# The contrasting regulatory effects of valproic acid on ferroptosis and disulfidptosis in hepatocellular carcinoma

**DOI:** 10.7150/thno.115661

**Published:** 2025-08-16

**Authors:** Rongrong Liu, Xinyan Li, Jiayi Xu, Liangwen Yan, Kailing Hu, Mengjiao Shi, Yinggang Zhang, Yaping Zhao, Yudan Fan, Gang Wang, Ying Guo, Yetong Feng, Pengfei Liu

**Affiliations:** 1 Department of Critical Care Medicine, National & Local Joint Engineering Research Center of Biodiagnosis and Biotherapy, The Second Affiliated Hospital of Xi'an Jiaotong University, Xi'an, China.; 2 International Joint Research Center on Cell Stress and Disease Diagnosis and Therapy, National & Local Joint Engineering Research Center of Biodiagnosis and Biotherapy, The Second Affiliated Hospital of Xi'an Jiaotong University, Xi'an, China.; 3 Shaanxi Provincial Clinical Research Center for Hepatic & Splenic Diseases, The Second Affiliated Hospital of Xi'an Jiaotong University, Xi'an, China.; 4 Key Laboratory of Surgical Critical Care and Life Support, Xi'an Jiaotong University, Ministry of Education of China, Xi'an, China.; 5 Core Research Laboratory, The Second Affiliated Hospital of Xi'an Jiaotong University, Xi'an, China.; 6 Key Laboratory of Environment and Genes Related to Diseases, Xi'an Jiaotong University, Ministry of Education of China, Xi'an, China.

**Keywords:** valproic acid, ferroptosis, disulfidptosis, labile iron pool, G6PD

## Abstract

**Background**: Valproic acid (VPA), a branched short-chain fatty acid, is extensively utilized as both an antiepileptic medication and a mood stabilizer. However, the complete pharmacological functions of VPA on programmed cell death are still not fully understood. In this study, we investigated the role of VPA in modulating ferroptosis and disulfidptosis, which are emerging forms of programmed cell death triggered by lipid peroxidation and disulfide stress respectively.

**Methods**: Herein, the network pharmacology analysis, genome-wide mRNA transcription assay and metabolomics analysis were performed to predict the major pharmacological action and potential targets of VPA. To confirm the hypothesis, pharmacological targeting model and gene knockdown model was created in our work. The pharmacological action of VPA on ferroptosis and disulfidptosis was evaluated respectively.

**Results**: Our findings primarily indicated that the potential targets of VPA were linked to hepatocarcinogenesis and programmed cell death. Additionally, omics data suggested that VPA could significantly influence iron transport and glucose homeostasis. Notably, VPA heightened the susceptibility of hepatocellular carcinoma (HCC) cells to ferroptosis by increasing the labile iron pool, facilitating the accumulation of free iron through enhanced cellular ferritinophagy and reduced ferritin expression. Furthermore, VPA promoted the transcription of glucose-6-phosphate dehydrogenase (G6PD) and impacted glutathione (GSH) metabolism. The activation of the NRF2-G6PD pathway induced by VPA further augmented the production of NADPH and GSH, which subsequently inhibited the formation of disulfide bonds among various cytoskeletal proteins, as well as disulfidptosis in HCC cells.

**Conclusion**: Overall, our results highlight the significant role of VPA in differentially regulating ferroptosis and disulfidptosis in HCC cells, thereby offering a precise avenue for addressing drug-resistant HCC in clinical practice.

## Introduction

Distinct from apoptosis, necroptosis or autophagic cell death, ferroptosis is a novel form of programmed cell death, and characterized by iron-dependent lipid peroxidation as well as and the lethal reactive oxygen species (ROS) derived from iron metabolism 1, 2. Numerous studies currently suggest that ferroptosis is implicated in a wide range of diseases, such as ischemia-reperfusion injury, Parkinson's diseases, Alzheimer's diseases and stroke 3-6. Furthermore, the involvement of ferroptosis in various biological processes, including mitochondrial metabolism and fatty acid metabolism, has been validated by multiple research groups 1, 7-11. Given the crucial position of glutathione (GSH), the substrate of phospholipid hydroperoxidases in the regulation of cellular redox contents, cysteine/glutamate antiporter (system Xc^-^) play a significant role in maintaining redox homeostasis, and disruption of cystine transport impairs intracellular glutamate metabolism and GSH production process, ultimately leading to ferroptosis. Therefore, numerous genes with anti-oxidant properties, including glutathione peroxidase 4 (GPX4), ferroptosis suppressor protein 1 (FSP1), nuclear factor erythroid 2-related factor 2 (NRF2), dihydroorotate dehydrogenase (DHODH), et al., have been identified as suppressors of ferroptosis 1, 11-15. Additionally, the level of free reactive iron also determines cellular response to ferroptosis inducers. Typically, intracellular iron in the form of ferritin which consists of ferritin heavy chain (FTH1) and ferritin light chain (FTL). Differently, FTH is known as an iron reductase, while FTL serves as the primary carrier for storing iron 16. The degradation of ferritin leads to cellular iron overload and promote ferroptosis by increasing ROS derived from iron metabolism 17-19. Therefore, the iron chelation therapy is considered as an important strategy against ferroptosis-related diseases.

Serving as a crucial antiporter on the cell membrane as well as a canonical ferroptosis suppressor in system Xc^-^, solute carrier family 7 member 11 (SLC7A11) has been found to play an unexpected role in regulating disulfidptosis, a recently identified form of cell death. Disulfidptosis primarily occurs in glucose-deprived cells that exhibit high SLC7A11 expression. Under the condition of glucose deprivation, SLC7A11-induced excessive cystine importation and cystine reduction to cysteine deplete nicotinamide adenine dinucleotide phosphate (NADPH) and disrupt the redox system, leading to significant cell death characterized by aberrant increased intracellular disulfide stress and enhanced disulfide bonds in actin cytoskeleton. Therefore, disulfidptosis provides a novel insight into the therapeutic strategy against cancers with high level of SCL7A11 expression. Research has shown that the Rac1-WRC pathway-mediated actin polymerization plays a critical role in disulfidptosis regulation and promotes the cell death progress 20, 21. Recently, thioredoxin reductase 1 (TrxR1) has been identified as a new disulfidptosis suppresser, and the inhibition of TrxR1 effectively sensitizes glucose-starved glioblastoma cells to disulfidptosis, highlighting the potential of targeting TrxR1 and disulfidptosis regulation as a novel therapeutic strategy in glioblastoma 22. In addition, comprehensive multi-omics analysis has elucidated the molecular landmarks of disulfidptosis across different cancer types, which provide opportunities to relieve drug resistance in clinical settings 23. However, the precise mechanisms underlying disulfidptosis regulation as well as the effective strategy to induce disulfidptosis *in vivo* haven't been fully elucidated. Notably, the current discovery of effective small molecular modulators targeting disulfidptosis is limited, which restricts the clinical application of the disulfidptosis model.

While ferroptosis exploits iron metabolic vulnerabilities, disulfidptosis targets cytoskeletal redox homeostasis. Both of them represent non-apoptotic death pathways effective against apoptosis-resistant malignancies. Critically, their activation shares dependence on amino acid transporters, SLC7A11, although the regulatory mechanisms differ significantly between the two models of cell death 21, 24. Ferroptosis is characterized by iron-dependent lipid peroxidation mediated by system Xc⁻ inhibition and other regulators, such as GPX4. Consequently, the reduced activity of SLC7A11 inhibits the synthesis of GSH, thereby promoting the ferroptosis process. Disulfidptosis is marked by an abnormal increase in intracellular disulfide stress due to the depletion of NADPH pools, a process that can be exacerbated by the overexpression of SLC7A11. This duality positions SLC7A11 could be considered as a context-dependent therapeutic switch, in which its inhibition prevents ferroptosis but may induce disulfidptosis during glucose limitation.

As a highly prevalent medication, valproic acid (VPA) holds multifaceted therapeutic action against various neurological and psychiatric disorders. Currently, VPA is mainly considered as a well-tolerated anticonvulsive drug as well as a short-chain fatty acid histone deacetylase (HDAC) inhibitor. Because of the important position of HDAC in the regulation of gene transcription, HDAC is regarded as a target in the treatment of some diseases, such as hypertension, myocardial infarction injury, glomerular diseases and cancer, and VPA also holds great value in the treatment of those diseases 25-29. For example, VPA treatments significantly rescue cardiac damage after myocardial infarction, and upregulate the transcription of *Foxm1* gene in heart, identifying *Foxm1* as a potential key target of VPA. Meanwhile, the suppression of FOXM1 activity blocks the cardiac protective function of VPA, indicating the cardiac protective effect of VPA is dependent of *Foxm1* activation 28. Differently, VPA hold the potential to arrest cell growth and enhance apoptosis and mitochondrial perturbations in cancer cells, and the combined treatment with VPA and 5'-deoxy-5-fluorouridine shows more effective therapeutic action than single treatment 30, 31. Therefore, VPA could play different role in cancer cells and normal cells. Recently, some researchers has reported that VPA also hold promising potential in the treatment of ferroptosis-related diseases. The treatment with VPA ameliorates cauda equina injury via relieve ferroptosis and neuroinflammation, and the therapeutic action of VPA totally depends on the suppressing of HDAC2 32. Additionally, our previous result also indicated that VPA treatment relieves lipid peroxidation and ferroptosis in tubular epithelial cells in cisplatin-induced acute kidney injury 33. However, the detailed mechanism need to be investigated intensively. Especially, the association between VPA and ferroptosis in cancer cells is still unclear for us.

Hepatocellular carcinoma (HCC) is considered as the most prevalent form of liver cancer, has emerged as one of the deadliest malignancies globally. In addition, both patients with hepatitis B or C infection and patients with other chronic liver diseases face a significantly elevated risk of developing HCC. Currently, HCC patients are often diagnosed at an advanced stage and characterized by marked resistance to chemotherapy, which not only limits the efficacy of chemotherapeutic interventions but also heightens the risk of recurrence post-treatment 34-36. Consequently, the development of novel strategies to combat drug resistance is critical for improving HCC treatment outcomes. Some studies have revealed the potential of VPA in HCC treatment. For example, VPA influences the expression of genes involved in apoptosis as well as ROS production, and enhances the effects of chemotherapy 37, 38. In this work, our findings primarily suggested that the treatment with VPA heightened the susceptibility of HCC to ferroptosis via altering the labile iron pool. Differently, VPA protected cells against glucose starvation-induced disulfidptosis by enhancing the transcription of glucose-6-phosphate dehydrogenase (G6PD), an essential enzyme for the synthesis of NADPH. The contrasting regulatory effects of VPA on ferroptosis and disulfidptosis offer a new perspective on the precise cancer treatment using VPA.

## Methods

### Chemicals and cell culture

Erastin (HY-15763), Sorafenib (HY-10201), RSL3 (HY-100218A), Cisplatin (HY-17394), Valproic acid (HY-10585), Deferasirox (HY-17359), Deferoxamine mesylate (HY-B0988), 6-Aminonicotinamide (HY-136057), Polydatin (HY-17589A), Sulforaphane (HY-13755), Vorinostat (HY-10221), Trichostatin A (HY-15144) and A-485 (HY-107455) were sourced from MCE. Hepatocellular carcinoma cell lines, HepG2 and Hep3B, were obtained from the American Type Culture Collection (ATCC, USA). MHCC97H cell line was sourced from the China Center for Type Culture Collection (China), while BEL-7404 cell line was purchased from the Cell Bank of Chinese Academy of Sciences (China). All of HCC cells were maintained in 37 °C incubator with 5% CO_2_. Dulbecco's Modified Eagle Medium (DMEM, High Glucose) with 10% FBS, 100 U/mL penicillin, and 0.1 g/mL streptomycin was utilized for cell culture. In our current work, different HCC cell lines were passaged approximately every three to four days, while the culture medium was refreshed every two days.

*G6PD* knockdown cells and *NCOA4* knockdown cells were generated using siRNA transfection method. The human *G6PD*-specific siRNA (RY204682) and *NCOA*-specific siRNA (RY25008426) were purchased from GENECARER, China. The transfection of siRNA were performed using Lipofectamine 3000 solution (Thermo Fisher Scientific) according to the manufacturer's instructions. Meanwhile, *NRF2* knockout cell line was established using CRISPR/Cas9 as our previous work 39.

### Cell viability assay

The cell viability was evaluated using CCK-8 method first. Briefly, 1×10^5^ cells were seeded into each well of 96-well plate containing 100 μL medium. After drug treatment, 10 μL of CCK-8 solution (Dojindo) were added into each well and the cells in different groups were further incubated at 37 °C for 1-2 h. Finally, the OD at 450 nm was measured using Microplate Reader. Herein, the well only containing medium was used as blank group, and the cell viability of ctrl group without any treatment was considered as “100 %”. The relative cell viability in different groups was calculated respectively. In addition, crystal violet staining was also performed in our work to evaluated cell viability in different groups. The cells were fixed with 4 % paraformaldehyde (Solarbio), then stained with crystal violet staining solution (Beyotine) for 10 min. After washed with PBS for three times, the stained cells in each group were captured using a scanner. Besides, the cell death in each group was also determined via propidium iodide (PI) staining. The PI staining solution (YEASEN) was added into each well and the cell samples were incubated at 37 °C for 20 min, then the stained cells were observed using a fluorescence microscope (Zeiss).

### RT-qPCR

RT-qPCR was utilized to determine gene transcription level in different groups, and the related operation is same as our previous work 40, 41. Herein, the total RNA from the various groups were extracted using TRIzol solution (Thermo Fisher Scientific), and 1 μg of total RNA was utilized for cDNA synthesis via reverse transcription using PrimeScript RT Reagent Kit (Takara). In our current work, normalization was performed using GAPDH as a reference gene, and all items were conducted in triplicate. The primer sequences (5'-3') used in RT-qPCR are summarized in Table S1.

### Chromatin immunoprecipitation assay (ChIP)-qPCR

In this study, the ChIP-qPCR assay was performed using EZChIP Kit (Merck). The operation is same as per our previous study 42. Herein, 1 μL of purified DNA sample in each group was used for qPCR assay, to evaluate the fucntion of potential ARE sequence located in human *G6PD* promoter. The primers (5'-3') specific for the sequence flanking potential ARE were shown as follow:

Human-*G6PD*-ARE1-F 5-TCTGAAGGCAGGTGCAGCAT-3

Human-*G6PD*-ARE1-R 5-CCCCAGAACTCATAGGCTTT-3

Human-*G6PD*-ARE2-F 5-AATCGCTTGAATCCAGGAGG-3

Human-*G6PD*-ARE2-R 5-ATATCCCTCGCAATCCTCTC-3

### Western blot

In our current work, the process of western blot assay was same as our previous description 43, 44. The primary antibodies used in our work were as follows: anti-SLC7A11 (1:1000; Proteintech, 26864-1-AP), anti-GPX4 (1:1000; Proteintech, 67763-1-Ig), anti-FTL (1:1000; Proteintech, 10727-1-AP), anti-GAPDH (1:3000; Proteintech, 60004-1-Ig), anti-LC3 (1:1000; Proteintech, 14600-1-AP), anti-p62 (1:1000; Proteintech, 18420-1-AP), anti-NRF2 (1:1000; Proteintech, 16396-1-AP), anti-FLNA (1:3000; Proteintech, 67133-1-Ig) and anti-DREBRIN (1:3000; Proteintech, 10260-1-AP). In addition, HRP-labeled secondary antibodies, including anti-rabbit IgG (1:3000; ABclonal, AS014) and anti-mouse IgG (1:3000; ABclonal, AS003), were used in this study. The protein samples harvested using sample buffer without any reducing agents were used for non-reducing western blot. Finally, the results of western blot were quantified across three biological replicates using ImageJ software. Herein, one-way ANOVA followed by Benjamini-Hochberg correction was performed to analyze the difference among three or more groups, and the adjust P-values for multiple comparisons were calculated in western blot analyses.

### Evaluation of malondialdehyde (MDA)

In order to assess lipid peroxidation in different groups, the Lipid Peroxidation (MDA) Assay Kit (MAK085, Sigma-Aldrich) was used to evaluate the MDA level. The cell samples were prepared following the manufacturer's instructions, and the signal in each group was measured using a Microplate Reader eventually.

### BODIPY staining and intracellular iron measurement

For the evaluation of lipid peroxidation and intracellular iron content, cell samples in different groups were initially rinsed with PBS. They were then incubated with 1 μM BODIPY 581/591 C11 (Thermo Fisher) and FerroOrange solution (Dojindo) for 30 min at 37 °C respectively. Following the incubation, different cell samples were washed twice with PBS. At last, the level of lipid peroxidation and intracellular iron in each group were measured using the FACSAria II Flow Cytometer (BD Biosciences). In our work, data interpretation was analyzed using the FlowJo 7.6.1 software. In addition, the level of free iron in tumor tissues were measured using Total Iron Colorimetric Assay Kit (Elabscience).

### Network pharmacology analysis

The Network pharmacology analysis were carried out according to our previous study 45. Based on the chemical structure of VPA, both TargetNet 46 and SwissTargetPrediction database 47 were used to predicted the potential bioavailable targets. The overlap section of two different databases was chosen for cluster analysis and interaction networks assay using Metascape 48 and STING database 49 respectively. The DisGeNET database (https://disgenet.com/) was used to check genes and variants associated with hepatocarcinogenesis.

### RNA sequencing and Metabolomics assay

Herein, both VPA-treated and VPA-untreated HCC cells were prepared for RNA sequencing and Metabolomics assay, which were performed as our previous work 45. In brief, the sequencing libraries were prepared using NEBNext® Ultra™ RNA Library Prep Kit for Illumina® (NEB), then underwent sequencing using Illumina Novaseq 6000 platform (Novogene Beijing, China). The metabolomics assay were completed by PANOMIX Biomedical Tech Co., LTD in Suzhou, China.

### Live cell immunofluorescence microscopy

The ptf-LC3 vector (mRFP-GFP-LC3 reporter construct) was used for cell transfection to assess the impact of VPA on autophagic flow. Both MHCC97-H cells and HepG2 cells were transfected with ptf-LC3 vector using Lipofectamine 3000 solution (Thermo Fisher Scientific), then treated with VPA for 24 h. Both VPA-treated cells and VPA-untreated cells were imaged using a Zeiss fluorescence microscope.

### Luciferase reporter gene assay

The 41-bp potential ARE-containing sequences were cloned into pGL4.22-luciferase vector, and the vector were transfected into MHCC97-H cells and HepG2 cells using Lipofectamine 3000 solution (Thermo Fisher Scientific). Additionally, the dual luciferase reporter assay kit (Promega) was used to assess luciferase activity. As our previous work 42, the relative luciferase activity (value of Firefly luciferase normalized to Renilla luciferase) was measured to determine the function of potential ARE-containing sequences.

### Cytoskeleton staining

In current study, Actin-Tracker Red-Rhodamine (Beyotime) was used to stain the cytoskeleton across various groups. The cells were firstly fixed using 4 % paraformaldehyde (Solarbio) for 20 min at room temperature. Then, the samples were rinsed three times with PBS containing 0.1 % Triton X-100. Actin-Tracker Red-Rhodamine solution (1:200) was next used to stain the cytoskeleton structure for 30 min. At last, DAPI (Beyotime) was used as a stain for nuclear quantitation, and the samples were imaged using a Zeiss fluorescence microscope.

### Analysis of G6PD activity and Glutathione Reductase (GR) activity

The activity of G6PD and GR across various groups was determined using G6PD Activity Assay Kit (BC0260, Solarbio) and GR Activity Assay Kit (BC1165, Solarbio) respectively. The cell samples were prepared following the manufacturer's instructions, and the signal in each group was measured using a Microplate Reader eventually.

### Evaluation of NADP+-NADPH metabolism and GSH-GSSH metabolism

Both NADP^+^-NADPH metabolism and GSH-GSSH metabolism were evaluated in different groups. Cell samples were prepared according to the manufacturer's instructions, and the levels of NADP^+^/NADPH and GSH/GSSH were determined using NADP^+^/NADPH Assay Kit (Beyotime Biotechnology, S0180S) and GSH and GSSG Assay Kit (Beyotime Biotechnology, S0053) respectively.

### Animal studies

All animal experiments were approved by The Biomedical Ethics Committee of Health Science Center of Xi'an Jiaotong University (Approval number: 2022-1371) on June 9th 2022. SCID Beige Mice obtained from Charles River Laboratories were utilized in the xenograft mouse model. The male mice (weight = 22-24g), aged 7-8 weeks, were injected with a cancer cell suspension containing 5×10^6^ cells per mouse intradermally. The mice were divided into six groups: Ctrl, VPA, Erastin, Erastin+VPA, Erastin+DFS and Erastin+VPA+DFS. Tumor measurements were taken using a vernier caliper, and the tumor volume was calculated using the formula: Volume = π/6 × Length × Width². In this study, both Erastin (15 mg/kg) and VPA (200 mg/kg) were dissolved in a 5% DMSO/corn oil solution and administered intraperitoneally to the mice twice weekly for a duration of five weeks. Meanwhile, Deferasirox (DSF, 100 mg/kg) were administered by oral gavage. After the treatment concluded, the mice were euthanized, and the tumor weight, metabolic activity as well as gene expression were assessed across the different groups (n = 6).

### Statistical analysis

In this work, the results are presented as mean ± SD. Power analyses (power = 0.8) was performed to ensure sufficient sample sizes for different assays. SPSS version 17.0 software package was used for all of statistical analysis in the study. Herein, Unpaired Student's t-tests were used to compare two groups. Moreover, one-way ANOVA followed by Benjamini-Hochberg correction was performed to analyze the difference among three or more groups. In addition, Student's t-test was operated as a one-tailed test. The p-value less than 0.05 is considered statistically significant. For the significant comparison, effect size assay was further performed and the value of Cohen's d over 0.8 represents a large effect size.

## Results

### Network pharmacology analysis of VPA potential targets and function

In our investigation, we utilized both SwissTargetPrediction and TargetNet databases to predict the potential bioavailable targets of VPA, relying on its chemical structure as illustrated in Figure S1A. The SwissTargetPrediction database identified 100 potential bioavailable targets of VPA, while the TargetNet database revealed 202 potential targets. Notably, there were 32 common targets identified between the two databases (Figure S1B-C and Table S2), which were subsequently selected for cluster analysis via Metascape. Initially, a protein-protein interaction network was constructed based on the 32 common targets (Figure S1D). Furthermore, the top 20 enriched terms from the cluster analysis were compiled into a network plot, revealing that Metabolism of lipids, Prostaglandin synthesis and regulation and small molecule biosynthetic process were the most pertinent biological processes and pathways underlying VPA pharmacological function. Besides, the regulation of protein and some other metabolic processes were also identified as potential signaling targets of VPA (Figures S1E-G). Additionally, the cluster analysis highlighted a robust connection between cancer-related pathways/metabolic processes and the potential targets of VPA. This finding implies that VPA may possess therapeutic action in metabolic cancer treatment, likely through its influence on fatty acid metabolism as well as one-carbon metabolic process (Figure S1G). Based on the important position of HCC in metabolic cancer field, the genes related with hepatocarcinogenesis were compiled using the DisGeNET database (https://disgenet.com/), revealing that 5 potential bioavailable targets of VPA, including the key ferroptosis maker PTGS2, overlapped with genes associated with hepatocarcinogenesis (Figure S1H-I). This suggests that VPA treatment could represent a novel strategy against HCC, with the ferroptosis-related pathway being potential mechanisms of action.

### VPA treatment sensitizes HCC to ferroptosis

**Figure 1:**
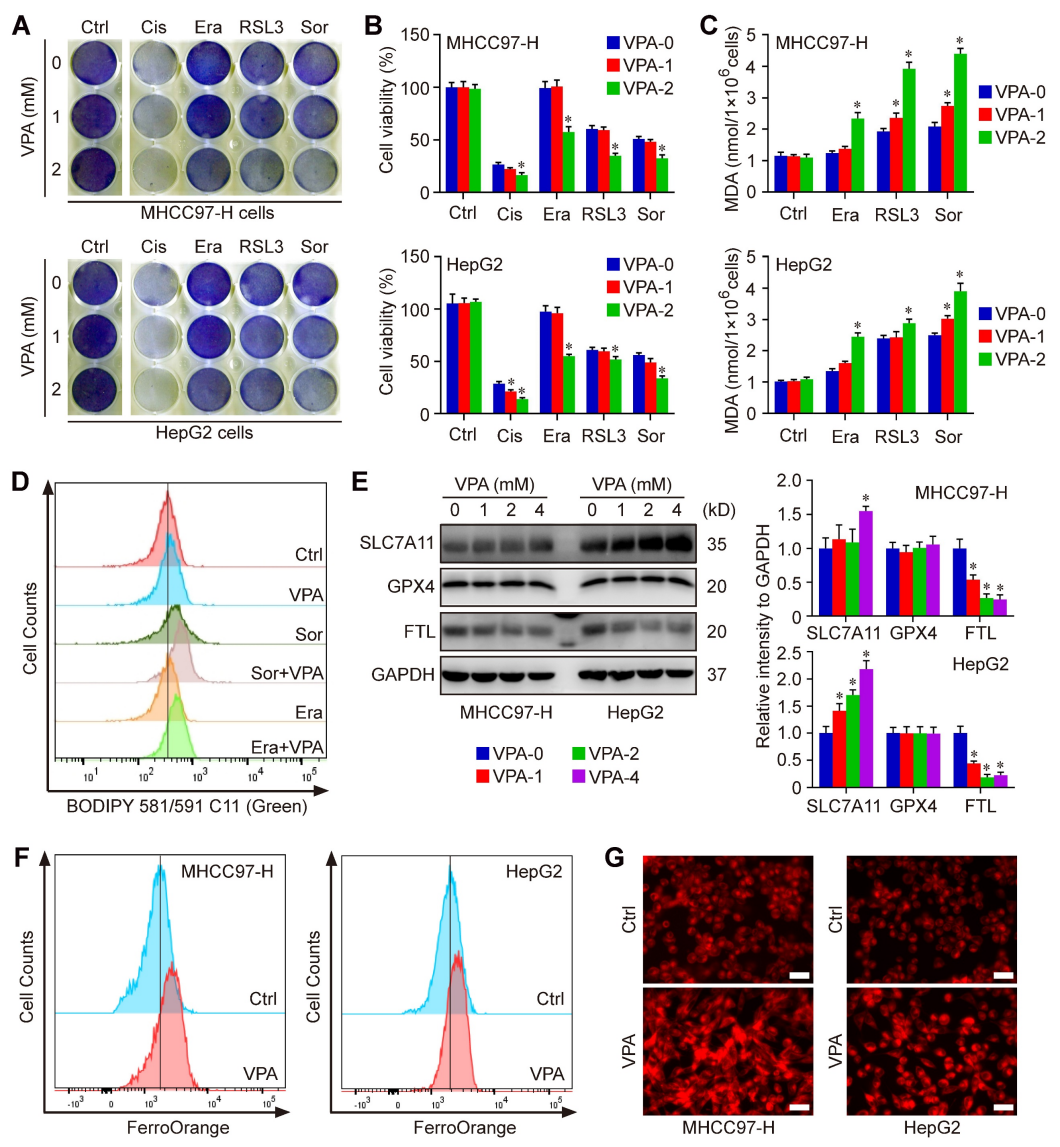
** VPA treatment sensitizes HCC to ferroptosis**. Both MHCC97-H and HepG2 cells were subjected to VPA treatment (1 mM and 2 mM) for a duration of 24 h, then exposed to Erastin (Era, 5 μM), Sorafenib (Sor, 1 μM), RSL3 (2 μM)) and Cisplatin (Cis, 10 μM) to induce cell death. Both crystal violet staining (A) and CCK-8 assay (B) were utilized to evaluate cell viability in different groups. In addition, the level of MDA was measured to access the lipid peroxidation (C), which was further confirmed via BODIPY staining (D). Additionally, the impact of VPA on different ferroptosis regulators was examined using western blot (E) and the level of free Fe^2+^ was measured using FerroOrange staining (F-G, Scale bar = 50 μm). Data are expressed as mean ± SD. The P value less than 0.05 was considered statistically significant and the value of Cohen's d over 0.8 represents a large effect size. *: P < 0.05 and Cohen's d > 0.8 compared to Ctrl group.

The network pharmacology analysis has revealed the close relationship between VPA potential targets and ferroptosis regulation. We next tested the effect of VPA on different compounds-induced ferroptosis. Herein, the system Xc^-^ inhibitors, Erastin and Sorafenib, as well as the GPX4 inhibitor, RSL3, were employed to pharmacologically induce ferroptosis in MHCC97-H and HepG2 cells. Both crystal violet staining (Figure 1A) and CCK-8 assay (Figure 1B) indicated that the VPA treatment heightened the susceptibility of HCC cells to different compounds-induced ferroptosis. In addition, the response of VPA-treated HCC cells were more sensitive to Cisplatin-induced cell death, indicating the potential of VAP in apoptosis regulation as well (Figure 1A-B). Additionally, the amount of MDA was further measured to evaluate the lipid peroxidation in each group. Similarly, our findings showed that pretreatment with VPA markedly increased the level of MDA in different ferroptosis models (Figure 1C). Then, lipid peroxidation across various groups were further quantified using BODIPY staining. The results indicated that both Erastin-induced and Sorafenib-induced lipid peroxidation and cell ferroptosis could be further enhanced by VPA treatment (Figure 1D).

To investigate the underlying mechanism associated with VPA and ferroptosis, the impact of VPA on different ferroptosis regulator was examined subsequently. Our data revealed that VPA treatment didn't affect the expression of GPX4, and even increased the protein level of SLC7A11. Both GPX4 and SLC7A11 were considered as most principal ferroptosis suppressors. Therefore, the pharmacological function of VAP on ferroptosis regulation was independent of GPX4 or SLC7A11 signaling pathway. In addition, the level of FTL, the primary carrier for storing iron, was measured herein. The results indicated that the protein level of FTL was decreased by VPA treatment in a dose-dependent manner, highlighting the significance of iron transport in VPA function on modulating ferroptosis (Figure 1E). Subsequently, the level of free Fe^2+^ was measured using FerroOrange staining, and the results further indicated that VPA treatment increased the amount of free Fe^2+^ in HCC cells (Figure 1F-G). Consequently, the treatment with VPA promotes the conversion from ferritin-bound Fe^3+^ to free Fe^2+^, which contributes to the labile iron pool and sensitizes HCC to ferroptosis.

To validate the above conclusion, we conducted time-course experiments to assess the impact of VPA on iron accumulation and lipid peroxidation. In this study, both MHCC97-H and HepG2 cells were subjected to VPA treatment for durations of 8 h, 16 h, and 24 h, respectively. Subsequently, the cell samples were collected for FerroOrange staining. The findings revealed that VPA treatment led to an increase in the levels of free Fe^2+^ in HCC cells in a time-dependent manner (Figure S2A). The cells treated with VPA were then further exposed to Sorafenib. Following this, lipid peroxidation across different groups was quantified using BODIPY staining. The results showed that the lipid peroxidation and cell ferroptosis induced by Sorafenib could also be augmented by VPA treatment in a time-dependent manner (Figure S2B).

### VPA modulates multiple genes pivotal for ferroptosis and iron transport regulation

**Figure 2:**
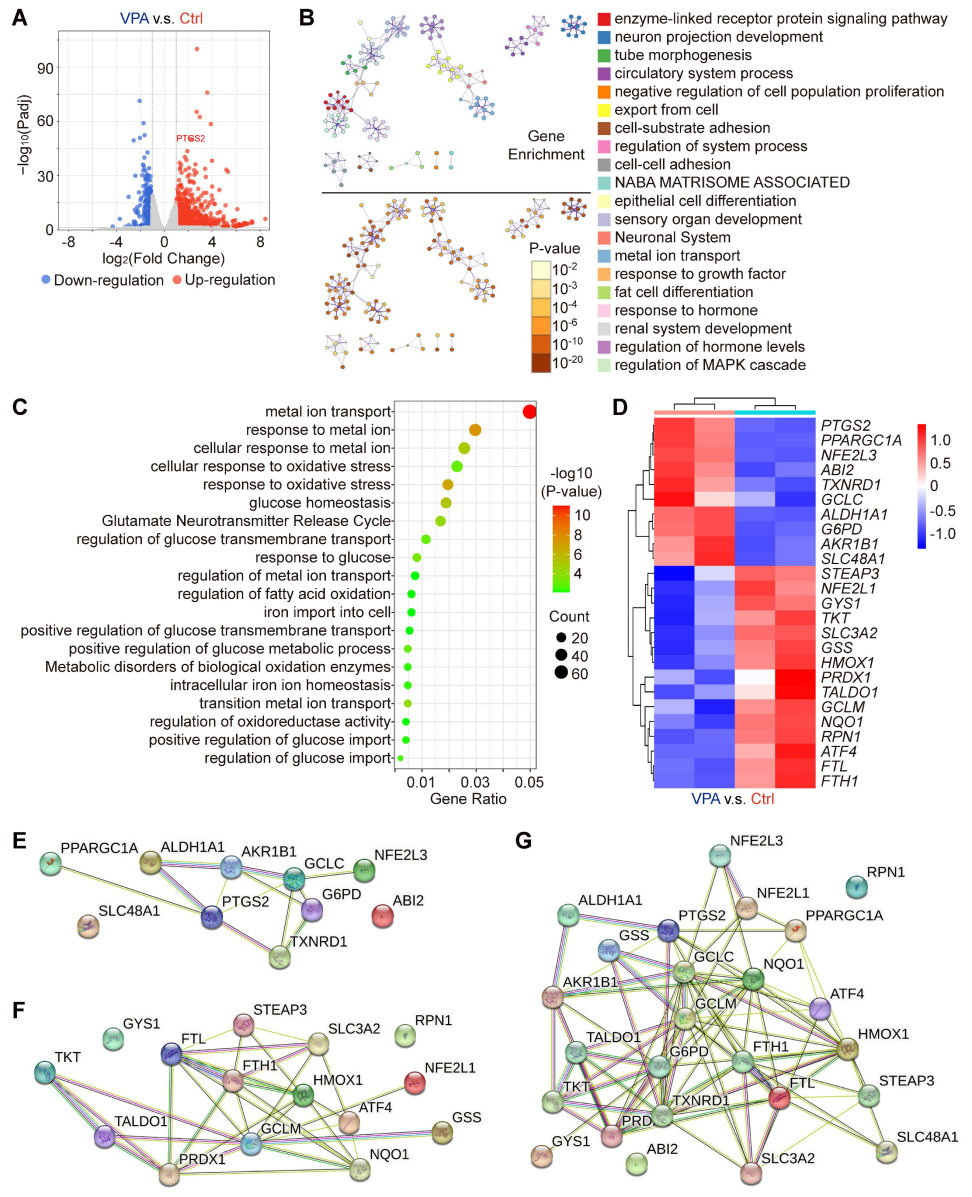
** VPA influences various genes essential for the regulation of oxidative stress and ferroptosis.** In this study, the HCC cell line MHCC97-H was subjected to VPA treatment (2 mM) for a duration of 24 h. Subsequently, the cell samples were collected for RNA sequencing analysis. Initially, a volcano plot was created to assess the impact of VPA treatment on gene transcription (A). Following this, differentially expressed genes were utilized for cluster analysis via Metascape. The network illustrating the top 20 enriched terms for these differentially expressed genes was color-coded by cluster ID, with the same terms further distinguished by p-value (B). The elements related to metal ion transport and oxidative stress were compiled in Figure 2C, while the key genes were presented in a heatmap (D). Furthermore, both upregulated genes (E) and downregulated genes (F) depicted in the heatmap were analyzed using the STING database, and the interactions among all aforementioned genes were illustrated in a separate Protein-Protein Interaction Network (G).

Our results have indicated the potential therapeutic effects of VPA in the context of hepatocarcinogenesis via regulating ferroptosis. To substantiate the effect and explore related key mechanism, an RNA sequencing assay was conducted in the current study to examine the variations in genome-wide mRNA expression profiles. The HCC cell line, MHCC97-H, was subjected to VPA (2 mM) treatment. Following the 24-h treatment, the transcription levels of 981 genes were upregulated, and 298 genes were downregulated in the VPA group relative to the Ctrl group (Figure 2A and Table S3), which were selected for further cluster analysis. The top 20 enriched terms from the cluster analysis of VPA-modulated genes were represented as network plots, and the pathways and processes most relevant to VPA function included enzyme-linked receptor protein signaling pathway, neuron projection development and tube morphogenesis. Notably, metal ion transport emerged as the primary pathways linked to VPA-modulated genes (Figure 2B). In addition, genes related with cellular response to oxidative stress (such as *GCLM*, *HMOX1* and *NQO1*), fatty acid oxidation (such as *PPARGC1A* and *ATF4*), iron transport (such as *FTL* and *FTH1*) and glucose homeostasis (such as *G6PD* and *GYS1*) were also involved in VPA pharmacological function (Figure 2C-D). Differently, VPA treatment enhanced the transcription of *G6PD*, but suppressed the expression of various anti-oxidative stress genes (such as *GCLM*, *HMOX1* and *NQO1*). The protein-protein interaction network was constructed using STING database based on the identified upregulated (Figure 2E) and downregulated genes (Figure 2F), and another network was also created based on all of those VPA-modulated genes (Figure 2G). The results further confirmed the important position of iron transport-related genes as well as anti-oxidative stress genes, indicating close association between VPA function and ferroptosis regulation.

### Ferritinophagy plays a significant role underlying VPA action concerning ferroptosis

**Figure 3:**
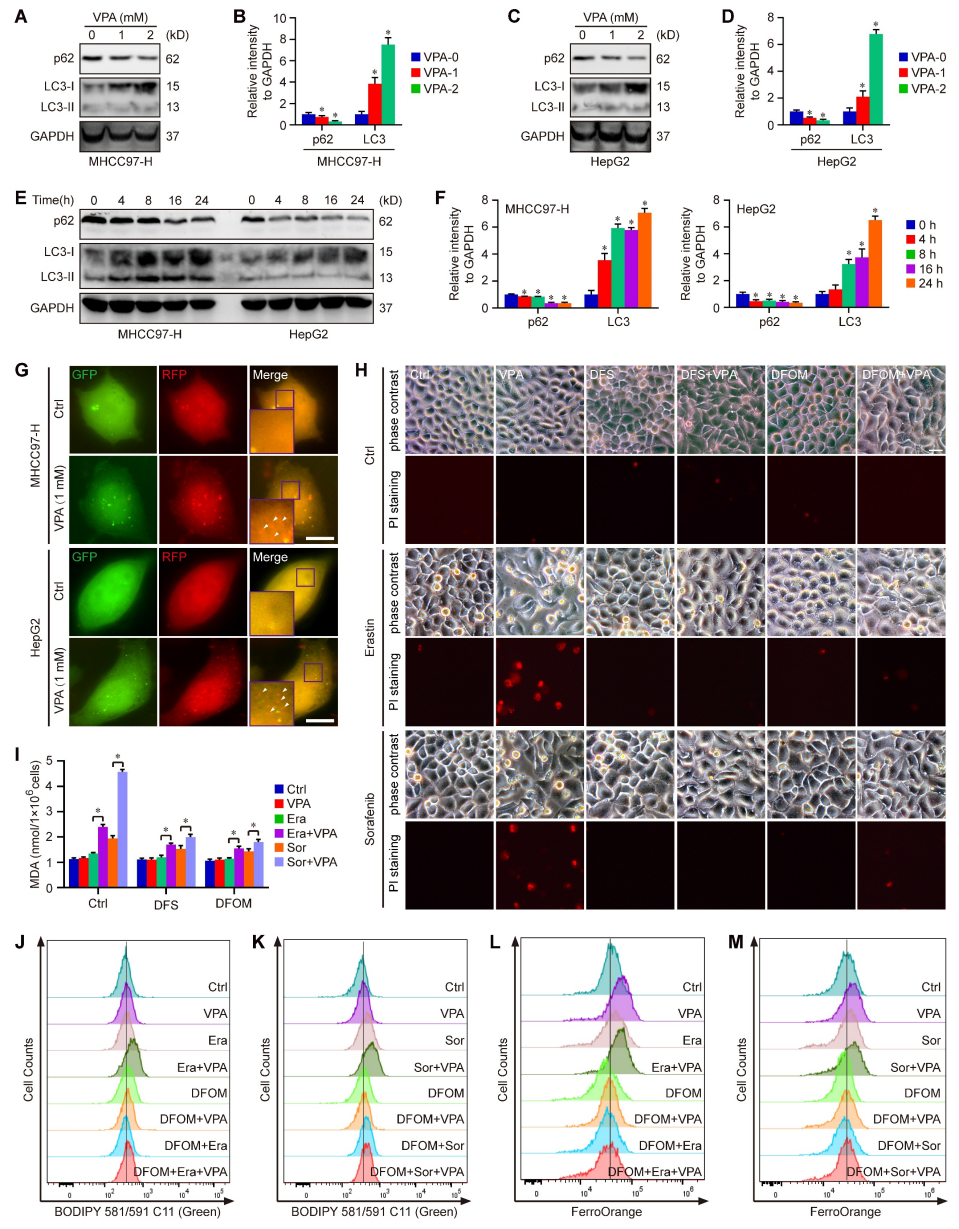
** Ferritinophagy holds a crucial position underlying the action of VPA concerning ferroptosis.** Both MHCC97-H and HepG2 cells were subjected to VPA treatment (1 mM and 2 mM) for a duration of 24 h, then harvested for western blot assay. The autophagy markers, p62 and LC3, were measured in our work (A-D). In addition, the effect of VPA treatment in different time points was also analyzed (E-F). The impact of VPA treatment on autophagic flux was evaluated using live cell immunofluorescence assay, and the red puncta (white arrow) represented autolysosome (G, Scale bar = 10 μm). Moreover, MHCC97-H cells were treated with VPA plus iron chelators (Deferasirox (DFS) and Deferoxamine mesylate (DFOM)), then exposed to ferroptosis inducers. Cell death was analyzed in each group via PI staining (H, Scale bar = 10 μm). The levels of lipid peroxidation across various groups were accessed via MDA measurement (I) and BODIPY staining (J-K). Finally, the level of free Fe^2+^ was determined using FerroOrange staining (L-M). Data are expressed as mean ± SD. The P value less than 0.05 was considered statistically significant and the value of Cohen's d over 0.8 represents a large effect size. *: P < 0.05 and Cohen's d > 0.8 compared to Ctrl group or compared between different groups.

Moreover, the degradation process of ferritin primarily depends on autophagy, which is considered as ferritinophagy 17, 50, 51. Thus, the effect of VPA on cell autophagy was assessed in MHCC97-H and HepG2 cells. As expected, our results showed that the treatment with VPA decreased the protein level of p62 and increased the protein level of LC3 in both dose-dependent and time-dependent manners (Figure 3A-F). Besides, the effect of VPA treatment on autophagic flux in HCC was also evaluated via tandem mRFP-GFP-LC3 reporter construct. Herein, both MHCC97-H and HepG2 cells were transfected with the construct, then treated with VPA for 24 h. We observed that the amount of red puncta was increased in VPA-treated cells compared with the untreated cells, indicating that VPA treatment enhanced the formation of autolysosome and promoted autophagy process in HCC cells (Figure 3G). Therefore, VPA could contribute to labile iron pool via enhancing ferritinophagy and FTL degradation.

To further confirm the above conclusion, two different iron chelators, Deferasirox (DFS) and Deferoxamine mesylate (DFOM), were utilized in our current work. Herein, MHCC97-H cells were treated with VPA plus iron chelator, then exposed to ferroptosis inducers (Erastin and Sorafenib). Cell death was analyzed in each group via PI staining. The results showed that both DFS and DFOM obviously effectively blocked the effect of VPA on ferroptosis and rescued Erastin or Sorafenib-induced cell death (Figure 3H). Additionally, the lipid peroxidation was further evaluated through measuring MDA amount and BODIPY staining in different groups. As shown in Figure 3I-K, the pretreatment with iron chelator inhibited VPA function and suppressed MDA production as well as lipid peroxidation in ferroptosis model. Finally, the level of free Fe^2+^ was measured using FerroOrange staining, and the results further confirmed the blocking effect of DFOM on VPA function in ferroptosis. The findings provided additional evidence supporting the inhibitory impact of iron chelator on VPA function in the context of ferroptosis (Figure 3L-M).

**Figure 4:**
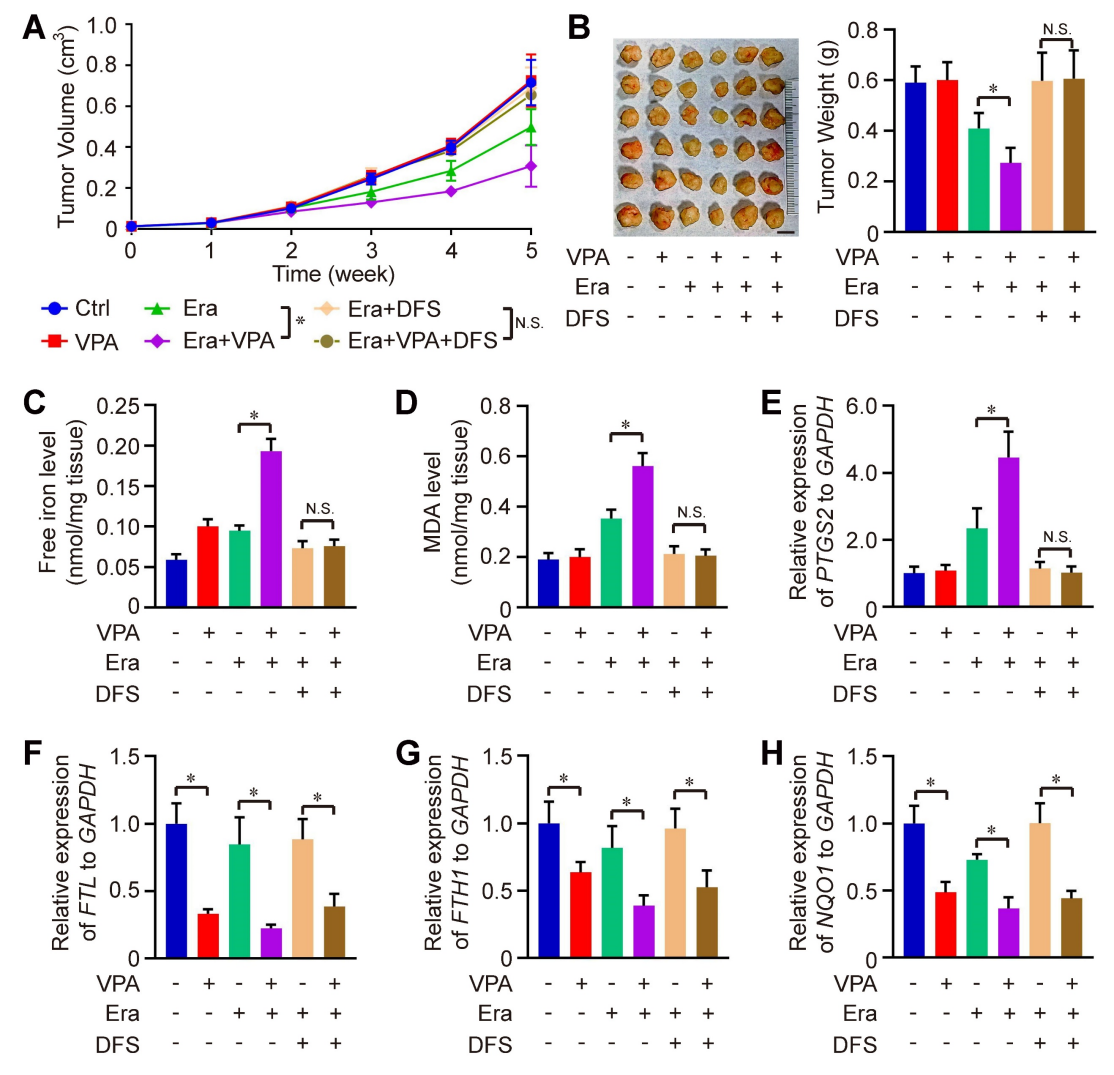
** The therapeutic effectiveness of the combination of Erastin and VPA *in vivo* against HCC.** Wild type HCC cells were injected into SCID mice, and the mice were further treated with Erastin (Era), VPA and DFS. Herein, tumor volume (A) and tumor weight (B, Scale bar = 1 cm) were measured respectively. In addition, the levels of free iron (C), MDA (D) and expression of *PTGS2* (E) were determined in tumor tissues from each group, and the transcription of *FTL*, *FTH1* and *NQO1* in tumor tissues were evaluated using RT-qPCR in our work (F-H). Data are expressed as mean ± SD. The P value less than 0.05 was considered statistically significant and the value of Cohen's d over 0.8 represents a large effect size. *: P < 0.05 and Cohen's d > 0.8 compared between different groups.

We also evaluated the therapeutic effectiveness of the combination of Erastin and VPA *in vivo* against HCC. Remarkably, the combined treatment of Erastin and VPA demonstrated enhanced therapeutic results compared to Erastin treatment alone, with significant reductions in both tumor volume and weight observed in the combination group when compared to the individual groups receiving Erastin. The effect could be totally blocked under the condition of DSF treatment (Figure 4A-B). Consistent with* in vitro* results, the levels of free iron, MDA and expression of *PTGS2* were higher in the combination group than in the groups treated with Erastin alone, which could be rescued by iron chelators DFS treatment, indicating that enhanced labile iron pool could play a significant role underlying VPA action concerning ferroptosis (Figure 4C-E). In addition, RNA-seq data indicated the effect of VPA on genes related with iron transport (such as *FTL* and *FTH1)* and anti-oxidative stress (such as *NQO1*) (Figure 2D), so the function of VPA *in vivo* was evaluated herein. We observed that VPA effectively blocked the transcription of *FTL*, *FTH1* and *NQO1* in tumor tissues, with or without Erastin treatment, and DFS treatment didn't affect the function of VPA (Figure 4F-H).

Furthermore, to assess the significance of ferritinophagy in the function of VPA, a cell model with *NCOA4* knockdown was created using the siRNA transfection method, which impeded the ferritinophagy process. Initially, the effectiveness of the *NCOA4* knockdown was measured using qPCR, and the findings revealed that the specific *NCOA4*-siRNA-#3 exhibited the most significant effect and was selected for subsequent assays (Figure S3A). Both wild type and *NCOA4* knockdown cells were subjected to VPA treatment followed by glucose deprivation. The findings indicated that *NCOA4* knockdown effectively hindered the impact of VPA on ferroptosis and mitigated Sorafenib-induced cell death (Figure S3B). Additionally, the levels of free Fe^2+^ and lipid peroxidation were further assessed through FerroOrange staining and BODIPY staining across various groups. The results indicated that *NCOA4* knockdown diminished VPA function and decreased the levels of free iron as well as lipid peroxidation in the ferroptosis model (Figure S3C-D). Therefore, VPA-induced downregulation of *FTL* transcription could contribute to labile iron pool and enhance the susceptibility of HCC to ferroptosis.

Due to the recognition of VPA as a potent HDAC inhibitor, the histone acetyltransferase p300/CBP inhibitor (A-485) was utilized to verify the role of histone acetylation in the modulation of ferroptosis induced by VPA. Our findings showed that VPA treatment heightened the susceptibility of HCC cells to Sorafenib-induced ferroptosis, a phenomenon that was inhibited by concurrent treatment with the p300/CBP inhibitor (Figure S4A). Additionally, we assessed the levels of free Fe^2+^ and lipid peroxidation in different groups, and the results revealed that the co-treatment with the p300/CBP inhibitor diminished the efficacy of VPA and reduced the levels of free Fe^2+^ and lipid peroxidation within the ferroptosis model (Figure S4B-C).

### VPA modulates multiple metabolites related with programmed cell death

**Figure 5:**
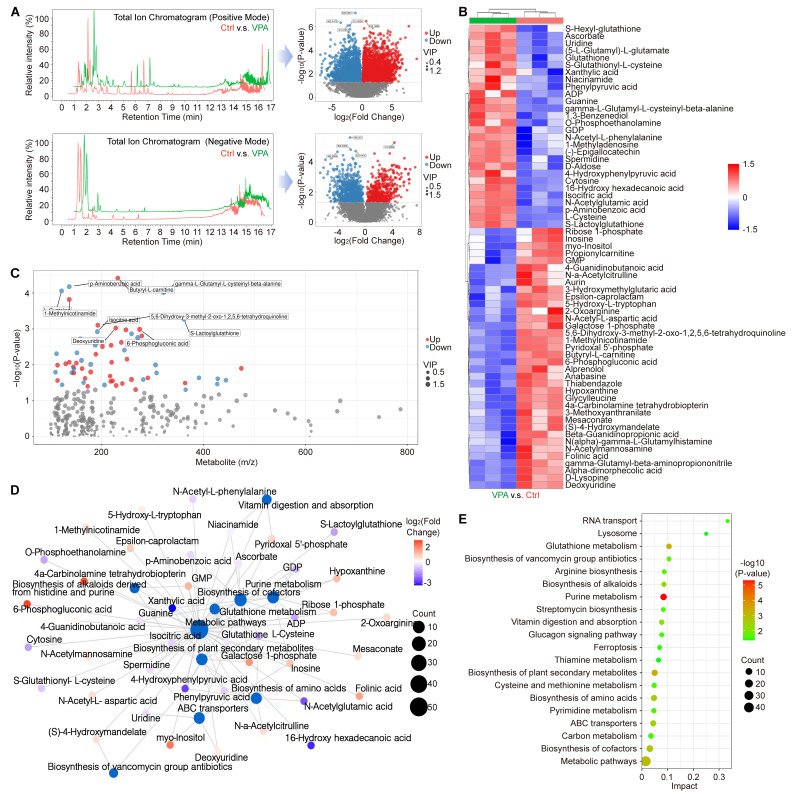
** VPA modulates multiple metabolites related with programmed cell death.** In this study, the HCC cell line MHCC97-H underwent treatment with VPA (2 mM) for a duration of 24 h. Subsequently, the cell samples were collected for metabolomics analysis. The primary metabolic alterations were assessed using both Positive Mode and Negative Mode (A). The analysis of secondary metabolites highlighted both upregulated and downregulated metabolites, as illustrated in Figure 5B. Furthermore, the relationship between mass-to-charge ratio and P value was examined in Figure 5C, while the interactions among differential metabolites and key metabolic pathways were depicted in a network plot (D). Lastly, Figure 5E presented the significant metabolic pathways in HCC cells that were affected by VPA.

Ferroptosis represents a form of programmed cell death characterized by the accumulation of free iron and lethal lipid peroxidation, which arises from an imbalance in redox homeostasis and cellular metabolism. To investigate this phenomenon and the related metabolic mechanism, a metabolomics analysis was conducted to assess the metabolic profiles between ctrl group and VPA treated group. In current research, we examined the primary metabolic alterations utilizing both Positive Mode and Negative Mode methodologies. The results in Positive Mode revealed that in VPA-treated MHCC97-H cells, there were 3,224 metabolites that were downregulated and 2,412 metabolites that were upregulated when compared to untreated cells. In the Negative Mode analysis, 562 metabolites were downregulated and 1018 metabolites were upregulated in the VPA group relative to the control group (Figure 5A). Furthermore, in the secondary metabolites assessment, our data indicated that 36 metabolites were downregulated and 28 metabolites were upregulated in the VPA-treated group compared to the control group (Figure 5B and Table S4). Among the metabolites studied, the concentrations of GSH, S-Lactoylglutathione, (5-L-Glutamyl)-L-glutamate, S-Glutathionyl-L-cysteine, and L-cysteine were found to be elevated in the VPA-treated group when compared to the Ctrl group. This suggests that the GSH metabolic process may be enhanced by VPA treatment, which could also facilitate the generation of NADPH due to the interaction between NADPH metabolism and GSH metabolism 52, 53. Nevertheless, no metabolites that are directly associated with NADPH-related fluxes were identified among the differential metabolites. In addition, the relationship between the mass-to-charge ratio and the P value is illustrated in Figure 5C, while the interactions among the differential metabolites and key metabolic pathways are depicted in a network plot (Figure 5D). Our results implied that VPA treatment could influence Glutathione metabolism, Glucagon signaling pathway and Cysteine and methionine metabolism, which were closely associated with programmed cell death. Additionally, the regulation of ferroptosis was found to be associated with VPA treatment in the metabolomics analysis (Figure 5E).

### VPA treatment suppresses the susceptibility of HCC cells to disulfidptosis

**Figure 6:**
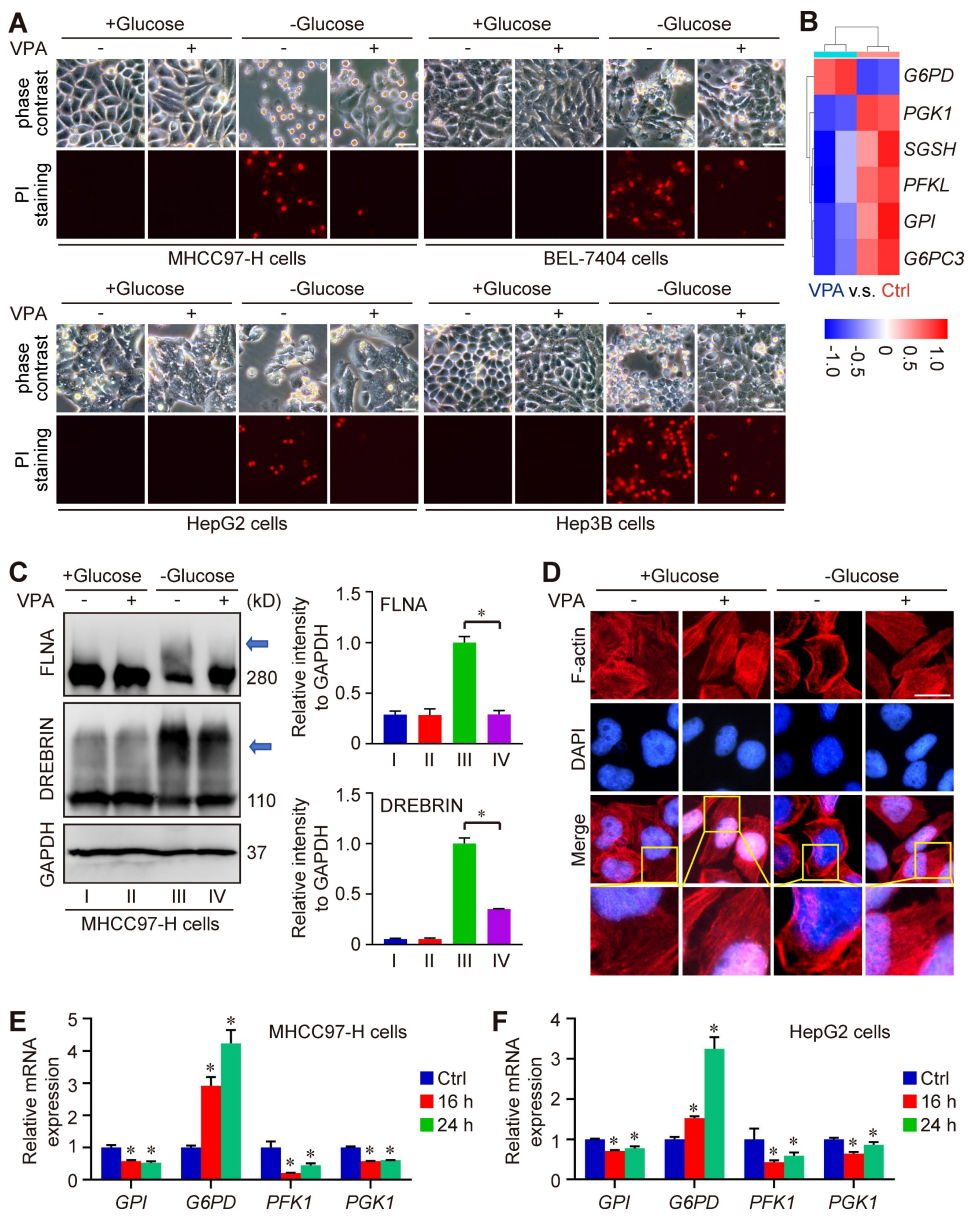
** VPA suppresses the sensitivity of HCC cells to disulfidptosis.** Herein, MHCC97-H, Hep3B, HepG2 and BEL-7404 cells were initially subjected to treatment with VPA (2 mM) for a duration of 24 h, followed by glucose deprivation for 8 to 12 h to induce disulfidptosis. The subsequent changes in cell morphology and cell death were assessed across each experimental group (A, Scale bar = 20 μm). A heatmap was generated to represent the expression of genes associated with glucose and energy metabolism as derived from RNA-seq data (B). Furthermore, the formation of disulfide bonds in cytoskeletal proteins, specifically FLNA and DREBRIN, induced by glucose starvation was analyzed through non-reducing western blotting (C). The expression level of Group III was considered as “1”. Additionally, phalloidin staining was utilized to examine the presence of actin filaments (F-actin) in the various groups (D, Scale bar = 10 μm). The transcription levels of several critical enzymes involved in glycolysis and the pentose phosphate pathway (PPP) were further assessed using qPCR (E-F). Data are expressed as mean ± SD. The P value less than 0.05 was considered statistically significant and the value of Cohen's d over 0.8 represents a large effect size. *: P < 0.05 and Cohen's d > 0.8 compared to Ctrl group or compared between different groups.

Metabolomics analysis indicated the important position of glutathione metabolism underlying pharmacological function of VPA. Notably, our data indicated that VPA treatment increased the level of SLC7A11, an essential element of system Xc^-^ and the upstream of glutathione metabolism (Figure 1E). Recent reports have revealed that both SLC7A11 and glutathione metabolism are pivotal in the modulation of disulfidptosis. Consequently, we proceeded to examine the effect of VPA on disulfidptosis in HCC cells. In our investigation, four HCC cell lines (MHCC97-H, BEL-7404, HepG2 and Hep3B) were initially subjected to treatment with VPA for a duration of 24 h, followed by a period of glucose deprivation (10 to 12 h) to induce disulfidptosis. Different form other reports 20, 21, our findings revealed that the prior administration of VPA significantly decreased the susceptibility of HCC cells to disulfidptosis induced by glucose starvation (Figure 6A), even though the level of SLC7A11 could be upregulated after VPA treatment (Figure 1E). Our RNA-seq data also indicated that several key enzymes involved in glycolysis and the pentose phosphate pathway (PPP) were affected by VPA treatement, which could be the potential mechanism (Figure 6B). Furthermore, the formation of disulfide bonds in cytoskeletal proteins caused by glucose starvation was assessed using non-reducing western blotting, and the results indicated that both FLNA and DREBRIN displayed markedly slower migration patterns with smears, a phenomenon that was diminished by VPA pretreatment (Figure 6C). Additionally, phalloidin staining was utilized to analyze the organization of actin filaments (F-actin) across the various experimental groups. Our results showed that under normal conditions, F-actin was predominantly arranged in the cell cortex and stress fibers. In contrast, glucose starvation induced notable morphological alterations, which were characterized by F-actin contraction and marginal clustering. VPA pretreatment effectively rescued these morphological changes in F-actin, aligning with other observations related to disulfidptosis (Figure 6D). Therefore, VPA treatment suppresses the susceptibility of HCC cells to disulfidptosis. To confirm the conclusion, we next conducted time-course experiments to assess the impact of VPA on cytoskeletal disulfide bond formation. Herein, HCC cells were subjected to VPA treatment for durations of 8 h, 16 h, and 24 h, respectively. Subsequently, the cells were then further exposed to glucose starvation to induce disulfidptosis. The results showed that cell death induced by glucose starvation could be rescued by VPA treatment in a time-dependent manner (Figure S5A). Following this, the structure of F-actin was analyzed via phalloidin staining, and the results showed that F-actin contraction and marginal clustering induced by glucose starvation could also be suppressed by VPA treatment in a time-dependent manner (Figure S5B). Furthermore, some reports have demonstrated that reducing agents and endoplasmic reticulum stress activators are regarded as inhibitors of disulfidptosis 20, 54. Consequently, both the reducing agent DL-dithiothreitol (DTT) and the endoplasmic reticulum stress activator cinchonine (CCN) were utilized as positive controls to validate the function of VPA. As anticipated, our findings revealed that treatment with DTT and CCN significantly mitigated cell death induced by glucose deprivation (Figure S6A). The arrangement of F-actin across the different experimental groups was subsequently examined through phalloidin staining (Figure S6B). The findings demonstrated that both DTT and CCN treatments inhibited glucose starvation-induced F-actin contraction and marginal clustering in HCC cells, mirroring the effects observed with VPA treatment. Due to the role of VPA in HDAC suppression, the histone acetyltransferase p300/CBP inhibitor was employed to assess the significance of histone acetylation in VPA-induced disulfidptotic modulation. In this study, the p300/CBP inhibitor was administered alongside VPA, and the treated cells were subsequently subjected to glucose starvation condition. Our findings indicated that VPA conferred protection to HCC cells against glucose starvation-induced disulfidptosis, and the effect that was suppressed by the co-treatment with the p300/CBP inhibitor (Figure S7A). Furthermore, non-reducing western blot analysis revealed that VPA treatment rescued the slower migration of cytoskeletal proteins, whereas the p300/CBP inhibitor effectively negated the effects of VPA (Figure S7B-D). Additionally, phalloidin staining illustrated that VPA treatment inhibited F-actin contraction and marginal clustering in HCC cells, with this effect being obstructed by the p300/CBP inhibitor, thereby underscoring the critical role of histone acetylation in the pharmacological action of VPA (Figure S7E).

Furthermore, to reveal the underlying mechanism of VPA function, we explored the impact of VPA treatment on the transcription of genes associated with disulfidptosis (*GYS1*, *NDUFS1*, *OXSM*, *LRPPRC*, *NDUFA11*, *NUBPL*, *NCKAP1*, *RPN1*, *SLC3A2* and *SLC7A11*) as the report (20). The findings revealed that VPA treatment could activate the transcription of *NDUFA11* and suppress the transcription of *GYS1* modestly (Figure S8A-S8B), underscoring the limited influence of these genes in the regulatory effects of VPA on disulfidptosis. In addition, the strong link between disulfidptosis and glucose/energy metabolism has been validated through both *in vitro* and *in vivo* studies 20, 22, 23. Consequently, some related genes in RNA-seq data were analyzed. Especially, we conducted further investigations into the impact of VPA on the transcription of several key enzymes involved in glycolysis and the PPP, including Phosphofructokinase 1 (PFK1), Phosphoglycerate kinase 1 (PGK1), glucose-6-phosphate isomerase (GPI), and G6PD. Notably, our results indicated that VPA treatment obviously enhanced the transcription of *G6PD*, the rate-limiting enzyme in the oxidative PPP for NADPH production, in both MHCC97-H and HepG2 cells (Figure 6B, E-F), which could be essential mechanism underlying the function of VPA in disulfidptosis regulation.

### The activation of G6PD transcription is a crucial mechanism of VPA function on disulfidptosis

**Figure 7:**
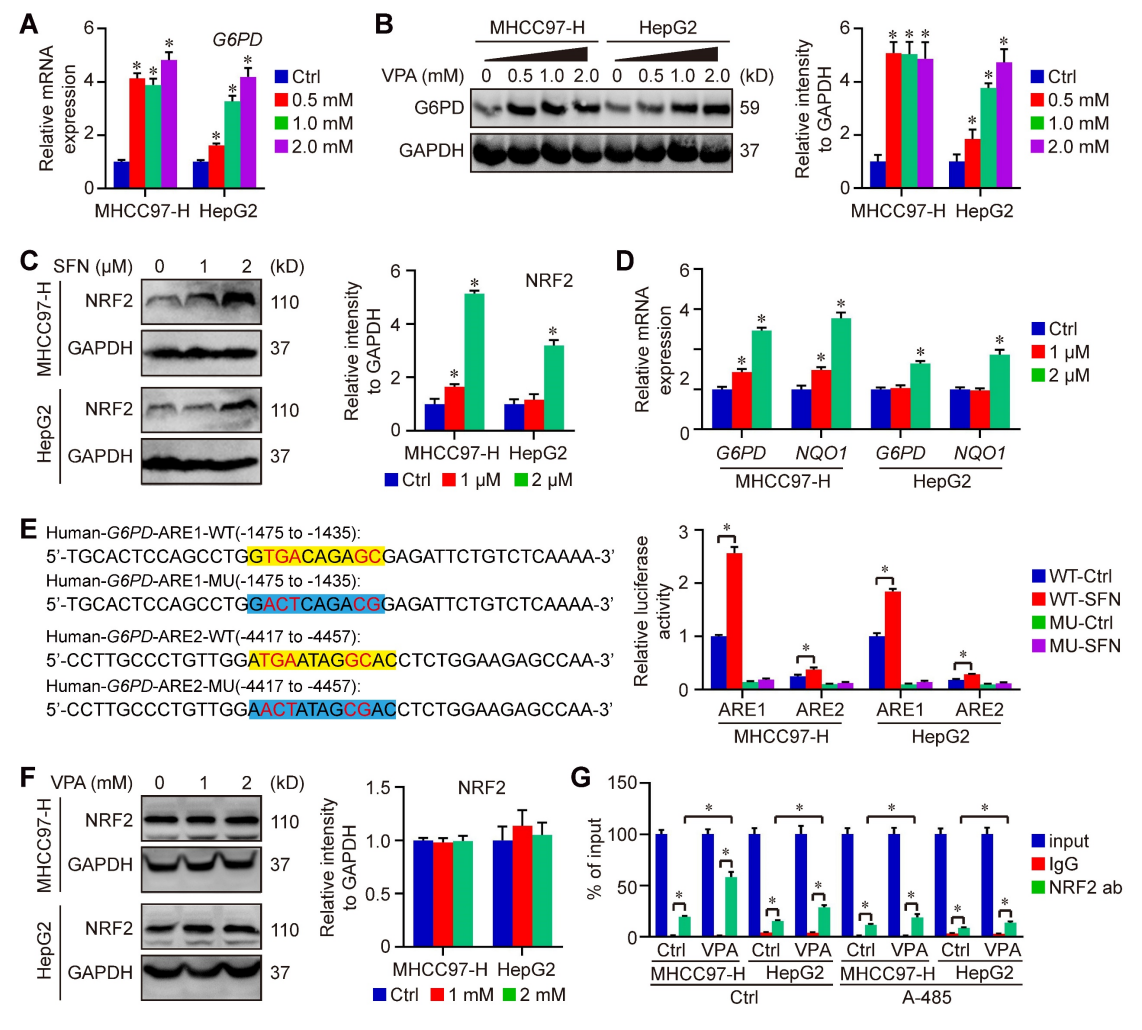
** VPA treatment promotes the activation of G6PD transcription in HCC cells.** Both MHCC97-H and HepG2 cells were subjected to VPA treatment for a duration of 24 h, then harvested for qPCR and western blot assay. The mRNA and protein levels of G6PD were measured in our work (A-B). In addition, HCC cells were treated with SFN (1 and 2 μM) for 16 h, and the protein level of NRF2 was determined in each group (C). The transcription of *G6PD* and another NRF2 target gene, *NQO1*, was accessed using qPCR (D). Moreover, the function of potential AREs in human *G6PD* promotor region was analyzed via luciferase assay. MHCC97-H and HepG2 cells transfected with ARE-firefly luciferase or TK-renilla luciferase vectors for 24 h were left untreated or treated with SFN (2 μM) for 16 h. The cells were harvested for dual luciferase assay (E). Finally, The influence of VPA treatment on NRF2 expression was further determined using immunoblot (F), and the endogenous interaction between NRF2 and ARE1 in human *G6PD* gene was validated through ChIP-qPCR (G). Data are expressed as mean ± SD. The P value less than 0.05 was considered statistically significant and the value of Cohen's d over 0.8 represents a large effect size. *: P < 0.05 and Cohen's d > 0.8 compared to Ctrl group or compared between different groups.

To further confirm the impact of VPA on* G6PD* expression, HCC cells were subjected to treatment with VPA in different dose, and the expression of G6PD were assessed through qPCR and western blot analysis. Our findings indicated that both mRNA and protein levels of G6PD were elevated in a dose-dependent manner following VPA treatment (Figure 7A-B). VPA has been recognized as an effective HDAC inhibitor, which typically promotes gene transcription through enhancing the acetylation of hyperacetylated histones and inhibiting the binding between histone and DNA 55, 56. Therefore, it's possible that VPA treatment increases the expression of* G6PD* by modulating gene transcription.

Additionally, some studies also reported that nuclear factor erythroid 2 (NFE2)-related factor 2 (NRF2), an important transcription factor that regulates cellular antioxidant responses, serves as the upstream of *G6PD* and regulates* G6PD* transcription 57, 58. Next, the effect of VPA on NRF2-G6PD axis was investigated in our work. Initially, NRF2 activator, Sulforaphane (SFN), was employed in our work to explore the connection between between NRF2 and G6PD in HCC cells. Our results indicated that the treatment with SFN obviously increased the protein level of NRF2 as well as the transcription of *G6PD* and other NRF2 target gene (*NQO1*) in both MHCC97-H and HepG2 cells (Figure 7C-D). NRF2 regulates the expression of its target genes containing antioxidant response elements (AREs) within their regulatory regions, so the potential ARE sequences in human *G6PD* promotor were predicted using JASPAR (https://jaspar.elixir.no/) 59. Computational analysis has revealed the presence of two potential AREs within the promoter region of *G6PD*. To confirm the validity of these two potential AREs, both wild type (WT) and mutated (MU) 41-bp sequences containing the AREs were cloned into the pGL4.22-luciferase vector. MHCC97-H and HepG2 cells were transfected with the various ARE-reporter plasmids and subsequently treated with SFN for 16 h before being harvested for luciferase assays. The results indicated that the relative luciferase activity was increased in response to SFN only in cells transfected with the reporter plasmids containing ARE1/2-WT, while no increase was observed with ARE1/2-MU. Furthermore, the activity of ARE1 was significantly higher than that of ARE2 (Figure 7E). The impact of VPA on NRF2 expression was also assessed, revealing that VPA treatment did not affect NRF2 level in HCC cells (Figure 7F). Additionally, the endogenous interaction of NRF2 with ARE1 of human *G6PD* was validated through ChIP-qPCR, and the results indicated that VPA treatment enhanced the binding of NRF2 to the ARE sequence. Additionally, the co-treatment with histone acetyltransferase p300/CBP inhibitor (A-485) limited the function of VPA, suggesting the importance of histone acetylation in the pharmacological fucntion of VPA (Figure 7G). In summary, these findings confirm the existence of two functional AREs in the promoter region of *G6PD*, and indicate that VPA treatment promotes *G6PD* transcription by facilitating the binding of the transcription factor to the promoter sequence.

**Figure 8:**
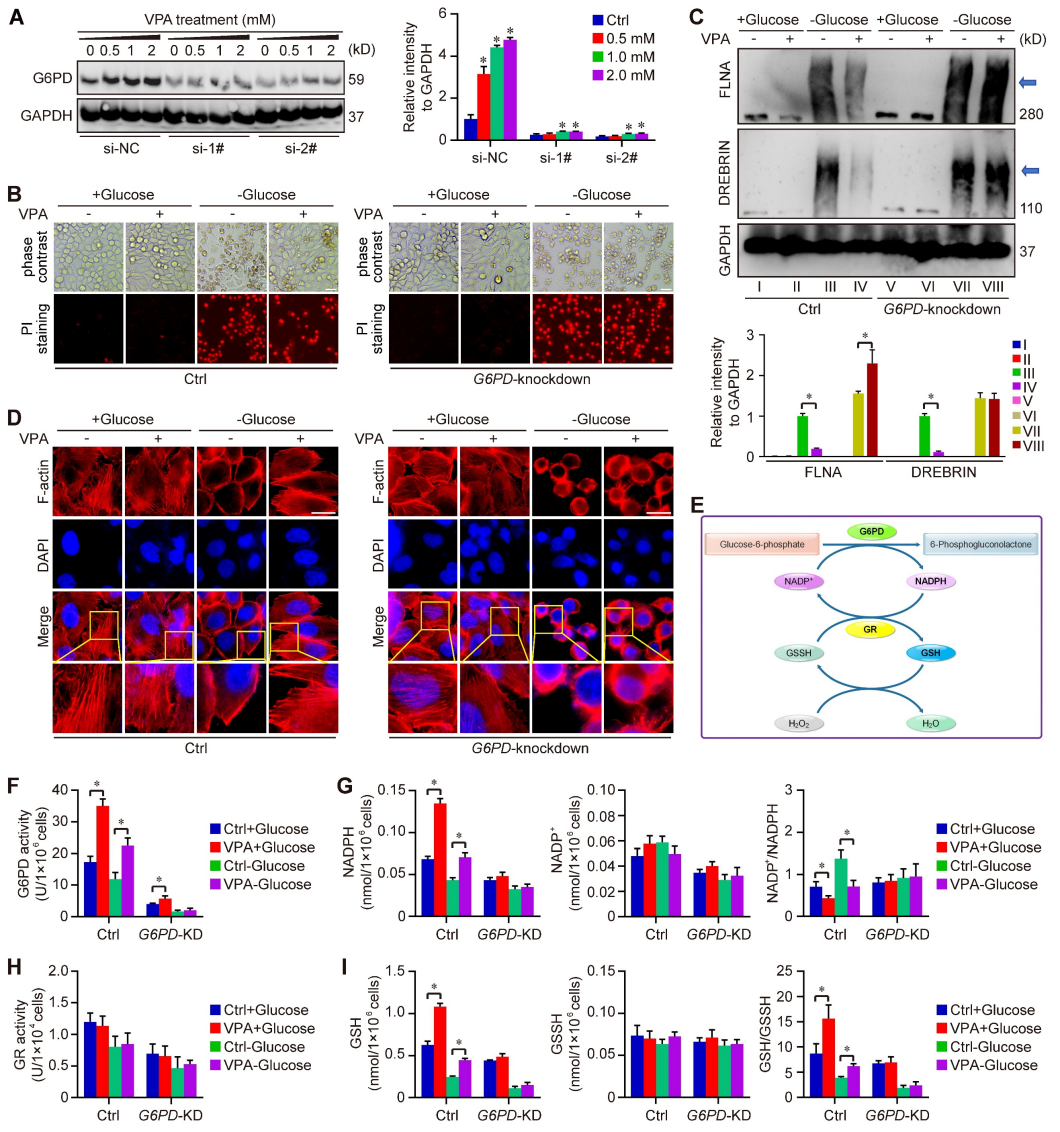
** G6PD plays a pivotal role in the action of VPA on disulfidptosis.** *G6PD* knockdown cells were generated through siRNA transfection, and the effectiveness of the knockdown model was assessed via western blot analysis. HCC cells (MHCC97-H) were exposed to varying concentrations of VPA for 24 h, and the protein levels of G6PD were measured to verify the knockdown effect (A), and si-2# was chosen for following assays. Furthermore, both wild type and *G6PD* knockdown HCC cells underwent treatment with VPA for 24 h, followed by glucose deprivation (10-12 h) to trigger disulfidptosis. The morphological changes and cell death were subsequently analyzed in each group (B, Scale bar = 20 μm). Additionally, the formation of disulfide bonds in cytoskeletal proteins (FLNA and DREBRIN) induced by glucose starvation was assessed using non-reducing western blot (C). The expression level of Group III was considered as “1”. Phalloidin staining was then utilized to examine the actin filament (F-actin) in the various groups (D, Scale bar = 10 μm). Given the significance of G6PD in the pentose phosphate pathway and NADPH production (E), the activity of G6PD (F) and NADP^+^-NADPH metabolism (G) were measured herein. Moreover, the activity of GR (H) and GSH-GSSH metabolism (I) were also investigated in this study. Data are expressed as mean ± SD. The P value less than 0.05 was considered statistically significant and the value of Cohen's d over 0.8 represents a large effect size. *: P < 0.05 and Cohen's d > 0.8 compared to Ctrl group or compared between different groups.

Furthermore, our study utilized two distinct G6PD chemical inhibitors, 6-Aminonicotinamide and Polydatin, to further elucidate the role of G6PD in the pharmacological effects of VPA concerning disulfidptosis regulation. The findings demonstrated that the protective effect of VPA against disulfidptosis was diminished when co-treated with G6PD inhibitors (Figure S9). Additionally, a* G6PD* knockdown model was created through siRNA transfection techniques (Figure 8A). The impact of VPA on glucose starvation-induced disulfidptosis was assessed in both wild type and *G6PD* knockdown HCC cells. Our results indicated that VPA decreased the susceptibility of HCC cells to disulfidptosis, a protective effect that was negated by *G6PD* knockdown (Figure 8B). Moreover, non-reducing western blot analysis revealed that VPA treatment inhibited the slower migration of cytoskeletal proteins, while *G6PD* knockdown effectively countered the action of VPA (Figure 8C). The arrangement of F-actin in various groups was further examined through phalloidin staining, revealing that VPA treatment prevented glucose starvation-induced F-actin contraction and marginal clustering in wild type HCC cells, and the effect was not observed in *G6PD* knockdown HCC cells (Figure 8D). These results indicate that G6PD plays a pivotal role in mediating the effects of VPA on disulfidptosis regulation.

As the rate-limiting enzyme in the oxidative PPP responsible for NADPH production, G6PD primarily facilitates the conversion of glucose-6-phosphate to 6-phosphogluconolactone. The synthesis of glutathione (GSH) is intricately linked to the activity of G6PD and the production of NADPH. Our metabolomics analysis indicated that GSH levels were influenced by treatment with VPA (Figure 5B). Consequently, we assessed the impact of VPA on NADP^+^-NADPH metabolism and GSH synthesis using both wild type and *G6PD* knockdown HCC (Figure 8E). Our findings revealed that VPA treatment enhanced G6PD activity, leading to an increase in NADPH levels and a decrease in the NADP^+^/NADPH ratio in wild type cells, a response that was not observed in *G6PD* knockdown HCC cells (Figure 8F-G). Notably, VPA did not alter the activity of glutathione reductase (GR) in either wild type or *G6PD* knockdown HCC cells (Figure 8H). Additionally, VPA treatment resulted in elevated GSH levels and an increased GSH/GSSH ratio in wild type cells, an effect that was inhibited in *G6PD* knockdown cells (Figure 8I). In summary, VPA enhances the sensitivity of HCC cells to disulfidptosis by modulating G6PD function, with the maintenance of GSH synthesis and NADPH production serving as critical underlying metabolic mechanisms.

Furthermore, *NRF2* knockout cell line was established using CRISPR/Cas9 as our previous work 39. The influence of VPA on disulfidptosis was evaluated in both wild type and *NRF2* knockout HCC cells. Our previous study has revealed that *NRF2* knockout rescued disulfidptosis via regulating SLC7A11 39. Herein, our findings indicated that VPA reduced the sensitivity of wild type HCC cells to disulfidptosis, and *NRF2* knockout also showed an obvious protective effect. Therefore, there were no difference beween VPA treated and untreated *NRF2* knockout cells in disulfidptosis model (Figure S10A-B). Additionally, the knockout of* NRF2* resulted in a significant downregulation of G6PD in HCC cells (Figure S10C). The non-reducing western blot analysis indicated that VPA treatment inhibited the slower migration of cytoskeletal proteins, whereas *NRF2* knockout effectively counteracted the effects of VPA (Figure S10D). The organization of F-actin across different groups was further analyzed using phalloidin staining, which showed that VPA treatment suppressed glucose starvation-induced F-actin contraction and marginal clustering in wild type HCC cells, an effect that was not observed in *NRF2* knockout HCC cells (Figure S10E-F). These findings suggest that NRF2-GPX6 pathway is crucial in mediating the effects of VPA on disulfidptosis.

## Discussion

One of the most important characteristics of cancer is chemotherapy resistance. According to recent reports, cellular metabolism imbalance leads to distinct types of programmed cell death which are different form traditional apoptosis, such as ferroptosis, lysozincrosis, alkaliptosis, cuproptosis, and disulfidptosis. All of the cell death types provide novel insights into innovative therapeutic strategies for cancer treatment 24, 60. However, the close association can be established among different cell death types based on the intricacy of the metabolic pathways. For instance, the cystine/glutamate antiporter SLC7A11 holds an important role in ferroptosis regulation, and the overexpression of SLC7A11 enhances tumor growth and promotes cancer escape from ferroptosis via regulating GSH production and lipid peroxidation. However, the high expression of SLC7A11 promotes the transport of cysteine and the generation of intercellular cysteine, which exhausts NADPH and increases disulfide stress, and SLC7A11 is identified as a disulfidptosis enhancer under the condition of glucose starvation 21, 24. Taken together, it's reasonable that the same factor plays opposite roles in different cell death models. In this study, our findings mainly revealed the contrasting regulatory effects of VPA on ferroptosis and disulfidptosis of HCC. We observed that the treatment with VPA enhanced the susceptibility of HCC to ferroptosis via contributing to the labile iron pool, in which VPA promotes accumulation of free iron through promoting cell autophagy and suppressing the expression of ferritin. Differently, VPA treatment protected cells from glucose starvation-induced disulfidptosis. Omics assay indicated that VPA activated the transcription of *G6PD* gene together with GSH metabolism, and the production efficiency of NADPH and GSH was increased in VPA-treated cells compared with VPA-untreated cells. Taken together, VPA regulates ferroptosis and disulfidptosis through different signaling pathway, offering a novel insight into the precise cancer treatment using VPA. Nonetheless, as a functional HDAC inhibitor, VPA can regulate the transcription of various genes through the modulation of histone acetylation. It is conceivable that additional genes associated with iron metabolism or glucose metabolism may also play a role in the pharmacological effects of VPA, contributing to the opposing regulatory impacts of VPA on ferroptosis and disulfidptosis. Consequently, it is essential to consider the metabolic and epigenetic crosstalk when analyzing the pharmacological metabolism of VPA across different cell death models.

As a well-tolerated anticonvulsive drug, VPA has shown multiple therapeutic action against different diseases. In cancer treatment, VPA holds great potential in the combining treatment with other conventional chemotherapeutic agents. Recent research also indicated the potential of VAP in the regulation of ferroptosis. However, the detailed mechanism still needs further investigation. In our study, we noticed that the treatment with VPA enhanced cell autophagy and promoted autophagic flux. Some studies have revealed that ferritinophagy, a process involving the degradation of ferritin and iron in autophagosomes, could be regulated by autophagy, and enhanced autophagy contributes to ferroptosis process via promoting degradation of ferritin. Consistently, the protein level of FTL was decreased in HCC after VPA treatment, which was accompanied with increased free iron. Meanwhile, our RNA-seq data and qPCR results suggested that VPA treatment suppressed the transcription of *FTH1* and *FTL* as well. Currently, histone acetylation is regarded as an important modification which influences the transcription of various functional genes. HDAC is able to remove acetylation form hyperacetylated histone and strengthen the binding between histone and DNA. Therefore, as an effective HDAC inhibitor, VPA usually activates the process of gene transcription. The accurate regulatory mechanisms about the regulation of VPA on *FTH1* and *FTL* transcription remain to be fully elucidated currently. Meanwhile, the role of VPA in ferroptosis and disulfidptosis was further compared with other HDAC inhibitors, specifically vorinostat (SAHA) and trichostatin A (TSA). The findings revealed that both SAHA and TSA treatments increased the sensitivity of HCC cells to ferroptosis inducers (Figure S11A). However, these treatments did not influence disulfidptosis induced by glucose starvation (Figure S11B). The protein levels of G6PD and FTL were subsequently assessed in HCC cells treated with varying concentrations of SAHA and TSA. The results indicated that FTL expression remained unaffected by either SAHA or TSA treatment. In contrast, the level of G6PD was elevated following TSA treatment and showed a slight decrease with SAHA treatment in HepG2 cells, while G6PD expression was not impacted by these HDAC inhibitors in MHCC97-H cells (Figure S11C). Consequently, it is plausible that different HDAC inhibitors modulate ferroptosis and disulfidptosis via distinct signaling pathways, even though other HDAC inhibitors apart from VPA may influence the sensitivity of HCC cells to ferroptosis or disulfidptosis. The precise mechanisms underlying the effects of various HDAC inhibitors on cell death necessitate further exploration.

In addition, besides ferroptosis and disulfidptosis, cuproptosis was also separate from existing other kinds of cell death, which is a copper-dependent programmed cell death and characterized by accumulation of intracellular copper and disturbed copper homeostasis 61. The expression of key genes (*FDX1*, *ATP7B* and *SLC31A1*) in cuproptosis regulation was also analyzed in VPA-treated cells. Our qPCR results indicated that the transcription of all those genes were upregulated by VPA treatment (Figure S12A-B), suggesting the potential of VPA in cuproptosis regulation. Besides, some reports showed the close association between epithelial mesenchymal transition (EMT) and different programed cell death 62, 63. In our work, we also observed that the morphology of VPA-treated HCC cells was different from untreated cells. Therefore, epithelial makers (*EрCAM*, *CDH1* and *KRT8*) and mesenchymal makers (*CDH2*, *SNAI1* and *VIM*) were measured in current work. However, the results indicated that the transcription of both epithelial makers (*EрCAM* and *CDH1*) and mesenchymal makers (*CDH2* and *SNAI1*) were upregulated in VPA-treated cells (Figure S13A-S13B). The role of VPA in the regulation of EMT remains ambiguous at present 64-66. It is conceivable that VPA influences EMT-related genes in diverse ways due to the tissue specificity of genetic regulation across various tissues. The precise mechanisms by which VPA regulates EMT have yet to be thoroughly clarified. Furthermore, we have noticed the limitations associated with VPA treatment. For instance, the most common general side effects of VPA therapy include vomiting, heartburn, and nausea. Approximately 10% of individuals in the population report experiencing dermatological side effects such as alopecia or rash, dose-dependent tremors as well as neurological effects like ataxia, drowsiness, and irritability 67, 68. As a potent HDAC inhibitor, VPA treatment can regulate the transcription of various genes in different organs. Consequently, the potential off-target effects of VPA may significantly contribute to the diverse side effects and toxicity, which in turn restricts VPA-related precise clinical treatment. Greater efforts should be directed towards identifying the key targets of VPA in various tissues, and optimal dosages must also be evaluated both i*n vitro* and* in vivo*, enabling us to establish a precise approach for treating different diseases in clinical settings.

G6PD is considered as the key rate-limiting enzyme in the pentose phosphate pathway, a glucose-oxidizing pathway for ribose 5-phosphate and NADPH production. The significance and function of G6PD in physiology and pathophysiology have been well studied for years, because of the important position of NADPH in various cellular systems, such as antioxidant pathways as well as nitric oxide synthase. The pathophysiologic roles for G6PD have been confirmed in the progression of multiple diseases (e.g. diabetes, cancer, and virus infection). For example, G6PD activity is increased by carbohydrate-rich diet and insulin, and the suppression of G6PD relieves high fat diet-induced weight gain as well as the related metabolic reprogramming in liver and visceral fat, suggesting that G6PD acts as a functional regulator of high fat diet -induced obesity and fatty liver 69. Additionally, G6PD also holds antioxidant properties and decreases cellular oxidative stress. The upregulation of G6PD shows potential benefits in the treatment of neurodegenerative disease 70. In this work, we observed that the treatment with VPA directly activated the transcription of G6PD and improve the protein level of G6PD, together with the production of NAPDH and GSH which played an important position in disulfidptosis. The knockdown of *G6PD* blocked the protective function of VPA against disulfidptosis, indicating that the anti-disulfidptosis effect of VPA was dependent of G6PD regulation. However, some researchers reported that G6PD was associated with ferroptosis as well, and G6PD suppressed ferroptosis through regulating cytochrome P450 oxidoreductase that was usually downregulated in HCC and significantly correlated with the prognosis. G6PD deficiency suppressed tumor growth and metastasis *in vivo* 71, 72. Differently, our current work showed opposite results, in which VPA heightened the susceptibility of HCC to ferroptosis even though the transcription of *G6PD* was activated effectively by VPA treatment. It's possible that the labile iron pool and ferritin degradation hold more important position compared with activated G6PD in our VPA-related ferroptosis model, which still needs more convincing evidence.

SLC7A11 promotes the uptake of cystine and the production of GSH and is considered as an important regulator to detoxify lipid peroxidation and ferroptosis. In our study, we noticed the treatment with VPA increased the protein level of SLC7A11, while the RNA-seq and qPCR data suggested that VPA treatment affect the transcription of *SLC7A11* modestly. The upregulated SLC7A11 could contribute to GSH production in VPA-treated cells, which should suppress ferroptosis in theory. However, it seems that VPA-enhanced the labile iron pool played a more critical role compared with GSH production, leading to the promoting effect of VPA on ferroptosis. Therefore, it's possible that SLC7A11 inhibitor could further strengthen the effect of VPA on ferroptosis regulation. On the other hand, the VPA-induced SLC7A11 expression didn't enhance the susceptibility of HCC to disulfidptosis, which was inconsistent with other reports. Therefore, the complex mechanism still need further investigation in VPA-based treatment in clinical settings.

Currently, the fundamental mechanism related to disulfidptosis requires additional exploration, and the exact sequence of molecular events that connect disulfide stress to cytoskeletal collapse is not clearly defined. Notably, the contradictory reliance on SLC7A11 overexpression also lacks a mechanistic explanation in different cell death models. Furthermore, disulfidptosis demonstrates significant metabolic flexibility. Its initiation necessitates glucose deprivation but shows inconsistent manifestation across various cell types under similar nutrient stress 39, 73. The specific quantitative thresholds for NADPH depletion that are required to initiate pathological disulfide bonding remain unclear, and the interactions with glycolysis/TCA cycle intermediates have yet to be mapped. Additionally, while our current research has validated the influence of the NRF2-G6PD axis on disulfidptosis *in vitro*, more convincing *in vivo* models are essential to substantiate this conclusion. Nevertheless, both *NRF2* knockout and *G6PD* knockout HCC cell lines can't form tumor in xenograft model due to the essential roles of NRF2 and G6PD in cell grow and metabolism. In addition, the available *in vivo* models for disulfidptosis are limited, and there is a lack of specific small-molecule inducers or inhibitors for disulfidptosis, which poses challenges to the clinical relevance of our current findings as well as those of other researchers in the field of disulfidptosis. The absence of biomarkers that correlate disulfidptotic potential with tumor stage and therapy resistance should also be considered in future preclinical and clinical studies.

## Conclusion

In summary, our research primarily exhibited that VPA, a widely used medication, increased the susceptibility of HCC cells to ferroptosis by enhancing the labile iron pool, in which VPA facilitated the accumulation of free iron through the promotion of cellular ferritinophagy and the inhibition of ferritin expression. Additionally, VPA stimulated the transcription of the *G6PD* gene and influenced GSH metabolism. The activation of the NRF2-G6PD pathway further enhanced the production of NADPH and GSH, which in turn suppressed the formation of disulfide bonds among various cytoskeletal proteins, as well as disulfidptosis in HCC cells. Collectively, our findings indicate that VPA modulates ferroptosis and disulfidptosis via distinct signaling pathways, providing new insights into targeted cancer therapies utilizing VPA. Furthermore, pharmacological intervention targeting the NRF2-G6PD signaling pathway may represent a promising strategy for the treatment of disulfidptosis-related diseases in clinical settings.

## Supplementary Material

Supplementary figures and tables.

## Abbreviations

VPA: valproic acid
HCC: hepatocellular carcinoma
G6PD: glucose-6-phosphate dehydrogenase
GSH: glutathione
ROS: reactive oxygen species
GPX4: glutathione peroxidase 4
FSP1: ferroptosis suppressor protein 1
NRF2: nuclear factor erythroid 2-related factor 2
DHODH: dihydroorotate dehydrogenase
FTH: ferritin heavy chain
FTL: ferritin light chain
SLC7A11: solute carrier family 7 member 11
NADPH: nicotinamide adenine dinucleotide phosphate
TrxR1: thioredoxin reductase 1
ChIP: chromatin immunoprecipitation assay
MDA: malondialdehyde
DFS: deferasirox
DFOM: deferoxamine mesylate
PPP: pentose phosphate pathway
PFK1: phosphofructokinase 1
PGK1: phosphoglycerate kinase 1
GPI: glucose-6-phosphate isomerase
ARE: antioxidant response elements
GR: glutathione reductase
SAHA: vorinostat
TSA: trichostatin A
DTT: DL-dithiothreitol
CCN: cinchonine
