# Supplementary material for: The contrasting regulatory effects of valproic acid on ferroptosis and disulfidptosis in hepatocellular carcinoma
Source: Theranostics. 2025 Aug 16;15(17):9091–113. doi: 10.7150/thno.115661 (PMC12439337; doi:10.7150/thno.115661)
Supplement: Supplementary file 1 — Supplementary figures and tables. [file thnov15p9091s1.pdf]

Supplementary Materials for

The contrasting regulatory effects of valproic acid on ferroptosis and disulfidptosis in hepatocellular carcinoma

Rongrong Liu [1,2,3], Xinyan Li [2], Jiayi Xu [2], Liangwen Yan [2], Kailing Hu [2], Mengjiao Shi [2,3], Yinggang Zhang [2,3], Yaping Zhao [2,3], Yudan Fan [2], Gang Wang [1,4], Ying Guo [1,2,3]*, Yetong Feng [5]*, Pengfei Liu [2,3,6]*

1. Department of Critical Care Medicine, National & Local Joint Engineering Research Center of Biodiagnosis and Biotherapy, The Second Affiliated Hospital of Xi’an Jiaotong University, Xi’an, China
2. International Joint Research Center on Cell Stress and Disease Diagnosis and Therapy, National & Local Joint Engineering Research Center of Biodiagnosis and Biotherapy, The Second Affiliated Hospital of Xi’an Jiaotong University, Xi’an, China
3. Shaanxi Provincial Clinical Research Center for Hepatic & Splenic Diseases, The Second Affiliated Hospital of Xi’an Jiaotong University, Xi’an, China
4. Key Laboratory of Surgical Critical Care and Life Support, Xi'an Jiaotong University, Ministry of Education of China, Xi'an, China
5. Core Research Laboratory, The Second Affiliated Hospital of Xi'an Jiaotong University, Xi'an, China
6. Key Laboratory of Environment and Genes Related To Diseases, Xi'an Jiaotong University, Ministry of Education of China, Xi'an, China.

* Corresponding author:
Pengfei Liu, E-mail address: liupengfei@xjtu.edu.cn
Yetong Feng, E-mail address: fengyetong@xjtu.edu.cn
Ying Guo, E-mail address: guoying.2yuan@xjtu.edu.cn

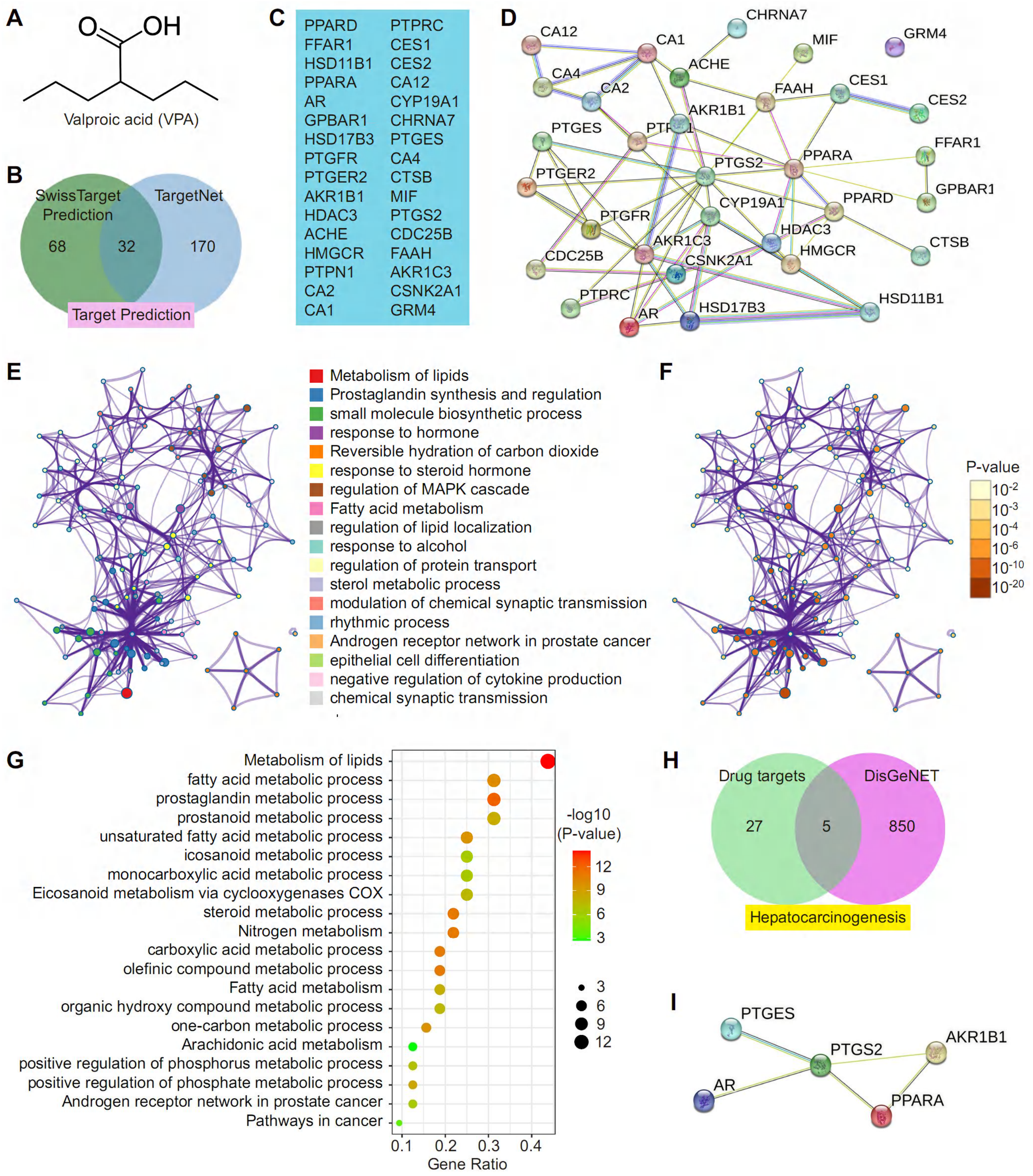

Figure S1. Network pharmacology assay of the potential bioavailable targets and therapeutic effects of VPA. Initially, the chemical structure of VPA is presented in Figure S1A. The potential bioavailable targets of VPA were identified using the SwissTargetPrediction and TargetNet databases, as shown in Figure S1B. The overlapping results (C) from these two databases were utilized to construct a protein-protein interaction network, depicted in Figure S1D. Furthermore, the overlapping targets were subjected to

cluster analysis via Metascape, with the top 20 enriched terms represented in a network plot (E-F). The items pertaining to metabolic processes are summarized in Figure S1G. Finally, the potential bioavailable targets of VPA related to hepatocarcinogenesis are compiled in Figure S1H-I.

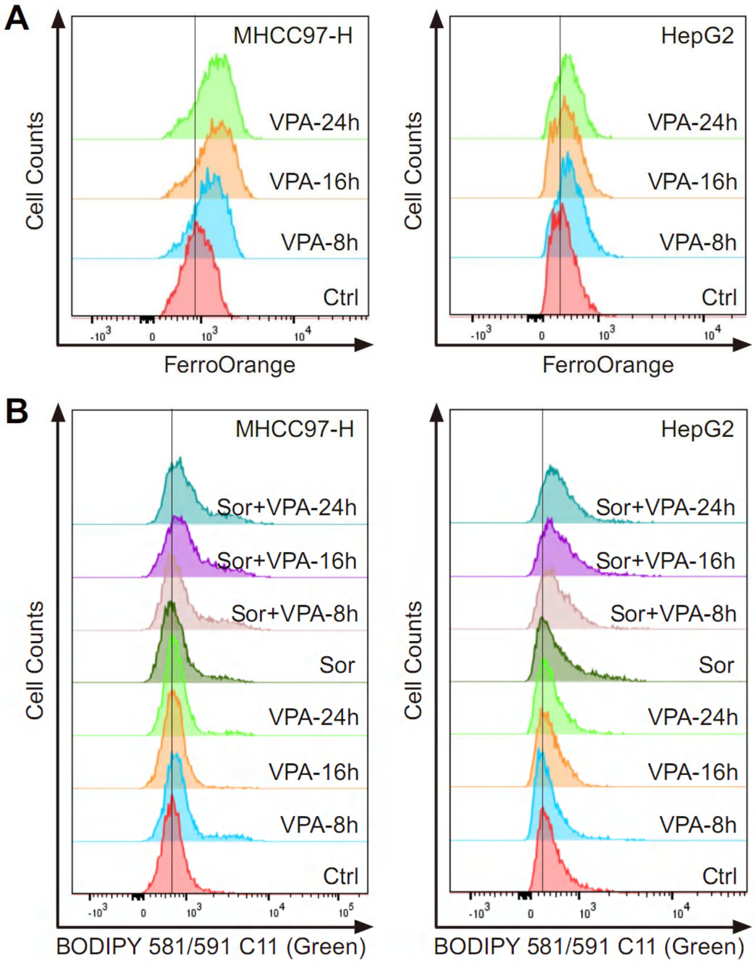

Figure S2. Time-course assay of VPA action on ferroptosis. Both MHCC97-H and HepG2 cells were subjected to VPA treatment (2 mM) for durations of 8 h, 16 h, and 24 h, respectively. Subsequently, the cell samples were collected for FerroOrange staining (A). The cells treated with VPA were then further exposed to Sorafenib (Sor, 1 μM). Following this, lipid peroxidation across different groups was quantified using BODIPY staining (B).

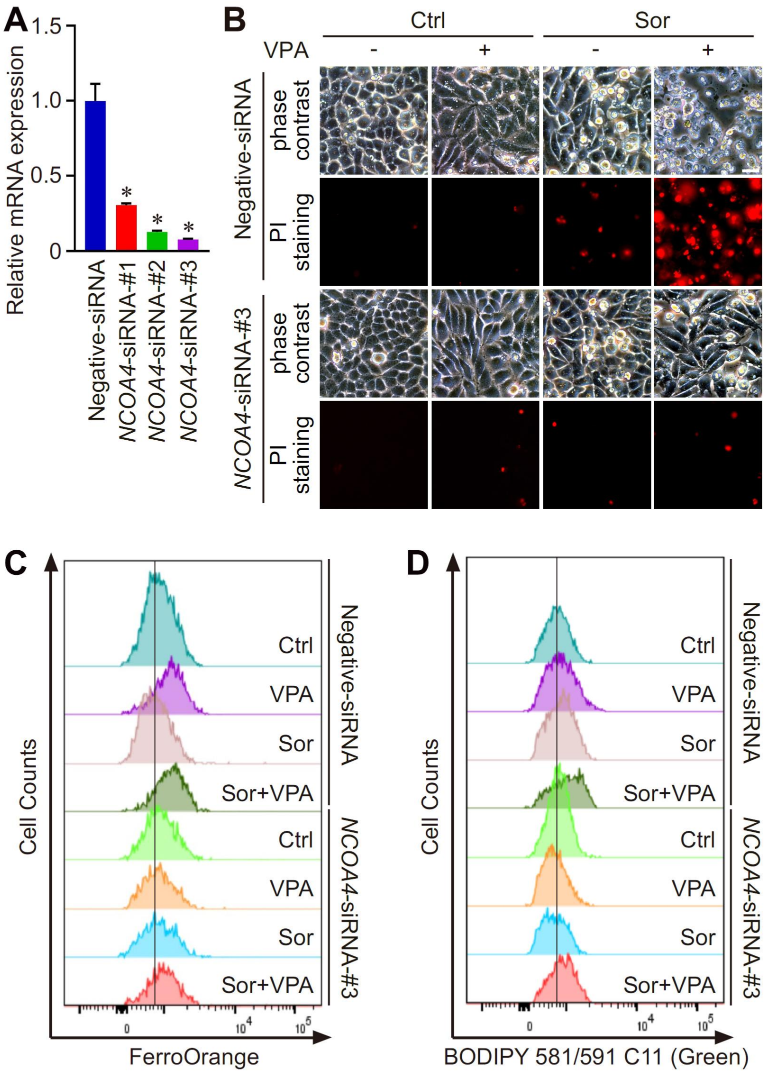

Figure S3. *NCOA4* knockdown suppresses VPA function on ferroptosis. Herein, *NCOA4* knockdown was created using the siRNA transfection method, and the effectiveness of the *NCOA4* knockdown was measured using qPCR (A). Then both wild-type and *NCOA4* knockdown cells were subjected to VPA treatment followed by Sorafenib (Sor, 1 μM) exposure. The subsequent changes in cell morphology and cell death were assessed across each experimental group (B, Scale bar = 20 μm). Additionally, the levels of free Fe^{2+} and lipid peroxidation were further assessed through FerroOrange staining (C) and BODIPY staining (D) across various groups. Data are expressed as mean ± SD. The P value less than 0.05 was considered statistically significant and the value of Cohen's d over 0.8 represents a large effect size. *: $P < 0.05$ and Cohen's d > 0.8 compared to Ctrl group.

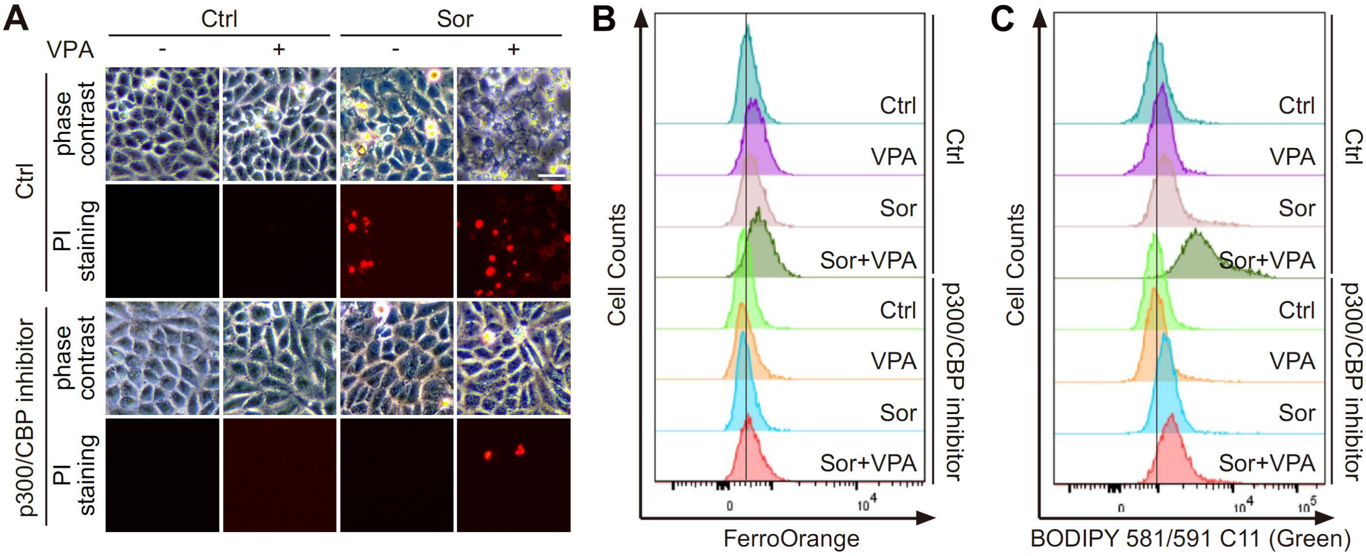

Figure S4. The position of histone acetylation in the pharmacological action of VPA on ferroptosis. Herein, MHCC97-H cells were treated with VPA (2 mM) and p300/CBP inhibitor (A-485, 1 μM), followed by Sorafenib (Sor, 1 μM) exposure. The subsequent changes in cell morphology and cell death were assessed across each experimental group (A, Scale bar = 20 μm). Additionally, the levels of free Fe^{2+} and lipid peroxidation were further assessed through FerroOrange staining (B) and BODIPY staining (C) across various groups.

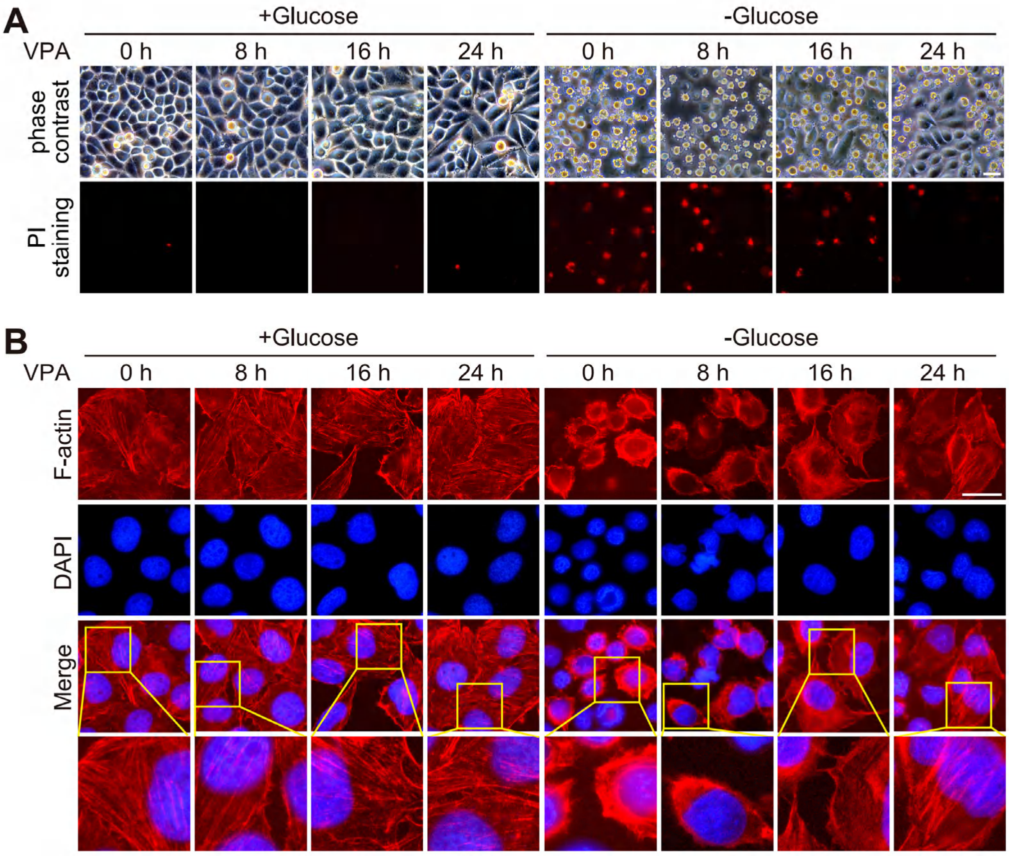

Figure S5. Time-course assay of VPA action on disulfidptosis. Herein, MHCC97-H cells were subjected to VPA treatment for durations of 8 h, 16 h, and 24 h, respectively. Subsequently, the cell samples were followed by glucose deprivation for 8 to 12 h to induce disulfidptosis. The subsequent changes in cell morphology and cell death were assessed across each experimental group (A, Scale bar = 20 μm). Furthermore, phalloidin staining was utilized to examine the presence of actin filaments (F-actin) in the various groups (B, Scale bar = 10 μm).

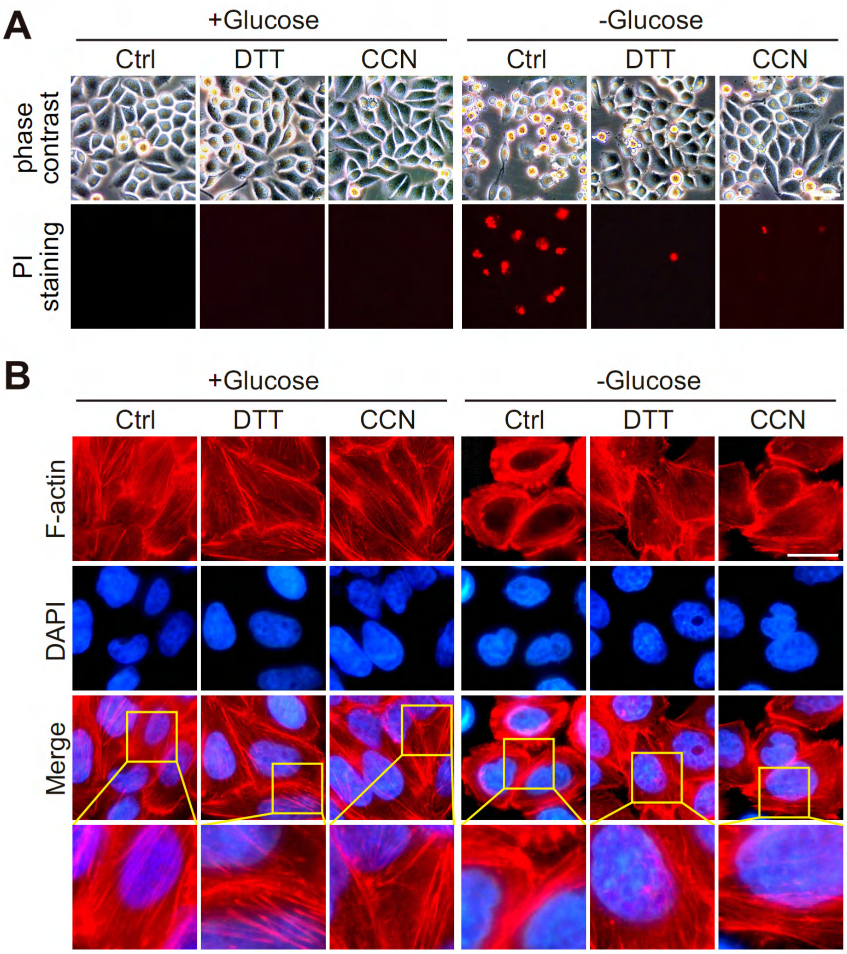

Figure S6. The impact of DTT and CCN on disulfidptosis. Both the reducing agent DL-dithiothreitol (DTT) and the endoplasmic reticulum stress activator cinchonine (CCN) were utilized as positive controls to validate the function of VPA. Herein, MHCC97-H cells were subjected to DTT (1 mM) and CCN (20 μM) treatment followed by glucose deprivation for 8 to 12 h to induce disulfidptosis. The subsequent changes in cell morphology and cell death were assessed across each experimental group (A, Scale bar = 20 μm). Furthermore, phalloidin staining was utilized to examine the presence of actin filaments (F-actin) in the various groups (B, Scale bar = 10 μm).

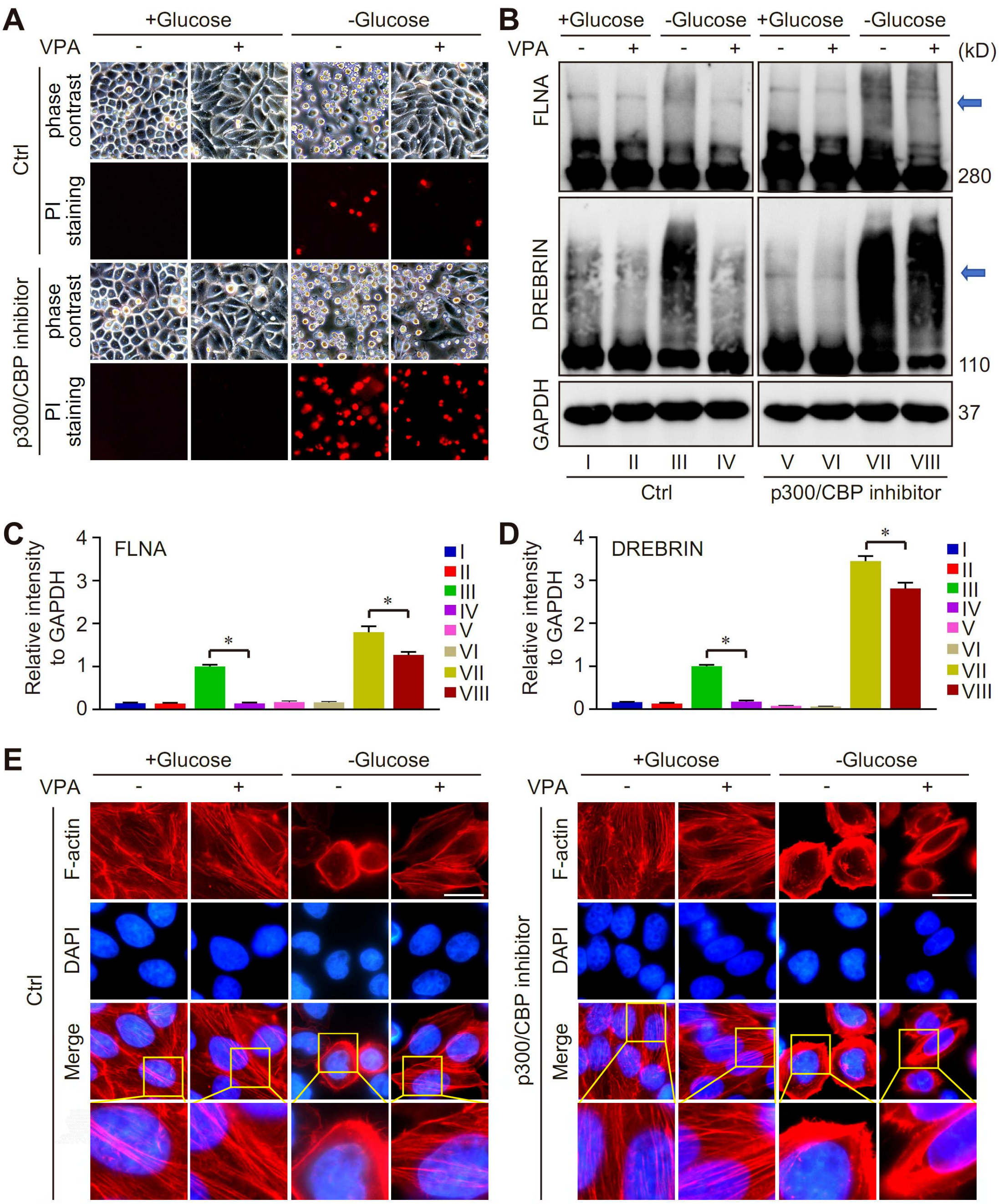

Figure S7. The position of histone acetylation in the pharmacological action of VPA on disulfidptosis. Herein, MHCC97-H cells were treated with VPA and p300/CBP inhibitor followed by glucose deprivation for 8 to 12 h to induce disulfidptosis. The subsequent changes in cell morphology and cell death were assessed across each experimental group (A, Scale bar = 20 μm). Furthermore, the formation of disulfide bonds in cytoskeletal proteins (FLNA and DREBRIN) induced by glucose starvation was assessed using non-reducing western blot (B). The quantified western blot results were shown in (C-D), and the expression level of Group III was considered as “1”. Phalloidin staining was then utilized to examine the actin filament (F-actin) in the various groups (E, Scale bar = 10 μm). Data are

expressed as mean ± SD. The P value less than 0.05 was considered statistically significant and the value of Cohen's d over 0.8 represents a large effect size. *: P < 0.05 and Cohen's d > 0.8 compared between different groups.

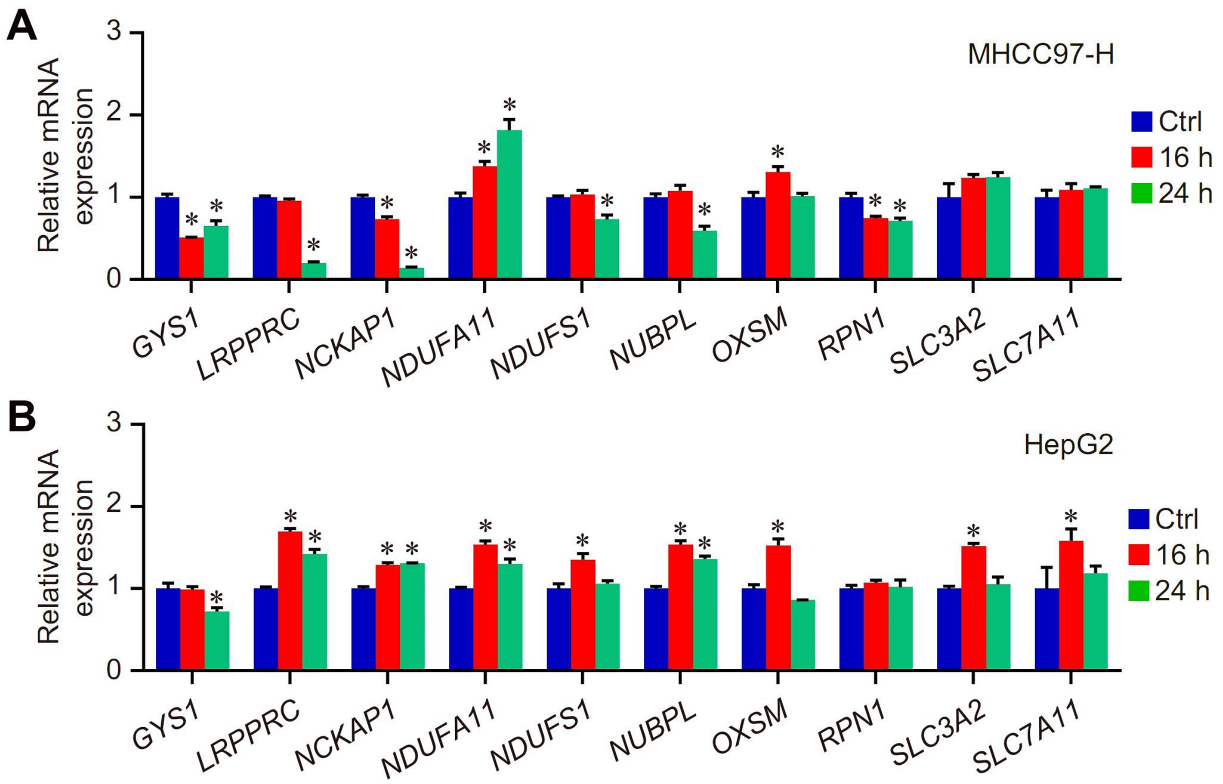

Figure S8. The effect of VPA treatment on transcription of disulfidptosis-related genes. Both MHCC97-H cells (A) and HepG2 cells (B) were treated with VPA (2 mM) for 16 h or 24 h, then the cells were harvested for qPCR measurement. (Data are expressed as mean ± SD. The P value less than 0.05 was considered statistically significant and the value of Cohen's d over 0.8 represents a large effect size. *: P < 0.05 and Cohen's d > 0.8 compared to Ctrl group.)

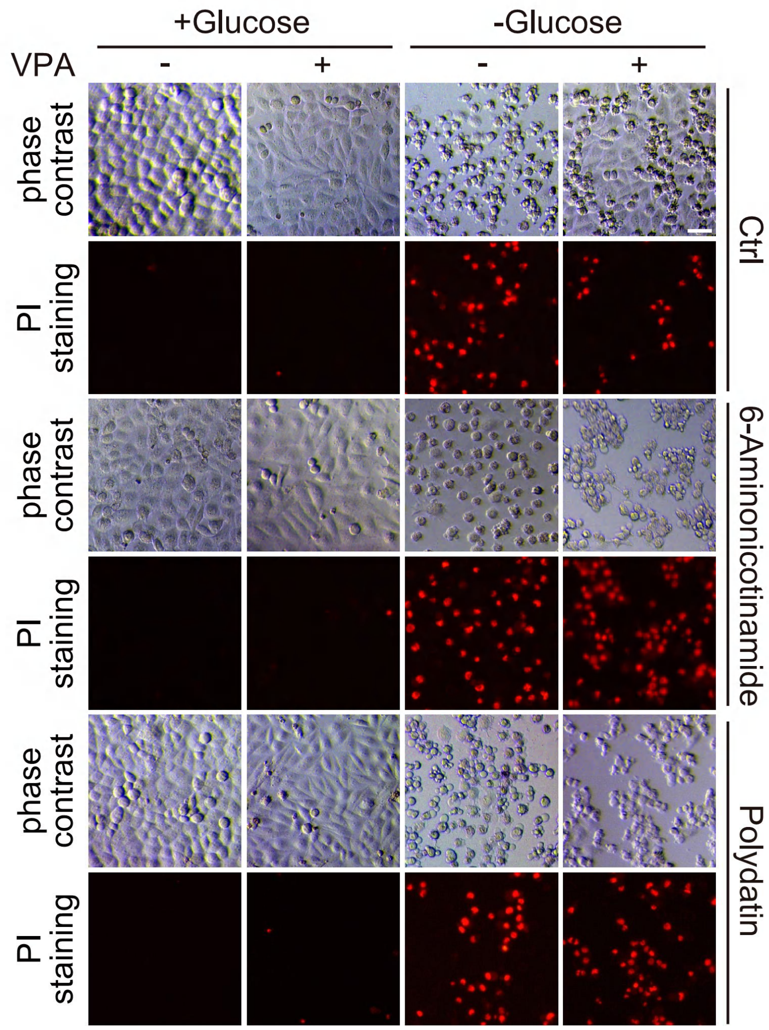

Figure S9. G6PD is essential for the action of VPA on disulfidptosis. Herein, two different G6PD inhibitors, 6-Aminonicotinamide and Polydatin, were used to further elucidate the role of G6PD in the pharmacological effects of VPA concerning disulfidptosis regulation. MHCC97-H cells were treated with VPA plus G6PD inhibitors (6-Aminonicotinamide (1 μM) and Polydatin (10 μM)), followed by glucose deprivation (10-12 h) to trigger disulfidptosis. Cell death was analyzed in each group via PI staining.

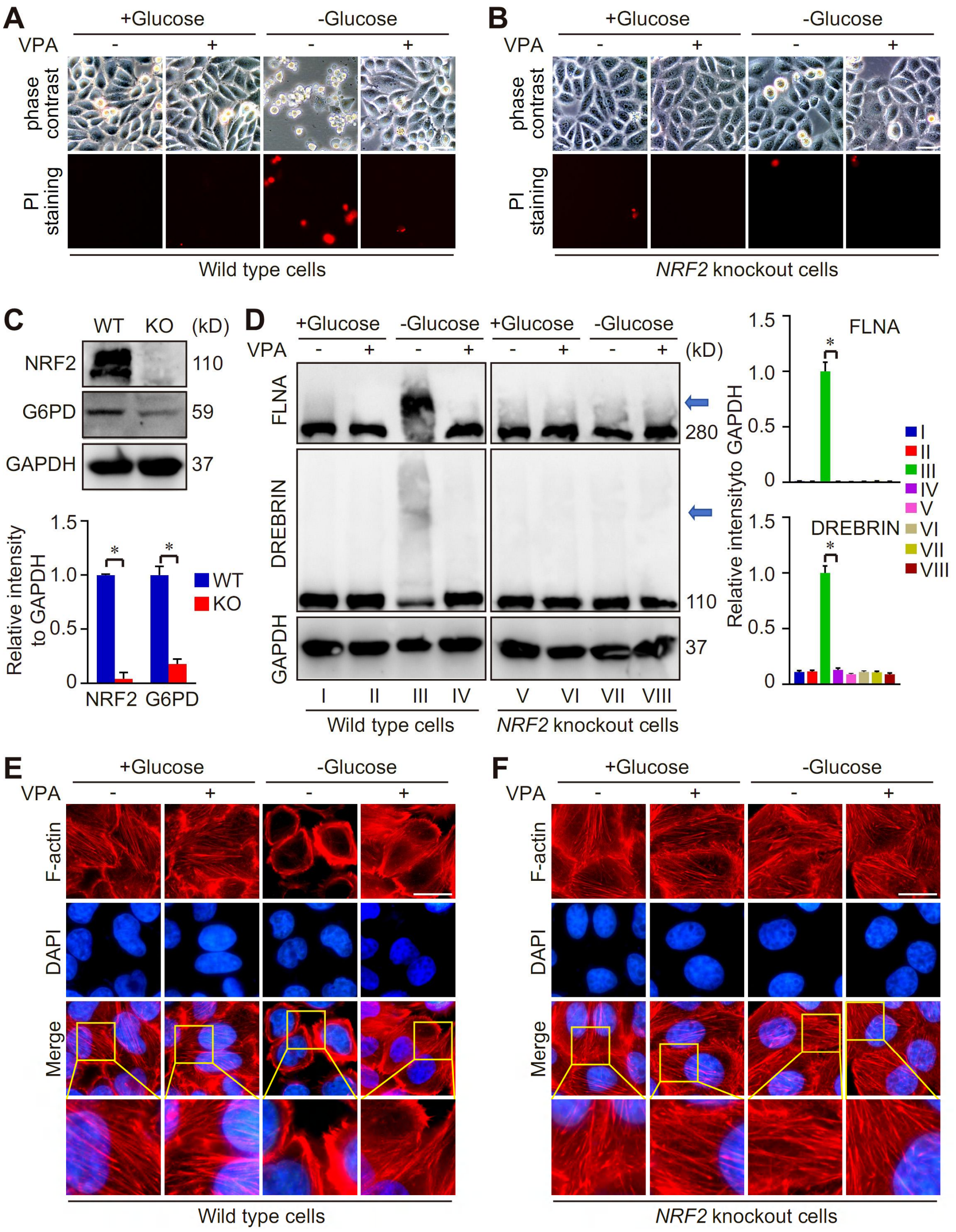

Figure S10. The position of NRF2 in the action of VPA on disulfidptosis. *NRF2* knockout cells were generated through CRISPR/Cas9. Both wild type (A) and *NRF2* knockout (B) HCC cells (MHCC97-H) were exposed to VPA for 24 h, followed by glucose deprivation (10-12 h) to trigger disulfidptosis. The morphological changes and cell death were subsequently analyzed in each group (A-B, Scale bar = 20 μm). Meanwhile, the protein levels of NRF2 and G6PD were measured to verify the knockdown effect (C). Additionally, the formation of disulfide bonds in cytoskeletal proteins (FLNA and DREBRIN) induced by glucose starvation was assessed using non-reducing western blot (D), and the expression level of Group III was considered as “1”. Phalloidin staining was then utilized to examine the

actin filament (F-actin) in the various groups (E-F, Scale bar = 10 μm). Data are expressed as mean ± SD. The P value less than 0.05 was considered statistically significant and the value of Cohen's d over 0.8 represents a large effect size. *: $P < 0.05$ and Cohen's $d > 0.8$ compared between different groups.

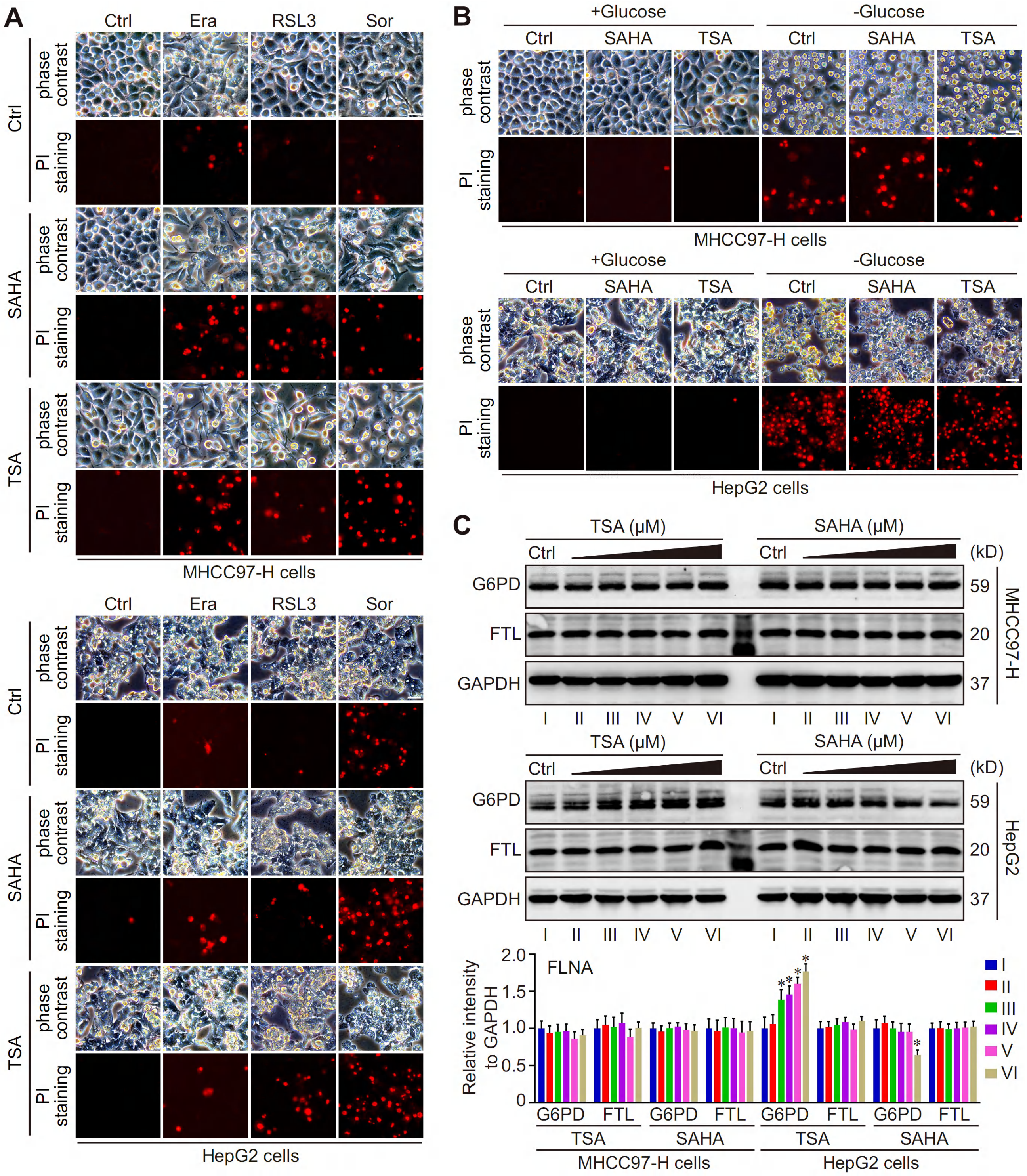

Figure S11. The function of HDAC inhibitors (vorinostat (SAHA) and trichostatin A (TSA)) in ferroptosis and disulfidptosis regulation. Both MHCC97-H and HepG2 cells were subjected to SAHA (0.5 μM) and TSA (0.25 μM) treatment for a duration of 24 h, then exposed to Erastin (Era, 5 μM), Sorafenib (Sor, 1 μM), RSL3 (2 μM)) to induce ferroptosis (A) or exposed to glucose free condition to induce disulfidptosis (B). The morphological changes and cell death were subsequently analyzed in each group (A-B, Scale bar = 20 μm). In

addition, the protein levels of G6PD and FTL in HCC cells treated with TSA (0, 0.0625, 0.125, 0.25, 0.5, 1 μM) and SAHA (0, 0.25, 0.5, 1, 2, 4 μM) were determined using western blot (C). Data are expressed as mean ± SD. The P value less than 0.05 was considered statistically significant and the value of Cohen's d over 0.8 represents a large effect size. *: P < 0.05 and Cohen's d > 0.8 compared to Ctrl group.

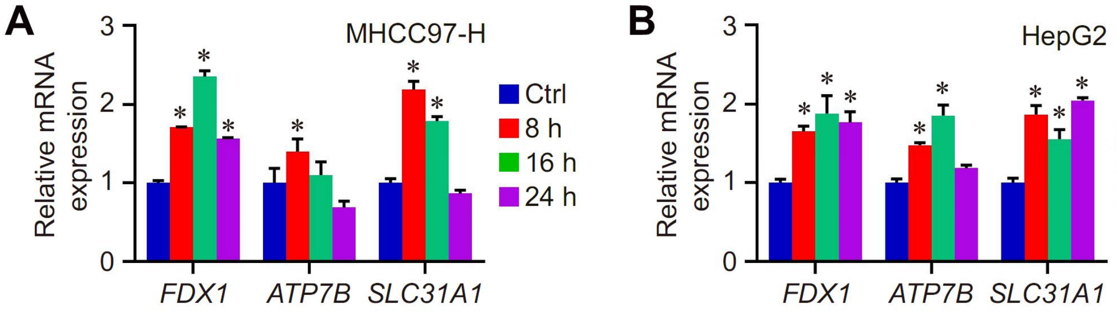

Figure S12. The effect of VPA treatment on transcription of cuproptosis-related genes. Both MHCC97-H cells and HepG2 cells were treated with VPA (2 mM) for 8 h, 16 h or 24 h, then the cells were harvested for qPCR measurement (A). (Data are expressed as mean ± SD. The P value less than 0.05 was considered statistically significant and the value of Cohen's d over 0.8 represents a large effect size. *: P < 0.05 and Cohen's d > 0.8 compared to Ctrl group)

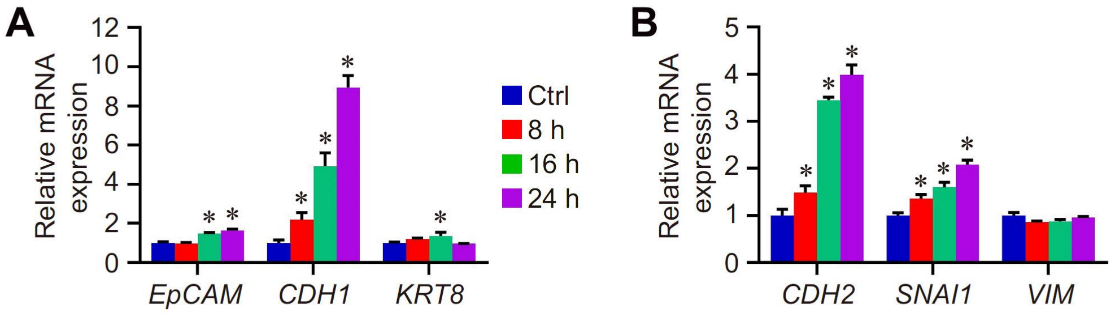

Figure S13. The effect of VPA treatment on transcription of EMT-related genes. Both MHCC97-H cells and HepG2 cells were treated with VPA (2 mM) for 8 h, 16 h or 24 h, then the cells were harvested for qPCR measurement (A). (Data are expressed as mean ± SD. The P value less than 0.05 was considered statistically significant and the value of Cohen's d over 0.8 represents a large effect size. *: P < 0.05 and Cohen's d > 0.8 compared to Ctrl group)

Table S1. The primers used in current study

Primer	Sequecne
Human-*NQO1*-F:	5'- ATGTATGACAAAGGACCCTTCC -3'
Human-*NQO1*-R:	5'- TCCCTTGCAGAGAGTACATGG -3'
Human-*FTL*-F:	5'- CAGGCCTCCTACACCTACCT -3'
Human-*FTL*-R:	5'- AGGGCCTGGTTCAGCTTTTT -3'
Human-*FTH1*-F:	5'- CCCCCATTTGTGTGACTTCAT -3'
Human-*FTH1*-R:	5'- GCCCGAGGCTTAGCTTTCATT -3'
Human-*G6PD*-F:	5'- CGAGGCCGTCACCAAGAAC -3'
Human-*G6PD*-R	5'- GTAGTGGTCGATGCGGTAGA -3'
Human-*SLC7A11*-F:	5'- TCTCCAAAGGAGGTTACCTGC -3'
Human-*SLC7A11*-R:	5'- AGACTCCCCTCAGTAAAGTGAC -3'
Human-*RPN1*-F:	5'- GGCCAAGATTTCAGTCATTGTGG -3'
Human-*RPN1*-R:	5'- CTTCGTTGGATAGGGAGAGTAGA -3'
Human-*NCKAP1*-F:	5'- TTGTACCCCATAGCAAGTCTCT -3'
Human-*NCKAP1*-R:	5'- GGGCATTTCTCCACTGGTCAG -3'
Human-*NUBPL*-F:	5'- CTGAGATGTTTCGCAGAGTCC -3'
Human-*NUBPL*-R:	5'- CAAGGGTCTGTGCTAGTTTCC -3'
Human-*LRPPRC*-F:	5'- GCTCATAGGATATGGGACACACT -3'
Human-*LRPPRC*-R:	5'- CCAGGAAATCAGTTGGTGAGAAT -3'
Human-*OXSM*-F:	5'- CAATATCCAGATTGCATAGGCGA -3'
Human-*OXSM*-R:	5'- CGATCCCAAACCAGGTGAGTT -3'
Human-*GYS1*-F:	5'- GCGCTCACGTCTTCACTACTG -3'
Human-*GYS1*-R:	5'- TCCAGATGCCCATAAAAATGGC -3'
Human-*SLC3A2*-F:	5'- TGAATGAGTTAGAGCCCGAGA -3'
Human-*SLC3A2*-R:	5'- GTCTTCCGCCACCTTGATCTT -3'
Human-*NDUFA11*-F:	5'- TTGAAGGAGTGGCTAAGGTTGG -3'
Human-*NDUFA11*-R:	5'- CACCGAGGAAGTAGTTCAGGG -3'
Human-*NDUFS1*-F:	5'- TTAGCAAATCACCCATTGGACTG -3'
Human-*NDUFS1*-R:	5'- CCCCTCTAAAAATCGGCTCCTA -3'
Human-*GSR*-F:	5'- AGGCTTCCTGCTGCTTCTG -3'

Human-*GSR*-R:	5’- GTAGTCATAGGAGGCCACGG -3’
Human-*PGK1*-F:	5’- GCTCATAAGGACTACCGACTTGG -3’
Human-*PGK1*-R:	5’- TGGACGTTAAAGGGAAGCGG -3’
Human-*PFK1*-F:	5’- GCTGGGCGGCACTATCATT -3’
Human-*PFK1*-R:	5’- TCAGGTGCGAGTAGGTCCG -3’
Human-*GPI*-F:	5’- CCAGGATGGGTGTGTTTGACC -3’
Human-*GPI*-R:	5’- CAAGGACCGCTTCAACCACTT -3’
Human-*FDX1*-F:	5’- TGCCAGATCGAGCATGTCATT -3’
Human-*FDX1*-R:	5’- TTCAACCTGTCACCTCATCTTTG -3’
Human-*ATP7B* -F:	5’- TGATAAGTGATGACGGCCTCT -3’
Human-*ATP7B* -R:	5’- GCCAGCATTGCAGAAGGAAAG -3’
Human-*SLC31A1*-F:	5’- TCACCAAACCGGAAAACAGTAG -3’
Human-*SLC31A1*-R:	5’- GGGGATGAGCTATATGGACTCC -3’
Human-*EpCAM*-F:	5’- CTTGTCTGTTCTTCTGACCCC -3’
Human-*EpCAM*-R:	5’- TGATCCTGACTGCGATGAGAG -3’
Human-*CDH1*-F:	5’- GTGTATGTGGCAATGCGTTC -3’
Human-*CDH1*-R:	5’- TGCCCAGAAAATGAAAAAGG -3’
Human-*KRT8*-F:	5’- CTCTGGTTGACCGTAACTGCG -3’
Human-*KRT8*-R:	5’- CAGAAGTCCTACAAGGTGTCCA -3’
Human-*CDH2*-F:	5’- TGCAGATCGGACCGGATACT -3’
Human-*CDH2*-R:	5’- TGGGAATCCGACGAATGG -3’
Human-*SNAI1*-F:	5’- GCACTGGTACTTCTTGACATCTG -3’
Human-*SNAI1*-R:	5’- ACTGCAACAAGGAATACCTCAG -3’
Human-*VIM*-F:	5’- CTTTGTCGTTGGTTAGCTGGT -3’
Human-*VIM*-R:	5’- GACGCCATCAACACCGAGTT -3’
Human-*NCOA4*-F:	5’- GAGGTGTAGTGATGCACGGAG -3’
Human-*NCOA4*-R:	5’- GACGGCTTATGCAACTGTGAA -3’
Human-*GAPDH*-F:	5’- CTGACTTCAACAGCGACACC -3’
Human-*GAPDH*-R:	5’- TGCTGTAGCCAAATTCGTTGT -3’

Table S2. The potential bioavailable targets of VPA predicted by SwissTargetPrediction and TargetNet

Database	Targets	Number
SwissTargetPrediction	PPARD FFAR1 FABP2 HSD11B1 FABP4 FABP3 AKR1B10 PPARA AR VDR NR1H4 POLB CDC25A GPBAR1 PHF8 FABP5 SERPINA6 SHBG HSD17B3 G6PD GABBR1 PTGFR SLC22A6 NPC1L1 PTGER2 AKR1B1 HSD11B2 HDAC3 ACHE HMGCR PLG PTPN1 GSTK1 KDM2A SAE1 UBA2 PTGER3 CA2 CA1 CDC45 PTPRC EGLN1 AKR1C1 KDM5C CES1 CES2 CCR2 CACNA2D1 PTGDR2 UGT2B7 HAO1 CA12 CPA3 CYP19A1 CHRNA7 CXCL8 FNTA FNTB TBXAS1 EDNRA GRM5 PTGES FDFT1 PTGDR NR4A1 PTPRG TRPM8 CA4 KMO CTSB SLC6A12 EGLN3 HSPA1A GABRA2 GABRB2 GABRG2 MIF PTGS2 ACLY LTA4H SLC22A12 F2 REN DAO CDC25B NR1I3 KDM4C FAAH TERT PEPD CPT1A SRD5A1 SRD5A2 AKR1C3 ITGAL ICAM1 ITGB2 CSNK2A1 CSNK2A2 GRIK1 GRIA1 ADORA3 GRIK5 SLC1A1 GRM4 GRM3	100
TargetNet	MME SIRT1 ACE ACE CA12 CA14 Nos2 NOS3 ECE1 PPO2 FOLH1 HTR1E S1PR5 CES1 CHRM4 Ace MMP3 S1PR2 ANPEP CES2 BCL2A1 CA7 FDPS GRIA2 Mme MGLL Alox5 lef CTSK CDC25B CA6 TLR9 CA5A PTGS1 PTGES Chrm1 HDAC4 CA2 CA1 GPR35 CA13 CA4 ALPL S1PR4 PTGFR PTGS1 CA5B MMP2 CA9 ACHE Akr1b1 ALOX5 MMP1 MMP8 SIRT2 EPHX2 NOS1 PTGER2 APOBEC3A HCAR2 CTSL Nos1 Cnr2 CTSB PLIN5 THRA APOBEC3G Chrm2 Chrna4 CYP1A2 NOS2 PTPRC CSNK2A1 PLA2G1B Adra2c ALOX15 MMP9 Chrm3 DPP7 Cnr2 PLIN1 MMP7 GRM2 RARB FAAH CTSG THRB CHRNA4 CHRM5 Fdft1 NR2F2 LIPE LTB4R Faah RXRB S1PR1 PPARG CHRM1 S1PR3 RIPK2 MCL1 ADRA2B ache HTR5A DUSP3 MIF RXRG HDAC6 HSD11B1 PTPN1 PPARA Ptgs2 CASP9 CHRM3 CACNA1H PTGS2 Ptger2 CTDSP1 RORA DRD2 GALR3 CHRM2 HDAC2 Cnr1 MMP12 APP Maoa PTGIR METAP2 PPARD MMP13 AHR DNMT1 Grin1 DPP8 ITGA2B AKR1B1 DPP9 CNR1 DRD5 PTPN22 CNR2 GPBAR1 RARA DPP4 PLA2G2A Htr2c AKR1C3 MTNR1B RARG PTGS2 GRM4 CAPN1 RELA Hrh1 PTGER4 HDAC3 TAAR1 PREP Maob CTSS MDM2 Chrna7 CHRNA7 HRH1 ABCG2 PTPN7 HDAC8 Htr3a ICMT CA4 CYP2D6 PNMT CHRFAM7A HMGCR Adora1 SOAT1 MAOB Htr4 CYP17A1 MAOA RXRA DRD1 Ar PNP CYP2C19 Soat1 ADRA2C Taar1 ESR1 AR Fnta HRH2 CYP19A1 Grin2b XDH NR1H3 GSK3A CACNA1B SLC6A3 GCGR HNF4A FFAR1 CYP2C9 ELANE TYMS HSD17B3	202
Overlapping targets	CA12 CES1 CES2 CDC25B PTGES CA2 CA1 CA4 PTGFR ACHE PTGER2 CTSB PTPRC CSNK2A1 FAAH MIF HSD11B1 PTPN1 PPARA PTGS2 PPARD AKR1B1 GPBAR1 AKR1C3 GRM4 HDAC3 CHRNA7 HMGCR AR CYP19A1 FFAR1 HSD17B3	32

Table S3. The differentially expressed gene list in RNA-seq assay

gene_id	h97H_VPA1	h97H_VPA2	h97H_ctrl_1	h97H_ctrl_2	log2FoldChange	pvalue	padj	gene_name	gene_description
ENSG00000123358	4579.5576	4261.71802	682.718844	644.054777	2.736189465	1.34E-118	2.27E-114	NR4A1	nuclear receptor subfamily 4 group A member 1 [Source:HGNC Symbol;Acc:HGNC:7980]
ENSG00000109321	1130.86432	1133.20943	86.3934339	101.132568	3.594059717	1.50E-80	1.27E-76	AREG	amphiregulin [Source:HGNC Symbol;Acc:HGNC:651]
ENSG00000186642	1184.01199	1155.35973	5015.03348	4657.42091	-2.04789075	9.11E-76	5.13E-72	PDE2A	phosphodiesterase 2A [Source:HGNC Symbol;Acc:HGNC:8777]
ENSG00000167772	3308.93459	2508.30015	425.645699	464.145261	2.708531822	1.30E-69	5.48E-66	ANGPTL4	angiopoietin like 4 [Source:HGNC Symbol;Acc:HGNC:16039]
ENSG00000169429	1187.94885	1321.04398	150.66172	169.263983	2.971730785	1.28E-66	4.34E-63	CXCL8	C-X-C motif chemokine ligand 8 [Source:HGNC Symbol;Acc:HGNC:6025]
ENSG00000149257	4170.12369	3725.68072	12825.2106	13387.8229	-1.731318097	3.63E-63	1.02E-59	SERPINH1	serpin family H member 1 [Source:HGNC Symbol;Acc:HGNC:1546]
ENSG00000266976	777.530733	783.234663	43.196717	61.7440943	3.895373969	1.43E-62	3.45E-59	AC079466.1	uncharacterized LOC102724908 [Source:NCBI gene;Acc:102724908]
ENSG00000170004	3098.31234	2792.71002	9178.77556	9183.90175	-1.640423486	2.18E-56	4.60E-53	CHD3	chromodomain helicase DNA binding protein 3 [Source:HGNC Symbol;Acc:HGNC:1918]
ENSG00000142227	713.556686	680.457263	2626.57111	3040.36437	-2.023578273	9.06E-55	1.70E-51	EMP3	epithelial membrane protein 3 [Source:HGNC Symbol;Acc:HGNC:3335]
ENSG00000073756	2250.90226	2698.79274	565.771634	496.081861	2.220948443	4.64E-54	7.84E-51	PTGS2	prostaglandin-endoperoxide synthase 2 [Source:HGNC Symbol;Acc:HGNC:9605]
ENSG00000176046	191.922143	201.124738	1100.98949	1198.68707	-2.548073516	2.27E-53	3.49E-50	NUPR1	nuclear protein 1, transcriptional regulator [Source:HGNC Symbol;Acc:HGNC:29990]
ENSG00000120738	3757.73714	4617.89487	1028.29258	1169.94413	1.93011487	2.47E-47	3.48E-44	EGR1	early growth response 1 [Source:HGNC Symbol;Acc:HGNC:3238]
ENSG00000143321	6774.35954	6683.18898	16943.6488	18341.1897	-1.390644503	1.27E-46	1.66E-43	HDGF	heparin binding growth factor [Source:HGNC Symbol;Acc:HGNC:4856]
ENSG00000140545	2405.42419	2329.32571	716.433354	713.250745	1.72751844	4.68E-44	5.65E-41	MFGE8	milk fat globule-EGF factor 8 protein [Source:HGNC Symbol;Acc:HGNC:7036]
ENSG00000168209	4825.61163	3822.25603	13555.3405	13649.7031	-1.653732858	1.06E-43	1.19E-40	DDIT4	DNA damage inducible transcript 4 [Source:HGNC Symbol;Acc:HGNC:24944]
ENSG00000105290	2388.69252	2089.21644	593.164674	646.183884	1.853043102	2.32E-42	2.45E-39	APLP1	amyloid beta precursor like protein 1 [Source:HGNC Symbol;Acc:HGNC:597]
ENSG00000074211	478.329033	445.664067	33.7145108	24.4847271	3.987852436	6.61E-42	6.57E-39	PPP2R2C	protein phosphatase 2 regulatory subunit Bgamma [Source:HGNC Symbol;Acc:HGNC:9306]
ENSG00000245532	18122.3714	17570.5052	7077.94011	7564.71611	1.285452858	8.68E-41	8.15E-38	NEAT1	nuclear paraspeckle assembly transcript 1 [Source:HGNC Symbol;Acc:HGNC:30815]
ENSG00000080573	843.473213	769.05847	146.447406	118.165422	2.606818178	7.68E-40	6.83E-37	COL5A3	collagen type V alpha 3 chain [Source:HGNC Symbol;Acc:HGNC:14864]
ENSG00000163283	531.476704	506.798899	1742.61878	1796.96606	-1.76973256	1.11E-39	9.36E-37	ALPP	alkaline phosphatase, placental [Source:HGNC Symbol;Acc:HGNC:439]
ENSG00000130066	5332.48293	5820.21323	2102.94261	2082.26635	1.414120752	1.67E-39	1.34E-36	SAT1	spermidine/spermine N1-acetyltransferase 1 [Source:HGNC Symbol;Acc:HGNC:10540]
ENSG00000135114	716.509334	708.809649	2157.72869	2453.79547	-1.693992856	1.51E-38	1.16E-35	OASL	2'-5'-oligoadenylate synthetase like [Source:HGNC Symbol;Acc:HGNC:8090]
ENSG00000023171	6361.97299	6055.89244	2510.67748	2393.11593	1.340377065	6.45E-38	4.74E-35	GRAMD1B	GRAM domain containing 1B [Source:HGNC Symbol;Acc:HGNC:29214]
ENSG00000156453	1726.31507	1363.57256	376.127511	357.689926	2.073548155	9.15E-38	6.44E-35	PCDH1	protocadherin 1 [Source:HGNC Symbol;Acc:HGNC:8655]
ENSG00000138119	3978.20155	3810.73788	9308.36571	9281.84066	-1.255127943	1.45E-37	9.78E-35	MYOF	myoferlin [Source:HGNC Symbol;Acc:HGNC:3656]
ENSG00000173868	501.95022	457.182224	37.9288246	52.1631142	3.412582499	3.02E-37	1.96E-34	PHOSPHO1	phosphoethanolamine/phosphocholine phosphatase [Source:HGNC Symbol;Acc:HGNC:16815]
ENSG00000124762	7564.68508	7002.15332	2960.55548	2926.45716	1.30700182	7.77E-37	4.87E-34	CDKN1A	cyclin dependent kinase inhibitor 1A [Source:HGNC Symbol;Acc:HGNC:1784]
ENSG00000163395	457.660495	352.632801	5.26789231	17.0328536	5.185572994	9.69E-37	5.85E-34	IGFN1	immunoglobulin-like and fibronectin type III domain containing 1 [Source:HGNC Symbol;Acc:HGNC:24607]
ENSG00000146072	2110.15936	1826.95687	601.593302	545.051315	1.779361578	1.41E-36	8.24E-34	TNFRSF21	TNF receptor superfamily member 21 [Source:HGNC Symbol;Acc:HGNC:13469]
ENSG00000039068	707.651389	746.022156	138.018779	118.165422	2.50440261	2.04E-36	1.15E-33	CDH1	cadherin 1 [Source:HGNC Symbol;Acc:HGNC:1748]
ENSG00000167965	501.95022	463.384308	1601.43926	1623.44386	-1.74073794	2.88E-36	1.57E-33	MLST8	MTOR associated protein, LST8 homolog [Source:HGNC Symbol;Acc:HGNC:24825]
ENSG00000160886	1097.40097	974.613268	2918.41234	3259.66236	-1.576620706	3.36E-36	1.77E-33	LY6K	lymphocyte antigen 6 family member K [Source:HGNC Symbol;Acc:HGNC:24225]
ENSG00000116983	364.159964	355.290837	7.37504924	10.6455335	5.320194808	1.72E-35	8.81E-33	HPCAL4	hippocalcin like 4 [Source:HGNC Symbol;Acc:HGNC:18212]
ENSG00000143375	721.430415	606.918262	91.6613262	112.842655	2.699226389	4.05E-35	2.01E-32	CGN	cingulin [Source:HGNC Symbol;Acc:HGNC:17429]
ENSG00000158050	3275.47124	3016.87107	1170.52567	1130.55566	1.451122154	6.62E-35	3.20E-32	DUSP2	dual specificity phosphatase 2 [Source:HGNC Symbol;Acc:HGNC:3068]
ENSG00000119403	1417.27121	1259.02314	3510.52344	3797.2618	-1.449630552	4.57E-34	2.14E-31	PHF19	PHD finger protein 19 [Source:HGNC Symbol;Acc:HGNC:24566]
ENSG00000135424	749.972682	621.980467	118.000788	119.229975	2.531319818	7.55E-34	3.45E-31	ITGA7	integrin subunit alpha 7 [Source:HGNC Symbol;Acc:HGNC:6143]
ENSG00000177614	1139.72226	952.462966	248.644517	240.589057	2.095902214	7.89E-34	3.51E-31	PGBD5	piggyBac transposable element derived 5 [Source:HGNC Symbol;Acc:HGNC:19405]
ENSG00000182551	337.586129	336.684583	1190.54366	1191.2352	-1.820663134	1.66E-33	7.21E-31	ADI1	acireductone dioxygenase 1 [Source:HGNC Symbol;Acc:HGNC:30576]
ENSG00000092929	1900.52132	1734.81162	4520.90518	4582.90217	-1.324679769	1.72E-33	7.26E-31	UNC13D	unc-13 homolog D [Source:HGNC Symbol;Acc:HGNC:23147]
ENSG00000119922	119.09015	132.015797	690.093893	640.861117	-2.404814642	3.34E-33	1.38E-30	IFIT2	interferon induced protein with tetratricopeptide repeats 2 [Source:HGNC Symbol;Acc:HGNC:5409]
ENSG00000120708	7858.9657	7688.81267	16591.7536	17474.6432	-1.131661104	7.40E-33	2.98E-30	TGFBI	transforming growth factor beta induced [Source:HGNC Symbol;Acc:HGNC:11771]
ENSG00000078018	1127.91167	1351.16839	335.037951	301.268598	1.962357054	9.11E-33	3.58E-30	MAP2	microtubule associated protein 2 [Source:HGNC Symbol;Acc:HGNC:6839]
ENSG00000043143	1020.63211	879.809977	2872.05489	2621.9949	-1.532219147	1.44E-32	5.51E-30	JADE2	jade family PHD finger 2 [Source:HGNC Symbol;Acc:HGNC:22984]
ENSG00000113389	373.017909	382.757211	34.7680893	33.0011539	3.479234359	1.65E-32	6.20E-30	NPR3	natriuretic peptide receptor 3 [Source:HGNC Symbol;Acc:HGNC:7945]
ENSG00000198830	4046.11246	4210.32932	9696.08259	11947.4822	-1.39028363	2.78E-32	1.02E-29	HMGN2	high mobility group nucleosomal binding domain 2 [Source:HGNC Symbol;Acc:HGNC:4986]
ENSG00000184363	935.989528	769.944482	2470.64149	2558.1217	-1.560543681	1.51E-31	5.41E-29	PKP3	plakophilin 3 [Source:HGNC Symbol;Acc:HGNC:9025]
ENSG00000196517	697.809228	613.120347	1890.11976	1958.77816	-1.554615498	4.69E-31	1.65E-28	SLC6A9	solute carrier family 6 member 9 [Source:HGNC Symbol;Acc:HGNC:11056]

ENSG00000102317	1277.51252	1201.43236	3075.39553	3136.17417	-1.32549527	9.37E-31	3.23E-28	RBM3	RNA binding motif protein 3 [Source:HGNC Symbol;Acc:HGNC:9900]
ENSG00000120129	4906.31735	4615.23683	2030.2457	2021.58681	1.232535653	9.81E-31	3.31E-28	DUSP1	dual specificity phosphatase 1 [Source:HGNC Symbol;Acc:HGNC:3064]
ENSG00000102007	2898.51647	2788.27996	6279.32764	6764.17199	-1.197725288	1.46E-30	4.82E-28	PLP2	proteolipid protein 2 [Source:HGNC Symbol;Acc:HGNC:9087]
ENSG00000106123	755.877979	728.301915	165.411819	172.457643	2.135053853	2.35E-30	7.63E-28	EPHB6	EPH receptor B6 [Source:HGNC Symbol;Acc:HGNC:3396]
ENSG00000125740	745.051601	611.348323	124.322259	138.391936	2.367761944	6.63E-30	2.11E-27	FOSB	FosB proto-oncogene, AP-1 transcription factor subunit [Source:HGNC Symbol;Acc:HGNC:3797]
ENSG00000160867	717.49355	734.503999	171.733289	160.747556	2.12669422	1.18E-29	3.68E-27	FGFR4	fibroblast growth factor receptor 4 [Source:HGNC Symbol;Acc:HGNC:3691]
ENSG00000143341	540.334649	564.389683	107.465003	90.4870348	2.48036531	3.17E-29	9.73E-27	HMCN1	hemicentin 1 [Source:HGNC Symbol;Acc:HGNC:19194]
ENSG00000064205	703.714525	643.244757	1818.47643	1898.09862	-1.464794137	1.30E-28	3.92E-26	WISP2	WNT1 inducible signaling pathway protein 2 [Source:HGNC Symbol;Acc:HGNC:12770]
ENSG00000102996	3165.23904	2686.38857	1100.98949	1118.84557	1.398126268	3.84E-28	1.14E-25	MMP15	matrix metallopeptidase 15 [Source:HGNC Symbol;Acc:HGNC:7161]
ENSG00000060656	2930.9956	2628.79779	1115.73959	1100.74816	1.326545453	1.07E-27	3.11E-25	PTPRU	protein tyrosine phosphatase, receptor type U [Source:HGNC Symbol;Acc:HGNC:9683]
ENSG00000105137	836.5837	680.457263	2094.51398	2285.59604	-1.530667431	1.09E-27	3.12E-25	SYDE1	synapse defective Rho GTPase homolog 1 [Source:HGNC Symbol;Acc:HGNC:25824]
ENSG00000142910	3099.29656	2583.61117	1071.4893	1067.74701	1.409241536	2.34E-27	6.59E-25	TINAGL1	tubulointerstitial nephritis antigen like 1 [Source:HGNC Symbol;Acc:HGNC:19168]
ENSG00000170345	6485	6305.74784	2593.91017	2982.87849	1.197600117	2.39E-27	6.63E-25	FOS	Fos proto-oncogene, AP-1 transcription factor subunit [Source:HGNC Symbol;Acc:HGNC:3796]
ENSG00000143222	292.312187	316.306306	978.774391	1070.94067	-1.751108923	2.70E-27	7.36E-25	UFC1	ubiquitin-fold modifier conjugating enzyme 1 [Source:HGNC Symbol;Acc:HGNC:26941]
ENSG00000111206	3351.25588	3069.14578	6908.31398	7027.11667	-1.118205652	3.92E-27	1.05E-24	FOXM1	forkhead box M1 [Source:HGNC Symbol;Acc:HGNC:3818]
ENSG00000006283	341.522993	253.39945	22.1251477	14.9037469	4.004190609	4.92E-27	1.30E-24	CACNA1G	calcium voltage-gated channel subunit alpha1 G [Source:HGNC Symbol;Acc:HGNC:1394]
ENSG00000155254	1323.77068	1315.72791	3065.91333	2996.71768	-1.19971098	3.01E-26	7.82E-24	MARVELD1	MARVEL domain containing 1 [Source:HGNC Symbol;Acc:HGNC:28674]
ENSG00000162729	3135.71255	2459.56948	1005.11385	1004.93836	1.47663721	3.50E-26	8.95E-24	IGSF8	immunoglobulin superfamily member 8 [Source:HGNC Symbol;Acc:HGNC:17813]
ENSG00000197702	844.457429	933.856713	2214.62193	2247.27212	-1.326632938	4.05E-26	1.02E-23	PARVA	parvin alpha [Source:HGNC Symbol;Acc:HGNC:14652]
ENSG00000120254	1218.45955	1228.89873	2779.33998	2889.19779	-1.211705113	7.85E-26	1.95E-23	MTHFD1L	methylenetetrahydrofolate dehydrogenase (NADP+ dependent) 1 like [Source:HGNC Symbol;Acc:HGNC:21055]
ENSG00000099812	940.910609	724.757867	2324.19409	2396.30959	-1.503915982	9.71E-26	2.35E-23	MISP	mitotic spindle positioning [Source:HGNC Symbol;Acc:HGNC:27000]
ENSG00000105696	402.544392	360.606909	57.9468154	47.9049008	2.849220705	9.74E-26	2.35E-23	TMEM59L	transmembrane protein 59 like [Source:HGNC Symbol;Acc:HGNC:13237]
ENSG00000175334	2011.73775	1957.20064	4225.90321	4789.42552	-1.183686679	1.83E-25	4.35E-23	BANF1	barrier to autointegration factor 1 [Source:HGNC Symbol;Acc:HGNC:17397]
ENSG00000160211	5638.57414	5304.55421	2449.56992	2615.60758	1.111284668	1.85E-25	4.35E-23	G6PD	glucose-6-phosphate dehydrogenase [Source:HGNC Symbol;Acc:HGNC:4057]
ENSG00000235823	965.516012	829.30729	229.680105	264.009231	1.861860345	1.90E-25	4.39E-23	OLMALINC	oligodendrocyte maturation-associated long intergenic non-coding RNA [Source:HGNC Symbol;Acc:HGNC:28060]
ENSG00000184900	1191.88572	1078.27668	2917.35876	2631.57588	-1.289845352	2.83E-25	6.46E-23	SUMO3	small ubiquitin-like modifier 3 [Source:HGNC Symbol;Acc:HGNC:11124]
ENSG00000095637	558.050539	595.400106	136.9652	117.100869	2.182704367	3.67E-25	8.26E-23	SORBS1	sorbin and SH3 domain containing 1 [Source:HGNC Symbol;Acc:HGNC:14565]
ENSG00000115009	232.275004	257.82951	6.32147077	13.8391936	4.605822248	4.41E-25	9.81E-23	CCL20	C-C motif chemokine ligand 20 [Source:HGNC Symbol;Acc:HGNC:10619]
ENSG00000166851	2145.59114	2005.93131	4411.33302	4589.28949	-1.116578634	5.31E-25	1.16E-22	PLK1	polo like kinase 1 [Source:HGNC Symbol;Acc:HGNC:9077]
ENSG00000061273	1270.62301	1109.2871	2807.7866	2912.61797	-1.265746071	5.68E-25	1.23E-22	HDAC7	histone deacetylase 7 [Source:HGNC Symbol;Acc:HGNC:14067]
ENSG00000129255	493.092275	496.166755	1325.40171	1384.98391	-1.454019855	7.58E-25	1.62E-22	MPDU1	mannose-P-dolichol utilization defect 1 [Source:HGNC Symbol;Acc:HGNC:7207]
ENSG00000112984	2407.39263	2289.45517	4865.42534	5296.15292	-1.113480047	8.67E-25	1.83E-22	KIF20A	kinesin family member 20A [Source:HGNC Symbol;Acc:HGNC:9787]
ENSG00000136938	2926.07452	2959.28029	5922.16454	6638.55469	-1.093680581	2.64E-24	5.51E-22	ANP32B	acidic nuclear phosphoprotein 32 family member B [Source:HGNC Symbol;Acc:HGNC:16677]
ENSG00000135709	859.220671	779.690614	246.53736	226.749864	1.791551721	4.01E-24	8.26E-22	KIAA0513	KIAA0513 [Source:HGNC Symbol;Acc:HGNC:29058]
ENSG00000141994	288.375323	279.093799	898.702428	892.095708	-1.658283027	5.83E-24	1.19E-21	DUS3L	dihydrouridine synthase 3 like [Source:HGNC Symbol;Acc:HGNC:26920]
ENSG00000118503	2954.61679	3013.32702	1315.9195	1370.08016	1.151831812	6.07E-24	1.22E-21	TNFAIP3	TNF alpha induced protein 3 [Source:HGNC Symbol;Acc:HGNC:11896]
ENSG00000060566	1460.57672	1164.21985	346.627314	436.466874	1.744643422	6.90E-24	1.37E-21	CREB3L3	cAMP responsive element binding protein 3 like 3 [Source:HGNC Symbol;Acc:HGNC:18855]
ENSG00000146834	828.709972	671.597143	2089.24609	1941.74531	-1.426882479	7.14E-24	1.40E-21	MEPCE	methylphosphate capping enzyme [Source:HGNC Symbol;Acc:HGNC:20247]
ENSG00000119917	137.790257	180.746461	618.450557	752.639219	-2.103161968	1.10E-23	2.14E-21	IFIT3	interferon induced protein with tetratricopeptide repeats 3 [Source:HGNC Symbol;Acc:HGNC:5411]
ENSG00000087077	1287.35468	1114.60317	2766.69704	2921.13439	-1.244215169	1.24E-23	2.38E-21	TRIP6	thyroid hormone receptor interactor 6 [Source:HGNC Symbol;Acc:HGNC:12311]
ENSG00000268388	1306.05479	1309.52583	2815.16165	3020.13785	-1.157649504	1.72E-23	3.27E-21	FENDRR	FOXF1 adjacent non-coding developmental regulatory RNA [Source:HGNC Symbol;Acc:HGNC:43894]
ENSG00000146592	258.848839	252.513438	22.1251477	22.3556204	3.523023308	1.86E-23	3.50E-21	CREB5	cAMP responsive element binding protein 5 [Source:HGNC Symbol;Acc:HGNC:16844]
ENSG00000175063	1271.60722	1265.22522	2740.35758	3108.49578	-1.205120214	2.33E-23	4.32E-21	UBE2C	ubiquitin conjugating enzyme E2 C [Source:HGNC Symbol;Acc:HGNC:15937]
ENSG00000171368	315.933374	256.943498	31.6073539	26.6138338	3.297566944	2.39E-23	4.40E-21	TPPP	tubulin polymerization promoting protein [Source:HGNC Symbol;Acc:HGNC:24164]
ENSG00000143061	2068.82228	1971.37684	870.25581	767.542966	1.302490605	3.09E-23	5.62E-21	IGSF3	immunoglobulin superfamily member 3 [Source:HGNC Symbol;Acc:HGNC:5950]
ENSG00000166741	1611.16179	1648.86845	3428.34432	3999.52694	-1.187968028	3.88E-23	6.98E-21	NNMT	nicotinamide N-methyltransferase [Source:HGNC Symbol;Acc:HGNC:7861]
ENSG00000050344	561.003187	629.954576	152.768877	139.456489	2.027258813	4.31E-23	7.61E-21	NFE2L3	nuclear factor, erythroid 2 like 3 [Source:HGNC Symbol;Acc:HGNC:7783]
ENSG00000101384	962.563364	959.551063	330.823637	275.719318	1.663843174	4.32E-23	7.61E-21	JAG1	jagged 1 [Source:HGNC Symbol;Acc:HGNC:6188]
ENSG00000101255	1348.37608	1175.73801	3004.80577	2844.48655	-1.213012804	4.89E-23	8.52E-21	TRIB3	tribbles pseudokinase 3 [Source:HGNC Symbol;Acc:HGNC:16228]

ENSG00000113739	1894.61603	1637.35029	3856.09717	4071.91656	-1.16690741	5.10E-23	8.79E-21	STC2	stanniocalcin 2 [Source:HGNC Symbol;Acc:HGNC:11374]
ENSG00000077782	2785.33161	2667.78232	1194.75798	1250.85019	1.156820153	5.40E-23	9.22E-21	FGFR1	fibroblast growth factor receptor 1 [Source:HGNC Symbol;Acc:HGNC:3688]
ENSG00000167074	348.412506	333.140535	997.738804	993.228276	-1.546940733	6.53E-23	1.10E-20	TEF	TEF, PAR bZIP transcription factor [Source:HGNC Symbol;Acc:HGNC:11722]
ENSG00000135127	810.009865	692.861432	210.715692	215.039777	1.819144892	1.19E-22	1.98E-20	BICDL1	BICD family like cargo adaptor 1 [Source:HGNC Symbol;Acc:HGNC:28095]
ENSG00000116711	1458.60829	1831.38693	611.075508	532.276675	1.525177637	1.77E-22	2.93E-20	PLA2G4A	phospholipase A2 group IVA [Source:HGNC Symbol;Acc:HGNC:9035]
ENSG00000162645	548.208378	436.803946	85.3398554	108.584442	2.344255999	2.75E-22	4.51E-20	GBP2	guanylate binding protein 2 [Source:HGNC Symbol;Acc:HGNC:4183]
ENSG00000164104	1489.11899	1510.65057	3095.41352	3389.53787	-1.11217425	4.24E-22	6.88E-20	HMGB2	high mobility group box 2 [Source:HGNC Symbol;Acc:HGNC:5000]
ENSG00000178685	590.529671	500.596815	1449.72396	1435.01792	-1.40363207	4.96E-22	7.98E-20	PARP10	poly(ADP-ribose) polymerase family member 10 [Source:HGNC Symbol;Acc:HGNC:25895]
ENSG00000164171	2221.37578	2414.38287	1039.88194	978.324529	1.19987962	5.13E-22	8.19E-20	ITGA2	integrin subunit alpha 2 [Source:HGNC Symbol;Acc:HGNC:6137]
ENSG00000185499	956.658067	862.089736	2055.53158	2252.59489	-1.244580849	6.81E-22	1.07E-19	MUC1	mucin 1, cell surface associated [Source:HGNC Symbol;Acc:HGNC:7508]
ENSG00000180921	3526.44635	2943.33207	7008.40393	6898.30571	-1.104302662	6.85E-22	1.07E-19	FAM83H	family with sequence similarity 83 member H [Source:HGNC Symbol;Acc:HGNC:24797]
ENSG00000118523	504.902869	541.35337	94.8220616	132.004615	2.206186155	7.17E-22	1.11E-19	CTGF	connective tissue growth factor [Source:HGNC Symbol;Acc:HGNC:2500]
ENSG00000198729	212.590682	218.844979	15.8036769	11.7100869	3.970300962	8.38E-22	1.29E-19	PPP1R14C	protein phosphatase 1 regulatory inhibitor subunit 14C [Source:HGNC Symbol;Acc:HGNC:14952]
ENSG00000170955	353.333586	275.549751	997.738804	976.195422	-1.652048865	9.48E-22	1.44E-19	CAVIN3	caveolae associated protein 3 [Source:HGNC Symbol;Acc:HGNC:9400]
ENSG00000092199	10178.7631	10948.451	19545.9876	20940.829	-0.93829403	1.00E-21	1.51E-19	HNRNPC	heterogeneous nuclear ribonucleoprotein C (C1/C2) [Source:HGNC Symbol;Acc:HGNC:5035]
ENSG00000163536	274.596297	307.446185	26.3394616	41.5175807	3.101739573	1.09E-21	1.63E-19	SERPINI1	serpin family I member 1 [Source:HGNC Symbol;Acc:HGNC:8943]
ENSG00000188643	3892.57475	3692.89827	7186.45869	7931.98701	-0.995080797	2.75E-21	4.08E-19	S100A16	S100 calcium binding protein A16 [Source:HGNC Symbol;Acc:HGNC:20441]
ENSG00000144802	897.6051	813.359073	241.269468	291.687618	1.682590992	3.44E-21	5.05E-19	NFKBIZ	NFKB inhibitor zeta [Source:HGNC Symbol;Acc:HGNC:29805]
ENSG00000063244	3835.49021	3392.54018	7137.99408	7427.38872	-1.011080481	4.18E-21	6.08E-19	U2AF2	U2 small nuclear RNA auxiliary factor 2 [Source:HGNC Symbol;Acc:HGNC:23156]
ENSG00000167656	32.4791319	36.3264945	253.912409	284.235745	-2.965418817	4.48E-21	6.47E-19	LY6D	lymphocyte antigen 6 family member D [Source:HGNC Symbol;Acc:HGNC:13348]
ENSG00000136813	1642.6567	1714.43334	3435.71937	3417.21625	-1.029360842	1.01E-20	1.45E-18	ECPAS	Ecm29 proteasome adaptor and scaffold [Source:HGNC Symbol;Acc:HGNC:29020]
ENSG00000160783	167.31674	147.078002	548.914379	620.634603	-1.896577939	1.21E-20	1.72E-18	PMF1	polyamine modulated factor 1 [Source:HGNC Symbol;Acc:HGNC:9112]
ENSG00000121753	2476.28775	2143.26318	995.631647	1023.03577	1.194074118	2.21E-20	3.11E-18	ADGRB2	adhesion G protein-coupled receptor B2 [Source:HGNC Symbol;Acc:HGNC:944]
ENSG00000079999	807.057217	615.778383	1842.70873	1858.71015	-1.380508598	3.19E-20	4.45E-18	KEAP1	kelch like ECH associated protein 1 [Source:HGNC Symbol;Acc:HGNC:23177]
ENSG00000105339	1589.50903	1465.46395	546.807222	652.571204	1.348785964	3.31E-20	4.58E-18	DENND3	DENN domain containing 3 [Source:HGNC Symbol;Acc:HGNC:29134]
ENSG00000170017	2230.23372	2605.76147	1088.34655	933.613288	1.258257073	3.36E-20	4.62E-18	ALCAM	activated leukocyte cell adhesion molecule [Source:HGNC Symbol;Acc:HGNC:400]
ENSG00000135604	305.106997	278.207787	47.4110308	35.1302606	2.820251204	3.82E-20	5.21E-18	STX11	syntaxin 11 [Source:HGNC Symbol;Acc:HGNC:11429]
ENSG00000064195	1082.63773	1050.81031	413.002757	397.0784	1.396920064	4.63E-20	6.25E-18	DLX3	distal-less homeobox 3 [Source:HGNC Symbol;Acc:HGNC:2916]
ENSG00000006007	3481.17241	3440.38484	1610.92147	1762.90035	1.036725988	5.16E-20	6.91E-18	GDE1	glycerophosphodiester phosphodiesterase 1 [Source:HGNC Symbol;Acc:HGNC:29644]
ENSG00000178172	3352.2401	3398.74227	6256.14891	6564.03596	-0.925212539	5.56E-20	7.40E-18	SPINK6	serine peptidase inhibitor, Kazal type 6 [Source:HGNC Symbol;Acc:HGNC:29486]
ENSG00000140092	1386.76051	1294.46362	562.610899	514.179268	1.315897357	6.60E-20	8.72E-18	FBLN5	fibulin 5 [Source:HGNC Symbol;Acc:HGNC:3602]
ENSG00000086475	1111.18	929.426653	2268.35443	2491.05484	-1.222465278	6.81E-20	8.92E-18	SEPHS1	selenophosphate synthetase 1 [Source:HGNC Symbol;Acc:HGNC:19685]
ENSG00000167972	1771.58901	1566.46933	717.486933	682.378698	1.25338784	7.75E-20	1.01E-17	ABCA3	ATP binding cassette subfamily A member 3 [Source:HGNC Symbol;Acc:HGNC:33]
ENSG00000163347	371.049477	342.000656	70.589757	67.0668611	2.372545012	8.53E-20	1.10E-17	CLDN1	claudin 1 [Source:HGNC Symbol;Acc:HGNC:2032]
ENSG00000111885	354.317803	371.239054	76.9112277	62.8086477	2.376430226	9.24E-20	1.18E-17	MAN1A1	mannosidase alpha class 1A member 1 [Source:HGNC Symbol;Acc:HGNC:6821]
ENSG00000067840	815.915162	661.85101	228.626526	216.10433	1.731724761	1.05E-19	1.34E-17	PDZD4	PDZ domain containing 4 [Source:HGNC Symbol;Acc:HGNC:21167]
ENSG00000106078	875.952345	790.322759	202.287065	285.300298	1.772909609	1.08E-19	1.36E-17	COBL	cordon-bleu WH2 repeat protein [Source:HGNC Symbol;Acc:HGNC:22199]
ENSG00000120896	655.487935	587.425997	1448.67039	1540.4087	-1.266595337	1.25E-19	1.57E-17	SORBS3	sorbin and SH3 domain containing 3 [Source:HGNC Symbol;Acc:HGNC:30907]
ENSG00000106537	647.614206	768.172458	187.536966	226.749864	1.77359526	1.33E-19	1.64E-17	TSPAN13	tetraspanin 13 [Source:HGNC Symbol;Acc:HGNC:21643]
ENSG00000111676	3220.35514	2942.44606	5828.39605	6303.22039	-0.977287724	1.33E-19	1.64E-17	ATN1	atrophin 1 [Source:HGNC Symbol;Acc:HGNC:3033]
ENSG00000224189	226.369707	192.264617	6.32147077	19.1619603	4.039889754	1.50E-19	1.84E-17	HAGLR	HOXD antisense growth-associated long non-coding RNA [Source:HGNC Symbol;Acc:HGNC:43755]
ENSG00000132128	1159.40659	1058.78441	2338.94419	2420.79432	-1.10188511	1.65E-19	2.00E-17	LRRC41	leucine rich repeat containing 41 [Source:HGNC Symbol;Acc:HGNC:16917]
ENSG00000123416	9743.73957	9591.96658	18151.0497	16940.2375	-0.859862116	2.14E-19	2.58E-17	TUBA1B	tubulin alpha 1b [Source:HGNC Symbol;Acc:HGNC:18809]
ENSG00000140682	317.901806	276.435763	862.880761	844.190807	-1.523307814	2.30E-19	2.76E-17	TGFB1I1	transforming growth factor beta 1 induced transcript 1 [Source:HGNC Symbol;Acc:HGNC:11767]
ENSG00000175040	257.864623	256.057486	36.8752462	31.9366005	2.900533822	2.40E-19	2.86E-17	CHST2	carbohydrate sulfotransferase 2 [Source:HGNC Symbol;Acc:HGNC:1970]
ENSG00000089220	4497.86766	4230.7076	7954.51739	8713.36917	-0.93333984	2.51E-19	2.97E-17	PEBP1	phosphatidylethanolamine binding protein 1 [Source:HGNC Symbol;Acc:HGNC:8630]
ENSG00000225614	141.727121	111.63752	501.503348	496.081861	-1.9797936	2.59E-19	3.03E-17	ZNF469	zinc finger protein 469 [Source:HGNC Symbol;Acc:HGNC:23216]
ENSG00000154146	1065.90606	963.981123	372.966776	385.368313	1.420201714	2.61E-19	3.04E-17	NRGN	neurogranin [Source:HGNC Symbol;Acc:HGNC:8000]
ENSG00000152137	2354.24495	2450.70936	1082.02508	1176.33145	1.089375776	3.20E-19	3.71E-17	HSPB8	heat shock protein family B (small) member 8 [Source:HGNC Symbol;Acc:HGNC:30171]
ENSG00000125845	1019.6479	992.333509	392.984766	373.658226	1.391858956	3.34E-19	3.84E-17	BMP2	bone morphogenetic protein 2 [Source:HGNC Symbol;Acc:HGNC:1069]
ENSG00000125266	478.329033	509.456936	134.858043	124.552742	1.929099841	3.71E-19	4.23E-17	EFNB2	ephrin B2 [Source:HGNC Symbol;Acc:HGNC:3227]
ENSG00000135476	1462.54515	1335.22018	2826.75101	2977.55572	-1.05317595	3.94E-19	4.46E-17	ESPL1	extra spindle pole bodies like 1, separase [Source:HGNC Symbol;Acc:HGNC:16856]

ENSG00000088325	4643.53165	4920.91099	8664.62927	9055.0908	-0.889509428	4.13E-19	4.65E-17	TPX2	TPX2, microtubule nucleation factor [Source:HGNC Symbol;Acc:HGNC:1249]
ENSG00000196154	1909.37927	1827.84288	3599.02403	4184.75922	-1.058602479	4.66E-19	5.22E-17	S100A4	S100 calcium binding protein A4 [Source:HGNC Symbol;Acc:HGNC:10494]
ENSG00000173457	4093.35484	3587.46284	7291.81654	8390.80951	-1.03001166	5.48E-19	6.10E-17	PPP1R14B	protein phosphatase 1 regulatory inhibitor subunit 14B [Source:HGNC Symbol;Acc:HGNC:9057]
ENSG00000102109	1848.35787	1655.07053	716.433354	792.027693	1.215484665	6.20E-19	6.81E-17	PCSK1N	proprotein convertase subtilisin/kexin type 1 inhibitor [Source:HGNC Symbol;Acc:HGNC:17301]
ENSG00000188559	1280.46517	1414.96126	574.200262	529.083015	1.288931656	6.21E-19	6.81E-17	RALGAPA2	Ral GTPase activating protein catalytic alpha subunit 2 [Source:HGNC Symbol;Acc:HGNC:16207]
ENSG00000179104	398.607528	427.943826	102.197111	89.4224814	2.108950766	9.07E-19	9.89E-17	TMTC2	transmembrane and tetratricopeptide repeat containing 2 [Source:HGNC Symbol;Acc:HGNC:25440]
ENSG00000196754	512.776598	365.922982	1204.24018	1367.95106	-1.55127705	9.28E-19	1.00E-16	S100A2	S100 calcium binding protein A2 [Source:HGNC Symbol;Acc:HGNC:10492]
ENSG00000119771	542.303081	461.612284	129.590151	134.133722	1.928005581	1.12E-18	1.21E-16	KLHL29	kelch like family member 29 [Source:HGNC Symbol;Acc:HGNC:29404]
ENSG00000115053	9228.99454	9223.38556	16015.4462	17700.3286	-0.869612342	1.15E-18	1.23E-16	NCL	nucleolin [Source:HGNC Symbol;Acc:HGNC:7667]
ENSG00000070915	6696.60647	6272.07939	11791.6502	11647.2782	-0.853953831	1.40E-18	1.48E-16	SLC12A3	solute carrier family 12 member 3 [Source:HGNC Symbol;Acc:HGNC:10912]
ENSG00000155115	338.570345	292.38398	917.666841	845.25536	-1.483514091	1.83E-18	1.94E-16	GTF3C6	general transcription factor IIIC subunit 6 [Source:HGNC Symbol;Acc:HGNC:20872]
ENSG00000233276	3989.02793	3461.64913	7200.15521	7226.18814	-0.953496589	1.86E-18	1.95E-16	GPX1	glutathione peroxidase 1 [Source:HGNC Symbol;Acc:HGNC:4553]
ENSG00000130477	220.46441	190.492593	22.1251477	15.9683003	3.430171644	1.90E-18	1.98E-16	UNC13A	unc-13 homolog A [Source:HGNC Symbol;Acc:HGNC:23150]
ENSG00000183762	2038.31158	1914.67207	933.470518	912.322221	1.098534138	2.23E-18	2.31E-16	KREMEN1	kringle containing transmembrane protein 1 [Source:HGNC Symbol;Acc:HGNC:17550]
ENSG00000104081	1162.35924	1087.1368	449.878003	459.887047	1.30584148	2.40E-18	2.47E-16	BMF	Bcl2 modifying factor [Source:HGNC Symbol;Acc:HGNC:24132]
ENSG00000108821	1679.0727	1348.51036	608.968351	598.278983	1.325948334	2.42E-18	2.48E-16	COL1A1	collagen type I alpha 1 chain [Source:HGNC Symbol;Acc:HGNC:2197]
ENSG00000003137	1303.10214	1110.17311	482.538936	469.468027	1.34148848	2.81E-18	2.86E-16	CYP26B1	cytochrome P450 family 26 subfamily B member 1 [Source:HGNC Symbol;Acc:HGNC:20581]
ENSG00000159753	288.375323	267.575643	44.2502954	46.8403474	2.609387844	2.96E-18	3.00E-16	CARMIL2	capping protein regulator and myosin 1 linker 2 [Source:HGNC Symbol;Acc:HGNC:27089]
ENSG00000142871	3729.19487	3472.28127	1620.40368	1872.54934	1.043787324	3.22E-18	3.23E-16	CYR61	cysteine rich angiogenic inducer 61 [Source:HGNC Symbol;Acc:HGNC:2654]
ENSG00000065989	428.134011	372.125066	83.2326985	97.9389082	2.142813559	3.48E-18	3.48E-16	PDE4A	phosphodiesterase 4A [Source:HGNC Symbol;Acc:HGNC:8780]
ENSG00000129474	1234.20701	1088.02281	2474.85581	2418.66521	-1.075876573	3.54E-18	3.52E-16	AJUBA	ajuba LIM protein [Source:HGNC Symbol;Acc:HGNC:20250]
ENSG00000187957	790.325543	864.747772	304.484176	291.687618	1.473362258	3.68E-18	3.64E-16	DNER	delta/notch like EGF repeat containing [Source:HGNC Symbol;Acc:HGNC:24456]
ENSG00000131652	520.650327	511.22896	1206.34734	1232.75278	-1.241189264	3.73E-18	3.66E-16	THOC6	THO complex 6 [Source:HGNC Symbol;Acc:HGNC:28369]
ENSG00000054118	3040.24359	2984.97464	5422.76835	5850.78521	-0.90388737	5.04E-18	4.92E-16	THRAP3	thyroid hormone receptor associated protein 3 [Source:HGNC Symbol;Acc:HGNC:22964]
ENSG00000049246	193.890575	193.150629	601.593302	603.60175	-1.638743404	6.03E-18	5.86E-16	PER3	period circadian regulator 3 [Source:HGNC Symbol;Acc:HGNC:8847]
ENSG00000173621	1075.74822	945.37487	2136.65712	2187.65713	-1.09787407	6.34E-18	6.12E-16	LRFN4	leucine rich repeat and fibronectin type III domain containing 4 [Source:HGNC Symbol;Acc:HGNC:28456]
ENSG00000135414	1825.7209	1866.82741	856.55929	881.450174	1.087255538	7.17E-18	6.86E-16	GDF11	growth differentiation factor 11 [Source:HGNC Symbol;Acc:HGNC:4216]
ENSG00000165548	177.158901	181.632473	9.48220616	15.9683003	3.81865562	7.18E-18	6.86E-16	TMEM63C	transmembrane protein 63C [Source:HGNC Symbol;Acc:HGNC:23787]
ENSG00000127399	975.358173	783.234663	2050.26369	1996.03753	-1.203099059	7.87E-18	7.48E-16	LRRC61	leucine rich repeat containing 61 [Source:HGNC Symbol;Acc:HGNC:21704]
ENSG00000047644	2881.78479	2881.31122	1486.59921	1458.43809	0.968549606	1.01E-17	9.57E-16	WWC3	WWC family member 3 [Source:HGNC Symbol;Acc:HGNC:29237]
ENSG00000126878	1351.32873	1264.33921	553.128693	572.729702	1.215979502	1.03E-17	9.58E-16	AIF1L	allograft inflammatory factor 1 like [Source:HGNC Symbol;Acc:HGNC:28904]
ENSG00000137819	1318.8496	1358.25649	548.914379	603.60175	1.216036489	1.03E-17	9.58E-16	PAQR5	progestin and adipoQ receptor family member 5 [Source:HGNC Symbol;Acc:HGNC:29645]
ENSG00000167702	985.200334	909.048375	339.252265	381.110099	1.394668673	1.16E-17	1.07E-15	KIFC2	kinesin family member C2 [Source:HGNC Symbol;Acc:HGNC:29530]
ENSG00000182326	1545.21931	1536.34492	2953.18043	3037.17071	-0.958992977	1.19E-17	1.10E-15	C1S	complement C1s [Source:HGNC Symbol;Acc:HGNC:1247]
ENSG00000248323	1732.22037	1735.69763	711.165462	832.48072	1.167818199	1.28E-17	1.18E-15	LUCAT1	lung cancer associated transcript 1 [Source:HGNC Symbol;Acc:HGNC:48498]
ENSG00000091640	444.865685	396.047392	1015.64964	1099.68361	-1.331624723	1.42E-17	1.30E-15	SPAG7	sperm associated antigen 7 [Source:HGNC Symbol;Acc:HGNC:11216]
ENSG00000009950	470.455304	466.928357	113.786474	138.391936	1.894393599	1.84E-17	1.67E-15	MLXIPL	MLX interacting protein like [Source:HGNC Symbol;Acc:HGNC:12744]
ENSG00000119508	301.170132	296.814041	55.8396585	58.5504343	2.386159545	1.94E-17	1.75E-15	NR4A3	nuclear receptor subfamily 4 group A member 3 [Source:HGNC Symbol;Acc:HGNC:7982]
ENSG00000181222	6020.45	5293.03606	10753.8754	10278.2626	-0.894705426	1.98E-17	1.78E-15	POLR2A	RNA polymerase II subunit A [Source:HGNC Symbol;Acc:HGNC:9187]
ENSG00000163430	3070.75429	3279.13064	6016.9866	5712.39328	-0.885192771	2.18E-17	1.95E-15	FSTL1	follistatin like 1 [Source:HGNC Symbol;Acc:HGNC:3972]
ENSG00000198598	630.882532	567.04772	181.215496	196.94237	1.663185451	2.33E-17	2.08E-15	MMP17	matrix metallopeptidase 17 [Source:HGNC Symbol;Acc:HGNC:7163]
ENSG00000024422	3027.44878	2703.2228	5381.67879	5422.83477	-0.915102965	2.72E-17	2.41E-15	EHD2	EH domain containing 2 [Source:HGNC Symbol;Acc:HGNC:3243]
ENSG00000142945	1316.88117	1344.0803	2557.03493	2716.74015	-0.986792946	2.75E-17	2.42E-15	KIF2C	kinesin family member 2C [Source:HGNC Symbol;Acc:HGNC:6393]
ENSG00000099783	6808.80711	6496.24044	11435.5406	12275.3647	-0.83362527	2.89E-17	2.53E-15	HNRNPM	heterogeneous nuclear ribonucleoprotein M [Source:HGNC Symbol;Acc:HGNC:5046]
ENSG00000069482	813.94673	667.167083	1644.63598	1789.51418	-1.214172469	3.48E-17	3.03E-15	GAL	galanin and GMAP prepropeptide [Source:HGNC Symbol;Acc:HGNC:4114]
ENSG00000151117	174.206253	151.508063	6.32147077	11.7100869	4.17603198	3.97E-17	3.44E-15	TMEM86A	transmembrane protein 86A [Source:HGNC Symbol;Acc:HGNC:26890]
ENSG00000244122	1737.14145	1841.13306	3333.52225	3567.31828	-0.94730982	4.22E-17	3.64E-15	UGT1A7	UDP glucuronosyltransferase family 1 member A7 [Source:HGNC Symbol;Acc:HGNC:12539]
ENSG00000179271	1548.17195	1435.33954	2834.12606	3108.49578	-0.994330513	4.98E-17	4.27E-15	GADD45GIP1	GADD45G interacting protein 1 [Source:HGNC Symbol;Acc:HGNC:29996]
ENSG00000104518	1059.01654	946.260882	2063.96021	2117.39661	-1.060666025	5.06E-17	4.32E-15	GSDMD	gasdermin D [Source:HGNC Symbol;Acc:HGNC:25697]
ENSG00000074657	518.681894	579.451888	1264.29415	1275.33491	-1.208877076	5.22E-17	4.43E-15	ZNF532	zinc finger protein 532 [Source:HGNC Symbol;Acc:HGNC:30940]

ENSG00000133466	630.882532	551.985514	1459.20617	1310.46517	-1.228201998	5.26E-17	4.44E-15	C1QTNF6	C1q and TNF related 6 [Source:HGNC Symbol;Acc:HGNC:14343]
ENSG00000113368	2164.29124	2085.67239	4143.72409	3866.45777	-0.914502735	5.50E-17	4.62E-15	LMNB1	lamin B1 [Source:HGNC Symbol;Acc:HGNC:6637]
ENSG00000182575	175.190469	142.647942	9.48220616	7.45187345	4.228952154	5.83E-17	4.87E-15	NXPH3	neurexophilin 3 [Source:HGNC Symbol;Acc:HGNC:8077]
ENSG00000165458	4779.35347	4366.26744	8150.48298	8320.54899	-0.848915393	7.61E-17	6.31E-15	INPPL1	inositol polyphosphate phosphatase like 1 [Source:HGNC Symbol;Acc:HGNC:6080]
ENSG00000184216	3001.85916	2369.19625	5510.21536	5561.2267	-1.04397317	7.61E-17	6.31E-15	IRAK1	interleukin 1 receptor associated kinase 1 [Source:HGNC Symbol;Acc:HGNC:6112]
ENSG00000008513	1119.05373	1079.16269	466.735259	466.274367	1.236250151	7.98E-17	6.57E-15	ST3GAL1	ST3 beta-galactoside alpha-2,3-sialyltransferase 1 [Source:HGNC Symbol;Acc:HGNC:10862]
ENSG00000153162	1549.15617	1567.35534	705.89757	744.122792	1.103924225	8.80E-17	7.21E-15	BMP6	bone morphogenetic protein 6 [Source:HGNC Symbol;Acc:HGNC:1073]
ENSG00000165272	2911.31128	2594.24332	1280.09783	1412.6623	1.031633317	9.03E-17	7.37E-15	AQP3	aquaporin 3 (Gill blood group) [Source:HGNC Symbol;Acc:HGNC:636]
ENSG00000075218	1128.89589	1077.39067	2187.22889	2229.17472	-1.001456514	9.14E-17	7.42E-15	GTSE1	G2 and S-phase expressed 1 [Source:HGNC Symbol;Acc:HGNC:13698]
ENSG00000180998	196.843224	191.378605	22.1251477	21.291067	3.160389803	9.52E-17	7.70E-15	GPR137C	G protein-coupled receptor 137C [Source:HGNC Symbol;Acc:HGNC:25445]
ENSG00000183044	204.716953	229.477124	32.6609323	20.2265137	3.036886632	1.03E-16	8.32E-15	ABAT	4-aminobutyrate aminotransferase [Source:HGNC Symbol;Acc:HGNC:23]
ENSG00000272405	535.413568	430.601862	127.482994	136.262829	1.872226167	1.04E-16	8.32E-15	AL365181.3	novel transcript, antisense to BCAN
ENSG00000136378	1004.88466	826.649254	319.234274	354.496266	1.44232841	1.10E-16	8.80E-15	ADAMTS7	ADAM metallopeptidase with thrombospondin type 1 motif 7 [Source:HGNC Symbol;Acc:HGNC:223]
ENSG00000100403	2407.39263	2023.65155	4341.79684	4408.31542	-0.982058471	1.11E-16	8.82E-15	ZC3H7B	zinc finger CCCH-type containing 7B [Source:HGNC Symbol;Acc:HGNC:30869]
ENSG00000179051	3725.25801	3465.19317	6360.45318	6649.20023	-0.855560914	1.16E-16	9.19E-15	RCC2	regulator of chromosome condensation 2 [Source:HGNC Symbol;Acc:HGNC:30297]
ENSG00000100105	547.224162	469.586393	1166.31136	1259.36661	-1.255236828	1.26E-16	9.91E-15	PATZ1	POZ/BTB and AT hook containing zinc finger 1 [Source:HGNC Symbol;Acc:HGNC:13071]
ENSG00000166888	2428.06116	2212.37212	4426.08312	4260.34251	-0.904751498	1.61E-16	1.26E-14	STAT6	signal transducer and activator of transcription 6 [Source:HGNC Symbol;Acc:HGNC:11368]
ENSG00000070540	469.471088	473.130441	115.893631	146.908362	1.842962305	1.66E-16	1.29E-14	WIPI1	WD repeat domain, phosphoinositide interacting 1 [Source:HGNC Symbol;Acc:HGNC:25471]
ENSG00000168528	2971.34846	2564.11891	1394.93788	1317.91705	1.028613079	1.87E-16	1.45E-14	SERINC2	serine incorporator 2 [Source:HGNC Symbol;Acc:HGNC:23231]
ENSG00000182175	205.701169	166.570268	21.0715692	7.45187345	3.703495796	2.04E-16	1.58E-14	RGMA	repulsive guidance molecule BMP co-receptor a [Source:HGNC Symbol;Acc:HGNC:30308]
ENSG00000148773	7262.53074	7235.1745	12224.6709	12600.0535	-0.775955206	2.11E-16	1.62E-14	MKI67	marker of proliferation Ki-67 [Source:HGNC Symbol;Acc:HGNC:7107]
ENSG00000182240	1082.63773	1154.47372	479.3782	484.371774	1.21511944	2.52E-16	1.93E-14	BACE2	beta-secretase 2 [Source:HGNC Symbol;Acc:HGNC:934]
ENSG00000182095	2356.21339	1960.74469	4371.29704	4186.88833	-0.987719352	2.86E-16	2.17E-14	TNRC18	trinucleotide repeat containing 18 [Source:HGNC Symbol;Acc:HGNC:11962]
ENSG00000204366	89.5636668	91.2592424	372.966776	350.238052	-1.99962813	2.98E-16	2.26E-14	ZBTB12	zinc finger and BTB domain containing 12 [Source:HGNC Symbol;Acc:HGNC:19066]
ENSG00000114942	3129.80726	3029.27524	5392.21457	6272.34834	-0.921388889	3.03E-16	2.28E-14	EEF1B2	eukaryotic translation elongation factor 1 beta 2 [Source:HGNC Symbol;Acc:HGNC:3208]
ENSG00000153885	528.524055	376.555126	1188.43651	1221.04269	-1.414351187	3.35E-16	2.51E-14	KCTD15	potassium channel tetramerization domain containing 15 [Source:HGNC Symbol;Acc:HGNC:23297]
ENSG00000215218	154.521931	140.875918	3.16073539	5.32276675	5.1230559	3.74E-16	2.79E-14	UBE2QL1	ubiquitin conjugating enzyme E2 Q family like 1 [Source:HGNC Symbol;Acc:HGNC:37269]
ENSG00000160299	1609.19335	1500.90443	2943.69822	2970.10385	-0.927373176	4.23E-16	3.15E-14	PCNT	pericentrin [Source:HGNC Symbol;Acc:HGNC:16068]
ENSG00000127954	350.380938	357.948873	92.7149047	75.5832879	2.073218154	4.26E-16	3.16E-14	STEAP4	STEAP4 metalloreductase [Source:HGNC Symbol;Acc:HGNC:21923]
ENSG00000128872	463.565792	481.10455	144.340249	139.456489	1.735058875	4.62E-16	3.41E-14	TMOD2	tropomodulin 2 [Source:HGNC Symbol;Acc:HGNC:11872]
ENSG00000180198	2062.91698	1915.55808	3596.91687	4063.40014	-0.945368305	4.92E-16	3.62E-14	RCC1	regulator of chromosome condensation 1 [Source:HGNC Symbol;Acc:HGNC:1913]
ENSG00000089127	1118.06951	1089.79484	470.949573	490.759094	1.198926302	5.22E-16	3.82E-14	OAS1	2'-5'-oligoadenylate synthetase 1 [Source:HGNC Symbol;Acc:HGNC:8086]
ENSG00000145819	557.066323	610.462311	206.501379	168.199429	1.639761709	5.71E-16	4.16E-14	ARHGAP26	Rho GTPase activating protein 26 [Source:HGNC Symbol;Acc:HGNC:17073]
ENSG00000169564	2804.03172	2348.81798	4879.12186	5102.40421	-0.954243957	6.01E-16	4.36E-14	PCBP1	poly(rC) binding protein 1 [Source:HGNC Symbol;Acc:HGNC:8647]
ENSG00000167900	2409.36106	2006.81732	4228.01037	4458.34943	-0.976358443	6.30E-16	4.53E-14	TK1	thymidine kinase 1 [Source:HGNC Symbol;Acc:HGNC:11830]
ENSG00000158292	561.987403	535.151285	182.269074	178.844963	1.603011578	6.31E-16	4.53E-14	GPR153	G protein-coupled receptor 153 [Source:HGNC Symbol;Acc:HGNC:23618]
ENSG00000145569	1011.77417	1038.40614	448.824425	426.885893	1.227273519	7.04E-16	5.04E-14	OTULINL	OTU deubiquitinase with linear linkage specificity like [Source:HGNC Symbol;Acc:HGNC:25629]
ENSG00000134057	3314.83989	3395.19822	5791.52081	6720.5253	-0.898867052	7.29E-16	5.20E-14	CCNB1	cyclin B1 [Source:HGNC Symbol;Acc:HGNC:1579]
ENSG00000132846	378.923206	409.337573	971.399342	927.225968	-1.267673892	7.69E-16	5.46E-14	ZBED3	zinc finger BED-type containing 3 [Source:HGNC Symbol;Acc:HGNC:20711]
ENSG00000023445	667.298528	810.701037	270.769665	186.296836	1.693365953	7.82E-16	5.53E-14	BIRC3	baculoviral IAP repeat containing 3 [Source:HGNC Symbol;Acc:HGNC:591]
ENSG00000152127	2813.87388	2749.29543	1510.83151	1342.40177	0.963218634	8.18E-16	5.76E-14	MGAT5	alpha-1,6-mannosylglycoprotein 6-beta-N-acetylglucosaminyltransferase [Source:HGNC Symbol;Acc:HGNC:7049]
ENSG00000174705	1665.29367	1460.14788	734.344188	708.992531	1.114294396	9.63E-16	6.75E-14	SH3PXD2B	SH3 and PX domains 2B [Source:HGNC Symbol;Acc:HGNC:29242]
ENSG00000132341	10701.3819	10222.8072	17260.7759	18549.8421	-0.775246597	1.05E-15	7.34E-14	RAN	RAN, member RAS oncogene family [Source:HGNC Symbol;Acc:HGNC:9846]
ENSG00000156508	80526.5944	83394.9993	132509.617	139408.584	-0.7301627	1.06E-15	7.36E-14	EEF1A1	eukaryotic translation elongation factor 1 alpha 1 [Source:HGNC Symbol;Acc:HGNC:3189]
ENSG00000091409	4785.25877	5623.51856	2739.304	2118.46117	1.099513448	1.06E-15	7.36E-14	ITGA6	integrin subunit alpha 6 [Source:HGNC Symbol;Acc:HGNC:6142]
ENSG00000065978	17946.1967	17356.9763	28799.5673	31744.9809	-0.778211212	1.08E-15	7.43E-14	YBX1	Y-box binding protein 1 [Source:HGNC Symbol;Acc:HGNC:8014]
ENSG00000169136	191.922143	199.352714	555.23585	588.698003	-1.547370138	1.12E-15	7.68E-14	ATF5	activating transcription factor 5 [Source:HGNC Symbol;Acc:HGNC:790]
ENSG00000167601	1406.44483	1162.44783	2675.03572	2591.12285	-1.036248854	1.18E-15	8.07E-14	AXL	AXL receptor tyrosine kinase [Source:HGNC Symbol;Acc:HGNC:905]
ENSG00000139874	691.903931	666.28107	1485.54563	1399.88766	-1.087341435	1.18E-15	8.07E-14	SSTR1	somatostatin receptor 1 [Source:HGNC Symbol;Acc:HGNC:11330]

ENSG00000073792	999.963576	937.400761	347.680893	423.692233	1.328546298	1.25E-15	8.46E-14	IGF2BP2	insulin like growth factor 2 mRNA binding protein 2 [Source:HGNC Symbol;Acc:HGNC:28867]
ENSG00000131650	509.823949	423.513766	133.804465	137.327382	1.782764315	1.36E-15	9.17E-14	KREMEN2	kringle containing transmembrane protein 2 [Source:HGNC Symbol;Acc:HGNC:18797]
ENSG00000276043	2780.41053	2380.71441	4845.40735	4869.26702	-0.912813863	1.48E-15	9.93E-14	UHRF1	ubiquitin like with PHD and ring finger domains 1 [Source:HGNC Symbol;Acc:HGNC:12556]
ENSG00000161618	261.801487	193.150629	658.486539	702.605211	-1.583287205	1.50E-15	1.01E-13	ALDH16A1	aldehyde dehydrogenase 16 family member A1 [Source:HGNC Symbol;Acc:HGNC:28114]
ENSG00000108551	191.922143	177.202412	17.9108339	24.4847271	3.12242557	1.52E-15	1.02E-13	RASD1	ras related dexamethasone induced 1 [Source:HGNC Symbol;Acc:HGNC:15828]
ENSG00000187778	1036.37957	948.032906	1935.42364	2062.03984	-1.01075607	1.73E-15	1.15E-13	MCRS1	microspherule protein 1 [Source:HGNC Symbol;Acc:HGNC:6960]
ENSG00000156299	793.278191	846.141519	342.413	290.623065	1.372903542	1.86E-15	1.23E-13	TIAM1	T cell lymphoma invasion and metastasis 1 [Source:HGNC Symbol;Acc:HGNC:11805]
ENSG00000210082	82245.0357	102006.569	45892.8242	51024.0421	0.926871352	1.88E-15	1.24E-13	MT-RNR2	mitochondrially encoded 16S RNA [Source:HGNC Symbol;Acc:HGNC:7471]
ENSG00000188483	919.257854	1072.07459	1982.83467	2163.17241	-1.057343665	2.00E-15	1.32E-13	IER5L	immediate early response 5 like [Source:HGNC Symbol;Acc:HGNC:23679]
ENSG00000211448	5428.93611	6264.99129	11491.3803	10205.873	-0.891618009	2.07E-15	1.36E-13	DIO2	iodothyronine deiodinase 2 [Source:HGNC Symbol;Acc:HGNC:2884]
ENSG00000214706	1476.32418	1375.09072	2757.21484	2648.60874	-0.923109898	2.61E-15	1.70E-13	IFRD2	interferon related developmental regulator 2 [Source:HGNC Symbol;Acc:HGNC:5457]
ENSG00000006534	723.398847	660.964998	1421.27735	1520.18218	-1.087778673	2.75E-15	1.79E-13	ALDH3B1	aldehyde dehydrogenase 3 family member B1 [Source:HGNC Symbol;Acc:HGNC:410]
ENSG00000140939	523.602975	428.829838	1139.9719	1109.26459	-1.240949312	3.09E-15	2.00E-13	NOL3	nucleolar protein 3 [Source:HGNC Symbol;Acc:HGNC:7869]
ENSG00000214022	2199.72302	2033.39768	3897.18673	3806.84278	-0.864120048	3.40E-15	2.19E-13	REPIN1	replication initiator 1 [Source:HGNC Symbol;Acc:HGNC:17922]
ENSG00000135069	2646.55714	2716.51298	4831.71083	4642.51716	-0.820890621	3.49E-15	2.24E-13	PSAT1	phosphoserine aminotransferase 1 [Source:HGNC Symbol;Acc:HGNC:19129]
ENSG00000158813	204.716953	174.544376	27.39304	18.097407	3.058220591	3.60E-15	2.30E-13	EDA	ectodysplasin A [Source:HGNC Symbol;Acc:HGNC:3157]
ENSG00000005339	1609.19335	1428.25144	3084.87774	2813.6145	-0.957888789	3.72E-15	2.37E-13	CREBBP	CREB binding protein [Source:HGNC Symbol;Acc:HGNC:2348]
ENSG00000047457	53.1476704	65.5648926	269.716086	292.752171	-2.241503277	3.91E-15	2.48E-13	CP	ceruloplasmin [Source:HGNC Symbol;Acc:HGNC:2295]
ENSG00000123268	446.834118	470.472405	1137.86474	995.357382	-1.217274	4.18E-15	2.65E-13	ATF1	activating transcription factor 1 [Source:HGNC Symbol;Acc:HGNC:783]
ENSG00000198624	356.286235	373.89709	104.304268	73.4541812	2.038092196	4.21E-15	2.65E-13	CCDC69	coiled-coil domain containing 69 [Source:HGNC Symbol;Acc:HGNC:24487]
ENSG00000196230	19766.9965	18465.3774	31075.2967	34360.5885	-0.775311378	5.45E-15	3.42E-13	TUBB	tubulin beta class I [Source:HGNC Symbol;Acc:HGNC:20778]
ENSG00000101298	828.709972	712.353698	291.841234	301.268598	1.377076235	5.58E-15	3.49E-13	SNPH	syntaphilin [Source:HGNC Symbol;Acc:HGNC:15931]
ENSG00000108312	1746.98361	1581.53153	3012.18082	3355.47216	-0.936164598	5.90E-15	3.68E-13	UBTF	upstream binding transcription factor [Source:HGNC Symbol;Acc:HGNC:12511]
ENSG00000151150	315.933374	347.316728	88.5005908	70.2605211	2.062756596	6.40E-15	3.96E-13	ANK3	ankyrin 3 [Source:HGNC Symbol;Acc:HGNC:494]
ENSG00000114948	844.457429	948.918918	375.073933	367.270906	1.272886736	6.42E-15	3.96E-13	ADAM23	ADAM metallopeptidase domain 23 [Source:HGNC Symbol;Acc:HGNC:202]
ENSG00000132639	178.143117	134.673833	13.69652	12.7746402	3.561676965	6.42E-15	3.96E-13	SNAP25	synaptosome associated protein 25 [Source:HGNC Symbol;Acc:HGNC:11132]
ENSG00000197457	249.006678	210.870871	37.9288246	40.4530273	2.552122599	6.56E-15	4.03E-13	STMN3	stathmin 3 [Source:HGNC Symbol;Acc:HGNC:15926]
ENSG00000182463	1249.95447	1212.0645	590.003939	469.468027	1.216280157	8.35E-15	5.11E-13	TSHZ2	teashirt zinc finger homeobox 2 [Source:HGNC Symbol;Acc:HGNC:13010]
ENSG00000135480	38252.5436	35076.3315	59610.4158	63463.348	-0.747087825	9.15E-15	5.58E-13	KRT7	keratin 7 [Source:HGNC Symbol;Acc:HGNC:6445]
ENSG00000204520	2070.79071	2089.21644	1020.91753	1115.65191	0.961353745	9.27E-15	5.62E-13	MICA	MHC class I polypeptide-related sequence A [Source:HGNC Symbol;Acc:HGNC:7090]
ENSG00000164574	1994.02186	1938.59439	1038.82836	976.195422	0.964589925	9.31E-15	5.62E-13	GALNT10	polypeptide N-acetylgalactosaminyltransferase 10 [Source:HGNC Symbol;Acc:HGNC:19873]
ENSG00000133131	835.599484	911.706412	374.020354	352.367159	1.266569831	9.32E-15	5.62E-13	MORC4	MORC family CW-type zinc finger 4 [Source:HGNC Symbol;Acc:HGNC:23485]
ENSG00000137699	3681.9525	3312.7991	6486.88259	6022.1783	-0.838825269	9.62E-15	5.78E-13	TRIM29	tripartite motif containing 29 [Source:HGNC Symbol;Acc:HGNC:17274]
ENSG00000168785	353.333586	408.45156	111.679317	76.6478412	2.016196974	1.00E-14	5.99E-13	TSPAN5	tetraspanin 5 [Source:HGNC Symbol;Acc:HGNC:17753]
ENSG00000116649	1507.81909	1403.44311	2668.71425	2763.5805	-0.900172324	1.01E-14	6.06E-13	SRM	spermidine synthase [Source:HGNC Symbol;Acc:HGNC:11296]
ENSG00000116991	794.262407	738.048047	311.859225	304.462258	1.313668945	1.28E-14	7.63E-13	SIPA1L2	signal induced proliferation associated 1 like 2 [Source:HGNC Symbol;Acc:HGNC:23800]
ENSG00000137261	200.780088	209.098847	33.7145108	30.8720472	2.665902072	1.43E-14	8.49E-13	KIAA0319	KIAA0319 [Source:HGNC Symbol;Acc:HGNC:21580]
ENSG00000277363	578.719078	509.456936	153.822455	196.94237	1.633143538	1.45E-14	8.59E-13	SRCIN1	SRC kinase signaling inhibitor 1 [Source:HGNC Symbol;Acc:HGNC:29506]
ENSG00000076382	2099.33298	2075.92626	3612.72055	3846.23125	-0.837128248	1.54E-14	9.07E-13	SPAG5	sperm associated antigen 5 [Source:HGNC Symbol;Acc:HGNC:13452]
ENSG00000109066	1044.2533	1066.75852	468.842416	485.436328	1.145548761	1.55E-14	9.10E-13	TMEM104	transmembrane protein 104 [Source:HGNC Symbol;Acc:HGNC:25984]
ENSG00000176907	808.041433	856.773664	303.430597	363.012692	1.321136022	1.58E-14	9.22E-13	TCIM	transcriptional and immune response regulator [Source:HGNC Symbol;Acc:HGNC:1357]
ENSG00000186480	4642.54743	4873.95235	2785.66145	2626.25312	0.814344365	1.69E-14	9.82E-13	INSIG1	insulin induced gene 1 [Source:HGNC Symbol;Acc:HGNC:6083]
ENSG00000129757	1351.32873	1285.6035	2378.98017	2703.96551	-0.946955653	1.82E-14	1.06E-12	CDKN1C	cyclin dependent kinase inhibitor 1C [Source:HGNC Symbol;Acc:HGNC:1786]
ENSG00000124126	2235.1548	2077.69828	1126.27538	1127.362	0.936214249	1.84E-14	1.07E-12	PREX1	phosphatidylinositol-3,4,5-trisphosphate dependent Rac exchange factor 1 [Source:HGNC Symbol;Acc:HGNC:32594]
ENSG00000164976	1248.97025	1242.18891	593.164674	590.827109	1.073138012	1.93E-14	1.11E-12	MYORG	myogenesis regulating glycosidase (putative) [Source:HGNC Symbol;Acc:HGNC:19918]
ENSG00000137959	135.821824	173.658364	488.860407	487.565434	-1.655381019	2.06E-14	1.19E-12	IFI44L	interferon induced protein 44 like [Source:HGNC Symbol;Acc:HGNC:17817]
ENSG00000041982	10787.0087	9525.51568	5982.21851	5555.90393	0.815871934	2.08E-14	1.19E-12	TNC	tenascin C [Source:HGNC Symbol;Acc:HGNC:5318]
ENSG00000119335	8099.11444	9505.1374	15218.9409	15907.6207	-0.822105046	2.09E-14	1.19E-12	SET	SET nuclear proto-oncogene [Source:HGNC Symbol;Acc:HGNC:10760]
ENSG00000168685	438.960389	445.664067	1021.97111	963.420782	-1.16620524	2.09E-14	1.19E-12	IL7R	interleukin 7 receptor [Source:HGNC Symbol;Acc:HGNC:6024]
ENSG00000012822	1356.24981	1282.94547	641.629284	622.76371	1.061464689	2.32E-14	1.32E-12	CALCOCO1	calcium binding and coiled-coil domain 1 [Source:HGNC Symbol;Acc:HGNC:29306]
ENSG00000146670	1135.7854	1110.17311	2110.31766	2126.97759	-0.915911692	2.38E-14	1.35E-12	CDCA5	cell division cycle associated 5 [Source:HGNC Symbol;Acc:HGNC:14626]
ENSG00000139289	834.615268	917.908496	372.966776	369.400013	1.239547797	2.41E-14	1.36E-12	PHLDA1	pleckstrin homology like domain family A member 1 [Source:HGNC Symbol;Acc:HGNC:8933]

ENSG00000138944	617.103506	557.301587	1266.40131	1231.68823	-1.089504913	2.87E-14	1.61E-12	SHISAL1	shisa like 1 [Source:HGNC Symbol;Acc:HGNC:29335]
ENSG00000131398	363.175748	337.570596	81.1255416	102.197122	1.934509044	2.97E-14	1.66E-12	KCNC3	potassium voltage-gated channel subfamily C member 3 [Source:HGNC Symbol;Acc:HGNC:6235]
ENSG00000162517	707.651389	693.747444	1372.81274	1514.85942	-1.043115834	3.01E-14	1.68E-12	PEF1	penta-EF-hand domain containing 1 [Source:HGNC Symbol;Acc:HGNC:30009]
ENSG00000138166	397.623312	375.669114	92.7149047	121.359082	1.853000996	3.41E-14	1.89E-12	DUSP5	dual specificity phosphatase 5 [Source:HGNC Symbol;Acc:HGNC:3071]
ENSG00000179950	3273.50281	2752.83948	5438.57202	5628.29356	-0.877200896	3.57E-14	1.98E-12	PUF60	poly(U) binding splicing factor 60 [Source:HGNC Symbol;Acc:HGNC:17042]
ENSG00000111640	81186.0192	77590.7343	124948.084	133460.924	-0.702661124	3.60E-14	1.98E-12	GAPDH	glyceraldehyde-3-phosphate dehydrogenase [Source:HGNC Symbol;Acc:HGNC:4141]
ENSG00000071246	842.488997	736.276023	324.502166	314.043238	1.305451266	3.60E-14	1.98E-12	VASH1	vasohibin 1 [Source:HGNC Symbol;Acc:HGNC:19964]
ENSG00000135678	1735.17302	1850.8792	893.434536	936.806948	0.970555639	3.65E-14	2.00E-12	CPM	carboxypeptidase M [Source:HGNC Symbol;Acc:HGNC:2311]
ENSG00000163235	473.407953	463.384308	153.822455	156.489342	1.593943247	3.84E-14	2.10E-12	TGFA	transforming growth factor alpha [Source:HGNC Symbol;Acc:HGNC:11765]
ENSG00000124006	619.071938	542.239382	1203.1866	1314.72339	-1.117187202	4.01E-14	2.18E-12	OBSL1	obscurin like 1 [Source:HGNC Symbol;Acc:HGNC:29092]
ENSG00000010404	988.152983	964.867135	420.377806	451.370621	1.163695099	4.52E-14	2.46E-12	IDS	iduronate 2-sulfatase [Source:HGNC Symbol;Acc:HGNC:5389]
ENSG00000074800	50309.1911	45008.5267	77387.4452	81745.9872	-0.739437901	5.59E-14	3.02E-12	ENO1	enolase 1 [Source:HGNC Symbol;Acc:HGNC:3350]
ENSG00000173272	1814.89452	1546.09105	3082.77058	3292.66351	-0.924085354	5.59E-14	3.02E-12	MZT2A	mitotic spindle organizing protein 2A [Source:HGNC Symbol;Acc:HGNC:33187]
ENSG00000164638	458.644711	397.819416	106.411425	141.585596	1.78787904	6.02E-14	3.24E-12	SLC29A4	solute carrier family 29 member 4 [Source:HGNC Symbol;Acc:HGNC:23097]
ENSG00000141510	996.026712	925.882605	1842.70873	1860.83926	-0.946708185	6.07E-14	3.26E-12	TP53	tumor protein p53 [Source:HGNC Symbol;Acc:HGNC:11998]
ENSG00000182199	4527.39414	4057.04923	7244.40551	7626.4602	-0.792854518	6.18E-14	3.30E-12	SHMT2	serine hydroxymethyltransferase 2 [Source:HGNC Symbol;Acc:HGNC:10852]
ENSG00000132589	2095.39612	1829.61491	3538.97005	3651.41799	-0.873737722	6.25E-14	3.33E-12	FLOT2	flotillin 2 [Source:HGNC Symbol;Acc:HGNC:3758]
ENSG00000197380	166.332524	126.699725	2.10715692	2.1291067	6.111253323	6.72E-14	3.57E-12	DACT3	dishevelled binding antagonist of beta catenin 3 [Source:HGNC Symbol;Acc:HGNC:30745]
ENSG00000049323	2899.50068	2612.84957	1528.74235	1411.59774	0.906459422	6.83E-14	3.62E-12	LTBP1	latent transforming growth factor beta binding protein 1 [Source:HGNC Symbol;Acc:HGNC:6714]
ENSG00000108518	11840.1199	10012.8223	18467.1233	20956.7973	-0.851326726	7.12E-14	3.76E-12	PFN1	profilin 1 [Source:HGNC Symbol;Acc:HGNC:8881]
ENSG00000071539	1540.29822	1519.51069	2745.62547	2746.54764	-0.843985912	7.90E-14	4.16E-12	TRIP13	thyroid hormone receptor interactor 13 [Source:HGNC Symbol;Acc:HGNC:12307]
ENSG00000175745	526.555623	518.317056	180.161917	191.619603	1.490800004	8.46E-14	4.43E-12	NR2F1	nuclear receptor subfamily 2 group F member 1 [Source:HGNC Symbol;Acc:HGNC:7975]
ENSG00000149218	421.244499	399.59144	132.750886	121.359082	1.691329477	8.47E-14	4.43E-12	ENDOD1	endonuclease domain containing 1 [Source:HGNC Symbol;Acc:HGNC:29129]
ENSG00000135829	5519.48399	5712.11976	9230.40091	9264.80781	-0.719539177	8.49E-14	4.43E-12	DHX9	DExH-box helicase 9 [Source:HGNC Symbol;Acc:HGNC:2750]
ENSG00000099956	971.421309	890.442122	1758.42245	1871.48479	-0.963570272	8.85E-14	4.60E-12	SMARCB1	SWI/SNF related, matrix associated, actin dependent regulator of chromatin, subfamily b, member 1 [Source:HGNC Symbol;Acc:HGNC:11103]
ENSG00000117724	3578.60981	3952.49981	6398.382	6533.16391	-0.779782009	9.31E-14	4.82E-12	CENPF	centromere protein F [Source:HGNC Symbol;Acc:HGNC:1857]
ENSG00000100823	3289.25027	3233.05801	5416.44687	5608.06705	-0.757292819	9.42E-14	4.87E-12	APEX1	apurinic/apyrimidinic endodeoxyribonuclease 1 [Source:HGNC Symbol;Acc:HGNC:587]
ENSG00000188486	2708.56276	2757.26954	4602.03072	4729.81054	-0.771671786	9.51E-14	4.90E-12	H2AFX	H2A histone family member X [Source:HGNC Symbol;Acc:HGNC:4739]
ENSG00000137440	558.050539	475.788477	177.001182	177.780409	1.542424151	9.70E-14	4.98E-12	FGFBP1	fibroblast growth factor binding protein 1 [Source:HGNC Symbol;Acc:HGNC:19695]
ENSG00000029363	1334.59706	1608.9979	3112.27078	2724.19202	-0.987015342	1.03E-13	5.27E-12	BCLAF1	BCL2 associated transcription factor 1 [Source:HGNC Symbol;Acc:HGNC:16863]
ENSG00000197111	2757.77356	2861.81896	4692.63847	4958.68951	-0.780166203	1.09E-13	5.57E-12	PCBP2	poly(rC) binding protein 2 [Source:HGNC Symbol;Acc:HGNC:8648]
ENSG00000023191	2007.80088	1692.28304	3400.95128	3509.8324	-0.901740595	1.14E-13	5.82E-12	RNH1	ribonuclease/angiogenin inhibitor 1 [Source:HGNC Symbol;Acc:HGNC:10074]
ENSG00000147889	7117.85097	6258.7892	11096.2884	12257.2673	-0.804040637	1.15E-13	5.82E-12	CDKN2A	cyclin dependent kinase inhibitor 2A [Source:HGNC Symbol;Acc:HGNC:1787]
ENSG00000135048	1227.3175	1397.24102	643.73644	596.149876	1.082180219	1.19E-13	6.03E-12	CEMIP2	cell migration inducing hyaluronidase 2 [Source:HGNC Symbol;Acc:HGNC:11869]
ENSG00000157456	1690.88329	1828.7289	3065.91333	3289.46985	-0.852304763	1.30E-13	6.53E-12	CCNB2	cyclin B2 [Source:HGNC Symbol;Acc:HGNC:1580]
ENSG00000187689	33.463348	57.590784	225.465791	272.525658	-2.444497203	1.39E-13	6.97E-12	AMTN	amelotin [Source:HGNC Symbol;Acc:HGNC:33188]
ENSG00000138772	686.982851	841.711459	1604.6	1585.11994	-1.060210242	1.42E-13	7.10E-12	ANXA3	annexin A3 [Source:HGNC Symbol;Acc:HGNC:541]
ENSG00000175832	1188.93307	1067.64453	536.271437	512.050161	1.105691669	1.45E-13	7.22E-12	ETV4	ETS variant 4 [Source:HGNC Symbol;Acc:HGNC:3493]
ENSG00000167779	3240.03946	2661.58023	5290.01746	5641.0682	-0.889581938	1.51E-13	7.55E-12	IGFBP6	insulin like growth factor binding protein 6 [Source:HGNC Symbol;Acc:HGNC:5475]
ENSG00000146950	333.649264	357.948873	80.0719631	105.390782	1.899388669	1.56E-13	7.76E-12	SHROOM2	shroom family member 2 [Source:HGNC Symbol;Acc:HGNC:630]
ENSG00000164548	573.797997	539.581346	1163.15062	1158.23405	-1.06042134	1.62E-13	8.04E-12	TRA2A	transformer 2 alpha homolog [Source:HGNC Symbol;Acc:HGNC:16645]
ENSG00000101216	567.8927	519.203068	1143.13263	1145.4594	-1.074530909	1.72E-13	8.49E-12	GMEB2	glucocorticoid modulatory element binding protein 2 [Source:HGNC Symbol;Acc:HGNC:4371]
ENSG00000170144	5856.0859	6376.62881	9926.81627	10729.6332	-0.755744887	1.76E-13	8.69E-12	HNRNPA3	heterogeneous nuclear ribonucleoprotein A3 [Source:HGNC Symbol;Acc:HGNC:24941]
ENSG00000110321	14900.0478	16580.8297	28035.7229	25300.1749	-0.760587392	1.82E-13	8.92E-12	EIF4G2	eukaryotic translation initiation factor 4 gamma 2 [Source:HGNC Symbol;Acc:HGNC:3297]
ENSG00000069011	3172.12855	2992.06273	5173.07025	5281.24917	-0.762241617	1.85E-13	9.04E-12	PITX1	paired like homeodomain 1 [Source:HGNC Symbol;Acc:HGNC:9004]
ENSG00000161847	854.299591	795.638831	1585.63559	1629.83118	-0.962968084	1.90E-13	9.28E-12	RAVER1	ribonucleoprotein, PTB binding 1 [Source:HGNC Symbol;Acc:HGNC:30296]
ENSG00000069275	3606.16786	4679.0297	8791.05869	7544.48959	-0.979157268	1.93E-13	9.38E-12	NUCKS1	nuclear casein kinase and cyclin dependent kinase substrate 1 [Source:HGNC Symbol;Acc:HGNC:29923]
ENSG00000132967	1086.57459	1111.05913	1967.03099	2331.37184	-0.967727145	2.01E-13	9.75E-12	HMGB1P5	high mobility group box 1 pseudogene 5 [Source:HGNC Symbol;Acc:HGNC:4997]
ENSG00000149823	653.519502	629.068564	1271.6692	1320.04615	-1.015057822	2.22E-13	1.08E-11	VPS51	VPS51, GARP complex subunit [Source:HGNC Symbol;Acc:HGNC:1172]
ENSG00000274523	965.516012	859.4317	1793.19054	1750.12571	-0.957788072	2.34E-13	1.13E-11	RCC1L	RCC1 like [Source:HGNC Symbol;Acc:HGNC:14948]
ENSG00000140450	475.376385	511.22896	178.05476	109.648995	1.77767145	2.46E-13	1.19E-11	ARRDC4	arrestin domain containing 4 [Source:HGNC Symbol;Acc:HGNC:28087]

ENSG00000050165	606.277129	660.078986	249.698096	249.105484	1.344457617	2.49E-13	1.20E-11	DKK3	dickkopf WNT signaling pathway inhibitor 3 [Source:HGNC Symbol;Acc:HGNC:2893]
ENSG00000273760	200.780088	194.036642	543.646487	521.631142	-1.432302525	2.52E-13	1.20E-11	AC245041.1	novel transcript
ENSG00000204673	988.152983	855.00164	1780.5476	1834.22542	-0.972358834	2.54E-13	1.21E-11	AKT1S1	AKT1 substrate 1 [Source:HGNC Symbol;Acc:HGNC:28426]
ENSG00000156650	383.844286	378.32715	875.523702	837.803487	-1.168735323	2.56E-13	1.22E-11	KAT6B	lysine acetyltransferase 6B [Source:HGNC Symbol;Acc:HGNC:17582]
ENSG00000066117	1542.26666	1478.75413	2659.23204	2735.90211	-0.836770013	2.62E-13	1.24E-11	SMARCD1	SWI/SNF related, matrix associated, actin dependent regulator of chromatin, subfamily d, member 1 [Source:HGNC Symbol;Acc:HGNC:11106]
ENSG00000185291	171.253605	139.103894	10.5357846	21.291067	3.28622665	2.79E-13	1.32E-11	IL3RA	interleukin 3 receptor subunit alpha [Source:HGNC Symbol;Acc:HGNC:6012]
ENSG00000130517	1496.99272	1267.88326	642.682862	677.055931	1.066559862	3.34E-13	1.57E-11	PGPEP1	pyroglutamyl-peptidase I [Source:HGNC Symbol;Acc:HGNC:13568]
ENSG00000105323	5756.68008	5055.58482	8967.00629	9638.46603	-0.783204732	3.36E-13	1.58E-11	HNRNPUL1	heterogeneous nuclear ribonucleoprotein U like 1 [Source:HGNC Symbol;Acc:HGNC:17011]
ENSG00000119125	3436.88268	4216.5314	2107.15692	1810.80525	0.966191033	3.38E-13	1.59E-11	GDA	guanine deaminase [Source:HGNC Symbol;Acc:HGNC:4212]
ENSG00000151726	1482.22947	1668.36071	823.898358	726.025385	1.023650196	3.63E-13	1.70E-11	ACSL1	acyl-CoA synthetase long chain family member 1 [Source:HGNC Symbol;Acc:HGNC:3569]
ENSG00000055163	256.880407	264.031594	66.3754431	51.0985608	2.148449171	3.78E-13	1.76E-11	CYFIP2	cytoplasmic FMR1 interacting protein 2 [Source:HGNC Symbol;Acc:HGNC:13760]
ENSG00000168488	2143.62271	2089.21644	3536.8629	3861.135	-0.805564913	4.05E-13	1.88E-11	ATXN2L	ataxin 2 like [Source:HGNC Symbol;Acc:HGNC:31326]
ENSG00000131116	137.790257	124.041689	429.860013	386.432866	-1.641547747	4.31E-13	2.00E-11	ZNF428	zinc finger protein 428 [Source:HGNC Symbol;Acc:HGNC:20804]
ENSG00000159217	775.562301	757.540313	1442.34891	1560.63521	-0.970040269	4.34E-13	2.01E-11	IGF2BP1	insulin like growth factor 2 mRNA binding protein 1 [Source:HGNC Symbol;Acc:HGNC:28866]
ENSG00000008838	965.516012	910.8204	1747.88667	1795.9015	-0.917643898	4.50E-13	2.08E-11	MED24	mediator complex subunit 24 [Source:HGNC Symbol;Acc:HGNC:22963]
ENSG00000084774	2580.61466	2336.41381	4578.852	4134.72521	-0.825719911	4.53E-13	2.09E-11	CAD	carbamoyl-phosphate synthetase 2, aspartate transcarbamylase, and dihydroorotase [Source:HGNC Symbol;Acc:HGNC:1424]
ENSG00000103202	753.909547	748.680192	1418.11661	1523.37584	-0.969111133	4.82E-13	2.22E-11	NME4	NME/NM23 nucleoside diphosphate kinase 4 [Source:HGNC Symbol;Acc:HGNC:7852]
ENSG00000050438	329.7124	346.430716	105.357846	73.4541812	1.918649857	4.91E-13	2.25E-11	SLC4A8	solute carrier family 4 member 8 [Source:HGNC Symbol;Acc:HGNC:11034]
ENSG00000134243	1966.4638	1890.74974	1065.16783	872.933747	0.99274618	5.12E-13	2.34E-11	SORT1	sortilin 1 [Source:HGNC Symbol;Acc:HGNC:11186]
ENSG00000006047	244.085597	195.808666	46.3574523	35.1302606	2.431281011	5.18E-13	2.36E-11	YBX2	Y-box binding protein 2 [Source:HGNC Symbol;Acc:HGNC:17948]
ENSG00000143344	631.866748	610.462311	240.215889	255.492804	1.325406154	5.43E-13	2.46E-11	RGL1	ral guanine nucleotide dissociation stimulator like 1 [Source:HGNC Symbol;Acc:HGNC:30281]
ENSG00000147872	3842.37973	3574.17266	2067.12094	2187.65713	0.801563206	5.44E-13	2.46E-11	PLIN2	perilipin 2 [Source:HGNC Symbol;Acc:HGNC:248]
ENSG00000160818	772.609653	735.390011	1413.9023	1568.08708	-0.983858861	5.88E-13	2.66E-11	GPATCH4	G-patch domain containing 4 [Source:HGNC Symbol;Acc:HGNC:25982]
ENSG00000142089	4093.35484	3449.24496	6473.18607	6915.33856	-0.828107902	5.93E-13	2.67E-11	IFITM3	interferon induced transmembrane protein 3 [Source:HGNC Symbol;Acc:HGNC:5414]
ENSG00000170921	2472.35089	2452.48139	1421.27735	1252.97929	0.880869559	5.94E-13	2.67E-11	TANC2	tetratricopeptide repeat, ankyrin repeat and coiled-coil containing 2 [Source:HGNC Symbol;Acc:HGNC:30212]
ENSG00000223501	311.012293	283.52386	782.808798	670.668611	-1.290407524	5.95E-13	2.67E-11	VPS52	VPS52, GARP complex subunit [Source:HGNC Symbol;Acc:HGNC:10518]
ENSG00000100285	2719.38913	2636.7719	4393.42219	5174.79384	-0.837091894	6.02E-13	2.69E-11	NEFH	neurofilament heavy [Source:HGNC Symbol;Acc:HGNC:7737]
ENSG00000142507	2199.72302	1871.25747	3574.79172	3884.55518	-0.874052287	6.05E-13	2.70E-11	PSMB6	proteasome subunit beta 6 [Source:HGNC Symbol;Acc:HGNC:9543]
ENSG00000159403	1671.19897	1453.94579	2799.35797	2916.87618	-0.871573809	6.30E-13	2.80E-11	C1R	complement C1r [Source:HGNC Symbol;Acc:HGNC:1246]
ENSG00000101224	4592.35241	4236.90968	7257.04845	7375.22561	-0.728917067	7.02E-13	3.11E-11	CDC25B	cell division cycle 25B [Source:HGNC Symbol;Acc:HGNC:1726]
ENSG00000186832	54.1318865	46.0726272	225.465791	235.26629	-2.203131742	7.95E-13	3.52E-11	KRT16	keratin 16 [Source:HGNC Symbol;Acc:HGNC:6423]
ENSG00000164825	154.521931	180.746461	17.9108339	27.6783871	2.879886167	8.07E-13	3.56E-11	DEFB1	defensin beta 1 [Source:HGNC Symbol;Acc:HGNC:2766]
ENSG00000137941	376.954773	471.358417	134.858043	134.133722	1.657838906	8.15E-13	3.59E-11	TTLL7	tubulin tyrosine ligase like 7 [Source:HGNC Symbol;Acc:HGNC:26242]
ENSG00000165671	2251.88648	2170.72955	4082.61654	3655.6762	-0.807229089	8.28E-13	3.63E-11	NSD1	nuclear receptor binding SET domain protein 1 [Source:HGNC Symbol;Acc:HGNC:14234]
ENSG00000049449	722.414631	790.322759	1499.24215	1454.17988	-0.964769388	8.81E-13	3.85E-11	RCN1	reticulocalbin 1 [Source:HGNC Symbol;Acc:HGNC:9934]
ENSG00000062282	757.846411	643.244757	283.412606	286.364851	1.297523417	8.82E-13	3.85E-11	DGAT2	diacylglycerol O-acyltransferase 2 [Source:HGNC Symbol;Acc:HGNC:16940]
ENSG00000124466	2794.18956	2380.71441	1281.15141	1431.82426	0.93141943	8.92E-13	3.88E-11	LYPD3	LY6/PLAUR domain containing 3 [Source:HGNC Symbol;Acc:HGNC:24880]
ENSG00000095752	630.882532	532.493249	175.947603	233.137184	1.507553216	9.14E-13	3.97E-11	IL11	interleukin 11 [Source:HGNC Symbol;Acc:HGNC:5966]
ENSG00000170421	34897.3509	30543.4938	52592.5297	55597.3633	-0.725330738	9.25E-13	4.01E-11	KRT8	keratin 8 [Source:HGNC Symbol;Acc:HGNC:6446]
ENSG00000101210	5657.27425	4910.27885	8732.0583	9417.03894	-0.7804148	9.64E-13	4.17E-11	EEF1A2	eukaryotic translation elongation factor 1 alpha 2 [Source:HGNC Symbol;Acc:HGNC:3192]
ENSG00000175505	223.417059	185.176521	522.574917	584.439789	-1.439448095	9.69E-13	4.18E-11	CLCF1	cardiotrophin like cytokine factor 1 [Source:HGNC Symbol;Acc:HGNC:17412]
ENSG00000103489	330.696616	342.000656	105.357846	88.3579281	1.795942104	9.84E-13	4.23E-11	XYLT1	xylosyltransferase 1 [Source:HGNC Symbol;Acc:HGNC:15516]
ENSG00000111328	2119.0173	2095.41853	3546.3451	3621.6105	-0.766251495	9.92E-13	4.26E-11	CDK2AP1	cyclin dependent kinase 2 associated protein 1 [Source:HGNC Symbol;Acc:HGNC:14002]
ENSG00000088340	235.227652	201.124738	46.3574523	40.4530273	2.328733311	1.00E-12	4.29E-11	FER1L4	fer-1 like family member 4, pseudogene [Source:HGNC Symbol;Acc:HGNC:15801]
ENSG00000158825	2259.76021	2044.02983	3624.30991	4448.76845	-0.907693872	1.16E-12	4.97E-11	CDA	cytidine deaminase [Source:HGNC Symbol;Acc:HGNC:1712]
ENSG00000160233	273.612081	240.109269	61.1075508	60.679541	2.076087398	1.18E-12	5.04E-11	LRRC3	leucine rich repeat containing 3 [Source:HGNC Symbol;Acc:HGNC:14965]
ENSG00000104611	616.11929	637.928684	1189.49008	1423.30783	-1.058772044	1.19E-12	5.04E-11	SH2D4A	SH2 domain containing 4A [Source:HGNC Symbol;Acc:HGNC:26102]
ENSG00000056097	1468.45045	1585.96159	3010.07367	2612.41392	-0.880103118	1.19E-12	5.04E-11	ZFR	zinc finger RNA binding protein [Source:HGNC Symbol;Acc:HGNC:17277]
ENSG00000117016	393.686447	360.606909	116.947209	121.359082	1.661993977	1.28E-12	5.39E-11	RIMS3	regulating synaptic membrane exocytosis 3 [Source:HGNC Symbol;Acc:HGNC:21292]
ENSG00000140044	440.928821	429.71585	881.845173	1046.45594	-1.147276335	1.32E-12	5.58E-11	JDP2	Jun dimerization protein 2 [Source:HGNC Symbol;Acc:HGNC:17546]
ENSG00000138685	804.104569	930.312665	1929.10216	1607.47556	-1.027369566	1.37E-12	5.74E-11	FGF2	fibroblast growth factor 2 [Source:HGNC Symbol;Acc:HGNC:3676]

ENSG00000128272	6877.70223	6550.28717	10528.4096	11237.4252	-0.696871884	1.54E-12	6.46E-11	ATF4	activating transcription factor 4 [Source:HGNC Symbol;Acc:HGNC:786]
ENSG00000186185	1035.39536	935.628737	1830.06579	1854.45194	-0.902979993	1.58E-12	6.61E-11	KIF18B	kinesin family member 18B [Source:HGNC Symbol;Acc:HGNC:27102]
ENSG00000171310	1270.62301	1218.26658	633.200656	620.634603	0.989007589	1.59E-12	6.64E-11	CHST11	carbohydrate sulfotransferase 11 [Source:HGNC Symbol;Acc:HGNC:17422]
ENSG00000092096	460.613143	378.32715	125.375837	143.714702	1.63989857	1.69E-12	7.03E-11	SLC22A17	solute carrier family 22 member 17 [Source:HGNC Symbol;Acc:HGNC:23095]
ENSG00000196352	12840.0835	13632.1816	8202.10833	8363.13112	0.676361077	1.76E-12	7.30E-11	CD55	CD55 molecule (Cromer blood group) [Source:HGNC Symbol;Acc:HGNC:2665]
ENSG00000274180	347.42829	300.358089	94.8220616	89.4224814	1.813247052	1.77E-12	7.32E-11	NATD1	N-acetyltransferase domain containing 1 [Source:HGNC Symbol;Acc:HGNC:30770]
ENSG00000108349	1374.94992	1403.44311	2426.3912	2462.3119	-0.81511752	1.82E-12	7.53E-11	CASC3	CASC3, exon junction complex subunit [Source:HGNC Symbol;Acc:HGNC:17040]
ENSG00000169891	501.95022	474.016453	184.376231	174.586749	1.44271346	1.87E-12	7.70E-11	REPS2	RALBP1 associated Eps domain containing 2 [Source:HGNC Symbol;Acc:HGNC:9963]
ENSG00000109501	2550.10396	2376.28435	1391.77715	1378.59659	0.83028924	1.90E-12	7.80E-11	WFS1	wolframin ER transmembrane glycoprotein [Source:HGNC Symbol;Acc:HGNC:12762]
ENSG00000158458	169.285172	155.052111	22.1251477	25.5492804	2.766112413	2.00E-12	8.22E-11	NRG2	neuregulin 2 [Source:HGNC Symbol;Acc:HGNC:7998]
ENSG00000103260	2137.71741	2134.40306	1169.47209	1213.59082	0.842148612	2.09E-12	8.57E-11	METRN	meteorin, glial cell differentiation regulator [Source:HGNC Symbol;Acc:HGNC:14151]
ENSG00000196531	4450.62529	4606.37671	7147.47629	7676.49421	-0.710764367	2.16E-12	8.81E-11	NACA	nascent polypeptide associated complex subunit alpha [Source:HGNC Symbol;Acc:HGNC:7629]
ENSG00000151348	5798.01715	5643.89683	3532.64858	3470.44392	0.708225621	2.20E-12	8.95E-11	EXT2	exostosin glycosyltransferase 2 [Source:HGNC Symbol;Acc:HGNC:3513]
ENSG00000125520	2216.4547	1901.38188	3631.68496	3668.45084	-0.826425718	2.24E-12	9.08E-11	SLC2A4RG	SLC2A4 regulator [Source:HGNC Symbol;Acc:HGNC:15930]
ENSG00000167653	1781.43117	1686.08095	2959.5019	3023.33151	-0.787105802	2.46E-12	9.95E-11	PSCA	prostate stem cell antigen [Source:HGNC Symbol;Acc:HGNC:9500]
ENSG00000259884	108.263773	107.207459	2.10715692	6.3873201	4.667605786	2.62E-12	1.06E-10	AC025259.3	novel transcript
ENSG00000116771	105.311125	123.155677	350.841628	376.851886	-1.669705135	2.71E-12	1.09E-10	AGMAT	agmatinase [Source:HGNC Symbol;Acc:HGNC:18407]
ENSG00000108561	3543.17803	3686.69619	5751.48483	6734.36449	-0.788171934	2.78E-12	1.12E-10	C1QBP	complement C1q binding protein [Source:HGNC Symbol;Acc:HGNC:1243]
ENSG00000100302	418.29185	356.176849	99.0363755	134.133722	1.731575413	3.06E-12	1.23E-10	RASD2	RASD family member 2 [Source:HGNC Symbol;Acc:HGNC:18229]
ENSG00000147403	8787.0815	7692.35672	13235.0526	14294.8224	-0.740432073	3.08E-12	1.23E-10	RPL10	ribosomal protein L10 [Source:HGNC Symbol;Acc:HGNC:10298]
ENSG00000105376	839.536349	650.332853	276.037557	315.107792	1.332958506	3.16E-12	1.26E-10	ICAM5	intercellular adhesion molecule 5 [Source:HGNC Symbol;Acc:HGNC:5348]
ENSG00000163590	604.308697	695.519469	282.359028	227.814417	1.349547957	3.35E-12	1.33E-10	PPM1L	protein phosphatase, Mg2+/Mn2+ dependent 1L [Source:HGNC Symbol;Acc:HGNC:16381]
ENSG00000135451	978.310821	894.872182	1673.0826	1921.5188	-0.940679667	3.43E-12	1.36E-10	TROAP	trophinin associated protein [Source:HGNC Symbol;Acc:HGNC:12327]
ENSG00000126749	1007.83731	869.177833	1722.60079	1889.5822	-0.945022594	3.46E-12	1.37E-10	EMG1	EMG1, N1-specific pseudouridine methyltransferase [Source:HGNC Symbol;Acc:HGNC:16912]
ENSG00000278259	1638.71984	1520.3967	2738.25042	2763.5805	-0.800648177	3.55E-12	1.41E-10	MYO19	myosin XIX [Source:HGNC Symbol;Acc:HGNC:26234]
ENSG00000111665	757.846411	735.390011	1362.27695	1557.44155	-0.967495677	3.71E-12	1.46E-10	CDCA3	cell division cycle associated 3 [Source:HGNC Symbol;Acc:HGNC:14624]
ENSG00000272888	442.897253	579.451888	1231.63322	1072.00522	-1.170752561	3.80E-12	1.50E-10	LINC01578	long intergenic non-protein coding RNA 1578 [Source:HGNC Symbol;Acc:HGNC:48626]
ENSG00000167996	55198.7768	57498.6388	85248.1941	97588.6702	-0.698096152	4.01E-12	1.58E-10	FTH1	ferritin heavy chain 1 [Source:HGNC Symbol;Acc:HGNC:3976]
ENSG00000143401	1392.66581	1609.88392	2767.75062	2641.15686	-0.848696264	5.09E-12	1.99E-10	ANP32E	acidic nuclear phosphoprotein 32 family member E [Source:HGNC Symbol;Acc:HGNC:16673]
ENSG00000204304	690.919715	623.752492	1241.11543	1371.14472	-0.991103008	5.25E-12	2.05E-10	PBX2	PBX homeobox 2 [Source:HGNC Symbol;Acc:HGNC:8633]
ENSG00000149182	669.26696	551.099502	1218.99028	1296.62598	-1.044569704	5.26E-12	2.05E-10	ARFGAP2	ADP ribosylation factor GTPase activating protein 2 [Source:HGNC Symbol;Acc:HGNC:13504]
ENSG00000103495	915.32099	940.94481	1728.92226	1674.54242	-0.874477653	5.26E-12	2.05E-10	MAZ	MYC associated zinc finger protein [Source:HGNC Symbol;Acc:HGNC:6914]
ENSG00000115380	6307.8411	6272.07939	10099.6031	9769.4061	-0.659407435	5.74E-12	2.23E-10	EFEMP1	EGF containing fibulin extracellular matrix protein 1 [Source:HGNC Symbol;Acc:HGNC:3218]
ENSG00000131016	3814.82167	3709.7325	2273.62232	2246.20757	0.735284767	5.75E-12	2.23E-10	AKAP12	A-kinase anchoring protein 12 [Source:HGNC Symbol;Acc:HGNC:370]
ENSG00000150907	249.006678	262.25957	70.589757	55.3567742	2.021152373	5.78E-12	2.23E-10	FOXO1	forkhead box O1 [Source:HGNC Symbol;Acc:HGNC:3819]
ENSG00000011347	736.193656	654.762914	291.841234	304.462258	1.221567709	5.79E-12	2.24E-10	SYT7	synaptotagmin 7 [Source:HGNC Symbol;Acc:HGNC:11514]
ENSG00000205220	42.3212931	36.3264945	178.05476	219.29799	-2.338978731	5.86E-12	2.25E-10	PSMB10	proteasome subunit beta 10 [Source:HGNC Symbol;Acc:HGNC:9538]
ENSG00000141101	933.03688	884.240038	1626.72515	1744.80294	-0.891856742	5.89E-12	2.26E-10	NOB1	NIN1 (RPN12) binding protein 1 homolog [Source:HGNC Symbol;Acc:HGNC:29540]
ENSG00000121743	190.937927	149.736038	31.6073539	19.1619603	2.744701902	6.86E-12	2.63E-10	GJA3	gap junction protein alpha 3 [Source:HGNC Symbol;Acc:HGNC:4277]
ENSG00000181163	12264.317	15917.2067	25561.9207	24697.6377	-0.834558239	7.83E-12	2.99E-10	NPM1	nucleophosmin 1 [Source:HGNC Symbol;Acc:HGNC:7910]
ENSG00000175274	1623.9566	1556.72319	860.773604	861.22366	0.885130652	8.05E-12	3.07E-10	TP53I11	tumor protein p53 inducible protein 11 [Source:HGNC Symbol;Acc:HGNC:16842]
ENSG00000166986	4110.08651	3860.35455	6296.18489	6694.97602	-0.704899836	8.16E-12	3.11E-10	MARS	methionyl-tRNA synthetase [Source:HGNC Symbol;Acc:HGNC:6898]
ENSG00000068079	177.158901	179.860449	442.502954	516.308375	-1.425045949	9.04E-12	3.43E-10	IFI35	interferon induced protein 35 [Source:HGNC Symbol;Acc:HGNC:5399]
ENSG00000101447	1330.66019	1229.78474	2254.65791	2260.04676	-0.818545777	9.10E-12	3.45E-10	FAM83D	family with sequence similarity 83 member D [Source:HGNC Symbol;Acc:HGNC:16122]
ENSG00000065320	226.369707	158.596159	33.7145108	35.1302606	2.482098186	9.90E-12	3.74E-10	NTN1	netrin 1 [Source:HGNC Symbol;Acc:HGNC:8029]
ENSG00000198825	1149.56443	1295.34963	625.825607	605.730856	0.989638114	9.91E-12	3.74E-10	INPP5F	inositol polyphosphate-5-phosphatase F [Source:HGNC Symbol;Acc:HGNC:17054]
ENSG00000205426	1074.764	893.100158	1776.33329	2137.62313	-0.992596409	1.06E-11	4.01E-10	KRT81	keratin 81 [Source:HGNC Symbol;Acc:HGNC:6458]
ENSG00000130956	843.473213	682.229288	306.591333	339.592519	1.238873081	1.07E-11	4.02E-10	HABP4	hyaluronan binding protein 4 [Source:HGNC Symbol;Acc:HGNC:17062]
ENSG00000081923	238.180301	257.82951	59.0003939	66.0023077	1.988884135	1.11E-11	4.16E-10	ATP8B1	ATPase phospholipid transporting 8B1 [Source:HGNC Symbol;Acc:HGNC:3706]
ENSG00000173221	1362.15511	1403.44311	712.219041	751.574665	0.918001598	1.21E-11	4.54E-10	GLRX	glutaredoxin [Source:HGNC Symbol;Acc:HGNC:4330]
ENSG00000121064	3104.21764	3022.18714	1653.06461	1873.6139	0.796724215	1.25E-11	4.67E-10	SCPEP1	serine carboxypeptidase 1 [Source:HGNC Symbol;Acc:HGNC:29507]

ENSG00000104936	678.124905	603.374214	1229.52607	1277.46402	-0.968747559	1.27E-11	4.74E-10	DMPK	DM1 protein kinase [Source:HGNC Symbol;Acc:HGNC:2933]
ENSG00000076641	191.922143	183.404497	29.5001969	41.5175807	2.402232552	1.40E-11	5.19E-10	PAG1	phosphoprotein membrane anchor with glycosphingolipid microdomains 1 [Source:HGNC Symbol;Acc:HGNC:30043]
ENSG00000265190	383.844286	405.793524	792.291004	932.548735	-1.126783414	1.43E-11	5.28E-10	ANXA8	annexin A8 [Source:HGNC Symbol;Acc:HGNC:546]
ENSG00000162384	322.822887	291.497968	698.522521	688.766018	-1.175977273	1.45E-11	5.35E-10	C1orf123	chromosome 1 open reading frame 123 [Source:HGNC Symbol;Acc:HGNC:26059]
ENSG00000126016	200.780088	239.223257	49.5181877	50.0340075	2.144706234	1.48E-11	5.47E-10	AMOT	angiomotin [Source:HGNC Symbol;Acc:HGNC:17810]
ENSG00000120756	749.972682	902.846291	383.50256	350.238052	1.172104612	1.51E-11	5.54E-10	PLS1	plastin 1 [Source:HGNC Symbol;Acc:HGNC:9090]
ENSG00000168077	2640.65185	2380.71441	4163.74208	4230.53501	-0.741572671	1.51E-11	5.56E-10	SCARA3	scavenger receptor class A member 3 [Source:HGNC Symbol;Acc:HGNC:19000]
ENSG00000091129	159.443011	174.544376	32.6609323	22.3556204	2.601590825	1.53E-11	5.59E-10	NRCAM	neuronal cell adhesion molecule [Source:HGNC Symbol;Acc:HGNC:7994]
ENSG00000188191	666.314312	564.389683	1220.04386	1234.88189	-0.997072707	1.55E-11	5.66E-10	PRKAR1B	protein kinase cAMP-dependent type I regulatory subunit beta [Source:HGNC Symbol;Acc:HGNC:9390]
ENSG00000148053	103.342692	106.321447	8.4286277	1.06455335	4.46107709	1.63E-11	5.96E-10	NTRK2	neurotrophic receptor tyrosine kinase 2 [Source:HGNC Symbol;Acc:HGNC:8032]
ENSG00000125944	4786.24298	5085.70923	7920.80288	7772.30401	-0.668634383	1.65E-11	6.00E-10	HNRNPR	heterogeneous nuclear ribonucleoprotein R [Source:HGNC Symbol;Acc:HGNC:5047]
ENSG00000165689	1435.97132	1336.9922	2335.78345	2529.37876	-0.811297537	1.66E-11	6.04E-10	ENTR1	endosome associated trafficking regulator 1 [Source:HGNC Symbol;Acc:HGNC:10667]
ENSG00000101940	806.073001	622.866479	1424.43808	1497.82656	-1.03322495	1.71E-11	6.20E-10	WDR13	WD repeat domain 13 [Source:HGNC Symbol;Acc:HGNC:14352]
ENSG00000078804	688.951283	736.276023	289.734077	335.334305	1.189456793	1.76E-11	6.35E-10	TP53INP2	tumor protein p53 inducible nuclear protein 2 [Source:HGNC Symbol;Acc:HGNC:16104]
ENSG00000139629	608.245561	507.684911	219.14432	222.49165	1.336674191	1.80E-11	6.50E-10	GALNT6	polypeptide N-acetylgalactosaminyltransferase 6 [Source:HGNC Symbol;Acc:HGNC:4128]
ENSG00000106327	176.174685	131.129785	445.66369	435.40232	-1.522148119	1.81E-11	6.52E-10	TFR2	transferrin receptor 2 [Source:HGNC Symbol;Acc:HGNC:11762]
ENSG00000189403	4054.97041	4940.40326	7761.71253	7663.71957	-0.777818201	1.96E-11	7.06E-10	HMGB1	high mobility group box 1 [Source:HGNC Symbol;Acc:HGNC:4983]
ENSG00000081377	762.767492	831.079314	342.413	385.368313	1.131321525	2.01E-11	7.20E-10	CDC14B	cell division cycle 14B [Source:HGNC Symbol;Acc:HGNC:1719]
ENSG00000168477	254.911975	268.461655	631.093499	591.891663	-1.224107703	2.01E-11	7.20E-10	TNXB	tenascin XB [Source:HGNC Symbol;Acc:HGNC:11976]
ENSG00000129351	9719.13417	8710.38458	14365.5423	15181.5953	-0.681081679	2.01E-11	7.20E-10	ILF3	interleukin enhancer binding factor 3 [Source:HGNC Symbol;Acc:HGNC:6038]
ENSG00000198910	11345.0592	9610.57283	17571.5816	16973.2386	-0.721233244	2.04E-11	7.26E-10	L1CAM	L1 cell adhesion molecule [Source:HGNC Symbol;Acc:HGNC:6470]
ENSG00000011451	1500.92958	1371.54667	2444.30203	2547.47617	-0.797570193	2.09E-11	7.42E-10	WIZ	widely interspaced zinc finger motifs [Source:HGNC Symbol;Acc:HGNC:30917]
ENSG00000104888	136.80604	97.4613268	3.16073539	12.7746402	3.879504096	2.11E-11	7.49E-10	SLC17A7	solute carrier family 17 member 7 [Source:HGNC Symbol;Acc:HGNC:16704]
ENSG00000188612	2977.25376	2908.7776	4688.42416	4806.45838	-0.689905221	2.20E-11	7.79E-10	SUMO2	small ubiquitin-like modifier 2 [Source:HGNC Symbol;Acc:HGNC:11125]
ENSG00000163125	1019.6479	967.525171	1784.76192	1770.35222	-0.839422757	2.20E-11	7.79E-10	RPRD2	regulation of nuclear pre-mRNA domain containing 2 [Source:HGNC Symbol;Acc:HGNC:29039]
ENSG00000143947	7991.83488	8441.03691	12471.2083	14236.272	-0.700607389	2.36E-11	8.31E-10	RPS27A	ribosomal protein S27a [Source:HGNC Symbol;Acc:HGNC:10417]
ENSG00000148180	4472.27804	4009.20458	2518.05252	2560.25081	0.73981668	2.37E-11	8.34E-10	GSN	gelsolin [Source:HGNC Symbol;Acc:HGNC:4620]
ENSG00000136205	2398.53468	2141.49115	3937.22271	3735.51771	-0.757333216	2.43E-11	8.55E-10	TNS3	tensin 3 [Source:HGNC Symbol;Acc:HGNC:21616]
ENSG00000108602	45.2739414	42.528579	194.912016	202.265137	-2.178285245	2.44E-11	8.57E-10	ALDH3A1	aldehyde dehydrogenase 3 family member A1 [Source:HGNC Symbol;Acc:HGNC:405]
ENSG00000134013	5135.6397	4376.01357	7768.034	8115.09019	-0.739923225	2.55E-11	8.94E-10	LOXL2	lysyl oxidase like 2 [Source:HGNC Symbol;Acc:HGNC:6666]
ENSG00000116852	718.477766	580.3379	280.251871	253.363697	1.282543863	2.72E-11	9.50E-10	KIF21B	kinesin family member 21B [Source:HGNC Symbol;Acc:HGNC:29442]
ENSG00000142733	432.070876	412.881621	858.666447	885.708387	-1.046063745	2.79E-11	9.73E-10	MAP3K6	mitogen-activated protein kinase kinase kinase 6 [Source:HGNC Symbol;Acc:HGNC:6858]
ENSG00000121152	878.904994	885.12605	1528.74235	1765.02945	-0.900793195	2.85E-11	9.90E-10	NCAPH	non-SMC condensin I complex subunit H [Source:HGNC Symbol;Acc:HGNC:1112]
ENSG00000152082	982.247686	795.638831	1664.65397	1762.90035	-0.947842449	2.85E-11	9.90E-10	MZT2B	mitotic spindle organizing protein 2B [Source:HGNC Symbol;Acc:HGNC:25886]
ENSG00000255112	1277.51252	1348.51036	673.236637	714.315298	0.920538535	2.91E-11	1.01E-09	CHMP1B	charged multivesicular body protein 1B [Source:HGNC Symbol;Acc:HGNC:24287]
ENSG00000107957	1527.50342	1306.86779	756.469336	701.540658	0.958583203	3.03E-11	1.05E-09	SH3PXD2A	SH3 and PX domains 2A [Source:HGNC Symbol;Acc:HGNC:23664]
ENSG00000172889	917.289422	809.815024	410.8956	404.530273	1.082292398	3.04E-11	1.05E-09	EGFL7	EGF like domain multiple 7 [Source:HGNC Symbol;Acc:HGNC:20594]
ENSG00000179943	150.585066	146.19199	384.556139	437.531427	-1.47013339	3.18E-11	1.09E-09	FIZ1	FLT3 interacting zinc finger 1 [Source:HGNC Symbol;Acc:HGNC:25917]
ENSG00000117411	1303.10214	1139.41151	2118.74629	2267.49864	-0.845094149	3.21E-11	1.10E-09	B4GALT2	beta-1,4-galactosyltransferase 2 [Source:HGNC Symbol;Acc:HGNC:925]
ENSG00000134333	35492.8017	37472.9941	55034.7246	56061.5085	-0.606504283	3.26E-11	1.12E-09	LDHA	lactate dehydrogenase A [Source:HGNC Symbol;Acc:HGNC:6535]
ENSG00000104967	165.348308	132.901809	17.9108339	25.5492804	2.778544706	3.28E-11	1.12E-09	NOVA2	NOVA alternative splicing regulator 2 [Source:HGNC Symbol;Acc:HGNC:7887]
ENSG00000165283	944.847473	873.607893	1599.33211	1724.57643	-0.870505143	3.36E-11	1.15E-09	STOML2	stomatin like 2 [Source:HGNC Symbol;Acc:HGNC:14559]
ENSG00000188015	241.132949	190.492593	523.628496	572.729702	-1.346662829	3.57E-11	1.22E-09	S100A3	S100 calcium binding protein A3 [Source:HGNC Symbol;Acc:HGNC:10493]
ENSG00000149136	5810.81196	5207.9789	8600.36099	9311.64815	-0.701084725	3.58E-11	1.22E-09	SSRP1	structure specific recognition protein 1 [Source:HGNC Symbol;Acc:HGNC:11327]
ENSG00000173391	13247.549	15796.709	8668.84359	8892.21413	0.725949522	3.65E-11	1.24E-09	OLR1	oxidized low density lipoprotein receptor 1 [Source:HGNC Symbol;Acc:HGNC:8133]
ENSG00000163898	299.2017	322.50839	95.8756401	97.9389082	1.681904395	3.80E-11	1.29E-09	LIPH	lipase H [Source:HGNC Symbol;Acc:HGNC:18483]
ENSG00000132938	226.369707	180.746461	49.5181877	30.8720472	2.338915162	3.97E-11	1.34E-09	MTUS2	microtubule associated scaffold protein 2 [Source:HGNC Symbol;Acc:HGNC:20595]
ENSG00000204264	353.333586	304.788149	695.361785	779.253052	-1.164884653	3.99E-11	1.35E-09	PSMB8	proteasome subunit beta 8 [Source:HGNC Symbol;Acc:HGNC:9545]
ENSG00000106211	10019.3201	9095.79983	14632.0977	15743.6795	-0.668275703	4.17E-11	1.40E-09	HSPB1	heat shock protein family B (small) member 1 [Source:HGNC Symbol;Acc:HGNC:5246]
ENSG00000205542	33336.3841	36020.8204	51885.5785	59956.7092	-0.689331184	4.18E-11	1.40E-09	TMSB4X	thymosin beta 4 X-linked [Source:HGNC Symbol;Acc:HGNC:11881]
ENSG00000100297	3644.55229	3130.28061	5506.00104	5890.17369	-0.750538189	4.26E-11	1.43E-09	MCM5	minichromosome maintenance complex component 5 [Source:HGNC Symbol;Acc:HGNC:6948]
ENSG00000137807	2192.83351	2256.67272	3529.48785	3854.74768	-0.730710662	4.31E-11	1.44E-09	KIF23	kinesin family member 23 [Source:HGNC Symbol;Acc:HGNC:6392]

ENSG00000125726	2325.70269	2150.35127	3655.91726	3730.19494	-0.722796268	4.37E-11	1.46E-09	CD70	CD70 molecule [Source:HGNC Symbol;Acc:HGNC:11937]
ENSG00000167880	893.668235	738.93406	1506.6172	1636.2185	-0.945683326	4.57E-11	1.52E-09	EVPL	envoplakin [Source:HGNC Symbol;Acc:HGNC:3503]
ENSG00000111057	9481.93808	8541.15628	13837.6995	14906.9406	-0.673523407	4.83E-11	1.61E-09	KRT18	keratin 18 [Source:HGNC Symbol;Acc:HGNC:6430]
ENSG00000133069	721.430415	615.778383	265.501773	306.591365	1.224476437	5.39E-11	1.79E-09	TMCC2	transmembrane and coiled-coil domain family 2 [Source:HGNC Symbol;Acc:HGNC:24239]
ENSG00000137727	138.774473	159.482171	27.39304	18.097407	2.712754138	5.68E-11	1.88E-09	ARHGAP20	Rho GTPase activating protein 20 [Source:HGNC Symbol;Acc:HGNC:18357]
ENSG00000111424	1062.95341	937.400761	514.14629	463.080707	1.033029804	5.71E-11	1.89E-09	VDR	vitamin D receptor [Source:HGNC Symbol;Acc:HGNC:12679]
ENSG00000166508	6021.43421	5161.90627	9200.90071	9078.51097	-0.709041627	5.84E-11	1.93E-09	MCM7	minichromosome maintenance complex component 7 [Source:HGNC Symbol;Acc:HGNC:6950]
ENSG00000259781	681.077554	879.809977	1500.29573	1608.54011	-0.992973209	6.18E-11	2.03E-09	HMGB1P6	high mobility group box 1 pseudogene 6 [Source:HGNC Symbol;Acc:HGNC:4998]
ENSG00000019485	142.711337	129.357761	417.217071	356.625372	-1.509059365	6.27E-11	2.06E-09	PRDM11	PR/SET domain 11 [Source:HGNC Symbol;Acc:HGNC:13996]
ENSG00000167553	7139.50372	6981.77505	10667.4819	11121.3889	-0.625742614	6.48E-11	2.13E-09	TUBA1C	tubulin alpha 1c [Source:HGNC Symbol;Acc:HGNC:20768]
ENSG00000068615	406.481257	505.912887	179.108339	151.166576	1.46660277	6.57E-11	2.15E-09	REEP1	receptor accessory protein 1 [Source:HGNC Symbol;Acc:HGNC:25786]
ENSG00000066735	9607.91775	8935.43164	14581.5259	14137.2685	-0.631156735	6.83E-11	2.23E-09	KIF26A	kinesin family member 26A [Source:HGNC Symbol;Acc:HGNC:20226]
ENSG00000108773	1147.59599	1002.96565	1881.69113	1983.26289	-0.84626072	7.20E-11	2.35E-09	KAT2A	lysine acetyltransferase 2A [Source:HGNC Symbol;Acc:HGNC:4201]
ENSG00000073464	561.987403	546.669442	240.215889	231.008077	1.234181233	7.34E-11	2.39E-09	CLCN4	chloride voltage-gated channel 4 [Source:HGNC Symbol;Acc:HGNC:2022]
ENSG00000211584	1311.96009	1077.39067	549.967957	620.634603	1.028925091	7.39E-11	2.40E-09	SLC48A1	solute carrier family 48 member 1 [Source:HGNC Symbol;Acc:HGNC:26035]
ENSG00000179091	3950.6435	3474.05329	5961.14694	6161.63479	-0.707527581	7.39E-11	2.40E-09	CYC1	cytochrome c1 [Source:HGNC Symbol;Acc:HGNC:2579]
ENSG00000185236	1021.61633	848.799555	1724.70794	1753.31937	-0.895659794	7.55E-11	2.44E-09	RAB11B	RAB11B, member RAS oncogene family [Source:HGNC Symbol;Acc:HGNC:9761]
ENSG00000185745	385.812718	459.84026	882.898751	888.902047	-1.065982624	7.56E-11	2.44E-09	IFIT1	interferon induced protein with tetratricopeptide repeats 1 [Source:HGNC Symbol;Acc:HGNC:5407]
ENSG00000143198	3221.33935	3319.00118	1869.04819	2053.52341	0.737649538	7.60E-11	2.45E-09	MGST3	microsomal glutathione S-transferase 3 [Source:HGNC Symbol;Acc:HGNC:7064]
ENSG00000085662	65359.824	58312.8838	37850.8598	40811.7818	0.652764585	7.72E-11	2.48E-09	AKR1B1	aldo-keto reductase family 1 member B [Source:HGNC Symbol;Acc:HGNC:381]
ENSG00000096746	2897.53225	2843.21271	4434.51175	5107.72697	-0.733108459	7.95E-11	2.55E-09	HNRNPH3	heterogeneous nuclear ribonucleoprotein H3 [Source:HGNC Symbol;Acc:HGNC:5043]
ENSG00000163159	612.182426	566.161707	1092.56087	1183.78333	-0.950373123	7.99E-11	2.56E-09	VPS72	vacuolar protein sorting 72 homolog [Source:HGNC Symbol;Acc:HGNC:11644]
ENSG00000161682	974.373957	880.695989	415.109914	479.049008	1.052642572	8.47E-11	2.71E-09	FAM171A2	family with sequence similarity 171 member A2 [Source:HGNC Symbol;Acc:HGNC:30480]
ENSG00000134030	1271.60722	1139.41151	622.664871	636.602903	0.936733832	8.67E-11	2.77E-09	CTIF	cap binding complex dependent translation initiation factor [Source:HGNC Symbol;Acc:HGNC:23925]
ENSG00000107738	850.362726	804.498952	349.788049	421.563127	1.101199073	8.76E-11	2.79E-09	VSIR	V-set immunoregulatory receptor [Source:HGNC Symbol;Acc:HGNC:30085]
ENSG00000119383	3732.14752	3059.39965	5659.8235	5949.78867	-0.7738029	8.81E-11	2.80E-09	PTPA	protein phosphatase 2 phosphatase activator [Source:HGNC Symbol;Acc:HGNC:9308]
ENSG00000130779	2489.08256	2757.26954	4687.37058	4146.4353	-0.751525693	8.82E-11	2.80E-09	CLIP1	CAP-Gly domain containing linker protein 1 [Source:HGNC Symbol;Acc:HGNC:10461]
ENSG00000167680	421.244499	342.000656	111.679317	141.585596	1.590987945	8.95E-11	2.84E-09	SEMA6B	semaphorin 6B [Source:HGNC Symbol;Acc:HGNC:10739]
ENSG00000116350	1981.22705	1856.19527	3054.32396	3558.80185	-0.785337324	8.97E-11	2.84E-09	SRSF4	serine and arginine rich splicing factor 4 [Source:HGNC Symbol;Acc:HGNC:10786]
ENSG00000122862	890.715587	903.732303	443.556533	451.370621	1.003763168	9.02E-11	2.85E-09	SRGN	serglycin [Source:HGNC Symbol;Acc:HGNC:9361]
ENSG00000188505	349.396722	270.233679	92.7149047	93.6806948	1.732080886	9.06E-11	2.85E-09	NCCRP1	non-specific cytotoxic cell receptor protein 1 homolog (zebrafish) [Source:HGNC Symbol;Acc:HGNC:33739]
ENSG00000171954	147.632418	124.041689	20.0179928	19.1619603	2.792919679	9.07E-11	2.85E-09	CYP4F22	cytochrome P450 family 4 subfamily F member 22 [Source:HGNC Symbol;Acc:HGNC:26820]
ENSG00000135446	2964.45895	2666.01029	4663.13827	4526.48085	-0.706985567	9.29E-11	2.92E-09	CDK4	cyclin dependent kinase 4 [Source:HGNC Symbol;Acc:HGNC:1773]
ENSG00000135318	445.849902	450.980139	182.269074	167.134876	1.359900819	9.32E-11	2.92E-09	NT5E	5'-nucleotidase ecto [Source:HGNC Symbol;Acc:HGNC:8021]
ENSG00000196782	152.553498	154.166099	30.5537754	20.2265137	2.593952458	9.58E-11	3.00E-09	MAML3	mastermind like transcriptional coactivator 3 [Source:HGNC Symbol;Acc:HGNC:16272]
ENSG00000184584	445.849902	396.047392	843.916348	887.837494	-1.04126677	1.02E-10	3.18E-09	TMEM173	transmembrane protein 173 [Source:HGNC Symbol;Acc:HGNC:27962]
ENSG00000169857	98.4216118	90.3732303	279.198293	323.624218	-1.675748777	1.05E-10	3.27E-09	AVEN	apoptosis and caspase activation inhibitor [Source:HGNC Symbol;Acc:HGNC:13509]
ENSG00000165424	878.904994	801.840916	400.359816	414.111253	1.044864074	1.06E-10	3.31E-09	ZCCHC24	zinc finger CCHC-type containing 24 [Source:HGNC Symbol;Acc:HGNC:26911]
ENSG00000148337	3506.76203	3008.89696	5361.66079	5413.25379	-0.72596368	1.09E-10	3.39E-09	CIZ1	CDKN1A interacting zinc finger protein 1 [Source:HGNC Symbol;Acc:HGNC:16744]
ENSG00000146733	525.571407	515.65902	978.774391	1051.77871	-0.963673155	1.09E-10	3.39E-09	PSPH	phosphoserine phosphatase [Source:HGNC Symbol;Acc:HGNC:9577]
ENSG00000136826	1247.98604	1231.55677	679.558108	653.635757	0.89512065	1.10E-10	3.40E-09	KLF4	Kruppel like factor 4 [Source:HGNC Symbol;Acc:HGNC:6348]
ENSG00000175221	835.599484	751.338228	1448.67039	1457.37354	-0.87333434	1.11E-10	3.44E-09	MED16	mediator complex subunit 16 [Source:HGNC Symbol;Acc:HGNC:17556]
ENSG00000108176	332.665048	397.819416	135.911622	101.132568	1.624039928	1.12E-10	3.45E-09	DNAJC12	DnaJ heat shock protein family (Hsp40) member C12 [Source:HGNC Symbol;Acc:HGNC:28908]
ENSG00000196262	31194.7299	31893.7762	46051.9146	51112.4	-0.62304199	1.12E-10	3.45E-09	PPIA	peptidylprolyl isomerase A [Source:HGNC Symbol;Acc:HGNC:9253]
ENSG00000122966	1568.84049	1399.01305	2499.08811	2577.28366	-0.774758327	1.17E-10	3.59E-09	CIT	citron rho-interacting serine/threonine kinase [Source:HGNC Symbol;Acc:HGNC:1985]
ENSG00000099901	2926.07452	3062.05769	4624.15587	5050.24109	-0.691956569	1.19E-10	3.66E-09	RANBP1	RAN binding protein 1 [Source:HGNC Symbol;Acc:HGNC:9847]
ENSG00000197558	138.774473	116.953592	18.9644123	14.9037469	2.915496226	1.20E-10	3.67E-09	SSPO	SCO-spondin [Source:HGNC Symbol;Acc:HGNC:21998]
ENSG00000136827	1421.20807	1339.65024	2386.35522	2294.11247	-0.761767794	1.20E-10	3.67E-09	TOR1A	torsin family 1 member A [Source:HGNC Symbol;Acc:HGNC:3098]
ENSG00000173905	4418.14615	4553.21599	2812.00092	2837.03468	0.667371436	1.28E-10	3.89E-09	GOLIM4	golgi integral membrane protein 4 [Source:HGNC Symbol;Acc:HGNC:15448]
ENSG00000075213	287.391107	334.026547	97.982797	100.068015	1.650330618	1.29E-10	3.92E-09	SEMA3A	semaphorin 3A [Source:HGNC Symbol;Acc:HGNC:10723]
ENSG00000155265	181.095766	140.875918	21.0715692	33.0011539	2.573738066	1.30E-10	3.94E-09	GOLGA7B	golgin A7 family member B [Source:HGNC Symbol;Acc:HGNC:31668]

ENSG00000140105	2800.09486	2864.477	4384.99356	4687.2284	-0.679425716	1.32E-10	3.99E-09	WARS	tryptophanyl-tRNA synthetase [Source:HGNC Symbol;Acc:HGNC:12729]
ENSG00000169567	3772.50038	3444.8149	5596.60879	6050.92124	-0.690639713	1.33E-10	4.03E-09	HINT1	histidine triad nucleotide binding protein 1 [Source:HGNC Symbol;Acc:HGNC:4912]
ENSG00000168389	362.191531	323.394403	96.9292185	126.681849	1.616192953	1.47E-10	4.44E-09	MFSD2A	major facilitator superfamily domain containing 2A [Source:HGNC Symbol;Acc:HGNC:25897]
ENSG00000213339	530.492488	407.565548	967.185028	1015.5839	-1.081154431	1.48E-10	4.45E-09	QTRT1	queuine tRNA-ribosyltransferase catalytic subunit 1 [Source:HGNC Symbol;Acc:HGNC:23797]
ENSG00000166444	283.454242	272.891715	585.789625	669.604057	-1.174315634	1.48E-10	4.45E-09	ST5	suppression of tumorigenicity 5 [Source:HGNC Symbol;Acc:HGNC:11350]
ENSG00000115468	397.623312	310.990234	121.161523	112.842655	1.597474303	1.49E-10	4.48E-09	EFHD1	EF-hand domain family member D1 [Source:HGNC Symbol;Acc:HGNC:29556]
ENSG00000148120	954.689635	862.089736	1567.72475	1733.09285	-0.861859221	1.50E-10	4.50E-09	C9orf3	chromosome 9 open reading frame 3 [Source:HGNC Symbol;Acc:HGNC:1361]
ENSG00000100813	4068.74943	3602.52504	5979.05777	6625.78005	-0.716614096	1.51E-10	4.53E-09	ACIN1	apoptotic chromatin condensation inducer 1 [Source:HGNC Symbol;Acc:HGNC:17066]
ENSG00000167721	1346.40765	1480.52616	2338.94419	2503.82948	-0.776248097	1.52E-10	4.53E-09	TSR1	TSR1, ribosome maturation factor [Source:HGNC Symbol;Acc:HGNC:25542]
ENSG00000134070	1296.21263	1173.96598	684.826001	588.698003	0.955427737	1.52E-10	4.54E-09	IRAK2	interleukin 1 receptor associated kinase 2 [Source:HGNC Symbol;Acc:HGNC:6113]
ENSG00000125445	1088.54303	994.105533	1749.99383	2020.52226	-0.856658705	1.54E-10	4.59E-09	MRPS7	mitochondrial ribosomal protein S7 [Source:HGNC Symbol;Acc:HGNC:14499]
ENSG00000235162	1994.02186	2142.37717	1093.61444	1245.52742	0.822609079	1.55E-10	4.61E-09	C12orf75	chromosome 12 open reading frame 75 [Source:HGNC Symbol;Acc:HGNC:35164]
ENSG00000173210	1374.94992	1284.71749	737.504924	707.927978	0.879512738	1.59E-10	4.74E-09	ABLIM3	actin binding LIM protein family member 3 [Source:HGNC Symbol;Acc:HGNC:29132]
ENSG00000162496	755.877979	721.213818	1326.45528	1362.62829	-0.864595194	1.65E-10	4.88E-09	DHRS3	dehydrogenase/reductase 3 [Source:HGNC Symbol;Acc:HGNC:17693]
ENSG00000272068	161.411443	97.4613268	14.7500985	15.9683003	3.073523542	1.65E-10	4.88E-09	AL365181.2	novel transcript
ENSG00000033327	344.475641	299.472077	86.3934339	114.971762	1.676905966	1.66E-10	4.90E-09	GAB2	GRB2 associated binding protein 2 [Source:HGNC Symbol;Acc:HGNC:14458]
ENSG00000188707	228.338139	207.326822	527.84281	507.791948	-1.250045769	1.67E-10	4.92E-09	ZBED6CL	ZBED6 C-terminal like [Source:HGNC Symbol;Acc:HGNC:21720]
ENSG00000189091	7522.36379	7039.36583	11257.4859	11117.1306	-0.619753375	1.68E-10	4.94E-09	SF3B3	splicing factor 3b subunit 3 [Source:HGNC Symbol;Acc:HGNC:10770]
ENSG00000131981	3790.21627	3978.19416	5897.93223	6478.87169	-0.671853975	1.80E-10	5.28E-09	LGALS3	galectin 3 [Source:HGNC Symbol;Acc:HGNC:6563]
ENSG00000065923	708.635605	749.566204	362.430991	315.107792	1.105910174	1.81E-10	5.30E-09	SLC9A7	solute carrier family 9 member A7 [Source:HGNC Symbol;Acc:HGNC:17123]
ENSG00000137745	196.843224	229.477124	49.5181877	55.3567742	2.024043861	1.82E-10	5.33E-09	MMP13	matrix metallopeptidase 13 [Source:HGNC Symbol;Acc:HGNC:7159]
ENSG00000101361	3561.87813	3377.47798	5285.80315	6063.69588	-0.709829273	1.83E-10	5.34E-09	NOP56	NOP56 ribonucleoprotein [Source:HGNC Symbol;Acc:HGNC:15911]
ENSG00000089685	1842.45257	1874.80152	2923.68023	3313.95458	-0.746687807	1.86E-10	5.43E-09	BIRC5	baculoviral IAP repeat containing 5 [Source:HGNC Symbol;Acc:HGNC:593]
ENSG00000005889	833.631052	954.234991	1605.65358	1628.76663	-0.854660016	1.87E-10	5.44E-09	ZFX	zinc finger protein X-linked [Source:HGNC Symbol;Acc:HGNC:12869]
ENSG00000168028	21665.5494	20163.8625	31041.5822	32570.0098	-0.604798674	1.92E-10	5.58E-09	RPSA	ribosomal protein SA [Source:HGNC Symbol;Acc:HGNC:6502]
ENSG00000174938	1743.04675	1503.56247	872.362967	904.870348	0.868947091	2.11E-10	6.13E-09	SEZ6L2	seizure related 6 homolog like 2 [Source:HGNC Symbol;Acc:HGNC:30844]
ENSG00000182718	19636.0958	19727.0586	28667.87	30740.0425	-0.593805127	2.17E-10	6.30E-09	ANXA2	annexin A2 [Source:HGNC Symbol;Acc:HGNC:537]
ENSG00000178252	3071.7385	2451.59537	4810.63926	4737.26241	-0.790030303	2.18E-10	6.32E-09	WDR6	WD repeat domain 6 [Source:HGNC Symbol;Acc:HGNC:12758]
ENSG00000117713	2356.21339	2133.51704	3792.88246	3580.09292	-0.715881907	2.19E-10	6.33E-09	ARID1A	AT-rich interaction domain 1A [Source:HGNC Symbol;Acc:HGNC:11110]
ENSG00000181472	254.911975	260.487546	653.218647	541.857655	-1.213253192	2.23E-10	6.44E-09	ZBTB2	zinc finger and BTB domain containing 2 [Source:HGNC Symbol;Acc:HGNC:20868]
ENSG00000175216	4703.56883	4796.8693	7212.79815	7409.29132	-0.622048897	2.25E-10	6.47E-09	CKAP5	cytoskeleton associated protein 5 [Source:HGNC Symbol;Acc:HGNC:28959]
ENSG00000111602	2081.61709	2236.29444	3402.00485	3688.67736	-0.715385719	2.26E-10	6.50E-09	TIMELESS	timeless circadian regulator [Source:HGNC Symbol;Acc:HGNC:11813]
ENSG00000130749	964.531796	824.87723	1584.58201	1684.1234	-0.869888154	2.32E-10	6.67E-09	ZC3H4	zinc finger CCCH-type containing 4 [Source:HGNC Symbol;Acc:HGNC:17808]
ENSG00000112983	1438.92396	1326.36006	2270.46159	2430.3753	-0.765765946	2.36E-10	6.77E-09	BRD8	bromodomain containing 8 [Source:HGNC Symbol;Acc:HGNC:19874]
ENSG00000189221	210.622249	228.591112	57.9468154	56.4213276	1.941547828	2.40E-10	6.87E-09	MAOA	monoamine oxidase A [Source:HGNC Symbol;Acc:HGNC:6833]
ENSG00000154237	602.340264	480.218537	1098.88234	1076.26344	-1.007849878	2.47E-10	7.05E-09	LRRK1	leucine rich repeat kinase 1 [Source:HGNC Symbol;Acc:HGNC:18608]
ENSG00000138668	5276.38261	4826.1077	7662.67616	8378.03487	-0.66713532	2.52E-10	7.17E-09	HNRNPD	heterogeneous nuclear ribonucleoprotein D [Source:HGNC Symbol;Acc:HGNC:5036]
ENSG00000120306	1350.34451	1273.19933	693.254628	737.735472	0.87435853	2.53E-10	7.20E-09	CYSTM1	cysteine rich transmembrane module containing 1 [Source:HGNC Symbol;Acc:HGNC:30239]
ENSG00000115129	433.055092	371.239054	787.023111	905.934901	-1.074641626	2.59E-10	7.34E-09	TP53I3	tumor protein p53 inducible protein 3 [Source:HGNC Symbol;Acc:HGNC:19373]
ENSG00000198517	2085.55395	1543.43301	905.023899	932.548735	0.981258606	2.66E-10	7.53E-09	MAFK	MAF bZIP transcription factor K [Source:HGNC Symbol;Acc:HGNC:6782]
ENSG00000138193	797.215056	861.203724	418.27065	399.207506	1.020800932	2.69E-10	7.61E-09	PLCE1	phospholipase C epsilon 1 [Source:HGNC Symbol;Acc:HGNC:17175]
ENSG00000057252	4639.59478	5048.49673	3125.9673	2874.29405	0.691276672	2.79E-10	7.89E-09	SOAT1	sterol O-acyltransferase 1 [Source:HGNC Symbol;Acc:HGNC:11177]
ENSG00000134690	1033.42692	949.80493	1722.60079	1741.60928	-0.805046983	2.82E-10	7.95E-09	CDCA8	cell division cycle associated 8 [Source:HGNC Symbol;Acc:HGNC:14629]
ENSG00000112715	3400.46669	2780.30585	5028.73	5570.80768	-0.778443764	2.89E-10	8.13E-09	VEGFA	vascular endothelial growth factor A [Source:HGNC Symbol;Acc:HGNC:12680]
ENSG00000149929	404.512825	342.000656	758.576493	814.383313	-1.076304962	2.95E-10	8.31E-09	HIRIP3	HIRA interacting protein 3 [Source:HGNC Symbol;Acc:HGNC:4917]
ENSG00000198576	201.764304	142.647942	22.1251477	38.3239206	2.509964609	2.99E-10	8.39E-09	ARC	activity regulated cytoskeleton associated protein [Source:HGNC Symbol;Acc:HGNC:648]
ENSG00000102870	729.304144	621.094455	1292.74077	1252.97929	-0.915504269	3.08E-10	8.64E-09	ZNF629	zinc finger protein 629 [Source:HGNC Symbol;Acc:HGNC:29008]
ENSG00000183092	271.643649	197.58069	583.682468	566.342382	-1.295553302	3.14E-10	8.79E-09	BEGAIN	brain enriched guanylate kinase associated [Source:HGNC Symbol;Acc:HGNC:24163]
ENSG00000123485	595.450752	545.78343	1064.11425	1098.61906	-0.922756107	3.15E-10	8.80E-09	HJURP	Holliday junction recognition protein [Source:HGNC Symbol;Acc:HGNC:25444]
ENSG00000113070	297.233268	281.751836	97.982797	86.2288214	1.651789449	3.25E-10	9.06E-09	HBEGF	heparin binding EGF like growth factor [Source:HGNC Symbol;Acc:HGNC:3059]
ENSG00000104980	751.941114	625.524516	1266.40131	1358.37007	-0.931028311	3.27E-10	9.11E-09	TIMM44	translocase of inner mitochondrial membrane 44 [Source:HGNC Symbol;Acc:HGNC:17316]
ENSG00000261371	327.743967	336.684583	82.1791201	125.617295	1.677457027	3.30E-10	9.16E-09	PECAM1	platelet and endothelial cell adhesion molecule 1 [Source:HGNC Symbol;Acc:HGNC:8823]
ENSG00000172534	3097.32812	2644.746	5027.67642	4574.38575	-0.742072118	3.32E-10	9.20E-09	HCFC1	host cell factor C1 [Source:HGNC Symbol;Acc:HGNC:4839]

ENSG00000132507	14092.9906	11894.7119	20059.0803	22051.1581	-0.696416963	3.35E-10	9.28E-09	EIF5A	eukaryotic translation initiation factor 5A [Source:HGNC Symbol;Acc:HGNC:3300]
ENSG00000163874	2801.07907	2530.45045	1585.63559	1605.34645	0.740346786	3.49E-10	9.64E-09	ZC3H12A	zinc finger CCCH-type containing 12A [Source:HGNC Symbol;Acc:HGNC:26259]
ENSG00000101945	417.307634	365.036969	762.790807	873.998301	-1.065783185	3.52E-10	9.73E-09	SUV39H1	suppressor of variegation 3-9 homolog 1 [Source:HGNC Symbol;Acc:HGNC:11479]
ENSG00000124216	1064.92184	933.856713	473.05673	531.212122	0.992639126	3.57E-10	9.83E-09	SNAI1	snail family transcriptional repressor 1 [Source:HGNC Symbol;Acc:HGNC:11128]
ENSG00000095397	2189.88086	1842.01908	1075.70361	1166.75047	0.84605733	3.75E-10	1.03E-08	WHRN	whirlin [Source:HGNC Symbol;Acc:HGNC:16361]
ENSG00000122566	20615.3908	22370.9185	31819.1231	33318.3908	-0.599579659	3.76E-10	1.03E-08	HNRNPA2B1	heterogeneous nuclear ribonucleoprotein A2/B1 [Source:HGNC Symbol;Acc:HGNC:5033]
ENSG00000183688	283.454242	272.005703	77.9648062	93.6806948	1.694292104	3.78E-10	1.04E-08	RFLNB	refilin B [Source:HGNC Symbol;Acc:HGNC:28705]
ENSG00000179388	117.121718	109.865496	11.5893631	15.9683003	3.042450164	3.87E-10	1.06E-08	EGR3	early growth response 3 [Source:HGNC Symbol;Acc:HGNC:3240]
ENSG00000135486	20045.5297	21005.574	29786.7703	33302.4225	-0.619951905	3.99E-10	1.09E-08	HNRNPA1	heterogeneous nuclear ribonucleoprotein A1 [Source:HGNC Symbol;Acc:HGNC:5031]
ENSG00000104998	563.955836	493.508718	1021.97111	1021.97122	-0.951522711	4.01E-10	1.09E-08	IL27RA	interleukin 27 receptor subunit alpha [Source:HGNC Symbol;Acc:HGNC:17290]
ENSG00000132481	811.978298	738.048047	1350.68759	1470.14818	-0.864267086	4.04E-10	1.10E-08	TRIM47	tripartite motif containing 47 [Source:HGNC Symbol;Acc:HGNC:19020]
ENSG00000136943	781.467598	795.638831	383.50256	397.0784	1.014753849	4.05E-10	1.10E-08	CTSV	cathepsin V [Source:HGNC Symbol;Acc:HGNC:2538]
ENSG00000137834	2524.51434	2460.4555	1526.63519	1504.21388	0.717797552	4.06E-10	1.10E-08	SMAD6	SMAD family member 6 [Source:HGNC Symbol;Acc:HGNC:6772]
ENSG00000105357	489.155411	366.808994	153.822455	158.618449	1.452995457	4.20E-10	1.14E-08	MYH14	myosin heavy chain 14 [Source:HGNC Symbol;Acc:HGNC:23212]
ENSG00000108953	8525.28002	9302.24064	13256.1242	14443.8599	-0.635704806	4.21E-10	1.14E-08	YWHAE	tyrosine 3-monooxygenase/tryptophan 5-monooxygenase activation protein epsilon [Source:HGNC Symbol;Acc:HGNC:12851]
ENSG00000170775	97.4373957	98.3473389	9.48220616	8.5164268	3.443108118	4.26E-10	1.15E-08	GPR37	G protein-coupled receptor 37 [Source:HGNC Symbol;Acc:HGNC:4494]
ENSG00000140945	1412.35013	1410.5312	2406.37321	2280.27328	-0.731406387	4.31E-10	1.16E-08	CDH13	cadherin 13 [Source:HGNC Symbol;Acc:HGNC:1753]
ENSG00000130635	27528.5248	24826.058	39822.1051	39642.9022	-0.602035247	4.37E-10	1.18E-08	COL5A1	collagen type V alpha 1 chain [Source:HGNC Symbol;Acc:HGNC:2209]
ENSG00000159335	9256.55259	8489.76758	13140.2306	14861.1648	-0.658036614	4.59E-10	1.23E-08	PTMS	parathymosin [Source:HGNC Symbol;Acc:HGNC:9629]
ENSG00000101126	1889.69495	1959.85868	3129.12803	3069.10731	-0.68705725	4.60E-10	1.24E-08	ADNP	activity dependent neuroprotector homeobox [Source:HGNC Symbol;Acc:HGNC:15766]
ENSG00000139211	1548.17195	1530.14283	2741.41116	2431.43985	-0.748884035	4.65E-10	1.25E-08	AMIGO2	adhesion molecule with Ig like domain 2 [Source:HGNC Symbol;Acc:HGNC:24073]
ENSG00000106688	301.170132	287.067908	100.089954	92.6161415	1.609703968	4.67E-10	1.25E-08	SLC1A1	solute carrier family 1 member 1 [Source:HGNC Symbol;Acc:HGNC:10939]
ENSG00000182481	5259.65094	5272.65778	7975.58896	8006.50575	-0.601630728	4.68E-10	1.25E-08	KPNA2	karyopherin subunit alpha 2 [Source:HGNC Symbol;Acc:HGNC:6395]
ENSG00000111678	570.845349	438.575971	987.203019	1103.94182	-1.052036238	4.72E-10	1.26E-08	C12orf57	chromosome 12 open reading frame 57 [Source:HGNC Symbol;Acc:HGNC:29521]
ENSG00000100219	1490.1032	1483.18419	2392.67669	2504.89403	-0.720012755	4.84E-10	1.29E-08	XBP1	X-box binding protein 1 [Source:HGNC Symbol;Acc:HGNC:12801]
ENSG00000130726	7862.90257	6388.14697	11487.166	11969.8379	-0.719107983	5.11E-10	1.36E-08	TRIM28	tripartite motif containing 28 [Source:HGNC Symbol;Acc:HGNC:16384]
ENSG00000149532	1890.67916	1812.78068	2925.78739	3079.75284	-0.697540406	5.13E-10	1.36E-08	CPSF7	cleavage and polyadenylation specific factor 7 [Source:HGNC Symbol;Acc:HGNC:30098]
ENSG00000235173	802.136136	660.078986	1451.83112	1320.04615	-0.923600222	5.22E-10	1.39E-08	HGH1	HGH1 homolog [Source:HGNC Symbol;Acc:HGNC:24161]
ENSG00000177548	203.732736	159.482171	443.556533	476.919901	-1.343518638	5.31E-10	1.41E-08	RABEP2	rabaptin, RAB GTPase binding effector protein 2 [Source:HGNC Symbol;Acc:HGNC:24817]
ENSG00000165617	138.774473	116.953592	16.8572554	21.291067	2.744649704	5.48E-10	1.45E-08	DACT1	dishevelled binding antagonist of beta catenin 1 [Source:HGNC Symbol;Acc:HGNC:17748]
ENSG00000105088	203.732736	183.404497	49.5181877	43.6466874	2.054373143	5.51E-10	1.46E-08	OLFM2	olfactomedin 2 [Source:HGNC Symbol;Acc:HGNC:17189]
ENSG00000152527	201.764304	196.694678	53.7325016	44.7112407	2.01670364	5.56E-10	1.47E-08	PLEKHH2	pleckstrin homology, MyTH4 and FERM domain containing H2 [Source:HGNC Symbol;Acc:HGNC:30506]
ENSG00000214049	8008.56655	8208.01574	5208.89192	5435.60941	0.607391684	5.67E-10	1.49E-08	UCA1	urothelial cancer associated 1 [Source:HGNC Symbol;Acc:HGNC:37126]
ENSG00000054179	297.233268	238.337245	77.9648062	81.970608	1.742765419	5.75E-10	1.51E-08	ENTPD2	ectonucleoside triphosphate diphosphohydrolase 2 [Source:HGNC Symbol;Acc:HGNC:3364]
ENSG00000145555	5224.21916	4926.22706	3333.52225	3096.7857	0.658490295	5.77E-10	1.51E-08	MYO10	myosin X [Source:HGNC Symbol;Acc:HGNC:7593]
ENSG00000082014	593.482319	575.90784	249.698096	276.783871	1.151268643	5.79E-10	1.52E-08	SMARCD3	SWI/SNF related, matrix associated, actin dependent regulator of chromatin, subfamily d, member 3 [Source:HGNC Symbol;Acc:HGNC:11108]
ENSG00000234616	827.725755	782.348651	1558.24255	1363.69284	-0.860098622	5.81E-10	1.52E-08	JRK	Jrk helix-turn-helix protein [Source:HGNC Symbol;Acc:HGNC:6199]
ENSG00000169696	478.329033	428.829838	867.095074	922.967755	-0.981241665	5.89E-10	1.54E-08	ASPSCR1	ASPSCR1, UBX domain containing tether for SLC2A4 [Source:HGNC Symbol;Acc:HGNC:13825]
ENSG00000163939	735.20944	770.830494	1377.02705	1316.85249	-0.838694638	5.92E-10	1.54E-08	PBRM1	polybromo 1 [Source:HGNC Symbol;Acc:HGNC:30064]
ENSG00000158186	910.399909	917.022484	430.913591	495.017308	0.980951404	5.98E-10	1.56E-08	MRAS	muscle RAS oncogene homolog [Source:HGNC Symbol;Acc:HGNC:7227]
ENSG00000173207	1465.4978	1401.67108	2315.76546	2427.18164	-0.726308563	6.12E-10	1.59E-08	CKS1B	CDC28 protein kinase regulatory subunit 1B [Source:HGNC Symbol;Acc:HGNC:19083]
ENSG00000175061	1410.3817	1347.62435	2205.13972	2441.02083	-0.752550636	6.16E-10	1.60E-08	LRRC75A-AS1	LRRC75A antisense RNA 1 [Source:HGNC Symbol;Acc:HGNC:28619]
ENSG00000105771	459.628927	359.720897	852.344976	860.159107	-1.06497954	6.50E-10	1.68E-08	SMG9	SMG9, nonsense mediated mRNA decay factor [Source:HGNC Symbol;Acc:HGNC:25763]
ENSG00000129354	270.659433	218.844979	546.807222	585.504343	-1.211425785	6.65E-10	1.72E-08	AP1M2	adaptor related protein complex 1 subunit mu 2 [Source:HGNC Symbol;Acc:HGNC:558]
ENSG00000143324	1767.65215	2054.66197	1134.704	967.678995	0.862663681	6.66E-10	1.72E-08	XPR1	xenotropic and polytropic retrovirus receptor 1 [Source:HGNC Symbol;Acc:HGNC:12827]
ENSG00000100300	3187.87601	2757.26954	4717.92435	4941.65665	-0.700519257	6.71E-10	1.73E-08	TSPO	translocator protein [Source:HGNC Symbol;Acc:HGNC:1158]
ENSG00000116032	111.216421	113.409544	10.5357846	17.0328536	3.027402009	6.93E-10	1.79E-08	GRIN3B	glutamate ionotropic receptor NMDA type subunit 3B [Source:HGNC Symbol;Acc:HGNC:16768]
ENSG00000160447	638.756261	570.591768	1107.31096	1150.78217	-0.901486367	7.13E-10	1.83E-08	PKN3	protein kinase N3 [Source:HGNC Symbol;Acc:HGNC:17999]
ENSG00000238227	2101.30141	1867.71343	3207.09284	3286.27619	-0.71050673	7.26E-10	1.86E-08	TMEM250	transmembrane protein 250 [Source:HGNC Symbol;Acc:HGNC:31009]
ENSG00000047634	745.051601	791.208771	395.091923	357.689926	1.029264815	7.36E-10	1.89E-08	SCML1	Scm polycomb group protein like 1 [Source:HGNC Symbol;Acc:HGNC:10580]

ENSG00000125977	2920.16922	3090.41007	4555.67327	4982.10968	-0.666018914	7.48E-10	1.91E-08	EIF2S2	eukaryotic translation initiation factor 2 subunit beta [Source:HGNC Symbol;Acc:HGNC:3266]
ENSG00000183684	2170.19654	1901.38188	3214.46789	3657.80531	-0.755492768	7.48E-10	1.91E-08	ALYREF	Aly/REF export factor [Source:HGNC Symbol;Acc:HGNC:19071]
ENSG00000137809	91.532099	97.4613268	9.48220616	7.45187345	3.480047073	7.56E-10	1.93E-08	ITGA11	integrin subunit alpha 11 [Source:HGNC Symbol;Acc:HGNC:6136]
ENSG00000128564	167.31674	158.596159	34.7680893	34.0657072	2.243039757	7.78E-10	1.98E-08	VGF	VGF nerve growth factor inducible [Source:HGNC Symbol;Acc:HGNC:12684]
ENSG00000100387	943.863257	974.613268	1580.36769	1795.9015	-0.815295447	7.83E-10	1.99E-08	RBX1	ring-box 1 [Source:HGNC Symbol;Acc:HGNC:9928]
ENSG00000173517	1625.92503	1820.75479	1028.29258	850.578127	0.875507726	7.86E-10	2.00E-08	PEAK1	pseudopodium enriched atypical kinase 1 [Source:HGNC Symbol;Acc:HGNC:29431]
ENSG00000070814	3646.52072	3087.75204	5327.94628	5696.42498	-0.711363922	8.22E-10	2.08E-08	TCOF1	treacle ribosome biogenesis factor 1 [Source:HGNC Symbol;Acc:HGNC:11654]
ENSG00000115350	738.162089	647.674817	1200.02587	1462.6963	-0.942667197	8.22E-10	2.08E-08	POLE4	DNA polymerase epsilon 4, accessory subunit [Source:HGNC Symbol;Acc:HGNC:18755]
ENSG00000156482	3349.28745	2790.05198	4852.7824	5514.38635	-0.756139475	8.46E-10	2.14E-08	RPL30	ribosomal protein L30 [Source:HGNC Symbol;Acc:HGNC:10333]
ENSG00000053501	83.65837	67.3369167	262.341037	241.653611	-1.741316015	8.60E-10	2.17E-08	USE1	unconventional SNARE in the ER 1 [Source:HGNC Symbol;Acc:HGNC:30882]
ENSG00000186907	353.333586	334.026547	116.947209	132.004615	1.465090083	8.63E-10	2.18E-08	RTN4RL2	reticulon 4 receptor like 2 [Source:HGNC Symbol;Acc:HGNC:23053]
ENSG00000196419	10782.0876	10924.5287	15666.7117	17285.1527	-0.602210997	8.88E-10	2.24E-08	XRCC6	X-ray repair cross complementing 6 [Source:HGNC Symbol;Acc:HGNC:4055]
ENSG00000179314	341.522993	312.762258	112.732895	119.229975	1.495712469	9.06E-10	2.28E-08	WSCD1	WSC domain containing 1 [Source:HGNC Symbol;Acc:HGNC:29060]
ENSG00000108424	7252.68858	7256.43879	10623.2316	11174.6165	-0.587220283	9.27E-10	2.33E-08	KPNB1	karyopherin subunit beta 1 [Source:HGNC Symbol;Acc:HGNC:6400]
ENSG00000144063	190.937927	305.674161	66.3754431	59.6149876	1.980189063	9.39E-10	2.35E-08	MALL	mal, T cell differentiation protein like [Source:HGNC Symbol;Acc:HGNC:6818]
ENSG00000086504	1803.08393	1403.44311	2740.35758	3006.29866	-0.84229347	1.00E-09	2.50E-08	MRPL28	mitochondrial ribosomal protein L28 [Source:HGNC Symbol;Acc:HGNC:14484]
ENSG00000105698	1629.86189	1393.69697	2504.356	2621.9949	-0.76215751	1.03E-09	2.56E-08	USF2	upstream transcription factor 2, c-fos interacting [Source:HGNC Symbol;Acc:HGNC:12594]
ENSG00000186951	701.746092	625.524516	329.770059	282.106638	1.116624492	1.04E-09	2.60E-08	PPARA	peroxisome proliferator activated receptor alpha [Source:HGNC Symbol;Acc:HGNC:9232]
ENSG00000135046	10078.3731	10950.2231	6405.75705	7114.41004	0.637304522	1.12E-09	2.80E-08	ANXA1	annexin A1 [Source:HGNC Symbol;Acc:HGNC:533]
ENSG00000165119	12745.5987	13058.9318	18601.9813	19949.7298	-0.579151945	1.13E-09	2.81E-08	HNRNPK	heterogeneous nuclear ribonucleoprotein K [Source:HGNC Symbol;Acc:HGNC:5044]
ENSG00000124882	821.820459	889.55611	460.413788	384.303759	1.018753902	1.14E-09	2.84E-08	EREG	epiregulin [Source:HGNC Symbol;Acc:HGNC:3443]
ENSG00000108557	1675.13583	1377.74876	2728.76822	2553.86349	-0.791645552	1.15E-09	2.87E-08	RAI1	retinoic acid induced 1 [Source:HGNC Symbol;Acc:HGNC:9834]
ENSG00000140600	78.7372895	117.839604	10.5357846	7.45187345	3.450714772	1.17E-09	2.91E-08	SH3GL3	SH3 domain containing GRB2 like 3, endophilin A3 [Source:HGNC Symbol;Acc:HGNC:10832]
ENSG00000128928	1024.56898	929.426653	1619.3501	1811.8698	-0.812667411	1.18E-09	2.93E-08	IVD	isovaleryl-CoA dehydrogenase [Source:HGNC Symbol;Acc:HGNC:6186]
ENSG00000181804	96.4531796	115.181568	12.6429415	12.7746402	3.058397281	1.24E-09	3.06E-08	SLC9A9	solute carrier family 9 member A9 [Source:HGNC Symbol;Acc:HGNC:20653]
ENSG00000166965	997.995144	892.214146	1613.02863	1674.54242	-0.798959021	1.28E-09	3.16E-08	RCCD1	RCC1 domain containing 1 [Source:HGNC Symbol;Acc:HGNC:30457]
ENSG00000178605	1100.35362	888.670098	1779.49402	1801.22427	-0.848977042	1.32E-09	3.24E-08	GTPBP6	GTP binding protein 6 (putative) [Source:HGNC Symbol;Acc:HGNC:30189]
ENSG00000135617	909.415693	817.789133	447.770846	433.273214	0.970756603	1.33E-09	3.27E-08	PRADC1	protease associated domain containing 1 [Source:HGNC Symbol;Acc:HGNC:16047]
ENSG00000064300	102.358476	62.9068564	3.16073539	2.1291067	4.96267797	1.35E-09	3.32E-08	NGFR	nerve growth factor receptor [Source:HGNC Symbol;Acc:HGNC:7809]
ENSG00000198467	3985.09106	3199.38956	5706.18095	6626.84461	-0.779828665	1.35E-09	3.32E-08	TPM2	tropomyosin 2 [Source:HGNC Symbol;Acc:HGNC:12011]
ENSG00000108370	149.60085	186.948545	41.08956	28.7429405	2.269193633	1.35E-09	3.32E-08	RGS9	regulator of G protein signaling 9 [Source:HGNC Symbol;Acc:HGNC:10004]
ENSG00000137331	3941.78555	3877.18878	2220.9434	2582.60643	0.702892606	1.37E-09	3.35E-08	IER3	immediate early response 3 [Source:HGNC Symbol;Acc:HGNC:5392]
ENSG00000135390	3083.5491	2694.36268	4453.47616	4993.81977	-0.709587028	1.42E-09	3.48E-08	ATP5MC2	ATP synthase membrane subunit c locus 2 [Source:HGNC Symbol;Acc:HGNC:842]
ENSG00000145675	290.343755	261.373558	91.6613262	88.3579281	1.615286964	1.45E-09	3.54E-08	PIK3R1	phosphoinositide-3-kinase regulatory subunit 1 [Source:HGNC Symbol;Acc:HGNC:8979]
ENSG00000175426	325.775535	380.099175	136.9652	121.359082	1.450726754	1.51E-09	3.67E-08	PCSK1	proprotein convertase subtilisin/kexin type 1 [Source:HGNC Symbol;Acc:HGNC:8743]
ENSG00000130396	1393.65002	1487.61425	839.702034	806.931439	0.807355071	1.51E-09	3.68E-08	AFDN	afadin, adherens junction formation factor [Source:HGNC Symbol;Acc:HGNC:7137]
ENSG00000164236	330.696616	287.95392	112.732895	100.068015	1.538922114	1.57E-09	3.82E-08	ANKRD33B	ankyrin repeat domain 33B [Source:HGNC Symbol;Acc:HGNC:35240]
ENSG00000081277	39.3686447	45.1866152	186.483388	167.134876	-2.062488837	1.68E-09	4.08E-08	PKP1	plakophilin 1 [Source:HGNC Symbol;Acc:HGNC:9023]
ENSG00000163814	4155.36045	3845.29235	2597.07091	2470.82833	0.658594941	1.69E-09	4.09E-08	CDCP1	CUB domain containing protein 1 [Source:HGNC Symbol;Acc:HGNC:24357]
ENSG00000174547	1278.49674	1216.49456	1971.2453	2366.5021	-0.798035963	1.74E-09	4.20E-08	MRPL11	mitochondrial ribosomal protein L11 [Source:HGNC Symbol;Acc:HGNC:14042]
ENSG00000135736	154.521931	128.471749	361.377413	380.045546	-1.391180298	1.75E-09	4.24E-08	CCDC102A	coiled-coil domain containing 102A [Source:HGNC Symbol;Acc:HGNC:28097]
ENSG00000115255	495.060707	404.0215	158.036769	194.813263	1.348856698	1.76E-09	4.25E-08	REEP6	receptor accessory protein 6 [Source:HGNC Symbol;Acc:HGNC:30078]
ENSG00000111669	25762.8411	23136.433	36040.812	37567.0232	-0.590079043	1.77E-09	4.26E-08	TPI1	triosephosphate isomerase 1 [Source:HGNC Symbol;Acc:HGNC:12009]
ENSG00000100889	858.236455	686.659348	1465.52764	1400.95221	-0.892724354	1.82E-09	4.38E-08	PCK2	phosphoenolpyruvate carboxykinase 2, mitochondrial [Source:HGNC Symbol;Acc:HGNC:8725]
ENSG00000114631	4289.21384	3721.25066	2342.10492	2554.92804	0.709808452	1.83E-09	4.39E-08	PODXL2	podocalyxin like 2 [Source:HGNC Symbol;Acc:HGNC:17936]
ENSG00000186868	812.962514	744.250132	391.931188	392.820186	0.988332628	1.93E-09	4.61E-08	MAPT	microtubule associated protein tau [Source:HGNC Symbol;Acc:HGNC:6893]
ENSG00000177731	4261.65579	3629.10541	6295.13131	6265.96102	-0.670964316	1.98E-09	4.74E-08	FLII	FLII, actin remodeling protein [Source:HGNC Symbol;Acc:HGNC:3750]
ENSG00000073910	429.118228	496.166755	198.072751	190.55505	1.252050515	2.00E-09	4.78E-08	FRY	FRY microtubule binding protein [Source:HGNC Symbol;Acc:HGNC:20367]
ENSG00000028137	162.39566	108.979484	23.1787262	21.291067	2.60779413	2.01E-09	4.80E-08	TNFRSF1B	TNF receptor superfamily member 1B [Source:HGNC Symbol;Acc:HGNC:11917]
ENSG00000283265	191.922143	166.570268	47.4110308	36.1948139	2.099325252	2.02E-09	4.81E-08	AL356234.3	novel transcript
ENSG00000248109	469.471088	591.856057	236.001576	213.975223	1.238674866	2.10E-09	4.99E-08	MARCOL	MARCO like [Source:HGNC Symbol;Acc:HGNC:53644]
ENSG00000147852	555.097891	598.058142	272.876822	261.880124	1.108896105	2.21E-09	5.26E-08	VLDLR	very low density lipoprotein receptor [Source:HGNC Symbol;Acc:HGNC:12698]

ENSG00000065911	1804.06814	1781.77026	2821.48312	2863.64851	-0.664920678	2.22E-09	5.27E-08	MTHFD2	methylenetetrahydrofolate dehydrogenase (NADP+ dependent) 2, methenyltetrahydrofolate cyclohydrolase [Source:HGNC Symbol;Acc:HGNC:7434]
ENSG00000163993	5351.18303	4247.54182	2767.75062	2912.61797	0.756644488	2.33E-09	5.52E-08	S100P	S100 calcium binding protein P [Source:HGNC Symbol;Acc:HGNC:10504]
ENSG00000233913	1043.26909	940.94481	1683.61838	1703.28536	-0.771845697	2.39E-09	5.66E-08	RPL10P9	ribosomal protein L10 pseudogene 9 [Source:HGNC Symbol;Acc:HGNC:35579]
ENSG00000186862	296.249052	227.7051	86.3934339	71.3250745	1.730880048	2.44E-09	5.77E-08	PDZD7	PDZ domain containing 7 [Source:HGNC Symbol;Acc:HGNC:26257]
ENSG00000090266	958.626499	915.25046	1546.65318	1656.44501	-0.77363242	2.50E-09	5.90E-08	NDUFB2	NADH:ubiquinone oxidoreductase subunit B2 [Source:HGNC Symbol;Acc:HGNC:7697]
ENSG00000109819	246.05403	285.295884	85.3398554	86.2288214	1.631510613	2.51E-09	5.92E-08	PPARGC1A	PPARG coactivator 1 alpha [Source:HGNC Symbol;Acc:HGNC:9237]
ENSG00000265241	1223.38063	1209.40646	1928.04859	2132.30036	-0.73901124	2.55E-09	6.00E-08	RBM8A	RNA binding motif protein 8A [Source:HGNC Symbol;Acc:HGNC:9905]
ENSG00000114646	243.101381	245.425341	84.286277	50.0340075	1.862226368	2.63E-09	6.19E-08	CSPG5	chondroitin sulfate proteoglycan 5 [Source:HGNC Symbol;Acc:HGNC:2467]
ENSG00000204371	1590.49325	1398.12703	2543.33841	2412.27789	-0.730013484	2.64E-09	6.21E-08	EHMT2	euchromatic histone lysine methyltransferase 2 [Source:HGNC Symbol;Acc:HGNC:14129]
ENSG00000107798	2108.19093	2526.9064	1416.00945	1168.87958	0.84273008	2.75E-09	6.44E-08	LIPA	lipase A, lysosomal acid type [Source:HGNC Symbol;Acc:HGNC:6617]
ENSG00000142784	2181.02292	2160.09741	1344.36612	1327.49803	0.700190949	2.77E-09	6.49E-08	WDTC1	WD and tetratricopeptide repeats 1 [Source:HGNC Symbol;Acc:HGNC:29175]
ENSG00000263001	810.994081	882.468014	1584.58201	1404.14587	-0.819213332	2.80E-09	6.55E-08	GTF2I	general transcription factor IIi [Source:HGNC Symbol;Acc:HGNC:4659]
ENSG00000204568	869.062832	784.120675	1383.34852	1543.60236	-0.824584868	2.82E-09	6.57E-08	MRPS18B	mitochondrial ribosomal protein S18B [Source:HGNC Symbol;Acc:HGNC:14516]
ENSG00000239672	1580.65109	1661.27262	2510.67748	2763.5805	-0.701936585	2.89E-09	6.73E-08	NME1	NME/NM23 nucleoside diphosphate kinase 1 [Source:HGNC Symbol;Acc:HGNC:7849]
ENSG00000071282	553.129458	534.265273	230.733683	262.944678	1.139204795	2.99E-09	6.96E-08	LMCD1	LIM and cysteine rich domains 1 [Source:HGNC Symbol;Acc:HGNC:6633]
ENSG00000145494	3393.57718	2613.73558	4901.24701	5472.86877	-0.788544723	3.08E-09	7.17E-08	NDUFS6	NADH:ubiquinone oxidoreductase subunit S6 [Source:HGNC Symbol;Acc:HGNC:7713]
ENSG00000244405	919.257854	825.763242	467.788837	435.40232	0.949721096	3.14E-09	7.29E-08	ETV5	ETS variant 5 [Source:HGNC Symbol;Acc:HGNC:3494]
ENSG00000133265	963.54758	754.882277	1528.74235	1636.2185	-0.882002596	3.18E-09	7.37E-08	HSPBP1	HSPA (Hsp70) binding protein 1 [Source:HGNC Symbol;Acc:HGNC:24989]
ENSG00000158457	362.191531	310.990234	131.697308	113.907208	1.453897211	3.19E-09	7.39E-08	TSPAN33	tetraspanin 33 [Source:HGNC Symbol;Acc:HGNC:28743]
ENSG00000105204	147.632418	132.901809	350.841628	368.335459	-1.359123323	3.26E-09	7.53E-08	DYRK1B	dual specificity tyrosine phosphorylation regulated kinase 1B [Source:HGNC Symbol;Acc:HGNC:3092]
ENSG00000204386	1919.22143	1842.90509	1065.16783	1175.2669	0.747711061	3.35E-09	7.74E-08	NEU1	neuraminidase 1 [Source:HGNC Symbol;Acc:HGNC:7758]
ENSG00000165175	1213.53847	1109.2871	1924.88785	1938.55165	-0.734366207	3.37E-09	7.78E-08	MID1IP1	MID1 interacting protein 1 [Source:HGNC Symbol;Acc:HGNC:20715]
ENSG00000139318	190.937927	191.378605	47.4110308	53.2276675	1.925745612	3.47E-09	7.99E-08	DUSP6	dual specificity phosphatase 6 [Source:HGNC Symbol;Acc:HGNC:3072]
ENSG00000116254	108.263773	101.005375	7.37504924	17.0328536	3.101186162	3.51E-09	8.07E-08	CHD5	chromodomain helicase DNA binding protein 5 [Source:HGNC Symbol;Acc:HGNC:16816]
ENSG00000150753	14109.7223	13729.6429	19951.6153	20765.1776	-0.548512528	3.52E-09	8.08E-08	CCT5	chaperonin containing TCP1 subunit 5 [Source:HGNC Symbol;Acc:HGNC:1618]
ENSG00000168481	76.7688572	81.5131097	5.26789231	5.32276675	3.901886948	3.60E-09	8.26E-08	LGI3	leucine rich repeat LGI family member 3 [Source:HGNC Symbol;Acc:HGNC:18711]
ENSG00000135316	2261.72864	2703.2228	4497.72646	3862.19955	-0.751406605	3.70E-09	8.46E-08	SYNCRIP	synaptotagmin binding cytoplasmic RNA interacting protein [Source:HGNC Symbol;Acc:HGNC:16918]
ENSG00000121966	767.688572	734.503999	1291.68719	1317.91705	-0.796984925	4.03E-09	9.20E-08	CXCR4	C-X-C motif chemokine receptor 4 [Source:HGNC Symbol;Acc:HGNC:2561]
ENSG00000090889	1745.99939	1986.43904	2947.91254	3106.36668	-0.697468764	4.06E-09	9.27E-08	KIF4A	kinesin family member 4A [Source:HGNC Symbol;Acc:HGNC:13339]
ENSG00000100350	3081.58067	2794.48204	4749.53171	4443.44568	-0.645894189	4.09E-09	9.33E-08	FOXRED2	FAD dependent oxidoreductase domain containing 2 [Source:HGNC Symbol;Acc:HGNC:26264]
ENSG00000149806	4368.93535	3684.03815	6235.07734	6602.35988	-0.672994687	4.10E-09	9.35E-08	FAU	FAU, ubiquitin like and ribosomal protein S30 fusion [Source:HGNC Symbol;Acc:HGNC:3597]
ENSG00000132005	302.154348	308.332198	597.378988	668.539504	-1.051959288	4.12E-09	9.37E-08	RFX1	regulatory factor X1 [Source:HGNC Symbol;Acc:HGNC:9982]
ENSG00000249992	1165.31188	1187.25616	635.307813	686.636911	0.831681708	4.25E-09	9.65E-08	TMEM158	transmembrane protein 158 (gene/pseudogene) [Source:HGNC Symbol;Acc:HGNC:30293]
ENSG00000154277	426.165579	426.171802	193.858437	155.424789	1.286827578	4.28E-09	9.71E-08	UCHL1	ubiquitin C-terminal hydrolase L1 [Source:HGNC Symbol;Acc:HGNC:12513]
ENSG00000117399	3710.49477	3283.5607	5366.92869	5514.38635	-0.637865146	4.29E-09	9.72E-08	CDC20	cell division cycle 20 [Source:HGNC Symbol;Acc:HGNC:1723]
ENSG00000178537	711.588253	660.078986	355.055942	318.301452	1.026122115	4.33E-09	9.78E-08	SLC25A20	solute carrier family 25 member 20 [Source:HGNC Symbol;Acc:HGNC:1421]
ENSG00000173805	121.058583	96.5753147	10.5357846	18.097407	2.926257126	4.39E-09	9.91E-08	HAP1	huntingtin associated protein 1 [Source:HGNC Symbol;Acc:HGNC:4812]
ENSG00000109654	414.354986	443.006031	195.965594	143.714702	1.335700163	4.50E-09	1.02E-07	TRIM2	tripartite motif containing 2 [Source:HGNC Symbol;Acc:HGNC:15974]
ENSG00000126464	958.626499	859.4317	1513.99225	1618.12109	-0.785201848	4.56E-09	1.03E-07	PRR12	proline rich 12 [Source:HGNC Symbol;Acc:HGNC:29217]
ENSG00000178999	1031.45849	861.203724	1587.74274	1770.35222	-0.827887474	4.79E-09	1.08E-07	AURKB	aurora kinase B [Source:HGNC Symbol;Acc:HGNC:11390]
ENSG00000179967	355.302019	273.777727	651.11149	697.282444	-1.101552649	4.82E-09	1.08E-07	PPP1R14BP3	protein phosphatase 1 regulatory inhibitor subunit 14B pseudogene 3 [Source:HGNC Symbol;Acc:HGNC:16330]
ENSG00000127528	387.781151	391.617331	155.929612	163.941216	1.284962912	4.93E-09	1.11E-07	KLF2	Kruppel like factor 2 [Source:HGNC Symbol;Acc:HGNC:6347]
ENSG00000159692	1274.55987	1185.48414	1939.63795	2170.62428	-0.74078414	4.99E-09	1.12E-07	CTBP1	C-terminal binding protein 1 [Source:HGNC Symbol;Acc:HGNC:2494]
ENSG00000141314	289.359539	231.249148	84.286277	81.970608	1.645846134	5.01E-09	1.12E-07	RHBDL3	rhomboid like 3 [Source:HGNC Symbol;Acc:HGNC:16502]
ENSG00000088298	906.463045	913.478436	459.36021	509.921055	0.90900172	5.04E-09	1.13E-07	EDEM2	ER degradation enhancing alpha-mannosidase like protein 2 [Source:HGNC Symbol;Acc:HGNC:15877]
ENSG00000108342	137.790257	92.1452544	16.8572554	15.9683003	2.806759552	5.06E-09	1.13E-07	CSF3	colony stimulating factor 3 [Source:HGNC Symbol;Acc:HGNC:2438]
ENSG00000197956	26784.4574	21911.9643	36785.692	40753.2314	-0.671149291	5.07E-09	1.13E-07	S100A6	S100 calcium binding protein A6 [Source:HGNC Symbol;Acc:HGNC:10496]
ENSG00000153187	13743.5939	14207.2034	20138.0987	20472.4255	-0.538942945	5.10E-09	1.14E-07	HNRNPU	heterogeneous nuclear ribonucleoprotein U [Source:HGNC Symbol;Acc:HGNC:5048]
ENSG00000115461	110.232205	88.6012062	11.5893631	12.7746402	3.028064636	5.13E-09	1.14E-07	IGFBP5	insulin like growth factor binding protein 5 [Source:HGNC Symbol;Acc:HGNC:5474]

ENSG00000079308	935.005312	928.540641	1577.20696	1548.92512	-0.746367331	5.14E-09	1.14E-07	TNS1	tensin 1 [Source:HGNC Symbol;Acc:HGNC:11973]
ENSG00000144554	853.315374	812.473061	1383.34852	1476.5355	-0.779968054	5.23E-09	1.16E-07	FANCD2	FA complementation group D2 [Source:HGNC Symbol;Acc:HGNC:3585]
ENSG00000014216	2082.60131	1775.56817	3077.50269	3220.27388	-0.707328232	5.27E-09	1.16E-07	CAPN1	calpain 1 [Source:HGNC Symbol;Acc:HGNC:1476]
ENSG00000141905	5508.65761	4663.96749	8304.30544	7752.0775	-0.658648923	5.27E-09	1.16E-07	NFIC	nuclear factor I C [Source:HGNC Symbol;Acc:HGNC:7786]
ENSG00000006459	290.343755	352.632801	112.732895	120.294529	1.465117116	5.29E-09	1.17E-07	KDM7A	lysine demethylase 7A [Source:HGNC Symbol;Acc:HGNC:22224]
ENSG00000081052	180.11155	168.342292	38.9824031	46.8403474	2.021499696	5.35E-09	1.18E-07	COL4A4	collagen type IV alpha 4 chain [Source:HGNC Symbol;Acc:HGNC:2206]
ENSG00000233608	104.326909	102.777399	287.62692	289.558511	-1.478826612	5.52E-09	1.22E-07	TWIST2	twist family bHLH transcription factor 2 [Source:HGNC Symbol;Acc:HGNC:20670]
ENSG00000165097	703.714525	757.540313	391.931188	327.882432	1.021643086	5.62E-09	1.24E-07	KDM1B	lysine demethylase 1B [Source:HGNC Symbol;Acc:HGNC:21577]
ENSG00000198034	23945.9782	23053.1478	33244.6148	35202.6502	-0.542371175	5.66E-09	1.24E-07	RPS4X	ribosomal protein S4 X-linked [Source:HGNC Symbol;Acc:HGNC:10424]
ENSG00000158234	97.4373957	135.559845	330.823637	323.624218	-1.487030257	5.66E-09	1.24E-07	FAIM	Fas apoptotic inhibitory molecule [Source:HGNC Symbol;Acc:HGNC:18703]
ENSG00000049130	488.171195	621.980467	264.448194	194.813263	1.273869423	5.73E-09	1.26E-07	KITLG	KIT ligand [Source:HGNC Symbol;Acc:HGNC:6343]
ENSG00000124588	1875.91592	1761.39198	992.470912	1142.26574	0.768722468	5.77E-09	1.26E-07	NQO2	N-ribosyldihydronicotinamide:quinone reductase 2 [Source:HGNC Symbol;Acc:HGNC:7856]
ENSG00000182307	1511.75596	1545.20504	2368.44438	2524.05599	-0.6783812	5.82E-09	1.27E-07	C8orf33	chromosome 8 open reading frame 33 [Source:HGNC Symbol;Acc:HGNC:26104]
ENSG00000123643	819.852026	769.944482	387.716874	431.144107	0.956998789	5.98E-09	1.31E-07	SLC36A1	solute carrier family 36 member 1 [Source:HGNC Symbol;Acc:HGNC:18761]
ENSG00000071655	1251.9229	1127.00734	1940.69153	1998.16664	-0.727865774	5.99E-09	1.31E-07	MBD3	methyl-CpG binding domain protein 3 [Source:HGNC Symbol;Acc:HGNC:6918]
ENSG00000079616	1426.12916	1266.11124	2140.87144	2323.91996	-0.730173106	6.11E-09	1.33E-07	KIF22	kinesin family member 22 [Source:HGNC Symbol;Acc:HGNC:6391]
ENSG00000104369	250.97511	264.917606	82.1791201	89.4224814	1.588339964	6.14E-09	1.34E-07	JPH1	junctophilin 1 [Source:HGNC Symbol;Acc:HGNC:14201]
ENSG00000118162	118.105934	106.321447	319.234274	292.752171	-1.44831967	6.22E-09	1.35E-07	KPTN	kaptin, actin binding protein [Source:HGNC Symbol;Acc:HGNC:6404]
ENSG00000178498	234.243436	254.285462	502.556927	562.084169	-1.123172722	6.25E-09	1.36E-07	DTX3	deltex E3 ubiquitin ligase 3 [Source:HGNC Symbol;Acc:HGNC:24457]
ENSG00000108960	482.265898	562.617659	247.590939	218.233437	1.165960199	6.30E-09	1.37E-07	MMD	monocyte to macrophage differentiation associated [Source:HGNC Symbol;Acc:HGNC:7153]
ENSG00000110400	1872.96327	1620.51606	1024.07827	1021.97122	0.77146679	6.50E-09	1.41E-07	NECTIN1	nectin cell adhesion molecule 1 [Source:HGNC Symbol;Acc:HGNC:9706]
ENSG00000006118	6371.81515	5293.92207	3589.54182	3699.32289	0.678343628	6.52E-09	1.41E-07	TMEM132A	transmembrane protein 132A [Source:HGNC Symbol;Acc:HGNC:31092]
ENSG00000188846	9291.98437	9490.96121	13311.9639	16044.9481	-0.644255017	6.57E-09	1.42E-07	RPL14	ribosomal protein L14 [Source:HGNC Symbol;Acc:HGNC:10305]
ENSG00000111684	1650.53043	1515.96664	955.595665	911.257668	0.762018283	6.57E-09	1.42E-07	LPCAT3	lysophosphatidylcholine acyltransferase 3 [Source:HGNC Symbol;Acc:HGNC:30244]
ENSG00000242265	4608.09987	4889.01456	3196.55705	2999.91134	0.616110646	6.70E-09	1.44E-07	PEG10	paternally expressed 10 [Source:HGNC Symbol;Acc:HGNC:14005]
ENSG00000132434	131.88496	148.850026	330.823637	391.755633	-1.362800714	6.80E-09	1.46E-07	LANCL2	LanC like 2 [Source:HGNC Symbol;Acc:HGNC:6509]
ENSG00000138061	620.056154	739.820072	1370.70558	1163.55681	-0.897359036	6.93E-09	1.49E-07	CYP1B1	cytochrome P450 family 1 subfamily B member 1 [Source:HGNC Symbol;Acc:HGNC:2597]
ENSG00000145860	2581.59888	2806.88621	4207.99238	4103.85317	-0.625098921	7.11E-09	1.52E-07	RNF145	ring finger protein 145 [Source:HGNC Symbol;Acc:HGNC:20853]
ENSG00000100227	1247.98604	1072.96061	1928.04859	1975.81102	-0.750741855	7.23E-09	1.55E-07	POLDIP3	DNA polymerase delta interacting protein 3 [Source:HGNC Symbol;Acc:HGNC:23782]
ENSG00000111639	2566.83564	2403.75072	3680.14957	4444.51024	-0.7089944	7.25E-09	1.55E-07	MRPL51	mitochondrial ribosomal protein L51 [Source:HGNC Symbol;Acc:HGNC:14044]
ENSG00000071073	166.332524	195.808666	48.4646093	44.7112407	1.959102582	7.43E-09	1.59E-07	MGAT4A	alpha-1,3-mannosyl-glycoprotein 4-beta-N-acetylglucosaminyltransferase A [Source:HGNC Symbol;Acc:HGNC:7047]
ENSG00000178921	1602.30384	1255.47909	2404.26605	2557.05715	-0.796469638	7.46E-09	1.59E-07	PFAS	phosphoribosylformylglycinamidine synthase [Source:HGNC Symbol;Acc:HGNC:8863]
ENSG00000028310	1136.76962	1029.54602	1739.45804	1904.48594	-0.750625503	7.46E-09	1.59E-07	BRD9	bromodomain containing 9 [Source:HGNC Symbol;Acc:HGNC:25818]
ENSG00000173926	231.290788	237.451233	80.0719631	66.0023077	1.681976529	7.51E-09	1.60E-07	3-Mar	membrane associated ring-CH-type finger 3 [Source:HGNC Symbol;Acc:HGNC:28728]
ENSG00000090447	364.159964	323.394403	670.075902	705.798871	-1.001595861	7.52E-09	1.60E-07	TFAP4	transcription factor AP-4 [Source:HGNC Symbol;Acc:HGNC:11745]
ENSG00000161179	610.213993	505.026875	1027.239	1053.90782	-0.901013129	7.58E-09	1.61E-07	YDJC	YdjC chitooligosaccharide deacetylase homolog [Source:HGNC Symbol;Acc:HGNC:27158]
ENSG00000176014	2764.66308	2459.56948	3997.27669	4186.88833	-0.647868729	7.61E-09	1.61E-07	TUBB6	tubulin beta 6 class V [Source:HGNC Symbol;Acc:HGNC:20776]
ENSG00000141404	279.517378	278.207787	97.982797	99.0034616	1.501449808	7.63E-09	1.62E-07	GNAL	G protein subunit alpha L [Source:HGNC Symbol;Acc:HGNC:4388]
ENSG00000165672	1889.69495	2005.93131	3451.52304	2920.06984	-0.709679667	7.64E-09	1.62E-07	PRDX3	peroxiredoxin 3 [Source:HGNC Symbol;Acc:HGNC:9354]
ENSG00000158792	543.287297	432.373886	218.090742	199.071476	1.224872085	7.68E-09	1.62E-07	SPATA2L	spermatogenesis associated 2 like [Source:HGNC Symbol;Acc:HGNC:28393]
ENSG00000197122	349.396722	284.409872	614.236244	738.800025	-1.095296634	7.82E-09	1.65E-07	SRC	SRC proto-oncogene, non-receptor tyrosine kinase [Source:HGNC Symbol;Acc:HGNC:11283]
ENSG00000128965	237.196084	245.425341	490.967563	558.890509	-1.120911105	7.84E-09	1.65E-07	CHAC1	ChaC glutathione specific gamma-glutamylcyclotransferase 1 [Source:HGNC Symbol;Acc:HGNC:28680]
ENSG00000173482	705.682957	702.607565	347.680893	369.400013	0.973766096	7.88E-09	1.66E-07	PTPRM	protein tyrosine phosphatase, receptor type M [Source:HGNC Symbol;Acc:HGNC:9675]
ENSG00000254986	798.199272	762.856385	1287.47288	1415.85596	-0.792405399	7.96E-09	1.67E-07	DPP3	dipeptidyl peptidase 3 [Source:HGNC Symbol;Acc:HGNC:3008]
ENSG00000176692	257.864623	257.82951	80.0719631	92.6161415	1.578510874	8.36E-09	1.76E-07	FOXC2	forkhead box C2 [Source:HGNC Symbol;Acc:HGNC:3801]
ENSG00000130332	1102.32205	963.981123	1746.83309	1736.28651	-0.753877556	8.54E-09	1.79E-07	LSM7	LSM7 homolog, U6 small nuclear RNA and mRNA degradation associated [Source:HGNC Symbol;Acc:HGNC:20470]
ENSG00000115414	12439.5075	11340.9544	8246.35862	7260.25385	0.616831306	8.76E-09	1.83E-07	FN1	fibronectin 1 [Source:HGNC Symbol;Acc:HGNC:3778]
ENSG00000188042	351.365154	384.529235	151.715299	145.843809	1.306668729	8.84E-09	1.85E-07	ARL4C	ADP ribosylation factor like GTPase 4C [Source:HGNC Symbol;Acc:HGNC:698]
ENSG00000005238	659.424799	567.933732	273.9304	307.655918	1.077034538	8.95E-09	1.87E-07	FAM214B	family with sequence similarity 214 member B [Source:HGNC Symbol;Acc:HGNC:25666]
ENSG00000145386	852.331158	1056.12638	1748.94025	1627.70207	-0.822408443	8.99E-09	1.88E-07	CCNA2	cyclin A2 [Source:HGNC Symbol;Acc:HGNC:1578]
ENSG00000119915	280.501594	283.52386	96.9292185	104.326228	1.486859969	9.23E-09	1.92E-07	ELOVL3	ELOVL fatty acid elongase 3 [Source:HGNC Symbol;Acc:HGNC:18047]

ENSG00000196159	311.012293	313.64827	126.429415	104.326228	1.436543529	9.35E-09	1.95E-07	FAT4	FAT atypical cadherin 4 [Source:HGNC Symbol;Acc:HGNC:23109]
ENSG00000177106	1057.04811	912.592424	1642.52882	1713.93089	-0.769596629	9.38E-09	1.95E-07	EPS8L2	EPS8 like 2 [Source:HGNC Symbol;Acc:HGNC:21296]
ENSG00000225138	805.088785	582.995937	272.876822	338.527965	1.182179717	9.53E-09	1.98E-07	SLC9A3-AS1	SLC9A3 antisense RNA 1 [Source:HGNC Symbol;Acc:HGNC:40550]
ENSG00000186684	117.121718	111.63752	301.32344	307.655918	-1.413025212	9.55E-09	1.98E-07	CYP27C1	cytochrome P450 family 27 subfamily C member 1 [Source:HGNC Symbol;Acc:HGNC:33480]
ENSG00000139343	1832.61041	1740.12769	2815.16165	2775.29058	-0.646104811	9.58E-09	1.98E-07	SNRPF	small nuclear ribonucleoprotein polypeptide F [Source:HGNC Symbol;Acc:HGNC:11162]
ENSG00000111432	464.550008	489.078658	886.059487	875.062854	-0.88467708	9.59E-09	1.98E-07	FZD10	frizzled class receptor 10 [Source:HGNC Symbol;Acc:HGNC:4039]
ENSG00000071242	595.450752	606.918262	288.680499	300.204045	1.029932446	9.60E-09	1.98E-07	RPS6KA2	ribosomal protein S6 kinase A2 [Source:HGNC Symbol;Acc:HGNC:10431]
ENSG00000092820	6201.54576	6112.59721	8753.12986	9689.56459	-0.582741157	9.66E-09	1.99E-07	EZR	ezrin [Source:HGNC Symbol;Acc:HGNC:12691]
ENSG00000184634	1606.2407	1626.71815	2627.62469	2479.34475	-0.659583559	9.67E-09	1.99E-07	MED12	mediator complex subunit 12 [Source:HGNC Symbol;Acc:HGNC:11957]
ENSG00000119801	685.998634	731.845963	382.448982	322.559665	1.008095396	9.87E-09	2.03E-07	YPEL5	yippee like 5 [Source:HGNC Symbol;Acc:HGNC:18329]
ENSG00000140479	637.772045	591.856057	1114.68601	1069.87612	-0.829553769	9.89E-09	2.03E-07	PCSK6	proprotein convertase subtilisin/kexin type 6 [Source:HGNC Symbol;Acc:HGNC:8569]
ENSG00000100029	1936.93732	1577.10147	2854.14405	3003.105	-0.73759781	1.00E-08	2.06E-07	PES1	pescadillo ribosomal biogenesis factor 1 [Source:HGNC Symbol;Acc:HGNC:8848]
ENSG00000101181	838.552133	733.617987	1355.95548	1373.27382	-0.796366069	1.02E-08	2.09E-07	MTG2	mitochondrial ribosome associated GTPase 2 [Source:HGNC Symbol;Acc:HGNC:16239]
ENSG00000100867	274.596297	265.803619	104.304268	70.2605211	1.629732673	1.04E-08	2.13E-07	DHRS2	dehydrogenase/reductase 2 [Source:HGNC Symbol;Acc:HGNC:18349]
ENSG00000177380	489.155411	373.89709	187.536966	157.553896	1.321399217	1.05E-08	2.16E-07	PPFIA3	PTPRF interacting protein alpha 3 [Source:HGNC Symbol;Acc:HGNC:9247]
ENSG00000197822	121.058583	155.052111	29.5001969	25.5492804	2.327220316	1.06E-08	2.16E-07	OCLN	occludin [Source:HGNC Symbol;Acc:HGNC:8104]
ENSG00000160949	1167.28032	995.877557	1797.40486	1859.7747	-0.758188519	1.07E-08	2.18E-07	TONSL	tonsoku like, DNA repair protein [Source:HGNC Symbol;Acc:HGNC:7801]
ENSG00000143630	519.66611	467.814369	204.394222	237.395397	1.160107495	1.08E-08	2.21E-07	HCN3	hyperpolarization activated cyclic nucleotide gated potassium channel 3 [Source:HGNC Symbol;Acc:HGNC:19183]
ENSG00000072501	6732.03825	6776.22025	10231.3004	9623.56229	-0.555648615	1.09E-08	2.22E-07	SMC1A	structural maintenance of chromosomes 1A [Source:HGNC Symbol;Acc:HGNC:11111]
ENSG00000117114	122.042799	132.901809	24.2323046	24.4847271	2.38806085	1.10E-08	2.23E-07	ADGRL2	adhesion G protein-coupled receptor L2 [Source:HGNC Symbol;Acc:HGNC:18582]
ENSG00000234741	1278.49674	1258.13713	2044.9958	2022.65137	-0.681347105	1.10E-08	2.23E-07	GAS5	growth arrest specific 5 [Source:HGNC Symbol;Acc:HGNC:16355]
ENSG00000065427	3912.25907	3985.28225	5671.41286	6467.1616	-0.620079255	1.11E-08	2.26E-07	KARS	lysyl-tRNA synthetase [Source:HGNC Symbol;Acc:HGNC:6215]
ENSG00000119333	4344.32995	3732.76882	6234.02376	6276.60655	-0.631469483	1.14E-08	2.31E-07	WDR34	WD repeat domain 34 [Source:HGNC Symbol;Acc:HGNC:28296]
ENSG00000165521	176.174685	166.570268	45.3038739	42.582134	1.963101706	1.17E-08	2.37E-07	EML5	echinoderm microtubule associated protein like 5 [Source:HGNC Symbol;Acc:HGNC:18197]
ENSG00000141753	746.035818	702.607565	1210.56165	1300.88419	-0.794098365	1.17E-08	2.37E-07	IGFBP4	insulin like growth factor binding protein 4 [Source:HGNC Symbol;Acc:HGNC:5473]
ENSG00000145741	5736.99575	6413.84132	9008.09585	9245.64585	-0.587007249	1.18E-08	2.38E-07	BTF3	basic transcription factor 3 [Source:HGNC Symbol;Acc:HGNC:1125]
ENSG00000162302	1918.23721	1616.086	2784.60788	3026.52517	-0.717815939	1.19E-08	2.39E-07	RPS6KA4	ribosomal protein S6 kinase A4 [Source:HGNC Symbol;Acc:HGNC:10433]
ENSG00000213563	456.676279	362.378933	812.308994	805.866886	-0.983684996	1.22E-08	2.46E-07	C8orf82	chromosome 8 open reading frame 82 [Source:HGNC Symbol;Acc:HGNC:33826]
ENSG00000167747	1598.36698	1332.56214	2427.44478	2429.31075	-0.729184251	1.24E-08	2.50E-07	C19orf48	chromosome 19 open reading frame 48 [Source:HGNC Symbol;Acc:HGNC:29667]
ENSG00000119899	609.229777	599.830166	303.430597	293.816725	1.017395634	1.26E-08	2.52E-07	SLC17A5	solute carrier family 17 member 5 [Source:HGNC Symbol;Acc:HGNC:10933]
ENSG00000126709	2171.18076	1868.59944	1091.50729	1264.68938	0.777570302	1.27E-08	2.55E-07	IFI6	interferon alpha inducible protein 6 [Source:HGNC Symbol;Acc:HGNC:4054]
ENSG00000139546	430.102444	445.664067	779.648062	878.256514	-0.92047509	1.27E-08	2.55E-07	TARBP2	TARBP2, RISC loading complex RNA binding subunit [Source:HGNC Symbol;Acc:HGNC:11569]
ENSG00000106244	2913.27971	2393.11858	4146.88483	4471.12407	-0.69996931	1.29E-08	2.58E-07	PDAP1	PDGFA associated protein 1 [Source:HGNC Symbol;Acc:HGNC:14634]
ENSG00000105676	802.136136	668.939107	1248.49048	1402.01676	-0.850159851	1.29E-08	2.59E-07	ARMC6	armadillo repeat containing 6 [Source:HGNC Symbol;Acc:HGNC:25049]
ENSG00000128512	341.522993	355.290837	147.500985	129.875509	1.328970137	1.30E-08	2.61E-07	DOCK4	dedicator of cytokinesis 4 [Source:HGNC Symbol;Acc:HGNC:19192]
ENSG00000171456	2839.4635	2678.41446	4127.92042	4253.95519	-0.603291739	1.31E-08	2.62E-07	ASXL1	ASXL transcriptional regulator 1 [Source:HGNC Symbol;Acc:HGNC:18318]
ENSG00000074582	438.960389	365.036969	802.826788	766.478412	-0.966009504	1.32E-08	2.64E-07	BCS1L	BCS1 homolog, ubiquinol-cytochrome c reductase complex chaperone [Source:HGNC Symbol;Acc:HGNC:1020]
ENSG00000196586	668.282744	702.607565	358.216677	341.721625	0.969973827	1.33E-08	2.65E-07	MYO6	myosin VI [Source:HGNC Symbol;Acc:HGNC:7605]
ENSG00000110675	202.74852	183.404497	53.7325016	55.3567742	1.823265151	1.34E-08	2.66E-07	ELMOD1	ELMO domain containing 1 [Source:HGNC Symbol;Acc:HGNC:25334]
ENSG00000224877	818.86781	782.348651	1319.08023	1404.14587	-0.766352996	1.35E-08	2.68E-07	NDUFAF8	NADH:ubiquinone oxidoreductase complex assembly factor 8 [Source:HGNC Symbol;Acc:HGNC:33551]
ENSG00000053254	1258.81242	1422.04936	802.826788	728.154492	0.808531678	1.41E-08	2.80E-07	FOXN3	forkhead box N3 [Source:HGNC Symbol;Acc:HGNC:1928]
ENSG00000095319	5106.11322	4753.45471	7409.81733	7230.44635	-0.570453284	1.41E-08	2.80E-07	NUP188	nucleoporin 188 [Source:HGNC Symbol;Acc:HGNC:17859]
ENSG00000188613	147.632418	131.129785	30.5537754	28.7429405	2.232416207	1.42E-08	2.81E-07	NANOS1	nanos C2HC-type zinc finger 1 [Source:HGNC Symbol;Acc:HGNC:23044]
ENSG00000099203	146.648202	110.751508	368.752462	316.172345	-1.414404876	1.42E-08	2.81E-07	TMED1	transmembrane p24 trafficking protein 1 [Source:HGNC Symbol;Acc:HGNC:17291]
ENSG00000175352	258.848839	295.928029	99.0363755	97.9389082	1.494454961	1.44E-08	2.84E-07	NRIP3	nuclear receptor interacting protein 3 [Source:HGNC Symbol;Acc:HGNC:1167]
ENSG00000087191	2454.635	2204.39801	3531.59501	3740.84047	-0.642659862	1.46E-08	2.88E-07	PSMC5	proteasome 26S subunit, ATPase 5 [Source:HGNC Symbol;Acc:HGNC:9552]
ENSG00000075223	2166.25968	2588.04123	1485.54563	1341.33722	0.750308137	1.47E-08	2.90E-07	SEMA3C	semaphorin 3C [Source:HGNC Symbol;Acc:HGNC:10725]
ENSG00000176978	1515.69282	1281.17344	2210.40761	2487.86118	-0.748793615	1.48E-08	2.91E-07	DPP7	dipeptidyl peptidase 7 [Source:HGNC Symbol;Acc:HGNC:14892]
ENSG00000136854	4327.59827	3943.63969	2648.69625	2738.03122	0.618561895	1.52E-08	2.99E-07	STXBP1	syntaxin binding protein 1 [Source:HGNC Symbol;Acc:HGNC:11444]
ENSG00000101158	2786.31583	2475.5177	3907.72252	4500.93156	-0.676519592	1.55E-08	3.04E-07	NELFCD	negative elongation factor complex member C/D [Source:HGNC Symbol;Acc:HGNC:15934]
ENSG00000140465	383.844286	358.834885	166.465397	127.746402	1.335378124	1.57E-08	3.08E-07	CYP1A1	cytochrome P450 family 1 subfamily A member 1 [Source:HGNC Symbol;Acc:HGNC:2595]

ENSG00000165916	3237.08681	2880.42521	4563.04832	4915.04282	-0.631864647	1.57E-08	3.08E-07	PSMC3	proteasome 26S subunit, ATPase 3 [Source:HGNC Symbol;Acc:HGNC:9549]
ENSG00000158856	124.995447	123.155677	322.395009	312.978685	-1.356543132	1.58E-08	3.09E-07	DMTN	dematin actin binding protein [Source:HGNC Symbol;Acc:HGNC:3382]
ENSG00000166441	15914.7746	14489.8413	21511.965	23993.968	-0.581803444	1.60E-08	3.12E-07	RPL27A	ribosomal protein L27a [Source:HGNC Symbol;Acc:HGNC:10329]
ENSG00000233621	406.481257	337.570596	138.018779	158.618449	1.32610122	1.60E-08	3.12E-07	LINC01137	long intergenic non-protein coding RNA 1137 [Source:HGNC Symbol;Acc:HGNC:49453]
ENSG00000100505	88.5794506	112.523532	11.5893631	15.9683003	2.868917476	1.60E-08	3.12E-07	TRIM9	tripartite motif containing 9 [Source:HGNC Symbol;Acc:HGNC:16288]
ENSG00000198171	730.28836	599.830166	1165.25778	1241.26921	-0.856303308	1.63E-08	3.17E-07	DDRGK1	DDRGK domain containing 1 [Source:HGNC Symbol;Acc:HGNC:16110]
ENSG00000185269	329.7124	257.82951	591.057517	655.764864	-1.087045717	1.65E-08	3.22E-07	NOTUM	notum, palmitoleoyl-protein carboxylesterase [Source:HGNC Symbol;Acc:HGNC:27106]
ENSG00000149212	73.8162089	76.1970373	6.32147077	5.32276675	3.687121786	1.71E-08	3.33E-07	SESN3	sestrin 3 [Source:HGNC Symbol;Acc:HGNC:23060]
ENSG00000104824	3652.42601	3145.34282	5071.92672	5658.10106	-0.658743762	1.75E-08	3.40E-07	HNRNPL	heterogeneous nuclear ribonucleoprotein L [Source:HGNC Symbol;Acc:HGNC:5045]
ENSG00000184887	1076.73243	881.582001	1654.11819	1726.70553	-0.788505099	1.76E-08	3.41E-07	BTBD6	BTB domain containing 6 [Source:HGNC Symbol;Acc:HGNC:19897]
ENSG00000148719	849.37851	750.452216	1305.38371	1501.02022	-0.811301801	1.77E-08	3.42E-07	DNAJB12	DnaJ heat shock protein family (Hsp40) member B12 [Source:HGNC Symbol;Acc:HGNC:14891]
ENSG00000105662	261.801487	230.363136	489.913985	574.858809	-1.114198455	1.85E-08	3.58E-07	CRTC1	CREB regulated transcription coactivator 1 [Source:HGNC Symbol;Acc:HGNC:16062]
ENSG00000147684	1830.64198	1761.39198	2667.66067	3114.8831	-0.686978488	1.85E-08	3.58E-07	NDUFB9	NADH:ubiquinone oxidoreductase subunit B9 [Source:HGNC Symbol;Acc:HGNC:7704]
ENSG00000143772	272.627865	291.497968	554.182271	601.472643	-1.03409165	1.88E-08	3.63E-07	ITPKB	inositol-trisphosphate 3-kinase B [Source:HGNC Symbol;Acc:HGNC:6179]
ENSG00000101150	3222.32357	2990.29071	4541.97675	4956.5604	-0.612640325	1.90E-08	3.66E-07	TPD52L2	tumor protein D52 like 2 [Source:HGNC Symbol;Acc:HGNC:12007]
ENSG00000054148	1595.41433	1321.04398	2363.17649	2468.69922	-0.728915552	1.92E-08	3.70E-07	PHPT1	phosphohistidine phosphatase 1 [Source:HGNC Symbol;Acc:HGNC:30033]
ENSG00000166582	657.456367	668.053095	1116.79317	1177.39601	-0.791330467	1.94E-08	3.72E-07	CENPV	centromere protein V [Source:HGNC Symbol;Acc:HGNC:29920]
ENSG00000182134	412.386554	497.938779	203.340643	195.877816	1.189904786	1.94E-08	3.73E-07	TDRKH	tudor and KH domain containing [Source:HGNC Symbol;Acc:HGNC:11713]
ENSG00000065268	1089.52724	963.981123	1633.04662	1843.8064	-0.760130526	1.97E-08	3.79E-07	WDR18	WD repeat domain 18 [Source:HGNC Symbol;Acc:HGNC:17956]
ENSG00000137752	162.39566	213.528907	416.163493	476.919901	-1.246251722	2.01E-08	3.84E-07	CASP1	caspase 1 [Source:HGNC Symbol;Acc:HGNC:1499]
ENSG00000186193	1339.51814	1115.48919	2050.26369	2049.2652	-0.740351655	2.02E-08	3.87E-07	SAPCD2	suppressor APC domain containing 2 [Source:HGNC Symbol;Acc:HGNC:28055]
ENSG00000169439	427.149795	464.27032	205.4478	193.74871	1.15930341	2.03E-08	3.87E-07	SDC2	syndecan 2 [Source:HGNC Symbol;Acc:HGNC:10659]
ENSG00000012779	86.6110184	86.8291821	12.6429415	4.2582134	3.357228748	2.03E-08	3.87E-07	ALOX5	arachidonate 5-lipoxygenase [Source:HGNC Symbol;Acc:HGNC:435]
ENSG00000120162	147.632418	139.103894	33.7145108	30.8720472	2.150033354	2.03E-08	3.88E-07	MOB3B	MOB kinase activator 3B [Source:HGNC Symbol;Acc:HGNC:23825]
ENSG00000134684	3473.29868	3481.14139	5054.01588	5285.50738	-0.572151999	2.07E-08	3.94E-07	YARS	tyrosyl-tRNA synthetase [Source:HGNC Symbol;Acc:HGNC:12840]
ENSG00000149792	1024.56898	1087.1368	1676.24333	1783.12686	-0.711829992	2.08E-08	3.95E-07	MRPL49	mitochondrial ribosomal protein L49 [Source:HGNC Symbol;Acc:HGNC:1176]
ENSG00000085185	715.525118	659.192974	364.538148	339.592519	0.964853524	2.15E-08	4.09E-07	BCORL1	BCL6 corepressor like 1 [Source:HGNC Symbol;Acc:HGNC:25657]
ENSG00000106733	185.03263	233.021172	48.4646093	69.1959678	1.830337023	2.16E-08	4.10E-07	NMRK1	nicotinamide riboside kinase 1 [Source:HGNC Symbol;Acc:HGNC:26057]
ENSG00000087586	2132.79633	2151.23729	3144.93171	3530.05891	-0.639751591	2.18E-08	4.13E-07	AURKA	aurora kinase A [Source:HGNC Symbol;Acc:HGNC:11393]
ENSG00000164176	482.265898	573.249804	262.341037	211.846117	1.154861208	2.20E-08	4.16E-07	EDIL3	EGF like repeats and discoidin domains 3 [Source:HGNC Symbol;Acc:HGNC:3173]
ENSG00000270647	5094.30263	4609.03475	7005.2432	7723.33456	-0.602186279	2.25E-08	4.26E-07	TAF15	TATA-box binding protein associated factor 15 [Source:HGNC Symbol;Acc:HGNC:11547]
ENSG00000124193	2085.55395	2269.9629	3259.77176	3498.12231	-0.633495442	2.30E-08	4.34E-07	SRSF6	serine and arginine rich splicing factor 6 [Source:HGNC Symbol;Acc:HGNC:10788]
ENSG00000168002	951.736986	885.12605	1497.13499	1549.98968	-0.730527428	2.31E-08	4.36E-07	POLR2G	RNA polymerase II subunit G [Source:HGNC Symbol;Acc:HGNC:9194]
ENSG00000125458	250.97511	225.933076	480.431779	541.857655	-1.100764978	2.33E-08	4.39E-07	NT5C	5', 3'-nucleotidase, cytosolic [Source:HGNC Symbol;Acc:HGNC:17144]
ENSG00000168003	12881.4206	10983.0055	17777.0294	18208.1205	-0.592626274	2.34E-08	4.40E-07	SLC3A2	solute carrier family 3 member 2 [Source:HGNC Symbol;Acc:HGNC:11026]
ENSG00000240891	363.175748	377.441138	162.251083	147.972916	1.255494454	2.36E-08	4.43E-07	PLCXD2	phosphatidylinositol specific phospholipase C X domain containing 2 [Source:HGNC Symbol;Acc:HGNC:26462]
ENSG00000156011	694.856579	798.296868	406.681286	354.496266	0.972439613	2.41E-08	4.53E-07	PSD3	pleckstrin and Sec7 domain containing 3 [Source:HGNC Symbol;Acc:HGNC:19093]
ENSG00000006704	933.03688	870.949857	1444.45607	1577.66807	-0.744656495	2.43E-08	4.55E-07	GTF2IRD1	GTF2I repeat domain containing 1 [Source:HGNC Symbol;Acc:HGNC:4661]
ENSG00000013810	2343.41858	2249.58462	3379.87971	3673.77361	-0.619022486	2.46E-08	4.62E-07	TACC3	transforming acidic coiled-coil containing protein 3 [Source:HGNC Symbol;Acc:HGNC:11524]
ENSG00000129187	774.578085	661.85101	1286.4193	1227.43001	-0.808176779	2.46E-08	4.62E-07	DCTD	dCMP deaminase [Source:HGNC Symbol;Acc:HGNC:2710]
ENSG00000162244	13542.8138	11479.1723	18211.1037	20292.516	-0.621872041	2.52E-08	4.71E-07	RPL29	ribosomal protein L29 [Source:HGNC Symbol;Acc:HGNC:10331]
ENSG00000067225	84891.5928	77267.3399	115504.86	118181.39	-0.527173787	2.53E-08	4.74E-07	PKM	pyruvate kinase M1/2 [Source:HGNC Symbol;Acc:HGNC:9021]
ENSG00000069812	317.901806	292.38398	105.357846	125.617295	1.401572981	2.74E-08	5.12E-07	HES2	hes family bHLH transcription factor 2 [Source:HGNC Symbol;Acc:HGNC:16005]
ENSG00000213949	561.003187	645.016781	315.01996	208.652457	1.203642876	2.80E-08	5.22E-07	ITGA1	integrin subunit alpha 1 [Source:HGNC Symbol;Acc:HGNC:6134]
ENSG00000130255	5512.59448	4675.48565	7584.71135	8135.3167	-0.625902286	2.82E-08	5.25E-07	RPL36	ribosomal protein L36 [Source:HGNC Symbol;Acc:HGNC:13631]
ENSG00000182944	5224.21916	4865.09223	7176.97649	8004.37664	-0.589554	2.87E-08	5.34E-07	EWSR1	EWS RNA binding protein 1 [Source:HGNC Symbol;Acc:HGNC:3508]
ENSG00000089009	15418.7297	16319.4562	22016.6291	26332.7917	-0.607251975	2.93E-08	5.45E-07	RPL6	ribosomal protein L6 [Source:HGNC Symbol;Acc:HGNC:10362]
ENSG00000136942	19218.7881	18717.8908	26154.0317	31094.5388	-0.593650956	3.00E-08	5.58E-07	RPL35	ribosomal protein L35 [Source:HGNC Symbol;Acc:HGNC:10344]
ENSG00000136271	1684.97799	1524.82676	2480.1237	2577.28366	-0.65622588	3.10E-08	5.75E-07	DDX56	DEAD-box helicase 56 [Source:HGNC Symbol;Acc:HGNC:18193]
ENSG00000107829	338.570345	295.042017	616.3434	644.054777	-0.993143423	3.11E-08	5.77E-07	FBXW4	F-box and WD repeat domain containing 4 [Source:HGNC Symbol;Acc:HGNC:10847]
ENSG00000168802	1712.53605	1520.3967	2624.46395	2503.82948	-0.666015983	3.13E-08	5.79E-07	CHTF8	chromosome transmission fidelity factor 8 [Source:HGNC Symbol;Acc:HGNC:24353]
ENSG00000164587	12327.3069	11174.3841	16676.0399	17871.7216	-0.555885253	3.21E-08	5.94E-07	RPS14	ribosomal protein S14 [Source:HGNC Symbol;Acc:HGNC:10387]
ENSG00000146223	2029.45364	1986.43904	2979.51989	3171.30443	-0.615108335	3.28E-08	6.05E-07	RPL7L1	ribosomal protein L7 like 1 [Source:HGNC Symbol;Acc:HGNC:21370]

ENSG00000137198	133.853392	108.093472	25.2858831	20.2265137	2.409143088	3.36E-08	6.19E-07	GMPR	guanosine monophosphate reductase [Source:HGNC Symbol;Acc:HGNC:4376]
ENSG00000153234	169.285172	178.088424	49.5181877	45.7757941	1.866149861	3.36E-08	6.20E-07	NR4A2	nuclear receptor subfamily 4 group A member 2 [Source:HGNC Symbol;Acc:HGNC:7981]
ENSG00000099282	795.246624	653.876902	357.163099	376.851886	0.980671193	3.41E-08	6.28E-07	TSPAN15	tetraspanin 15 [Source:HGNC Symbol;Acc:HGNC:23298]
ENSG00000124942	19394.9628	19582.6386	29957.45	26786.2914	-0.541815624	3.42E-08	6.29E-07	AHNAK	AHNAK nucleoprotein [Source:HGNC Symbol;Acc:HGNC:347]
ENSG00000142541	17404.8778	15204.853	23280.9233	25608.8954	-0.584283023	3.48E-08	6.39E-07	RPL13A	ribosomal protein L13a [Source:HGNC Symbol;Acc:HGNC:10304]
ENSG00000137693	1887.72651	1940.36642	3266.09323	2802.96897	-0.664804503	3.55E-08	6.51E-07	YAP1	Yes associated protein 1 [Source:HGNC Symbol;Acc:HGNC:16262]
ENSG00000108826	755.877979	707.923637	1178.9543	1361.56373	-0.795666806	3.57E-08	6.55E-07	MRPL27	mitochondrial ribosomal protein L27 [Source:HGNC Symbol;Acc:HGNC:14483]
ENSG00000105185	810.994081	847.913543	1490.81352	1317.91705	-0.75951961	3.60E-08	6.59E-07	PDCD5	programmed cell death 5 [Source:HGNC Symbol;Acc:HGNC:8764]
ENSG00000278845	674.188041	759.312337	1176.84714	1325.36892	-0.803064932	3.64E-08	6.65E-07	MRPL45	mitochondrial ribosomal protein L45 [Source:HGNC Symbol;Acc:HGNC:16651]
ENSG00000136504	598.4034	707.037625	1107.31096	1216.78448	-0.831299048	3.80E-08	6.93E-07	KAT7	lysine acetyltransferase 7 [Source:HGNC Symbol;Acc:HGNC:17016]
ENSG00000167676	1947.7637	1535.4589	1023.02469	974.066315	0.802020035	3.80E-08	6.93E-07	PLIN4	perilipin 4 [Source:HGNC Symbol;Acc:HGNC:29393]
ENSG00000177954	7232.02004	8116.7565	10964.5911	12018.8073	-0.582364199	3.82E-08	6.96E-07	RPS27	ribosomal protein S27 [Source:HGNC Symbol;Acc:HGNC:10416]
ENSG00000182379	937.957961	870.949857	408.788443	531.212122	0.944319691	3.87E-08	7.04E-07	NXPH4	neurexophilin 4 [Source:HGNC Symbol;Acc:HGNC:8078]
ENSG00000014257	66.926696	67.3369167	5.26789231	2.1291067	4.179814672	3.90E-08	7.09E-07	ACPP	acid phosphatase, prostate [Source:HGNC Symbol;Acc:HGNC:125]
ENSG00000133316	710.604037	598.944154	1116.79317	1205.07439	-0.827020336	3.95E-08	7.18E-07	WDR74	WD repeat domain 74 [Source:HGNC Symbol;Acc:HGNC:25529]
ENSG00000196498	7697.55426	6519.27675	10785.4827	10731.7623	-0.598038379	3.99E-08	7.25E-07	NCOR2	nuclear receptor corepressor 2 [Source:HGNC Symbol;Acc:HGNC:7673]
ENSG00000197345	552.145242	501.482827	912.398948	976.195422	-0.842477326	4.01E-08	7.26E-07	MRPL21	mitochondrial ribosomal protein L21 [Source:HGNC Symbol;Acc:HGNC:14479]
ENSG00000188229	15601.7939	13753.5652	21150.5876	22041.5771	-0.557205572	4.01E-08	7.26E-07	TUBB4B	tubulin beta 4B class IVb [Source:HGNC Symbol;Acc:HGNC:20771]
ENSG00000049249	429.118228	460.726272	177.001182	218.233437	1.171300469	4.08E-08	7.38E-07	TNFRSF9	TNF receptor superfamily member 9 [Source:HGNC Symbol;Acc:HGNC:11924]
ENSG00000186648	99.4058279	85.0571579	15.8036769	5.32276675	3.123821744	4.17E-08	7.53E-07	CARMIL3	capping protein regulator and myosin 1 linker 3 [Source:HGNC Symbol;Acc:HGNC:20272]
ENSG00000125843	227.353923	202.01075	462.520945	462.016154	-1.107473152	4.18E-08	7.54E-07	AP5S1	adaptor related protein complex 5 subunit sigma 1 [Source:HGNC Symbol;Acc:HGNC:15875]
ENSG00000182872	2118.03309	1752.53186	3043.78818	3179.82086	-0.685655703	4.20E-08	7.57E-07	RBM10	RNA binding motif protein 10 [Source:HGNC Symbol;Acc:HGNC:9896]
ENSG00000114867	15971.8592	14287.8305	21668.9482	22473.7858	-0.544832307	4.21E-08	7.57E-07	EIF4G1	eukaryotic translation initiation factor 4 gamma 1 [Source:HGNC Symbol;Acc:HGNC:3296]
ENSG00000170889	10496.6649	8595.20301	14110.5763	15663.838	-0.641222939	4.21E-08	7.57E-07	RPS9	ribosomal protein S9 [Source:HGNC Symbol;Acc:HGNC:10442]
ENSG00000136877	2717.4207	2305.40338	3898.24031	3947.36382	-0.643730089	4.31E-08	7.75E-07	FPGS	folylpolyglutamate synthase [Source:HGNC Symbol;Acc:HGNC:3824]
ENSG00000143162	2076.69601	2268.19088	1412.84872	1308.33607	0.675255241	4.33E-08	7.78E-07	CREG1	cellular repressor of E1A stimulated genes 1 [Source:HGNC Symbol;Acc:HGNC:2351]
ENSG00000137547	575.766429	623.752492	1073.59645	1020.90666	-0.803726506	4.35E-08	7.80E-07	MRPL15	mitochondrial ribosomal protein L15 [Source:HGNC Symbol;Acc:HGNC:14054]
ENSG00000244509	851.346942	807.156988	1382.29494	1363.69284	-0.727709742	4.51E-08	8.08E-07	APOBEC3C	apolipoprotein B mRNA editing enzyme catalytic subunit 3C [Source:HGNC Symbol;Acc:HGNC:17353]
ENSG00000251562	2675.09941	3096.61216	1649.90387	1902.35684	0.700532416	4.54E-08	8.13E-07	MALAT1	metastasis associated lung adenocarcinoma transcript 1 [Source:HGNC Symbol;Acc:HGNC:29665]
ENSG00000106366	3012.68554	2653.60613	4272.26066	4376.37882	-0.610327473	4.59E-08	8.20E-07	SERPINE1	serpin family E member 1 [Source:HGNC Symbol;Acc:HGNC:8583]
ENSG00000165092	67.9109122	62.0208443	3.16073539	1.06455335	4.93930785	4.63E-08	8.27E-07	ALDH1A1	aldehyde dehydrogenase 1 family member A1 [Source:HGNC Symbol;Acc:HGNC:402]
ENSG00000105197	1215.50691	1025.11596	1814.26211	1900.22773	-0.729870079	4.67E-08	8.34E-07	TIMM50	translocase of inner mitochondrial membrane 50 [Source:HGNC Symbol;Acc:HGNC:23656]
ENSG00000175264	59.0529671	76.1970373	0	0	8.440248869	4.68E-08	8.34E-07	CHST1	carbohydrate sulfotransferase 1 [Source:HGNC Symbol;Acc:HGNC:1969]
ENSG00000152795	2849.30566	3192.30146	4531.44097	4607.3869	-0.596843977	4.75E-08	8.45E-07	HNRNPDL	heterogeneous nuclear ribonucleoprotein D like [Source:HGNC Symbol;Acc:HGNC:5037]
ENSG00000159784	167.31674	143.533954	45.3038739	24.4847271	2.153822508	4.77E-08	8.49E-07	FAM131B	family with sequence similarity 131 member B [Source:HGNC Symbol;Acc:HGNC:22202]
ENSG00000183617	395.65488	282.637848	679.558108	733.477258	-1.060651231	4.78E-08	8.50E-07	MRPL54	mitochondrial ribosomal protein L54 [Source:HGNC Symbol;Acc:HGNC:16685]
ENSG00000169242	1963.51116	1778.22621	2813.05449	3010.55687	-0.638474115	4.79E-08	8.50E-07	EFNA1	ephrin A1 [Source:HGNC Symbol;Acc:HGNC:3221]
ENSG00000137801	2233.18637	2197.30991	1475.00985	1309.40062	0.670014106	4.87E-08	8.63E-07	THBS1	thrombospondin 1 [Source:HGNC Symbol;Acc:HGNC:11785]
ENSG00000148840	1124.95902	1073.84662	1702.5828	1847.00006	-0.691096718	4.87E-08	8.63E-07	PPRC1	peroxisome proliferator-activated receptor gamma, coactivator-related 1 [Source:HGNC Symbol;Acc:HGNC:30025]
ENSG00000233695	4.92108059	5.31607237	72.6969139	60.679541	-3.701945213	4.92E-08	8.71E-07	GAS6-AS1	GAS6 antisense RNA 1 [Source:HGNC Symbol;Acc:HGNC:39826]
ENSG00000163191	6305.87267	6136.51954	8670.95074	9648.04701	-0.558098211	5.08E-08	8.97E-07	S100A11	S100 calcium binding protein A11 [Source:HGNC Symbol;Acc:HGNC:10488]
ENSG00000106263	5350.19882	4827.87972	7453.01404	7564.71611	-0.561339483	5.13E-08	9.06E-07	EIF3B	eukaryotic translation initiation factor 3 subunit B [Source:HGNC Symbol;Acc:HGNC:3280]
ENSG00000141349	1660.37259	1466.34996	2401.10532	2558.1217	-0.66583676	5.19E-08	9.15E-07	G6PC3	glucose-6-phosphatase catalytic subunit 3 [Source:HGNC Symbol;Acc:HGNC:24861]
ENSG00000116898	1248.97025	1222.69665	1846.92304	2384.5995	-0.77570488	5.26E-08	9.27E-07	MRPS15	mitochondrial ribosomal protein S15 [Source:HGNC Symbol;Acc:HGNC:14504]
ENSG00000182809	1397.58689	1186.37015	733.29061	760.091092	0.790559238	5.27E-08	9.28E-07	CRIP2	cysteine rich protein 2 [Source:HGNC Symbol;Acc:HGNC:2361]
ENSG00000102038	208.653817	243.653317	490.967563	473.726241	-1.091592431	5.30E-08	9.33E-07	SMARCA1	SWI/SNF related, matrix associated, actin dependent regulator of chromatin, subfamily a, member 1 [Source:HGNC Symbol;Acc:HGNC:11097]
ENSG00000143569	3908.32221	3693.78429	5469.1258	5739.00711	-0.560170794	5.37E-08	9.44E-07	UBAP2L	ubiquitin associated protein 2 like [Source:HGNC Symbol;Acc:HGNC:29877]
ENSG00000106105	4278.38747	4647.13326	6475.29323	6656.6521	-0.556939914	5.56E-08	9.75E-07	GARS	glycyl-tRNA synthetase [Source:HGNC Symbol;Acc:HGNC:4162]
ENSG00000094916	2422.15587	2617.27963	3730.72134	3864.32866	-0.591601723	5.69E-08	9.97E-07	CBX5	chromobox 5 [Source:HGNC Symbol;Acc:HGNC:1555]
ENSG00000203727	111.216421	137.33187	26.3394616	24.4847271	2.290700561	5.74E-08	1.00E-06	SAMD5	sterile alpha motif domain containing 5 [Source:HGNC Symbol;Acc:HGNC:21180]
ENSG00000065882	1084.60616	1145.6136	635.307813	667.474951	0.775802927	5.74E-08	1.00E-06	TBC1D1	TBC1 domain family member 1 [Source:HGNC Symbol;Acc:HGNC:11578]

ENSG00000142039	463.565792	422.627753	839.702034	784.575819	-0.874705466	5.77E-08	1.01E-06	CCDC97	coiled-coil domain containing 97 [Source:HGNC Symbol;Acc:HGNC:28289]
ENSG00000115539	298.217484	275.549751	520.46776	698.346998	-1.087325765	5.79E-08	1.01E-06	PDCL3	phosducin like 3 [Source:HGNC Symbol;Acc:HGNC:28860]
ENSG00000105640	13146.1747	11759.1521	17548.4029	19186.445	-0.560752838	5.88E-08	1.02E-06	RPL18A	ribosomal protein L18a [Source:HGNC Symbol;Acc:HGNC:10311]
ENSG00000182685	255.896191	226.819088	74.8040708	90.4870348	1.545860354	5.89E-08	1.02E-06	BRICD5	BRICHOS domain containing 5 [Source:HGNC Symbol;Acc:HGNC:28309]
ENSG00000167969	2159.37016	1772.02412	3056.43112	3278.82432	-0.688804396	5.89E-08	1.02E-06	ECI1	enoyl-CoA delta isomerase 1 [Source:HGNC Symbol;Acc:HGNC:2703]
ENSG00000112081	4313.81925	4906.7348	7835.46302	6591.71434	-0.645733433	5.94E-08	1.03E-06	SRSF3	serine and arginine rich splicing factor 3 [Source:HGNC Symbol;Acc:HGNC:10785]
ENSG00000090861	8840.22917	7760.57965	12104.563	12380.7555	-0.560769702	5.98E-08	1.04E-06	AARS	alanyl-tRNA synthetase [Source:HGNC Symbol;Acc:HGNC:20]
ENSG00000260027	325.775535	353.518813	663.754431	639.796564	-0.939787569	6.01E-08	1.04E-06	HOXB7	homeobox B7 [Source:HGNC Symbol;Acc:HGNC:5118]
ENSG00000105568	4540.18895	3839.97628	6338.32803	6454.38696	-0.610500628	6.03E-08	1.05E-06	PPP2R1A	protein phosphatase 2 scaffold subunit Aalpha [Source:HGNC Symbol;Acc:HGNC:9302]
ENSG00000171490	2857.17939	3168.37913	4352.33263	4941.65665	-0.624997456	6.06E-08	1.05E-06	RSL1D1	ribosomal L1 domain containing 1 [Source:HGNC Symbol;Acc:HGNC:24534]
ENSG00000084710	193.890575	186.062533	63.2147077	48.9694541	1.759465979	6.18E-08	1.07E-06	EFR3B	EFR3 homolog B [Source:HGNC Symbol;Acc:HGNC:29155]
ENSG00000204469	8240.84156	7232.51646	11522.9876	11313.0085	-0.561639744	6.21E-08	1.07E-06	PRRC2A	proline rich coiled-coil 2A [Source:HGNC Symbol;Acc:HGNC:13918]
ENSG00000106789	733.241008	678.685239	364.538148	384.303759	0.914671398	6.35E-08	1.10E-06	CORO2A	coronin 2A [Source:HGNC Symbol;Acc:HGNC:2255]
ENSG00000007520	962.563364	925.882605	1509.77794	1548.92512	-0.695891988	6.37E-08	1.10E-06	TSR3	TSR3, acp transferase ribosome maturation factor [Source:HGNC Symbol;Acc:HGNC:14175]
ENSG00000162613	2815.84231	2917.63772	4089.99159	4605.25779	-0.600724626	6.42E-08	1.11E-06	FUBP1	far upstream element binding protein 1 [Source:HGNC Symbol;Acc:HGNC:4004]
ENSG00000105220	11548.7919	10110.2836	15325.3523	17290.4755	-0.590671142	6.46E-08	1.11E-06	GPI	glucose-6-phosphate isomerase [Source:HGNC Symbol;Acc:HGNC:4458]
ENSG00000102103	1027.52163	787.664723	1536.1174	1667.09055	-0.820298556	6.51E-08	1.12E-06	PQBP1	polyglutamine binding protein 1 [Source:HGNC Symbol;Acc:HGNC:9330]
ENSG00000276850	239.164517	222.389028	502.556927	464.145261	-1.067177642	6.53E-08	1.12E-06	AC245041.2	novel transcript
ENSG00000169813	6255.67765	6063.86655	8685.70084	9042.31616	-0.525117697	6.53E-08	1.12E-06	HNRNPF	heterogeneous nuclear ribonucleoprotein F [Source:HGNC Symbol;Acc:HGNC:5039]
ENSG00000084070	1439.90818	1324.58803	824.951936	843.126253	0.728587846	6.63E-08	1.14E-06	SMAP2	small ArfGAP2 [Source:HGNC Symbol;Acc:HGNC:25082]
ENSG00000099822	1232.23858	1112.83115	680.611687	693.024231	0.771314889	6.65E-08	1.14E-06	HCN2	hyperpolarization activated cyclic nucleotide gated potassium and sodium channel 2 [Source:HGNC Symbol;Acc:HGNC:4846]
ENSG00000175220	1491.08742	1384.83685	2248.33644	2232.36838	-0.639967629	6.90E-08	1.18E-06	ARHGAP1	Rho GTPase activating protein 1 [Source:HGNC Symbol;Acc:HGNC:673]
ENSG00000076770	261.801487	309.21821	672.183059	529.083015	-1.071986843	6.92E-08	1.18E-06	MBNL3	muscleblind like splicing regulator 3 [Source:HGNC Symbol;Acc:HGNC:20564]
ENSG00000163739	124.995447	163.026219	34.7680893	33.0011539	2.088487769	7.04E-08	1.20E-06	CXCL1	C-X-C motif chemokine ligand 1 [Source:HGNC Symbol;Acc:HGNC:4602]
ENSG00000163513	2238.10745	2449.82335	1554.02823	1404.14587	0.66439887	7.09E-08	1.21E-06	TGFBR2	transforming growth factor beta receptor 2 [Source:HGNC Symbol;Acc:HGNC:11773]
ENSG00000105649	241.132949	197.58069	66.3754431	75.5832879	1.627121177	7.14E-08	1.22E-06	RAB3A	RAB3A, member RAS oncogene family [Source:HGNC Symbol;Acc:HGNC:9777]
ENSG00000142173	558.050539	479.332525	256.019566	241.653611	1.05903392	7.21E-08	1.23E-06	COL6A2	collagen type VI alpha 2 chain [Source:HGNC Symbol;Acc:HGNC:2212]
ENSG00000160094	332.665048	287.067908	595.271831	625.95737	-0.97961144	7.31E-08	1.24E-06	ZNF362	zinc finger protein 362 [Source:HGNC Symbol;Acc:HGNC:18079]
ENSG00000197467	107.279557	65.5648926	9.48220616	10.6455335	3.100669745	7.35E-08	1.25E-06	COL13A1	collagen type XIII alpha 1 chain [Source:HGNC Symbol;Acc:HGNC:2190]
ENSG00000166145	1154.48551	1065.87251	637.41497	660.023077	0.774880773	7.37E-08	1.25E-06	SPINT1	serine peptidase inhibitor, Kunitz type 1 [Source:HGNC Symbol;Acc:HGNC:11246]
ENSG00000198218	1774.54166	1677.22083	2598.12449	2686.93266	-0.614760324	7.41E-08	1.26E-06	QRICH1	glutamine rich 1 [Source:HGNC Symbol;Acc:HGNC:24713]
ENSG00000184575	3335.50842	3689.35423	5490.19737	5067.27395	-0.587556977	7.59E-08	1.28E-06	XPOT	exportin for tRNA [Source:HGNC Symbol;Acc:HGNC:12826]
ENSG00000122591	1094.44832	1399.01305	722.754825	654.70031	0.856686512	7.66E-08	1.30E-06	FAM126A	family with sequence similarity 126 member A [Source:HGNC Symbol;Acc:HGNC:24587]
ENSG00000179292	168.300956	170.114316	34.7680893	54.2922209	1.926532252	7.71E-08	1.30E-06	TMEM151A	transmembrane protein 151A [Source:HGNC Symbol;Acc:HGNC:28497]
ENSG00000170473	544.271513	500.596815	898.702428	948.517035	-0.822502858	7.71E-08	1.30E-06	PYM1	PYM homolog 1, exon junction complex associated factor [Source:HGNC Symbol;Acc:HGNC:30258]
ENSG00000004142	3421.13523	3072.68983	4773.76401	4944.85031	-0.581883458	7.81E-08	1.32E-06	POLDIP2	DNA polymerase delta interacting protein 2 [Source:HGNC Symbol;Acc:HGNC:23781]
ENSG00000116990	142.711337	123.155677	24.2323046	34.0657072	2.189068531	7.81E-08	1.32E-06	MYCL	MYCL proto-oncogene, bHLH transcription factor [Source:HGNC Symbol;Acc:HGNC:7555]
ENSG00000141569	867.0944	753.996265	1316.97308	1422.24328	-0.757400341	7.94E-08	1.34E-06	TRIM65	tripartite motif containing 65 [Source:HGNC Symbol;Acc:HGNC:27316]
ENSG00000160789	13446.3606	11613.8461	18270.1041	18621.1672	-0.55795991	8.08E-08	1.36E-06	LMNA	lamin A/C [Source:HGNC Symbol;Acc:HGNC:6636]
ENSG00000123064	2311.92366	1943.02445	3238.70019	3478.96035	-0.659200077	8.22E-08	1.38E-06	DDX54	DEAD-box helicase 54 [Source:HGNC Symbol;Acc:HGNC:20084]
ENSG00000101844	453.72363	483.762586	231.787262	212.91067	1.076168098	8.23E-08	1.38E-06	ATG4A	autophagy related 4A cysteine peptidase [Source:HGNC Symbol;Acc:HGNC:16489]
ENSG00000182899	7335.36273	7292.76528	10063.7815	12383.9491	-0.617817476	8.32E-08	1.39E-06	RPL35A	ribosomal protein L35a [Source:HGNC Symbol;Acc:HGNC:10345]
ENSG00000020256	727.335711	690.203396	1180.00788	1192.29975	-0.743175548	8.50E-08	1.42E-06	ZFP64	ZFP64 zinc finger protein [Source:HGNC Symbol;Acc:HGNC:15940]
ENSG00000160703	501.95022	401.363464	816.523308	881.450174	-0.911708601	8.51E-08	1.42E-06	NLRX1	NLR family member X1 [Source:HGNC Symbol;Acc:HGNC:29890]
ENSG00000197903	649.582638	657.42095	1112.57886	1094.36084	-0.755724231	8.54E-08	1.43E-06	HIST1H2BK	histone cluster 1 H2B family member k [Source:HGNC Symbol;Acc:HGNC:13954]
ENSG00000148291	389.749583	350.860776	658.486539	754.768325	-0.932846467	8.63E-08	1.44E-06	SURF2	surfeit 2 [Source:HGNC Symbol;Acc:HGNC:11475]
ENSG00000185201	1761.74685	1559.38123	2555.98135	2618.80124	-0.640193576	8.70E-08	1.45E-06	IFITM2	interferon induced transmembrane protein 2 [Source:HGNC Symbol;Acc:HGNC:5413]
ENSG00000117280	1043.26909	985.245413	560.503742	607.859963	0.795787325	8.70E-08	1.45E-06	RAB29	RAB29, member RAS oncogene family [Source:HGNC Symbol;Acc:HGNC:9789]
ENSG00000134775	311.012293	221.503015	94.8220616	89.4224814	1.529873787	8.78E-08	1.46E-06	FHOD3	formin homology 2 domain containing 3 [Source:HGNC Symbol;Acc:HGNC:26178]
ENSG00000179111	411.402337	385.415247	151.715299	193.74871	1.205668414	9.07E-08	1.51E-06	HES7	hes family bHLH transcription factor 7 [Source:HGNC Symbol;Acc:HGNC:15977]
ENSG00000010810	901.541964	840.825447	473.05673	503.533735	0.835010946	9.12E-08	1.52E-06	FYN	FYN proto-oncogene, Src family tyrosine kinase [Source:HGNC Symbol;Acc:HGNC:4037]
ENSG00000124406	82.6741539	101.005375	16.8572554	6.3873201	2.981316004	9.22E-08	1.53E-06	ATP8A1	ATPase phospholipid transporting 8A1 [Source:HGNC Symbol;Acc:HGNC:13531]
ENSG00000137501	460.613143	507.684911	250.751674	175.651303	1.183295146	9.31E-08	1.54E-06	SYTL2	synaptotagmin like 2 [Source:HGNC Symbol;Acc:HGNC:15585]
ENSG00000104964	4169.13948	3350.01161	5889.5036	5875.26994	-0.646122594	9.32E-08	1.54E-06	AES	amino-terminal enhancer of split [Source:HGNC Symbol;Acc:HGNC:307]

ENSG00000121774	4119.92867	4210.32932	6013.82586	6015.79098	-0.530118862	9.38E-08	1.55E-06	KHDRBS1	KH RNA binding domain containing, signal transduction associated 1 [Source:HGNC Symbol;Acc:HGNC:18116]
ENSG00000177606	5696.64289	4753.45471	3405.16559	3376.76323	0.623558756	9.43E-08	1.56E-06	JUN	Jun proto-oncogene, AP-1 transcription factor subunit [Source:HGNC Symbol;Acc:HGNC:6204]
ENSG00000167978	11856.8516	10764.1605	16324.1447	16174.8236	-0.522796493	9.44E-08	1.56E-06	SRRM2	serine/arginine repetitive matrix 2 [Source:HGNC Symbol;Acc:HGNC:16639]
ENSG00000105711	189.953711	133.787821	42.1431385	40.4530273	1.969273932	9.55E-08	1.58E-06	SCN1B	sodium voltage-gated channel beta subunit 1 [Source:HGNC Symbol;Acc:HGNC:10586]
ENSG00000178464	634.819396	599.830166	1027.239	1083.71531	-0.774086855	9.70E-08	1.60E-06	RPL10P16	ribosomal protein L10 pseudogene 16 [Source:HGNC Symbol;Acc:HGNC:36882]
ENSG00000133243	1721.39399	1556.72319	2469.58792	2623.05946	-0.635848812	9.75E-08	1.61E-06	BTBD2	BTB domain containing 2 [Source:HGNC Symbol;Acc:HGNC:15504]
ENSG00000182612	119.09015	113.409544	274.983979	311.914132	-1.336279569	1.01E-07	1.66E-06	TSPAN10	tetraspanin 10 [Source:HGNC Symbol;Acc:HGNC:29942]
ENSG00000145246	469.471088	494.39473	243.376625	220.362544	1.055650944	1.01E-07	1.66E-06	ATP10D	ATPase phospholipid transporting 10D (putative) [Source:HGNC Symbol;Acc:HGNC:13549]
ENSG00000168393	698.793444	631.7266	1097.82876	1171.00869	-0.770461847	1.01E-07	1.66E-06	DTYMK	deoxythymidylate kinase [Source:HGNC Symbol;Acc:HGNC:3061]
ENSG00000105447	1136.76962	1033.97608	1729.97584	1738.41562	-0.676466477	1.03E-07	1.69E-06	GRWD1	glutamate rich WD repeat containing 1 [Source:HGNC Symbol;Acc:HGNC:21270]
ENSG00000197594	318.886022	323.394403	138.018779	128.810955	1.26727739	1.04E-07	1.71E-06	ENPP1	ectonucleotide pyrophosphatase/phosphodiesterase 1 [Source:HGNC Symbol;Acc:HGNC:3356]
ENSG00000006016	488.171195	497.052767	234.947997	246.976377	1.03176412	1.05E-07	1.73E-06	CRLF1	cytokine receptor like factor 1 [Source:HGNC Symbol;Acc:HGNC:2364]
ENSG00000071564	3201.65503	2713.85495	4600.97714	4467.93041	-0.616733864	1.06E-07	1.73E-06	TCF3	transcription factor 3 [Source:HGNC Symbol;Acc:HGNC:11633]
ENSG00000100726	1146.61178	958.665051	1658.3325	1868.29113	-0.744876727	1.06E-07	1.73E-06	TELO2	telomere maintenance 2 [Source:HGNC Symbol;Acc:HGNC:29099]
ENSG00000137364	664.34588	746.908168	387.716874	361.948139	0.913048224	1.06E-07	1.74E-06	TPMT	thiopurine S-methyltransferase [Source:HGNC Symbol;Acc:HGNC:12014]
ENSG00000117751	957.642283	940.94481	1560.3497	1490.37469	-0.684321851	1.07E-07	1.75E-06	PPP1R8	protein phosphatase 1 regulatory subunit 8 [Source:HGNC Symbol;Acc:HGNC:9296]
ENSG00000129195	468.486872	418.197693	799.666053	805.866886	-0.857259223	1.08E-07	1.75E-06	PIMREG	PICALM interacting mitotic regulator [Source:HGNC Symbol;Acc:HGNC:25483]
ENSG00000165804	543.287297	468.700381	909.238213	897.418474	-0.83697759	1.08E-07	1.77E-06	ZNF219	zinc finger protein 219 [Source:HGNC Symbol;Acc:HGNC:13011]
ENSG00000140859	2074.72758	1757.84793	3019.55587	2977.55572	-0.646381775	1.10E-07	1.78E-06	KIFC3	kinesin family member C3 [Source:HGNC Symbol;Acc:HGNC:6326]
ENSG00000101182	7255.64122	6446.62376	9695.02901	10544.4009	-0.562860569	1.16E-07	1.89E-06	PSMA7	proteasome subunit alpha 7 [Source:HGNC Symbol;Acc:HGNC:9536]
ENSG00000063046	5893.48612	6645.09046	9279.9191	9075.31731	-0.549689494	1.17E-07	1.89E-06	EIF4B	eukaryotic translation initiation factor 4B [Source:HGNC Symbol;Acc:HGNC:3285]
ENSG00000011304	10368.7168	9313.75879	13663.8591	15750.0668	-0.579648048	1.17E-07	1.90E-06	PTBP1	polypyrimidine tract binding protein 1 [Source:HGNC Symbol;Acc:HGNC:9583]
ENSG00000196155	945.83169	809.815024	1495.02784	1427.56604	-0.735923222	1.17E-07	1.90E-06	PLEKHG4	pleckstrin homology and RhoGEF domain containing G4 [Source:HGNC Symbol;Acc:HGNC:24501]
ENSG00000158220	227.353923	209.984859	79.0183847	69.1959678	1.560564028	1.20E-07	1.94E-06	ESYT3	extended synaptotagmin 3 [Source:HGNC Symbol;Acc:HGNC:24295]
ENSG00000271601	722.414631	751.338228	1215.82955	1221.04269	-0.725329964	1.20E-07	1.94E-06	LIX1L	limb and CNS expressed 1 like [Source:HGNC Symbol;Acc:HGNC:28715]
ENSG00000169435	65.9424799	56.704772	4.21431385	1.06455335	4.534322777	1.20E-07	1.94E-06	RASSF6	Ras association domain family member 6 [Source:HGNC Symbol;Acc:HGNC:20796]
ENSG00000106028	1157.43816	1359.1425	1984.94182	2068.42716	-0.68709762	1.22E-07	1.97E-06	SSBP1	single stranded DNA binding protein 1 [Source:HGNC Symbol;Acc:HGNC:11317]
ENSG00000117519	1311.96009	1367.11661	820.737622	821.835186	0.705910737	1.26E-07	2.02E-06	CNN3	calponin 3 [Source:HGNC Symbol;Acc:HGNC:2157]
ENSG00000053372	1224.36485	1164.21985	1778.44044	2105.68653	-0.701567235	1.26E-07	2.03E-06	MRTO4	MRT4 homolog, ribosome maturation factor [Source:HGNC Symbol;Acc:HGNC:18477]
ENSG00000149503	2290.27091	1932.39231	3317.71858	3252.21049	-0.638130375	1.28E-07	2.05E-06	INCENP	inner centromere protein [Source:HGNC Symbol;Acc:HGNC:6058]
ENSG00000164442	1325.73911	1393.69697	857.612868	802.673226	0.711994706	1.30E-07	2.10E-06	CITED2	Cbp/p300 interacting transactivator with Glu/Asp rich carboxy-terminal domain 2 [Source:HGNC Symbol;Acc:HGNC:1987]
ENSG00000074219	511.792381	479.332525	861.827182	880.385621	-0.814171406	1.31E-07	2.10E-06	TEAD2	TEA domain transcription factor 2 [Source:HGNC Symbol;Acc:HGNC:11715]
ENSG00000157557	1595.41433	1442.42764	2309.44399	2402.69691	-0.63366146	1.31E-07	2.10E-06	ETS2	ETS proto-oncogene 2, transcription factor [Source:HGNC Symbol;Acc:HGNC:3489]
ENSG00000167964	415.339202	294.156005	138.018779	143.714702	1.331282196	1.31E-07	2.10E-06	RAB26	RAB26, member RAS oncogene family [Source:HGNC Symbol;Acc:HGNC:14259]
ENSG00000242372	1195.82258	1022.45792	1757.36888	1845.93551	-0.700446864	1.32E-07	2.12E-06	EIF6	eukaryotic translation initiation factor 6 [Source:HGNC Symbol;Acc:HGNC:6159]
ENSG00000120437	434.039308	454.524188	784.915954	803.737779	-0.8379543	1.33E-07	2.13E-06	ACAT2	acetyl-CoA acetyltransferase 2 [Source:HGNC Symbol;Acc:HGNC:94]
ENSG00000101955	198.811656	149.736038	52.6789231	44.7112407	1.838167106	1.35E-07	2.16E-06	SRPX	sushi repeat containing protein X-linked [Source:HGNC Symbol;Acc:HGNC:11309]
ENSG00000176438	396.639096	387.187271	182.269074	174.586749	1.135046518	1.35E-07	2.16E-06	SYNE3	spectrin repeat containing nuclear envelope family member 3 [Source:HGNC Symbol;Acc:HGNC:19861]
ENSG00000196205	2527.46699	2964.59636	4356.54694	4080.43299	-0.619080626	1.39E-07	2.21E-06	EEF1A1P5	eukaryotic translation elongation factor 1 alpha 1 pseudogene 5 [Source:HGNC Symbol;Acc:HGNC:3200]
ENSG00000169093	1121.02216	946.260882	574.200262	599.343536	0.816365818	1.39E-07	2.22E-06	ASMTL	acetylserotonin O-methyltransferase like [Source:HGNC Symbol;Acc:HGNC:751]
ENSG00000137460	329.7124	363.264945	135.911622	158.618449	1.23494682	1.39E-07	2.22E-06	FHDC1	FH2 domain containing 1 [Source:HGNC Symbol;Acc:HGNC:29363]
ENSG00000130826	2038.31158	2114.91079	2975.30558	3405.50617	-0.61938973	1.40E-07	2.22E-06	DKC1	dyskerin pseudouridine synthase 1 [Source:HGNC Symbol;Acc:HGNC:2890]
ENSG00000145882	546.239946	560.845635	288.680499	276.783871	0.969335162	1.40E-07	2.23E-06	PCYOX1L	prenylcysteine oxidase 1 like [Source:HGNC Symbol;Acc:HGNC:28477]
ENSG00000134278	901.541964	918.794508	522.574917	526.953908	0.794539329	1.40E-07	2.23E-06	SPIRE1	spire type actin nucleation factor 1 [Source:HGNC Symbol;Acc:HGNC:30622]
ENSG00000198231	2242.04432	2078.5843	3120.69941	3474.70214	-0.610389594	1.42E-07	2.25E-06	DDX42	DEAD-box helicase 42 [Source:HGNC Symbol;Acc:HGNC:18676]
ENSG00000134440	1935.9531	2160.09741	3075.39553	3150.01336	-0.603633747	1.43E-07	2.26E-06	NARS	asparaginyl-tRNA synthetase [Source:HGNC Symbol;Acc:HGNC:7643]
ENSG00000236081	62.9898316	53.1607237	178.05476	194.813263	-1.68452393	1.44E-07	2.27E-06	ELFN1-AS1	ELFN1 antisense RNA 1 [Source:HGNC Symbol;Acc:HGNC:39071]
ENSG00000196743	1340.50235	1342.30827	682.718844	865.481874	0.793263136	1.44E-07	2.28E-06	GM2A	GM2 ganglioside activator [Source:HGNC Symbol;Acc:HGNC:4367]

ENSG00000133816	1053.11125	965.753147	593.164674	582.310683	0.779988534	1.45E-07	2.30E-06	MICAL2	microtubule associated monooxygenase, calponin and LIM domain containing 2 [Source:HGNC Symbol;Acc:HGNC:24693]
ENSG00000111961	871.031265	886.898074	499.396191	507.791948	0.80362285	1.46E-07	2.31E-06	SASH1	SAM and SH3 domain containing 1 [Source:HGNC Symbol;Acc:HGNC:19182]
ENSG00000133794	757.846411	738.048047	420.377806	406.65938	0.854856505	1.49E-07	2.35E-06	ARNTL	aryl hydrocarbon receptor nuclear translocator like [Source:HGNC Symbol;Acc:HGNC:701]
ENSG00000087085	57.0845349	61.1348323	2.10715692	2.1291067	4.802812313	1.51E-07	2.37E-06	ACHE	acetylcholinesterase (Cartwright blood group) [Source:HGNC Symbol;Acc:HGNC:108]
ENSG00000150782	1353.29716	1352.94042	857.612868	805.866886	0.70204997	1.55E-07	2.44E-06	IL18	interleukin 18 [Source:HGNC Symbol;Acc:HGNC:5986]
ENSG00000124562	1348.37608	1377.74876	2021.81707	2223.85195	-0.639037599	1.55E-07	2.44E-06	SNRPC	small nuclear ribonucleoprotein polypeptide C [Source:HGNC Symbol;Acc:HGNC:11157]
ENSG00000198816	279.517378	284.409872	547.8608	550.374082	-0.961474293	1.57E-07	2.47E-06	ZNF358	zinc finger protein 358 [Source:HGNC Symbol;Acc:HGNC:16838]
ENSG00000125651	1787.33647	1590.39165	2499.08811	2789.12978	-0.647036569	1.59E-07	2.49E-06	GTF2F1	general transcription factor IIF subunit 1 [Source:HGNC Symbol;Acc:HGNC:4652]
ENSG00000011105	932.052664	844.369495	507.824819	501.404628	0.81535737	1.60E-07	2.51E-06	TSPAN9	tetraspanin 9 [Source:HGNC Symbol;Acc:HGNC:21640]
ENSG00000120733	2012.72196	1878.34557	2872.05489	2993.52402	-0.592301279	1.60E-07	2.51E-06	KDM3B	lysine demethylase 3B [Source:HGNC Symbol;Acc:HGNC:1337]
ENSG00000110367	1571.79314	1723.29346	2640.26763	2446.3436	-0.626121096	1.61E-07	2.52E-06	DDX6	DEAD-box helicase 6 [Source:HGNC Symbol;Acc:HGNC:2747]
ENSG00000010292	8302.84717	7493.00401	11093.1276	11789.9284	-0.534821409	1.64E-07	2.57E-06	NCAPD2	non-SMC condensin I complex subunit D2 [Source:HGNC Symbol;Acc:HGNC:24305]
ENSG00000121058	265.738352	261.373558	522.574917	518.437482	-0.981935903	1.66E-07	2.59E-06	COIL	coilin [Source:HGNC Symbol;Acc:HGNC:2184]
ENSG00000241468	1685.96221	1475.21008	2391.62311	2549.60527	-0.644801311	1.66E-07	2.59E-06	ATP5MF	ATP synthase membrane subunit f [Source:HGNC Symbol;Acc:HGNC:848]
ENSG00000197296	851.346942	735.390011	430.913591	438.59598	0.867281166	1.67E-07	2.60E-06	FITM2	fat storage inducing transmembrane protein 2 [Source:HGNC Symbol;Acc:HGNC:16135]
ENSG00000275832	2321.76582	2021.87953	3302.96848	3322.47101	-0.609451269	1.69E-07	2.63E-06	ARHGAP23	Rho GTPase activating protein 23 [Source:HGNC Symbol;Acc:HGNC:29293]
ENSG00000068078	524.587191	486.420622	244.430203	257.621911	1.009611824	1.69E-07	2.64E-06	FGFR3	fibroblast growth factor receptor 3 [Source:HGNC Symbol;Acc:HGNC:3690]
ENSG00000110076	94.4847473	49.6166755	4.21431385	7.45187345	3.625780067	1.70E-07	2.64E-06	NRXN2	neurexin 2 [Source:HGNC Symbol;Acc:HGNC:8009]
ENSG00000141002	1571.79314	1235.98683	2277.83664	2338.82371	-0.718073739	1.70E-07	2.65E-06	TCF25	transcription factor 25 [Source:HGNC Symbol;Acc:HGNC:29181]
ENSG00000130816	5695.65868	5139.75597	7681.64057	8120.41296	-0.544480712	1.70E-07	2.65E-06	DNMT1	DNA methyltransferase 1 [Source:HGNC Symbol;Acc:HGNC:2976]
ENSG00000113273	476.360601	484.648598	795.451739	928.290521	-0.842764763	1.73E-07	2.68E-06	ARSB	arylsulfatase B [Source:HGNC Symbol;Acc:HGNC:714]
ENSG00000076248	2354.24495	2111.36674	3315.61142	3418.28081	-0.592853873	1.73E-07	2.68E-06	UNG	uracil DNA glycosylase [Source:HGNC Symbol;Acc:HGNC:12572]
ENSG00000086619	232.275004	294.156005	106.411425	83.0351613	1.475124835	1.74E-07	2.69E-06	ERO1B	endoplasmic reticulum oxidoreductase 1 beta [Source:HGNC Symbol;Acc:HGNC:14355]
ENSG00000245910	427.149795	469.586393	782.808798	822.89974	-0.839850561	1.76E-07	2.72E-06	SNHG6	small nucleolar RNA host gene 6 [Source:HGNC Symbol;Acc:HGNC:32965]
ENSG00000103257	39091.0958	33646.308	52019.383	53426.739	-0.535765132	1.82E-07	2.82E-06	SLC7A5	solute carrier family 7 member 5 [Source:HGNC Symbol;Acc:HGNC:11063]
ENSG00000162415	509.823949	493.508718	269.716086	200.13603	1.094183214	1.83E-07	2.83E-06	ZSWIM5	zinc finger SWIM-type containing 5 [Source:HGNC Symbol;Acc:HGNC:29299]
ENSG00000117020	1265.70193	1389.26691	837.594878	767.542966	0.726228502	1.84E-07	2.83E-06	AKT3	AKT serine/threonine kinase 3 [Source:HGNC Symbol;Acc:HGNC:393]
ENSG00000101161	2657.38352	2380.71441	3656.97084	3946.29927	-0.593974065	1.85E-07	2.85E-06	PRPF6	pre-mRNA processing factor 6 [Source:HGNC Symbol;Acc:HGNC:15860]
ENSG00000111879	60.0371832	77.9690614	7.37504924	5.32276675	3.442377591	1.86E-07	2.86E-06	FAM184A	family with sequence similarity 184 member A [Source:HGNC Symbol;Acc:HGNC:20991]
ENSG00000173327	1418.25543	1245.73296	2117.69271	2067.36261	-0.652117106	1.88E-07	2.89E-06	MAP3K11	mitogen-activated protein kinase kinase kinase 11 [Source:HGNC Symbol;Acc:HGNC:6850]
ENSG00000144749	240.148733	242.767305	90.6077478	88.3579281	1.432110702	1.88E-07	2.89E-06	LRIG1	leucine rich repeats and immunoglobulin like domains 1 [Source:HGNC Symbol;Acc:HGNC:17360]
ENSG00000111203	236.211868	224.161052	446.717268	499.275521	-1.039377522	1.89E-07	2.90E-06	ITFG2	integrin alpha FG-GAP repeat containing 2 [Source:HGNC Symbol;Acc:HGNC:30879]
ENSG00000089847	98.4216118	101.005375	17.9108339	18.097407	2.469588139	1.91E-07	2.93E-06	ANKRD24	ankyrin repeat domain 24 [Source:HGNC Symbol;Acc:HGNC:29424]
ENSG00000176476	127.948095	112.523532	274.983979	321.495112	-1.311691319	1.95E-07	2.99E-06	SGF29	SAGA complex associated factor 29 [Source:HGNC Symbol;Acc:HGNC:25156]
ENSG00000174804	547.224162	656.534938	322.395009	284.235745	0.989218007	1.95E-07	2.99E-06	FZD4	frizzled class receptor 4 [Source:HGNC Symbol;Acc:HGNC:4042]
ENSG00000187653	714.540902	747.79418	1133.65043	1364.7574	-0.772474759	1.96E-07	2.99E-06	TMSB4XP8	TMSB4X pseudogene 8 [Source:HGNC Symbol;Acc:HGNC:11885]
ENSG00000101365	1244.04917	1127.00734	1867.99461	1855.51649	-0.651521622	1.96E-07	3.00E-06	IDH3B	isocitrate dehydrogenase 3 (NAD(+)) beta [Source:HGNC Symbol;Acc:HGNC:5385]
ENSG00000163468	9533.11732	9001.88255	12650.3166	14113.8483	-0.530087871	2.01E-07	3.07E-06	CCT3	chaperonin containing TCP1 subunit 3 [Source:HGNC Symbol;Acc:HGNC:1616]
ENSG00000109971	24394.7807	26422.6517	35476.094	35849.8986	-0.489078085	2.01E-07	3.07E-06	HSPA8	heat shock protein family A (Hsp70) member 8 [Source:HGNC Symbol;Acc:HGNC:5241]
ENSG00000143379	334.63348	367.695006	639.522127	671.733164	-0.900074263	2.03E-07	3.10E-06	SETDB1	SET domain bifurcated 1 [Source:HGNC Symbol;Acc:HGNC:10761]
ENSG00000152061	450.770982	558.187599	252.858831	229.943524	1.06407938	2.08E-07	3.17E-06	RABGAP1L	RAB GTPase activating protein 1 like [Source:HGNC Symbol;Acc:HGNC:24663]
ENSG00000092841	11474.9757	9313.75879	15643.533	15667.0317	-0.590960977	2.15E-07	3.27E-06	MYL6	myosin light chain 6 [Source:HGNC Symbol;Acc:HGNC:7587]
ENSG00000151229	222.432843	276.435763	102.197111	61.7440943	1.605751063	2.16E-07	3.28E-06	SLC2A13	solute carrier family 2 member 13 [Source:HGNC Symbol;Acc:HGNC:15956]
ENSG00000147130	1880.837	1725.06548	2756.16126	2696.51364	-0.596872841	2.16E-07	3.28E-06	ZMYM3	zinc finger MYM-type containing 3 [Source:HGNC Symbol;Acc:HGNC:13054]
ENSG00000197958	14498.4876	13870.5188	19164.5922	21680.6935	-0.525871509	2.17E-07	3.30E-06	RPL12	ribosomal protein L12 [Source:HGNC Symbol;Acc:HGNC:10302]
ENSG00000100395	570.845349	535.151285	936.631253	955.968909	-0.775389176	2.19E-07	3.32E-06	L3MBTL2	L3MBTL2, polycomb repressive complex 1 subunit [Source:HGNC Symbol;Acc:HGNC:18594]
ENSG00000100461	702.730308	746.908168	1189.49008	1195.49341	-0.717968398	2.19E-07	3.32E-06	RBM23	RNA binding motif protein 23 [Source:HGNC Symbol;Acc:HGNC:20155]
ENSG00000170873	127.948095	108.979484	28.4466185	22.3556204	2.220454101	2.19E-07	3.32E-06	MTSS1	MTSS1, I-BAR domain containing [Source:HGNC Symbol;Acc:HGNC:20443]
ENSG00000096384	27374.9871	26284.4338	36248.367	39106.3673	-0.489877353	2.22E-07	3.36E-06	HSP90AB1	heat shock protein 90 alpha family class B member 1 [Source:HGNC Symbol;Acc:HGNC:5258]
ENSG00000275023	2529.43542	2194.65188	3699.11398	3489.60588	-0.606029233	2.29E-07	3.46E-06	MLLT6	MLLT6, PHD finger containing [Source:HGNC Symbol;Acc:HGNC:7138]
ENSG00000106829	283.454242	302.130113	119.054366	121.359082	1.284665459	2.37E-07	3.58E-06	TLE4	transducin like enhancer of split 4 [Source:HGNC Symbol;Acc:HGNC:11840]
ENSG00000128283	2557.97769	2224.77629	3569.52383	3663.12808	-0.596998133	2.38E-07	3.60E-06	CDC42EP1	CDC42 effector protein 1 [Source:HGNC Symbol;Acc:HGNC:17014]
ENSG00000096092	974.373957	1071.18858	504.664083	636.602903	0.842248609	2.40E-07	3.62E-06	TMEM14A	transmembrane protein 14A [Source:HGNC Symbol;Acc:HGNC:21076]

ENSG00000169718	1305.07057	1133.20943	1844.81589	2085.46001	-0.689210322	2.40E-07	3.62E-06	DUS1L	dihydrouridine synthase 1 like [Source:HGNC Symbol;Acc:HGNC:30086]
ENSG00000196218	220.46441	194.036642	422.484963	447.112407	-1.069952209	2.43E-07	3.65E-06	RYR1	ryanodine receptor 1 [Source:HGNC Symbol;Acc:HGNC:10483]
ENSG00000100077	502.934436	567.04772	274.983979	267.202891	0.981188485	2.47E-07	3.71E-06	GRK3	G protein-coupled receptor kinase 3 [Source:HGNC Symbol;Acc:HGNC:290]
ENSG00000162542	173.222037	138.217882	366.645305	348.108946	-1.200377936	2.47E-07	3.71E-06	TMCO4	transmembrane and coiled-coil domains 4 [Source:HGNC Symbol;Acc:HGNC:27393]
ENSG00000275216	4841.35909	4834.96782	2786.71503	3460.86294	0.631198692	2.48E-07	3.73E-06	AL161431.1	novel transcript
ENSG00000125505	4176.02899	3618.47326	5645.0734	5952.98233	-0.573565517	2.57E-07	3.85E-06	MBOAT7	membrane bound O-acyltransferase domain containing 7 [Source:HGNC Symbol;Acc:HGNC:15505]
ENSG00000108298	16545.6572	16564.8815	22250.5235	24579.4723	-0.500138224	2.58E-07	3.87E-06	RPL19	ribosomal protein L19 [Source:HGNC Symbol;Acc:HGNC:10312]
ENSG00000138074	449.786766	431.487874	763.844385	795.221353	-0.823264772	2.61E-07	3.91E-06	SLC5A6	solute carrier family 5 member 6 [Source:HGNC Symbol;Acc:HGNC:11041]
ENSG00000013374	2102.28563	2269.07689	1418.11661	1435.01792	0.615724037	2.62E-07	3.91E-06	NUB1	negative regulator of ubiquitin like proteins 1 [Source:HGNC Symbol;Acc:HGNC:17623]
ENSG00000221869	764.735924	856.773664	1283.25857	1383.91936	-0.717431491	2.67E-07	3.99E-06	CEBPD	CCAAT enhancer binding protein delta [Source:HGNC Symbol;Acc:HGNC:1835]
ENSG00000140365	1870.99484	1675.44881	2636.05331	2768.90326	-0.608223997	2.69E-07	4.02E-06	COMMD4	COMM domain containing 4 [Source:HGNC Symbol;Acc:HGNC:26027]
ENSG00000167513	1837.53149	1660.3866	2611.82101	2698.64274	-0.602641889	2.69E-07	4.02E-06	CDT1	chromatin licensing and DNA replication factor 1 [Source:HGNC Symbol;Acc:HGNC:24576]
ENSG00000109685	3309.91881	3038.13536	4546.19106	4751.1016	-0.550661937	2.70E-07	4.02E-06	NSD2	nuclear receptor binding SET domain protein 2 [Source:HGNC Symbol;Acc:HGNC:12766]
ENSG00000073417	1477.30839	1545.20504	966.13145	941.065162	0.664417233	2.73E-07	4.06E-06	PDE8A	phosphodiesterase 8A [Source:HGNC Symbol;Acc:HGNC:8793]
ENSG00000163704	400.57596	356.176849	183.322652	154.360236	1.163517884	2.77E-07	4.12E-06	PRRT3	proline rich transmembrane protein 3 [Source:HGNC Symbol;Acc:HGNC:26591]
ENSG00000166165	10470.0911	9126.81025	6600.66907	6832.3034	0.544758353	2.78E-07	4.14E-06	CKB	creatine kinase B [Source:HGNC Symbol;Acc:HGNC:1991]
ENSG00000161800	2265.6655	2391.34655	3419.91569	3428.92634	-0.556310577	2.82E-07	4.18E-06	RACGAP1	Rac GTPase activating protein 1 [Source:HGNC Symbol;Acc:HGNC:9804]
ENSG00000073605	180.11155	189.606581	53.7325016	63.873201	1.652900693	2.84E-07	4.21E-06	GSDMB	gasdermin B [Source:HGNC Symbol;Acc:HGNC:23690]
ENSG00000180340	650.566854	633.498624	1052.52488	1079.4571	-0.731613348	2.90E-07	4.30E-06	FZD2	frizzled class receptor 2 [Source:HGNC Symbol;Acc:HGNC:4040]
ENSG00000115525	225.385491	179.860449	37.9288246	75.5832879	1.835905166	2.91E-07	4.31E-06	ST3GAL5	ST3 beta-galactoside alpha-2,3-sialyltransferase 5 [Source:HGNC Symbol;Acc:HGNC:10872]
ENSG00000264230	232.275004	187.834557	453.038739	434.337767	-1.080396274	2.96E-07	4.37E-06	ANXA8L1	annexin A8 like 1 [Source:HGNC Symbol;Acc:HGNC:23334]
ENSG00000179409	1388.72894	1219.1526	2061.85305	2014.13494	-0.644736577	2.96E-07	4.38E-06	GEMIN4	gem nuclear organelle associated protein 4 [Source:HGNC Symbol;Acc:HGNC:15717]
ENSG00000052802	885.794506	1245.73296	614.236244	444.9833	1.009381884	2.98E-07	4.40E-06	MSMO1	methylsterol monooxygenase 1 [Source:HGNC Symbol;Acc:HGNC:10545]
ENSG00000185787	4910.25421	5604.9123	8413.8776	7358.19276	-0.584769472	2.99E-07	4.41E-06	MORF4L1	mortality factor 4 like 1 [Source:HGNC Symbol;Acc:HGNC:16989]
ENSG00000185920	612.182426	578.565876	336.091529	275.719318	0.960356678	3.00E-07	4.43E-06	PTCH1	patched 1 [Source:HGNC Symbol;Acc:HGNC:9585]
ENSG00000160888	3040.24359	2675.75643	1821.63716	1918.32514	0.611762958	3.01E-07	4.43E-06	IER2	immediate early response 2 [Source:HGNC Symbol;Acc:HGNC:28871]
ENSG00000169410	1128.89589	1203.20438	1874.31608	1761.83579	-0.640547381	3.08E-07	4.53E-06	PTPN9	protein tyrosine phosphatase, non-receptor type 9 [Source:HGNC Symbol;Acc:HGNC:9661]
ENSG00000110090	3018.59083	2776.7618	1978.62035	1875.743	0.588223207	3.11E-07	4.57E-06	CPT1A	carnitine palmitoyltransferase 1A [Source:HGNC Symbol;Acc:HGNC:2328]
ENSG00000206077	65.9424799	63.7928684	0	2.1291067	5.935626169	3.12E-07	4.58E-06	ZDHHC11B	zinc finger DHHC-type containing 11B [Source:HGNC Symbol;Acc:HGNC:32962]
ENSG00000126247	4573.6523	4182.86294	6064.39763	6955.79159	-0.572427346	3.14E-07	4.61E-06	CAPNS1	calpain small subunit 1 [Source:HGNC Symbol;Acc:HGNC:1481]
ENSG00000100564	497.02914	530.721225	272.876822	246.976377	0.983504665	3.16E-07	4.63E-06	PIGH	phosphatidylinositol glycan anchor biosynthesis class H [Source:HGNC Symbol;Acc:HGNC:8964]
ENSG00000105516	194.874791	200.238726	382.448982	463.080707	-1.097294011	3.18E-07	4.65E-06	DBP	D-box binding PAR bZIP transcription factor [Source:HGNC Symbol;Acc:HGNC:2697]
ENSG00000157514	373.017909	322.50839	632.147077	668.539504	-0.904024967	3.18E-07	4.66E-06	TSC22D3	TSC22 domain family member 3 [Source:HGNC Symbol;Acc:HGNC:3051]
ENSG00000096070	486.202762	472.244429	823.898358	837.803487	-0.794064131	3.20E-07	4.68E-06	BRPF3	bromodomain and PHD finger containing 3 [Source:HGNC Symbol;Acc:HGNC:14256]
ENSG00000167549	407.465473	335.798571	162.251083	168.199429	1.168679088	3.24E-07	4.73E-06	CORO6	coronin 6 [Source:HGNC Symbol;Acc:HGNC:21356]
ENSG00000089154	6386.57839	5685.5394	9166.13262	8442.97262	-0.544773988	3.26E-07	4.76E-06	GCN1	GCN1, eIF2 alpha kinase activator homolog [Source:HGNC Symbol;Acc:HGNC:4199]
ENSG00000135457	626.945667	555.529563	979.82797	1031.5522	-0.767006089	3.49E-07	5.09E-06	TFCP2	transcription factor CP2 [Source:HGNC Symbol;Acc:HGNC:11748]
ENSG00000125648	2975.28533	2746.63739	1864.83388	1953.4554	0.583428793	3.56E-07	5.19E-06	SLC25A23	solute carrier family 25 member 23 [Source:HGNC Symbol;Acc:HGNC:19375]
ENSG00000197959	223.417059	236.56522	94.8220616	66.0023077	1.515949241	3.64E-07	5.30E-06	DNM3	dynamin 3 [Source:HGNC Symbol;Acc:HGNC:29125]
ENSG00000123989	2504.83002	2090.10245	1508.72436	1366.8865	0.675808885	3.64E-07	5.30E-06	CHPF	chondroitin polymerizing factor [Source:HGNC Symbol;Acc:HGNC:24291]
ENSG00000256663	299.2017	254.285462	538.378594	542.922209	-0.967292451	3.65E-07	5.30E-06	AC112777.1	ubiquitin-like with PHD and ring finger domains 1 (UHRF1) pseudogene
ENSG00000143575	1495.02428	1463.69193	2177.74668	2299.43524	-0.597680544	3.66E-07	5.31E-06	HAX1	HCLS1 associated protein X-1 [Source:HGNC Symbol;Acc:HGNC:16915]
ENSG00000127328	545.255729	562.617659	261.287459	307.655918	0.961684482	3.66E-07	5.32E-06	RAB3IP	RAB3A interacting protein [Source:HGNC Symbol;Acc:HGNC:16508]
ENSG00000049618	1209.60161	1155.35973	1963.87025	1746.93205	-0.650119327	3.69E-07	5.36E-06	ARID1B	AT-rich interaction domain 1B [Source:HGNC Symbol;Acc:HGNC:18040]
ENSG00000110619	1626.90924	1523.94075	2301.01536	2493.18395	-0.605742321	3.71E-07	5.38E-06	CARS	cysteinyl-tRNA synthetase [Source:HGNC Symbol;Acc:HGNC:1493]
ENSG00000106635	806.073001	691.089408	1195.81155	1307.27151	-0.742143822	3.72E-07	5.38E-06	BCL7B	BCL tumor suppressor 7B [Source:HGNC Symbol;Acc:HGNC:1005]
ENSG00000063176	174.206253	157.710147	357.163099	369.400013	-1.131095979	3.72E-07	5.39E-06	SPHK2	sphingosine kinase 2 [Source:HGNC Symbol;Acc:HGNC:18859]
ENSG00000142409	616.11929	593.628081	1003.0067	1013.45479	-0.737325875	3.81E-07	5.51E-06	ZNF787	zinc finger protein 787 [Source:HGNC Symbol;Acc:HGNC:26998]
ENSG00000166510	277.548945	379.213162	141.179514	125.617295	1.300678021	3.84E-07	5.55E-06	CCDC68	coiled-coil domain containing 68 [Source:HGNC Symbol;Acc:HGNC:24350]
ENSG00000205730	1002.91622	999.421606	1601.43926	1531.89227	-0.646042318	3.92E-07	5.66E-06	ITPRIPL2	ITPRIP like 2 [Source:HGNC Symbol;Acc:HGNC:27257]
ENSG00000184743	2132.79633	2189.3358	3097.52068	3263.92057	-0.557525993	3.93E-07	5.67E-06	ATL3	atlastin GTPase 3 [Source:HGNC Symbol;Acc:HGNC:24526]
ENSG00000213619	1001.93201	1010.05375	1509.77794	1672.41331	-0.661334663	3.96E-07	5.70E-06	NDUFS3	NADH:ubiquinone oxidoreductase core subunit S3 [Source:HGNC Symbol;Acc:HGNC:7710]
ENSG00000122026	3236.1026	4526.63562	5980.11135	6998.37372	-0.741180911	3.98E-07	5.73E-06	RPL21	ribosomal protein L21 [Source:HGNC Symbol;Acc:HGNC:10313]

ENSG00000169715	574.782213	626.410528	1008.27459	1007.06747	-0.746082321	4.00E-07	5.75E-06	MT1E	metallothionein 1E [Source:HGNC Symbol;Acc:HGNC:7397]
ENSG00000145592	17322.2037	15638.1129	22349.5599	25587.6043	-0.540450619	4.00E-07	5.75E-06	RPL37	ribosomal protein L37 [Source:HGNC Symbol;Acc:HGNC:10347]
ENSG00000008283	1456.63985	1313.95589	2063.96021	2216.40008	-0.627870954	4.02E-07	5.76E-06	CYB561	cytochrome b561 [Source:HGNC Symbol;Acc:HGNC:2571]
ENSG00000140548	431.08666	378.32715	739.612081	718.573511	-0.850055842	4.02E-07	5.76E-06	ZNF710	zinc finger protein 710 [Source:HGNC Symbol;Acc:HGNC:25352]
ENSG00000110711	991.105631	901.960279	1470.79553	1540.4087	-0.670014081	4.03E-07	5.77E-06	AIP	aryl hydrocarbon receptor interacting protein [Source:HGNC Symbol;Acc:HGNC:358]
ENSG00000034152	2197.75459	1923.53219	1296.95509	1344.53088	0.641455055	4.11E-07	5.88E-06	MAP2K3	mitogen-activated protein kinase kinase 3 [Source:HGNC Symbol;Acc:HGNC:6843]
ENSG00000100599	491.123843	372.125066	770.165856	848.44902	-0.908305923	4.11E-07	5.88E-06	RIN3	Ras and Rab interactor 3 [Source:HGNC Symbol;Acc:HGNC:18751]
ENSG00000100804	2683.95735	2400.20668	3647.48864	3938.8474	-0.577626813	4.14E-07	5.92E-06	PSMB5	proteasome subunit beta 5 [Source:HGNC Symbol;Acc:HGNC:9542]
ENSG00000185650	1431.05024	1632.03422	2407.42679	2307.95166	-0.621984917	4.17E-07	5.96E-06	ZFP36L1	ZFP36 ring finger protein like 1 [Source:HGNC Symbol;Acc:HGNC:1107]
ENSG00000105939	1330.66019	1313.06988	2108.2105	1942.80986	-0.615782744	4.17E-07	5.96E-06	ZC3HAV1	zinc finger CCCH-type containing, antiviral 1 [Source:HGNC Symbol;Acc:HGNC:23721]
ENSG00000164576	1029.49006	1072.96061	548.914379	669.604057	0.787174775	4.22E-07	6.01E-06	SAP30L	SAP30 like [Source:HGNC Symbol;Acc:HGNC:25663]
ENSG00000011201	53.1476704	63.7928684	2.10715692	5.32276675	3.979095297	4.28E-07	6.10E-06	ANOS1	anosmin 1 [Source:HGNC Symbol;Acc:HGNC:6211]
ENSG00000138271	214.559114	256.057486	478.324622	474.790794	-1.016796874	4.29E-07	6.11E-06	GPR87	G protein-coupled receptor 87 [Source:HGNC Symbol;Acc:HGNC:4538]
ENSG00000164715	1826.70512	1646.21041	1147.34695	1046.45594	0.662410864	4.43E-07	6.30E-06	LMTK2	lemur tyrosine kinase 2 [Source:HGNC Symbol;Acc:HGNC:17880]
ENSG00000101443	302.154348	256.057486	112.732895	112.842655	1.306483604	4.48E-07	6.36E-06	WFDC2	WAP four-disulfide core domain 2 [Source:HGNC Symbol;Acc:HGNC:15939]
ENSG00000235097	55.1161026	69.1089408	5.26789231	5.32276675	3.552993245	4.51E-07	6.41E-06	LINC00330	long intergenic non-protein coding RNA 330 [Source:HGNC Symbol;Acc:HGNC:42047]
ENSG00000067141	3613.05737	3429.75269	2444.30203	2380.34129	0.545626653	4.60E-07	6.52E-06	NEO1	neogenin 1 [Source:HGNC Symbol;Acc:HGNC:7754]
ENSG00000106868	120.074366	115.181568	295.001969	270.396551	-1.265466677	4.73E-07	6.70E-06	SUSD1	sushi domain containing 1 [Source:HGNC Symbol;Acc:HGNC:25413]
ENSG00000177666	1948.74791	1712.66132	1113.63243	1202.94529	0.660125019	4.74E-07	6.71E-06	PNPLA2	patatin like phospholipase domain containing 2 [Source:HGNC Symbol;Acc:HGNC:30802]
ENSG00000188735	730.28836	662.737022	350.841628	406.65938	0.878647494	4.86E-07	6.88E-06	TMEM120B	transmembrane protein 120B [Source:HGNC Symbol;Acc:HGNC:32008]
ENSG00000100523	1006.85309	1191.68622	649.004333	649.377544	0.760342178	4.90E-07	6.93E-06	DDHD1	DDHD domain containing 1 [Source:HGNC Symbol;Acc:HGNC:19714]
ENSG00000171608	562.97162	522.747116	302.377019	250.170037	0.97405397	4.91E-07	6.94E-06	PIK3CD	phosphatidylinositol-4,5-bisphosphate 3-kinase catalytic subunit delta [Source:HGNC Symbol;Acc:HGNC:8977]
ENSG00000135842	1128.89589	1204.09039	744.879973	669.604057	0.722048469	4.94E-07	6.97E-06	FAM129A	family with sequence similarity 129 member A [Source:HGNC Symbol;Acc:HGNC:16784]
ENSG00000141854	193.890575	178.088424	66.3754431	55.3567742	1.610922531	4.98E-07	7.02E-06	MISP3	MISP family member 3 [Source:HGNC Symbol;Acc:HGNC:26963]
ENSG00000123095	195.859008	264.917606	55.8396585	92.6161415	1.635510039	5.07E-07	7.14E-06	BHLHE41	basic helix-loop-helix family member e41 [Source:HGNC Symbol;Acc:HGNC:16617]
ENSG00000087301	498.997572	595.400106	300.269862	241.653611	1.014439367	5.07E-07	7.15E-06	TXNDC16	thioredoxin domain containing 16 [Source:HGNC Symbol;Acc:HGNC:19965]
ENSG00000099194	43667.7007	45713.7923	32285.8584	32829.7608	0.456982067	5.19E-07	7.31E-06	SCD	stearoyl-CoA desaturase [Source:HGNC Symbol;Acc:HGNC:10571]
ENSG00000143612	2966.42738	3021.30113	4220.63532	4378.50793	-0.522136216	5.22E-07	7.34E-06	C1orf43	chromosome 1 open reading frame 43 [Source:HGNC Symbol;Acc:HGNC:29876]
ENSG00000117481	346.444074	339.34262	623.71845	627.021923	-0.867092086	5.26E-07	7.39E-06	NSUN4	NOP2/Sun RNA methyltransferase family member 4 [Source:HGNC Symbol;Acc:HGNC:31802]
ENSG00000138496	408.449689	489.078658	787.023111	815.447866	-0.835221504	5.27E-07	7.40E-06	PARP9	poly(ADP-ribose) polymerase family member 9 [Source:HGNC Symbol;Acc:HGNC:24118]
ENSG00000035681	693.872363	738.048047	1211.61523	1127.362	-0.707640146	5.29E-07	7.42E-06	NSMAF	neutral sphingomyelinase activation associated factor [Source:HGNC Symbol;Acc:HGNC:8017]
ENSG00000110042	1284.40203	1221.81063	764.897964	794.156799	0.684694658	5.40E-07	7.56E-06	DTX4	deltex E3 ubiquitin ligase 4 [Source:HGNC Symbol;Acc:HGNC:29151]
ENSG00000111110	910.399909	963.095111	539.432173	570.600596	0.755368125	5.44E-07	7.62E-06	PPM1H	protein phosphatase, Mg2+/Mn2+ dependent 1H [Source:HGNC Symbol;Acc:HGNC:18583]
ENSG00000204681	478.329033	409.337573	185.429809	232.07263	1.087822373	5.46E-07	7.64E-06	GABBR1	gamma-aminobutyric acid type B receptor subunit 1 [Source:HGNC Symbol;Acc:HGNC:4070]
ENSG00000214595	330.696616	337.570596	126.429415	162.876663	1.208141251	5.48E-07	7.67E-06	EML6	echinoderm microtubule associated protein like 6 [Source:HGNC Symbol;Acc:HGNC:35412]
ENSG00000076201	1350.34451	1088.02281	2081.87104	1882.13032	-0.701718706	5.50E-07	7.68E-06	PTPN23	protein tyrosine phosphatase, non-receptor type 23 [Source:HGNC Symbol;Acc:HGNC:14406]
ENSG00000116396	362.191531	287.95392	151.715299	113.907208	1.290292027	5.64E-07	7.87E-06	KCNC4	potassium voltage-gated channel subfamily C member 4 [Source:HGNC Symbol;Acc:HGNC:6236]
ENSG00000187266	553.129458	492.622706	265.501773	273.590211	0.955484687	5.67E-07	7.91E-06	EPOR	erythropoietin receptor [Source:HGNC Symbol;Acc:HGNC:3416]
ENSG00000177189	3356.17696	4080.08554	2552.82061	2091.84733	0.679176009	5.73E-07	7.98E-06	RPS6KA3	ribosomal protein S6 kinase A3 [Source:HGNC Symbol;Acc:HGNC:10432]
ENSG00000115268	11108.8473	10726.062	14706.9018	15755.3896	-0.480409505	5.85E-07	8.15E-06	RPS15	ribosomal protein S15 [Source:HGNC Symbol;Acc:HGNC:10388]
ENSG00000103723	132.869176	99.2333509	25.2858831	26.6138338	2.159833808	6.06E-07	8.43E-06	AP3B2	adaptor related protein complex 3 subunit beta 2 [Source:HGNC Symbol;Acc:HGNC:567]
ENSG00000112096	3615.0258	3972.87809	2675.03572	2454.86003	0.564895461	6.09E-07	8.46E-06	SOD2	superoxide dismutase 2 [Source:HGNC Symbol;Acc:HGNC:11180]
ENSG00000122877	60.0371832	49.6166755	4.21431385	1.06455335	4.372713502	6.23E-07	8.65E-06	EGR2	early growth response 2 [Source:HGNC Symbol;Acc:HGNC:3239]
ENSG00000139636	691.903931	538.695334	324.502166	307.655918	0.960120608	6.30E-07	8.73E-06	LMBR1L	limb development membrane protein 1 like [Source:HGNC Symbol;Acc:HGNC:18268]
ENSG00000066136	664.34588	544.011406	1006.16743	1068.81156	-0.780997808	6.31E-07	8.75E-06	NFYC	nuclear transcription factor Y subunit gamma [Source:HGNC Symbol;Acc:HGNC:7806]
ENSG00000155506	5887.58082	5383.40929	7897.62415	8091.67002	-0.50460328	6.32E-07	8.76E-06	LARP1	La ribonucleoprotein domain family member 1 [Source:HGNC Symbol;Acc:HGNC:29531]
ENSG00000153291	124.995447	147.964014	32.6609323	39.388474	1.92259868	6.35E-07	8.78E-06	SLC25A27	solute carrier family 25 member 27 [Source:HGNC Symbol;Acc:HGNC:21065]
ENSG00000170540	1585.57217	2082.12835	3113.32436	2835.97013	-0.697306238	6.43E-07	8.89E-06	ARL6IP1	ADP ribosylation factor like GTPase 6 interacting protein 1 [Source:HGNC Symbol;Acc:HGNC:697]

ENSG00000160679	1411.36591	1246.61897	1978.62035	2140.81679	-0.632515484	6.45E-07	8.91E-06	CHTOP	chromatin target of PRMT1 [Source:HGNC Symbol;Acc:HGNC:24511]
ENSG00000177096	82.6741539	94.8032906	222.305056	243.782717	-1.391578588	6.53E-07	9.01E-06	PHETA2	PH domain containing endocytic trafficking adaptor 2 [Source:HGNC Symbol;Acc:HGNC:27161]
ENSG00000128165	164.364092	124.927701	330.823637	335.334305	-1.205572285	6.58E-07	9.08E-06	ADM2	adrenomedullin 2 [Source:HGNC Symbol;Acc:HGNC:28898]
ENSG00000138326	10835.2352	12192.412	15628.7829	17963.2732	-0.544684745	6.70E-07	9.23E-06	RPS24	ribosomal protein S24 [Source:HGNC Symbol;Acc:HGNC:10411]
ENSG00000146963	470.455304	408.45156	883.95233	719.638065	-0.868348442	6.83E-07	9.40E-06	LUC7L2	LUC7 like 2, pre-mRNA splicing factor [Source:HGNC Symbol;Acc:HGNC:21608]
ENSG00000092621	6372.79937	5496.81883	8368.57373	8925.21529	-0.543128223	6.84E-07	9.41E-06	PHGDH	phosphoglycerate dehydrogenase [Source:HGNC Symbol;Acc:HGNC:8923]
ENSG00000174428	101.37426	80.6270976	244.430203	232.07263	-1.390750492	6.84E-07	9.41E-06	GTF2IRD2B	GTF2I repeat domain containing 2B [Source:HGNC Symbol;Acc:HGNC:33125]
ENSG00000142459	1325.73911	1134.98145	746.98713	747.316452	0.719150481	7.01E-07	9.63E-06	EVI5L	ecotropic viral integration site 5 like [Source:HGNC Symbol;Acc:HGNC:30464]
ENSG00000130159	491.123843	458.068236	822.844779	806.931439	-0.780335572	7.03E-07	9.66E-06	ECSIT	ECSIT signalling integrator [Source:HGNC Symbol;Acc:HGNC:29548]
ENSG00000083799	872.999697	957.779039	559.450163	516.308375	0.767378608	7.05E-07	9.67E-06	CYLD	CYLD lysine 63 deubiquitinase [Source:HGNC Symbol;Acc:HGNC:2584]
ENSG00000164970	1475.33996	1247.50498	790.183847	863.352767	0.719156277	7.08E-07	9.70E-06	FAM219A	family with sequence similarity 219 member A [Source:HGNC Symbol;Acc:HGNC:19920]
ENSG00000167107	695.840796	653.876902	363.48457	388.561973	0.843564662	7.30E-07	9.99E-06	ACSF2	acyl-CoA synthetase family member 2 [Source:HGNC Symbol;Acc:HGNC:26101]
ENSG00000277586	2422.15587	2412.61084	1475.00985	1699.02715	0.607166907	7.31E-07	1.00E-05	NEFL	neurofilament light [Source:HGNC Symbol;Acc:HGNC:7739]
ENSG00000204060	400.57596	369.46703	172.786868	191.619603	1.079130792	7.66E-07	1.05E-05	FOXO6	forkhead box O6 [Source:HGNC Symbol;Acc:HGNC:24814]
ENSG00000087258	2257.79178	1918.21611	1363.33053	1313.65883	0.641151037	7.75E-07	1.06E-05	GNAO1	G protein subunit alpha o1 [Source:HGNC Symbol;Acc:HGNC:4389]
ENSG00000235750	473.407953	458.954248	251.805252	215.039777	0.997689908	7.76E-07	1.06E-05	KIAA0040	KIAA0040 [Source:HGNC Symbol;Acc:HGNC:28950]
ENSG00000170776	4663.21597	4879.26842	3444.14799	3198.98282	0.52254859	7.83E-07	1.07E-05	AKAP13	A-kinase anchoring protein 13 [Source:HGNC Symbol;Acc:HGNC:371]
ENSG00000163110	1568.84049	1695.82709	1094.66802	981.518189	0.653158329	7.85E-07	1.07E-05	PDLIM5	PDZ and LIM domain 5 [Source:HGNC Symbol;Acc:HGNC:17468]
ENSG00000105281	4461.45166	3855.92449	5892.66434	6303.22039	-0.552392306	7.86E-07	1.07E-05	SLC1A5	solute carrier family 1 member 5 [Source:HGNC Symbol;Acc:HGNC:10943]
ENSG00000089157	36974.0469	34329.4233	48788.0579	49238.7861	-0.459222182	7.95E-07	1.08E-05	RPLP0	ribosomal protein lateral stalk subunit P0 [Source:HGNC Symbol;Acc:HGNC:10371]
ENSG00000169714	5175.99257	5352.39887	7370.83492	7330.51437	-0.481615214	8.15E-07	1.11E-05	CNBP	CCHC-type zinc finger nucleic acid binding protein [Source:HGNC Symbol;Acc:HGNC:13164]
ENSG00000186204	194.874791	215.300931	67.4290216	78.7769479	1.488854639	8.16E-07	1.11E-05	CYP4F12	cytochrome P450 family 4 subfamily F member 12 [Source:HGNC Symbol;Acc:HGNC:18857]
ENSG00000140511	226.369707	161.254195	49.5181877	71.3250745	1.680658172	8.25E-07	1.12E-05	HAPLN3	hyaluronan and proteoglycan link protein 3 [Source:HGNC Symbol;Acc:HGNC:21446]
ENSG00000123610	161.411443	167.45628	57.9468154	42.582134	1.709686504	8.34E-07	1.13E-05	TNFAIP6	TNF alpha induced protein 6 [Source:HGNC Symbol;Acc:HGNC:11898]
ENSG00000197081	12338.1333	11791.0485	9001.77438	7809.56338	0.521307157	8.36E-07	1.13E-05	IGF2R	insulin like growth factor 2 receptor [Source:HGNC Symbol;Acc:HGNC:5467]
ENSG00000242574	64.9582638	52.2747116	2.10715692	0	5.790717263	8.50E-07	1.15E-05	HLA-DMB	major histocompatibility complex, class II, DM beta [Source:HGNC Symbol;Acc:HGNC:4935]
ENSG00000123411	83.65837	94.8032906	220.197899	243.782717	-1.377179469	8.52E-07	1.15E-05	IKZF4	IKAROS family zinc finger 4 [Source:HGNC Symbol;Acc:HGNC:13179]
ENSG00000122390	999.963576	944.488858	1504.51004	1513.79486	-0.63462885	8.53E-07	1.15E-05	NAA60	N(alpha)-acetyltransferase 60, NatF catalytic subunit [Source:HGNC Symbol;Acc:HGNC:25875]
ENSG00000157240	746.035818	722.09983	433.020748	406.65938	0.805901258	8.63E-07	1.17E-05	FZD1	frizzled class receptor 1 [Source:HGNC Symbol;Acc:HGNC:4038]
ENSG00000107262	2720.37335	2425.01501	3592.70256	4189.01743	-0.597008881	8.74E-07	1.18E-05	BAG1	BCL2 associated athanogene 1 [Source:HGNC Symbol;Acc:HGNC:937]
ENSG00000106571	814.930946	751.338228	476.217465	411.982147	0.817993976	8.74E-07	1.18E-05	GLI3	GLI family zinc finger 3 [Source:HGNC Symbol;Acc:HGNC:4319]
ENSG00000136286	181.095766	154.166099	369.80604	352.367159	-1.10839962	8.83E-07	1.19E-05	MYO1G	myosin IG [Source:HGNC Symbol;Acc:HGNC:13880]
ENSG00000182796	219.480194	233.907184	86.3934339	87.2933747	1.384558517	8.85E-07	1.19E-05	TMEM198B	transmembrane protein 198B (pseudogene) [Source:HGNC Symbol;Acc:HGNC:43629]
ENSG00000185664	154.521931	137.33187	48.4646093	30.8720472	1.878158669	8.93E-07	1.20E-05	PMEL	premelanosome protein [Source:HGNC Symbol;Acc:HGNC:10880]
ENSG00000087086	31873.839	33249.3746	43087.1448	47171.4235	-0.470879857	8.94E-07	1.20E-05	FTL	ferritin light chain [Source:HGNC Symbol;Acc:HGNC:3999]
ENSG00000136709	1570.80892	1500.90443	2263.08654	2300.49979	-0.571279732	9.09E-07	1.22E-05	WDR33	WD repeat domain 33 [Source:HGNC Symbol;Acc:HGNC:25651]
ENSG00000186468	5149.41873	5699.71559	7373.99566	8535.58876	-0.55219567	9.12E-07	1.22E-05	RPS23	ribosomal protein S23 [Source:HGNC Symbol;Acc:HGNC:10410]
ENSG00000081059	257.864623	251.627426	104.304268	103.261675	1.295366322	9.15E-07	1.23E-05	TCF7	transcription factor 7 [Source:HGNC Symbol;Acc:HGNC:11639]
ENSG00000135677	5826.55942	6614.96605	4486.13709	3901.58803	0.568904262	9.19E-07	1.23E-05	GNS	glucosamine (N-acetyl)-6-sulfatase [Source:HGNC Symbol;Acc:HGNC:4422]
ENSG00000164651	158.458795	144.419966	31.6073539	52.1631142	1.854452421	9.25E-07	1.24E-05	SP8	Sp8 transcription factor [Source:HGNC Symbol;Acc:HGNC:19196]
ENSG00000160799	407.465473	328.710475	635.307813	740.929132	-0.903776422	9.37E-07	1.25E-05	CCDC12	coiled-coil domain containing 12 [Source:HGNC Symbol;Acc:HGNC:28332]
ENSG00000135632	531.476704	488.192646	842.86277	885.708387	-0.761954404	9.37E-07	1.25E-05	SMYD5	SMYD family member 5 [Source:HGNC Symbol;Acc:HGNC:16258]
ENSG00000204054	4054.97041	3602.52504	2445.35561	2707.15917	0.571449901	9.60E-07	1.28E-05	LINC00963	long intergenic non-protein coding RNA 963 [Source:HGNC Symbol;Acc:HGNC:48716]
ENSG00000204253	330.696616	295.928029	536.271437	658.958524	-0.932231044	9.67E-07	1.29E-05	HNRNPCP2	heterogeneous nuclear ribonucleoprotein C pseudogene 2 [Source:HGNC Symbol;Acc:HGNC:48814]
ENSG00000175183	1337.5497	1420.27734	860.773604	894.224814	0.652268364	9.67E-07	1.29E-05	CSRP2	cysteine and glycine rich protein 2 [Source:HGNC Symbol;Acc:HGNC:2470]
ENSG00000179674	146.648202	155.052111	35.8216677	51.0985608	1.796021921	9.67E-07	1.29E-05	ARL14	ADP ribosylation factor like GTPase 14 [Source:HGNC Symbol;Acc:HGNC:22974]
ENSG00000128917	85.6268023	78.8550735	7.37504924	17.0328536	2.753550919	9.81E-07	1.31E-05	DLL4	delta like canonical Notch ligand 4 [Source:HGNC Symbol;Acc:HGNC:2910]
ENSG00000159871	106.295341	86.8291821	23.1787262	11.7100869	2.466916555	9.81E-07	1.31E-05	LYPD5	LY6/PLAUR domain containing 5 [Source:HGNC Symbol;Acc:HGNC:26397]
ENSG00000001036	1802.09971	1831.38693	2643.42836	2666.70614	-0.547339866	9.87E-07	1.31E-05	FUCA2	alpha-L-fucosidase 2 [Source:HGNC Symbol;Acc:HGNC:4008]
ENSG00000184470	455.692063	397.819416	708.004727	814.383313	-0.835582289	9.88E-07	1.31E-05	TXNRD2	thioredoxin reductase 2 [Source:HGNC Symbol;Acc:HGNC:18155]
ENSG00000250303	86.6110184	61.1348323	2.10715692	12.7746402	3.31310984	9.92E-07	1.32E-05	AP002884.1	uncharacterized LOC283140 [Source:NCBI gene;Acc:283140]

ENSG00000126698	2169.21232	2441.84924	3316.665	3491.73499	-0.561922972	9.95E-07	1.32E-05	DNAJC8	DnaJ heat shock protein family (Hsp40) member C8 [Source:HGNC Symbol;Acc:HGNC:15470]
ENSG00000165802	1760.76264	1668.36071	2446.40919	2654.99606	-0.573202741	9.95E-07	1.32E-05	NSMF	NMDA receptor synaptonuclear signaling and neuronal migration factor [Source:HGNC Symbol;Acc:HGNC:29843]
ENSG00000181856	77.7530733	77.9690614	7.37504924	14.9037469	2.806443943	1.00E-06	1.33E-05	SLC2A4	solute carrier family 2 member 4 [Source:HGNC Symbol;Acc:HGNC:11009]
ENSG00000113742	230.306572	211.756883	93.7684831	63.873201	1.486878355	1.02E-06	1.35E-05	CPEB4	cytoplasmic polyadenylation element binding protein 4 [Source:HGNC Symbol;Acc:HGNC:21747]
ENSG00000165929	176.174685	220.617003	76.9112277	56.4213276	1.573925177	1.04E-06	1.37E-05	TC2N	tandem C2 domains, nuclear [Source:HGNC Symbol;Acc:HGNC:19859]
ENSG00000104381	574.782213	582.109925	326.609323	302.333151	0.879247921	1.04E-06	1.37E-05	GDAP1	ganglioside induced differentiation associated protein 1 [Source:HGNC Symbol;Acc:HGNC:15968]
ENSG00000140691	186.016846	145.305978	341.359422	388.561973	-1.141352582	1.05E-06	1.38E-05	ARMC5	armadillo repeat containing 5 [Source:HGNC Symbol;Acc:HGNC:25781]
ENSG00000124160	830.678404	793.866807	1217.9367	1398.8231	-0.687904497	1.06E-06	1.40E-05	NCOA5	nuclear receptor coactivator 5 [Source:HGNC Symbol;Acc:HGNC:15909]
ENSG00000095261	1338.53392	1595.70772	2313.6583	2222.7874	-0.628057966	1.06E-06	1.40E-05	PSMD5	proteasome 26S subunit, non-ATPase 5 [Source:HGNC Symbol;Acc:HGNC:9563]
ENSG00000178053	499.981788	563.503671	296.055548	256.557357	0.944819845	1.07E-06	1.41E-05	MLF1	myeloid leukemia factor 1 [Source:HGNC Symbol;Acc:HGNC:7125]
ENSG00000172794	60.0371832	48.7306634	3.16073539	4.2582134	3.873809854	1.07E-06	1.41E-05	RAB37	RAB37, member RAS oncogene family [Source:HGNC Symbol;Acc:HGNC:30268]
ENSG00000104897	1393.65002	1173.96598	1903.81628	2157.84964	-0.662141355	1.08E-06	1.42E-05	SF3A2	splicing factor 3a subunit 2 [Source:HGNC Symbol;Acc:HGNC:10766]
ENSG00000204387	312.980726	222.389028	492.021142	627.021923	-1.065470261	1.09E-06	1.43E-05	C6orf48	chromosome 6 open reading frame 48 [Source:HGNC Symbol;Acc:HGNC:19078]
ENSG00000073921	5334.45136	6196.76836	4102.63453	2937.10269	0.712005597	1.10E-06	1.45E-05	PICALM	phosphatidylinositol binding clathrin assembly protein [Source:HGNC Symbol;Acc:HGNC:15514]
ENSG00000159733	287.391107	260.487546	113.786474	117.100869	1.246250902	1.11E-06	1.45E-05	ZFYVE28	zinc finger FYVE-type containing 28 [Source:HGNC Symbol;Acc:HGNC:29334]
ENSG00000173083	463.565792	535.151285	240.215889	270.396551	0.968507363	1.11E-06	1.45E-05	HPSE	heparanase [Source:HGNC Symbol;Acc:HGNC:5164]
ENSG00000008394	2550.10396	2908.7776	3981.47301	4008.04336	-0.549224417	1.11E-06	1.45E-05	MGST1	microsomal glutathione S-transferase 1 [Source:HGNC Symbol;Acc:HGNC:7061]
ENSG00000101997	390.733799	379.213162	650.057911	713.250745	-0.824434709	1.13E-06	1.47E-05	CCDC22	coiled-coil domain containing 22 [Source:HGNC Symbol;Acc:HGNC:28909]
ENSG00000137497	2736.12081	2466.65758	3726.50702	3835.58572	-0.539735464	1.13E-06	1.47E-05	NUMA1	nuclear mitotic apparatus protein 1 [Source:HGNC Symbol;Acc:HGNC:8059]
ENSG00000109814	632.850964	697.291493	1073.59645	1091.16718	-0.702117705	1.13E-06	1.48E-05	UGDH	UDP-glucose 6-dehydrogenase [Source:HGNC Symbol;Acc:HGNC:12525]
ENSG00000125877	905.478829	770.830494	1331.72318	1365.82195	-0.687048448	1.13E-06	1.48E-05	ITPA	inosine triphosphatase [Source:HGNC Symbol;Acc:HGNC:6176]
ENSG00000204256	3392.59296	3232.172	4567.26263	4889.49354	-0.513561638	1.15E-06	1.50E-05	BRD2	bromodomain containing 2 [Source:HGNC Symbol;Acc:HGNC:1103]
ENSG00000165912	1381.83943	1154.47372	1888.0126	2129.1067	-0.663954518	1.16E-06	1.51E-05	PACSIN3	protein kinase C and casein kinase substrate in neurons 3 [Source:HGNC Symbol;Acc:HGNC:8572]
ENSG00000112902	70.8635605	59.3628081	8.4286277	6.3873201	3.134529384	1.16E-06	1.52E-05	SEMA5A	semaphorin 5A [Source:HGNC Symbol;Acc:HGNC:10736]
ENSG00000084072	815.915162	805.384964	1257.97268	1290.23866	-0.652388957	1.17E-06	1.52E-05	PPIE	peptidylprolyl isomerase E [Source:HGNC Symbol;Acc:HGNC:9258]
ENSG00000147274	3396.52982	3647.71166	5160.42731	4887.36443	-0.512236631	1.17E-06	1.52E-05	RBMX	RNA binding motif protein X-linked [Source:HGNC Symbol;Acc:HGNC:9910]
ENSG00000221963	576.750645	579.451888	994.578068	916.580435	-0.725059612	1.19E-06	1.55E-05	APOL6	apolipoprotein L6 [Source:HGNC Symbol;Acc:HGNC:14870]
ENSG00000198918	6363.94142	6526.36485	8613.00393	9556.49543	-0.495195467	1.20E-06	1.56E-05	RPL39	ribosomal protein L39 [Source:HGNC Symbol;Acc:HGNC:10350]
ENSG00000174306	1111.18	1033.97608	606.861194	691.959678	0.723721335	1.20E-06	1.56E-05	ZHX3	zinc fingers and homeoboxes 3 [Source:HGNC Symbol;Acc:HGNC:15935]
ENSG00000100612	1517.66125	1533.68688	886.059487	1040.06862	0.663842161	1.20E-06	1.56E-05	DHRS7	dehydrogenase/reductase 7 [Source:HGNC Symbol;Acc:HGNC:21524]
ENSG00000076984	607.261345	582.995937	934.524096	1033.6813	-0.725789505	1.22E-06	1.59E-05	MAP2K7	mitogen-activated protein kinase kinase 7 [Source:HGNC Symbol;Acc:HGNC:6847]
ENSG00000108883	3982.13841	3711.50453	5347.96427	5547.38751	-0.502094092	1.23E-06	1.60E-05	EFTUD2	elongation factor Tu GTP binding domain containing 2 [Source:HGNC Symbol;Acc:HGNC:30858]
ENSG00000182768	634.819396	682.229288	1014.59606	1157.16949	-0.721156857	1.24E-06	1.60E-05	NGRN	neugrin, neurite outgrowth associated [Source:HGNC Symbol;Acc:HGNC:18077]
ENSG00000072832	308.059645	271.119691	125.375837	124.552742	1.2119175	1.24E-06	1.60E-05	CRMP1	collapsin response mediator protein 1 [Source:HGNC Symbol;Acc:HGNC:2365]
ENSG00000088881	145.663985	129.357761	35.8216677	41.5175807	1.829938979	1.27E-06	1.64E-05	EBF4	early B cell factor 4 [Source:HGNC Symbol;Acc:HGNC:29278]
ENSG00000100360	661.393231	598.944154	340.305843	357.689926	0.85216025	1.27E-06	1.64E-05	IFT27	intraflagellar transport 27 [Source:HGNC Symbol;Acc:HGNC:18626]
ENSG00000071626	3150.47579	2859.16092	4222.74248	4448.76845	-0.529195519	1.27E-06	1.64E-05	DAZAP1	DAZ associated protein 1 [Source:HGNC Symbol;Acc:HGNC:2683]
ENSG00000156381	2933.94825	2624.36773	1833.22652	1910.87326	0.569824501	1.27E-06	1.64E-05	ANKRD9	ankyrin repeat domain 9 [Source:HGNC Symbol;Acc:HGNC:20096]
ENSG00000221890	1567.85628	1424.7074	989.310176	919.774095	0.648195992	1.27E-06	1.64E-05	NPTXR	neuronal pentraxin receptor [Source:HGNC Symbol;Acc:HGNC:7954]
ENSG00000113407	5998.79724	7013.67148	9233.56164	9499.00954	-0.525502455	1.27E-06	1.64E-05	TARS	threonyl-tRNA synthetase [Source:HGNC Symbol;Acc:HGNC:11572]
ENSG00000184371	367.112612	328.710475	621.611293	630.215583	-0.847973167	1.32E-06	1.70E-05	CSF1	colony stimulating factor 1 [Source:HGNC Symbol;Acc:HGNC:2432]
ENSG00000141736	2061.93277	1821.6408	2995.32357	2793.38799	-0.576199968	1.32E-06	1.70E-05	ERBB2	erb-b2 receptor tyrosine kinase 2 [Source:HGNC Symbol;Acc:HGNC:3430]
ENSG00000167088	1985.16391	2142.37717	2914.19803	3147.88426	-0.554309574	1.33E-06	1.71E-05	SNRPD1	small nuclear ribonucleoprotein D1 polypeptide [Source:HGNC Symbol;Acc:HGNC:11158]
ENSG00000154153	384.828502	443.892043	211.769271	193.74871	1.031641965	1.35E-06	1.73E-05	RETREG1	reticulophagy regulator 1 [Source:HGNC Symbol;Acc:HGNC:25964]
ENSG00000134317	142.711337	204.668786	62.1611293	40.4530273	1.760121828	1.36E-06	1.74E-05	GRHL1	grainyhead like transcription factor 1 [Source:HGNC Symbol;Acc:HGNC:17923]
ENSG00000156869	392.702231	446.550079	202.287065	212.91067	1.015895397	1.36E-06	1.74E-05	FRRS1	ferric chelate reductase 1 [Source:HGNC Symbol;Acc:HGNC:27622]
ENSG00000196368	49.2108059	51.3886996	3.16073539	1.06455335	4.570822825	1.36E-06	1.74E-05	NUDT11	nudix hydrolase 11 [Source:HGNC Symbol;Acc:HGNC:18011]
ENSG00000127616	4481.13599	4060.59328	5955.87905	6178.66764	-0.506669436	1.37E-06	1.75E-05	SMARCA4	SWI/SNF related, matrix associated, actin dependent regulator of chromatin, subfamily a, member 4 [Source:HGNC Symbol;Acc:HGNC:11100]

ENSG00000038532	1864.10533	1645.3244	1110.4717	1160.36315	0.627714916	1.41E-06	1.80E-05	CLEC16A	C-type lectin domain containing 16A [Source:HGNC Symbol;Acc:HGNC:29013]
ENSG00000132153	3143.58628	2893.71539	4221.6889	4435.99381	-0.520241717	1.41E-06	1.81E-05	DHX30	DExH-box helicase 30 [Source:HGNC Symbol;Acc:HGNC:16716]
ENSG00000119547	177.158901	191.378605	67.4290216	60.679541	1.524673447	1.42E-06	1.81E-05	ONECUT2	one cut homeobox 2 [Source:HGNC Symbol;Acc:HGNC:8139]
ENSG00000075945	776.546517	852.343603	510.985554	381.110099	0.86872943	1.43E-06	1.82E-05	KIFAP3	kinesin associated protein 3 [Source:HGNC Symbol;Acc:HGNC:17060]
ENSG00000055118	62.0056154	48.7306634	2.10715692	0	5.708435616	1.45E-06	1.84E-05	KCNH2	potassium voltage-gated channel subfamily H member 2 [Source:HGNC Symbol;Acc:HGNC:6251]
ENSG00000180720	77.7530733	81.5131097	12.6429415	13.8391936	2.588703916	1.47E-06	1.87E-05	CHRM4	cholinergic receptor muscarinic 4 [Source:HGNC Symbol;Acc:HGNC:1953]
ENSG00000185298	810.994081	744.250132	1215.82955	1249.78563	-0.665215461	1.47E-06	1.87E-05	CCDC137	coiled-coil domain containing 137 [Source:HGNC Symbol;Acc:HGNC:33451]
ENSG00000182004	677.140689	815.131097	1419.17019	1133.74932	-0.773999867	1.47E-06	1.87E-05	SNRPE	small nuclear ribonucleoprotein polypeptide E [Source:HGNC Symbol;Acc:HGNC:11161]
ENSG00000163840	492.108059	552.871527	888.166644	868.675534	-0.748857703	1.48E-06	1.88E-05	DTX3L	deltex E3 ubiquitin ligase 3L [Source:HGNC Symbol;Acc:HGNC:30323]
ENSG00000198931	3259.72378	2635.88588	4305.97518	4619.09699	-0.598554932	1.49E-06	1.89E-05	APRT	adenine phosphoribosyltransferase [Source:HGNC Symbol;Acc:HGNC:626]
ENSG00000090013	3263.66065	2678.41446	1838.49442	2017.3286	0.623655781	1.49E-06	1.89E-05	BLVRB	biliverdin reductase B [Source:HGNC Symbol;Acc:HGNC:1063]
ENSG00000259330	668.282744	707.037625	375.073933	407.723933	0.813336694	1.50E-06	1.90E-05	INAFM2	InaF motif containing 2 [Source:HGNC Symbol;Acc:HGNC:35165]
ENSG00000169252	175.190469	163.026219	54.78608	57.4858809	1.5906783	1.50E-06	1.90E-05	ADRB2	adrenoceptor beta 2 [Source:HGNC Symbol;Acc:HGNC:286]
ENSG00000140988	49137.9739	42211.3866	62629.9717	66726.204	-0.501904199	1.52E-06	1.92E-05	RPS2	ribosomal protein S2 [Source:HGNC Symbol;Acc:HGNC:10404]
ENSG00000110955	20833.8868	19946.7895	27087.5023	28623.7105	-0.450098153	1.52E-06	1.92E-05	ATP5F1B	ATP synthase F1 subunit beta [Source:HGNC Symbol;Acc:HGNC:830]
ENSG00000144381	9673.86023	11502.2086	15510.7821	14944.1999	-0.524144217	1.52E-06	1.92E-05	HSPD1	heat shock protein family D (Hsp60) member 1 [Source:HGNC Symbol;Acc:HGNC:5261]
ENSG00000164659	480.297466	536.923309	268.662508	268.267444	0.922291707	1.53E-06	1.93E-05	KIAA1324L	KIAA1324 like [Source:HGNC Symbol;Acc:HGNC:21945]
ENSG00000184307	379.907422	346.430716	185.429809	154.360236	1.095449621	1.54E-06	1.94E-05	ZDHHC23	zinc finger DHHC-type containing 23 [Source:HGNC Symbol;Acc:HGNC:28654]
ENSG00000139266	866.110184	788.550735	476.217465	499.275521	0.762015249	1.55E-06	1.95E-05	9-Mar	membrane associated ring-CH-type finger 9 [Source:HGNC Symbol;Acc:HGNC:25139]
ENSG00000167470	1492.07164	1344.0803	2029.19212	2322.85541	-0.618052721	1.56E-06	1.96E-05	MIDN	midnolin [Source:HGNC Symbol;Acc:HGNC:16298]
ENSG00000112306	6162.17712	5874.25997	8059.87524	8867.72941	-0.492016313	1.56E-06	1.96E-05	RPS12	ribosomal protein S12 [Source:HGNC Symbol;Acc:HGNC:10385]
ENSG00000110888	544.271513	564.389683	285.519763	315.107792	0.884502116	1.56E-06	1.96E-05	CAPRIN2	caprin family member 2 [Source:HGNC Symbol;Acc:HGNC:21259]
ENSG00000090674	792.293975	724.757867	449.878003	431.144107	0.783650699	1.56E-06	1.96E-05	MCOLN1	mucolipin 1 [Source:HGNC Symbol;Acc:HGNC:13356]
ENSG00000103544	872.015481	792.094783	1273.77636	1355.17641	-0.660159145	1.57E-06	1.97E-05	VPS35L	VPS35 endosomal protein sorting factor like [Source:HGNC Symbol;Acc:HGNC:24641]
ENSG00000167515	926.147367	800.068892	1369.652	1369.01561	-0.666498606	1.57E-06	1.97E-05	TRAPPC2L	trafficking protein particle complex 2 like [Source:HGNC Symbol;Acc:HGNC:30887]
ENSG00000132326	103.342692	88.6012062	259.180302	222.49165	-1.328877154	1.58E-06	1.98E-05	PER2	period circadian regulator 2 [Source:HGNC Symbol;Acc:HGNC:8846]
ENSG00000106025	283.454242	309.21821	132.750886	129.875509	1.174596394	1.59E-06	1.99E-05	TSPAN12	tetraspanin 12 [Source:HGNC Symbol;Acc:HGNC:21641]
ENSG00000196358	101.37426	68.2229288	1.05357846	0	7.322089836	1.59E-06	1.99E-05	NTNG2	netrin G2 [Source:HGNC Symbol;Acc:HGNC:14288]
ENSG00000168792	1001.93201	835.509374	533.110702	543.986762	0.769973481	1.60E-06	2.00E-05	ABHD15	abhydrolase domain containing 15 [Source:HGNC Symbol;Acc:HGNC:26971]
ENSG00000112379	690.919715	734.503999	439.342219	345.979839	0.860091775	1.61E-06	2.02E-05	ARFGEF3	ARFGEF family member 3 [Source:HGNC Symbol;Acc:HGNC:21213]
ENSG00000035141	784.420246	801.840916	1221.09744	1267.88304	-0.649801688	1.62E-06	2.02E-05	FAM136A	family with sequence similarity 136 member A [Source:HGNC Symbol;Acc:HGNC:25911]
ENSG00000160213	2124.9226	1912.90004	2828.85817	3217.08022	-0.582622759	1.62E-06	2.02E-05	CSTB	cystatin B [Source:HGNC Symbol;Acc:HGNC:2482]
ENSG00000144476	197.82744	155.938123	380.341825	369.400013	-1.08545104	1.63E-06	2.03E-05	ACKR3	atypical chemokine receptor 3 [Source:HGNC Symbol;Acc:HGNC:23692]
ENSG00000261236	2957.56944	2385.14447	3924.57977	4181.56556	-0.601808312	1.64E-06	2.04E-05	BOP1	block of proliferation 1 [Source:HGNC Symbol;Acc:HGNC:15519]
ENSG00000108654	12031.0578	13055.3877	17023.7208	17558.743	-0.46307973	1.65E-06	2.05E-05	DDX5	DEAD-box helicase 5 [Source:HGNC Symbol;Acc:HGNC:2746]
ENSG00000173898	1077.71665	835.509374	1543.49245	1585.11994	-0.710383705	1.69E-06	2.11E-05	SPTBN2	spectrin beta, non-erythrocytic 2 [Source:HGNC Symbol;Acc:HGNC:11276]
ENSG00000124249	95.4689635	104.549423	264.448194	227.814417	-1.29852041	1.69E-06	2.11E-05	KCNK15	potassium two pore domain channel subfamily K member 15 [Source:HGNC Symbol;Acc:HGNC:13814]
ENSG00000004660	438.960389	331.368511	660.593696	784.575819	-0.90908024	1.71E-06	2.12E-05	CAMKK1	calcium/calmodulin dependent protein kinase kinase 1 [Source:HGNC Symbol;Acc:HGNC:1469]
ENSG00000159840	3565.815	2923.8398	4757.96034	4898.00996	-0.573587258	1.72E-06	2.14E-05	ZYX	zyxin [Source:HGNC Symbol;Acc:HGNC:13200]
ENSG00000173786	2315.86053	2021.87953	3056.43112	3452.34651	-0.585727528	1.72E-06	2.14E-05	CNP	2',3'-cyclic nucleotide 3' phosphodiesterase [Source:HGNC Symbol;Acc:HGNC:2158]
ENSG00000142920	142.711337	113.409544	22.1251477	40.4530273	2.032971914	1.73E-06	2.14E-05	AZIN2	antizyme inhibitor 2 [Source:HGNC Symbol;Acc:HGNC:29957]
ENSG00000081181	137.790257	130.243773	40.0359816	36.1948139	1.813584424	1.73E-06	2.15E-05	ARG2	arginase 2 [Source:HGNC Symbol;Acc:HGNC:664]
ENSG00000166166	592.498103	458.068236	925.04189	905.934901	-0.802699128	1.76E-06	2.18E-05	TRMT61A	tRNA methyltransferase 61A [Source:HGNC Symbol;Acc:HGNC:23790]
ENSG00000172239	1984.17969	2290.34118	3365.12961	3035.0416	-0.582035606	1.78E-06	2.20E-05	PAIP1	poly(A) binding protein interacting protein 1 [Source:HGNC Symbol;Acc:HGNC:16945]
ENSG00000119720	218.495978	262.25957	451.98516	489.694541	-0.968597198	1.81E-06	2.24E-05	NRDE2	NRDE-2, necessary for RNA interference, domain containing [Source:HGNC Symbol;Acc:HGNC:20186]
ENSG00000196453	385.812718	393.389355	636.361391	744.122792	-0.824921231	1.82E-06	2.26E-05	ZNF777	zinc finger protein 777 [Source:HGNC Symbol;Acc:HGNC:22213]
ENSG00000060688	1436.95553	1400.78507	2022.87065	2211.07731	-0.577326459	1.83E-06	2.26E-05	SNRNP40	small nuclear ribonucleoprotein U5 subunit 40 [Source:HGNC Symbol;Acc:HGNC:30857]
ENSG00000186174	3407.3562	3174.58122	4988.69402	4486.02782	-0.525716497	1.83E-06	2.26E-05	BCL9L	B cell CLL/lymphoma 9 like [Source:HGNC Symbol;Acc:HGNC:23688]
ENSG00000011426	3090.43861	3871.87271	5447.00065	5071.53216	-0.594998459	1.84E-06	2.27E-05	ANLN	anillin actin binding protein [Source:HGNC Symbol;Acc:HGNC:14082]
ENSG00000131747	5936.79162	7232.51646	10607.428	9130.67409	-0.583665438	1.85E-06	2.28E-05	TOP2A	DNA topoisomerase II alpha [Source:HGNC Symbol;Acc:HGNC:11989]
ENSG00000105767	391.718015	310.104222	143.286671	172.457643	1.151599401	1.86E-06	2.29E-05	CADM4	cell adhesion molecule 4 [Source:HGNC Symbol;Acc:HGNC:30825]
ENSG00000126457	4080.56003	3435.95478	5325.83913	5714.52238	-0.55489126	1.86E-06	2.29E-05	PRMT1	protein arginine methyltransferase 1 [Source:HGNC Symbol;Acc:HGNC:5187]

ENSG00000110107	4470.30961	3966.676	5970.62915	6026.43652	-0.508063641	1.90E-06	2.34E-05	PRPF19	pre-mRNA processing factor 19 [Source:HGNC Symbol;Acc:HGNC:17896]
ENSG00000122140	1253.89133	1156.24574	1814.26211	1816.12802	-0.591326601	1.92E-06	2.36E-05	MRPS2	mitochondrial ribosomal protein S2 [Source:HGNC Symbol;Acc:HGNC:14495]
ENSG00000132603	794.262407	738.93406	1194.75798	1222.10725	-0.656944157	1.93E-06	2.37E-05	NIP7	NIP7, nucleolar pre-rRNA processing protein [Source:HGNC Symbol;Acc:HGNC:24328]
ENSG00000116667	389.749583	386.301259	199.126329	184.16773	1.01759103	1.94E-06	2.39E-05	C1orf21	chromosome 1 open reading frame 21 [Source:HGNC Symbol;Acc:HGNC:15494]
ENSG00000131018	406.481257	351.746789	192.804859	170.328536	1.061418992	1.95E-06	2.39E-05	SYNE1	spectrin repeat containing nuclear envelope protein 1 [Source:HGNC Symbol;Acc:HGNC:17089]
ENSG00000009307	6105.09258	6895.83188	9567.54602	8890.08503	-0.50548724	1.96E-06	2.41E-05	CSDE1	cold shock domain containing E1 [Source:HGNC Symbol;Acc:HGNC:29905]
ENSG00000140941	3484.12506	3737.19888	2325.24767	2618.80124	0.546709542	1.98E-06	2.42E-05	MAP1LC3B	microtubule associated protein 1 light chain 3 beta [Source:HGNC Symbol;Acc:HGNC:13352]
ENSG00000138614	743.083169	791.208771	1166.31136	1264.68938	-0.663645099	2.00E-06	2.44E-05	INTS14	integrator complex subunit 14 [Source:HGNC Symbol;Acc:HGNC:25372]
ENSG00000164120	65.9424799	74.4250132	10.5357846	9.58098015	2.803064824	2.01E-06	2.46E-05	HPGD	15-hydroxyprostaglandin dehydrogenase [Source:HGNC Symbol;Acc:HGNC:5154]
ENSG00000144821	212.590682	191.378605	77.9648062	72.3896278	1.425317663	2.01E-06	2.46E-05	MYH15	myosin heavy chain 15 [Source:HGNC Symbol;Acc:HGNC:31073]
ENSG00000123609	74.800425	103.663411	265.501773	208.652457	-1.406990139	2.02E-06	2.46E-05	NMI	N-myc and STAT interactor [Source:HGNC Symbol;Acc:HGNC:7854]
ENSG00000181649	1673.1674	1572.67141	902.916742	1123.10378	0.679886015	2.02E-06	2.46E-05	PHLDA2	pleckstrin homology like domain family A member 2 [Source:HGNC Symbol;Acc:HGNC:12385]
ENSG00000246695	461.597359	433.259898	224.412212	237.395397	0.954135484	2.02E-06	2.46E-05	RASSF8-AS1	RASSF8 antisense RNA 1 [Source:HGNC Symbol;Acc:HGNC:48637]
ENSG00000163623	55.1161026	57.590784	5.26789231	5.32276675	3.411908319	2.02E-06	2.46E-05	NKX6-1	NK6 homeobox 1 [Source:HGNC Symbol;Acc:HGNC:7839]
ENSG00000188368	19.6843224	9.74613268	79.0183847	89.4224814	-2.52696607	2.03E-06	2.48E-05	PRR19	proline rich 19 [Source:HGNC Symbol;Acc:HGNC:33728]
ENSG00000105127	579.703294	496.166755	857.612868	969.808102	-0.765063042	2.06E-06	2.51E-05	AKAP8	A-kinase anchoring protein 8 [Source:HGNC Symbol;Acc:HGNC:378]
ENSG00000183856	1383.80786	1255.47909	2020.76349	1941.74531	-0.58662854	2.07E-06	2.52E-05	IQGAP3	IQ motif containing GTPase activating protein 3 [Source:HGNC Symbol;Acc:HGNC:20669]
ENSG00000164403	461.597359	409.337573	714.326197	794.156799	-0.793126066	2.13E-06	2.59E-05	SHROOM1	shroom family member 1 [Source:HGNC Symbol;Acc:HGNC:24084]
ENSG00000162736	1692.85172	1689.625	2451.67708	2455.92458	-0.536944164	2.16E-06	2.62E-05	NCSTN	nicastrin [Source:HGNC Symbol;Acc:HGNC:17091]
ENSG00000105373	1587.5406	1355.59845	2132.44281	2386.72861	-0.619136154	2.17E-06	2.63E-05	NOP53	NOP53 ribosome biogenesis factor [Source:HGNC Symbol;Acc:HGNC:4333]
ENSG00000148303	23095.6154	20605.9825	29067.1762	32533.8149	-0.495299923	2.22E-06	2.69E-05	RPL7A	ribosomal protein L7a [Source:HGNC Symbol;Acc:HGNC:10364]
ENSG00000136068	13721.9411	12094.0646	18565.1061	17578.9695	-0.485564122	2.23E-06	2.70E-05	FLNB	filamin B [Source:HGNC Symbol;Acc:HGNC:3755]
ENSG00000228300	667.298528	616.664395	1012.4889	1056.03692	-0.688402274	2.24E-06	2.71E-05	C19orf24	chromosome 19 open reading frame 24 [Source:HGNC Symbol;Acc:HGNC:26073]
ENSG00000177169	4926.98589	4438.03442	6821.92054	6426.70858	-0.500642001	2.24E-06	2.72E-05	ULK1	unc-51 like autophagy activating kinase 1 [Source:HGNC Symbol;Acc:HGNC:12558]
ENSG00000047230	811.978298	822.219193	1223.20459	1333.88535	-0.645830648	2.26E-06	2.73E-05	CTPS2	CTP synthase 2 [Source:HGNC Symbol;Acc:HGNC:2520]
ENSG00000172403	67.9109122	73.5390011	12.6429415	7.45177345	2.814775053	2.26E-06	2.73E-05	SYNPO2	synaptopodin 2 [Source:HGNC Symbol;Acc:HGNC:17732]
ENSG00000083857	3392.59296	3226.85593	2413.74826	1999.23119	0.584838872	2.27E-06	2.74E-05	FAT1	FAT atypical cadherin 1 [Source:HGNC Symbol;Acc:HGNC:3595]
ENSG00000170323	34.4475641	65.5648926	2.10715692	1.06455335	4.978959662	2.30E-06	2.77E-05	FABP4	fatty acid binding protein 4 [Source:HGNC Symbol;Acc:HGNC:3559]
ENSG00000140451	252.943542	143.533954	427.752856	482.242668	-1.201646993	2.34E-06	2.82E-05	PIF1	PIF1 5'-to-3' DNA helicase [Source:HGNC Symbol;Acc:HGNC:26220]
ENSG00000169100	11778.1143	9894.98271	15316.9237	15605.2876	-0.512837396	2.36E-06	2.84E-05	SLC25A6	solute carrier family 25 member 6 [Source:HGNC Symbol;Acc:HGNC:10992]
ENSG00000141076	1603.28806	1640.00833	2307.33683	2427.18164	-0.545667439	2.39E-06	2.88E-05	UTP4	UTP4, small subunit processome component [Source:HGNC Symbol;Acc:HGNC:1983]
ENSG00000123080	1658.40416	1388.3809	2266.24727	2368.6312	-0.605748938	2.43E-06	2.92E-05	CDKN2C	cyclin dependent kinase inhibitor 2C [Source:HGNC Symbol;Acc:HGNC:1789]
ENSG00000090621	3142.60207	3082.43596	4241.70689	4545.64281	-0.497374693	2.44E-06	2.93E-05	PABPC4	poly(A) binding protein cytoplasmic 4 [Source:HGNC Symbol;Acc:HGNC:8557]
ENSG00000144583	48.2265898	50.5026875	1.05357846	4.2582134	4.219508222	2.44E-06	2.93E-05	4-Mar	membrane associated ring-CH-type finger 4 [Source:HGNC Symbol;Acc:HGNC:29269]
ENSG00000132563	192.906359	141.76193	57.9468154	48.9694541	1.644800513	2.44E-06	2.93E-05	REEP2	receptor accessory protein 2 [Source:HGNC Symbol;Acc:HGNC:17975]
ENSG00000139637	876.936561	711.467686	1305.38371	1270.01215	-0.69809079	2.48E-06	2.97E-05	C12orf10	chromosome 12 open reading frame 10 [Source:HGNC Symbol;Acc:HGNC:17590]
ENSG00000008735	506.871301	403.135488	209.662114	241.653611	1.010992944	2.48E-06	2.97E-05	MAPK8IP2	mitogen-activated protein kinase 8 interacting protein 2 [Source:HGNC Symbol;Acc:HGNC:6883]
ENSG00000115109	298.217484	331.368511	139.072357	150.102022	1.123005147	2.49E-06	2.98E-05	EPB41L5	erythrocyte membrane protein band 4.1 like 5 [Source:HGNC Symbol;Acc:HGNC:19819]
ENSG00000176890	914.336774	903.732303	1346.47327	1455.24443	-0.623931243	2.50E-06	3.00E-05	TYMS	thymidylate synthetase [Source:HGNC Symbol;Acc:HGNC:12441]
ENSG00000095321	432.070876	415.539657	220.197899	215.039777	0.961403532	2.52E-06	3.02E-05	CRAT	carnitine O-acetyltransferase [Source:HGNC Symbol;Acc:HGNC:2342]
ENSG00000215021	4387.63545	3975.53612	5731.46684	6103.08436	-0.501027934	2.52E-06	3.02E-05	PHB2	prohibitin 2 [Source:HGNC Symbol;Acc:HGNC:30306]
ENSG00000099381	1473.37153	1342.30827	2099.78188	2080.13725	-0.57032274	2.53E-06	3.02E-05	SETD1A	SET domain containing 1A [Source:HGNC Symbol;Acc:HGNC:29010]
ENSG00000008086	520.650327	618.436419	318.180696	298.074938	0.886889687	2.54E-06	3.03E-05	CDKL5	cyclin dependent kinase like 5 [Source:HGNC Symbol;Acc:HGNC:11411]
ENSG00000126461	2354.24495	1902.2679	3141.77097	3311.82547	-0.600870372	2.60E-06	3.10E-05	SCAF1	SR-related CTD associated factor 1 [Source:HGNC Symbol;Acc:HGNC:30403]
ENSG00000139620	684.030202	662.737022	1026.18542	1130.55566	-0.6794788	2.61E-06	3.11E-05	KANSL2	KAT8 regulatory NSL complex subunit 2 [Source:HGNC Symbol;Acc:HGNC:26024]
ENSG00000224259	124.995447	121.383652	257.073145	305.526812	-1.191403113	2.62E-06	3.12E-05	LINC01133	long intergenic non-protein coding RNA 1133 [Source:HGNC Symbol;Acc:HGNC:49447]
ENSG00000112139	284.438458	248.969389	99.0363755	124.552742	1.254040602	2.64E-06	3.14E-05	MDGA1	MAM domain containing glycosylphosphatidylinositol anchor 1 [Source:HGNC Symbol;Acc:HGNC:19267]
ENSG00000187840	1667.2621	1512.42259	2271.51516	2439.95628	-0.56758295	2.66E-06	3.16E-05	EIF4EBP1	eukaryotic translation initiation factor 4E binding protein 1 [Source:HGNC Symbol;Acc:HGNC:3288]
ENSG00000174231	13155.0326	11710.4214	17616.8855	16908.3009	-0.473576064	2.66E-06	3.16E-05	PRPF8	pre-mRNA processing factor 8 [Source:HGNC Symbol;Acc:HGNC:17340]
ENSG00000083937	1189.91729	1503.56247	851.291398	786.704926	0.718065371	2.67E-06	3.18E-05	CHMP2B	charged multivesicular body protein 2B [Source:HGNC Symbol;Acc:HGNC:24537]

ENSG00000146731	5151.38716	5395.81346	7104.27957	7498.7138	-0.469333352	2.68E-06	3.19E-05	CCT6A	chaperonin containing TCP1 subunit 6A [Source:HGNC Symbol;Acc:HGNC:1620]
ENSG00000131876	998.97936	1119.03323	1517.15299	1868.29113	-0.676209746	2.70E-06	3.20E-05	SNRPA1	small nuclear ribonucleoprotein polypeptide A' [Source:HGNC Symbol;Acc:HGNC:11152]
ENSG00000168672	256.880407	266.689631	116.947209	108.584442	1.215151136	2.70E-06	3.20E-05	FAM84B	family with sequence similarity 84 member B [Source:HGNC Symbol;Acc:HGNC:24166]
ENSG00000131015	557.066323	565.275695	296.055548	324.688772	0.854572407	2.71E-06	3.21E-05	ULBP2	UL16 binding protein 2 [Source:HGNC Symbol;Acc:HGNC:14894]
ENSG00000172009	2673.13098	2353.24804	3653.81011	3616.28773	-0.532744924	2.72E-06	3.21E-05	THOP1	thimet oligopeptidase 1 [Source:HGNC Symbol;Acc:HGNC:11793]
ENSG00000153044	351.365154	342.000656	708.004727	561.019616	-0.872281916	2.75E-06	3.25E-05	CENPH	centromere protein H [Source:HGNC Symbol;Acc:HGNC:17268]
ENSG00000113594	733.241008	908.162363	509.931976	371.529119	0.897347755	2.77E-06	3.27E-05	LIFR	LIF receptor alpha [Source:HGNC Symbol;Acc:HGNC:6597]
ENSG00000133835	3637.66277	3696.44232	2651.85699	2534.70153	0.499859574	2.79E-06	3.30E-05	HSD17B4	hydroxysteroid 17-beta dehydrogenase 4 [Source:HGNC Symbol;Acc:HGNC:5213]
ENSG00000101347	1224.36485	1301.55172	815.46973	810.1251	0.636035531	2.80E-06	3.30E-05	SAMHD1	SAM and HD domain containing deoxynucleoside triphosphate triphosphohydrolase 1 [Source:HGNC Symbol;Acc:HGNC:15925]
ENSG00000137154	9768.34497	9385.52577	12485.9584	14881.3913	-0.514836511	2.82E-06	3.33E-05	RPS6	ribosomal protein S6 [Source:HGNC Symbol;Acc:HGNC:10429]
ENSG00000113552	831.66262	889.55611	1326.45528	1330.69169	-0.626129585	2.82E-06	3.33E-05	GNPDA1	glucosamine-6-phosphate deaminase 1 [Source:HGNC Symbol;Acc:HGNC:4417]
ENSG00000185504	559.034755	479.332525	902.916742	840.997147	-0.748870865	2.88E-06	3.39E-05	FAAP100	FA core complex associated protein 100 [Source:HGNC Symbol;Acc:HGNC:26171]
ENSG00000090539	392.702231	347.316728	153.822455	195.877816	1.081149943	2.89E-06	3.40E-05	CHRD	chordin [Source:HGNC Symbol;Acc:HGNC:1949]
ENSG00000103740	78.7372895	84.1711459	10.5357846	18.097407	2.509432682	2.90E-06	3.41E-05	ACSBG1	acyl-CoA synthetase bubblegum family member 1 [Source:HGNC Symbol;Acc:HGNC:29567]
ENSG00000006327	3596.3257	2884.85527	4616.78082	5174.79384	-0.595578951	2.92E-06	3.43E-05	TNFRSF12A	TNF receptor superfamily member 12A [Source:HGNC Symbol;Acc:HGNC:18152]
ENSG00000167881	2125.90682	1888.97772	2799.35797	3164.91711	-0.571253703	2.94E-06	3.45E-05	SRP68	signal recognition particle 68 [Source:HGNC Symbol;Acc:HGNC:11302]
ENSG00000106089	359.238883	370.353042	136.9652	196.94237	1.128039386	2.94E-06	3.45E-05	STX1A	syntaxin 1A [Source:HGNC Symbol;Acc:HGNC:11433]
ENSG00000162433	1018.66368	1068.53055	1665.70755	1515.92397	-0.608034625	2.97E-06	3.48E-05	AK4	adenylate kinase 4 [Source:HGNC Symbol;Acc:HGNC:363]
ENSG00000073711	155.506147	176.3164	64.2682862	41.5175807	1.649292672	2.99E-06	3.50E-05	PPP2R3A	protein phosphatase 2 regulatory subunit B''alpha [Source:HGNC Symbol;Acc:HGNC:9307]
ENSG00000155363	1125.94324	972.841244	1544.54603	1730.96375	-0.642669448	2.99E-06	3.50E-05	MOV10	Mov10 RISC complex RNA helicase [Source:HGNC Symbol;Acc:HGNC:7200]
ENSG00000132906	735.20944	601.60219	369.80604	376.851886	0.839573187	3.01E-06	3.52E-05	CASP9	caspase 9 [Source:HGNC Symbol;Acc:HGNC:1511]
ENSG00000184428	666.314312	568.819744	950.327773	1113.5228	-0.741353243	3.02E-06	3.53E-05	TOP1MT	DNA topoisomerase I mitochondrial [Source:HGNC Symbol;Acc:HGNC:29787]
ENSG00000112186	231.290788	217.072955	451.98516	414.111253	-0.950382659	3.03E-06	3.55E-05	CAP2	cyclase associated actin cytoskeleton regulatory protein 2 [Source:HGNC Symbol;Acc:HGNC:20039]
ENSG00000130475	410.418121	351.746789	184.376231	189.490496	1.02694072	3.04E-06	3.55E-05	FCHO1	FCH domain only 1 [Source:HGNC Symbol;Acc:HGNC:29002]
ENSG00000105518	457.660495	341.114644	184.376231	194.813263	1.073825552	3.08E-06	3.60E-05	TMEM205	transmembrane protein 205 [Source:HGNC Symbol;Acc:HGNC:29631]
ENSG00000167522	6059.81864	5521.62717	7932.39224	8153.41411	-0.474084451	3.09E-06	3.60E-05	ANKRD11	ankyrin repeat domain 11 [Source:HGNC Symbol;Acc:HGNC:21316]
ENSG00000110628	417.307634	358.834885	207.554957	163.941216	1.062194704	3.11E-06	3.63E-05	SLC22A18	solute carrier family 22 member 18 [Source:HGNC Symbol;Acc:HGNC:10964]
ENSG00000173171	281.48581	235.679208	459.36021	532.276675	-0.940322097	3.14E-06	3.66E-05	MTX1	metaxin 1 [Source:HGNC Symbol;Acc:HGNC:7504]
ENSG00000111596	851.346942	841.711459	1308.54445	1291.30321	-0.618855468	3.21E-06	3.73E-05	CNOT2	CCR4-NOT transcription complex subunit 2 [Source:HGNC Symbol;Acc:HGNC:7878]
ENSG00000124733	984.216118	852.343603	1397.04504	1465.88996	-0.641073761	3.22E-06	3.74E-05	MEA1	male-enhanced antigen 1 [Source:HGNC Symbol;Acc:HGNC:6986]
ENSG00000266714	269.675216	221.503015	430.913591	540.793102	-0.985453135	3.23E-06	3.76E-05	MYO15B	myosin XVB [Source:HGNC Symbol;Acc:HGNC:14083]
ENSG00000090530	1145.62756	1117.26121	701.683256	738.800025	0.651555408	3.24E-06	3.77E-05	P3H2	prolyl 3-hydroxylase 2 [Source:HGNC Symbol;Acc:HGNC:19317]
ENSG00000196526	6247.80392	6086.90286	4358.6541	4566.93387	0.466681442	3.26E-06	3.78E-05	AFAP1	actin filament associated protein 1 [Source:HGNC Symbol;Acc:HGNC:24017]
ENSG00000132749	15.7474579	13.2901809	71.6433354	86.2288214	-2.445287391	3.32E-06	3.85E-05	TESMIN	testis expressed metallothionein like protein [Source:HGNC Symbol;Acc:HGNC:7446]
ENSG00000142534	9677.79709	8704.1825	12536.5301	12857.6754	-0.46628997	3.32E-06	3.85E-05	RPS11	ribosomal protein S11 [Source:HGNC Symbol;Acc:HGNC:10384]
ENSG00000147065	18065.2868	17333.94	23669.6937	23940.7403	-0.427579853	3.32E-06	3.85E-05	MSN	moesin [Source:HGNC Symbol;Acc:HGNC:7373]
ENSG00000136930	4453.57793	4414.9981	5998.02219	6222.31433	-0.46251922	3.34E-06	3.86E-05	PSMB7	proteasome subunit beta 7 [Source:HGNC Symbol;Acc:HGNC:9544]
ENSG00000114686	1951.70056	2056.434	2785.66145	2993.52402	-0.527771647	3.42E-06	3.95E-05	MRPL3	mitochondrial ribosomal protein L3 [Source:HGNC Symbol;Acc:HGNC:10379]
ENSG00000107731	1124.95902	958.665051	646.897176	630.215583	0.705688122	3.42E-06	3.95E-05	UNC5B	unc-5 netrin receptor B [Source:HGNC Symbol;Acc:HGNC:12568]
ENSG00000073150	254.911975	230.363136	463.574523	453.499727	-0.919000485	3.44E-06	3.97E-05	PANX2	pannexin 2 [Source:HGNC Symbol;Acc:HGNC:8600]
ENSG00000143499	471.439521	458.954248	772.273013	774.994839	-0.73397396	3.44E-06	3.97E-05	SMYD2	SET and MYND domain containing 2 [Source:HGNC Symbol;Acc:HGNC:20982]
ENSG00000110237	825.757323	807.156988	1234.79396	1284.91589	-0.625905284	3.44E-06	3.97E-05	ARHGEF17	Rho guanine nucleotide exchange factor 17 [Source:HGNC Symbol;Acc:HGNC:21726]
ENSG00000128335	700.761876	553.757539	1034.61405	1057.10148	-0.738612638	3.47E-06	4.00E-05	APOL2	apolipoprotein L2 [Source:HGNC Symbol;Acc:HGNC:619]
ENSG00000166025	970.437093	979.92934	627.932764	586.568896	0.683374815	3.48E-06	4.00E-05	AMOTL1	angiomotin like 1 [Source:HGNC Symbol;Acc:HGNC:17811]
ENSG00000112667	832.646836	599.830166	1203.1866	1244.46287	-0.774060744	3.49E-06	4.02E-05	DNPH1	2'-deoxynucleoside 5'-phosphate N-hydrolase 1 [Source:HGNC Symbol;Acc:HGNC:21218]
ENSG00000231991	1220.42799	1013.5978	1655.17176	1841.6773	-0.647004505	3.50E-06	4.03E-05	ANXA2P2	annexin A2 pseudogene 2 [Source:HGNC Symbol;Acc:HGNC:539]
ENSG00000166987	1073.77978	979.043328	1607.76073	1516.98852	-0.606531544	3.53E-06	4.06E-05	MBD6	methyl-CpG binding domain protein 6 [Source:HGNC Symbol;Acc:HGNC:20445]
ENSG00000168280	106.295341	113.409544	33.7145108	15.9683003	2.144041565	3.55E-06	4.08E-05	KIF5C	kinesin family member 5C [Source:HGNC Symbol;Acc:HGNC:6325]
ENSG00000169783	72.8319927	43.414591	5.26789231	6.3873201	3.316640643	3.56E-06	4.08E-05	LINGO1	leucine rich repeat and Ig domain containing 1 [Source:HGNC Symbol;Acc:HGNC:21205]
ENSG00000158402	308.059645	288.839932	526.789231	550.374082	-0.852110808	3.58E-06	4.11E-05	CDC25C	cell division cycle 25C [Source:HGNC Symbol;Acc:HGNC:1727]
ENSG00000100836	1520.6139	1257.25112	1957.54878	2586.86464	-0.71049888	3.60E-06	4.13E-05	PABPN1	poly(A) binding protein nuclear 1 [Source:HGNC Symbol;Acc:HGNC:8565]
ENSG00000137818	24639.8505	20089.4375	30747.6338	34081.6755	-0.535474713	3.60E-06	4.13E-05	RPLP1	ribosomal protein lateral stalk subunit P1 [Source:HGNC Symbol;Acc:HGNC:10372]
ENSG00000051128	569.861132	463.384308	813.362573	980.453636	-0.796762003	3.69E-06	4.22E-05	HOMER3	homer scaffold protein 3 [Source:HGNC Symbol;Acc:HGNC:17514]

ENSG00000158201	1217.47534	1239.53087	814.416151	771.801179	0.631341703	3.69E-06	4.22E-05	ABHD3	abhydrolase domain containing 3 [Source:HGNC Symbol;Acc:HGNC:18718]
ENSG00000171223	11991.6892	10426.5899	7655.30111	8227.93284	0.497097268	3.70E-06	4.23E-05	JUNB	JunB proto-oncogene, AP-1 transcription factor subunit [Source:HGNC Symbol;Acc:HGNC:6205]
ENSG00000002834	5287.20899	4817.24758	6974.68942	7052.66595	-0.473379249	3.75E-06	4.28E-05	LASP1	LIM and SH3 protein 1 [Source:HGNC Symbol;Acc:HGNC:6513]
ENSG00000173744	1517.66125	1656.84256	1092.56087	976.195422	0.617952294	3.76E-06	4.30E-05	AGFG1	ArfGAP with FG repeats 1 [Source:HGNC Symbol;Acc:HGNC:5175]
ENSG00000001461	1198.77523	1179.28205	743.826394	785.640372	0.63672928	3.80E-06	4.34E-05	NIPAL3	NIPA like domain containing 3 [Source:HGNC Symbol;Acc:HGNC:25233]
ENSG00000196937	670.251176	665.395058	304.484176	419.43402	0.883805328	3.90E-06	4.45E-05	FAM3C	family with sequence similarity 3 member C [Source:HGNC Symbol;Acc:HGNC:18664]
ENSG00000171824	1137.75383	1229.78474	1706.79711	1850.19372	-0.586950331	3.91E-06	4.45E-05	EXOSC10	exosome component 10 [Source:HGNC Symbol;Acc:HGNC:9138]
ENSG00000115641	1287.35468	1258.13713	1875.36966	1867.22658	-0.556186818	3.95E-06	4.50E-05	FHL2	four and a half LIM domains 2 [Source:HGNC Symbol;Acc:HGNC:3703]
ENSG00000117600	63.9740477	74.4250132	11.5893631	9.58098015	2.709006781	4.02E-06	4.57E-05	PLPPR4	phospholipid phosphatase related 4 [Source:HGNC Symbol;Acc:HGNC:23496]
ENSG00000166228	278.533161	270.233679	461.467366	570.600596	-0.911383965	4.03E-06	4.59E-05	PCBD1	pterin-4 alpha-carbinolamine dehydratase 1 [Source:HGNC Symbol;Acc:HGNC:8646]
ENSG00000095739	624.977235	615.778383	348.734471	365.141799	0.797444604	4.05E-06	4.60E-05	BAMBI	BMP and activin membrane bound inhibitor [Source:HGNC Symbol;Acc:HGNC:30251]
ENSG00000198755	8394.37927	8454.32709	11047.8238	11980.4834	-0.450758028	4.06E-06	4.61E-05	RPL10A	ribosomal protein L10a [Source:HGNC Symbol;Acc:HGNC:10299]
ENSG00000177697	4081.54424	3308.36904	5351.12501	5503.74082	-0.55499632	4.07E-06	4.62E-05	CD151	CD151 molecule (Raph blood group) [Source:HGNC Symbol;Acc:HGNC:1630]
ENSG00000077312	1869.02641	1717.09138	2471.69507	2850.87387	-0.569896715	4.08E-06	4.63E-05	SNRPA	small nuclear ribonucleoprotein polypeptide A [Source:HGNC Symbol;Acc:HGNC:11151]
ENSG00000187678	165.348308	135.559845	52.6789231	42.582134	1.658254034	4.11E-06	4.66E-05	SPRY4	sprouty RTK signaling antagonist 4 [Source:HGNC Symbol;Acc:HGNC:15533]
ENSG00000005007	3235.11838	2722.71507	4330.20748	4321.02205	-0.538427544	4.13E-06	4.68E-05	UPF1	UPF1, RNA helicase and ATPase [Source:HGNC Symbol;Acc:HGNC:9962]
ENSG00000198589	1586.55638	1721.52144	1159.98989	949.581588	0.649153612	4.17E-06	4.72E-05	LRBA	LPS responsive beige-like anchor protein [Source:HGNC Symbol;Acc:HGNC:1742]
ENSG00000092201	5310.83017	5846.7936	7543.62179	7975.6337	-0.475905814	4.22E-06	4.77E-05	SUPT16H	SPT16 homolog, facilitates chromatin remodeling subunit [Source:HGNC Symbol;Acc:HGNC:11465]
ENSG00000257732	162.39566	178.974436	52.6789231	66.0023077	1.524930534	4.22E-06	4.78E-05	AC089983.1	novel transcript, antisense to TXNRD1
ENSG00000025039	158.458795	201.124738	56.893237	69.1959678	1.513103429	4.28E-06	4.83E-05	RRAGD	Ras related GTP binding D [Source:HGNC Symbol;Acc:HGNC:19903]
ENSG00000188636	1324.7549	1346.73833	2120.85344	1867.22658	-0.578024866	4.28E-06	4.83E-05	RTL6	retrotransposon Gag like 6 [Source:HGNC Symbol;Acc:HGNC:13343]
ENSG00000162909	4081.54424	3928.57748	5559.73355	5480.32065	-0.462922403	4.29E-06	4.83E-05	CAPN2	calpain 2 [Source:HGNC Symbol;Acc:HGNC:1479]
ENSG00000198176	3017.60662	2954.85023	4047.84845	4321.02205	-0.486742942	4.30E-06	4.85E-05	TFDP1	transcription factor Dp-1 [Source:HGNC Symbol;Acc:HGNC:11749]
ENSG00000240342	9973.06193	7716.27905	12582.8876	13767.8685	-0.575096092	4.33E-06	4.88E-05	RPS2P5	ribosomal protein S2 pseudogene 5 [Source:HGNC Symbol;Acc:HGNC:31386]
ENSG00000133083	106.295341	126.699725	33.7145108	28.7429405	1.899908949	4.38E-06	4.93E-05	DCLK1	doublecortin like kinase 1 [Source:HGNC Symbol;Acc:HGNC:2700]
ENSG00000107581	5101.19214	5033.43452	7162.22639	6751.39735	-0.457231953	4.40E-06	4.95E-05	EIF3A	eukaryotic translation initiation factor 3 subunit A [Source:HGNC Symbol;Acc:HGNC:3271]
ENSG00000123104	1433.01867	1588.61963	1037.77479	914.451328	0.630428815	4.46E-06	5.02E-05	ITPR2	inositol 1,4,5-trisphosphate receptor type 2 [Source:HGNC Symbol;Acc:HGNC:6181]
ENSG00000147119	212.590682	197.58069	77.9648062	83.0351613	1.348893898	4.47E-06	5.02E-05	CHST7	carbohydrate sulfotransferase 7 [Source:HGNC Symbol;Acc:HGNC:13817]
ENSG00000113272	187.001062	210.870871	384.556139	399.207506	-0.977160322	4.49E-06	5.04E-05	THG1L	tRNA-histidine guanylyltransferase 1 like [Source:HGNC Symbol;Acc:HGNC:26053]
ENSG00000102974	1375.93413	1369.77465	1978.62035	2024.78047	-0.544057627	4.51E-06	5.06E-05	CTCF	CCCTC-binding factor [Source:HGNC Symbol;Acc:HGNC:13723]
ENSG00000197756	8540.04326	7679.95255	10790.7506	11861.2534	-0.481946144	4.55E-06	5.10E-05	RPL37A	ribosomal protein L37a [Source:HGNC Symbol;Acc:HGNC:10348]
ENSG00000007038	1621.00395	1282.05945	2264.14012	2214.27097	-0.626045706	4.55E-06	5.10E-05	PRSS21	serine protease 21 [Source:HGNC Symbol;Acc:HGNC:9485]
ENSG00000183828	180.11155	155.938123	337.145108	353.431712	-1.040272169	4.58E-06	5.13E-05	NUDT14	nudix hydrolase 14 [Source:HGNC Symbol;Acc:HGNC:20141]
ENSG00000196642	3551.05175	3032.81929	4547.24464	5024.69181	-0.54011481	4.64E-06	5.20E-05	RABL6	RAB, member RAS oncogene family like 6 [Source:HGNC Symbol;Acc:HGNC:24703]
ENSG00000091947	286.40689	264.917606	473.05673	545.051315	-0.885391814	4.68E-06	5.23E-05	TMEM101	transmembrane protein 101 [Source:HGNC Symbol;Acc:HGNC:28653]
ENSG00000160685	2444.79284	2028.96762	3212.36073	3414.02259	-0.567120432	4.71E-06	5.26E-05	ZBTB7B	zinc finger and BTB domain containing 7B [Source:HGNC Symbol;Acc:HGNC:18668]
ENSG00000149260	598.4034	532.493249	307.644911	323.624218	0.84070136	4.71E-06	5.26E-05	CAPN5	calpain 5 [Source:HGNC Symbol;Acc:HGNC:1482]
ENSG00000128050	5069.69722	5855.65372	7548.88968	7864.92015	-0.496375699	4.74E-06	5.29E-05	PAICS	phosphoribosylaminoimidazole carboxylase and phosphoribosylaminoimidazolesuccinocarboxamide synthase [Source:HGNC Symbol;Acc:HGNC:8587]
ENSG00000175866	633.83518	629.068564	968.238607	1035.81041	-0.666187206	4.78E-06	5.33E-05	BAIAP2	BAI1 associated protein 2 [Source:HGNC Symbol;Acc:HGNC:947]
ENSG00000143418	1567.85628	1436.22555	2207.24688	2179.14071	-0.546411027	4.80E-06	5.35E-05	CERS2	ceramide synthase 2 [Source:HGNC Symbol;Acc:HGNC:14076]
ENSG00000089006	1303.10214	1464.57794	1989.15614	2131.23581	-0.573696075	4.80E-06	5.35E-05	SNX5	sorting nexin 5 [Source:HGNC Symbol;Acc:HGNC:14969]
ENSG00000172915	574.782213	629.068564	370.859619	261.880124	0.928044767	4.81E-06	5.35E-05	NBEA	neurobeachin [Source:HGNC Symbol;Acc:HGNC:7648]
ENSG00000112972	2524.51434	2676.64244	1864.83388	1735.22196	0.530914361	4.81E-06	5.35E-05	HMGCS1	3-hydroxy-3-methylglutaryl-CoA synthase 1 [Source:HGNC Symbol;Acc:HGNC:5007]
ENSG00000120885	28925.1275	25692.5778	18894.8761	20554.3961	0.469342874	4.82E-06	5.36E-05	CLU	clusterin [Source:HGNC Symbol;Acc:HGNC:2095]
ENSG00000206053	2500.89316	2791.82401	3686.47104	3864.32866	-0.512370342	4.82E-06	5.36E-05	JPT2	Jupiter microtubule associated homolog 2 [Source:HGNC Symbol;Acc:HGNC:14137]
ENSG00000124570	3239.05524	3426.20864	4506.15508	4821.36212	-0.484710557	4.84E-06	5.37E-05	SERPINB6	serpin family B member 6 [Source:HGNC Symbol;Acc:HGNC:8950]
ENSG00000134574	1038.348	924.996593	1446.56323	1559.57066	-0.615044767	4.92E-06	5.45E-05	DDB2	damage specific DNA binding protein 2 [Source:HGNC Symbol;Acc:HGNC:2718]
ENSG00000166446	1091.49568	1018.91387	694.308207	633.409243	0.668290815	4.94E-06	5.48E-05	CDYL2	chromodomain Y like 2 [Source:HGNC Symbol;Acc:HGNC:23030]
ENSG00000154217	449.786766	401.363464	201.233486	235.26629	0.963084531	4.96E-06	5.49E-05	PITPNC1	phosphatidylinositol transfer protein cytoplasmic 1 [Source:HGNC Symbol;Acc:HGNC:21045]
ENSG00000204628	20076.0404	18343.9937	25291.151	27242.9848	-0.45142942	4.96E-06	5.49E-05	RACK1	receptor for activated C kinase 1 [Source:HGNC Symbol;Acc:HGNC:4399]
ENSG00000101871	1836.54728	1735.69763	1241.11543	1164.62137	0.570171817	5.01E-06	5.54E-05	MID1	midline 1 [Source:HGNC Symbol;Acc:HGNC:7095]

ENSG00000159176	5345.27774	4567.39218	3207.09284	3597.12577	0.542696994	5.04E-06	5.58E-05	CSRP1	cysteine and glycine rich protein 1 [Source:HGNC Symbol;Acc:HGNC:2469]
ENSG00000170837	95.4689635	91.2592424	214.930006	234.201737	-1.266585563	5.16E-06	5.71E-05	GPR27	G protein-coupled receptor 27 [Source:HGNC Symbol;Acc:HGNC:4482]
ENSG00000187017	151.569282	137.33187	50.5717662	41.5175807	1.648789087	5.19E-06	5.73E-05	ESPN	espin [Source:HGNC Symbol;Acc:HGNC:13281]
ENSG00000051825	558.050539	641.472733	1032.50689	926.161415	-0.706755582	5.25E-06	5.79E-05	MPHOSPH9	M-phase phosphoprotein 9 [Source:HGNC Symbol;Acc:HGNC:7215]
ENSG00000174444	21133.0885	20825.7135	26857.8222	31874.8564	-0.485190331	5.25E-06	5.79E-05	RPL4	ribosomal protein L4 [Source:HGNC Symbol;Acc:HGNC:10353]
ENSG00000121067	768.672788	750.452216	1172.63283	1168.87958	-0.624323106	5.25E-06	5.79E-05	SPOP	speckle type BTB/POZ protein [Source:HGNC Symbol;Acc:HGNC:11254]
ENSG00000198794	1163.34345	1017.14185	638.468548	715.379851	0.687230089	5.26E-06	5.79E-05	SCAMP5	secretory carrier membrane protein 5 [Source:HGNC Symbol;Acc:HGNC:30386]
ENSG00000155367	73.8162089	55.8187599	5.26789231	11.7100869	2.933054335	5.28E-06	5.81E-05	PPM1J	protein phosphatase, Mg2+/Mn2+ dependent 1J [Source:HGNC Symbol;Acc:HGNC:20785]
ENSG00000112208	596.434968	561.731647	892.380958	974.066315	-0.688743928	5.28E-06	5.81E-05	BAG2	BCL2 associated athanogene 2 [Source:HGNC Symbol;Acc:HGNC:938]
ENSG00000225663	82.6741539	83.2851338	194.912016	220.362544	-1.323102109	5.34E-06	5.87E-05	MCRIP1	MAPK regulated corepressor interacting protein 1 [Source:HGNC Symbol;Acc:HGNC:28007]
ENSG00000103657	2723.326	2752.83948	2001.79908	1784.19142	0.532480776	5.34E-06	5.87E-05	HERC1	HECT and RLD domain containing E3 ubiquitin protein ligase family member 1 [Source:HGNC Symbol;Acc:HGNC:4867]
ENSG00000176974	1894.61603	1777.3402	2525.42757	2809.35629	-0.539038402	5.47E-06	6.01E-05	SHMT1	serine hydroxymethyltransferase 1 [Source:HGNC Symbol;Acc:HGNC:10850]
ENSG00000132196	496.044924	498.824791	256.019566	287.429405	0.872476694	5.48E-06	6.01E-05	HSD17B7	hydroxysteroid 17-beta dehydrogenase 7 [Source:HGNC Symbol;Acc:HGNC:5215]
ENSG00000204231	587.577023	460.726272	867.095074	909.128561	-0.761928046	5.48E-06	6.01E-05	RXRB	retinoid X receptor beta [Source:HGNC Symbol;Acc:HGNC:10478]
ENSG00000078401	116.137502	134.673833	29.5001969	41.5175807	1.821407923	5.56E-06	6.10E-05	EDN1	endothelin 1 [Source:HGNC Symbol;Acc:HGNC:3176]
ENSG00000169902	1067.87449	1156.24574	729.076296	689.830571	0.648691358	5.57E-06	6.10E-05	TPST1	tyrosylprotein sulfotransferase 1 [Source:HGNC Symbol;Acc:HGNC:12020]
ENSG00000122952	2211.53362	2222.11825	3130.18161	3101.04391	-0.491009166	5.70E-06	6.24E-05	ZWINT	ZW10 interacting kinetochore protein [Source:HGNC Symbol;Acc:HGNC:13195]
ENSG00000040633	894.652451	857.659676	1321.18739	1336.01445	-0.600843427	5.73E-06	6.27E-05	PHF23	PHD finger protein 23 [Source:HGNC Symbol;Acc:HGNC:28428]
ENSG00000144647	1127.91167	1014.48381	602.64688	716.444405	0.699451529	5.77E-06	6.31E-05	POMGNT2	protein O-linked mannose N-acetylglucosaminyltransferase 2 (beta 1,4-) [Source:HGNC Symbol;Acc:HGNC:25902]
ENSG00000057294	756.862195	684.001312	424.59212	431.144107	0.751311275	5.82E-06	6.35E-05	PKP2	plakophilin 2 [Source:HGNC Symbol;Acc:HGNC:9024]
ENSG00000175445	243.101381	254.285462	111.679317	105.390782	1.196359026	5.89E-06	6.43E-05	LPL	lipoprotein lipase [Source:HGNC Symbol;Acc:HGNC:6677]
ENSG00000008277	518.681894	552.871527	325.555745	232.07263	0.942322931	5.91E-06	6.45E-05	ADAM22	ADAM metallopeptidase domain 22 [Source:HGNC Symbol;Acc:HGNC:201]
ENSG00000166250	362.191531	411.995609	767.005121	611.053623	-0.831258412	5.95E-06	6.49E-05	CLMP	CXADR like membrane protein [Source:HGNC Symbol;Acc:HGNC:24039]
ENSG00000119986	2133.78054	1751.64585	2799.35797	3004.16955	-0.579297783	5.95E-06	6.49E-05	AVPI1	arginine vasopressin induced 1 [Source:HGNC Symbol;Acc:HGNC:30898]
ENSG00000006712	1171.21718	1013.5978	1640.42167	1667.09055	-0.598780477	6.00E-06	6.53E-05	PAF1	PAF1 homolog, Paf1/RNA polymerase II complex component [Source:HGNC Symbol;Acc:HGNC:25459]
ENSG00000161638	12497.5763	11318.8041	16273.5729	16129.0478	-0.444220408	6.00E-06	6.53E-05	ITGA5	integrin subunit alpha 5 [Source:HGNC Symbol;Acc:HGNC:6141]
ENSG00000137767	164.364092	166.570268	35.8216677	68.1314144	1.671310838	6.02E-06	6.54E-05	SQOR	sulfide quinone oxidoreductase [Source:HGNC Symbol;Acc:HGNC:20390]
ENSG00000111012	328.728183	275.549751	142.233092	137.327382	1.111247159	6.02E-06	6.54E-05	CYP27B1	cytochrome P450 family 27 subfamily B member 1 [Source:HGNC Symbol;Acc:HGNC:2606]
ENSG00000103034	287.391107	248.083377	119.054366	119.229975	1.167471001	6.03E-06	6.55E-05	NDRG4	NDRG family member 4 [Source:HGNC Symbol;Acc:HGNC:14466]
ENSG00000182795	98.4216118	108.093472	30.5537754	19.1619603	2.054232311	6.04E-06	6.56E-05	C1orf116	chromosome 1 open reading frame 116 [Source:HGNC Symbol;Acc:HGNC:28667]
ENSG00000196781	884.81029	914.364448	594.218253	498.210968	0.719804808	6.04E-06	6.56E-05	TLE1	transducin like enhancer of split 1 [Source:HGNC Symbol;Acc:HGNC:11837]
ENSG00000152990	1673.1674	1635.57827	1107.31096	1131.62021	0.563417235	6.13E-06	6.64E-05	ADGRA3	adhesion G protein-coupled receptor A3 [Source:HGNC Symbol;Acc:HGNC:13839]
ENSG00000140350	3767.5793	3779.72746	4924.42573	6102.0198	-0.546907873	6.13E-06	6.64E-05	ANP32A	acidic nuclear phosphoprotein 32 family member A [Source:HGNC Symbol;Acc:HGNC:13233]
ENSG00000108797	1453.68721	1215.60855	2077.65673	1959.84272	-0.597575234	6.15E-06	6.66E-05	CNTNAP1	contactin associated protein 1 [Source:HGNC Symbol;Acc:HGNC:8011]
ENSG00000132478	419.276066	393.389355	697.468942	671.733164	-0.753023988	6.19E-06	6.70E-05	UNK	unkempt family zinc finger [Source:HGNC Symbol;Acc:HGNC:29369]
ENSG00000122870	410.418121	503.254851	244.430203	233.137184	0.936746769	6.22E-06	6.72E-05	BICC1	BicC family RNA binding protein 1 [Source:HGNC Symbol;Acc:HGNC:19351]
ENSG00000182870	217.511762	213.528907	396.145502	425.82134	-0.93136337	6.23E-06	6.73E-05	GALNT9	polypeptide N-acetylgalactosaminyltransferase 9 [Source:HGNC Symbol;Acc:HGNC:4131]
ENSG00000100982	577.734861	573.249804	889.220222	949.581588	-0.675914231	6.28E-06	6.78E-05	PCIF1	PDX1 C-terminal inhibiting factor 1 [Source:HGNC Symbol;Acc:HGNC:16200]
ENSG00000170899	54.1318865	78.8550735	7.37504924	11.7100869	2.803015259	6.34E-06	6.84E-05	GSTA4	glutathione S-transferase alpha 4 [Source:HGNC Symbol;Acc:HGNC:4629]
ENSG00000175467	1789.3049	1475.21008	2317.87262	2659.25427	-0.608893357	6.35E-06	6.85E-05	SART1	SART1, U4/U6.U5 tri-snRNP-associated protein 1 [Source:HGNC Symbol;Acc:HGNC:10538]
ENSG00000008441	583.640158	474.016453	970.345764	826.0934	-0.765306447	6.37E-06	6.86E-05	NFIX	nuclear factor I X [Source:HGNC Symbol;Acc:HGNC:7788]
ENSG00000106305	398.607528	457.182224	690.093893	766.478412	-0.766410522	6.37E-06	6.86E-05	AIMP2	aminoacyl tRNA synthetase complex interacting multifunctional protein 2 [Source:HGNC Symbol;Acc:HGNC:20609]
ENSG00000146066	550.17681	497.052767	792.291004	967.678995	-0.749411241	6.37E-06	6.86E-05	HIGD2A	HIG1 hypoxia inducible domain family member 2A [Source:HGNC Symbol;Acc:HGNC:28311]
ENSG00000128595	9422.88512	11237.291	7447.74615	7131.44289	0.503039421	6.38E-06	6.86E-05	CALU	calumenin [Source:HGNC Symbol;Acc:HGNC:1458]
ENSG00000143442	1284.40203	1200.54634	1785.81549	1873.6139	-0.558646796	6.44E-06	6.93E-05	POGZ	pogo transposable element derived with ZNF domain [Source:HGNC Symbol;Acc:HGNC:18801]
ENSG00000166451	558.050539	645.016781	1011.43532	935.742395	-0.693963613	6.55E-06	7.04E-05	CENPN	centromere protein N [Source:HGNC Symbol;Acc:HGNC:30873]
ENSG00000144713	14953.1955	14958.5416	19216.2176	21588.0774	-0.448004452	6.64E-06	7.13E-05	RPL32	ribosomal protein L32 [Source:HGNC Symbol;Acc:HGNC:10336]
ENSG00000170364	190.937927	191.378605	350.841628	403.46572	-0.980299672	6.68E-06	7.16E-05	SETMAR	SET domain and mariner transposase fusion gene [Source:HGNC Symbol;Acc:HGNC:10762]
ENSG00000177359	43.3055092	47.8446513	4.21431385	1.06455335	4.107283505	6.69E-06	7.17E-05	AC024940.1	alpha-2-macroglobulin like 1 pseudogene [Source:NCBI gene;Acc:144203]

ENSG00000039560	1863.12111	2020.99351	1273.77636	1362.62829	0.559245092	6.69E-06	7.17E-05	RAI14	retinoic acid induced 14 [Source:HGNC Symbol;Acc:HGNC:14873]
ENSG00000099800	1281.44939	1054.35435	1731.02941	1856.58104	-0.619728201	6.69E-06	7.17E-05	TIMM13	translocase of inner mitochondrial membrane 13 [Source:HGNC Symbol;Acc:HGNC:11816]
ENSG00000177469	9884.48248	8775.06346	12695.6205	12925.8068	-0.457526759	6.71E-06	7.18E-05	CAVIN1	caveolae associated protein 1 [Source:HGNC Symbol;Acc:HGNC:9688]
ENSG00000099622	1308.02322	1090.68085	1695.20775	2112.07385	-0.666964588	6.80E-06	7.27E-05	CIRBP	cold inducible RNA binding protein [Source:HGNC Symbol;Acc:HGNC:1982]
ENSG00000135372	1374.94992	1299.77969	1909.08417	1993.90843	-0.54538359	6.86E-06	7.34E-05	NAT10	N-acetyltransferase 10 [Source:HGNC Symbol;Acc:HGNC:29830]
ENSG00000143850	1025.5532	898.416231	1459.20617	1468.01907	-0.605996202	6.90E-06	7.37E-05	PLEKHA6	pleckstrin homology domain containing A6 [Source:HGNC Symbol;Acc:HGNC:17053]
ENSG00000079785	2231.21794	2276.16499	3134.39593	3180.88541	-0.486498419	6.93E-06	7.39E-05	DDX1	DEAD-box helicase 1 [Source:HGNC Symbol;Acc:HGNC:2734]
ENSG00000137878	157.474579	220.617003	69.5361785	68.1314144	1.458901825	6.96E-06	7.43E-05	GCOM1	GRINL1A complex locus 1 [Source:HGNC Symbol;Acc:HGNC:26424]
ENSG00000116096	933.03688	855.00164	1318.02666	1406.27498	-0.607874559	6.99E-06	7.46E-05	SPR	sepiapterin reductase [Source:HGNC Symbol;Acc:HGNC:11257]
ENSG00000198242	5411.22022	5366.57506	6971.52869	8383.35763	-0.510629244	7.02E-06	7.48E-05	RPL23A	ribosomal protein L23a [Source:HGNC Symbol;Acc:HGNC:10317]
ENSG00000127554	567.8927	424.399778	885.005908	829.28706	-0.790157751	7.07E-06	7.53E-05	GFER	growth factor, augmenter of liver regeneration [Source:HGNC Symbol;Acc:HGNC:4236]
ENSG00000069399	1666.27789	1505.33449	2216.72908	2430.3753	-0.551404073	7.08E-06	7.53E-05	BCL3	B cell CLL/lymphoma 3 [Source:HGNC Symbol;Acc:HGNC:998]
ENSG00000183570	75.7846411	62.0208443	9.48220616	12.7746402	2.629993604	7.09E-06	7.54E-05	PCBP3	poly(rC) binding protein 3 [Source:HGNC Symbol;Acc:HGNC:8651]
ENSG00000170293	190.937927	209.984859	85.3398554	72.3896278	1.346130556	7.19E-06	7.64E-05	CMTM8	CKLF like MARVEL transmembrane domain containing 8 [Source:HGNC Symbol;Acc:HGNC:19179]
ENSG00000100321	696.825012	596.286118	366.645305	378.980993	0.793749057	7.19E-06	7.64E-05	SYNGR1	synaptogyrin 1 [Source:HGNC Symbol;Acc:HGNC:11498]
ENSG00000144579	1175.15405	940.058798	1557.18897	1788.44963	-0.662150813	7.22E-06	7.66E-05	CTDSP1	CTD small phosphatase 1 [Source:HGNC Symbol;Acc:HGNC:21614]
ENSG00000177192	979.295038	870.063845	1373.86631	1445.66345	-0.608917143	7.24E-06	7.68E-05	PUS1	pseudouridylate synthase 1 [Source:HGNC Symbol;Acc:HGNC:15508]
ENSG00000101236	2832.57399	2891.05736	2085.03178	1953.4554	0.503137454	7.30E-06	7.73E-05	RNF24	ring finger protein 24 [Source:HGNC Symbol;Acc:HGNC:13779]
ENSG00000175806	48.2265898	60.2488202	144.340249	172.457643	-1.543788025	7.32E-06	7.75E-05	MSRA	methionine sulfoxide reductase A [Source:HGNC Symbol;Acc:HGNC:7377]
ENSG00000151014	297.233268	318.07833	153.822455	139.456489	1.06926252	7.35E-06	7.78E-05	NOCT	nocturnin [Source:HGNC Symbol;Acc:HGNC:14254]
ENSG00000176658	2113.11201	2145.92121	1476.06343	1486.11648	0.523909226	7.38E-06	7.81E-05	MYO1D	myosin ID [Source:HGNC Symbol;Acc:HGNC:7598]
ENSG00000136933	795.246624	676.027203	1119.95391	1208.26805	-0.662868119	7.43E-06	7.85E-05	RABEPK	Rab9 effector protein with kelch motifs [Source:HGNC Symbol;Acc:HGNC:16896]
ENSG00000036672	119.09015	106.321447	23.1787262	36.1948139	1.924765184	7.48E-06	7.91E-05	USP2	ubiquitin specific peptidase 2 [Source:HGNC Symbol;Acc:HGNC:12618]
ENSG00000272391	1314.91273	1279.40142	1858.51241	1914.06692	-0.540294841	7.55E-06	7.98E-05	POM121C	POM121 transmembrane nucleoporin C [Source:HGNC Symbol;Acc:HGNC:34005]
ENSG00000170653	352.34937	339.34262	586.843204	602.537196	-0.782250409	7.75E-06	8.18E-05	ATF7	activating transcription factor 7 [Source:HGNC Symbol;Acc:HGNC:792]
ENSG00000106034	116.137502	90.3732303	27.39304	24.4847271	1.991795389	7.78E-06	8.20E-05	CPED1	cadherin like and PC-esterase domain containing 1 [Source:HGNC Symbol;Acc:HGNC:26159]
ENSG00000099385	558.050539	485.53461	790.183847	968.743549	-0.753755369	7.88E-06	8.31E-05	BCL7C	BCL tumor suppressor 7C [Source:HGNC Symbol;Acc:HGNC:1006]
ENSG00000171206	574.782213	473.130441	817.576887	941.065162	-0.747850224	7.89E-06	8.31E-05	TRIM8	tripartite motif containing 8 [Source:HGNC Symbol;Acc:HGNC:15579]
ENSG00000100296	779.499166	787.664723	1232.6868	1163.55681	-0.612588806	7.89E-06	8.31E-05	THOC5	THO complex 5 [Source:HGNC Symbol;Acc:HGNC:19074]
ENSG00000080986	423.212931	598.944154	916.613262	877.191961	-0.80994242	7.90E-06	8.32E-05	NDC80	NDC80, kinetochore complex component [Source:HGNC Symbol;Acc:HGNC:16909]
ENSG00000232022	73.8162089	72.6529891	11.5893631	14.9037469	2.467253783	7.95E-06	8.36E-05	FAAHP1	fatty acid amide hydrolase pseudogene 1 [Source:HGNC Symbol;Acc:HGNC:50679]
ENSG00000184661	726.351495	785.892699	1262.187	1106.07093	-0.64681985	7.96E-06	8.36E-05	CDCA2	cell division cycle associated 2 [Source:HGNC Symbol;Acc:HGNC:14623]
ENSG00000134996	801.15192	764.628409	477.271043	483.307221	0.704730917	8.07E-06	8.48E-05	OSTF1	osteoclast stimulating factor 1 [Source:HGNC Symbol;Acc:HGNC:8510]
ENSG00000011376	1139.72226	978.157316	1537.17098	1701.15625	-0.613156687	8.08E-06	8.48E-05	LARS2	leucyl-tRNA synthetase 2, mitochondrial [Source:HGNC Symbol;Acc:HGNC:17095]
ENSG00000198826	855.283807	832.851338	1354.9019	1224.23635	-0.61161541	8.13E-06	8.52E-05	ARHGAP11A	Rho GTPase activating protein 11A [Source:HGNC Symbol;Acc:HGNC:15783]
ENSG00000133138	233.25922	288.839932	108.518582	120.294529	1.191168202	8.17E-06	8.57E-05	TBC1D8B	TBC1 domain family member 8B [Source:HGNC Symbol;Acc:HGNC:24715]
ENSG00000143669	627.929883	703.493577	410.8956	365.141799	0.779123897	8.21E-06	8.60E-05	LYST	lysosomal trafficking regulator [Source:HGNC Symbol;Acc:HGNC:1968]
ENSG00000138760	3672.11034	4139.44835	2867.84057	2579.41277	0.520225973	8.21E-06	8.60E-05	SCARB2	scavenger receptor class B member 2 [Source:HGNC Symbol;Acc:HGNC:1665]
ENSG00000211450	458.644711	413.767633	784.915954	685.572358	-0.753861257	8.22E-06	8.60E-05	SELENOH	selenoprotein H [Source:HGNC Symbol;Acc:HGNC:18251]
ENSG00000133884	839.536349	795.638831	1172.63283	1376.46748	-0.640722816	8.35E-06	8.73E-05	DPF2	double PHD fingers 2 [Source:HGNC Symbol;Acc:HGNC:9964]
ENSG00000064309	759.814843	778.804602	503.610505	399.207506	0.76907556	8.38E-06	8.75E-05	CDON	cell adhesion associated, oncogene regulated [Source:HGNC Symbol;Acc:HGNC:17104]
ENSG00000126756	711.588253	743.36412	1060.95351	1221.04269	-0.649071067	8.38E-06	8.76E-05	UXT	ubiquitously expressed prefoldin like chaperone [Source:HGNC Symbol;Acc:HGNC:12641]
ENSG00000174373	734.225224	891.328134	521.521339	406.65938	0.808875607	8.40E-06	8.76E-05	RALGAPA1	Ral GTPase activating protein catalytic alpha subunit 1 [Source:HGNC Symbol;Acc:HGNC:17770]
ENSG00000240583	124.011231	98.3473389	232.84084	281.042084	-1.21042284	8.44E-06	8.81E-05	AQP1	aquaporin 1 (Colton blood group) [Source:HGNC Symbol;Acc:HGNC:633]
ENSG00000170291	969.452876	847.913543	1398.09862	1373.27382	-0.609328728	8.45E-06	8.81E-05	ELP5	elongator acetyltransferase complex subunit 5 [Source:HGNC Symbol;Acc:HGNC:30617]
ENSG00000125968	17426.5306	15917.2067	11949.6869	12627.7318	0.440045005	8.51E-06	8.87E-05	ID1	inhibitor of DNA binding 1, HLH protein [Source:HGNC Symbol;Acc:HGNC:5360]
ENSG00000099875	4831.51692	4283.86832	6268.79185	6383.06189	-0.473142658	8.55E-06	8.90E-05	MKNK2	MAP kinase interacting serine/threonine kinase 2 [Source:HGNC Symbol;Acc:HGNC:7111]
ENSG00000130940	302.154348	253.39945	114.840052	135.198275	1.15116901	8.59E-06	8.94E-05	CASZ1	castor zinc finger 1 [Source:HGNC Symbol;Acc:HGNC:26002]
ENSG00000103126	1100.35362	971.06922	1536.1174	1577.66807	-0.588540748	8.62E-06	8.96E-05	AXIN1	axin 1 [Source:HGNC Symbol;Acc:HGNC:903]
ENSG00000197892	649.582638	631.7266	390.87761	365.141799	0.760959936	8.68E-06	9.02E-05	KIF13B	kinesin family member 13B [Source:HGNC Symbol;Acc:HGNC:14405]
ENSG00000197724	705.682957	636.15666	1030.39974	1070.94067	-0.64761025	8.73E-06	9.07E-05	PHF2	PHD finger protein 2 [Source:HGNC Symbol;Acc:HGNC:8920]
ENSG00000203867	174.206253	128.471749	44.2502954	54.2922209	1.617929565	8.76E-06	9.09E-05	RBM20	RNA binding motif protein 20 [Source:HGNC Symbol;Acc:HGNC:27424]
ENSG00000188895	1882.80543	1786.20032	2564.40998	2640.09231	-0.504526708	8.86E-06	9.18E-05	MSL1	male specific lethal 1 homolog [Source:HGNC Symbol;Acc:HGNC:27905]

ENSG00000033050	447.818334	402.249476	652.165068	821.835186	-0.794572235	8.86E-06	9.18E-05	ABCF2	ATP binding cassette subfamily F member 2 [Source:HGNC Symbol;Acc:HGNC:71]
ENSG00000068394	790.325543	666.28107	1105.20381	1201.88073	-0.664191563	8.86E-06	9.18E-05	GPKOW	G-patch domain and KOW motifs [Source:HGNC Symbol;Acc:HGNC:30677]
ENSG00000125430	122.042799	114.295556	35.8216677	33.0011539	1.779488101	8.87E-06	9.19E-05	HS3ST3B1	heparan sulfate-glucosamine 3-sulfotransferase 3B1 [Source:HGNC Symbol;Acc:HGNC:5198]
ENSG00000075624	84669.16	77194.6869	103652.103	120995.005	-0.472886206	8.91E-06	9.22E-05	ACTB	actin beta [Source:HGNC Symbol;Acc:HGNC:132]
ENSG00000041880	303.138564	303.016125	561.55732	508.856501	-0.820459587	8.96E-06	9.27E-05	PARP3	poly(ADP-ribose) polymerase family member 3 [Source:HGNC Symbol;Acc:HGNC:273]
ENSG00000015171	460.613143	508.570923	832.326985	760.091092	-0.715848709	9.06E-06	9.36E-05	ZMYND11	zinc finger MYND-type containing 11 [Source:HGNC Symbol;Acc:HGNC:16966]
ENSG00000144028	8919.95068	8006.891	11880.1507	11283.201	-0.452624974	9.09E-06	9.39E-05	SNRNP200	small nuclear ribonucleoprotein U5 subunit 200 [Source:HGNC Symbol;Acc:HGNC:30859]
ENSG00000143502	44.2897253	20.3782774	0	0	7.372506635	9.21E-06	9.50E-05	SUSD4	sushi domain containing 4 [Source:HGNC Symbol;Acc:HGNC:25470]
ENSG00000219507	209.638033	173.658364	358.216677	399.207506	-0.983997808	9.23E-06	9.52E-05	FTH1P8	ferritin heavy chain 1 pseudogene 8 [Source:HGNC Symbol;Acc:HGNC:3995]
ENSG00000179163	690.919715	681.343276	395.091923	426.885893	0.739382335	9.26E-06	9.54E-05	FUCA1	alpha-L-fucosidase 1 [Source:HGNC Symbol;Acc:HGNC:4006]
ENSG00000162227	227.353923	217.958967	395.091923	443.918747	-0.914129547	9.29E-06	9.58E-05	TAF6L	TATA-box binding protein associated factor 6 like [Source:HGNC Symbol;Acc:HGNC:17305]
ENSG00000044090	366.128396	303.016125	571.039527	608.924516	-0.819507589	9.31E-06	9.59E-05	CUL7	cullin 7 [Source:HGNC Symbol;Acc:HGNC:21024]
ENSG00000137714	999.963576	1099.54097	649.004333	690.895124	0.648280281	9.49E-06	9.77E-05	FDX1	ferredoxin 1 [Source:HGNC Symbol;Acc:HGNC:3638]
ENSG00000172375	546.239946	410.223585	261.287459	233.137184	0.950876321	9.50E-06	9.77E-05	C2CD2L	C2CD2 like [Source:HGNC Symbol;Acc:HGNC:29000]
ENSG00000188322	243.101381	206.44081	102.197111	84.0997147	1.269946039	9.57E-06	9.83E-05	SBK1	SH3 domain binding kinase 1 [Source:HGNC Symbol;Acc:HGNC:17699]
ENSG00000197860	524.587191	531.607237	317.127117	275.719318	0.833096124	9.58E-06	9.84E-05	SGTB	small glutamine rich tetratricopeptide repeat containing beta [Source:HGNC Symbol;Acc:HGNC:23567]
ENSG00000003989	348.412506	376.555126	197.019172	168.199429	0.989361104	9.62E-06	9.88E-05	SLC7A2	solute carrier family 7 member 2 [Source:HGNC Symbol;Acc:HGNC:11060]
ENSG00000172113	388.765367	335.798571	581.575311	688.766018	-0.810826644	9.65E-06	9.90E-05	NME6	NME/NM23 nucleoside diphosphate kinase 6 [Source:HGNC Symbol;Acc:HGNC:20567]
ENSG00000104833	168.300956	144.419966	48.4646093	59.6149876	1.532334346	9.67E-06	9.92E-05	TUBB4A	tubulin beta 4A class IVa [Source:HGNC Symbol;Acc:HGNC:20774]
ENSG00000121769	185.03263	148.850026	68.4826001	44.7112407	1.55923536	9.68E-06	9.92E-05	FABP3	fatty acid binding protein 3 [Source:HGNC Symbol;Acc:HGNC:3557]
ENSG00000253368	809.025649	767.286446	439.342219	513.114715	0.726731556	9.70E-06	9.94E-05	TRNP1	TMF1-regulated nuclear protein 1 [Source:HGNC Symbol;Acc:HGNC:34348]
ENSG00000079819	1927.09516	2026.30959	1387.56283	1346.65999	0.532084784	9.72E-06	9.94E-05	EPB41L2	erythrocyte membrane protein band 4.1 like 2 [Source:HGNC Symbol;Acc:HGNC:3379]
ENSG00000142937	15198.2653	13948.4879	18713.6606	22030.9316	-0.483304259	9.74E-06	9.96E-05	RPS8	ribosomal protein S8 [Source:HGNC Symbol;Acc:HGNC:10441]
ENSG00000269729	115.153286	116.06758	26.3394616	38.3239206	1.83876014	9.74E-06	9.96E-05	AC006262.2	novel transcript
ENSG00000131408	1311.96009	1052.58233	1740.51162	1906.61505	-0.625854343	9.82E-06	0.0001003	NR1H2	nuclear receptor subfamily 1 group H member 2 [Source:HGNC Symbol;Acc:HGNC:7965]
ENSG00000071575	233.25922	194.922654	76.9112277	95.8098015	1.309220921	9.83E-06	0.0001004	TRIB2	tribbles pseudokinase 2 [Source:HGNC Symbol;Acc:HGNC:30809]
ENSG00000146469	32.4791319	56.704772	3.16073539	2.1291067	4.076473754	9.85E-06	0.0001005	VIP	vasoactive intestinal peptide [Source:HGNC Symbol;Acc:HGNC:12693]
ENSG00000162063	1174.16983	1132.32341	1679.40407	1690.51072	-0.547158375	9.97E-06	0.0001017	CCNF	cyclin F [Source:HGNC Symbol;Acc:HGNC:1591]
ENSG00000182606	1408.41327	1458.37585	990.363755	929.355075	0.57861711	1.00E-05	0.000102	TRAK1	trafficking kinesin protein 1 [Source:HGNC Symbol;Acc:HGNC:29947]
ENSG00000109113	2477.27197	2246.04058	3291.37912	3358.66582	-0.493803674	1.02E-05	0.000104	RAB34	RAB34, member RAS oncogene family [Source:HGNC Symbol;Acc:HGNC:16519]
ENSG00000005156	575.766429	584.767961	876.577281	965.549889	-0.666460323	1.03E-05	0.0001052	LIG3	DNA ligase 3 [Source:HGNC Symbol;Acc:HGNC:6600]
ENSG00000176697	249.006678	204.668786	100.089954	90.4870348	1.250319703	1.06E-05	0.0001077	BDNF	brain derived neurotrophic factor [Source:HGNC Symbol;Acc:HGNC:1033]
ENSG00000100216	1360.18668	1307.7538	1799.51201	2311.14532	-0.623699782	1.06E-05	0.0001079	TOMM22	translocase of outer mitochondrial membrane 22 [Source:HGNC Symbol;Acc:HGNC:18002]
ENSG00000165655	1050.1586	791.208771	1437.08102	1510.6012	-0.679725148	1.07E-05	0.0001089	ZNF503	zinc finger protein 503 [Source:HGNC Symbol;Acc:HGNC:23589]
ENSG00000109805	949.768554	1154.47372	1796.35128	1508.4721	-0.650696637	1.08E-05	0.0001093	NCAPG	non-SMC condensin I complex subunit G [Source:HGNC Symbol;Acc:HGNC:24304]
ENSG00000131831	32.4791319	28.352386	0	0	7.286172383	1.08E-05	0.0001098	RAI2	retinoic acid induced 2 [Source:HGNC Symbol;Acc:HGNC:9835]
ENSG00000100410	865.125968	881.582001	1243.22259	1422.24328	-0.609623841	1.08E-05	0.00011	PHF5A	PHD finger protein 5A [Source:HGNC Symbol;Acc:HGNC:18000]
ENSG00000116525	326.759751	303.902137	152.768877	156.489342	1.027740999	1.09E-05	0.0001102	TRIM62	tripartite motif containing 62 [Source:HGNC Symbol;Acc:HGNC:25574]
ENSG00000211455	722.414631	948.032906	523.628496	430.079554	0.809246034	1.10E-05	0.0001113	STK38L	serine/threonine kinase 38 like [Source:HGNC Symbol;Acc:HGNC:17848]
ENSG00000130762	393.686447	310.990234	656.379382	590.827109	-0.825065376	1.10E-05	0.0001115	ARHGEF16	Rho guanine nucleotide exchange factor 16 [Source:HGNC Symbol;Acc:HGNC:15515]
ENSG00000116251	1170.23296	1179.28205	1662.54681	1770.35222	-0.547013968	1.11E-05	0.0001119	RPL22	ribosomal protein L22 [Source:HGNC Symbol;Acc:HGNC:10315]
ENSG00000162458	724.383063	663.623034	1065.16783	1080.52165	-0.628867933	1.12E-05	0.0001129	FBLIM1	filamin binding LIM protein 1 [Source:HGNC Symbol;Acc:HGNC:24686]
ENSG00000153404	123.027015	92.1452544	243.376625	251.234591	-1.20326402	1.12E-05	0.0001131	PLEKHG4B	pleckstrin homology and RhoGEF domain containing G4B [Source:HGNC Symbol;Acc:HGNC:29399]
ENSG00000198917	667.298528	601.60219	958.756401	1041.13318	-0.656838029	1.13E-05	0.0001141	SPOUT1	SPOUT domain containing methyltransferase 1 [Source:HGNC Symbol;Acc:HGNC:26933]
ENSG00000214078	1914.30035	1731.26757	2501.19527	2758.25773	-0.529025606	1.13E-05	0.0001141	CPNE1	copine 1 [Source:HGNC Symbol;Acc:HGNC:2314]
ENSG00000054277	336.601912	269.347667	144.340249	140.521042	1.087965292	1.15E-05	0.0001157	OPN3	opsin 3 [Source:HGNC Symbol;Acc:HGNC:14007]
ENSG00000111581	1540.29822	1804.80657	2485.39159	2400.5678	-0.546152339	1.15E-05	0.0001158	NUP107	nucleoporin 107 [Source:HGNC Symbol;Acc:HGNC:29914]
ENSG00000137203	787.372895	687.54536	1148.40052	1135.87842	-0.631749527	1.15E-05	0.0001163	TFAP2A	transcription factor AP-2 alpha [Source:HGNC Symbol;Acc:HGNC:11742]
ENSG00000118777	605.292913	700.835541	333.984373	410.917593	0.810806972	1.16E-05	0.0001164	ABCG2	ATP binding cassette subfamily G member 2 (Junior blood group) [Source:HGNC Symbol;Acc:HGNC:74]
ENSG00000153006	179.127334	211.756883	364.538148	396.013846	-0.959006546	1.16E-05	0.0001169	SREK1IP1	SREK1 interacting protein 1 [Source:HGNC Symbol;Acc:HGNC:26716]
ENSG00000163156	395.65488	367.695006	599.486145	707.927978	-0.776670372	1.16E-05	0.0001171	SCNM1	sodium channel modifier 1 [Source:HGNC Symbol;Acc:HGNC:23136]

ENSG00000232472	125.979663	115.181568	238.108732	295.945831	-1.147641746	1.17E-05	0.0001173	EEF1B2P3	eukaryotic translation elongation factor 1 beta 2 pseudogene 3 [Source:HGNC Symbol;Acc:HGNC:3210]
ENSG00000130770	2778.4421	2104.27865	3434.66579	4144.30619	-0.634693225	1.17E-05	0.0001177	ATP5IF1	ATP synthase inhibitory factor subunit 1 [Source:HGNC Symbol;Acc:HGNC:871]
ENSG00000171793	2243.02853	2148.57925	3073.28837	3048.8808	-0.479415431	1.17E-05	0.0001177	CTPS1	CTP synthase 1 [Source:HGNC Symbol;Acc:HGNC:2519]
ENSG00000149599	93.5005312	83.2851338	25.2858831	11.7100869	2.255073683	1.19E-05	0.0001192	DUSP15	dual specificity phosphatase 15 [Source:HGNC Symbol;Acc:HGNC:16236]
ENSG00000111252	1026.53741	989.675473	674.290216	618.505496	0.640974894	1.21E-05	0.0001212	SH2B3	SH2B adaptor protein 3 [Source:HGNC Symbol;Acc:HGNC:29605]
ENSG00000141576	512.776598	492.622706	300.269862	260.815571	0.841201827	1.21E-05	0.0001212	RNF157	ring finger protein 157 [Source:HGNC Symbol;Acc:HGNC:29402]
ENSG00000103241	749.972682	738.048047	1062.00709	1271.0767	-0.6488561	1.22E-05	0.0001224	FOXF1	forkhead box F1 [Source:HGNC Symbol;Acc:HGNC:3809]
ENSG00000057704	94.4847473	117.839604	30.5537754	26.6138338	1.89378034	1.23E-05	0.0001235	TMCC3	transmembrane and coiled-coil domain family 3 [Source:HGNC Symbol;Acc:HGNC:29199]
ENSG00000020577	515.729246	487.306634	290.787656	274.654764	0.826630434	1.24E-05	0.0001236	SAMD4A	sterile alpha motif domain containing 4A [Source:HGNC Symbol;Acc:HGNC:23023]
ENSG00000079246	5216.34543	5629.72064	7144.31555	7674.3651	-0.450140359	1.25E-05	0.0001247	XRCC5	X-ray repair cross complementing 5 [Source:HGNC Symbol;Acc:HGNC:12833]
ENSG00000196549	228.338139	266.689631	118.000788	100.068015	1.183254853	1.27E-05	0.0001272	MME	membrane metalloendopeptidase [Source:HGNC Symbol;Acc:HGNC:7154]
ENSG00000138759	1275.54409	1256.3651	891.327379	613.18273	0.750767514	1.28E-05	0.0001276	FRAS1	Fraser extracellular matrix complex subunit 1 [Source:HGNC Symbol;Acc:HGNC:19185]
ENSG00000131944	91.532099	87.7151941	197.019172	228.87897	-1.248881151	1.29E-05	0.0001287	FAAP24	FA core complex associated protein 24 [Source:HGNC Symbol;Acc:HGNC:28467]
ENSG00000105372	11687.5664	10484.1807	14401.364	16157.7908	-0.462940736	1.29E-05	0.0001287	RPS19	ribosomal protein S19 [Source:HGNC Symbol;Acc:HGNC:10402]
ENSG00000136463	482.265898	422.627753	700.629677	811.189653	-0.741135857	1.29E-05	0.0001291	TACO1	translational activator of cytochrome c oxidase I [Source:HGNC Symbol;Acc:HGNC:24316]
ENSG00000173113	4022.49128	3494.43157	5038.21221	5617.64803	-0.503626239	1.30E-05	0.0001291	TRMT112	tRNA methyltransferase subunit 11-2 [Source:HGNC Symbol;Acc:HGNC:26940]
ENSG00000136052	554.113675	658.306962	372.966776	311.914132	0.824462193	1.30E-05	0.0001292	SLC41A2	solute carrier family 41 member 2 [Source:HGNC Symbol;Acc:HGNC:31045]
ENSG00000197256	2947.72727	2630.56981	3806.57898	4022.94711	-0.489328424	1.30E-05	0.0001293	KANK2	KN motif and ankyrin repeat domains 2 [Source:HGNC Symbol;Acc:HGNC:29300]
ENSG00000119707	1218.45955	1368.00262	1921.72712	1861.90381	-0.548388155	1.30E-05	0.0001296	RBM25	RNA binding motif protein 25 [Source:HGNC Symbol;Acc:HGNC:23244]
ENSG00000120071	743.083169	722.985842	1114.68601	1115.65191	-0.605449535	1.30E-05	0.0001296	KANSL1	KAT8 regulatory NSL complex subunit 1 [Source:HGNC Symbol;Acc:HGNC:24565]
ENSG00000100393	2394.59782	2279.70903	3528.43427	3106.36668	-0.505438987	1.32E-05	0.0001307	EP300	E1A binding protein p300 [Source:HGNC Symbol;Acc:HGNC:3373]
ENSG00000090316	1182.04356	1052.58233	1614.0822	1695.83349	-0.567192527	1.32E-05	0.0001307	MAEA	macrophage erythroblast attacher [Source:HGNC Symbol;Acc:HGNC:13731]
ENSG00000226479	470.455304	500.596815	853.398554	737.735472	-0.71214779	1.32E-05	0.0001308	TMEM185B	transmembrane protein 185B [Source:HGNC Symbol;Acc:HGNC:18896]
ENSG00000107159	1044.2533	776.146566	1411.79514	1512.73031	-0.684884885	1.33E-05	0.0001317	CA9	carbonic anhydrase 9 [Source:HGNC Symbol;Acc:HGNC:1383]
ENSG00000106546	503.918653	437.689959	276.037557	232.07263	0.88929948	1.33E-05	0.0001322	AHR	aryl hydrocarbon receptor [Source:HGNC Symbol;Acc:HGNC:348]
ENSG00000114251	1676.12005	1857.96729	2961.60906	2361.17933	-0.590651349	1.34E-05	0.0001327	WNT5A	Wnt family member 5A [Source:HGNC Symbol;Acc:HGNC:12784]
ENSG00000166173	2271.5708	2100.7346	1539.27813	1513.79486	0.517935931	1.35E-05	0.0001333	LARP6	La ribonucleoprotein domain family member 6 [Source:HGNC Symbol;Acc:HGNC:24012]
ENSG00000181035	331.680832	323.394403	166.465397	162.876663	0.991935902	1.35E-05	0.0001333	SLC25A42	solute carrier family 25 member 42 [Source:HGNC Symbol;Acc:HGNC:28380]
ENSG00000101337	2270.58658	2156.55336	3095.41352	3066.9782	-0.477259252	1.35E-05	0.0001334	TM9SF4	transmembrane 9 superfamily member 4 [Source:HGNC Symbol;Acc:HGNC:30797]
ENSG00000074416	3580.57824	3062.05769	2345.26566	2295.17702	0.517239559	1.36E-05	0.0001343	MGLL	monoglyceride lipase [Source:HGNC Symbol;Acc:HGNC:17038]
ENSG00000237499	141.727121	191.378605	64.2682862	54.2922209	1.491434042	1.36E-05	0.0001344	AL357060.1	uncharacterized LOC100130476 [Source:NCBI gene;Acc:100130476]
ENSG00000161547	2027.4852	2292.99922	3061.69901	3061.65544	-0.502817566	1.37E-05	0.0001347	SRSF2	serine and arginine rich splicing factor 2 [Source:HGNC Symbol;Acc:HGNC:10783]
ENSG00000147604	9563.62802	10508.1031	12943.2114	14795.1625	-0.466651381	1.37E-05	0.0001347	RPL7	ribosomal protein L7 [Source:HGNC Symbol;Acc:HGNC:10363]
ENSG00000229807	3521.52527	3853.26646	2573.89218	2708.22372	0.481647517	1.37E-05	0.0001349	XIST	X inactive specific transcript [Source:HGNC Symbol;Acc:HGNC:12810]
ENSG00000261786	139.758689	154.166099	33.7145108	59.6149876	1.656029886	1.38E-05	0.0001357	AC006058.1	novel transcript
ENSG00000158710	15776.9844	14429.5924	19360.5578	22207.6474	-0.460651711	1.39E-05	0.0001368	TAGLN2	transgelin 2 [Source:HGNC Symbol;Acc:HGNC:11554]
ENSG00000175756	2128.85946	1882.77563	2797.25082	2917.94073	-0.510926447	1.40E-05	0.0001375	AURKAIP1	aurora kinase A interacting protein 1 [Source:HGNC Symbol;Acc:HGNC:24114]
ENSG00000152253	149.60085	187.834557	379.288246	312.978685	-1.035136993	1.41E-05	0.0001383	SPC25	SPC25, NDC80 kinetochore complex component [Source:HGNC Symbol;Acc:HGNC:24031]
ENSG00000107968	460.613143	427.943826	258.126723	223.556204	0.882954708	1.41E-05	0.0001385	MAP3K8	mitogen-activated protein kinase kinase kinase 8 [Source:HGNC Symbol;Acc:HGNC:6860]
ENSG00000095303	312.980726	276.435763	136.9652	145.843809	1.058951767	1.41E-05	0.0001386	PTGS1	prostaglandin-endoperoxide synthase 1 [Source:HGNC Symbol;Acc:HGNC:9604]
ENSG00000167889	462.581576	514.773008	799.666053	780.317606	-0.692347645	1.42E-05	0.0001395	MGAT5B	alpha-1,6-mannosylglycoprotein 6-beta-N-acetylglucosaminyltransferase B [Source:HGNC Symbol;Acc:HGNC:24140]
ENSG00000100320	2651.47822	2658.03619	3782.34668	3551.34998	-0.465967165	1.42E-05	0.0001395	RBFOX2	RNA binding fox-1 homolog 2 [Source:HGNC Symbol;Acc:HGNC:9906]
ENSG00000150990	1195.82258	1054.35435	1609.86789	1736.28651	-0.572911622	1.42E-05	0.0001395	DHX37	DEAH-box helicase 37 [Source:HGNC Symbol;Acc:HGNC:17210]
ENSG00000128228	1522.58233	1186.37015	787.023111	899.547581	0.683135017	1.43E-05	0.0001405	SDF2L1	stromal cell derived factor 2 like 1 [Source:HGNC Symbol;Acc:HGNC:10676]
ENSG00000198373	1129.8801	1002.07964	1549.81392	1615.99199	-0.570853127	1.44E-05	0.0001407	WWP2	WW domain containing E3 ubiquitin protein ligase 2 [Source:HGNC Symbol;Acc:HGNC:16804]
ENSG00000172292	747.020034	809.815024	515.199868	324.688772	0.890328792	1.44E-05	0.0001412	CERS6	ceramide synthase 6 [Source:HGNC Symbol;Acc:HGNC:23826]
ENSG00000187672	36.4159964	49.6166755	4.21431385	0	4.34737344	1.44E-05	0.0001413	ERC2	ELKS/RAB6-interacting/CAST family member 2 [Source:HGNC Symbol;Acc:HGNC:31922]
ENSG00000254615	125.979663	132.015797	31.6073539	47.9049008	1.698784986	1.47E-05	0.0001435	AC027031.2	novel transcript
ENSG00000160293	1278.49674	1198.77432	1731.02941	1884.25943	-0.54557368	1.47E-05	0.0001435	VAV2	vav guanine nucleotide exchange factor 2 [Source:HGNC Symbol;Acc:HGNC:12658]
ENSG00000115677	7817.62863	7477.9418	10130.1569	10260.1652	-0.41481621	1.47E-05	0.0001435	HDLBP	high density lipoprotein binding protein [Source:HGNC Symbol;Acc:HGNC:4857]
ENSG00000154734	715.525118	677.799227	434.074326	414.111253	0.715827782	1.49E-05	0.0001459	ADAMTS1	ADAM metallopeptidase with thrombospondin type 1 motif 1 [Source:HGNC Symbol;Acc:HGNC:217]
ENSG00000100353	2492.03521	2378.94239	3363.02245	3367.18225	-0.466559108	1.50E-05	0.0001462	EIF3D	eukaryotic translation initiation factor 3 subunit D [Source:HGNC Symbol;Acc:HGNC:3278]

ENSG00000139971	58.068751	54.9327478	0	1.06455335	6.736931911	1.51E-05	0.0001468	ARMH4	armadillo-like helical domain containing 4 [Source:HGNC Symbol;Acc:HGNC:19846]
ENSG00000145425	6354.09926	7098.72864	8688.86158	10352.7813	-0.501147046	1.52E-05	0.0001476	RPS3A	ribosomal protein S3A [Source:HGNC Symbol;Acc:HGNC:10421]
ENSG00000161654	375.970557	404.0215	632.147077	667.474951	-0.736079522	1.52E-05	0.0001476	LSM12	LSM12 homolog [Source:HGNC Symbol;Acc:HGNC:26407]
ENSG00000182568	57.0845349	75.3110253	9.48220616	12.7746402	2.574165522	1.53E-05	0.0001491	SATB1	SATB homeobox 1 [Source:HGNC Symbol;Acc:HGNC:10541]
ENSG00000167862	395.65488	364.150957	590.003939	711.121638	-0.776468518	1.54E-05	0.0001496	MRPL58	mitochondrial ribosomal protein L58 [Source:HGNC Symbol;Acc:HGNC:5359]
ENSG00000177181	187.985279	213.528907	91.6613262	69.1959678	1.319942329	1.54E-05	0.00015	RIMKLA	ribosomal modification protein rimK like family member A [Source:HGNC Symbol;Acc:HGNC:28725]
ENSG00000214063	1122.00637	907.276351	1570.88549	1534.02138	-0.614338459	1.55E-05	0.000151	TSPAN4	tetraspanin 4 [Source:HGNC Symbol;Acc:HGNC:11859]
ENSG00000128973	517.697678	415.539657	735.397767	835.67438	-0.752504431	1.56E-05	0.0001516	CLN6	CLN6, transmembrane ER protein [Source:HGNC Symbol;Acc:HGNC:2077]
ENSG00000103148	1314.91273	1135.86746	1737.35088	1908.74416	-0.573580299	1.56E-05	0.0001516	NPRL3	NPR3 like, GATOR1 complex subunit [Source:HGNC Symbol;Acc:HGNC:14124]
ENSG00000155380	2343.41858	2673.9844	4067.86644	3324.60011	-0.558912068	1.57E-05	0.0001521	SLC16A1	solute carrier family 16 member 1 [Source:HGNC Symbol;Acc:HGNC:10922]
ENSG00000138311	131.88496	147.078002	38.9824031	53.2276675	1.597914763	1.58E-05	0.0001533	ZNF365	zinc finger protein 365 [Source:HGNC Symbol;Acc:HGNC:18194]
ENSG00000072062	4210.47655	3749.60305	2720.33959	2942.42546	0.491125962	1.59E-05	0.0001537	PRKACA	protein kinase cAMP-activated catalytic subunit alpha [Source:HGNC Symbol;Acc:HGNC:9380]
ENSG00000160710	6153.31917	6136.51954	8613.00393	7949.01987	-0.430424382	1.59E-05	0.0001537	ADAR	adenosine deaminase, RNA specific [Source:HGNC Symbol;Acc:HGNC:225]
ENSG00000197766	1602.30384	1382.17882	985.095862	999.615596	0.588155338	1.59E-05	0.0001538	CFD	complement factor D [Source:HGNC Symbol;Acc:HGNC:2771]
ENSG00000072958	1242.08074	1115.48919	1725.76152	1722.44732	-0.54895781	1.59E-05	0.0001538	AP1M1	adaptor related protein complex 1 subunit mu 1 [Source:HGNC Symbol;Acc:HGNC:13667]
ENSG00000093000	1554.07725	1663.04464	2319.97977	2249.40123	-0.506012676	1.60E-05	0.0001543	NUP50	nucleoporin 50 [Source:HGNC Symbol;Acc:HGNC:8065]
ENSG00000100342	205.701169	204.668786	381.395403	387.497419	-0.905888813	1.60E-05	0.0001546	APOL1	apolipoprotein L1 [Source:HGNC Symbol;Acc:HGNC:618]
ENSG00000187210	299.2017	303.016125	164.35824	112.842655	1.119095983	1.60E-05	0.0001546	GCNT1	glucosaminyl (N-acetyl) transferase 1, core 2 [Source:HGNC Symbol;Acc:HGNC:4203]
ENSG00000172831	721.430415	656.534938	1085.18582	1036.87496	-0.623417736	1.61E-05	0.0001556	CES2	carboxylesterase 2 [Source:HGNC Symbol;Acc:HGNC:1864]
ENSG00000164949	127.948095	82.3991217	29.5001969	24.4847271	1.960168134	1.62E-05	0.0001559	GEM	GTP binding protein overexpressed in skeletal muscle [Source:HGNC Symbol;Acc:HGNC:4234]
ENSG00000132591	1104.29048	983.473389	1523.47446	1569.15164	-0.567331173	1.62E-05	0.000156	ERAL1	Era like 12S mitochondrial rRNA chaperone 1 [Source:HGNC Symbol;Acc:HGNC:3424]
ENSG00000110881	1000.94779	865.633784	528.896388	621.699157	0.697650272	1.62E-05	0.000156	ASIC1	acid sensing ion channel subunit 1 [Source:HGNC Symbol;Acc:HGNC:100]
ENSG00000115875	2344.40279	2377.17036	3148.09245	3429.99089	-0.478347179	1.62E-05	0.000156	SRSF7	serine and arginine rich splicing factor 7 [Source:HGNC Symbol;Acc:HGNC:10789]
ENSG00000160310	1041.30065	914.364448	1422.33092	1508.4721	-0.58414446	1.64E-05	0.0001576	PRMT2	protein arginine methyltransferase 2 [Source:HGNC Symbol;Acc:HGNC:5186]
ENSG00000172296	92.5163151	122.269665	28.4466185	30.8720472	1.857650012	1.65E-05	0.0001589	SPTLC3	serine palmitoyltransferase long chain base subunit 3 [Source:HGNC Symbol;Acc:HGNC:16253]
ENSG00000104823	1693.83594	1575.32945	1082.02508	1148.65306	0.551261682	1.66E-05	0.0001592	ECH1	enoyl-CoA hydratase 1 [Source:HGNC Symbol;Acc:HGNC:3149]
ENSG00000187498	5653.33738	5069.76102	4033.09835	3671.64451	0.476763698	1.67E-05	0.0001602	COL4A1	collagen type IV alpha 1 chain [Source:HGNC Symbol;Acc:HGNC:2202]
ENSG00000099284	351.365154	309.21821	538.378594	606.79541	-0.794514752	1.67E-05	0.0001605	H2AFY2	H2A histone family member Y2 [Source:HGNC Symbol;Acc:HGNC:14453]
ENSG00000182759	116.137502	134.673833	37.9288246	41.5175807	1.659332887	1.70E-05	0.0001631	MAFA	MAF bZIP transcription factor A [Source:HGNC Symbol;Acc:HGNC:23145]
ENSG00000115271	172.237821	204.668786	66.3754431	81.970608	1.346218105	1.72E-05	0.0001646	GCA	grancalcin [Source:HGNC Symbol;Acc:HGNC:15990]
ENSG00000078098	69.8793444	62.0208443	159.090348	184.16773	-1.380968633	1.72E-05	0.0001648	FAP	fibroblast activation protein alpha [Source:HGNC Symbol;Acc:HGNC:3590]
ENSG00000113013	9784.09243	10093.4494	12777.7996	13755.0938	-0.416613302	1.72E-05	0.000165	HSPA9	heat shock protein family A (Hsp70) member 9 [Source:HGNC Symbol;Acc:HGNC:5244]
ENSG00000006747	89.5636668	63.7928684	13.69652	17.0328536	2.318136486	1.74E-05	0.0001664	SCIN	scinderin [Source:HGNC Symbol;Acc:HGNC:21695]
ENSG00000102854	2954.61679	2456.02544	3805.52541	3935.65374	-0.51709342	1.75E-05	0.0001672	MSLN	mesothelin [Source:HGNC Symbol;Acc:HGNC:7371]
ENSG00000062822	1669.23054	1396.35501	2229.37203	2260.04676	-0.55087876	1.76E-05	0.0001686	POLD1	DNA polymerase delta 1, catalytic subunit [Source:HGNC Symbol;Acc:HGNC:9175]
ENSG00000164032	4555.93641	4656.8794	6714.45554	5940.20769	-0.457938438	1.77E-05	0.0001691	H2AFZ	H2A histone family member Z [Source:HGNC Symbol;Acc:HGNC:4741]
ENSG00000162419	257.864623	234.793196	443.556533	444.9833	-0.851535335	1.79E-05	0.0001707	GMEB1	glucocorticoid modulatory element binding protein 1 [Source:HGNC Symbol;Acc:HGNC:4370]
ENSG00000183691	177.158901	121.383652	46.3574523	53.2276675	1.582550766	1.79E-05	0.0001712	NOG	noggin [Source:HGNC Symbol;Acc:HGNC:7866]
ENSG00000184828	73.8162089	99.2333509	12.6429415	24.4847271	2.22264492	1.81E-05	0.0001724	ZBTB7C	zinc finger and BTB domain containing 7C [Source:HGNC Symbol;Acc:HGNC:31700]
ENSG00000111859	389.749583	312.762258	167.518976	184.16773	0.997399186	1.81E-05	0.0001728	NEDD9	neural precursor cell expressed, developmentally down-regulated 9 [Source:HGNC Symbol;Acc:HGNC:7733]
ENSG00000167685	305.106997	313.64827	525.735653	537.599442	-0.780952943	1.82E-05	0.0001733	ZNF444	zinc finger protein 444 [Source:HGNC Symbol;Acc:HGNC:16052]
ENSG00000070214	3528.41478	3791.24561	2762.48273	2429.31075	0.495622972	1.82E-05	0.0001738	SLC44A1	solute carrier family 44 member 1 [Source:HGNC Symbol;Acc:HGNC:18798]
ENSG00000164061	151.569282	176.3164	63.2147077	59.6149876	1.417164815	1.83E-05	0.000174	BSN	bassoon presynaptic cytomatrix protein [Source:HGNC Symbol;Acc:HGNC:1117]
ENSG00000172977	179.127334	155.052111	329.770059	330.011539	-0.982521111	1.83E-05	0.000174	KAT5	lysine acetyltransferase 5 [Source:HGNC Symbol;Acc:HGNC:5275]
ENSG00000121775	376.954773	354.404825	581.575311	651.50665	-0.753956042	1.84E-05	0.0001749	TMEM39B	transmembrane protein 39B [Source:HGNC Symbol;Acc:HGNC:25510]
ENSG00000118898	3196.73395	2781.19186	4196.40302	4159.20994	-0.483370101	1.84E-05	0.0001753	PPL	periplakin [Source:HGNC Symbol;Acc:HGNC:9273]
ENSG00000101197	19.6843224	9.74613268	63.2147077	89.4224814	-2.384017237	1.85E-05	0.0001756	BIRC7	baculoviral IAP repeat containing 7 [Source:HGNC Symbol;Acc:HGNC:13702]
ENSG00000142864	7354.06284	8149.53894	10145.9606	10924.4465	-0.442518223	1.85E-05	0.0001759	SERBP1	SERPINE1 mRNA binding protein 1 [Source:HGNC Symbol;Acc:HGNC:17860]
ENSG00000111144	2974.30111	3150.65889	2157.72869	2249.40123	0.474987512	1.88E-05	0.0001785	LTA4H	leukotriene A4 hydrolase [Source:HGNC Symbol;Acc:HGNC:6710]
ENSG00000185989	401.560176	336.684583	619.504136	632.34469	-0.762955595	1.89E-05	0.0001791	RASA3	RAS p21 protein activator 3 [Source:HGNC Symbol;Acc:HGNC:20331]

ENSG00000169193	187.001062	179.860449	77.9648062	69.1959678	1.317531491	1.94E-05	0.0001836	CCDC126	coiled-coil domain containing 126 [Source:HGNC Symbol;Acc:HGNC:22398]
ENSG00000020633	577.734861	484.648598	296.055548	306.591365	0.817245312	1.96E-05	0.0001856	RUNX3	runt related transcription factor 3 [Source:HGNC Symbol;Acc:HGNC:10473]
ENSG00000136935	689.935499	682.229288	1082.02508	1012.39024	-0.610177977	1.97E-05	0.0001864	GOLGA1	golgin A1 [Source:HGNC Symbol;Acc:HGNC:4424]
ENSG00000184281	506.871301	384.529235	716.433354	807.995993	-0.775430577	1.97E-05	0.0001866	TSSC4	tumor suppressing subtransferable candidate 4 [Source:HGNC Symbol;Acc:HGNC:12386]
ENSG00000005020	749.972682	1054.35435	553.128693	491.823648	0.788801918	1.99E-05	0.0001878	SKAP2	src kinase associated phosphoprotein 2 [Source:HGNC Symbol;Acc:HGNC:15687]
ENSG00000164294	755.877979	933.856713	1322.24097	1291.30321	-0.628383528	2.00E-05	0.0001886	GPX8	glutathione peroxidase 8 (putative) [Source:HGNC Symbol;Acc:HGNC:33100]
ENSG00000196562	1680.05691	1517.73866	1068.32856	1109.26459	0.55407548	2.00E-05	0.0001892	SULF2	sulfatase 2 [Source:HGNC Symbol;Acc:HGNC:20392]
ENSG00000184924	108.263773	95.6893027	214.930006	244.847271	-1.173740455	2.01E-05	0.0001898	PTRHD1	peptidyl-tRNA hydrolase domain containing 1 [Source:HGNC Symbol;Acc:HGNC:33782]
ENSG00000048342	127.948095	149.736038	288.680499	285.300298	-1.046251002	2.01E-05	0.0001898	CC2D2A	coiled-coil and C2 domain containing 2A [Source:HGNC Symbol;Acc:HGNC:29253]
ENSG00000181610	869.062832	811.587049	1230.57964	1279.59313	-0.579073708	2.03E-05	0.0001911	MRPS23	mitochondrial ribosomal protein S23 [Source:HGNC Symbol;Acc:HGNC:14509]
ENSG00000147676	273.612081	334.026547	149.608142	145.843809	1.041136518	2.05E-05	0.0001927	MAL2	mal, T cell differentiation protein 2 (gene/pseudogene) [Source:HGNC Symbol;Acc:HGNC:13634]
ENSG00000095587	46.2581576	60.2488202	7.37504924	6.3873201	2.952941426	2.05E-05	0.0001927	TLL2	tolloid like 2 [Source:HGNC Symbol;Acc:HGNC:11844]
ENSG00000196924	41863.6326	34948.7458	55330.7801	50935.6841	-0.468305386	2.05E-05	0.0001927	FLNA	filamin A [Source:HGNC Symbol;Acc:HGNC:3754]
ENSG00000175826	1429.0818	1184.59813	1879.58398	2001.3603	-0.570891427	2.08E-05	0.0001962	CTDNEP1	CTD nuclear envelope phosphatase 1 [Source:HGNC Symbol;Acc:HGNC:19085]
ENSG00000151692	166.332524	146.19199	57.9468154	57.4858809	1.436306517	2.11E-05	0.0001981	RNF144A	ring finger protein 144A [Source:HGNC Symbol;Acc:HGNC:20457]
ENSG00000134107	6255.67765	5944.25492	7981.91043	8334.38818	-0.419499444	2.12E-05	0.000199	BHLHE40	basic helix-loop-helix family member e40 [Source:HGNC Symbol;Acc:HGNC:1046]
ENSG00000170485	654.503719	660.964998	404.57413	399.207506	0.710733123	2.15E-05	0.0002017	NPAS2	neuronal PAS domain protein 2 [Source:HGNC Symbol;Acc:HGNC:7895]
ENSG00000059145	484.23433	414.653645	702.736834	776.059392	-0.719037489	2.15E-05	0.000202	UNKL	unkempt family like zinc finger [Source:HGNC Symbol;Acc:HGNC:14184]
ENSG00000106723	1694.82016	1886.31968	2905.7694	2380.34129	-0.561573526	2.16E-05	0.0002026	SPIN1	spindlin 1 [Source:HGNC Symbol;Acc:HGNC:11243]
ENSG00000003402	304.122781	310.990234	535.217859	518.437482	-0.776340002	2.17E-05	0.0002036	CFLAR	CASP8 and FADD like apoptosis regulator [Source:HGNC Symbol;Acc:HGNC:1876]
ENSG00000110066	285.422674	272.891715	494.128299	480.113561	-0.803536473	2.18E-05	0.0002045	KMT5B	lysine methyltransferase 5B [Source:HGNC Symbol;Acc:HGNC:24283]
ENSG00000101331	55.1161026	50.5026875	1.05357846	0	6.639908388	2.19E-05	0.0002052	CCM2L	CCM2 like scaffold protein [Source:HGNC Symbol;Acc:HGNC:16153]
ENSG00000136840	2282.39718	1992.64113	1358.06264	1548.92512	0.556177603	2.19E-05	0.0002052	ST6GALNAC4	ST6 N-acetylgalactosaminide alpha-2,6-sialyltransferase 4 [Source:HGNC Symbol;Acc:HGNC:17846]
ENSG00000081479	50.195022	57.590784	9.48220616	4.2582134	2.970766332	2.21E-05	0.0002068	LRP2	LDL receptor related protein 2 [Source:HGNC Symbol;Acc:HGNC:6694]
ENSG00000176783	866.110184	842.597471	1201.07945	1376.46748	-0.593167767	2.22E-05	0.0002073	RUFY1	RUN and FYVE domain containing 1 [Source:HGNC Symbol;Acc:HGNC:19760]
ENSG00000168904	489.155411	549.327478	280.251871	312.978685	0.808369093	2.24E-05	0.0002095	LRRC28	leucine rich repeat containing 28 [Source:HGNC Symbol;Acc:HGNC:28355]
ENSG00000124207	3701.63682	4641.81719	6320.4172	5728.36158	-0.529925555	2.29E-05	0.000214	CSE1L	chromosome segregation 1 like [Source:HGNC Symbol;Acc:HGNC:2431]
ENSG00000104763	1341.48657	1479.64014	972.452921	932.548735	0.566752618	2.30E-05	0.0002145	ASAH1	N-acylsphingosine amidohydrolase 1 [Source:HGNC Symbol;Acc:HGNC:735]
ENSG00000106086	908.431477	1002.07964	639.522127	584.439789	0.642688711	2.30E-05	0.0002147	PLEKHA8	pleckstrin homology domain containing A8 [Source:HGNC Symbol;Acc:HGNC:30037]
ENSG00000164070	1041.30065	1380.40679	813.362573	636.602903	0.740487672	2.30E-05	0.0002148	HSPA4L	heat shock protein family A (Hsp70) member 4 like [Source:HGNC Symbol;Acc:HGNC:17041]
ENSG00000115207	904.494613	838.16741	1252.70479	1345.59543	-0.576592532	2.33E-05	0.0002174	GTF3C2	general transcription factor IIIC subunit 2 [Source:HGNC Symbol;Acc:HGNC:4665]
ENSG00000113555	70.8635605	78.8550735	15.8036769	15.9683003	2.236912334	2.34E-05	0.0002176	PCDH12	protocadherin 12 [Source:HGNC Symbol;Acc:HGNC:8657]
ENSG00000102225	4886.63303	4373.35554	6370.98896	6235.08897	-0.445198607	2.34E-05	0.0002182	CDK16	cyclin dependent kinase 16 [Source:HGNC Symbol;Acc:HGNC:8749]
ENSG00000167695	668.282744	555.529563	962.970715	967.678995	-0.658601952	2.35E-05	0.0002184	FAM57A	family with sequence similarity 57 member A [Source:HGNC Symbol;Acc:HGNC:29646]
ENSG00000007402	69.8793444	66.4509046	16.8572554	5.32276675	2.617941549	2.35E-05	0.0002187	CACNA2D2	calcium voltage-gated channel auxiliary subunit alpha2delta 2 [Source:HGNC Symbol;Acc:HGNC:1400]
ENSG00000163220	56.1003187	48.7306634	7.37504924	6.3873201	2.928400503	2.36E-05	0.0002194	S100A9	S100 calcium binding protein A9 [Source:HGNC Symbol;Acc:HGNC:10499]
ENSG00000104490	42.3212931	37.2125066	0	2.1291067	5.228893879	2.38E-05	0.0002214	NCALD	neurocalcin delta [Source:HGNC Symbol;Acc:HGNC:7655]
ENSG00000196576	6991.8713	6086.90286	9081.84635	8777.24237	-0.449571477	2.39E-05	0.000222	PLXNB2	plexin B2 [Source:HGNC Symbol;Acc:HGNC:9104]
ENSG00000088247	6256.66186	5487.0727	7758.5518	8363.13112	-0.457244742	2.39E-05	0.000222	KHSRP	KH-type splicing regulatory protein [Source:HGNC Symbol;Acc:HGNC:6316]
ENSG00000124243	650.566854	547.555454	360.323834	340.657072	0.772627417	2.41E-05	0.0002231	BCAS4	breast carcinoma amplified sequence 4 [Source:HGNC Symbol;Acc:HGNC:14367]
ENSG00000178202	1202.7121	1360.91453	1930.15574	1802.28882	-0.541526269	2.41E-05	0.0002237	KDELC2	KDEL motif containing 2 [Source:HGNC Symbol;Acc:HGNC:28496]
ENSG00000170801	1412.35013	1257.25112	1931.20932	1915.13148	-0.527272317	2.42E-05	0.0002241	HTRA3	HtrA serine peptidase 3 [Source:HGNC Symbol;Acc:HGNC:30406]
ENSG00000164754	3679.98407	4148.30847	6170.80905	5061.95118	-0.520814548	2.42E-05	0.0002245	RAD21	RAD21 cohesin complex component [Source:HGNC Symbol;Acc:HGNC:9811]
ENSG00000169184	806.073001	772.602518	1208.4545	1151.84672	-0.580480609	2.43E-05	0.0002246	MN1	MN1 proto-oncogene, transcriptional regulator [Source:HGNC Symbol;Acc:HGNC:7180]
ENSG00000171004	452.739414	387.187271	820.737622	628.086477	-0.787406702	2.43E-05	0.0002246	HS6ST2	heparan sulfate 6-O-sulfotransferase 2 [Source:HGNC Symbol;Acc:HGNC:19133]
ENSG00000162733	187.001062	193.150629	448.824425	314.043238	-1.004784935	2.43E-05	0.0002246	DDR2	discoidin domain receptor tyrosine kinase 2 [Source:HGNC Symbol;Acc:HGNC:2731]
ENSG00000204866	78.7372895	66.4509046	15.8036769	13.8391936	2.291222955	2.44E-05	0.0002255	IGFL2	IGF like family member 2 [Source:HGNC Symbol;Acc:HGNC:32929]
ENSG00000198125	74.800425	63.7928684	170.679711	177.780409	-1.331692484	2.44E-05	0.0002256	MB	myoglobin [Source:HGNC Symbol;Acc:HGNC:6915]
ENSG00000198157	198.811656	364.150957	127.482994	96.8743549	1.328705403	2.45E-05	0.000226	HMGN5	high mobility group nucleosome binding domain 5 [Source:HGNC Symbol;Acc:HGNC:8013]
ENSG00000172936	791.309759	670.711131	1135.75758	1112.45825	-0.621578122	2.47E-05	0.000228	MYD88	MYD88, innate immune signal transduction adaptor [Source:HGNC Symbol;Acc:HGNC:7562]
ENSG00000105221	2313.89209	2028.96762	3044.84176	3058.46178	-0.491270285	2.49E-05	0.0002292	AKT2	AKT serine/threonine kinase 2 [Source:HGNC Symbol;Acc:HGNC:392]

ENSG00000150593	809.025649	932.970701	1410.74156	1244.46287	-0.60754821	2.51E-05	0.0002315	PDCD4	programmed cell death 4 [Source:HGNC Symbol;Acc:HGNC:8763]
ENSG00000135052	3102.2492	3039.90738	2196.71109	2269.62774	0.459620921	2.52E-05	0.0002316	GOLM1	golgi membrane protein 1 [Source:HGNC Symbol;Acc:HGNC:15451]
ENSG00000260231	121.058583	104.549423	38.9824031	27.6783871	1.757846941	2.55E-05	0.0002346	KDM7A-DT	KDM7A divergent transcript [Source:HGNC Symbol;Acc:HGNC:48959]
ENSG00000095370	83.65837	77.0830494	17.9108339	19.1619603	2.116042342	2.57E-05	0.0002363	SH2D3C	SH2 domain containing 3C [Source:HGNC Symbol;Acc:HGNC:16884]
ENSG00000057019	1781.43117	2038.71375	1394.93788	1126.29744	0.599689745	2.57E-05	0.0002363	DCBLD2	discoidin, CUB and LCCL domain containing 2 [Source:HGNC Symbol;Acc:HGNC:24627]
ENSG00000070182	96.4531796	90.3732303	21.0715692	27.6783871	1.938305982	2.59E-05	0.0002376	SPTB	spectrin beta, erythrocytic [Source:HGNC Symbol;Acc:HGNC:11274]
ENSG00000135624	9420.91668	8570.39467	11807.4538	12241.299	-0.418737307	2.61E-05	0.0002398	CCT7	chaperonin containing TCP1 subunit 7 [Source:HGNC Symbol;Acc:HGNC:1622]
ENSG00000151572	267.706784	266.689631	131.697308	125.617295	1.054313992	2.64E-05	0.000242	ANO4	anoctamin 4 [Source:HGNC Symbol;Acc:HGNC:23837]
ENSG00000151743	241.132949	265.803619	126.429415	109.648995	1.102851909	2.64E-05	0.000242	AMN1	antagonist of mitotic exit network 1 homolog [Source:HGNC Symbol;Acc:HGNC:27281]
ENSG00000122034	1401.52375	1567.35534	2029.19212	2265.36953	-0.532223317	2.64E-05	0.000242	GTF3A	general transcription factor IIIA [Source:HGNC Symbol;Acc:HGNC:4662]
ENSG00000178568	45.2739414	37.2125066	3.16073539	3.19366005	3.697575995	2.65E-05	0.0002433	ERBB4	erb-b2 receptor tyrosine kinase 4 [Source:HGNC Symbol;Acc:HGNC:3432]
ENSG00000148229	2184.95978	2091.87448	2815.16165	3208.5638	-0.49419452	2.67E-05	0.0002445	POLE3	DNA polymerase epsilon 3, accessory subunit [Source:HGNC Symbol;Acc:HGNC:13546]
ENSG00000090372	1471.4031	1322.81601	1941.74511	2073.74993	-0.52347318	2.67E-05	0.0002447	STRN4	striatin 4 [Source:HGNC Symbol;Acc:HGNC:15721]
ENSG00000198900	1642.6567	1830.50092	2414.80184	2493.18395	-0.498552555	2.68E-05	0.0002456	TOP1	DNA topoisomerase I [Source:HGNC Symbol;Acc:HGNC:11986]
ENSG00000141664	539.350433	529.835213	312.912803	317.236898	0.762677782	2.72E-05	0.0002491	ZCCHC2	zinc finger CCHC-type containing 2 [Source:HGNC Symbol;Acc:HGNC:22916]
ENSG00000100600	3418.18258	3282.67469	2456.94497	2443.14994	0.451456402	2.74E-05	0.00025	LGMN	legumain [Source:HGNC Symbol;Acc:HGNC:9472]
ENSG00000169084	408.449689	458.068236	714.326197	691.959678	-0.697916045	2.75E-05	0.0002508	DHRSX	dehydrogenase/reductase X-linked [Source:HGNC Symbol;Acc:HGNC:18399]
ENSG00000113649	1340.50235	1455.71782	2068.17452	1931.09978	-0.515995074	2.76E-05	0.0002516	TCERG1	transcription elongation regulator 1 [Source:HGNC Symbol;Acc:HGNC:15630]
ENSG00000260604	177.158901	169.228304	80.0719631	41.5175807	1.509595763	2.78E-05	0.0002534	AL590004.3	novel transcript
ENSG00000144655	870.047048	679.571251	418.27065	495.017308	0.762126118	2.78E-05	0.0002534	CSRNP1	cysteine and serine rich nuclear protein 1 [Source:HGNC Symbol;Acc:HGNC:14300]
ENSG00000142197	456.676279	432.373886	239.162311	259.751017	0.833297842	2.81E-05	0.0002562	DOP1B	DOP1 leucine zipper like protein B [Source:HGNC Symbol;Acc:HGNC:1291]
ENSG00000169174	5372.83579	4497.39723	3481.02324	3553.47908	0.488442692	2.81E-05	0.0002564	PCSK9	proprotein convertase subtilisin/kexin type 9 [Source:HGNC Symbol;Acc:HGNC:20001]
ENSG00000120802	1928.07938	2471.08764	3412.54064	3096.7857	-0.564839533	2.82E-05	0.0002565	TMPO	thymopoietin [Source:HGNC Symbol;Acc:HGNC:11875]
ENSG00000176619	5877.73866	5052.04078	7332.9061	7696.72072	-0.459709209	2.83E-05	0.0002574	LMNB2	lamin B2 [Source:HGNC Symbol;Acc:HGNC:6638]
ENSG00000105520	1357.23403	1239.53087	877.630859	873.998301	0.567728035	2.84E-05	0.0002585	PLPPR2	phospholipid phosphatase related 2 [Source:HGNC Symbol;Acc:HGNC:29566]
ENSG00000162385	703.714525	691.97542	1006.16743	1116.71646	-0.605091334	2.85E-05	0.0002588	MAGOH	mago homolog, exon junction complex subunit [Source:HGNC Symbol;Acc:HGNC:6815]
ENSG00000196465	800.167704	708.809649	1084.13224	1228.49457	-0.616456741	2.87E-05	0.0002603	MYL6B	myosin light chain 6B [Source:HGNC Symbol;Acc:HGNC:29823]
ENSG00000112855	575.766429	585.653973	849.184241	964.485335	-0.642881804	2.87E-05	0.0002609	HARS2	histidyl-tRNA synthetase 2, mitochondrial [Source:HGNC Symbol;Acc:HGNC:4817]
ENSG00000167775	2098.34876	1855.30926	2702.42876	2877.48771	-0.497364447	2.88E-05	0.0002615	CD320	CD320 molecule [Source:HGNC Symbol;Acc:HGNC:16692]
ENSG00000171863	6216.309	6882.5417	8326.43059	10108.9986	-0.492946195	2.90E-05	0.0002631	RPS7	ribosomal protein S7 [Source:HGNC Symbol;Acc:HGNC:10440]
ENSG00000142186	1666.27789	1358.25649	2176.6931	2260.04676	-0.553340821	2.93E-05	0.0002659	SCYL1	SCY1 like pseudokinase 1 [Source:HGNC Symbol;Acc:HGNC:14372]
ENSG00000168758	572.813781	448.322103	848.130662	819.70608	-0.709014513	2.95E-05	0.0002671	SEMA4C	semaphorin 4C [Source:HGNC Symbol;Acc:HGNC:10731]
ENSG00000008294	3371.92442	3505.94973	2621.30321	2321.79086	0.476582614	2.95E-05	0.0002675	SPAG9	sperm associated antigen 9 [Source:HGNC Symbol;Acc:HGNC:14524]
ENSG00000175029	1222.39642	1314.8419	1826.90505	1798.03061	-0.514425147	2.97E-05	0.0002684	CTBP2	C-terminal binding protein 2 [Source:HGNC Symbol;Acc:HGNC:2495]
ENSG00000143797	722.414631	812.473061	502.556927	448.17696	0.691377218	2.97E-05	0.0002687	MBOAT2	membrane bound O-acyltransferase domain containing 2 [Source:HGNC Symbol;Acc:HGNC:25193]
ENSG00000130304	416.323418	392.503343	212.822849	231.008077	0.865621612	3.00E-05	0.0002711	SLC27A1	solute carrier family 27 member 1 [Source:HGNC Symbol;Acc:HGNC:10995]
ENSG00000160179	576.750645	515.65902	298.162705	337.463412	0.78089786	3.01E-05	0.0002724	ABCG1	ATP binding cassette subfamily G member 1 [Source:HGNC Symbol;Acc:HGNC:73]
ENSG00000100316	23837.7144	21191.6365	28853.2998	31980.2472	-0.434034472	3.02E-05	0.000273	RPL3	ribosomal protein L3 [Source:HGNC Symbol;Acc:HGNC:10332]
ENSG00000048707	2009.76931	1959.85868	1460.25975	1323.23981	0.512002666	3.02E-05	0.000273	VPS13D	vacuolar protein sorting 13 homolog D [Source:HGNC Symbol;Acc:HGNC:23595]
ENSG00000100299	1395.61846	1291.80559	908.184635	918.709541	0.556591911	3.05E-05	0.000275	ARSA	arylsulfatase A [Source:HGNC Symbol;Acc:HGNC:713]
ENSG00000139437	416.323418	349.088752	631.093499	644.054777	-0.737403413	3.05E-05	0.0002751	TCHP	trichoplein keratin filament binding [Source:HGNC Symbol;Acc:HGNC:28135]
ENSG00000147140	13517.2242	13517.886	17172.2754	18352.8998	-0.394004097	3.06E-05	0.0002762	NONO	non-POU domain containing octamer binding [Source:HGNC Symbol;Acc:HGNC:7871]
ENSG00000027697	1162.35924	1329.01809	867.095074	770.736626	0.605487051	3.07E-05	0.0002766	IFNGR1	interferon gamma receptor 1 [Source:HGNC Symbol;Acc:HGNC:5439]
ENSG00000080824	32387.5998	39009.3391	51293.4674	47076.6783	-0.462328518	3.13E-05	0.000282	HSP90AA1	heat shock protein 90 alpha family class A member 1 [Source:HGNC Symbol;Acc:HGNC:5253]
ENSG00000116584	1481.24526	1248.391	1971.2453	1999.23119	-0.541140046	3.17E-05	0.0002857	ARHGEF2	Rho/Rac guanine nucleotide exchange factor 2 [Source:HGNC Symbol;Acc:HGNC:682]
ENSG00000167112	1748.95204	1641.78035	2332.62272	2404.82602	-0.482708129	3.18E-05	0.0002857	TRUB2	TruB pseudouridine synthase family member 2 [Source:HGNC Symbol;Acc:HGNC:17170]
ENSG00000093009	786.388678	737.162035	1151.56126	1123.10378	-0.578544214	3.21E-05	0.0002888	CDC45	cell division cycle 45 [Source:HGNC Symbol;Acc:HGNC:1739]
ENSG00000133134	92.5163151	95.6893027	16.8572554	30.8720472	1.980271992	3.22E-05	0.000289	BEX2	brain expressed X-linked 2 [Source:HGNC Symbol;Acc:HGNC:30933]
ENSG00000185507	95.4689635	69.9949529	173.840446	234.201737	-1.304694988	3.22E-05	0.0002895	IRF7	interferon regulatory factor 7 [Source:HGNC Symbol;Acc:HGNC:6122]
ENSG00000143390	1235.19123	1293.57761	1764.74392	1835.28998	-0.509386026	3.24E-05	0.000291	RFX5	regulatory factor X5 [Source:HGNC Symbol;Acc:HGNC:9986]
ENSG00000100626	102.358476	97.4613268	28.4466185	28.7429405	1.804662551	3.27E-05	0.000293	GALNT16	polypeptide N-acetylgalactosaminyltransferase 16 [Source:HGNC Symbol;Acc:HGNC:23233]
ENSG00000130707	7907.19229	7203.27806	9702.40406	10734.956	-0.435735315	3.30E-05	0.000296	ASS1	argininosuccinate synthase 1 [Source:HGNC Symbol;Acc:HGNC:758]

ENSG00000181929	897.6051	850.571579	1236.90111	1349.85365	-0.565503095	3.32E-05	0.0002973	PRKAG1	protein kinase AMP-activated non-catalytic subunit gamma 1 [Source:HGNC Symbol;Acc:HGNC:9385]
ENSG00000167182	382.86007	310.990234	603.700459	576.987916	-0.768202988	3.32E-05	0.0002976	SP2	Sp2 transcription factor [Source:HGNC Symbol;Acc:HGNC:11207]
ENSG00000160193	630.882532	544.897418	876.577281	969.808102	-0.651778407	3.35E-05	0.0002996	WDR4	WD repeat domain 4 [Source:HGNC Symbol;Acc:HGNC:12756]
ENSG00000084764	1221.4122	998.535594	611.075508	765.413859	0.689130429	3.35E-05	0.0002998	MAPRE3	microtubule associated protein RP/EB family member 3 [Source:HGNC Symbol;Acc:HGNC:6892]
ENSG00000185324	804.104569	635.270648	1104.15023	1147.58851	-0.646559952	3.36E-05	0.0003001	CDK10	cyclin dependent kinase 10 [Source:HGNC Symbol;Acc:HGNC:1770]
ENSG00000184564	92.5163151	105.435435	27.39304	28.7429405	1.81880921	3.41E-05	0.000305	SLITRK6	SLIT and NTRK like family member 6 [Source:HGNC Symbol;Acc:HGNC:23503]
ENSG00000114982	1197.79102	1083.59275	1654.11819	1638.34761	-0.529654117	3.42E-05	0.0003051	KANSL3	KAT8 regulatory NSL complex subunit 3 [Source:HGNC Symbol;Acc:HGNC:25473]
ENSG00000198947	337.586129	356.176849	199.126329	159.683003	0.951275694	3.43E-05	0.0003063	DMD	dystrophin [Source:HGNC Symbol;Acc:HGNC:2928]
ENSG00000188985	259.833055	176.3164	403.520551	441.78964	-0.956973461	3.44E-05	0.0003071	DHFRP1	dihydrofolate reductase pseudogene 1 [Source:HGNC Symbol;Acc:HGNC:2862]
ENSG00000197329	268.691	238.337245	121.161523	119.229975	1.076104457	3.44E-05	0.0003072	PELI1	pellino E3 ubiquitin protein ligase 1 [Source:HGNC Symbol;Acc:HGNC:8827]
ENSG00000266401	130.900744	126.699725	47.4110308	40.4530273	1.551449369	3.45E-05	0.0003074	AP002478.1	novel transcript, antisense to DLGAP1
ENSG00000138448	3202.63925	3485.57145	2557.03493	1886.38854	0.58998675	3.46E-05	0.0003082	ITGAV	integrin subunit alpha V [Source:HGNC Symbol;Acc:HGNC:6150]
ENSG00000071082	8191.63075	7818.17043	10105.9246	11537.6292	-0.435011791	3.47E-05	0.000309	RPL31	ribosomal protein L31 [Source:HGNC Symbol;Acc:HGNC:10334]
ENSG00000127884	2746.94719	2254.01469	3424.13	3749.3569	-0.520821134	3.49E-05	0.0003103	ECHS1	enoyl-CoA hydratase, short chain 1 [Source:HGNC Symbol;Acc:HGNC:3151]
ENSG00000245848	134.837608	122.269665	43.196717	44.7112407	1.547886774	3.49E-05	0.0003105	CEBPA	CCAAT enhancer binding protein alpha [Source:HGNC Symbol;Acc:HGNC:1833]
ENSG00000180537	879.88921	843.483483	587.896782	507.791948	0.653160998	3.51E-05	0.0003122	RNF182	ring finger protein 182 [Source:HGNC Symbol;Acc:HGNC:28522]
ENSG00000188986	3237.08681	2741.32132	4165.84924	4178.3719	-0.481316506	3.52E-05	0.000313	NELFB	negative elongation factor complex member B [Source:HGNC Symbol;Acc:HGNC:24324]
ENSG00000112578	598.4034	555.529563	893.434536	883.579281	-0.623309901	3.57E-05	0.000317	BYSL	bystin like [Source:HGNC Symbol;Acc:HGNC:1157]
ENSG00000085382	278.533161	304.788149	156.983191	130.940062	1.018850086	3.58E-05	0.0003174	HACE1	HECT domain and ankyrin repeat containing E3 ubiquitin protein ligase 1 [Source:HGNC Symbol;Acc:HGNC:21033]
ENSG00000114019	1435.97132	1334.33417	2042.88864	1898.09862	-0.508787903	3.58E-05	0.0003176	AMOTL2	angiomotin like 2 [Source:HGNC Symbol;Acc:HGNC:17812]
ENSG00000135074	245.069813	259.601534	95.8756401	134.133722	1.134187025	3.59E-05	0.000318	ADAM19	ADAM metallopeptidase domain 19 [Source:HGNC Symbol;Acc:HGNC:197]
ENSG00000163644	180.11155	216.186943	92.7149047	72.3896278	1.263761714	3.59E-05	0.0003186	PPM1K	protein phosphatase, Mg2+/Mn2+ dependent 1K [Source:HGNC Symbol;Acc:HGNC:25415]
ENSG00000168653	3933.91182	3629.99142	4931.80078	5360.02612	-0.444419654	3.61E-05	0.0003199	NDUFS5	NADH:ubiquinone oxidoreductase subunit S5 [Source:HGNC Symbol;Acc:HGNC:7712]
ENSG00000142676	11600.9554	13076.652	15649.8545	18400.8047	-0.464417664	3.64E-05	0.000322	RPL11	ribosomal protein L11 [Source:HGNC Symbol;Acc:HGNC:10301]
ENSG00000158716	1101.33784	964.867135	660.593696	688.766018	0.614291027	3.66E-05	0.0003239	DUSP23	dual specificity phosphatase 23 [Source:HGNC Symbol;Acc:HGNC:21480]
ENSG00000128849	36.4159964	36.3264945	2.10715692	1.06455335	4.517621648	3.67E-05	0.0003241	CGNL1	cingulin like 1 [Source:HGNC Symbol;Acc:HGNC:25931]
ENSG00000154310	514.74503	544.897418	331.877216	287.429405	0.774984322	3.69E-05	0.0003259	TNIK	TRAF2 and NCK interacting kinase [Source:HGNC Symbol;Acc:HGNC:30765]
ENSG00000136828	248.022462	256.943498	132.750886	102.197122	1.103763951	3.72E-05	0.0003285	RALGPS1	Ral GEF with PH domain and SH3 binding motif 1 [Source:HGNC Symbol;Acc:HGNC:16851]
ENSG00000067064	879.88921	1125.23532	678.50453	531.212122	0.729509809	3.74E-05	0.0003304	IDI1	isopentenyl-diphosphate delta isomerase 1 [Source:HGNC Symbol;Acc:HGNC:5387]
ENSG00000186594	388.765367	455.4102	233.894419	233.137184	0.854699301	3.75E-05	0.0003313	MIR22HG	MIR22 host gene [Source:HGNC Symbol;Acc:HGNC:28219]
ENSG00000102858	1088.54303	1024.22994	1504.51004	1539.34414	-0.527010374	3.81E-05	0.0003361	MGRN1	mahogunin ring finger 1 [Source:HGNC Symbol;Acc:HGNC:20254]
ENSG00000116157	53.1476704	91.2592424	192.804859	183.103176	-1.375760387	3.81E-05	0.0003361	GPX7	glutathione peroxidase 7 [Source:HGNC Symbol;Acc:HGNC:4559]
ENSG00000166902	1437.93975	1382.17882	1902.7627	2104.62197	-0.507012839	3.82E-05	0.0003365	MRPL16	mitochondrial ribosomal protein L16 [Source:HGNC Symbol;Acc:HGNC:14476]
ENSG00000012232	1864.10533	1747.21579	1282.20499	1259.36661	0.506619698	3.82E-05	0.0003368	EXTL3	exostosin like glycosyltransferase 3 [Source:HGNC Symbol;Acc:HGNC:3518]
ENSG00000087842	1537.34558	1773.79615	1060.95351	1183.78333	0.56117429	3.84E-05	0.0003376	PIR	pirin [Source:HGNC Symbol;Acc:HGNC:30048]
ENSG00000145198	59.0529671	52.2747116	11.5893631	1.06455335	3.133838195	3.85E-05	0.0003386	VWA5B2	von Willebrand factor A domain containing 5B2 [Source:HGNC Symbol;Acc:HGNC:25144]
ENSG00000134910	3245.94476	3352.66964	4375.51135	4482.83416	-0.424802397	3.85E-05	0.0003386	STT3A	STT3A, catalytic subunit of the oligosaccharyltransferase complex [Source:HGNC Symbol;Acc:HGNC:6172]
ENSG00000051108	990.121415	992.333509	1392.83073	1476.5355	-0.533414211	3.91E-05	0.0003436	HERPUD1	homocysteine inducible ER protein with ubiquitin like domain 1 [Source:HGNC Symbol;Acc:HGNC:13744]
ENSG00000084623	5099.22371	4404.36596	6232.97018	6907.88669	-0.467679901	3.92E-05	0.0003445	EIF3I	eukaryotic translation initiation factor 3 subunit I [Source:HGNC Symbol;Acc:HGNC:3272]
ENSG00000233493	145.663985	152.394075	281.305449	307.655918	-0.982160651	3.94E-05	0.0003457	TMEM238	transmembrane protein 238 [Source:HGNC Symbol;Acc:HGNC:40042]
ENSG00000136527	3210.51298	3155.97496	4431.35101	4158.14539	-0.43212272	3.94E-05	0.0003459	TRA2B	transformer 2 beta homolog [Source:HGNC Symbol;Acc:HGNC:10781]
ENSG00000243701	146.648202	164.798243	62.1611293	57.4858809	1.38066094	3.96E-05	0.0003469	DUBR	DPPA2 upstream binding RNA [Source:HGNC Symbol;Acc:HGNC:48569]
ENSG00000104524	485.218546	343.77268	683.772422	747.316452	-0.789269059	3.96E-05	0.0003475	PYCR3	pyrroline-5-carboxylate reductase 3 [Source:HGNC Symbol;Acc:HGNC:25846]
ENSG00000101493	233.25922	224.161052	115.893631	90.4870348	1.147800482	3.99E-05	0.0003494	ZNF516	zinc finger protein 516 [Source:HGNC Symbol;Acc:HGNC:28990]
ENSG00000100714	4884.66459	4884.5845	6268.79185	6765.23654	-0.415954127	4.00E-05	0.0003498	MTHFD1	methylenetetrahydrofolate dehydrogenase, cyclohydrolase and formyltetrahydrofolate synthetase 1 [Source:HGNC Symbol;Acc:HGNC:7432]
ENSG00000158106	340.538777	303.902137	175.947603	155.424789	0.958976889	4.00E-05	0.0003498	RHPN1	rhophilin Rho GTPase binding protein 1 [Source:HGNC Symbol;Acc:HGNC:19973]
ENSG00000148200	295.264835	240.109269	115.893631	137.327382	1.079411084	4.01E-05	0.0003503	NR6A1	nuclear receptor subfamily 6 group A member 1 [Source:HGNC Symbol;Acc:HGNC:7985]
ENSG00000198829	16.731674	33.6684583	0	0	7.017468949	4.12E-05	0.0003605	SUCNR1	succinate receptor 1 [Source:HGNC Symbol;Acc:HGNC:4542]
ENSG00000077150	2694.78373	2391.34655	1607.76073	1907.6796	0.532701657	4.13E-05	0.0003611	NFKB2	nuclear factor kappa B subunit 2 [Source:HGNC Symbol;Acc:HGNC:7795]
ENSG00000115204	1037.36379	906.390339	632.147077	632.34469	0.619825923	4.15E-05	0.0003627	MPV17	MPV17, mitochondrial inner membrane protein [Source:HGNC Symbol;Acc:HGNC:7224]

ENSG00000198162	1142.67491	1414.96126	901.863164	679.185037	0.694266688	4.16E-05	0.0003627	MAN1A2	mannosidase alpha class 1A member 2 [Source:HGNC Symbol;Acc:HGNC:6822]
ENSG00000120800	1249.95447	1423.82138	1987.04898	1858.71015	-0.523973416	4.18E-05	0.0003649	UTP20	UTP20, small subunit processome component [Source:HGNC Symbol;Acc:HGNC:17897]
ENSG00000111321	3068.78586	2724.48709	3859.25791	4125.14423	-0.463030768	4.19E-05	0.0003653	LTBR	lymphotoxin beta receptor [Source:HGNC Symbol;Acc:HGNC:6718]
ENSG00000132773	364.159964	318.964342	532.057123	628.086477	-0.764812146	4.21E-05	0.0003665	TOE1	target of EGR1, exonuclease [Source:HGNC Symbol;Acc:HGNC:15954]
ENSG00000236682	166.332524	236.56522	79.0183847	87.2933747	1.278047859	4.23E-05	0.000368	AC068282.1	novel transcript
ENSG00000138347	2079.64866	2015.67744	2905.7694	2727.38568	-0.460068193	4.23E-05	0.0003685	MYPN	myopalladin [Source:HGNC Symbol;Acc:HGNC:23246]
ENSG00000101972	1621.00395	2047.57387	3254.50387	2438.89173	-0.633716093	4.27E-05	0.0003718	STAG2	stromal antigen 2 [Source:HGNC Symbol;Acc:HGNC:11355]
ENSG00000214274	165.348308	147.078002	57.9468154	62.8086477	1.370964629	4.29E-05	0.0003735	ANG	angiogenin [Source:HGNC Symbol;Acc:HGNC:483]
ENSG00000237686	615.135074	515.65902	318.180696	345.979839	0.767105245	4.33E-05	0.0003761	AL109615.3	uncharacterized LOC101929705 [Source:NCBI gene;Acc:101929705]
ENSG00000164674	141.727121	128.471749	40.0359816	54.2922209	1.518162763	4.35E-05	0.0003781	SYTL3	synaptotagmin like 3 [Source:HGNC Symbol;Acc:HGNC:15587]
ENSG00000130203	315.933374	283.52386	133.804465	165.005769	1.004132264	4.35E-05	0.0003781	APOE	apolipoprotein E [Source:HGNC Symbol;Acc:HGNC:613]
ENSG00000172893	3447.70906	3100.1562	2353.69428	2399.50325	0.461941704	4.36E-05	0.0003787	DHCR7	7-dehydrocholesterol reductase [Source:HGNC Symbol;Acc:HGNC:2860]
ENSG00000120693	259.833055	250.741413	135.911622	104.326228	1.087263848	4.40E-05	0.0003819	SMAD9	SMAD family member 9 [Source:HGNC Symbol;Acc:HGNC:6774]
ENSG00000242960	220.46441	179.860449	346.627314	408.788486	-0.917452357	4.46E-05	0.0003865	FTH1P23	ferritin heavy chain 1 pseudogene 23 [Source:HGNC Symbol;Acc:HGNC:37641]
ENSG00000166411	1645.60935	1676.33482	1182.11503	1158.23405	0.505349675	4.47E-05	0.0003876	IDH3A	isocitrate dehydrogenase 3 (NAD(+)) alpha [Source:HGNC Symbol;Acc:HGNC:5384]
ENSG00000178695	308.059645	308.332198	172.786868	139.456489	0.980997902	4.48E-05	0.0003877	KCTD12	potassium channel tetramerization domain containing 12 [Source:HGNC Symbol;Acc:HGNC:14678]
ENSG00000115649	372.033693	351.746789	577.360599	608.924516	-0.713166414	4.50E-05	0.0003897	CNPPD1	cyclin Pas1/PHO80 domain containing 1 [Source:HGNC Symbol;Acc:HGNC:25220]
ENSG00000136944	175.190469	147.078002	61.1075508	64.9377544	1.353575914	4.52E-05	0.0003914	LMX1B	LIM homeobox transcription factor 1 beta [Source:HGNC Symbol;Acc:HGNC:6654]
ENSG00000013275	2234.17059	1922.64617	2789.87577	3111.68944	-0.505942188	4.53E-05	0.0003914	PSMC4	proteasome 26S subunit, ATPase 4 [Source:HGNC Symbol;Acc:HGNC:9551]
ENSG00000089597	12828.2729	12071.0283	16449.5205	16089.6593	-0.386114082	4.54E-05	0.0003921	GANAB	glucosidase II alpha subunit [Source:HGNC Symbol;Acc:HGNC:4138]
ENSG00000136842	270.659433	278.207787	118.000788	149.037469	1.039738716	4.54E-05	0.0003925	TMOD1	tropomodulin 1 [Source:HGNC Symbol;Acc:HGNC:11871]
ENSG00000113657	200.780088	221.503015	93.7684831	95.8098015	1.155904921	4.55E-05	0.0003926	DPYSL3	dihydropyrimidinase like 3 [Source:HGNC Symbol;Acc:HGNC:3015]
ENSG00000197375	1111.18	1108.40109	736.451345	762.220199	0.566613188	4.55E-05	0.0003931	SLC22A5	solute carrier family 22 member 5 [Source:HGNC Symbol;Acc:HGNC:10969]
ENSG00000079150	151.569282	209.098847	65.3218647	77.7123946	1.33581848	4.57E-05	0.0003942	FKBP7	FK506 binding protein 7 [Source:HGNC Symbol;Acc:HGNC:3723]
ENSG00000131979	439.944605	576.793852	278.144714	292.752171	0.833657213	4.60E-05	0.0003968	GCH1	GTP cyclohydrolase 1 [Source:HGNC Symbol;Acc:HGNC:4193]
ENSG00000184254	309.043861	293.269992	525.735653	492.888201	-0.758424406	4.61E-05	0.0003969	ALDH1A3	aldehyde dehydrogenase 1 family member A3 [Source:HGNC Symbol;Acc:HGNC:409]
ENSG00000102978	1533.40871	1470.78002	2102.94261	2083.33091	-0.478840782	4.61E-05	0.000397	POLR2C	RNA polymerase II subunit C [Source:HGNC Symbol;Acc:HGNC:9189]
ENSG00000213079	579.703294	592.742069	908.184635	878.256514	-0.607458536	4.62E-05	0.000398	SCAF8	SR-related CTD associated factor 8 [Source:HGNC Symbol;Acc:HGNC:20959]
ENSG00000164818	869.062832	830.193302	1201.07945	1296.62598	-0.555882781	4.64E-05	0.0003995	DNAAF5	dynein axonemal assembly factor 5 [Source:HGNC Symbol;Acc:HGNC:26013]
ENSG00000242732	61.0213993	62.9068564	11.5893631	11.7100869	2.411285947	4.66E-05	0.0004011	RTL5	retrotransposon Gag like 5 [Source:HGNC Symbol;Acc:HGNC:29430]
ENSG00000223959	265.738352	233.907184	409.842022	481.178114	-0.835344466	4.71E-05	0.0004046	AFG3L1P	AFG3 like matrix AAA peptidase subunit 1, pseudogene [Source:HGNC Symbol;Acc:HGNC:314]
ENSG00000172819	2131.81211	1865.9414	2797.25082	2781.6779	-0.481148561	4.72E-05	0.0004051	RARG	retinoic acid receptor gamma [Source:HGNC Symbol;Acc:HGNC:9866]
ENSG00000115652	1204.68053	1348.51036	882.898751	845.25536	0.563396932	4.72E-05	0.0004051	UXS1	UDP-glucuronate decarboxylase 1 [Source:HGNC Symbol;Acc:HGNC:17729]
ENSG00000220412	59.0529671	61.1348323	1.05357846	13.8391936	3.015499894	4.75E-05	0.0004078	AL356234.1	protein tyrosine phosphatase, non-receptor type 11 (PTPN11) pseudogene
ENSG00000116406	953.705419	1084.47876	721.701247	500.340075	0.738135835	4.75E-05	0.0004078	EDEM3	ER degradation enhancing alpha-mannosidase like protein 3 [Source:HGNC Symbol;Acc:HGNC:16787]
ENSG00000213977	79.7215056	101.005375	182.269074	240.589057	-1.224242683	4.77E-05	0.0004089	TAX1BP3	Tax1 binding protein 3 [Source:HGNC Symbol;Acc:HGNC:30684]
ENSG00000171861	196.843224	217.072955	365.591726	389.626526	-0.866786778	4.79E-05	0.0004104	MRM3	mitochondrial rRNA methyltransferase 3 [Source:HGNC Symbol;Acc:HGNC:18485]
ENSG00000177098	82.6741539	76.1970373	22.1251477	15.9683003	2.059420836	4.82E-05	0.0004126	SCN4B	sodium voltage-gated channel beta subunit 4 [Source:HGNC Symbol;Acc:HGNC:10592]
ENSG00000152661	48.2265898	33.6684583	5.26789231	1.06455335	3.688656151	4.84E-05	0.0004142	GJA1	gap junction protein alpha 1 [Source:HGNC Symbol;Acc:HGNC:4274]
ENSG00000108946	1788.32069	2013.90542	2806.73302	2547.47617	-0.493539958	4.85E-05	0.0004146	PRKAR1A	protein kinase cAMP-dependent type I regulatory subunit alpha [Source:HGNC Symbol;Acc:HGNC:9388]
ENSG00000156675	892.684019	861.203724	597.378988	541.857655	0.622297453	4.85E-05	0.0004147	RAB11FIP1	RAB11 family interacting protein 1 [Source:HGNC Symbol;Acc:HGNC:30265]
ENSG00000147883	1056.06389	979.043328	1585.63559	1394.56489	-0.55063859	4.88E-05	0.0004173	CDKN2B	cyclin dependent kinase inhibitor 2B [Source:HGNC Symbol;Acc:HGNC:1788]
ENSG00000197043	3268.58173	2812.20228	2021.81707	2265.36953	0.504014824	4.92E-05	0.0004199	ANXA6	annexin A6 [Source:HGNC Symbol;Acc:HGNC:544]
ENSG00000106080	682.06177	776.146566	473.05673	438.59598	0.678079532	4.92E-05	0.0004205	FKBP14	FK506 binding protein 14 [Source:HGNC Symbol;Acc:HGNC:18625]
ENSG00000187514	14867.5687	15733.8022	18846.4115	24057.8412	-0.487499102	4.93E-05	0.0004211	PTMA	prothymosin alpha [Source:HGNC Symbol;Acc:HGNC:9623]
ENSG00000167526	25755.9516	22237.1307	31004.707	33585.5936	-0.428529694	4.94E-05	0.0004214	RPL13	ribosomal protein L13 [Source:HGNC Symbol;Acc:HGNC:10303]
ENSG00000143258	469.471088	388.959295	666.915167	734.541812	-0.708151793	4.95E-05	0.0004217	USP21	ubiquitin specific peptidase 21 [Source:HGNC Symbol;Acc:HGNC:12620]
ENSG00000087253	900.557748	1002.96565	639.522127	606.79541	0.611350412	4.95E-05	0.0004217	LPCAT2	lysophosphatidylcholine acyltransferase 2 [Source:HGNC Symbol;Acc:HGNC:26032]
ENSG00000127980	532.46092	547.555454	305.537754	339.592519	0.743586591	4.95E-05	0.0004217	PEX1	peroxisomal biogenesis factor 1 [Source:HGNC Symbol;Acc:HGNC:8850]
ENSG00000107816	2047.16953	1782.65627	2534.90978	2961.58742	-0.521525322	4.97E-05	0.0004231	LZTS2	leucine zipper tumor suppressor 2 [Source:HGNC Symbol;Acc:HGNC:29381]
ENSG00000157657	1062.95341	984.359401	1499.24215	1450.98622	-0.527429418	4.98E-05	0.0004235	ZNF618	zinc finger protein 618 [Source:HGNC Symbol;Acc:HGNC:29416]
ENSG00000105173	1159.40659	1158.01776	773.326591	802.673226	0.556270699	5.00E-05	0.0004249	CCNE1	cyclin E1 [Source:HGNC Symbol;Acc:HGNC:1589]

ENSG00000247199	160.427227	139.103894	49.5181877	62.8086477	1.414614327	5.01E-05	0.0004262	AC091948.1	uncharacterized LOC102546294 [Source:NCBI gene;Acc:102546294]
ENSG00000100162	574.782213	510.342948	838.648456	843.126253	-0.632771099	5.02E-05	0.0004262	CENPM	centromere protein M [Source:HGNC Symbol;Acc:HGNC:18352]
ENSG00000130766	661.393231	558.187599	911.34537	980.453636	-0.634169625	5.02E-05	0.0004264	SESN2	sestrin 2 [Source:HGNC Symbol;Acc:HGNC:20746]
ENSG00000198356	2134.76476	1818.09675	2724.5539	2848.74477	-0.496022166	5.02E-05	0.0004264	ASNA1	arsA arsenite transporter, ATP-binding, homolog 1 (bacterial) [Source:HGNC Symbol;Acc:HGNC:752]
ENSG00000139880	1066.89027	869.177833	571.039527	651.50665	0.66270499	5.05E-05	0.0004288	CDH24	cadherin 24 [Source:HGNC Symbol;Acc:HGNC:14265]
ENSG00000066827	202.74852	149.736038	348.734471	336.398859	-0.960994812	5.06E-05	0.0004294	ZFAT	zinc finger and AT-hook domain containing [Source:HGNC Symbol;Acc:HGNC:19899]
ENSG00000166257	54.1318865	40.7565548	7.37504924	4.2582134	3.025804226	5.11E-05	0.0004331	SCN3B	sodium voltage-gated channel beta subunit 3 [Source:HGNC Symbol;Acc:HGNC:20665]
ENSG00000100348	819.852026	793.866807	1168.41851	1196.55797	-0.551583988	5.11E-05	0.0004331	TXN2	thioredoxin 2 [Source:HGNC Symbol;Acc:HGNC:17772]
ENSG00000109475	3989.02793	4127.04418	5082.4625	6369.22269	-0.496643911	5.14E-05	0.0004353	RPL34	ribosomal protein L34 [Source:HGNC Symbol;Acc:HGNC:10340]
ENSG00000133710	221.448627	247.197365	103.250689	116.036315	1.096301003	5.15E-05	0.0004357	SPINK5	serine peptidase inhibitor, Kazal type 5 [Source:HGNC Symbol;Acc:HGNC:15464]
ENSG00000213719	5583.45804	5254.93754	7015.77898	7347.54722	-0.406315098	5.17E-05	0.0004374	CLIC1	chloride intracellular channel 1 [Source:HGNC Symbol;Acc:HGNC:2062]
ENSG00000068489	1205.66474	1140.29752	1566.67117	1864.03292	-0.548467985	5.18E-05	0.0004382	PRR11	proline rich 11 [Source:HGNC Symbol;Acc:HGNC:25619]
ENSG00000175793	4292.16649	3715.93459	5336.37491	5610.19616	-0.451153381	5.21E-05	0.00044	SFN	stratifin [Source:HGNC Symbol;Acc:HGNC:10773]
ENSG00000234975	369.081044	359.720897	548.914379	665.345844	-0.736551299	5.22E-05	0.0004409	FTH1P2	ferritin heavy chain 1 pseudogene 2 [Source:HGNC Symbol;Acc:HGNC:3989]
ENSG00000249353	275.580513	341.114644	488.860407	590.827109	-0.806669306	5.25E-05	0.0004435	NPM1P27	nucleophosmin 1 pseudogene 27 [Source:HGNC Symbol;Acc:HGNC:45206]
ENSG00000110328	151.569282	117.839604	30.5537754	56.4213276	1.630797256	5.29E-05	0.0004465	GALNT18	polypeptide N-acetylgalactosaminyltransferase 18 [Source:HGNC Symbol;Acc:HGNC:30488]
ENSG00000058063	1520.6139	1742.78573	1200.02587	958.098015	0.596808266	5.36E-05	0.0004519	ATP11B	ATPase phospholipid transporting 11B (putative) [Source:HGNC Symbol;Acc:HGNC:13553]
ENSG00000196083	479.31325	502.368839	285.519763	293.816725	0.761085348	5.38E-05	0.0004532	IL1RAP	interleukin 1 receptor accessory protein [Source:HGNC Symbol;Acc:HGNC:5995]
ENSG00000203875	1078.70087	1033.09006	1428.65239	1650.05769	-0.54398047	5.41E-05	0.000456	SNHG5	small nucleolar RNA host gene 5 [Source:HGNC Symbol;Acc:HGNC:21026]
ENSG00000131043	646.62999	603.374214	999.845961	903.805794	-0.607230678	5.42E-05	0.000456	AAR2	AAR2 splicing factor homolog [Source:HGNC Symbol;Acc:HGNC:15886]
ENSG00000161996	881.857642	692.861432	1162.09704	1270.01215	-0.628005361	5.42E-05	0.0004562	WDR90	WD repeat domain 90 [Source:HGNC Symbol;Acc:HGNC:26960]
ENSG00000111142	1618.0513	2009.47536	2979.51989	2431.43985	-0.576501753	5.43E-05	0.0004568	METAP2	methionyl aminopeptidase 2 [Source:HGNC Symbol;Acc:HGNC:16672]
ENSG00000018610	399.591744	375.669114	650.057911	603.60175	-0.693813406	5.44E-05	0.000457	CXorf56	chromosome X open reading frame 56 [Source:HGNC Symbol;Acc:HGNC:26239]
ENSG00000137285	86.6110184	70.8809649	17.9108339	20.2265137	2.045288991	5.44E-05	0.0004574	TUBB2B	tubulin beta 2B class IIb [Source:HGNC Symbol;Acc:HGNC:30829]
ENSG00000137269	374.002125	365.036969	217.037163	184.16773	0.881070058	5.45E-05	0.0004577	LRRC1	leucine rich repeat containing 1 [Source:HGNC Symbol;Acc:HGNC:14307]
ENSG00000100632	2553.05661	2509.18616	3416.75495	3400.1834	-0.429392684	5.46E-05	0.0004583	ERH	ERH, mRNA splicing and mitosis factor [Source:HGNC Symbol;Acc:HGNC:3447]
ENSG00000267530	91.532099	80.6270976	170.679711	233.137184	-1.230910872	5.50E-05	0.0004611	LINC01836	long intergenic non-protein coding RNA 1836 [Source:HGNC Symbol;Acc:HGNC:52652]
ENSG00000128567	10601.976	9754.9928	7916.58857	7442.29247	0.40638343	5.50E-05	0.0004615	PODXL	podocalyxin like [Source:HGNC Symbol;Acc:HGNC:9171]
ENSG00000213442	1171.21718	905.504327	1502.40289	1657.50957	-0.606349721	5.52E-05	0.0004629	RPL18AP3	ribosomal protein L18a pseudogene 3 [Source:HGNC Symbol;Acc:HGNC:31387]
ENSG00000166681	1452.70299	1152.70169	1833.22652	2043.94243	-0.574110765	5.53E-05	0.0004637	BEX3	brain expressed X-linked 3 [Source:HGNC Symbol;Acc:HGNC:13388]
ENSG00000138463	573.797997	670.711131	387.716874	369.400013	0.717564606	5.55E-05	0.000465	DIRC2	disrupted in renal carcinoma 2 [Source:HGNC Symbol;Acc:HGNC:16628]
ENSG00000063177	8649.29125	7552.36681	10370.3728	11734.5716	-0.448319685	5.58E-05	0.0004669	RPL18	ribosomal protein L18 [Source:HGNC Symbol;Acc:HGNC:10310]
ENSG00000005249	257.864623	293.269992	150.66172	117.100869	1.041786907	5.63E-05	0.0004711	PRKAR2B	protein kinase cAMP-dependent type II regulatory subunit beta [Source:HGNC Symbol;Acc:HGNC:9392]
ENSG00000125398	343.491425	365.036969	190.697702	194.813263	0.878355307	5.64E-05	0.0004717	SOX9	SRY-box 9 [Source:HGNC Symbol;Acc:HGNC:11204]
ENSG00000099250	512.776598	501.482827	770.165856	797.350459	-0.628168818	5.67E-05	0.0004734	NRP1	neuropilin 1 [Source:HGNC Symbol;Acc:HGNC:8004]
ENSG00000105248	253.927758	210.870871	407.734865	423.692233	-0.840269843	5.71E-05	0.0004771	YJU2	YJU2 splicing factor homolog [Source:HGNC Symbol;Acc:HGNC:25518]
ENSG00000006025	580.68751	522.747116	306.591333	350.238052	0.748077133	5.77E-05	0.0004813	OSBPL7	oxysterol binding protein like 7 [Source:HGNC Symbol;Acc:HGNC:16387]
ENSG00000104973	797.215056	676.027203	1115.73959	1105.00638	-0.592800321	5.77E-05	0.0004815	MED25	mediator complex subunit 25 [Source:HGNC Symbol;Acc:HGNC:28845]
ENSG00000087510	821.820459	866.519796	494.128299	584.439789	0.646769076	5.78E-05	0.0004817	TFAP2C	transcription factor AP-2 gamma [Source:HGNC Symbol;Acc:HGNC:11744]
ENSG00000012171	3551.05175	3097.49817	4401.85082	4720.22956	-0.456547263	5.79E-05	0.0004828	SEMA3B	semaphorin 3B [Source:HGNC Symbol;Acc:HGNC:10724]
ENSG00000110002	186.016846	176.3164	75.8576493	78.7769479	1.228234933	5.81E-05	0.0004845	VWA5A	von Willebrand factor A domain containing 5A [Source:HGNC Symbol;Acc:HGNC:6658]
ENSG00000119698	52.1634543	46.0726272	6.32147077	7.45187345	2.834158108	5.83E-05	0.0004856	PPP4R4	protein phosphatase 4 regulatory subunit 4 [Source:HGNC Symbol;Acc:HGNC:23788]
ENSG00000136830	11102.942	9494.50525	14107.4156	13617.7665	-0.428831299	5.88E-05	0.0004898	FAM129B	family with sequence similarity 129 member B [Source:HGNC Symbol;Acc:HGNC:25282]
ENSG00000164855	582.655942	455.4102	822.844779	839.932593	-0.680880273	5.89E-05	0.0004901	TMEM184A	transmembrane protein 184A [Source:HGNC Symbol;Acc:HGNC:28797]
ENSG00000077157	741.114737	740.706084	510.985554	399.207506	0.702984427	5.90E-05	0.0004909	PPP1R12B	protein phosphatase 1 regulatory subunit 12B [Source:HGNC Symbol;Acc:HGNC:7619]
ENSG00000163041	5591.33177	5849.45163	7321.31673	7794.65963	-0.401823605	5.92E-05	0.000492	H3F3A	H3 histone family member 3A [Source:HGNC Symbol;Acc:HGNC:4764]
ENSG00000236882	61.0213993	51.3886996	8.4286277	10.6455335	2.558746091	5.97E-05	0.0004961	LINC01554	long intergenic non-protein coding RNA 1554 [Source:HGNC Symbol;Acc:HGNC:24687]
ENSG00000197324	4679.94764	4161.59865	3225.00367	3289.46985	0.44049043	6.06E-05	0.0005031	LRP10	LDL receptor related protein 10 [Source:HGNC Symbol;Acc:HGNC:14553]
ENSG00000189171	947.800122	851.457591	1250.59763	1404.14587	-0.561574676	6.20E-05	0.0005142	S100A13	S100 calcium binding protein A13 [Source:HGNC Symbol;Acc:HGNC:10490]
ENSG00000237649	1473.37153	1319.27196	1955.44163	1979.00468	-0.494904261	6.21E-05	0.000515	KIFC1	kinesin family member C1 [Source:HGNC Symbol;Acc:HGNC:6389]
ENSG00000143924	1046.22173	1259.90915	823.898358	559.955062	0.737009357	6.24E-05	0.0005171	EML4	echinoderm microtubule associated protein like 4 [Source:HGNC Symbol;Acc:HGNC:1316]
ENSG00000160117	309.043861	270.233679	454.092317	551.438635	-0.796386237	6.24E-05	0.0005171	ANKLE1	ankyrin repeat and LEM domain containing 1 [Source:HGNC Symbol;Acc:HGNC:26812]
ENSG00000164091	2924.10609	2863.59098	3902.45462	3833.45661	-0.41863385	6.27E-05	0.000519	WDR82	WD repeat domain 82 [Source:HGNC Symbol;Acc:HGNC:28826]

ENSG00000109062	2332.5922	2052.88995	1486.59921	1609.60467	0.501983991	6.29E-05	0.0005207	SLC9A3R1	SLC9A3 regulator 1 [Source:HGNC Symbol;Acc:HGNC:11075]
ENSG00000101246	789.341327	666.28107	1057.79278	1149.71762	-0.60150358	6.29E-05	0.0005207	ARFRP1	ADP ribosylation factor related protein 1 [Source:HGNC Symbol;Acc:HGNC:662]
ENSG00000146674	2293.22356	2311.60547	2954.23401	3439.57187	-0.473481192	6.32E-05	0.0005227	IGFBP3	insulin like growth factor binding protein 3 [Source:HGNC Symbol;Acc:HGNC:5472]
ENSG00000114316	647.614206	625.524516	932.416939	977.259976	-0.585104422	6.32E-05	0.0005227	USP4	ubiquitin specific peptidase 4 [Source:HGNC Symbol;Acc:HGNC:12627]
ENSG00000145526	26.5738352	19.4922654	0	0	6.88446638	6.34E-05	0.0005239	CDH18	cadherin 18 [Source:HGNC Symbol;Acc:HGNC:1757]
ENSG00000128573	27.5580513	60.2488202	5.26789231	3.19366005	3.376958921	6.36E-05	0.0005254	FOXP2	forkhead box P2 [Source:HGNC Symbol;Acc:HGNC:13875]
ENSG00000160145	88.5794506	93.0312665	30.5537754	18.097407	1.899843958	6.38E-05	0.0005266	KALRN	kalirin RhoGEF kinase [Source:HGNC Symbol;Acc:HGNC:4814]
ENSG00000101189	1167.28032	1078.27668	1526.63519	1707.54357	-0.526589916	6.38E-05	0.0005266	MRGBP	MRG domain binding protein [Source:HGNC Symbol;Acc:HGNC:15866]
ENSG00000115541	529.508272	628.182552	1035.66763	822.89974	-0.682279501	6.39E-05	0.0005266	HSPE1	heat shock protein family E (Hsp10) member 1 [Source:HGNC Symbol;Acc:HGNC:5269]
ENSG00000130119	664.34588	614.006359	941.899145	980.453636	-0.588982914	6.43E-05	0.0005304	GNL3L	G protein nucleolar 3 like [Source:HGNC Symbol;Acc:HGNC:25553]
ENSG00000213694	783.43603	773.48853	504.664083	505.662841	0.623826392	6.48E-05	0.0005337	S1PR3	sphingosine-1-phosphate receptor 3 [Source:HGNC Symbol;Acc:HGNC:3167]
ENSG00000170214	234.243436	155.938123	83.2326985	76.6478412	1.28547317	6.52E-05	0.0005365	ADRA1B	adrenoceptor alpha 1B [Source:HGNC Symbol;Acc:HGNC:278]
ENSG00000033100	3576.64137	3212.67974	2528.58831	2437.82717	0.450867944	6.52E-05	0.0005369	CHPF2	chondroitin polymerizing factor 2 [Source:HGNC Symbol;Acc:HGNC:29270]
ENSG00000219607	358.254667	334.026547	536.271437	605.730856	-0.722523967	6.53E-05	0.0005372	PPP1R3G	protein phosphatase 1 regulatory subunit 3G [Source:HGNC Symbol;Acc:HGNC:14945]
ENSG00000019549	335.617696	391.617331	203.340643	191.619603	0.881346991	6.53E-05	0.0005372	SNAI2	snail family transcriptional repressor 2 [Source:HGNC Symbol;Acc:HGNC:11094]
ENSG00000135077	35.4317803	34.5544704	2.10715692	2.1291067	4.046129529	6.57E-05	0.0005403	HAVCR2	hepatitis A virus cellular receptor 2 [Source:HGNC Symbol;Acc:HGNC:18437]
ENSG00000204498	247.038246	164.798243	375.073933	419.43402	-0.950388927	6.63E-05	0.000545	NFKBIL1	NFKB inhibitor like 1 [Source:HGNC Symbol;Acc:HGNC:7800]
ENSG00000228594	402.544392	363.264945	605.807616	630.215583	-0.691272568	6.64E-05	0.0005454	FNDC10	fibronectin type III domain containing 10 [Source:HGNC Symbol;Acc:HGNC:42951]
ENSG00000270885	56.1003187	52.2747116	141.179514	141.585596	-1.384306168	6.65E-05	0.0005456	RASL10B	RAS like family 10 member B [Source:HGNC Symbol;Acc:HGNC:30295]
ENSG00000218891	385.812718	320.736366	583.682468	585.504343	-0.727768707	6.66E-05	0.0005464	ZNF579	zinc finger protein 579 [Source:HGNC Symbol;Acc:HGNC:26646]
ENSG00000174780	1357.23403	1742.78573	2599.17807	2100.36376	-0.599757094	6.70E-05	0.0005489	SRP72	signal recognition particle 72 [Source:HGNC Symbol;Acc:HGNC:11303]
ENSG00000166685	404.512825	380.099175	630.03992	624.892817	-0.677957325	6.70E-05	0.0005489	COG1	component of oligomeric golgi complex 1 [Source:HGNC Symbol;Acc:HGNC:6545]
ENSG00000070081	993.074063	1277.62939	787.023111	632.34469	0.678380589	6.73E-05	0.0005514	NUCB2	nucleobindin 2 [Source:HGNC Symbol;Acc:HGNC:8044]
ENSG00000145907	3073.70694	3211.79372	4288.06434	4111.30504	-0.418167862	6.82E-05	0.0005586	G3BP1	G3BP stress granule assembly factor 1 [Source:HGNC Symbol;Acc:HGNC:30292]
ENSG00000204876	42.3212931	58.4767961	7.37504924	7.45187345	2.766516979	6.89E-05	0.0005641	AC021218.1	uncharacterized LOC389602 [Source:NCBI gene;Acc:389602]
ENSG00000136237	95.4689635	154.166099	33.7145108	45.7757941	1.653111747	6.92E-05	0.000566	RAPGEF5	Rap guanine nucleotide exchange factor 5 [Source:HGNC Symbol;Acc:HGNC:16862]
ENSG00000126254	1809.97344	1546.97706	2277.83664	2486.79663	-0.505618249	6.94E-05	0.0005672	RBM42	RNA binding motif protein 42 [Source:HGNC Symbol;Acc:HGNC:28117]
ENSG00000104852	5037.21809	4157.16859	6138.14812	6582.13336	-0.468519569	6.97E-05	0.0005692	SNRNP70	small nuclear ribonucleoprotein U1 subunit 70 [Source:HGNC Symbol;Acc:HGNC:11150]
ENSG00000221887	91.532099	67.3369167	18.9644123	20.2265137	2.018009126	6.98E-05	0.0005702	HMSD	histocompatibility minor serpin domain containing [Source:HGNC Symbol;Acc:HGNC:23037]
ENSG00000143147	1292.27576	1181.94009	846.023505	849.513574	0.544939916	6.99E-05	0.0005702	GPR161	G protein-coupled receptor 161 [Source:HGNC Symbol;Acc:HGNC:23694]
ENSG00000100647	607.261345	543.125394	339.252265	360.883586	0.715993138	6.99E-05	0.0005702	SUSD6	sushi domain containing 6 [Source:HGNC Symbol;Acc:HGNC:19956]
ENSG00000197894	1580.65109	1796.83246	2335.78345	2381.40584	-0.481596219	7.00E-05	0.0005713	ADH5	alcohol dehydrogenase 5 (class III), chi polypeptide [Source:HGNC Symbol;Acc:HGNC:253]
ENSG00000148187	1026.53741	1148.27163	1478.17058	1691.57527	-0.543038239	7.01E-05	0.0005717	MRRF	mitochondrial ribosome recycling factor [Source:HGNC Symbol;Acc:HGNC:7234]
ENSG00000096060	911.384125	1081.82073	636.361391	667.474951	0.612900175	7.02E-05	0.0005719	FKBP5	FK506 binding protein 5 [Source:HGNC Symbol;Acc:HGNC:3721]
ENSG00000129932	397.623312	279.979812	586.843204	591.891663	-0.800545201	7.03E-05	0.0005728	DOHH	deoxyhypusine hydroxylase [Source:HGNC Symbol;Acc:HGNC:28662]
ENSG00000100650	2582.58309	2530.45045	3247.12882	3868.58687	-0.47684187	7.05E-05	0.0005735	SRSF5	serine and arginine rich splicing factor 5 [Source:HGNC Symbol;Acc:HGNC:10787]
ENSG00000182704	2354.24495	2015.67744	3071.18122	3000.97589	-0.474969727	7.11E-05	0.0005783	TSKU	tsukushi, small leucine rich proteoglycan [Source:HGNC Symbol;Acc:HGNC:28850]
ENSG00000100292	2624.90439	2375.39834	3297.70059	3534.31712	-0.450510669	7.19E-05	0.0005849	HMOX1	heme oxygenase 1 [Source:HGNC Symbol;Acc:HGNC:5013]
ENSG00000137563	1574.74579	1822.52681	1229.52607	1114.58736	0.535652129	7.20E-05	0.0005849	GGH	gamma-glutamyl hydrolase [Source:HGNC Symbol;Acc:HGNC:4248]
ENSG00000135378	333.649264	325.166427	161.197505	189.490496	0.909705174	7.23E-05	0.0005871	PRRG4	proline rich and Gla domain 4 [Source:HGNC Symbol;Acc:HGNC:30799]
ENSG00000176170	971.421309	846.141519	598.432567	589.762556	0.61274008	7.26E-05	0.0005896	SPHK1	sphingosine kinase 1 [Source:HGNC Symbol;Acc:HGNC:11240]
ENSG00000125835	5851.16482	5216.83902	7120.08325	7761.65848	-0.427271463	7.29E-05	0.0005916	SNRPB	small nuclear ribonucleoprotein polypeptides B and B1 [Source:HGNC Symbol;Acc:HGNC:11153]
ENSG00000111670	2045.20109	2246.04058	1620.40368	1382.8548	0.515009719	7.30E-05	0.000592	GNPTAB	N-acetylglucosamine-1-phosphate transferase subunits alpha and beta [Source:HGNC Symbol;Acc:HGNC:29670]
ENSG00000168939	115.153286	104.549423	35.8216677	36.1948139	1.608711925	7.33E-05	0.0005946	SPRY3	sprouty RTK signaling antagonist 3 [Source:HGNC Symbol;Acc:HGNC:11271]
ENSG00000187479	30.5106997	37.2125066	1.05357846	1.06455335	4.999552279	7.35E-05	0.0005954	C11orf96	chromosome 11 open reading frame 96 [Source:HGNC Symbol;Acc:HGNC:38675]
ENSG00000159202	2665.25725	2485.26383	3379.87971	3592.86756	-0.437160669	7.36E-05	0.0005958	UBE2Z	ubiquitin conjugating enzyme E2 Z [Source:HGNC Symbol;Acc:HGNC:25847]
ENSG00000196950	530.492488	588.312009	1096.77518	763.284752	-0.733093993	7.38E-05	0.0005978	SLC39A10	solute carrier family 39 member 10 [Source:HGNC Symbol;Acc:HGNC:20861]
ENSG00000163297	413.37077	360.606909	236.001576	178.844963	0.898978668	7.44E-05	0.000602	ANTXR2	ANTXR cell adhesion molecule 2 [Source:HGNC Symbol;Acc:HGNC:21732]
ENSG00000109919	2302.0815	2229.20635	2937.37675	3253.27504	-0.45022967	7.44E-05	0.000602	MTCH2	mitochondrial carrier 2 [Source:HGNC Symbol;Acc:HGNC:17587]
ENSG00000086589	706.667173	764.628409	1027.239	1177.39601	-0.583046067	7.45E-05	0.000602	RBM22	RNA binding motif protein 22 [Source:HGNC Symbol;Acc:HGNC:25503]
ENSG00000115604	69.8793444	84.1711459	18.9644123	19.1619603	2.01538573	7.48E-05	0.0006041	IL18R1	interleukin 18 receptor 1 [Source:HGNC Symbol;Acc:HGNC:5988]
ENSG00000106780	2638.68341	3034.59131	2134.54996	1900.22773	0.49190009	7.55E-05	0.0006095	MEGF9	multiple EGF like domains 9 [Source:HGNC Symbol;Acc:HGNC:3234]
ENSG00000172590	849.37851	645.902793	1089.40013	1256.17295	-0.65046657	7.63E-05	0.0006157	MRPL52	mitochondrial ribosomal protein L52 [Source:HGNC Symbol;Acc:HGNC:16655]

ENSG00000138316	68.8951283	46.0726272	10.5357846	9.58098015	2.51296079	7.64E-05	0.0006164	ADAMTS14	ADAM metallopeptidase with thrombospondin type 1 motif 14 [Source:HGNC Symbol;Acc:HGNC:14899]
ENSG00000084652	2218.42313	2130.85901	2872.05489	3024.39607	-0.439167916	7.65E-05	0.0006169	TXLNA	taxilin alpha [Source:HGNC Symbol;Acc:HGNC:30685]
ENSG00000145362	140.742905	135.559845	52.6789231	52.1631142	1.397840563	7.69E-05	0.0006201	ANK2	ankyrin 2 [Source:HGNC Symbol;Acc:HGNC:493]
ENSG00000104213	307.075429	334.912559	142.233092	188.425943	0.957786705	7.71E-05	0.0006209	PDGFRL	platelet derived growth factor receptor like [Source:HGNC Symbol;Acc:HGNC:8805]
ENSG00000141524	776.546517	713.23971	495.181877	458.822494	0.642658612	7.73E-05	0.0006229	TMC6	transmembrane channel like 6 [Source:HGNC Symbol;Acc:HGNC:18021]
ENSG00000130723	7369.81029	6695.59315	10044.8171	8867.72941	-0.427287876	7.75E-05	0.0006237	PRRC2B	proline rich coiled-coil 2B [Source:HGNC Symbol;Acc:HGNC:28121]
ENSG00000160471	56.1003187	48.7306634	7.37504924	9.58098015	2.628051628	7.78E-05	0.0006263	COX6B2	cytochrome c oxidase subunit 6B2 [Source:HGNC Symbol;Acc:HGNC:24380]
ENSG00000145041	1208.61739	1154.47372	1708.90427	1620.2502	-0.494678116	7.80E-05	0.0006276	DCAF1	DDB1 and CUL4 associated factor 1 [Source:HGNC Symbol;Acc:HGNC:30911]
ENSG00000172586	932.052664	901.960279	1248.49048	1426.50149	-0.544624244	7.81E-05	0.0006276	CHCHD1	coiled-coil-helix-coiled-coil-helix domain containing 1 [Source:HGNC Symbol;Acc:HGNC:23518]
ENSG00000112893	1014.72682	1251.93504	804.933945	615.311836	0.674802156	7.85E-05	0.0006304	MAN2A1	mannosidase alpha class 2A member 1 [Source:HGNC Symbol;Acc:HGNC:6824]
ENSG00000050555	1197.79102	1077.39067	1681.51123	1568.08708	-0.514700334	7.88E-05	0.0006325	LAMC3	laminin subunit gamma 3 [Source:HGNC Symbol;Acc:HGNC:6494]
ENSG00000119616	824.773107	939.172785	1256.91911	1325.36892	-0.549273455	7.90E-05	0.0006338	FCF1	FCF1, rRNA-processing protein [Source:HGNC Symbol;Acc:HGNC:20220]
ENSG00000159140	5027.37593	5137.98395	7255.99487	6366.02903	-0.422266187	7.93E-05	0.0006362	SON	SON DNA binding protein [Source:HGNC Symbol;Acc:HGNC:11183]
ENSG00000115112	62.0056154	60.2488202	11.5893631	12.7746402	2.32709103	7.95E-05	0.0006373	TFCP2L1	transcription factor CP2 like 1 [Source:HGNC Symbol;Acc:HGNC:17925]
ENSG00000137962	1087.55881	1305.09577	849.184241	713.250745	0.615214481	7.95E-05	0.0006376	ARHGAP29	Rho GTPase activating protein 29 [Source:HGNC Symbol;Acc:HGNC:30207]
ENSG00000123739	419.276066	488.192646	273.9304	249.105484	0.795506441	8.02E-05	0.0006427	PLA2G12A	phospholipase A2 group XIIA [Source:HGNC Symbol;Acc:HGNC:18554]
ENSG00000087269	1011.77417	929.426653	1372.81274	1412.6623	-0.52132864	8.05E-05	0.0006445	NOP14	NOP14 nucleolar protein [Source:HGNC Symbol;Acc:HGNC:16821]
ENSG00000139278	161.411443	199.352714	79.0183847	74.5187345	1.233375197	8.06E-05	0.0006451	GLIPR1	GLI pathogenesis related 1 [Source:HGNC Symbol;Acc:HGNC:17001]
ENSG00000117394	3925.05388	3373.93393	4856.99671	5086.43591	-0.446278843	8.09E-05	0.0006474	SLC2A1	solute carrier family 2 member 1 [Source:HGNC Symbol;Acc:HGNC:11005]
ENSG00000135387	3955.56458	4505.37133	6397.32842	5369.6071	-0.475711455	8.12E-05	0.0006493	CAPRIN1	cell cycle associated protein 1 [Source:HGNC Symbol;Acc:HGNC:6743]
ENSG00000104738	10273.2478	10635.6888	14327.6135	13054.6177	-0.389101087	8.14E-05	0.0006504	MCM4	minichromosome maintenance complex component 4 [Source:HGNC Symbol;Acc:HGNC:6947]
ENSG00000136159	154.521931	213.528907	368.752462	336.398859	-0.935879455	8.14E-05	0.0006504	NUDT15	nudix hydrolase 15 [Source:HGNC Symbol;Acc:HGNC:23063]
ENSG00000141522	3432.94582	3024.84518	4339.68969	4394.47623	-0.435862039	8.20E-05	0.0006551	ARHGDIA	Rho GDP dissociation inhibitor alpha [Source:HGNC Symbol;Acc:HGNC:678]
ENSG00000128944	1080.6693	1171.30795	1498.18857	1780.99775	-0.541824112	8.25E-05	0.0006588	KNSTRN	kinetochore localized astrin (SPAG5) binding protein [Source:HGNC Symbol;Acc:HGNC:30767]
ENSG00000175197	102.358476	93.9172785	200.179908	222.49165	-1.10737122	8.26E-05	0.0006593	DDIT3	DNA damage inducible transcript 3 [Source:HGNC Symbol;Acc:HGNC:2726]
ENSG00000197580	165.348308	179.860449	63.2147077	80.9060546	1.260826716	8.28E-05	0.00066	BCO2	beta-carotene oxygenase 2 [Source:HGNC Symbol;Acc:HGNC:18503]
ENSG00000146425	2161.3386	2182.24771	1402.31293	1663.89689	0.502505912	8.28E-05	0.0006602	DYNLT1	dynein light chain Tctex-type 1 [Source:HGNC Symbol;Acc:HGNC:11697]
ENSG00000065361	278.533161	285.295884	156.983191	129.875509	0.974861539	8.29E-05	0.0006603	ERBB3	erb-b2 receptor tyrosine kinase 3 [Source:HGNC Symbol;Acc:HGNC:3431]
ENSG00000198492	1621.98816	1822.52681	2315.76546	2482.53841	-0.477872059	8.33E-05	0.0006633	YTHDF2	YTH N6-methyladenosine RNA binding protein 2 [Source:HGNC Symbol;Acc:HGNC:31675]
ENSG00000187608	1626.90924	1461.03389	2071.33526	2257.91766	-0.487781715	8.34E-05	0.0006635	ISG15	ISG15 ubiquitin-like modifier [Source:HGNC Symbol;Acc:HGNC:4053]
ENSG00000115839	1158.42237	1201.43236	1638.31451	1671.34876	-0.487833368	8.36E-05	0.0006648	RAB3GAP1	RAB3 GTPase activating protein catalytic subunit 1 [Source:HGNC Symbol;Acc:HGNC:17063]
ENSG00000105552	933.03688	836.395386	1222.15102	1378.59659	-0.556051344	8.37E-05	0.0006654	BCAT2	branched chain amino acid transaminase 2 [Source:HGNC Symbol;Acc:HGNC:977]
ENSG00000081791	808.041433	723.871854	1092.56087	1169.94413	-0.563070572	8.40E-05	0.000668	DELE1	DAP3 binding cell death enhancer 1 [Source:HGNC Symbol;Acc:HGNC:28969]
ENSG00000100304	3338.46107	2894.60141	4210.09954	4269.92349	-0.444387676	8.42E-05	0.0006691	TTLL12	tubulin tyrosine ligase like 12 [Source:HGNC Symbol;Acc:HGNC:28974]
ENSG00000135821	1481.24526	1470.78002	1058.84635	1026.22943	0.501568048	8.47E-05	0.0006727	GLUL	glutamate-ammonia ligase [Source:HGNC Symbol;Acc:HGNC:4341]
ENSG00000133321	7.87372895	9.74613268	52.6789231	51.0985608	-2.554770215	8.50E-05	0.0006748	RARRES3	retinoic acid receptor responder 3 [Source:HGNC Symbol;Acc:HGNC:9869]
ENSG00000149480	3207.56033	2855.61688	4030.9912	4164.53271	-0.434986208	8.56E-05	0.0006787	MTA2	metastasis associated 1 family member 2 [Source:HGNC Symbol;Acc:HGNC:7411]
ENSG00000127564	1067.87449	892.214146	1424.43808	1442.46979	-0.549254919	8.59E-05	0.0006815	PKMYT1	protein kinase, membrane associated tyrosine/threonine 1 [Source:HGNC Symbol;Acc:HGNC:29650]
ENSG00000152642	807.057217	815.131097	564.718056	482.242668	0.631677509	8.61E-05	0.0006827	GPD1L	glycerol-3-phosphate dehydrogenase 1 like [Source:HGNC Symbol;Acc:HGNC:28956]
ENSG00000113048	1924.14251	2096.30454	2703.48233	2783.80701	-0.448491916	8.64E-05	0.0006845	MRPS27	mitochondrial ribosomal protein S27 [Source:HGNC Symbol;Acc:HGNC:14512]
ENSG00000137656	318.886022	329.596487	480.431779	624.892817	-0.76903583	8.67E-05	0.0006867	BUD13	BUD13 homolog [Source:HGNC Symbol;Acc:HGNC:28199]
ENSG00000122406	11963.1469	12845.4029	15371.7098	17725.8778	-0.415844826	8.71E-05	0.0006895	RPL5	ribosomal protein L5 [Source:HGNC Symbol;Acc:HGNC:10360]
ENSG00000198535	41.337077	28.352386	1.05357846	3.19366005	4.037472738	8.72E-05	0.00069	C2CD4A	C2 calcium dependent domain containing 4A [Source:HGNC Symbol;Acc:HGNC:33627]
ENSG00000138378	51.1792381	86.8291821	16.8572554	11.7100869	2.273813878	8.74E-05	0.0006908	STAT4	signal transducer and activator of transcription 4 [Source:HGNC Symbol;Acc:HGNC:11365]
ENSG00000144711	1264.71771	1149.15764	855.505711	792.027693	0.550700189	8.75E-05	0.0006914	IQSEC1	IQ motif and Sec7 domain 1 [Source:HGNC Symbol;Acc:HGNC:29112]
ENSG00000140525	2806.00015	2959.28029	3886.65095	3804.71367	-0.415732504	8.80E-05	0.0006951	FANCI	FA complementation group I [Source:HGNC Symbol;Acc:HGNC:25568]
ENSG00000215246	40.3528608	46.0726272	7.37504924	2.1291067	3.183125741	8.87E-05	0.0007001	AC116351.1	uncharacterized LOC100506688 [Source:NCBI gene;Acc:100506688]
ENSG00000142552	94.4847473	69.9949529	15.8036769	25.5492804	1.991239991	8.92E-05	0.0007043	RCN3	reticulocalbin 3 [Source:HGNC Symbol;Acc:HGNC:21145]
ENSG00000214900	174.206253	162.140207	76.9112277	63.873201	1.255929897	9.09E-05	0.0007173	LINC01588	long intergenic non-protein coding RNA 1588 [Source:HGNC Symbol;Acc:HGNC:27503]

ENSG00000005187	47.2423737	23.9223257	1.05357846	3.19366005	4.066734677	9.10E-05	0.0007179	ACSM3	acyl-CoA synthetase medium chain family member 3 [Source:HGNC Symbol;Acc:HGNC:10522]
ENSG00000204335	129.916528	128.471749	263.394616	249.105484	-0.988136697	9.12E-05	0.0007185	SP5	Sp5 transcription factor [Source:HGNC Symbol;Acc:HGNC:14529]
ENSG00000173638	744.067385	601.60219	978.774391	1090.10263	-0.621395689	9.17E-05	0.0007226	SLC19A1	solute carrier family 19 member 1 [Source:HGNC Symbol;Acc:HGNC:10937]
ENSG00000073737	37.4002125	43.414591	1.05357846	0	6.254654917	9.19E-05	0.0007234	DHRS9	dehydrogenase/reductase 9 [Source:HGNC Symbol;Acc:HGNC:16888]
ENSG00000158195	3281.37654	3291.53481	4430.29743	4265.66527	-0.403813564	9.21E-05	0.0007245	WASF2	WAS protein family member 2 [Source:HGNC Symbol;Acc:HGNC:12733]
ENSG00000082074	95.4689635	116.06758	36.8752462	30.8720472	1.643340225	9.21E-05	0.0007245	FYB1	FYN binding protein 1 [Source:HGNC Symbol;Acc:HGNC:4036]
ENSG00000169116	23.6211868	19.4922654	0	0	6.789296452	9.25E-05	0.0007272	PARM1	prostate androgen-regulated mucin-like protein 1 [Source:HGNC Symbol;Acc:HGNC:24536]
ENSG00000115484	4833.48536	4610.80677	5946.39684	6639.61925	-0.414359601	9.35E-05	0.0007347	CCT4	chaperonin containing TCP1 subunit 4 [Source:HGNC Symbol;Acc:HGNC:1617]
ENSG00000137135	187.985279	179.860449	304.484176	382.174653	-0.900707068	9.37E-05	0.0007364	ARHGEF39	Rho guanine nucleotide exchange factor 39 [Source:HGNC Symbol;Acc:HGNC:25909]
ENSG00000142731	327.743967	404.0215	693.254628	547.180422	-0.760395638	9.38E-05	0.0007367	PLK4	polo like kinase 4 [Source:HGNC Symbol;Acc:HGNC:11397]
ENSG00000165280	6993.83974	6467.88805	8684.64726	8933.73172	-0.388301375	9.42E-05	0.0007396	VCP	valosin containing protein [Source:HGNC Symbol;Acc:HGNC:12666]
ENSG00000117650	837.567917	847.913543	1187.38293	1242.33376	-0.527552342	9.43E-05	0.0007396	NEK2	NIMA related kinase 2 [Source:HGNC Symbol;Acc:HGNC:7745]
ENSG00000101311	580.68751	569.705756	907.131056	830.351613	-0.595011786	9.45E-05	0.0007408	FERMT1	fermitin family member 1 [Source:HGNC Symbol;Acc:HGNC:15889]
ENSG00000100023	588.561239	535.151285	826.005514	880.385621	-0.603161149	9.68E-05	0.000759	PPIL2	peptidylprolyl isomerase like 2 [Source:HGNC Symbol;Acc:HGNC:9261]
ENSG00000136451	732.256792	720.327806	1148.40052	1004.93836	-0.568079202	9.80E-05	0.0007671	VEZF1	vascular endothelial zinc finger 1 [Source:HGNC Symbol;Acc:HGNC:12949]
ENSG00000111674	3630.77326	3093.06811	2259.9258	2547.47617	0.483824515	9.80E-05	0.0007671	ENO2	enolase 2 [Source:HGNC Symbol;Acc:HGNC:3353]
ENSG00000177707	219.480194	279.979812	126.429415	114.971762	1.049912686	9.80E-05	0.0007671	NECTIN3	nectin cell adhesion molecule 3 [Source:HGNC Symbol;Acc:HGNC:17664]
ENSG00000214046	298.217484	287.95392	476.217465	497.146415	-0.731877831	9.81E-05	0.0007675	SMIM7	small integral membrane protein 7 [Source:HGNC Symbol;Acc:HGNC:28419]
ENSG00000169894	39.3686447	28.352386	2.10715692	2.1291067	3.99749807	9.84E-05	0.0007693	MUC3A	mucin 3A, cell surface associated [Source:HGNC Symbol;Acc:HGNC:7513]
ENSG00000132382	1963.51116	1482.29818	2481.17728	2562.37991	-0.550188804	9.85E-05	0.0007698	MYBBP1A	MYB binding protein 1a [Source:HGNC Symbol;Acc:HGNC:7546]
ENSG00000276293	1437.93975	1453.94579	2008.12055	1969.4237	-0.459833292	9.85E-05	0.0007699	PIP4K2B	phosphatidylinositol-5-phosphate 4-kinase type 2 beta [Source:HGNC Symbol;Acc:HGNC:8998]
ENSG00000216775	147.632418	171.88634	72.6969139	56.4213276	1.307635045	9.87E-05	0.0007708	AL109918.1	uncharacterized LOC730101 [Source:NCBI gene;Acc:730101]
ENSG00000164051	576.750645	543.125394	832.326985	856.965447	-0.593401655	9.94E-05	0.0007762	CCDC51	coiled-coil domain containing 51 [Source:HGNC Symbol;Acc:HGNC:25714]
ENSG00000119318	4414.20929	4538.15378	6168.7019	5662.35927	-0.402207723	9.98E-05	0.0007789	RAD23B	RAD23 homolog B, nucleotide excision repair protein [Source:HGNC Symbol;Acc:HGNC:9813]
ENSG00000003756	869.062832	828.421278	1182.11503	1272.14125	-0.532080719	0.0001001	0.0007803	RBM5	RNA binding motif protein 5 [Source:HGNC Symbol;Acc:HGNC:9902]
ENSG00000064687	1782.41539	1516.85265	1089.40013	1191.2352	0.532351641	0.0001001	0.0007803	ABCA7	ATP binding cassette subfamily A member 7 [Source:HGNC Symbol;Acc:HGNC:37]
ENSG00000137309	6091.31356	5554.40962	7677.42625	7625.39565	-0.394111652	0.0001009	0.0007863	HMGA1	high mobility group AT-hook 1 [Source:HGNC Symbol;Acc:HGNC:5010]
ENSG00000165868	482.265898	538.695334	309.752068	306.591365	0.728591209	0.000101	0.0007863	HSPA12A	heat shock protein family A (Hsp70) member 12A [Source:HGNC Symbol;Acc:HGNC:19022]
ENSG00000110200	124.995447	128.471749	268.662508	237.395397	-0.997337217	0.000101	0.0007863	ANAPC15	anaphase promoting complex subunit 15 [Source:HGNC Symbol;Acc:HGNC:24531]
ENSG00000100242	1931.03202	1701.14316	2643.42836	2412.27789	-0.477437409	0.0001012	0.0007872	SUN2	Sad1 and UNC84 domain containing 2 [Source:HGNC Symbol;Acc:HGNC:14210]
ENSG00000138821	2640.65185	2745.75138	2103.99619	1624.50841	0.530722784	0.0001012	0.0007872	SLC39A8	solute carrier family 39 member 8 [Source:HGNC Symbol;Acc:HGNC:20862]
ENSG00000171792	1219.44377	1218.26658	1619.3501	1820.38623	-0.496740285	0.0001022	0.0007941	RHNO1	RAD9-HUS1-RAD1 interacting nuclear orphan 1 [Source:HGNC Symbol;Acc:HGNC:28206]
ENSG00000141959	3525.46214	2843.21271	4352.33263	4521.15808	-0.478829383	0.0001023	0.0007944	PFKL	phosphofructokinase, liver type [Source:HGNC Symbol;Acc:HGNC:8876]
ENSG00000171241	926.147367	954.234991	1344.36612	1330.69169	-0.508412747	0.0001025	0.0007956	SHCBP1	SHC binding and spindle associated 1 [Source:HGNC Symbol;Acc:HGNC:29547]
ENSG00000088367	1010.78995	1081.82073	1461.31333	1501.02022	-0.501137029	0.0001025	0.0007956	EPB41L1	erythrocyte membrane protein band 4.1 like 1 [Source:HGNC Symbol;Acc:HGNC:3378]
ENSG00000269335	223.417059	185.176521	372.966776	363.012692	-0.850373228	0.0001026	0.0007957	IKBKG	inhibitor of nuclear factor kappa B kinase subunit gamma [Source:HGNC Symbol;Acc:HGNC:5961]
ENSG00000105576	1531.44028	1290.03356	1965.97741	2037.55511	-0.505340168	0.0001026	0.0007957	TNPO2	transportin 2 [Source:HGNC Symbol;Acc:HGNC:19998]
ENSG00000133985	239.164517	270.233679	107.465003	140.521042	1.039295977	0.0001028	0.000797	TTC9	tetratricopeptide repeat domain 9 [Source:HGNC Symbol;Acc:HGNC:20267]
ENSG00000139842	1900.52132	1970.49083	2719.28601	2542.1534	-0.442657843	0.0001032	0.0007995	CUL4A	cullin 4A [Source:HGNC Symbol;Acc:HGNC:2554]
ENSG00000051596	1259.79663	1420.27734	1885.90545	1871.48479	-0.48704594	0.0001041	0.0008065	THOC3	THO complex 3 [Source:HGNC Symbol;Acc:HGNC:19072]
ENSG00000268223	171.253605	171.88634	75.8576493	72.3896278	1.210760215	0.0001049	0.0008117	ARL14EPL	ADP ribosylation factor like GTPase 14 effector protein like [Source:HGNC Symbol;Acc:HGNC:44201]
ENSG00000198478	50.195022	52.2747116	11.5893631	3.19366005	2.791382495	0.0001051	0.000813	SH3BGRL2	SH3 domain binding glutamate rich protein like 2 [Source:HGNC Symbol;Acc:HGNC:15567]
ENSG00000176049	45.2739414	57.590784	9.48220616	7.45187345	2.603339522	0.0001056	0.000816	JAKMIP2	janus kinase and microtubule interacting protein 2 [Source:HGNC Symbol;Acc:HGNC:29067]
ENSG00000127124	319.870238	319.850354	169.626132	177.780409	0.880857009	0.0001056	0.000816	HIVEP3	human immunodeficiency virus type I enhancer binding protein 3 [Source:HGNC Symbol;Acc:HGNC:13561]
ENSG00000203666	104.326909	116.953592	212.822849	246.976377	-1.054078099	0.0001068	0.0008253	EFCAB2	EF-hand calcium binding domain 2 [Source:HGNC Symbol;Acc:HGNC:28166]
ENSG00000176108	116.137502	108.093472	218.090742	242.718164	-1.039748704	0.0001072	0.0008276	CHMP6	charged multivesicular body protein 6 [Source:HGNC Symbol;Acc:HGNC:25675]
ENSG00000167770	1771.58901	1531.02884	2309.44399	2288.7897	-0.477895932	0.0001081	0.0008344	OTUB1	OTU deubiquitinase, ubiquitin aldehyde binding 1 [Source:HGNC Symbol;Acc:HGNC:23077]

ENSG00000232573	813.94673	587.425997	1025.13184	1215.71993	-0.678259958	0.0001084	0.0008367	RPL3P4	ribosomal protein L3 pseudogene 4 [Source:HGNC Symbol;Acc:HGNC:19805]
ENSG00000116221	3134.72834	3085.98001	4004.65174	4243.30965	-0.406981727	0.0001088	0.0008392	MRPL37	mitochondrial ribosomal protein L37 [Source:HGNC Symbol;Acc:HGNC:14034]
ENSG00000164418	318.886022	332.254523	180.161917	175.651303	0.872022289	0.0001089	0.0008393	GRIK2	glutamate ionotropic receptor kainate type subunit 2 [Source:HGNC Symbol;Acc:HGNC:4580]
ENSG00000165138	739.146305	635.270648	445.66369	422.62768	0.66195872	0.000109	0.0008396	ANKS6	ankyrin repeat and sterile alpha motif domain containing 6 [Source:HGNC Symbol;Acc:HGNC:26724]
ENSG00000105486	1022.60055	856.773664	1360.16979	1380.7257	-0.545080034	0.0001098	0.0008453	LIG1	DNA ligase 1 [Source:HGNC Symbol;Acc:HGNC:6598]
ENSG00000185164	2326.6869	2006.81732	1639.36809	1356.24097	0.532361456	0.0001102	0.0008487	NOMO2	NODAL modulator 2 [Source:HGNC Symbol;Acc:HGNC:22652]
ENSG00000244313	1727.29929	1555.83718	2189.33604	2360.11478	-0.470913322	0.0001104	0.0008496	AC024293.1	ribosomal protein S10 (RPS10) pseudogene
ENSG00000112992	1529.47185	1460.14788	2080.81746	2023.71592	-0.457426369	0.0001109	0.0008532	NNT	nicotinamide nucleotide transhydrogenase [Source:HGNC Symbol;Acc:HGNC:7863]
ENSG00000107223	5557.86842	4272.35016	6412.07852	7819.14436	-0.533957409	0.0001112	0.0008546	EDF1	endothelial differentiation related factor 1 [Source:HGNC Symbol;Acc:HGNC:3164]
ENSG00000196116	519.66611	556.415575	317.127117	342.786179	0.705788728	0.0001112	0.0008546	TDRD7	tudor domain containing 7 [Source:HGNC Symbol;Acc:HGNC:30831]
ENSG00000167323	1885.75808	1626.71815	1255.86553	1215.71993	0.506671653	0.0001113	0.0008546	STIM1	stromal interaction molecule 1 [Source:HGNC Symbol;Acc:HGNC:11386]
ENSG00000110063	506.871301	431.487874	697.468942	779.253052	-0.655004376	0.0001113	0.0008546	DCPS	decapping enzyme, scavenger [Source:HGNC Symbol;Acc:HGNC:29812]
ENSG00000197785	1141.6907	971.06922	1451.83112	1618.12109	-0.539629334	0.000112	0.0008595	ATAD3A	ATPase family, AAA domain containing 3A [Source:HGNC Symbol;Acc:HGNC:25567]
ENSG00000277791	3444.75641	3195.84551	4256.45699	4627.61341	-0.420033847	0.000113	0.0008664	PSMB3	proteasome subunit beta 3 [Source:HGNC Symbol;Acc:HGNC:9540]
ENSG00000185634	43.3055092	45.1866152	1.05357846	8.5164268	3.212090095	0.000113	0.0008664	SHC4	SHC adaptor protein 4 [Source:HGNC Symbol;Acc:HGNC:16743]
ENSG00000198959	2490.06678	2050.23191	1668.86828	1499.95567	0.518455045	0.0001131	0.0008664	TGM2	transglutaminase 2 [Source:HGNC Symbol;Acc:HGNC:11778]
ENSG00000116675	436.991956	505.026875	279.198293	278.912978	0.755821713	0.0001137	0.0008706	DNAJC6	DnaJ heat shock protein family (Hsp40) member C6 [Source:HGNC Symbol;Acc:HGNC:15469]
ENSG00000261115	30.5106997	47.8446513	0	1.06455335	6.210302975	0.0001139	0.0008722	TMEM178B	transmembrane protein 178B [Source:HGNC Symbol;Acc:HGNC:44112]
ENSG00000090776	972.405525	825.763242	615.289822	556.761402	0.61689911	0.0001142	0.0008736	EFNB1	ephrin B1 [Source:HGNC Symbol;Acc:HGNC:3226]
ENSG00000165861	513.760814	467.814369	301.32344	289.558511	0.731788222	0.0001142	0.0008739	ZFYVE1	zinc finger FYVE-type containing 1 [Source:HGNC Symbol;Acc:HGNC:13180]
ENSG00000158941	1657.41994	1328.13208	2038.67432	2289.85426	-0.536415651	0.0001145	0.0008752	CCAR2	cell cycle and apoptosis regulator 2 [Source:HGNC Symbol;Acc:HGNC:23360]
ENSG00000111331	4112.05494	3966.676	3156.52107	2893.45601	0.417122142	0.0001149	0.0008784	OAS3	2'-5'-oligoadenylate synthetase 3 [Source:HGNC Symbol;Acc:HGNC:8088]
ENSG00000171522	187.985279	186.062533	80.0719631	88.3579281	1.151123412	0.0001158	0.0008848	PTGER4	prostaglandin E receptor 4 [Source:HGNC Symbol;Acc:HGNC:9596]
ENSG00000004799	113.184854	124.927701	49.5181877	31.9366005	1.547515761	0.0001166	0.0008904	PDK4	pyruvate dehydrogenase kinase 4 [Source:HGNC Symbol;Acc:HGNC:8812]
ENSG00000078369	7320.59949	6757.614	9059.7212	9289.29253	-0.38232095	0.0001167	0.0008904	GNB1	G protein subunit beta 1 [Source:HGNC Symbol;Acc:HGNC:4396]
ENSG00000148296	896.620884	788.550735	1166.31136	1308.33607	-0.554824667	0.0001171	0.0008933	SURF6	surfeit 6 [Source:HGNC Symbol;Acc:HGNC:11478]
ENSG00000099795	1309.00744	1110.17311	1654.11819	1819.32168	-0.522361934	0.0001174	0.0008955	NDUFB7	NADH:ubiquinone oxidoreductase subunit B7 [Source:HGNC Symbol;Acc:HGNC:7702]
ENSG00000162426	116.137502	83.2851338	37.9288246	14.9037469	1.914222227	0.0001179	0.0008987	SLC45A1	solute carrier family 45 member 1 [Source:HGNC Symbol;Acc:HGNC:17939]
ENSG00000185885	29.5264835	20.3782774	79.0183847	95.8098015	-1.812976866	0.0001185	0.0009025	IFITM1	interferon induced transmembrane protein 1 [Source:HGNC Symbol;Acc:HGNC:5412]
ENSG00000178035	5397.44119	5004.19612	6671.25882	6976.0181	-0.391900466	0.0001186	0.0009029	IMPDH2	inosine monophosphate dehydrogenase 2 [Source:HGNC Symbol;Acc:HGNC:6053]
ENSG00000068028	789.341327	715.897746	1118.90033	1079.4571	-0.546913597	0.0001191	0.0009066	RASSF1	Ras association domain family member 1 [Source:HGNC Symbol;Acc:HGNC:9882]
ENSG00000137103	266.722568	271.119691	154.876034	111.778102	1.012012206	0.0001201	0.0009137	TMEM8B	transmembrane protein 8B [Source:HGNC Symbol;Acc:HGNC:21427]
ENSG00000101558	2695.76795	3205.59164	4300.70728	3867.52232	-0.468701304	0.0001205	0.0009161	VAPA	VAMP associated protein A [Source:HGNC Symbol;Acc:HGNC:12648]
ENSG00000161671	2654.43087	2341.72988	3325.09363	3453.41107	-0.44042033	0.0001209	0.0009192	EMC10	ER membrane protein complex subunit 10 [Source:HGNC Symbol;Acc:HGNC:27609]
ENSG00000080189	608.245561	629.954576	898.702428	936.806948	-0.567730474	0.000121	0.0009192	SLC35C2	solute carrier family 35 member C2 [Source:HGNC Symbol;Acc:HGNC:17117]
ENSG00000119673	432.070876	411.995609	664.80801	649.377544	-0.639045267	0.0001218	0.0009243	ACOT2	acyl-CoA thioesterase 2 [Source:HGNC Symbol;Acc:HGNC:18431]
ENSG00000187605	528.524055	513.886996	848.130662	746.251899	-0.61327955	0.0001218	0.0009243	TET3	tet methylcytosine dioxygenase 3 [Source:HGNC Symbol;Acc:HGNC:28313]
ENSG00000170779	1676.12005	1475.21008	2231.47918	2141.88134	-0.473182103	0.0001224	0.0009285	CDCA4	cell division cycle associated 4 [Source:HGNC Symbol;Acc:HGNC:14625]
ENSG00000145912	1507.81909	1276.74338	1896.44123	2055.65252	-0.505650147	0.0001228	0.0009315	NHP2	NHP2 ribonucleoprotein [Source:HGNC Symbol;Acc:HGNC:14377]
ENSG00000162676	31.4949158	37.2125066	2.10715692	3.19366005	3.697804049	0.000124	0.0009401	GFI1	growth factor independent 1 transcriptional repressor [Source:HGNC Symbol;Acc:HGNC:4237]
ENSG00000135063	43.3055092	40.7565548	2.10715692	7.45187345	3.138488368	0.0001243	0.0009416	FAM189A2	family with sequence similarity 189 member A2 [Source:HGNC Symbol;Acc:HGNC:24820]
ENSG00000110700	4674.04235	5376.32119	6349.91739	7395.45212	-0.451537201	0.0001245	0.0009427	RPS13	ribosomal protein S13 [Source:HGNC Symbol;Acc:HGNC:10386]
ENSG00000100731	1854.26317	1924.4182	1438.1346	1254.04385	0.489148989	0.0001252	0.0009479	PCNX1	pecanex 1 [Source:HGNC Symbol;Acc:HGNC:19740]
ENSG00000213420	219.480194	199.352714	87.4470124	107.519888	1.102894816	0.0001254	0.0009493	GPC2	glypican 2 [Source:HGNC Symbol;Acc:HGNC:4450]
ENSG00000172757	14862.6476	13397.3884	17600.0282	19750.6583	-0.402415972	0.0001263	0.0009554	CFL1	cofilin 1 [Source:HGNC Symbol;Acc:HGNC:1874]
ENSG00000087087	3294.17135	3116.10442	4151.09914	4330.60303	-0.404074563	0.0001267	0.0009583	SRRT	serrate, RNA effector molecule [Source:HGNC Symbol;Acc:HGNC:24101]
ENSG00000136448	3000.87494	3082.43596	3941.43703	4093.20763	-0.401311436	0.0001271	0.0009603	NMT1	N-myristoyltransferase 1 [Source:HGNC Symbol;Acc:HGNC:7857]
ENSG00000197106	361.207315	308.332198	179.108339	186.296836	0.87299061	0.0001272	0.0009611	SLC6A17	solute carrier family 6 member 17 [Source:HGNC Symbol;Acc:HGNC:31399]
ENSG00000137842	239.164517	260.487546	433.020748	417.304913	-0.766501203	0.0001274	0.0009617	TMEM62	transmembrane protein 62 [Source:HGNC Symbol;Acc:HGNC:26269]
ENSG00000170515	3279.40811	3120.53448	3965.66933	4943.78576	-0.477325468	0.0001279	0.0009653	PA2G4	proliferation-associated 2G4 [Source:HGNC Symbol;Acc:HGNC:8550]
ENSG00000232187	392.702231	292.38398	523.628496	654.70031	-0.783780708	0.000129	0.000973	FTH1P7	ferritin heavy chain 1 pseudogene 7 [Source:HGNC Symbol;Acc:HGNC:3994]

ENSG00000126561	610.213993	474.902465	816.523308	886.772941	-0.651602207	0.0001295	0.0009763	STAT5A	signal transducer and activator of transcription 5A [Source:HGNC Symbol;Acc:HGNC:11366]
ENSG00000121653	378.923206	320.736366	206.501379	174.586749	0.875686582	0.0001295	0.0009763	MAPK8IP1	mitogen-activated protein kinase 8 interacting protein 1 [Source:HGNC Symbol;Acc:HGNC:6882]
ENSG00000227036	180.11155	191.378605	318.180696	354.496266	-0.856072215	0.0001296	0.0009764	LINC00511	long intergenic non-protein coding RNA 511 [Source:HGNC Symbol;Acc:HGNC:43564]
ENSG00000082641	5537.19988	4963.43957	7168.54786	6735.42905	-0.405163619	0.0001301	0.0009792	NFE2L1	nuclear factor, erythroid 2 like 1 [Source:HGNC Symbol;Acc:HGNC:7781]
ENSG00000101444	3884.70102	3303.93898	4781.13906	4954.43129	-0.437794453	0.0001301	0.0009792	AHCY	adenosylhomocysteinase [Source:HGNC Symbol;Acc:HGNC:343]
ENSG00000130787	2322.75004	2053.77596	1513.99225	1630.89573	0.476530632	0.0001303	0.0009801	HIP1R	huntingtin interacting protein 1 related [Source:HGNC Symbol;Acc:HGNC:18415]
ENSG00000214510	241.132949	264.917606	116.947209	137.327382	0.993474415	0.0001312	0.0009867	SPINK13	serine peptidase inhibitor, Kazal type 13 (putative) [Source:HGNC Symbol;Acc:HGNC:27200]
ENSG00000181444	102.358476	81.5131097	29.5001969	25.5492804	1.738688468	0.0001314	0.0009877	ZNF467	zinc finger protein 467 [Source:HGNC Symbol;Acc:HGNC:23154]
ENSG00000174501	196.843224	197.58069	102.197111	75.5832879	1.149383689	0.0001318	0.0009903	ANKRD36C	ankyrin repeat domain 36C [Source:HGNC Symbol;Acc:HGNC:32946]
ENSG00000183283	3327.6347	3557.33843	4484.02994	4598.87047	-0.399567867	0.0001341	0.0010074	DAZAP2	DAZ associated protein 2 [Source:HGNC Symbol;Acc:HGNC:2684]
ENSG00000067182	5364.96206	4886.35652	6596.45475	6881.27286	-0.394888287	0.0001343	0.0010079	TNFRSF1A	TNF receptor superfamily member 1A [Source:HGNC Symbol;Acc:HGNC:11916]
ENSG00000135519	77.7530733	66.4509046	16.8572554	19.1619603	2.000745967	0.0001347	0.0010107	KCNH3	potassium voltage-gated channel subfamily H member 3 [Source:HGNC Symbol;Acc:HGNC:6252]
ENSG00000203499	66.926696	61.1348323	140.125935	169.263983	-1.273365176	0.0001351	0.0010135	IQANK1	IQ motif and ankyrin repeat containing 1 [Source:HGNC Symbol;Acc:HGNC:49576]
ENSG00000010310	110.232205	112.523532	42.1431385	35.1302606	1.52730005	0.0001354	0.0010148	GIPR	gastric inhibitory polypeptide receptor [Source:HGNC Symbol;Acc:HGNC:4271]
ENSG00000157637	3772.50038	3372.16191	2660.28562	2652.86695	0.427110127	0.0001355	0.0010154	SLC38A10	solute carrier family 38 member 10 [Source:HGNC Symbol;Acc:HGNC:28237]
ENSG00000102003	83.65837	62.9068564	22.1251477	12.7746402	2.068347413	0.0001361	0.0010195	SYP	synaptophysin [Source:HGNC Symbol;Acc:HGNC:11506]
ENSG00000182871	1404.4764	1332.56214	972.452921	963.420782	0.499456889	0.0001362	0.0010196	COL18A1	collagen type XVIII alpha 1 chain [Source:HGNC Symbol;Acc:HGNC:2195]
ENSG00000149547	2370.97663	2476.40371	3170.21759	3293.72807	-0.415090406	0.0001363	0.0010199	EI24	EI24, autophagy associated transmembrane protein [Source:HGNC Symbol;Acc:HGNC:13276]
ENSG00000277161	242.117165	248.969389	421.431385	411.982147	-0.762864225	0.0001367	0.0010221	PIGW	phosphatidylinositol glycan anchor biosynthesis class W [Source:HGNC Symbol;Acc:HGNC:23213]
ENSG00000150054	255.896191	326.938451	163.304662	133.069169	0.976507851	0.0001368	0.0010221	MPP7	membrane palmitoylated protein 7 [Source:HGNC Symbol;Acc:HGNC:26542]
ENSG00000106031	277.548945	327.824463	168.572554	152.231129	0.916795989	0.0001368	0.0010221	HOXA13	homeobox A13 [Source:HGNC Symbol;Acc:HGNC:5102]
ENSG00000196305	4449.64107	4758.77078	6437.3644	5783.71835	-0.408267011	0.0001372	0.0010253	IARS	isoleucyl-tRNA synthetase [Source:HGNC Symbol;Acc:HGNC:5330]
ENSG00000154511	236.211868	319.850354	160.143926	85.164268	1.181125634	0.0001393	0.0010402	FAM69A	family with sequence similarity 69 member A [Source:HGNC Symbol;Acc:HGNC:32213]
ENSG00000177946	158.458795	119.611628	260.23388	288.493958	-0.982726065	0.0001398	0.0010431	CENPBD1	CENPB DNA-binding domain containing 1 [Source:HGNC Symbol;Acc:HGNC:28272]
ENSG00000116455	571.829565	566.161707	815.46973	887.837494	-0.581867722	0.0001414	0.001055	WDR77	WD repeat domain 77 [Source:HGNC Symbol;Acc:HGNC:29652]
ENSG00000125901	1431.05024	1027.77399	1748.94025	1988.58566	-0.604865096	0.0001422	0.0010603	MRPS26	mitochondrial ribosomal protein S26 [Source:HGNC Symbol;Acc:HGNC:14045]
ENSG00000168005	1143.65913	845.255507	1453.93828	1549.98968	-0.595753226	0.0001424	0.0010613	SPINDOC	spindlin interactor and repressor of chromatin binding [Source:HGNC Symbol;Acc:HGNC:25115]
ENSG00000143436	515.729246	549.327478	740.665659	912.322221	-0.633761003	0.0001424	0.0010613	MRPL9	mitochondrial ribosomal protein L9 [Source:HGNC Symbol;Acc:HGNC:14277]
ENSG00000075234	730.28836	701.721553	1090.45371	1000.68015	-0.546473375	0.0001429	0.0010642	TTC38	tetratricopeptide repeat domain 38 [Source:HGNC Symbol;Acc:HGNC:26082]
ENSG00000118495	1630.84611	1562.03926	1139.9719	1155.04039	0.476234975	0.0001442	0.0010731	PLAGL1	PLAG1 like zinc finger 1 [Source:HGNC Symbol;Acc:HGNC:9046]
ENSG00000232956	659.424799	564.389683	881.845173	965.549889	-0.594826088	0.0001447	0.001077	SNHG15	small nucleolar RNA host gene 15 [Source:HGNC Symbol;Acc:HGNC:27797]
ENSG00000214194	433.055092	466.042345	261.287459	275.719318	0.743908341	0.0001453	0.001081	SMIM30	small integral membrane protein 30 [Source:HGNC Symbol;Acc:HGNC:48953]
ENSG00000171503	634.819396	655.648926	387.716874	435.40232	0.648929376	0.0001455	0.0010816	ETFDH	electron transfer flavoprotein dehydrogenase [Source:HGNC Symbol;Acc:HGNC:3483]
ENSG00000071894	2937.88511	2507.41413	3678.04241	3724.87217	-0.44339288	0.0001459	0.0010841	CPSF1	cleavage and polyadenylation specific factor 1 [Source:HGNC Symbol;Acc:HGNC:2324]
ENSG00000163888	463.565792	437.689959	272.876822	267.202891	0.738486257	0.0001467	0.0010895	CAMK2N2	calcium/calmodulin dependent protein kinase II inhibitor 2 [Source:HGNC Symbol;Acc:HGNC:24197]
ENSG00000115306	8107.97238	7910.31569	10554.749	10067.481	-0.364509146	0.0001468	0.0010895	SPTBN1	spectrin beta, non-erythrocytic 1 [Source:HGNC Symbol;Acc:HGNC:11275]
ENSG00000022567	492.108059	384.529235	231.787262	266.138338	0.815203406	0.0001473	0.0010931	SLC45A4	solute carrier family 45 member 4 [Source:HGNC Symbol;Acc:HGNC:29196]
ENSG00000106526	52.1634543	56.704772	130.643729	143.714702	-1.332580996	0.0001478	0.0010963	ACTR3C	ARP3 actin related protein 3 homolog C [Source:HGNC Symbol;Acc:HGNC:37282]
ENSG00000144730	257.864623	287.95392	146.447406	138.391936	0.938744484	0.0001482	0.0010985	IL17RD	interleukin 17 receptor D [Source:HGNC Symbol;Acc:HGNC:17616]
ENSG00000160214	944.847473	676.027203	1214.77597	1296.62598	-0.632778064	0.0001486	0.001101	RRP1	ribosomal RNA processing 1 [Source:HGNC Symbol;Acc:HGNC:18785]
ENSG00000126895	56.1003187	30.1244101	118.000788	132.004615	-1.541526168	0.0001489	0.0011033	AVPR2	arginine vasopressin receptor 2 [Source:HGNC Symbol;Acc:HGNC:897]
ENSG00000138735	177.158901	197.58069	99.0363755	55.3567742	1.279197269	0.0001494	0.001106	PDE5A	phosphodiesterase 5A [Source:HGNC Symbol;Acc:HGNC:8784]
ENSG00000228106	132.869176	125.813713	42.1431385	55.3567742	1.407757453	0.0001506	0.0011147	AL392172.1	dispatched RND transporter family member 1 [Source:NCBI gene;Acc:84976]
ENSG00000105135	1007.83731	908.162363	1423.3845	1314.72339	-0.515536119	0.0001509	0.0011161	ILVBL	ilvB acetolactate synthase like [Source:HGNC Symbol;Acc:HGNC:6041]
ENSG00000106804	137.790257	128.471749	59.0003939	41.5175807	1.404702182	0.0001516	0.0011215	C5	complement C5 [Source:HGNC Symbol;Acc:HGNC:1331]
ENSG00000119042	265.738352	280.865824	148.554563	138.391936	0.929917066	0.0001521	0.0011239	SATB2	SATB homeobox 2 [Source:HGNC Symbol;Acc:HGNC:21637]
ENSG00000166311	1123.97481	1041.95018	767.005121	722.831725	0.539535753	0.0001521	0.0011239	SMPD1	sphingomyelin phosphodiesterase 1 [Source:HGNC Symbol;Acc:HGNC:11120]
ENSG00000074071	3803.01108	3187.8714	4675.78122	4814.9748	-0.441317225	0.0001524	0.0011253	MRPS34	mitochondrial ribosomal protein S34 [Source:HGNC Symbol;Acc:HGNC:16618]

ENSG00000136111	587.577023	617.550407	408.788443	342.786179	0.681278939	0.0001527	0.0011272	TBC1D4	TBC1 domain family member 4 [Source:HGNC Symbol;Acc:HGNC:19165]
ENSG00000116985	355.302019	334.026547	191.75128	195.877816	0.830240512	0.0001533	0.0011312	BMP8B	bone morphogenetic protein 8b [Source:HGNC Symbol;Acc:HGNC:1075]
ENSG00000242485	1029.49006	942.716834	1306.43729	1556.377	-0.537895423	0.0001534	0.0011312	MRPL20	mitochondrial ribosomal protein L20 [Source:HGNC Symbol;Acc:HGNC:14478]
ENSG00000131773	56.1003187	45.1866152	4.21431385	11.7100869	2.669886168	0.0001536	0.0011323	KHDRBS3	KH RNA binding domain containing, signal transduction associated 3 [Source:HGNC Symbol;Acc:HGNC:18117]
ENSG00000166387	120.074366	132.901809	41.08956	53.2276675	1.424162992	0.0001536	0.0011323	PPFIBP2	PPFIA binding protein 2 [Source:HGNC Symbol;Acc:HGNC:9250]
ENSG00000166897	1062.95341	926.768617	1527.68877	1347.72454	-0.531747941	0.0001539	0.0011336	ELFN2	extracellular leucine rich repeat and fibronectin type III domain containing 2 [Source:HGNC Symbol;Acc:HGNC:29396]
ENSG00000172985	277.548945	264.917606	129.590151	152.231129	0.944676561	0.0001543	0.0011362	SH3RF3	SH3 domain containing ring finger 3 [Source:HGNC Symbol;Acc:HGNC:24699]
ENSG00000169057	898.589316	873.607893	1275.88352	1238.07555	-0.504563598	0.0001545	0.0011374	MECP2	methyl-CpG binding protein 2 [Source:HGNC Symbol;Acc:HGNC:6990]
ENSG00000215440	164.364092	161.254195	281.305449	322.559665	-0.89112421	0.0001556	0.0011449	NPEPL1	aminopeptidase like 1 [Source:HGNC Symbol;Acc:HGNC:16244]
ENSG00000170876	2623.92017	2744.86537	3579.00604	3521.54248	-0.403229813	0.0001564	0.00115	TMEM43	transmembrane protein 43 [Source:HGNC Symbol;Acc:HGNC:28472]
ENSG00000260565	400.57596	334.026547	200.179908	211.846117	0.833479888	0.0001571	0.001155	ERVK13-1	endogenous retrovirus group K13 member 1 [Source:HGNC Symbol;Acc:HGNC:27548]
ENSG00000233476	177.158901	176.3164	311.859225	326.817879	-0.853490259	0.0001573	0.0011558	EEF1A1P6	eukaryotic translation elongation factor 1 alpha 1 pseudogene 6 [Source:HGNC Symbol;Acc:HGNC:3201]
ENSG00000170854	275.580513	304.788149	473.05673	483.307221	-0.719895836	0.0001582	0.0011616	RIOX2	ribosomal oxygenase 2 [Source:HGNC Symbol;Acc:HGNC:19441]
ENSG00000185163	513.760814	447.436091	721.701247	755.832879	-0.621036925	0.0001583	0.0011616	DDX51	DEAD-box helicase 51 [Source:HGNC Symbol;Acc:HGNC:20082]
ENSG00000139112	4550.03111	4388.41774	3265.03965	3513.02606	0.39911264	0.0001589	0.0011656	GABARAPL1	GABA type A receptor associated protein like 1 [Source:HGNC Symbol;Acc:HGNC:4068]
ENSG00000213347	424.197147	371.239054	590.003939	675.991377	-0.671161527	0.0001594	0.001169	MXD3	MAX dimerization protein 3 [Source:HGNC Symbol;Acc:HGNC:14008]
ENSG00000177675	73.8162089	72.6529891	12.6429415	23.4201737	2.022749416	0.0001595	0.001169	CD163L1	CD163 molecule like 1 [Source:HGNC Symbol;Acc:HGNC:30375]
ENSG00000181396	782.451814	684.001312	1050.41773	1097.5545	-0.551253101	0.00016	0.0011727	OGFOD3	2-oxoglutarate and iron dependent oxygenase domain containing 3 [Source:HGNC Symbol;Acc:HGNC:26174]
ENSG00000122299	779.499166	893.100158	1200.02587	1218.91359	-0.531682299	0.0001612	0.0011804	ZC3H7A	zinc finger CCCH-type containing 7A [Source:HGNC Symbol;Acc:HGNC:30959]
ENSG00000121892	2179.05449	2370.08227	3123.86014	2983.94304	-0.424857163	0.0001615	0.0011821	PDS5A	PDS5 cohesin associated factor A [Source:HGNC Symbol;Acc:HGNC:29088]
ENSG00000173198	27.5580513	36.3264945	0	3.19366005	4.328385948	0.000162	0.0011852	CYSLTR1	cysteinyl leukotriene receptor 1 [Source:HGNC Symbol;Acc:HGNC:17451]
ENSG00000198626	122.042799	129.357761	56.893237	29.8074938	1.535454532	0.0001624	0.0011878	RYR2	ryanodine receptor 2 [Source:HGNC Symbol;Acc:HGNC:10484]
ENSG00000153094	611.198209	682.229288	399.306237	426.885893	0.647135791	0.0001625	0.0011879	BCL2L11	BCL2 like 11 [Source:HGNC Symbol;Acc:HGNC:994]
ENSG00000114054	1140.70648	1000.30762	1461.31333	1584.05539	-0.508789856	0.0001627	0.0011889	PCCB	propionyl-CoA carboxylase subunit beta [Source:HGNC Symbol;Acc:HGNC:8654]
ENSG00000145730	5038.20231	5538.4614	4169.00998	3764.26065	0.414989216	0.0001628	0.0011892	PAM	peptidylglycine alpha-amidating monooxygenase [Source:HGNC Symbol;Acc:HGNC:8596]
ENSG00000128510	270.659433	289.725944	162.251083	128.810955	0.945202739	0.0001628	0.0011892	CPA4	carboxypeptidase A4 [Source:HGNC Symbol;Acc:HGNC:15740]
ENSG00000164683	131.88496	181.632473	65.3218647	62.8086477	1.292291354	0.000163	0.0011901	HEY1	hes related family bHLH transcription factor with YRPW motif 1 [Source:HGNC Symbol;Acc:HGNC:4880]
ENSG00000104687	1926.11094	2067.06614	1423.3845	1488.24558	0.455908378	0.0001633	0.0011912	GSR	glutathione-disulfide reductase [Source:HGNC Symbol;Acc:HGNC:4623]
ENSG00000114779	771.625437	667.167083	1024.07827	1095.4254	-0.55951026	0.0001636	0.0011928	ABHD14B	abhydrolase domain containing 14B [Source:HGNC Symbol;Acc:HGNC:28235]
ENSG00000176788	9626.61785	9692.97196	11807.4538	13513.4402	-0.390250876	0.0001639	0.0011946	BASP1	brain abundant membrane attached signal protein 1 [Source:HGNC Symbol;Acc:HGNC:957]
ENSG00000130305	683.045986	529.835213	905.023899	963.420782	-0.624481873	0.0001642	0.0011967	NSUN5	NOP2/Sun RNA methyltransferase family member 5 [Source:HGNC Symbol;Acc:HGNC:16385]
ENSG00000187051	1007.83731	941.830822	1302.22298	1488.24558	-0.51750508	0.0001656	0.0012064	RPS19BP1	ribosomal protein S19 binding protein 1 [Source:HGNC Symbol;Acc:HGNC:28749]
ENSG00000072071	1824.73668	1526.59878	1231.63322	1091.16718	0.528422888	0.0001664	0.0012115	ADGRL1	adhesion G protein-coupled receptor L1 [Source:HGNC Symbol;Acc:HGNC:20973]
ENSG00000170265	1065.90606	902.846291	1334.88391	1535.08593	-0.544302969	0.0001666	0.0012125	ZNF282	zinc finger protein 282 [Source:HGNC Symbol;Acc:HGNC:13076]
ENSG00000168078	564.940052	689.317384	1113.63243	860.159107	-0.653446523	0.0001673	0.001217	PBK	PDZ binding kinase [Source:HGNC Symbol;Acc:HGNC:18282]
ENSG00000145687	252.943542	261.373558	134.858043	132.004615	0.946700677	0.0001676	0.0012188	SSBP2	single stranded DNA binding protein 2 [Source:HGNC Symbol;Acc:HGNC:15831]
ENSG00000143486	731.272576	726.529891	1013.54248	1099.68361	-0.535652154	0.0001682	0.0012222	EIF2D	eukaryotic translation initiation factor 2D [Source:HGNC Symbol;Acc:HGNC:6583]
ENSG00000055070	2007.80088	1857.08128	2525.42757	2709.28828	-0.437888829	0.0001682	0.0012222	SZRD1	SUZ RNA binding domain containing 1 [Source:HGNC Symbol;Acc:HGNC:30232]
ENSG00000189306	912.368342	690.203396	1113.63243	1374.33838	-0.635416289	0.0001685	0.0012236	RRP7A	ribosomal RNA processing 7 homolog A [Source:HGNC Symbol;Acc:HGNC:24286]
ENSG00000129933	945.83169	800.954904	1291.68719	1241.26921	-0.536811551	0.000169	0.0012269	MAU2	MAU2 sister chromatid cohesion factor [Source:HGNC Symbol;Acc:HGNC:29140]
ENSG00000066135	543.287297	481.990562	789.130268	765.413859	-0.601150712	0.0001692	0.0012273	KDM4A	lysine demethylase 4A [Source:HGNC Symbol;Acc:HGNC:22978]
ENSG00000087365	5084.46047	4650.67731	6114.9694	6779.07573	-0.405536146	0.0001695	0.0012291	SF3B2	splicing factor 3b subunit 2 [Source:HGNC Symbol;Acc:HGNC:10769]
ENSG00000164347	1331.64441	1633.80624	1086.23939	914.451328	0.568144438	0.0001708	0.0012379	GFM2	G elongation factor mitochondrial 2 [Source:HGNC Symbol;Acc:HGNC:29682]
ENSG00000087245	1763.71528	1627.60416	2313.6583	2286.6606	-0.440143155	0.0001709	0.001238	MMP2	matrix metallopeptidase 2 [Source:HGNC Symbol;Acc:HGNC:7166]
ENSG00000117226	180.11155	209.098847	339.252265	354.496266	-0.832733968	0.0001714	0.0012416	GBP3	guanylate binding protein 3 [Source:HGNC Symbol;Acc:HGNC:4184]
ENSG00000236753	94.4847473	126.699725	38.9824031	37.2593673	1.537857281	0.0001735	0.0012558	MKLN1-AS	MKLN1 antisense RNA [Source:HGNC Symbol;Acc:HGNC:40374]
ENSG00000102024	2863.08469	3342.92351	4343.904	4061.27103	-0.437352454	0.0001738	0.0012576	PLS3	plastin 3 [Source:HGNC Symbol;Acc:HGNC:9091]
ENSG00000079156	343.491425	325.166427	201.233486	168.199429	0.855563179	0.0001739	0.0012579	OSBPL6	oxysterol binding protein like 6 [Source:HGNC Symbol;Acc:HGNC:16388]
ENSG00000056998	112.200637	72.6529891	18.9644123	33.0011539	1.829729355	0.0001751	0.001266	GYG2	glycogenin 2 [Source:HGNC Symbol;Acc:HGNC:4700]

ENSG00000214253	852.331158	759.312337	1129.43611	1199.75163	-0.531791717	0.0001763	0.0012739	FIS1	fission, mitochondrial 1 [Source:HGNC Symbol;Acc:HGNC:21689]
ENSG00000151090	369.081044	339.34262	218.090742	175.651303	0.846830694	0.0001766	0.0012755	THRB	thyroid hormone receptor beta [Source:HGNC Symbol;Acc:HGNC:11799]
ENSG00000177646	1165.31188	1148.27163	1524.52803	1716.06	-0.486145747	0.0001769	0.0012771	ACAD9	acyl-CoA dehydrogenase family member 9 [Source:HGNC Symbol;Acc:HGNC:21497]
ENSG00000172939	3393.57718	3422.66459	2525.42757	2634.76954	0.401578192	0.000178	0.001285	OXSR1	oxidative stress responsive 1 [Source:HGNC Symbol;Acc:HGNC:8508]
ENSG00000137076	5298.03536	4508.91538	6668.09809	6435.225	-0.418253698	0.0001784	0.0012873	TLN1	talin 1 [Source:HGNC Symbol;Acc:HGNC:11845]
ENSG00000172809	5747.82213	5141.52799	6778.72383	7852.14551	-0.426201236	0.0001786	0.0012884	RPL38	ribosomal protein L38 [Source:HGNC Symbol;Acc:HGNC:10349]
ENSG00000197563	903.510397	1137.63949	717.486933	608.924516	0.622397665	0.0001792	0.0012922	PIGN	phosphatidylinositol glycan anchor biosynthesis class N [Source:HGNC Symbol;Acc:HGNC:8967]
ENSG00000101843	795.246624	847.027531	1214.77597	1138.00753	-0.518406035	0.0001793	0.0012922	PSMD10	proteasome 26S subunit, non-ATPase 10 [Source:HGNC Symbol;Acc:HGNC:9555]
ENSG00000141458	2964.45895	2774.10377	2147.19291	2147.20411	0.41809498	0.0001795	0.0012923	NPC1	NPC intracellular cholesterol transporter 1 [Source:HGNC Symbol;Acc:HGNC:7897]
ENSG00000132376	601.356048	594.514093	370.859619	392.820186	0.647011343	0.0001795	0.0012923	INPP5K	inositol polyphosphate-5-phosphatase K [Source:HGNC Symbol;Acc:HGNC:33882]
ENSG00000100558	1165.31188	1108.40109	750.147865	826.0934	0.528443221	0.0001797	0.0012934	PLEK2	pleckstrin 2 [Source:HGNC Symbol;Acc:HGNC:19238]
ENSG00000268621	1671.19897	1615.19999	1083.07866	1250.85019	0.493714412	0.000181	0.0013023	IGFL2-AS1	IGFL2 antisense RNA 1 [Source:HGNC Symbol;Acc:HGNC:52559]
ENSG00000127022	10846.0616	11931.0384	15471.7997	14202.2062	-0.381560986	0.0001817	0.0013064	CANX	calnexin [Source:HGNC Symbol;Acc:HGNC:1473]
ENSG00000198719	20.6685385	17.7202412	0	0	6.621974721	0.0001818	0.0013064	DLL1	delta like canonical Notch ligand 1 [Source:HGNC Symbol;Acc:HGNC:2908]
ENSG00000265763	299.2017	281.751836	170.679711	136.262829	0.91999721	0.0001824	0.0013103	ZNF488	zinc finger protein 488 [Source:HGNC Symbol;Acc:HGNC:23535]
ENSG00000140807	31.4949158	51.3886996	5.26789231	5.32276675	2.970223563	0.0001832	0.0013158	NKD1	naked cuticle homolog 1 [Source:HGNC Symbol;Acc:HGNC:17045]
ENSG00000181827	282.470026	307.446185	556.289428	437.531427	-0.752067037	0.0001839	0.0013198	RFX7	regulatory factor X7 [Source:HGNC Symbol;Acc:HGNC:25777]
ENSG00000122705	2226.29686	2034.28369	2769.85778	2978.62027	-0.432346438	0.0001851	0.0013283	CLTA	clathrin light chain A [Source:HGNC Symbol;Acc:HGNC:2090]
ENSG00000101670	100.390044	92.1452544	37.9288246	20.2265137	1.72606935	0.0001853	0.0013284	LIPG	lipase G, endothelial type [Source:HGNC Symbol;Acc:HGNC:6623]
ENSG00000083845	9485.87495	8430.40477	11587.2559	11730.3134	-0.380239131	0.0001854	0.0013284	RPS5	ribosomal protein S5 [Source:HGNC Symbol;Acc:HGNC:10426]
ENSG00000172531	3325.66626	2770.55972	3981.47301	4379.57248	-0.456049316	0.0001854	0.0013284	PPP1CA	protein phosphatase 1 catalytic subunit alpha [Source:HGNC Symbol;Acc:HGNC:9281]
ENSG00000229117	19831.9548	20127.536	24449.3418	26787.356	-0.358630244	0.0001857	0.0013305	RPL41	ribosomal protein L41 [Source:HGNC Symbol;Acc:HGNC:10354]
ENSG00000183864	2436.91911	2191.99384	3068.02048	3141.49694	-0.424063282	0.0001868	0.0013378	TOB2	transducer of ERBB2, 2 [Source:HGNC Symbol;Acc:HGNC:11980]
ENSG00000162377	839.536349	859.4317	1171.57925	1243.39831	-0.507220022	0.0001876	0.0013423	COA7	cytochrome c oxidase assembly factor 7 (putative) [Source:HGNC Symbol;Acc:HGNC:25716]
ENSG00000133027	370.06526	265.803619	512.039133	569.536042	-0.76805147	0.0001884	0.0013479	PEMT	phosphatidylethanolamine N-methyltransferase [Source:HGNC Symbol;Acc:HGNC:8830]
ENSG00000172301	898.589316	846.141519	1207.40092	1270.01215	-0.506084752	0.0001887	0.0013494	COPRS	coordinator of PRMT5 and differentiation stimulator [Source:HGNC Symbol;Acc:HGNC:28848]
ENSG00000168175	1242.08074	1426.47942	1922.78069	1804.41793	-0.481592372	0.000189	0.0013506	MAPK1IP1L	mitogen-activated protein kinase 1 interacting protein 1 like [Source:HGNC Symbol;Acc:HGNC:19840]
ENSG00000115844	355.302019	312.762258	190.697702	182.038623	0.841204984	0.0001903	0.0013592	DLX2	distal-less homeobox 2 [Source:HGNC Symbol;Acc:HGNC:2915]
ENSG00000124496	573.797997	514.773008	840.755613	793.092246	-0.586447104	0.0001904	0.0013592	TRERF1	transcriptional regulating factor 1 [Source:HGNC Symbol;Acc:HGNC:18273]
ENSG00000155846	387.781151	357.948873	201.233486	226.749864	0.800829386	0.0001904	0.0013592	PPARGC1B	PPARG coactivator 1 beta [Source:HGNC Symbol;Acc:HGNC:30022]
ENSG00000165959	488.171195	485.53461	299.216283	301.268598	0.697338314	0.0001906	0.0013599	CLMN	calmin [Source:HGNC Symbol;Acc:HGNC:19972]
ENSG00000100888	2838.47928	2662.46625	3652.75653	3606.70675	-0.400334207	0.0001906	0.0013599	CHD8	chromodomain helicase DNA binding protein 8 [Source:HGNC Symbol;Acc:HGNC:20153]
ENSG00000006468	86.6110184	96.5753147	35.8216677	14.9037469	1.85198208	0.000194	0.001383	ETV1	ETS variant 1 [Source:HGNC Symbol;Acc:HGNC:3490]
ENSG00000049192	108.263773	123.155677	44.2502954	40.4530273	1.45052759	0.0001948	0.0013881	ADAMTS6	ADAM metallopeptidase with thrombospondin type 1 motif 6 [Source:HGNC Symbol;Acc:HGNC:222]
ENSG00000103089	98.4216118	116.953592	35.8216677	39.388474	1.518770001	0.0001955	0.0013918	FA2H	fatty acid 2-hydroxylase [Source:HGNC Symbol;Acc:HGNC:21197]
ENSG00000105928	769.657004	800.068892	551.021536	488.629988	0.594488272	0.0001956	0.0013918	GSDME	gasdermin E [Source:HGNC Symbol;Acc:HGNC:2810]
ENSG00000139926	800.167704	822.219193	556.289428	534.405782	0.572953163	0.0001956	0.0013918	FRMD6	FERM domain containing 6 [Source:HGNC Symbol;Acc:HGNC:19839]
ENSG00000099364	1009.80574	900.188255	1335.93749	1370.08016	-0.50307525	0.0001956	0.0013918	FBXL19	F-box and leucine rich repeat protein 19 [Source:HGNC Symbol;Acc:HGNC:25300]
ENSG00000120318	836.5837	640.586721	1122.06106	1106.07093	-0.59403153	0.000196	0.0013942	ARAP3	ArfGAP with RhoGAP domain, ankyrin repeat and PH domain 3 [Source:HGNC Symbol;Acc:HGNC:24097]
ENSG00000089063	1440.8924	1490.27229	1108.36454	958.098015	0.504343293	0.0001965	0.0013972	TMEM230	transmembrane protein 230 [Source:HGNC Symbol;Acc:HGNC:15876]
ENSG00000010704	345.459857	352.632801	519.414182	596.149876	-0.676091433	0.0001969	0.0013991	HFE	homeostatic iron regulator [Source:HGNC Symbol;Acc:HGNC:4886]
ENSG00000198142	1420.22386	1262.56719	1000.89954	840.997147	0.542159304	0.0001974	0.0014019	SOWAHC	sosondowah ankyrin repeat domain family member C [Source:HGNC Symbol;Acc:HGNC:26149]
ENSG00000165934	1371.01305	1676.33482	2297.85463	2037.55511	-0.508122694	0.000198	0.0014059	CPSF2	cleavage and polyadenylation specific factor 2 [Source:HGNC Symbol;Acc:HGNC:2325]
ENSG00000144815	1590.49325	1526.59878	1103.09665	1146.52396	0.470412137	0.0001983	0.0014075	NXPE3	neurexophilin and PC-esterase domain family member 3 [Source:HGNC Symbol;Acc:HGNC:28238]
ENSG00000083123	359.238883	362.378933	200.179908	215.039777	0.79745945	0.0001986	0.0014089	BCKDHB	branched chain keto acid dehydrogenase E1 subunit beta [Source:HGNC Symbol;Acc:HGNC:987]
ENSG00000100764	1338.53392	1325.47404	1716.27932	2005.61851	-0.482430872	0.0001996	0.001415	PSMC1	proteasome 26S subunit, ATPase 1 [Source:HGNC Symbol;Acc:HGNC:9547]
ENSG00000125611	328.728183	285.295884	506.77124	492.888201	-0.704078355	0.0002002	0.001419	CHCHD5	coiled-coil-helix-coiled-coil-helix domain containing 5 [Source:HGNC Symbol;Acc:HGNC:17840]

ENSG00000167986	7219.22523	6706.2253	9078.68561	8879.43949	-0.366994823	0.0002007	0.001422	DDB1	damage specific DNA binding protein 1 [Source:HGNC Symbol;Acc:HGNC:2717]
ENSG00000213585	5976.16027	6218.03265	7614.21155	8159.80143	-0.371300285	0.0002015	0.0014267	VDAC1	voltage dependent anion channel 1 [Source:HGNC Symbol;Acc:HGNC:12669]
ENSG00000262814	477.344817	349.088752	649.004333	695.153338	-0.70322629	0.000202	0.0014302	MRPL12	mitochondrial ribosomal protein L12 [Source:HGNC Symbol;Acc:HGNC:10378]
ENSG00000104219	782.451814	965.753147	576.307419	571.665149	0.607463834	0.0002022	0.001431	ZDHHC2	zinc finger DHHC-type containing 2 [Source:HGNC Symbol;Acc:HGNC:18469]
ENSG00000154122	3076.65959	2858.27491	2142.97859	2287.72515	0.421570825	0.0002024	0.0014314	ANKH	ANKH inorganic pyrophosphate transport regulator [Source:HGNC Symbol;Acc:HGNC:15492]
ENSG00000091986	941.894825	1057.01239	692.20105	674.926824	0.548451221	0.0002025	0.0014317	CCDC80	coiled-coil domain containing 80 [Source:HGNC Symbol;Acc:HGNC:30649]
ENSG00000130158	841.504781	785.006687	573.146684	506.727395	0.590588151	0.0002037	0.0014395	DOCK6	dedicator of cytokinesis 6 [Source:HGNC Symbol;Acc:HGNC:19189]
ENSG00000234771	209.638033	189.606581	86.3934339	102.197122	1.081699689	0.000204	0.0014411	SLC25A25-AS1	SLC25A25 antisense RNA 1 [Source:HGNC Symbol;Acc:HGNC:27844]
ENSG00000167615	2288.30247	1904.03992	1431.81313	1546.79602	0.492769323	0.0002041	0.0014412	LENG8	leukocyte receptor cluster member 8 [Source:HGNC Symbol;Acc:HGNC:15500]
ENSG00000159377	5004.73896	4692.31988	6011.71871	6801.43135	-0.402075941	0.0002045	0.0014433	PSMB4	proteasome subunit beta 4 [Source:HGNC Symbol;Acc:HGNC:9541]
ENSG00000078061	1347.39187	1199.66033	1725.76152	1809.7407	-0.473482586	0.0002047	0.0014441	ARAF	A-Raf proto-oncogene, serine/threonine kinase [Source:HGNC Symbol;Acc:HGNC:646]
ENSG00000117395	1796.19442	1687.85298	2234.63992	2534.70153	-0.453172084	0.000205	0.0014458	EBNA1BP2	EBNA1 binding protein 2 [Source:HGNC Symbol;Acc:HGNC:15531]
ENSG00000090975	709.619821	620.208443	1017.75679	943.194268	-0.560985932	0.0002051	0.0014459	PITPNM2	phosphatidylinositol transfer protein membrane associated 2 [Source:HGNC Symbol;Acc:HGNC:21044]
ENSG00000133216	2527.46699	2357.6781	1670.97544	1898.09862	0.452747278	0.0002061	0.0014515	EPHB2	EPH receptor B2 [Source:HGNC Symbol;Acc:HGNC:3393]
ENSG00000185585	1773.55744	1700.25715	1320.13381	1183.78333	0.472177507	0.0002061	0.0014515	OLFML2A	olfactomedin like 2A [Source:HGNC Symbol;Acc:HGNC:27270]
ENSG00000187094	65.9424799	57.590784	16.8572554	10.6455335	2.165996172	0.0002066	0.0014544	CCK	cholecystokinin [Source:HGNC Symbol;Acc:HGNC:1569]
ENSG00000164099	100.390044	77.9690614	30.5537754	23.4201737	1.723000332	0.0002073	0.0014585	PRSS12	serine protease 12 [Source:HGNC Symbol;Acc:HGNC:9477]
ENSG00000105613	158.458795	166.570268	73.7504924	69.1959678	1.185271836	0.0002073	0.0014585	MAST1	microtubule associated serine/threonine kinase 1 [Source:HGNC Symbol;Acc:HGNC:19034]
ENSG00000149541	1554.07725	1265.22522	987.203019	955.968909	0.536387818	0.0002074	0.0014585	B3GAT3	beta-1,3-glucuronyltransferase 3 [Source:HGNC Symbol;Acc:HGNC:923]
ENSG00000140575	7937.70299	8761.77328	11273.2895	10472.0113	-0.380823635	0.0002078	0.0014605	IQGAP1	IQ motif containing GTPase activating protein 1 [Source:HGNC Symbol;Acc:HGNC:6110]
ENSG00000123810	60.0371832	59.3628081	145.393828	140.521042	-1.259914059	0.0002079	0.0014608	B9D2	B9 domain containing 2 [Source:HGNC Symbol;Acc:HGNC:28636]
ENSG00000162923	2655.41509	3048.7675	2210.40761	1908.74416	0.469861379	0.0002091	0.0014688	WDR26	WD repeat domain 26 [Source:HGNC Symbol;Acc:HGNC:21208]
ENSG00000267321	374.002125	342.886668	187.536966	219.29799	0.81703946	0.0002097	0.0014723	LINC02001	long intergenic non-protein coding RNA 2001 [Source:HGNC Symbol;Acc:HGNC:52836]
ENSG00000277972	1191.88572	955.121003	1515.04583	1585.11994	-0.530738578	0.0002114	0.0014835	CISD3	CDGSH iron sulfur domain 3 [Source:HGNC Symbol;Acc:HGNC:27578]
ENSG00000179262	2225.31264	1876.57355	2732.98253	2903.03699	-0.458774165	0.0002118	0.001486	RAD23A	RAD23 homolog A, nucleotide excision repair protein [Source:HGNC Symbol;Acc:HGNC:9812]
ENSG00000167258	1527.50342	1748.1018	2522.26684	2102.49287	-0.497319049	0.000212	0.0014862	CDK12	cyclin dependent kinase 12 [Source:HGNC Symbol;Acc:HGNC:24224]
ENSG00000157895	512.776598	466.042345	706.951148	781.382159	-0.605070596	0.0002128	0.0014912	C12orf43	chromosome 12 open reading frame 43 [Source:HGNC Symbol;Acc:HGNC:25719]
ENSG00000139651	1351.32873	1282.05945	1835.33368	1768.22311	-0.452708389	0.0002131	0.0014924	ZNF740	zinc finger protein 740 [Source:HGNC Symbol;Acc:HGNC:27465]
ENSG00000187867	110.232205	102.777399	36.8752462	38.3239206	1.501839067	0.0002131	0.0014924	PALM3	paralemmin 3 [Source:HGNC Symbol;Acc:HGNC:33274]
ENSG00000269893	151.569282	146.19199	272.876822	279.977531	-0.893024234	0.000214	0.0014981	SNHG8	small nucleolar RNA host gene 8 [Source:HGNC Symbol;Acc:HGNC:33098]
ENSG00000107771	619.071938	716.783758	458.306631	388.561973	0.657986002	0.0002144	0.0014999	CCSER2	coiled-coil serine rich protein 2 [Source:HGNC Symbol;Acc:HGNC:29197]
ENSG00000143590	305.106997	252.513438	145.393828	149.037469	0.920510355	0.0002146	0.0015009	EFNA3	ephrin A3 [Source:HGNC Symbol;Acc:HGNC:3223]
ENSG00000153132	257.864623	369.46703	172.786868	152.231129	0.949958548	0.000215	0.0015034	CLGN	calmegin [Source:HGNC Symbol;Acc:HGNC:2060]
ENSG00000139946	26.5738352	42.528579	1.05357846	0	6.02991286	0.0002156	0.0015065	PELI2	pellino E3 ubiquitin protein ligase family member 2 [Source:HGNC Symbol;Acc:HGNC:8828]
ENSG00000196756	744.067385	689.317384	1010.38175	1060.29514	-0.531033866	0.000216	0.0015092	SNHG17	small nucleolar RNA host gene 17 [Source:HGNC Symbol;Acc:HGNC:48600]
ENSG00000171858	8872.70831	8196.49758	10602.1601	11557.8557	-0.376622772	0.0002163	0.0015104	RPS21	ribosomal protein S21 [Source:HGNC Symbol;Acc:HGNC:10409]
ENSG00000233016	953.705419	761.084361	1229.52607	1289.17411	-0.555481105	0.000217	0.0015145	SNHG7	small nucleolar RNA host gene 7 [Source:HGNC Symbol;Acc:HGNC:28254]
ENSG00000136810	5862.97542	6528.13687	7693.22993	8751.69309	-0.408221495	0.0002186	0.0015251	TXN	thioredoxin [Source:HGNC Symbol;Acc:HGNC:12435]
ENSG00000147123	2993.98543	2866.24902	3804.47183	3870.71598	-0.389338814	0.0002193	0.0015293	NDUFB11	NADH:ubiquinone oxidoreductase subunit B11 [Source:HGNC Symbol;Acc:HGNC:20372]
ENSG00000130589	1388.72894	1096.88293	1837.44084	1736.28651	-0.524527874	0.0002196	0.0015306	HELZ2	helicase with zinc finger 2 [Source:HGNC Symbol;Acc:HGNC:30021]
ENSG00000272333	1205.66474	990.561485	1601.43926	1536.15048	-0.515311458	0.0002206	0.0015369	KMT2B	lysine methyltransferase 2B [Source:HGNC Symbol;Acc:HGNC:15840]
ENSG00000070614	1274.55987	1237.75885	1763.69035	1679.86519	-0.455007322	0.000221	0.0015395	NDST1	N-deacetylase and N-sulfotransferase 1 [Source:HGNC Symbol;Acc:HGNC:7680]
ENSG00000134313	1607.22492	1718.8634	1235.84754	1173.13779	0.465570045	0.0002212	0.0015396	KIDINS220	kinase D interacting substrate 220 [Source:HGNC Symbol;Acc:HGNC:29508]
ENSG00000123892	59.0529671	46.9586393	4.21431385	13.8391936	2.554694999	0.0002212	0.0015396	RAB38	RAB38, member RAS oncogene family [Source:HGNC Symbol;Acc:HGNC:9776]
ENSG00000072210	2174.13341	2324.89565	1639.36809	1693.70438	0.432934796	0.000223	0.0015515	ALDH3A2	aldehyde dehydrogenase 3 family member A2 [Source:HGNC Symbol;Acc:HGNC:403]
ENSG00000104529	2206.61254	2104.27865	2725.60748	3103.17302	-0.435297681	0.0002233	0.0015528	EEF1D	eukaryotic translation elongation factor 1 delta [Source:HGNC Symbol;Acc:HGNC:3211]
ENSG00000170915	128.932311	124.927701	51.6253447	47.9049008	1.350588887	0.0002241	0.0015577	PAQR8	progestin and adipoQ receptor family member 8 [Source:HGNC Symbol;Acc:HGNC:15708]
ENSG00000179772	64.9582638	35.4404825	118.000788	156.489342	-1.456084918	0.0002263	0.0015723	FOXS1	forkhead box S1 [Source:HGNC Symbol;Acc:HGNC:3735]
ENSG00000181038	719.461982	671.597143	448.824425	466.274367	0.603926302	0.000227	0.0015765	METTL23	methyltransferase like 23 [Source:HGNC Symbol;Acc:HGNC:26988]
ENSG00000139767	62.0056154	40.7565548	6.32147077	11.7100869	2.51002554	0.0002275	0.0015796	SRRM4	serine/arginine repetitive matrix 4 [Source:HGNC Symbol;Acc:HGNC:29389]
ENSG00000106459	327.743967	320.736366	506.77124	525.889355	-0.671357641	0.0002277	0.0015803	NRF1	nuclear respiratory factor 1 [Source:HGNC Symbol;Acc:HGNC:7996]
ENSG00000160993	169.285172	181.632473	288.680499	349.173499	-0.861479653	0.000228	0.0015816	ALKBH4	alkB homolog 4, lysine demethylase [Source:HGNC Symbol;Acc:HGNC:21900]

ENSG00000137288	472.423737	388.959295	607.914773	785.640372	-0.694873338	0.0002284	0.0015837	UQCC2	ubiquinol-cytochrome c reductase complex assembly factor 2 [Source:HGNC Symbol;Acc:HGNC:21237]
ENSG00000166002	320.854455	270.233679	153.822455	165.005769	0.889870374	0.0002286	0.0015845	SMCO4	single-pass membrane protein with coiled-coil domains 4 [Source:HGNC Symbol;Acc:HGNC:24810]
ENSG00000121578	685.998634	701.721553	455.145896	462.016154	0.597572962	0.0002297	0.0015911	B4GALT4	beta-1,4-galactosyltransferase 4 [Source:HGNC Symbol;Acc:HGNC:927]
ENSG00000143367	612.182426	650.332853	382.448982	430.079554	0.636133307	0.0002299	0.0015922	TUFT1	tuftelin 1 [Source:HGNC Symbol;Acc:HGNC:12422]
ENSG00000170340	544.271513	589.198021	360.323834	360.883586	0.652603391	0.0002302	0.0015938	B3GNT2	UDP-GlcNAc:betaGal beta-1,3-N-acetylglucosaminyltransferase 2 [Source:HGNC Symbol;Acc:HGNC:15629]
ENSG00000205885	194.874791	206.44081	341.359422	356.625372	-0.798000685	0.0002312	0.0015998	C1RL-AS1	C1RL antisense RNA 1 [Source:HGNC Symbol;Acc:HGNC:27461]
ENSG00000100926	101.37426	102.777399	210.715692	204.394243	-1.023747166	0.0002315	0.0016014	TM9SF1	transmembrane 9 superfamily member 1 [Source:HGNC Symbol;Acc:HGNC:11864]
ENSG00000133226	1661.35681	1890.74974	2371.60512	2466.57011	-0.445415212	0.0002318	0.0016025	SRRM1	serine and arginine repetitive matrix 1 [Source:HGNC Symbol;Acc:HGNC:16638]
ENSG00000185112	220.46441	244.539329	129.590151	101.132568	1.011332146	0.0002319	0.0016025	FAM43A	family with sequence similarity 43 member A [Source:HGNC Symbol;Acc:HGNC:26888]
ENSG00000112118	6118.87161	5559.72569	7475.13919	7678.62332	-0.375922007	0.000232	0.0016025	MCM3	minichromosome maintenance complex component 3 [Source:HGNC Symbol;Acc:HGNC:6945]
ENSG00000169129	83.65837	93.0312665	20.0179908	33.0011539	1.737692464	0.0002321	0.0016025	AFAP1L2	actin filament associated protein 1 like 2 [Source:HGNC Symbol;Acc:HGNC:25901]
ENSG00000159069	2934.93246	2679.30047	3675.93526	3739.77592	-0.401692966	0.0002327	0.0016063	FBXW5	F-box and WD repeat domain containing 5 [Source:HGNC Symbol;Acc:HGNC:13613]
ENSG00000077348	513.760814	363.264945	662.700853	777.123946	-0.716648346	0.0002351	0.0016221	EXOSC5	exosome component 5 [Source:HGNC Symbol;Acc:HGNC:24662]
ENSG00000148730	2740.05767	2815.74633	2079.76388	2113.1384	0.406111643	0.0002353	0.0016231	EIF4EBP2	eukaryotic translation initiation factor 4E binding protein 2 [Source:HGNC Symbol;Acc:HGNC:3289]
ENSG00000083457	499.981788	490.850682	723.808404	760.091092	-0.582765511	0.0002367	0.0016318	ITGAE	integrin subunit alpha E [Source:HGNC Symbol;Acc:HGNC:6147]
ENSG00000171314	11501.5496	10591.3882	13837.6995	14533.2823	-0.360884906	0.000237	0.0016333	PGAM1	phosphoglycerate mutase 1 [Source:HGNC Symbol;Acc:HGNC:8888]
ENSG00000169105	875.952345	839.053423	1165.25778	1266.81849	-0.504138905	0.0002383	0.0016415	CHST14	carbohydrate sulfotransferase 14 [Source:HGNC Symbol;Acc:HGNC:24464]
ENSG00000198223	360.223099	359.720897	215.983585	201.200583	0.787135717	0.0002385	0.0016421	CSF2RA	colony stimulating factor 2 receptor alpha subunit [Source:HGNC Symbol;Acc:HGNC:2435]
ENSG00000178896	642.693125	644.130769	935.577675	928.290521	-0.534476718	0.0002405	0.0016554	EXOSC4	exosome component 4 [Source:HGNC Symbol;Acc:HGNC:18189]
ENSG00000117410	3568.76764	3130.28061	2383.19448	2581.54187	0.432047386	0.0002406	0.0016554	ATP6V0B	ATPase H+ transporting V0 subunit b [Source:HGNC Symbol;Acc:HGNC:861]
ENSG00000160190	375.970557	357.062861	230.733683	187.36139	0.809662537	0.0002407	0.0016557	SLC37A1	solute carrier family 37 member 1 [Source:HGNC Symbol;Acc:HGNC:11024]
ENSG00000167778	1107.24313	957.779039	1423.3845	1493.56835	-0.498844115	0.0002426	0.0016681	SPRYD3	SPRY domain containing 3 [Source:HGNC Symbol;Acc:HGNC:25920]
ENSG00000111962	614.150858	691.97542	441.449376	401.336613	0.632465512	0.0002453	0.0016857	UST	uronyl 2-sulfotransferase [Source:HGNC Symbol;Acc:HGNC:17223]
ENSG00000168243	95.4689635	85.94317	14.7500985	36.1948139	1.832831935	0.0002457	0.0016877	GNG4	G protein subunit gamma 4 [Source:HGNC Symbol;Acc:HGNC:4407]
ENSG00000155792	83.65837	95.6893027	28.4466185	28.7429405	1.649578118	0.0002462	0.0016908	DEPTOR	DEP domain containing MTOR interacting protein [Source:HGNC Symbol;Acc:HGNC:22953]
ENSG00000066427	172.237821	169.228304	325.555745	289.558511	-0.849309347	0.0002465	0.0016919	ATXN3	ataxin 3 [Source:HGNC Symbol;Acc:HGNC:7106]
ENSG00000134901	712.57247	649.446841	405.627708	473.726241	0.630974057	0.0002466	0.001692	KDELC1	KDEL motif containing 1 [Source:HGNC Symbol;Acc:HGNC:19350]
ENSG00000155893	249.006678	269.347667	138.018779	136.262829	0.918649994	0.0002481	0.0017017	PXYLP1	2-phosphoxylose phosphatase 1 [Source:HGNC Symbol;Acc:HGNC:26303]
ENSG00000198814	412.386554	485.53461	287.62692	245.911824	0.751551862	0.00025	0.0017139	GK	glycerol kinase [Source:HGNC Symbol;Acc:HGNC:4289]
ENSG00000162062	424.197147	407.565548	665.861588	612.118176	-0.619902045	0.0002508	0.0017184	TEDC2	tubulin epsilon and delta complex 2 [Source:HGNC Symbol;Acc:HGNC:25849]
ENSG00000204370	317.901806	310.104222	505.717662	496.081861	-0.673920857	0.0002513	0.0017211	SDHD	succinate dehydrogenase complex subunit D [Source:HGNC Symbol;Acc:HGNC:10683]
ENSG00000141456	2011.73775	1795.94645	2462.21287	2718.86926	-0.444613932	0.0002527	0.0017302	PELP1	proline, glutamate and leucine rich protein 1 [Source:HGNC Symbol;Acc:HGNC:30134]
ENSG00000138160	1322.78646	1660.3866	2543.33841	1908.74416	-0.577228916	0.0002529	0.0017308	KIF11	kinesin family member 11 [Source:HGNC Symbol;Acc:HGNC:6388]
ENSG00000197971	194.874791	222.389028	84.286277	113.907208	1.074900324	0.0002549	0.001744	MBP	myelin basic protein [Source:HGNC Symbol;Acc:HGNC:6925]
ENSG00000236552	562.97162	578.565876	914.506105	787.769479	-0.576405737	0.0002551	0.0017448	RPL13AP5	ribosomal protein L13a pseudogene 5 [Source:HGNC Symbol;Acc:HGNC:23736]
ENSG00000115457	39.3686447	25.6943498	0	4.2582134	3.936804083	0.0002554	0.0017461	IGFBP2	insulin like growth factor binding protein 2 [Source:HGNC Symbol;Acc:HGNC:5471]
ENSG00000118193	762.767492	898.416231	1445.50965	1083.71531	-0.606030759	0.0002558	0.0017478	KIF14	kinesin family member 14 [Source:HGNC Symbol;Acc:HGNC:19181]
ENSG00000161249	917.289422	769.05847	1194.75798	1239.1401	-0.53007787	0.0002563	0.0017507	DMKN	dermokine [Source:HGNC Symbol;Acc:HGNC:25063]
ENSG00000241878	400.57596	398.705428	598.432567	631.280137	-0.6215499	0.0002573	0.0017565	PISD	phosphatidylserine decarboxylase [Source:HGNC Symbol;Acc:HGNC:8999]
ENSG00000099995	2928.04295	2792.71002	3665.39947	3831.32751	-0.390153887	0.0002574	0.0017565	SF3A1	splicing factor 3a subunit 1 [Source:HGNC Symbol;Acc:HGNC:10765]
ENSG00000183048	291.327971	228.591112	413.002757	472.661688	-0.769915778	0.0002591	0.0017674	SLC25A10	solute carrier family 25 member 10 [Source:HGNC Symbol;Acc:HGNC:10980]
ENSG00000144485	218.495978	174.544376	74.8040708	104.326228	1.133015607	0.00026	0.0017731	HES6	hes family bHLH transcription factor 6 [Source:HGNC Symbol;Acc:HGNC:18254]
ENSG00000186395	670.251176	653.876902	932.416939	980.453636	-0.53080432	0.0002604	0.0017745	KRT10	keratin 10 [Source:HGNC Symbol;Acc:HGNC:6413]
ENSG00000170791	522.618759	604.260226	354.002363	357.689926	0.663612473	0.0002604	0.0017745	CHCHD7	coiled-coil-helix-coiled-coil-helix domain containing 7 [Source:HGNC Symbol;Acc:HGNC:28314]
ENSG00000100983	1449.75034	1253.70707	1818.47643	1930.03522	-0.471966163	0.0002622	0.0017859	GSS	glutathione synthetase [Source:HGNC Symbol;Acc:HGNC:4624]
ENSG00000215105	207.669601	240.109269	129.590151	63.873201	1.210694665	0.0002625	0.0017869	TTC3P1	tetratricopeptide repeat domain 3 pseudogene 1 [Source:HGNC Symbol;Acc:HGNC:23318]
ENSG00000161526	930.084232	895.758194	1231.63322	1336.01445	-0.492021231	0.0002627	0.0017876	SAP30BP	SAP30 binding protein [Source:HGNC Symbol;Acc:HGNC:30785]
ENSG00000112149	303.138564	275.549751	134.858043	173.522196	0.907861678	0.000263	0.001789	CD83	CD83 molecule [Source:HGNC Symbol;Acc:HGNC:1703]
ENSG00000274080	26.5738352	10.6321447	0	0	6.574569685	0.0002649	0.0018016	AC005089.1	novel transcript

ENSG00000053747	1207.63318	1239.53087	887.113065	854.83634	0.490478252	0.0002654	0.0018043	LAMA3	laminin subunit alpha 3 [Source:HGNC Symbol;Acc:HGNC:6483]
ENSG00000096093	460.613143	479.332525	280.251871	300.204045	0.69562193	0.0002671	0.001815	EFHC1	EF-hand domain containing 1 [Source:HGNC Symbol;Acc:HGNC:16406]
ENSG00000187720	1145.62756	1176.62402	856.55929	776.059392	0.508375896	0.0002686	0.0018242	THSD4	thrombospondin type 1 domain containing 4 [Source:HGNC Symbol;Acc:HGNC:25835]
ENSG00000162585	541.318865	471.358417	721.701247	816.51242	-0.603752276	0.0002707	0.0018377	FAAP20	FA core complex associated protein 20 [Source:HGNC Symbol;Acc:HGNC:26428]
ENSG00000083097	371.049477	434.14591	248.644517	224.620757	0.767293655	0.0002724	0.0018484	DOP1A	DOP1 leucine zipper like protein A [Source:HGNC Symbol;Acc:HGNC:21194]
ENSG00000109736	695.840796	605.146238	920.827576	988.970062	-0.554457943	0.0002726	0.0018489	MFSD10	major facilitator superfamily domain containing 10 [Source:HGNC Symbol;Acc:HGNC:16894]
ENSG00000079950	492.108059	507.684911	327.662902	295.945831	0.681052102	0.0002731	0.0018521	STX7	syntaxin 7 [Source:HGNC Symbol;Acc:HGNC:11442]
ENSG00000183431	2487.11413	2645.63202	3196.55705	3730.19494	-0.432296931	0.0002737	0.0018555	SF3A3	splicing factor 3a subunit 3 [Source:HGNC Symbol;Acc:HGNC:10767]
ENSG00000047849	9538.0384	8707.72654	11281.7182	12385.0137	-0.375361833	0.0002745	0.0018602	MAP4	microtubule associated protein 4 [Source:HGNC Symbol;Acc:HGNC:6862]
ENSG00000170631	138.774473	122.269665	254.965988	242.718164	-0.932003771	0.0002755	0.0018657	ZNF16	zinc finger protein 16 [Source:HGNC Symbol;Acc:HGNC:12947]
ENSG00000184678	417.307634	414.653645	210.715692	274.654764	0.77759057	0.0002759	0.001868	HIST2H2BE	histone cluster 2 H2B family member e [Source:HGNC Symbol;Acc:HGNC:4760]
ENSG00000129355	424.197147	324.280415	192.804859	225.68531	0.837831801	0.0002764	0.0018701	CDKN2D	cyclin dependent kinase inhibitor 2D [Source:HGNC Symbol;Acc:HGNC:1790]
ENSG00000126803	370.06526	341.114644	200.179908	211.846117	0.787142994	0.0002764	0.0018701	HSPA2	heat shock protein family A (Hsp70) member 2 [Source:HGNC Symbol;Acc:HGNC:5235]
ENSG00000186141	430.102444	420.855729	611.075508	696.217891	-0.619492253	0.0002781	0.0018804	POLR3C	RNA polymerase III subunit C [Source:HGNC Symbol;Acc:HGNC:30076]
ENSG00000205213	471.439521	555.529563	335.037951	297.010385	0.700864021	0.0002816	0.0019035	LGR4	leucine rich repeat containing G protein-coupled receptor 4 [Source:HGNC Symbol;Acc:HGNC:13299]
ENSG00000149243	386.796934	283.52386	553.128693	551.438635	-0.72223647	0.0002822	0.0019067	KLHL35	kelch like family member 35 [Source:HGNC Symbol;Acc:HGNC:26597]
ENSG00000182810	334.63348	280.865824	526.789231	474.790794	-0.70358258	0.0002837	0.0019159	DDX28	DEAD-box helicase 28 [Source:HGNC Symbol;Acc:HGNC:17330]
ENSG00000163686	667.298528	597.17213	374.020354	435.40232	0.643235024	0.0002848	0.0019229	ABHD6	abhydrolase domain containing 6 [Source:HGNC Symbol;Acc:HGNC:21398]
ENSG00000101413	602.340264	677.799227	1024.07827	876.127407	-0.569367118	0.0002851	0.0019241	RPRD1B	regulation of nuclear pre-mRNA domain containing 1B [Source:HGNC Symbol;Acc:HGNC:16209]
ENSG00000204356	1387.74473	1176.62402	1742.61878	1833.16087	-0.48017721	0.0002853	0.0019245	NELFE	negative elongation factor complex member E [Source:HGNC Symbol;Acc:HGNC:13974]
ENSG00000196363	1828.67355	1740.12769	2287.31884	2518.73323	-0.429534432	0.0002863	0.0019304	WDR5	WD repeat domain 5 [Source:HGNC Symbol;Acc:HGNC:12757]
ENSG00000174243	3430.97739	3242.80415	4275.4214	4405.12176	-0.379389221	0.0002864	0.0019304	DDX23	DEAD-box helicase 23 [Source:HGNC Symbol;Acc:HGNC:17347]
ENSG00000149328	433.055092	430.601862	256.019566	270.396551	0.714270209	0.0002867	0.0019315	GLB1L2	galactosidase beta 1 like 2 [Source:HGNC Symbol;Acc:HGNC:25129]
ENSG00000162520	178.143117	178.088424	303.430597	326.817879	-0.823068824	0.0002868	0.0019319	SYNC	syncoilin, intermediate filament protein [Source:HGNC Symbol;Acc:HGNC:28897]
ENSG00000184232	3087.48596	2753.72549	2131.38923	2229.17472	0.421548911	0.0002874	0.001935	OAF	out at first homolog [Source:HGNC Symbol;Acc:HGNC:28752]
ENSG00000077549	2387.7083	2419.69894	3065.91333	3263.92057	-0.396861114	0.000288	0.0019383	CAPZB	capping actin protein of muscle Z-line subunit beta [Source:HGNC Symbol;Acc:HGNC:1491]
ENSG00000140443	6171.03506	5673.13523	7754.33748	7512.55299	-0.366334822	0.0002888	0.001943	IGF1R	insulin like growth factor 1 receptor [Source:HGNC Symbol;Acc:HGNC:5465]
ENSG00000176444	579.703294	523.633129	791.237425	843.126253	-0.567374396	0.0002925	0.0019667	CLK2	CDC like kinase 2 [Source:HGNC Symbol;Acc:HGNC:2069]
ENSG00000169020	373.017909	217.958967	495.181877	576.987916	-0.861586126	0.0002931	0.0019699	ATP5ME	ATP synthase membrane subunit e [Source:HGNC Symbol;Acc:HGNC:846]
ENSG00000261061	116.137502	155.052111	214.930006	349.173499	-1.054719807	0.0002932	0.00197	AC092718.4	novel transcript
ENSG00000164850	83.65837	64.6788805	160.143926	171.393089	-1.162580057	0.0002944	0.0019769	GPER1	G protein-coupled estrogen receptor 1 [Source:HGNC Symbol;Acc:HGNC:4485]
ENSG00000161011	10379.5432	9726.64041	12903.1754	12668.1849	-0.346942743	0.0002962	0.0019883	SQSTM1	sequestosome 1 [Source:HGNC Symbol;Acc:HGNC:11280]
ENSG00000073050	548.208378	427.943826	721.701247	793.092246	-0.635097664	0.0002972	0.0019943	XRCC1	X-ray repair cross complementing 1 [Source:HGNC Symbol;Acc:HGNC:12828]
ENSG00000221983	9390.40598	8085.74608	11102.6098	11781.4119	-0.389058146	0.0002987	0.0020035	UBA52	ubiquitin A-52 residue ribosomal protein fusion product 1 [Source:HGNC Symbol;Acc:HGNC:12458]
ENSG00000026025	3036.30672	3354.44167	4135.29546	4252.89063	-0.392174759	0.0002988	0.0020036	VIM	vimentin [Source:HGNC Symbol;Acc:HGNC:12692]
ENSG00000188211	701.746092	741.592096	522.574917	399.207506	0.646948411	0.0002992	0.0020052	NCR3LG1	natural killer cell cytotoxicity receptor 3 ligand 1 [Source:HGNC Symbol;Acc:HGNC:42400]
ENSG00000104419	9783.10821	8464.07323	11698.9352	12072.035	-0.381629997	0.0002998	0.0020088	NDRG1	N-myc downstream regulated 1 [Source:HGNC Symbol;Acc:HGNC:7679]
ENSG00000174939	221.448627	152.394075	91.6613262	74.5187345	1.167986616	0.0003	0.0020091	ASPHD1	aspartate beta-hydroxylase domain containing 1 [Source:HGNC Symbol;Acc:HGNC:27380]
ENSG00000150991	17730.6534	16889.1619	21723.7343	21926.6054	-0.33441867	0.000301	0.0020155	UBC	ubiquitin C [Source:HGNC Symbol;Acc:HGNC:12468]
ENSG00000170604	751.941114	616.664395	1021.97111	992.163722	-0.558344164	0.0003033	0.0020299	IRF2BP1	interferon regulatory factor 2 binding protein 1 [Source:HGNC Symbol;Acc:HGNC:21728]
ENSG00000157617	989.137199	975.49928	675.343794	696.217891	0.51840786	0.0003035	0.0020303	C2CD2	C2 calcium dependent domain containing 2 [Source:HGNC Symbol;Acc:HGNC:1266]
ENSG00000148411	1259.79663	1118.14722	1628.8323	1654.31591	-0.465800891	0.0003046	0.0020371	NACC2	NACC family member 2 [Source:HGNC Symbol;Acc:HGNC:23846]
ENSG00000149554	585.60859	709.695661	984.042284	933.613288	-0.565194588	0.0003048	0.0020376	CHEK1	checkpoint kinase 1 [Source:HGNC Symbol;Acc:HGNC:1925]
ENSG00000142544	154.521931	171.000328	266.555351	332.140645	-0.878215852	0.0003051	0.0020386	CTU1	cytosolic thiouridylase subunit 1 [Source:HGNC Symbol;Acc:HGNC:29590]
ENSG00000143028	67.9109122	70.8809649	21.0715692	15.9683003	1.90558791	0.0003072	0.0020516	SYPL2	synaptophysin like 2 [Source:HGNC Symbol;Acc:HGNC:27638]
ENSG00000188917	223.417059	260.487546	403.520551	407.723933	-0.744345485	0.0003073	0.0020519	TRMT2B	tRNA methyltransferase 2 homolog B [Source:HGNC Symbol;Acc:HGNC:25748]
ENSG00000173237	2040.28001	1655.07053	2455.8914	2684.80355	-0.476709797	0.000309	0.0020621	C11orf86	chromosome 11 open reading frame 86 [Source:HGNC Symbol;Acc:HGNC:34442]
ENSG00000100519	1150.54864	1390.15292	1741.5652	1822.51534	-0.487697875	0.0003095	0.0020646	PSMC6	proteasome 26S subunit, ATPase 6 [Source:HGNC Symbol;Acc:HGNC:9553]
ENSG00000134202	1499.94536	1441.54162	964.024293	1127.362	0.492041524	0.0003117	0.0020788	GSTM3	glutathione S-transferase mu 3 [Source:HGNC Symbol;Acc:HGNC:4635]
ENSG00000158526	1028.50584	902.846291	1281.15141	1474.40639	-0.51317679	0.0003128	0.0020843	TSR2	TSR2, ribosome maturation factor [Source:HGNC Symbol;Acc:HGNC:25455]
ENSG00000173511	839.536349	809.815024	1079.91792	1305.14241	-0.532212597	0.0003128	0.0020843	VEGFB	vascular endothelial growth factor B [Source:HGNC Symbol;Acc:HGNC:12681]

ENSG00000106682	8807.75004	8217.76187	10769.679	10923.3819	-0.349598089	0.0003129	0.0020844	EIF4H	eukaryotic translation initiation factor 4H [Source:HGNC Symbol;Acc:HGNC:12741]
ENSG00000137161	734.225224	591.856057	928.202625	1057.10148	-0.583049446	0.0003144	0.0020934	CNPY3	canopy FGF signaling regulator 3 [Source:HGNC Symbol;Acc:HGNC:11968]
ENSG00000247315	764.735924	635.270648	977.720813	1083.71531	-0.558980395	0.0003159	0.0021027	ZCCHC3	zinc finger CCHC-type containing 3 [Source:HGNC Symbol;Acc:HGNC:16230]
ENSG00000105705	575.766429	474.016453	738.558502	871.869194	-0.618204303	0.0003167	0.0021072	SUGP1	SURP and G-patch domain containing 1 [Source:HGNC Symbol;Acc:HGNC:18643]
ENSG00000112297	1546.20352	1715.31935	2313.6583	2119.52572	-0.442511203	0.0003189	0.0021207	CRYBG1	crystallin beta-gamma domain containing 1 [Source:HGNC Symbol;Acc:HGNC:356]
ENSG00000158773	699.77766	664.509046	927.149047	1050.71416	-0.535996757	0.0003195	0.0021239	USF1	upstream transcription factor 1 [Source:HGNC Symbol;Acc:HGNC:12593]
ENSG00000113621	603.32448	687.54536	901.863164	990.034616	-0.550829215	0.0003207	0.0021307	TXNDC15	thioredoxin domain containing 15 [Source:HGNC Symbol;Acc:HGNC:20652]
ENSG00000177030	486.202762	404.0215	692.20105	673.862271	-0.618810011	0.0003208	0.0021307	DEAF1	DEAF1, transcription factor [Source:HGNC Symbol;Acc:HGNC:14677]
ENSG00000101811	722.414631	738.048047	979.82797	1126.29744	-0.52801579	0.0003219	0.0021372	CSTF2	cleavage stimulation factor subunit 2 [Source:HGNC Symbol;Acc:HGNC:2484]
ENSG00000268879	368.096828	297.700053	178.05476	194.813263	0.835567253	0.0003226	0.0021409	IGFL1P1	IGF like family member 1 pseudogene 1 [Source:HGNC Symbol;Acc:HGNC:32956]
ENSG00000018699	317.901806	294.156005	487.806828	487.565434	-0.672805956	0.000323	0.002143	TTC27	tetratricopeptide repeat domain 27 [Source:HGNC Symbol;Acc:HGNC:25986]
ENSG00000170037	1067.87449	887.784086	1344.36612	1444.5989	-0.512730788	0.0003237	0.0021458	CNTROB	centrobin, centriole duplication and spindle assembly protein [Source:HGNC Symbol;Acc:HGNC:29616]
ENSG00000130921	625.961451	675.141191	891.327379	1007.06747	-0.544630731	0.0003237	0.0021458	C12orf65	chromosome 12 open reading frame 65 [Source:HGNC Symbol;Acc:HGNC:26784]
ENSG00000171703	1289.32311	1142.06955	808.094681	894.224814	0.513959297	0.0003241	0.002148	TCEA2	transcription elongation factor A2 [Source:HGNC Symbol;Acc:HGNC:11614]
ENSG00000243199	243.101381	189.606581	361.377413	389.626526	-0.797055484	0.0003252	0.0021539	AC115223.1	ribosomal protein L6 (RPL6) pseudogene
ENSG00000115594	205.701169	225.047064	105.357846	111.778102	0.988739775	0.0003259	0.0021573	IL1R1	interleukin 1 receptor type 1 [Source:HGNC Symbol;Acc:HGNC:5993]
ENSG00000173473	3346.3348	3572.40063	4871.74681	4280.56902	-0.403536953	0.0003259	0.0021573	SMARCC1	SWI/SNF related, matrix associated, actin dependent regulator of chromatin subfamily c member 1 [Source:HGNC Symbol;Acc:HGNC:11104]
ENSG00000205937	4714.39521	4314.87874	5609.25173	6222.31433	-0.390069307	0.0003261	0.0021573	RNPS1	RNA binding protein with serine rich domain 1 [Source:HGNC Symbol;Acc:HGNC:10080]
ENSG00000123933	1379.871	1280.28743	1837.44084	1776.73954	-0.442438178	0.0003268	0.0021611	MXD4	MAX dimerization protein 4 [Source:HGNC Symbol;Acc:HGNC:13906]
ENSG00000160862	21.6527546	13.2901809	0	0	6.485185742	0.000327	0.0021619	AZGP1	alpha-2-glycoprotein 1, zinc-binding [Source:HGNC Symbol;Acc:HGNC:910]
ENSG00000138621	229.322356	175.430388	341.359422	372.593673	-0.820550383	0.0003292	0.0021758	PPCDC	phosphopantothenoylcysteine decarboxylase [Source:HGNC Symbol;Acc:HGNC:28107]
ENSG00000118263	605.292913	674.255179	940.845567	916.580435	-0.53713438	0.0003297	0.0021777	KLF7	Kruppel like factor 7 [Source:HGNC Symbol;Acc:HGNC:6350]
ENSG00000168743	113.184854	142.647942	61.1075508	30.8720472	1.476048497	0.0003298	0.0021777	NPNT	nephronectin [Source:HGNC Symbol;Acc:HGNC:27405]
ENSG00000256732	22.6369707	12.4041689	0	0	6.488893837	0.0003314	0.002187	AC006065.4	uncharacterized LOC100996671 [Source:NCBI gene;Acc:100996671]
ENSG00000204574	2930.9956	2643.85999	3524.21996	3888.81339	-0.411319617	0.0003315	0.002187	ABCF1	ATP binding cassette subfamily F member 1 [Source:HGNC Symbol;Acc:HGNC:70]
ENSG00000167815	4191.77645	3927.69147	5010.81917	5653.84284	-0.393463677	0.0003325	0.002193	PRDX2	peroxiredoxin 2 [Source:HGNC Symbol;Acc:HGNC:9353]
ENSG00000166963	770.641221	677.799227	450.931582	503.533735	0.601320114	0.0003356	0.0022127	MAP1A	microtubule associated protein 1A [Source:HGNC Symbol;Acc:HGNC:6835]
ENSG00000260822	577.734861	688.431372	421.431385	392.820186	0.637545256	0.0003358	0.0022128	AC004656.1	novel transcript, overlapping to PDK3
ENSG00000255717	1285.38625	1306.86779	1669.92186	1885.32398	-0.455649358	0.000337	0.0022203	SNHG1	small nucleolar RNA host gene 1 [Source:HGNC Symbol;Acc:HGNC:32688]
ENSG00000124145	4842.3433	4459.29871	3397.79054	3699.32289	0.390158328	0.0003377	0.0022239	SDC4	syndecan 4 [Source:HGNC Symbol;Acc:HGNC:10661]
ENSG00000111077	676.156473	548.441466	871.309388	960.227122	-0.581653281	0.0003385	0.0022283	TNS2	tensin 2 [Source:HGNC Symbol;Acc:HGNC:19737]
ENSG00000129460	533.445136	507.684911	735.397767	809.060546	-0.56918274	0.0003391	0.0022314	NGDN	neuroguidin [Source:HGNC Symbol;Acc:HGNC:20271]
ENSG00000134825	1449.75034	1313.95589	1778.44044	2041.81333	-0.467334987	0.0003401	0.0022371	TMEM258	transmembrane protein 258 [Source:HGNC Symbol;Acc:HGNC:1164]
ENSG00000204138	1066.89027	1134.09544	1470.79553	1558.5061	-0.460571339	0.0003418	0.0022475	PHACTR4	phosphatase and actin regulator 4 [Source:HGNC Symbol;Acc:HGNC:25793]
ENSG00000147799	1188.93307	936.514749	726.969139	705.798871	0.568277447	0.0003431	0.0022552	ARHGAP39	Rho GTPase activating protein 39 [Source:HGNC Symbol;Acc:HGNC:29351]
ENSG00000109466	529.508272	536.923309	346.627314	336.398859	0.642819346	0.0003436	0.0022576	KLHL2	kelch like family member 2 [Source:HGNC Symbol;Acc:HGNC:6353]
ENSG00000181027	357.270451	306.560173	521.521339	530.147568	-0.664747179	0.0003451	0.0022661	FKRP	fukutin related protein [Source:HGNC Symbol;Acc:HGNC:17997]
ENSG00000100796	1407.42905	1608.9979	2060.79947	2047.13609	-0.445155635	0.0003452	0.0022665	PPP4R3A	protein phosphatase 4 regulatory subunit 3A [Source:HGNC Symbol;Acc:HGNC:20219]
ENSG00000168398	342.507209	310.990234	203.340643	162.876663	0.834885315	0.0003477	0.002282	BDKRB2	bradykinin receptor B2 [Source:HGNC Symbol;Acc:HGNC:1030]
ENSG00000164251	350.380938	316.306306	499.396191	555.696849	-0.662881559	0.0003481	0.0022838	F2RL1	F2R like trypsin receptor 1 [Source:HGNC Symbol;Acc:HGNC:3538]
ENSG00000173914	427.149795	489.96467	679.558108	708.992531	-0.597615385	0.0003509	0.0023012	RBM4B	RNA binding motif protein 4B [Source:HGNC Symbol;Acc:HGNC:28842]
ENSG00000164924	6989.90287	8090.17614	10344.0333	9453.23375	-0.392545623	0.000354	0.0023204	YWHAZ	tyrosine 3-monooxygenase/tryptophan 5-monooxygenase activation protein zeta [Source:HGNC Symbol;Acc:HGNC:12855]
ENSG00000172053	3570.73608	3117.87645	4351.27905	4467.93041	-0.399176759	0.0003543	0.0023215	QARS	glutaminyl-tRNA synthetase [Source:HGNC Symbol;Acc:HGNC:9751]
ENSG00000109861	4890.56989	5097.22739	3741.25712	3984.62319	0.370536761	0.0003547	0.0023232	CTSC	cathepsin C [Source:HGNC Symbol;Acc:HGNC:2528]
ENSG00000111605	1909.37927	1910.24201	2434.81983	2641.15686	-0.41023449	0.000356	0.0023309	CPSF6	cleavage and polyadenylation specific factor 6 [Source:HGNC Symbol;Acc:HGNC:13871]
ENSG00000064651	808.041433	916.136472	641.629284	438.59598	0.674727744	0.0003562	0.0023309	SLC12A2	solute carrier family 12 member 2 [Source:HGNC Symbol;Acc:HGNC:10911]
ENSG00000122257	1422.19229	1453.94579	1903.81628	1956.64906	-0.424554117	0.0003567	0.0023333	RBBP6	RB binding protein 6, ubiquitin ligase [Source:HGNC Symbol;Acc:HGNC:9889]
ENSG00000169045	7530.23752	6926.8423	9022.84595	9525.62338	-0.359603057	0.0003568	0.0023333	HNRNPH1	heterogeneous nuclear ribonucleoprotein H1 [Source:HGNC Symbol;Acc:HGNC:5041]
ENSG00000163808	707.651389	836.395386	1115.73959	1106.07093	-0.524293746	0.0003569	0.0023333	KIF15	kinesin family member 15 [Source:HGNC Symbol;Acc:HGNC:17273]
ENSG00000153914	756.862195	922.338556	1298.00867	1146.52396	-0.541076733	0.0003582	0.0023406	SREK1	splicing regulatory glutamic acid and lysine rich protein 1 [Source:HGNC Symbol;Acc:HGNC:17882]
ENSG00000275700	1834.57884	1686.08095	2234.63992	2544.28251	-0.441040143	0.0003588	0.0023434	AATF	apoptosis antagonizing transcription factor [Source:HGNC Symbol;Acc:HGNC:19235]
ENSG00000164300	553.129458	606.03225	401.413394	334.269752	0.656166047	0.0003589	0.0023434	SERINC5	serine incorporator 5 [Source:HGNC Symbol;Acc:HGNC:18825]

ENSG00000185475	497.02914	567.04772	290.787656	365.141799	0.698627443	0.0003605	0.0023526	TMEM179B	transmembrane protein 179B [Source:HGNC Symbol;Acc:HGNC:33744]
ENSG00000125538	54.1318865	56.704772	15.8036769	7.45187345	2.251909784	0.0003609	0.0023547	IL1B	interleukin 1 beta [Source:HGNC Symbol;Acc:HGNC:5992]
ENSG00000102178	1114.13265	950.690942	1447.61681	1445.66345	-0.487287928	0.0003628	0.0023658	UBL4A	ubiquitin like 4A [Source:HGNC Symbol;Acc:HGNC:12505]
ENSG00000067992	989.137199	1051.69632	746.98713	675.991377	0.52040561	0.0003629	0.002366	PDK3	pyruvate dehydrogenase kinase 3 [Source:HGNC Symbol;Acc:HGNC:8811]
ENSG00000133657	2399.5189	2753.72549	2055.53158	1395.62944	0.578481249	0.000364	0.0023717	ATP13A3	ATPase 13A3 [Source:HGNC Symbol;Acc:HGNC:24113]
ENSG00000164010	86.6110184	80.6270976	171.733289	180.97407	-1.077192524	0.0003641	0.0023717	ERMAP	erythroblast membrane associated protein (Scianna blood group) [Source:HGNC Symbol;Acc:HGNC:15743]
ENSG00000163565	762.767492	980.815352	1322.24097	1229.55912	-0.548598484	0.0003664	0.0023859	IFI16	interferon gamma inducible protein 16 [Source:HGNC Symbol;Acc:HGNC:5395]
ENSG00000154473	2046.18531	2437.41918	3207.09284	2904.10154	-0.446449215	0.0003693	0.0024038	BUB3	BUB3, mitotic checkpoint protein [Source:HGNC Symbol;Acc:HGNC:1151]
ENSG00000141738	399.591744	341.114644	551.021536	608.924516	-0.647969488	0.000371	0.0024139	GRB7	growth factor receptor bound protein 7 [Source:HGNC Symbol;Acc:HGNC:4567]
ENSG00000279495	39.3686447	31.0104222	96.9292185	101.132568	-1.495294436	0.0003718	0.0024184	AL928654.4	novel transcript
ENSG00000111087	135.821824	153.280087	60.0539724	64.9377544	1.210413714	0.0003733	0.0024272	GLI1	GLI family zinc finger 1 [Source:HGNC Symbol;Acc:HGNC:4317]
ENSG00000118007	886.778722	1130.55139	1619.3501	1347.72454	-0.555919964	0.0003748	0.0024356	STAG1	stromal antigen 1 [Source:HGNC Symbol;Acc:HGNC:11354]
ENSG00000182179	50.195022	44.3006031	113.786474	124.552742	-1.3359275	0.0003754	0.0024389	UBA7	ubiquitin like modifier activating enzyme 7 [Source:HGNC Symbol;Acc:HGNC:12471]
ENSG00000214655	1760.76264	1492.04431	1204.24018	1118.84557	0.485204929	0.0003759	0.0024409	ZSWIM8	zinc finger SWIM-type containing 8 [Source:HGNC Symbol;Acc:HGNC:23528]
ENSG00000115234	1543.25087	1426.47942	1926.99501	2093.97644	-0.437445906	0.000376	0.0024409	SNX17	sorting nexin 17 [Source:HGNC Symbol;Acc:HGNC:14979]
ENSG00000197905	933.03688	785.006687	1207.40092	1241.26921	-0.511928139	0.0003796	0.0024632	TEAD4	TEA domain transcription factor 4 [Source:HGNC Symbol;Acc:HGNC:11717]
ENSG00000205581	3662.26818	4259.05998	5335.32133	5114.11429	-0.399394805	0.0003803	0.0024663	HMGN1	high mobility group nucleosome binding domain 1 [Source:HGNC Symbol;Acc:HGNC:4984]
ENSG00000105141	232.275004	256.057486	118.000788	138.391936	0.930107923	0.0003804	0.0024663	CASP14	caspase 14 [Source:HGNC Symbol;Acc:HGNC:1502]
ENSG00000176723	79.7215056	64.6788805	25.2858831	12.7746402	1.922004465	0.0003809	0.0024688	ZNF843	zinc finger protein 843 [Source:HGNC Symbol;Acc:HGNC:28710]
ENSG00000100462	1164.32767	1064.9865	1550.8675	1509.53665	-0.45748325	0.0003832	0.002483	PRMT5	protein arginine methyltransferase 5 [Source:HGNC Symbol;Acc:HGNC:10894]
ENSG00000046651	666.314312	677.799227	990.363755	931.484181	-0.515776941	0.0003841	0.0024875	OFD1	OFD1, centriole and centriolar satellite protein [Source:HGNC Symbol;Acc:HGNC:2567]
ENSG00000183032	6.88951283	2.65803619	40.0359816	33.0011539	-2.951542347	0.0003879	0.0025117	SLC25A21	solute carrier family 25 member 21 [Source:HGNC Symbol;Acc:HGNC:14411]
ENSG00000106397	3372.90864	2816.63234	4118.43821	4157.08083	-0.419321635	0.0003888	0.0025162	PLOD3	procollagen-lysine,2-oxoglutarate 5-dioxygenase 3 [Source:HGNC Symbol;Acc:HGNC:9083]
ENSG00000175283	602.340264	529.835213	913.452527	777.123946	-0.579094152	0.00039	0.0025232	DOLK	dolichol kinase [Source:HGNC Symbol;Acc:HGNC:23406]
ENSG00000179115	2391.64517	2132.63103	2947.91254	3055.26812	-0.408312784	0.0003921	0.0025356	FARSA	phenylalanyl-tRNA synthetase subunit alpha [Source:HGNC Symbol;Acc:HGNC:3592]
ENSG00000111196	359.238883	310.104222	525.735653	528.018462	-0.655642585	0.0003931	0.0025414	MAGOHB	mago homolog B, exon junction complex subunit [Source:HGNC Symbol;Acc:HGNC:25504]
ENSG00000142230	3580.57824	3353.55565	4380.77925	4603.12869	-0.373743957	0.0003981	0.0025727	SAE1	SUMO1 activating enzyme subunit 1 [Source:HGNC Symbol;Acc:HGNC:30660]
ENSG00000158555	1359.20246	1248.391	986.149441	861.22366	0.496933971	0.0003987	0.0025757	GDPD5	glycerophosphodiester phosphodiesterase domain containing 5 [Source:HGNC Symbol;Acc:HGNC:28804]
ENSG00000000971	29.5264835	31.0104222	2.10715692	3.19366005	3.514653104	0.0003996	0.00258	CFH	complement factor H [Source:HGNC Symbol;Acc:HGNC:4883]
ENSG00000112419	350.380938	422.627753	256.019566	170.328536	0.858833968	0.0003998	0.0025807	PHACTR2	phosphatase and actin regulator 2 [Source:HGNC Symbol;Acc:HGNC:20956]
ENSG00000111716	1154.48551	1299.77969	1778.44044	1611.73377	-0.46568891	0.0004016	0.0025905	LDHB	lactate dehydrogenase B [Source:HGNC Symbol;Acc:HGNC:6541]
ENSG00000110696	1597.38276	1688.73899	2135.60354	2251.53034	-0.416699706	0.0004016	0.0025905	C11orf58	chromosome 11 open reading frame 58 [Source:HGNC Symbol;Acc:HGNC:16990]
ENSG00000186501	518.681894	484.648598	705.89757	786.704926	-0.573360184	0.0004019	0.0025909	TMEM222	transmembrane protein 222 [Source:HGNC Symbol;Acc:HGNC:25363]
ENSG00000114735	369.081044	371.239054	221.251477	221.427097	0.741917972	0.0004029	0.0025968	HEMK1	HemK methyltransferase family member 1 [Source:HGNC Symbol;Acc:HGNC:24923]
ENSG00000173193	704.698741	874.493905	1282.20499	1061.35969	-0.568802172	0.0004042	0.002604	PARP14	poly(ADP-ribose) polymerase family member 14 [Source:HGNC Symbol;Acc:HGNC:29232]
ENSG00000137094	417.307634	387.187271	213.876428	264.009231	0.751250737	0.0004054	0.0026109	DNAJB5	DnaJ heat shock protein family (Hsp40) member B5 [Source:HGNC Symbol;Acc:HGNC:14887]
ENSG00000106258	35.4317803	46.0726272	6.32147077	6.3873201	2.682175493	0.0004057	0.0026114	CYP3A5	cytochrome P450 family 3 subfamily A member 5 [Source:HGNC Symbol;Acc:HGNC:2638]
ENSG00000158769	956.658067	936.514749	546.807222	715.379851	0.584942162	0.0004075	0.0026223	F11R	F11 receptor [Source:HGNC Symbol;Acc:HGNC:14685]
ENSG00000131069	912.368342	838.16741	601.593302	606.79541	0.534394213	0.0004081	0.0026249	ACSS2	acyl-CoA synthetase short chain family member 2 [Source:HGNC Symbol;Acc:HGNC:15814]
ENSG00000119185	1149.56443	1104.85704	1520.31372	1548.92512	-0.445279334	0.0004089	0.0026294	ITGB1BP1	integrin subunit beta 1 binding protein 1 [Source:HGNC Symbol;Acc:HGNC:23927]
ENSG00000139239	336.601912	279.093799	442.502954	580.181576	-0.732985849	0.0004091	0.0026295	RPL14P1	ribosomal protein L14 pseudogene 1 [Source:HGNC Symbol;Acc:HGNC:31384]
ENSG00000117643	129.916528	108.979484	46.3574523	47.9049008	1.340823759	0.0004104	0.0026361	MAN1C1	mannosidase alpha class 1C member 1 [Source:HGNC Symbol;Acc:HGNC:19080]
ENSG00000109756	1568.84049	1510.65057	1177.90072	1061.35969	0.459515906	0.0004104	0.0026361	RAPGEF2	Rap guanine nucleotide exchange factor 2 [Source:HGNC Symbol;Acc:HGNC:16854]
ENSG00000169679	1568.84049	1803.03455	2369.49796	2205.75454	-0.439928111	0.0004124	0.0026478	BUB1	BUB1 mitotic checkpoint serine/threonine kinase [Source:HGNC Symbol;Acc:HGNC:1148]
ENSG00000104312	493.092275	498.824791	341.359422	272.525658	0.692147124	0.0004137	0.0026549	RIPK2	receptor interacting serine/threonine kinase 2 [Source:HGNC Symbol;Acc:HGNC:10020]
ENSG00000134042	13.7790257	19.4922654	0	0	6.417291179	0.0004143	0.0026582	MRO	maestro [Source:HGNC Symbol;Acc:HGNC:24121]
ENSG00000171169	162.39566	146.19199	278.144714	277.848424	-0.85021974	0.0004147	0.0026596	NAIF1	nuclear apoptosis inducing factor 1 [Source:HGNC Symbol;Acc:HGNC:25446]
ENSG00000138622	53.1476704	53.1607237	12.6429415	10.6455335	2.190303762	0.000415	0.0026605	HCN4	hyperpolarization activated cyclic nucleotide gated potassium channel 4 [Source:HGNC Symbol;Acc:HGNC:16882]
ENSG00000137509	2427.07695	2564.11891	1901.70912	1877.87211	0.401282264	0.0004156	0.0026634	PRCP	prolylcarboxypeptidase [Source:HGNC Symbol;Acc:HGNC:9344]
ENSG00000106665	1427.11337	1274.08534	953.488508	985.776402	0.477759359	0.0004161	0.0026649	CLIP2	CAP-Gly domain containing linker protein 2 [Source:HGNC Symbol;Acc:HGNC:2586]
ENSG00000167967	872.015481	787.664723	1110.4717	1245.52742	-0.505829154	0.0004162	0.0026649	E4F1	E4F transcription factor 1 [Source:HGNC Symbol;Acc:HGNC:3121]

ENSG00000140854	1818.83139	1515.08063	1191.59724	1197.62252	0.480234718	0.000417	0.0026681	KATNB1	katanin regulatory subunit B1 [Source:HGNC Symbol;Acc:HGNC:6217]
ENSG00000147041	146.648202	164.798243	83.2326985	38.3239206	1.357133966	0.0004171	0.0026681	SYTL5	synaptotagmin like 5 [Source:HGNC Symbol;Acc:HGNC:15589]
ENSG00000160256	898.589316	738.048047	1131.54327	1229.55912	-0.529476875	0.0004172	0.0026681	FAM207A	family with sequence similarity 207 member A [Source:HGNC Symbol;Acc:HGNC:15811]
ENSG00000171365	403.528608	436.803946	265.501773	249.105484	0.707802845	0.000419	0.002679	CLCN5	chloride voltage-gated channel 5 [Source:HGNC Symbol;Acc:HGNC:2023]
ENSG00000163697	564.940052	528.949201	368.752462	334.269752	0.637473949	0.0004244	0.0027123	APBB2	amyloid beta precursor protein binding family B member 2 [Source:HGNC Symbol;Acc:HGNC:582]
ENSG00000118181	6554.87935	6767.36013	7973.4818	9568.20551	-0.396909724	0.0004259	0.002721	RPS25	ribosomal protein S25 [Source:HGNC Symbol;Acc:HGNC:10413]
ENSG00000069329	2134.76476	2505.64211	3285.05765	2981.81393	-0.433176784	0.0004267	0.0027252	VPS35	VPS35, retromer complex component [Source:HGNC Symbol;Acc:HGNC:13487]
ENSG00000169992	2519.59326	2053.77596	1659.38608	1657.50957	0.463037942	0.0004285	0.0027356	NLGN2	neuroligin 2 [Source:HGNC Symbol;Acc:HGNC:14290]
ENSG00000115524	4267.56109	4827.87972	6092.84425	5758.16907	-0.381632128	0.0004296	0.0027416	SF3B1	splicing factor 3b subunit 1 [Source:HGNC Symbol;Acc:HGNC:10768]
ENSG00000243742	33.463348	30.1244101	4.21431385	2.1291067	3.323575581	0.00043	0.0027429	RPLP0P2	ribosomal protein lateral stalk subunit P0 pseudogene 2 [Source:HGNC Symbol;Acc:HGNC:17960]
ENSG00000233251	111.216421	135.559845	53.7325016	44.7112407	1.32655926	0.0004312	0.0027493	AC007743.1	uncharacterized LOC100129434 [Source:NCBI gene;Acc:100129434]
ENSG00000118363	1084.60616	1226.24069	1558.24255	1621.31475	-0.459938321	0.0004319	0.0027533	SPCS2	signal peptidase complex subunit 2 [Source:HGNC Symbol;Acc:HGNC:28962]
ENSG00000232774	142.711337	175.430388	295.001969	281.042084	-0.854981761	0.0004326	0.0027557	FLJ22447	uncharacterized LOC400221 [Source:NCBI gene;Acc:400221]
ENSG00000113648	4491.96236	4365.38143	5392.21457	6149.9247	-0.381989029	0.0004327	0.0027557	H2AFY	H2A histone family member Y [Source:HGNC Symbol;Acc:HGNC:4740]
ENSG00000075415	10425.8013	10502.787	12617.6557	14107.461	-0.352709313	0.0004353	0.0027718	SLC25A3	solute carrier family 25 member 3 [Source:HGNC Symbol;Acc:HGNC:10989]
ENSG00000144036	1634.78297	1615.19999	1242.16901	1147.58851	0.443499071	0.0004356	0.0027723	EXOC6B	exocyst complex component 6B [Source:HGNC Symbol;Acc:HGNC:17085]
ENSG00000139160	84.6425862	93.9172785	25.2858831	34.0657072	1.589885325	0.0004388	0.0027916	ETFBKMT	electron transfer flavoprotein subunit beta lysine methyltransferase [Source:HGNC Symbol;Acc:HGNC:28739]
ENSG00000272767	117.121718	115.181568	41.08956	50.0340075	1.350249641	0.0004394	0.0027943	JMJD1C-AS1	JMJD1C antisense RNA 1 [Source:HGNC Symbol;Acc:HGNC:28222]
ENSG00000175573	422.228715	354.404825	596.32541	602.537196	-0.627376512	0.0004396	0.0027949	C11orf68	chromosome 11 open reading frame 68 [Source:HGNC Symbol;Acc:HGNC:28801]
ENSG00000147526	1863.12111	2053.77596	1480.27774	1423.30783	0.432107652	0.0004415	0.002806	TACC1	transforming acidic coiled-coil containing protein 1 [Source:HGNC Symbol;Acc:HGNC:11522]
ENSG00000168056	1123.97481	932.084689	1391.77715	1513.79486	-0.4995729	0.0004423	0.0028101	LTBP3	latent transforming growth factor beta binding protein 3 [Source:HGNC Symbol;Acc:HGNC:6716]
ENSG00000117122	96.4531796	69.9949529	187.536966	169.263983	-1.10281078	0.0004432	0.0028141	MFAP2	microfibril associated protein 2 [Source:HGNC Symbol;Acc:HGNC:7033]
ENSG00000165474	116.137502	126.699725	56.893237	37.2593673	1.366866783	0.000444	0.0028182	GJB2	gap junction protein beta 2 [Source:HGNC Symbol;Acc:HGNC:4284]
ENSG00000088899	397.623312	286.181896	190.697702	189.490496	0.845588403	0.0004458	0.0028283	LZTS3	leucine zipper tumor suppressor family member 3 [Source:HGNC Symbol;Acc:HGNC:30139]
ENSG00000134109	2326.6869	2348.81798	1811.10138	1725.64098	0.402707164	0.0004459	0.0028283	EDEM1	ER degradation enhancing alpha-mannosidase like protein 1 [Source:HGNC Symbol;Acc:HGNC:18967]
ENSG00000169991	450.770982	400.477452	251.805252	269.331998	0.707437252	0.0004484	0.0028431	IFFO2	intermediate filament family orphan 2 [Source:HGNC Symbol;Acc:HGNC:27006]
ENSG00000111667	3333.53999	2798.9121	4004.65174	4168.79092	-0.41477328	0.0004488	0.0028446	USP5	ubiquitin specific peptidase 5 [Source:HGNC Symbol;Acc:HGNC:12628]
ENSG00000166716	1566.87206	1370.66066	2062.90663	1933.22888	-0.444432771	0.0004497	0.002849	ZNF592	zinc finger protein 592 [Source:HGNC Symbol;Acc:HGNC:28986]
ENSG00000204272	1532.4245	1533.68688	986.149441	1199.75163	0.488258615	0.0004503	0.002852	NBDY	negative regulator of P-body association [Source:HGNC Symbol;Acc:HGNC:50713]
ENSG00000174903	1661.35681	1495.58836	2136.65712	2098.23465	-0.424134258	0.0004517	0.0028599	RAB1B	RAB1B, member RAS oncogene family [Source:HGNC Symbol;Acc:HGNC:18370]
ENSG00000213462	131.88496	155.052111	64.2682862	60.679541	1.200129199	0.0004531	0.0028674	ERV3-1	endogenous retrovirus group 3 member 1, envelope [Source:HGNC Symbol;Acc:HGNC:3454]
ENSG00000164742	163.379876	139.989906	72.6969139	61.7440943	1.173204196	0.0004536	0.0028698	ADCY1	adenylate cyclase 1 [Source:HGNC Symbol;Acc:HGNC:232]
ENSG00000132357	163.379876	165.684256	271.823243	314.043238	-0.832021113	0.0004542	0.0028718	CARD6	caspase recruitment domain family member 6 [Source:HGNC Symbol;Acc:HGNC:16394]
ENSG00000170962	64.9582638	76.1970373	18.9644123	21.291067	1.810915051	0.0004543	0.0028718	PDGFD	platelet derived growth factor D [Source:HGNC Symbol;Acc:HGNC:30620]
ENSG00000103653	2332.5922	2146.80723	2881.53709	3011.62143	-0.395939233	0.0004547	0.0028736	CSK	C-terminal Src kinase [Source:HGNC Symbol;Acc:HGNC:2444]
ENSG00000107960	303.138564	316.306306	161.197505	190.55505	0.81674873	0.0004556	0.0028779	STN1	STN1, CST complex subunit [Source:HGNC Symbol;Acc:HGNC:26200]
ENSG00000081189	156.490363	139.103894	49.5181877	75.5832879	1.240407654	0.0004558	0.0028783	MEF2C	myocyte enhancer factor 2C [Source:HGNC Symbol;Acc:HGNC:6996]
ENSG00000136327	117.121718	100.119363	45.3038739	36.1948139	1.413461919	0.0004565	0.0028817	NKX2-8	NK2 homeobox 8 [Source:HGNC Symbol;Acc:HGNC:16364]
ENSG00000132570	87.5952345	100.119363	185.429809	195.877816	-1.02121556	0.0004573	0.0028853	PCBD2	pterin-4 alpha-carbinolamine dehydratase 2 [Source:HGNC Symbol;Acc:HGNC:24474]
ENSG00000147804	222.432843	212.642895	339.252265	394.949293	-0.755152484	0.0004575	0.0028854	SLC39A4	solute carrier family 39 member 4 [Source:HGNC Symbol;Acc:HGNC:17129]
ENSG00000162869	549.192594	660.078986	411.949179	361.948139	0.644531258	0.0004577	0.0028857	PPP1R21	protein phosphatase 1 regulatory subunit 21 [Source:HGNC Symbol;Acc:HGNC:30595]
ENSG00000113369	1129.8801	1372.43268	1915.40564	1631.96029	-0.502972605	0.0004588	0.0028918	ARRDC3	arrestin domain containing 3 [Source:HGNC Symbol;Acc:HGNC:29263]
ENSG00000271122	348.412506	319.850354	172.786868	210.781563	0.800704166	0.0004601	0.002899	AC018647.2	uncharacterized LOC101930085 [Source:NCBI gene;Acc:101930085]
ENSG00000214026	312.980726	314.534282	422.484963	646.183884	-0.767932274	0.000462	0.0029096	MRPL23	mitochondrial ribosomal protein L23 [Source:HGNC Symbol;Acc:HGNC:10322]
ENSG00000223496	992.089847	910.8204	1235.84754	1446.728	-0.49568934	0.0004673	0.0029419	EXOSC6	exosome component 6 [Source:HGNC Symbol;Acc:HGNC:19055]
ENSG00000086061	4922.06481	5422.39382	6526.91857	6771.62386	-0.362277487	0.0004678	0.0029439	DNAJA1	DnaJ heat shock protein family (Hsp40) member A1 [Source:HGNC Symbol;Acc:HGNC:5229]
ENSG00000169957	715.525118	654.762914	906.077478	1094.36084	-0.546159169	0.0004687	0.0029484	ZNF768	zinc finger protein 768 [Source:HGNC Symbol;Acc:HGNC:26273]
ENSG00000248487	52.1634543	60.2488202	121.161523	145.843809	-1.246587967	0.0004694	0.0029518	ABHD14A	abhydrolase domain containing 14A [Source:HGNC Symbol;Acc:HGNC:24538]

ENSG00000106771	2410.34527	2629.6838	2034.46001	1494.6329	0.514188244	0.0004702	0.0029559	TMEM245	transmembrane protein 245 [Source:HGNC Symbol;Acc:HGNC:1363]
ENSG00000181284	371.049477	367.695006	208.608536	233.137184	0.741904047	0.0004706	0.0029572	TMEM102	transmembrane protein 102 [Source:HGNC Symbol;Acc:HGNC:26722]
ENSG00000149557	25.5896191	45.1866152	3.16073539	5.32276675	3.063670751	0.0004715	0.0029616	FEZ1	fasciculation and elongation protein zeta 1 [Source:HGNC Symbol;Acc:HGNC:3659]
ENSG00000105538	1201.72788	1040.17816	1516.09941	1581.92628	-0.467137123	0.0004722	0.0029654	RASIP1	Ras interacting protein 1 [Source:HGNC Symbol;Acc:HGNC:24716]
ENSG00000105974	2148.54379	2367.42423	2923.68023	3023.33151	-0.396878204	0.0004734	0.0029715	CAV1	caveolin 1 [Source:HGNC Symbol;Acc:HGNC:1527]
ENSG00000151914	4892.53832	5222.15509	4092.09875	3690.80647	0.37813503	0.0004778	0.002998	DST	dystonin [Source:HGNC Symbol;Acc:HGNC:1090]
ENSG00000166226	5798.01715	6267.64933	7547.8361	7848.95185	-0.351624083	0.0004794	0.0030072	CCT2	chaperonin containing TCP1 subunit 2 [Source:HGNC Symbol;Acc:HGNC:1615]
ENSG00000158715	432.070876	356.176849	258.126723	198.006923	0.78827199	0.0004796	0.0030072	SLC45A3	solute carrier family 45 member 3 [Source:HGNC Symbol;Acc:HGNC:8642]
ENSG00000140474	821.820459	779.690614	1079.91792	1166.75047	-0.48856859	0.0004822	0.0030222	ULK3	unc-51 like kinase 3 [Source:HGNC Symbol;Acc:HGNC:19703]
ENSG00000133315	608.245561	563.503671	834.434142	858.03	-0.530858803	0.0004825	0.0030232	MACROD1	MACRO domain containing 1 [Source:HGNC Symbol;Acc:HGNC:29598]
ENSG00000162694	422.228715	571.47778	326.609323	264.009231	0.751467648	0.0004863	0.0030443	EXTL2	exostosin like glycosyltransferase 2 [Source:HGNC Symbol;Acc:HGNC:3516]
ENSG00000181690	79.7215056	124.927701	41.08956	28.7429405	1.552607257	0.0004864	0.0030443	PLAG1	PLAG1 zinc finger [Source:HGNC Symbol;Acc:HGNC:9045]
ENSG00000114391	5387.59903	5714.7778	6579.5975	8452.5536	-0.437115825	0.0004864	0.0030443	RPL24	ribosomal protein L24 [Source:HGNC Symbol;Acc:HGNC:10325]
ENSG00000142453	1772.57323	1632.03422	2257.81864	2263.24042	-0.409429698	0.0004868	0.0030453	CARM1	coactivator associated arginine methyltransferase 1 [Source:HGNC Symbol;Acc:HGNC:23393]
ENSG00000253276	673.203825	610.462311	897.64885	945.323375	-0.522237415	0.0004873	0.0030474	CCDC71L	coiled-coil domain containing 71 like [Source:HGNC Symbol;Acc:HGNC:26685]
ENSG00000247271	202.74852	225.933076	94.8220616	121.359082	0.988381002	0.0004882	0.0030519	ZBED5-AS1	ZBED5 antisense RNA 1 [Source:HGNC Symbol;Acc:HGNC:48646]
ENSG00000149273	25577.8085	22519.7686	29532.8579	32275.1285	-0.361863079	0.0004908	0.0030671	RPS3	ribosomal protein S3 [Source:HGNC Symbol;Acc:HGNC:10420]
ENSG00000168675	88.5794506	96.5753147	36.8752462	26.6138338	1.544169629	0.000491	0.0030674	LDLRAD4	low density lipoprotein receptor class A domain containing 4 [Source:HGNC Symbol;Acc:HGNC:1224]
ENSG00000167186	169.285172	185.176521	302.377019	314.043238	-0.797578638	0.0004943	0.0030865	COQ7	coenzyme Q7, hydroxylase [Source:HGNC Symbol;Acc:HGNC:2244]
ENSG00000052841	908.431477	934.742725	1242.16901	1298.75509	-0.463016515	0.0004949	0.0030892	TTC17	tetratricopeptide repeat domain 17 [Source:HGNC Symbol;Acc:HGNC:25596]
ENSG00000173530	185.03263	179.860449	331.877216	299.139491	-0.790473499	0.0005005	0.0031232	TNFRSF10D	TNF receptor superfamily member 10d [Source:HGNC Symbol;Acc:HGNC:11907]
ENSG00000164744	28.5422674	31.8964342	1.05357846	4.2582134	3.511301523	0.0005011	0.0031259	SUN3	Sad1 and UNC84 domain containing 3 [Source:HGNC Symbol;Acc:HGNC:22429]
ENSG00000227097	4815.76947	4423.85822	5824.18174	6029.63018	-0.359566453	0.000503	0.0031362	RPS28P7	ribosomal protein S28 pseudogene 7 [Source:HGNC Symbol;Acc:HGNC:35787]
ENSG00000141441	311.012293	318.07833	195.965594	163.941216	0.805604477	0.0005041	0.003142	GAREM1	GRB2 associated regulator of MAPK1 subtype 1 [Source:HGNC Symbol;Acc:HGNC:26136]
ENSG00000130529	292.312187	230.363136	153.822455	120.294529	0.929932682	0.0005053	0.0031486	TRPM4	transient receptor potential cation channel subfamily M member 4 [Source:HGNC Symbol;Acc:HGNC:17993]
ENSG00000137628	141.727121	213.528907	401.413394	278.912978	-0.935248957	0.0005081	0.0031645	DDX60	DExD/H-box helicase 60 [Source:HGNC Symbol;Acc:HGNC:25942]
ENSG00000109689	653.519502	707.037625	433.020748	476.919901	0.580730155	0.0005084	0.0031651	STIM2	stromal interaction molecule 2 [Source:HGNC Symbol;Acc:HGNC:19205]
ENSG00000110852	160.427227	232.13516	361.377413	339.592519	-0.834152242	0.0005088	0.003167	CLEC2B	C-type lectin domain family 2 member B [Source:HGNC Symbol;Acc:HGNC:2053]
ENSG00000185347	569.861132	513.000984	802.826788	778.188499	-0.546584393	0.0005099	0.0031724	TEDC1	tubulin epsilon and delta complex 1 [Source:HGNC Symbol;Acc:HGNC:20127]
ENSG00000168461	2890.64274	3314.57112	2404.26605	2263.24042	0.411048429	0.0005102	0.0031734	RAB31	RAB31, member RAS oncogene family [Source:HGNC Symbol;Acc:HGNC:9771]
ENSG00000165801	1452.70299	1282.94547	1009.32817	960.227122	0.473622571	0.000511	0.0031768	ARHGEF40	Rho guanine nucleotide exchange factor 40 [Source:HGNC Symbol;Acc:HGNC:25516]
ENSG00000104290	264.754136	342.000656	180.161917	154.360236	0.859961902	0.0005116	0.0031791	FZD3	frizzled class receptor 3 [Source:HGNC Symbol;Acc:HGNC:4041]
ENSG00000076242	1097.40097	1037.52012	1460.25975	1446.728	-0.445579003	0.0005117	0.0031791	MLH1	mutL homolog 1 [Source:HGNC Symbol;Acc:HGNC:7127]
ENSG00000105341	591.513887	532.493249	788.07669	851.64268	-0.545316436	0.0005127	0.0031841	DMAC2	distal membrane arm assembly complex 2 [Source:HGNC Symbol;Acc:HGNC:25496]
ENSG00000146411	61.0213993	47.8446513	7.37504924	15.9683003	2.221552986	0.0005151	0.0031978	SLC2A12	solute carrier family 2 member 12 [Source:HGNC Symbol;Acc:HGNC:18067]
ENSG00000188818	171.253605	93.0312665	46.3574523	53.2276675	1.406000924	0.0005171	0.003208	ZDHHC11	zinc finger DHHC-type containing 11 [Source:HGNC Symbol;Acc:HGNC:19158]
ENSG00000172348	19.6843224	12.4041689	0	0	6.362321331	0.0005171	0.003208	RCAN2	regulator of calcineurin 2 [Source:HGNC Symbol;Acc:HGNC:3041]
ENSG00000180354	495.060707	487.306634	338.198686	277.848424	0.673022208	0.0005183	0.0032138	MTURN	maturin, neural progenitor differentiation regulator homolog [Source:HGNC Symbol;Acc:HGNC:25457]
ENSG00000100401	4615.97359	3792.13162	5568.16217	5585.71143	-0.407940143	0.0005189	0.0032166	RANGAP1	Ran GTPase activating protein 1 [Source:HGNC Symbol;Acc:HGNC:9854]
ENSG00000100425	834.615268	822.219193	1135.75758	1162.49226	-0.472168261	0.0005191	0.0032166	BRD1	bromodomain containing 1 [Source:HGNC Symbol;Acc:HGNC:1102]
ENSG00000233006	107.279557	120.49764	34.7680893	52.1631142	1.390626399	0.0005209	0.0032265	MIR3936HG	MIR3936 host gene [Source:HGNC Symbol;Acc:HGNC:40538]
ENSG00000106992	846.425862	679.571251	408.788443	554.632295	0.663056716	0.0005212	0.0032271	AK1	adenylate kinase 1 [Source:HGNC Symbol;Acc:HGNC:361]
ENSG00000066468	34.4475641	23.0363136	0	1.06455335	5.760335653	0.0005214	0.0032275	FGFR2	fibroblast growth factor receptor 2 [Source:HGNC Symbol;Acc:HGNC:3689]
ENSG00000103978	561.003187	689.317384	883.95233	957.033462	-0.557228414	0.0005226	0.0032337	TMEM87A	transmembrane protein 87A [Source:HGNC Symbol;Acc:HGNC:24522]
ENSG00000165685	856.268023	993.219521	1460.25975	1198.68707	-0.523270048	0.0005229	0.0032341	TMEM52B	transmembrane protein 52B [Source:HGNC Symbol;Acc:HGNC:26438]
ENSG00000007923	783.43603	699.063517	1049.36415	1046.45594	-0.500022608	0.0005233	0.0032347	DNAJC11	DnaJ heat shock protein family (Hsp40) member C11 [Source:HGNC Symbol;Acc:HGNC:25570]
ENSG00000251151	20.6685385	12.4041689	63.2147077	62.8086477	-1.936328726	0.0005235	0.0032347	HOXC-AS3	HOXC cluster antisense RNA 3 [Source:HGNC Symbol;Acc:HGNC:43751]
ENSG00000131389	6110.99788	5486.18669	4528.28023	4471.12407	0.365746971	0.0005236	0.0032347	SLC6A6	solute carrier family 6 member 6 [Source:HGNC Symbol;Acc:HGNC:11052]
ENSG00000170852	685.014418	846.141519	1126.27538	1083.71531	-0.528570079	0.000524	0.0032361	KBTBD2	kelch repeat and BTB domain containing 2 [Source:HGNC Symbol;Acc:HGNC:21751]
ENSG00000114346	1091.49568	1547.86307	2250.4436	1736.28651	-0.594402064	0.0005243	0.003237	ECT2	epithelial cell transforming 2 [Source:HGNC Symbol;Acc:HGNC:3155]
ENSG00000227500	760.799059	645.016781	973.506499	1051.77871	-0.527427908	0.0005249	0.0032397	SCAMP4	secretory carrier membrane protein 4 [Source:HGNC Symbol;Acc:HGNC:30385]

ENSG00000001617	2271.5708	2067.06614	1616.18936	1638.34761	0.414566502	0.0005268	0.0032499	SEMA3F	semaphorin 3F [Source:HGNC Symbol;Acc:HGNC:10728]
ENSG00000176022	770.641221	706.151613	1012.4889	1073.06978	-0.498360852	0.0005271	0.003251	B3GALT6	beta-1,3-galactosyltransferase 6 [Source:HGNC Symbol;Acc:HGNC:17978]
ENSG00000119392	1704.66232	1688.73899	2164.05016	2330.30728	-0.405392557	0.0005302	0.0032688	GLE1	GLE1, RNA export mediator [Source:HGNC Symbol;Acc:HGNC:4315]
ENSG00000071054	3175.0812	2937.12998	3948.81208	3950.55748	-0.37020709	0.0005306	0.0032697	MAP4K4	mitogen-activated protein kinase kinase kinase kinase 4 [Source:HGNC Symbol;Acc:HGNC:6866]
ENSG00000256542	53.1476704	49.6166755	127.482994	119.229975	-1.264219393	0.0005311	0.0032716	AC148477.2	novel transcript
ENSG00000052850	607.261345	557.301587	842.86277	836.738933	-0.528792996	0.0005326	0.0032799	ALX4	ALX homeobox 4 [Source:HGNC Symbol;Acc:HGNC:450]
ENSG00000162670	14.7632418	16.8342292	0	0	6.342092771	0.0005364	0.0033021	BRINP3	BMP/retinoic acid inducible neural specific 3 [Source:HGNC Symbol;Acc:HGNC:22393]
ENSG00000111799	10120.6943	10704.7977	14772.2236	12381.82	-0.382792853	0.0005378	0.0033097	COL12A1	collagen type XII alpha 1 chain [Source:HGNC Symbol;Acc:HGNC:2188]
ENSG00000140526	2320.78161	2331.09773	1865.88746	1576.60351	0.434319399	0.0005383	0.0033116	ABHD2	abhydrolase domain containing 2 [Source:HGNC Symbol;Acc:HGNC:18717]
ENSG00000101417	694.856579	660.078986	466.735259	447.112407	0.567955531	0.0005389	0.0033136	PXMP4	peroxisomal membrane protein 4 [Source:HGNC Symbol;Acc:HGNC:15920]
ENSG00000150764	652.535286	761.084361	503.610505	426.885893	0.603771944	0.0005402	0.0033207	DIXDC1	DIX domain containing 1 [Source:HGNC Symbol;Acc:HGNC:23695]
ENSG00000136247	432.070876	446.550079	625.825607	686.636911	-0.578727352	0.0005427	0.0033348	ZDHHC4	zinc finger DHHC-type containing 4 [Source:HGNC Symbol;Acc:HGNC:18471]
ENSG00000196976	628.9141	476.674489	864.987918	807.995993	-0.598840454	0.000543	0.0033353	LAGE3	L antigen family member 3 [Source:HGNC Symbol;Acc:HGNC:26058]
ENSG00000167136	355.302019	323.394403	510.985554	539.728549	-0.631105315	0.0005444	0.0033426	ENDOG	endonuclease G [Source:HGNC Symbol;Acc:HGNC:3346]
ENSG00000123136	2532.38807	1998.84321	3007.96651	3202.17648	-0.455154144	0.0005465	0.0033544	DDX39A	DExD-box helicase 39A [Source:HGNC Symbol;Acc:HGNC:17821]
ENSG00000139579	1442.86083	1233.32879	1816.36927	1834.22542	-0.448457405	0.0005488	0.0033672	NABP2	nucleic acid binding protein 2 [Source:HGNC Symbol;Acc:HGNC:28412]
ENSG00000160767	1419.23964	1259.90915	1800.56559	1821.45078	-0.435424511	0.000549	0.0033672	FAM189B	family with sequence similarity 189 member B [Source:HGNC Symbol;Acc:HGNC:1233]
ENSG00000102057	127.948095	116.06758	42.1431385	56.4213276	1.307684166	0.0005498	0.00337	KCND1	potassium voltage-gated channel subfamily D member 1 [Source:HGNC Symbol;Acc:HGNC:6237]
ENSG00000258429	289.359539	245.425341	413.002757	455.628834	-0.700812895	0.0005498	0.00337	PDF	peptide deformylase, mitochondrial [Source:HGNC Symbol;Acc:HGNC:30012]
ENSG00000156587	846.425862	827.535266	563.664477	599.343536	0.525352514	0.0005505	0.003373	UBE2L6	ubiquitin conjugating enzyme E2 L6 [Source:HGNC Symbol;Acc:HGNC:12490]
ENSG00000119866	52.1634543	46.0726272	12.6429415	7.45187345	2.288092944	0.0005509	0.0033741	BCL11A	B cell CLL/lymphoma 11A [Source:HGNC Symbol;Acc:HGNC:13221]
ENSG00000101445	35.4317803	29.238398	4.21431385	3.19366005	3.124588187	0.0005519	0.0033787	PPP1R16B	protein phosphatase 1 regulatory subunit 16B [Source:HGNC Symbol;Acc:HGNC:15850]
ENSG00000007168	2129.84368	2186.67777	2758.26841	2865.77762	-0.381656848	0.0005553	0.0033985	PAFAH1B1	platelet activating factor acetylhydrolase 1b regulatory subunit 1 [Source:HGNC Symbol;Acc:HGNC:8574]
ENSG00000170633	977.326605	913.478436	1294.84793	1301.94875	-0.458026613	0.0005577	0.0034119	RNF34	ring finger protein 34 [Source:HGNC Symbol;Acc:HGNC:17297]
ENSG00000136490	90.5478829	98.3473389	180.161917	199.071476	-1.004724625	0.0005596	0.0034225	LIMD2	LIM domain containing 2 [Source:HGNC Symbol;Acc:HGNC:28142]
ENSG00000002822	549.192594	491.736694	743.826394	781.382159	-0.551715234	0.0005604	0.003426	MAD1L1	mitotic arrest deficient 1 like 1 [Source:HGNC Symbol;Acc:HGNC:6762]
ENSG00000168066	4331.53514	4364.49542	5440.67918	5600.61518	-0.344463242	0.0005608	0.0034271	SF1	splicing factor 1 [Source:HGNC Symbol;Acc:HGNC:12950]
ENSG00000122507	476.360601	531.607237	315.01996	330.011539	0.644520021	0.0005617	0.0034317	BBS9	Bardet-Biedl syndrome 9 [Source:HGNC Symbol;Acc:HGNC:30000]
ENSG00000144369	178.143117	194.036642	107.465003	59.6149876	1.155314724	0.000563	0.0034384	FAM171B	family with sequence similarity 171 member B [Source:HGNC Symbol;Acc:HGNC:29412]
ENSG00000141452	486.202762	443.892043	256.019566	318.301452	0.695296398	0.0005671	0.0034621	RMC1	regulator of MON1-CCZ1 [Source:HGNC Symbol;Acc:HGNC:24326]
ENSG00000139514	3013.66975	2870.67908	3879.2759	3709.96843	-0.367189002	0.0005681	0.0034671	SLC7A1	solute carrier family 7 member 1 [Source:HGNC Symbol;Acc:HGNC:11057]
ENSG00000081721	392.702231	420.855729	592.111096	633.409243	-0.590627718	0.0005692	0.0034726	DUSP12	dual specificity phosphatase 12 [Source:HGNC Symbol;Acc:HGNC:3067]
ENSG00000244300	1157.43816	1057.8984	792.291004	790.963139	0.484321175	0.0005697	0.0034743	GATA2-AS1	GATA2 antisense RNA 1 [Source:HGNC Symbol;Acc:HGNC:51108]
ENSG00000049759	1223.38063	1178.39604	889.220222	848.44902	0.466796256	0.00057	0.003475	NEDD4L	neural precursor cell expressed, developmentally down-regulated 4-like, E3 ubiquitin protein ligase [Source:HGNC Symbol;Acc:HGNC:7728]
ENSG00000036549	508.839733	564.389683	893.434536	717.508958	-0.585541447	0.0005709	0.0034787	AC118549.1	zinc finger ZZ-type containing 3 [Source:NCBI gene;Acc:26009]
ENSG00000172046	2017.64304	1724.17947	2490.65948	2524.05599	-0.422832081	0.0005716	0.0034819	USP19	ubiquitin specific peptidase 19 [Source:HGNC Symbol;Acc:HGNC:12617]
ENSG00000168092	1550.14039	1726.83751	2286.26526	2108.88019	-0.423245902	0.0005721	0.0034837	PAFAH1B2	platelet activating factor acetylhydrolase 1b catalytic subunit 2 [Source:HGNC Symbol;Acc:HGNC:8575]
ENSG00000076924	1187.94885	1076.50466	1478.17058	1625.57297	-0.455181259	0.0005738	0.0034927	XAB2	XPA binding protein 2 [Source:HGNC Symbol;Acc:HGNC:14089]
ENSG00000176101	1693.83594	1321.04398	2017.60276	2204.68999	-0.486511058	0.0005754	0.0035014	SSNA1	SS nuclear autoantigen 1 [Source:HGNC Symbol;Acc:HGNC:11321]
ENSG00000148400	1737.14145	1435.33954	2286.26526	2071.62082	-0.45853294	0.0005827	0.0035442	NOTCH1	notch 1 [Source:HGNC Symbol;Acc:HGNC:7881]
ENSG00000110931	1172.2014	1103.97103	1523.47446	1554.24789	-0.435486732	0.0005838	0.0035501	CAMKK2	calcium/calmodulin dependent protein kinase kinase 2 [Source:HGNC Symbol;Acc:HGNC:1470]
ENSG00000168404	357.270451	426.171802	566.825213	642.990224	-0.625856854	0.0005851	0.0035568	MLKL	mixed lineage kinase domain like pseudokinase [Source:HGNC Symbol;Acc:HGNC:26617]
ENSG00000205683	78.7372895	72.6529891	20.0179908	26.6138338	1.698870367	0.0005886	0.0035763	DPF3	double PHD fingers 3 [Source:HGNC Symbol;Acc:HGNC:17427]
ENSG00000160877	4650.42116	3921.48939	5602.93026	5630.42267	-0.3903257	0.0005892	0.0035786	NACC1	nucleus accumbens associated 1 [Source:HGNC Symbol;Acc:HGNC:20967]
ENSG00000172183	63.9740477	75.3110253	134.858043	174.586749	-1.150064361	0.0005907	0.0035866	ISG20	interferon stimulated exonuclease gene 20 [Source:HGNC Symbol;Acc:HGNC:6130]
ENSG00000111371	5971.23919	6926.8423	9907.85186	7694.59162	-0.448533931	0.0005911	0.0035879	SLC38A1	solute carrier family 38 member 1 [Source:HGNC Symbol;Acc:HGNC:13447]
ENSG00000231770	152.553498	122.269665	67.4290216	47.9049008	1.251371675	0.0005933	0.0035991	TMEM44-AS1	TMEM44 antisense RNA 1 [Source:HGNC Symbol;Acc:HGNC:44272]
ENSG00000204642	241.132949	230.363136	116.947209	135.198275	0.902895867	0.0005934	0.0035991	HLA-F	major histocompatibility complex, class I, F [Source:HGNC Symbol;Acc:HGNC:4963]
ENSG00000165282	520.650327	442.120019	791.237425	665.345844	-0.598200727	0.0005941	0.0036021	PIGO	phosphatidylinositol glycan anchor biosynthesis class O [Source:HGNC Symbol;Acc:HGNC:23215]

ENSG00000114529	200.780088	233.021172	105.357846	119.229975	0.950572832	0.0005951	0.0036066	C3orf52	chromosome 3 open reading frame 52 [Source:HGNC Symbol;Acc:HGNC:26255]
ENSG00000067798	198.811656	165.684256	93.7684831	83.0351613	1.042775573	0.0005954	0.0036069	NAV3	neuron navigator 3 [Source:HGNC Symbol;Acc:HGNC:15998]
ENSG00000112110	1429.0818	1197.0023	1746.83309	1864.03292	-0.459964534	0.0005955	0.0036069	MRPL18	mitochondrial ribosomal protein L18 [Source:HGNC Symbol;Acc:HGNC:14477]
ENSG00000152377	1667.2621	1808.35062	2429.55193	2204.68999	-0.414855469	0.0005978	0.0036191	SPOCK1	SPARC (osteonectin), cwcv and kazal like domains proteoglycan 1 [Source:HGNC Symbol;Acc:HGNC:11251]
ENSG00000205918	25.5896191	63.7928684	4.21431385	9.58098015	2.699966414	0.0005992	0.0036263	PDPK2P	3-phosphoinositide dependent protein kinase 2, pseudogene [Source:HGNC Symbol;Acc:HGNC:49897]
ENSG00000163257	735.20944	741.592096	545.753643	438.59598	0.585142245	0.0006	0.0036298	DCAF16	DDB1 and CUL4 associated factor 16 [Source:HGNC Symbol;Acc:HGNC:25987]
ENSG00000116690	81.6899378	111.63752	38.9824031	28.7429405	1.514308924	0.0006005	0.0036318	PRG4	proteoglycan 4 [Source:HGNC Symbol;Acc:HGNC:9364]
ENSG00000126934	4260.67158	3745.17299	5107.74839	5271.66819	-0.374793967	0.0006017	0.0036374	MAP2K2	mitogen-activated protein kinase kinase 2 [Source:HGNC Symbol;Acc:HGNC:6842]
ENSG00000166452	830.678404	734.503999	1031.45331	1205.07439	-0.515386093	0.0006019	0.0036374	AKIP1	A-kinase interacting protein 1 [Source:HGNC Symbol;Acc:HGNC:1170]
ENSG00000123179	374.002125	413.767633	590.003939	600.40809	-0.594992015	0.0006032	0.0036443	EBPL	EBP like [Source:HGNC Symbol;Acc:HGNC:18061]
ENSG00000104472	753.909547	723.871854	980.881548	1108.20004	-0.499579053	0.0006038	0.0036463	CHRAC1	chromatin accessibility complex subunit 1 [Source:HGNC Symbol;Acc:HGNC:13544]
ENSG00000105429	2067.83806	1962.51672	1620.40368	1272.14125	0.478398192	0.0006052	0.0036537	MEGF8	multiple EGF like domains 8 [Source:HGNC Symbol;Acc:HGNC:3233]
ENSG00000084636	335.617696	270.233679	478.324622	487.565434	-0.674213955	0.000609	0.0036752	COL16A1	collagen type XVI alpha 1 chain [Source:HGNC Symbol;Acc:HGNC:2193]
ENSG00000141543	3484.12506	3047.88149	4075.24149	4538.19093	-0.399268403	0.0006099	0.0036797	EIF4A3	eukaryotic translation initiation factor 4A3 [Source:HGNC Symbol;Acc:HGNC:18683]
ENSG00000168090	2961.5063	2614.62159	3507.3627	3836.65027	-0.397533572	0.0006121	0.0036916	COPS6	COP9 signalosome subunit 6 [Source:HGNC Symbol;Acc:HGNC:21749]
ENSG00000113494	15.7474579	15.0622051	0	0	6.305053457	0.0006129	0.0036948	PRLR	prolactin receptor [Source:HGNC Symbol;Acc:HGNC:9446]
ENSG00000198561	4114.02337	4322.85285	5351.12501	5374.92987	-0.346253621	0.0006132	0.0036955	CTNND1	catenin delta 1 [Source:HGNC Symbol;Acc:HGNC:2515]
ENSG00000138604	962.563364	1169.53592	803.880367	625.95737	0.576808067	0.000614	0.0036986	GLCE	glucuronic acid epimerase [Source:HGNC Symbol;Acc:HGNC:17855]
ENSG00000214029	236.211868	229.477124	115.893631	133.069169	0.903418169	0.0006161	0.00371	ZNF891	zinc finger protein 891 [Source:HGNC Symbol;Acc:HGNC:38709]
ENSG00000102934	138.774473	142.647942	52.6789231	70.2605211	1.195207616	0.0006163	0.0037103	PLLP	plasmolipin [Source:HGNC Symbol;Acc:HGNC:18553]
ENSG00000185483	128.932311	103.663411	47.4110308	45.7757941	1.318551804	0.000618	0.0037188	ROR1	receptor tyrosine kinase like orphan receptor 1 [Source:HGNC Symbol;Acc:HGNC:10256]
ENSG00000128294	491.123843	434.14591	285.519763	298.074938	0.66439242	0.0006188	0.0037226	TPST2	tyrosylprotein sulfotransferase 2 [Source:HGNC Symbol;Acc:HGNC:12021]
ENSG00000080503	971.421309	994.105533	739.612081	637.667457	0.513097012	0.0006205	0.0037313	SMARCA2	SWI/SNF related, matrix associated, actin dependent regulator of chromatin, subfamily a, member 2 [Source:HGNC Symbol;Acc:HGNC:11098]
ENSG00000196975	720.446199	703.493577	996.685225	997.486489	-0.486021965	0.0006207	0.0037313	ANXA4	annexin A4 [Source:HGNC Symbol;Acc:HGNC:542]
ENSG00000152402	193.890575	215.300931	122.215102	68.1314144	1.104091727	0.0006214	0.0037339	GUCY1A2	guanylate cyclase 1 soluble subunit alpha 2 [Source:HGNC Symbol;Acc:HGNC:4684]
ENSG00000173852	1311.96009	1445.08567	1047.25699	949.581588	0.465647567	0.0006233	0.0037441	DPY19L1	dpy-19 like C-mannosyltransferase 1 [Source:HGNC Symbol;Acc:HGNC:22205]
ENSG00000151276	492.108059	461.612284	313.966382	294.881278	0.64715028	0.0006237	0.0037452	MAGI1	membrane associated guanylate kinase, WW and PDZ domain containing 1 [Source:HGNC Symbol;Acc:HGNC:946]
ENSG00000278311	1104.29048	1195.23027	1491.8671	1643.67037	-0.447055293	0.0006255	0.0037546	GGNBP2	gametogenetin binding protein 2 [Source:HGNC Symbol;Acc:HGNC:19357]
ENSG00000197451	6724.16452	6538.76902	7891.30268	9347.84297	-0.378300569	0.0006307	0.0037849	HNRNPAB	heterogeneous nuclear ribonucleoprotein A/B [Source:HGNC Symbol;Acc:HGNC:5034]
ENSG00000022267	480.297466	475.788477	263.394616	333.205199	0.680494752	0.0006333	0.0037988	FHL1	four and a half LIM domains 1 [Source:HGNC Symbol;Acc:HGNC:3702]
ENSG00000104093	955.673851	1050.81031	765.951542	602.537196	0.552252003	0.0006337	0.0037997	DMXL2	Dmx like 2 [Source:HGNC Symbol;Acc:HGNC:2938]
ENSG00000198113	1752.88891	1359.1425	2192.49678	2135.49402	-0.476459187	0.0006347	0.0038044	TOR4A	torsin family 4 member A [Source:HGNC Symbol;Acc:HGNC:25981]
ENSG00000135723	1848.35787	1465.46395	1138.91832	1215.71993	0.492508793	0.0006357	0.0038093	FHOD1	formin homology 2 domain containing 1 [Source:HGNC Symbol;Acc:HGNC:17905]
ENSG00000154930	75.7846411	87.7151941	28.4466185	25.5492804	1.598925854	0.0006364	0.0038118	ACSS1	acyl-CoA synthetase short chain family member 1 [Source:HGNC Symbol;Acc:HGNC:16091]
ENSG00000169905	1750.92047	1899.60986	1308.54445	1407.33953	0.426926228	0.0006369	0.0038137	TOR1AIP2	torsin 1A interacting protein 2 [Source:HGNC Symbol;Acc:HGNC:24055]
ENSG00000143156	544.271513	677.799227	424.59212	355.560819	0.648155346	0.0006396	0.0038287	NME7	NME/NM23 family member 7 [Source:HGNC Symbol;Acc:HGNC:20461]
ENSG00000136444	821.820459	660.964998	495.181877	491.823648	0.586420536	0.0006398	0.0038287	RSAD1	radical S-adenosyl methionine domain containing 1 [Source:HGNC Symbol;Acc:HGNC:25634]
ENSG00000011198	887.762939	905.504327	653.218647	607.859963	0.507966671	0.0006454	0.0038606	ABHD5	abhydrolase domain containing 5 [Source:HGNC Symbol;Acc:HGNC:21396]
ENSG00000165526	414.354986	411.995609	585.789625	653.635757	-0.584829114	0.0006473	0.0038705	RPUSD4	RNA pseudouridylate synthase domain containing 4 [Source:HGNC Symbol;Acc:HGNC:25898]
ENSG00000164054	3242.99211	2996.49279	4037.31267	3992.07506	-0.364028473	0.0006496	0.0038822	SHISA5	shisa family member 5 [Source:HGNC Symbol;Acc:HGNC:30376]
ENSG00000240694	77.7530733	91.2592424	29.5001969	27.6783871	1.564262972	0.0006497	0.0038822	PNMA2	PNMA family member 2 [Source:HGNC Symbol;Acc:HGNC:9159]
ENSG00000231584	124.995447	85.0571579	35.8216677	41.5175807	1.439956409	0.000652	0.0038946	FAHD2CP	fumarylacetoacetate hydrolase domain containing 2C, pseudogene [Source:HGNC Symbol;Acc:HGNC:44135]
ENSG00000104907	1042.28487	868.291821	1314.86592	1356.24097	-0.4841102	0.0006562	0.0039181	TRMT1	tRNA methyltransferase 1 [Source:HGNC Symbol;Acc:HGNC:25980]
ENSG00000198585	1106.25892	995.877557	753.308601	743.058238	0.490019335	0.0006568	0.0039205	NUDT16	nudix hydrolase 16 [Source:HGNC Symbol;Acc:HGNC:26442]
ENSG00000102898	2648.52557	2206.17003	3020.60945	3622.67505	-0.452802411	0.0006598	0.0039372	NUTF2	nuclear transport factor 2 [Source:HGNC Symbol;Acc:HGNC:13722]
ENSG00000276710	308.059645	260.487546	138.018779	175.651303	0.857493817	0.0006609	0.0039421	CSPG4P10	chondroitin sulfate proteoglycan 4 pseudogene 10 [Source:HGNC Symbol;Acc:HGNC:48361]
ENSG00000122515	2528.45121	2094.53251	1580.36769	1788.44963	0.456277268	0.0006616	0.0039448	ZMIZ2	zinc finger MIZ-type containing 2 [Source:HGNC Symbol;Acc:HGNC:22229]
ENSG00000140990	2564.8672	2285.02511	3022.71661	3409.76438	-0.407644813	0.0006631	0.0039513	NDUFB10	NADH:ubiquinone oxidoreductase subunit B10 [Source:HGNC Symbol;Acc:HGNC:7696]

ENSG00000280789	108.263773	80.6270976	171.733289	216.10433	-1.040127025	0.0006631	0.0039513	PAGR1	PAXIP1 associated glutamate rich protein 1 [Source:HGNC Symbol;Acc:HGNC:28707]
ENSG00000108387	62.0056154	76.1970373	17.9108339	22.3556204	1.780361403	0.0006637	0.003953	4-Sep	septin 4 [Source:HGNC Symbol;Acc:HGNC:9165]
ENSG00000186529	62.0056154	47.8446513	7.37504924	17.0328536	2.170194905	0.0006647	0.003958	CYP4F3	cytochrome P450 family 4 subfamily F member 3 [Source:HGNC Symbol;Acc:HGNC:2646]
ENSG00000122565	3057.95948	3873.64473	5254.19579	4324.21571	-0.466349214	0.000665	0.0039584	CBX3	chromobox 3 [Source:HGNC Symbol;Acc:HGNC:1553]
ENSG00000188112	151.569282	155.938123	278.144714	265.073784	-0.820712322	0.0006654	0.0039589	C6orf132	chromosome 6 open reading frame 132 [Source:HGNC Symbol;Acc:HGNC:21288]
ENSG00000163933	471.439521	467.814369	673.236637	702.605211	-0.550753211	0.0006693	0.0039811	RFT1	RFT1 homolog [Source:HGNC Symbol;Acc:HGNC:30220]
ENSG00000065029	299.2017	287.067908	448.824425	469.468027	-0.647646669	0.0006729	0.004001	ZNF76	zinc finger protein 76 [Source:HGNC Symbol;Acc:HGNC:13149]
ENSG00000124191	381.875854	393.389355	249.698096	226.749864	0.702435849	0.0006791	0.0040362	TOX2	TOX high mobility group box family member 2 [Source:HGNC Symbol;Acc:HGNC:16095]
ENSG00000198000	628.9141	790.322759	1005.11385	1051.77871	-0.534393721	0.000681	0.0040461	NOL8	nucleolar protein 8 [Source:HGNC Symbol;Acc:HGNC:23387]
ENSG00000093010	1264.71771	1116.3752	1609.86789	1620.2502	-0.440416657	0.0006814	0.0040473	COMT	catechol-O-methyltransferase [Source:HGNC Symbol;Acc:HGNC:2228]
ENSG00000182771	50.195022	38.9845307	10.5357846	6.3873201	2.395860434	0.0006829	0.0040549	GRID1	glutamate ionotropic receptor delta type subunit 1 [Source:HGNC Symbol;Acc:HGNC:4575]
ENSG00000108840	1374.94992	1259.90915	934.524096	984.711849	0.456941721	0.0006851	0.0040663	HDAC5	histone deacetylase 5 [Source:HGNC Symbol;Acc:HGNC:14068]
ENSG00000128245	2043.23266	2041.37179	2782.50072	2558.1217	-0.38683417	0.0006859	0.0040694	YWHAH	tyrosine 3-monooxygenase/tryptophan 5-monooxygenase activation protein eta [Source:HGNC Symbol;Acc:HGNC:12853]
ENSG00000091732	407.465473	341.114644	542.592908	612.118176	-0.626258038	0.0006885	0.0040828	ZC3HC1	zinc finger C3HC-type containing 1 [Source:HGNC Symbol;Acc:HGNC:29913]
ENSG00000140563	311.012293	303.016125	183.322652	175.651303	0.774262881	0.0006886	0.0040828	MCTP2	multiple C2 and transmembrane domain containing 2 [Source:HGNC Symbol;Acc:HGNC:25636]
ENSG00000072682	3677.03142	3227.74194	2547.55272	2709.28828	0.393202932	0.0006901	0.0040905	P4HA2	prolyl 4-hydroxylase subunit alpha 2 [Source:HGNC Symbol;Acc:HGNC:8547]
ENSG00000140986	28.5422674	38.0985187	5.26789231	3.19366005	2.977630912	0.0006905	0.0040908	RPL3L	ribosomal protein L3 like [Source:HGNC Symbol;Acc:HGNC:10351]
ENSG00000268089	793.278191	818.675145	556.289428	568.471489	0.519331728	0.0006907	0.0040908	GABRQ	gamma-aminobutyric acid type A receptor theta subunit [Source:HGNC Symbol;Acc:HGNC:14454]
ENSG00000108587	629.898316	620.208443	886.059487	882.514727	-0.500620067	0.000693	0.0041029	GOSR1	golgi SNAP receptor complex member 1 [Source:HGNC Symbol;Acc:HGNC:4430]
ENSG00000138172	94.4847473	67.3369167	175.947603	166.070323	-1.082549566	0.0006935	0.0041045	CALHM2	calcium homeostasis modulator family member 2 [Source:HGNC Symbol;Acc:HGNC:23493]
ENSG00000151065	200.780088	212.642895	318.180696	377.916439	-0.751180314	0.0006945	0.0041091	DCP1B	decapping mRNA 1B [Source:HGNC Symbol;Acc:HGNC:24451]
ENSG00000163932	1133.81697	1042.8362	793.344582	768.607519	0.478452853	0.0006978	0.0041267	PRKCD	protein kinase C delta [Source:HGNC Symbol;Acc:HGNC:9399]
ENSG00000112742	291.327971	377.441138	618.450557	473.726241	-0.706440777	0.000698	0.0041267	TTK	TTK protein kinase [Source:HGNC Symbol;Acc:HGNC:12401]
ENSG00000148334	2087.52239	1655.07053	2549.65988	2561.31536	-0.45007396	0.0006984	0.0041278	PTGES2	prostaglandin E synthase 2 [Source:HGNC Symbol;Acc:HGNC:17822]
ENSG00000162174	336.601912	293.269992	151.715299	203.32969	0.826700176	0.0006999	0.0041339	ASRGL1	asparaginase like 1 [Source:HGNC Symbol;Acc:HGNC:16448]
ENSG00000050748	2015.67461	2075.04025	1603.54642	1483.98737	0.405943788	0.0006999	0.0041339	MAPK9	mitogen-activated protein kinase 9 [Source:HGNC Symbol;Acc:HGNC:6886]
ENSG00000140416	1864.10533	1850.8792	1370.70558	1430.7597	0.407171721	0.0007023	0.0041466	TPM1	tropomyosin 1 [Source:HGNC Symbol;Acc:HGNC:12010]
ENSG00000138642	186.016846	221.503015	332.930794	354.496266	-0.75307047	0.0007041	0.0041556	HERC6	HECT and RLD domain containing E3 ubiquitin protein ligase family member 6 [Source:HGNC Symbol;Acc:HGNC:26072]
ENSG00000163295	6.88951283	7.97410856	45.3038739	36.1948139	-2.452799155	0.0007057	0.0041639	ALPI	alkaline phosphatase, intestinal [Source:HGNC Symbol;Acc:HGNC:437]
ENSG00000059691	438.960389	438.575971	626.879185	669.604057	-0.563047201	0.0007071	0.0041708	GATB	glutamyl-tRNA amidotransferase subunit B [Source:HGNC Symbol;Acc:HGNC:8849]
ENSG00000276170	61.0213993	52.2747116	124.322259	136.262829	-1.203097656	0.0007099	0.0041858	AC244153.1	uncharacterized LOC101929494 [Source:NCBI gene;Acc:101929494]
ENSG00000112367	332.665048	322.50839	199.126329	190.55505	0.749394991	0.0007108	0.0041896	FIG4	FIG4 phosphoinositide 5-phosphatase [Source:HGNC Symbol;Acc:HGNC:16873]
ENSG00000168575	293.296403	301.244101	440.395797	491.823648	-0.648664091	0.0007131	0.0042013	SLC20A2	solute carrier family 20 member 2 [Source:HGNC Symbol;Acc:HGNC:10947]
ENSG00000189180	147.632418	163.912231	352.948785	231.008077	-0.905926761	0.0007152	0.0042125	ZNF33A	zinc finger protein 33A [Source:HGNC Symbol;Acc:HGNC:13096]
ENSG00000106049	2607.1885	3001.80887	2120.85344	2123.78393	0.402366517	0.0007168	0.0042204	HIBADH	3-hydroxyisobutyrate dehydrogenase [Source:HGNC Symbol;Acc:HGNC:4907]
ENSG00000198522	310.028077	396.047392	508.878397	614.247283	-0.668277167	0.0007175	0.0042229	GPN1	GPN-loop GTPase 1 [Source:HGNC Symbol;Acc:HGNC:17030]
ENSG00000102699	1779.46274	1870.37146	2357.9086	2414.407	-0.386699258	0.0007202	0.0042376	PARP4	poly(ADP-ribose) polymerase family member 4 [Source:HGNC Symbol;Acc:HGNC:271]
ENSG00000089057	1569.82471	1485.84223	1181.06146	1069.87612	0.440760092	0.000721	0.0042409	SLC23A2	solute carrier family 23 member 2 [Source:HGNC Symbol;Acc:HGNC:10973]
ENSG00000266028	1396.60267	1391.03894	1001.95312	1056.03692	0.437820674	0.0007219	0.0042443	SRGAP2	SLIT-ROBO Rho GTPase activating protein 2 [Source:HGNC Symbol;Acc:HGNC:19751]
ENSG00000188186	998.97936	849.685567	1269.56205	1300.88419	-0.476170588	0.0007233	0.0042511	LAMTOR4	late endosomal/lysosomal adaptor, MAPK and MTOR activator 4 [Source:HGNC Symbol;Acc:HGNC:33772]
ENSG00000104783	1360.18668	1094.2249	1676.24333	1725.64098	-0.471613992	0.0007242	0.0042553	KCNN4	potassium calcium-activated channel subfamily N member 4 [Source:HGNC Symbol;Acc:HGNC:6293]
ENSG00000237506	1315.89695	1502.67646	1825.85148	1985.392	-0.434858926	0.0007257	0.004262	RPSAP15	ribosomal protein SA pseudogene 15 [Source:HGNC Symbol;Acc:HGNC:31464]
ENSG00000100065	1923.15829	1665.70268	2398.99816	2376.08308	-0.412395438	0.0007259	0.004262	CARD10	caspase recruitment domain family member 10 [Source:HGNC Symbol;Acc:HGNC:16422]
ENSG00000108582	2983.15905	3771.75335	2709.80381	1994.97298	0.521981547	0.0007267	0.0042641	CPD	carboxypeptidase D [Source:HGNC Symbol;Acc:HGNC:2301]
ENSG00000006282	1654.46729	1361.80054	1981.78109	2141.88134	-0.451672303	0.0007267	0.0042641	SPATA20	spermatogenesis associated 20 [Source:HGNC Symbol;Acc:HGNC:26125]
ENSG00000113448	771.625437	955.121003	642.682862	485.436328	0.61457867	0.0007289	0.0042751	PDE4D	phosphodiesterase 4D [Source:HGNC Symbol;Acc:HGNC:8783]
ENSG00000141564	2012.72196	1730.38156	2451.67708	2532.57242	-0.413528728	0.0007316	0.0042896	RPTOR	regulatory associated protein of MTOR complex 1 [Source:HGNC Symbol;Acc:HGNC:30287]
ENSG00000185721	1011.77417	1000.30762	1289.58004	1474.40639	-0.458062909	0.0007321	0.0042913	DRG1	developmentally regulated GTP binding protein 1 [Source:HGNC Symbol;Acc:HGNC:3029]
ENSG00000172456	161.411443	168.342292	89.5541693	64.9377544	1.093751803	0.0007335	0.0042977	FGGY	FGGY carbohydrate kinase domain containing [Source:HGNC Symbol;Acc:HGNC:25610]
ENSG00000255769	368.096828	308.332198	190.697702	207.587903	0.763405038	0.0007354	0.0043073	GOLGA2P10	GOLGA2 pseudogene 10 [Source:HGNC Symbol;Acc:HGNC:26229]

ENSG00000213178	310.028077	317.192318	473.05673	496.081861	-0.627554994	0.0007367	0.0043132	RPL22P1	ribosomal protein L22 pseudogene 1 [Source:HGNC Symbol;Acc:HGNC:17998]
ENSG00000062038	273.612081	258.715522	140.125935	158.618449	0.833237101	0.0007369	0.0043132	CDH3	cadherin 3 [Source:HGNC Symbol;Acc:HGNC:1762]
ENSG00000087152	1957.60586	1665.70268	2335.78345	2531.50787	-0.426203597	0.0007374	0.0043145	ATXN7L3	ataxin 7 like 3 [Source:HGNC Symbol;Acc:HGNC:25416]
ENSG00000263266	615.135074	645.902793	822.844779	1011.32568	-0.540223482	0.000738	0.0043168	RPS7P1	ribosomal protein S7 pseudogene 1 [Source:HGNC Symbol;Acc:HGNC:17081]
ENSG00000105426	3878.79572	3275.58659	2748.78621	2665.64159	0.401764088	0.0007387	0.0043193	PTPRS	protein tyrosine phosphatase, receptor type S [Source:HGNC Symbol;Acc:HGNC:9681]
ENSG00000148680	63.9740477	59.3628081	12.6429415	20.2265137	1.908073821	0.0007398	0.0043241	HTR7	5-hydroxytryptamine receptor 7 [Source:HGNC Symbol;Acc:HGNC:5302]
ENSG00000101363	634.819396	535.151285	832.326985	863.352767	-0.53621583	0.0007411	0.0043305	MANBAL	mannosidase beta like [Source:HGNC Symbol;Acc:HGNC:15799]
ENSG00000134531	1939.88997	1890.74974	1438.1346	1462.6963	0.401054623	0.0007437	0.004344	EMP1	epithelial membrane protein 1 [Source:HGNC Symbol;Acc:HGNC:3333]
ENSG00000120948	1776.51009	1894.29379	2609.71385	2278.14417	-0.412966098	0.0007446	0.004347	TARDBP	TAR DNA binding protein [Source:HGNC Symbol;Acc:HGNC:11571]
ENSG00000135999	184.048414	195.808666	364.538148	289.558511	-0.783726976	0.0007449	0.004347	EPC2	enhancer of polycomb homolog 2 [Source:HGNC Symbol;Acc:HGNC:24543]
ENSG00000134308	6279.29883	7232.51646	9352.61601	8251.35302	-0.381568384	0.000745	0.004347	YWHAQ	tyrosine 3-monooxygenase/tryptophan 5-monooxygenase activation protein theta [Source:HGNC Symbol;Acc:HGNC:12854]
ENSG00000183087	701.746092	657.42095	414.056336	495.017308	0.580111754	0.0007484	0.0043656	GAS6	growth arrest specific 6 [Source:HGNC Symbol;Acc:HGNC:4168]
ENSG00000205302	1336.56549	1535.4589	1018.81037	1072.00522	0.458415676	0.0007487	0.0043656	SNX2	sorting nexin 2 [Source:HGNC Symbol;Acc:HGNC:11173]
ENSG00000150867	595.450752	604.260226	372.966776	424.756787	0.588872105	0.000749	0.0043656	PIP4K2A	phosphatidylinositol-5-phosphate 4-kinase type 2 alpha [Source:HGNC Symbol;Acc:HGNC:8997]
ENSG00000105393	545.255729	457.182224	692.20105	803.737779	-0.578346131	0.0007522	0.0043829	BABAM1	BRISC and BRCA1 A complex member 1 [Source:HGNC Symbol;Acc:HGNC:25008]
ENSG00000150687	8436.70057	8822.0221	6585.91897	7086.73165	0.336080037	0.0007525	0.0043834	PRSS23	serine protease 23 [Source:HGNC Symbol;Acc:HGNC:14370]
ENSG00000135749	704.698741	670.711131	487.806828	449.241514	0.55341073	0.0007532	0.0043857	PCNX2	pecanex 2 [Source:HGNC Symbol;Acc:HGNC:8736]
ENSG00000069998	1156.45394	987.903449	1461.31333	1481.85826	-0.457403296	0.0007586	0.0044158	HDHD5	haloacid dehalogenase like hydrolase domain containing 5 [Source:HGNC Symbol;Acc:HGNC:1843]
ENSG00000115993	1123.97481	1210.29248	889.220222	784.575819	0.480022139	0.0007598	0.0044211	TRAK2	trafficking kinesin protein 2 [Source:HGNC Symbol;Acc:HGNC:13206]
ENSG00000137312	2938.86933	2505.64211	3498.93407	3669.5154	-0.397159305	0.0007617	0.0044308	FLOT1	flotillin 1 [Source:HGNC Symbol;Acc:HGNC:3757]
ENSG00000187837	2849.30566	3023.07315	1831.11937	2418.66521	0.466688501	0.0007622	0.0044323	HIST1H1C	histone cluster 1 H1 family member c [Source:HGNC Symbol;Acc:HGNC:4716]
ENSG00000146859	321.838671	345.544704	204.394222	194.813263	0.741679361	0.0007646	0.0044443	TMEM140	transmembrane protein 140 [Source:HGNC Symbol;Acc:HGNC:21870]
ENSG00000122085	206.685385	214.414919	327.662902	371.529119	-0.731196689	0.0007698	0.0044728	MTERF4	mitochondrial transcription termination factor 4 [Source:HGNC Symbol;Acc:HGNC:28785]
ENSG00000092068	281.48581	309.21821	180.161917	160.747556	0.793399311	0.00077	0.0044728	SLC7A8	solute carrier family 7 member 8 [Source:HGNC Symbol;Acc:HGNC:11066]
ENSG00000221843	20.6685385	24.8083377	67.4290216	77.7123946	-1.672037661	0.0007708	0.0044757	C2orf16	chromosome 2 open reading frame 16 [Source:HGNC Symbol;Acc:HGNC:25275]
ENSG00000163399	12007.4366	11066.2907	8918.54168	9392.55421	0.333484405	0.0007737	0.0044912	ATP1A1	ATPase Na+/K+ transporting subunit alpha 1 [Source:HGNC Symbol;Acc:HGNC:799]
ENSG00000243678	47.2423737	30.1244101	99.0363755	106.455335	-1.413686151	0.0007742	0.0044923	NME2	NME/NM23 nucleoside diphosphate kinase 2 [Source:HGNC Symbol;Acc:HGNC:7850]
ENSG00000125895	62.9898316	46.9586393	8.4286277	17.0328536	2.110179002	0.0007754	0.0044978	TMEM74B	transmembrane protein 74B [Source:HGNC Symbol;Acc:HGNC:15893]
ENSG00000185518	33.463348	38.9845307	3.16073539	7.45187345	2.773240544	0.0007757	0.0044983	SV2B	synaptic vesicle glycoprotein 2B [Source:HGNC Symbol;Acc:HGNC:16874]
ENSG00000254166	366.128396	395.16138	221.251477	245.911824	0.70495216	0.0007775	0.0045065	CASC19	cancer susceptibility 19 [Source:HGNC Symbol;Acc:HGNC:49476]
ENSG00000166689	607.261345	535.151285	354.002363	394.949293	0.608693426	0.0007777	0.0045065	PLEKHA7	pleckstrin homology domain containing A7 [Source:HGNC Symbol;Acc:HGNC:27049]
ENSG00000163811	880.873426	974.613268	1289.58004	1256.17295	-0.455872385	0.0007779	0.0045066	WDR43	WD repeat domain 43 [Source:HGNC Symbol;Acc:HGNC:28945]
ENSG00000095209	624.977235	700.835541	447.770846	449.241514	0.564146876	0.000779	0.0045109	TMEM38B	transmembrane protein 38B [Source:HGNC Symbol;Acc:HGNC:25535]
ENSG00000197444	98.4216118	80.6270976	26.3394616	36.1948139	1.517083373	0.0007795	0.0045123	OGDHL	oxoglutarate dehydrogenase like [Source:HGNC Symbol;Acc:HGNC:25590]
ENSG00000185630	1383.80786	1442.42764	1891.17334	1847.00006	-0.403310027	0.0007802	0.0045149	PBX1	PBX homeobox 1 [Source:HGNC Symbol;Acc:HGNC:8632]
ENSG00000169826	413.37077	456.296212	296.055548	243.782717	0.688217692	0.0007806	0.0045156	CSGALNACT2	chondroitin sulfate N-acetylgalactosaminyltransferase 2 [Source:HGNC Symbol;Acc:HGNC:24292]
ENSG00000123384	16164.7655	13957.348	12551.2802	9985.51043	0.418483773	0.0007816	0.0045201	LRP1	LDL receptor related protein 1 [Source:HGNC Symbol;Acc:HGNC:6692]
ENSG00000184378	38.3844286	31.8964342	109.57216	81.970608	-1.448528289	0.0007824	0.0045232	ACTRT3	actin related protein T3 [Source:HGNC Symbol;Acc:HGNC:24022]
ENSG00000139438	253.927758	256.943498	133.804465	151.166576	0.842307318	0.0007854	0.0045389	FAM222A	family with sequence similarity 222 member A [Source:HGNC Symbol;Acc:HGNC:25915]
ENSG00000059804	791.309759	794.752819	1103.09665	1082.65076	-0.462663577	0.0007857	0.0045389	SLC2A3	solute carrier family 2 member 3 [Source:HGNC Symbol;Acc:HGNC:11007]
ENSG00000103018	4988.9915	5242.53337	3668.56021	4225.21225	0.374315607	0.0007889	0.0045561	CYB5B	cytochrome b5 type B [Source:HGNC Symbol;Acc:HGNC:24374]
ENSG00000135638	11.8105934	17.7202412	0	0	6.24542608	0.0007907	0.0045648	EMX1	empty spiracles homeobox 1 [Source:HGNC Symbol;Acc:HGNC:3340]
ENSG00000099377	24.605403	15.9482171	66.3754431	70.2605211	-1.757498726	0.0007928	0.0045756	HSD3B7	hydroxy-delta-5-steroid dehydrogenase, 3 beta- and steroid delta-isomerase 7 [Source:HGNC Symbol;Acc:HGNC:18324]
ENSG00000164307	391.718015	410.223585	588.95036	604.666303	-0.573473277	0.0007942	0.0045822	ERAP1	endoplasmic reticulum aminopeptidase 1 [Source:HGNC Symbol;Acc:HGNC:18173]
ENSG00000100416	1081.65351	928.540641	1323.29455	1461.63175	-0.470836245	0.0007993	0.0046098	TRMU	tRNA 5-methylaminomethyl-2-thiouridylate methyltransferase [Source:HGNC Symbol;Acc:HGNC:25481]
ENSG00000277258	863.157536	757.540313	1069.38214	1211.46171	-0.493452485	0.0007996	0.00461	PCGF2	polycomb group ring finger 2 [Source:HGNC Symbol;Acc:HGNC:12929]
ENSG00000231652	54.1318865	87.7151941	22.1251477	19.1619603	1.782415073	0.0008019	0.0046215	AL590428.1	uncharacterized LOC101928489 [Source:NCBI gene;Acc:101928489]
ENSG00000038274	912.368342	912.592424	1335.93749	1178.46056	-0.462390189	0.0008074	0.0046505	MAT2B	methionine adenosyltransferase 2B [Source:HGNC Symbol;Acc:HGNC:6905]
ENSG00000121281	114.16907	113.409544	218.090742	208.652457	-0.907084176	0.0008075	0.0046505	ADCY7	adenylate cyclase 7 [Source:HGNC Symbol;Acc:HGNC:238]
ENSG00000110108	2077.68023	1862.39735	2550.71346	2617.73669	-0.39179899	0.0008128	0.0046797	TMEM109	transmembrane protein 109 [Source:HGNC Symbol;Acc:HGNC:28771]

ENSG00000106689	483.250114	458.954248	313.966382	292.752171	0.634727008	0.000815	0.0046878	LHX2	LIM homeobox 2 [Source:HGNC Symbol;Acc:HGNC:6594]
ENSG00000173706	11419.8596	11731.6857	9724.52921	8370.58299	0.355511581	0.0008152	0.0046878	HEG1	heart development protein with EGF like domains 1 [Source:HGNC Symbol;Acc:HGNC:29227]
ENSG00000182118	84.6425862	92.1452544	37.9288246	22.3556204	1.551947148	0.0008153	0.0046878	FAM89A	family with sequence similarity 89 member A [Source:HGNC Symbol;Acc:HGNC:25057]
ENSG00000163629	594.466535	676.027203	402.466973	446.047854	0.582949852	0.0008153	0.0046878	PTPN13	protein tyrosine phosphatase, non-receptor type 13 [Source:HGNC Symbol;Acc:HGNC:9646]
ENSG00000137145	692.888147	845.255507	568.93237	434.337767	0.616928198	0.0008161	0.0046908	DENND4C	DENN domain containing 4C [Source:HGNC Symbol;Acc:HGNC:26079]
ENSG00000168079	235.227652	199.352714	121.161523	107.519888	0.92539216	0.0008187	0.0047042	SCARA5	scavenger receptor class A member 5 [Source:HGNC Symbol;Acc:HGNC:28701]
ENSG00000164342	63.9740477	100.119363	194.912016	158.618449	-1.103963777	0.0008192	0.0047051	TLR3	toll like receptor 3 [Source:HGNC Symbol;Acc:HGNC:11849]
ENSG00000057935	582.655942	637.042672	817.576887	935.742395	-0.523076678	0.0008199	0.0047079	MTA3	metastasis associated 1 family member 3 [Source:HGNC Symbol;Acc:HGNC:23784]
ENSG00000158104	46.2581576	30.1244101	109.57216	93.6806948	-1.416260832	0.0008208	0.0047113	HPD	4-hydroxyphenylpyruvate dioxygenase [Source:HGNC Symbol;Acc:HGNC:5147]
ENSG00000124228	1622.97238	1352.94042	1930.15574	2123.78393	-0.446472412	0.0008224	0.0047186	DDX27	DEAD-box helicase 27 [Source:HGNC Symbol;Acc:HGNC:15837]
ENSG00000138623	281.48581	206.44081	140.125935	116.036315	0.928172908	0.0008261	0.0047385	SEMA7A	semaphorin 7A (John Milton Hagen blood group) [Source:HGNC Symbol;Acc:HGNC:10741]
ENSG00000178719	3742.9739	3446.58692	4497.72646	4695.74483	-0.354848882	0.0008303	0.0047612	GRINA	glutamate ionotropic receptor NMDA type subunit associated protein 1 [Source:HGNC Symbol;Acc:HGNC:4589]
ENSG00000055483	868.078616	857.659676	1177.90072	1178.46056	-0.449403336	0.0008378	0.0048014	USP36	ubiquitin specific peptidase 36 [Source:HGNC Symbol;Acc:HGNC:20062]
ENSG00000170325	165.348308	185.176521	309.752068	290.623065	-0.775529226	0.0008379	0.0048014	PRDM10	PR/SET domain 10 [Source:HGNC Symbol;Acc:HGNC:13995]
ENSG00000214717	2110.15936	1969.60481	2598.12449	2709.28828	-0.37970467	0.0008399	0.0048109	ZBED1	zinc finger BED-type containing 1 [Source:HGNC Symbol;Acc:HGNC:447]
ENSG00000110697	3210.51298	2871.56509	2336.83703	2332.43639	0.381155887	0.0008421	0.0048218	PITPNM1	phosphatidylinositol transfer protein membrane associated 1 [Source:HGNC Symbol;Acc:HGNC:9003]
ENSG00000138363	1511.75596	1644.43839	2040.78148	2126.97759	-0.400803733	0.0008427	0.0048236	ATIC	5-aminoimidazole-4-carboxamide ribonucleotide formyltransferase/IMP cyclohydrolase [Source:HGNC Symbol;Acc:HGNC:794]
ENSG00000161016	40239.676	31121.1737	45016.247	49924.3585	-0.411926053	0.0008433	0.0048258	RPL8	ribosomal protein L8 [Source:HGNC Symbol;Acc:HGNC:10368]
ENSG00000205250	2097.36455	1901.38188	2584.42797	2640.09231	-0.386014793	0.0008444	0.0048303	E2F4	E2F transcription factor 4 [Source:HGNC Symbol;Acc:HGNC:3118]
ENSG00000031698	3052.05418	2851.18681	3686.47104	3895.20071	-0.361143277	0.0008451	0.0048325	SARS	seryl-tRNA synthetase [Source:HGNC Symbol;Acc:HGNC:10537]
ENSG00000143643	602.340264	720.327806	436.181483	449.241514	0.579711452	0.0008455	0.0048328	TTC13	tetratricopeptide repeat domain 13 [Source:HGNC Symbol;Acc:HGNC:26204]
ENSG00000101191	2851.27409	2722.71507	3634.8457	3503.44508	-0.356982264	0.000846	0.0048328	DIDO1	death inducer-obliterator 1 [Source:HGNC Symbol;Acc:HGNC:2680]
ENSG00000119689	2024.53256	2035.16971	2554.92777	2711.41738	-0.375398483	0.000846	0.0048328	DLST	dihydrolipoamide S-succinyltransferase [Source:HGNC Symbol;Acc:HGNC:2911]
ENSG00000102048	254.911975	261.373558	422.484963	397.0784	-0.666544258	0.0008506	0.0048573	ASB9	ankyrin repeat and SOCS box containing 9 [Source:HGNC Symbol;Acc:HGNC:17184]
ENSG00000121691	1902.48976	1852.65122	1425.49166	1424.37238	0.397899003	0.0008516	0.0048619	CAT	catalase [Source:HGNC Symbol;Acc:HGNC:1516]
ENSG00000154118	924.178935	755.768289	578.414576	572.729702	0.544662376	0.000852	0.0048623	JPH3	junctophilin 3 [Source:HGNC Symbol;Acc:HGNC:14203]
ENSG00000183751	1451.71877	1236.87284	1780.5476	1855.51649	-0.436025576	0.0008529	0.0048655	TBL3	transducin beta like 3 [Source:HGNC Symbol;Acc:HGNC:11587]
ENSG00000229931	60.0371832	40.7565548	13.69652	8.5164268	2.179636183	0.0008546	0.0048739	AL137003.1	uncharacterized LOC101928433 [Source:NCBI gene;Acc:101928433]
ENSG00000112276	103.342692	107.207459	35.8216677	46.8403474	1.349335772	0.0008551	0.0048748	BVES	blood vessel epicardial substance [Source:HGNC Symbol;Acc:HGNC:1152]
ENSG00000122692	1026.53741	1119.03323	1400.20578	1510.6012	-0.439695672	0.0008611	0.0049073	SMU1	SMU1, DNA replication regulator and spliceosomal factor [Source:HGNC Symbol;Acc:HGNC:18247]
ENSG00000141655	180.11155	201.124738	89.5541693	104.326228	0.976194151	0.000863	0.0049169	TNFRSF11A	TNF receptor superfamily member 11a [Source:HGNC Symbol;Acc:HGNC:11908]
ENSG00000162444	74.800425	94.8032906	32.6609323	25.5492804	1.543623464	0.0008645	0.0049234	RBP7	retinol binding protein 7 [Source:HGNC Symbol;Acc:HGNC:30316]
ENSG00000139131	800.167704	904.618315	1144.18621	1218.91359	-0.470542293	0.000865	0.0049245	YARS2	tyrosyl-tRNA synthetase 2 [Source:HGNC Symbol;Acc:HGNC:24249]
ENSG00000153786	1647.57778	1544.31902	2058.69232	2136.55857	-0.394544399	0.0008678	0.0049393	ZDHHC7	zinc finger DHHC-type containing 7 [Source:HGNC Symbol;Acc:HGNC:18459]
ENSG00000120837	452.739414	601.60219	885.005908	727.089938	-0.61150223	0.0008685	0.0049413	NFYB	nuclear transcription factor Y subunit beta [Source:HGNC Symbol;Acc:HGNC:7805]
ENSG00000178917	166.332524	144.419966	73.7504924	73.4541812	1.077241028	0.0008748	0.004975	ZNF852	zinc finger protein 852 [Source:HGNC Symbol;Acc:HGNC:27713]
ENSG00000125818	2023.54834	2005.93131	2537.01694	2686.93266	-0.374558271	0.000875	0.004975	PSMF1	proteasome inhibitor subunit 1 [Source:HGNC Symbol;Acc:HGNC:9571]
ENSG00000173559	381.875854	445.664067	283.412606	217.168883	0.725650928	0.0008813	0.0050093	NABP1	nucleic acid binding protein 1 [Source:HGNC Symbol;Acc:HGNC:26232]
ENSG00000180329	251.959326	323.394403	451.98516	468.403474	-0.676277642	0.0008821	0.005011	CCDC43	coiled-coil domain containing 43 [Source:HGNC Symbol;Acc:HGNC:26472]
ENSG00000183763	257.864623	264.917606	419.324228	406.65938	-0.659730873	0.0008822	0.005011	TRAIP	TRAF interacting protein [Source:HGNC Symbol;Acc:HGNC:30764]
ENSG00000160221	223.417059	268.461655	405.627708	390.69108	-0.693837808	0.0008915	0.0050597	GATD3A	glutamine amidotransferase like class 1 domain containing 3A [Source:HGNC Symbol;Acc:HGNC:1273]
ENSG00000142546	848.394294	658.306962	1066.2214	1101.81272	-0.525959757	0.0008916	0.0050597	NOSIP	nitric oxide synthase interacting protein [Source:HGNC Symbol;Acc:HGNC:17946]
ENSG00000147044	1014.72682	1142.06955	808.094681	730.283598	0.4878143	0.0008918	0.0050597	CASK	calcium/calmodulin dependent serine protein kinase [Source:HGNC Symbol;Acc:HGNC:1497]
ENSG00000240065	391.718015	375.669114	258.126723	210.781563	0.71031575	0.000892	0.0050597	PSMB9	proteasome subunit beta 9 [Source:HGNC Symbol;Acc:HGNC:9546]
ENSG00000197170	1124.95902	1337.87821	1778.44044	1602.15279	-0.456438279	0.0008931	0.0050635	PSMD12	proteasome 26S subunit, non-ATPase 12 [Source:HGNC Symbol;Acc:HGNC:9557]
ENSG00000141179	234.243436	229.477124	134.858043	118.165422	0.873766514	0.0008933	0.0050635	PCTP	phosphatidylcholine transfer protein [Source:HGNC Symbol;Acc:HGNC:8752]
ENSG00000161999	551.161026	467.814369	742.772816	744.122792	-0.546046482	0.0008949	0.005071	JMJD8	jumonji domain containing 8 [Source:HGNC Symbol;Acc:HGNC:14148]
ENSG00000115163	403.528608	394.275367	552.075114	647.248437	-0.588178059	0.0008964	0.0050781	CENPA	centromere protein A [Source:HGNC Symbol;Acc:HGNC:1851]

ENSG00000078403	451.755198	488.192646	774.38017	629.15103	-0.578095664	0.0009001	0.0050974	MLLT10	MLLT10, histone lysine methyltransferase DOT1L cofactor [Source:HGNC Symbol;Acc:HGNC:16063]
ENSG00000166669	176.174685	201.124738	102.197111	89.4224814	0.977983371	0.0009013	0.005102	ATF7IP2	activating transcription factor 7 interacting protein 2 [Source:HGNC Symbol;Acc:HGNC:20397]
ENSG00000167447	490.139627	489.078658	715.379776	698.346998	-0.529824823	0.0009034	0.0051121	SMG8	SMG8, nonsense mediated mRNA decay factor [Source:HGNC Symbol;Acc:HGNC:25551]
ENSG00000182858	210.622249	186.948545	318.180696	344.915285	-0.738828383	0.0009069	0.0051308	ALG12	ALG12, alpha-1,6-mannosyltransferase [Source:HGNC Symbol;Acc:HGNC:19358]
ENSG00000165185	524.587191	478.446513	361.377413	260.815571	0.688418208	0.0009115	0.0051546	KIAA1958	KIAA1958 [Source:HGNC Symbol;Acc:HGNC:23427]
ENSG00000183258	1900.52132	1546.09105	2352.64071	2300.49979	-0.433542254	0.0009178	0.0051888	DDX41	DEAD-box helicase 41 [Source:HGNC Symbol;Acc:HGNC:18674]
ENSG00000167118	1499.94536	1228.89873	1822.69074	1897.03407	-0.447481383	0.0009199	0.0051987	URM1	ubiquitin related modifier 1 [Source:HGNC Symbol;Acc:HGNC:28378]
ENSG00000178814	60.0371832	68.2229288	139.072357	140.521042	-1.123052469	0.0009204	0.0051987	OPLAH	5-oxoprolinase, ATP-hydrolysing [Source:HGNC Symbol;Acc:HGNC:8149]
ENSG00000152078	326.759751	371.239054	234.947997	171.393089	0.780823773	0.0009206	0.0051987	TMEM56	transmembrane protein 56 [Source:HGNC Symbol;Acc:HGNC:26477]
ENSG00000163528	452.739414	460.726272	676.397373	653.635757	-0.541952311	0.0009208	0.0051987	CHCHD4	coiled-coil-helix-coiled-coil-helix domain containing 4 [Source:HGNC Symbol;Acc:HGNC:26467]
ENSG00000148842	758.830627	683.1153	522.574917	460.951601	0.551507043	0.000922	0.0052038	CNNM2	cyclin and CBS domain divalent metal cation transport mediator 2 [Source:HGNC Symbol;Acc:HGNC:103]
ENSG00000104866	433.055092	428.829838	590.003939	691.959678	-0.572773724	0.0009227	0.0052057	PPP1R37	protein phosphatase 1 regulatory subunit 37 [Source:HGNC Symbol;Acc:HGNC:27607]
ENSG00000097007	4902.38048	4342.34511	5896.87865	5907.20654	-0.352753524	0.0009319	0.0052563	ABL1	ABL proto-oncogene 1, non-receptor tyrosine kinase [Source:HGNC Symbol;Acc:HGNC:76]
ENSG00000102144	34795.9766	34361.3198	42703.6422	42220.1859	-0.296291606	0.0009419	0.005311	PGK1	phosphoglycerate kinase 1 [Source:HGNC Symbol;Acc:HGNC:8896]
ENSG00000155850	428.134011	505.026875	317.127117	272.525658	0.662834433	0.0009429	0.0053146	SLC26A2	solute carrier family 26 member 2 [Source:HGNC Symbol;Acc:HGNC:10994]
ENSG00000178982	2069.8065	1863.28337	2549.65988	2585.80009	-0.385115885	0.0009463	0.0053314	EIF3K	eukaryotic translation initiation factor 3 subunit K [Source:HGNC Symbol;Acc:HGNC:24656]
ENSG00000231500	14181.57	12665.5424	16268.305	17763.1372	-0.342155325	0.0009468	0.0053314	RPS18	ribosomal protein S18 [Source:HGNC Symbol;Acc:HGNC:10401]
ENSG00000060762	216.527546	212.642895	113.786474	117.100869	0.894296264	0.000947	0.0053314	MPC1	mitochondrial pyruvate carrier 1 [Source:HGNC Symbol;Acc:HGNC:21606]
ENSG00000130985	16922.6119	14987.78	20327.7429	19829.4353	-0.331688266	0.0009473	0.0053314	UBA1	ubiquitin like modifier activating enzyme 1 [Source:HGNC Symbol;Acc:HGNC:12469]
ENSG00000229132	219.480194	222.389028	349.788049	367.270906	-0.698350992	0.0009475	0.0053314	EIF4A1P10	eukaryotic translation initiation factor 4A1 pseudogene 10 [Source:HGNC Symbol;Acc:HGNC:37930]
ENSG00000257365	102.358476	93.9172785	168.572554	218.233437	-0.979299974	0.0009514	0.005352	FNTB	farnesyltransferase, CAAX box, beta [Source:HGNC Symbol;Acc:HGNC:3785]
ENSG00000117266	1099.3694	859.4317	649.004333	697.282444	0.540309884	0.000952	0.0053535	CDK18	cyclin dependent kinase 18 [Source:HGNC Symbol;Acc:HGNC:8751]
ENSG00000174837	121.058583	132.901809	48.4646093	61.7440943	1.205061238	0.0009524	0.005354	ADGRE1	adhesion G protein-coupled receptor E1 [Source:HGNC Symbol;Acc:HGNC:3336]
ENSG00000285437	55.1161026	53.1607237	12.6429415	14.9037469	1.974839729	0.0009546	0.0053643	POLR2J3	RNA polymerase II subunit J3 [Source:HGNC Symbol;Acc:HGNC:33853]
ENSG00000160695	419.276066	358.834885	595.271831	573.794256	-0.58823659	0.0009574	0.0053783	VPS11	VPS11, CORVET/HOPS core subunit [Source:HGNC Symbol;Acc:HGNC:14583]
ENSG00000187634	3741.00547	3537.84616	2799.35797	2896.64967	0.353668666	0.000963	0.0054083	SAMD11	sterile alpha motif domain containing 11 [Source:HGNC Symbol;Acc:HGNC:28706]
ENSG00000103005	2603.25163	2262.87481	1850.08378	1834.22542	0.401086025	0.000966	0.0054231	USB1	U6 snRNA biogenesis phosphodiesterase 1 [Source:HGNC Symbol;Acc:HGNC:25792]
ENSG00000166123	1108.22735	859.4317	1311.70519	1486.11648	-0.50858397	0.00097	0.0054439	GPT2	glutamic--pyruvic transaminase 2 [Source:HGNC Symbol;Acc:HGNC:18062]
ENSG00000161082	101.37426	84.1711459	26.3394616	40.4530273	1.473650436	0.0009724	0.0054552	CELF5	CUGBP Elav-like family member 5 [Source:HGNC Symbol;Acc:HGNC:14058]
ENSG00000116679	1705.64653	1765.82204	2422.17688	2152.52687	-0.398061059	0.0009729	0.0054564	IVNS1ABP	influenza virus NS1A binding protein [Source:HGNC Symbol;Acc:HGNC:16951]
ENSG00000140678	47.2423737	33.6684583	8.4286277	6.3873201	2.44728907	0.0009752	0.0054677	ITGAX	integrin subunit alpha X [Source:HGNC Symbol;Acc:HGNC:6152]
ENSG00000135049	408.449689	453.638176	292.894813	249.105484	0.66988108	0.0009777	0.0054799	AGTPBP1	ATP/GTP binding protein 1 [Source:HGNC Symbol;Acc:HGNC:17258]
ENSG00000271303	404.512825	333.140535	219.14432	228.87897	0.718545319	0.0009792	0.0054863	SRXN1	sulfiredoxin 1 [Source:HGNC Symbol;Acc:HGNC:16132]
ENSG00000134490	383.844286	387.187271	215.983585	259.751017	0.696807798	0.0009824	0.0055025	TMEM241	transmembrane protein 241 [Source:HGNC Symbol;Acc:HGNC:31723]
ENSG00000132669	481.281682	477.560501	699.576099	684.507804	-0.529621815	0.0009884	0.0055339	RIN2	Ras and Rab interactor 2 [Source:HGNC Symbol;Acc:HGNC:18750]
ENSG00000143614	937.957961	981.701364	1236.90111	1381.79025	-0.44777018	0.0009889	0.0055353	GATAD2B	GATA zinc finger domain containing 2B [Source:HGNC Symbol;Acc:HGNC:30778]
ENSG00000182154	2056.02747	1508.87854	1169.47209	1322.17526	0.516272476	0.0009908	0.005544	MRPL41	mitochondrial ribosomal protein L41 [Source:HGNC Symbol;Acc:HGNC:14492]
ENSG00000074356	1091.49568	1125.23532	1444.45607	1521.24674	-0.419799102	0.0009951	0.0055663	NCBP3	nuclear cap binding subunit 3 [Source:HGNC Symbol;Acc:HGNC:24612]
ENSG00000089280	8160.13584	6574.2095	9231.45449	10150.5162	-0.395675725	0.0009957	0.0055677	FUS	FUS RNA binding protein [Source:HGNC Symbol;Acc:HGNC:4010]
ENSG00000235552	737.177873	721.213818	897.64885	1262.56027	-0.566778882	0.0009975	0.005576	RPL6P27	ribosomal protein L6 pseudogene 27 [Source:HGNC Symbol;Acc:HGNC:36133]
ENSG00000171617	345.459857	344.658692	231.787262	178.844963	0.748796633	0.0009997	0.0055864	ENC1	ectodermal-neural cortex 1 [Source:HGNC Symbol;Acc:HGNC:3345]
ENSG00000080561	309.043861	312.762258	184.376231	187.36139	0.742252097	0.0010013	0.0055924	MID2	midline 2 [Source:HGNC Symbol;Acc:HGNC:7096]
ENSG00000153879	574.782213	654.762914	927.149047	831.416167	-0.515690151	0.0010018	0.0055924	CEBPG	CCAAT enhancer binding protein gamma [Source:HGNC Symbol;Acc:HGNC:1837]
ENSG00000012963	1659.38838	1735.69763	1230.57964	1323.23981	0.410948437	0.0010018	0.0055924	UBR7	ubiquitin protein ligase E3 component n-recognin 7 (putative) [Source:HGNC Symbol;Acc:HGNC:20344]
ENSG00000169230	1809.97344	1588.61963	2221.99698	2264.30498	-0.400962828	0.001005	0.0056082	PRELID1	PRELI domain containing 1 [Source:HGNC Symbol;Acc:HGNC:30255]
ENSG00000142655	426.165579	394.275367	578.414576	638.73201	-0.56944354	0.001008	0.0056231	PEX14	peroxisomal biogenesis factor 14 [Source:HGNC Symbol;Acc:HGNC:8856]
ENSG00000275464	268.691	321.622378	477.271043	448.17696	-0.6475738	0.0010092	0.0056283	FP565260.1	periodic tryptophan protein 2 homolog [Source:NCBI gene;Acc:102724159]
ENSG00000075043	54.1318865	49.6166755	16.8572554	6.3873201	2.156514995	0.0010127	0.0056457	KCNQ2	potassium voltage-gated channel subfamily Q member 2 [Source:HGNC Symbol;Acc:HGNC:6296]
ENSG00000129244	45.2739414	41.6425669	5.26789231	11.7100869	2.356800322	0.0010159	0.0056617	ATP1B2	ATPase Na+/K+ transporting subunit beta 2 [Source:HGNC Symbol;Acc:HGNC:805]

ENSG00000185561	595.450752	607.804274	366.645305	436.466874	0.583460794	0.001019	0.0056773	TLCD2	TLC domain containing 2 [Source:HGNC Symbol;Acc:HGNC:33522]
ENSG00000136770	952.721202	930.312665	674.290216	680.249591	0.475169355	0.0010206	0.0056844	DNAJC1	DnaJ heat shock protein family (Hsp40) member C1 [Source:HGNC Symbol;Acc:HGNC:20090]
ENSG00000136718	1247.00182	1054.35435	1647.79672	1501.02022	-0.452911671	0.0010216	0.0056882	IMP4	IMP4, U3 small nucleolar ribonucleoprotein [Source:HGNC Symbol;Acc:HGNC:30856]
ENSG00000141933	113.184854	128.471749	229.680105	215.039777	-0.878942292	0.0010226	0.0056916	TPGS1	tubulin polyglutamylase complex subunit 1 [Source:HGNC Symbol;Acc:HGNC:25058]
ENSG00000167632	592.498103	486.420622	343.466579	358.754479	0.61882982	0.0010248	0.005702	TRAPPC9	trafficking protein particle complex 9 [Source:HGNC Symbol;Acc:HGNC:30832]
ENSG00000151552	446.834118	373.89709	601.593302	624.892817	-0.580534913	0.0010361	0.0057629	QDPR	quinoid dihydropteridine reductase [Source:HGNC Symbol;Acc:HGNC:9752]
ENSG00000125991	3007.76446	2358.56411	3453.6302	3790.87448	-0.433319445	0.0010365	0.0057631	ERGIC3	ERGIC and golgi 3 [Source:HGNC Symbol;Acc:HGNC:15927]
ENSG00000056487	124.011231	119.611628	55.8396585	50.0340075	1.202009215	0.0010393	0.005777	PHF21B	PHD finger protein 21B [Source:HGNC Symbol;Acc:HGNC:25161]
ENSG00000100380	4000.83852	4241.33974	5047.69441	5401.5437	-0.342194789	0.0010417	0.0057884	ST13	ST13, Hsp70 interacting protein [Source:HGNC Symbol;Acc:HGNC:11343]
ENSG00000240849	937.957961	1018.02786	620.557714	751.574665	0.511827561	0.0010437	0.0057976	TMEM189	transmembrane protein 189 [Source:HGNC Symbol;Acc:HGNC:16735]
ENSG00000001497	2548.13553	2383.37245	3079.60985	3267.11423	-0.364132125	0.0010465	0.0058113	LAS1L	LAS1 like, ribosome biogenesis factor [Source:HGNC Symbol;Acc:HGNC:25726]
ENSG00000174516	515.729246	416.425669	267.608929	316.172345	0.67441861	0.0010472	0.0058134	PELI3	pellino E3 ubiquitin protein ligase family member 3 [Source:HGNC Symbol;Acc:HGNC:30010]
ENSG00000166197	3286.29762	3339.37946	4144.77767	4228.40591	-0.337671223	0.0010487	0.005818	NOLC1	nucleolar and coiled-body phosphoprotein 1 [Source:HGNC Symbol;Acc:HGNC:15608]
ENSG00000107815	675.172257	580.3379	850.237819	943.194268	-0.515119768	0.0010487	0.005818	TWNK	twinkle mtDNA helicase [Source:HGNC Symbol;Acc:HGNC:1160]
ENSG00000128185	694.856579	663.623034	942.952724	944.258822	-0.474497852	0.0010494	0.0058199	DGCR6L	DiGeorge syndrome critical region gene 6 like [Source:HGNC Symbol;Acc:HGNC:18551]
ENSG00000236017	107.279557	85.0571579	28.4466185	42.582134	1.436556751	0.0010524	0.0058335	ASMTL-AS1	ASMTL antisense RNA 1 [Source:HGNC Symbol;Acc:HGNC:25811]
ENSG00000171604	761.783275	682.229288	975.613656	1037.93952	-0.480154407	0.0010526	0.0058335	CXXC5	CXXC finger protein 5 [Source:HGNC Symbol;Acc:HGNC:26943]
ENSG00000013588	14567.3828	13437.2589	10748.6075	11578.0822	0.326859226	0.001055	0.0058439	GPRC5A	G protein-coupled receptor class C group 5 member A [Source:HGNC Symbol;Acc:HGNC:9836]
ENSG00000174446	197.82744	204.668786	310.805646	355.560819	-0.727026046	0.0010551	0.0058439	SNAPC5	small nuclear RNA activating complex polypeptide 5 [Source:HGNC Symbol;Acc:HGNC:15484]
ENSG00000136895	120.074366	102.777399	43.196717	48.9694541	1.273153334	0.0010583	0.0058593	GARNL3	GTPase activating Rap/RanGAP domain like 3 [Source:HGNC Symbol;Acc:HGNC:25425]
ENSG00000157570	44.2897253	28.352386	5.26789231	6.3873201	2.638329826	0.0010586	0.0058593	TSPAN18	tetraspanin 18 [Source:HGNC Symbol;Acc:HGNC:20660]
ENSG00000105193	9907.11945	8931.88759	11438.7014	12354.1416	-0.336876204	0.001063	0.0058816	RPS16	ribosomal protein S16 [Source:HGNC Symbol;Acc:HGNC:10396]
ENSG00000067955	902.52618	984.359401	1387.56283	1208.26805	-0.459894585	0.0010694	0.0059149	CBFB	core-binding factor subunit beta [Source:HGNC Symbol;Acc:HGNC:1539]
ENSG00000174606	261.801487	311.876246	466.735259	434.337767	-0.6503306	0.0010726	0.005931	ANGEL2	angel homolog 2 [Source:HGNC Symbol;Acc:HGNC:30534]
ENSG00000275874	645.645774	511.22896	827.059093	856.965447	-0.542729575	0.0010761	0.0059481	PICSAR	P38 inhibited cutaneous squamous cell carcinoma associated lincRNA [Source:HGNC Symbol;Acc:HGNC:19725]
ENSG00000235437	749.972682	703.493577	466.735259	534.405782	0.537684101	0.0010779	0.0059561	LINC01278	long intergenic non-protein coding RNA 1278 [Source:HGNC Symbol;Acc:HGNC:28090]
ENSG00000124226	2324.71847	2186.67777	1661.49324	1788.44963	0.386881319	0.0010842	0.0059891	RNF114	ring finger protein 114 [Source:HGNC Symbol;Acc:HGNC:13094]
ENSG00000186493	162.39566	143.533954	243.376625	295.945831	-0.818764058	0.0010864	0.0059996	C5orf38	chromosome 5 open reading frame 38 [Source:HGNC Symbol;Acc:HGNC:24226]
ENSG00000107959	903.510397	823.991217	1119.95391	1263.62483	-0.464785051	0.0010907	0.006021	PITRM1	pitrilysin metallopeptidase 1 [Source:HGNC Symbol;Acc:HGNC:17663]
ENSG00000124688	363.175748	364.150957	508.878397	587.633449	-0.592162913	0.0010963	0.0060495	MAD2L1BP	MAD2L1 binding protein [Source:HGNC Symbol;Acc:HGNC:21059]
ENSG00000121039	484.23433	548.441466	336.091529	343.850732	0.603459408	0.0010966	0.0060495	RDH10	retinol dehydrogenase 10 [Source:HGNC Symbol;Acc:HGNC:19975]
ENSG00000124587	652.535286	539.581346	388.770453	402.401166	0.590741666	0.0010988	0.0060598	PEX6	peroxisomal biogenesis factor 6 [Source:HGNC Symbol;Acc:HGNC:8859]
ENSG00000179348	3869.93778	3351.78363	2687.67866	2857.26119	0.380966985	0.0010998	0.0060634	GATA2	GATA binding protein 2 [Source:HGNC Symbol;Acc:HGNC:4171]
ENSG00000196220	85.6268023	85.94317	38.9824031	17.0328536	1.614074074	0.0011015	0.0060709	SRGAP3	SLIT-ROBO Rho GTPase activating protein 3 [Source:HGNC Symbol;Acc:HGNC:19744]
ENSG00000120616	120.074366	116.06758	205.4478	228.87897	-0.879347431	0.0011023	0.0060732	EPC1	enhancer of polycomb homolog 1 [Source:HGNC Symbol;Acc:HGNC:19876]
ENSG00000111707	1695.80437	1817.21074	1279.04425	1370.08016	0.407410417	0.0011036	0.0060783	SUDS3	SDS3 homolog, SIN3A corepressor complex component [Source:HGNC Symbol;Acc:HGNC:29545]
ENSG00000128016	305.106997	315.420294	192.804859	178.844963	0.739651403	0.001111	0.0061173	ZFP36	ZFP36 ring finger protein [Source:HGNC Symbol;Acc:HGNC:12862]
ENSG00000148297	1238.14388	990.561485	1530.84951	1543.60236	-0.464839094	0.0011144	0.006134	MED22	mediator complex subunit 22 [Source:HGNC Symbol;Acc:HGNC:11477]
ENSG00000163624	267.706784	312.762258	189.644123	136.262829	0.833154014	0.0011183	0.0061536	CDS1	CDP-diacylglycerol synthase 1 [Source:HGNC Symbol;Acc:HGNC:1800]
ENSG00000122420	160.427227	178.088424	310.805646	269.331998	-0.776474176	0.0011243	0.0061843	PTGFR	prostaglandin F receptor [Source:HGNC Symbol;Acc:HGNC:9600]
ENSG00000224631	233.25922	225.047064	343.466579	397.0784	-0.692450236	0.0011267	0.0061956	RPS27AP16	ribosomal protein S27a pseudogene 16 [Source:HGNC Symbol;Acc:HGNC:36855]
ENSG00000174197	1646.59357	1755.18989	2400.05174	2099.29921	-0.403278297	0.0011273	0.0061967	MGA	MGA, MAX dimerization protein [Source:HGNC Symbol;Acc:HGNC:14010]
ENSG00000122694	272.627865	277.321775	169.626132	149.037469	0.78724004	0.0011276	0.0061967	GLIPR2	GLI pathogenesis related 2 [Source:HGNC Symbol;Acc:HGNC:18007]
ENSG00000108379	255.896191	226.819088	369.80604	402.401166	-0.678598547	0.0011293	0.006204	WNT3	Wnt family member 3 [Source:HGNC Symbol;Acc:HGNC:12782]
ENSG00000132305	2364.08712	2496.78199	3038.52029	3194.7246	-0.358622344	0.0011346	0.0062306	IMMT	inner membrane mitochondrial protein [Source:HGNC Symbol;Acc:HGNC:6047]
ENSG00000165355	629.898316	655.648926	464.628102	411.982147	0.552464478	0.0011356	0.0062345	FBXO33	F-box protein 33 [Source:HGNC Symbol;Acc:HGNC:19833]
ENSG00000140459	373.017909	269.347667	486.75325	535.470335	-0.671913223	0.0011376	0.0062431	CYP11A1	cytochrome P450 family 11 subfamily A member 1 [Source:HGNC Symbol;Acc:HGNC:2590]
ENSG00000128266	269.675216	240.995281	136.9652	152.231129	0.819888922	0.001139	0.006249	GNAZ	G protein subunit alpha z [Source:HGNC Symbol;Acc:HGNC:4395]
ENSG00000198720	1024.56898	979.92934	734.344188	719.638065	0.463054221	0.0011411	0.0062586	ANKRD13B	ankyrin repeat domain 13B [Source:HGNC Symbol;Acc:HGNC:26363]
ENSG00000107736	73.8162089	61.1348323	17.9108339	23.4201737	1.706607253	0.0011419	0.006261	CDH23	cadherin related 23 [Source:HGNC Symbol;Acc:HGNC:13733]

ENSG00000140983	1806.03658	1591.27766	2167.2109	2316.46809	-0.400632038	0.0011437	0.0062681	RHOT2	ras homolog family member T2 [Source:HGNC Symbol;Acc:HGNC:21169]
ENSG00000128563	476.360601	434.14591	643.73644	681.314144	-0.541812731	0.001144	0.0062681	PRKRIP1	PRKR interacting protein 1 [Source:HGNC Symbol;Acc:HGNC:21894]
ENSG00000137802	660.409015	596.286118	448.824425	401.336613	0.563343273	0.0011463	0.0062789	MAPKBP1	mitogen-activated protein kinase binding protein 1 [Source:HGNC Symbol;Acc:HGNC:29536]
ENSG00000239521	191.922143	188.720569	103.250689	95.8098015	0.935075517	0.0011494	0.0062939	CASTOR3	CASTOR family member 3 [Source:HGNC Symbol;Acc:HGNC:29954]
ENSG00000126903	760.799059	699.949529	959.809979	1076.26344	-0.479421029	0.0011524	0.0063082	SLC10A3	solute carrier family 10 member 3 [Source:HGNC Symbol;Acc:HGNC:22979]
ENSG00000115282	341.522993	294.156005	462.520945	517.372928	-0.625189674	0.0011569	0.0063305	TTC31	tetratricopeptide repeat domain 31 [Source:HGNC Symbol;Acc:HGNC:25759]
ENSG00000141698	1383.80786	1250.16302	1687.8327	1820.38623	-0.413825234	0.00116	0.0063456	NT5C3B	5'-nucleotidase, cytosolic IIIB [Source:HGNC Symbol;Acc:HGNC:28300]
ENSG00000164849	13.7790257	13.2901809	65.3218647	41.5175807	-1.981398962	0.0011605	0.0063466	GPR146	G protein-coupled receptor 146 [Source:HGNC Symbol;Acc:HGNC:21718]
ENSG00000078081	54.1318865	65.5648926	15.8036769	18.097407	1.821045601	0.0011647	0.0063675	LAMP3	lysosomal associated membrane protein 3 [Source:HGNC Symbol;Acc:HGNC:14582]
ENSG00000103549	1581.6353	1323.70202	1987.04898	1913.00237	-0.425318869	0.0011703	0.006396	RNF40	ring finger protein 40 [Source:HGNC Symbol;Acc:HGNC:16867]
ENSG00000197747	6477.12627	7000.3813	7814.39146	10056.8355	-0.40701638	0.0011712	0.0063988	S100A10	S100 calcium binding protein A10 [Source:HGNC Symbol;Acc:HGNC:10487]
ENSG00000183741	3223.30779	2844.98473	2184.06815	2441.02083	0.391625962	0.0011727	0.0064046	CBX6	chromobox 6 [Source:HGNC Symbol;Acc:HGNC:1556]
ENSG00000139180	403.528608	335.798571	526.789231	597.214429	-0.605339458	0.0011736	0.0064074	NDUFA9	NADH:ubiquinone oxidoreductase subunit A9 [Source:HGNC Symbol;Acc:HGNC:7693]
ENSG00000101639	396.639096	442.120019	689.040314	568.471489	-0.583744058	0.0011747	0.0064113	CEP192	centrosomal protein 192 [Source:HGNC Symbol;Acc:HGNC:25515]
ENSG00000139364	30.5106997	23.0363136	2.10715692	3.19366005	3.336265855	0.001175	0.0064113	TMEM132B	transmembrane protein 132B [Source:HGNC Symbol;Acc:HGNC:29397]
ENSG00000099889	201.764304	180.746461	93.7684831	105.390782	0.941142495	0.0011792	0.0064312	ARVCF	ARVCF, delta catenin family member [Source:HGNC Symbol;Acc:HGNC:728]
ENSG00000198786	36110.8894	34953.1758	41830.2257	46651.9215	-0.316271218	0.0011794	0.0064312	MT-ND5	mitochondrially encoded NADH:ubiquinone oxidoreductase core subunit 5 [Source:HGNC Symbol;Acc:HGNC:7461]
ENSG00000103187	8743.77599	7813.74037	9941.56637	11166.1001	-0.350358723	0.0011835	0.0064482	COTL1	coactosin like F-actin binding protein 1 [Source:HGNC Symbol;Acc:HGNC:18304]
ENSG00000198586	385.812718	426.171802	734.344188	528.018462	-0.636254293	0.0011837	0.0064482	TLK1	tousled like kinase 1 [Source:HGNC Symbol;Acc:HGNC:11841]
ENSG00000074201	2242.04432	2322.23761	2878.37636	2961.58742	-0.355470785	0.0011837	0.0064482	CLNS1A	chloride nucleotide-sensitive channel 1A [Source:HGNC Symbol;Acc:HGNC:2080]
ENSG00000247516	327.743967	303.016125	189.644123	190.55505	0.729957445	0.0011852	0.0064545	MIR4458HG	MIR4458 host gene [Source:HGNC Symbol;Acc:HGNC:49008]
ENSG00000181873	100.390044	90.3732303	192.804859	175.651303	-0.950661217	0.0011869	0.0064612	IBA57	IBA57, iron-sulfur cluster assembly [Source:HGNC Symbol;Acc:HGNC:27302]
ENSG00000151491	1242.08074	1300.66571	993.52449	867.61098	0.450285187	0.0011896	0.0064736	EPS8	epidermal growth factor receptor pathway substrate 8 [Source:HGNC Symbol;Acc:HGNC:3420]
ENSG00000168237	61.0213993	31.8964342	105.357846	129.875509	-1.345412637	0.0011899	0.0064736	GLYCTK	glycerate kinase [Source:HGNC Symbol;Acc:HGNC:24247]
ENSG00000105419	859.220671	761.970373	565.771634	573.794256	0.508119958	0.0011919	0.0064826	MEIS3	Meis homeobox 3 [Source:HGNC Symbol;Acc:HGNC:29537]
ENSG00000100335	1184.99621	1219.1526	1649.90387	1540.4087	-0.408085144	0.0011943	0.0064932	MIEF1	mitochondrial elongation factor 1 [Source:HGNC Symbol;Acc:HGNC:25979]
ENSG00000004897	1106.25892	1439.7696	2047.10295	1602.15279	-0.518809314	0.0011948	0.0064933	CDC27	cell division cycle 27 [Source:HGNC Symbol;Acc:HGNC:1728]
ENSG00000105443	786.388678	649.446841	980.881548	1046.45594	-0.498492267	0.0011953	0.0064933	CYTH2	cytohesin 2 [Source:HGNC Symbol;Acc:HGNC:9502]
ENSG00000168259	1215.50691	1305.09577	1708.90427	1630.89573	-0.405744683	0.0011955	0.0064933	DNAJC7	DnaJ heat shock protein family (Hsp40) member C7 [Source:HGNC Symbol;Acc:HGNC:12392]
ENSG00000078808	3505.77781	3091.29608	4142.67051	4330.60303	-0.361311519	0.001196	0.0064941	SDF4	stromal cell derived factor 4 [Source:HGNC Symbol;Acc:HGNC:24188]
ENSG00000087274	2045.20109	2124.65692	2568.62429	2837.03468	-0.374349091	0.001198	0.0065031	ADD1	adducin 1 [Source:HGNC Symbol;Acc:HGNC:243]
ENSG00000104549	2135.74898	2296.54326	1543.49245	1794.83695	0.409125955	0.0011986	0.0065032	SQLE	squalene epoxidase [Source:HGNC Symbol;Acc:HGNC:11279]
ENSG00000018408	738.162089	782.348651	578.414576	455.628834	0.556331125	0.0011988	0.0065032	WWTR1	WW domain containing transcription regulator 1 [Source:HGNC Symbol;Acc:HGNC:24042]
ENSG00000158470	3547.11489	3638.85154	4530.38739	4505.18978	-0.330385397	0.0012013	0.0065146	B4GALT5	beta-1,4-galactosyltransferase 5 [Source:HGNC Symbol;Acc:HGNC:928]
ENSG00000272695	49.2108059	50.5026875	13.69652	10.6455335	2.034090326	0.0012065	0.0065407	GAS6-DT	GAS6 divergent transcript [Source:HGNC Symbol;Acc:HGNC:43694]
ENSG00000107295	229.322356	225.933076	132.750886	119.229975	0.853192661	0.0012077	0.0065453	SH3GL2	SH3 domain containing GRB2 like 2, endophilin A1 [Source:HGNC Symbol;Acc:HGNC:10831]
ENSG00000126001	1428.09759	1220.03861	1712.065	1851.25828	-0.428720905	0.0012111	0.0065614	CEP250	centrosomal protein 250 [Source:HGNC Symbol;Acc:HGNC:1859]
ENSG00000121068	233.25922	184.290509	118.000788	101.132568	0.928930045	0.0012121	0.0065647	TBX2	T-box 2 [Source:HGNC Symbol;Acc:HGNC:11597]
ENSG00000160633	2927.05874	2408.18078	3309.28995	3821.74653	-0.418858797	0.0012128	0.0065664	SAFB	scaffold attachment factor B [Source:HGNC Symbol;Acc:HGNC:10520]
ENSG00000109472	1456.63985	1367.11661	1051.47131	1060.29514	0.418967314	0.0012132	0.0065666	CPE	carboxypeptidase E [Source:HGNC Symbol;Acc:HGNC:2303]
ENSG00000125351	369.081044	452.752164	607.914773	620.634603	-0.578910349	0.0012171	0.0065855	UPF3B	UPF3B, regulator of nonsense mediated mRNA decay [Source:HGNC Symbol;Acc:HGNC:20439]
ENSG00000128039	377.938989	428.829838	251.805252	258.686464	0.660865937	0.0012182	0.0065892	SRD5A3	steroid 5 alpha-reductase 3 [Source:HGNC Symbol;Acc:HGNC:25812]
ENSG00000248508	96.4531796	87.7151941	28.4466185	40.4530273	1.418401474	0.0012246	0.006622	SRP14-AS1	SRP14 antisense RNA1 (head to head) [Source:HGNC Symbol;Acc:HGNC:48619]
ENSG00000139292	36.4159964	27.4663739	6.32147077	2.1291067	2.915333863	0.0012319	0.0066594	LGR5	leucine rich repeat containing G protein-coupled receptor 5 [Source:HGNC Symbol;Acc:HGNC:4504]
ENSG00000133250	159.443011	150.622051	280.251871	254.428251	-0.786598029	0.0012332	0.006664	ZNF414	zinc finger protein 414 [Source:HGNC Symbol;Acc:HGNC:20630]
ENSG00000156853	217.511762	206.44081	349.788049	336.398859	-0.695100283	0.0012457	0.0067294	ZNF689	zinc finger protein 689 [Source:HGNC Symbol;Acc:HGNC:25173]
ENSG00000100324	609.229777	499.710803	819.684044	781.382159	-0.530827837	0.0012486	0.006743	TAB1	TGF-beta activated kinase 1 (MAP3K7) binding protein 1 [Source:HGNC Symbol;Acc:HGNC:18157]
ENSG00000148158	862.17332	910.8204	659.540117	606.79541	0.485688405	0.0012492	0.0067438	SNX30	sorting nexin family member 30 [Source:HGNC Symbol;Acc:HGNC:23685]

ENSG00000109016	519.66611	504.140863	322.395009	356.625372	0.592351585	0.0012512	0.0067527	DHRS7B	dehydrogenase/reductase 7B [Source:HGNC Symbol;Acc:HGNC:24547]
ENSG00000130935	1135.7854	1339.65024	1756.3153	1598.95913	-0.438245856	0.0012541	0.0067664	NOL11	nucleolar protein 11 [Source:HGNC Symbol;Acc:HGNC:24557]
ENSG00000182979	2386.72409	2140.60514	2846.76901	3016.94419	-0.373405451	0.0012557	0.006771	MTA1	metastasis associated 1 [Source:HGNC Symbol;Acc:HGNC:7410]
ENSG00000143315	687.967067	739.820072	490.967563	505.662841	0.51896807	0.0012558	0.006771	PIGM	phosphatidylinositol glycan anchor biosynthesis class M [Source:HGNC Symbol;Acc:HGNC:18858]
ENSG00000125107	5640.54257	5961.97516	7962.94602	6864.24	-0.353762264	0.0012573	0.006777	CNOT1	CCR4-NOT transcription complex subunit 1 [Source:HGNC Symbol;Acc:HGNC:7877]
ENSG00000105364	802.136136	785.006687	1090.45371	1070.94067	-0.445641668	0.0012587	0.0067804	MRPL4	mitochondrial ribosomal protein L4 [Source:HGNC Symbol;Acc:HGNC:14276]
ENSG00000111247	365.14418	427.943826	708.004727	523.760248	-0.634532238	0.0012587	0.0067804	RAD51AP1	RAD51 associated protein 1 [Source:HGNC Symbol;Acc:HGNC:16956]
ENSG00000204291	71.8477766	76.1970373	23.1787262	26.6138338	1.572483512	0.0012642	0.0068079	COL15A1	collagen type XV alpha 1 chain [Source:HGNC Symbol;Acc:HGNC:2192]
ENSG00000167085	2924.10609	2742.20733	3571.63099	3627.99782	-0.34565393	0.0012651	0.0068105	PHB	prohibitin [Source:HGNC Symbol;Acc:HGNC:8912]
ENSG00000103966	1721.39399	1599.25177	1227.41891	1280.65768	0.404688535	0.0012678	0.0068224	EHD4	EH domain containing 4 [Source:HGNC Symbol;Acc:HGNC:3245]
ENSG00000160746	953.705419	939.172785	673.236637	697.282444	0.465819817	0.001269	0.0068268	ANO10	anoctamin 10 [Source:HGNC Symbol;Acc:HGNC:25519]
ENSG00000118518	281.48581	252.513438	446.717268	392.820186	-0.653531339	0.0012701	0.0068305	RNF146	ring finger protein 146 [Source:HGNC Symbol;Acc:HGNC:21336]
ENSG00000171148	2384.75565	2183.13372	2779.33998	3203.24103	-0.389407961	0.0012708	0.0068326	TADA3	transcriptional adaptor 3 [Source:HGNC Symbol;Acc:HGNC:19422]
ENSG00000177045	235.227652	172.772352	342.413	342.786179	-0.749942729	0.0012737	0.0068459	SIX5	SIX homeobox 5 [Source:HGNC Symbol;Acc:HGNC:10891]
ENSG00000174227	709.619821	669.825119	898.702428	1031.5522	-0.484919938	0.0012758	0.0068549	PIGG	phosphatidylinositol glycan anchor biosynthesis class G [Source:HGNC Symbol;Acc:HGNC:25985]
ENSG00000157423	131.88496	96.5753147	205.4478	224.620757	-0.914956899	0.0012783	0.0068659	HYDIN	HYDIN, axonemal central pair apparatus protein [Source:HGNC Symbol;Acc:HGNC:19368]
ENSG00000105808	41.337077	31.8964342	7.37504924	5.32276675	2.526261539	0.0012802	0.0068741	RASA4	RAS p21 protein activator 4 [Source:HGNC Symbol;Acc:HGNC:23181]
ENSG00000100591	3616.99423	3348.23958	4257.51057	4613.77422	-0.349097184	0.0012857	0.0069014	AHSA1	activator of HSP90 ATPase activity 1 [Source:HGNC Symbol;Acc:HGNC:1189]
ENSG00000108639	2274.52345	2048.45989	1626.72515	1675.60697	0.388303096	0.0012877	0.0069098	SYNGR2	synaptogyrin 2 [Source:HGNC Symbol;Acc:HGNC:11499]
ENSG00000003249	363.175748	329.596487	512.039133	526.953908	-0.585330136	0.0012929	0.0069356	DBNDD1	dysbindin domain containing 1 [Source:HGNC Symbol;Acc:HGNC:28455]
ENSG00000137070	97.4373957	76.1970373	33.7145108	29.8074938	1.449425109	0.0012992	0.0069674	IL11RA	interleukin 11 receptor subunit alpha [Source:HGNC Symbol;Acc:HGNC:5967]
ENSG00000174744	1024.56898	943.602846	1311.70519	1332.82079	-0.426494759	0.0013018	0.0069776	BRMS1	BRMS1, transcriptional repressor and anoikis regulator [Source:HGNC Symbol;Acc:HGNC:17262]
ENSG00000117707	10.8263773	15.9482171	0	0	6.104009073	0.001302	0.0069776	PROX1	prospero homeobox 1 [Source:HGNC Symbol;Acc:HGNC:9459]
ENSG00000124422	4213.4292	3917.05933	5133.03427	5097.08144	-0.331533674	0.0013064	0.0069991	USP22	ubiquitin specific peptidase 22 [Source:HGNC Symbol;Acc:HGNC:12621]
ENSG00000182473	868.078616	776.146566	1249.54406	1047.5205	-0.482880093	0.0013093	0.0070117	EXOC7	exocyst complex component 7 [Source:HGNC Symbol;Acc:HGNC:23214]
ENSG00000229891	154.521931	132.015797	52.6789231	77.7123946	1.135531336	0.0013096	0.0070117	LINC01315	long intergenic non-protein coding RNA 1315 [Source:HGNC Symbol;Acc:HGNC:50513]
ENSG00000104774	1595.41433	1432.6815	1083.07866	1171.00869	0.425594985	0.0013114	0.0070195	MAN2B1	mannosidase alpha class 2B member 1 [Source:HGNC Symbol;Acc:HGNC:6826]
ENSG00000159307	13.7790257	37.2125066	0	3.19366005	4.004607669	0.0013152	0.0070374	SCUBE1	signal peptide, CUB domain and EGF like domain containing 1 [Source:HGNC Symbol;Acc:HGNC:13441]
ENSG00000167987	429.118228	338.456608	552.075114	614.247283	-0.604802902	0.0013166	0.0070428	VPS37C	VPS37C, ESCRT-I subunit [Source:HGNC Symbol;Acc:HGNC:26097]
ENSG00000101057	4732.1111	4403.47995	5607.14458	5884.85092	-0.33116193	0.0013171	0.0070431	MYBL2	MYB proto-oncogene like 2 [Source:HGNC Symbol;Acc:HGNC:7548]
ENSG00000148362	1047.20595	832.851338	1246.38332	1384.98391	-0.485782624	0.001319	0.0070512	PAXX	PAXX, non-homologous end joining factor [Source:HGNC Symbol;Acc:HGNC:27849]
ENSG00000103381	79.7215056	83.2851338	172.786868	153.295682	-0.999985593	0.0013226	0.0070683	CPPED1	calcineurin like phosphoesterase domain containing 1 [Source:HGNC Symbol;Acc:HGNC:25632]
ENSG00000185359	1947.7637	1713.54733	2406.37321	2376.08308	-0.385749803	0.0013256	0.0070821	HGS	hepatocyte growth factor-regulated tyrosine kinase substrate [Source:HGNC Symbol;Acc:HGNC:4897]
ENSG00000163104	290.343755	349.974764	605.807616	427.950447	-0.690249228	0.0013273	0.0070859	SMARCAD1	SWI/SNF-related, matrix-associated actin-dependent regulator of chromatin, subfamily a, containing DEAD/H box 1 [Source:HGNC Symbol;Acc:HGNC:18398]
ENSG00000221955	1290.30733	1255.47909	1621.45725	1735.22196	-0.399003528	0.0013275	0.0070859	SLC12A8	solute carrier family 12 member 8 [Source:HGNC Symbol;Acc:HGNC:15595]
ENSG00000124374	212.590682	256.943498	133.804465	126.681849	0.850874636	0.0013278	0.0070859	PAIP2B	poly(A) binding protein interacting protein 2B [Source:HGNC Symbol;Acc:HGNC:29200]
ENSG00000130513	585.60859	528.949201	768.058699	812.254206	-0.504252565	0.001328	0.0070859	GDF15	growth differentiation factor 15 [Source:HGNC Symbol;Acc:HGNC:30142]
ENSG00000175984	128.932311	183.404497	85.3398554	56.4213276	1.140694973	0.0013315	0.0071021	DENND2C	DENN domain containing 2C [Source:HGNC Symbol;Acc:HGNC:24748]
ENSG00000125449	395.65488	341.114644	565.771634	536.534889	-0.582148111	0.0013365	0.007125	ARMC7	armadillo repeat containing 7 [Source:HGNC Symbol;Acc:HGNC:26168]
ENSG00000137411	632.850964	547.555454	809.148259	866.546427	-0.506164334	0.0013366	0.007125	VARS2	valyl-tRNA synthetase 2, mitochondrial [Source:HGNC Symbol;Acc:HGNC:21642]
ENSG00000179922	72.8319927	66.4509046	139.072357	151.166576	-1.060016715	0.0013404	0.0071427	ZNF784	zinc finger protein 784 [Source:HGNC Symbol;Acc:HGNC:33111]
ENSG00000135643	354.317803	335.798571	221.251477	205.458797	0.693265937	0.0013408	0.0071427	KCNMB4	potassium calcium-activated channel subfamily M regulatory beta subunit 4 [Source:HGNC Symbol;Acc:HGNC:6289]
ENSG00000101474	5075.60252	4702.06601	3725.45344	3992.07506	0.341261977	0.0013474	0.0071758	APMAP	adipocyte plasma membrane associated protein [Source:HGNC Symbol;Acc:HGNC:13238]
ENSG00000239306	2086.53817	1807.46461	2401.10532	2776.35514	-0.411290483	0.00136	0.0072402	RBM14	RNA binding motif protein 14 [Source:HGNC Symbol;Acc:HGNC:14219]
ENSG00000158467	572.813781	645.902793	404.57413	424.756787	0.555872837	0.0013604	0.0072402	AHCYL2	adenosylhomocysteinase like 2 [Source:HGNC Symbol;Acc:HGNC:22204]
ENSG00000149679	699.77766	661.85101	456.199474	489.694541	0.525395136	0.0013617	0.007245	CABLES2	Cdk5 and Abl enzyme substrate 2 [Source:HGNC Symbol;Acc:HGNC:16143]
ENSG00000115977	1027.52163	1138.5255	807.041102	771.801179	0.456526833	0.0013638	0.0072538	AAK1	AP2 associated kinase 1 [Source:HGNC Symbol;Acc:HGNC:19679]
ENSG00000249306	336.601912	356.176849	219.14432	210.781563	0.688549552	0.0013648	0.0072567	LINC01411	long intergenic non-protein coding RNA 1411 [Source:HGNC Symbol;Acc:HGNC:50703]

ENSG00000163743	77.7530733	93.0312665	225.465791	135.198275	-1.077390387	0.0013655	0.0072582	RCHY1	ring finger and CHY zinc finger domain containing 1 [Source:HGNC Symbol;Acc:HGNC:17479]
ENSG00000276550	1065.90606	1052.58233	735.397767	810.1251	0.454945443	0.0013724	0.0072928	HERC2P2	hect domain and RLD 2 pseudogene 2 [Source:HGNC Symbol;Acc:HGNC:4870]
ENSG00000229953	68.8951283	59.3628081	17.9108339	21.291067	1.709573615	0.0013737	0.0072974	AL590666.2	novel transcript
ENSG00000167552	6152.33495	6056.77845	4988.69402	4809.65204	0.317322665	0.0013748	0.0073008	TUBA1A	tubulin alpha 1a [Source:HGNC Symbol;Acc:HGNC:20766]
ENSG00000132313	601.356048	663.623034	859.720025	910.193114	-0.484046829	0.0013758	0.0073041	MRPL35	mitochondrial ribosomal protein L35 [Source:HGNC Symbol;Acc:HGNC:14489]
ENSG00000169031	71.8477766	73.5390011	16.8572554	29.8074938	1.640253831	0.0013778	0.0073122	COL4A3	collagen type IV alpha 3 chain [Source:HGNC Symbol;Acc:HGNC:2204]
ENSG00000044115	10992.7098	10768.5906	13058.0515	13801.9342	-0.303705518	0.0013786	0.0073141	CTNNA1	catenin alpha 1 [Source:HGNC Symbol;Acc:HGNC:2509]
ENSG00000185697	427.149795	466.928357	315.01996	255.492804	0.648372839	0.001385	0.0073457	MYBL1	MYB proto-oncogene like 1 [Source:HGNC Symbol;Acc:HGNC:7547]
ENSG00000135828	158.458795	185.176521	94.8220616	77.7123946	0.994554827	0.0013861	0.0073492	RNASEL	ribonuclease L [Source:HGNC Symbol;Acc:HGNC:10050]
ENSG00000171097	596.434968	545.78343	347.680893	414.111253	0.584122433	0.0013888	0.0073614	KYAT1	kynurenine aminotransferase 1 [Source:HGNC Symbol;Acc:HGNC:1564]
ENSG00000245849	142.711337	118.725616	63.2147077	55.3567742	1.139679681	0.0013944	0.0073877	RAD51-AS1	RAD51 antisense RNA 1 [Source:HGNC Symbol;Acc:HGNC:48621]
ENSG00000170786	90.5478829	84.1711459	30.5537754	35.1302606	1.411224987	0.0013946	0.0073877	SDR16C5	short chain dehydrogenase/reductase family 16C member 5 [Source:HGNC Symbol;Acc:HGNC:30311]
ENSG00000143368	2608.17271	2487.92187	3083.82416	3489.60588	-0.367333868	0.0013951	0.007388	SF3B4	splicing factor 3b subunit 4 [Source:HGNC Symbol;Acc:HGNC:10771]
ENSG00000116489	1871.97906	2319.57958	3016.39514	2631.57588	-0.42985475	0.0013962	0.0073915	CAPZA1	capping actin protein of muscle Z-line subunit alpha 1 [Source:HGNC Symbol;Acc:HGNC:1488]
ENSG00000071462	1851.31052	1632.03422	2176.6931	2418.66521	-0.400018249	0.0014005	0.007411	BUD23	BUD23, rRNA methyltransferase and ribosome maturation factor [Source:HGNC Symbol;Acc:HGNC:16405]
ENSG00000177508	265.738352	276.435763	391.931188	454.564281	-0.642414277	0.0014008	0.007411	IRX3	iroquois homeobox 3 [Source:HGNC Symbol;Acc:HGNC:14360]
ENSG00000154839	231.290788	223.27504	326.609323	413.0467	-0.702485161	0.0014018	0.0074138	SKA1	spindle and kinetochore associated complex subunit 1 [Source:HGNC Symbol;Acc:HGNC:28109]
ENSG00000160948	930.084232	865.633784	1181.06146	1245.52742	-0.434664487	0.0014075	0.0074419	VPS28	VPS28, ESCRT-I subunit [Source:HGNC Symbol;Acc:HGNC:18178]
ENSG00000074590	822.804675	744.250132	581.575311	515.243822	0.51428723	0.001412	0.0074634	NUAK1	NUAK family kinase 1 [Source:HGNC Symbol;Acc:HGNC:14311]
ENSG00000169962	22.6369707	10.6321447	57.9468154	61.7440943	-1.855555412	0.0014138	0.0074704	TAS1R3	taste 1 receptor member 3 [Source:HGNC Symbol;Acc:HGNC:15661]
ENSG00000162545	601.356048	651.218865	424.59212	437.531427	0.539289577	0.0014174	0.0074869	CAMK2N1	calcium/calmodulin dependent protein kinase II inhibitor 1 [Source:HGNC Symbol;Acc:HGNC:24190]
ENSG00000132680	1301.13371	1292.6916	966.13145	976.195422	0.417278107	0.0014187	0.0074914	KHDC4	KH domain containing 4, pre-mRNA splicing factor [Source:HGNC Symbol;Acc:HGNC:29145]
ENSG00000153406	1117.08529	973.727256	1330.6696	1523.37584	-0.449388922	0.0014191	0.0074915	NMRAL1	NmrA like redox sensor 1 [Source:HGNC Symbol;Acc:HGNC:24987]
ENSG00000122786	2556.99348	2760.81358	3605.3455	3240.5004	-0.364254793	0.0014243	0.0075167	CALD1	caldesmon 1 [Source:HGNC Symbol;Acc:HGNC:1441]
ENSG00000162650	49.2108059	60.2488202	102.197111	151.166576	-1.208848783	0.0014252	0.0075188	ATXN7L2	ataxin 7 like 2 [Source:HGNC Symbol;Acc:HGNC:28713]
ENSG00000161270	35.4317803	29.238398	3.16073539	6.3873201	2.760250286	0.0014301	0.0075422	NPHS1	NPHS1, nephrin [Source:HGNC Symbol;Acc:HGNC:7908]
ENSG00000134419	2459.55608	2507.41413	2858.35837	3993.13962	-0.463988826	0.0014343	0.007562	RPS15A	ribosomal protein S15a [Source:HGNC Symbol;Acc:HGNC:10389]
ENSG00000234518	21.6527546	36.3264945	148.554563	46.8403474	-1.749135136	0.0014362	0.0075701	PTGES3P1	prostaglandin E synthase 3 pseudogene 1 [Source:HGNC Symbol;Acc:HGNC:43824]
ENSG00000125841	819.852026	787.664723	1078.86435	1101.81272	-0.440124437	0.001447	0.0076244	NRSN2	neurensin 2 [Source:HGNC Symbol;Acc:HGNC:16229]
ENSG00000002726	12.7948095	13.2901809	0	0	6.065177577	0.0014474	0.0076244	AOC1	amine oxidase, copper containing 1 [Source:HGNC Symbol;Acc:HGNC:80]
ENSG00000169239	348.412506	354.404825	230.733683	206.52335	0.684663303	0.0014493	0.007632	CA5B	carbonic anhydrase 5B [Source:HGNC Symbol;Acc:HGNC:1378]
ENSG00000113083	1291.29155	1240.41689	1732.08299	1605.34645	-0.398798322	0.0014567	0.0076686	LOX	lysyl oxidase [Source:HGNC Symbol;Acc:HGNC:6664]
ENSG00000091127	551.161026	555.529563	775.433748	778.188499	-0.489338468	0.0014603	0.0076851	PUS7	pseudouridylate synthase 7 [Source:HGNC Symbol;Acc:HGNC:26033]
ENSG00000198520	74.800425	57.590784	22.1251477	19.1619603	1.679654506	0.0014636	0.0076999	ARMH1	armadillo-like helical domain containing 1 [Source:HGNC Symbol;Acc:HGNC:34345]
ENSG00000101109	1501.9138	1622.28809	1252.70479	1054.97237	0.437178463	0.0014671	0.0077158	STK4	serine/threonine kinase 4 [Source:HGNC Symbol;Acc:HGNC:11408]
ENSG00000121454	96.4531796	102.777399	46.3574523	33.0011539	1.32791697	0.0014714	0.0077361	LHX4	LIM homeobox 4 [Source:HGNC Symbol;Acc:HGNC:21734]
ENSG00000108828	6460.3946	5723.63792	4812.74642	4824.55578	0.338160552	0.0014754	0.0077547	VAT1	vesicle amine transport 1 [Source:HGNC Symbol;Acc:HGNC:16919]
ENSG00000134982	1549.15617	1756.96192	1321.18739	1110.32914	0.443515013	0.0014793	0.0077726	APC	APC, WNT signaling pathway regulator [Source:HGNC Symbol;Acc:HGNC:583]
ENSG00000064490	799.183488	640.586721	978.774391	1057.10148	-0.500685785	0.0014827	0.007788	RFXANK	regulatory factor X associated ankyrin containing protein [Source:HGNC Symbol;Acc:HGNC:9987]
ENSG00000151718	1613.13022	1804.80657	1310.65161	1272.14125	0.404485876	0.0014875	0.0078111	WWC2	WW and C2 domain containing 2 [Source:HGNC Symbol;Acc:HGNC:24148]
ENSG00000159348	845.441646	722.09983	520.46776	571.665149	0.520838947	0.0014912	0.0078282	CYB5R1	cytochrome b5 reductase 1 [Source:HGNC Symbol;Acc:HGNC:13397]
ENSG00000143776	1969.41645	2160.98342	1647.79672	1495.69746	0.394098107	0.0014922	0.007831	CDC42BPA	CDC42 binding protein kinase alpha [Source:HGNC Symbol;Acc:HGNC:1737]
ENSG00000204618	147.632418	139.103894	71.6433354	66.0023077	1.05838318	0.0014942	0.0078387	RNF39	ring finger protein 39 [Source:HGNC Symbol;Acc:HGNC:18064]
ENSG00000168291	947.800122	1093.33888	1292.74077	1508.4721	-0.456138153	0.0014979	0.0078559	PDHB	pyruvate dehydrogenase E1 beta subunit [Source:HGNC Symbol;Acc:HGNC:8808]
ENSG00000107175	997.995144	956.893027	709.058305	717.508958	0.454385621	0.0015014	0.0078715	CREB3	cAMP responsive element binding protein 3 [Source:HGNC Symbol;Acc:HGNC:2347]
ENSG00000110717	1818.83139	1725.9515	2215.67551	2369.69576	-0.371481987	0.0015042	0.007884	NDUFS8	NADH:ubiquinone oxidoreductase core subunit S8 [Source:HGNC Symbol;Acc:HGNC:7715]
ENSG00000112183	66.926696	63.7928684	22.1251477	19.1619603	1.662298362	0.0015082	0.0079025	RBM24	RNA binding motif protein 24 [Source:HGNC Symbol;Acc:HGNC:21539]
ENSG00000160352	229.322356	241.881293	387.716874	357.689926	-0.661345936	0.0015109	0.0079144	ZNF714	zinc finger protein 714 [Source:HGNC Symbol;Acc:HGNC:27124]

ENSG00000100577	323.807103	314.534282	445.66369	526.953908	-0.607644846	0.0015152	0.0079342	GSTZ1	glutathione S-transferase zeta 1 [Source:HGNC Symbol;Acc:HGNC:4643]
ENSG00000167646	193.890575	136.445858	71.6433354	87.2933747	1.054138797	0.0015159	0.0079353	DNAAF3	dynein axonemal assembly factor 3 [Source:HGNC Symbol;Acc:HGNC:30492]
ENSG00000213523	632.850964	543.125394	793.344582	878.256514	-0.508068294	0.0015192	0.0079501	SRA1	steroid receptor RNA activator 1 [Source:HGNC Symbol;Acc:HGNC:11281]
ENSG00000115561	414.354986	526.291165	713.272619	673.862271	-0.559227339	0.0015257	0.0079816	CHMP3	charged multivesicular body protein 3 [Source:HGNC Symbol;Acc:HGNC:29865]
ENSG00000205269	148.616634	174.544376	95.8756401	51.0985608	1.136804879	0.0015265	0.0079837	TMEM170B	transmembrane protein 170B [Source:HGNC Symbol;Acc:HGNC:34244]
ENSG00000197217	1135.7854	1122.57728	840.755613	833.545273	0.431671616	0.0015276	0.0079867	ENTPD4	ectonucleoside triphosphate diphosphohydrolase 4 [Source:HGNC Symbol;Acc:HGNC:14573]
ENSG00000152102	2892.61117	2815.74633	3596.91687	3596.06122	-0.333576206	0.0015308	0.0080011	FAM168B	family with sequence similarity 168 member B [Source:HGNC Symbol;Acc:HGNC:27016]
ENSG00000109320	1484.19791	1450.40175	1949.12016	1865.09747	-0.378317663	0.001532	0.0080027	NFKB1	nuclear factor kappa B subunit 1 [Source:HGNC Symbol;Acc:HGNC:7794]
ENSG00000129128	761.783275	892.214146	604.754037	557.825956	0.509143536	0.0015321	0.0080027	SPCS3	signal peptidase complex subunit 3 [Source:HGNC Symbol;Acc:HGNC:26212]
ENSG00000235174	578.719078	551.985514	754.362179	840.997147	-0.496860547	0.0015336	0.0080083	RPL39P3	ribosomal protein L39 pseudogene 3 [Source:HGNC Symbol;Acc:HGNC:21383]
ENSG00000125746	1041.30065	762.856385	595.271831	631.280137	0.555902291	0.0015388	0.0080332	EML2	echinoderm microtubule associated protein like 2 [Source:HGNC Symbol;Acc:HGNC:18035]
ENSG00000203883	188.969495	191.378605	301.32344	321.495112	-0.711362353	0.0015469	0.0080727	SOX18	SRY-box 18 [Source:HGNC Symbol;Acc:HGNC:11194]
ENSG00000175137	965.516012	876.265929	1279.04425	1207.2035	-0.433298881	0.0015513	0.0080934	SH3BP5L	SH3 binding domain protein 5 like [Source:HGNC Symbol;Acc:HGNC:29360]
ENSG00000159720	3355.19275	2909.66361	2348.42639	2483.60297	0.374424178	0.0015623	0.008148	ATP6V0D1	ATPase H+ transporting V0 subunit d1 [Source:HGNC Symbol;Acc:HGNC:13724]
ENSG00000106443	647.614206	679.571251	880.791594	955.968909	-0.468519364	0.0015759	0.0082163	PHF14	PHD finger protein 14 [Source:HGNC Symbol;Acc:HGNC:22203]
ENSG00000285523	21.6527546	33.6684583	4.21431385	2.1291067	3.12490813	0.0015809	0.00824	AC005906.3	novel transcript
ENSG00000006432	963.54758	946.260882	732.237031	647.248437	0.46916747	0.0015817	0.0082417	MAP3K9	mitogen-activated protein kinase kinase kinase 9 [Source:HGNC Symbol;Acc:HGNC:6861]
ENSG00000123444	98.4216118	93.0312665	167.518976	198.006923	-0.93336822	0.0015908	0.0082864	KBTBD4	kelch repeat and BTB domain containing 4 [Source:HGNC Symbol;Acc:HGNC:23761]
ENSG00000140406	641.708909	622.866479	870.25581	877.191961	-0.466749859	0.0015961	0.0083118	TLNRD1	talin rod domain containing 1 [Source:HGNC Symbol;Acc:HGNC:13519]
ENSG00000135597	896.620884	1033.97608	653.218647	727.089938	0.484581519	0.0015985	0.0083215	REPS1	RALBP1 associated Eps domain containing 1 [Source:HGNC Symbol;Acc:HGNC:15578]
ENSG00000114796	446.834118	473.130441	336.091529	243.782717	0.665856078	0.0016046	0.0083509	KLHL24	kelch like family member 24 [Source:HGNC Symbol;Acc:HGNC:25947]
ENSG00000026652	479.31325	467.814369	299.216283	327.882432	0.594820115	0.0016068	0.0083596	AGPAT4	1-acylglycerol-3-phosphate O-acyltransferase 4 [Source:HGNC Symbol;Acc:HGNC:20885]
ENSG00000130193	1416.28699	1277.62939	1036.72121	966.614442	0.42695591	0.0016081	0.0083635	THEM6	thioesterase superfamily member 6 [Source:HGNC Symbol;Acc:HGNC:29656]
ENSG00000115866	1035.39536	1225.35468	1569.83191	1480.79371	-0.431740381	0.0016099	0.0083703	DARS	aspartyl-tRNA synthetase [Source:HGNC Symbol;Acc:HGNC:2678]
ENSG00000076513	1145.62756	1181.05408	837.594878	888.902047	0.430561724	0.0016113	0.0083752	ANKRD13A	ankyrin repeat domain 13A [Source:HGNC Symbol;Acc:HGNC:21268]
ENSG00000106268	595.450752	429.71585	709.058305	835.67438	-0.59274947	0.0016123	0.0083777	NUDT1	nudix hydrolase 1 [Source:HGNC Symbol;Acc:HGNC:8048]
ENSG00000149600	299.2017	330.482499	483.592514	467.338921	-0.594089117	0.0016156	0.0083923	COMMD7	COMM domain containing 7 [Source:HGNC Symbol;Acc:HGNC:16223]
ENSG00000066230	148.616634	115.181568	63.2147077	57.4858809	1.126696703	0.0016352	0.0084918	SLC9A3	solute carrier family 9 member A3 [Source:HGNC Symbol;Acc:HGNC:11073]
ENSG00000120913	177.158901	155.938123	252.858831	317.236898	-0.776050864	0.0016388	0.0085068	PDLIM2	PDZ and LIM domain 2 [Source:HGNC Symbol;Acc:HGNC:13992]
ENSG00000178075	64.9582638	83.2851338	24.2323046	26.6138338	1.545035438	0.0016391	0.0085068	GRAMD1C	GRAM domain containing 1C [Source:HGNC Symbol;Acc:HGNC:25252]
ENSG00000131475	1172.2014	1145.6136	1529.79593	1515.92397	-0.394113549	0.0016422	0.0085195	VPS25	vacuolar protein sorting 25 homolog [Source:HGNC Symbol;Acc:HGNC:28122]
ENSG00000008988	8164.0727	8211.55979	9448.49165	11568.5013	-0.359989616	0.0016426	0.0085195	RPS20	ribosomal protein S20 [Source:HGNC Symbol;Acc:HGNC:10405]
ENSG00000173653	536.397784	486.420622	670.075902	811.189653	-0.534687254	0.0016485	0.008545	RCE1	Ras converting CAAX endopeptidase 1 [Source:HGNC Symbol;Acc:HGNC:13721]
ENSG00000143420	3741.98968	3741.62894	4434.51175	5099.21055	-0.349284175	0.0016485	0.008545	ENSA	endosulfine alpha [Source:HGNC Symbol;Acc:HGNC:3360]
ENSG00000111653	289.359539	273.777727	413.002757	450.306067	-0.616714954	0.0016507	0.0085538	ING4	inhibitor of growth family member 4 [Source:HGNC Symbol;Acc:HGNC:19423]
ENSG00000163820	1418.25543	1431.79549	1117.84675	1028.35854	0.40919209	0.0016515	0.0085553	FYCO1	FYVE and coiled-coil domain containing 1 [Source:HGNC Symbol;Acc:HGNC:14673]
ENSG00000171840	55.1161026	59.3628081	113.786474	137.327382	-1.132465562	0.0016529	0.0085598	NINJ2	ninjurin 2 [Source:HGNC Symbol;Acc:HGNC:7825]
ENSG00000101017	154.521931	148.850026	75.8576493	74.5187345	1.012301061	0.0016545	0.0085655	CD40	CD40 molecule [Source:HGNC Symbol;Acc:HGNC:11919]
ENSG00000154309	259.833055	240.995281	143.286671	146.908362	0.786938088	0.0016556	0.0085684	DISP1	dispatched RND transporter family member 1 [Source:HGNC Symbol;Acc:HGNC:19711]
ENSG00000072954	274.596297	264.031594	160.143926	158.618449	0.756602451	0.0016616	0.0085962	TMEM38A	transmembrane protein 38A [Source:HGNC Symbol;Acc:HGNC:28462]
ENSG00000182934	3501.84095	3384.56608	4363.92199	4259.27795	-0.324544605	0.001662	0.0085962	SRPRA	SRP receptor subunit alpha [Source:HGNC Symbol;Acc:HGNC:11307]
ENSG00000025770	1107.24313	951.576954	1358.06264	1417.98506	-0.431770916	0.0016669	0.0086193	NCAPH2	non-SMC condensin II complex subunit H2 [Source:HGNC Symbol;Acc:HGNC:25071]
ENSG00000112699	569.861132	532.493249	388.770453	358.754479	0.560043727	0.0016691	0.0086281	GMDS	GDP-mannose 4,6-dehydratase [Source:HGNC Symbol;Acc:HGNC:4369]
ENSG00000197565	999.963576	994.105533	745.933551	717.508958	0.44630596	0.0016701	0.0086304	COL4A6	collagen type IV alpha 6 chain [Source:HGNC Symbol;Acc:HGNC:2208]
ENSG00000115317	849.37851	730.073939	536.271437	573.794256	0.508261385	0.0016782	0.0086691	HTRA2	HtrA serine peptidase 2 [Source:HGNC Symbol;Acc:HGNC:14348]
ENSG00000090097	876.936561	700.835541	514.14629	571.665149	0.538437778	0.0016786	0.0086691	PCBP4	poly(rC) binding protein 4 [Source:HGNC Symbol;Acc:HGNC:8652]
ENSG00000027075	129.916528	140.875918	61.1075508	67.0668611	1.079579422	0.0016839	0.0086939	PRKCH	protein kinase C eta [Source:HGNC Symbol;Acc:HGNC:9403]
ENSG00000198952	2275.50767	1864.16938	2663.44635	2798.71076	-0.400377256	0.0016884	0.0087145	SMG5	SMG5, nonsense mediated mRNA decay factor [Source:HGNC Symbol;Acc:HGNC:24644]
ENSG00000242802	431.08666	426.171802	579.468154	668.539504	-0.541825506	0.001689	0.0087146	AP5Z1	adaptor related protein complex 5 subunit zeta 1 [Source:HGNC Symbol;Acc:HGNC:22197]
ENSG00000100263	701.746092	571.47778	868.148653	936.806948	-0.504358515	0.0016899	0.0087146	RHBDD3	rhomboid domain containing 3 [Source:HGNC Symbol;Acc:HGNC:1308]
ENSG00000088992	98.4216118	84.1711459	43.196717	24.4847271	1.430530788	0.00169	0.0087146	TESC	tescalcin [Source:HGNC Symbol;Acc:HGNC:26065]
ENSG00000106355	210.622249	322.50839	582.62889	358.754479	-0.818746436	0.0016917	0.0087208	LSM5	LSM5 homolog, U6 small nuclear RNA and mRNA degradation associated [Source:HGNC Symbol;Acc:HGNC:17162]
ENSG00000185024	847.410078	669.825119	1069.38214	1059.23058	-0.489399141	0.0016957	0.0087389	BRF1	BRF1, RNA polymerase III transcription initiation factor subunit [Source:HGNC Symbol;Acc:HGNC:11551]

ENSG00000096968	108.263773	158.596159	67.4290216	51.0985608	1.172227558	0.001703	0.0087733	JAK2	Janus kinase 2 [Source:HGNC Symbol;Acc:HGNC:6192]
ENSG00000125995	2095.39612	1465.46395	2404.26605	2578.34821	-0.485277674	0.0017039	0.0087757	ROMO1	reactive oxygen species modulator 1 [Source:HGNC Symbol;Acc:HGNC:16185]
ENSG00000088038	1178.10669	1077.39067	1481.33132	1503.14933	-0.404378357	0.0017061	0.0087815	CNOT3	CCR4-NOT transcription complex subunit 3 [Source:HGNC Symbol;Acc:HGNC:7879]
ENSG00000163661	90.5478829	102.777399	41.08956	37.2593673	1.303560099	0.0017061	0.0087815	PTX3	pentraxin 3 [Source:HGNC Symbol;Acc:HGNC:9692]
ENSG00000133687	1112.16421	1232.44278	933.470518	741.993685	0.484968342	0.0017112	0.0088049	TMTC1	transmembrane and tetratricopeptide repeat containing 1 [Source:HGNC Symbol;Acc:HGNC:24099]
ENSG00000075142	1393.65002	1491.1583	1804.77991	1972.61736	-0.388668166	0.0017119	0.008806	SRI	sorcin [Source:HGNC Symbol;Acc:HGNC:11292]
ENSG00000204463	5034.26544	4281.21028	5803.11017	6100.95525	-0.353948184	0.0017126	0.0088069	BAG6	BCL2 associated athanogene 6 [Source:HGNC Symbol;Acc:HGNC:13919]
ENSG00000165757	109.247989	88.6012062	38.9824031	41.5175807	1.296393562	0.0017158	0.0088207	JCAD	junctional cadherin 5 associated [Source:HGNC Symbol;Acc:HGNC:29283]
ENSG00000259527	12.7948095	12.4041689	0	0	6.015081985	0.0017179	0.0088287	LINC00052	long intergenic non-protein coding RNA 52 [Source:HGNC Symbol;Acc:HGNC:26455]
ENSG00000048052	62.9898316	69.1089408	24.2323046	18.097407	1.641947498	0.0017214	0.0088439	HDAC9	histone deacetylase 9 [Source:HGNC Symbol;Acc:HGNC:14065]
ENSG00000197562	951.736986	744.250132	1205.29376	1162.49226	-0.482314708	0.0017282	0.0088763	RAB40C	RAB40C, member RAS oncogene family [Source:HGNC Symbol;Acc:HGNC:18285]
ENSG00000110318	128.932311	162.140207	73.7504924	66.0023077	1.059490845	0.0017377	0.0089225	CEP126	centrosomal protein 126 [Source:HGNC Symbol;Acc:HGNC:29264]
ENSG00000102531	1204.68053	1360.02851	1023.02469	823.964293	0.473847329	0.0017387	0.008925	FNDC3A	fibronectin type III domain containing 3A [Source:HGNC Symbol;Acc:HGNC:20296]
ENSG00000179532	365.14418	247.197365	177.001182	173.522196	0.803330989	0.0017424	0.008941	DNHD1	dynein heavy chain domain 1 [Source:HGNC Symbol;Acc:HGNC:26532]
ENSG00000170558	416.323418	442.120019	288.680499	272.525658	0.613430822	0.001743	0.0089413	CDH2	cadherin 2 [Source:HGNC Symbol;Acc:HGNC:1759]
ENSG00000257103	1426.12916	1464.57794	1942.79868	1816.12802	-0.378826605	0.0017435	0.0089413	LSM14A	LSM14A, mRNA processing body assembly factor [Source:HGNC Symbol;Acc:HGNC:24489]
ENSG00000163995	226.369707	149.736038	106.411425	78.7769479	1.020302376	0.001751	0.0089758	ABLIM2	actin binding LIM protein family member 2 [Source:HGNC Symbol;Acc:HGNC:19195]
ENSG00000118402	156.490363	186.062533	104.304268	56.4213276	1.091909156	0.0017515	0.0089758	ELOVL4	ELOVL fatty acid elongase 4 [Source:HGNC Symbol;Acc:HGNC:14415]
ENSG00000173848	1045.23752	1086.25079	1407.58083	1407.33953	-0.401072342	0.0017518	0.0089758	NET1	neuroepithelial cell transforming 1 [Source:HGNC Symbol;Acc:HGNC:14592]
ENSG00000103197	1718.44134	1481.41217	2130.33565	2068.42716	-0.392397622	0.0017593	0.0090109	TSC2	TSC complex subunit 2 [Source:HGNC Symbol;Acc:HGNC:12363]
ENSG00000165480	229.322356	317.192318	467.788837	409.85304	-0.681641452	0.0017597	0.0090109	SKA3	spindle and kinetochore associated complex subunit 3 [Source:HGNC Symbol;Acc:HGNC:20262]
ENSG00000154917	573.797997	508.570923	358.216677	372.593673	0.566128479	0.0017605	0.0090121	RAB6B	RAB6B, member RAS oncogene family [Source:HGNC Symbol;Acc:HGNC:14902]
ENSG00000141013	283.454242	240.109269	384.556139	435.40232	-0.648187396	0.0017705	0.0090603	GAS8	growth arrest specific 8 [Source:HGNC Symbol;Acc:HGNC:4166]
ENSG00000197989	272.627865	234.793196	345.573736	481.178114	-0.704973062	0.0017719	0.0090651	SNHG12	small nucleolar RNA host gene 12 [Source:HGNC Symbol;Acc:HGNC:30062]
ENSG00000179083	30.5106997	37.2125066	5.26789231	6.3873201	2.539864407	0.0017731	0.0090682	FAM133A	family with sequence similarity 133 member A [Source:HGNC Symbol;Acc:HGNC:26748]
ENSG00000174177	507.855517	435.917934	653.218647	708.992531	-0.530218725	0.0017743	0.0090716	CTU2	cytosolic thiouridylase subunit 2 [Source:HGNC Symbol;Acc:HGNC:28005]
ENSG00000166106	455.692063	443.892043	309.752068	283.171191	0.601226748	0.0017787	0.0090916	ADAMTS15	ADAM metallopeptidase with thrombospondin type 1 motif 15 [Source:HGNC Symbol;Acc:HGNC:16305]
ENSG00000164967	281.48581	275.549751	428.806434	420.498573	-0.608668944	0.0017805	0.009098	RPP25L	ribonuclease P/MRP subunit p25 like [Source:HGNC Symbol;Acc:HGNC:19909]
ENSG00000100908	434.039308	384.529235	590.003939	601.472643	-0.542277939	0.0017814	0.0091	EMC9	ER membrane protein complex subunit 9 [Source:HGNC Symbol;Acc:HGNC:20273]
ENSG00000273841	1119.05373	1228.89873	1529.79593	1570.21619	-0.400507408	0.0017833	0.0091068	TAF9	TATA-box binding protein associated factor 9 [Source:HGNC Symbol;Acc:HGNC:11542]
ENSG00000184613	31.4949158	41.6425669	9.48220616	3.19366005	2.52801861	0.001784	0.0091075	NELL2	neural EGFL like 2 [Source:HGNC Symbol;Acc:HGNC:7751]
ENSG00000103245	572.813781	452.752164	693.254628	811.189653	-0.553812673	0.001785	0.0091099	CIAO3	cytosolic iron-sulfur assembly component 3 [Source:HGNC Symbol;Acc:HGNC:14179]
ENSG00000143333	49.2108059	27.4663739	4.21431385	9.58098015	2.473622123	0.0017923	0.0091446	RGS16	regulator of G protein signaling 16 [Source:HGNC Symbol;Acc:HGNC:9997]
ENSG00000111906	572.813781	515.65902	745.933551	787.769479	-0.4952483	0.001793	0.0091454	HDDC2	HD domain containing 2 [Source:HGNC Symbol;Acc:HGNC:21078]
ENSG00000109265	669.26696	663.623034	472.003151	465.209814	0.508062862	0.0017988	0.0091719	KIAA1211	KIAA1211 [Source:HGNC Symbol;Acc:HGNC:29219]
ENSG00000079459	6559.80043	6369.54071	4991.85475	5387.70451	0.316882422	0.0017994	0.0091725	FDFT1	farnesyl-diphosphate farnesyltransferase 1 [Source:HGNC Symbol;Acc:HGNC:3629]
ENSG00000166473	56.1003187	38.9845307	14.7500985	7.45187345	2.096103465	0.0018006	0.0091732	PKD1L2	polycystin 1 like 2 (gene/pseudogene) [Source:HGNC Symbol;Acc:HGNC:21715]
ENSG00000158552	149.60085	123.155677	226.519369	253.363697	-0.816490078	0.0018006	0.0091732	ZFAND2B	zinc finger AN1-type containing 2B [Source:HGNC Symbol;Acc:HGNC:25206]
ENSG00000125457	648.598422	547.555454	421.431385	389.626526	0.559808063	0.0018075	0.0092054	MIF4GD	MIF4G domain containing [Source:HGNC Symbol;Acc:HGNC:24030]
ENSG00000224078	416.323418	398.705428	279.198293	246.976377	0.63101946	0.0018142	0.0092367	SNHG14	small nucleolar RNA host gene 14 [Source:HGNC Symbol;Acc:HGNC:37462]
ENSG00000204856	205.701169	217.072955	309.752068	374.722779	-0.694648021	0.0018151	0.0092383	FAM216A	family with sequence similarity 216 member A [Source:HGNC Symbol;Acc:HGNC:30180]
ENSG00000015133	429.118228	337.570596	598.432567	551.438635	-0.586072585	0.0018172	0.0092459	CCDC88C	coiled-coil domain containing 88C [Source:HGNC Symbol;Acc:HGNC:19967]
ENSG00000136367	140.742905	101.891387	55.8396585	51.0985608	1.180426441	0.0018177	0.0092459	ZFHX2	zinc finger homeobox 2 [Source:HGNC Symbol;Acc:HGNC:20152]
ENSG00000174032	247.038246	310.990234	166.465397	160.747556	0.771125955	0.0018197	0.0092536	SLC25A30	solute carrier family 25 member 30 [Source:HGNC Symbol;Acc:HGNC:27371]
ENSG00000113194	2062.91698	2082.12835	2570.73145	2708.22372	-0.348825337	0.0018248	0.0092764	FAF2	Fas associated factor family member 2 [Source:HGNC Symbol;Acc:HGNC:24666]
ENSG00000183955	1450.73456	1402.55709	1779.49402	1939.6162	-0.382420113	0.0018279	0.0092895	KMT5A	lysine methyltransferase 5A [Source:HGNC Symbol;Acc:HGNC:29489]
ENSG00000164258	510.808165	555.529563	750.147865	749.445559	-0.491447835	0.0018291	0.009293	NDUFS4	NADH:ubiquinone oxidoreductase subunit S4 [Source:HGNC Symbol;Acc:HGNC:7711]
ENSG00000113269	1368.0604	1409.64519	1070.43572	1031.5522	0.40221962	0.00183	0.0092944	RNF130	ring finger protein 130 [Source:HGNC Symbol;Acc:HGNC:18280]
ENSG00000226237	39.3686447	45.1866152	9.48220616	9.58098015	2.149744251	0.0018358	0.0093215	GAS1RR	GAS1 adjacent regulatory RNA [Source:HGNC Symbol;Acc:HGNC:52261]
ENSG00000183578	36.4159964	31.0104222	5.26789231	6.3873201	2.531965268	0.001837	0.0093244	TNFAIP8L3	TNF alpha induced protein 8 like 3 [Source:HGNC Symbol;Acc:HGNC:20620]
ENSG00000213553	1417.27121	1262.56719	1796.35128	1725.64098	-0.394651093	0.001838	0.0093271	RPLP0P6	ribosomal protein lateral stalk subunit P0 pseudogene 6 [Source:HGNC Symbol;Acc:HGNC:36404]
ENSG00000173575	2327.67112	2179.58967	2956.34117	2790.19433	-0.350623639	0.0018455	0.0093619	CHD2	chromodomain helicase DNA binding protein 2 [Source:HGNC Symbol;Acc:HGNC:1917]

ENSG00000181773	154.521931	98.3473389	45.3038739	62.8086477	1.224323643	0.0018471	0.0093668	GPR3	G protein-coupled receptor 3 [Source:HGNC Symbol;Acc:HGNC:4484]
ENSG00000170191	333.649264	407.565548	243.376625	219.29799	0.680687498	0.0018484	0.0093668	NANP	N-acetylneuraminic acid phosphatase [Source:HGNC Symbol;Acc:HGNC:16140]
ENSG00000143157	1754.85734	1674.5628	2130.33565	2293.04792	-0.367309932	0.0018485	0.0093668	POGK	pogo transposable element derived with KRAB domain [Source:HGNC Symbol;Acc:HGNC:18800]
ENSG00000188910	580.68751	494.39473	775.433748	750.510112	-0.506091059	0.0018486	0.0093668	GJB3	gap junction protein beta 3 [Source:HGNC Symbol;Acc:HGNC:4285]
ENSG00000143067	257.864623	328.710475	193.858437	142.650149	0.802418085	0.0018506	0.0093719	ZNF697	zinc finger protein 697 [Source:HGNC Symbol;Acc:HGNC:32034]
ENSG00000129484	825.757323	808.929012	1018.81037	1235.94644	-0.463976934	0.0018508	0.0093719	PARP2	poly(ADP-ribose) polymerase 2 [Source:HGNC Symbol;Acc:HGNC:272]
ENSG00000136295	5858.05434	5134.4399	7017.88614	6823.78698	-0.332656979	0.0018524	0.0093739	TTYH3	tweety family member 3 [Source:HGNC Symbol;Acc:HGNC:22222]
ENSG00000100225	1783.39961	1804.80657	2189.33604	2452.73092	-0.371441922	0.001853	0.0093739	FBXO7	F-box protein 7 [Source:HGNC Symbol;Acc:HGNC:13586]
ENSG00000198133	40.3528608	32.7824463	8.4286277	5.32276675	2.409351961	0.0018533	0.0093739	TMEM229B	transmembrane protein 229B [Source:HGNC Symbol;Acc:HGNC:20130]
ENSG00000006652	1033.42692	1261.68118	1581.42127	1521.24674	-0.434306628	0.0018534	0.0093739	IFRD1	interferon related developmental regulator 1 [Source:HGNC Symbol;Acc:HGNC:5456]
ENSG00000179886	618.087722	556.415575	801.77321	837.803487	-0.481797403	0.0018559	0.0093837	TIGD5	tigger transposable element derived 5 [Source:HGNC Symbol;Acc:HGNC:18336]
ENSG00000126351	1700.72545	1554.06516	2114.53197	2095.04099	-0.371396981	0.0018591	0.0093974	THRA	thyroid hormone receptor alpha [Source:HGNC Symbol;Acc:HGNC:11796]
ENSG00000169727	2516.64061	2166.29949	2918.41234	3156.40068	-0.375732889	0.0018608	0.0094009	GPS1	G protein pathway suppressor 1 [Source:HGNC Symbol;Acc:HGNC:4549]
ENSG00000213463	757.846411	896.644207	639.522127	488.629988	0.552790004	0.0018609	0.0094009	SYNJ2BP	synaptojanin 2 binding protein [Source:HGNC Symbol;Acc:HGNC:18955]
ENSG00000159055	339.554561	357.948873	536.271437	496.081861	-0.565372189	0.0018649	0.0094157	MIS18A	MIS18 kinetochore protein A [Source:HGNC Symbol;Acc:HGNC:1286]
ENSG00000117322	23.6211868	19.4922654	1.05357846	0	5.347147366	0.001865	0.0094157	CR2	complement C3d receptor 2 [Source:HGNC Symbol;Acc:HGNC:2336]
ENSG00000120549	591.513887	506.798899	380.341825	359.819032	0.56869945	0.0018697	0.0094357	KIAA1217	KIAA1217 [Source:HGNC Symbol;Acc:HGNC:25428]
ENSG00000221818	9.84216118	15.0622051	0	0	5.999665618	0.00187	0.0094357	EBF2	early B cell factor 2 [Source:HGNC Symbol;Acc:HGNC:19090]
ENSG00000170190	461.597359	434.14591	613.182665	673.862271	-0.52321283	0.0018714	0.0094396	SLC16A5	solute carrier family 16 member 5 [Source:HGNC Symbol;Acc:HGNC:10926]
ENSG00000087116	47.2423737	32.7824463	11.5893631	3.19366005	2.433423667	0.0018793	0.0094768	ADAMTS2	ADAM metallopeptidase with thrombospondin type 1 motif 2 [Source:HGNC Symbol;Acc:HGNC:218]
ENSG00000205363	269.675216	242.767305	154.876034	144.779256	0.7735116	0.0018822	0.0094861	INSYN1	inhibitory synaptic factor 1 [Source:HGNC Symbol;Acc:HGNC:33753]
ENSG00000197162	72.8319927	64.6788805	125.375837	160.747556	-1.058002313	0.0018823	0.0094861	ZNF785	zinc finger protein 785 [Source:HGNC Symbol;Acc:HGNC:26496]
ENSG00000242759	32.4791319	23.0363136	5.26789231	1.06455335	3.128159823	0.0018853	0.0094984	LINC00882	long intergenic non-protein coding RNA 882 [Source:HGNC Symbol;Acc:HGNC:48568]
ENSG00000212978	135.821824	168.342292	290.787656	238.45995	-0.797671085	0.0018905	0.009522	AC016747.1	uncharacterized LOC339803 [Source:NCBI gene;Acc:339803]
ENSG00000103194	2715.45227	2453.3674	3367.23677	3217.08022	-0.349436003	0.0018915	0.0095222	USP10	ubiquitin specific peptidase 10 [Source:HGNC Symbol;Acc:HGNC:12608]
ENSG00000171450	113.184854	145.305978	40.0359816	71.3250745	1.216374265	0.0018917	0.0095222	CDK5R2	cyclin dependent kinase 5 regulatory subunit 2 [Source:HGNC Symbol;Acc:HGNC:1776]
ENSG00000140396	356.286235	377.441138	558.396585	519.502035	-0.554584822	0.0018988	0.0095549	NCOA2	nuclear receptor coactivator 2 [Source:HGNC Symbol;Acc:HGNC:7669]
ENSG00000131351	442.897253	381.871199	572.093105	635.53835	-0.550899405	0.0019104	0.0096106	HAUS8	HAUS augmin like complex subunit 8 [Source:HGNC Symbol;Acc:HGNC:30532]
ENSG00000010256	7306.82046	6461.68597	8384.3774	8892.21413	-0.327559474	0.0019112	0.0096117	UQCRC1	ubiquinol-cytochrome c reductase core protein 1 [Source:HGNC Symbol;Acc:HGNC:12585]
ENSG00000011485	1653.48308	1551.40712	1970.19172	2207.88365	-0.382725727	0.0019127	0.0096132	PPP5C	protein phosphatase 5 catalytic subunit [Source:HGNC Symbol;Acc:HGNC:9322]
ENSG00000143641	9266.39475	8566.85063	7332.9061	7059.05327	0.309236194	0.0019127	0.0096132	GALNT2	polypeptide N-acetylgalactosaminyltransferase 2 [Source:HGNC Symbol;Acc:HGNC:4124]
ENSG00000183605	227.353923	259.601534	359.270256	407.723933	-0.654484714	0.0019132	0.0096132	SFXN4	sideroflexin 4 [Source:HGNC Symbol;Acc:HGNC:16088]
ENSG00000172071	345.459857	376.555126	229.680105	228.87897	0.655331954	0.001918	0.0096344	EIF2AK3	eukaryotic translation initiation factor 2 alpha kinase 3 [Source:HGNC Symbol;Acc:HGNC:3255]
ENSG00000232931	137.790257	167.45628	73.7504924	77.7123946	1.012013927	0.0019263	0.0096731	LINC00342	long intergenic non-protein coding RNA 342 [Source:HGNC Symbol;Acc:HGNC:42470]
ENSG00000171634	1810.95766	1881.88962	2520.15968	2251.53034	-0.369686861	0.0019285	0.0096814	BPTF	bromodomain PHD finger transcription factor [Source:HGNC Symbol;Acc:HGNC:3581]
ENSG00000082684	32.4791319	45.1866152	8.4286277	7.45187345	2.291260871	0.0019315	0.0096936	SEMA5B	semaphorin 5B [Source:HGNC Symbol;Acc:HGNC:10737]
ENSG00000172508	47.2423737	31.0104222	8.4286277	7.45187345	2.298896157	0.0019378	0.0097221	CARNS1	carnosine synthase 1 [Source:HGNC Symbol;Acc:HGNC:29268]
ENSG00000077238	711.588253	567.933732	413.002757	452.435174	0.56334446	0.0019388	0.0097245	IL4R	interleukin 4 receptor [Source:HGNC Symbol;Acc:HGNC:6015]
ENSG00000128191	652.535286	649.446841	828.112671	993.228276	-0.484245245	0.0019412	0.0097337	DGCR8	DGCR8, microprocessor complex subunit [Source:HGNC Symbol;Acc:HGNC:2847]
ENSG00000143578	151.569282	145.305978	237.055154	270.396551	-0.77366747	0.0019493	0.0097712	CREB3L4	cAMP responsive element binding protein 3 like 4 [Source:HGNC Symbol;Acc:HGNC:18854]
ENSG00000104267	50.195022	48.7306634	9.48220616	15.9683003	1.959223423	0.0019568	0.0098059	CA2	carbonic anhydrase 2 [Source:HGNC Symbol;Acc:HGNC:1373]
ENSG00000163328	93.5005312	82.3991217	38.9824031	28.7429405	1.37604664	0.0019574	0.0098059	GPR155	G protein-coupled receptor 155 [Source:HGNC Symbol;Acc:HGNC:22951]
ENSG00000007314	43.3055092	36.3264945	9.48220616	7.45187345	2.232259426	0.0019611	0.009822	SCN4A	sodium voltage-gated channel alpha subunit 4 [Source:HGNC Symbol;Acc:HGNC:10591]
ENSG00000108239	317.901806	281.751836	191.75128	171.393089	0.722923596	0.0019623	0.0098251	TBC1D12	TBC1 domain family member 12 [Source:HGNC Symbol;Acc:HGNC:29082]
ENSG00000112679	426.165579	387.187271	236.001576	284.235745	0.644422342	0.001964	0.0098305	DUSP22	dual specificity phosphatase 22 [Source:HGNC Symbol;Acc:HGNC:16077]
ENSG00000101098	2184.95978	1920.87415	1580.36769	1567.02253	0.383210318	0.0019676	0.0098455	RIMS4	regulating synaptic membrane exocytosis 4 [Source:HGNC Symbol;Acc:HGNC:16183]
ENSG00000184271	110.232205	104.549423	189.644123	202.265137	-0.868055639	0.0019749	0.0098784	POU6F1	POU class 6 homeobox 1 [Source:HGNC Symbol;Acc:HGNC:9224]
ENSG00000103150	308.059645	301.244101	189.644123	185.232283	0.700615759	0.0019753	0.0098784	MLYCD	malonyl-CoA decarboxylase [Source:HGNC Symbol;Acc:HGNC:7150]
ENSG00000094631	751.941114	641.472733	938.73841	990.034616	-0.469760039	0.0019761	0.0098791	HDAC6	histone deacetylase 6 [Source:HGNC Symbol;Acc:HGNC:14064]
ENSG00000130713	1661.35681	1492.04431	1994.42403	2106.75108	-0.379446108	0.0019774	0.009883	EXOSC2	exosome component 2 [Source:HGNC Symbol;Acc:HGNC:17097]
ENSG00000126602	1739.10988	1580.64552	2079.76388	2223.85195	-0.374742354	0.0019812	0.0098992	TRAP1	TNF receptor associated protein 1 [Source:HGNC Symbol;Acc:HGNC:16264]
ENSG00000116874	176.174685	195.808666	351.895206	270.396551	-0.741771513	0.0019861	0.0099174	WARS2	tryptophanyl tRNA synthetase 2, mitochondrial [Source:HGNC Symbol;Acc:HGNC:12730]
ENSG00000020129	923.194719	836.395386	1188.43651	1174.20235	-0.425596944	0.0019861	0.0099174	NCDN	neurochondrin [Source:HGNC Symbol;Acc:HGNC:17597]

ENSG00000224660	139.758689	163.026219	70.589757	79.8415013	1.01006487	0.0019894	0.0099313	SH3BP5-AS1	SH3BP5 antisense RNA 1 [Source:HGNC Symbol;Acc:HGNC:44501]
ENSG00000122068	1537.34558	1760.50597	1313.81234	1148.65306	0.421705763	0.0019971	0.0099668	FYTTD1	forty-two-three domain containing 1 [Source:HGNC Symbol;Acc:HGNC:25407]
ENSG00000101400	1806.03658	1710.88929	1364.38411	1343.46633	0.377005446	0.0020011	0.0099813	SNTA1	syntrophin alpha 1 [Source:HGNC Symbol;Acc:HGNC:11167]
ENSG00000125971	1403.49218	1458.37585	1806.88706	1900.22773	-0.373189605	0.0020012	0.0099813	DYNLRB1	dynein light chain roadblock-type 1 [Source:HGNC Symbol;Acc:HGNC:15468]
ENSG00000146233	35.4317803	39.8705428	5.26789231	9.58098015	2.343771838	0.0020069	0.0100069	CYP39A1	cytochrome P450 family 39 subfamily A member 1 [Source:HGNC Symbol;Acc:HGNC:17449]
ENSG00000136270	2530.41964	2212.37212	3034.30597	3047.81624	-0.359144873	0.0020094	0.0100161	TBRG4	transforming growth factor beta regulator 4 [Source:HGNC Symbol;Acc:HGNC:17443]
ENSG00000147535	184.048414	174.544376	270.769665	322.559665	-0.72677827	0.0020148	0.0100401	PLPP5	phospholipid phosphatase 5 [Source:HGNC Symbol;Acc:HGNC:25026]
ENSG00000171766	109.247989	147.964014	48.4646093	67.0668611	1.156310559	0.0020311	0.0101181	GATM	glycine amidinotransferase [Source:HGNC Symbol;Acc:HGNC:4175]
ENSG00000153944	1675.13583	1690.51101	2131.38923	2174.88249	-0.355519803	0.0020418	0.0101687	MSI2	musashi RNA binding protein 2 [Source:HGNC Symbol;Acc:HGNC:18585]
ENSG00000186318	1053.11125	1042.8362	789.130268	766.478412	0.430071395	0.0020428	0.0101708	BACE1	beta-secretase 1 [Source:HGNC Symbol;Acc:HGNC:933]
ENSG00000143195	12.7948095	11.5181568	0	0	5.963184436	0.0020491	0.0101988	ILDR2	immunoglobulin like domain containing receptor 2 [Source:HGNC Symbol;Acc:HGNC:18131]
ENSG00000168275	216.527546	197.58069	316.073539	347.044392	-0.679860526	0.0020497	0.0101988	COA6	cytochrome c oxidase assembly factor 6 [Source:HGNC Symbol;Acc:HGNC:18025]
ENSG00000218336	2456.60343	2526.9064	2070.28168	1755.44847	0.38143641	0.0020519	0.0102071	TENM3	teneurin transmembrane protein 3 [Source:HGNC Symbol;Acc:HGNC:29944]
ENSG00000280649	91.532099	55.8187599	28.4466185	20.2265137	1.595615485	0.0020535	0.0102119	AC245100.8	TEC
ENSG00000176903	1372.98148	1271.42731	1822.69074	1648.99314	-0.392981725	0.0020545	0.0102137	PNMA1	PNMA family member 1 [Source:HGNC Symbol;Acc:HGNC:9158]
ENSG00000050030	7.87372895	16.8342292	0	0	5.989232062	0.0020604	0.0102403	NEXMIF	neurite extension and migration factor [Source:HGNC Symbol;Acc:HGNC:29433]
ENSG00000153823	467.502656	478.446513	317.127117	318.301452	0.574143455	0.0020611	0.0102408	PID1	phosphotyrosine interaction domain containing 1 [Source:HGNC Symbol;Acc:HGNC:26084]
ENSG00000204619	1253.89133	1162.44783	1557.18897	1607.47556	-0.389506294	0.002065	0.0102573	PPP1R11	protein phosphatase 1 regulatory inhibitor subunit 11 [Source:HGNC Symbol;Acc:HGNC:9285]
ENSG00000042493	2970.36424	2428.55906	3439.93368	3590.73845	-0.381341111	0.0020665	0.0102614	CAPG	capping actin protein, gelsolin like [Source:HGNC Symbol;Acc:HGNC:1474]
ENSG00000159251	882.841858	816.017109	597.378988	630.215583	0.468458161	0.0020676	0.0102637	ACTC1	actin, alpha, cardiac muscle 1 [Source:HGNC Symbol;Acc:HGNC:143]
ENSG00000240376	723.398847	738.048047	954.542087	1031.5522	-0.442408299	0.0020701	0.0102732	AC010343.1	ribosomal protein S8 (RPS8) pseudogene
ENSG00000134809	660.409015	612.234335	819.684044	955.968909	-0.4808181	0.0020754	0.0102966	TIMM10	translocase of inner mitochondrial membrane 10 [Source:HGNC Symbol;Acc:HGNC:11814]
ENSG00000267002	207.669601	205.554798	108.518582	121.359082	0.846106703	0.0020829	0.0103306	AC060780.1	novel transcript
ENSG00000101695	51.1792381	104.549423	32.6609323	14.9037469	1.71266379	0.0020937	0.0103802	RNF125	ring finger protein 125 [Source:HGNC Symbol;Acc:HGNC:21150]
ENSG00000170448	205.701169	182.518485	300.269862	328.946985	-0.697494432	0.0020941	0.0103802	NFXL1	nuclear transcription factor, X-box binding like 1 [Source:HGNC Symbol;Acc:HGNC:18726]
ENSG00000125741	593.482319	457.182224	744.879973	776.059392	-0.534835943	0.0020949	0.0103813	OPA3	OPA3, outer mitochondrial membrane lipid metabolism regulator [Source:HGNC Symbol;Acc:HGNC:8142]
ENSG00000079215	926.147367	986.131425	694.308207	710.057085	0.445623416	0.002096	0.0103836	SLC1A3	solute carrier family 1 member 3 [Source:HGNC Symbol;Acc:HGNC:10941]
ENSG00000185000	1198.77523	1053.46834	853.398554	804.802333	0.441296154	0.0020968	0.0103845	DGAT1	diacylglycerol O-acyltransferase 1 [Source:HGNC Symbol;Acc:HGNC:2843]
ENSG00000141429	1220.42799	1531.02884	1097.82876	832.48072	0.51172375	0.0020996	0.010395	GALNT1	polypeptide N-acetylgalactosaminyltransferase 1 [Source:HGNC Symbol;Acc:HGNC:4123]
ENSG00000125355	99.4058279	131.129785	0	6.3873201	5.175312257	0.0021054	0.01042	TMEM255A	transmembrane protein 255A [Source:HGNC Symbol;Acc:HGNC:26086]
ENSG00000011143	238.180301	202.01075	317.127117	394.949293	-0.6948524	0.0021058	0.01042	MKS1	Meckel syndrome, type 1 [Source:HGNC Symbol;Acc:HGNC:7121]
ENSG00000132854	357.270451	345.544704	526.789231	504.598288	-0.553602869	0.0021074	0.0104248	KANK4	KN motif and ankyrin repeat domains 4 [Source:HGNC Symbol;Acc:HGNC:27263]
ENSG00000070159	960.594931	1035.7481	698.522521	764.349305	0.44887001	0.0021096	0.0104323	PTPN3	protein tyrosine phosphatase, non-receptor type 3 [Source:HGNC Symbol;Acc:HGNC:9655]
ENSG00000172340	504.902869	569.705756	749.094287	761.155645	-0.490328052	0.0021121	0.0104419	SUCLG2	succinate-CoA ligase GDP-forming beta subunit [Source:HGNC Symbol;Acc:HGNC:11450]
ENSG00000140391	2654.43087	2549.94271	3302.96848	3239.43584	-0.330198928	0.0021192	0.0104741	TSPAN3	tetraspanin 3 [Source:HGNC Symbol;Acc:HGNC:17752]
ENSG00000159363	2247.94961	1928.84826	1562.45686	1625.57297	0.389403913	0.0021206	0.0104776	ATP13A2	ATPase cation transporting 13A2 [Source:HGNC Symbol;Acc:HGNC:30213]
ENSG00000122035	133.853392	148.850026	69.5361785	69.1959678	1.027519655	0.0021232	0.0104873	RASL11A	RAS like family 11 member A [Source:HGNC Symbol;Acc:HGNC:23802]
ENSG00000127191	1066.89027	905.504327	1295.90151	1361.56373	-0.430704867	0.0021241	0.0104887	TRAF2	TNF receptor associated factor 2 [Source:HGNC Symbol;Acc:HGNC:12032]
ENSG00000102409	17.7158901	23.9223257	2.10715692	0	4.300358489	0.0021263	0.0104966	BEX4	brain expressed X-linked 4 [Source:HGNC Symbol;Acc:HGNC:25475]
ENSG00000125730	13917.8001	12384.6766	10637.9817	10578.4666	0.309950641	0.0021336	0.0105295	C3	complement C3 [Source:HGNC Symbol;Acc:HGNC:1318]
ENSG00000119977	1247.00182	1288.26154	968.238607	946.387928	0.405172522	0.0021479	0.0105972	TCTN3	tectonic family member 3 [Source:HGNC Symbol;Acc:HGNC:24519]
ENSG00000156697	509.823949	550.21349	652.165068	925.096861	-0.57290186	0.0021493	0.0106009	UTP14A	UTP14A, small subunit processome component [Source:HGNC Symbol;Acc:HGNC:10665]
ENSG00000104064	258.848839	277.321775	434.074326	388.561973	-0.617161492	0.0021546	0.0106238	GABPB1	GA binding protein transcription factor subunit beta 1 [Source:HGNC Symbol;Acc:HGNC:4074]
ENSG00000225648	309.043861	307.446185	447.770846	472.661688	-0.578240809	0.0021695	0.0106943	SBDSP1	SBDS, ribosome maturation factor pseudogene 1 [Source:HGNC Symbol;Acc:HGNC:21646]
ENSG00000011332	222.432843	205.554798	133.804465	102.197122	0.858177958	0.0021724	0.0107056	DPF1	double PHD fingers 1 [Source:HGNC Symbol;Acc:HGNC:20225]
ENSG00000116752	484.23433	528.949201	807.041102	653.635757	-0.527395168	0.0021789	0.0107342	BCAS2	BCAS2, pre-mRNA processing factor [Source:HGNC Symbol;Acc:HGNC:975]
ENSG00000133858	1071.81135	1460.14788	948.220616	832.48072	0.508425294	0.0021796	0.0107345	ZFC3H1	zinc finger C3H1-type containing [Source:HGNC Symbol;Acc:HGNC:28328]
ENSG00000233237	77.7530733	56.704772	21.0715692	23.4201737	1.594213614	0.0021943	0.0108022	LINC00472	long intergenic non-protein coding RNA 472 [Source:HGNC Symbol;Acc:HGNC:21380]
ENSG00000134222	351.365154	354.404825	510.985554	521.631142	-0.548969608	0.0021951	0.0108022	PSRC1	proline and serine rich coiled-coil 1 [Source:HGNC Symbol;Acc:HGNC:24472]
ENSG00000218283	1057.04811	1311.29785	1521.3673	1693.70438	-0.440302427	0.0021952	0.0108022	MORF4L1P1	mortality factor 4 like 1 pseudogene 1 [Source:HGNC Symbol;Acc:HGNC:20400]
ENSG00000169213	21.6527546	21.2642895	2.10715692	1.06455335	3.756554562	0.0021981	0.0108134	RAB3B	RAB3B, member RAS oncogene family [Source:HGNC Symbol;Acc:HGNC:9778]

ENSG00000154803	363.175748	299.472077	455.145896	555.696849	-0.610219315	0.0021992	0.0108156	FLCN	folliculin [Source:HGNC Symbol;Acc:HGNC:27310]
ENSG00000204103	86.6110184	98.3473389	31.6073539	42.582134	1.318841457	0.0022048	0.0108398	MAFB	MAF bZIP transcription factor B [Source:HGNC Symbol;Acc:HGNC:6408]
ENSG00000156256	292.312187	362.378933	625.825607	422.62768	-0.678539921	0.0022097	0.0108576	USP16	ubiquitin specific peptidase 16 [Source:HGNC Symbol;Acc:HGNC:12614]
ENSG00000131051	3257.75535	3672.52	4205.88522	4606.32235	-0.346379288	0.0022099	0.0108576	RBM39	RNA binding motif protein 39 [Source:HGNC Symbol;Acc:HGNC:15923]
ENSG00000178802	1067.87449	881.582001	1290.63362	1350.9182	-0.438997343	0.0022103	0.0108576	MPI	mannose phosphate isomerase [Source:HGNC Symbol;Acc:HGNC:7216]
ENSG00000141696	1398.5711	1252.82106	1717.33289	1745.86749	-0.385733505	0.0022119	0.0108622	P3H4	prolyl 3-hydroxylase family member 4 (non-enzymatic) [Source:HGNC Symbol;Acc:HGNC:16946]
ENSG00000181555	2112.12779	2066.18013	2621.30321	2663.51248	-0.338993328	0.0022129	0.010864	SETD2	SET domain containing 2 [Source:HGNC Symbol;Acc:HGNC:18420]
ENSG00000137478	549.192594	547.555454	394.038345	358.754479	0.542831104	0.0022182	0.0108867	FCHSD2	FCH and double SH3 domains 2 [Source:HGNC Symbol;Acc:HGNC:29114]
ENSG00000148346	211.606465	215.300931	109.57216	129.875509	0.834445771	0.0022194	0.0108895	LCN2	lipocalin 2 [Source:HGNC Symbol;Acc:HGNC:6526]
ENSG00000066739	1184.99621	1344.0803	1016.70322	812.254206	0.46782381	0.0022274	0.0109255	ATG2B	autophagy related 2B [Source:HGNC Symbol;Acc:HGNC:20187]
ENSG00000099341	2614.07801	2514.50223	3239.75377	3201.11192	-0.328794084	0.0022308	0.0109392	PSMD8	proteasome 26S subunit, non-ATPase 8 [Source:HGNC Symbol;Acc:HGNC:9566]
ENSG00000198324	564.940052	474.902465	331.877216	364.077246	0.578647366	0.0022329	0.0109451	PHETA1	PH domain containing endocytic trafficking adaptor 1 [Source:HGNC Symbol;Acc:HGNC:26509]
ENSG00000154265	191.922143	280.865824	132.750886	127.746402	0.861456584	0.0022333	0.0109451	ABCA5	ATP binding cassette subfamily A member 5 [Source:HGNC Symbol;Acc:HGNC:35]
ENSG00000110721	865.125968	839.053423	659.540117	563.148722	0.478824581	0.0022376	0.010963	CHKA	choline kinase alpha [Source:HGNC Symbol;Acc:HGNC:1937]
ENSG00000167528	312.980726	340.228632	211.769271	196.94237	0.676808735	0.0022418	0.0109803	ZNF641	zinc finger protein 641 [Source:HGNC Symbol;Acc:HGNC:31834]
ENSG00000067221	344.475641	266.689631	184.376231	183.103176	0.732769697	0.0022453	0.0109944	STOML1	stomatin like 1 [Source:HGNC Symbol;Acc:HGNC:14560]
ENSG00000145623	4498.85188	4765.85888	3857.15075	3522.60704	0.328235076	0.0022487	0.0110076	OSMR	oncostatin M receptor [Source:HGNC Symbol;Acc:HGNC:8507]
ENSG00000169972	292.312187	240.995281	378.234668	456.693387	-0.647758633	0.0022531	0.0110248	PUSL1	pseudouridylate synthase like 1 [Source:HGNC Symbol;Acc:HGNC:26914]
ENSG00000277443	1805.05236	1993.52714	2638.16047	2303.69345	-0.379373234	0.0022535	0.0110248	MARCKS	myristoylated alanine rich protein kinase C substrate [Source:HGNC Symbol;Acc:HGNC:6759]
ENSG00000129347	913.352558	804.498952	1110.4717	1214.65537	-0.437199334	0.0022581	0.0110443	KRI1	KRI1 homolog [Source:HGNC Symbol;Acc:HGNC:25769]
ENSG00000125970	3918.16437	3567.08456	4599.92357	4778.77999	-0.325494554	0.002269	0.0110924	RALY	RALY heterogeneous nuclear ribonucleoprotein [Source:HGNC Symbol;Acc:HGNC:15921]
ENSG00000156603	354.317803	379.213162	516.253447	555.696849	-0.54685528	0.0022693	0.0110924	MED19	mediator complex subunit 19 [Source:HGNC Symbol;Acc:HGNC:29600]
ENSG00000197943	301.170132	303.016125	194.912016	177.780409	0.696963685	0.0022759	0.0111208	PLCG2	phospholipase C gamma 2 [Source:HGNC Symbol;Acc:HGNC:9066]
ENSG00000162772	644.661557	559.073611	401.413394	427.950447	0.53689795	0.0022764	0.0111208	ATF3	activating transcription factor 3 [Source:HGNC Symbol;Acc:HGNC:785]
ENSG00000181938	403.528608	432.373886	559.450163	654.70031	-0.538086188	0.0022779	0.0111247	GINS3	GINS complex subunit 3 [Source:HGNC Symbol;Acc:HGNC:25851]
ENSG00000139641	4069.73365	3838.20425	4981.31897	4847.97596	-0.313893722	0.0022785	0.0111247	ESYT1	extended synaptotagmin 1 [Source:HGNC Symbol;Acc:HGNC:29534]
ENSG00000147650	561.003187	598.944154	377.18109	422.62768	0.536683592	0.0022822	0.0111395	LRP12	LDL receptor related protein 12 [Source:HGNC Symbol;Acc:HGNC:31708]
ENSG00000276234	234.243436	213.528907	352.948785	349.173499	-0.649617607	0.0022832	0.0111413	TADA2A	transcriptional adaptor 2A [Source:HGNC Symbol;Acc:HGNC:11531]
ENSG00000103319	956.658067	885.12605	1244.27616	1206.13895	-0.412271435	0.0022866	0.0111516	EEF2K	eukaryotic elongation factor 2 kinase [Source:HGNC Symbol;Acc:HGNC:24615]
ENSG00000084073	838.552133	994.105533	1323.29455	1176.33145	-0.447181662	0.0022867	0.0111516	ZMPSTE24	zinc metallopeptidase STE24 [Source:HGNC Symbol;Acc:HGNC:12877]
ENSG00000163435	1732.22037	1490.27229	2098.7283	2099.29921	-0.381970374	0.0022948	0.0111878	ELF3	E74 like ETS transcription factor 3 [Source:HGNC Symbol;Acc:HGNC:3318]
ENSG00000099849	561.987403	472.244429	720.647668	746.251899	-0.505085694	0.0023023	0.0112206	RASSF7	Ras association domain family member 7 [Source:HGNC Symbol;Acc:HGNC:1166]
ENSG00000165732	3815.80589	4360.06536	5906.36086	4776.65088	-0.385744946	0.0023028	0.0112206	DDX21	DExD-box helicase 21 [Source:HGNC Symbol;Acc:HGNC:2744]
ENSG00000119906	436.991956	548.441466	757.522914	672.797717	-0.536476981	0.0023044	0.0112213	SLF2	SMC5-SMC6 complex localization factor 2 [Source:HGNC Symbol;Acc:HGNC:17814]
ENSG00000165775	705.682957	734.503999	1010.38175	943.194268	-0.439694797	0.0023046	0.0112213	FUNDC2	FUN14 domain containing 2 [Source:HGNC Symbol;Acc:HGNC:24925]
ENSG00000145220	462.581576	504.140863	659.540117	708.992531	-0.500945594	0.0023054	0.0112213	LYAR	Ly1 antibody reactive [Source:HGNC Symbol;Acc:HGNC:26021]
ENSG00000176720	419.276066	329.596487	248.644517	219.29799	0.677304505	0.0023056	0.0112213	BOK	BOK, BCL2 family apoptosis regulator [Source:HGNC Symbol;Acc:HGNC:1087]
ENSG00000132294	1149.56443	1337.87821	996.685225	792.027693	0.476031022	0.002308	0.0112297	EFR3A	EFR3 homolog A [Source:HGNC Symbol;Acc:HGNC:28970]
ENSG00000183128	132.869176	115.181568	228.626526	208.652457	-0.819098748	0.0023125	0.0112482	CALHM3	calcium homeostasis modulator 3 [Source:HGNC Symbol;Acc:HGNC:23458]
ENSG00000108592	2556.00926	2270.84891	3020.60945	3129.78685	-0.349860585	0.0023152	0.0112584	FTSJ3	FtsJ RNA methyltransferase homolog 3 [Source:HGNC Symbol;Acc:HGNC:17136]
ENSG00000120451	1138.73805	1089.79484	860.773604	800.544119	0.423575864	0.0023177	0.0112672	SNX19	sorting nexin 19 [Source:HGNC Symbol;Acc:HGNC:21532]
ENSG00000112029	444.865685	583.881949	737.504924	754.768325	-0.535379371	0.002325	0.0112993	FBXO5	F-box protein 5 [Source:HGNC Symbol;Acc:HGNC:13584]
ENSG00000122482	379.907422	477.560501	746.98713	555.696849	-0.602444259	0.0023307	0.0113236	ZNF644	zinc finger protein 644 [Source:HGNC Symbol;Acc:HGNC:29222]
ENSG00000075290	28.5422674	13.2901809	0	1.06455335	5.300463634	0.0023435	0.0113799	WNT8B	Wnt family member 8B [Source:HGNC Symbol;Acc:HGNC:12789]
ENSG00000075914	774.578085	607.804274	954.542087	983.647296	-0.488531858	0.002344	0.0113799	EXOSC7	exosome component 7 [Source:HGNC Symbol;Acc:HGNC:28112]
ENSG00000164896	1570.80892	1328.13208	1804.77991	2048.20065	-0.410883873	0.0023446	0.0113799	FASTK	Fas activated serine/threonine kinase [Source:HGNC Symbol;Acc:HGNC:24676]
ENSG00000112964	18.7001062	23.9223257	1.05357846	2.1291067	3.745884707	0.0023449	0.0113799	GHR	growth hormone receptor [Source:HGNC Symbol;Acc:HGNC:4263]
ENSG00000167264	310.028077	276.435763	433.020748	450.306067	-0.591631378	0.0023484	0.0113922	DUS2	dihydrouridine synthase 2 [Source:HGNC Symbol;Acc:HGNC:26014]
ENSG00000145284	565.924268	597.17213	382.448982	422.62768	0.531068773	0.0023488	0.0113922	SCD5	stearoyl-CoA desaturase 5 [Source:HGNC Symbol;Acc:HGNC:21088]
ENSG00000145088	75.7846411	84.1711459	20.0179908	37.2593673	1.482749259	0.0023507	0.0113978	EAF2	ELL associated factor 2 [Source:HGNC Symbol;Acc:HGNC:23115]
ENSG00000171872	6.88951283	7.08809649	34.7680893	35.1302606	-2.321701308	0.0023515	0.0113988	KLF17	Kruppel like factor 17 [Source:HGNC Symbol;Acc:HGNC:18830]
ENSG00000174013	865.125968	949.80493	673.236637	652.571204	0.453376164	0.0023544	0.0114096	FBXO45	F-box protein 45 [Source:HGNC Symbol;Acc:HGNC:29148]
ENSG00000130227	2066.85385	2110.48073	2669.76782	2608.15571	-0.337334074	0.0023555	0.0114113	XPO7	exportin 7 [Source:HGNC Symbol;Acc:HGNC:14108]

ENSG00000099625	170.269388	131.129785	71.6433354	77.7123946	1.011755533	0.0023592	0.0114263	CBARP	CACN subunit beta associated regulatory protein [Source:HGNC Symbol;Acc:HGNC:28617]
ENSG00000135631	1990.08499	1702.02917	1393.88431	1415.85596	0.393638468	0.0023774	0.0115112	RAB11FIP5	RAB11 family interacting protein 5 [Source:HGNC Symbol;Acc:HGNC:24845]
ENSG00000105327	216.527546	164.798243	346.627314	287.429405	-0.735418138	0.0023797	0.0115188	BBC3	BCL2 binding component 3 [Source:HGNC Symbol;Acc:HGNC:17868]
ENSG00000147894	114.16907	161.254195	75.8576493	50.0340075	1.130533654	0.0023864	0.0115482	C9orf72	chromosome 9 open reading frame 72 [Source:HGNC Symbol;Acc:HGNC:28337]
ENSG00000121858	166.332524	136.445858	264.448194	249.105484	-0.763756488	0.0023925	0.0115743	TNFSF10	TNF superfamily member 10 [Source:HGNC Symbol;Acc:HGNC:11925]
ENSG00000189280	39.3686447	41.6425669	93.7684831	97.9389082	-1.242121514	0.0024073	0.0116422	GJB5	gap junction protein beta 5 [Source:HGNC Symbol;Acc:HGNC:4287]
ENSG00000153107	1401.52375	1556.72319	2090.29967	1805.48248	-0.396903005	0.0024101	0.0116526	ANAPC1	anaphase promoting complex subunit 1 [Source:HGNC Symbol;Acc:HGNC:19988]
ENSG00000181350	168.300956	154.166099	87.4470124	79.8415013	0.946282517	0.0024179	0.0116869	LRRC75A	leucine rich repeat containing 75A [Source:HGNC Symbol;Acc:HGNC:32403]
ENSG00000144040	321.838671	282.637848	440.395797	467.338921	-0.587381836	0.0024231	0.0117078	SFXN5	sideroflexin 5 [Source:HGNC Symbol;Acc:HGNC:16073]
ENSG00000118513	150.585066	152.394075	83.2326985	70.2605211	0.980942013	0.0024236	0.0117078	MYB	MYB proto-oncogene, transcription factor [Source:HGNC Symbol;Acc:HGNC:7545]
ENSG00000108107	8321.54728	7261.75486	9336.81233	10253.7779	-0.330261709	0.002426	0.011716	RPL28	ribosomal protein L28 [Source:HGNC Symbol;Acc:HGNC:10330]
ENSG00000196510	1065.90606	1018.91387	1336.99107	1403.08132	-0.394460934	0.0024344	0.0117531	ANAPC7	anaphase promoting complex subunit 7 [Source:HGNC Symbol;Acc:HGNC:17380]
ENSG00000266338	609.229777	598.944154	435.127905	410.917593	0.513908495	0.0024353	0.0117544	NBPF15	NBPF member 15 [Source:HGNC Symbol;Acc:HGNC:28791]
ENSG00000107485	45.2739414	36.3264945	6.32147077	11.7100869	2.177990697	0.0024387	0.0117671	GATA3	GATA binding protein 3 [Source:HGNC Symbol;Acc:HGNC:4172]
ENSG00000129538	13.7790257	9.74613268	0	0	5.914841112	0.0024434	0.0117866	RNASE1	ribonuclease A family member 1, pancreatic [Source:HGNC Symbol;Acc:HGNC:10044]
ENSG00000134748	903.510397	1115.48919	1405.47367	1332.82079	-0.438928105	0.0024525	0.0118273	PRPF38A	pre-mRNA processing factor 38A [Source:HGNC Symbol;Acc:HGNC:25930]
ENSG00000168952	12.7948095	10.6321447	0	0	5.909351034	0.002457	0.0118457	STXBP6	syntaxin binding protein 6 [Source:HGNC Symbol;Acc:HGNC:19666]
ENSG00000118276	120.074366	114.295556	66.3754431	20.2265137	1.435328089	0.0024678	0.011894	B4GALT6	beta-1,4-galactosyltransferase 6 [Source:HGNC Symbol;Acc:HGNC:929]
ENSG00000274267	58.068751	62.0208443	16.8572554	21.291067	1.655026608	0.002482	0.0119591	HIST1H3B	histone cluster 1 H3 family member b [Source:HGNC Symbol;Acc:HGNC:4776]
ENSG00000127507	153.537714	192.264617	85.3398554	95.8098015	0.933943105	0.0024837	0.0119639	ADGRE2	adhesion G protein-coupled receptor E2 [Source:HGNC Symbol;Acc:HGNC:3337]
ENSG00000145979	564.940052	616.664395	344.520157	450.306067	0.572499844	0.0024848	0.011966	TBC1D7	TBC1 domain family member 7 [Source:HGNC Symbol;Acc:HGNC:21066]
ENSG00000162521	2740.05767	3133.82466	3869.79369	3610.96496	-0.348629197	0.0024867	0.0119717	RBBP4	RB binding protein 4, chromatin remodeling factor [Source:HGNC Symbol;Acc:HGNC:9887]
ENSG00000164087	570.845349	531.607237	730.129874	806.931439	-0.479788471	0.0024951	0.0120086	POC1A	POC1 centriolar protein A [Source:HGNC Symbol;Acc:HGNC:24488]
ENSG00000108039	1479.27683	1531.02884	1132.59685	1177.39601	0.38214595	0.0025016	0.0120365	XPNPEP1	X-prolyl aminopeptidase 1 [Source:HGNC Symbol;Acc:HGNC:12822]
ENSG00000185215	9840.19275	9313.75879	11388.1296	12108.2298	-0.294835159	0.0025057	0.012049	TNFAIP2	TNF alpha induced protein 2 [Source:HGNC Symbol;Acc:HGNC:11895]
ENSG00000140386	542.303081	540.467358	384.556139	363.012692	0.534394395	0.002506	0.012049	SCAPER	S-phase cyclin A associated protein in the ER [Source:HGNC Symbol;Acc:HGNC:13081]
ENSG00000164647	77.7530733	96.5753147	37.9288246	30.8720472	1.34211754	0.0025063	0.012049	STEAP1	STEAP family member 1 [Source:HGNC Symbol;Acc:HGNC:11378]
ENSG00000181192	1265.70193	1124.34931	857.612868	921.903201	0.425212985	0.0025184	0.0121033	DHTKD1	dehydrogenase E1 and transketolase domain containing 1 [Source:HGNC Symbol;Acc:HGNC:23537]
ENSG00000136875	2177.08605	2064.4081	2621.30321	2746.54764	-0.339906001	0.002527	0.0121412	PRPF4	pre-mRNA processing factor 4 [Source:HGNC Symbol;Acc:HGNC:17349]
ENSG00000168936	574.782213	447.436091	753.308601	717.508958	-0.526103034	0.0025297	0.0121511	TMEM129	transmembrane protein 129 [Source:HGNC Symbol;Acc:HGNC:25137]
ENSG00000141526	14751.4312	11495.1205	16882.5413	16879.5579	-0.363373014	0.0025319	0.012158	SLC16A3	solute carrier family 16 member 3 [Source:HGNC Symbol;Acc:HGNC:10924]
ENSG00000126216	671.235393	767.286446	990.363755	970.872655	-0.446565293	0.002533	0.01216	TUBGCP3	tubulin gamma complex associated protein 3 [Source:HGNC Symbol;Acc:HGNC:18598]
ENSG00000136643	691.903931	956.893027	581.575311	548.244975	0.546193263	0.0025338	0.0121603	RPS6KC1	ribosomal protein S6 kinase C1 [Source:HGNC Symbol;Acc:HGNC:10439]
ENSG00000133401	506.871301	502.368839	369.80604	310.849578	0.568119952	0.0025354	0.0121626	PDZD2	PDZ domain containing 2 [Source:HGNC Symbol;Acc:HGNC:18486]
ENSG00000141084	714.540902	674.255179	950.327773	930.419628	-0.437770176	0.0025357	0.0121626	RANBP10	RAN binding protein 10 [Source:HGNC Symbol;Acc:HGNC:29285]
ENSG00000148341	3016.6224	2569.43498	2206.1933	2122.71938	0.36752767	0.0025457	0.0122069	SH3GLB2	SH3 domain containing GRB2 like, endophilin B2 [Source:HGNC Symbol;Acc:HGNC:10834]
ENSG00000151224	10.8263773	12.4041689	0	0	5.898320224	0.0025466	0.0122077	MAT1A	methionine adenosyltransferase 1A [Source:HGNC Symbol;Acc:HGNC:6903]
ENSG00000103932	1127.91167	966.639159	1441.29534	1353.04731	-0.416453117	0.0025479	0.0122107	RPAP1	RNA polymerase II associated protein 1 [Source:HGNC Symbol;Acc:HGNC:24567]
ENSG00000105755	609.229777	481.990562	333.984373	390.69108	0.589772189	0.002554	0.0122364	ETHE1	ETHE1, persulfide dioxygenase [Source:HGNC Symbol;Acc:HGNC:23287]
ENSG00000165591	76.7688572	105.435435	38.9824031	34.0657072	1.319970937	0.0025624	0.0122732	FAAH2	fatty acid amide hydrolase 2 [Source:HGNC Symbol;Acc:HGNC:26440]
ENSG00000172671	160.427227	187.834557	95.8756401	89.4224814	0.911043621	0.002566	0.0122868	ZFAND4	zinc finger AN1-type containing 4 [Source:HGNC Symbol;Acc:HGNC:23504]
ENSG00000110713	3723.28958	3674.29202	4637.85239	4514.77076	-0.307161691	0.0025709	0.012307	NUP98	nucleoporin 98 [Source:HGNC Symbol;Acc:HGNC:8068]
ENSG00000073060	8140.45151	7191.75991	9452.70596	9574.59283	-0.311615154	0.0025863	0.012377	SCARB1	scavenger receptor class B member 1 [Source:HGNC Symbol;Acc:HGNC:1664]
ENSG00000006744	2848.32145	2560.57486	3366.18319	3462.99205	-0.336603335	0.0025924	0.0124029	ELAC2	elaC ribonuclease Z 2 [Source:HGNC Symbol;Acc:HGNC:14198]
ENSG00000143126	2666.24146	2466.65758	2107.15692	1897.03407	0.358072836	0.0025981	0.0124251	CELSR2	cadherin EGF LAG seven-pass G-type receptor 2 [Source:HGNC Symbol;Acc:HGNC:3231]
ENSG00000107077	427.149795	389.845307	270.769665	267.202891	0.602354322	0.0025985	0.0124251	KDM4C	lysine demethylase 4C [Source:HGNC Symbol;Acc:HGNC:17071]
ENSG00000164199	55.1161026	60.2488202	16.8572554	19.1619603	1.679955258	0.0026007	0.0124316	ADGRV1	adhesion G protein-coupled receptor V1 [Source:HGNC Symbol;Acc:HGNC:17416]
ENSG00000184939	615.135074	649.446841	438.28864	454.564281	0.502424329	0.0026035	0.0124417	ZFP90	ZFP90 zinc finger protein [Source:HGNC Symbol;Acc:HGNC:23329]
ENSG00000109332	2361.13447	2518.04628	3385.1476	2889.19779	-0.362721344	0.0026066	0.0124529	UBE2D3	ubiquitin conjugating enzyme E2 D3 [Source:HGNC Symbol;Acc:HGNC:12476]
ENSG00000137414	440.928821	467.814369	342.413	231.008077	0.664260223	0.0026108	0.0124696	FAM8A1	family with sequence similarity 8 member A1 [Source:HGNC Symbol;Acc:HGNC:16372]
ENSG00000127804	921.226287	908.162363	1227.41891	1189.10609	-0.401648738	0.0026143	0.0124803	METTL16	methyltransferase like 16 [Source:HGNC Symbol;Acc:HGNC:28484]
ENSG00000151320	94.4847473	122.269665	56.893237	34.0657072	1.253392798	0.0026145	0.0124803	AKAP6	A-kinase anchoring protein 6 [Source:HGNC Symbol;Acc:HGNC:376]
ENSG00000244398	1281.44939	1336.9922	1590.90348	1851.25828	-0.394425987	0.002622	0.0125123	AC116533.1	ribosomal protein L36a (RPL36A) pseudogene
ENSG00000100031	409.433905	307.446185	244.430203	191.619603	0.716004442	0.0026234	0.0125154	GGT1	gamma-glutamyltransferase 1 [Source:HGNC Symbol;Acc:HGNC:4250]
ENSG00000119041	608.245561	659.192974	908.184635	832.48072	-0.45735953	0.0026307	0.012547	GTF3C3	general transcription factor IIIC subunit 3 [Source:HGNC Symbol;Acc:HGNC:4666]

ENSG00000168264	1420.22386	1484.95622	1883.79829	1845.93551	-0.360294712	0.0026324	0.0125516	IRF2BP2	interferon regulatory factor 2 binding protein 2 [Source:HGNC Symbol;Acc:HGNC:21729]
ENSG00000221838	184.048414	149.736038	252.858831	307.655918	-0.749162237	0.0026346	0.0125583	AP4M1	adaptor related protein complex 4 subunit mu 1 [Source:HGNC Symbol;Acc:HGNC:574]
ENSG00000106628	4125.83397	3538.73217	4744.26382	4961.88317	-0.340917564	0.0026386	0.0125737	POLD2	DNA polymerase delta 2, accessory subunit [Source:HGNC Symbol;Acc:HGNC:9176]
ENSG00000196605	81.6899378	118.725616	40.0359816	43.6466874	1.261782879	0.0026425	0.0125889	ZNF846	zinc finger protein 846 [Source:HGNC Symbol;Acc:HGNC:27260]
ENSG00000175792	2265.6655	2054.66197	2682.41077	2809.35629	-0.346369626	0.0026434	0.0125895	RUVBL1	RuvB like AAA ATPase 1 [Source:HGNC Symbol;Acc:HGNC:10474]
ENSG00000225173	34.4475641	33.6684583	4.21431385	8.5164268	2.42067914	0.0026751	0.0127373	AL662890.1	novel transcript
ENSG00000063322	808.041433	884.240038	1151.56126	1109.26459	-0.417492839	0.0026845	0.0127783	MED29	mediator complex subunit 29 [Source:HGNC Symbol;Acc:HGNC:23074]
ENSG00000259523	17.7158901	22.1503015	2.10715692	0	4.23739988	0.0026876	0.0127894	AC022613.2	novel transcript, antisense to TJP1
ENSG00000033030	663.361664	646.788805	869.202231	910.193114	-0.441764594	0.0027006	0.0128474	ZCCHC8	zinc finger CCHC-type containing 8 [Source:HGNC Symbol;Acc:HGNC:25265]
ENSG00000075188	613.166642	739.820072	906.077478	967.678995	-0.468936629	0.0027015	0.0128481	NUP37	nucleoporin 37 [Source:HGNC Symbol;Acc:HGNC:29929]
ENSG00000111276	776.546517	753.996265	1045.14983	1004.93836	-0.421800956	0.0027037	0.0128552	CDKN1B	cyclin dependent kinase inhibitor 1B [Source:HGNC Symbol;Acc:HGNC:1785]
ENSG00000143093	683.045986	673.369167	940.845567	895.289368	-0.43696681	0.0027129	0.0128953	STRIP1	striatin interacting protein 1 [Source:HGNC Symbol;Acc:HGNC:25916]
ENSG00000003436	350.380938	446.550079	645.843597	541.857655	-0.57451766	0.0027222	0.0129358	TFPI	tissue factor pathway inhibitor [Source:HGNC Symbol;Acc:HGNC:11760]
ENSG00000103042	728.319927	647.674817	939.791988	932.548735	-0.444935416	0.0027252	0.0129466	SLC38A7	solute carrier family 38 member 7 [Source:HGNC Symbol;Acc:HGNC:25582]
ENSG00000244716	915.32099	714.125722	1171.57925	1084.77986	-0.470536948	0.0027274	0.012953	BX679664.3	ribosomal protein L17 (RPL17) pseudogene
ENSG00000205189	143.695553	183.404497	94.8220616	71.3250745	0.978125576	0.0027282	0.0129533	ZBTB10	zinc finger and BTB domain containing 10 [Source:HGNC Symbol;Acc:HGNC:30953]
ENSG00000069431	251.959326	311.876246	470.949573	399.207506	-0.624824795	0.0027322	0.0129687	ABCC9	ATP binding cassette subfamily C member 9 [Source:HGNC Symbol;Acc:HGNC:60]
ENSG00000112218	38.3844286	38.0985187	8.4286277	8.5164268	2.174253389	0.0027331	0.0129694	GPR63	G protein-coupled receptor 63 [Source:HGNC Symbol;Acc:HGNC:13302]
ENSG00000159352	3372.90864	2727.14513	3812.90046	4094.27219	-0.374656742	0.0027487	0.0130397	PSMD4	proteasome 26S subunit, non-ATPase 4 [Source:HGNC Symbol;Acc:HGNC:9561]
ENSG00000118816	2656.3993	2840.55467	3496.82692	3387.40876	-0.324518117	0.0027517	0.0130484	CCNI	cyclin I [Source:HGNC Symbol;Acc:HGNC:1595]
ENSG00000073146	9.84216118	9.74613268	49.5181877	33.0011539	-2.075069943	0.0027521	0.0130484	MOV10L1	Mov10 like RISC complex RNA helicase 1 [Source:HGNC Symbol;Acc:HGNC:7201]
ENSG00000181019	8132.57778	8519.00597	10019.5312	10303.8119	-0.287431384	0.0027545	0.0130564	NQO1	NAD(P)H quinone dehydrogenase 1 [Source:HGNC Symbol;Acc:HGNC:2874]
ENSG00000205534	51.1792381	62.9068564	109.57216	135.198275	-1.099340422	0.0027621	0.0130852	SMG1P2	SMG1 pseudogene 2 [Source:HGNC Symbol;Acc:HGNC:49859]
ENSG00000125735	38.3844286	26.5803619	5.26789231	6.3873201	2.477430901	0.0027622	0.0130852	TNFSF14	TNF superfamily member 14 [Source:HGNC Symbol;Acc:HGNC:11930]
ENSG00000163083	24.605403	20.3782774	3.16073539	1.06455335	3.409349153	0.002773	0.0131328	INHBB	inhibin subunit beta B [Source:HGNC Symbol;Acc:HGNC:6067]
ENSG00000151500	551.161026	559.959623	738.558502	796.285906	-0.465957929	0.0027898	0.0132088	THYN1	thymocyte nuclear protein 1 [Source:HGNC Symbol;Acc:HGNC:29560]
ENSG00000140463	616.11929	621.094455	369.80604	476.919901	0.547299672	0.0027919	0.0132149	BBS4	Bardet-Biedl syndrome 4 [Source:HGNC Symbol;Acc:HGNC:969]
ENSG00000013503	365.14418	407.565548	563.664477	551.438635	-0.528530899	0.0027941	0.0132215	POLR3B	RNA polymerase III subunit B [Source:HGNC Symbol;Acc:HGNC:30348]
ENSG00000100099	493.092275	472.244429	638.468548	722.831725	-0.496062237	0.0027957	0.0132256	HPS4	HPS4, biogenesis of lysosomal organelles complex 3 subunit 2 [Source:HGNC Symbol;Acc:HGNC:15844]
ENSG00000175182	511.792381	474.902465	336.091529	337.463412	0.550468259	0.0027965	0.0132256	FAM131A	family with sequence similarity 131 member A [Source:HGNC Symbol;Acc:HGNC:28308]
ENSG00000168273	299.2017	266.689631	387.716874	479.049008	-0.615712297	0.002802	0.0132478	SMIM4	small integral membrane protein 4 [Source:HGNC Symbol;Acc:HGNC:37257]
ENSG00000090273	4746.87434	4114.64001	5251.03506	6074.34142	-0.354087678	0.0028032	0.0132499	NUDC	nuclear distribution C, dynein complex regulator [Source:HGNC Symbol;Acc:HGNC:8045]
ENSG00000170442	52.1634543	53.1607237	120.107945	107.519888	-1.111722362	0.002814	0.0132941	KRT86	keratin 86 [Source:HGNC Symbol;Acc:HGNC:6463]
ENSG00000238266	97.4373957	101.005375	33.7145108	50.0340075	1.245159813	0.0028141	0.0132941	LINC00707	long intergenic non-protein coding RNA 707 [Source:HGNC Symbol;Acc:HGNC:44691]
ENSG00000146729	451.755198	471.358417	775.433748	578.052469	-0.552009822	0.0028211	0.0133231	NIPSNAP2	nipsnap homolog 2 [Source:HGNC Symbol;Acc:HGNC:4179]
ENSG00000142700	277.548945	208.212835	371.913197	393.88474	-0.658433954	0.0028227	0.0133247	DMRTA2	DMRT like family A2 [Source:HGNC Symbol;Acc:HGNC:13908]
ENSG00000137776	1802.09971	1953.6566	2307.33683	2489.99029	-0.352882708	0.002823	0.0133247	SLTM	SAFB like transcription modulator [Source:HGNC Symbol;Acc:HGNC:20709]
ENSG00000154920	215.54333	228.591112	346.627314	343.850732	-0.636185093	0.0028282	0.0133457	EME1	essential meiotic structure-specific endonuclease 1 [Source:HGNC Symbol;Acc:HGNC:24965]
ENSG00000128284	92.5163151	63.7928684	143.286671	168.199429	-0.997653816	0.002842	0.0134072	APOL3	apolipoprotein L3 [Source:HGNC Symbol;Acc:HGNC:14868]
ENSG00000116017	612.182426	518.317056	808.094681	769.672072	-0.481764849	0.0028443	0.0134142	ARID3A	AT-rich interaction domain 3A [Source:HGNC Symbol;Acc:HGNC:3031]
ENSG00000163884	3.93686447	20.3782774	0	0	5.96776364	0.0028461	0.013419	KLF15	Kruppel like factor 15 [Source:HGNC Symbol;Acc:HGNC:14536]
ENSG00000123570	15.7474579	23.9223257	2.10715692	0	4.23098536	0.0028487	0.0134237	RAB9B	RAB9B, member RAS oncogene family [Source:HGNC Symbol;Acc:HGNC:14090]
ENSG00000151689	587.577023	531.607237	395.091923	381.110099	0.527482565	0.0028492	0.0134237	INPP1	inositol polyphosphate-1-phosphatase [Source:HGNC Symbol;Acc:HGNC:6071]
ENSG00000144048	322.822887	357.062861	470.949573	531.212122	-0.559083678	0.0028495	0.0134237	DUSP11	dual specificity phosphatase 11 [Source:HGNC Symbol;Acc:HGNC:3066]
ENSG00000197872	7.87372895	15.0622051	0	0	5.881558298	0.0028592	0.0134658	FAM49A	family with sequence similarity 49 member A [Source:HGNC Symbol;Acc:HGNC:25373]
ENSG00000246705	3208.54455	2828.1505	2217.78266	2483.60297	0.360487016	0.0028757	0.0135397	H2AFJ	H2A histone family member J [Source:HGNC Symbol;Acc:HGNC:14456]
ENSG00000167716	754.893763	601.60219	999.845961	896.353921	-0.484184793	0.0028798	0.0135521	WDR81	WD repeat domain 81 [Source:HGNC Symbol;Acc:HGNC:26600]
ENSG00000112773	1650.53043	1587.73361	1320.13381	1150.78217	0.390017187	0.00288	0.0135521	TENT5A	terminal nucleotidyltransferase 5A [Source:HGNC Symbol;Acc:HGNC:18345]
ENSG00000110492	686.982851	588.312009	851.291398	906.999454	-0.464048387	0.0028811	0.0135536	MDK	midkine [Source:HGNC Symbol;Acc:HGNC:6972]
ENSG00000262580	116.137502	108.093472	48.4646093	54.2922209	1.125504332	0.0028821	0.0135546	AC087741.1	novel transcript, antisense to CARD14
ENSG00000100379	841.504781	707.923637	555.23585	550.374082	0.486254667	0.0028862	0.0135703	KCTD17	potassium channel tetramerization domain containing 17 [Source:HGNC Symbol;Acc:HGNC:25705]
ENSG00000168394	1301.13371	1149.15764	897.64885	940.000608	0.414719118	0.0028972	0.0136182	TAP1	transporter 1, ATP binding cassette subfamily B member [Source:HGNC Symbol;Acc:HGNC:43]

ENSG00000085840	1124.95902	1033.97608	1355.95548	1485.05192	-0.396375625	0.0028983	0.0136193	ORC1	origin recognition complex subunit 1 [Source:HGNC Symbol;Acc:HGNC:8487]
ENSG00000113068	806.073001	910.8204	1066.2214	1275.33491	-0.447150639	0.002905	0.0136471	PFDN1	prefoldin subunit 1 [Source:HGNC Symbol;Acc:HGNC:8866]
ENSG00000233593	71.8477766	95.6893027	34.7680893	30.8720472	1.353054351	0.0029062	0.0136491	AL590094.1	uncharacterized LOC105378853 [Source:NCBI gene;Acc:105378853]
ENSG00000105821	452.739414	537.809322	711.165462	689.830571	-0.499270615	0.0029152	0.0136876	DNAJC2	DnaJ heat shock protein family (Hsp40) member C2 [Source:HGNC Symbol;Acc:HGNC:13192]
ENSG00000131508	1679.0727	1836.703	2484.33801	2100.36376	-0.382795229	0.0029191	0.0137018	UBE2D2	ubiquitin conjugating enzyme E2 D2 [Source:HGNC Symbol;Acc:HGNC:12475]
ENSG00000053918	87.5952345	95.6893027	159.090348	183.103176	-0.899907814	0.002923	0.0137166	KCNQ1	potassium voltage-gated channel subfamily Q member 1 [Source:HGNC Symbol;Acc:HGNC:6294]
ENSG00000123191	362.191531	348.20274	223.358634	236.330844	0.627811254	0.0029472	0.0138232	ATP7B	ATPase copper transporting beta [Source:HGNC Symbol;Acc:HGNC:870]
ENSG00000183207	3051.06997	2353.24804	3347.21877	3822.81108	-0.408223254	0.0029476	0.0138232	RUVBL2	RuvB like AAA ATPase 2 [Source:HGNC Symbol;Acc:HGNC:10475]
ENSG00000130699	837.567917	834.623362	1071.4893	1151.84672	-0.41097402	0.0029482	0.0138232	TAF4	TATA-box binding protein associated factor 4 [Source:HGNC Symbol;Acc:HGNC:11537]
ENSG00000174080	1345.42343	1235.98683	1021.97111	924.032308	0.407337514	0.0029616	0.0138821	CTSF	cathepsin F [Source:HGNC Symbol;Acc:HGNC:2531]
ENSG00000198108	12.7948095	9.74613268	0	0	5.853432452	0.0029626	0.0138828	CHSY3	chondroitin sulfate synthase 3 [Source:HGNC Symbol;Acc:HGNC:24293]
ENSG00000144635	746.035818	719.441794	985.095862	978.324529	-0.422180232	0.0029661	0.0138931	DYNC1LI1	dynein cytoplasmic 1 light intermediate chain 1 [Source:HGNC Symbol;Acc:HGNC:18745]
ENSG00000051523	3733.13174	3018.64309	4159.52777	4581.83762	-0.372873223	0.0029664	0.0138931	CYBA	cytochrome b-245 alpha chain [Source:HGNC Symbol;Acc:HGNC:2577]
ENSG00000128692	208.653817	176.3164	276.037557	353.431712	-0.710368918	0.0029905	0.0139989	EIF2S2P4	eukaryotic translation initiation factor 2 subunit 2 beta pseudogene 4 [Source:HGNC Symbol;Acc:HGNC:37626]
ENSG00000165240	1158.42237	1400.78507	1031.45331	809.060546	0.475937283	0.0029906	0.0139989	ATP7A	ATPase copper transporting alpha [Source:HGNC Symbol;Acc:HGNC:869]
ENSG00000173812	7675.90151	7368.96232	8698.34378	10046.19	-0.317222837	0.0030081	0.0140769	EIF1	eukaryotic translation initiation factor 1 [Source:HGNC Symbol;Acc:HGNC:3249]
ENSG00000128607	957.642283	948.918918	697.468942	721.767171	0.425841665	0.0030151	0.0141054	KLHDC10	kelch domain containing 10 [Source:HGNC Symbol;Acc:HGNC:22194]
ENSG00000228223	207.669601	228.591112	379.288246	310.849578	-0.661156352	0.0030219	0.0141335	HCG11	HLA complex group 11 [Source:HGNC Symbol;Acc:HGNC:17707]
ENSG00000122863	2828.63712	2623.48171	3584.27393	3282.01798	-0.332892089	0.0030261	0.014149	CHST3	carbohydrate sulfotransferase 3 [Source:HGNC Symbol;Acc:HGNC:1971]
ENSG00000105991	106.295341	114.295556	61.1075508	33.0011539	1.228719911	0.0030286	0.0141567	HOXA1	homeobox A1 [Source:HGNC Symbol;Acc:HGNC:5099]
ENSG00000181090	2342.43436	2078.5843	2754.0541	2862.58396	-0.345611688	0.00303	0.0141583	EHMT1	euchromatic histone lysine methyltransferase 1 [Source:HGNC Symbol;Acc:HGNC:24650]
ENSG00000132692	67.9109122	61.1348323	22.1251477	22.3556204	1.53612751	0.0030306	0.0141583	BCAN	brevican [Source:HGNC Symbol;Acc:HGNC:23059]
ENSG00000108064	772.609653	906.390339	1390.72357	1000.68015	-0.509863225	0.0030359	0.0141793	TFAM	transcription factor A, mitochondrial [Source:HGNC Symbol;Acc:HGNC:11741]
ENSG00000204767	50.195022	51.3886996	14.7500985	14.9037469	1.776502635	0.0030427	0.014207	INSYN2B	inhibitory synaptic factor family member 2B [Source:HGNC Symbol;Acc:HGNC:37271]
ENSG00000203857	10.8263773	11.5181568	0	0	5.841961769	0.0030493	0.0142332	HSD3B1	hydroxy-delta-5-steroid dehydrogenase, 3 beta- and steroid delta-isomerase 1 [Source:HGNC Symbol;Acc:HGNC:5217]
ENSG00000100221	3382.7508	3156.86098	2533.8562	2678.41623	0.327177602	0.00305	0.0142332	JOSD1	Josephin domain containing 1 [Source:HGNC Symbol;Acc:HGNC:28953]
ENSG00000136731	4936.82805	5308.98427	4278.58214	3961.20302	0.314435513	0.0030535	0.014242	UGGT1	UDP-glucose glycoprotein glucosyltransferase 1 [Source:HGNC Symbol;Acc:HGNC:15663]
ENSG00000224578	215.54333	275.549751	374.020354	391.755633	-0.639367455	0.0030535	0.014242	HNRNPA1P48	heterogeneous nuclear ribonucleoprotein A1 pseudogene 48 [Source:HGNC Symbol;Acc:HGNC:48778]
ENSG00000160688	1857.21582	1843.7911	1456.04543	1442.46979	0.352579246	0.0030544	0.0142422	FLAD1	flavin adenine dinucleotide synthetase 1 [Source:HGNC Symbol;Acc:HGNC:24671]
ENSG00000220842	154.521931	187.834557	242.323046	339.592519	-0.763941175	0.0030618	0.0142727	RPL21P16	ribosomal protein L21 pseudogene 16 [Source:HGNC Symbol;Acc:HGNC:31396]
ENSG00000158164	59.0529671	54.9327478	22.1251477	12.7746402	1.706565925	0.0030636	0.0142772	TMSB15A	thymosin beta 15a [Source:HGNC Symbol;Acc:HGNC:30744]
ENSG00000119574	311.012293	273.777727	425.645699	448.17696	-0.580215695	0.0030668	0.0142845	ZBTB45	zinc finger and BTB domain containing 45 [Source:HGNC Symbol;Acc:HGNC:23715]
ENSG00000139737	15.7474579	27.4663739	0	3.19366005	3.764686735	0.003067	0.0142845	SLAIN1	SLAIN motif family member 1 [Source:HGNC Symbol;Acc:HGNC:26387]
ENSG00000112320	249.990894	275.549751	144.340249	171.393089	0.735699583	0.0030677	0.0142845	SOBP	sine oculis binding protein homolog [Source:HGNC Symbol;Acc:HGNC:29256]
ENSG00000204392	854.299591	838.16741	1038.82836	1250.85019	-0.436031396	0.0030705	0.0142936	LSM2	LSM2 homolog, U6 small nuclear RNA and mRNA degradation associated [Source:HGNC Symbol;Acc:HGNC:13940]
ENSG00000189410	65.9424799	61.1348323	23.1787262	20.2265137	1.549229185	0.003079	0.0143289	SH2D5	SH2 domain containing 5 [Source:HGNC Symbol;Acc:HGNC:28819]
ENSG00000164841	10.8263773	28.352386	1.05357846	1.06455335	4.21246657	0.00308	0.01433	TMEM74	transmembrane protein 74 [Source:HGNC Symbol;Acc:HGNC:26409]
ENSG00000106348	1995.99029	1677.22083	2322.08693	2436.76262	-0.373995048	0.0030883	0.0143645	IMPDH1	inosine monophosphate dehydrogenase 1 [Source:HGNC Symbol;Acc:HGNC:6052]
ENSG00000072121	1019.6479	858.545688	1246.38332	1265.75393	-0.420227323	0.0030902	0.0143675	ZFYVE26	zinc finger FYVE-type containing 26 [Source:HGNC Symbol;Acc:HGNC:20761]
ENSG00000115648	48.2265898	38.9845307	11.5893631	10.6455335	1.970585907	0.0030906	0.0143675	MLPH	melanophilin [Source:HGNC Symbol;Acc:HGNC:29643]
ENSG00000133067	120.074366	105.435435	187.536966	212.91067	-0.829394221	0.0030928	0.0143731	LGR6	leucine rich repeat containing G protein-coupled receptor 6 [Source:HGNC Symbol;Acc:HGNC:19719]
ENSG00000104723	17.7158901	23.0363136	2.10715692	1.06455335	3.683029544	0.0030936	0.0143731	TUSC3	tumor suppressor candidate 3 [Source:HGNC Symbol;Acc:HGNC:30242]
ENSG00000053371	1057.04811	925.882605	1270.61563	1359.43463	-0.407947093	0.0031039	0.0144173	AKR7A2	aldo-keto reductase family 7 member A2 [Source:HGNC Symbol;Acc:HGNC:389]
ENSG00000165312	1267.67036	1286.48951	1988.10256	1485.05192	-0.443421694	0.0031121	0.0144511	OTUD1	OTU deubiquitinase 1 [Source:HGNC Symbol;Acc:HGNC:27346]
ENSG00000236242	0	1.77202412	17.9108339	18.097407	-4.294103255	0.0031132	0.0144525	MYO16-AS1	MYO16 antisense RNA 1 [Source:HGNC Symbol;Acc:HGNC:39913]
ENSG00000123124	734.225224	784.120675	604.754037	446.047854	0.53106928	0.003127	0.0145124	WWP1	WW domain containing E3 ubiquitin protein ligase 1 [Source:HGNC Symbol;Acc:HGNC:17004]
ENSG00000148335	879.88921	855.887652	1102.04307	1199.75163	-0.407268091	0.0031285	0.0145155	NTMT1	N-terminal Xaa-Pro-Lys N-methyltransferase 1 [Source:HGNC Symbol;Acc:HGNC:23373]
ENSG00000165795	333.649264	311.876246	207.554957	203.32969	0.651391063	0.0031305	0.0145206	NDRG2	NDRG family member 2 [Source:HGNC Symbol;Acc:HGNC:14460]

ENSG00000143653	1435.97132	1589.50564	1881.69113	2019.45771	-0.366397411	0.0031435	0.014577	SCCPDH	saccharopine dehydrogenase (putative) [Source:HGNC Symbol;Acc:HGNC:24275]
ENSG00000161010	444.865685	367.695006	266.555351	265.073784	0.611211989	0.0031497	0.0145992	MRNIP	MRN complex interacting protein [Source:HGNC Symbol;Acc:HGNC:30817]
ENSG00000139746	505.887085	610.462311	878.684438	715.379851	-0.513183427	0.00315	0.0145992	RBM26	RNA binding motif protein 26 [Source:HGNC Symbol;Acc:HGNC:20327]
ENSG00000196588	1422.19229	1161.56181	1674.13618	1745.86749	-0.405126662	0.0031534	0.014611	MRTFA	myocardin related transcription factor A [Source:HGNC Symbol;Acc:HGNC:14334]
ENSG00000135406	71.8477766	92.1452544	31.6073539	33.0011539	1.345084397	0.0031593	0.0146343	PRPH	peripherin [Source:HGNC Symbol;Acc:HGNC:9461]
ENSG00000166295	1285.38625	1321.93	1662.54681	1685.18795	-0.36050967	0.0031694	0.0146751	ANAPC16	anaphase promoting complex subunit 16 [Source:HGNC Symbol;Acc:HGNC:26976]
ENSG00000233077	81.6899378	52.2747116	23.1787262	22.3556204	1.554795363	0.0031699	0.0146751	LINC01271	long intergenic non-protein coding RNA 1271 [Source:HGNC Symbol;Acc:HGNC:50327]
ENSG00000056291	30.5106997	27.4663739	5.26789231	4.2582134	2.604686299	0.0031764	0.0147016	NPFFR2	neuropeptide FF receptor 2 [Source:HGNC Symbol;Acc:HGNC:4525]
ENSG00000134504	333.649264	360.606909	485.699671	524.824802	-0.541045105	0.0031816	0.0147215	KCTD1	potassium channel tetramerization domain containing 1 [Source:HGNC Symbol;Acc:HGNC:18249]
ENSG00000089639	469.471088	400.477452	576.307419	679.185037	-0.530004748	0.0031852	0.0147285	GMIP	GEM interacting protein [Source:HGNC Symbol;Acc:HGNC:24852]
ENSG00000137877	117.121718	86.8291821	48.4646093	39.388474	1.213447983	0.0031854	0.0147285	SPTBN5	spectrin beta, non-erythrocytic 5 [Source:HGNC Symbol;Acc:HGNC:15680]
ENSG00000244879	319.870238	361.492921	211.769271	224.620757	0.64343219	0.0031858	0.0147285	GABPB1-AS1	GABPB1 antisense RNA 1 [Source:HGNC Symbol;Acc:HGNC:44157]
ENSG00000181523	799.183488	653.876902	947.167038	1054.97237	-0.463226692	0.0031916	0.0147516	SGSH	N-sulfoglucosamine sulfohydrolase [Source:HGNC Symbol;Acc:HGNC:10818]
ENSG00000037897	212.590682	191.378605	309.752068	326.817879	-0.656796581	0.0032085	0.0148258	METTL1	methyltransferase like 1 [Source:HGNC Symbol;Acc:HGNC:7030]
ENSG00000162804	70.8635605	79.7410856	26.3394616	30.8720472	1.397149221	0.0032146	0.0148496	SNED1	sushi, nidogen and EGF like domains 1 [Source:HGNC Symbol;Acc:HGNC:24696]
ENSG00000176092	151.569282	106.321447	46.3574523	71.3250745	1.13075855	0.0032227	0.0148829	CRYBG2	crystallin beta-gamma domain containing 2 [Source:HGNC Symbol;Acc:HGNC:17295]
ENSG00000196843	306.091213	305.674161	174.894025	207.587903	0.677712691	0.0032341	0.0149315	ARID5A	AT-rich interaction domain 5A [Source:HGNC Symbol;Acc:HGNC:17361]
ENSG00000273018	167.31674	165.684256	93.7684831	85.164268	0.895968861	0.0032404	0.0149568	FAM106A	family with sequence similarity 106 member A [Source:NCBI gene;Acc:80039]
ENSG00000112182	28.5422674	26.5803619	1.05357846	6.3873201	2.891416044	0.0032483	0.014989	BACH2	BTB domain and CNC homolog 2 [Source:HGNC Symbol;Acc:HGNC:14078]
ENSG00000103522	39.3686447	29.238398	3.16073539	9.58098015	2.429189938	0.0032568	0.0150239	IL21R	interleukin 21 receptor [Source:HGNC Symbol;Acc:HGNC:6006]
ENSG00000108381	23.6211868	40.7565548	5.26789231	6.3873201	2.468131097	0.0032605	0.0150369	ASPA	aspartoacylase [Source:HGNC Symbol;Acc:HGNC:756]
ENSG00000187049	189.953711	177.202412	277.091136	311.914132	-0.682327874	0.0032619	0.0150396	TMEM216	transmembrane protein 216 [Source:HGNC Symbol;Acc:HGNC:25018]
ENSG00000164823	420.260282	583.881949	342.413	314.043238	0.614277007	0.0032713	0.0150789	OSGIN2	oxidative stress induced growth inhibitor family member 2 [Source:HGNC Symbol;Acc:HGNC:1355]
ENSG00000163930	2189.88086	1857.96729	2558.08851	2641.15686	-0.361534088	0.0032836	0.0151314	BAP1	BRCA1 associated protein 1 [Source:HGNC Symbol;Acc:HGNC:950]
ENSG00000100220	2079.64866	2005.0453	2499.08811	2643.28597	-0.332295232	0.0033047	0.0152242	RTCB	RNA 2',3'-cyclic phosphate and 5'-OH ligase [Source:HGNC Symbol;Acc:HGNC:26935]
ENSG00000079387	618.087722	749.566204	936.631253	941.065162	-0.456409835	0.0033078	0.0152345	SENP1	SUMO specific peptidase 1 [Source:HGNC Symbol;Acc:HGNC:17927]
ENSG00000149564	136.80604	137.33187	57.9468154	77.7123946	1.015208272	0.0033133	0.0152558	ESAM	endothelial cell adhesion molecule [Source:HGNC Symbol;Acc:HGNC:17474]
ENSG00000088836	236.211868	221.503015	115.893631	149.037469	0.788731664	0.0033169	0.015268	SLC4A11	solute carrier family 4 member 11 [Source:HGNC Symbol;Acc:HGNC:16438]
ENSG00000110900	38.3844286	23.0363136	5.26789231	5.32276675	2.533831495	0.003319	0.0152702	TSPAN11	tetraspanin 11 [Source:HGNC Symbol;Acc:HGNC:30795]
ENSG00000176410	152.553498	130.243773	231.787262	244.847271	-0.754292587	0.0033193	0.0152702	DNAJC30	DnaJ heat shock protein family (Hsp40) member C30 [Source:HGNC Symbol;Acc:HGNC:16410]
ENSG00000165233	790.325543	593.628081	450.931582	498.210968	0.543237981	0.0033201	0.0152702	CARD19	caspase recruitment domain family member 19 [Source:HGNC Symbol;Acc:HGNC:28148]
ENSG00000127589	166.332524	157.710147	253.912409	275.719318	-0.709163074	0.0033231	0.0152783	TUBBP1	tubulin beta pseudogene 1 [Source:HGNC Symbol;Acc:HGNC:12414]
ENSG00000140319	3657.3471	4049.07512	4826.44294	4746.84339	-0.312784598	0.0033236	0.0152783	SRP14	signal recognition particle 14 [Source:HGNC Symbol;Acc:HGNC:11299]
ENSG00000013523	1001.93201	868.291821	690.093893	684.507804	0.443687188	0.0033323	0.0153139	ANGEL1	angel homolog 1 [Source:HGNC Symbol;Acc:HGNC:19961]
ENSG00000115194	193.890575	196.694678	85.3398554	126.681849	0.881805816	0.0033367	0.0153298	SLC30A3	solute carrier family 30 member 3 [Source:HGNC Symbol;Acc:HGNC:11014]
ENSG00000175463	32.4791319	15.0622051	4.21431385	0	3.489253259	0.0033379	0.0153313	TBC1D10C	TBC1 domain family member 10C [Source:HGNC Symbol;Acc:HGNC:24702]
ENSG00000283041	2439.87176	2286.79713	3017.44872	2905.16609	-0.325582767	0.0033392	0.0153329	AC008038.1	eukaryotic translation elongation factor 1 gamma (
ENSG00000132017	769.657004	604.260226	940.845567	963.420782	-0.471921381	0.0033571	0.0154112	DCAF15	DDB1 and CUL4 associated factor 15 [Source:HGNC Symbol;Acc:HGNC:25095]
ENSG00000106266	674.188041	601.60219	859.720025	876.127407	-0.44480688	0.003361	0.0154249	SNX8	sorting nexin 8 [Source:HGNC Symbol;Acc:HGNC:14972]
ENSG00000164684	175.190469	185.176521	91.6613262	106.455335	0.863530575	0.0033623	0.0154264	ZNF704	zinc finger protein 704 [Source:HGNC Symbol;Acc:HGNC:32291]
ENSG00000025772	1675.13583	1588.61963	2018.65633	2133.36491	-0.347435006	0.0033719	0.0154664	TOMM34	translocase of outer mitochondrial membrane 34 [Source:HGNC Symbol;Acc:HGNC:15746]
ENSG00000022277	1674.15162	1705.57322	2004.95981	2364.37299	-0.370417684	0.003375	0.0154765	RTF2	replication termination factor 2 [Source:HGNC Symbol;Acc:HGNC:15890]
ENSG00000095564	1685.96221	1896.95182	1466.58122	1262.56027	0.392910317	0.0033765	0.0154789	BTAF1	B-TFIID TATA-box binding protein associated factor 1 [Source:HGNC Symbol;Acc:HGNC:17307]
ENSG00000123700	19.6843224	17.7202412	0	1.06455335	5.141198296	0.0033796	0.0154867	KCNJ2	potassium voltage-gated channel subfamily J member 2 [Source:HGNC Symbol;Acc:HGNC:6263]
ENSG00000182752	252.943542	191.378605	114.840052	136.262829	0.822222783	0.00338	0.0154867	PAPPA	pappalysin 1 [Source:HGNC Symbol;Acc:HGNC:8602]
ENSG00000177732	2228.26529	1963.40273	2537.01694	2840.22834	-0.359614042	0.0033867	0.0155131	SOX12	SRY-box 12 [Source:HGNC Symbol;Acc:HGNC:11198]
ENSG00000071537	1820.79982	2138.83312	1676.24333	1150.78217	0.486222991	0.0034163	0.0156448	SEL1L	SEL1L, ERAD E3 ligase adaptor subunit [Source:HGNC Symbol;Acc:HGNC:10717]
ENSG00000167658	45969.7822	39145.7849	52593.5833	52495.2548	-0.304140357	0.0034181	0.0156485	EEF2	eukaryotic translation elongation factor 2 [Source:HGNC Symbol;Acc:HGNC:3214]
ENSG00000123975	1128.89589	1249.27701	1532.95666	1554.24789	-0.376065346	0.0034213	0.0156589	CKS2	CDC28 protein kinase regulatory subunit 2 [Source:HGNC Symbol;Acc:HGNC:2000]
ENSG00000148399	534.429352	467.814369	659.540117	750.510112	-0.493139542	0.0034284	0.0156872	DPH7	diphthamide biosynthesis 7 [Source:HGNC Symbol;Acc:HGNC:25199]
ENSG00000150540	160.427227	197.58069	301.32344	279.977531	-0.697873439	0.0034338	0.0157078	HNMT	histamine N-methyltransferase [Source:HGNC Symbol;Acc:HGNC:5028]

ENSG00000129219	477.344817	441.234007	617.396979	672.797717	-0.490516184	0.0034521	0.0157873	PLD2	phospholipase D2 [Source:HGNC Symbol;Acc:HGNC:9068]
ENSG00000172466	617.103506	685.773336	871.309388	896.353921	-0.439609494	0.0034615	0.0158259	ZNF24	zinc finger protein 24 [Source:HGNC Symbol;Acc:HGNC:13032]
ENSG00000234743	141.727121	106.321447	222.305056	211.846117	-0.809738612	0.0034637	0.0158276	EIF5AP4	eukaryotic translation initiation factor 5A pseudogene 4 [Source:HGNC Symbol;Acc:HGNC:31442]
ENSG00000068305	1184.01199	1274.08534	991.417333	850.578127	0.416427326	0.0034638	0.0158276	MEF2A	myocyte enhancer factor 2A [Source:HGNC Symbol;Acc:HGNC:6993]
ENSG00000108651	1103.30627	1091.56686	1350.68759	1518.05308	-0.386282319	0.0034674	0.0158372	UTP6	UTP6, small subunit processome component [Source:HGNC Symbol;Acc:HGNC:18279]
ENSG00000280206	140.742905	156.824135	209.662114	305.526812	-0.791010515	0.0034677	0.0158372	AC026401.3	novel transcript
ENSG00000127463	2153.46487	1990.8691	2574.94576	2644.35052	-0.332923027	0.0034694	0.0158375	EMC1	ER membrane protein complex subunit 1 [Source:HGNC Symbol;Acc:HGNC:28957]
ENSG00000203778	254.911975	246.311353	166.465397	132.004615	0.747519249	0.0034697	0.0158375	FAM229B	family with sequence similarity 229 member B [Source:HGNC Symbol;Acc:HGNC:33858]
ENSG00000163961	999.963576	836.395386	692.20105	642.990224	0.459171532	0.0034715	0.0158416	RNF168	ring finger protein 168 [Source:HGNC Symbol;Acc:HGNC:26661]
ENSG00000149926	37.4002125	27.4663739	3.16073539	8.5164268	2.474013314	0.0034729	0.0158437	FAM57B	family with sequence similarity 57 member B [Source:HGNC Symbol;Acc:HGNC:25295]
ENSG00000068971	548.208378	424.399778	313.966382	333.205199	0.586754371	0.0034862	0.0158977	PPP2R5B	protein phosphatase 2 regulatory subunit B'beta [Source:HGNC Symbol;Acc:HGNC:9310]
ENSG00000124172	5148.43451	5075.07709	6217.16651	6251.05727	-0.286385943	0.0034866	0.0158977	ATP5F1E	ATP synthase F1 subunit epsilon [Source:HGNC Symbol;Acc:HGNC:838]
ENSG00000134668	115.153286	126.699725	204.394222	213.975223	-0.789855276	0.0034903	0.0159103	SPOCD1	SPOC domain containing 1 [Source:HGNC Symbol;Acc:HGNC:26338]
ENSG00000135363	59.0529671	49.6166755	16.8572554	17.0328536	1.680194138	0.0034982	0.0159406	LMO2	LIM domain only 2 [Source:HGNC Symbol;Acc:HGNC:6642]
ENSG00000129667	780.483382	678.685239	490.967563	550.374082	0.486242797	0.0034989	0.0159406	RHBDF2	rhomboid 5 homolog 2 [Source:HGNC Symbol;Acc:HGNC:20788]
ENSG00000123144	3434.91425	3000.03684	3801.31109	4427.47738	-0.354939312	0.0035092	0.0159834	TRIR	telomerase RNA component interacting RNase [Source:HGNC Symbol;Acc:HGNC:28424]
ENSG00000169855	1437.93975	1386.60888	1144.18621	1011.32568	0.389829428	0.0035107	0.0159859	ROBO1	roundabout guidance receptor 1 [Source:HGNC Symbol;Acc:HGNC:10249]
ENSG00000198873	1020.63211	958.665051	773.326591	699.411551	0.426211783	0.0035272	0.0160566	GRK5	G protein-coupled receptor kinase 5 [Source:HGNC Symbol;Acc:HGNC:4544]
ENSG00000157326	216.527546	159.482171	278.144714	341.721625	-0.722987106	0.0035299	0.0160648	DHRS4	dehydrogenase/reductase 4 [Source:HGNC Symbol;Acc:HGNC:16985]
ENSG00000155324	566.908484	594.514093	379.288246	434.337767	0.51374179	0.0035375	0.0160951	GRAMD2B	GRAM domain containing 2B [Source:HGNC Symbol;Acc:HGNC:24911]
ENSG00000168569	387.781151	367.695006	483.592514	630.215583	-0.560212226	0.0035424	0.0161057	TMEM223	transmembrane protein 223 [Source:HGNC Symbol;Acc:HGNC:28464]
ENSG00000109171	844.457429	799.18288	619.504136	588.698003	0.443783911	0.0035425	0.0161057	SLAIN2	SLAIN motif family member 2 [Source:HGNC Symbol;Acc:HGNC:29282]
ENSG00000003393	707.651389	702.607565	516.253447	506.727395	0.463138713	0.0035427	0.0161057	ALS2	ALS2, alsin Rho guanine nucleotide exchange factor [Source:HGNC Symbol;Acc:HGNC:443]
ENSG00000035687	907.447261	1098.65496	1627.77872	1188.04154	-0.488730532	0.0035446	0.01611	ADSS	adenylosuccinate synthase [Source:HGNC Symbol;Acc:HGNC:292]
ENSG00000275993	1170.23296	1153.5877	1419.17019	1611.73377	-0.383261519	0.0035465	0.0161144	SIK1B	salt inducible kinase 1B (putative) [Source:HGNC Symbol;Acc:HGNC:52389]
ENSG00000228232	331.680832	314.534282	445.66369	501.404628	-0.551727544	0.0035606	0.016174	GAPDHP1	glyceraldehyde-3-phosphate dehydrogenase pseudogene 1 [Source:HGNC Symbol;Acc:HGNC:4159]
ENSG00000161513	430.102444	363.264945	531.003545	620.634603	-0.538463694	0.0035727	0.0162244	FDXR	ferredoxin reductase [Source:HGNC Symbol;Acc:HGNC:3642]
ENSG00000145349	290.343755	344.658692	203.340643	198.006923	0.662694406	0.0035759	0.0162345	CAMK2D	calcium/calmodulin dependent protein kinase II delta [Source:HGNC Symbol;Acc:HGNC:1462]
ENSG00000169554	236.211868	228.591112	350.841628	358.754479	-0.61059788	0.0035848	0.0162708	ZEB2	zinc finger E-box binding homeobox 2 [Source:HGNC Symbol;Acc:HGNC:14881]
ENSG00000154188	20.6685385	36.3264945	2.10715692	6.3873201	2.750116547	0.0035898	0.0162892	ANGPT1	angiopoietin 1 [Source:HGNC Symbol;Acc:HGNC:484]
ENSG00000161395	145.663985	125.813713	215.983585	244.847271	-0.764451367	0.0035945	0.0163058	PGAP3	post-GPI attachment to proteins 3 [Source:HGNC Symbol;Acc:HGNC:23719]
ENSG00000125848	26.5738352	38.0985187	3.16073539	8.5164268	2.472371602	0.0036015	0.0163336	FLRT3	fibronectin leucine rich transmembrane protein 3 [Source:HGNC Symbol;Acc:HGNC:3762]
ENSG00000179057	13.7790257	7.97410856	0	0	5.80121628	0.0036029	1	IGSF22	immunoglobulin superfamily member 22 [Source:HGNC Symbol;Acc:HGNC:26750]
ENSG00000198355	3656.36288	3167.49312	4156.36703	4437.05836	-0.332872849	0.0036053	0.0163464	PIM3	Pim-3 proto-oncogene, serine/threonine kinase [Source:HGNC Symbol;Acc:HGNC:19310]
ENSG00000048828	4377.79329	4093.37573	5672.46644	4938.46299	-0.325028909	0.003613	0.0163768	FAM120A	family with sequence similarity 120A [Source:HGNC Symbol;Acc:HGNC:13247]
ENSG00000167699	1111.18	1052.58233	1308.54445	1555.31244	-0.404555514	0.0036156	0.0163843	GLOD4	glyoxalase domain containing 4 [Source:HGNC Symbol;Acc:HGNC:14111]
ENSG00000115514	227.353923	281.751836	374.020354	407.723933	-0.617347419	0.0036202	0.0164007	TXNDC9	thioredoxin domain containing 9 [Source:HGNC Symbol;Acc:HGNC:24110]
ENSG00000172986	280.501594	324.280415	192.804859	187.36139	0.670459915	0.0036304	0.0164423	GXYLT2	glucoside xylosyltransferase 2 [Source:HGNC Symbol;Acc:HGNC:33383]
ENSG00000173456	1339.51814	1204.9764	1670.97544	1617.05654	-0.37023154	0.0036361	0.0164636	RNF26	ring finger protein 26 [Source:HGNC Symbol;Acc:HGNC:14646]
ENSG00000140199	642.693125	660.964998	463.574523	473.726241	0.47611741	0.0036379	0.0164676	SLC12A6	solute carrier family 12 member 6 [Source:HGNC Symbol;Acc:HGNC:10914]
ENSG00000105483	170.269388	181.632473	267.608929	298.074938	-0.684290406	0.0036423	0.0164819	CARD8	caspase recruitment domain family member 8 [Source:HGNC Symbol;Acc:HGNC:17057]
ENSG00000115318	25.5896191	23.0363136	53.7325016	83.0351613	-1.492822741	0.003643	0.0164819	LOXL3	lysyl oxidase like 3 [Source:HGNC Symbol;Acc:HGNC:13869]
ENSG00000112651	564.940052	411.109597	697.468942	716.444405	-0.536042779	0.0036466	0.0164938	MRPL2	mitochondrial ribosomal protein L2 [Source:HGNC Symbol;Acc:HGNC:14056]
ENSG00000165113	98.4216118	129.357761	57.9468154	46.8403474	1.121255737	0.0036566	0.0165345	GKAP1	G kinase anchoring protein 1 [Source:HGNC Symbol;Acc:HGNC:17496]
ENSG00000146232	386.796934	297.700053	194.912016	232.07263	0.679906465	0.0036576	0.0165347	NFKBIE	NFKB inhibitor epsilon [Source:HGNC Symbol;Acc:HGNC:7799]
ENSG00000100949	278.533161	303.016125	465.68168	402.401166	-0.57747355	0.0036634	0.0165565	RABGGTA	Rab geranylgeranyltransferase subunit alpha [Source:HGNC Symbol;Acc:HGNC:9795]
ENSG00000141639	10.8263773	10.6321447	0	0	5.783311483	0.003671	1	MAPK4	mitogen-activated protein kinase 4 [Source:HGNC Symbol;Acc:HGNC:6878]
ENSG00000008300	2736.12081	2287.68314	3419.91569	3051.0099	-0.365540306	0.0036792	0.0166232	CELSR3	cadherin EGF LAG seven-pass G-type receptor 3 [Source:HGNC Symbol;Acc:HGNC:3230]
ENSG00000067208	530.492488	522.747116	396.145502	327.882432	0.540534943	0.0036813	0.0166284	EVI5	ecotropic viral integration site 5 [Source:HGNC Symbol;Acc:HGNC:3501]
ENSG00000198331	139.758689	138.217882	212.822849	256.557357	-0.755777904	0.0036997	0.0167072	HYLS1	HYLS1, centriolar and ciliogenesis associated [Source:HGNC Symbol;Acc:HGNC:26558]
ENSG00000085117	824.773107	768.172458	548.914379	612.118176	0.456075398	0.0037061	0.0167315	CD82	CD82 molecule [Source:HGNC Symbol;Acc:HGNC:6210]
ENSG00000118640	920.242071	853.229616	1105.20381	1251.91474	-0.410715549	0.003708	0.0167337	VAMP8	vesicle associated membrane protein 8 [Source:HGNC Symbol;Acc:HGNC:12647]
ENSG00000238105	314.949158	315.420294	173.840446	221.427097	0.673552798	0.0037086	0.0167337	GOLGA2P5	GOLGA2 pseudogene 5 [Source:HGNC Symbol;Acc:HGNC:25315]

ENSG00000124766	1407.42905	1568.24135	1959.65594	1860.83926	-0.360213853	0.0037107	0.0167386	SOX4	SRY-box 4 [Source:HGNC Symbol;Acc:HGNC:11200]
ENSG00000225178	498.013356	461.612284	608.968351	755.832879	-0.508436247	0.0037162	0.016759	RPSAP58	ribosomal protein SA pseudogene 58 [Source:HGNC Symbol;Acc:HGNC:36809]
ENSG00000143811	612.182426	481.990562	736.451345	807.995993	-0.498290565	0.0037317	0.0168246	PYCR2	pyrroline-5-carboxylate reductase 2 [Source:HGNC Symbol;Acc:HGNC:30262]
ENSG00000176422	235.227652	244.539329	321.341431	430.079554	-0.646923084	0.0037428	0.01687	SPRYD4	SPRY domain containing 4 [Source:HGNC Symbol;Acc:HGNC:27468]
ENSG00000117419	1300.14949	1266.99725	1594.06421	1701.15625	-0.360283625	0.0037464	0.0168818	ERI3	ERI1 exoribonuclease family member 3 [Source:HGNC Symbol;Acc:HGNC:17276]
ENSG00000012048	1123.97481	1406.10114	1835.33368	1567.02253	-0.426793545	0.0037568	0.016924	BRCA1	BRCA1, DNA repair associated [Source:HGNC Symbol;Acc:HGNC:1100]
ENSG00000083454	190.937927	122.269665	261.287459	276.783871	-0.783405162	0.0037615	0.0169406	P2RX5	purinergic receptor P2X 5 [Source:HGNC Symbol;Acc:HGNC:8536]
ENSG00000161243	201.764304	193.150629	286.573342	337.463412	-0.660309462	0.0037648	0.0169481	FBXO27	F-box protein 27 [Source:HGNC Symbol;Acc:HGNC:18753]
ENSG00000173039	1830.64198	1547.86307	2138.76428	2219.59374	-0.367842468	0.0037651	0.0169481	RELA	RELA proto-oncogene, NF-kB subunit [Source:HGNC Symbol;Acc:HGNC:9955]
ENSG00000183722	182.079982	170.114316	84.286277	107.519888	0.876603057	0.0037685	0.0169588	LHFPL6	LHFPL tetraspan subfamily member 6 [Source:HGNC Symbol;Acc:HGNC:6586]
ENSG00000261594	71.8477766	46.9586393	25.2858831	9.58098015	1.766064039	0.0037742	0.0169797	TPBGL	trophoblast glycoprotein like [Source:HGNC Symbol;Acc:HGNC:44159]
ENSG00000175048	475.376385	466.042345	313.966382	333.205199	0.540637271	0.0037758	0.0169825	ZDHHC14	zinc finger DHHC-type containing 14 [Source:HGNC Symbol;Acc:HGNC:20341]
ENSG00000087263	952.721202	987.903449	1196.86513	1360.49918	-0.397947903	0.0037804	0.0169989	OGFOD1	2-oxoglutarate and iron dependent oxygenase domain containing 1 [Source:HGNC Symbol;Acc:HGNC:25585]
ENSG00000161956	579.703294	476.674489	656.379382	877.191961	-0.538457815	0.0037841	0.0170109	SENP3	SUMO specific peptidase 3 [Source:HGNC Symbol;Acc:HGNC:17862]
ENSG00000189343	2304.04993	2380.71441	2912.09087	2916.87618	-0.315176879	0.0038053	0.0171017	RPS2P46	ribosomal protein S2 pseudogene 46 [Source:HGNC Symbol;Acc:HGNC:35839]
ENSG00000258101	1.96843224	2.65803619	13.69652	29.8074938	-3.226985709	0.0038068	0.0171038	AC010173.1	novel transcript, antisense to TUBA1C
ENSG00000180098	686.982851	716.783758	896.595271	990.034616	-0.426264802	0.0038123	0.0171239	TRNAU1AP	tRNA selenocysteine 1 associated protein 1 [Source:HGNC Symbol;Acc:HGNC:30813]
ENSG00000132334	432.070876	349.974764	271.823243	235.26629	0.624028704	0.0038216	0.017161	PTPRE	protein tyrosine phosphatase, receptor type E [Source:HGNC Symbol;Acc:HGNC:9669]
ENSG00000115896	68.8951283	101.005375	41.08956	24.4847271	1.37458185	0.0038236	0.0171655	PLCL1	phospholipase C like 1 (inactive) [Source:HGNC Symbol;Acc:HGNC:9063]
ENSG00000062485	4703.56883	4741.05054	5504.94747	6170.15122	-0.305844789	0.0038312	0.0171937	CS	citrate synthase [Source:HGNC Symbol;Acc:HGNC:2422]
ENSG00000114547	18.7001062	18.6062533	0	2.1291067	4.136202786	0.0038319	0.0171937	ROPN1B	rhophilin associated tail protein 1B [Source:HGNC Symbol;Acc:HGNC:31927]
ENSG00000213024	1904.45819	1678.10685	2188.28247	2405.89057	-0.359126144	0.0038331	0.0171944	NUP62	nucleoporin 62 [Source:HGNC Symbol;Acc:HGNC:8066]
ENSG00000106392	457.660495	575.90784	388.770453	290.623065	0.605925803	0.0038375	0.0172099	C1GALT1	core 1 synthase, glycoprotein-N-acetylgalactosamine 3-beta-galactosyltransferase 1 [Source:HGNC Symbol;Acc:HGNC:24337]
ENSG00000239213	165.348308	161.254195	91.6613262	85.164268	0.885010335	0.0038506	0.017264	NCK1-DT	NCK1 divergent transcript [Source:HGNC Symbol;Acc:HGNC:49645]
ENSG00000149089	191.922143	235.679208	319.234274	352.367159	-0.649931705	0.0038657	0.0173269	APIP	APAF1 interacting protein [Source:HGNC Symbol;Acc:HGNC:17581]
ENSG00000107020	177.158901	212.642895	102.197111	117.100869	0.830850026	0.0038691	0.0173375	PLGRKT	plasminogen receptor with a C-terminal lysine [Source:HGNC Symbol;Acc:HGNC:23633]
ENSG00000126012	6430.86812	5451.63222	7476.19277	7350.74088	-0.319550364	0.0038706	0.0173397	KDM5C	lysine demethylase 5C [Source:HGNC Symbol;Acc:HGNC:11114]
ENSG00000168143	49.2108059	61.1348323	158.036769	88.3579281	-1.157488655	0.003883	0.0173906	FAM83B	family with sequence similarity 83 member B [Source:HGNC Symbol;Acc:HGNC:21357]
ENSG00000135919	4265.59266	4700.29399	3549.50584	3682.29004	0.310238565	0.0038871	0.0174042	SERPINE2	serpin family E member 2 [Source:HGNC Symbol;Acc:HGNC:8951]
ENSG00000196715	1162.35924	1259.90915	945.059881	899.547581	0.393291165	0.0039004	0.0174562	VKORC1L1	vitamin K epoxide reductase complex subunit 1 like 1 [Source:HGNC Symbol;Acc:HGNC:21492]
ENSG00000176248	1840.48414	1430.90948	2189.33604	2126.97759	-0.400484061	0.0039007	0.0174562	ANAPC2	anaphase promoting complex subunit 2 [Source:HGNC Symbol;Acc:HGNC:19989]
ENSG00000230183	72.8319927	68.2229288	24.2323046	28.7429405	1.412741243	0.0039023	0.0174585	CNOT6LP1	CCR4-NOT transcription complex subunit 6-like pseudogene 1 [Source:HGNC Symbol;Acc:HGNC:32355]
ENSG00000112576	2427.07695	2066.18013	1649.90387	1821.45078	0.371969356	0.0039037	0.0174602	CCND3	cyclin D3 [Source:HGNC Symbol;Acc:HGNC:1585]
ENSG00000223764	698.793444	505.026875	385.609717	424.756787	0.569981659	0.0039121	0.0174931	LINC02593	long intergenic non-protein coding RNA 2593 [Source:HGNC Symbol;Acc:HGNC:53933]
ENSG00000178952	5283.27212	4627.641	5968.52199	6363.89993	-0.31552347	0.0039178	0.0175141	TUFM	Tu translation elongation factor, mitochondrial [Source:HGNC Symbol;Acc:HGNC:12420]
ENSG00000133706	2106.22249	2452.48139	2925.78739	2862.58396	-0.34419439	0.0039195	0.0175171	LARS	leucyl-tRNA synthetase [Source:HGNC Symbol;Acc:HGNC:6512]
ENSG00000164509	18.7001062	26.5803619	0	4.2582134	3.415919631	0.0039263	0.0175426	IL31RA	interleukin 31 receptor A [Source:HGNC Symbol;Acc:HGNC:18969]
ENSG00000126746	741.114737	643.244757	970.345764	903.805794	-0.437694029	0.0039319	0.0175633	ZNF384	zinc finger protein 384 [Source:HGNC Symbol;Acc:HGNC:11955]
ENSG00000196814	311.996509	258.715522	420.377806	432.20866	-0.580238353	0.0039368	0.0175804	MVB12B	multivesicular body subunit 12B [Source:HGNC Symbol;Acc:HGNC:23368]
ENSG00000100911	1859.18425	1631.14821	2082.92462	2454.86003	-0.378907232	0.003956	0.0176616	PSME2	proteasome activator subunit 2 [Source:HGNC Symbol;Acc:HGNC:9569]
ENSG00000166734	1023.58476	1092.45287	804.933945	795.221353	0.403383936	0.0039574	0.0176632	CASC4	cancer susceptibility 4 [Source:HGNC Symbol;Acc:HGNC:24892]
ENSG00000163714	2515.6564	3458.10508	4360.76126	3670.57995	-0.426663345	0.0039608	0.0176737	U2SURP	U2 snRNP associated SURP domain containing [Source:HGNC Symbol;Acc:HGNC:30855]
ENSG00000012660	2537.30915	3023.07315	2331.56914	1897.03407	0.395208752	0.0039862	0.0177823	ELOVL5	ELOVL fatty acid elongase 5 [Source:HGNC Symbol;Acc:HGNC:21308]
ENSG00000148481	249.990894	340.228632	185.429809	174.586749	0.714443749	0.0039877	0.017784	MINDY3	MINDY lysine 48 deubiquitinase 3 [Source:HGNC Symbol;Acc:HGNC:23578]
ENSG00000073803	450.770982	576.793852	338.198686	357.689926	0.563284331	0.0039937	0.0178063	MAP3K13	mitogen-activated protein kinase kinase kinase 13 [Source:HGNC Symbol;Acc:HGNC:6852]
ENSG00000162129	854.299591	693.747444	1065.16783	1033.6813	-0.439998016	0.0040003	0.017831	CLPB	ClpB homolog, mitochondrial AAA ATPase chaperonin [Source:HGNC Symbol;Acc:HGNC:30664]
ENSG00000102977	730.28836	720.327806	994.578068	936.806948	-0.413062174	0.0040032	0.0178391	ACD	ACD, shelterin complex subunit and telomerase recruitment factor [Source:HGNC Symbol;Acc:HGNC:25070]
ENSG00000137310	2720.37335	2609.30552	2117.69271	2151.46232	0.32000845	0.0040044	0.01784	TCF19	transcription factor 19 [Source:HGNC Symbol;Acc:HGNC:11629]
ENSG00000169217	1811.94187	1624.94612	2177.74668	2186.59258	-0.344982935	0.0040079	0.0178491	CD2BP2	CD2 cytoplasmic tail binding protein 2 [Source:HGNC Symbol;Acc:HGNC:1656]
ENSG00000185100	824.773107	723.871854	1082.02508	994.292829	-0.423619838	0.0040096	0.0178491	ADSSL1	adenylosuccinate synthase like 1 [Source:HGNC Symbol;Acc:HGNC:20093]

ENSG00000138674	2104.25406	2226.54831	2601.28522	2848.74477	-0.331464756	0.0040096	0.0178491	SEC31A	SEC31 homolog A, COPII coat complex component [Source:HGNC Symbol;Acc:HGNC:17052]
ENSG00000148926	1661.35681	1460.14788	1928.04859	2086.52457	-0.363364766	0.0040138	0.017863	ADM	adrenomedullin [Source:HGNC Symbol;Acc:HGNC:259]
ENSG00000065150	2998.90651	3418.23453	4157.42061	3893.0716	-0.326924879	0.0040175	0.0178738	IPO5	importin 5 [Source:HGNC Symbol;Acc:HGNC:6402]
ENSG00000177352	434.039308	437.689959	561.55732	677.055931	-0.506647672	0.0040184	0.0178738	CCDC71	coiled-coil domain containing 71 [Source:HGNC Symbol;Acc:HGNC:25760]
ENSG00000115183	1478.29261	1406.10114	1129.43611	1106.07093	0.36749683	0.0040201	0.0178765	TANC1	tetratricopeptide repeat, ankyrin repeat and coiled-coil containing 1 [Source:HGNC Symbol;Acc:HGNC:29364]
ENSG00000215030	837.567917	669.825119	989.310176	1070.94067	-0.451614427	0.0040245	0.0178914	RPL13P12	ribosomal protein L13 pseudogene 12 [Source:HGNC Symbol;Acc:HGNC:35701]
ENSG00000084463	3236.1026	3244.57617	3883.49021	4103.85317	-0.301555096	0.0040273	0.0178994	WBP11	WW domain binding protein 11 [Source:HGNC Symbol;Acc:HGNC:16461]
ENSG00000166575	468.486872	598.944154	396.145502	317.236898	0.582123317	0.004039	0.0179468	TMEM135	transmembrane protein 135 [Source:HGNC Symbol;Acc:HGNC:26167]
ENSG00000076716	60.0371832	46.9586393	20.0179908	12.7746402	1.70444274	0.0040638	0.0180476	GPC4	glypican 4 [Source:HGNC Symbol;Acc:HGNC:4452]
ENSG00000176393	2375.89771	2292.99922	2791.98293	3054.20356	-0.324481951	0.0040639	0.0180476	RNPEP	arginyl aminopeptidase [Source:HGNC Symbol;Acc:HGNC:10078]
ENSG00000149150	53.1476704	50.5026875	10.5357846	20.2265137	1.753054909	0.0040807	0.0181177	SLC43A1	solute carrier family 43 member 1 [Source:HGNC Symbol;Acc:HGNC:9225]
ENSG00000107372	2570.7725	3059.39965	2298.9082	2079.07269	0.363176832	0.0040823	0.0181199	ZFAND5	zinc finger AN1-type containing 5 [Source:HGNC Symbol;Acc:HGNC:13008]
ENSG00000187554	14.7632418	35.4404825	4.21431385	2.1291067	2.986347185	0.0040934	0.0181644	TLR5	toll like receptor 5 [Source:HGNC Symbol;Acc:HGNC:11851]
ENSG00000114021	703.714525	755.768289	903.970321	1074.13433	-0.438293624	0.0041066	0.0182183	NIT2	nitrilase family member 2 [Source:HGNC Symbol;Acc:HGNC:29878]
ENSG00000214944	439.944605	500.596815	305.537754	336.398859	0.551692516	0.0041092	0.0182251	ARHGEF28	Rho guanine nucleotide exchange factor 28 [Source:HGNC Symbol;Acc:HGNC:30322]
ENSG00000055917	1156.45394	1167.7639	1657.27892	1391.37123	-0.391443401	0.0041127	0.0182358	PUM2	pumilio RNA binding family member 2 [Source:HGNC Symbol;Acc:HGNC:14958]
ENSG00000273274	52.1634543	31.8964342	10.5357846	10.6455335	1.986510843	0.004119	0.0182589	ZBTB8B	zinc finger and BTB domain containing 8B [Source:HGNC Symbol;Acc:HGNC:37057]
ENSG00000186814	167.31674	194.922654	83.2326985	113.907208	0.878688562	0.004124	0.0182763	ZSCAN30	zinc finger and SCAN domain containing 30 [Source:HGNC Symbol;Acc:HGNC:33517]
ENSG00000105983	951.736986	1044.60822	770.165856	726.025385	0.416370279	0.0041263	0.0182786	LMBR1	limb development membrane protein 1 [Source:HGNC Symbol;Acc:HGNC:13243]
ENSG00000103254	461.597359	357.948873	572.093105	614.247283	-0.53488312	0.0041267	0.0182786	FAM173A	family with sequence similarity 173 member A [Source:HGNC Symbol;Acc:HGNC:14152]
ENSG00000224032	165.348308	124.041689	207.554957	297.010385	-0.803674761	0.0041411	0.0183375	EPB41L4A-AS1	EPB41L4A antisense RNA 1 [Source:HGNC Symbol;Acc:HGNC:30749]
ENSG00000125686	1682.02535	1825.18485	2271.51516	2165.30151	-0.338969593	0.0041552	0.0183952	MED1	mediator complex subunit 1 [Source:HGNC Symbol;Acc:HGNC:9234]
ENSG00000186522	1493.05585	1737.46965	1331.72318	1091.16718	0.415309873	0.0041585	0.0184052	10-Sep	septin 10 [Source:HGNC Symbol;Acc:HGNC:14349]
ENSG00000091622	448.80255	425.28579	596.32541	624.892817	-0.482762783	0.0041616	0.0184128	PITPNM3	PITPNM family member 3 [Source:HGNC Symbol;Acc:HGNC:21043]
ENSG00000180370	1799.14706	2083.90037	2535.96336	2415.47155	-0.350297162	0.0041624	0.0184128	PAK2	p21 (RAC1) activated kinase 2 [Source:HGNC Symbol;Acc:HGNC:8591]
ENSG00000107554	679.109122	628.182552	477.271043	464.145261	0.473326308	0.0041647	0.0184182	DNMBP	dynamin binding protein [Source:HGNC Symbol;Acc:HGNC:30373]
ENSG00000155760	475.376385	444.778055	310.805646	321.495112	0.540977777	0.0041709	0.0184405	FZD7	frizzled class receptor 7 [Source:HGNC Symbol;Acc:HGNC:4045]
ENSG00000213866	205.701169	207.326822	291.841234	355.560819	-0.648268719	0.004183	0.0184891	YBX1P10	Y-box binding protein 1 pseudogene 10 [Source:HGNC Symbol;Acc:HGNC:42432]
ENSG00000127863	39.3686447	42.528579	13.69652	6.3873201	2.027095663	0.0041927	0.0185272	TNFRSF19	TNF receptor superfamily member 19 [Source:HGNC Symbol;Acc:HGNC:11915]
ENSG00000253981	74.800425	45.1866152	21.0715692	18.097407	1.612723364	0.0042013	0.0185606	ALG1L13P	asparagine-linked glycosylation 1-like 13, pseudogene [Source:HGNC Symbol;Acc:HGNC:44382]
ENSG00000139354	518.681894	586.539985	404.57413	368.335459	0.516425306	0.0042049	0.0185715	GAS2L3	growth arrest specific 2 like 3 [Source:HGNC Symbol;Acc:HGNC:27475]
ENSG00000111860	92.5163151	110.751508	48.4646093	43.6466874	1.14271962	0.0042143	0.018608	CEP85L	centrosomal protein 85 like [Source:HGNC Symbol;Acc:HGNC:21638]
ENSG00000137404	691.903931	638.814697	841.809191	961.291675	-0.438595193	0.0042168	0.0186144	NRM	nurim [Source:HGNC Symbol;Acc:HGNC:8003]
ENSG00000160111	45.2739414	27.4663739	6.32147077	9.58098015	2.192018393	0.0042228	0.0186357	CPAMD8	C3 and PZP like, alpha-2-macroglobulin domain containing 8 [Source:HGNC Symbol;Acc:HGNC:23228]
ENSG00000132600	1137.75383	1067.64453	1370.70558	1488.24558	-0.374665135	0.0042358	0.0186849	PRMT7	protein arginine methyltransferase 7 [Source:HGNC Symbol;Acc:HGNC:25557]
ENSG00000253626	355.302019	320.736366	489.913985	485.436328	-0.529459656	0.0042361	0.0186849	EIF5AL1	eukaryotic translation initiation factor 5A-like 1 [Source:HGNC Symbol;Acc:HGNC:17419]
ENSG00000101457	933.03688	825.763242	1136.81116	1184.84788	-0.401065307	0.0042538	0.0187579	DNTTIP1	deoxynucleotidyltransferase terminal interacting protein 1 [Source:HGNC Symbol;Acc:HGNC:16160]
ENSG00000094880	884.81029	956.007015	1295.90151	1138.00753	-0.402663729	0.0042565	0.018765	CDC23	cell division cycle 23 [Source:HGNC Symbol;Acc:HGNC:1724]
ENSG00000187713	1067.87449	998.535594	1286.4193	1405.21042	-0.381589623	0.0042755	0.018844	TMEM203	transmembrane protein 203 [Source:HGNC Symbol;Acc:HGNC:28217]
ENSG00000162591	260.817271	243.653317	361.377413	397.0784	-0.588704035	0.0042831	0.0188726	MEGF6	multiple EGF like domains 6 [Source:HGNC Symbol;Acc:HGNC:3232]
ENSG00000213281	727.335711	909.048375	1314.86592	987.905509	-0.492260831	0.0042855	0.0188782	NRAS	NRAS proto-oncogene, GTPase [Source:HGNC Symbol;Acc:HGNC:7989]
ENSG00000079805	3444.75641	2864.477	3955.13355	4029.33443	-0.340031559	0.0042916	0.0189001	DNM2	dynamin 2 [Source:HGNC Symbol;Acc:HGNC:2974]
ENSG00000115339	29.5264835	36.3264945	9.48220616	3.19366005	2.376464912	0.0043049	0.0189536	GALNT3	polypeptide N-acetylgalactosaminyltransferase 3 [Source:HGNC Symbol;Acc:HGNC:4125]
ENSG00000163938	1758.7942	2011.24738	2245.1757	2628.38222	-0.370053016	0.0043165	0.0189999	GNL3	G protein nucleolar 3 [Source:HGNC Symbol;Acc:HGNC:29931]
ENSG00000122687	809.025649	805.384964	1032.50689	1093.29629	-0.396998548	0.0043265	0.0190388	MRM2	mitochondrial rRNA methyltransferase 2 [Source:HGNC Symbol;Acc:HGNC:16352]
ENSG00000072110	8805.78161	7901.45557	10163.8714	10236.745	-0.288232547	0.0043417	0.0191009	ACTN1	actinin alpha 1 [Source:HGNC Symbol;Acc:HGNC:163]
ENSG00000100739	366.128396	353.518813	194.912016	265.073784	0.645759056	0.0043599	0.0191757	BDKRB1	bradykinin receptor B1 [Source:HGNC Symbol;Acc:HGNC:1029]
ENSG00000173674	2408.37684	2875.99515	3826.59698	3084.01106	-0.386841362	0.0043711	0.0192201	EIF1AX	eukaryotic translation initiation factor 1A X-linked [Source:HGNC Symbol;Acc:HGNC:3250]
ENSG00000174917	665.330096	577.679864	843.916348	843.126253	-0.441349365	0.0043785	0.0192477	C19orf70	chromosome 19 open reading frame 70 [Source:HGNC Symbol;Acc:HGNC:33702]
ENSG00000144645	1136.76962	1150.04366	906.077478	837.803487	0.391039091	0.0043952	0.0193159	OSBPL10	oxysterol binding protein like 10 [Source:HGNC Symbol;Acc:HGNC:16395]

ENSG00000130706	2491.051	2238.95248	2889.96572	3041.42892	-0.326770922	0.0044128	0.0193882	ADRM1	adhesion regulating molecule 1 [Source:HGNC Symbol;Acc:HGNC:15759]
ENSG00000273151	44.2897253	38.0985187	8.4286277	12.7746402	1.958040411	0.0044146	0.019391	AC073957.3	novel transcript, antisense to GET4
ENSG00000019102	24.605403	23.0363136	2.10715692	4.2582134	2.904927212	0.0044312	0.0194591	VSIG2	V-set and immunoglobulin domain containing 2 [Source:HGNC Symbol;Acc:HGNC:17149]
ENSG00000173077	10.8263773	9.74613268	0	0	5.722175725	0.0044444	1	1-Dec	deleted in esophageal cancer 1 [Source:HGNC Symbol;Acc:HGNC:23658]
ENSG00000271503	3.93686447	11.5181568	28.4466185	44.7112407	-2.229731593	0.0044594	0.0195777	CCL5	C-C motif chemokine ligand 5 [Source:HGNC Symbol;Acc:HGNC:10632]
ENSG00000244731	18.7001062	18.6062533	70.589757	43.6466874	-1.614820407	0.0044679	0.019609	C4A	complement C4A (Rodgers blood group) [Source:HGNC Symbol;Acc:HGNC:1323]
ENSG00000153936	811.978298	1073.84662	737.504924	588.698003	0.508500421	0.0044688	0.019609	HS2ST1	heparan sulfate 2-O-sulfotransferase 1 [Source:HGNC Symbol;Acc:HGNC:5193]
ENSG00000010165	1446.79769	1188.14217	1714.17216	1726.70553	-0.38559873	0.0044827	0.0196648	METTL13	methyltransferase like 13 [Source:HGNC Symbol;Acc:HGNC:24248]
ENSG00000126603	691.903931	460.726272	816.523308	859.094554	-0.541151005	0.0045042	0.0197541	GLIS2	GLIS family zinc finger 2 [Source:HGNC Symbol;Acc:HGNC:29450]
ENSG00000116691	380.891638	350.860776	531.003545	510.985608	-0.510435636	0.004515	0.019796	MIIP	migration and invasion inhibitory protein [Source:HGNC Symbol;Acc:HGNC:25715]
ENSG00000259768	191.922143	202.01075	99.0363755	125.617295	0.810697962	0.0045161	0.019796	AC004943.2	novel transcript, antisense to ZFHX3
ENSG00000108813	437.976173	420.855729	287.62692	299.139491	0.549427212	0.0045206	0.0198083	DLX4	distal-less homeobox 4 [Source:HGNC Symbol;Acc:HGNC:2917]
ENSG00000171109	1092.47989	1264.33921	950.327773	802.673226	0.427355883	0.0045213	0.0198083	MFN1	mitofusin 1 [Source:HGNC Symbol;Acc:HGNC:18262]
ENSG00000101193	1620.01973	1485.84223	1886.95903	2078.00814	-0.352554052	0.0045237	0.0198136	GID8	GID complex subunit 8 homolog [Source:HGNC Symbol;Acc:HGNC:15857]
ENSG00000165752	568.876916	412.881621	303.430597	344.915285	0.597561699	0.0045302	0.0198371	STK32C	serine/threonine kinase 32C [Source:HGNC Symbol;Acc:HGNC:21332]
ENSG00000103647	17.7158901	20.3782774	2.10715692	1.06455335	3.585274121	0.0045504	0.0199156	CORO2B	coronin 2B [Source:HGNC Symbol;Acc:HGNC:2256]
ENSG00000109323	601.356048	630.840588	414.056336	466.274367	0.4853904	0.0045514	0.0199156	MANBA	mannosidase beta [Source:HGNC Symbol;Acc:HGNC:6831]
ENSG00000134909	599.387616	634.384636	458.306631	426.885893	0.47921334	0.0045517	0.0199156	ARHGAP32	Rho GTPase activating protein 32 [Source:HGNC Symbol;Acc:HGNC:17399]
ENSG00000117385	2616.04644	2300.97332	1822.69074	2029.03869	0.352054629	0.0045544	0.0199224	P3H1	prolyl 3-hydroxylase 1 [Source:HGNC Symbol;Acc:HGNC:19316]
ENSG00000134014	531.476704	582.995937	708.004727	830.351613	-0.464513747	0.0045612	0.0199472	ELP3	elongator acetyltransferase complex subunit 3 [Source:HGNC Symbol;Acc:HGNC:20696]
ENSG00000241553	1933.00046	1713.54733	2150.35364	2570.89634	-0.372881964	0.0045766	0.0200092	ARPC4	actin related protein 2/3 complex subunit 4 [Source:HGNC Symbol;Acc:HGNC:707]
ENSG00000117528	1324.7549	1603.68183	1235.84754	821.835186	0.509312265	0.0045832	0.0200329	ABCD3	ATP binding cassette subfamily D member 3 [Source:HGNC Symbol;Acc:HGNC:67]
ENSG00000004487	7475.12142	7341.49594	8913.27379	8947.57091	-0.269603667	0.0046046	0.020121	KDM1A	lysine demethylase 1A [Source:HGNC Symbol;Acc:HGNC:29079]
ENSG00000132718	89.5636668	59.3628081	18.9644123	35.1302606	1.45997086	0.004608	0.0201307	SYT11	synaptotagmin 11 [Source:HGNC Symbol;Acc:HGNC:19239]
ENSG00000170100	601.356048	513.886996	419.324228	352.367159	0.53054865	0.0046111	0.0201393	ZNF778	zinc finger protein 778 [Source:HGNC Symbol;Acc:HGNC:26479]
ENSG00000141985	2766.63151	2326.66767	3149.14602	3309.69637	-0.343006664	0.0046248	0.020192	SH3GL1	SH3 domain containing GRB2 like 1, endophilin A2 [Source:HGNC Symbol;Acc:HGNC:10830]
ENSG00000103253	370.06526	334.912559	492.021142	517.372928	-0.518427178	0.0046261	0.020192	HAGHL	hydroxyacylglutathione hydrolase like [Source:HGNC Symbol;Acc:HGNC:14177]
ENSG00000029153	1248.97025	1373.3187	1072.54287	910.193114	0.403535558	0.0046268	0.020192	ARNTL2	aryl hydrocarbon receptor nuclear translocator like 2 [Source:HGNC Symbol;Acc:HGNC:18984]
ENSG00000141867	2138.70162	2007.70333	2515.94537	2673.09346	-0.323762901	0.0046343	0.0202197	BRD4	bromodomain containing 4 [Source:HGNC Symbol;Acc:HGNC:13575]
ENSG00000161888	557.066323	472.244429	675.343794	757.961985	-0.478438275	0.0046357	0.0202206	SPC24	SPC24, NDC80 kinetochore complex component [Source:HGNC Symbol;Acc:HGNC:26913]
ENSG00000100503	1403.49218	1605.45386	1193.7044	1120.97468	0.378794901	0.0046483	0.0202702	NIN	ninein [Source:HGNC Symbol;Acc:HGNC:14906]
ENSG00000182158	2195.78616	2493.23794	1900.65555	1792.70784	0.344607371	0.0046512	0.0202774	CREB3L2	cAMP responsive element binding protein 3 like 2 [Source:HGNC Symbol;Acc:HGNC:23720]
ENSG00000104320	341.522993	475.788477	773.326591	501.404628	-0.640202655	0.0046632	0.0203249	NBN	nibrin [Source:HGNC Symbol;Acc:HGNC:7652]
ENSG00000167011	8.85794506	11.5181568	0	0	5.709606268	0.0046633	1	NAT16	N-acetyltransferase 16 (putative) [Source:HGNC Symbol;Acc:HGNC:22030]
ENSG00000176678	107.279557	98.3473389	49.5181877	45.7757941	1.109045481	0.0046834	0.0204024	FOXL1	forkhead box L1 [Source:HGNC Symbol;Acc:HGNC:3817]
ENSG00000256894	40.3528608	40.7565548	9.48220616	11.7100869	1.936672136	0.0046834	0.0204024	AC022509.3	novel transcript, antisense to SSPN
ENSG00000158545	2463.49294	2319.57958	2835.17964	3159.59434	-0.325884583	0.0046865	0.0204103	ZC3H18	zinc finger CCCH-type containing 18 [Source:HGNC Symbol;Acc:HGNC:25091]
ENSG00000151893	1476.32418	1654.18452	1306.43729	1050.71416	0.409540232	0.0046887	0.0204145	CACUL1	CDK2 associated cullin domain 1 [Source:HGNC Symbol;Acc:HGNC:23727]
ENSG00000166471	795.246624	870.063845	635.307813	598.278983	0.433246164	0.0047077	0.0204922	TMEM41B	transmembrane protein 41B [Source:HGNC Symbol;Acc:HGNC:28948]
ENSG00000104812	1970.40067	1757.84793	2384.24806	2321.79086	-0.336339136	0.0047105	0.0204949	GYS1	glycogen synthase 1 [Source:HGNC Symbol;Acc:HGNC:4706]
ENSG00000100865	646.62999	675.141191	826.005514	961.291675	-0.435046685	0.0047107	0.0204949	CINP	cyclin dependent kinase 2 interacting protein [Source:HGNC Symbol;Acc:HGNC:23789]
ENSG00000118197	330.696616	334.912559	466.735259	488.629988	-0.521272591	0.004723	0.0205429	DDX59	DEAD-box helicase 59 [Source:HGNC Symbol;Acc:HGNC:25360]
ENSG00000067369	2237.12324	2200.85396	2712.96454	2791.25888	-0.31067454	0.0047265	0.020553	TP53BP1	tumor protein p53 binding protein 1 [Source:HGNC Symbol;Acc:HGNC:11999]
ENSG00000108175	2093.42768	1771.13811	2542.28483	2394.18048	-0.353589744	0.0047337	0.020579	ZMIZ1	zinc finger MIZ-type containing 1 [Source:HGNC Symbol;Acc:HGNC:16493]
ENSG00000145390	308.059645	347.316728	222.305056	202.265137	0.626821694	0.0047451	0.0206233	USP53	ubiquitin specific peptidase 53 [Source:HGNC Symbol;Acc:HGNC:29255]
ENSG00000103035	1258.81242	1189.9142	1609.86789	1524.4404	-0.356345111	0.0047488	0.020634	PSMD7	proteasome 26S subunit, non-ATPase 7 [Source:HGNC Symbol;Acc:HGNC:9565]
ENSG00000285966	9.84216118	27.4663739	0	2.1291067	4.139332991	0.0047582	0.0206695	AC007686.4	novel transcript
ENSG00000167113	1031.45849	932.084689	1215.82955	1359.43463	-0.391616047	0.0047666	0.0207005	COQ4	coenzyme Q4 [Source:HGNC Symbol;Acc:HGNC:19693]
ENSG00000169371	251.959326	220.617003	345.573736	370.464566	-0.600347511	0.0047846	0.0207734	SNUPN	snurportin 1 [Source:HGNC Symbol;Acc:HGNC:14245]
ENSG00000026297	354.317803	282.637848	187.536966	215.039777	0.66103306	0.0047919	0.0208	RNASET2	ribonuclease T2 [Source:HGNC Symbol;Acc:HGNC:21686]
ENSG00000128591	54.1318865	45.1866152	124.322259	90.4870348	-1.114703628	0.0048082	0.0208651	FLNC	filamin C [Source:HGNC Symbol;Acc:HGNC:3756]
ENSG00000156170	146.648202	185.176521	268.662508	269.331998	-0.695524033	0.0048109	0.0208714	NDUFAF6	NADH:ubiquinone oxidoreductase complex assembly factor 6 [Source:HGNC Symbol;Acc:HGNC:28625]
ENSG00000106367	2649.50979	2545.51265	2002.85266	2154.65598	0.321344144	0.0048188	0.0209006	AP1S1	adaptor related protein complex 1 subunit sigma 1 [Source:HGNC Symbol;Acc:HGNC:559]

ENSG00000138375	326.759751	337.570596	448.824425	510.985608	-0.530584082	0.0048226	0.0209065	SMARCAL1	SWI/SNF related, matrix associated, actin dependent regulator of chromatin, subfamily a like 1 [Source:HGNC Symbol;Acc:HGNC:11102]
ENSG00000188677	285.422674	243.653317	382.448982	407.723933	-0.579681233	0.0048237	0.0209065	PARVB	parvin beta [Source:HGNC Symbol;Acc:HGNC:14653]
ENSG00000170145	1077.71665	1083.59275	846.023505	803.737779	0.389641896	0.0048239	0.0209065	SIK2	salt inducible kinase 2 [Source:HGNC Symbol;Acc:HGNC:21680]
ENSG00000085872	1367.07619	1120.80526	1559.29612	1709.67268	-0.394490136	0.0048253	0.0209072	CHERP	calcium homeostasis endoplasmic reticulum protein [Source:HGNC Symbol;Acc:HGNC:16930]
ENSG00000116560	7094.22978	7680.83856	8623.53971	9458.55652	-0.29131522	0.0048268	0.0209081	SFPQ	splicing factor proline and glutamine rich [Source:HGNC Symbol;Acc:HGNC:10774]
ENSG00000068724	1160.3908	993.219521	1393.88431	1415.85596	-0.384242733	0.004843	0.0209731	TTC7A	tetratricopeptide repeat domain 7A [Source:HGNC Symbol;Acc:HGNC:19750]
ENSG00000143252	1620.01973	1759.61995	2054.478	2225.98106	-0.340629529	0.0048449	0.0209758	SDHC	succinate dehydrogenase complex subunit C [Source:HGNC Symbol;Acc:HGNC:10682]
ENSG00000140853	583.640158	505.912887	762.790807	730.283598	-0.45529924	0.0048493	0.0209895	NLRC5	NLR family CARD domain containing 5 [Source:HGNC Symbol;Acc:HGNC:29933]
ENSG00000141551	2876.86371	2441.84924	3316.665	3385.27965	-0.333821194	0.0048509	0.020991	CSNK1D	casein kinase 1 delta [Source:HGNC Symbol;Acc:HGNC:2452]
ENSG00000095787	1640.68827	1624.06011	2138.76428	1977.94012	-0.334572845	0.0048558	0.0210068	WAC	WW domain containing adaptor with coiled-coil [Source:HGNC Symbol;Acc:HGNC:17327]
ENSG00000218537	4.92108059	15.9482171	0	0	5.746623536	0.0048611	1	MIF-AS1	MIF antisense RNA 1 [Source:HGNC Symbol;Acc:HGNC:27669]
ENSG00000174775	961.579147	799.18288	1149.4541	1191.2352	-0.41144033	0.0048619	0.0210282	HRAS	HRas proto-oncogene, GTPase [Source:HGNC Symbol;Acc:HGNC:5173]
ENSG00000140743	917.289422	953.348978	692.20105	718.573511	0.407212283	0.004892	0.0211526	CDR2	cerebellar degeneration related protein 2 [Source:HGNC Symbol;Acc:HGNC:1799]
ENSG00000225151	156.490363	104.549423	59.0003939	68.1314144	1.036281275	0.0048944	0.0211543	GOLGA2P7	GOLGA2 pseudogene 7 [Source:HGNC Symbol;Acc:HGNC:49516]
ENSG00000167671	1516.67704	1235.98683	1785.81549	1799.09516	-0.381697996	0.0048949	0.0211543	UBXN6	UBX domain protein 6 [Source:HGNC Symbol;Acc:HGNC:14928]
ENSG00000214544	93.5005312	87.7151941	146.447406	185.232283	-0.872474831	0.0049076	0.021204	GTF2IRD2P1	GTF2I repeat domain containing 2 pseudogene 1 [Source:HGNC Symbol;Acc:HGNC:33127]
ENSG00000119285	1696.78859	1887.20569	2400.05174	2158.91419	-0.346877456	0.004918	0.0212395	HEATR1	HEAT repeat containing 1 [Source:HGNC Symbol;Acc:HGNC:25517]
ENSG00000163931	20106.5511	18138.4389	22586.6151	23779.9927	-0.277854241	0.0049183	0.0212395	TKT	transketolase [Source:HGNC Symbol;Acc:HGNC:11834]
ENSG00000005194	1500.92958	1255.47909	1781.60118	1784.19142	-0.371974247	0.0049407	0.0213305	CIAPIN1	cytokine induced apoptosis inhibitor 1 [Source:HGNC Symbol;Acc:HGNC:28050]
ENSG00000166938	689.935499	782.348651	982.988705	977.259976	-0.412404656	0.0049439	0.021339	DIS3L	DIS3 like exosome 3'-5' exoribonuclease [Source:HGNC Symbol;Acc:HGNC:28698]
ENSG00000210176	102.358476	76.1970373	143.286671	191.619603	-0.909466126	0.0049605	0.021405	MT-TH	mitochondrially encoded tRNA histidine [Source:HGNC Symbol;Acc:HGNC:7487]
ENSG00000167861	65.9424799	50.5026875	11.5893631	25.5492804	1.648404523	0.0049637	0.0214135	HID1	HID1 domain containing [Source:HGNC Symbol;Acc:HGNC:15736]
ENSG00000067829	766.704356	704.379589	948.220616	1002.80926	-0.407733371	0.0049662	0.0214187	IDH3G	isocitrate dehydrogenase 3 (NAD(+)) gamma [Source:HGNC Symbol;Acc:HGNC:5386]
ENSG00000116459	2425.10852	2782.07787	3168.11044	3372.50501	-0.328628176	0.0049762	0.0214536	ATP5PB	ATP synthase peripheral stalk-membrane subunit b [Source:HGNC Symbol;Acc:HGNC:840]
ENSG00000171475	1567.85628	1499.13241	1879.58398	1993.90843	-0.336943639	0.0049768	0.0214536	WIPF2	WAS/WASL interacting protein family member 2 [Source:HGNC Symbol;Acc:HGNC:30923]
ENSG00000143543	310.028077	318.964342	461.467366	446.047854	-0.528712661	0.0049797	0.0214608	JTB	jumping translocation breakpoint [Source:HGNC Symbol;Acc:HGNC:6201]
ENSG00000005073	211.606465	163.912231	302.377019	293.816725	-0.668628175	0.0049825	0.0214622	HOXA11	homeobox A11 [Source:HGNC Symbol;Acc:HGNC:5101]
ENSG00000249915	2703.64168	2665.12428	3326.14721	3276.69521	-0.298534857	0.0049826	0.0214622	PDCD6	programmed cell death 6 [Source:HGNC Symbol;Acc:HGNC:8765]
ENSG00000105722	1286.37047	1089.79484	1492.92068	1604.2819	-0.382859122	0.0049933	0.0215027	ERF	ETS2 repressor factor [Source:HGNC Symbol;Acc:HGNC:3444]
ENSG00000213199	80.7057217	73.5390011	34.7680893	27.6783871	1.303816551	0.0049971	0.0215069	ASIC3	acid sensing ion channel subunit 3 [Source:HGNC Symbol;Acc:HGNC:101]
ENSG00000155561	3282.36075	3597.20897	4305.97518	4171.98458	-0.301234201	0.0049976	0.0215069	NUP205	nucleoporin 205 [Source:HGNC Symbol;Acc:HGNC:18658]
ENSG00000184922	756.862195	666.28107	906.077478	1003.87381	-0.424986542	0.0049981	0.0215069	FMNL1	formin like 1 [Source:HGNC Symbol;Acc:HGNC:1212]
ENSG00000166801	661.393231	771.716506	935.577675	987.905509	-0.423863913	0.0050041	0.0215273	FAM111A	family with sequence similarity 111 member A [Source:HGNC Symbol;Acc:HGNC:24725]
ENSG00000136108	517.697678	656.534938	962.970715	721.767171	-0.520033131	0.0050085	0.0215408	CKAP2	cytoskeleton associated protein 2 [Source:HGNC Symbol;Acc:HGNC:1990]
ENSG00000073578	7527.28487	6830.26698	8608.78962	8869.85851	-0.283878476	0.0050173	0.0215729	SDHA	succinate dehydrogenase complex flavoprotein subunit A [Source:HGNC Symbol;Acc:HGNC:10680]
ENSG00000204843	3426.05631	2935.35796	4053.11634	3906.9108	-0.323699716	0.0050216	0.0215839	DCTN1	dynactin subunit 1 [Source:HGNC Symbol;Acc:HGNC:2711]
ENSG00000214975	61.0213993	45.1866152	111.679317	110.713548	-1.068925071	0.0050224	0.0215839	PPIAP29	peptidylprolyl isomerase A pseudogene 29 [Source:HGNC Symbol;Acc:HGNC:43021]
ENSG00000157827	976.342389	1060.55644	756.469336	786.704926	0.400793256	0.0050339	0.0216282	FMNL2	formin like 2 [Source:HGNC Symbol;Acc:HGNC:18267]
ENSG00000233532	33.463348	38.9845307	6.32147077	10.6455335	2.095638979	0.0050397	0.0216475	LINC00460	long intergenic non-protein coding RNA 460 [Source:HGNC Symbol;Acc:HGNC:42809]
ENSG00000136436	1570.80892	1613.42796	1207.40092	1289.17411	0.351104754	0.0050702	0.0217729	CALCOCO2	calcium binding and coiled-coil domain 2 [Source:HGNC Symbol;Acc:HGNC:29912]
ENSG00000261556	25.5896191	22.1503015	63.2147077	64.9377544	-1.426155493	0.005077	0.0217946	SMG1P7	SMG1 pseudogene 7 [Source:HGNC Symbol;Acc:HGNC:49864]
ENSG00000113719	5390.55168	4910.27885	4240.65331	4159.20994	0.29419751	0.0050778	0.0217946	ERGIC1	endoplasmic reticulum-golgi intermediate compartment 1 [Source:HGNC Symbol;Acc:HGNC:29205]
ENSG00000112304	733.241008	722.985842	476.217465	576.987916	0.467500221	0.0050815	0.0218035	ACOT13	acyl-CoA thioesterase 13 [Source:HGNC Symbol;Acc:HGNC:20999]
ENSG00000134330	362.191531	334.026547	498.342613	493.952755	-0.511740101	0.0050825	0.0218035	IAH1	isoamyl acetate hydrolyzing esterase 1 (putative) [Source:HGNC Symbol;Acc:HGNC:27696]
ENSG00000167705	121.058583	111.63752	220.197899	180.97407	-0.786508012	0.0050906	0.0218328	RILP	Rab interacting lysosomal protein [Source:HGNC Symbol;Acc:HGNC:30266]
ENSG00000065057	578.719078	446.550079	680.611687	768.607519	-0.500379033	0.0050934	0.0218393	NTHL1	nth like DNA glycosylase 1 [Source:HGNC Symbol;Acc:HGNC:8028]
ENSG00000117036	719.461982	614.892371	456.199474	500.340075	0.479690068	0.0051178	0.0219384	ETV3	ETS variant 3 [Source:HGNC Symbol;Acc:HGNC:3492]
ENSG00000185015	45.2739414	55.8187599	14.7500985	17.0328536	1.670528469	0.0051293	0.021982	CA13	carbonic anhydrase 13 [Source:HGNC Symbol;Acc:HGNC:14914]
ENSG00000225697	1135.7854	991.447497	793.344582	812.254206	0.405419429	0.0051381	0.0220142	SLC26A6	solute carrier family 26 member 6 [Source:HGNC Symbol;Acc:HGNC:14472]
ENSG00000066583	595.450752	569.705756	392.984766	438.59598	0.486473755	0.0051396	0.0220149	ISOC1	isochorismatase domain containing 1 [Source:HGNC Symbol;Acc:HGNC:24254]
ENSG00000215305	805.088785	764.628409	549.967957	608.924516	0.437611946	0.0051473	0.0220374	VPS16	VPS16, CORVET/HOPS core subunit [Source:HGNC Symbol;Acc:HGNC:14584]
ENSG00000113758	3718.36849	3216.22378	4145.83125	4537.12638	-0.324591171	0.0051475	0.0220374	DBN1	drebrin 1 [Source:HGNC Symbol;Acc:HGNC:2695]

ENSG00000070882	794.262407	800.068892	619.504136	562.084169	0.432197596	0.0051524	0.0220531	OSBPL3	oxysterol binding protein like 3 [Source:HGNC Symbol;Acc:HGNC:16370]
ENSG00000101665	1227.3175	1126.12133	935.577675	861.22366	0.3890164	0.005156	0.0220617	SMAD7	SMAD family member 7 [Source:HGNC Symbol;Acc:HGNC:6773]
ENSG00000103855	7972.15056	6827.60895	6158.16611	5775.20193	0.310439555	0.005157	0.0220617	CD276	CD276 molecule [Source:HGNC Symbol;Acc:HGNC:19137]
ENSG00000034693	509.823949	551.099502	407.734865	326.817879	0.530559814	0.0051657	0.0220932	PEX3	peroxisomal biogenesis factor 3 [Source:HGNC Symbol;Acc:HGNC:8858]
ENSG00000153904	293.296403	328.710475	222.305056	170.328536	0.664058291	0.0051779	0.0221398	DDAH1	dimethylarginine dimethylaminohydrolase 1 [Source:HGNC Symbol;Acc:HGNC:2715]
ENSG00000136425	225.385491	182.518485	111.679317	124.552742	0.787097404	0.0051842	0.0221609	CIB2	calcium and integrin binding family member 2 [Source:HGNC Symbol;Acc:HGNC:24579]
ENSG00000173581	60.0371832	73.5390011	124.322259	137.327382	-0.968153715	0.0052026	0.0222341	CCDC106	coiled-coil domain containing 106 [Source:HGNC Symbol;Acc:HGNC:30181]
ENSG00000103196	157.474579	166.570268	75.8576493	100.068015	0.881744354	0.0052102	0.0222608	CRISPLD2	cysteine rich secretory protein LCCL domain containing 2 [Source:HGNC Symbol;Acc:HGNC:25248]
ENSG00000204564	245.069813	217.072955	312.912803	400.27206	-0.626574064	0.0052376	0.0223722	C6orf136	chromosome 6 open reading frame 136 [Source:HGNC Symbol;Acc:HGNC:21301]
ENSG00000254726	267.706784	239.223257	352.948785	407.723933	-0.586143527	0.0052412	0.0223819	MEX3A	mex-3 RNA binding family member A [Source:HGNC Symbol;Acc:HGNC:33482]
ENSG00000178028	817.883594	729.187927	1012.4889	1031.5522	-0.402398745	0.0052466	0.0223994	DMAP1	DNA methyltransferase 1 associated protein 1 [Source:HGNC Symbol;Acc:HGNC:18291]
ENSG00000167291	1303.10214	1142.95556	976.667235	879.321067	0.397829416	0.0052519	0.0224163	TBC1D16	TBC1 domain family member 16 [Source:HGNC Symbol;Acc:HGNC:28356]
ENSG00000272325	110.232205	106.321447	207.554957	170.328536	-0.803610431	0.0052729	0.0225004	NUDT3	nudix hydrolase 3 [Source:HGNC Symbol;Acc:HGNC:8050]
ENSG00000099917	1655.45151	1372.43268	1893.2805	2018.39315	-0.369975455	0.0053088	0.0226477	MED15	mediator complex subunit 15 [Source:HGNC Symbol;Acc:HGNC:14248]
ENSG00000142002	2216.4547	1871.25747	2562.30282	2624.12401	-0.343841419	0.0053227	0.0227012	DPP9	dipeptidyl peptidase 9 [Source:HGNC Symbol;Acc:HGNC:18648]
ENSG00000166855	857.252239	924.11058	1146.29337	1173.13779	-0.380450129	0.0053245	0.0227032	CLPX	caseinolytic mitochondrial matrix peptidase chaperone subunit [Source:HGNC Symbol;Acc:HGNC:2088]
ENSG00000123685	97.4373957	71.766977	38.9824031	31.9366005	1.25286385	0.005332	0.0227295	BATF3	basic leucine zipper ATF-like transcription factor 3 [Source:HGNC Symbol;Acc:HGNC:28915]
ENSG00000055813	125.979663	120.49764	69.5361785	53.2276675	1.005092704	0.0053402	0.0227588	CCDC85A	coiled-coil domain containing 85A [Source:HGNC Symbol;Acc:HGNC:29400]
ENSG00000236104	312.980726	225.047064	375.073933	454.564281	-0.626465125	0.0053562	0.0228213	ZBTB22	zinc finger and BTB domain containing 22 [Source:HGNC Symbol;Acc:HGNC:13085]
ENSG00000171159	1693.83594	1477.86812	1215.82955	1245.52742	0.365439529	0.0053576	0.0228215	C9orf16	chromosome 9 open reading frame 16 [Source:HGNC Symbol;Acc:HGNC:17823]
ENSG00000175879	139.758689	148.850026	59.0003939	89.4224814	0.96004989	0.005361	0.0228304	HOXD8	homeobox D8 [Source:HGNC Symbol;Acc:HGNC:5139]
ENSG00000105835	1558.99833	1957.20064	1446.56323	1177.39601	0.422633244	0.0053652	0.0228422	NAMPT	nicotinamide phosphoribosyltransferase [Source:HGNC Symbol;Acc:HGNC:30092]
ENSG00000196668	24.605403	9.74613268	0	1.06455335	5.01559763	0.0053674	0.0228461	LINC00173	long intergenic non-protein coding RNA 173 [Source:HGNC Symbol;Acc:HGNC:33791]
ENSG00000109738	66.926696	89.4872182	34.7680893	28.7429405	1.301467397	0.0053781	0.0228858	GLRB	glycine receptor beta [Source:HGNC Symbol;Acc:HGNC:4329]
ENSG00000132801	213.574898	209.098847	134.858043	116.036315	0.752229258	0.0053799	0.0228875	ZSWIM3	zinc finger SWIM-type containing 3 [Source:HGNC Symbol;Acc:HGNC:16157]
ENSG00000171307	990.121415	939.172785	1201.07945	1300.88419	-0.375179182	0.0053908	0.0229282	ZDHHC16	zinc finger DHHC-type containing 16 [Source:HGNC Symbol;Acc:HGNC:20714]
ENSG00000152234	7588.30627	7616.15968	9127.15022	9126.41587	-0.263679311	0.0054033	0.0229758	ATP5F1A	ATP synthase F1 subunit alpha [Source:HGNC Symbol;Acc:HGNC:823]
ENSG00000135917	29.5264835	20.3782774	5.26789231	2.1291067	2.751128484	0.0054167	0.0230262	SLC19A3	solute carrier family 19 member 3 [Source:HGNC Symbol;Acc:HGNC:16266]
ENSG00000185238	601.356048	638.814697	895.541693	780.317606	-0.434126841	0.0054188	0.0230262	PRMT3	protein arginine methyltransferase 3 [Source:HGNC Symbol;Acc:HGNC:30163]
ENSG00000163832	915.32099	782.348651	1045.14983	1229.55912	-0.422659942	0.0054193	0.0230262	ELP6	elongator acetyltransferase complex subunit 6 [Source:HGNC Symbol;Acc:HGNC:25976]
ENSG00000105223	2648.52557	2339.95786	2048.15653	1902.35684	0.336287398	0.0054351	0.0230876	PLD3	phospholipase D family member 3 [Source:HGNC Symbol;Acc:HGNC:17158]
ENSG00000166783	611.198209	661.85101	842.86277	858.03	-0.417592265	0.0054375	0.0230919	MARF1	meiosis regulator and mRNA stability factor 1 [Source:HGNC Symbol;Acc:HGNC:29562]
ENSG00000148343	553.129458	493.508718	705.89757	721.767171	-0.448493877	0.0054477	0.0231294	MIGA2	mitoguardin 2 [Source:HGNC Symbol;Acc:HGNC:23621]
ENSG00000198837	1372.98148	1179.28205	1614.0822	1674.54242	-0.366199035	0.0054515	0.0231394	DENND4B	DENN domain containing 4B [Source:HGNC Symbol;Acc:HGNC:29044]
ENSG00000132912	680.093338	838.16741	1111.52528	954.904355	-0.443983564	0.0054535	0.0231394	DCTN4	dynactin subunit 4 [Source:HGNC Symbol;Acc:HGNC:15518]
ENSG00000160183	925.163151	861.203724	1125.2218	1202.94529	-0.382450941	0.0054541	0.0231394	TMPRSS3	transmembrane serine protease 3 [Source:HGNC Symbol;Acc:HGNC:11877]
ENSG00000267519	727.335711	608.690286	384.556139	533.341228	0.541162635	0.0054596	0.0231569	AC020916.1	novel transcript
ENSG00000182180	2051.10639	2220.34623	2710.85738	2608.15571	-0.316230879	0.0054824	0.0232477	MRPS16	mitochondrial ribosomal protein S16 [Source:HGNC Symbol;Acc:HGNC:14048]
ENSG00000231133	24.605403	23.0363136	1.05357846	5.32276675	2.903640252	0.0054867	0.0232566	HAR1B	highly accelerated region 1B [Source:HGNC Symbol;Acc:HGNC:33118]
ENSG00000136628	4568.73122	5369.23309	6042.27248	6288.31664	-0.311025046	0.0054873	0.0232566	EPRS	glutamyl-prolyl-tRNA synthetase [Source:HGNC Symbol;Acc:HGNC:3418]
ENSG00000177105	1126.92746	972.841244	1326.45528	1408.40408	-0.381759132	0.0054905	0.0232646	RHOG	ras homolog family member G [Source:HGNC Symbol;Acc:HGNC:672]
ENSG00000138593	799.183488	884.240038	697.468942	476.919901	0.519598357	0.0054976	0.0232888	SECISBP2L	SECIS binding protein 2 like [Source:HGNC Symbol;Acc:HGNC:28997]
ENSG00000143458	177.158901	183.404497	292.894813	271.461104	-0.646140285	0.0055118	0.0233428	GABPB2	GA binding protein transcription factor subunit beta 2 [Source:HGNC Symbol;Acc:HGNC:28441]
ENSG00000175213	383.844286	303.016125	460.413788	548.244975	-0.555519733	0.0055139	0.0233459	ZNF408	zinc finger protein 408 [Source:HGNC Symbol;Acc:HGNC:20041]
ENSG00000234996	52.1634543	48.7306634	8.4286277	21.291067	1.764110757	0.0055234	0.0233804	AC098934.2	actin gamma 1 pseudogene [Source:NCBI gene;Acc:148709]
ENSG00000189143	685.014418	605.146238	455.145896	476.919901	0.468563209	0.0055253	0.0233826	CLDN4	claudin 4 [Source:HGNC Symbol;Acc:HGNC:2046]
ENSG00000109390	550.17681	598.944154	740.665659	816.51242	-0.437929442	0.0055297	0.0233955	NDUFC1	NADH:ubiquinone oxidoreductase subunit C1 [Source:HGNC Symbol;Acc:HGNC:7705]
ENSG00000230699	150.585066	148.850026	72.6969139	88.3579281	0.89481084	0.0055317	0.0233978	AL645608.2	novel transcript
ENSG00000117118	1658.40416	1762.27799	2051.31727	2264.30498	-0.335079998	0.0055359	0.0234075	SDHB	succinate dehydrogenase complex iron sulfur subunit B [Source:HGNC Symbol;Acc:HGNC:10681]
ENSG00000253669	363.175748	331.368511	220.197899	239.524504	0.594924178	0.0055367	0.0234075	GASAL1	growth arrest associated lncRNA 1 [Source:HGNC Symbol;Acc:HGNC:53461]
ENSG00000165943	609.229777	525.405153	431.96717	361.948139	0.514491748	0.0055455	0.0234388	MOAP1	modulator of apoptosis 1 [Source:HGNC Symbol;Acc:HGNC:16658]

ENSG00000169851	2771.55259	2776.7618	2336.83703	2121.65483	0.315470317	0.0055528	0.0234637	PCDH7	protocadherin 7 [Source:HGNC Symbol;Acc:HGNC:8659]
ENSG00000173267	95.4689635	57.590784	141.179514	157.553896	-0.968469967	0.0055581	0.02348	SNCG	synuclein gamma [Source:HGNC Symbol;Acc:HGNC:11141]
ENSG00000020181	778.514949	707.037625	560.503742	533.341228	0.441174366	0.0055628	0.0234897	ADGRA2	adhesion G protein-coupled receptor A2 [Source:HGNC Symbol;Acc:HGNC:17849]
ENSG00000122042	394.670663	515.65902	315.01996	298.074938	0.5712935	0.0055634	0.0234897	UBL3	ubiquitin like 3 [Source:HGNC Symbol;Acc:HGNC:12504]
ENSG00000231711	99.4058279	72.6529891	154.876034	162.876663	-0.887476071	0.0055645	0.0234897	LINC00899	long intergenic non-protein coding RNA 899 [Source:HGNC Symbol;Acc:HGNC:48583]
ENSG00000197885	127.948095	159.482171	83.2326985	67.0668611	0.936236302	0.0055709	0.0235109	NKIRAS1	NFKB inhibitor interacting Ras like 1 [Source:HGNC Symbol;Acc:HGNC:17899]
ENSG00000277969	150.585066	143.533954	87.4470124	69.1959678	0.9084659	0.0055857	0.0235672	AC006449.6	novel transcript
ENSG00000087460	28281.4502	26993.2435	31579.9608	35198.392	-0.272771521	0.0055877	0.0235699	GNAS	GNAS complex locus [Source:HGNC Symbol;Acc:HGNC:4392]
ENSG00000102226	2505.81424	2076.81227	2882.59067	2928.58627	-0.343044641	0.0056022	0.023625	USP11	ubiquitin specific peptidase 11 [Source:HGNC Symbol;Acc:HGNC:12609]
ENSG00000182621	132.869176	126.699725	74.8040708	57.4858809	0.971930111	0.005608	0.0236437	PLCB1	phospholipase C beta 1 [Source:HGNC Symbol;Acc:HGNC:15917]
ENSG00000175662	1065.90606	994.991545	793.344582	778.188499	0.390837188	0.0056268	0.0237172	TOM1L2	target of myb1 like 2 membrane trafficking protein [Source:HGNC Symbol;Acc:HGNC:11984]
ENSG00000127948	4370.90378	3865.67063	3194.4499	3436.37821	0.312696027	0.0056455	0.02379	POR	cytochrome p450 oxidoreductase [Source:HGNC Symbol;Acc:HGNC:9208]
ENSG00000157911	237.196084	220.617003	330.823637	358.754479	-0.59141139	0.0056558	0.0238275	PEX10	peroxisomal biogenesis factor 10 [Source:HGNC Symbol;Acc:HGNC:8851]
ENSG00000183010	1017.67947	839.053423	1207.40092	1244.46287	-0.40182244	0.0056723	0.0238875	PYCR1	pyrroline-5-carboxylate reductase 1 [Source:HGNC Symbol;Acc:HGNC:9721]
ENSG00000197147	249.006678	253.39945	171.733289	137.327382	0.700871961	0.0056729	0.0238875	LRRC8B	leucine rich repeat containing 8 VRAC subunit B [Source:HGNC Symbol;Acc:HGNC:30692]
ENSG00000185567	8869.75566	7944.87016	11199.5391	9632.07871	-0.309144886	0.0057039	0.0240121	AHNAK2	AHNAK nucleoprotein 2 [Source:HGNC Symbol;Acc:HGNC:20125]
ENSG00000153012	14.7632418	23.9223257	3.16073539	0	3.610738852	0.0057101	0.0240321	LGI2	leucine rich repeat LGI family member 2 [Source:HGNC Symbol;Acc:HGNC:18710]
ENSG00000204410	74.800425	42.528579	21.0715692	18.097407	1.580188846	0.0057199	0.0240628	MSH5	mutS homolog 5 [Source:HGNC Symbol;Acc:HGNC:7328]
ENSG00000185386	408.449689	336.684583	251.805252	242.718164	0.590565182	0.0057202	0.0240628	MAPK11	mitogen-activated protein kinase 11 [Source:HGNC Symbol;Acc:HGNC:6873]
ENSG00000090615	2645.57293	2549.0567	1954.38805	2194.04445	0.324410015	0.0057262	0.024082	GOLGA3	golgin A3 [Source:HGNC Symbol;Acc:HGNC:4426]
ENSG00000164056	82.6741539	78.8550735	38.9824031	28.7429405	1.253468765	0.0057283	0.024085	SPRY1	sprouty RTK signaling antagonist 1 [Source:HGNC Symbol;Acc:HGNC:11269]
ENSG00000233901	41.337077	42.528579	93.7684831	90.4870348	-1.135299161	0.0057453	0.0241502	LINC01503	long intergenic non-protein coding RNA 1503 [Source:HGNC Symbol;Acc:HGNC:51184]
ENSG00000037474	4685.85294	4455.75466	5450.16139	5645.32642	-0.279527381	0.0057568	0.0241925	NSUN2	NOP2/Sun RNA methyltransferase family member 2 [Source:HGNC Symbol;Acc:HGNC:25994]
ENSG00000127419	376.954773	318.964342	224.412212	234.201737	0.600891444	0.0057716	0.0242486	TMEM175	transmembrane protein 175 [Source:HGNC Symbol;Acc:HGNC:28709]
ENSG00000171056	77.7530733	69.1089408	120.107945	161.812109	-0.94164019	0.0057779	0.0242694	SOX7	SRY-box 7 [Source:HGNC Symbol;Acc:HGNC:18196]
ENSG00000133703	1279.48095	1363.57256	1129.43611	790.963139	0.460821514	0.0057868	0.0243006	KRAS	KRAS proto-oncogene, GTPase [Source:HGNC Symbol;Acc:HGNC:6407]
ENSG00000137804	465.534224	524.519141	722.754825	642.990224	-0.463540092	0.0057949	0.0243285	NUSAP1	nucleolar and spindle associated protein 1 [Source:HGNC Symbol;Acc:HGNC:18538]
ENSG00000143294	1841.46836	1790.63038	2181.961	2357.98567	-0.321934374	0.0058129	0.0243982	PRCC	proline rich mitotic checkpoint control factor [Source:HGNC Symbol;Acc:HGNC:9343]
ENSG00000237441	343.491425	265.803619	440.395797	457.757941	-0.56126962	0.0058229	0.0244341	RGL2	ral guanine nucleotide dissociation stimulator like 2 [Source:HGNC Symbol;Acc:HGNC:9769]
ENSG00000172062	206.685385	237.451233	296.055548	397.0784	-0.641168304	0.0058255	0.0244387	SMN1	survival of motor neuron 1, telomeric [Source:HGNC Symbol;Acc:HGNC:11117]
ENSG00000184402	675.172257	625.524516	485.699671	462.016154	0.45640158	0.0058328	0.0244633	SS18L1	SS18L1, nBAF chromatin remodeling complex subunit [Source:HGNC Symbol;Acc:HGNC:15592]
ENSG00000058262	7701.49112	7199.73401	9152.4361	8831.53459	-0.271352792	0.0058406	0.02449	SEC61A1	Sec61 translocon alpha 1 subunit [Source:HGNC Symbol;Acc:HGNC:18276]
ENSG00000102879	102.358476	96.5753147	45.3038739	47.9049008	1.09350729	0.0058433	0.024495	CORO1A	coronin 1A [Source:HGNC Symbol;Acc:HGNC:2252]
ENSG00000196776	937.957961	1148.27163	860.773604	627.021923	0.488074732	0.0058471	0.0245049	CD47	CD47 molecule [Source:HGNC Symbol;Acc:HGNC:1682]
ENSG00000100109	526.555623	539.581346	697.468942	745.187345	-0.436171122	0.0058572	0.0245411	TFIP11	tuftelin interacting protein 11 [Source:HGNC Symbol;Acc:HGNC:17165]
ENSG00000183520	573.797997	742.478108	889.220222	915.515881	-0.454271287	0.0058735	0.0245991	UTP11	UTP11, small subunit processome component [Source:HGNC Symbol;Acc:HGNC:24329]
ENSG00000180758	508.839733	475.788477	321.341431	367.270906	0.515677548	0.0058739	0.0245991	GPR157	G protein-coupled receptor 157 [Source:HGNC Symbol;Acc:HGNC:23687]
ENSG00000255571	68.8951283	79.7410856	12.6429415	39.388474	1.515838467	0.0058963	0.0246828	MIR9-3HG	MIR9-3 host gene [Source:HGNC Symbol;Acc:HGNC:27388]
ENSG00000135241	531.476704	707.923637	478.324622	363.012692	0.559599809	0.0058968	0.0246828	PNPLA8	patatin like phospholipase domain containing 8 [Source:HGNC Symbol;Acc:HGNC:28900]
ENSG00000139044	113.184854	90.3732303	52.6789231	41.5175807	1.110367437	0.0058998	0.0246861	B4GALNT3	beta-1,4-N-acetyl-galactosaminyltransferase 3 [Source:HGNC Symbol;Acc:HGNC:24137]
ENSG00000122642	3206.57611	3003.58089	3711.75692	3912.23356	-0.296040777	0.0059005	0.0246861	FKBP9	FK506 binding protein 9 [Source:HGNC Symbol;Acc:HGNC:3725]
ENSG00000108469	415.339202	349.088752	532.057123	550.374082	-0.50279553	0.005904	0.0246892	RECQL5	RecQ like helicase 5 [Source:HGNC Symbol;Acc:HGNC:9950]
ENSG00000171877	138.774473	112.523532	48.4646093	74.5187345	1.030369053	0.0059042	0.0246892	FRMD5	FERM domain containing 5 [Source:HGNC Symbol;Acc:HGNC:28214]
ENSG00000214113	353.333586	356.176849	445.66369	587.633449	-0.542202512	0.0059117	0.0247144	LYRM4	LYR motif containing 4 [Source:HGNC Symbol;Acc:HGNC:21365]
ENSG00000173320	192.906359	272.005703	159.090348	103.261675	0.826424358	0.005919	0.0247387	STOX2	storkhead box 2 [Source:HGNC Symbol;Acc:HGNC:25450]
ENSG00000164346	1236.17544	1480.52616	1636.20735	1921.5188	-0.388574874	0.0059208	0.0247403	NSA2	NSA2, ribosome biogenesis homolog [Source:HGNC Symbol;Acc:HGNC:30728]
ENSG00000157193	1776.51009	1764.05002	1440.24176	1370.08016	0.333202156	0.005926	0.0247559	LRP8	LDL receptor related protein 8 [Source:HGNC Symbol;Acc:HGNC:6700]
ENSG00000198780	86.6110184	136.445858	209.662114	189.490496	-0.836496284	0.0059297	0.0247652	FAM169A	family with sequence similarity 169 member A [Source:HGNC Symbol;Acc:HGNC:29138]
ENSG00000065485	1257.8282	1163.33384	917.666841	951.710695	0.372894464	0.0059357	0.0247843	PDIA5	protein disulfide isomerase family A member 5 [Source:HGNC Symbol;Acc:HGNC:24811]
ENSG00000168159	3666.20504	3199.38956	4223.79606	4269.92349	-0.307243925	0.0059402	0.0247969	RNF187	ring finger protein 187 [Source:HGNC Symbol;Acc:HGNC:27146]
ENSG00000137936	1434.00288	1314.8419	1788.97623	1696.89804	-0.343008842	0.0059597	0.0248719	BCAR3	BCAR3, NSP family adaptor protein [Source:HGNC Symbol;Acc:HGNC:973]
ENSG00000174748	15988.5908	16537.4151	18417.6051	21153.7396	-0.282842593	0.0059694	0.0248984	RPL15	ribosomal protein L15 [Source:HGNC Symbol;Acc:HGNC:10306]

ENSG00000171700	529.508272	493.508718	689.040314	699.411551	-0.441029774	0.00597	0.0248984	RGS19	regulator of G protein signaling 19 [Source:HGNC Symbol;Acc:HGNC:13735]
ENSG00000213672	1363.13932	1160.6758	1665.70755	1587.24905	-0.366683707	0.0059704	0.0248984	NCKIPSD	NCK interacting protein with SH3 domain [Source:HGNC Symbol;Acc:HGNC:15486]
ENSG00000175265	908.431477	707.037625	553.128693	609.98907	0.473214703	0.0059799	0.0249318	GOLGA8A	golgin A8 family member A [Source:HGNC Symbol;Acc:HGNC:31972]
ENSG00000205517	172.237821	164.798243	93.7684831	96.8743549	0.821828791	0.0059822	0.0249352	RGL3	ral guanine nucleotide dissociation stimulator like 3 [Source:HGNC Symbol;Acc:HGNC:30282]
ENSG00000204348	206.685385	167.45628	253.912409	350.238052	-0.692454847	0.0059994	0.0250009	DXO	decapping exoribonuclease [Source:HGNC Symbol;Acc:HGNC:2992]
ENSG00000224861	159.443011	125.813713	229.680105	239.524504	-0.719660335	0.0060011	0.0250017	YBX1P1	Y-box binding protein 1 pseudogene 1 [Source:HGNC Symbol;Acc:HGNC:8015]
ENSG00000143621	5648.4163	5709.46173	6863.0101	6802.49591	-0.266831969	0.0060057	0.0250148	ILF2	interleukin enhancer binding factor 2 [Source:HGNC Symbol;Acc:HGNC:6037]
ENSG00000100036	442.897253	382.757211	274.983979	287.429405	0.553270285	0.0060105	0.0250284	SLC35E4	solute carrier family 35 member E4 [Source:HGNC Symbol;Acc:HGNC:17058]
ENSG00000116260	6165.12976	5365.68905	4705.28141	4663.80823	0.299362671	0.0060183	0.0250528	QSOX1	quiescin sulfhydryl oxidase 1 [Source:HGNC Symbol;Acc:HGNC:9756]
ENSG00000160124	248.022462	300.358089	347.680893	500.340075	-0.627822506	0.0060193	0.0250528	CCDC58	coiled-coil domain containing 58 [Source:HGNC Symbol;Acc:HGNC:31136]
ENSG00000189067	2594.39369	2726.25911	3122.80656	3474.70214	-0.310189883	0.0060387	0.0251271	LITAF	lipopolysaccharide induced TNF factor [Source:HGNC Symbol;Acc:HGNC:16841]
ENSG00000179715	168.300956	138.217882	271.823243	227.814417	-0.706384024	0.006053	0.0251806	PCED1B	PC-esterase domain containing 1B [Source:HGNC Symbol;Acc:HGNC:28255]
ENSG00000170312	1633.79876	2028.08161	2665.55351	2157.84964	-0.397088848	0.006065	0.0252241	CDK1	cyclin dependent kinase 1 [Source:HGNC Symbol;Acc:HGNC:1722]
ENSG00000172262	883.826074	879.809977	1152.61484	1127.362	-0.370495956	0.006068	0.0252247	ZNF131	zinc finger protein 131 [Source:HGNC Symbol;Acc:HGNC:12915]
ENSG00000143382	868.078616	753.110253	573.146684	623.828263	0.437182255	0.0060681	0.0252247	ADAMTSL4	ADAMTS like 4 [Source:HGNC Symbol;Acc:HGNC:19706]
ENSG00000182534	1518.64547	1442.42764	1124.16822	1196.55797	0.351411217	0.0060948	0.0253296	MXRA7	matrix remodeling associated 7 [Source:HGNC Symbol;Acc:HGNC:7541]
ENSG00000145777	6.88951283	12.4041689	0	0	5.631900778	0.0060955	1	TSLP	thymic stromal lymphopoietin [Source:HGNC Symbol;Acc:HGNC:30743]
ENSG00000170745	6.88951283	12.4041689	0	0	5.631900778	0.0060955	1	KCNS3	potassium voltage-gated channel modifier subfamily S member 3 [Source:HGNC Symbol;Acc:HGNC:6302]
ENSG00000100664	3247.91319	3776.18341	4584.11989	4184.75922	-0.319860862	0.0061024	0.0253547	EIF5	eukaryotic translation initiation factor 5 [Source:HGNC Symbol;Acc:HGNC:3299]
ENSG00000100417	897.6051	789.436747	634.254234	623.828263	0.42277668	0.0061222	0.0254307	PMM1	phosphomannomutase 1 [Source:HGNC Symbol;Acc:HGNC:9114]
ENSG00000129518	398.607528	572.363792	722.754825	675.991377	-0.52519463	0.0061294	0.0254546	EAPP	E2F associated phosphoprotein [Source:HGNC Symbol;Acc:HGNC:19312]
ENSG00000130830	721.430415	659.192974	502.556927	512.050161	0.444032388	0.0061658	0.0255992	MPP1	membrane palmitoylated protein 1 [Source:HGNC Symbol;Acc:HGNC:7219]
ENSG00000163291	438.960389	509.456936	375.073933	241.653611	0.621132894	0.006182	0.0256602	PAQR3	progestin and adipoQ receptor family member 3 [Source:HGNC Symbol;Acc:HGNC:30130]
ENSG00000006576	679.109122	976.385292	642.682862	493.952755	0.543225235	0.0061911	0.025692	PHTF2	putative homeodomain transcription factor 2 [Source:HGNC Symbol;Acc:HGNC:13411]
ENSG00000033011	530.492488	529.835213	701.683256	728.154492	-0.431330813	0.0061975	0.0257121	ALG1	ALG1, chitobiosyldiphosphodolichol beta-mannosyltransferase [Source:HGNC Symbol;Acc:HGNC:18294]
ENSG00000158301	103.342692	118.725616	201.233486	179.909516	-0.778295618	0.0062085	0.0257356	GPRASP2	G protein-coupled receptor associated sorting protein 2 [Source:HGNC Symbol;Acc:HGNC:25169]
ENSG00000235531	93.5005312	96.5753147	49.5181877	37.2593673	1.131053496	0.0062091	0.0257356	MSC-AS1	MSC antisense RNA 1 [Source:HGNC Symbol;Acc:HGNC:48724]
ENSG00000116191	660.409015	811.587049	603.700459	388.561973	0.569302208	0.0062092	0.0257356	RALGPS2	Ral GEF with PH domain and SH3 binding motif 2 [Source:HGNC Symbol;Acc:HGNC:30279]
ENSG00000183508	44.2897253	48.7306634	15.8036769	12.7746402	1.702805875	0.0062093	0.0257356	TENT5C	terminal nucleotidyltransferase 5C [Source:HGNC Symbol;Acc:HGNC:24712]
ENSG00000150776	477.344817	545.78343	728.022717	672.797717	-0.452623759	0.0062151	0.0257536	NKAPD1	NKAP domain containing 1 [Source:HGNC Symbol;Acc:HGNC:25569]
ENSG00000250182	34.4475641	20.3782774	68.4826001	72.3896278	-1.366580764	0.0062178	0.0257583	EEF1A1P13	eukaryotic translation elongation factor 1 alpha 1 pseudogene 13 [Source:HGNC Symbol;Acc:HGNC:3196]
ENSG00000232653	19.6843224	25.6943498	4.21431385	2.1291067	2.838554245	0.0062482	0.0258778	GOLGA8N	golgin A8 family member N [Source:HGNC Symbol;Acc:HGNC:44405]
ENSG00000167202	2050.12217	2005.0453	1635.15377	1615.99199	0.318748507	0.0062614	0.0259262	TBC1D2B	TBC1 domain family member 2B [Source:HGNC Symbol;Acc:HGNC:29183]
ENSG00000139428	521.634543	501.482827	657.43296	735.606365	-0.445425077	0.0062736	0.0259706	MMAB	methylmalonic aciduria (cobalamin deficiency) cblB type [Source:HGNC Symbol;Acc:HGNC:19331]
ENSG00000181135	423.212931	378.32715	569.985948	548.244975	-0.481045642	0.0062979	0.0260646	ZNF707	zinc finger protein 707 [Source:HGNC Symbol;Acc:HGNC:27815]
ENSG00000167595	223.417059	220.617003	303.430597	372.593673	-0.606384411	0.0063379	0.0262236	PROSER3	proline and serine rich 3 [Source:HGNC Symbol;Acc:HGNC:25204]
ENSG00000119953	253.927758	279.093799	448.824425	350.238052	-0.58366806	0.0063407	0.026229	SMNDC1	survival motor neuron domain containing 1 [Source:HGNC Symbol;Acc:HGNC:16900]
ENSG00000186566	1543.25087	1446.8577	1835.33368	1923.6479	-0.330346035	0.0063472	0.0262494	GPATCH8	G-patch domain containing 8 [Source:HGNC Symbol;Acc:HGNC:29066]
ENSG00000247626	425.181363	435.917934	305.537754	292.752171	0.525423846	0.006356	0.0262742	MARS2	methionyl-tRNA synthetase 2, mitochondrial [Source:HGNC Symbol;Acc:HGNC:25133]
ENSG00000163659	716.509334	837.281398	611.075508	521.631142	0.456477932	0.0063563	0.0262742	TIPARP	TCDD inducible poly(ADP-ribose) polymerase [Source:HGNC Symbol;Acc:HGNC:23696]
ENSG00000182950	81.6899378	70.8809649	33.7145108	29.8074938	1.263303801	0.0063693	0.0263206	ODF3L1	outer dense fiber of sperm tails 3 like 1 [Source:HGNC Symbol;Acc:HGNC:28735]
ENSG00000105617	96.4531796	103.663411	170.679711	177.780409	-0.799535443	0.0063707	0.0263206	LENG1	leukocyte receptor cluster member 1 [Source:HGNC Symbol;Acc:HGNC:15502]
ENSG00000165269	19.6843224	12.4041689	1.05357846	0	4.920229724	0.0063919	0.026402	AQP7	aquaporin 7 [Source:HGNC Symbol;Acc:HGNC:640]
ENSG00000166068	437.976173	516.545032	380.341825	231.008077	0.643018653	0.0064007	0.026432	SPRED1	sprouty related EVH1 domain containing 1 [Source:HGNC Symbol;Acc:HGNC:20249]
ENSG00000099957	87.5952345	87.7151941	42.1431385	36.1948139	1.161974044	0.0064123	0.0264731	P2RX6	purinergic receptor P2X 6 [Source:HGNC Symbol;Acc:HGNC:8538]
ENSG00000107789	1077.71665	1191.68622	954.542087	712.186191	0.445436431	0.0064256	0.0265219	MINPP1	multiple inositol-polyphosphate phosphatase 1 [Source:HGNC Symbol;Acc:HGNC:7102]
ENSG00000153827	800.167704	825.763242	1115.73959	1009.19658	-0.386051614	0.0064461	0.0265999	TRIP12	thyroid hormone receptor interactor 12 [Source:HGNC Symbol;Acc:HGNC:12306]
ENSG00000170965	34.4475641	28.352386	6.32147077	7.45187345	2.188252614	0.0064709	0.0266902	PLAC1	placenta specific 1 [Source:HGNC Symbol;Acc:HGNC:9044]
ENSG00000174564	638.756261	590.970045	789.130268	853.771787	-0.418266581	0.0064712	0.0266902	IL20RB	interleukin 20 receptor subunit beta [Source:HGNC Symbol;Acc:HGNC:6004]
ENSG00000131469	10667.9185	10438.9941	12015.0088	13553.8933	-0.276681854	0.0064988	0.0267975	RPL27	ribosomal protein L27 [Source:HGNC Symbol;Acc:HGNC:10328]

ENSG00000169994	15.7474579	28.352386	53.7325016	70.2605211	-1.485423425	0.0065036	0.0268107	MYO7B	myosin VIIB [Source:HGNC Symbol;Acc:HGNC:7607]
ENSG00000141504	300.185916	296.814041	420.377806	437.531427	-0.523147247	0.0065068	0.0268176	SAT2	spermidine/spermine N1-acetyltransferase family member 2 [Source:HGNC Symbol;Acc:HGNC:23160]
ENSG00000162849	20.6685385	30.1244101	3.16073539	5.32276675	2.584359122	0.0065159	0.0268484	KIF26B	kinesin family member 26B [Source:HGNC Symbol;Acc:HGNC:25484]
ENSG00000241697	17.7158901	15.9482171	0	2.1291067	3.987457597	0.0065187	0.0268533	TMEFF1	transmembrane protein with EGF like and two follistatin like domains 1 [Source:HGNC Symbol;Acc:HGNC:11866]
ENSG00000138442	700.761876	782.348651	935.577675	1026.22943	-0.403027886	0.0065314	0.0268928	WDR12	WD repeat domain 12 [Source:HGNC Symbol;Acc:HGNC:14098]
ENSG00000163482	257.864623	277.321775	401.413394	381.110099	-0.547636201	0.0065314	0.0268928	STK36	serine/threonine kinase 36 [Source:HGNC Symbol;Acc:HGNC:17209]
ENSG00000136153	688.951283	769.05847	910.291791	1027.29398	-0.409729164	0.0065369	0.0269088	LMO7	LIM domain 7 [Source:HGNC Symbol;Acc:HGNC:6646]
ENSG00000124098	954.689635	975.49928	743.826394	732.412705	0.386890548	0.006551	0.0269604	FAM210B	family with sequence similarity 210 member B [Source:HGNC Symbol;Acc:HGNC:16102]
ENSG00000100811	2906.3902	2944.21808	3685.41746	3469.37937	-0.290307942	0.0065548	0.0269694	YY1	YY1 transcription factor [Source:HGNC Symbol;Acc:HGNC:12856]
ENSG00000123091	1008.82152	1170.42193	886.059487	746.251899	0.417272969	0.0065666	0.0270067	RNF11	ring finger protein 11 [Source:HGNC Symbol;Acc:HGNC:10056]
ENSG00000141519	150.585066	144.419966	75.8576493	85.164268	0.873371979	0.0065671	0.0270067	CCDC40	coiled-coil domain containing 40 [Source:HGNC Symbol;Acc:HGNC:26090]
ENSG00000154727	238.180301	321.622378	485.699671	371.529119	-0.613352339	0.0065779	0.0270447	GABPA	GA binding protein transcription factor subunit alpha [Source:HGNC Symbol;Acc:HGNC:4071]
ENSG00000143622	346.444074	316.306306	493.07472	451.370621	-0.511606944	0.0066107	0.0271713	RIT1	Ras like without CAAX 1 [Source:HGNC Symbol;Acc:HGNC:10023]
ENSG00000261324	52.1634543	61.1348323	21.0715692	19.1619603	1.494317047	0.0066119	0.0271713	AC010168.2	novel transcript, overlapping HIST4H4
ENSG00000146776	89.5636668	85.0571579	152.768877	159.683003	-0.839820298	0.0066149	0.0271767	ATXN7L1	ataxin 7 like 1 [Source:HGNC Symbol;Acc:HGNC:22210]
ENSG00000148672	1704.66232	1857.96729	2118.74629	2368.6312	-0.332666483	0.0066286	0.0272267	GLUD1	glutamate dehydrogenase 1 [Source:HGNC Symbol;Acc:HGNC:4335]
ENSG00000002549	801.15192	710.581674	969.292185	1020.90666	-0.397230061	0.0066346	0.0272406	LAP3	leucine aminopeptidase 3 [Source:HGNC Symbol;Acc:HGNC:18449]
ENSG00000134291	6252.725	6334.10023	5069.81956	5363.21978	0.270779057	0.0066352	0.0272406	TMEM106C	transmembrane protein 106C [Source:HGNC Symbol;Acc:HGNC:28775]
ENSG00000178409	176.174685	158.596159	295.001969	238.45995	-0.673053672	0.0066446	0.0272726	BEND3	BEN domain containing 3 [Source:HGNC Symbol;Acc:HGNC:23040]
ENSG00000177283	273.612081	241.881293	164.35824	160.747556	0.664408022	0.0066675	0.0273597	FZD8	frizzled class receptor 8 [Source:HGNC Symbol;Acc:HGNC:4046]
ENSG00000088756	45.2739414	59.3628081	15.8036769	19.1619603	1.582893319	0.0066821	0.0274131	ARHGAP28	Rho GTPase activating protein 28 [Source:HGNC Symbol;Acc:HGNC:25509]
ENSG00000133812	1595.41433	1824.29884	1375.97347	1305.14241	0.35137139	0.0067012	0.0274845	SBF2	SET binding factor 2 [Source:HGNC Symbol;Acc:HGNC:2135]
ENSG00000134716	21.6527546	14.176193	2.10715692	1.06455335	3.494619499	0.0067077	0.0274992	CYP2J2	cytochrome P450 family 2 subfamily J member 2 [Source:HGNC Symbol;Acc:HGNC:2634]
ENSG00000140564	9505.55927	8324.08332	7434.04963	7189.99333	0.285833155	0.006708	0.0274992	FURIN	furin, paired basic amino acid cleaving enzyme [Source:HGNC Symbol;Acc:HGNC:8568]
ENSG00000143549	8678.81773	9022.26083	10017.424	11601.5024	-0.288419297	0.0067256	0.0275647	TPM3	tropomyosin 3 [Source:HGNC Symbol;Acc:HGNC:12012]
ENSG00000278535	272.627865	248.969389	371.913197	392.820186	-0.552596224	0.0067427	0.0276282	DHRS11	dehydrogenase/reductase 11 [Source:HGNC Symbol;Acc:HGNC:28639]
ENSG00000123815	477.344817	404.907512	624.772028	598.278983	-0.472133411	0.0067459	0.0276343	COQ8B	coenzyme Q8B [Source:HGNC Symbol;Acc:HGNC:19041]
ENSG00000198546	179.127334	128.471749	243.376625	259.751017	-0.712068345	0.0067539	0.0276606	ZNF511	zinc finger protein 511 [Source:HGNC Symbol;Acc:HGNC:28445]
ENSG00000237624	26.5738352	31.8964342	71.6433354	71.3250745	-1.288046069	0.0067569	0.027666	OXCT2P1	3-oxoacid CoA-transferase 2 pseudogene 1 [Source:HGNC Symbol;Acc:HGNC:21627]
ENSG00000148798	1066.89027	1139.41151	908.184635	764.349305	0.399724045	0.0067603	0.0276733	INA	internexin neuronal intermediate filament protein alpha [Source:HGNC Symbol;Acc:HGNC:6057]
ENSG00000139726	1707.61497	2064.4081	2795.14366	2171.68883	-0.396683287	0.0067666	0.0276905	DENR	density regulated re-initiation and release factor [Source:HGNC Symbol;Acc:HGNC:2769]
ENSG00000156970	1061.96919	1240.41689	1561.40328	1420.11417	-0.372423287	0.0067678	0.0276905	BUB1B	BUB1 mitotic checkpoint serine/threonine kinase B [Source:HGNC Symbol;Acc:HGNC:1149]
ENSG00000095203	321.838671	360.606909	250.751674	194.813263	0.61540042	0.0067715	0.0276991	EPB41L4B	erythrocyte membrane protein band 4.1 like 4B [Source:HGNC Symbol;Acc:HGNC:19818]
ENSG00000133313	786.388678	600.716178	887.113065	1015.5839	-0.456918601	0.0067989	0.0278045	CNDP2	carnosine dipeptidase 2 [Source:HGNC Symbol;Acc:HGNC:24437]
ENSG00000103351	99.4058279	100.119363	165.411819	180.97407	-0.795699186	0.0068023	0.0278114	CLUAP1	clusterin associated protein 1 [Source:HGNC Symbol;Acc:HGNC:19009]
ENSG00000279821	124.011231	85.0571579	44.2502954	53.2276675	1.099265952	0.0068106	0.0278386	AC145098.2	TEC
ENSG00000175970	1126.92746	1140.29752	1451.83112	1422.24328	-0.342129685	0.0068362	0.0279348	UNC119B	unc-119 lipid binding chaperone B [Source:HGNC Symbol;Acc:HGNC:16488]
ENSG00000168067	702.730308	693.747444	513.092711	523.760248	0.429538282	0.0068374	0.0279348	MAP4K2	mitogen-activated protein kinase kinase kinase kinase 2 [Source:HGNC Symbol;Acc:HGNC:6864]
ENSG00000253729	18834.9439	19637.5713	24446.1811	21886.1523	-0.268179006	0.0068573	0.0280092	PRKDC	protein kinase, DNA-activated, catalytic subunit [Source:HGNC Symbol;Acc:HGNC:9413]
ENSG00000183978	618.087722	565.275695	750.147865	839.932593	-0.426601219	0.0068753	0.0280762	COA3	cytochrome c oxidase assembly factor 3 [Source:HGNC Symbol;Acc:HGNC:24990]
ENSG00000213740	224.401275	205.554798	320.287853	326.817879	-0.590417158	0.0068782	0.0280811	SERBP1P1	SERPINE1 mRNA binding protein 1 pseudogene 1 [Source:HGNC Symbol;Acc:HGNC:32247]
ENSG00000123159	2991.03278	2536.65253	3398.84412	3498.12231	-0.319594273	0.0069057	0.0281866	GIPC1	GIPC PDZ domain containing family member 1 [Source:HGNC Symbol;Acc:HGNC:1226]
ENSG00000145901	2806.98437	2504.7561	2140.87144	2130.17125	0.314359115	0.006927	0.0282667	TNIP1	TNFAIP3 interacting protein 1 [Source:HGNC Symbol;Acc:HGNC:16903]
ENSG00000175115	2763.67886	2656.26416	3306.12921	3312.89003	-0.288433681	0.0069314	0.0282779	PACS1	phosphofurin acidic cluster sorting protein 1 [Source:HGNC Symbol;Acc:HGNC:30032]
ENSG00000125755	1855.24738	1476.09609	2068.17452	2234.49748	-0.369648724	0.0069335	0.0282796	SYMPK	symplekin [Source:HGNC Symbol;Acc:HGNC:22935]
ENSG00000166278	47.2423737	48.7306634	93.7684831	106.455335	-1.06055016	0.0069692	0.0284184	C2	complement C2 [Source:HGNC Symbol;Acc:HGNC:1248]
ENSG00000173465	539.350433	471.358417	660.593696	718.573511	-0.449093938	0.0069746	0.0284336	SSSCA1	Sjogren syndrome/scleroderma autoantigen 1 [Source:HGNC Symbol;Acc:HGNC:11328]
ENSG00000102096	787.372895	702.607565	475.163887	601.472643	0.468495394	0.0069893	0.0284863	PIM2	Pim-2 proto-oncogene, serine/threonine kinase [Source:HGNC Symbol;Acc:HGNC:8987]
ENSG00000100347	1255.85977	1103.97103	1414.95587	1643.67037	-0.374573319	0.0070031	0.0285361	SAMM50	SAMM50 sorting and assembly machinery component [Source:HGNC Symbol;Acc:HGNC:24276]
ENSG00000103512	4722.26894	4394.61983	3883.49021	3568.38283	0.290821845	0.007022	0.0286061	NOMO1	NODAL modulator 1 [Source:HGNC Symbol;Acc:HGNC:30060]

ENSG00000155016	211.606465	228.591112	136.9652	133.069169	0.705382223	0.0070529	0.0287239	CYP2U1	cytochrome P450 family 2 subfamily U member 1 [Source:HGNC Symbol;Acc:HGNC:20582]
ENSG00000130811	2263.69707	1974.03487	2550.71346	2763.5805	-0.326891091	0.0070543	0.0287239	EIF3G	eukaryotic translation initiation factor 3 subunit G [Source:HGNC Symbol;Acc:HGNC:3274]
ENSG00000163902	6490.9053	6498.01246	7501.47865	8154.47866	-0.269420538	0.0070807	0.0288244	RPN1	ribophorin I [Source:HGNC Symbol;Acc:HGNC:10381]
ENSG00000198963	95.4689635	115.181568	55.8396585	45.7757941	1.052469287	0.0070928	0.0288666	RORB	RAR related orphan receptor B [Source:HGNC Symbol;Acc:HGNC:10259]
ENSG00000156931	858.236455	985.245413	715.379776	673.862271	0.408594284	0.0071135	0.0289439	VPS8	VPS8, CORVET complex subunit [Source:HGNC Symbol;Acc:HGNC:29122]
ENSG00000233223	213.574898	178.974436	282.359028	323.624218	-0.62752464	0.0071173	0.0289524	AC016876.1	uncharacterized LOC100996842 [Source:NCBI gene;Acc:100996842]
ENSG00000169758	58.068751	53.1607237	13.69652	24.4847271	1.542857247	0.0071515	0.0290834	TMEM266	transmembrane protein 266 [Source:HGNC Symbol;Acc:HGNC:26763]
ENSG00000105325	1558.01412	1251.93504	1853.24452	1777.80409	-0.370452518	0.007153	0.0290834	FZR1	fizzy and cell division cycle 20 related 1 [Source:HGNC Symbol;Acc:HGNC:24824]
ENSG00000248538	30.5106997	26.5803619	6.32147077	5.32276675	2.29274809	0.0071588	0.0291002	AC022784.1	uncharacterized LOC101929128 [Source:NCBI gene;Acc:101929128]
ENSG00000181004	129.916528	150.622051	65.3218647	84.0997147	0.909763591	0.0071719	0.0291463	BBS12	Bardet-Biedl syndrome 12 [Source:HGNC Symbol;Acc:HGNC:26648]
ENSG00000158828	1643.64092	1560.26724	1261.13342	1280.65768	0.33383557	0.0071773	0.0291615	PINK1	PTEN induced putative kinase 1 [Source:HGNC Symbol;Acc:HGNC:14581]
ENSG00000109436	948.784338	958.665051	762.790807	690.895124	0.391916091	0.0071894	0.0292033	TBC1D9	TBC1 domain family member 9 [Source:HGNC Symbol;Acc:HGNC:21710]
ENSG00000131711	1727.29929	1821.6408	1452.8847	1372.20927	0.329216889	0.0072011	0.0292438	MAP1B	microtubule associated protein 1B [Source:HGNC Symbol;Acc:HGNC:6836]
ENSG00000106591	629.898316	671.597143	847.077084	870.804641	-0.400124463	0.0072227	0.0293248	MRPL32	mitochondrial ribosomal protein L32 [Source:HGNC Symbol;Acc:HGNC:14035]
ENSG00000085760	526.555623	605.146238	803.880367	727.089938	-0.43531357	0.0072648	0.0294838	MTIF2	mitochondrial translational initiation factor 2 [Source:HGNC Symbol;Acc:HGNC:7441]
ENSG00000115568	613.166642	528.949201	774.38017	762.220199	-0.428778767	0.0072666	0.0294838	ZNF142	zinc finger protein 142 [Source:HGNC Symbol;Acc:HGNC:12927]
ENSG00000281571	55.1161026	46.0726272	16.8572554	17.0328536	1.577253387	0.0072671	0.0294838	AC241585.2	TEC
ENSG00000084444	430.102444	399.59144	315.01996	248.040931	0.558820019	0.0072693	0.0294854	FAM234B	family with sequence similarity 234 member B [Source:HGNC Symbol;Acc:HGNC:29288]
ENSG00000229809	99.4058279	87.7151941	163.304662	165.005769	-0.812117818	0.0072799	0.0295214	ZNF688	zinc finger protein 688 [Source:HGNC Symbol;Acc:HGNC:30489]
ENSG00000157020	3735.10017	3475.82532	4235.38542	4573.32119	-0.288860133	0.0072838	0.0295303	SEC13	SEC13 homolog, nuclear pore and COPII coat complex component [Source:HGNC Symbol;Acc:HGNC:10697]
ENSG00000245614	114.16907	85.0571579	50.5717662	42.582134	1.095132403	0.0072942	0.0295652	DDX11-AS1	DDX11 antisense RNA 1 [Source:HGNC Symbol;Acc:HGNC:44176]
ENSG00000187045	34.4475641	20.3782774	2.10715692	7.45187345	2.519695476	0.0073305	0.0296991	TMPRSS6	transmembrane serine protease 6 [Source:HGNC Symbol;Acc:HGNC:16517]
ENSG00000171533	237.196084	253.39945	163.304662	144.779256	0.67144629	0.0073307	0.0296991	MAP6	microtubule associated protein 6 [Source:HGNC Symbol;Acc:HGNC:6868]
ENSG00000103510	488.171195	438.575971	590.003939	688.766018	-0.465000845	0.0073815	0.0298917	KAT8	lysine acetyltransferase 8 [Source:HGNC Symbol;Acc:HGNC:17933]
ENSG00000163870	2481.20883	2114.02478	1829.01221	1816.12802	0.333827494	0.0073818	0.0298917	TPRA1	transmembrane protein adipocyte associated 1 [Source:HGNC Symbol;Acc:HGNC:30413]
ENSG00000150995	860.204887	731.845963	650.057911	490.759094	0.480220934	0.0074018	0.0299653	ITPR1	inositol 1,4,5-trisphosphate receptor type 1 [Source:HGNC Symbol;Acc:HGNC:6180]
ENSG00000005001	38.3844286	31.8964342	5.26789231	11.7100869	2.049734754	0.0074078	0.0299826	PRSS22	serine protease 22 [Source:HGNC Symbol;Acc:HGNC:14368]
ENSG00000141030	1588.52481	1672.79077	1936.47721	2155.72053	-0.327237172	0.007427	0.0300529	COPS3	COP9 signalosome subunit 3 [Source:HGNC Symbol;Acc:HGNC:2239]
ENSG00000178773	694.856579	579.451888	803.880367	917.644988	-0.434711538	0.0074688	0.0302148	CPNE7	copine 7 [Source:HGNC Symbol;Acc:HGNC:2320]
ENSG00000090470	619.071938	659.192974	799.666053	900.612134	-0.411231547	0.0074756	0.0302352	PDCD7	programmed cell death 7 [Source:HGNC Symbol;Acc:HGNC:8767]
ENSG00000249437	14.7632418	23.9223257	3.16073539	1.06455335	3.194516335	0.0074794	0.0302431	NAIP	NLR family apoptosis inhibitory protein [Source:HGNC Symbol;Acc:HGNC:7634]
ENSG00000178033	14.7632418	31.0104222	0	5.32276675	3.110191908	0.0074942	0.0302961	CALHM5	calcium homeostasis modulator family member 5 [Source:HGNC Symbol;Acc:HGNC:21568]
ENSG00000212864	21.6527546	22.1503015	52.6789231	64.9377544	-1.424629481	0.0075005	0.0303141	RNF208	ring finger protein 208 [Source:HGNC Symbol;Acc:HGNC:25420]
ENSG00000123364	909.415693	940.058798	682.718844	729.219045	0.389601396	0.0075159	0.0303692	HOXC13	homeobox C13 [Source:HGNC Symbol;Acc:HGNC:5125]
ENSG00000169288	137.790257	200.238726	318.180696	239.524504	-0.72031303	0.0075204	0.03038	MRPL1	mitochondrial ribosomal protein L1 [Source:HGNC Symbol;Acc:HGNC:14275]
ENSG00000177565	841.504781	1046.38024	1524.52803	1086.90897	-0.467592821	0.0075438	0.0304607	TBL1XR1	transducin beta like 1 X-linked receptor 1 [Source:HGNC Symbol;Acc:HGNC:29529]
ENSG00000087884	177.158901	186.948545	103.250689	110.713548	0.767336446	0.007544	0.0304607	AAMDC	adipogenesis associated Mth938 domain containing [Source:HGNC Symbol;Acc:HGNC:30205]
ENSG00000085721	992.089847	1071.18858	1286.4193	1350.9182	-0.353815856	0.0075492	0.0304744	RRN3	RRN3 homolog, RNA polymerase I transcription factor [Source:HGNC Symbol;Acc:HGNC:30346]
ENSG00000128908	988.152983	963.981123	1290.63362	1207.2035	-0.355757115	0.0075561	0.030495	INO80	INO80 complex subunit [Source:HGNC Symbol;Acc:HGNC:26956]
ENSG00000179583	113.184854	116.953592	49.5181877	66.0023077	0.994788925	0.0075704	0.0305453	CIITA	class II major histocompatibility complex transactivator [Source:HGNC Symbol;Acc:HGNC:7067]
ENSG00000161970	491.123843	318.07833	514.14629	716.444405	-0.606188537	0.0075934	0.0306312	RPL26	ribosomal protein L26 [Source:HGNC Symbol;Acc:HGNC:10327]
ENSG00000185043	3029.41721	2557.91682	3429.39789	3528.99436	-0.316904492	0.0076082	0.0306835	CIB1	calcium and integrin binding 1 [Source:HGNC Symbol;Acc:HGNC:16920]
ENSG00000215193	973.389741	984.359401	1216.88312	1283.85134	-0.353087864	0.0076212	0.0307283	PEX26	peroxisomal biogenesis factor 26 [Source:HGNC Symbol;Acc:HGNC:22965]
ENSG00000175581	381.875854	424.399778	537.325016	581.246129	-0.471666262	0.0076241	0.030729	MRPL48	mitochondrial ribosomal protein L48 [Source:HGNC Symbol;Acc:HGNC:16653]
ENSG00000162623	177.158901	258.715522	368.752462	315.107792	-0.64775563	0.0076263	0.030729	TYW3	tRNA-yW synthesizing protein 3 homolog [Source:HGNC Symbol;Acc:HGNC:24757]
ENSG00000102359	140.742905	104.549423	68.4826001	55.3567742	0.984431537	0.0076268	0.030729	SRPX2	sushi repeat containing protein X-linked 2 [Source:HGNC Symbol;Acc:HGNC:30668]
ENSG00000150457	438.960389	442.120019	594.218253	606.79541	-0.446854521	0.0076525	0.0308251	LATS2	large tumor suppressor kinase 2 [Source:HGNC Symbol;Acc:HGNC:6515]
ENSG00000111837	30.5106997	24.8083377	3.16073539	7.45187345	2.38240902	0.0076574	0.0308376	MAK	male germ cell associated kinase [Source:HGNC Symbol;Acc:HGNC:6816]
ENSG00000115355	1621.00395	1952.77058	1491.8671	1250.85019	0.3821694	0.0076592	0.0308377	CCDC88A	coiled-coil domain containing 88A [Source:HGNC Symbol;Acc:HGNC:25523]
ENSG00000090006	3072.72272	2731.57519	2325.24767	2364.37299	0.307427616	0.0076624	0.0308429	LTBP4	latent transforming growth factor beta binding protein 4 [Source:HGNC Symbol;Acc:HGNC:6717]
ENSG00000187446	4128.78662	4196.15312	3360.91529	3511.9615	0.276562235	0.0076721	0.0308747	CHP1	calcineurin like EF-hand protein 1 [Source:HGNC Symbol;Acc:HGNC:17433]

ENSG00000100167	459.628927	378.32715	260.23388	308.720472	0.557861338	0.0076769	0.0308795	3-Sep	septin 3 [Source:HGNC Symbol;Acc:HGNC:10750]
ENSG00000280435	61.0213993	61.1348323	13.69652	29.8074938	1.490322323	0.0076769	0.0308795	AC006058.4	TEC
ENSG00000162522	3591.40462	3248.12022	4153.2063	4193.27565	-0.287456351	0.0076848	0.0309038	KIAA1522	KIAA1522 [Source:HGNC Symbol;Acc:HGNC:29301]
ENSG00000157625	700.761876	826.649254	630.03992	446.047854	0.505601724	0.0076951	0.0309378	TAB3	TGF-beta activated kinase 1 (MAP3K7) binding protein 3 [Source:HGNC Symbol;Acc:HGNC:30681]
ENSG00000088205	1538.32979	1938.59439	2297.85463	2170.62428	-0.361455276	0.0077028	0.0309613	DDX18	DEAD-box helicase 18 [Source:HGNC Symbol;Acc:HGNC:2741]
ENSG00000136244	288.375323	325.166427	201.233486	203.32969	0.601396152	0.0077051	0.0309633	IL6	interleukin 6 [Source:HGNC Symbol;Acc:HGNC:6018]
ENSG00000130487	23.6211868	25.6943498	64.2682862	61.7440943	-1.352575812	0.007708	0.0309675	KLHDC7B	kelch domain containing 7B [Source:HGNC Symbol;Acc:HGNC:25145]
ENSG00000244038	5885.61239	5328.47654	6771.34878	6784.3985	-0.273715754	0.0077111	0.0309719	DDOST	dolichyl-diphosphooligosaccharide--protein glycosyltransferase non-catalytic subunit [Source:HGNC Symbol;Acc:HGNC:2728]
ENSG00000168439	8374.69495	7825.25853	9179.82914	10584.854	-0.28697818	0.0077127	0.0309719	STIP1	stress induced phosphoprotein 1 [Source:HGNC Symbol;Acc:HGNC:11387]
ENSG00000170315	5350.19882	4702.95202	5939.02179	6357.51261	-0.290754769	0.0077295	0.0310317	UBB	ubiquitin B [Source:HGNC Symbol;Acc:HGNC:12463]
ENSG00000125249	381.875854	466.928357	320.287853	251.234591	0.571257724	0.0077347	0.0310455	RAP2A	RAP2A, member of RAS oncogene family [Source:HGNC Symbol;Acc:HGNC:9861]
ENSG00000171453	318.886022	288.839932	406.681286	467.338921	-0.524784238	0.0077731	0.0311922	POLR1C	RNA polymerase I and III subunit C [Source:HGNC Symbol;Acc:HGNC:20194]
ENSG00000205155	171.253605	171.000328	229.680105	317.236898	-0.676111176	0.0077754	0.0311938	PSENEN	presenilin enhancer, gamma-secretase subunit [Source:HGNC Symbol;Acc:HGNC:30100]
ENSG00000156273	547.224162	632.612612	445.66369	405.594826	0.471455243	0.0077892	0.0312391	BACH1	BTB domain and CNC homolog 1 [Source:HGNC Symbol;Acc:HGNC:935]
ENSG00000011260	947.800122	1151.81568	1500.29573	1268.94759	-0.398807407	0.0077904	0.0312391	UTP18	UTP18, small subunit processome component [Source:HGNC Symbol;Acc:HGNC:24274]
ENSG00000119655	2485.1457	2401.9787	1885.90545	2059.91073	0.308611822	0.0078085	0.0313045	NPC2	NPC intracellular cholesterol transporter 2 [Source:HGNC Symbol;Acc:HGNC:14537]
ENSG00000197582	45.2739414	35.4404825	81.1255416	95.8098015	-1.134537165	0.0078205	0.0313452	GPX1P1	glutathione peroxidase pseudogene 1 [Source:HGNC Symbol;Acc:HGNC:4560]
ENSG00000117620	187.985279	248.083377	145.393828	112.842655	0.756883058	0.0078259	0.0313593	SLC35A3	solute carrier family 35 member A3 [Source:HGNC Symbol;Acc:HGNC:11023]
ENSG00000240399	35.4317803	24.8083377	4.21431385	8.5164268	2.241916306	0.0078371	0.0313965	AC004801.2	ribosomal protein L37 (RPL37) pseudogene
ENSG00000166189	570.845349	588.312009	776.487327	761.155645	-0.407492137	0.0078811	0.0315632	HPS6	HPS6, biogenesis of lysosomal organelles complex 2 subunit 3 [Source:HGNC Symbol;Acc:HGNC:18817]
ENSG00000148468	226.369707	245.425341	136.9652	157.553896	0.680318016	0.007883	0.0315632	FAM171A1	family with sequence similarity 171 member A1 [Source:HGNC Symbol;Acc:HGNC:23522]
ENSG00000177076	41.337077	51.3886996	14.7500985	14.9037469	1.645805319	0.0078845	0.0315632	ACER2	alkaline ceramidase 2 [Source:HGNC Symbol;Acc:HGNC:23675]
ENSG00000234420	355.302019	388.959295	257.073145	252.299144	0.547514308	0.0078861	0.0315632	ZNF37BP	zinc finger protein 37B, pseudogene [Source:HGNC Symbol;Acc:HGNC:13103]
ENSG00000125656	1363.13932	1105.74305	1561.40328	1629.83118	-0.370881009	0.0079078	0.0316425	CLPP	caseinolytic mitochondrial matrix peptidase proteolytic subunit [Source:HGNC Symbol;Acc:HGNC:2084]
ENSG00000260528	50.195022	30.1244101	10.5357846	11.7100869	1.850109306	0.0079179	0.0316752	FAM157C	family with sequence similarity 157 member C [Source:HGNC Symbol;Acc:HGNC:34081]
ENSG00000164713	1377.90257	1453.94579	1680.45765	1897.03407	-0.336996914	0.0079241	0.031689	BRI3	brain protein I3 [Source:HGNC Symbol;Acc:HGNC:1109]
ENSG00000231312	108.263773	103.663411	40.0359816	61.7440943	1.05830966	0.0079251	0.031689	AC007388.1	uncharacterized LOC728730 [Source:NCBI gene;Acc:728730]
ENSG00000114166	195.859008	202.896762	104.304268	133.069169	0.748720738	0.0079309	0.031703	KAT2B	lysine acetyltransferase 2B [Source:HGNC Symbol;Acc:HGNC:8638]
ENSG00000107951	229.322356	248.969389	355.055942	349.173499	-0.557602013	0.0079323	0.031703	MTPAP	mitochondrial poly(A) polymerase [Source:HGNC Symbol;Acc:HGNC:25532]
ENSG00000111737	2112.12779	1828.7289	2414.80184	2512.34591	-0.322597843	0.0079443	0.0317432	RAB35	RAB35, member RAS oncogene family [Source:HGNC Symbol;Acc:HGNC:9774]
ENSG00000198369	907.447261	921.452544	735.397767	655.764864	0.394682111	0.0079544	0.0317763	SPRED2	sprouty related EVH1 domain containing 2 [Source:HGNC Symbol;Acc:HGNC:17722]
ENSG00000255135	196.843224	175.430388	110.625739	109.648995	0.756455135	0.0079589	0.0317865	AP002360.1	novel transcript
ENSG00000117632	3609.12051	4809.27347	5398.53604	5342.99326	-0.351299058	0.0079647	0.0318024	STMN1	stathmin 1 [Source:HGNC Symbol;Acc:HGNC:6510]
ENSG00000080845	1138.73805	1119.03323	772.273013	945.323375	0.394541407	0.0079684	0.0318095	DLGAP4	DLG associated protein 4 [Source:HGNC Symbol;Acc:HGNC:24476]
ENSG00000140829	1279.48095	1046.38024	1490.81352	1514.85942	-0.370566929	0.0079748	0.0318277	DHX38	DEAH-box helicase 38 [Source:HGNC Symbol;Acc:HGNC:17211]
ENSG00000148339	831.66262	802.726928	626.879185	612.118176	0.399420329	0.0079785	0.0318347	SLC25A25	solute carrier family 25 member 25 [Source:HGNC Symbol;Acc:HGNC:20663]
ENSG00000168010	285.422674	277.321775	181.215496	186.296836	0.614561549	0.0079878	0.0318646	ATG16L2	autophagy related 16 like 2 [Source:HGNC Symbol;Acc:HGNC:25464]
ENSG00000111671	492.108059	371.239054	298.162705	287.429405	0.558906489	0.0080088	0.0319394	SPSB2	splA/ryanodine receptor domain and SOCS box containing 2 [Source:HGNC Symbol;Acc:HGNC:29522]
ENSG00000163479	2738.08924	2803.34216	3267.14681	3494.92865	-0.287140144	0.0080104	0.0319394	SSR2	signal sequence receptor subunit 2 [Source:HGNC Symbol;Acc:HGNC:11324]
ENSG00000091651	663.361664	637.042672	791.237425	945.323375	-0.417387735	0.0080124	0.0319398	ORC6	origin recognition complex subunit 6 [Source:HGNC Symbol;Acc:HGNC:17151]
ENSG00000113712	2244.01275	2390.46054	3148.09245	2659.25427	-0.325372579	0.0080314	0.0320081	CSNK1A1	casein kinase 1 alpha 1 [Source:HGNC Symbol;Acc:HGNC:2451]
ENSG00000125637	254.911975	210.870871	330.823637	365.141799	-0.580531067	0.0080646	0.0321328	PSD4	pleckstrin and Sec7 domain containing 4 [Source:HGNC Symbol;Acc:HGNC:19096]
ENSG00000176225	792.293975	934.742725	663.754431	631.280137	0.415867551	0.0080665	0.032133	RTTN	rotatin [Source:HGNC Symbol;Acc:HGNC:18654]
ENSG00000160404	305.106997	316.306306	448.824425	432.20866	-0.503430413	0.0080731	0.0321492	TOR2A	torsin family 2 member A [Source:HGNC Symbol;Acc:HGNC:11996]
ENSG00000136720	1130.86432	1025.11596	850.237819	812.254206	0.374631508	0.0080744	0.0321492	HS6ST1	heparan sulfate 6-O-sulfotransferase 1 [Source:HGNC Symbol;Acc:HGNC:5201]
ENSG00000003147	46.2581576	60.2488202	25.2858831	6.3873201	1.749283442	0.008086	0.0321878	ICA1	islet cell autoantigen 1 [Source:HGNC Symbol;Acc:HGNC:5343]
ENSG00000094841	502.934436	485.53461	370.859619	334.269752	0.487077618	0.008096	0.0322201	UPRT	uracil phosphoribosyltransferase homolog [Source:HGNC Symbol;Acc:HGNC:28334]
ENSG00000167548	4017.57019	3583.91879	5201.51687	4312.50562	-0.323942683	0.0081183	0.032301	KMT2D	lysine methyltransferase 2D [Source:HGNC Symbol;Acc:HGNC:7133]
ENSG00000159082	507.855517	511.22896	396.145502	325.753325	0.497310833	0.0081248	0.0323195	SYNJ1	synaptojanin 1 [Source:HGNC Symbol;Acc:HGNC:11503]
ENSG00000017260	3010.71711	3530.75807	2716.12528	2531.50787	0.318183914	0.0081293	0.0323241	ATP2C1	ATPase secretory pathway Ca2+ transporting 1 [Source:HGNC Symbol;Acc:HGNC:13211]
ENSG00000168522	511.792381	510.342948	679.558108	689.830571	-0.421954601	0.0081298	0.0323241	FNTA	farnesyltransferase, CAAX box, alpha [Source:HGNC Symbol;Acc:HGNC:3782]

ENSG00000147164	2202.67567	2081.24233	2586.53512	2677.35168	-0.297343167	0.0081503	0.0323957	SNX12	sorting nexin 12 [Source:HGNC Symbol;Acc:HGNC:14976]
ENSG00000100106	1725.33086	1556.72319	2016.54918	2080.13725	-0.320164101	0.0081517	0.0323957	TRIOBP	TRIO and F-actin binding protein [Source:HGNC Symbol;Acc:HGNC:17009]
ENSG00000171316	976.342389	880.695989	724.861982	689.830571	0.392108136	0.008166	0.0324452	CHD7	chromodomain helicase DNA binding protein 7 [Source:HGNC Symbol;Acc:HGNC:20626]
ENSG00000174156	10.8263773	7.08809649	0	0	5.52150722	0.008187	1	GSTA3	glutathione S-transferase alpha 3 [Source:HGNC Symbol;Acc:HGNC:4628]
ENSG00000132429	49.2108059	79.7410856	27.39304	21.291067	1.407173074	0.0081941	0.032549	POPDC3	popeye domain containing 3 [Source:HGNC Symbol;Acc:HGNC:17649]
ENSG00000082515	662.377448	719.441794	797.558896	1106.07093	-0.461805088	0.0082	0.0325648	MRPL22	mitochondrial ribosomal protein L22 [Source:HGNC Symbol;Acc:HGNC:14480]
ENSG00000238160	11.8105934	6.20208443	0	0	5.52870724	0.0082065	1	AC116366.2	novel transcript
ENSG00000211459	9646.30217	10292.8021	7748.01601	8719.75649	0.276010014	0.0082151	0.0326172	MT-RNR1	mitochondrially encoded 12S RNA [Source:HGNC Symbol;Acc:HGNC:7470]
ENSG00000265778	32.4791319	37.2125066	8.4286277	9.58098015	1.953035313	0.0082208	0.0326323	AC018413.1	uncharacterized LOC101927989 [Source:NCBI gene;Acc:101927989]
ENSG00000269927	64.9582638	56.704772	17.9108339	27.6783871	1.415964222	0.0082261	0.0326454	AC004817.3	novel transcript
ENSG00000236088	258.848839	272.891715	156.983191	184.16773	0.640698453	0.0082458	0.032716	COX10-AS1	COX10 antisense RNA 1 [Source:HGNC Symbol;Acc:HGNC:38873]
ENSG00000109534	379.907422	390.731319	525.735653	537.599442	-0.464287023	0.0082515	0.0327309	GAR1	GAR1 ribonucleoprotein [Source:HGNC Symbol;Acc:HGNC:14264]
ENSG00000180035	307.075429	233.907184	392.984766	405.594826	-0.563432096	0.0082747	0.0328154	ZNF48	zinc finger protein 48 [Source:HGNC Symbol;Acc:HGNC:13114]
ENSG00000139190	424.197147	411.109597	244.430203	323.624218	0.556323665	0.0082784	0.0328221	VAMP1	vesicle associated membrane protein 1 [Source:HGNC Symbol;Acc:HGNC:12642]
ENSG00000175110	733.241008	793.866807	938.73841	1062.42424	-0.389640368	0.0082811	0.0328254	MRPS22	mitochondrial ribosomal protein S22 [Source:HGNC Symbol;Acc:HGNC:14508]
ENSG00000221968	2957.56944	2621.70969	2184.06815	2314.33898	0.310447045	0.0082852	0.0328339	FADS3	fatty acid desaturase 3 [Source:HGNC Symbol;Acc:HGNC:3576]
ENSG00000136193	3267.59751	3294.19285	4220.63532	3790.87448	-0.287988011	0.0082917	0.0328517	SCRN1	secernin 1 [Source:HGNC Symbol;Acc:HGNC:22192]
ENSG00000233762	322.822887	227.7051	426.699277	397.0784	-0.583335098	0.0082959	0.0328608	AC007969.1	ribosomal protein S15 (RPS15) pseudogene
ENSG00000157168	248.022462	256.057486	173.840446	145.843809	0.65702392	0.0083147	0.0329277	NRG1	neuregulin 1 [Source:HGNC Symbol;Acc:HGNC:7997]
ENSG00000148843	1701.70967	1507.99253	1973.35246	2047.13609	-0.325285128	0.0083225	0.0329508	PDCD11	programmed cell death 11 [Source:HGNC Symbol;Acc:HGNC:13408]
ENSG00000169607	438.960389	534.265273	687.986736	647.248437	-0.455289446	0.0083308	0.0329759	CKAP2L	cytoskeleton associated protein 2 like [Source:HGNC Symbol;Acc:HGNC:26877]
ENSG00000134072	131.88496	120.49764	193.858437	220.362544	-0.715411496	0.0083401	0.033005	CAMK1	calcium/calmodulin dependent protein kinase I [Source:HGNC Symbol;Acc:HGNC:1459]
ENSG00000104131	1132.83275	1520.3967	2052.37084	1553.18334	-0.441941517	0.0083474	0.0330256	EIF3J	eukaryotic translation initiation factor 3 subunit J [Source:HGNC Symbol;Acc:HGNC:3270]
ENSG00000068903	817.883594	796.524844	600.539724	623.828263	0.398881596	0.0083492	0.0330256	SIRT2	sirtuin 2 [Source:HGNC Symbol;Acc:HGNC:10886]
ENSG00000141756	2575.69358	2171.61556	2984.78778	2937.10269	-0.31931077	0.008362	0.0330682	FKBP10	FK506 binding protein 10 [Source:HGNC Symbol;Acc:HGNC:18169]
ENSG00000185033	3053.0384	2547.28468	3621.14917	3375.69867	-0.321525541	0.0083643	0.0330698	SEMA4B	semaphorin 4B [Source:HGNC Symbol;Acc:HGNC:10730]
ENSG00000123983	2307.00258	2545.51265	2113.4784	1662.83233	0.361860497	0.0083743	0.0331016	ACSL3	acyl-CoA synthetase long chain family member 3 [Source:HGNC Symbol;Acc:HGNC:3570]
ENSG00000126458	969.452876	902.846291	700.629677	733.477258	0.384412508	0.0083767	0.0331033	RRAS	RAS related [Source:HGNC Symbol;Acc:HGNC:10447]
ENSG00000102387	303.138564	335.798571	208.608536	218.233437	0.582510745	0.0083823	0.0331176	TAF7L	TATA-box binding protein associated factor 7 like [Source:HGNC Symbol;Acc:HGNC:11548]
ENSG00000249602	26.5738352	5.31607237	1.05357846	0	4.908792571	0.0084081	0.0332118	AL589765.4	novel transcript
ENSG00000139405	887.762939	821.333181	1152.61484	1057.10148	-0.370996421	0.0084196	0.0332493	RITA1	RBPJ interacting and tubulin associated 1 [Source:HGNC Symbol;Acc:HGNC:25925]
ENSG00000114790	125.979663	121.383652	69.5361785	60.679541	0.925403845	0.0084389	0.033318	ARHGEF26	Rho guanine nucleotide exchange factor 26 [Source:HGNC Symbol;Acc:HGNC:24490]
ENSG00000183671	32.4791319	30.1244101	73.7504924	72.3896278	-1.223811331	0.008445	0.033334	GPR1	G protein-coupled receptor 1 [Source:HGNC Symbol;Acc:HGNC:4463]
ENSG00000172725	2008.7851	1764.93603	2308.39041	2394.18048	-0.317798713	0.0084679	0.0334161	CORO1B	coronin 1B [Source:HGNC Symbol;Acc:HGNC:2253]
ENSG00000127329	143.695553	152.394075	238.108732	231.008077	-0.663483422	0.0084697	0.0334161	PTPRB	protein tyrosine phosphatase, receptor type B [Source:HGNC Symbol;Acc:HGNC:9665]
ENSG00000153071	798.199272	783.234663	596.32541	602.537196	0.399494162	0.0084744	0.0334266	DAB2	DAB2, clathrin adaptor protein [Source:HGNC Symbol;Acc:HGNC:2662]
ENSG00000114126	1273.57566	1420.27734	1084.13224	1024.10032	0.35394841	0.0084808	0.0334441	TFDP2	transcription factor Dp-2 [Source:HGNC Symbol;Acc:HGNC:11751]
ENSG00000105619	118.105934	84.1711459	156.983191	200.13603	-0.822380153	0.0084847	0.0334512	TFPT	TCF3 fusion partner [Source:HGNC Symbol;Acc:HGNC:13630]
ENSG00000122873	1039.33222	1214.72254	854.452133	878.256514	0.380003512	0.0084883	0.0334512	CISD1	CDGSH iron sulfur domain 1 [Source:HGNC Symbol;Acc:HGNC:30880]
ENSG00000162706	18.7001062	13.2901809	2.10715692	0	3.918122626	0.0084885	0.0334512	CADM3	cell adhesion molecule 3 [Source:HGNC Symbol;Acc:HGNC:17601]
ENSG00000278864	149.60085	98.3473389	66.3754431	58.5504343	0.986973473	0.0084923	0.0334585	AC055811.4	novel transcript
ENSG00000132164	96.4531796	81.5131097	152.768877	160.747556	-0.818291712	0.0085319	0.0336067	SLC6A11	solute carrier family 6 member 11 [Source:HGNC Symbol;Acc:HGNC:11044]
ENSG00000137642	2032.40628	2080.35632	1819.53	1277.46402	0.409197746	0.008534	0.0336071	SORL1	sortilin related receptor 1 [Source:HGNC Symbol;Acc:HGNC:11185]
ENSG00000169871	1292.27576	1047.26626	1557.18897	1470.14818	-0.372494101	0.0085363	0.033608	TRIM56	tripartite motif containing 56 [Source:HGNC Symbol;Acc:HGNC:19028]
ENSG00000181789	4717.34785	4396.39185	5406.96467	5578.25956	-0.2695532	0.0085389	0.0336107	COPG1	coatomer protein complex subunit gamma 1 [Source:HGNC Symbol;Acc:HGNC:2236]
ENSG00000164379	359.238883	318.964342	447.770846	512.050161	-0.501682482	0.0085518	0.0336537	FOXQ1	forkhead box Q1 [Source:HGNC Symbol;Acc:HGNC:20951]
ENSG00000213376	8.85794506	21.2642895	1.05357846	0	4.833675648	0.0085749	0.0337366	GAPDHP71	glyceraldehyde-3-phosphate dehydrogenase pseudogene 71 [Source:HGNC Symbol;Acc:HGNC:4149]
ENSG00000156976	3286.29762	3880.73283	4430.29743	4409.37998	-0.302361872	0.0086129	0.0338783	EIF4A2	eukaryotic translation initiation factor 4A2 [Source:HGNC Symbol;Acc:HGNC:3284]
ENSG00000250312	82.6741539	95.6893027	45.3038739	37.2593673	1.11178567	0.0086528	0.0340272	ZNF718	zinc finger protein 718 [Source:HGNC Symbol;Acc:HGNC:26889]
ENSG00000197728	5810.81196	5500.36288	6558.52593	7054.79505	-0.267330726	0.0086756	0.0341089	RPS26	ribosomal protein S26 [Source:HGNC Symbol;Acc:HGNC:10414]
ENSG00000163162	906.463045	942.716834	1269.56205	1114.58736	-0.366476429	0.0087225	0.0342853	RNF149	ring finger protein 149 [Source:HGNC Symbol;Acc:HGNC:23137]
ENSG00000280179	39.3686447	38.0985187	11.5893631	10.6455335	1.800484862	0.0087257	0.03429	CU639417.4	uncharacterized CH507-42P11.6 [Source:NCBI gene;Acc:102724398]
ENSG00000166503	792.293975	911.706412	1150.50768	1066.68246	-0.379258591	0.0087316	0.0343051	HDGFL3	HDGF like 3 [Source:HGNC Symbol;Acc:HGNC:24937]
ENSG00000177303	954.689635	761.970373	1125.2218	1134.81387	-0.397571826	0.0087612	0.0344074	CASKIN2	CASK interacting protein 2 [Source:HGNC Symbol;Acc:HGNC:18200]
ENSG00000196365	5019.5022	4439.80644	5664.03781	5827.36504	-0.280910956	0.0087632	0.0344074	LONP1	lon peptidase 1, mitochondrial [Source:HGNC Symbol;Acc:HGNC:9479]

ENSG00000126653	488.171195	523.633129	656.379382	703.669765	-0.426314757	0.0087637	0.0344074	NSRP1	nuclear speckle splicing regulatory protein 1 [Source:HGNC Symbol;Acc:HGNC:25305]
ENSG00000179862	873.983913	846.141519	658.486539	656.829417	0.386972317	0.0087767	0.0344504	CITED4	Cbp/p300 interacting transactivator with Glu/Asp rich carboxy-terminal domain 4 [Source:HGNC Symbol;Acc:HGNC:18696]
ENSG00000168701	748.988466	591.856057	882.898751	915.515881	-0.42454513	0.0087945	0.0345123	TMEM208	transmembrane protein 208 [Source:HGNC Symbol;Acc:HGNC:25015]
ENSG00000101076	18.7001062	10.6321447	40.0359816	52.1631142	-1.658407202	0.0088105	0.0345669	HNF4A	hepatocyte nuclear factor 4 alpha [Source:HGNC Symbol;Acc:HGNC:5024]
ENSG00000205238	62.0056154	51.3886996	10.5357846	27.6783871	1.569371163	0.0088125	0.0345669	SPDYE2	speedy/RINGO cell cycle regulator family member E2 [Source:HGNC Symbol;Acc:HGNC:33841]
ENSG00000087088	1591.47746	1259.90915	1823.74432	1844.87096	-0.364189032	0.0088189	0.0345837	BAX	BCL2 associated X, apoptosis regulator [Source:HGNC Symbol;Acc:HGNC:959]
ENSG00000124097	48.2265898	51.3886996	20.0179908	13.8391936	1.556763911	0.0088449	0.0346777	HMGB1P1	high mobility group box 1 pseudogene 1 [Source:HGNC Symbol;Acc:HGNC:4993]
ENSG00000237989	174.206253	188.720569	258.126723	300.204045	-0.62079774	0.0088828	0.0348121	LINC01679	long intergenic non-protein coding RNA 1679 [Source:HGNC Symbol;Acc:HGNC:52469]
ENSG00000114861	236.211868	259.601534	145.393828	169.263983	0.65660175	0.0088833	0.0348121	FOXP1	forkhead box P1 [Source:HGNC Symbol;Acc:HGNC:3823]
ENSG00000134644	2218.42313	2308.06142	2854.14405	2689.06176	-0.292240741	0.008898	0.0348553	PUM1	pumilio RNA binding family member 1 [Source:HGNC Symbol;Acc:HGNC:14957]
ENSG00000121104	111.216421	92.1452544	40.0359816	57.4858809	1.059690023	0.0088984	0.0348553	FAM117A	family with sequence similarity 117 member A [Source:HGNC Symbol;Acc:HGNC:24179]
ENSG00000124067	1918.23721	1601.0238	1391.77715	1371.14472	0.348641526	0.0089098	0.0348916	SLC12A4	solute carrier family 12 member 4 [Source:HGNC Symbol;Acc:HGNC:10913]
ENSG00000149380	60.0371832	63.7928684	25.2858831	23.4201737	1.34640581	0.0089314	0.0349543	P4HA3	prolyl 4-hydroxylase subunit alpha 3 [Source:HGNC Symbol;Acc:HGNC:30135]
ENSG00000162976	143.695553	159.482171	91.6613262	78.7769479	0.831313376	0.0089316	0.0349543	PQLC3	PQ loop repeat containing 3 [Source:HGNC Symbol;Acc:HGNC:28503]
ENSG00000177410	257.864623	310.104222	363.48457	479.049008	-0.567812373	0.008932	0.0349543	ZFAS1	ZNFX1 antisense RNA 1 [Source:HGNC Symbol;Acc:HGNC:33101]
ENSG00000182963	1218.45955	1253.70707	1015.64964	917.644988	0.354762488	0.0089341	0.0349544	GJC1	gap junction protein gamma 1 [Source:HGNC Symbol;Acc:HGNC:4280]
ENSG00000249115	696.825012	581.223913	826.005514	877.191961	-0.415100014	0.008962	0.0350553	HAUS5	HAUS augmin like complex subunit 5 [Source:HGNC Symbol;Acc:HGNC:29130]
ENSG00000027644	22.6369707	9.74613268	0	2.1291067	3.928876259	0.008971	0.0350826	INSRR	insulin receptor related receptor [Source:HGNC Symbol;Acc:HGNC:6093]
ENSG00000120594	218.495978	267.575643	161.197505	143.714702	0.673653509	0.0089731	0.0350826	PLXDC2	plexin domain containing 2 [Source:HGNC Symbol;Acc:HGNC:21013]
ENSG00000134369	1166.2961	1236.87284	984.042284	888.902047	0.35977743	0.0089914	0.0351461	NAV1	neuron navigator 1 [Source:HGNC Symbol;Acc:HGNC:15989]
ENSG00000184292	111.216421	138.217882	191.75128	221.427097	-0.726427545	0.0089966	0.0351584	TACSTD2	tumor associated calcium signal transducer 2 [Source:HGNC Symbol;Acc:HGNC:11530]
ENSG00000108559	1400.53954	1508.87854	1793.19054	1834.22542	-0.317951066	0.0089994	0.035161	NUP88	nucleoporin 88 [Source:HGNC Symbol;Acc:HGNC:8067]
ENSG00000172137	109.247989	107.207459	1.05357846	15.9683003	3.669398101	0.0090081	0.0351871	CALB2	calbindin 2 [Source:HGNC Symbol;Acc:HGNC:1435]
ENSG00000178741	3912.25907	3780.61347	4537.76244	4725.55232	-0.268061491	0.0090258	0.0352464	COX5A	cytochrome c oxidase subunit 5A [Source:HGNC Symbol;Acc:HGNC:2267]
ENSG00000198805	1729.26772	1644.43839	2024.9778	2153.59143	-0.308813976	0.0090275	0.0352464	PNP	purine nucleoside phosphorylase [Source:HGNC Symbol;Acc:HGNC:7892]
ENSG00000144589	214.559114	156.824135	269.716086	311.914132	-0.649078841	0.0090636	0.0353793	STK11IP	serine/threonine kinase 11 interacting protein [Source:HGNC Symbol;Acc:HGNC:19184]
ENSG00000197879	4804.94309	4197.92515	5501.78673	5456.90047	-0.28380596	0.0090727	0.0354064	MYO1C	myosin IC [Source:HGNC Symbol;Acc:HGNC:7597]
ENSG00000157540	769.657004	803.61294	1032.50689	996.421936	-0.366763732	0.0090811	0.035431	DYRK1A	dual specificity tyrosine phosphorylation regulated kinase 1A [Source:HGNC Symbol;Acc:HGNC:3091]
ENSG00000120658	161.411443	157.710147	70.589757	106.455335	0.850216484	0.0091179	0.0355665	ENOX1	ecto-NOX disulfide-thiol exchanger 1 [Source:HGNC Symbol;Acc:HGNC:25474]
ENSG00000233429	200.780088	209.098847	126.429415	125.617295	0.701714154	0.0091336	0.0356195	HOTAIRM1	HOXA transcript antisense RNA, myeloid-specific 1 [Source:HGNC Symbol;Acc:HGNC:37117]
ENSG00000108784	538.366217	467.814369	657.43296	701.540658	-0.434332161	0.0091393	0.0356336	NAGLU	N-acetyl-alpha-glucosaminidase [Source:HGNC Symbol;Acc:HGNC:7632]
ENSG00000162402	1954.65321	1958.97267	2662.39277	2239.82025	-0.324959468	0.0091488	0.0356623	USP24	ubiquitin specific peptidase 24 [Source:HGNC Symbol;Acc:HGNC:12623]
ENSG00000119777	1439.90818	1310.41184	1862.72672	1623.44386	-0.342372869	0.0091528	0.0356653	TMEM214	transmembrane protein 214 [Source:HGNC Symbol;Acc:HGNC:25983]
ENSG00000163975	1086.57459	988.789461	753.308601	840.997147	0.380166868	0.0091538	0.0356653	MELTF	melanotransferrin [Source:HGNC Symbol;Acc:HGNC:7037]
ENSG00000136522	658.440583	818.675145	944.006302	1020.90666	-0.410792018	0.0091578	0.0356692	MRPL47	mitochondrial ribosomal protein L47 [Source:HGNC Symbol;Acc:HGNC:16652]
ENSG00000072864	643.677341	732.731975	848.130662	977.259976	-0.406678781	0.009159	0.0356692	NDE1	nudE neurodevelopment protein 1 [Source:HGNC Symbol;Acc:HGNC:17619]
ENSG00000093144	477.344817	676.913215	815.46973	790.963139	-0.475592492	0.0091616	0.0356712	ECHDC1	ethylmalonyl-CoA decarboxylase 1 [Source:HGNC Symbol;Acc:HGNC:21489]
ENSG00000172840	369.081044	350.860776	258.126723	235.26629	0.54482765	0.0091666	0.0356824	PDP2	pyruvate dehyrogenase phosphatase catalytic subunit 2 [Source:HGNC Symbol;Acc:HGNC:30263]
ENSG00000010244	2217.43891	2354.13405	2738.25042	2855.13209	-0.290866149	0.0091711	0.0356895	ZNF207	zinc finger protein 207 [Source:HGNC Symbol;Acc:HGNC:12998]
ENSG00000125826	1937.92154	1764.05002	2199.87183	2401.63236	-0.31404689	0.0091726	0.0356895	RBCK1	RANBP2-type and C3HC4-type zinc finger containing 1 [Source:HGNC Symbol;Acc:HGNC:15864]
ENSG00000253304	187.001062	169.228304	279.198293	266.138338	-0.615090457	0.0091778	0.0357012	TMEM200B	transmembrane protein 200B [Source:HGNC Symbol;Acc:HGNC:33785]
ENSG00000275052	1062.95341	1235.10081	1698.36848	1327.49803	-0.396581249	0.0092082	0.0358115	PPP4R3B	protein phosphatase 4 regulatory subunit 3B [Source:HGNC Symbol;Acc:HGNC:29267]
ENSG00000125319	246.05403	238.337245	351.895206	353.431712	-0.542336107	0.0092119	0.0358176	C17orf53	chromosome 17 open reading frame 53 [Source:HGNC Symbol;Acc:HGNC:28460]
ENSG00000204219	677.140689	635.270648	860.773604	854.83634	-0.386826778	0.0092164	0.0358265	TCEA3	transcription elongation factor A3 [Source:HGNC Symbol;Acc:HGNC:11615]
ENSG00000159592	1703.6781	1753.41787	2176.6931	2086.52457	-0.30230358	0.0092222	0.0358409	GPBP1L1	GC-rich promoter binding protein 1 like 1 [Source:HGNC Symbol;Acc:HGNC:28843]
ENSG00000185800	865.125968	720.327806	1039.88194	1035.81041	-0.389452274	0.0092347	0.0358814	DMWD	DM1 locus, WD repeat containing [Source:HGNC Symbol;Acc:HGNC:2936]
ENSG00000247596	1373.9657	1184.59813	1021.97111	965.549889	0.363896008	0.0092593	0.0359687	TWF2	twinfilin actin binding protein 2 [Source:HGNC Symbol;Acc:HGNC:9621]
ENSG00000124767	2147.55957	2922.06778	2108.2105	1725.64098	0.403422168	0.0092718	0.0360088	GLO1	glyoxalase I [Source:HGNC Symbol;Acc:HGNC:4323]
ENSG00000001460	316.91759	292.38398	214.930006	189.490496	0.590809764	0.0092922	0.0360799	STPG1	sperm tail PG-rich repeat containing 1 [Source:HGNC Symbol;Acc:HGNC:28070]
ENSG00000094914	1080.6693	1023.34393	1244.27616	1457.37354	-0.36084396	0.0093004	0.0361035	AAAS	aladin WD repeat nucleoporin [Source:HGNC Symbol;Acc:HGNC:13666]

ENSG00000162702	955.673851	1041.06417	813.362573	715.379851	0.385531571	0.0093051	0.0361132	ZNF281	zinc finger protein 281 [Source:HGNC Symbol;Acc:HGNC:13075]
ENSG00000196700	1259.79663	1056.12638	1464.47406	1498.89112	-0.356236213	0.0093148	0.0361367	ZNF512B	zinc finger protein 512B [Source:HGNC Symbol;Acc:HGNC:29212]
ENSG00000179041	625.961451	550.21349	738.558502	833.545273	-0.419160436	0.0093154	0.0361367	RRS1	ribosome biogenesis regulator homolog [Source:HGNC Symbol;Acc:HGNC:17083]
ENSG00000145476	85.6268023	93.0312665	46.3574523	37.2593673	1.095534986	0.0093275	0.0361754	CYP4V2	cytochrome P450 family 4 subfamily V member 2 [Source:HGNC Symbol;Acc:HGNC:23198]
ENSG00000236871	104.326909	48.7306634	31.6073539	28.7429405	1.339647603	0.00933	0.0361768	LINC00106	long intergenic non-protein coding RNA 106 [Source:HGNC Symbol;Acc:HGNC:31843]
ENSG00000171320	308.059645	326.938451	443.556533	449.241514	-0.491195044	0.0093523	0.036255	ESCO2	establishment of sister chromatid cohesion N-acetyltransferase 2 [Source:HGNC Symbol;Acc:HGNC:27230]
ENSG00000171940	1498.96115	1649.75446	1311.70519	1171.00869	0.343064183	0.0093578	0.0362679	ZNF217	zinc finger protein 217 [Source:HGNC Symbol;Acc:HGNC:13009]
ENSG00000181449	5.90529671	11.5181568	0	0	5.485040983	0.0093603	1	SOX2	SRY-box 2 [Source:HGNC Symbol;Acc:HGNC:11195]
ENSG00000112561	302.154348	269.347667	410.8956	405.594826	-0.515424426	0.0093608	0.0362711	TFEB	transcription factor EB [Source:HGNC Symbol;Acc:HGNC:11753]
ENSG00000118689	1007.83731	1149.15764	879.738016	770.736626	0.386495911	0.0093747	0.0363167	FOXO3	forkhead box O3 [Source:HGNC Symbol;Acc:HGNC:3821]
ENSG00000123349	4230.16088	4215.64539	4944.44372	5192.89124	-0.263369497	0.009381	0.0363328	PFDN5	prefoldin subunit 5 [Source:HGNC Symbol;Acc:HGNC:8869]
ENSG00000155158	48.2265898	40.7565548	13.69652	14.9037469	1.63679487	0.0093835	0.036334	TTC39B	tetratricopeptide repeat domain 39B [Source:HGNC Symbol;Acc:HGNC:23704]
ENSG00000068366	1562.9352	1974.92089	1491.8671	1185.91243	0.402189414	0.0093882	0.0363441	ACSL4	acyl-CoA synthetase long chain family member 4 [Source:HGNC Symbol;Acc:HGNC:3571]
ENSG00000137266	427.149795	410.223585	309.752068	277.848424	0.510768212	0.009399	0.0363775	SLC22A23	solute carrier family 22 member 23 [Source:HGNC Symbol;Acc:HGNC:21106]
ENSG00000132470	1081.65351	849.685567	1256.91911	1272.14125	-0.389806353	0.0094025	0.0363828	ITGB4	integrin subunit beta 4 [Source:HGNC Symbol;Acc:HGNC:6158]
ENSG00000170703	22.6369707	16.8342292	3.16073539	2.1291067	2.897492696	0.0094109	0.0364069	TTLL6	tubulin tyrosine ligase like 6 [Source:HGNC Symbol;Acc:HGNC:26664]
ENSG00000198431	16154.9234	17164.7117	14234.8986	13872.1947	0.245466656	0.0094174	0.0364236	TXNRD1	thioredoxin reductase 1 [Source:HGNC Symbol;Acc:HGNC:12437]
ENSG00000171492	1045.23752	1121.69127	1425.49166	1322.17526	-0.342307025	0.0094358	0.0364838	LRRC8D	leucine rich repeat containing 8 VRAC subunit D [Source:HGNC Symbol;Acc:HGNC:16992]
ENSG00000091157	497.02914	492.622706	361.377413	353.431712	0.469305689	0.0094372	0.0364838	WDR7	WD repeat domain 7 [Source:HGNC Symbol;Acc:HGNC:13490]
ENSG00000135454	796.23084	740.706084	595.271831	566.342382	0.403604006	0.0094538	0.0365394	B4GALNT1	beta-1,4-N-acetyl-galactosaminyltransferase 1 [Source:HGNC Symbol;Acc:HGNC:4117]
ENSG00000179859	146.648202	141.76193	202.287065	261.880124	-0.686629398	0.0094879	0.0366605	RNF227	ring finger protein 227 [Source:HGNC Symbol;Acc:HGNC:27571]
ENSG00000117614	472.423737	512.114972	647.950754	673.862271	-0.424534512	0.0094895	0.0366605	SYF2	SYF2 pre-mRNA splicing factor [Source:HGNC Symbol;Acc:HGNC:19824]
ENSG00000100156	132.869176	109.865496	59.0003939	68.1314144	0.932227836	0.0095024	0.0367021	SLC16A8	solute carrier family 16 member 8 [Source:HGNC Symbol;Acc:HGNC:16270]
ENSG00000153015	469.471088	608.690286	735.397767	738.800025	-0.450197316	0.0095214	0.0367669	CWC27	CWC27 spliceosome associated protein homolog [Source:HGNC Symbol;Acc:HGNC:10664]
ENSG00000183808	269.675216	332.254523	414.056336	452.435174	-0.524325676	0.0095248	0.0367719	RBM12B	RNA binding motif protein 12B [Source:HGNC Symbol;Acc:HGNC:32310]
ENSG00000179046	214.559114	168.342292	116.947209	110.713548	0.748861566	0.009529	0.0367795	TRIML2	tripartite motif family like 2 [Source:HGNC Symbol;Acc:HGNC:26378]
ENSG00000173614	103.342692	103.663411	203.340643	152.231129	-0.780596468	0.00954	0.0368135	NMNAT1	nicotinamide nucleotide adenylyltransferase 1 [Source:HGNC Symbol;Acc:HGNC:17877]
ENSG00000060982	188.969495	223.27504	462.520945	232.07263	-0.752165971	0.0095606	0.0368847	BCAT1	branched chain amino acid transaminase 1 [Source:HGNC Symbol;Acc:HGNC:976]
ENSG00000110171	556.082107	439.461983	374.020354	321.495112	0.516413957	0.0095955	0.0370079	TRIM3	tripartite motif containing 3 [Source:HGNC Symbol;Acc:HGNC:10064]
ENSG00000169427	11.8105934	16.8342292	1.05357846	0	4.759500771	0.0095969	0.0370079	KCNK9	potassium two pore domain channel subfamily K member 9 [Source:HGNC Symbol;Acc:HGNC:6283]
ENSG00000152936	15.7474579	24.8083377	1.05357846	4.2582134	2.93666718	0.0095994	0.0370092	LMNTD1	lamin tail domain containing 1 [Source:HGNC Symbol;Acc:HGNC:26683]
ENSG00000060339	1834.57884	2127.31496	2570.73145	2377.14763	-0.320278915	0.0096128	0.0370524	CCAR1	cell division cycle and apoptosis regulator 1 [Source:HGNC Symbol;Acc:HGNC:24236]
ENSG00000150961	1155.46972	1175.73801	930.309782	901.676688	0.347715593	0.0096186	0.0370595	SEC24D	SEC24 homolog D, COPII coat complex component [Source:HGNC Symbol;Acc:HGNC:10706]
ENSG00000236901	242.117165	221.503015	149.608142	143.714702	0.659961666	0.0096191	0.0370595	MIR600HG	MIR600 host gene [Source:HGNC Symbol;Acc:HGNC:23642]
ENSG00000140937	11.8105934	19.4922654	0	2.1291067	3.88459191	0.009628	0.0370855	CDH11	cadherin 11 [Source:HGNC Symbol;Acc:HGNC:1750]
ENSG00000141424	2188.89665	2421.47096	1922.78069	1795.9015	0.310296605	0.0096444	0.0371388	SLC39A6	solute carrier family 39 member 6 [Source:HGNC Symbol;Acc:HGNC:18607]
ENSG00000105409	13.7790257	25.6943498	0	4.2582134	3.218496709	0.0096462	0.0371388	ATP1A3	ATPase Na+/K+ transporting subunit alpha 3 [Source:HGNC Symbol;Acc:HGNC:801]
ENSG00000057608	2574.70937	2938.90201	3387.25476	3379.95689	-0.295293692	0.0096533	0.0371541	GDI2	GDP dissociation inhibitor 2 [Source:HGNC Symbol;Acc:HGNC:4227]
ENSG00000142621	37.4002125	33.6684583	7.37504924	11.7100869	1.89690418	0.0096546	0.0371541	FHAD1	forkhead associated phosphopeptide binding domain 1 [Source:HGNC Symbol;Acc:HGNC:29408]
ENSG00000150459	1354.28138	1390.15292	1586.68916	1899.16318	-0.344876095	0.0096603	0.0371674	SAP18	Sin3A associated protein 18 [Source:HGNC Symbol;Acc:HGNC:10530]
ENSG00000136518	1332.62862	1391.92495	1628.8323	1780.99775	-0.323502318	0.0096659	0.0371805	ACTL6A	actin like 6A [Source:HGNC Symbol;Acc:HGNC:24124]
ENSG00000186104	172.237821	171.88634	93.7684831	108.584442	0.766172818	0.0096753	0.0372078	CYP2R1	cytochrome P450 family 2 subfamily R member 1 [Source:HGNC Symbol;Acc:HGNC:20580]
ENSG00000137965	95.4689635	163.912231	220.197899	224.620757	-0.774894044	0.0096774	0.0372078	IFI44	interferon induced protein 44 [Source:HGNC Symbol;Acc:HGNC:16938]
ENSG00000130962	205.701169	299.472077	181.215496	117.100869	0.760974753	0.0096911	0.0372522	PRRG1	proline rich and Gla domain 1 [Source:HGNC Symbol;Acc:HGNC:9469]
ENSG00000182325	590.529671	556.415575	730.129874	787.769479	-0.404555247	0.0096966	0.0372648	FBXL6	F-box and leucine rich repeat protein 6 [Source:HGNC Symbol;Acc:HGNC:13603]
ENSG00000172164	48.2265898	61.1348323	12.6429415	25.5492804	1.519550341	0.0097126	0.0373124	SNTB1	syntrophin beta 1 [Source:HGNC Symbol;Acc:HGNC:11168]
ENSG00000116353	363.175748	369.46703	485.699671	526.953908	-0.466813222	0.0097155	0.0373124	MECR	mitochondrial trans-2-enoyl-CoA reductase [Source:HGNC Symbol;Acc:HGNC:19691]
ENSG00000149179	600.371832	535.151285	710.111884	809.060546	-0.420433623	0.0097156	0.0373124	C11orf49	chromosome 11 open reading frame 49 [Source:HGNC Symbol;Acc:HGNC:28720]
ENSG00000129646	69.8793444	60.2488202	25.2858831	27.6783871	1.296194983	0.0097229	0.0373318	QRICH2	glutamine rich 2 [Source:HGNC Symbol;Acc:HGNC:25326]
ENSG00000183655	476.360601	429.71585	316.073539	327.882432	0.492216433	0.00974	0.0373866	KLHL25	kelch like family member 25 [Source:HGNC Symbol;Acc:HGNC:25732]
ENSG00000100097	18892.0284	15649.631	20496.3154	21631.7241	-0.286506962	0.0097416	0.0373866	LGALS1	galectin 1 [Source:HGNC Symbol;Acc:HGNC:6561]
ENSG00000109180	768.672788	819.561157	1031.45331	1012.39024	-0.363570788	0.0097546	0.037428	OCIAD1	OCIA domain containing 1 [Source:HGNC Symbol;Acc:HGNC:16074]

ENSG00000087111	2543.21445	2326.66767	2024.9778	1924.71246	0.301932353	0.0097667	0.0374661	PIGS	phosphatidylinositol glycan anchor biosynthesis class S [Source:HGNC Symbol;Acc:HGNC:14937]
ENSG00000157150	705.682957	674.255179	520.46776	516.308375	0.412297702	0.0098116	0.0376296	TIMP4	TIMP metallopeptidase inhibitor 4 [Source:HGNC Symbol;Acc:HGNC:11823]
ENSG00000259956	2671.16254	2324.00964	3031.14524	3123.39953	-0.301412281	0.0098344	0.0377087	RBM15B	RNA binding motif protein 15B [Source:HGNC Symbol;Acc:HGNC:24303]
ENSG00000144158	174.206253	156.824135	218.090742	310.849578	-0.676642869	0.0098533	0.0377725	AL078621.1	small nuclear ribonucleoprotein polypeptide A' (SNRPA1) pseudogene
ENSG00000168610	4060.8757	3885.16289	3290.32554	3304.3736	0.26885371	0.009905	0.0379622	STAT3	signal transducer and activator of transcription 3 [Source:HGNC Symbol;Acc:HGNC:11364]
ENSG00000135916	3129.80726	2509.18616	2214.62193	2257.91766	0.33400126	0.0099143	0.0379891	ITM2C	integral membrane protein 2C [Source:HGNC Symbol;Acc:HGNC:6175]
ENSG00000140553	3399.48247	2809.54425	3762.32869	3944.17016	-0.312009359	0.0099176	0.0379932	UNC45A	unc-45 myosin chaperone A [Source:HGNC Symbol;Acc:HGNC:30594]
ENSG00000185651	1875.91592	2065.29412	2308.39041	2592.18741	-0.314045383	0.0099227	0.0380042	UBE2L3	ubiquitin conjugating enzyme E2 L3 [Source:HGNC Symbol;Acc:HGNC:12488]
ENSG00000177556	999.963576	862.975748	693.254628	727.089938	0.390844933	0.0099293	0.0380208	ATOX1	antioxidant 1 copper chaperone [Source:HGNC Symbol;Acc:HGNC:798]
ENSG00000132275	374.002125	337.570596	517.307025	472.661688	-0.477027274	0.0099403	0.0380542	RRP8	ribosomal RNA processing 8 [Source:HGNC Symbol;Acc:HGNC:29030]
ENSG00000149654	15.7474579	15.0622051	0	2.1291067	3.859718026	0.0099465	0.0380693	CDH22	cadherin 22 [Source:HGNC Symbol;Acc:HGNC:13251]
ENSG00000130821	1569.82471	1361.80054	1807.94064	1872.54934	-0.328624982	0.0099574	0.0380984	SLC6A8	solute carrier family 6 member 8 [Source:HGNC Symbol;Acc:HGNC:11055]
ENSG00000113966	159.443011	127.585737	90.6077478	64.9377544	0.882538778	0.0099586	0.0380984	ARL6	ADP ribosylation factor like GTPase 6 [Source:HGNC Symbol;Acc:HGNC:13210]
ENSG00000183853	2849.30566	2759.92757	3574.79172	3256.4687	-0.284435386	0.0099731	0.0381453	KIRREL1	kirre like nephrin family adhesion molecule 1 [Source:HGNC Symbol;Acc:HGNC:15734]
ENSG00000174945	382.86007	335.798571	566.825213	451.370621	-0.503428353	0.0099898	0.0382005	AMZ1	archaelysin family metallopeptidase 1 [Source:HGNC Symbol;Acc:HGNC:22231]
ENSG00000110218	1296.21263	1293.57761	1048.31057	1002.80926	0.336395944	0.0099922	0.0382011	PANX1	pannexin 1 [Source:HGNC Symbol;Acc:HGNC:8599]
ENSG00000178996	403.528608	426.171802	321.341431	251.234591	0.535198046	0.0100024	0.0382311	SNX18	sorting nexin 18 [Source:HGNC Symbol;Acc:HGNC:19245]
ENSG00000177700	3584.5151	3033.7053	3862.41864	4361.47508	-0.313611522	0.0100313	0.0383331	POLR2L	RNA polymerase II subunit L [Source:HGNC Symbol;Acc:HGNC:9199]
ENSG00000130731	1763.71528	1526.59878	2002.85266	2114.20295	-0.323781013	0.0100413	0.0383628	METTL26	methyltransferase like 26 [Source:HGNC Symbol;Acc:HGNC:14141]
ENSG00000186591	2737.10502	2757.26954	2301.01536	2213.20642	0.283482737	0.0100502	0.0383878	UBE2H	ubiquitin conjugating enzyme E2 H [Source:HGNC Symbol;Acc:HGNC:12484]
ENSG00000167130	589.545455	544.011406	700.629677	813.31876	-0.417790001	0.010076	0.038478	DOLPP1	dolichyldiphosphatase 1 [Source:HGNC Symbol;Acc:HGNC:29565]
ENSG00000140740	2895.56382	3288.87677	3685.41746	3883.49062	-0.291197066	0.0100953	0.0385429	UQCRC2	ubiquinol-cytochrome c reductase core protein 2 [Source:HGNC Symbol;Acc:HGNC:12586]
ENSG00000119321	1421.20807	1294.46362	1672.02902	1720.31821	-0.321289247	0.0101334	0.0386797	FKBP15	FK506 binding protein 15 [Source:HGNC Symbol;Acc:HGNC:23397]
ENSG00000156521	1079.68508	885.12605	1243.22259	1298.75509	-0.372252169	0.0101574	0.0387622	TYSND1	trypsin domain containing 1 [Source:HGNC Symbol;Acc:HGNC:28531]
ENSG00000134215	277.548945	307.446185	418.27065	411.982147	-0.504477327	0.010162	0.0387711	VAV3	vav guanine nucleotide exchange factor 3 [Source:HGNC Symbol;Acc:HGNC:12659]
ENSG00000157045	524.587191	549.327478	351.895206	422.62768	0.471804896	0.01017	0.038793	NTAN1	N-terminal asparagine amidase [Source:HGNC Symbol;Acc:HGNC:29909]
ENSG00000112977	4371.888	4554.102	3644.3279	3782.35805	0.26536493	0.0101803	0.0388234	DAP	death associated protein [Source:HGNC Symbol;Acc:HGNC:2672]
ENSG00000260139	53.1476704	42.528579	20.0179908	11.7100869	1.59074617	0.0101917	0.0388582	CSPG4P13	chondroitin sulfate proteoglycan 4 pseudogene 13 [Source:HGNC Symbol;Acc:HGNC:49195]
ENSG00000166292	9.84216118	7.08809649	0	0	5.440299277	0.0101978	1	TMEM100	transmembrane protein 100 [Source:HGNC Symbol;Acc:HGNC:25607]
ENSG00000165322	350.380938	414.653645	297.109126	211.846117	0.588424389	0.0102173	0.038947	ARHGAP12	Rho GTPase activating protein 12 [Source:HGNC Symbol;Acc:HGNC:16348]
ENSG00000166224	1489.11899	1410.5312	1820.58358	1774.61043	-0.310386878	0.0102226	0.0389584	SGPL1	sphingosine-1-phosphate lyase 1 [Source:HGNC Symbol;Acc:HGNC:10817]
ENSG00000259672	32.4791319	36.3264945	77.9648062	75.5832879	-1.157006565	0.0102274	0.0389677	AC087612.1	novel transcript
ENSG00000177138	14.7632418	13.2901809	1.05357846	0	4.727691119	0.0102357	0.0389907	FAM9B	family with sequence similarity 9 member B [Source:HGNC Symbol;Acc:HGNC:18404]
ENSG00000148082	120.074366	113.409544	74.8040708	40.4530273	1.01771331	0.0102501	0.0390366	SHC3	SHC adaptor protein 3 [Source:HGNC Symbol;Acc:HGNC:18181]
ENSG00000214176	269.675216	211.756883	142.233092	159.683003	0.672142486	0.0102562	0.039051	PLEKHM1P1	pleckstrin homology and RUN domain containing M1 pseudogene 1 [Source:HGNC Symbol;Acc:HGNC:35411]
ENSG00000070495	1487.15055	1420.27734	1146.29337	1173.13779	0.325834951	0.0102601	0.0390574	JMJD6	jumonji domain containing 6, arginine demethylase and lysine hydroxylase [Source:HGNC Symbol;Acc:HGNC:19355]
ENSG00000150051	86.6110184	95.6893027	170.679711	145.843809	-0.795277278	0.010271	0.0390901	MKX	mohawk homeobox [Source:HGNC Symbol;Acc:HGNC:23729]
ENSG00000182687	4.92108059	6.20208443	21.0715692	31.9366005	-2.249069938	0.0102744	0.0390941	GALR2	galanin receptor 2 [Source:HGNC Symbol;Acc:HGNC:4133]
ENSG00000177311	1513.72439	1670.13274	2137.7107	1862.96836	-0.329228605	0.0102933	0.0391574	ZBTB38	zinc finger and BTB domain containing 38 [Source:HGNC Symbol;Acc:HGNC:26636]
ENSG00000181830	740.130521	680.457263	889.220222	958.098015	-0.379309682	0.0103038	0.0391883	SLC35C1	solute carrier family 35 member C1 [Source:HGNC Symbol;Acc:HGNC:20197]
ENSG00000143228	345.459857	408.45156	618.450557	460.951601	-0.517083734	0.0103141	0.0392188	NUF2	NUF2, NDC80 kinetochore complex component [Source:HGNC Symbol;Acc:HGNC:14621]
ENSG00000181852	1425.14494	1346.73833	1704.68995	1738.41562	-0.313038881	0.0103223	0.039241	RNF41	ring finger protein 41 [Source:HGNC Symbol;Acc:HGNC:18401]
ENSG00000113763	8.85794506	7.97410856	0	0	5.432668064	0.0103236	1	UNC5A	unc-5 netrin receptor A [Source:HGNC Symbol;Acc:HGNC:12567]
ENSG00000073536	530.492488	487.306634	681.665265	674.926824	-0.415003412	0.0103345	0.0392785	NLE1	notchless homolog 1 [Source:HGNC Symbol;Acc:HGNC:19889]
ENSG00000110315	604.308697	677.799227	484.646093	467.338921	0.429967389	0.0103396	0.0392892	RNF141	ring finger protein 141 [Source:HGNC Symbol;Acc:HGNC:21159]
ENSG00000198680	353.333586	268.461655	447.770846	446.047854	-0.525066383	0.0103443	0.0392981	TUSC1	tumor suppressor candidate 1 [Source:HGNC Symbol;Acc:HGNC:31010]
ENSG00000173992	606.277129	409.337573	693.254628	740.929132	-0.499328291	0.010353	0.0393222	CCS	copper chaperone for superoxide dismutase [Source:HGNC Symbol;Acc:HGNC:1613]
ENSG00000109111	4636.64213	4143.87841	5577.64438	5099.21055	-0.282275122	0.0103627	0.0393504	SUPT6H	SPT6 homolog, histone chaperone [Source:HGNC Symbol;Acc:HGNC:11470]
ENSG00000055609	1879.85279	2015.67744	1656.22534	1458.43809	0.322870057	0.0103756	0.0393904	KMT2C	lysine methyltransferase 2C [Source:HGNC Symbol;Acc:HGNC:13726]
ENSG00000168958	2130.8279	2395.77662	1701.52922	1920.45424	0.321928063	0.0103785	0.0393927	MFF	mitochondrial fission factor [Source:HGNC Symbol;Acc:HGNC:24858]
ENSG00000143977	2220.39156	2361.22214	2828.85817	2753.99952	-0.284994943	0.0104012	0.0394701	SNRPG	small nuclear ribonucleoprotein polypeptide G [Source:HGNC Symbol;Acc:HGNC:11163]
ENSG00000049541	1186.96464	1117.26121	1378.08063	1531.89227	-0.336911386	0.0104653	0.0397042	RFC2	replication factor C subunit 2 [Source:HGNC Symbol;Acc:HGNC:9970]

ENSG00000121904	12.7948095	4.43006031	0	0	5.463089236	0.0104708	1	CSMD2	CUB and Sushi multiple domains 2 [Source:HGNC Symbol;Acc:HGNC:19290]
ENSG00000203685	12.7948095	4.43006031	0	0	5.463089236	0.0104708	1	STUM	stum, mechanosensory transduction mediator homolog [Source:HGNC Symbol;Acc:HGNC:30491]
ENSG00000187091	22.6369707	21.2642895	54.78608	58.5504343	-1.368916593	0.0105499	0.0400162	PLCD1	phospholipase C delta 1 [Source:HGNC Symbol;Acc:HGNC:9060]
ENSG00000166484	490.139627	460.726272	599.486145	683.443251	-0.432400411	0.0105532	0.0400197	MAPK7	mitogen-activated protein kinase 7 [Source:HGNC Symbol;Acc:HGNC:6880]
ENSG00000019186	298.217484	317.192318	190.697702	221.427097	0.578878377	0.01057	0.0400743	CYP24A1	cytochrome P450 family 24 subfamily A member 1 [Source:HGNC Symbol;Acc:HGNC:2602]
ENSG00000101040	1079.68508	1080.0487	1316.97308	1399.88766	-0.331063648	0.0105787	0.0400985	ZMYND8	zinc finger MYND-type containing 8 [Source:HGNC Symbol;Acc:HGNC:9397]
ENSG00000105298	526.555623	426.171802	679.558108	621.699157	-0.450845221	0.0105854	0.0401149	CACTIN	cactin, spliceosome C complex subunit [Source:HGNC Symbol;Acc:HGNC:29938]
ENSG00000116141	419.276066	404.0215	296.055548	286.364851	0.4991485	0.0106226	0.0402466	MARK1	microtubule affinity regulating kinase 1 [Source:HGNC Symbol;Acc:HGNC:6896]
ENSG00000163812	2429.04538	2407.29477	1938.58437	2021.58681	0.288344586	0.0106249	0.0402466	ZDHHC3	zinc finger DHHC-type containing 3 [Source:HGNC Symbol;Acc:HGNC:18470]
ENSG00000223745	60.0371832	98.3473389	28.4466185	39.388474	1.225779394	0.010629	0.0402531	CCDC18-AS1	CCDC18 antisense RNA 1 [Source:HGNC Symbol;Acc:HGNC:52262]
ENSG00000075891	39.3686447	31.0104222	11.5893631	7.45187345	1.884330438	0.0106368	0.0402733	PAX2	paired box 2 [Source:HGNC Symbol;Acc:HGNC:8616]
ENSG00000100767	189.953711	156.824135	109.57216	93.6806948	0.769653369	0.0106406	0.040279	PAPLN	papilin, proteoglycan like sulfated glycoprotein [Source:HGNC Symbol;Acc:HGNC:19262]
ENSG00000157851	1198.77523	995.877557	850.237819	836.738933	0.378970382	0.0106714	0.0403862	DPYSL5	dihydropyrimidinase like 5 [Source:HGNC Symbol;Acc:HGNC:20637]
ENSG00000259494	164.364092	193.150629	292.894813	255.492804	-0.616154057	0.0106843	0.0404262	MRPL46	mitochondrial ribosomal protein L46 [Source:HGNC Symbol;Acc:HGNC:1192]
ENSG00000166526	404.512825	381.871199	520.46776	553.567742	-0.450047756	0.0107006	0.0404788	ZNF3	zinc finger protein 3 [Source:HGNC Symbol;Acc:HGNC:13089]
ENSG00000175155	181.095766	180.746461	126.429415	83.0351613	0.788321331	0.0107061	0.0404905	YPEL2	yippee like 2 [Source:HGNC Symbol;Acc:HGNC:18326]
ENSG00000158864	1867.05798	1687.85298	2148.24648	2243.01391	-0.305102038	0.0107174	0.040524	NDUFS2	NADH:ubiquinone oxidoreductase core subunit S2 [Source:HGNC Symbol;Acc:HGNC:7708]
ENSG00000106479	162.39566	154.166099	91.6613262	92.6161415	0.780340202	0.0107387	0.0405955	ZNF862	zinc finger protein 862 [Source:HGNC Symbol;Acc:HGNC:34519]
ENSG00000233927	3101.26499	2117.56883	3301.9149	3548.15632	-0.39283164	0.0108378	0.0409609	RPS28	ribosomal protein S28 [Source:HGNC Symbol;Acc:HGNC:10418]
ENSG00000144909	1000.94779	1092.45287	809.148259	822.89974	0.359489038	0.0108471	0.040987	OSBPL11	oxysterol binding protein like 11 [Source:HGNC Symbol;Acc:HGNC:16397]
ENSG00000250337	214.559114	179.860449	131.697308	106.455335	0.726820674	0.010908	0.041208	PURPL	p53 upregulated regulator of p53 levels [Source:HGNC Symbol;Acc:HGNC:48995]
ENSG00000249026	6.88951283	9.74613268	0	0	5.417284904	0.0109114	1	CTNNA1P1	catenin alpha 1 pseudogene 1 [Source:HGNC Symbol;Acc:HGNC:2513]
ENSG00000285631	6.88951283	9.74613268	0	0	5.417284904	0.0109114	1	AC097105.1	
ENSG00000099290	837.567917	857.659676	1065.16783	1094.36084	-0.34911771	0.0109196	0.0412427	WASHC2A	WASH complex subunit 2A [Source:HGNC Symbol;Acc:HGNC:23416]
ENSG00000084628	62.0056154	53.1607237	20.0179908	24.4847271	1.371219066	0.0109302	0.0412733	NKAIN1	sodium/potassium transporting ATPase interacting 1 [Source:HGNC Symbol;Acc:HGNC:25743]
ENSG00000100028	2647.54136	2717.39899	3119.64583	3396.98974	-0.280488731	0.0109454	0.0413216	SNRPD3	small nuclear ribonucleoprotein D3 polypeptide [Source:HGNC Symbol;Acc:HGNC:11160]
ENSG00000179988	23.6211868	38.9845307	79.0183847	68.1314144	-1.228378464	0.0109508	0.0413326	PSTK	phosphoseryl-tRNA kinase [Source:HGNC Symbol;Acc:HGNC:28578]
ENSG00000136478	1213.53847	1046.38024	909.238213	839.932593	0.369097174	0.0109561	0.0413399	TEX2	testis expressed 2 [Source:HGNC Symbol;Acc:HGNC:30884]
ENSG00000108671	3410.30885	3426.20864	3945.65134	4306.1183	-0.271417854	0.0109576	0.0413399	PSMD11	proteasome 26S subunit, non-ATPase 11 [Source:HGNC Symbol;Acc:HGNC:9556]
ENSG00000178764	183.064198	197.58069	282.359028	288.493958	-0.584114777	0.0109745	0.0413946	ZHX2	zinc fingers and homeoboxes 2 [Source:HGNC Symbol;Acc:HGNC:18513]
ENSG00000108599	541.318865	567.933732	764.897964	701.540658	-0.402516054	0.0109979	0.0414735	AKAP10	A-kinase anchoring protein 10 [Source:HGNC Symbol;Acc:HGNC:368]
ENSG00000225313	77.7530733	73.5390011	33.7145108	34.0657072	1.158121463	0.0110174	0.0415376	AL513327.1	novel transcript
ENSG00000012211	699.77766	689.317384	527.84281	522.695695	0.402944799	0.0110447	0.0416313	PRICKLE3	prickle planar cell polarity protein 3 [Source:HGNC Symbol;Acc:HGNC:6645]
ENSG00000188338	38.3844286	45.1866152	14.7500985	11.7100869	1.659667088	0.0110549	0.0416606	SLC38A3	solute carrier family 38 member 3 [Source:HGNC Symbol;Acc:HGNC:18044]
ENSG00000115694	1310.97587	1040.17816	1454.99186	1580.86173	-0.369392224	0.0110756	0.0417292	STK25	serine/threonine kinase 25 [Source:HGNC Symbol;Acc:HGNC:11404]
ENSG00000081154	947.800122	973.727256	1329.61602	1133.74932	-0.358327523	0.0110948	0.0417924	PCNP	PEST proteolytic signal containing nuclear protein [Source:HGNC Symbol;Acc:HGNC:30023]
ENSG00000143036	40.3528608	48.7306634	6.32147077	20.2265137	1.748521596	0.0111475	0.0419813	SLC44A3	solute carrier family 44 member 3 [Source:HGNC Symbol;Acc:HGNC:28689]
ENSG00000007392	806.073001	716.783758	922.934733	1064.55335	-0.384605079	0.0111658	0.0420411	LUC7L	LUC7 like [Source:HGNC Symbol;Acc:HGNC:6723]
ENSG00000104863	124.011231	120.49764	53.7325016	75.5832879	0.91915287	0.0111716	0.0420534	LIN7B	lin-7 homolog B, crumbs cell polarity complex component [Source:HGNC Symbol;Acc:HGNC:17788]
ENSG00000198695	8402.253	8099.03626	8874.29139	11827.1877	-0.327161274	0.0111856	0.0420969	MT-ND6	mitochondrially encoded NADH:ubiquinone oxidoreductase core subunit 6 [Source:HGNC Symbol;Acc:HGNC:7462]
ENSG00000213995	957.642283	912.592424	1153.66842	1216.78448	-0.342130436	0.0112187	0.0422118	NAXD	NAD(P)HX dehydratase [Source:HGNC Symbol;Acc:HGNC:25576]
ENSG00000113838	423.212931	430.601862	287.62692	319.366005	0.492398049	0.0112528	0.0423306	TBCCD1	TBCC domain containing 1 [Source:HGNC Symbol;Acc:HGNC:25546]
ENSG00000143493	806.073001	916.136472	1118.90033	1091.16718	-0.359298645	0.0112603	0.0423494	INTS7	integrator complex subunit 7 [Source:HGNC Symbol;Acc:HGNC:24484]
ENSG00000228672	473.407953	349.088752	593.164674	559.955062	-0.488923464	0.0112666	0.0423614	PROB1	proline rich basic protein 1 [Source:HGNC Symbol;Acc:HGNC:41906]
ENSG00000119718	748.00425	699.063517	945.059881	920.838648	-0.367075198	0.0112701	0.0423614	EIF2B2	eukaryotic translation initiation factor 2B subunit beta [Source:HGNC Symbol;Acc:HGNC:3258]
ENSG00000154874	139.758689	132.015797	90.6077478	53.2276675	0.917311267	0.011271	0.0423614	CCDC144B	coiled-coil domain containing 144B (pseudogene) [Source:HGNC Symbol;Acc:HGNC:26704]
ENSG00000145214	465.534224	428.829838	267.608929	354.496266	0.523543833	0.0112756	0.0423693	DGKQ	diacylglycerol kinase theta [Source:HGNC Symbol;Acc:HGNC:2856]
ENSG00000099617	30.5106997	28.352386	60.0539724	77.7123946	-1.227378297	0.0112789	0.0423725	EFNA2	ephrin A2 [Source:HGNC Symbol;Acc:HGNC:3222]
ENSG00000055044	1544.23509	1836.703	2200.92541	2043.94243	-0.327864489	0.011289	0.0424009	NOP58	NOP58 ribonucleoprotein [Source:HGNC Symbol;Acc:HGNC:29926]
ENSG00000186603	850.362726	668.939107	979.82797	1025.16488	-0.401092236	0.0113114	0.0424756	HPDL	4-hydroxyphenylpyruvate dioxygenase like [Source:HGNC Symbol;Acc:HGNC:28242]

ENSG00000165304	1005.86887	1181.94009	1393.88431	1389.24212	-0.346645168	0.0113225	0.0425077	MELK	maternal embryonic leucine zipper kinase [Source:HGNC Symbol;Acc:HGNC:16870]
ENSG00000170619	768.672788	634.384636	920.827576	920.838648	-0.393265807	0.0113343	0.0425422	COMMD5	COMM domain containing 5 [Source:HGNC Symbol;Acc:HGNC:17902]
ENSG00000136147	28.5422674	36.3264945	77.9648062	69.1959678	-1.179518034	0.0113367	0.0425422	PHF11	PHD finger protein 11 [Source:HGNC Symbol;Acc:HGNC:17024]
ENSG00000113300	809.025649	897.530219	1313.81234	968.743549	-0.419318376	0.0113747	0.0426753	CNOT6	CCR4-NOT transcription complex subunit 6 [Source:HGNC Symbol;Acc:HGNC:14099]
ENSG00000129991	5.90529671	10.6321447	0	0	5.40950936	0.0113882	1	TNNI3	troponin I3, cardiac type [Source:HGNC Symbol;Acc:HGNC:11947]
ENSG00000185339	15.7474579	14.176193	0	2.1291067	3.817344234	0.0113928	0.0427315	TCN2	transcobalamin 2 [Source:HGNC Symbol;Acc:HGNC:11653]
ENSG00000142619	43.3055092	68.2229288	23.1787262	18.097407	1.435816978	0.0113947	0.0427315	PADI3	peptidyl arginine deiminase 3 [Source:HGNC Symbol;Acc:HGNC:18337]
ENSG00000262468	96.4531796	85.0571579	42.1431385	45.7757941	1.045236511	0.0114333	0.0428666	LINC01569	long intergenic non-protein coding RNA 1569 [Source:HGNC Symbol;Acc:HGNC:51380]
ENSG00000156374	214.559114	198.466702	355.055942	269.331998	-0.596832687	0.0114674	0.0429849	PCGF6	polycomb group ring finger 6 [Source:HGNC Symbol;Acc:HGNC:21156]
ENSG00000215158	119.09015	106.321447	49.5181877	67.0668611	0.950893848	0.0114865	0.0430468	AC138409.2	Putative beta-glucuronidase-like protein FLJ75287 pseudogene
ENSG00000278540	4489.99393	4260.83201	5560.78712	4995.94887	-0.270764127	0.0115176	0.0431541	ACACA	acetyl-CoA carboxylase alpha [Source:HGNC Symbol;Acc:HGNC:84]
ENSG00000121864	398.607528	460.726272	580.521733	586.568896	-0.440817134	0.0115235	0.0431665	ZNF639	zinc finger protein 639 [Source:HGNC Symbol;Acc:HGNC:30950]
ENSG00000108604	1631.83032	1527.48479	1909.08417	1986.45655	-0.302414396	0.0115456	0.0432334	SMARCD2	SWI/SNF related, matrix associated, actin dependent regulator of chromatin, subfamily d, member 2 [Source:HGNC Symbol;Acc:HGNC:11107]
ENSG00000165915	894.652451	783.234663	627.932764	651.50665	0.390653258	0.0115465	0.0432334	SLC39A13	solute carrier family 39 member 13 [Source:HGNC Symbol;Acc:HGNC:20859]
ENSG00000182195	736.193656	641.472733	482.538936	542.922209	0.425486185	0.0115685	0.0432979	LDOC1	LDOC1, regulator of NFKB signaling [Source:HGNC Symbol;Acc:HGNC:6548]
ENSG00000127423	275.580513	262.25957	351.895206	421.563127	-0.524365288	0.0115688	0.0432979	AUNIP	aurora kinase A and ninein interacting protein [Source:HGNC Symbol;Acc:HGNC:28363]
ENSG00000183474	226.369707	205.554798	342.413	295.945831	-0.564310864	0.0115751	0.0433118	GTF2H2C	GTF2H2 family member C [Source:HGNC Symbol;Acc:HGNC:31394]
ENSG00000138398	558.050539	661.85101	839.702034	777.123946	-0.405641626	0.0115811	0.0433237	PPIG	peptidylprolyl isomerase G [Source:HGNC Symbol;Acc:HGNC:14650]
ENSG00000089248	3438.85112	3131.16663	3842.40065	4123.01513	-0.278013514	0.0115834	0.0433237	ERP29	endoplasmic reticulum protein 29 [Source:HGNC Symbol;Acc:HGNC:13799]
ENSG00000260260	346.444074	332.254523	463.574523	473.726241	-0.465992482	0.0115879	0.043331	SNHG19	small nucleolar RNA host gene 19 [Source:HGNC Symbol;Acc:HGNC:49574]
ENSG00000138092	498.013356	503.254851	621.611293	718.573511	-0.420483825	0.0115994	0.0433642	CENPO	centromere protein O [Source:HGNC Symbol;Acc:HGNC:28152]
ENSG00000187068	13.7790257	13.2901809	1.05357846	0	4.676445775	0.0116109	0.0433976	C3orf70	chromosome 3 open reading frame 70 [Source:HGNC Symbol;Acc:HGNC:33731]
ENSG00000102054	7985.92958	7813.74037	9390.54483	9264.80781	-0.239719113	0.0116221	0.0434298	RBBP7	RB binding protein 7, chromatin remodeling factor [Source:HGNC Symbol;Acc:HGNC:9890]
ENSG00000106344	948.784338	959.551063	1110.4717	1348.78909	-0.365806752	0.0116537	0.0435383	RBM28	RNA binding motif protein 28 [Source:HGNC Symbol;Acc:HGNC:21863]
ENSG00000123977	53.1476704	86.8291821	27.39304	30.8720472	1.266820009	0.0116782	0.0436201	DAW1	dynein assembly factor with WD repeats 1 [Source:HGNC Symbol;Acc:HGNC:26383]
ENSG00000100722	948.784338	1027.77399	1212.66881	1289.17411	-0.339652151	0.0117248	0.0437848	ZC3H14	zinc finger CCCH-type containing 14 [Source:HGNC Symbol;Acc:HGNC:20509]
ENSG00000187123	402.544392	432.373886	302.377019	291.687618	0.49133363	0.0117671	0.0439329	LYPD6	LY6/PLAUR domain containing 6 [Source:HGNC Symbol;Acc:HGNC:28751]
ENSG00000170445	1717.45713	1532.80087	1928.04859	2116.33206	-0.315667559	0.011775	0.0439526	HARS	histidyl-tRNA synthetase [Source:HGNC Symbol;Acc:HGNC:4816]
ENSG00000151474	48.2265898	28.352386	10.5357846	11.7100869	1.781197842	0.0118073	0.0440635	FRMD4A	FERM domain containing 4A [Source:HGNC Symbol;Acc:HGNC:25491]
ENSG00000163319	150.585066	215.300931	269.716086	300.204045	-0.637144391	0.0118102	0.0440646	MRPS18C	mitochondrial ribosomal protein S18C [Source:HGNC Symbol;Acc:HGNC:16633]
ENSG00000170275	4853.16968	4991.79196	5703.02022	6033.88839	-0.253545683	0.0118157	0.0440737	CRTAP	cartilage associated protein [Source:HGNC Symbol;Acc:HGNC:2379]
ENSG00000071794	1567.85628	2250.47064	1569.83191	1276.39947	0.424336186	0.0118179	0.0440737	HLTF	helicase like transcription factor [Source:HGNC Symbol;Acc:HGNC:11099]
ENSG00000135801	562.97162	547.555454	742.772816	715.379851	-0.393064643	0.0118216	0.0440778	TAF5L	TATA-box binding protein associated factor 5 like [Source:HGNC Symbol;Acc:HGNC:17304]
ENSG00000145332	349.396722	412.881621	289.734077	232.07263	0.547365406	0.0118322	0.0441076	KLHL8	kelch like family member 8 [Source:HGNC Symbol;Acc:HGNC:18644]
ENSG00000218175	235.227652	205.554798	322.395009	323.624218	-0.552425017	0.0118383	0.0441207	AC016739.1	ribosomal protein, large, P1 (RPLP1) pseudogene
ENSG00000230202	2185.944	2967.25439	1958.60236	2045.00699	0.364591809	0.0118502	0.0441555	AL450405.1	ribosomal protein L29 (RPL29) pseudogene
ENSG00000107036	788.357111	874.493905	673.236637	590.827109	0.395901987	0.0118885	0.0442882	RIC1	RIC1 homolog, RAB6A GEF complex partner 1 [Source:HGNC Symbol;Acc:HGNC:17686]
ENSG00000189339	905.478829	905.504327	1075.70361	1241.26921	-0.355416745	0.0118929	0.044295	SLC35E2B	solute carrier family 35 member E2B [Source:HGNC Symbol;Acc:HGNC:33941]
ENSG00000177963	2288.30247	1908.46998	2547.55272	2673.09346	-0.315342207	0.0118967	0.0442995	RIC8A	RIC8 guanine nucleotide exchange factor A [Source:HGNC Symbol;Acc:HGNC:29550]
ENSG00000005102	211.606465	206.44081	296.055548	319.366005	-0.558040184	0.0119085	0.0443335	MEOX1	mesenchyme homeobox 1 [Source:HGNC Symbol;Acc:HGNC:7013]
ENSG00000092330	629.898316	595.400106	788.07669	807.995993	-0.381673357	0.01195	0.044478	TINF2	TERF1 interacting nuclear factor 2 [Source:HGNC Symbol;Acc:HGNC:11824]
ENSG00000103024	959.610715	753.110253	1049.36415	1210.39716	-0.400651384	0.0119727	0.0445473	NME3	NME/NM23 nucleoside diphosphate kinase 3 [Source:HGNC Symbol;Acc:HGNC:7851]
ENSG00000162889	2502.86159	2339.95786	1931.20932	2030.10324	0.289728121	0.0119738	0.0445473	MAPKAPK2	mitogen-activated protein kinase-activated protein kinase 2 [Source:HGNC Symbol;Acc:HGNC:6887]
ENSG00000165714	331.680832	350.860776	219.14432	250.170037	0.540731287	0.0120107	0.0446747	BORCS5	BLOC-1 related complex subunit 5 [Source:HGNC Symbol;Acc:HGNC:17950]
ENSG00000140995	1534.39293	1380.40679	1713.11858	1938.55165	-0.325443597	0.012036	0.0447587	DEF8	differentially expressed in FDCP 8 homolog [Source:HGNC Symbol;Acc:HGNC:25969]
ENSG00000280063	58.068751	52.2747116	18.9644123	23.4201737	1.380106971	0.012044	0.0447789	AC012676.5	tec
ENSG00000260563	174.206253	140.875918	71.6433354	105.390782	0.831047252	0.0120496	0.0447897	AC132872.1	novel transcript
ENSG00000162688	763.751708	960.437075	706.951148	563.148722	0.441525401	0.012059	0.0448149	AGL	amylo-alpha-1, 6-glucosidase, 4-alpha-glucanotransferase [Source:HGNC Symbol;Acc:HGNC:321]
ENSG00000172888	546.239946	505.026875	697.468942	690.895124	-0.401686674	0.0120809	0.0448863	ZNF621	zinc finger protein 621 [Source:HGNC Symbol;Acc:HGNC:24787]
ENSG00000099968	1077.71665	1029.54602	1268.50847	1384.98391	-0.332672154	0.0121173	0.0450115	BCL2L13	BCL2 like 13 [Source:HGNC Symbol;Acc:HGNC:17164]
ENSG00000205795	90.5478829	52.2747116	23.1787262	35.1302606	1.290528088	0.0121218	0.045014	CYS1	cystin 1 [Source:HGNC Symbol;Acc:HGNC:18525]
ENSG00000100311	470.455304	448.322103	361.377413	290.623065	0.494499799	0.0121233	0.045014	PDGFB	platelet derived growth factor subunit B [Source:HGNC Symbol;Acc:HGNC:8800]
ENSG00000185668	17.7158901	19.4922654	0	4.2582134	3.13147126	0.0121691	0.0451744	POU3F1	POU class 3 homeobox 1 [Source:HGNC Symbol;Acc:HGNC:9214]

ENSG00000185880	489.155411	508.570923	354.002363	374.722779	0.453488926	0.0121868	0.0452209	TRIM69	tripartite motif containing 69 [Source:HGNC Symbol;Acc:HGNC:17857]
ENSG00000040487	281.48581	249.855401	395.091923	364.077246	-0.515593298	0.012187	0.0452209	PQLC2	PQ loop repeat containing 2 [Source:HGNC Symbol;Acc:HGNC:26001]
ENSG00000276345	18.7001062	15.0622051	197.019172	36.1948139	-2.788981965	0.0121933	0.0452344	AC004556.1	39S ribosomal protein L23, mitochondrial-like [Source:NCBI gene;Acc:107987373]
ENSG00000261040	668.282744	706.151613	850.237819	930.419628	-0.373270012	0.0122144	0.0453027	WFDC21P	WAP four-disulfide core domain 21, pseudogene [Source:HGNC Symbol;Acc:HGNC:50357]
ENSG00000125744	339.554561	301.244101	211.769271	224.620757	0.55370856	0.012222	0.045321	RTN2	reticulon 2 [Source:HGNC Symbol;Acc:HGNC:10468]
ENSG00000164902	748.988466	841.711459	989.310176	1058.16603	-0.363650098	0.0122309	0.045344	PHAX	phosphorylated adaptor for RNA export [Source:HGNC Symbol;Acc:HGNC:10241]
ENSG00000237732	10.8263773	15.9482171	0	1.06455335	4.660426967	0.0122558	0.0454264	AC010980.1	uncharacterized LOC440934 [Source:NCBI gene;Acc:440934]
ENSG00000023839	185.03263	217.072955	118.000788	130.940062	0.692661993	0.0122693	0.0454665	ABCC2	ATP binding cassette subfamily C member 2 [Source:HGNC Symbol;Acc:HGNC:53]
ENSG00000187147	1449.75034	1097.76894	1624.61799	1669.21965	-0.37139473	0.0122768	0.0454841	RNF220	ring finger protein 220 [Source:HGNC Symbol;Acc:HGNC:25552]
ENSG00000174327	281.48581	226.819088	153.822455	173.522196	0.633953455	0.0122878	0.045515	SLC16A13	solute carrier family 16 member 13 [Source:HGNC Symbol;Acc:HGNC:31037]
ENSG00000126107	1375.93413	1266.99725	1035.66763	1065.6179	0.3306107	0.0123033	0.0455625	HECTD3	HECT domain E3 ubiquitin protein ligase 3 [Source:HGNC Symbol;Acc:HGNC:26117]
ENSG00000139613	3131.77569	2873.33712	3564.25594	3688.67736	-0.272544697	0.0123119	0.0455841	SMARCC2	SWI/SNF related, matrix associated, actin dependent regulator of chromatin subfamily c member 2 [Source:HGNC Symbol;Acc:HGNC:11105]
ENSG00000151461	607.261345	704.379589	880.791594	835.67438	-0.387401425	0.0123355	0.0456529	UPF2	UPF2, regulator of nonsense mediated mRNA decay [Source:HGNC Symbol;Acc:HGNC:17854]
ENSG00000258711	129.916528	113.409544	187.536966	205.458797	-0.692613248	0.0123359	0.0456529	AL358334.2	novel transcript
ENSG00000077721	1014.72682	1194.34426	1489.75995	1331.75624	-0.352520223	0.0123438	0.0456724	UBE2A	ubiquitin conjugating enzyme E2 A [Source:HGNC Symbol;Acc:HGNC:12472]
ENSG00000130204	2978.23797	2394.00459	3278.73617	3410.82893	-0.316753779	0.0123519	0.0456921	TOMM40	translocase of outer mitochondrial membrane 40 [Source:HGNC Symbol;Acc:HGNC:18001]
ENSG00000116863	641.708909	490.850682	740.665659	788.834033	-0.434589594	0.0123884	0.0458171	ADPRHL2	ADP-ribosylhydrolase like 2 [Source:HGNC Symbol;Acc:HGNC:21304]
ENSG00000133119	627.929883	690.203396	870.25581	840.997147	-0.376108488	0.0124026	0.0458598	RFC3	replication factor C subunit 3 [Source:HGNC Symbol;Acc:HGNC:9971]
ENSG00000166889	1756.82577	1726.83751	2110.31766	2141.88134	-0.287654511	0.0124233	0.0459173	PATL1	PAT1 homolog 1, processing body mRNA decay factor [Source:HGNC Symbol;Acc:HGNC:26721]
ENSG00000066654	420.260282	441.234007	647.950754	529.083015	-0.450076984	0.0124236	0.0459173	THUMPD1	THUMP domain containing 1 [Source:HGNC Symbol;Acc:HGNC:23807]
ENSG00000127580	447.818334	405.793524	576.307419	573.794256	-0.430677879	0.0124273	0.045921	WDR24	WD repeat domain 24 [Source:HGNC Symbol;Acc:HGNC:20852]
ENSG00000119328	287.391107	308.332198	422.484963	411.982147	-0.485773292	0.0124306	0.0459231	FAM206A	family with sequence similarity 206 member A [Source:HGNC Symbol;Acc:HGNC:1364]
ENSG00000136807	2386.72409	2044.02983	2629.73184	2849.80932	-0.306827675	0.0124515	0.0459902	CDK9	cyclin dependent kinase 9 [Source:HGNC Symbol;Acc:HGNC:1780]
ENSG00000168517	21.6527546	29.238398	53.7325016	71.3250745	-1.293974705	0.0124684	0.0460424	HEXIM2	hexamethylene bisacetamide inducible 2 [Source:HGNC Symbol;Acc:HGNC:28591]
ENSG00000158008	19.6843224	18.6062533	4.21431385	1.06455335	2.856232572	0.012495	0.0461234	EXTL1	exostosin like glycosyltransferase 1 [Source:HGNC Symbol;Acc:HGNC:3515]
ENSG00000214021	188.969495	156.824135	103.250689	103.261675	0.742731115	0.0124957	0.0461234	TTLL3	tubulin tyrosine ligase like 3 [Source:HGNC Symbol;Acc:HGNC:24483]
ENSG00000179431	342.507209	306.560173	450.931582	450.306067	-0.47422793	0.0125082	0.0461506	FJX1	four jointed box 1 [Source:HGNC Symbol;Acc:HGNC:17166]
ENSG00000108771	9.84216118	4.43006031	24.2323046	35.1302606	-2.066257049	0.0125089	0.0461506	DHX58	DExH-box helicase 58 [Source:HGNC Symbol;Acc:HGNC:29517]
ENSG00000047365	281.48581	377.441138	230.733683	208.652457	0.585791986	0.0125113	0.0461506	ARAP2	ArfGAP with RhoGAP domain, ankyrin repeat and PH domain 2 [Source:HGNC Symbol;Acc:HGNC:16924]
ENSG00000126453	720.446199	574.135816	834.434142	881.450174	-0.407406071	0.0125297	0.0462085	BCL2L12	BCL2 like 12 [Source:HGNC Symbol;Acc:HGNC:13787]
ENSG00000119640	315.933374	297.700053	202.287065	215.039777	0.555945114	0.0125625	0.0463177	ACYP1	acylphosphatase 1 [Source:HGNC Symbol;Acc:HGNC:179]
ENSG00000131400	32.4791319	29.238398	7.37504924	8.5164268	1.957136639	0.0125648	0.0463177	NAPSA	napsin A aspartic peptidase [Source:HGNC Symbol;Acc:HGNC:13395]
ENSG00000163453	1158.42237	1107.51508	935.577675	845.25536	0.347354196	0.0125723	0.046327	IGFBP7	insulin like growth factor binding protein 7 [Source:HGNC Symbol;Acc:HGNC:5476]
ENSG00000152104	1769.62058	1961.6307	1641.47524	1252.97929	0.366488495	0.0125737	0.046327	PTPN14	protein tyrosine phosphatase, non-receptor type 14 [Source:HGNC Symbol;Acc:HGNC:9647]
ENSG00000176915	3330.58734	3171.92318	3838.18634	3961.20302	-0.262458274	0.0125756	0.046327	ANKLE2	ankyrin repeat and LEM domain containing 2 [Source:HGNC Symbol;Acc:HGNC:29101]
ENSG00000100138	3012.68554	2678.41446	3297.70059	3668.45084	-0.291857972	0.0125999	0.0464066	SNU13	small nuclear ribonucleoprotein 13 [Source:HGNC Symbol;Acc:HGNC:7819]
ENSG00000066629	1047.20595	972.841244	777.540905	802.673226	0.354012283	0.0126257	0.0464915	EML1	echinoderm microtubule associated protein like 1 [Source:HGNC Symbol;Acc:HGNC:3330]
ENSG00000134955	76.7688572	54.9327478	28.4466185	26.6138338	1.256485594	0.0126346	0.0465139	SLC37A2	solute carrier family 37 member 2 [Source:HGNC Symbol;Acc:HGNC:20644]
ENSG00000197142	640.724693	668.939107	883.95233	814.383313	-0.374747832	0.0126697	0.0466332	ACSL5	acyl-CoA synthetase long chain family member 5 [Source:HGNC Symbol;Acc:HGNC:16526]
ENSG00000159247	9.84216118	6.20208443	0	0	5.362312975	0.0126974	1	TUBBP5	tubulin beta pseudogene 5 [Source:HGNC Symbol;Acc:HGNC:23674]
ENSG00000159388	507.855517	538.695334	340.305843	417.304913	0.466480275	0.0126991	0.0467311	BTG2	BTG anti-proliferation factor 2 [Source:HGNC Symbol;Acc:HGNC:1131]
ENSG00000068878	2068.82228	2279.70903	1871.15535	1622.37931	0.316006193	0.0127059	0.0467372	PSME4	proteasome activator subunit 4 [Source:HGNC Symbol;Acc:HGNC:20635]
ENSG00000159259	1076.73243	1057.8984	1257.97268	1438.21158	-0.336953993	0.0127063	0.0467372	CHAF1B	chromatin assembly factor 1 subunit B [Source:HGNC Symbol;Acc:HGNC:1911]
ENSG00000065183	1123.97481	1245.73296	1476.06343	1487.18103	-0.322085355	0.0127171	0.0467574	WDR3	WD repeat domain 3 [Source:HGNC Symbol;Acc:HGNC:12755]
ENSG00000176142	683.045986	694.633456	894.488115	877.191961	-0.362802363	0.0127173	0.0467574	TMEM39A	transmembrane protein 39A [Source:HGNC Symbol;Acc:HGNC:25600]
ENSG00000155329	93.5005312	132.015797	171.733289	207.587903	-0.74769765	0.0127602	0.0469049	ZCCHC10	zinc finger CCHC-type containing 10 [Source:HGNC Symbol;Acc:HGNC:25954]
ENSG00000279602	98.4216118	52.2747116	29.5001969	34.0657072	1.242814839	0.0128019	0.0470481	AC109326.1	TEC
ENSG00000204624	101.37426	91.2592424	56.893237	37.2593673	1.031883887	0.0128108	0.0470703	DISP3	dispatched RND transporter family member 3 [Source:HGNC Symbol;Acc:HGNC:29251]
ENSG00000063245	3320.74518	2754.6115	3705.43545	3765.3252	-0.298597101	0.0128632	0.0472477	EPN1	epsin 1 [Source:HGNC Symbol;Acc:HGNC:21604]
ENSG00000130714	255.896191	210.870871	307.644911	382.174653	-0.564576662	0.0128646	0.0472477	POMT1	protein O-mannosyltransferase 1 [Source:HGNC Symbol;Acc:HGNC:9202]
ENSG00000167771	48.2265898	57.590784	93.7684831	112.842655	-0.963678285	0.0128683	0.0472508	RCOR2	REST corepressor 2 [Source:HGNC Symbol;Acc:HGNC:27455]

ENSG00000109881	439.944605	554.643551	698.522521	649.377544	-0.437479025	0.0128741	0.0472619	CCDC34	coiled-coil domain containing 34 [Source:HGNC Symbol;Acc:HGNC:25079]
ENSG00000137462	30.5106997	33.6684583	10.5357846	6.3873201	1.92287833	0.0128849	0.0472912	TLR2	toll like receptor 2 [Source:HGNC Symbol;Acc:HGNC:11848]
ENSG00000126067	3621.91531	3567.08456	4197.45659	4391.28257	-0.256676511	0.0129279	0.0474389	PSMB2	proteasome subunit beta 2 [Source:HGNC Symbol;Acc:HGNC:9539]
ENSG00000140264	3892.57475	3210.90771	4183.76007	4579.70851	-0.30323142	0.0129499	0.047509	SERF2	small EDRK-rich factor 2 [Source:HGNC Symbol;Acc:HGNC:10757]
ENSG00000179240	134.837608	124.041689	75.8576493	68.1314144	0.845780857	0.0129643	0.0475517	GVQW3	GVQW motif containing 3 [Source:HGNC Symbol;Acc:HGNC:51239]
ENSG00000080839	335.617696	335.798571	503.610505	429.015	-0.474166077	0.0129705	0.0475642	RBL1	RB transcriptional corepressor like 1 [Source:HGNC Symbol;Acc:HGNC:9893]
ENSG00000225920	8.85794506	17.7202412	1.05357846	0	4.652657112	0.0129737	0.0475656	RIMKLBP2	ribosomal modification protein rimK like family member B pseudogene 2 [Source:HGNC Symbol;Acc:HGNC:39163]
ENSG00000187391	21.6527546	18.6062533	2.10715692	4.2582134	2.66154892	0.0129945	0.0476314	MAGI2	membrane associated guanylate kinase, WW and PDZ domain containing 2 [Source:HGNC Symbol;Acc:HGNC:18957]
ENSG00000100413	1327.70754	1066.75852	1478.17058	1581.92628	-0.354511671	0.0129995	0.0476396	POLR3H	RNA polymerase III subunit H [Source:HGNC Symbol;Acc:HGNC:30349]
ENSG00000089116	16.731674	12.4041689	0	2.1291067	3.778075431	0.0130222	0.0477061	LHX5	LIM homeobox 5 [Source:HGNC Symbol;Acc:HGNC:14216]
ENSG00000168938	763.751708	757.540313	524.682074	625.95737	0.402923833	0.0130233	0.0477061	PPIC	peptidylprolyl isomerase C [Source:HGNC Symbol;Acc:HGNC:9256]
ENSG00000175575	315.933374	326.052439	468.842416	422.62768	-0.473502612	0.0130289	0.047716	PAAF1	proteasomal ATPase associated factor 1 [Source:HGNC Symbol;Acc:HGNC:25687]
ENSG00000177108	7.87372895	7.97410856	0	0	5.346151346	0.0130339	1	ZDHHC22	zinc finger DHHC-type containing 22 [Source:HGNC Symbol;Acc:HGNC:20106]
ENSG00000100034	919.257854	871.835869	1178.9543	1091.16718	-0.34218663	0.0130366	0.0477254	PPM1F	protein phosphatase, Mg2+/Mn2+ dependent 1F [Source:HGNC Symbol;Acc:HGNC:19388]
ENSG00000138246	1439.90818	1599.25177	1961.7631	1809.7407	-0.311167246	0.0130371	0.0477254	DNAJC13	DnaJ heat shock protein family (Hsp40) member C13 [Source:HGNC Symbol;Acc:HGNC:30343]
ENSG00000081320	272.627865	279.979812	197.019172	171.393089	0.584960752	0.0130504	0.0477638	STK17B	serine/threonine kinase 17b [Source:HGNC Symbol;Acc:HGNC:11396]
ENSG00000259863	44.2897253	39.8705428	8.4286277	18.097407	1.666144587	0.0131134	0.0479841	SH3RF3-AS1	SH3RF3 antisense RNA 1 [Source:HGNC Symbol;Acc:HGNC:44168]
ENSG00000165359	101.37426	80.6270976	43.196717	45.7757941	1.031409991	0.0131166	0.0479853	INTS6L	integrator complex subunit 6 like [Source:HGNC Symbol;Acc:HGNC:27334]
ENSG00000231993	31.4949158	46.0726272	14.7500985	8.5164268	1.73820958	0.0131802	0.0482022	EP300-AS1	EP300 antisense RNA 1 [Source:HGNC Symbol;Acc:HGNC:50504]
ENSG00000121350	132.869176	171.000328	279.198293	206.52335	-0.675220127	0.0131816	0.0482022	PYROXD1	pyridine nucleotide-disulphide oxidoreductase domain 1 [Source:HGNC Symbol;Acc:HGNC:26162]
ENSG00000125356	1603.28806	1490.27229	1914.35207	1887.45309	-0.297661288	0.0132204	0.0483338	NDUFA1	NADH:ubiquinone oxidoreductase subunit A1 [Source:HGNC Symbol;Acc:HGNC:7683]
ENSG00000146414	466.51844	586.539985	417.217071	327.882432	0.499758869	0.013246	0.0484168	SHPRH	SNF2 histone linker PHD RING helicase [Source:HGNC Symbol;Acc:HGNC:19336]
ENSG00000083544	115.153286	118.725616	185.429809	191.619603	-0.688731702	0.0132631	0.0484687	TDRD3	tudor domain containing 3 [Source:HGNC Symbol;Acc:HGNC:20612]
ENSG00000104154	91.532099	93.0312665	54.78608	34.0657072	1.054278592	0.0133159	0.0486512	SLC30A4	solute carrier family 30 member 4 [Source:HGNC Symbol;Acc:HGNC:11015]
ENSG00000176406	20.6685385	12.4041689	0	3.19366005	3.374686056	0.0133393	0.0487261	RIMS2	regulating synaptic membrane exocytosis 2 [Source:HGNC Symbol;Acc:HGNC:17283]
ENSG00000251493	801.15192	829.30729	559.450163	678.120484	0.397987362	0.0133461	0.0487406	FOXD1	forkhead box D1 [Source:HGNC Symbol;Acc:HGNC:3802]
ENSG00000055130	1930.04781	1898.72385	2290.47958	2359.05022	-0.280244085	0.0133553	0.0487634	CUL1	cullin 1 [Source:HGNC Symbol;Acc:HGNC:2551]
ENSG00000153208	33.463348	31.8964342	9.48220616	8.5164268	1.860135519	0.0133725	0.0488097	MERTK	MER proto-oncogene, tyrosine kinase [Source:HGNC Symbol;Acc:HGNC:7027]
ENSG00000214517	1995.99029	1811.00865	2200.92541	2507.02314	-0.306659039	0.0133737	0.0488097	PPME1	protein phosphatase methylesterase 1 [Source:HGNC Symbol;Acc:HGNC:30178]
ENSG00000059758	724.383063	811.587049	619.504136	545.051315	0.399730457	0.0133778	0.0488141	CDK17	cyclin dependent kinase 17 [Source:HGNC Symbol;Acc:HGNC:8750]
ENSG00000196275	18.7001062	6.20208443	36.8752462	45.7757941	-1.741621581	0.0134394	0.0490281	GTF2IRD2	GTF2I repeat domain containing 2 [Source:HGNC Symbol;Acc:HGNC:30775]
ENSG00000235092	31.4949158	38.0985187	9.48220616	10.6455335	1.790824905	0.0134883	0.0491907	ID2-AS1	ID2 antisense RNA 1 [Source:HGNC Symbol;Acc:HGNC:51103]
ENSG00000160781	158.458795	115.181568	56.893237	89.4224814	0.902189243	0.0134898	0.0491907	PAQR6	progestin and adipoQ receptor family member 6 [Source:HGNC Symbol;Acc:HGNC:30132]
ENSG00000126814	342.507209	401.363464	480.431779	546.115869	-0.463740893	0.0135251	0.0493089	TRMT5	tRNA methyltransferase 5 [Source:HGNC Symbol;Acc:HGNC:23141]
ENSG00000113638	427.149795	458.954248	342.413	291.687618	0.482981927	0.0135345	0.0493327	TTC33	tetratricopeptide repeat domain 33 [Source:HGNC Symbol;Acc:HGNC:29959]
ENSG00000168487	2214.48627	2018.33548	2559.14208	2591.12285	-0.283268735	0.0135399	0.0493416	BMP1	bone morphogenetic protein 1 [Source:HGNC Symbol;Acc:HGNC:1067]
ENSG00000163875	639.740477	688.431372	775.433748	985.776402	-0.40674494	0.013556	0.0493897	MEAF6	MYST/Esa1 associated factor 6 [Source:HGNC Symbol;Acc:HGNC:25674]
ENSG00000167123	3447.70906	3008.01095	2637.10689	2660.31882	0.285053098	0.0135647	0.0494106	CERCAM	cerebral endothelial cell adhesion molecule [Source:HGNC Symbol;Acc:HGNC:23723]
ENSG00000076706	5602.15814	5228.35718	6347.81024	6518.26016	-0.248558925	0.0135683	0.0494133	MCAM	melanoma cell adhesion molecule [Source:HGNC Symbol;Acc:HGNC:6934]
ENSG00000132199	609.229777	551.099502	694.308207	850.578127	-0.4133391	0.0135743	0.0494243	ENOSF1	enolase superfamily member 1 [Source:HGNC Symbol;Acc:HGNC:30365]
ENSG00000179820	412.386554	401.363464	543.646487	550.374082	-0.427138035	0.013579	0.0494308	MYADM	myeloid associated differentiation marker [Source:HGNC Symbol;Acc:HGNC:7544]
ENSG00000140534	1567.85628	1506.22051	1853.24452	1915.13148	-0.293914788	0.0135914	0.0494654	TICRR	TOPBP1 interacting checkpoint and replication regulator [Source:HGNC Symbol;Acc:HGNC:28704]
ENSG00000172771	20.6685385	20.3782774	5.26789231	1.06455335	2.694063708	0.0135967	0.0494739	EFCAB12	EF-hand calcium binding domain 12 [Source:HGNC Symbol;Acc:HGNC:28061]
ENSG00000183662	0	0	8.4286277	6.3873201	-5.430621655	0.0136275	1	FAM19A1	family with sequence similarity 19 member A1, C-C motif chemokine like [Source:HGNC Symbol;Acc:HGNC:21587]
ENSG00000126088	1512.74017	1252.82106	1728.92226	1746.93205	-0.330349355	0.0136533	0.0496693	UROD	uroporphyrinogen decarboxylase [Source:HGNC Symbol;Acc:HGNC:12591]
ENSG00000111912	1729.26772	2029.85363	2417.96257	2245.14302	-0.310517893	0.0136582	0.0496762	NCOA7	nuclear receptor coactivator 7 [Source:HGNC Symbol;Acc:HGNC:21081]
ENSG00000198088	196.843224	174.544376	113.786474	114.971762	0.698481646	0.0136815	0.0497504	NUP62CL	nucleoporin 62 C-terminal like [Source:HGNC Symbol;Acc:HGNC:25960]
ENSG00000100258	2141.65427	1732.15358	2398.99816	2438.89173	-0.321095528	0.0136943	0.0497863	LMF2	lipase maturation factor 2 [Source:HGNC Symbol;Acc:HGNC:25096]
ENSG00000152503	209.638033	273.777727	162.251083	146.908362	0.646071497	0.0137123	0.049841	TRIM36	tripartite motif containing 36 [Source:HGNC Symbol;Acc:HGNC:16280]
ENSG00000110046	1485.18212	1266.11124	1109.41812	1063.4888	0.340011354	0.0137555	0.0499871	ATG2A	autophagy related 2A [Source:HGNC Symbol;Acc:HGNC:29028]

ENSG00000163472	132.869176	99.2333509	183.322652	196.94237	-0.714333305	0.0137697	0.0500279	TMEM79	transmembrane protein 79 [Source:HGNC Symbol;Acc:HGNC:28196]
ENSG00000101019	467.502656	557.301587	664.80801	702.605211	-0.415199934	0.0137871	0.0500806	UQCC1	ubiquinol-cytochrome c reductase complex assembly factor 1 [Source:HGNC Symbol;Acc:HGNC:15891]
ENSG00000186469	27.5580513	20.3782774	6.32147077	3.19366005	2.330464612	0.0137984	0.0501108	GNG2	G protein subunit gamma 2 [Source:HGNC Symbol;Acc:HGNC:4404]
ENSG00000103528	300.185916	293.269992	203.340643	201.200583	0.552725616	0.0138145	0.0501585	SYT17	synaptotagmin 17 [Source:HGNC Symbol;Acc:HGNC:24119]
ENSG00000157379	419.276066	361.492921	272.876822	277.848424	0.502879819	0.0138236	0.0501786	DHRS1	dehydrogenase/reductase 1 [Source:HGNC Symbol;Acc:HGNC:16445]
ENSG00000008311	12.7948095	21.2642895	2.10715692	2.1291067	3.009273472	0.0138289	0.0501786	AASS	aminoadipate-semialdehyde synthase [Source:HGNC Symbol;Acc:HGNC:17366]
ENSG00000136960	12.7948095	21.2642895	2.10715692	2.1291067	3.009273472	0.0138289	0.0501786	ENPP2	ectonucleotide pyrophosphatase/phosphodiesterase 2 [Source:HGNC Symbol;Acc:HGNC:3357]
ENSG00000133065	1687.93064	1538.11694	1368.59842	1206.13895	0.325049694	0.0138467	0.0502321	SLC41A1	solute carrier family 41 member 1 [Source:HGNC Symbol;Acc:HGNC:19429]
ENSG00000132825	285.422674	342.000656	209.662114	217.168883	0.556661428	0.0138788	0.0503278	PPP1R3D	protein phosphatase 1 regulatory subunit 3D [Source:HGNC Symbol;Acc:HGNC:9294]
ENSG00000172379	180.11155	171.88634	110.625739	104.326228	0.711252889	0.013879	0.0503278	ARNT2	aryl hydrocarbon receptor nuclear translocator 2 [Source:HGNC Symbol;Acc:HGNC:16876]
ENSG00000146457	1147.59599	1367.11661	1702.5828	1492.5038	-0.344978277	0.0139011	0.0503971	WTAP	WT1 associated protein [Source:HGNC Symbol;Acc:HGNC:16846]
ENSG00000123360	5.90529671	9.74613268	0	0	5.329798594	0.0139412	1	PDE1B	phosphodiesterase 1B [Source:HGNC Symbol;Acc:HGNC:8775]
ENSG00000115738	686.982851	786.778711	552.075114	568.471489	0.395852058	0.0139666	0.0506236	ID2	inhibitor of DNA binding 2 [Source:HGNC Symbol;Acc:HGNC:5361]
ENSG00000165886	535.413568	454.524188	361.377413	357.689926	0.460487949	0.0139786	0.0506564	UBTD1	ubiquitin domain containing 1 [Source:HGNC Symbol;Acc:HGNC:25683]
ENSG00000001629	1725.33086	1865.9414	1558.24255	1298.75509	0.330118198	0.0139867	0.0506749	ANKIB1	ankyrin repeat and IBR domain containing 1 [Source:HGNC Symbol;Acc:HGNC:22215]
ENSG00000164050	920.242071	722.09983	1109.41812	1034.74586	-0.385557923	0.0139929	0.0506864	PLXNB1	plexin B1 [Source:HGNC Symbol;Acc:HGNC:9103]
ENSG00000132361	2761.71043	2306.2894	3125.9673	3105.30212	-0.298464734	0.0139959	0.0506865	CLUH	clustered mitochondria homolog [Source:HGNC Symbol;Acc:HGNC:29094]
ENSG00000157349	348.412506	288.839932	419.324228	474.790794	-0.489611914	0.0140009	0.0506937	DDX19B	DEAD-box helicase 19B [Source:HGNC Symbol;Acc:HGNC:2742]
ENSG00000138443	607.261345	647.674817	461.467366	484.371774	0.408258803	0.0140053	0.0506986	ABI2	abl interactor 2 [Source:HGNC Symbol;Acc:HGNC:24011]
ENSG00000156463	301.170132	281.751836	211.769271	180.97407	0.56927031	0.0140165	0.0507117	SH3RF2	SH3 domain containing ring finger 2 [Source:HGNC Symbol;Acc:HGNC:26299]
ENSG00000159593	1077.71665	1322.81601	1577.20696	1475.47094	-0.346110368	0.014017	0.0507117	NAE1	NEDD8 activating enzyme E1 subunit 1 [Source:HGNC Symbol;Acc:HGNC:621]
ENSG00000100142	1705.64653	1354.71244	1839.548	2048.20065	-0.34576731	0.0140179	0.0507117	POLR2F	RNA polymerase II subunit F [Source:HGNC Symbol;Acc:HGNC:9193]
ENSG00000174485	614.150858	776.146566	588.95036	382.174653	0.518071418	0.0140362	0.050767	DENND4A	DENN domain containing 4A [Source:HGNC Symbol;Acc:HGNC:24321]
ENSG00000182389	9.84216118	15.9482171	1.05357846	0	4.608575724	0.0140483	0.0508001	CACNB4	calcium voltage-gated channel auxiliary subunit beta 4 [Source:HGNC Symbol;Acc:HGNC:1404]
ENSG00000151623	14.7632418	19.4922654	1.05357846	3.19366005	3.014857918	0.0140521	0.0508009	NR3C2	nuclear receptor subfamily 3 group C member 2 [Source:HGNC Symbol;Acc:HGNC:7979]
ENSG00000181409	19.6843224	8.86012062	41.08956	45.7757941	-1.613835915	0.0140546	0.0508009	AATK	apoptosis associated tyrosine kinase [Source:HGNC Symbol;Acc:HGNC:21]
ENSG00000078674	1304.08636	1411.41721	1796.35128	1589.37815	-0.318027883	0.0140854	0.0509013	PCM1	pericentriolar material 1 [Source:HGNC Symbol;Acc:HGNC:8727]
ENSG00000102172	6379.68888	6830.26698	7475.13919	8310.96801	-0.256950336	0.0140974	0.0509237	SMS	spermine synthase [Source:HGNC Symbol;Acc:HGNC:11123]
ENSG00000154065	89.5636668	135.559845	54.78608	61.7440943	0.952023733	0.0140976	0.0509237	ANKRD29	ankyrin repeat domain 29 [Source:HGNC Symbol;Acc:HGNC:27110]
ENSG00000079313	771.625437	633.498624	871.309388	969.808102	-0.39065535	0.0141018	0.050928	REXO1	RNA exonuclease 1 homolog [Source:HGNC Symbol;Acc:HGNC:24616]
ENSG00000155666	29.5264835	39.8705428	76.9112277	74.5187345	-1.122887941	0.0141105	0.0509484	KDM8	lysine demethylase 8 [Source:HGNC Symbol;Acc:HGNC:25840]
ENSG00000186834	3753.80027	3260.52439	4053.11634	4500.93156	-0.286505018	0.0141159	0.0509541	HEXIM1	hexamethylene bisacetamide inducible 1 [Source:HGNC Symbol;Acc:HGNC:24953]
ENSG00000154845	1611.16179	1600.13778	2039.7279	1893.84041	-0.292725455	0.0141181	0.0509541	PPP4R1	protein phosphatase 4 regulatory subunit 1 [Source:HGNC Symbol;Acc:HGNC:9320]
ENSG00000163132	324.791319	319.850354	233.894419	210.781563	0.53558834	0.0141322	0.0509942	MSX1	msh homeobox 1 [Source:HGNC Symbol;Acc:HGNC:7391]
ENSG00000138430	1074.764	1086.25079	1409.68798	1291.30321	-0.321769359	0.0141639	0.0510978	OLA1	Obg like ATPase 1 [Source:HGNC Symbol;Acc:HGNC:28833]
ENSG00000160932	6673.9695	5944.25492	7415.08522	7647.75127	-0.255605887	0.0141725	0.0511178	LY6E	lymphocyte antigen 6 family member E [Source:HGNC Symbol;Acc:HGNC:6727]
ENSG00000105732	441.913037	356.176849	538.378594	553.567742	-0.45341498	0.0141862	0.0511505	ZNF574	zinc finger protein 574 [Source:HGNC Symbol;Acc:HGNC:26166]
ENSG00000073169	817.883594	676.027203	947.167038	991.099169	-0.376446932	0.0141881	0.0511505	SELENOO	selenoprotein O [Source:HGNC Symbol;Acc:HGNC:30395]
ENSG00000172465	116.137502	103.663411	164.35824	195.877816	-0.713519695	0.0141906	0.0511505	TCEAL1	transcription elongation factor A like 1 [Source:HGNC Symbol;Acc:HGNC:11616]
ENSG00000131242	175.190469	157.710147	114.840052	79.8415013	0.77318949	0.0141959	0.0511586	RAB11FIP4	RAB11 family interacting protein 4 [Source:HGNC Symbol;Acc:HGNC:30267]
ENSG00000185049	636.787828	561.731647	742.772816	829.28706	-0.391955594	0.014201	0.0511661	NELFA	negative elongation factor complex member A [Source:HGNC Symbol;Acc:HGNC:12768]
ENSG00000125388	150.585066	152.394075	97.982797	78.7769479	0.777299725	0.0142091	0.0511844	GRK4	G protein-coupled receptor kinase 4 [Source:HGNC Symbol;Acc:HGNC:4543]
ENSG00000175203	3113.07558	2860.04694	3530.54143	3655.6762	-0.266916741	0.0142411	0.0512886	DCTN2	dynactin subunit 2 [Source:HGNC Symbol;Acc:HGNC:2712]
ENSG00000183814	238.180301	350.860776	442.502954	417.304913	-0.543756722	0.0142599	0.0513454	LIN9	lin-9 DREAM MuvB core complex component [Source:HGNC Symbol;Acc:HGNC:30830]
ENSG00000096080	221.448627	189.606581	278.144714	331.076092	-0.568555799	0.0142965	0.0514662	MRPS18A	mitochondrial ribosomal protein S18A [Source:HGNC Symbol;Acc:HGNC:14515]
ENSG00000166037	765.72014	920.566532	1190.54366	1007.06747	-0.381480767	0.0142996	0.0514663	CEP57	centrosomal protein 57 [Source:HGNC Symbol;Acc:HGNC:30794]
ENSG00000164611	1996.9745	2174.2736	2334.72987	2875.3586	-0.320615614	0.0143294	0.0515626	PTTG1	pituitary tumor-transforming 1 [Source:HGNC Symbol;Acc:HGNC:9690]
ENSG00000233058	30.5106997	34.5544704	10.5357846	7.45187345	1.854989625	0.0143454	0.0516091	LINC00884	long intergenic non-protein coding RNA 884 [Source:HGNC Symbol;Acc:HGNC:48570]
ENSG00000103044	118.105934	134.673833	81.1255416	56.4213276	0.878277943	0.014363	0.0516617	HAS3	hyaluronan synthase 3 [Source:HGNC Symbol;Acc:HGNC:4820]
ENSG00000068438	1639.70405	1547.86307	2022.87065	1887.45309	-0.29503549	0.0143692	0.0516729	FTSJ1	FtsJ RNA methyltransferase homolog 1 [Source:HGNC Symbol;Acc:HGNC:13254]
ENSG00000074855	1072.79557	906.390339	723.808404	801.608673	0.37520519	0.0143811	0.0517045	ANO8	anoctamin 8 [Source:HGNC Symbol;Acc:HGNC:29329]
ENSG00000278600	51.1792381	53.1607237	18.9644123	21.291067	1.374360244	0.0143991	0.0517584	AC015871.3	novel transcript, sense intronic to ST20
ENSG00000004700	1673.1674	2026.30959	1594.06421	1291.30321	0.358868644	0.0144037	0.051764	RECQL	RecQ like helicase [Source:HGNC Symbol;Acc:HGNC:9948]

ENSG00000180901	750.956898	713.23971	960.863558	910.193114	-0.35401313	0.0144112	0.0517798	KCTD2	potassium channel tetramerization domain containing 2 [Source:HGNC Symbol;Acc:HGNC:21294]
ENSG00000125633	1217.47534	1230.67075	1011.43532	940.000608	0.32715652	0.0144358	0.0518572	CCDC93	coiled-coil domain containing 93 [Source:HGNC Symbol;Acc:HGNC:25611]
ENSG00000276180	199.795872	226.819088	333.984373	291.687618	-0.551701219	0.0144661	0.0519452	HIST1H4I	histone cluster 1 H4 family member i [Source:HGNC Symbol;Acc:HGNC:4793]
ENSG00000136114	68.8951283	87.7151941	36.8752462	36.1948139	1.101028828	0.0144692	0.0519452	THSD1	thrombospondin type 1 domain containing 1 [Source:HGNC Symbol;Acc:HGNC:17754]
ENSG00000080031	97.4373957	71.766977	30.5537754	47.9049008	1.107776347	0.0144695	0.0519452	PTPRH	protein tyrosine phosphatase, receptor type H [Source:HGNC Symbol;Acc:HGNC:9672]
ENSG00000169914	353.333586	404.907512	274.983979	259.751017	0.504436814	0.014513	0.0520901	OTUD3	OTU deubiquitinase 3 [Source:HGNC Symbol;Acc:HGNC:29038]
ENSG00000163491	73.8162089	83.2851338	42.1431385	30.8720472	1.105718172	0.0145257	0.0521249	NEK10	NIMA related kinase 10 [Source:HGNC Symbol;Acc:HGNC:18592]
ENSG00000227671	227.353923	311.876246	204.394222	129.875509	0.690735218	0.014595	0.0523622	AL390728.4	zinc finger protein pseudogene
ENSG00000226887	24.605403	19.4922654	1.05357846	6.3873201	2.568581784	0.0146044	0.052385	ERVMER34-1	endogenous retrovirus group MER34 member 1, envelope [Source:HGNC Symbol;Acc:HGNC:42970]
ENSG00000141570	409.433905	319.850354	496.235456	514.179268	-0.471694029	0.0146268	0.0524401	CBX8	chromobox 8 [Source:HGNC Symbol;Acc:HGNC:15962]
ENSG00000259230	46.2581576	33.6684583	75.8576493	91.5515881	-1.06939918	0.0146282	0.0524401	LINC02323	long intergenic non-protein coding RNA 2323 [Source:HGNC Symbol;Acc:HGNC:53242]
ENSG00000062582	47.2423737	34.5544704	81.1255416	88.3579281	-1.053825099	0.0146291	0.0524401	MRPS24	mitochondrial ribosomal protein S24 [Source:HGNC Symbol;Acc:HGNC:14510]
ENSG00000064703	394.670663	495.280743	656.379382	562.084169	-0.45224844	0.014658	0.0525326	DDX20	DEAD-box helicase 20 [Source:HGNC Symbol;Acc:HGNC:2743]
ENSG00000261143	15.7474579	9.74613268	0	1.06455335	4.586433126	0.0147036	0.0526798	ADAMTS7P3	ADAMTS7 pseudogene 3 [Source:HGNC Symbol;Acc:HGNC:49409]
ENSG00000066923	62.9898316	71.766977	31.6073539	27.6783871	1.185125688	0.0147053	0.0526798	STAG3	stromal antigen 3 [Source:HGNC Symbol;Acc:HGNC:11356]
ENSG00000273706	109.247989	84.1711459	164.35824	160.747556	-0.751207691	0.0147633	0.0528763	LHX1	LIM homeobox 1 [Source:HGNC Symbol;Acc:HGNC:6593]
ENSG00000174282	1573.76157	1384.83685	1831.11937	1825.709	-0.306082433	0.0147769	0.0529137	ZBTB4	zinc finger and BTB domain containing 4 [Source:HGNC Symbol;Acc:HGNC:23847]
ENSG00000182670	3156.38109	3379.25	2842.55469	2546.41161	0.278411303	0.0147881	0.0529425	TTC3	tetratricopeptide repeat domain 3 [Source:HGNC Symbol;Acc:HGNC:12393]
ENSG00000179029	395.65488	410.223585	269.716086	305.526812	0.486649207	0.014802	0.0529812	TMEM107	transmembrane protein 107 [Source:HGNC Symbol;Acc:HGNC:28128]
ENSG00000174371	1215.50691	1293.57761	950.327773	1045.39139	0.330496656	0.0148135	0.0530112	EXO1	exonuclease 1 [Source:HGNC Symbol;Acc:HGNC:3511]
ENSG00000171723	709.619821	727.415903	563.664477	538.663995	0.382620618	0.0148385	0.0530894	GPHN	gephyrin [Source:HGNC Symbol;Acc:HGNC:15465]
ENSG00000042286	1539.31401	1389.26691	1738.40446	1884.25943	-0.307149634	0.0148493	0.0531069	AIFM2	apoptosis inducing factor, mitochondria associated 2 [Source:HGNC Symbol;Acc:HGNC:21411]
ENSG00000251432	40.3528608	38.9845307	15.8036769	9.58098015	1.643264383	0.0148497	0.0531069	AC108062.1	uncharacterized LOC100507487 [Source:NCBI gene;Acc:100507487]
ENSG00000100234	6063.7555	5951.34302	7194.88732	6968.56623	-0.237352748	0.0148667	0.0531511	TIMP3	TIMP metallopeptidase inhibitor 3 [Source:HGNC Symbol;Acc:HGNC:11822]
ENSG00000236698	245.069813	271.119691	404.57413	335.334305	-0.518915714	0.0148683	0.0531511	EIF1AXP1	EIF1AX pseudogene 1 [Source:HGNC Symbol;Acc:HGNC:19900]
ENSG00000232324	15.7474579	15.9482171	42.1431385	46.8403474	-1.489050431	0.0148863	0.0532041	AC008440.3	novel transcript
ENSG00000100580	930.084232	930.312665	754.362179	702.605211	0.352605209	0.0148923	0.0532041	TMED8	transmembrane p24 trafficking protein family member 8 [Source:HGNC Symbol;Acc:HGNC:18633]
ENSG00000010270	504.902869	552.871527	398.252659	385.368313	0.433194626	0.0148926	0.0532041	STARD3NL	STARD3 N-terminal like [Source:HGNC Symbol;Acc:HGNC:19169]
ENSG00000176490	45.2739414	35.4404825	13.69652	12.7746402	1.607132436	0.014904	0.0532336	DIRAS1	DIRAS family GTPase 1 [Source:HGNC Symbol;Acc:HGNC:19127]
ENSG00000116574	328.728183	321.622378	229.680105	222.49165	0.524211202	0.0149458	0.0533717	RHOU	ras homolog family member U [Source:HGNC Symbol;Acc:HGNC:17794]
ENSG00000043591	22.6369707	26.5803619	4.21431385	6.3873201	2.216240949	0.0149601	0.0534113	ADRB1	adrenoceptor beta 1 [Source:HGNC Symbol;Acc:HGNC:285]
ENSG00000163798	510.808165	569.705756	666.915167	761.155645	-0.401753512	0.0149817	0.053477	SLC4A1AP	solute carrier family 4 member 1 adaptor protein [Source:HGNC Symbol;Acc:HGNC:13813]
ENSG00000136371	58.068751	54.0467358	103.250689	107.519888	-0.911304817	0.0150615	0.0537505	MTHFS	methenyltetrahydrofolate synthetase [Source:HGNC Symbol;Acc:HGNC:7437]
ENSG00000138639	156.490363	132.015797	72.6969139	92.6161415	0.802761907	0.0150662	0.053756	ARHGAP24	Rho GTPase activating protein 24 [Source:HGNC Symbol;Acc:HGNC:25361]
ENSG00000106052	2279.44453	2806.88621	2095.56756	1991.77932	0.315807814	0.0150711	0.0537622	TAX1BP1	Tax1 binding protein 1 [Source:HGNC Symbol;Acc:HGNC:11575]
ENSG00000162972	189.953711	180.746461	348.734471	226.749864	-0.634989116	0.0150988	0.0538496	MAIP1	matrix AAA peptidase interacting protein 1 [Source:HGNC Symbol;Acc:HGNC:26198]
ENSG00000146830	1683.99378	1409.64519	1873.26251	1984.32744	-0.318870068	0.0151147	0.0538949	GIGYF1	GRB10 interacting GYF protein 1 [Source:HGNC Symbol;Acc:HGNC:9126]
ENSG00000106785	1087.55881	1111.94514	1368.59842	1359.43463	-0.310595021	0.0151367	0.0539621	TRIM14	tripartite motif containing 14 [Source:HGNC Symbol;Acc:HGNC:16283]
ENSG00000186666	157.474579	157.710147	82.1791201	104.326228	0.757191897	0.0151553	0.0540168	BCDIN3D	BCDIN3 domain containing RNA methyltransferase [Source:HGNC Symbol;Acc:HGNC:27050]
ENSG00000143633	268.691	304.788149	378.234668	429.015	-0.49243662	0.0151671	0.0540476	C1orf131	chromosome 1 open reading frame 131 [Source:HGNC Symbol;Acc:HGNC:25332]
ENSG00000116212	1091.49568	1087.1368	1370.70558	1332.82079	-0.311446469	0.0152025	0.0541554	LRRC42	leucine rich repeat containing 42 [Source:HGNC Symbol;Acc:HGNC:28792]
ENSG00000171033	54.1318865	85.0571579	32.6609323	27.6783871	1.207794978	0.0152038	0.0541554	PKIA	cAMP-dependent protein kinase inhibitor alpha [Source:HGNC Symbol;Acc:HGNC:9017]
ENSG00000079974	207.669601	186.948545	277.091136	303.397705	-0.557489846	0.0152259	0.0542228	RABL2B	RAB, member of RAS oncogene family like 2B [Source:HGNC Symbol;Acc:HGNC:9800]
ENSG00000132702	21.6527546	26.5803619	2.10715692	7.45187345	2.337656304	0.0152424	0.0542636	HAPLN2	hyaluronan and proteoglycan link protein 2 [Source:HGNC Symbol;Acc:HGNC:17410]
ENSG00000173486	1050.1586	886.898074	1142.07905	1349.85365	-0.363917492	0.0152438	0.0542636	FKBP2	FK506 binding protein 2 [Source:HGNC Symbol;Acc:HGNC:3718]
ENSG00000158711	412.386554	481.10455	787.023111	496.081861	-0.521697987	0.0152493	0.0542719	ELK4	ELK4, ETS transcription factor [Source:HGNC Symbol;Acc:HGNC:3326]
ENSG00000108641	363.175748	304.788149	466.735259	455.628834	-0.466607752	0.0152659	0.0543196	B9D1	B9 domain containing 1 [Source:HGNC Symbol;Acc:HGNC:24123]
ENSG00000162341	304.122781	247.197365	400.359816	381.110099	-0.504581728	0.0152825	0.054367	TPCN2	two pore segment channel 2 [Source:HGNC Symbol;Acc:HGNC:20820]
ENSG00000162852	889.731371	946.260882	730.129874	707.927978	0.35266097	0.0152918	0.0543887	CNST	consortin, connexin sorting protein [Source:HGNC Symbol;Acc:HGNC:26486]
ENSG00000107679	472.423737	590.970045	414.056336	354.496266	0.469222042	0.0153037	0.0544195	PLEKHA1	pleckstrin homology domain containing A1 [Source:HGNC Symbol;Acc:HGNC:14335]
ENSG00000159200	705.682957	749.566204	584.736047	528.018462	0.387318656	0.0153105	0.0544323	RCAN1	regulator of calcineurin 1 [Source:HGNC Symbol;Acc:HGNC:3040]

ENSG00000126804	461.597359	655.648926	828.112671	708.992531	-0.459048936	0.0153144	0.0544348	ZBTB1	zinc finger and BTB domain containing 1 [Source:HGNC Symbol;Acc:HGNC:20259]
ENSG00000060642	363.175748	337.570596	260.23388	229.943524	0.515145216	0.0153219	0.0544501	PIGV	phosphatidylinositol glycan anchor biosynthesis class V [Source:HGNC Symbol;Acc:HGNC:26031]
ENSG00000076826	180.11155	186.062533	110.625739	117.100869	0.68544838	0.0153478	0.0545192	CAMSAP3	calmodulin regulated spectrin associated protein family member 3 [Source:HGNC Symbol;Acc:HGNC:29307]
ENSG00000196150	349.396722	297.700053	208.608536	235.26629	0.543158783	0.0153508	0.0545192	ZNF250	zinc finger protein 250 [Source:HGNC Symbol;Acc:HGNC:13044]
ENSG00000133056	1289.32311	1136.75348	1567.72475	1461.63175	-0.320842833	0.0153511	0.0545192	PIK3C2B	phosphatidylinositol-4-phosphate 3-kinase catalytic subunit type 2 beta [Source:HGNC Symbol;Acc:HGNC:8972]
ENSG00000112394	71.8477766	85.0571579	30.5537754	42.582134	1.102404005	0.0153574	0.0545303	SLC16A10	solute carrier family 16 member 10 [Source:HGNC Symbol;Acc:HGNC:17027]
ENSG00000100116	207.669601	187.834557	263.394616	323.624218	-0.570269607	0.0153743	0.0545787	GCAT	glycine C-acetyltransferase [Source:HGNC Symbol;Acc:HGNC:4188]
ENSG00000188997	205.701169	163.026219	89.5541693	129.875509	0.748047834	0.0153786	0.0545825	KCTD21	potassium channel tetramerization domain containing 21 [Source:HGNC Symbol;Acc:HGNC:27452]
ENSG00000228873	51.1792381	55.8187599	21.0715692	21.291067	1.33717154	0.0153872	0.0546017	AC012307.1	novel transcript
ENSG00000105254	1240.11231	1119.03323	1365.43769	1613.86288	-0.336991687	0.0154051	0.0546536	TBCB	tubulin folding cofactor B [Source:HGNC Symbol;Acc:HGNC:1989]
ENSG00000176102	497.02914	520.08908	656.379382	677.055931	-0.390395193	0.015422	0.0547021	CSTF3	cleavage stimulation factor subunit 3 [Source:HGNC Symbol;Acc:HGNC:2485]
ENSG00000007384	494.076491	408.45156	333.984373	314.043238	0.477050988	0.015431	0.0547133	RHBDF1	rhomboid 5 homolog 1 [Source:HGNC Symbol;Acc:HGNC:20561]
ENSG00000115107	1170.23296	957.779039	1362.27695	1339.20811	-0.344930983	0.0154316	0.0547133	STEAP3	STEAP3 metalloreductase [Source:HGNC Symbol;Acc:HGNC:24592]
ENSG00000157214	51.1792381	84.1711459	24.2323046	33.0011539	1.244282391	0.0154528	0.054777	STEAP2	STEAP2 metalloreductase [Source:HGNC Symbol;Acc:HGNC:17885]
ENSG00000156858	565.924268	452.752164	697.468942	664.281291	-0.419846494	0.0154707	0.054829	PRR14	proline rich 14 [Source:HGNC Symbol;Acc:HGNC:28458]
ENSG00000166971	259.833055	265.803619	165.411819	186.296836	0.579893894	0.015487	0.0548752	AKTIP	AKT interacting protein [Source:HGNC Symbol;Acc:HGNC:16710]
ENSG00000204650	410.418121	385.415247	287.62692	283.171191	0.479166246	0.0154955	0.054887	LINC02210	long intergenic non-protein coding RNA 2210 [Source:HGNC Symbol;Acc:HGNC:26327]
ENSG00000249087	108.263773	102.777399	54.78608	57.4858809	0.910295408	0.0154968	0.054887	ZNF436-AS1	ZNF436 antisense RNA 1 [Source:HGNC Symbol;Acc:HGNC:25122]
ENSG00000140263	1036.37957	1004.73768	1332.77675	1219.97814	-0.322853439	0.0155318	0.0549992	SORD	sorbitol dehydrogenase [Source:HGNC Symbol;Acc:HGNC:11184]
ENSG00000224870	314.949158	290.611956	419.324228	418.369467	-0.468664899	0.0155991	0.0552261	AL391244.1	uncharacterized LOC148413 [Source:NCBI gene;Acc:148413]
ENSG00000115216	2383.77144	2279.70903	2835.17964	2772.09692	-0.266014948	0.0156503	0.0553957	NRBP1	nuclear receptor binding protein 1 [Source:HGNC Symbol;Acc:HGNC:7993]
ENSG00000204175	21.6527546	26.5803619	53.7325016	62.8086477	-1.270564958	0.015666	0.0554395	GPRIN2	G protein regulated inducer of neurite outgrowth 2 [Source:HGNC Symbol;Acc:HGNC:23730]
ENSG00000102760	25.5896191	8.86012062	2.10715692	2.1291067	3.019842951	0.0156881	0.0555061	RGCC	regulator of cell cycle [Source:HGNC Symbol;Acc:HGNC:20369]
ENSG00000167971	31.4949158	39.8705428	5.26789231	14.9037469	1.825083454	0.015704	0.0555508	CASKIN1	CASK interacting protein 1 [Source:HGNC Symbol;Acc:HGNC:20879]
ENSG00000213445	1097.40097	1005.62369	835.487721	826.0934	0.339587366	0.0157309	0.0556342	SIPA1	signal-induced proliferation-associated 1 [Source:HGNC Symbol;Acc:HGNC:10885]
ENSG00000115761	392.702231	454.524188	546.807222	594.020769	-0.428426896	0.0157667	0.0557491	NOL10	nucleolar protein 10 [Source:HGNC Symbol;Acc:HGNC:25862]
ENSG00000050730	1.96843224	14.176193	0	0	5.377454612	0.0157678	1	TNIP3	TNFAIP3 interacting protein 3 [Source:HGNC Symbol;Acc:HGNC:19315]
ENSG00000105229	814.930946	691.97542	1013.54248	929.355075	-0.367337493	0.0157781	0.0557756	PIAS4	protein inhibitor of activated STAT 4 [Source:HGNC Symbol;Acc:HGNC:17002]
ENSG00000164053	496.044924	438.575971	638.468548	602.537196	-0.409750362	0.0157808	0.0557756	ATRIP	ATR interacting protein [Source:HGNC Symbol;Acc:HGNC:33499]
ENSG00000257702	60.0371832	76.1970373	120.107945	125.617295	-0.848946076	0.0157989	0.0558282	LBX2-AS1	LBX2 antisense RNA 1 [Source:HGNC Symbol;Acc:HGNC:25136]
ENSG00000104331	2854.22674	2869.79307	2525.42757	2163.17241	0.287843497	0.0158115	0.055861	IMPAD1	inositol monophosphatase domain containing 1 [Source:HGNC Symbol;Acc:HGNC:26019]
ENSG00000120217	40.3528608	38.0985187	7.37504924	17.0328536	1.685158901	0.0158474	0.055976	CD274	CD274 molecule [Source:HGNC Symbol;Acc:HGNC:17635]
ENSG00000236651	14.7632418	18.6062533	3.16073539	1.06455335	2.980415827	0.0158702	0.0560447	DLX2-DT	DLX2 divergent transcript [Source:HGNC Symbol;Acc:HGNC:50638]
ENSG00000141027	3156.38109	3132.93865	3883.49021	3629.06237	-0.256427855	0.0158893	0.0561005	NCOR1	nuclear receptor corepressor 1 [Source:HGNC Symbol;Acc:HGNC:7672]
ENSG00000052126	1270.62301	1346.73833	1057.79278	1046.45594	0.314998355	0.0158958	0.0561117	PLEKHA5	pleckstrin homology domain containing A5 [Source:HGNC Symbol;Acc:HGNC:30036]
ENSG00000198840	8521.34315	8927.45753	9212.49007	12592.6016	-0.321501996	0.0159089	0.0561392	MT-ND3	mitochondrially encoded NADH:ubiquinone oxidoreductase core subunit 3 [Source:HGNC Symbol;Acc:HGNC:7458]
ENSG00000152601	1442.86083	1655.95654	2326.30124	1691.57527	-0.374499239	0.0159102	0.0561392	MBNL1	muscleblind like splicing regulator 1 [Source:HGNC Symbol;Acc:HGNC:6923]
ENSG00000158125	28.5422674	39.8705428	10.5357846	9.58098015	1.767301793	0.0159257	0.0561775	XDH	xanthine dehydrogenase [Source:HGNC Symbol;Acc:HGNC:12805]
ENSG00000271614	59.0529671	59.3628081	25.2858831	24.4847271	1.25048538	0.0159277	0.0561775	ATP2B1-AS1	ATP2B1 antisense RNA 1 [Source:HGNC Symbol;Acc:HGNC:27883]
ENSG00000107937	1107.24313	1329.9041	1558.24255	1506.34299	-0.329921247	0.0159387	0.0562045	GTPBP4	GTP binding protein 4 [Source:HGNC Symbol;Acc:HGNC:21535]
ENSG00000157800	483.250114	481.10455	361.377413	350.238052	0.43842338	0.0159958	0.056394	SLC37A3	solute carrier family 37 member 3 [Source:HGNC Symbol;Acc:HGNC:20651]
ENSG00000157693	693.872363	761.084361	903.970321	955.968909	-0.353825592	0.0160132	0.0564435	TMEM268	transmembrane protein 268 [Source:HGNC Symbol;Acc:HGNC:24513]
ENSG00000277459	33.463348	26.5803619	70.589757	63.873201	-1.16543324	0.0160683	0.056626	AP001527.2	novel transcript
ENSG00000186462	28.5422674	21.2642895	8.4286277	1.06455335	2.387992021	0.0160958	0.0567014	NAP1L2	nucleosome assembly protein 1 like 2 [Source:HGNC Symbol;Acc:HGNC:7638]
ENSG00000136925	274.596297	237.451233	356.10952	369.400013	-0.503636118	0.0160987	0.0567014	TSTD2	thiosulfate sulfurtransferase like domain containing 2 [Source:HGNC Symbol;Acc:HGNC:30087]
ENSG00000105879	263.76992	293.269992	365.591726	418.369467	-0.492274074	0.0160997	0.0567014	CBLL1	Cbl proto-oncogene like 1 [Source:HGNC Symbol;Acc:HGNC:21225]
ENSG00000120699	502.934436	559.959623	614.236244	828.222506	-0.439988988	0.0161046	0.0567068	EXOSC8	exosome component 8 [Source:HGNC Symbol;Acc:HGNC:17035]
ENSG00000149596	357.270451	350.860776	520.46776	447.112407	-0.450545233	0.0161485	0.0568495	JPH2	junctophilin 2 [Source:HGNC Symbol;Acc:HGNC:14202]
ENSG00000126432	6008.6394	4721.55828	6392.06053	6809.94778	-0.299288504	0.0161571	0.056868	PRDX5	peroxiredoxin 5 [Source:HGNC Symbol;Acc:HGNC:9355]

ENSG00000106853	3088.47018	3030.16125	2535.96336	2578.34821	0.258633341	0.0161738	0.0569146	PTGR1	prostaglandin reductase 1 [Source:HGNC Symbol;Acc:HGNC:18429]
ENSG00000184260	17.7158901	17.7202412	36.8752462	59.6149876	-1.444914853	0.0161945	0.0569757	HIST2H2AC	histone cluster 2 H2A family member c [Source:HGNC Symbol;Acc:HGNC:4738]
ENSG00000121289	465.534224	484.648598	580.521733	684.507804	-0.412610974	0.0162111	0.0570223	CEP89	centrosomal protein 89 [Source:HGNC Symbol;Acc:HGNC:25907]
ENSG00000163449	14.7632418	15.0622051	1.05357846	2.1291067	3.229743099	0.0162463	0.0571342	TMEM169	transmembrane protein 169 [Source:HGNC Symbol;Acc:HGNC:25130]
ENSG00000197978	106.295341	116.953592	66.3754431	54.2922209	0.88792271	0.0162709	0.0572087	GOLGA6L9	golgin A6 family-like 9 [Source:HGNC Symbol;Acc:HGNC:37229]
ENSG00000108819	3005.79602	2316.92154	2168.26448	2067.36261	0.32919236	0.0162888	0.0572598	PPP1R9B	protein phosphatase 1 regulatory subunit 9B [Source:HGNC Symbol;Acc:HGNC:9298]
ENSG00000064652	321.838671	345.544704	213.876428	250.170037	0.524700349	0.0163001	0.0572687	SNX24	sorting nexin 24 [Source:HGNC Symbol;Acc:HGNC:21533]
ENSG00000114698	139.758689	160.368183	100.089954	74.5187345	0.781875701	0.0163003	0.0572687	PLSCR4	phospholipid scramblase 4 [Source:HGNC Symbol;Acc:HGNC:16497]
ENSG00000068654	2122.95417	1968.7188	2546.49914	2411.21334	-0.277201051	0.0163015	0.0572687	POLR1A	RNA polymerase I subunit A [Source:HGNC Symbol;Acc:HGNC:17264]
ENSG00000107521	560.018971	482.876574	687.986736	683.443251	-0.395851372	0.0164039	0.0576165	HPS1	HPS1, biogenesis of lysosomal organelles complex 3 subunit 1 [Source:HGNC Symbol;Acc:HGNC:5163]
ENSG00000130313	1131.84854	921.452544	1281.15141	1328.56258	-0.346637635	0.016424	0.0576751	PGLS	6-phosphogluconolactonase [Source:HGNC Symbol;Acc:HGNC:8903]
ENSG00000111335	10.8263773	9.74613268	45.3038739	24.4847271	-1.763817095	0.0164291	0.0576811	OAS2	2'-5'-oligoadenylate synthetase 2 [Source:HGNC Symbol;Acc:HGNC:8087]
ENSG00000146242	2050.12217	2086.55841	2463.26644	2516.60412	-0.267578686	0.0164432	0.0577186	TPBG	trophoblast glycoprotein [Source:HGNC Symbol;Acc:HGNC:12004]
ENSG00000172733	19.6843224	15.9482171	2.10715692	3.19366005	2.748752159	0.0164646	0.0577709	PURG	purine rich element binding protein G [Source:HGNC Symbol;Acc:HGNC:17930]
ENSG00000110057	841.504781	704.379589	952.43493	1039.00407	-0.366070044	0.0164649	0.0577709	UNC93B1	unc-93 homolog B1, TLR signaling regulator [Source:HGNC Symbol;Acc:HGNC:13481]
ENSG00000104765	1364.12354	1450.40175	2109.26408	1527.63406	-0.369743626	0.0165008	0.0578846	BNIP3L	BCL2 interacting protein 3 like [Source:HGNC Symbol;Acc:HGNC:1085]
ENSG00000030582	12511.3553	10997.1817	9860.44083	9951.44472	0.246742737	0.0165174	0.057931	GRN	granulin precursor [Source:HGNC Symbol;Acc:HGNC:4601]
ENSG00000243547	172.237821	197.58069	247.590939	306.591365	-0.582529506	0.0165481	0.0580265	HNRNPKP4	heterogeneous nuclear ribonucleoprotein K pseudogene 4 [Source:HGNC Symbol;Acc:HGNC:42377]
ENSG00000005022	6129.69798	6428.90352	7779.62337	7104.82906	-0.245086559	0.016567	0.0580778	SLC25A5	solute carrier family 25 member 5 [Source:HGNC Symbol;Acc:HGNC:10991]
ENSG00000174791	264.754136	221.503015	383.50256	318.301452	-0.530521907	0.0165704	0.0580778	RIN1	Ras and Rab interactor 1 [Source:HGNC Symbol;Acc:HGNC:18749]
ENSG00000096717	488.171195	567.04772	846.023505	602.537196	-0.456614443	0.0165774	0.0580778	SIRT1	sirtuin 1 [Source:HGNC Symbol;Acc:HGNC:14929]
ENSG00000265354	1855.24738	1773.79615	2109.26408	2309.01622	-0.284001065	0.0165777	0.0580778	TIMM23	translocase of inner mitochondrial membrane 23 [Source:HGNC Symbol;Acc:HGNC:17312]
ENSG00000188070	573.797997	513.886996	678.50453	746.251899	-0.389971559	0.0165816	0.0580778	C11orf95	chromosome 11 open reading frame 95 [Source:HGNC Symbol;Acc:HGNC:28449]
ENSG00000139178	762.767492	644.130769	863.934339	959.162569	-0.374552973	0.0165834	0.0580778	C1RL	complement C1r subcomponent like [Source:HGNC Symbol;Acc:HGNC:21265]
ENSG00000152689	6.88951283	7.97410856	0	0	5.254112441	0.016584	1	RASGRP3	RAS guanyl releasing protein 3 [Source:HGNC Symbol;Acc:HGNC:14545]
ENSG00000259683	6.88951283	7.97410856	0	0	5.254112441	0.016584	1	AC243562.2	golgin A6 family-like 9 (GOLGA6L9) pseudogene
ENSG00000153253	6.88951283	7.97410856	0	0	5.254112441	0.016584	1	SCN3A	sodium voltage-gated channel alpha subunit 3 [Source:HGNC Symbol;Acc:HGNC:10590]
ENSG00000113318	210.622249	229.477124	339.252265	297.010385	-0.531292775	0.0166298	0.0582284	MSH3	mutS homolog 3 [Source:HGNC Symbol;Acc:HGNC:7326]
ENSG00000283674	47.2423737	27.4663739	14.7500985	7.45187345	1.747502508	0.0166342	0.0582318	AC068587.4	uncharacterized LOC729732 [Source:NCBI gene;Acc:729732]
ENSG00000088986	4630.73684	4698.52196	5259.46368	5851.84977	-0.252160103	0.0166496	0.0582738	DYNLL1	dynein light chain LC8-type 1 [Source:HGNC Symbol;Acc:HGNC:15476]
ENSG00000147316	246.05403	274.663739	386.663296	349.173499	-0.498232493	0.0166921	0.0584025	MCPH1	microcephalin 1 [Source:HGNC Symbol;Acc:HGNC:6954]
ENSG00000166780	1269.63879	1064.9865	929.256204	905.934901	0.346712622	0.0166955	0.0584025	C16orf45	chromosome 16 open reading frame 45 [Source:HGNC Symbol;Acc:HGNC:19213]
ENSG00000119705	1657.41994	1936.82237	2000.7455	2563.44447	-0.344320975	0.0166968	0.0584025	SLIRP	SRA stem-loop interacting RNA binding protein [Source:HGNC Symbol;Acc:HGNC:20495]
ENSG00000204128	96.4531796	71.766977	38.9824031	42.582134	1.042946158	0.0167037	0.0584147	C2orf72	chromosome 2 open reading frame 72 [Source:HGNC Symbol;Acc:HGNC:27418]
ENSG00000237289	21.6527546	25.6943498	7.37504924	2.1291067	2.315658973	0.0167078	0.0584169	CKMT1B	creatine kinase, mitochondrial 1B [Source:HGNC Symbol;Acc:HGNC:1995]
ENSG00000010818	802.136136	793.866807	579.468154	654.70031	0.370949217	0.0167484	0.0585465	HIVEP2	human immunodeficiency virus type I enhancer binding protein 2 [Source:HGNC Symbol;Acc:HGNC:4921]
ENSG00000244414	7.87372895	16.8342292	0	1.06455335	4.545671479	0.0167727	0.0586193	CFHR1	complement factor H related 1 [Source:HGNC Symbol;Acc:HGNC:4888]
ENSG00000131584	1430.06602	1162.44783	969.292185	1060.29514	0.352649387	0.0167785	0.0586275	ACAP3	ArfGAP with coiled-coil, ankyrin repeat and PH domains 3 [Source:HGNC Symbol;Acc:HGNC:16754]
ENSG00000161204	1331.64441	1206.74843	1626.72515	1515.92397	-0.308430612	0.0167864	0.0586431	ABCF3	ATP binding cassette subfamily F member 3 [Source:HGNC Symbol;Acc:HGNC:72]
ENSG00000073350	949.768554	880.695989	720.647668	716.444405	0.348761826	0.0167934	0.0586554	LLGL2	LLGL2, scribble cell polarity complex component [Source:HGNC Symbol;Acc:HGNC:6629]
ENSG00000116741	121.058583	105.435435	61.1075508	62.8086477	0.869403325	0.016832	0.0587781	RGS2	regulator of G protein signaling 2 [Source:HGNC Symbol;Acc:HGNC:9998]
ENSG00000135245	1047.20595	820.447169	656.379382	759.026539	0.399369839	0.0168375	0.0587851	HILPDA	hypoxia inducible lipid droplet associated [Source:HGNC Symbol;Acc:HGNC:28859]
ENSG00000284727	12.7948095	11.5181568	0	1.06455335	4.519372782	0.0168529	0.0588266	AC116562.4	RNA binding protein, fox-1 homolog (C. elegans) 1 (RBFOX1) pseudogene
ENSG00000130294	64.9582638	44.3006031	0	6.3873201	4.097761238	0.0168787	0.0589045	KIF1A	kinesin family member 1A [Source:HGNC Symbol;Acc:HGNC:888]
ENSG00000163412	254.911975	296.814041	192.804859	179.909516	0.566581959	0.0169124	0.0589958	EIF4E3	eukaryotic translation initiation factor 4E family member 3 [Source:HGNC Symbol;Acc:HGNC:31837]
ENSG00000137876	843.473213	1172.19396	1288.52646	1349.85365	-0.387482713	0.0169138	0.0589958	RSL24D1	ribosomal L24 domain containing 1 [Source:HGNC Symbol;Acc:HGNC:18479]
ENSG00000165948	592.498103	479.332525	354.002363	423.692233	0.462101505	0.0169153	0.0589958	IFI27L1	interferon alpha inducible protein 27 like 1 [Source:HGNC Symbol;Acc:HGNC:19754]
ENSG00000179598	58.068751	48.7306634	85.3398554	120.294529	-0.946464471	0.0169523	0.0591128	PLD6	phospholipase D family member 6 [Source:HGNC Symbol;Acc:HGNC:30447]
ENSG00000077585	270.659433	306.560173	189.644123	204.394243	0.551460791	0.0170073	0.0592814	GPR137B	G protein-coupled receptor 137B [Source:HGNC Symbol;Acc:HGNC:11862]
ENSG00000125821	407.465473	324.280415	479.3782	526.953908	-0.460864588	0.0170077	0.0592814	DTD1	D-tyrosyl-tRNA deacylase 1 [Source:HGNC Symbol;Acc:HGNC:16219]
ENSG00000110436	11.8105934	12.4041689	0	1.06455335	4.514121931	0.0170546	0.0594325	SLC1A2	solute carrier family 1 member 2 [Source:HGNC Symbol;Acc:HGNC:10940]

ENSG00000196313	1176.13826	1088.02281	1459.20617	1353.04731	-0.313068972	0.0170602	0.0594354	POM121	POM121 transmembrane nucleoporin [Source:HGNC Symbol;Acc:HGNC:19702]
ENSG00000196391	186.016846	179.860449	132.750886	88.3579281	0.726141486	0.0170644	0.0594354	ZNF774	zinc finger protein 774 [Source:HGNC Symbol;Acc:HGNC:33108]
ENSG00000123607	344.475641	408.45156	268.662508	265.073784	0.497167418	0.0170682	0.0594354	TTC21B	tetratricopeptide repeat domain 21B [Source:HGNC Symbol;Acc:HGNC:25660]
ENSG00000198899	41789.8164	43395.9848	46877.9201	53861.0768	-0.241928913	0.0170695	0.0594354	MT-ATP6	mitochondrially encoded ATP synthase membrane subunit 6 [Source:HGNC Symbol;Acc:HGNC:7414]
ENSG00000283405	4.92108059	5.31607237	25.2858831	21.291067	-2.184757757	0.0170863	0.0594816	AL121759.2	novel transcript
ENSG00000143374	1147.59599	1112.83115	1374.91989	1415.85596	-0.304182996	0.0171003	0.059518	TARS2	threonyl-tRNA synthetase 2, mitochondrial [Source:HGNC Symbol;Acc:HGNC:30740]
ENSG00000134780	176.174685	128.471749	246.53736	225.68531	-0.63447015	0.0171093	0.0595373	DAGLA	diacylglycerol lipase alpha [Source:HGNC Symbol;Acc:HGNC:1165]
ENSG00000171435	67.9109122	80.6270976	26.3394616	41.5175807	1.131529739	0.0171264	0.0595847	KSR2	kinase suppressor of ras 2 [Source:HGNC Symbol;Acc:HGNC:18610]
ENSG00000180357	2089.49082	2009.47536	2523.32042	2416.53611	-0.269330195	0.0171478	0.0596466	ZNF609	zinc finger protein 609 [Source:HGNC Symbol;Acc:HGNC:29003]
ENSG00000174136	158.458795	194.922654	120.107945	94.7452482	0.718683456	0.0171672	0.0597017	RGMB	repulsive guidance molecule BMP co-receptor b [Source:HGNC Symbol;Acc:HGNC:26896]
ENSG00000248515	5.90529671	8.86012062	0	0	5.245420986	0.01717	1	AC024230.1	novel transcript
ENSG00000279289	5.90529671	8.86012062	0	0	5.245420986	0.01717	1	AL136164.3	TEC
ENSG00000185272	5.90529671	8.86012062	0	0	5.245420986	0.01717	1	RBM11	RNA binding motif protein 11 [Source:HGNC Symbol;Acc:HGNC:9897]
ENSG00000173890	50.195022	50.5026875	24.2323046	13.8391936	1.402662038	0.01718	0.0597267	GPR160	G protein-coupled receptor 160 [Source:HGNC Symbol;Acc:HGNC:23693]
ENSG00000178951	1436.95553	1400.78507	1704.68995	1759.70669	-0.287941491	0.0171826	0.0597267	ZBTB7A	zinc finger and BTB domain containing 7A [Source:HGNC Symbol;Acc:HGNC:18078]
ENSG00000178919	324.791319	320.736366	433.020748	448.17696	-0.449048175	0.0171849	0.0597267	FOXE1	forkhead box E1 [Source:HGNC Symbol;Acc:HGNC:3806]
ENSG00000105618	1016.69525	817.789133	1119.95391	1235.94644	-0.361626985	0.0171902	0.0597326	PRPF31	pre-mRNA processing factor 31 [Source:HGNC Symbol;Acc:HGNC:15446]
ENSG00000160051	71.8477766	59.3628081	107.465003	129.875509	-0.856556974	0.0172079	0.0597766	IQCC	IQ motif containing C [Source:HGNC Symbol;Acc:HGNC:25545]
ENSG00000174943	417.307634	396.933404	260.23388	320.430558	0.487663116	0.0172099	0.0597766	KCTD13	potassium channel tetramerization domain containing 13 [Source:HGNC Symbol;Acc:HGNC:22234]
ENSG00000134769	207.669601	217.958967	145.393828	129.875509	0.628911151	0.0172216	0.0597959	DTNA	dystrobrevin alpha [Source:HGNC Symbol;Acc:HGNC:3057]
ENSG00000163507	612.182426	777.032578	1066.2214	803.737779	-0.428040122	0.0172226	0.0597959	CIP2A	cell proliferation regulating inhibitor of protein phosphatase 2A [Source:HGNC Symbol;Acc:HGNC:29302]
ENSG00000151725	194.874791	227.7051	310.805646	302.333151	-0.535956158	0.0172308	0.0598121	CENPU	centromere protein U [Source:HGNC Symbol;Acc:HGNC:21348]
ENSG00000167985	264.754136	208.212835	329.770059	352.367159	-0.529771559	0.0172456	0.0598513	SDHAF2	succinate dehydrogenase complex assembly factor 2 [Source:HGNC Symbol;Acc:HGNC:26034]
ENSG00000145861	36.4159964	59.3628081	22.1251477	11.7100869	1.502585227	0.017252	0.0598613	C1QTNF2	C1q and TNF related 2 [Source:HGNC Symbol;Acc:HGNC:14325]
ENSG00000074276	81.6899378	53.1607237	35.8216677	21.291067	1.237092356	0.017261	0.0598737	CDHR2	cadherin related family member 2 [Source:HGNC Symbol;Acc:HGNC:18231]
ENSG00000131669	1210.58583	1167.7639	1365.43769	1609.60467	-0.323020619	0.0172627	0.0598737	NINJ1	ninjurin 1 [Source:HGNC Symbol;Acc:HGNC:7824]
ENSG00000214106	196.843224	160.368183	109.57216	110.713548	0.696368664	0.017273	0.0598974	PAXIP1-AS2	PAXIP1 antisense RNA 2 [Source:HGNC Symbol;Acc:HGNC:48958]
ENSG00000151923	1473.37153	1519.51069	1791.08339	1855.51649	-0.284899574	0.017278	0.0599022	TIAL1	TIA1 cytotoxic granule associated RNA binding protein like 1 [Source:HGNC Symbol;Acc:HGNC:11804]
ENSG00000139618	231.290788	266.689631	383.50256	327.882432	-0.513733189	0.0172835	0.0599089	BRCA2	BRCA2, DNA repair associated [Source:HGNC Symbol;Acc:HGNC:1101]
ENSG00000139372	624.977235	730.959951	929.256204	829.28706	-0.374452444	0.0172874	0.0599104	TDG	thymine DNA glycosylase [Source:HGNC Symbol;Acc:HGNC:11700]
ENSG00000099817	4090.40219	3274.70058	4395.52934	4642.51716	-0.29558603	0.0173319	0.0600522	POLR2E	RNA polymerase II subunit E [Source:HGNC Symbol;Acc:HGNC:9192]
ENSG00000006740	352.34937	313.64827	226.519369	239.524504	0.514519189	0.0173582	0.0601309	ARHGAP44	Rho GTPase activating protein 44 [Source:HGNC Symbol;Acc:HGNC:29096]
ENSG00000146007	1295.22841	1372.43268	1595.11779	1679.86519	-0.295688978	0.017383	0.0602047	ZMAT2	zinc finger matrin-type 2 [Source:HGNC Symbol;Acc:HGNC:26433]
ENSG00000103599	66.926696	78.8550735	44.2502954	17.0328536	1.250100462	0.0173896	0.0602151	IQCH	IQ motif containing H [Source:HGNC Symbol;Acc:HGNC:25721]
ENSG00000184486	31.4949158	27.4663739	7.37504924	8.5164268	1.891065134	0.0174125	0.060282	POU3F2	POU class 3 homeobox 2 [Source:HGNC Symbol;Acc:HGNC:9215]
ENSG00000173295	136.80604	129.357761	88.5005908	61.7440943	0.824419095	0.0174227	0.060305	FAM86B3P	family with sequence similarity 86 member B3, pseudogene [Source:HGNC Symbol;Acc:HGNC:44371]
ENSG00000100354	926.147367	878.923965	1124.16822	1132.68476	-0.322484729	0.0174321	0.060325	TNRC6B	trinucleotide repeat containing 6B [Source:HGNC Symbol;Acc:HGNC:29190]
ENSG00000184194	201.764304	164.798243	124.322259	101.132568	0.700068805	0.0174532	0.0603858	GPR173	G protein-coupled receptor 173 [Source:HGNC Symbol;Acc:HGNC:18186]
ENSG00000115423	37.4002125	29.238398	4.21431385	13.8391936	1.884414169	0.0174685	0.0604264	DNAH6	dynein axonemal heavy chain 6 [Source:HGNC Symbol;Acc:HGNC:2951]
ENSG00000131153	1225.34907	1183.71211	1438.1346	1530.82772	-0.301597075	0.0174725	0.0604279	GINS2	GINS complex subunit 2 [Source:HGNC Symbol;Acc:HGNC:24575]
ENSG00000133142	1183.02777	1223.58266	1333.83033	1739.48017	-0.352633173	0.0175106	0.0605473	TCEAL4	transcription elongation factor A like 4 [Source:HGNC Symbol;Acc:HGNC:26121]
ENSG00000273230	139.758689	120.49764	68.4826001	79.8415013	0.810547617	0.0175276	0.0605935	AC102953.2	novel transcript
ENSG00000101464	561.987403	521.861104	711.165462	696.217891	-0.377258114	0.0175378	0.0606166	PIGU	phosphatidylinositol glycan anchor biosynthesis class U [Source:HGNC Symbol;Acc:HGNC:15791]
ENSG00000101084	1090.51146	1289.14755	902.916742	973.001762	0.34369061	0.0175536	0.0606588	RAB5IF	RAB5 interacting factor [Source:HGNC Symbol;Acc:HGNC:15870]
ENSG00000273344	222.432843	178.088424	127.482994	125.617295	0.661049597	0.0175704	0.0607	PAXIP1-AS1	PAXIP1 antisense RNA 1 (head to head) [Source:HGNC Symbol;Acc:HGNC:27328]
ENSG00000082497	173.222037	222.389028	124.322259	124.552742	0.669885548	0.0175727	0.0607	SERTAD4	SERTA domain containing 4 [Source:HGNC Symbol;Acc:HGNC:25236]
ENSG00000126768	1319.83381	1116.3752	1480.27774	1564.89342	-0.322415318	0.0175907	0.0607317	TIMM17B	translocase of inner mitochondrial membrane 17B [Source:HGNC Symbol;Acc:HGNC:17310]
ENSG00000174109	361.207315	280.865824	409.842022	493.952755	-0.494486904	0.0175921	0.0607317	C16orf91	chromosome 16 open reading frame 91 [Source:HGNC Symbol;Acc:HGNC:27558]

ENSG00000274602	220.46441	240.995281	134.858043	166.070323	0.617385289	0.0175927	0.0607317	PI4KAP1	phosphatidylinositol 4-kinase alpha pseudogene 1 [Source:HGNC Symbol;Acc:HGNC:33576]
ENSG00000256223	138.774473	171.88634	88.5005908	96.8743549	0.746055515	0.0176015	0.0607496	ZNF10	zinc finger protein 10 [Source:HGNC Symbol;Acc:HGNC:12879]
ENSG00000269825	132.869176	169.228304	102.197111	72.3896278	0.791911463	0.0176318	0.0608329	AC022150.4	novel transcript, sense intronic to ZNF83
ENSG00000184990	3090.43861	2734.23322	3417.80853	3602.44854	-0.269577215	0.0176328	0.0608329	SIVA1	SIVA1 apoptosis inducing factor [Source:HGNC Symbol;Acc:HGNC:17712]
ENSG00000266472	1424.16072	1328.13208	1608.81431	1783.12686	-0.301676707	0.0176707	0.0609512	MRPS21	mitochondrial ribosomal protein S21 [Source:HGNC Symbol;Acc:HGNC:14046]
ENSG00000085871	471.439521	507.684911	566.825213	760.091092	-0.438082735	0.0176996	0.0610384	MGST2	microsomal glutathione S-transferase 2 [Source:HGNC Symbol;Acc:HGNC:7063]
ENSG00000036530	8.85794506	18.6062533	2.10715692	0	3.70219942	0.017707	0.0610515	CYP46A1	cytochrome P450 family 46 subfamily A member 1 [Source:HGNC Symbol;Acc:HGNC:2641]
ENSG00000173894	1781.43117	1760.50597	2094.51398	2187.65713	-0.273828095	0.0177662	0.0612429	CBX2	chromobox 2 [Source:HGNC Symbol;Acc:HGNC:1552]
ENSG00000197632	15.7474579	16.8342292	1.05357846	3.19366005	2.941693536	0.0178323	0.0614584	SERPINB2	serpin family B member 2 [Source:HGNC Symbol;Acc:HGNC:8584]
ENSG00000161057	2234.17059	2356.79208	2613.92817	2952.00644	-0.27767652	0.0178449	0.0614893	PSMC2	proteasome 26S subunit, ATPase 2 [Source:HGNC Symbol;Acc:HGNC:9548]
ENSG00000130558	3015.63819	2914.97968	2521.21326	2437.82717	0.258040215	0.0178528	0.0615038	OLFM1	olfactomedin 1 [Source:HGNC Symbol;Acc:HGNC:17187]
ENSG00000232533	248.022462	200.238726	134.858043	154.360236	0.631248318	0.0178851	0.0616025	AC093673.1	novel transcript
ENSG00000163935	432.070876	483.762586	595.271831	615.311836	-0.401906777	0.0179525	0.0618222	SFMBT1	Scm like with four mbt domains 1 [Source:HGNC Symbol;Acc:HGNC:20255]
ENSG00000120457	4.92108059	9.74613268	0	0	5.236652144	0.0179783	1	KCNJ5	potassium voltage-gated channel subfamily J member 5 [Source:HGNC Symbol;Acc:HGNC:6266]
ENSG00000188002	92.5163151	73.5390011	126.429415	159.683003	-0.786567447	0.0179871	0.0619286	AC026412.1	programmed cell death 6 pseudogene [Source:NCBI gene;Acc:728613]
ENSG00000123066	1864.10533	1920.87415	1661.49324	1396.694	0.307624085	0.0180139	0.0620084	MED13L	mediator complex subunit 13 like [Source:HGNC Symbol;Acc:HGNC:22962]
ENSG00000171992	1340.50235	1220.92462	1064.11425	986.840956	0.320321022	0.0180236	0.0620291	SYNPO	synaptopodin [Source:HGNC Symbol;Acc:HGNC:30672]
ENSG00000138795	64.9582638	78.8550735	30.5537754	36.1948139	1.108549332	0.0180817	0.0622164	LEF1	lymphoid enhancer binding factor 1 [Source:HGNC Symbol;Acc:HGNC:6551]
ENSG00000127585	1155.46972	954.234991	1325.40171	1333.88535	-0.334658746	0.0181149	0.0623179	FBXL16	F-box and leucine rich repeat protein 16 [Source:HGNC Symbol;Acc:HGNC:14150]
ENSG00000105438	3847.30081	3486.45746	4176.38502	4631.87163	-0.264446165	0.018142	0.06239	KDELR1	KDEL endoplasmic reticulum protein retention receptor 1 [Source:HGNC Symbol;Acc:HGNC:6304]
ENSG00000125386	464.550008	505.026875	595.271831	686.636911	-0.402369382	0.0181432	0.06239	FAM193A	family with sequence similarity 193 member A [Source:HGNC Symbol;Acc:HGNC:16822]
ENSG00000121417	232.275004	212.642895	155.929612	134.133722	0.616600557	0.018168	0.0624625	ZNF211	zinc finger protein 211 [Source:HGNC Symbol;Acc:HGNC:13003]
ENSG00000181790	33.463348	19.4922654	6.32147077	6.3873201	2.056599713	0.0181875	0.0625168	ADGRB1	adhesion G protein-coupled receptor B1 [Source:HGNC Symbol;Acc:HGNC:943]
ENSG00000132646	5100.20792	5618.20248	6324.63151	6361.77082	-0.243066002	0.0182058	0.062567	PCNA	proliferating cell nuclear antigen [Source:HGNC Symbol;Acc:HGNC:8729]
ENSG00000184517	109.247989	158.596159	259.180302	178.844963	-0.707578824	0.0182108	0.0625715	ZFP1	ZFP1 zinc finger protein [Source:HGNC Symbol;Acc:HGNC:23328]
ENSG00000130222	24.605403	23.0363136	4.21431385	6.3873201	2.168264747	0.0182253	0.0625989	GADD45G	growth arrest and DNA damage inducible gamma [Source:HGNC Symbol;Acc:HGNC:4097]
ENSG00000225921	1096.41676	1127.89335	1273.77636	1516.98852	-0.327151629	0.0182262	0.0625989	NOL7	nucleolar protein 7 [Source:HGNC Symbol;Acc:HGNC:21040]
ENSG00000185090	771.625437	769.05847	996.685225	946.387928	-0.334807124	0.0182413	0.0626379	MANEAL	mannosidase endo-alpha like [Source:HGNC Symbol;Acc:HGNC:26452]
ENSG00000121552	27.5580513	41.6425669	6.32147077	13.8391936	1.781948238	0.0182523	0.062663	CSTA	cystatin A [Source:HGNC Symbol;Acc:HGNC:2481]
ENSG00000120875	990.121415	973.727256	787.023111	772.865732	0.332166293	0.0182702	0.0627089	DUSP4	dual specificity phosphatase 4 [Source:HGNC Symbol;Acc:HGNC:3070]
ENSG00000158423	20.6685385	15.0622051	43.196717	51.0985608	-1.403305842	0.0182731	0.0627089	RIBC1	RIB43A domain with coiled-coils 1 [Source:HGNC Symbol;Acc:HGNC:26537]
ENSG00000177600	8848.1029	7599.32545	9102.91791	10911.6718	-0.283274205	0.0182779	0.0627127	RPLP2	ribosomal protein lateral stalk subunit P2 [Source:HGNC Symbol;Acc:HGNC:10377]
ENSG00000240859	19.6843224	13.2901809	35.8216677	55.3567742	-1.471232596	0.0182899	0.0627401	AC093627.4	uncharacterized LOC100507642 [Source:NCBI gene;Acc:100507642]
ENSG00000136279	2225.31264	1895.1798	2492.76664	2540.02429	-0.288919593	0.0182933	0.0627401	DBNL	drebrin like [Source:HGNC Symbol;Acc:HGNC:2696]
ENSG00000152133	461.597359	538.695334	400.359816	327.882432	0.458434945	0.0183015	0.0627554	GPATCH11	G-patch domain containing 11 [Source:HGNC Symbol;Acc:HGNC:26768]
ENSG00000135404	8673.89665	7460.22156	9200.90071	10143.0643	-0.261873696	0.0183098	0.0627711	CD63	CD63 molecule [Source:HGNC Symbol;Acc:HGNC:1692]
ENSG00000154429	494.076491	601.60219	402.466973	409.85304	0.432522732	0.0183229	0.0628033	CCSAP	centriole, cilia and spindle associated protein [Source:HGNC Symbol;Acc:HGNC:29578]
ENSG00000171202	362.191531	403.135488	465.68168	580.181576	-0.449890456	0.0183275	0.0628033	TMEM126A	transmembrane protein 126A [Source:HGNC Symbol;Acc:HGNC:25382]
ENSG00000171488	454.707847	523.633129	392.984766	317.236898	0.462511203	0.0183341	0.0628033	LRRC8C	leucine rich repeat containing 8 VRAC subunit C [Source:HGNC Symbol;Acc:HGNC:25075]
ENSG00000182774	276.564729	306.560173	400.359816	405.594826	-0.46623171	0.0183362	0.0628033	RPS17	ribosomal protein S17 [Source:HGNC Symbol;Acc:HGNC:10397]
ENSG00000203722	155.506147	178.974436	87.4470124	114.971762	0.725491432	0.0183423	0.0628033	RAET1G	retinoic acid early transcript 1G [Source:HGNC Symbol;Acc:HGNC:16795]
ENSG00000142082	320.854455	280.865824	449.878069	386.432866	-0.475810302	0.0183434	0.0628033	SIRT3	sirtuin 3 [Source:HGNC Symbol;Acc:HGNC:14931]
ENSG00000011028	7039.11368	5991.21356	5474.39369	5393.02727	0.261713317	0.0183452	0.0628033	MRC2	mannose receptor C type 2 [Source:HGNC Symbol;Acc:HGNC:16875]
ENSG00000126583	17.7158901	28.352386	3.16073539	6.3873201	2.273526795	0.0183559	0.0628272	PRKCG	protein kinase C gamma [Source:HGNC Symbol;Acc:HGNC:9402]
ENSG00000171444	355.302019	398.705428	304.484176	216.10433	0.534684824	0.0183644	0.0628436	MCC	MCC, WNT signaling pathway regulator [Source:HGNC Symbol;Acc:HGNC:6935]
ENSG00000140521	2904.42176	2549.0567	3217.62862	3362.92403	-0.271289535	0.0183761	0.0628693	POLG	DNA polymerase gamma, catalytic subunit [Source:HGNC Symbol;Acc:HGNC:9179]
ENSG00000179632	3760.68979	3354.44167	4221.6889	4279.50447	-0.25697184	0.0183794	0.0628693	MAF1	MAF1 homolog, negative regulator of RNA polymerase III [Source:HGNC Symbol;Acc:HGNC:24966]
ENSG00000198682	2317.82896	2452.48139	2922.62665	2796.58165	-0.261601291	0.0183971	0.0629173	PAPSS2	3'-phosphoadenosine 5'-phosphosulfate synthase 2 [Source:HGNC Symbol;Acc:HGNC:8604]
ENSG00000114978	1473.37153	1931.50629	2490.65948	1920.45424	-0.373115279	0.0184306	0.0630192	MOB1A	MOB kinase activator 1A [Source:HGNC Symbol;Acc:HGNC:16015]
ENSG00000115091	3336.49264	3825.80008	4431.35101	4182.63011	-0.266047049	0.0184393	0.0630277	ACTR3	ARP3 actin related protein 3 homolog [Source:HGNC Symbol;Acc:HGNC:170]
ENSG00000185519	263.76992	188.720569	136.9652	151.166576	0.649739785	0.0184406	0.0630277	FAM131C	family with sequence similarity 131 member C [Source:HGNC Symbol;Acc:HGNC:26717]

ENSG00000163376	39.3686447	45.1866152	17.9108339	11.7100869	1.5134386	0.0184671	0.0631055	KBTBD8	kelch repeat and BTB domain containing 8 [Source:HGNC Symbol;Acc:HGNC:30691]
ENSG00000185432	1472.38731	1474.32407	1190.54366	1211.46171	0.294878064	0.0185028	0.0632147	METTL7A	methyltransferase like 7A [Source:HGNC Symbol;Acc:HGNC:24550]
ENSG00000198722	1106.25892	994.105533	856.55929	805.866886	0.336946814	0.0185388	0.0633249	UNC13B	unc-13 homolog B [Source:HGNC Symbol;Acc:HGNC:12566]
ENSG00000142185	40.3528608	39.8705428	71.6433354	91.5515881	-1.024473938	0.0185504	0.0633517	TRPM2	transient receptor potential cation channel subfamily M member 2 [Source:HGNC Symbol;Acc:HGNC:12339]
ENSG00000005513	20.6685385	15.0622051	4.21431385	1.06455335	2.755428196	0.0185752	0.0634209	SOX8	SRY-box 8 [Source:HGNC Symbol;Acc:HGNC:11203]
ENSG00000129691	677.140689	716.783758	848.130662	929.355075	-0.350377638	0.0185782	0.0634209	ASH2L	ASH2 like, histone lysine methyltransferase complex subunit [Source:HGNC Symbol;Acc:HGNC:744]
ENSG00000157111	114.16907	133.787821	69.5361785	71.3250745	0.816671856	0.0186098	0.0635162	TMEM171	transmembrane protein 171 [Source:HGNC Symbol;Acc:HGNC:27031]
ENSG00000270276	5.90529671	40.7565548	2.10715692	5.32276675	2.656677903	0.0186644	0.0636896	HIST2H4B	histone cluster 2 H4 family member b [Source:HGNC Symbol;Acc:HGNC:29607]
ENSG00000168564	301.170132	308.332198	444.610111	393.88474	-0.460087064	0.0187156	0.0638453	CDKN2AIP	CDKN2A interacting protein [Source:HGNC Symbol;Acc:HGNC:24325]
ENSG00000008853	913.352558	826.649254	639.522127	715.379851	0.360589804	0.0187176	0.0638453	RHOBTB2	Rho related BTB domain containing 2 [Source:HGNC Symbol;Acc:HGNC:18756]
ENSG00000105819	1266.68614	1357.37048	1578.26054	1638.34761	-0.293480952	0.0187497	0.0639418	PMPCB	peptidase, mitochondrial processing beta subunit [Source:HGNC Symbol;Acc:HGNC:9119]
ENSG00000177337	224.401275	225.047064	143.286671	153.295682	0.599792398	0.0187745	0.0640116	DLGAP1-AS1	DLGAP1 antisense RNA 1 [Source:HGNC Symbol;Acc:HGNC:31676]
ENSG00000004864	903.510397	1027.77399	1239.00827	1182.71877	-0.325984575	0.0187777	0.0640116	SLC25A13	solute carrier family 25 member 13 [Source:HGNC Symbol;Acc:HGNC:10983]
ENSG00000100139	1436.95553	1274.08534	1660.43966	1673.47787	-0.298771968	0.0188151	0.0641261	MICALL1	MICAL like 1 [Source:HGNC Symbol;Acc:HGNC:29804]
ENSG00000004866	315.933374	328.710475	433.020748	443.918747	-0.443711346	0.018821	0.0641334	ST7	suppression of tumorigenicity 7 [Source:HGNC Symbol;Acc:HGNC:11351]
ENSG00000117143	2257.79178	2723.60108	2079.76388	1969.4237	0.299246988	0.01888	0.0643215	UAP1	UDP-N-acetylglucosamine pyrophosphorylase 1 [Source:HGNC Symbol;Acc:HGNC:12457]
ENSG00000172661	628.9141	681.343276	869.202231	807.995993	-0.355845597	0.0188908	0.0643428	WASHC2C	WASH complex subunit 2C [Source:HGNC Symbol;Acc:HGNC:23414]
ENSG00000104427	148.616634	152.394075	85.3398554	95.8098015	0.732868242	0.0188939	0.0643428	ZC2HC1A	zinc finger C2HC-type containing 1A [Source:HGNC Symbol;Acc:HGNC:24277]
ENSG00000164889	5017.53377	4284.75433	5624.00183	5530.35465	-0.26215187	0.0189131	0.0643876	SLC4A2	solute carrier family 4 member 2 [Source:HGNC Symbol;Acc:HGNC:11028]
ENSG00000090238	241.132949	260.487546	165.411819	172.457643	0.570564706	0.0189147	0.0643876	YPEL3	yippee like 3 [Source:HGNC Symbol;Acc:HGNC:18327]
ENSG00000185716	203.732736	213.528907	141.179514	129.875509	0.622545576	0.0189225	0.0644011	MOSMO	modulator of smoothened [Source:HGNC Symbol;Acc:HGNC:27087]
ENSG00000089060	1317.86538	1097.76894	947.167038	962.356229	0.338652422	0.0189339	0.0644045	SLC8B1	solute carrier family 8 member B1 [Source:HGNC Symbol;Acc:HGNC:26175]
ENSG00000127252	16.731674	22.1503015	1.05357846	5.32276675	2.611675024	0.0189359	0.0644045	HRASLS	HRAS like suppressor [Source:HGNC Symbol;Acc:HGNC:14922]
ENSG00000177990	9.84216118	15.9482171	1.05357846	1.06455335	3.607913762	0.0189385	0.0644045	DPY19L2	dpy-19 like 2 [Source:HGNC Symbol;Acc:HGNC:19414]
ENSG00000204946	245.069813	174.544376	291.841234	330.011539	-0.569426662	0.0189387	0.0644045	ZNF783	zinc finger family member 783 [Source:HGNC Symbol;Acc:HGNC:27222]
ENSG00000134532	13.7790257	9.74613268	0	1.06455335	4.470907237	0.0190125	0.0646426	SOX5	SRY-box 5 [Source:HGNC Symbol;Acc:HGNC:11201]
ENSG00000277196	36.4159964	18.6062533	3.16073539	9.58098015	2.109239515	0.0190224	0.064663	AC007325.2	proline dehydrogenase 1, mitochondrial [Source:NCBI gene;Acc:102724788]
ENSG00000254470	1011.77417	840.825447	1237.95469	1117.78102	-0.347325717	0.0190384	0.0647044	AP5B1	adaptor related protein complex 5 subunit beta 1 [Source:HGNC Symbol;Acc:HGNC:25104]
ENSG00000164438	109.247989	109.865496	160.143926	191.619603	-0.6827826	0.0190887	0.0648623	TLX3	T cell leukemia homeobox 3 [Source:HGNC Symbol;Acc:HGNC:13532]
ENSG00000154359	304.122781	328.710475	246.53736	187.36139	0.544629864	0.0191035	0.0648998	LONRF1	LON peptidase N-terminal domain and ring finger 1 [Source:HGNC Symbol;Acc:HGNC:26302]
ENSG00000168137	3900.44848	3842.63431	4449.26185	4703.1967	-0.241268689	0.0191242	0.064957	SETD5	SET domain containing 5 [Source:HGNC Symbol;Acc:HGNC:25566]
ENSG00000196914	4168.15526	4256.40194	3743.36428	3295.85717	0.259190914	0.0191437	0.0650101	ARHGEF12	Rho guanine nucleotide exchange factor 12 [Source:HGNC Symbol;Acc:HGNC:14193]
ENSG00000083093	410.418121	471.358417	591.057517	578.052469	-0.406176443	0.0191632	0.0650632	PALB2	partner and localizer of BRCA2 [Source:HGNC Symbol;Acc:HGNC:26144]
ENSG00000089693	5584.44225	5037.86458	6176.07695	6404.35296	-0.244210969	0.0192427	0.0653201	MLF2	myeloid leukemia factor 2 [Source:HGNC Symbol;Acc:HGNC:7126]
ENSG00000165923	10.8263773	21.2642895	2.10715692	2.1291067	2.924008811	0.0192729	0.065393	AGBL2	ATP/GTP binding protein like 2 [Source:HGNC Symbol;Acc:HGNC:26296]
ENSG00000068097	612.182426	531.607237	718.540511	766.478412	-0.377325148	0.0192735	0.065393	HEATR6	HEAT repeat containing 6 [Source:HGNC Symbol;Acc:HGNC:24076]
ENSG00000272578	34.4475641	22.1503015	7.37504924	7.45187345	1.930537522	0.0192758	0.065393	AP000347.1	glucuronidase, beta (GUSB) pseudogene
ENSG00000168101	327.743967	242.767305	394.038345	413.0467	-0.502094574	0.0192886	0.0654017	NUDT16L1	nudix hydrolase 16 like 1 [Source:HGNC Symbol;Acc:HGNC:28154]
ENSG00000110844	192.906359	132.015797	95.8756401	96.8743549	0.751627916	0.0192895	0.0654017	PRPF40B	pre-mRNA processing factor 40 homolog B [Source:HGNC Symbol;Acc:HGNC:25031]
ENSG00000164323	215.54333	251.627426	395.091923	288.493958	-0.548440303	0.01929	0.0654017	CFAP97	cilia and flagella associated protein 97 [Source:HGNC Symbol;Acc:HGNC:29276]
ENSG00000152240	487.186979	534.265273	635.307813	700.476104	-0.386537993	0.0193281	0.0655177	HAUS1	HAUS augmin like complex subunit 1 [Source:HGNC Symbol;Acc:HGNC:25174]
ENSG00000089177	453.72363	474.902465	343.466579	344.915285	0.43211052	0.0193795	0.0656788	KIF16B	kinesin family member 16B [Source:HGNC Symbol;Acc:HGNC:15869]
ENSG00000184500	447.818334	527.177177	372.966776	344.915285	0.44230851	0.0193861	0.065688	PROS1	protein S [Source:HGNC Symbol;Acc:HGNC:9456]
ENSG00000135930	908.431477	812.473061	1059.89993	1102.87727	-0.330179246	0.0194131	0.0657665	EIF4E2	eukaryotic translation initiation factor 4E family member 2 [Source:HGNC Symbol;Acc:HGNC:3293]
ENSG00000237550	2677.06784	3008.01095	3137.55666	3867.52232	-0.301009764	0.0194304	0.0658119	RPL9P9	ribosomal protein L9 pseudogene 9 [Source:HGNC Symbol;Acc:HGNC:17251]
ENSG00000025293	1253.89133	1176.62402	1538.22455	1449.92166	-0.298244627	0.0194392	0.065827	PHF20	PHD finger protein 20 [Source:HGNC Symbol;Acc:HGNC:16098]
ENSG00000131732	419.276066	413.767633	502.556927	616.37639	-0.425638577	0.0194427	0.065827	ZCCHC9	zinc finger CCHC-type containing 9 [Source:HGNC Symbol;Acc:HGNC:25424]
ENSG00000183072	60.0371832	60.2488202	90.6077478	132.004615	-0.887837353	0.0194845	0.0659554	NKX2-5	NK2 homeobox 5 [Source:HGNC Symbol;Acc:HGNC:2488]
ENSG00000002746	206.685385	209.098847	136.9652	134.133722	0.617060091	0.0194953	0.0659787	HECW1	HECT, C2 and WW domain containing E3 ubiquitin protein ligase 1 [Source:HGNC Symbol;Acc:HGNC:22195]
ENSG00000085741	224.401275	186.948545	131.697308	133.069169	0.634723338	0.0194993	0.0659791	WNT11	Wnt family member 11 [Source:HGNC Symbol;Acc:HGNC:12776]
ENSG00000198954	1121.02216	1071.18858	1322.24097	1380.7257	-0.302323252	0.0195075	0.0659937	KIF1BP	KIF1 binding protein [Source:HGNC Symbol;Acc:HGNC:23419]

ENSG00000150551	22.6369707	14.176193	35.8216677	62.8086477	-1.426124546	0.0195376	0.066082	LYPD1	LY6/PLAUR domain containing 1 [Source:HGNC Symbol;Acc:HGNC:28431]
ENSG00000148308	2467.42981	2168.95753	2774.07209	2825.32459	-0.272566334	0.0195583	0.0661289	GTF3C5	general transcription factor IIIC subunit 5 [Source:HGNC Symbol;Acc:HGNC:4668]
ENSG00000102221	841.504781	864.747772	714.326197	615.311836	0.359822797	0.0195592	0.0661289	JADE3	jade family PHD finger 3 [Source:HGNC Symbol;Acc:HGNC:22982]
ENSG00000130023	165.348308	194.922654	271.823243	259.751017	-0.560072941	0.0195687	0.0661476	ERMARD	ER membrane associated RNA degradation [Source:HGNC Symbol;Acc:HGNC:21056]
ENSG00000125753	2476.28775	2223.89028	2779.33998	2878.55226	-0.267797149	0.0195841	0.0661865	VASP	vasodilator stimulated phosphoprotein [Source:HGNC Symbol;Acc:HGNC:12652]
ENSG00000174292	251.959326	227.7051	306.591333	378.980993	-0.515803905	0.0195961	0.0662137	TNK1	tyrosine kinase non receptor 1 [Source:HGNC Symbol;Acc:HGNC:11940]
ENSG00000182218	22.6369707	33.6684583	70.589757	57.4858809	-1.182028498	0.0196217	0.0662868	HHIPL1	HHIP like 1 [Source:HGNC Symbol;Acc:HGNC:19710]
ENSG00000197670	36.4159964	30.1244101	5.26789231	13.8391936	1.800433442	0.0196463	0.0663568	AL157838.1	uncharacterized LOC101927770 [Source:NCBI gene;Acc:101927770]
ENSG00000177570	177.158901	210.870871	133.804465	111.778102	0.660680904	0.0196668	0.0664128	SAMD12	sterile alpha motif domain containing 12 [Source:HGNC Symbol;Acc:HGNC:31750]
ENSG00000010803	645.645774	582.109925	782.808798	792.027693	-0.359691744	0.0197438	0.0666594	SCMH1	Scm polycomb group protein homolog 1 [Source:HGNC Symbol;Acc:HGNC:19003]
ENSG00000103168	975.358173	813.359073	1086.23939	1184.84788	-0.345106696	0.0197817	0.066774	TAF1C	TATA-box binding protein associated factor, RNA polymerase I subunit C [Source:HGNC Symbol;Acc:HGNC:11534]
ENSG00000075856	1866.07376	1738.35567	2170.37163	2179.14071	-0.271294809	0.019875	0.0670755	SART3	squamous cell carcinoma antigen recognized by T cells 3 [Source:HGNC Symbol;Acc:HGNC:16860]
ENSG00000122547	183.064198	156.824135	115.893631	92.6161415	0.703988729	0.019942	0.0672883	EEPD1	endonuclease/exonuclease/phosphatase family domain containing 1 [Source:HGNC Symbol;Acc:HGNC:22223]
ENSG00000144736	611.198209	619.322431	464.628102	477.984454	0.384609135	0.01996	0.0673222	SHQ1	SHQ1, H/ACA ribonucleoprotein assembly factor [Source:HGNC Symbol;Acc:HGNC:25543]
ENSG00000095713	14.7632418	11.5181568	2.10715692	0	3.635152497	0.0199638	0.0673222	CRTAC1	cartilage acidic protein 1 [Source:HGNC Symbol;Acc:HGNC:14882]
ENSG00000156500	65.9424799	62.9068564	27.39304	30.8720472	1.144862262	0.019964	0.0673222	FAM122C	family with sequence similarity 122C [Source:HGNC Symbol;Acc:HGNC:25202]
ENSG00000160087	1049.17438	926.768617	1153.66842	1338.14356	-0.335050367	0.0199871	0.067385	UBE2J2	ubiquitin conjugating enzyme E2 J2 [Source:HGNC Symbol;Acc:HGNC:19268]
ENSG00000117335	3594.35726	4488.53711	3520.00564	3008.42777	0.308343625	0.0199926	0.067385	CD46	CD46 molecule [Source:HGNC Symbol;Acc:HGNC:6953]
ENSG00000004534	1507.81909	1335.22018	1672.02902	1827.8381	-0.300217978	0.0199946	0.067385	RBM6	RNA binding motif protein 6 [Source:HGNC Symbol;Acc:HGNC:9903]
ENSG00000122386	272.627865	230.363136	328.71648	384.303759	-0.504364255	0.0200245	0.0674724	ZNF205	zinc finger protein 205 [Source:HGNC Symbol;Acc:HGNC:12996]
ENSG00000163249	522.618759	525.405153	425.645699	353.431712	0.427743028	0.0200606	0.0675793	CCNYL1	cyclin Y like 1 [Source:HGNC Symbol;Acc:HGNC:26868]
ENSG00000119231	899.573532	871.835869	677.450951	720.702618	0.3412841	0.0200642	0.0675793	SENP5	SUMO specific peptidase 5 [Source:HGNC Symbol;Acc:HGNC:28407]
ENSG00000140022	289.359539	310.104222	210.715692	207.587903	0.519465753	0.0200894	0.0676506	STON2	stonin 2 [Source:HGNC Symbol;Acc:HGNC:30652]
ENSG00000237976	0.98421612	1.77202412	11.5893631	14.9037469	-3.252999573	0.0201021	0.0676799	AL391069.3	novel transcript
ENSG00000196470	94.4847473	103.663411	153.822455	168.199429	-0.699791663	0.0201175	0.0677182	SIAH1	siah E3 ubiquitin protein ligase 1 [Source:HGNC Symbol;Acc:HGNC:10857]
ENSG00000183527	796.23084	849.685567	1025.13184	1036.87496	-0.324857439	0.0201218	0.0677192	PSMG1	proteasome assembly chaperone 1 [Source:HGNC Symbol;Acc:HGNC:3043]
ENSG00000042088	728.319927	704.379589	854.452133	967.678995	-0.346993326	0.0201368	0.0677563	TDP1	tyrosyl-DNA phosphodiesterase 1 [Source:HGNC Symbol;Acc:HGNC:18884]
ENSG00000138101	341.522993	334.912559	231.787262	249.105484	0.492191398	0.0201434	0.067765	DTNB	dystrobrevin beta [Source:HGNC Symbol;Acc:HGNC:3058]
ENSG00000263155	86.6110184	100.119363	28.4466185	61.7440943	1.051121262	0.0201505	0.0677752	MYZAP	myocardial zonula adherens protein [Source:HGNC Symbol;Acc:HGNC:43444]
ENSG00000163273	9.84216118	4.43006031	0	0	5.192453648	0.020153	1	NPPC	natriuretic peptide C [Source:HGNC Symbol;Acc:HGNC:7941]
ENSG00000177875	67.9109122	46.9586393	20.0179908	27.6783871	1.26663344	0.0201709	0.0678178	CCDC184	coiled-coil domain containing 184 [Source:HGNC Symbol;Acc:HGNC:33749]
ENSG00000172830	730.28836	697.291493	869.202231	938.936055	-0.341120854	0.020174	0.0678178	SSH3	slingshot protein phosphatase 3 [Source:HGNC Symbol;Acc:HGNC:30581]
ENSG00000031823	1486.16634	1324.58803	1663.60039	1788.44963	-0.296843139	0.0201752	0.0678178	RANBP3	RAN binding protein 3 [Source:HGNC Symbol;Acc:HGNC:9850]
ENSG00000130720	1177.12248	1052.58233	834.434142	933.613288	0.334382132	0.0202032	0.0678984	FIBCD1	fibrinogen C domain containing 1 [Source:HGNC Symbol;Acc:HGNC:25922]
ENSG00000274487	77.7530733	78.8550735	107.465003	168.199429	-0.81542883	0.0202184	0.0679359	AC244154.1	aminopeptidase puromycin sensitive pseudogene [Source:NCBI gene;Acc:440434]
ENSG00000009694	13.7790257	12.4041689	0	2.1291067	3.624480706	0.0202313	0.0679659	TENM1	teneurin transmembrane protein 1 [Source:HGNC Symbol;Acc:HGNC:8117]
ENSG00000197279	26.5738352	38.0985187	72.6969139	67.0668611	-1.108470855	0.0202384	0.0679764	ZNF165	zinc finger protein 165 [Source:HGNC Symbol;Acc:HGNC:12953]
ENSG00000225439	23.6211868	23.0363136	36.8752462	79.8415013	-1.322714575	0.0202577	0.0680276	BOLA3-AS1	BOLA3 divergent transcript [Source:HGNC Symbol;Acc:HGNC:42922]
ENSG00000230513	98.4216118	124.041689	67.4290216	54.2922209	0.870950969	0.0203009	0.068159	THAP7-AS1	THAP7 antisense RNA 1 [Source:HGNC Symbol;Acc:HGNC:41013]
ENSG00000111843	1709.5834	2094.53251	2501.19527	2219.59374	-0.311054886	0.0203189	0.0682058	TMEM14C	transmembrane protein 14C [Source:HGNC Symbol;Acc:HGNC:20952]
ENSG00000171552	1170.23296	1081.82073	1382.29494	1390.30668	-0.300302756	0.0203438	0.068276	BCL2L1	BCL2 like 1 [Source:HGNC Symbol;Acc:HGNC:992]
ENSG00000101751	120.074366	149.736038	89.5541693	64.9377544	0.805205661	0.0203602	0.0683173	POLI	DNA polymerase iota [Source:HGNC Symbol;Acc:HGNC:9182]
ENSG00000164620	142.711337	118.725616	89.5541693	56.4213276	0.839527269	0.0203682	0.0683308	RELL2	RELT like 2 [Source:HGNC Symbol;Acc:HGNC:26902]
ENSG00000103591	1550.14039	1655.95654	1244.27616	1366.8865	0.29635001	0.0203851	0.068374	AAGAB	alpha and gamma adaptin binding protein [Source:HGNC Symbol;Acc:HGNC:25662]
ENSG00000204923	83.65837	85.94317	52.6789231	29.8074938	1.039534059	0.0204134	0.0684552	FBXO48	F-box protein 48 [Source:HGNC Symbol;Acc:HGNC:33857]
ENSG00000159216	1275.54409	1310.41184	1089.40013	1000.68015	0.307190855	0.020436	0.068516	RUNX1	runt related transcription factor 1 [Source:HGNC Symbol;Acc:HGNC:10471]
ENSG00000213903	441.913037	354.404825	295.001969	273.590211	0.484939405	0.0204419	0.068516	LTB4R	leukotriene B4 receptor [Source:HGNC Symbol;Acc:HGNC:6713]
ENSG00000120314	729.304144	679.571251	897.64885	884.643834	-0.339538859	0.0204437	0.068516	WDR55	WD repeat domain 55 [Source:HGNC Symbol;Acc:HGNC:25971]
ENSG00000011478	635.803612	520.975092	413.002757	450.306067	0.421425864	0.0204524	0.0685314	QPCTL	glutaminyl-peptide cyclotransferase like [Source:HGNC Symbol;Acc:HGNC:25952]
ENSG00000240445	43.3055092	45.1866152	81.1255416	91.5515881	-0.96399743	0.0204607	0.0685413	FOXO3B	forkhead box O3B pseudogene [Source:HGNC Symbol;Acc:HGNC:3822]
ENSG00000136783	170.269388	209.984859	287.62692	270.396551	-0.551997294	0.0204634	0.0685413	NIPSNAP3A	nipsnap homolog 3A [Source:HGNC Symbol;Acc:HGNC:23619]
ENSG00000133636	10.8263773	3.54404825	0	0	5.201549999	0.0205041	1	NTS	neurotensin [Source:HGNC Symbol;Acc:HGNC:8038]
ENSG00000103249	1593.4459	1402.55709	1901.70912	1772.48133	-0.294793276	0.0205142	0.0686979	CLCN7	chloride voltage-gated channel 7 [Source:HGNC Symbol;Acc:HGNC:2025]

ENSG00000167930	1757.80999	1437.11156	1208.4545	1337.07901	0.327374515	0.020527	0.0687272	FAM234A	family with sequence similarity 234 member A [Source:HGNC Symbol;Acc:HGNC:14163]
ENSG00000122729	1905.4424	1842.90509	1568.77833	1528.69861	0.275057088	0.0205488	0.068773	ACO1	aconitase 1 [Source:HGNC Symbol;Acc:HGNC:117]
ENSG00000172667	255.896191	348.20274	234.947997	161.812109	0.607423588	0.0205489	0.068773	ZMAT3	zinc finger matrin-type 3 [Source:HGNC Symbol;Acc:HGNC:29983]
ENSG00000149923	2404.43998	1914.67207	2533.8562	2824.26004	-0.311400078	0.0205849	0.06888	PPP4C	protein phosphatase 4 catalytic subunit [Source:HGNC Symbol;Acc:HGNC:9319]
ENSG00000165731	2.95264835	11.5181568	0	0	5.218796372	0.0206102	1	RET	ret proto-oncogene [Source:HGNC Symbol;Acc:HGNC:9967]
ENSG00000163864	6.88951283	7.08809649	0	0	5.165036401	0.0206478	1	NMNAT3	nicotinamide nucleotide adenylyltransferase 3 [Source:HGNC Symbol;Acc:HGNC:20989]
ENSG00000164287	6.88951283	7.08809649	0	0	5.165036401	0.0206478	1	CDC20B	cell division cycle 20B [Source:HGNC Symbol;Acc:HGNC:24222]
ENSG00000175130	2274.52345	2321.3516	2645.53552	2857.26119	-0.259755405	0.0206555	0.0691025	MARCKSL1	MARCKS like 1 [Source:HGNC Symbol;Acc:HGNC:7142]
ENSG00000151883	142.711337	189.606581	94.8220616	107.519888	0.717243004	0.0206605	0.0691056	PARP8	poly(ADP-ribose) polymerase family member 8 [Source:HGNC Symbol;Acc:HGNC:26124]
ENSG00000180104	2013.70618	1795.94645	2263.08654	2349.46924	-0.276207477	0.0206684	0.0691182	EXOC3	exocyst complex component 3 [Source:HGNC Symbol;Acc:HGNC:30378]
ENSG00000177156	3722.30536	3528.98604	4053.11634	4623.3552	-0.258937023	0.0207559	0.0693972	TALDO1	transaldolase 1 [Source:HGNC Symbol;Acc:HGNC:11559]
ENSG00000145191	1055.07968	1010.05375	1228.47249	1326.43347	-0.307182768	0.0207607	0.0693993	EIF2B5	eukaryotic translation initiation factor 2B subunit epsilon [Source:HGNC Symbol;Acc:HGNC:3261]
ENSG00000164465	1706.63075	1732.15358	2071.33526	2066.29805	-0.266860305	0.0207791	0.0694473	DCBLD1	discoidin, CUB and LCCL domain containing 1 [Source:HGNC Symbol;Acc:HGNC:21479]
ENSG00000157470	292.312187	323.394403	208.608536	222.49165	0.514774636	0.0207971	0.0694938	FAM81A	family with sequence similarity 81 member A [Source:HGNC Symbol;Acc:HGNC:28379]
ENSG00000099899	685.998634	500.596815	768.058699	809.060546	-0.411662321	0.0208319	0.0695961	TRMT2A	tRNA methyltransferase 2 homolog A [Source:HGNC Symbol;Acc:HGNC:24974]
ENSG00000173917	72.8319927	68.2229288	123.26868	121.359082	-0.794895723	0.0208506	0.069645	HOXB2	homeobox B2 [Source:HGNC Symbol;Acc:HGNC:5113]
ENSG00000259802	129.916528	89.4872182	65.3218647	52.1631142	0.899164532	0.0208726	0.0697045	AC012640.2	novel transcript, antisense to MARCH6
ENSG00000160957	1649.54621	1430.90948	1825.85148	1953.4554	-0.295374227	0.0209152	0.0698331	RECQL4	RecQ like helicase 4 [Source:HGNC Symbol;Acc:HGNC:9949]
ENSG00000171960	393.686447	435.031922	527.84281	571.665149	-0.407301404	0.0209253	0.0698431	PPIH	peptidylprolyl isomerase H [Source:HGNC Symbol;Acc:HGNC:14651]
ENSG00000006715	945.83169	1138.5255	886.059487	726.025385	0.371112221	0.0209274	0.0698431	VPS41	VPS41, HOPS complex subunit [Source:HGNC Symbol;Acc:HGNC:12713]
ENSG00000177426	1163.34345	988.789461	1208.4545	1553.18334	-0.360150668	0.0209309	0.0698431	TGIF1	TGFB induced factor homeobox 1 [Source:HGNC Symbol;Acc:HGNC:11776]
ENSG00000139531	547.224162	453.638176	658.486539	655.764864	-0.393938472	0.0209348	0.0698431	SUOX	sulfite oxidase [Source:HGNC Symbol;Acc:HGNC:11460]
ENSG00000001084	1040.31644	1210.29248	944.006302	832.48072	0.341695217	0.0209685	0.0699419	GCLC	glutamate-cysteine ligase catalytic subunit [Source:HGNC Symbol;Acc:HGNC:4311]
ENSG00000146278	1063.93762	1238.64486	1497.13499	1372.20927	-0.316982066	0.0209868	0.0699891	PNRC1	proline rich nuclear receptor coactivator 1 [Source:HGNC Symbol;Acc:HGNC:17278]
ENSG00000168298	35.4317803	29.238398	12.6429415	6.3873201	1.763076009	0.02103	0.0701193	HIST1H1E	histone cluster 1 H1 family member e [Source:HGNC Symbol;Acc:HGNC:4718]
ENSG00000184967	718.477766	648.560829	857.612868	878.256514	-0.345084302	0.0210543	0.0701866	NOC4L	nucleolar complex associated 4 homolog [Source:HGNC Symbol;Acc:HGNC:28461]
ENSG00000170855	846.425862	824.87723	1029.34616	1054.97237	-0.318710712	0.0210872	0.0702814	TRIAP1	TP53 regulated inhibitor of apoptosis 1 [Source:HGNC Symbol;Acc:HGNC:26937]
ENSG00000162408	390.733799	389.845307	494.128299	543.986762	-0.411314591	0.0210911	0.0702814	NOL9	nucleolar protein 9 [Source:HGNC Symbol;Acc:HGNC:26265]
ENSG00000164951	323.807103	305.674161	240.215889	199.071476	0.518586944	0.0211135	0.0703424	PDP1	pyruvate dehyrogenase phosphatase catalytic subunit 1 [Source:HGNC Symbol;Acc:HGNC:9279]
ENSG00000196415	56.1003187	54.0467358	25.2858831	21.291067	1.241359937	0.0211194	0.0703481	PRTN3	proteinase 3 [Source:HGNC Symbol;Acc:HGNC:9495]
ENSG00000117308	1555.06147	1373.3187	1741.5652	1843.8064	-0.292390836	0.0211736	0.0705148	GALE	UDP-galactose-4-epimerase [Source:HGNC Symbol;Acc:HGNC:4116]
ENSG00000006453	1042.28487	996.76357	771.219434	851.64268	0.329242456	0.0211861	0.0705426	BAIAP2L1	BAI1 associated protein 2 like 1 [Source:HGNC Symbol;Acc:HGNC:21649]
ENSG00000053900	196.843224	293.269992	385.609717	333.205199	-0.550527247	0.021192	0.0705481	ANAPC4	anaphase promoting complex subunit 4 [Source:HGNC Symbol;Acc:HGNC:19990]
ENSG00000197355	588.561239	543.125394	732.237031	721.767171	-0.361984371	0.0211966	0.0705497	UAP1L1	UDP-N-acetylglucosamine pyrophosphorylase 1 like 1 [Source:HGNC Symbol;Acc:HGNC:28082]
ENSG00000023318	1254.87555	1436.22555	1118.90033	1044.32684	0.315384021	0.021214	0.0705828	ERP44	endoplasmic reticulum protein 44 [Source:HGNC Symbol;Acc:HGNC:18311]
ENSG00000119650	713.556686	689.317384	483.592514	588.698003	0.387662091	0.0212149	0.0705828	IFT43	intraflagellar transport 43 [Source:HGNC Symbol;Acc:HGNC:29669]
ENSG00000175193	150.585066	146.19199	204.394222	244.847271	-0.598224353	0.0212325	0.0706274	PARL	presenilin associated rhomboid like [Source:HGNC Symbol;Acc:HGNC:18253]
ENSG00000104356	366.128396	337.570596	461.467366	483.307221	-0.425480293	0.021257	0.0706949	POP1	POP1 homolog, ribonuclease P/MRP subunit [Source:HGNC Symbol;Acc:HGNC:30129]
ENSG00000185352	58.068751	56.704772	24.2323046	25.5492804	1.205039099	0.0213481	0.0709837	HS6ST3	heparan sulfate 6-O-sulfotransferase 3 [Source:HGNC Symbol;Acc:HGNC:19134]
ENSG00000112079	1089.52724	1295.34963	1500.29573	1464.82541	-0.313611311	0.0213523	0.0709841	STK38	serine/threonine kinase 38 [Source:HGNC Symbol;Acc:HGNC:17847]
ENSG00000169490	838.552133	861.203724	586.843204	728.154492	0.37045995	0.0214099	0.0711614	TM2D2	TM2 domain containing 2 [Source:HGNC Symbol;Acc:HGNC:24127]
ENSG00000078142	311.012293	443.006031	254.965988	269.331998	0.525596114	0.02142	0.0711809	PIK3C3	phosphatidylinositol 3-kinase catalytic subunit type 3 [Source:HGNC Symbol;Acc:HGNC:8974]
ENSG00000133997	380.891638	402.249476	497.289034	543.986762	-0.410644864	0.0214661	0.0713201	MED6	mediator complex subunit 6 [Source:HGNC Symbol;Acc:HGNC:19970]
ENSG00000205352	1060.00076	1122.57728	1282.20499	1415.85596	-0.305637431	0.0215649	0.0716345	PRR13	proline rich 13 [Source:HGNC Symbol;Acc:HGNC:24528]
ENSG00000121236	37.4002125	46.9586393	13.69652	17.0328536	1.458293905	0.0216107	0.0717725	TRIM6	tripartite motif containing 6 [Source:HGNC Symbol;Acc:HGNC:16277]
ENSG00000114423	363.175748	325.166427	263.394616	223.556204	0.49871539	0.0216157	0.0717749	CBLB	Cbl proto-oncogene B [Source:HGNC Symbol;Acc:HGNC:1542]
ENSG00000122512	384.828502	402.249476	526.789231	515.243822	-0.40456999	0.0216382	0.0718355	PMS2	PMS1 homolog 2, mismatch repair system component [Source:HGNC Symbol;Acc:HGNC:9122]
ENSG00000081026	516.713462	575.90784	424.59212	400.27206	0.406009367	0.0216592	0.0718911	MAGI3	membrane associated guanylate kinase, WW and PDZ domain containing 3 [Source:HGNC Symbol;Acc:HGNC:29647]
ENSG00000181896	384.828502	352.632801	231.787262	291.687618	0.494207028	0.0216674	0.0719042	ZNF101	zinc finger protein 101 [Source:HGNC Symbol;Acc:HGNC:12881]

ENSG00000233270	98.4216118	82.3991217	125.375837	179.909516	-0.756703977	0.0217339	0.0721106	SNRPEP4	small nuclear ribonucleoprotein polypeptide E pseudogene 4 [Source:HGNC Symbol;Acc:HGNC:43446]
ENSG00000134779	2082.60131	1926.19022	2349.47997	2469.76377	-0.265840122	0.0217589	0.0721794	TPGS2	tubulin polyglutamylase complex subunit 2 [Source:HGNC Symbol;Acc:HGNC:24561]
ENSG00000162817	179.127334	174.544376	110.625739	113.907208	0.655368476	0.0217747	0.0722176	C1orf115	chromosome 1 open reading frame 115 [Source:HGNC Symbol;Acc:HGNC:25873]
ENSG00000274615	8.85794506	78.8550735	8.4286277	13.8391936	1.982250153	0.0218296	0.0723743	AC233968.1	aminopeptidase puromycin sensitive pseudogene [Source:NCBI gene;Acc:440434]
ENSG00000139725	529.508272	416.425669	340.305843	350.238052	0.453038735	0.0218305	0.0723743	RHOF	ras homolog family member F, filopodia associated [Source:HGNC Symbol;Acc:HGNC:15703]
ENSG00000215301	8371.7423	9723.98238	12065.5806	9891.82973	-0.278986935	0.0218388	0.0723876	DDX3X	DEAD-box helicase 3 X-linked [Source:HGNC Symbol;Acc:HGNC:2745]
ENSG00000145147	188.969495	269.347667	170.679711	117.100869	0.672502358	0.0218548	0.0724266	SLIT2	slit guidance ligand 2 [Source:HGNC Symbol;Acc:HGNC:11086]
ENSG00000197321	750.956898	675.141191	558.396585	547.180422	0.36682182	0.0218965	0.0725504	SVIL	supervillin [Source:HGNC Symbol;Acc:HGNC:11480]
ENSG00000102580	634.819396	678.685239	542.592908	460.951601	0.38849329	0.0219339	0.0726469	DNAJC3	DnaJ heat shock protein family (Hsp40) member C3 [Source:HGNC Symbol;Acc:HGNC:9439]
ENSG00000118579	474.392169	528.063189	626.879185	679.185037	-0.381087147	0.0219342	0.0726469	MED28	mediator complex subunit 28 [Source:HGNC Symbol;Acc:HGNC:24628]
ENSG00000152409	614.150858	678.685239	559.450163	388.561973	0.44770576	0.0219518	0.072691	JMY	junction mediating and regulatory protein, p53 cofactor [Source:HGNC Symbol;Acc:HGNC:28916]
ENSG00000169594	267.706784	274.663739	187.536966	187.36139	0.532912865	0.0219671	0.0727274	BNC1	basonuclin 1 [Source:HGNC Symbol;Acc:HGNC:1081]
ENSG00000143319	1668.24632	1614.31398	1275.88352	1410.53319	0.289082368	0.0219982	0.0728158	ISG20L2	interferon stimulated exonuclease gene 20 like 2 [Source:HGNC Symbol;Acc:HGNC:25745]
ENSG00000230409	235.227652	188.720569	442.502954	227.814417	-0.662047867	0.0220035	0.0728194	TCEA1P2	transcription elongation factor A1 pseudogene 2 [Source:HGNC Symbol;Acc:HGNC:29891]
ENSG00000065328	757.846411	722.09983	919.773998	938.936055	-0.328979931	0.0220246	0.0728747	MCM10	minichromosome maintenance 10 replication initiation factor [Source:HGNC Symbol;Acc:HGNC:18043]
ENSG00000164022	507.855517	637.042672	772.273013	726.025385	-0.387122277	0.0220479	0.0729375	AIMP1	aminoacyl tRNA synthetase complex interacting multifunctional protein 1 [Source:HGNC Symbol;Acc:HGNC:10648]
ENSG00000163682	3885.68523	4594.85855	4874.90755	5338.73505	-0.268046363	0.0220693	0.0729943	RPL9	ribosomal protein L9 [Source:HGNC Symbol;Acc:HGNC:10369]
ENSG00000137821	282.470026	395.16138	247.590939	218.233437	0.541970685	0.022084	0.0730195	LRRC49	leucine rich repeat containing 49 [Source:HGNC Symbol;Acc:HGNC:25965]
ENSG00000119004	53.1476704	53.1607237	89.5541693	107.519888	-0.890374808	0.0220856	0.0730195	CYP20A1	cytochrome P450 family 20 subfamily A member 1 [Source:HGNC Symbol;Acc:HGNC:20576]
ENSG00000129925	1982.21126	1708.23126	1558.24255	1444.5989	0.297084684	0.0221282	0.0731459	TMEM8A	transmembrane protein 8A [Source:HGNC Symbol;Acc:HGNC:17205]
ENSG00000145824	10.8263773	11.5181568	1.05357846	0	4.400189657	0.0221451	0.0731875	CXCL14	C-X-C motif chemokine ligand 14 [Source:HGNC Symbol;Acc:HGNC:10640]
ENSG00000140057	39.3686447	46.0726272	15.8036769	15.9683003	1.427977823	0.0221606	0.0732244	AK7	adenylate kinase 7 [Source:HGNC Symbol;Acc:HGNC:20091]
ENSG00000197415	53.1476704	56.704772	26.3394616	20.2265137	1.238259711	0.0221797	0.0732733	VEPH1	ventricular zone expressed PH domain containing 1 [Source:HGNC Symbol;Acc:HGNC:25735]
ENSG00000233121	4.92108059	8.86012062	0	0	5.146475722	0.0221881	1	VN1R20P	vomeronasal 1 receptor 20 pseudogene [Source:HGNC Symbol;Acc:HGNC:37335]
ENSG00000149927	71.8477766	51.3886996	23.1787262	30.8720472	1.187711298	0.02226	0.0735153	DOC2A	double C2 domain alpha [Source:HGNC Symbol;Acc:HGNC:2985]
ENSG00000082269	225.385491	261.373558	163.304662	165.005769	0.568910617	0.0222647	0.0735153	FAM135A	family with sequence similarity 135 member A [Source:HGNC Symbol;Acc:HGNC:21084]
ENSG00000128881	458.644711	457.182224	365.591726	310.849578	0.436990933	0.022266	0.0735153	TTBK2	tau tubulin kinase 2 [Source:HGNC Symbol;Acc:HGNC:19141]
ENSG00000142168	4887.61724	4611.69278	5300.55324	5973.20885	-0.247136608	0.0222705	0.0735157	SOD1	superoxide dismutase 1 [Source:HGNC Symbol;Acc:HGNC:11179]
ENSG00000110074	1116.10108	961.323087	1269.56205	1314.72339	-0.315510795	0.0222794	0.0735309	FOXRED1	FAD dependent oxidoreductase domain containing 1 [Source:HGNC Symbol;Acc:HGNC:26927]
ENSG00000028116	249.006678	265.803619	358.216677	355.560819	-0.471005054	0.0222943	0.0735655	VRK2	vaccinia related kinase 2 [Source:HGNC Symbol;Acc:HGNC:12719]
ENSG00000270641	1.96843224	0.88601206	8.4286277	18.097407	-3.231068869	0.0222994	0.0735681	TSIX	TSIX transcript, XIST antisense RNA [Source:HGNC Symbol;Acc:HGNC:12377]
ENSG00000171867	3106.18607	3239.2601	3897.18673	3625.86871	-0.245526576	0.0223134	0.0735999	PRNP	prion protein [Source:HGNC Symbol;Acc:HGNC:9449]
ENSG00000267922	163.379876	134.673833	81.1255416	97.9389082	0.734273501	0.022348	0.0736994	AC007785.1	novel transcript
ENSG00000139793	536.397784	624.638504	854.452133	675.991377	-0.397989643	0.0223559	0.0737111	MBNL2	muscleblind like splicing regulator 2 [Source:HGNC Symbol;Acc:HGNC:16746]
ENSG00000183729	76.7688572	83.2851338	42.1431385	38.3239206	0.99242447	0.0223614	0.073715	NPBWR1	neuropeptides B and W receptor 1 [Source:HGNC Symbol;Acc:HGNC:4522]
ENSG00000163634	442.897253	525.405153	620.557714	650.442097	-0.391539375	0.0223701	0.0737294	THOC7	THO complex 7 [Source:HGNC Symbol;Acc:HGNC:29874]
ENSG00000229167	30.5106997	24.8083377	9.48220616	5.32276675	1.899966519	0.0224323	0.0739197	AC114488.1	novel transcript
ENSG00000265415	115.153286	146.19199	200.179908	204.394243	-0.628717538	0.0224429	0.0739405	AC099850.3	novel transcript, antisense to PRR11
ENSG00000183495	2951.66414	2963.71035	3685.41746	3349.08484	-0.249989162	0.0224599	0.073982	EP400	E1A binding protein p400 [Source:HGNC Symbol;Acc:HGNC:11958]
ENSG00000150938	2546.1671	2368.31024	2177.74668	1857.6456	0.284146124	0.0224806	0.0740356	CRIM1	cysteine rich transmembrane BMP regulator 1 [Source:HGNC Symbol;Acc:HGNC:2359]
ENSG00000123595	1134.80118	1213.83652	878.684438	1001.7447	0.321036185	0.0225192	0.0741483	RAB9A	RAB9A, member RAS oncogene family [Source:HGNC Symbol;Acc:HGNC:9792]
ENSG00000152495	180.11155	235.679208	153.822455	107.519888	0.670813123	0.0225533	0.0742463	CAMK4	calcium/calmodulin dependent protein kinase IV [Source:HGNC Symbol;Acc:HGNC:1464]
ENSG00000185761	273.612081	277.321775	195.965594	186.296836	0.527346662	0.0225767	0.0743088	ADAMTSL5	ADAMTS like 5 [Source:HGNC Symbol;Acc:HGNC:27912]
ENSG00000125124	1314.91273	1379.52078	1090.45371	1107.13548	0.294227207	0.0225878	0.0743307	BBS2	Bardet-Biedl syndrome 2 [Source:HGNC Symbol;Acc:HGNC:967]
ENSG00000135636	167.31674	132.015797	95.8756401	84.0997147	0.732646793	0.0226171	0.0744128	DYSF	dysferlin [Source:HGNC Symbol;Acc:HGNC:3097]
ENSG00000133424	212.590682	205.554798	144.340249	130.940062	0.602845538	0.0226451	0.0744905	LARGE1	LARGE xylosyl- and glucuronyltransferase 1 [Source:HGNC Symbol;Acc:HGNC:6511]
ENSG00000263753	176.174685	224.161052	108.518582	144.779256	0.661682667	0.0226729	0.0745674	LINC00667	long intergenic non-protein coding RNA 667 [Source:HGNC Symbol;Acc:HGNC:27906]

ENSG00000185304	33.463348	53.1607237	16.8572554	14.9037469	1.449527899	0.022685	0.0745927	RGPD2	RANBP2-like and GRIP domain containing 2 [Source:HGNC Symbol;Acc:HGNC:32415]
ENSG00000214491	65.9424799	72.6529891	125.375837	114.971762	-0.793477924	0.022725	0.0747095	SEC14L6	SEC14 like lipid binding 6 [Source:HGNC Symbol;Acc:HGNC:40047]
ENSG00000185420	847.410078	738.048047	612.129087	624.892817	0.35750651	0.0227465	0.0747657	SMYD3	SET and MYND domain containing 3 [Source:HGNC Symbol;Acc:HGNC:15513]
ENSG00000135837	1755.84155	2153.00931	1715.22574	1383.91936	0.33517934	0.0227787	0.074857	CEP350	centrosomal protein 350 [Source:HGNC Symbol;Acc:HGNC:24238]
ENSG00000138764	519.66611	581.223913	783.862376	653.635757	-0.384442584	0.0228073	0.074931	CCNG2	cyclin G2 [Source:HGNC Symbol;Acc:HGNC:1593]
ENSG00000269113	78.7372895	54.0467358	27.39304	33.0011539	1.134961566	0.0228101	0.074931	TRABD2B	TraB domain containing 2B [Source:HGNC Symbol;Acc:HGNC:44200]
ENSG00000198807	189.953711	192.264617	122.215102	125.617295	0.625120035	0.0228189	0.0749453	PAX9	paired box 9 [Source:HGNC Symbol;Acc:HGNC:8623]
ENSG00000213551	1070.82714	1040.17816	1272.72278	1318.9816	-0.296076948	0.0228396	0.0749989	DNAJC9	DnaJ heat shock protein family (Hsp40) member C9 [Source:HGNC Symbol;Acc:HGNC:19123]
ENSG00000122965	940.910609	874.493905	1086.23939	1172.07324	-0.315233517	0.0228506	0.0750202	RBM19	RNA binding motif protein 19 [Source:HGNC Symbol;Acc:HGNC:29098]
ENSG00000107021	1174.16983	1022.45792	1353.84832	1362.62829	-0.306951856	0.0228752	0.0750865	TBC1D13	TBC1 domain family member 13 [Source:HGNC Symbol;Acc:HGNC:25571]
ENSG00000172732	562.97162	512.114972	685.879579	698.346998	-0.365118855	0.0228852	0.0751047	MUS81	MUS81 structure-specific endonuclease subunit [Source:HGNC Symbol;Acc:HGNC:29814]
ENSG00000156110	1408.41327	1583.30355	1764.74392	1886.38854	-0.286990718	0.0229216	0.0752097	ADK	adenosine kinase [Source:HGNC Symbol;Acc:HGNC:257]
ENSG00000131759	933.03688	838.16741	1054.63204	1162.49226	-0.32435985	0.0229298	0.0752219	RARA	retinoic acid receptor alpha [Source:HGNC Symbol;Acc:HGNC:9864]
ENSG00000233328	61.0213993	48.7306634	91.6613262	110.713548	-0.884582639	0.0229447	0.0752561	PFN1P1	profilin 1 pseudogene 1 [Source:HGNC Symbol;Acc:HGNC:42989]
ENSG00000168884	932.052664	749.566204	1016.70322	1129.4911	-0.352682699	0.0230651	0.0756341	TNIP2	TNFAIP3 interacting protein 2 [Source:HGNC Symbol;Acc:HGNC:19118]
ENSG00000187866	396.639096	441.234007	326.609323	286.364851	0.451309053	0.0230689	0.0756341	FAM122A	family with sequence similarity 122A [Source:HGNC Symbol;Acc:HGNC:23490]
ENSG00000184986	173.222037	185.176521	120.107945	108.584442	0.648426332	0.0231375	0.0758445	TMEM121	transmembrane protein 121 [Source:HGNC Symbol;Acc:HGNC:20511]
ENSG00000163946	1342.47079	1572.67141	2175.63952	1570.21619	-0.361471262	0.0231422	0.075845	FAM208A	family with sequence similarity 208 member A [Source:HGNC Symbol;Acc:HGNC:30314]
ENSG00000249679	13.7790257	10.6321447	1.05357846	1.06455335	3.525614421	0.0231529	0.0758654	AC106897.1	uncharacterized LOC105377590 [Source:NCBI gene;Acc:105377590]
ENSG00000139714	33.463348	48.7306634	18.9644123	9.58098015	1.526692749	0.023171	0.0759101	MORN3	MORN repeat containing 3 [Source:HGNC Symbol;Acc:HGNC:29807]
ENSG00000272142	62.9898316	55.8187599	26.3394616	26.6138338	1.165227489	0.0231786	0.07592	LYRM4-AS1	LYRM4 antisense RNA 1 [Source:NCBI gene;Acc:100129461]
ENSG00000138867	1466.48202	1337.87821	1720.49363	1688.38161	-0.281941954	0.0231835	0.0759203	GUCD1	guanylyl cyclase domain containing 1 [Source:HGNC Symbol;Acc:HGNC:14237]
ENSG00000165169	482.265898	621.980467	445.66369	364.077246	0.448314414	0.023194	0.0759203	DYNLT3	dynein light chain Tctex-type 3 [Source:HGNC Symbol;Acc:HGNC:11694]
ENSG00000077684	570.845349	566.161707	791.237425	675.991377	-0.367960805	0.0231944	0.0759203	JADE1	jade family PHD finger 1 [Source:HGNC Symbol;Acc:HGNC:30027]
ENSG00000196422	3642.58385	3252.55028	2757.21484	2997.78223	0.26059633	0.0231966	0.0759203	PPP1R26	protein phosphatase 1 regulatory subunit 26 [Source:HGNC Symbol;Acc:HGNC:29089]
ENSG00000037042	406.481257	353.518813	296.055548	249.105484	0.478573856	0.0232074	0.0759404	TUBG2	tubulin gamma 2 [Source:HGNC Symbol;Acc:HGNC:12419]
ENSG00000105202	4586.44711	3948.95576	4864.37176	5368.54255	-0.261867801	0.023213	0.0759404	FBL	fibrillarin [Source:HGNC Symbol;Acc:HGNC:3599]
ENSG00000212802	1024.56898	919.68052	1158.93631	1258.30206	-0.314541459	0.0232162	0.0759404	RPL15P3	ribosomal protein L15 pseudogene 3 [Source:HGNC Symbol;Acc:HGNC:21538]
ENSG00000165171	135.821824	132.015797	86.3934339	72.3896278	0.753993877	0.0232284	0.0759655	METTL27	methyltransferase like 27 [Source:HGNC Symbol;Acc:HGNC:19068]
ENSG00000135541	376.954773	430.601862	316.073539	269.331998	0.464620749	0.0232365	0.0759772	AHI1	Abelson helper integration site 1 [Source:HGNC Symbol;Acc:HGNC:21575]
ENSG00000186153	180.11155	152.394075	222.305056	273.590211	-0.577665946	0.0232729	0.0760815	WWOX	WW domain containing oxidoreductase [Source:HGNC Symbol;Acc:HGNC:12799]
ENSG00000196371	94.4847473	72.6529891	44.2502954	40.4530273	0.979116762	0.0232834	0.0760984	FUT4	fucosyltransferase 4 [Source:HGNC Symbol;Acc:HGNC:4015]
ENSG00000184481	804.104569	733.617987	562.610899	634.473797	0.36097829	0.0232871	0.0760984	FOXO4	forkhead box O4 [Source:HGNC Symbol;Acc:HGNC:7139]
ENSG00000149476	1091.49568	949.80493	1196.86513	1355.17641	-0.322616965	0.0233499	0.0762891	TKFC	triokinase and FMN cyclase [Source:HGNC Symbol;Acc:HGNC:24552]
ENSG00000174899	21.6527546	16.8342292	4.21431385	3.19366005	2.375630857	0.0233598	0.0763067	PQLC2L	PQ loop repeat containing 2 like [Source:HGNC Symbol;Acc:HGNC:25146]
ENSG00000228589	121.058583	55.8187599	181.215496	137.327382	-0.853081864	0.0233706	0.0763271	SPCS2P4	signal peptidase complex subunit 2 pseudogene 4 [Source:HGNC Symbol;Acc:HGNC:45237]
ENSG00000198920	417.307634	474.902465	555.23585	620.634603	-0.397536613	0.0234313	0.0765104	KIAA0753	KIAA0753 [Source:HGNC Symbol;Acc:HGNC:29110]
ENSG00000247077	1890.67916	1570.89939	2050.26369	2197.23811	-0.295630002	0.0234734	0.0766333	PGAM5	PGAM family member 5, mitochondrial serine/threonine protein phosphatase [Source:HGNC Symbol;Acc:HGNC:28763]
ENSG00000166436	314.949158	273.777727	211.769271	199.071476	0.518276439	0.0235001	0.0767054	TRIM66	tripartite motif containing 66 [Source:HGNC Symbol;Acc:HGNC:29005]
ENSG00000269516	12.7948095	12.4041689	0	2.1291067	3.569426918	0.02355	0.0768534	CYP4F23P	cytochrome P450 family 4 subfamily F member 23, pseudogene [Source:HGNC Symbol;Acc:HGNC:39944]
ENSG00000149357	1348.37608	1091.56686	1503.45647	1536.15048	-0.317675454	0.0235948	0.076985	LAMTOR1	late endosomal/lysosomal adaptor, MAPK and MTOR activator 1 [Source:HGNC Symbol;Acc:HGNC:26068]
ENSG00000224621	0	0	6.32147077	6.3873201	-5.209186569	0.0236003	1	AL451042.1	novel transcript
ENSG00000225177	54.1318865	49.6166755	23.1787262	20.2265137	1.256549127	0.0236014	0.0769917	FLJ46906	uncharacterized LOC441172 [Source:NCBI gene;Acc:441172]
ENSG00000198746	280.501594	261.373558	354.002363	392.820186	-0.463201192	0.0236639	0.0771807	GPATCH3	G-patch domain containing 3 [Source:HGNC Symbol;Acc:HGNC:25720]
ENSG00000143473	11.8105934	18.6062533	2.10715692	2.1291067	2.845937514	0.0237163	0.0773365	KCNH1	potassium voltage-gated channel subfamily H member 1 [Source:HGNC Symbol;Acc:HGNC:6250]
ENSG00000166012	510.808165	631.7266	910.291791	638.73201	-0.438500757	0.0237332	0.0773767	TAF1D	TATA-box binding protein associated factor, RNA polymerase I subunit D [Source:HGNC Symbol;Acc:HGNC:28759]
ENSG00000184602	371.049477	386.301259	488.860407	514.179268	-0.405077695	0.0237447	0.0773992	SNN	stannin [Source:HGNC Symbol;Acc:HGNC:11149]
ENSG00000198783	223.417059	225.933076	276.037557	370.464566	-0.524607536	0.0237884	0.0775269	ZNF830	zinc finger protein 830 [Source:HGNC Symbol;Acc:HGNC:28291]
ENSG00000185669	76.7688572	51.3886996	24.2323046	33.0011539	1.16139746	0.0238077	0.0775746	SNAI3	snail family transcriptional repressor 3 [Source:HGNC Symbol;Acc:HGNC:18411]

ENSG00000120256	148.616634	154.166099	88.5005908	97.9389082	0.699860551	0.023817	0.0775902	LRP11	LDL receptor related protein 11 [Source:HGNC Symbol;Acc:HGNC:16936]
ENSG00000011009	1653.48308	1306.86779	1795.2977	1887.45309	-0.315600792	0.0238216	0.0775902	LYPLA2	lysophospholipase II [Source:HGNC Symbol;Acc:HGNC:6738]
ENSG00000137409	4218.35028	3600.75302	4579.90558	4764.9408	-0.257391873	0.0238418	0.0776411	MTCH1	mitochondrial carrier 1 [Source:HGNC Symbol;Acc:HGNC:17586]
ENSG00000275131	137.790257	118.725616	80.0719631	70.2605211	0.769991518	0.0239121	0.0778517	AC241952.1	phosphodiesterase 4D interacting protein-like [Source:NCBI gene;Acc:100996724]
ENSG00000169627	29.5264835	84.1711459	23.1787262	20.2265137	1.392718038	0.0239157	0.0778517	BOLA2B	bolA family member 2B [Source:HGNC Symbol;Acc:HGNC:32479]
ENSG00000197261	172.237821	158.596159	87.4470124	117.100869	0.693492785	0.0239557	0.0779669	C6orf141	chromosome 6 open reading frame 141 [Source:HGNC Symbol;Acc:HGNC:21351]
ENSG00000102554	2328.65534	2333.75577	2823.59028	2712.48194	-0.247790807	0.0239667	0.0779874	KLF5	Kruppel like factor 5 [Source:HGNC Symbol;Acc:HGNC:6349]
ENSG00000176771	98.4216118	85.0571579	50.5717662	46.8403474	0.912591968	0.0239953	0.0780657	NCKAP5	NCK associated protein 5 [Source:HGNC Symbol;Acc:HGNC:29847]
ENSG00000182459	57.0845349	40.7565548	21.0715692	18.097407	1.318908715	0.0240483	0.0782231	TEX19	testis expressed 19 [Source:HGNC Symbol;Acc:HGNC:33802]
ENSG00000250571	417.307634	352.632801	269.716086	287.429405	0.465954345	0.0240751	0.078295	GLI4	GLI family zinc finger 4 [Source:HGNC Symbol;Acc:HGNC:4320]
ENSG00000117153	656.472151	811.587049	972.452921	906.999454	-0.355579225	0.0241156	0.0784118	KLHL12	kelch like family member 12 [Source:HGNC Symbol;Acc:HGNC:19360]
ENSG00000236287	564.940052	667.167083	908.184635	710.057085	-0.392732712	0.0241271	0.0784341	ZBED5	zinc finger BED-type containing 5 [Source:HGNC Symbol;Acc:HGNC:30803]
ENSG00000277075	108.263773	85.0571579	51.6253447	52.1631142	0.896111981	0.0241511	0.078497	HIST1H2AE	histone cluster 1 H2A family member e [Source:HGNC Symbol;Acc:HGNC:4724]
ENSG00000017797	2217.43891	2239.83849	1822.69074	1904.48594	0.258119745	0.0241956	0.0786135	RALBP1	ralA binding protein 1 [Source:HGNC Symbol;Acc:HGNC:9841]
ENSG00000103429	1712.53605	1719.74941	1976.5132	2155.72053	-0.267715042	0.0241963	0.0786135	BFAR	bifunctional apoptosis regulator [Source:HGNC Symbol;Acc:HGNC:17613]
ENSG00000238363	0.98421612	0	8.4286277	11.7100869	-4.418026675	0.0242069	1	SNORA13	small nucleolar RNA, H/ACA box 13 [Source:HGNC Symbol;Acc:HGNC:32601]
ENSG00000112769	1174.16983	1281.17344	1582.47485	1431.82426	-0.295626788	0.0242132	0.0786518	LAMA4	laminin subunit alpha 4 [Source:HGNC Symbol;Acc:HGNC:6484]
ENSG00000065665	355.302019	330.482499	224.412212	265.073784	0.486256738	0.0242187	0.0786518	SEC61A2	Sec61 translocon alpha 2 subunit [Source:HGNC Symbol;Acc:HGNC:17702]
ENSG00000100568	812.962514	815.131097	1004.06027	1018.77756	-0.313178505	0.024222	0.0786518	VTI1B	vesicle transport through interaction with t-SNAREs 1B [Source:HGNC Symbol;Acc:HGNC:17793]
ENSG00000259677	0	0	4.21431385	8.5164268	-5.211425392	0.0242289	1	AC027176.2	Arginine/serine-rich coiled-coil protein (RSRC2) pseudogene
ENSG00000122545	1737.14145	2152.1233	2661.3392	2194.04445	-0.319719909	0.0242731	0.0788024	7-Sep	septin 7 [Source:HGNC Symbol;Acc:HGNC:1717]
ENSG00000158079	336.601912	341.114644	244.430203	243.782717	0.473237562	0.0242846	0.0788246	PTPDC1	protein tyrosine phosphatase domain containing 1 [Source:HGNC Symbol;Acc:HGNC:30184]
ENSG00000172270	21076.004	17702.521	21961.843	24160.0383	-0.250244349	0.0242929	0.0788283	BSG	basigin (Ok blood group) [Source:HGNC Symbol;Acc:HGNC:1116]
ENSG00000273038	22.6369707	26.5803619	47.4110308	66.0023077	-1.202602058	0.0242976	0.0788283	AL365203.2	novel transcript
ENSG00000067596	2198.73881	1998.84321	2557.03493	2470.82833	-0.260644777	0.0243036	0.0788283	DHX8	DEAH-box helicase 8 [Source:HGNC Symbol;Acc:HGNC:2749]
ENSG00000145040	324.791319	265.803619	214.930006	194.813263	0.526428943	0.0243044	0.0788283	UCN2	urocortin 2 [Source:HGNC Symbol;Acc:HGNC:18414]
ENSG00000171262	527.539839	611.348323	780.701641	693.024231	-0.371196161	0.0243145	0.078846	FAM98B	family with sequence similarity 98 member B [Source:HGNC Symbol;Acc:HGNC:26773]
ENSG00000124508	345.459857	363.264945	224.412212	281.042084	0.488025453	0.0243345	0.0788956	BTN2A2	butyrophilin subfamily 2 member A2 [Source:HGNC Symbol;Acc:HGNC:1137]
ENSG00000151690	629.898316	644.130769	500.44977	487.565434	0.366876957	0.0243854	0.0790455	MFSD6	major facilitator superfamily domain containing 6 [Source:HGNC Symbol;Acc:HGNC:24711]
ENSG00000139718	1783.39961	1544.31902	2073.44241	1973.68191	-0.282779042	0.0243961	0.0790521	SETD1B	SET domain containing 1B [Source:HGNC Symbol;Acc:HGNC:29187]
ENSG00000136141	368.096828	317.192318	246.53736	243.782717	0.482258032	0.0243979	0.0790521	LRCH1	leucine rich repeats and calponin homology domain containing 1 [Source:HGNC Symbol;Acc:HGNC:20309]
ENSG00000171798	65.9424799	66.4509046	29.5001969	33.0011539	1.083028573	0.0244014	0.0790521	KNDC1	kinase non-catalytic C-lobe domain containing 1 [Source:HGNC Symbol;Acc:HGNC:29374]
ENSG00000113811	827.725755	832.851338	622.664871	688.766018	0.340621364	0.0244203	0.079098	SELENOK	selenoprotein K [Source:HGNC Symbol;Acc:HGNC:30394]
ENSG00000159788	180.11155	132.015797	231.787262	238.45995	-0.593321452	0.0244732	0.0792541	RGS12	regulator of G protein signaling 12 [Source:HGNC Symbol;Acc:HGNC:9994]
ENSG00000177839	30.5106997	17.7202412	5.26789231	6.3873201	2.046866187	0.0244995	0.0793242	PCDHB9	protocadherin beta 9 [Source:HGNC Symbol;Acc:HGNC:8694]
ENSG00000143995	116.137502	164.798243	89.5541693	75.5832879	0.768081962	0.0245126	0.0793512	MEIS1	Meis homeobox 1 [Source:HGNC Symbol;Acc:HGNC:7000]
ENSG00000111801	191.922143	209.984859	320.287853	259.751017	-0.528812544	0.0245475	0.0794491	BTN3A3	butyrophilin subfamily 3 member A3 [Source:HGNC Symbol;Acc:HGNC:1140]
ENSG00000118849	33.463348	54.0467358	87.4470124	85.164268	-0.976032252	0.0245766	0.0795281	RARRES1	retinoic acid receptor responder 1 [Source:HGNC Symbol;Acc:HGNC:9867]
ENSG00000178209	28739.1107	23675.1283	31991.91	30309.963	-0.249362148	0.0246018	0.0795944	PLEC	plectin [Source:HGNC Symbol;Acc:HGNC:9069]
ENSG00000223478	268.691	277.321775	168.572554	207.587903	0.537894015	0.0246138	0.0796129	AL441992.1	uncharacterized LOC100506100 [Source:NCBI gene;Acc:100506100]
ENSG00000137337	835.599484	835.509374	1038.82836	1032.61675	-0.309834986	0.024617	0.0796129	MDC1	mediator of DNA damage checkpoint 1 [Source:HGNC Symbol;Acc:HGNC:21163]
ENSG00000065559	900.557748	936.514749	699.576099	763.284752	0.328810581	0.0246528	0.0797136	MAP2K4	mitogen-activated protein kinase kinase 4 [Source:HGNC Symbol;Acc:HGNC:6844]
ENSG00000180730	24.605403	33.6684583	9.48220616	7.45187345	1.784067522	0.0246945	0.079833	SHISA2	shisa family member 2 [Source:HGNC Symbol;Acc:HGNC:20366]
ENSG00000231908	28.5422674	13.2901809	3.16073539	5.32276675	2.299584445	0.0247228	0.0799094	IDH1-AS1	IDH1 antisense RNA 1 [Source:HGNC Symbol;Acc:HGNC:40292]
ENSG00000104969	1910.36349	1704.68721	2134.54996	2225.98106	-0.270795565	0.0247465	0.0799707	SGTA	small glutamine rich tetratricopeptide repeat containing alpha [Source:HGNC Symbol;Acc:HGNC:10819]
ENSG00000278828	90.5478829	101.891387	35.8216677	63.873201	0.949838872	0.0247835	0.0800747	HIST1H3H	histone cluster 1 H3 family member h [Source:HGNC Symbol;Acc:HGNC:4775]
ENSG00000204315	208.653817	186.948545	265.501773	302.333151	-0.522091956	0.0247979	0.080106	FKBPL	FK506 binding protein like [Source:HGNC Symbol;Acc:HGNC:13949]
ENSG00000116809	570.845349	509.456936	703.790413	684.507804	-0.362485448	0.0248075	0.0801218	ZBTB17	zinc finger and BTB domain containing 17 [Source:HGNC Symbol;Acc:HGNC:12936]
ENSG00000226580	12.7948095	8.86012062	0	1.06455335	4.351235987	0.0248129	0.0801239	RPL39P40	ribosomal protein L39 pseudogene 40 [Source:HGNC Symbol;Acc:HGNC:35823]
ENSG00000262172	0	0.88601206	13.69652	6.3873201	-4.437195459	0.0248696	1	AC116025.2	novel transcript
ENSG00000004478	6044.07118	5544.66348	6657.5623	6923.85499	-0.229011602	0.0249175	0.0804418	FKBP4	FK506 binding protein 4 [Source:HGNC Symbol;Acc:HGNC:3720]
ENSG00000185513	207.669601	202.01075	113.786474	152.231129	0.623063462	0.0249279	0.0804418	L3MBTL1	L3MBTL1, histone methyl-lysine binding protein [Source:HGNC Symbol;Acc:HGNC:15905]

ENSG00000005302	792.293975	852.343603	1028.29258	1017.713	-0.314714226	0.0249295	0.0804418	MSL3	MSL complex subunit 3 [Source:HGNC Symbol;Acc:HGNC:7370]
ENSG00000112624	266.722568	295.928029	409.842022	363.012692	-0.457376997	0.0249304	0.0804418	BICRAL	BRD4 interacting chromatin remodeling complex associated protein like [Source:HGNC Symbol;Acc:HGNC:21111]
ENSG00000110092	1702.69388	1647.09642	2028.13854	1987.5211	-0.261679409	0.024953	0.0804995	CCND1	cyclin D1 [Source:HGNC Symbol;Acc:HGNC:1582]
ENSG00000167005	1871.97906	2158.32538	2894.18004	2169.55973	-0.329117225	0.0249621	0.0805134	NUDT21	nudix hydrolase 21 [Source:HGNC Symbol;Acc:HGNC:13870]
ENSG00000120324	22.6369707	30.1244101	9.48220616	4.2582134	1.94127489	0.0249681	0.0805175	PCDHB10	protocadherin beta 10 [Source:HGNC Symbol;Acc:HGNC:8681]
ENSG00000143674	482.265898	433.259898	379.288246	286.364851	0.459246789	0.0249798	0.0805397	MAP3K21	mitogen-activated protein kinase kinase kinase 21 [Source:HGNC Symbol;Acc:HGNC:29798]
ENSG00000172057	1104.29048	869.177833	671.12948	840.997147	0.383618359	0.0249864	0.0805455	ORMDL3	ORMDL sphingolipid biosynthesis regulator 3 [Source:HGNC Symbol;Acc:HGNC:16038]
ENSG00000217801	279.517378	248.083377	151.715299	204.394243	0.566789034	0.0249939	0.0805545	AL390719.1	uncharacterized LOC100288175 [Source:NCBI gene;Acc:100288175]
ENSG00000159231	152.553498	96.5753147	184.376231	212.91067	-0.67608242	0.0250051	0.0805751	CBR3	carbonyl reductase 3 [Source:HGNC Symbol;Acc:HGNC:1549]
ENSG00000257337	111.216421	98.3473389	44.2502954	69.1959678	0.885171361	0.0250206	0.0806097	AC068888.1	uncharacterized LOC283335 [Source:NCBI gene;Acc:283335]
ENSG00000110013	705.682957	769.944482	608.968351	540.793102	0.36027413	0.0250258	0.0806112	SIAE	sialic acid acetylesterase [Source:HGNC Symbol;Acc:HGNC:18187]
ENSG00000173020	2562.89877	2276.16499	2876.2692	2907.2952	-0.25750177	0.0250407	0.0806439	GRK2	G protein-coupled receptor kinase 2 [Source:HGNC Symbol;Acc:HGNC:289]
ENSG00000164081	879.88921	750.452216	946.113459	1130.55566	-0.349626011	0.025058	0.080684	TEX264	testis expressed 264 [Source:HGNC Symbol;Acc:HGNC:30247]
ENSG00000185973	98.4216118	110.751508	159.090348	172.457643	-0.663555269	0.0251032	0.0808136	TMLHE	trimethyllysine hydroxylase, epsilon [Source:HGNC Symbol;Acc:HGNC:18308]
ENSG00000134569	572.813781	548.441466	472.003151	367.270906	0.417594468	0.0251078	0.0808136	LRP4	LDL receptor related protein 4 [Source:HGNC Symbol;Acc:HGNC:6696]
ENSG00000186577	274.596297	262.25957	164.35824	204.394243	0.541816473	0.0251415	0.0809066	SMIM29	small integral membrane protein 29 [Source:HGNC Symbol;Acc:HGNC:1340]
ENSG00000214273	2.95264835	10.6321447	0	0	5.127507219	0.0251694	1	AGGF1P1	angiogenic factor with G-patch and FHA domains 1 pseudogene 1 [Source:HGNC Symbol;Acc:HGNC:38051]
ENSG00000183340	279.517378	279.979812	189.644123	202.265137	0.513668991	0.0252377	0.0812008	JRKL	JRK like [Source:HGNC Symbol;Acc:HGNC:6200]
ENSG00000143158	474.392169	608.690286	446.717268	343.850732	0.454889327	0.025261	0.0812603	MPC2	mitochondrial pyruvate carrier 2 [Source:HGNC Symbol;Acc:HGNC:24515]
ENSG00000108256	1944.81105	2076.81227	2641.32121	2240.8848	-0.279637177	0.0253214	0.0814392	NUFIP2	NUFIP2, FMR1 interacting protein 2 [Source:HGNC Symbol;Acc:HGNC:17634]
ENSG00000272668	78.7372895	64.6788805	35.8216677	34.0657072	1.036026579	0.0253528	0.0815247	AL590560.1	uncharacterized LOC107985216 [Source:NCBI gene;Acc:107985216]
ENSG00000129235	1033.42692	878.923965	1207.40092	1174.20235	-0.317211839	0.0253853	0.0816136	TXNDC17	thioredoxin domain containing 17 [Source:HGNC Symbol;Acc:HGNC:28218]
ENSG00000259129	83.65837	85.0571579	41.08956	46.8403474	0.940383542	0.0253944	0.081618	LINC00648	long intergenic non-protein coding RNA 648 [Source:HGNC Symbol;Acc:HGNC:44302]
ENSG00000214485	1232.23858	1540.77498	1658.3325	1790.57874	-0.314070737	0.0253963	0.081618	RPL7P1	ribosomal protein L7 pseudogene 1 [Source:HGNC Symbol;Acc:HGNC:10367]
ENSG00000131873	1798.16285	1868.59944	1558.24255	1486.11648	0.268459144	0.0254041	0.0816275	CHSY1	chondroitin sulfate synthase 1 [Source:HGNC Symbol;Acc:HGNC:17198]
ENSG00000186335	8.85794506	4.43006031	0	0	5.089665104	0.0254392	1	SLC36A2	solute carrier family 36 member 2 [Source:HGNC Symbol;Acc:HGNC:18762]
ENSG00000145982	360.223099	307.446185	466.735259	433.273214	-0.43176645	0.0254398	0.0817265	FARS2	phenylalanyl-tRNA synthetase 2, mitochondrial [Source:HGNC Symbol;Acc:HGNC:21062]
ENSG00000228288	73.8162089	69.1089408	23.1787262	43.6466874	1.097063762	0.0254602	0.0817766	PCAT6	prostate cancer associated transcript 6 [Source:HGNC Symbol;Acc:HGNC:43714]
ENSG00000111728	5.90529671	15.9482171	1.05357846	0	4.371291105	0.0255473	0.0820407	ST8SIA1	ST8 alpha-N-acetyl-neuraminide alpha-2,8-sialyltransferase 1 [Source:HGNC Symbol;Acc:HGNC:10869]
ENSG00000135547	23.6211868	25.6943498	7.37504924	5.32276675	1.957390381	0.025601	0.0821869	HEY2	hes related family bHLH transcription factor with YRPW motif 2 [Source:HGNC Symbol;Acc:HGNC:4881]
ENSG00000163006	171.253605	208.212835	296.055548	255.492804	-0.538317422	0.0256025	0.0821869	CCDC138	coiled-coil domain containing 138 [Source:HGNC Symbol;Acc:HGNC:26531]
ENSG00000112406	237.196084	278.207787	212.822849	110.713548	0.671951248	0.0256384	0.0822863	HECA	hdc homolog, cell cycle regulator [Source:HGNC Symbol;Acc:HGNC:21041]
ENSG00000134709	45.2739414	67.3369167	29.5001969	18.097407	1.243713424	0.0256865	0.082407	HOOK1	hook microtubule tethering protein 1 [Source:HGNC Symbol;Acc:HGNC:19884]
ENSG00000184436	660.409015	654.762914	772.273013	899.547581	-0.346145692	0.0256903	0.082407	THAP7	THAP domain containing 7 [Source:HGNC Symbol;Acc:HGNC:23190]
ENSG00000178607	460.613143	485.53461	331.877216	376.851886	0.417154973	0.0256906	0.082407	ERN1	endoplasmic reticulum to nucleus signaling 1 [Source:HGNC Symbol;Acc:HGNC:3449]
ENSG00000144233	892.684019	886.012062	1099.93591	1093.29629	-0.302274819	0.0256986	0.0824171	AMMECR1L	AMMECR1 like [Source:HGNC Symbol;Acc:HGNC:28658]
ENSG00000138071	3673.09455	4247.54182	4885.44333	4556.28834	-0.253235438	0.0257188	0.082466	ACTR2	ARP2 actin related protein 2 homolog [Source:HGNC Symbol;Acc:HGNC:169]
ENSG00000117450	12421.7916	13334.4815	14042.0937	16372.8305	-0.239813382	0.0257495	0.0825398	PRDX1	peroxiredoxin 1 [Source:HGNC Symbol;Acc:HGNC:9352]
ENSG00000079435	61.0213993	57.590784	35.8216677	12.7746402	1.286206955	0.0257516	0.0825398	LIPE	lipase E, hormone sensitive type [Source:HGNC Symbol;Acc:HGNC:6621]
ENSG00000118246	368.096828	498.824791	663.754431	521.631142	-0.450222004	0.0258372	0.0827985	FASTKD2	FAST kinase domains 2 [Source:HGNC Symbol;Acc:HGNC:29160]
ENSG00000153048	1233.2228	1260.79516	945.059881	1074.13433	0.304817947	0.0258615	0.0828609	CARHSP1	calcium regulated heat stable protein 1 [Source:HGNC Symbol;Acc:HGNC:17150]
ENSG00000136167	6.88951283	6.20208443	0	0	5.070096155	0.0258855	1	LCP1	lymphocyte cytosolic protein 1 [Source:HGNC Symbol;Acc:HGNC:6528]
ENSG00000166402	245.069813	225.047064	175.947603	139.456489	0.575225233	0.0259004	0.0829696	TUB	tubby bipartite transcription factor [Source:HGNC Symbol;Acc:HGNC:12406]
ENSG00000176399	62.0056154	66.4509046	28.4466185	31.9366005	1.089545139	0.025911	0.0829878	DMRTA1	DMRT like family A1 [Source:HGNC Symbol;Acc:HGNC:13826]
ENSG00000104859	680.093338	600.716178	782.808798	845.25536	-0.346654821	0.025927	0.0830234	CLASRP	CLK4 associating serine/arginine rich protein [Source:HGNC Symbol;Acc:HGNC:17731]
ENSG00000257923	2358.18182	2206.17003	2731.92895	2690.12632	-0.24860604	0.0259541	0.0830944	CUX1	cut like homeobox 1 [Source:HGNC Symbol;Acc:HGNC:2557]
ENSG00000107371	451.755198	470.472405	626.879185	570.600596	-0.376627719	0.0260033	0.0832361	EXOSC3	exosome component 3 [Source:HGNC Symbol;Acc:HGNC:17944]
ENSG00000005483	993.074063	1044.60822	1305.38371	1200.81618	-0.298400339	0.0260154	0.0832592	KMT2E	lysine methyltransferase 2E [Source:HGNC Symbol;Acc:HGNC:18541]
ENSG00000115364	729.304144	871.835869	1059.89993	965.549889	-0.33847143	0.0260365	0.083311	MRPL19	mitochondrial ribosomal protein L19 [Source:HGNC Symbol;Acc:HGNC:14052]
ENSG00000165476	1466.48202	1903.15391	1377.02705	1314.72339	0.324573698	0.0260546	0.0833529	REEP3	receptor accessory protein 3 [Source:HGNC Symbol;Acc:HGNC:23711]
ENSG00000272720	57.0845349	56.704772	25.2858831	25.5492804	1.162441433	0.0260734	0.0833971	AL022322.1	novel transcript, antisense to BAIAP2L2

ENSG00000116062	3097.32812	3304.82499	3719.13197	3824.94019	-0.23664912	0.0260782	0.0833971	MSH6	mutS homolog 6 [Source:HGNC Symbol;Acc:HGNC:7329]
ENSG00000100055	17.7158901	18.6062533	5.26789231	1.06455335	2.51802272	0.0260995	0.0834404	CYTH4	cytohesin 4 [Source:HGNC Symbol;Acc:HGNC:9505]
ENSG00000089234	500.966004	481.10455	623.71845	639.796564	-0.363757	0.0261017	0.0834404	BRAP	BRCA1 associated protein [Source:HGNC Symbol;Acc:HGNC:1099]
ENSG00000127337	231.290788	324.280415	482.538936	327.882432	-0.543365125	0.0261293	0.0835069	YEATS4	YEATS domain containing 4 [Source:HGNC Symbol;Acc:HGNC:24859]
ENSG00000137491	8.85794506	12.4041689	0	1.06455335	4.327488638	0.0261323	0.0835069	SLCO2B1	solute carrier organic anion transporter family member 2B1 [Source:HGNC Symbol;Acc:HGNC:10962]
ENSG00000185834	24.605403	27.4663739	59.0003939	56.4213276	-1.147274642	0.0261604	0.0835679	RPL12P4	ribosomal protein L12 pseudogene 4 [Source:HGNC Symbol;Acc:HGNC:16587]
ENSG00000165733	2381.80301	2279.70903	2671.87498	2871.10039	-0.249955234	0.0261613	0.0835679	BMS1	BMS1, ribosome biogenesis factor [Source:HGNC Symbol;Acc:HGNC:23505]
ENSG00000197136	2825.68448	2412.61084	3249.23598	3049.94535	-0.266387443	0.0261902	0.0836442	PCNX3	pecanex 3 [Source:HGNC Symbol;Acc:HGNC:18760]
ENSG00000105671	1270.62301	1041.95018	1325.40171	1585.11994	-0.332295984	0.0262065	0.0836804	DDX49	DEAD-box helicase 49 [Source:HGNC Symbol;Acc:HGNC:18684]
ENSG00000184983	1916.26878	1802.14853	2142.97859	2316.46809	-0.262329836	0.0262215	0.0837126	NDUFA6	NADH:ubiquinone oxidoreductase subunit A6 [Source:HGNC Symbol;Acc:HGNC:7690]
ENSG00000162073	1564.90363	1391.92495	1254.81195	1153.97583	0.295376482	0.0263555	0.0841245	PAQR4	progestin and adipoQ receptor family member 4 [Source:HGNC Symbol;Acc:HGNC:26386]
ENSG00000121316	220.46441	265.803619	309.752068	376.851886	-0.496519918	0.0264218	0.0843201	PLBD1	phospholipase B domain containing 1 [Source:HGNC Symbol;Acc:HGNC:26215]
ENSG00000153767	371.049477	443.892043	318.180696	275.719318	0.457171291	0.0265083	0.0845802	GTF2E1	general transcription factor IIE subunit 1 [Source:HGNC Symbol;Acc:HGNC:4650]
ENSG00000115008	44.2897253	58.4767961	26.3394616	15.9683003	1.281171511	0.0265154	0.0845871	IL1A	interleukin 1 alpha [Source:HGNC Symbol;Acc:HGNC:5991]
ENSG00000090857	769.657004	762.856385	990.363755	919.774095	-0.31784974	0.0265528	0.0846786	PDPR	pyruvate dehydrogenase phosphatase regulatory subunit [Source:HGNC Symbol;Acc:HGNC:30264]
ENSG00000130054	81.6899378	62.9068564	29.5001969	40.4530273	1.046582319	0.0265541	0.0846786	FAM155B	family with sequence similarity 155 member B [Source:HGNC Symbol;Acc:HGNC:30701]
ENSG00000260671	5.90529671	7.08809649	0	0	5.060228224	0.0265605	1	AC010536.2	novel transcript, sense intronic to KLHDC4
ENSG00000144580	1271.60722	1256.3651	1502.40289	1557.44155	-0.275510547	0.0265737	0.084725	CNOT9	CCR4-NOT transcription complex subunit 9 [Source:HGNC Symbol;Acc:HGNC:10445]
ENSG00000070269	267.706784	286.181896	194.912016	193.74871	0.511427713	0.0265954	0.0847782	TMEM260	transmembrane protein 260 [Source:HGNC Symbol;Acc:HGNC:20185]
ENSG00000243836	43.3055092	34.5544704	76.9112277	76.6478412	-0.981902696	0.0266924	0.0850714	WDR86-AS1	WDR86 antisense RNA 1 [Source:HGNC Symbol;Acc:HGNC:41186]
ENSG00000242299	222.432843	189.606581	270.769665	318.301452	-0.516606425	0.0266988	0.0850758	AC073861.1	ribosomal protein S18 (RPS18) pseudogene
ENSG00000160999	381.875854	343.77268	273.9304	253.363697	0.460095313	0.0267076	0.0850878	SH2B2	SH2B adaptor protein 2 [Source:HGNC Symbol;Acc:HGNC:17381]
ENSG00000115286	416.323418	380.099175	537.325016	508.856501	-0.394081372	0.0267531	0.0852133	NDUFS7	NADH:ubiquinone oxidoreductase core subunit S7 [Source:HGNC Symbol;Acc:HGNC:7714]
ENSG00000088682	971.421309	860.317712	1138.91832	1129.4911	-0.308970067	0.0267571	0.0852133	COQ9	coenzyme Q9 [Source:HGNC Symbol;Acc:HGNC:25302]
ENSG00000075826	163.379876	127.585737	64.2682862	105.390782	0.777505903	0.0267814	0.0852744	SEC31B	SEC31 homolog B, COPII coat complex component [Source:HGNC Symbol;Acc:HGNC:23197]
ENSG00000176485	1413.33435	1172.19396	1017.75679	1058.16603	0.316191247	0.0268256	0.0853992	PLA2G16	phospholipase A2 group XVI [Source:HGNC Symbol;Acc:HGNC:17825]
ENSG00000183624	1796.19442	1651.52648	1971.2453	2192.9799	-0.272613527	0.0268338	0.0854094	HMCES	5-hydroxymethylcytosine binding, ES cell specific [Source:HGNC Symbol;Acc:HGNC:24446]
ENSG00000149657	1619.03551	1403.44311	1748.94025	1944.93897	-0.289782077	0.0268562	0.085461	LSM14B	LSM family member 14B [Source:HGNC Symbol;Acc:HGNC:15887]
ENSG00000169249	273.612081	262.25957	324.502166	422.62768	-0.479591277	0.0268602	0.085461	ZRSR2	zinc finger CCCH-type, RNA binding motif and serine/arginine rich 2 [Source:HGNC Symbol;Acc:HGNC:23019]
ENSG00000107859	37.4002125	19.4922654	9.48220616	6.3873201	1.838672157	0.0268696	0.0854723	PITX3	paired like homeodomain 3 [Source:HGNC Symbol;Acc:HGNC:9006]
ENSG00000216285	181.095766	165.684256	246.53736	255.492804	-0.534368334	0.0268739	0.0854723	AC078819.1	phosphoglycerate mutase 1 (brain) (PGAM1) pseudogene
ENSG00000276075	77.7530733	78.8550735	47.4110308	30.8720472	1.000052114	0.0268871	0.0854883	AC027682.6	uncharacterized LOC100505942 [Source:NCBI gene;Acc:100505942]
ENSG00000101901	241.132949	268.461655	339.252265	364.077246	-0.464118408	0.026889	0.0854883	ALG13	ALG13, UDP-N-acetylglucosaminyltransferase subunit [Source:HGNC Symbol;Acc:HGNC:30881]
ENSG00000150316	782.451814	901.960279	955.595665	1192.29975	-0.350117379	0.0268971	0.0854978	CWC15	CWC15 spliceosome associated protein homolog [Source:HGNC Symbol;Acc:HGNC:26939]
ENSG00000108439	422.228715	349.088752	472.003151	563.148722	-0.425340119	0.0269845	0.0857597	PNPO	pyridoxamine 5'-phosphate oxidase [Source:HGNC Symbol;Acc:HGNC:30260]
ENSG00000140374	4485.07285	4430.06031	3805.52541	3824.94019	0.224466302	0.0269966	0.085782	ETFA	electron transfer flavoprotein subunit alpha [Source:HGNC Symbol;Acc:HGNC:3481]
ENSG00000099937	14.7632418	15.0622051	1.05357846	3.19366005	2.813913587	0.0270333	0.0858823	SERPIND1	serpin family D member 1 [Source:HGNC Symbol;Acc:HGNC:4838]
ENSG00000226711	26.5738352	31.0104222	10.5357846	6.3873201	1.76675585	0.0270587	0.0859468	FAM66C	family with sequence similarity 66 member C [Source:HGNC Symbol;Acc:HGNC:21644]
ENSG00000137841	20.6685385	4.43006031	37.9288246	40.4530273	-1.655491628	0.0270645	0.0859493	PLCB2	phospholipase C beta 2 [Source:HGNC Symbol;Acc:HGNC:9055]
ENSG00000140931	109.247989	99.2333509	151.715299	177.780409	-0.660988883	0.0270851	0.0859868	CMTM3	CKLF like MARVEL transmembrane domain containing 3 [Source:HGNC Symbol;Acc:HGNC:19174]
ENSG00000084110	14.7632418	20.3782774	4.21431385	2.1291067	2.47013046	0.0270865	0.0859868	HAL	histidine ammonia-lyase [Source:HGNC Symbol;Acc:HGNC:4806]
ENSG00000213881	1.96843224	7.08809649	23.1787262	19.1619603	-2.20949918	0.0271366	0.0861019	NPM1P6	nucleophosmin 1 pseudogene 6 [Source:HGNC Symbol;Acc:HGNC:7926]
ENSG00000243244	68.8951283	63.7928684	29.5001969	34.0657072	1.061454829	0.0271367	0.0861019	STON1	stonin 1 [Source:HGNC Symbol;Acc:HGNC:17003]
ENSG00000130449	621.040371	616.664395	500.44977	458.822494	0.367571094	0.027138	0.0861019	ZSWIM6	zinc finger SWIM-type containing 6 [Source:HGNC Symbol;Acc:HGNC:29316]
ENSG00000214860	10.8263773	7.97410856	26.3394616	34.0657072	-1.687437437	0.0271567	0.0861449	EVPLL	envoplakin like [Source:HGNC Symbol;Acc:HGNC:35236]
ENSG00000278558	51.1792381	31.0104222	11.5893631	18.097407	1.467371138	0.027166	0.0861581	TMEM191B	transmembrane protein 191B [Source:HGNC Symbol;Acc:HGNC:33600]
ENSG00000223768	826.741539	683.1153	572.093105	600.40809	0.364147651	0.0272109	0.0862845	LINC00205	long intergenic non-protein coding RNA 205 [Source:HGNC Symbol;Acc:HGNC:16420]
ENSG00000136352	51.1792381	31.0104222	20.0179908	8.5164268	1.523284256	0.0272276	0.0863213	NKX2-1	NK2 homeobox 1 [Source:HGNC Symbol;Acc:HGNC:11825]
ENSG00000126261	1720.40977	2026.30959	2259.9258	2274.95051	-0.275020935	0.0272903	0.0865036	UBA2	ubiquitin like modifier activating enzyme 2 [Source:HGNC Symbol;Acc:HGNC:30661]

ENSG00000148660	1718.44134	1649.75446	1334.88391	1449.92166	0.274313422	0.0274001	0.0868354	CAMK2G	calcium/calmodulin dependent protein kinase II gamma [Source:HGNC Symbol;Acc:HGNC:1463]
ENSG00000177879	1256.84398	1451.28776	1226.36533	869.740087	0.369773846	0.027416	0.0868696	AP3S1	adaptor related protein complex 3 subunit sigma 1 [Source:HGNC Symbol;Acc:HGNC:2013]
ENSG00000167378	679.109122	612.234335	429.860013	550.374082	0.397426244	0.0274953	0.0871047	IRGQ	immunity related GTPase Q [Source:HGNC Symbol;Acc:HGNC:24868]
ENSG00000108852	720.446199	670.711131	546.807222	543.986762	0.350596604	0.0275046	0.0871121	MPP2	membrane palmitoylated protein 2 [Source:HGNC Symbol;Acc:HGNC:7220]
ENSG00000075651	633.83518	656.534938	521.521339	483.307221	0.360943908	0.027508	0.0871121	PLD1	phospholipase D1 [Source:HGNC Symbol;Acc:HGNC:9067]
ENSG00000101928	248.022462	289.725944	217.037163	140.521042	0.589103193	0.0275395	0.0871955	MOSPD1	motile sperm domain containing 1 [Source:HGNC Symbol;Acc:HGNC:25235]
ENSG00000108179	2099.33298	1851.76521	2262.03296	2508.08769	-0.272054896	0.0275801	0.0873078	PPIF	peptidylprolyl isomerase F [Source:HGNC Symbol;Acc:HGNC:9259]
ENSG00000185608	874.968129	963.095111	1049.36415	1254.04385	-0.325173714	0.0275857	0.087309	MRPL40	mitochondrial ribosomal protein L40 [Source:HGNC Symbol;Acc:HGNC:14491]
ENSG00000197226	4504.75717	3843.52032	4951.81877	4961.88317	-0.248165279	0.0276631	0.0875369	TBC1D9B	TBC1 domain family member 9B [Source:HGNC Symbol;Acc:HGNC:29097]
ENSG00000215883	375.970557	331.368511	247.590939	265.073784	0.463819599	0.027668	0.0875369	CYB5RL	cytochrome b5 reductase like [Source:HGNC Symbol;Acc:HGNC:32220]
ENSG00000158321	1.96843224	11.5181568	0	0	5.117786963	0.0277196	1	AUTS2	AUTS2, activator of transcription and developmental regulator [Source:HGNC Symbol;Acc:HGNC:14262]
ENSG00000196092	1.96843224	11.5181568	0	0	5.117786963	0.0277196	1	PAX5	paired box 5 [Source:HGNC Symbol;Acc:HGNC:8619]
ENSG00000279879	1.96843224	11.5181568	0	0	5.117786963	0.0277196	1	AC091152.4	TEC
ENSG00000087494	143.695553	149.736038	89.5541693	92.6161415	0.687989674	0.0277282	0.0877109	PTHLH	parathyroid hormone like hormone [Source:HGNC Symbol;Acc:HGNC:9607]
ENSG00000136603	1024.56898	1171.30795	976.667235	729.219045	0.364506835	0.0277581	0.087789	SKIL	SKI like proto-oncogene [Source:HGNC Symbol;Acc:HGNC:10897]
ENSG00000124541	883.826074	920.566532	1047.25699	1191.2352	-0.31080044	0.0277824	0.0878492	RRP36	ribosomal RNA processing 36 [Source:HGNC Symbol;Acc:HGNC:21374]
ENSG00000197857	140.742905	169.228304	89.5541693	103.261675	0.685977764	0.0277893	0.0878547	ZNF44	zinc finger protein 44 [Source:HGNC Symbol;Acc:HGNC:13110]
ENSG00000168806	384.828502	326.052439	462.520945	485.436328	-0.416139007	0.0278032	0.0878822	LCMT2	leucine carboxyl methyltransferase 2 [Source:HGNC Symbol;Acc:HGNC:17558]
ENSG00000204977	106.295341	155.052111	224.412212	184.16773	-0.642340022	0.0278222	0.0879259	TRIM13	tripartite motif containing 13 [Source:HGNC Symbol;Acc:HGNC:9976]
ENSG00000173548	1387.74473	1304.20975	1658.3325	1593.63637	-0.272892886	0.0278358	0.0879525	SNX33	sorting nexin 33 [Source:HGNC Symbol;Acc:HGNC:28468]
ENSG00000132932	84.6425862	77.9690614	43.196717	41.5175807	0.940272176	0.0278699	0.0880437	ATP8A2	ATPase phospholipid transporting 8A2 [Source:HGNC Symbol;Acc:HGNC:13533]
ENSG00000117543	275.580513	339.34262	433.020748	404.530273	-0.444605454	0.027904	0.0881348	DPH5	diphthamide biosynthesis 5 [Source:HGNC Symbol;Acc:HGNC:24270]
ENSG00000160285	2785.33161	2342.61589	2240.96139	1965.16548	0.285562036	0.0279372	0.0882232	LSS	lanosterol synthase [Source:HGNC Symbol;Acc:HGNC:6708]
ENSG00000148384	633.83518	517.431044	715.379776	768.607519	-0.367150774	0.0279557	0.0882653	INPP5E	inositol polyphosphate-5-phosphatase E [Source:HGNC Symbol;Acc:HGNC:21474]
ENSG00000177679	144.679769	158.596159	105.357846	81.970608	0.695322904	0.0279825	0.0883273	SRRM3	serine/arginine repetitive matrix 3 [Source:HGNC Symbol;Acc:HGNC:26729]
ENSG00000270231	485.218546	525.405153	398.252659	369.400013	0.397038002	0.0279858	0.0883273	NBPF8	NBPF member 8 [Source:HGNC Symbol;Acc:HGNC:31990]
ENSG00000081087	482.265898	447.436091	398.252659	271.461104	0.472763783	0.0279958	0.0883424	OSTM1	osteopetrosis associated transmembrane protein 1 [Source:HGNC Symbol;Acc:HGNC:21652]
ENSG00000259207	32.4791319	45.1866152	17.9108339	9.58098015	1.499088792	0.0280061	0.0883582	ITGB3	integrin subunit beta 3 [Source:HGNC Symbol;Acc:HGNC:6156]
ENSG00000130348	197.82744	258.715522	355.055942	297.010385	-0.512798914	0.0280311	0.0884137	QRSL1	QRSL1, glutaminyl-tRNA amidotransferase subunit A [Source:HGNC Symbol;Acc:HGNC:21020]
ENSG00000141646	557.066323	591.856057	858.666447	645.11933	-0.388171203	0.0280341	0.0884137	SMAD4	SMAD family member 4 [Source:HGNC Symbol;Acc:HGNC:6770]
ENSG00000171928	335.617696	394.275367	529.949967	449.241514	-0.423117229	0.0280685	0.0884951	TVP23B	trans-golgi network vesicle protein 23 homolog B [Source:HGNC Symbol;Acc:HGNC:20399]
ENSG00000136273	277.548945	288.839932	383.50256	383.239206	-0.436689573	0.0280704	0.0884951	HUS1	HUS1 checkpoint clamp component [Source:HGNC Symbol;Acc:HGNC:5309]
ENSG00000164778	137.790257	101.005375	67.4290216	70.2605211	0.792837175	0.0280894	0.0885386	EN2	engrailed homeobox 2 [Source:HGNC Symbol;Acc:HGNC:3343]
ENSG00000285812	17.7158901	15.0622051	1.05357846	4.2582134	2.626875599	0.0281588	0.0887407	AL390719.3	novel transcript, antisense to C1orf159
ENSG00000096401	934.021096	1116.3752	1326.45528	1224.23635	-0.314390757	0.0281771	0.0887625	CDC5L	cell division cycle 5 like [Source:HGNC Symbol;Acc:HGNC:1743]
ENSG00000104953	159.443011	139.103894	89.5541693	95.8098015	0.686927157	0.0281842	0.0887625	TLE6	transducin like enhancer of split 6 [Source:HGNC Symbol;Acc:HGNC:30788]
ENSG00000244627	77.7530733	81.5131097	42.1431385	40.4530273	0.947506061	0.0281899	0.0887625	TPTEP2	TPTE pseudogene 2 [Source:HGNC Symbol;Acc:HGNC:53828]
ENSG00000105701	5666.13219	4585.11242	6204.52356	6077.53508	-0.260969771	0.0281916	0.0887625	FKBP8	FK506 binding protein 8 [Source:HGNC Symbol;Acc:HGNC:3724]
ENSG00000142875	503.918653	614.006359	484.646093	322.559665	0.470217995	0.028192	0.0887625	PRKACB	protein kinase cAMP-activated catalytic subunit beta [Source:HGNC Symbol;Acc:HGNC:9381]
ENSG00000128463	1214.52269	1095.11091	1368.59842	1453.11532	-0.289266183	0.0282109	0.0888054	EMC4	ER membrane protein complex subunit 4 [Source:HGNC Symbol;Acc:HGNC:28032]
ENSG00000175294	8.85794506	8.86012062	34.7680893	23.4201737	-1.715708723	0.0282343	0.0888627	CATSPER1	cation channel sperm associated 1 [Source:HGNC Symbol;Acc:HGNC:17116]
ENSG00000165650	1057.04811	1127.89335	937.684831	819.70608	0.31431583	0.0283826	0.0893126	PDZD8	PDZ domain containing 8 [Source:HGNC Symbol;Acc:HGNC:26974]
ENSG00000256537	447.818334	549.327478	680.611687	620.634603	-0.383080028	0.0283925	0.089318	SMIM10L1	small integral membrane protein 10 like 1 [Source:HGNC Symbol;Acc:HGNC:49847]
ENSG00000090376	300.185916	399.59144	250.751674	246.976377	0.492751111	0.0283948	0.089318	IRAK3	interleukin 1 receptor associated kinase 3 [Source:HGNC Symbol;Acc:HGNC:17020]
ENSG00000139344	87.5952345	95.6893027	42.1431385	56.4213276	0.895673288	0.0284032	0.0893277	AMDHD1	amidohydrolase domain containing 1 [Source:HGNC Symbol;Acc:HGNC:28577]
ENSG00000164930	975.358173	993.219521	1229.52607	1178.46056	-0.290613433	0.0284321	0.0894019	FZD6	frizzled class receptor 6 [Source:HGNC Symbol;Acc:HGNC:4044]
ENSG00000198929	85.6268023	106.321447	65.3218647	34.0657072	0.949998614	0.0284539	0.0894538	NOS1AP	nitric oxide synthase 1 adaptor protein [Source:HGNC Symbol;Acc:HGNC:16859]
ENSG00000258881	13.7790257	7.08809649	1.05357846	0	4.298879298	0.02847	0.0894876	AC007040.2	novel protein
ENSG00000162300	122.042799	107.207459	180.161917	173.522196	-0.626551328	0.0284846	0.089517	ZFPL1	zinc finger protein like 1 [Source:HGNC Symbol;Acc:HGNC:12868]
ENSG00000158406	114.16907	103.663411	64.2682862	60.679541	0.80131764	0.0285037	0.0895603	HIST1H4H	histone cluster 1 H4 family member h [Source:HGNC Symbol;Acc:HGNC:4788]
ENSG00000100554	1175.15405	1394.58299	1014.59606	1060.29514	0.309101565	0.0285138	0.0895754	ATP6V1D	ATPase H+ transporting V1 subunit D [Source:HGNC Symbol;Acc:HGNC:13527]

ENSG00000136100	191.922143	202.01075	270.769665	287.429405	-0.502438566	0.0285272	0.0896009	VPS36	vacuolar protein sorting 36 homolog [Source:HGNC Symbol;Acc:HGNC:20312]
ENSG00000185262	1152.51707	1035.7481	1293.79435	1388.17757	-0.293880455	0.0285395	0.0896228	UBALD2	UBA like domain containing 2 [Source:HGNC Symbol;Acc:HGNC:28438]
ENSG00000196839	338.570345	337.570596	411.949179	495.017308	-0.423652447	0.0285769	0.0896974	ADA	adenosine deaminase [Source:HGNC Symbol;Acc:HGNC:186]
ENSG00000103269	59.0529671	57.590784	30.5537754	22.3556204	1.140057659	0.0285786	0.0896974	RHBDL1	rhomboid like 1 [Source:HGNC Symbol;Acc:HGNC:10007]
ENSG00000116688	2943.79041	2648.29005	3380.93329	3246.88772	-0.245379189	0.0285792	0.0896974	MFN2	mitofusin 2 [Source:HGNC Symbol;Acc:HGNC:16877]
ENSG00000128040	8.85794506	17.7202412	2.10715692	1.06455335	3.068324473	0.0286912	0.0900322	SPINK2	serine peptidase inhibitor, Kazal type 2 [Source:HGNC Symbol;Acc:HGNC:11245]
ENSG00000156298	9.84216118	14.176193	0	2.1291067	3.501599296	0.0287362	0.0901569	TSPAN7	tetraspanin 7 [Source:HGNC Symbol;Acc:HGNC:11854]
ENSG00000132356	1185.98042	1298.89368	1686.77912	1388.17757	-0.307174052	0.0287615	0.0902141	PRKAA1	protein kinase AMP-activated catalytic subunit alpha 1 [Source:HGNC Symbol;Acc:HGNC:9376]
ENSG00000086758	13520.1768	12650.4802	16727.6652	14151.1077	-0.238704248	0.0287652	0.0902141	HUWE1	HECT, UBA and WWE domain containing 1, E3 ubiquitin protein ligase [Source:HGNC Symbol;Acc:HGNC:30892]
ENSG00000196683	1938.90575	1539.00295	2072.38884	2201.49633	-0.297839835	0.0288112	0.0903417	TOMM7	translocase of outer mitochondrial membrane 7 [Source:HGNC Symbol;Acc:HGNC:21648]
ENSG00000170677	358.254667	419.969717	581.575311	463.080707	-0.424076647	0.0288328	0.0903928	SOCS6	suppressor of cytokine signaling 6 [Source:HGNC Symbol;Acc:HGNC:16833]
ENSG00000181061	530.492488	797.410856	494.128299	486.500881	0.438493478	0.0288531	0.0904395	HIGD1A	HIG1 hypoxia inducible domain family member 1A [Source:HGNC Symbol;Acc:HGNC:29527]
ENSG00000100603	1602.30384	1717.97739	1832.17295	2229.17472	-0.290433445	0.0288657	0.0904624	SNW1	SNW domain containing 1 [Source:HGNC Symbol;Acc:HGNC:16696]
ENSG00000264964	17.7158901	16.8342292	4.21431385	2.1291067	2.444019833	0.0288964	0.0905316	AP001033.1	novel transcript
ENSG00000265752	21.6527546	15.9482171	2.10715692	5.32276675	2.339405576	0.0288986	0.0905316	AC010754.1	novel transcript
ENSG00000272341	24.605403	51.3886996	16.8572554	8.5164268	1.584758687	0.0289168	0.0905721	AL137003.2	novel transcript
ENSG00000205693	11.8105934	17.7202412	1.05357846	3.19366005	2.801160141	0.0289412	0.0906316	MANSC4	MANSC domain containing 4 [Source:HGNC Symbol;Acc:HGNC:40023]
ENSG00000279278	71.8477766	52.2747116	18.9644123	36.1948139	1.169259736	0.0289584	0.0906687	AC245060.6	novel transcript
ENSG00000244588	3.93686447	8.86012062	0	0	5.040225174	0.0289744	1	RAD21L1	RAD21 cohesin complex component like 1 [Source:HGNC Symbol;Acc:HGNC:16271]
ENSG00000163605	499.981788	627.29654	860.773604	639.796564	-0.411935388	0.0290124	0.0908209	PPP4R2	protein phosphatase 4 regulatory subunit 2 [Source:HGNC Symbol;Acc:HGNC:18296]
ENSG00000259438	15.7474579	15.9482171	3.16073539	2.1291067	2.582348107	0.0290183	0.090822	MAPK6-DT	MAPK6 divergent transcript [Source:HGNC Symbol;Acc:HGNC:53900]
ENSG00000164405	3803.01108	3957.81588	4445.04753	4624.41975	-0.224729229	0.0290235	0.090822	UQCRQ	ubiquinol-cytochrome c reductase complex III subunit VII [Source:HGNC Symbol;Acc:HGNC:29594]
ENSG00000197530	974.373957	824.87723	1082.02508	1160.36315	-0.318253487	0.0290302	0.0908261	MIB2	mindbomb E3 ubiquitin protein ligase 2 [Source:HGNC Symbol;Acc:HGNC:30577]
ENSG00000082068	1043.26909	1073.84662	1261.13342	1317.91705	-0.284605477	0.0290451	0.0908561	WDR70	WD repeat domain 70 [Source:HGNC Symbol;Acc:HGNC:25495]
ENSG00000234287	118.105934	74.4250132	139.072357	179.909516	-0.731281229	0.0290744	0.0909191	AC099560.2	ribosomal protein S27 (RPS27) pseudogene
ENSG00000223638	9.84216118	10.6321447	1.05357846	0	4.274188867	0.0290765	1	RFPL4A	ret finger protein like 4A [Source:HGNC Symbol;Acc:HGNC:16449]
ENSG00000099219	1157.43816	1269.65528	1057.79278	893.160261	0.31525667	0.0290777	0.0909191	ERMP1	endoplasmic reticulum metallopeptidase 1 [Source:HGNC Symbol;Acc:HGNC:23703]
ENSG00000113812	410.418121	435.031922	575.25384	525.889355	-0.380925342	0.0290814	0.0909191	ACTR8	ARP8 actin related protein 8 homolog [Source:HGNC Symbol;Acc:HGNC:14672]
ENSG00000100104	205.701169	187.834557	246.53736	320.430558	-0.527213016	0.0291399	0.091085	SRRD	SRR1 domain containing [Source:HGNC Symbol;Acc:HGNC:33910]
ENSG00000263089	12.7948095	13.2901809	2.10715692	1.06455335	3.038708945	0.0291475	0.0910919	AC007114.2	novel transcript, antisense to AKAP1
ENSG00000251474	261.801487	211.756883	141.179514	176.715856	0.574160508	0.0292465	0.0913845	RPL32P3	ribosomal protein L32 pseudogene 3 [Source:HGNC Symbol;Acc:HGNC:27024]
ENSG00000176884	79.7215056	77.9690614	53.7325016	19.1619603	1.112359639	0.0293668	0.0917433	GRIN1	glutamate ionotropic receptor NMDA type subunit 1 [Source:HGNC Symbol;Acc:HGNC:4584]
ENSG00000175567	1430.06602	1326.36006	1695.20775	1628.76663	-0.270383732	0.0294339	0.0919332	UCP2	uncoupling protein 2 [Source:HGNC Symbol;Acc:HGNC:12518]
ENSG00000228794	275.580513	286.181896	189.644123	207.587903	0.500241618	0.0294384	0.0919332	LINC01128	long intergenic non-protein coding RNA 1128 [Source:HGNC Symbol;Acc:HGNC:49377]
ENSG00000203879	2374.91349	2130.85901	1916.45922	1845.93551	0.259860109	0.0294525	0.0919601	GDI1	GDP dissociation inhibitor 1 [Source:HGNC Symbol;Acc:HGNC:4226]
ENSG00000184640	7259.57809	6294.22969	7967.16033	7953.27808	-0.232318085	0.0294929	0.0920692	9-Sep	septin 9 [Source:HGNC Symbol;Acc:HGNC:7323]
ENSG00000224940	67.9109122	44.3006031	20.0179908	28.7429405	1.20078265	0.0295284	0.0921484	PRRT4	proline rich transmembrane protein 4 [Source:HGNC Symbol;Acc:HGNC:37280]
ENSG00000092094	514.74503	436.803946	644.790019	590.827109	-0.377757038	0.0295292	0.0921484	OSGEP	O-sialoglycoprotein endopeptidase [Source:HGNC Symbol;Acc:HGNC:18028]
ENSG00000170113	927.131583	919.68052	798.612474	667.474951	0.332953508	0.0295391	0.0921626	NIPA1	NIPA magnesium transporter 1 [Source:HGNC Symbol;Acc:HGNC:17043]
ENSG00000107140	516.713462	447.436091	365.591726	361.948139	0.405572205	0.0295622	0.0922064	TESK1	testis associated actin remodelling kinase 1 [Source:HGNC Symbol;Acc:HGNC:11731]
ENSG00000198399	582.655942	601.60219	477.271043	440.725087	0.367518089	0.0295641	0.0922064	ITSN2	intersectin 2 [Source:HGNC Symbol;Acc:HGNC:6184]
ENSG00000068745	1794.22598	1835.81699	2107.15692	2214.27097	-0.251437819	0.0295956	0.0922875	IP6K2	inositol hexakisphosphate kinase 2 [Source:HGNC Symbol;Acc:HGNC:17313]
ENSG00000152404	232.275004	269.347667	365.591726	327.882432	-0.466366802	0.029618	0.0923404	CWF19L2	CWF19 like cell cycle control factor 2 [Source:HGNC Symbol;Acc:HGNC:26508]
ENSG00000175877	28.5422674	25.6943498	8.4286277	7.45187345	1.771332246	0.0296618	0.09246	TMEM270	transmembrane protein 270 [Source:HGNC Symbol;Acc:HGNC:23018]
ENSG00000188021	1545.21931	1633.80624	2027.08496	1806.54704	-0.269992332	0.0297222	0.0926311	UBQLN2	ubiquilin 2 [Source:HGNC Symbol;Acc:HGNC:12509]
ENSG00000147813	329.7124	295.042017	400.359816	438.59598	-0.425932545	0.0297326	0.0926464	NAPRT	nicotinate phosphoribosyltransferase [Source:HGNC Symbol;Acc:HGNC:30450]
ENSG00000148057	31.4949158	24.8083377	54.78608	66.0023077	-1.103361489	0.029747	0.0926743	IDNK	IDNK, gluconokinase [Source:HGNC Symbol;Acc:HGNC:31367]
ENSG00000163082	17.7158901	19.4922654	2.10715692	5.32276675	2.325997293	0.0297786	0.0927556	SGPP2	sphingosine-1-phosphate phosphatase 2 [Source:HGNC Symbol;Acc:HGNC:19953]
ENSG00000105825	609.229777	628.182552	456.199474	505.662841	0.363618962	0.0297934	0.0927847	TFPI2	tissue factor pathway inhibitor 2 [Source:HGNC Symbol;Acc:HGNC:11761]
ENSG00000089195	630.882532	695.519469	868.148653	801.608673	-0.331692818	0.0298237	0.0928619	TRMT6	tRNA methyltransferase 6 [Source:HGNC Symbol;Acc:HGNC:20900]
ENSG00000130540	274.596297	207.326822	177.001182	145.843809	0.576563058	0.0298583	0.0929525	SULT4A1	sulfotransferase family 4A member 1 [Source:HGNC Symbol;Acc:HGNC:14903]

ENSG00000108669	972.405525	901.960279	710.111884	789.898586	0.32120643	0.0298715	0.0929762	CYTH1	cytohesin 1 [Source:HGNC Symbol;Acc:HGNC:9501]
ENSG00000130177	727.335711	757.540313	935.577675	908.064008	-0.312034444	0.0299235	0.093121	CDC16	cell division cycle 16 [Source:HGNC Symbol;Acc:HGNC:1720]
ENSG00000063180	177.158901	131.129785	225.465791	233.137184	-0.574941971	0.0300046	0.0933563	CA11	carbonic anhydrase 11 [Source:HGNC Symbol;Acc:HGNC:1370]
ENSG00000151694	1014.72682	1020.6859	818.630465	833.545273	0.300985764	0.0300227	0.0933953	ADAM17	ADAM metallopeptidase domain 17 [Source:HGNC Symbol;Acc:HGNC:195]
ENSG00000230882	22.6369707	9.74613268	3.16073539	2.1291067	2.609932868	0.0300681	0.0935195	AC005077.4	hypothetical protein LOC285908 (LOC285908) pseudogene
ENSG00000273802	83.65837	89.4872182	45.3038739	47.9049008	0.893865347	0.0301083	0.0936273	HIST1H2BG	histone cluster 1 H2B family member g [Source:HGNC Symbol;Acc:HGNC:4746]
ENSG00000172159	251.959326	301.244101	201.233486	186.296836	0.514321821	0.0301206	0.0936483	FRMD3	FERM domain containing 3 [Source:HGNC Symbol;Acc:HGNC:24125]
ENSG00000111652	1452.70299	1436.22555	1650.95745	1832.09632	-0.26982394	0.0301279	0.0936538	COPS7A	COP9 signalosome subunit 7A [Source:HGNC Symbol;Acc:HGNC:16758]
ENSG00000262587	32.4791319	23.9223257	8.4286277	8.5164268	1.73341826	0.0301474	0.0936971	AC133552.2	leucine carboxyl methyltransferase 1 pseudogene [Source:NCBI gene;Acc:554206]
ENSG00000149782	3637.66277	3227.74194	3943.54418	4171.98458	-0.241537487	0.0302239	0.0939174	PLCB3	phospholipase C beta 3 [Source:HGNC Symbol;Acc:HGNC:9056]
ENSG00000137221	511.792381	519.203068	657.43296	654.70031	-0.347799285	0.0302334	0.0939299	TJAP1	tight junction associated protein 1 [Source:HGNC Symbol;Acc:HGNC:17949]
ENSG00000140577	1616.08287	1468.12199	1900.65555	1808.67614	-0.266577612	0.0302864	0.0940677	CRTC3	CREB regulated transcription coactivator 3 [Source:HGNC Symbol;Acc:HGNC:26148]
ENSG00000188706	855.283807	835.509374	613.182665	724.960832	0.337464508	0.0302889	0.0940677	ZDHHC9	zinc finger DHHC-type containing 9 [Source:HGNC Symbol;Acc:HGNC:18475]
ENSG00000156802	2456.60343	3070.03179	3651.70295	3114.8831	-0.291722208	0.0302948	0.0940683	ATAD2	ATPase family, AAA domain containing 2 [Source:HGNC Symbol;Acc:HGNC:30123]
ENSG00000151468	117.121718	125.813713	79.0183847	64.9377544	0.755149366	0.0303051	0.0940683	CCDC3	coiled-coil domain containing 3 [Source:HGNC Symbol;Acc:HGNC:23813]
ENSG00000169062	626.945667	570.591768	736.451345	777.123946	-0.338337533	0.0303058	0.0940683	UPF3A	UPF3A, regulator of nonsense mediated mRNA decay [Source:HGNC Symbol;Acc:HGNC:20332]
ENSG00000221988	356.286235	318.964342	414.056336	490.759094	-0.422745248	0.0303299	0.0941257	PPT2	palmitoyl-protein thioesterase 2 [Source:HGNC Symbol;Acc:HGNC:9326]
ENSG00000068831	10.8263773	20.3782774	4.21431385	0	2.887090628	0.0303503	0.0941695	RASGRP2	RAS guanyl releasing protein 2 [Source:HGNC Symbol;Acc:HGNC:9879]
ENSG00000174721	35.4317803	43.414591	16.8572554	12.7746402	1.412575963	0.0303552	0.0941695	FGFBP3	fibroblast growth factor binding protein 3 [Source:HGNC Symbol;Acc:HGNC:23428]
ENSG00000171817	29.5264835	44.3006031	13.69652	12.7746402	1.481583938	0.0303745	0.0942122	ZNF540	zinc finger protein 540 [Source:HGNC Symbol;Acc:HGNC:25331]
ENSG00000119638	3630.77326	3319.00118	2957.39474	2941.36091	0.236393232	0.0303929	0.0942349	NEK9	NIMA related kinase 9 [Source:HGNC Symbol;Acc:HGNC:18591]
ENSG00000128710	358.254667	322.50839	409.842022	505.662841	-0.427893283	0.030393	0.0942349	HOXD10	homeobox D10 [Source:HGNC Symbol;Acc:HGNC:5133]
ENSG00000197386	3584.5151	3465.19317	3178.64622	2751.87041	0.249321997	0.0304089	0.0942671	HTT	huntingtin [Source:HGNC Symbol;Acc:HGNC:4851]
ENSG00000214796	124.011231	103.663411	73.7504924	57.4858809	0.793669327	0.0304213	0.094276	AC098934.1	tubulin, alpha (TUBA) pseudogene
ENSG00000204709	22.6369707	18.6062533	5.26789231	4.2582134	2.112962853	0.0304229	0.094276	LINC01556	long intergenic non-protein coding RNA 1556 [Source:HGNC Symbol;Acc:HGNC:21195]
ENSG00000229212	63.9740477	79.7410856	29.5001969	41.5175807	1.018389443	0.0305002	0.0944982	AC044860.1	golgin subfamily A member 2 [Source:NCBI gene;Acc:101929479]
ENSG00000113360	2337.51328	2368.31024	2767.75062	2773.16148	-0.235641319	0.030516	0.0945295	DROSHA	drosha ribonuclease III [Source:HGNC Symbol;Acc:HGNC:17904]
ENSG00000166477	814.930946	831.965326	1059.89993	975.130869	-0.305242558	0.0305652	0.0946648	LEO1	LEO1 homolog, Paf1/RNA polymerase II complex component [Source:HGNC Symbol;Acc:HGNC:30401]
ENSG00000039123	1042.28487	1414.96126	1662.54681	1453.11532	-0.341799701	0.0305977	0.0947481	MTREX	Mtr4 exosome RNA helicase [Source:HGNC Symbol;Acc:HGNC:18734]
ENSG00000279789	42.3212931	41.6425669	10.5357846	21.291067	1.400198022	0.030696	0.0950298	AC120114.3	novel transcript, sense intronic to KCTD13
ENSG00000144893	90.5478829	77.9690614	38.9824031	50.0340075	0.920208512	0.0307	0.0950298	MED12L	mediator complex subunit 12 like [Source:HGNC Symbol;Acc:HGNC:16050]
ENSG00000135900	351.365154	386.301259	453.038739	523.760248	-0.404488758	0.0307258	0.0950925	MRPL44	mitochondrial ribosomal protein L44 [Source:HGNC Symbol;Acc:HGNC:16650]
ENSG00000101473	569.861132	556.415575	423.538542	448.17696	0.369557609	0.0307342	0.0951011	ACOT8	acyl-CoA thioesterase 8 [Source:HGNC Symbol;Acc:HGNC:15919]
ENSG00000126970	114.16907	114.295556	53.7325016	77.7123946	0.797834042	0.0307404	0.0951029	ZC4H2	zinc finger C4H2-type containing [Source:HGNC Symbol;Acc:HGNC:24931]
ENSG00000157890	29.5264835	14.176193	2.10715692	7.45187345	2.191550968	0.0307903	0.0952384	MEGF11	multiple EGF like domains 11 [Source:HGNC Symbol;Acc:HGNC:29635]
ENSG00000258733	24.605403	16.8342292	7.37504924	1.06455335	2.292121499	0.0307955	0.0952384	LINC02328	long intergenic non-protein coding RNA 2328 [Source:HGNC Symbol;Acc:HGNC:53248]
ENSG00000189046	467.502656	413.767633	503.610505	669.604057	-0.413253067	0.0308958	0.0955311	ALKBH2	alkB homolog 2, alpha-ketoglutarate dependent dioxygenase [Source:HGNC Symbol;Acc:HGNC:32487]
ENSG00000226210	188.969495	146.19199	247.590939	241.653611	-0.547425045	0.0309667	0.0957328	WASH8P	WAS protein family homolog 8, pseudogene [Source:HGNC Symbol;Acc:HGNC:53913]
ENSG00000147955	3447.70906	3101.92823	3865.57938	3844.10215	-0.235456701	0.0309841	0.0957611	SIGMAR1	sigma non-opioid intracellular receptor 1 [Source:HGNC Symbol;Acc:HGNC:8157]
ENSG00000150630	494.076491	425.28579	311.859225	372.593673	0.42515264	0.0309872	0.0957611	VEGFC	vascular endothelial growth factor C [Source:HGNC Symbol;Acc:HGNC:12682]
ENSG00000153443	716.509334	627.29654	808.094681	885.708387	-0.334506517	0.0310241	0.0958577	UBALD1	UBA like domain containing 1 [Source:HGNC Symbol;Acc:HGNC:29576]
ENSG00000100442	1215.50691	1325.47404	1478.17058	1604.2819	-0.278366483	0.0310577	0.095944	FKBP3	FK506 binding protein 3 [Source:HGNC Symbol;Acc:HGNC:3719]
ENSG00000166548	305.106997	316.306306	397.19908	433.273214	-0.418108121	0.0310795	0.0959937	TK2	thymidine kinase 2, mitochondrial [Source:HGNC Symbol;Acc:HGNC:11831]
ENSG00000172264	29.5264835	30.1244101	9.48220616	9.58098015	1.645864828	0.0311185	0.0960967	MACROD2	MACRO domain containing 2 [Source:HGNC Symbol;Acc:HGNC:16126]
ENSG00000061936	1068.8587	986.131425	1157.88273	1389.24212	-0.309959547	0.0311387	0.0961412	SFSWAP	splicing factor SWAP [Source:HGNC Symbol;Acc:HGNC:10790]
ENSG00000165810	13.7790257	15.0622051	3.16073539	1.06455335	2.769548984	0.0311515	0.0961634	BTNL9	butyrophilin like 9 [Source:HGNC Symbol;Acc:HGNC:24176]
ENSG00000072736	950.75277	994.991545	824.951936	741.993685	0.312482054	0.0311605	0.0961736	NFATC3	nuclear factor of activated T cells 3 [Source:HGNC Symbol;Acc:HGNC:7777]
ENSG00000087095	474.392169	523.633129	389.824031	371.529119	0.390935197	0.0312329	0.0963793	NLK	nemo like kinase [Source:HGNC Symbol;Acc:HGNC:29858]
ENSG00000206532	12.7948095	9.74613268	1.05357846	1.06455335	3.410555034	0.0312538	0.0964263	AC117402.1	novel transcript
ENSG00000130958	89.5636668	57.590784	108.518582	145.843809	-0.792529016	0.0312664	0.0964477	SLC35D2	solute carrier family 35 member D2 [Source:HGNC Symbol;Acc:HGNC:20799]
ENSG00000206172	26.5738352	2.65803619	1.05357846	2.1291067	3.195186652	0.0312781	0.0964661	HBA1	hemoglobin subunit alpha 1 [Source:HGNC Symbol;Acc:HGNC:4823]
ENSG00000063601	548.208378	594.514093	664.80801	800.544119	-0.358321862	0.0312926	0.0964931	MTMR1	myotubularin related protein 1 [Source:HGNC Symbol;Acc:HGNC:7449]
ENSG00000151640	78.7372895	54.0467358	29.5001969	34.0657072	1.061041093	0.0313212	0.0965637	DPYSL4	dihydropyrimidinase like 4 [Source:HGNC Symbol;Acc:HGNC:3016]

ENSG00000172845	1079.68508	1286.48951	1598.27853	1344.53088	-0.31417398	0.0313721	0.0967031	SP3	Sp3 transcription factor [Source:HGNC Symbol;Acc:HGNC:11208]
ENSG00000175104	240.148733	262.25957	190.697702	156.489342	0.533438327	0.0314371	0.0968857	TRAF6	TNF receptor associated factor 6 [Source:HGNC Symbol;Acc:HGNC:12036]
ENSG00000101350	3715.41585	3823.14205	3289.27196	3156.40068	0.225999138	0.0314485	0.0969008	KIF3B	kinesin family member 3B [Source:HGNC Symbol;Acc:HGNC:6320]
ENSG00000198948	128.932311	150.622051	83.2326985	89.4224814	0.696123535	0.0314535	0.0969008	MFAP3L	microfibril associated protein 3 like [Source:HGNC Symbol;Acc:HGNC:29083]
ENSG00000104365	536.397784	530.721225	627.932764	738.800025	-0.356991642	0.0314667	0.0969227	IKBKB	inhibitor of nuclear factor kappa B kinase subunit beta [Source:HGNC Symbol;Acc:HGNC:5960]
ENSG00000167792	3071.7385	2651.8341	3309.28995	3503.44508	-0.251584193	0.0314721	0.0969227	NDUFV1	NADH:ubiquinone oxidoreductase core subunit V1 [Source:HGNC Symbol;Acc:HGNC:7716]
ENSG00000130600	83.65837	253.39945	604.754037	437.531427	-1.627929144	0.031504	0.0969906	H19	H19, imprinted maternally expressed transcript [Source:HGNC Symbol;Acc:HGNC:4713]
ENSG00000175899	11.8105934	10.6321447	1.05357846	1.06455335	3.404951938	0.0315056	0.0969906	A2M	alpha-2-macroglobulin [Source:HGNC Symbol;Acc:HGNC:7]
ENSG00000152818	1194.83837	1363.57256	1124.16822	928.290521	0.318177848	0.0315114	0.0969908	UTRN	utrophin [Source:HGNC Symbol;Acc:HGNC:12635]
ENSG00000141279	2116.06465	2473.74568	2624.46395	2896.64967	-0.266179628	0.0315643	0.097136	NPEPPS	aminopeptidase puromycin sensitive [Source:HGNC Symbol;Acc:HGNC:7900]
ENSG00000153982	188.969495	146.19199	119.054366	91.5515881	0.6688886	0.0315856	0.0971839	GDPD1	glycerophosphodiester phosphodiesterase domain containing 1 [Source:HGNC Symbol;Acc:HGNC:20883]
ENSG00000107537	307.075429	320.736366	413.002757	422.62768	-0.412246212	0.031596	0.0971982	PHYH	phytanoyl-CoA 2-hydroxylase [Source:HGNC Symbol;Acc:HGNC:8940]
ENSG00000175643	562.97162	567.933732	420.377806	455.628834	0.368554464	0.0316042	0.0972055	RMI2	RecQ mediated genome instability 2 [Source:HGNC Symbol;Acc:HGNC:28349]
ENSG00000105699	1360.18668	1167.7639	960.863558	1080.52165	0.308026036	0.0316247	0.0972411	LSR	lipolysis stimulated lipoprotein receptor [Source:HGNC Symbol;Acc:HGNC:29572]
ENSG00000179833	815.915162	995.877557	759.630071	665.345844	0.347053556	0.0316272	0.0972411	SERTAD2	SERTA domain containing 2 [Source:HGNC Symbol;Acc:HGNC:30784]
ENSG00000110536	93.5005312	98.3473389	146.447406	156.489342	-0.658613509	0.0316349	0.0972471	PTPMT1	protein tyrosine phosphatase, mitochondrial 1 [Source:HGNC Symbol;Acc:HGNC:26965]
ENSG00000073008	1891.66338	1662.15863	2142.97859	2117.39661	-0.261972233	0.0316411	0.0972483	PVR	poliovirus receptor [Source:HGNC Symbol;Acc:HGNC:9705]
ENSG00000128791	998.97936	1016.25583	833.380564	803.737779	0.299836851	0.0316579	0.0972658	TWSG1	twisted gastrulation BMP signaling modulator 1 [Source:HGNC Symbol;Acc:HGNC:12429]
ENSG00000165704	733.241008	945.37487	1192.65082	964.485335	-0.361121214	0.0316583	0.0972658	HPRT1	hypoxanthine phosphoribosyltransferase 1 [Source:HGNC Symbol;Acc:HGNC:5157]
ENSG00000138413	3949.65928	4352.97726	3558.98805	3516.21972	0.230944842	0.0316732	0.0972814	IDH1	isocitrate dehydrogenase (NADP(+)) 1, cytosolic [Source:HGNC Symbol;Acc:HGNC:5382]
ENSG00000182220	2126.89103	2134.40306	1780.5476	1811.8698	0.246351961	0.0316749	0.0972814	ATP6AP2	ATPase H+ transporting accessory protein 2 [Source:HGNC Symbol;Acc:HGNC:18305]
ENSG00000184304	33.463348	51.3886996	13.69652	19.1619603	1.371069419	0.0316878	0.0972979	PRKD1	protein kinase D1 [Source:HGNC Symbol;Acc:HGNC:9407]
ENSG00000125037	1647.57778	1631.14821	1327.50886	1403.08132	0.263913982	0.0316918	0.0972979	EMC3	ER membrane protein complex subunit 3 [Source:HGNC Symbol;Acc:HGNC:23999]
ENSG00000103145	998.97936	1049.92429	769.112277	881.450174	0.312129938	0.0317752	0.0975279	HCFC1R1	host cell factor C1 regulator 1 [Source:HGNC Symbol;Acc:HGNC:21198]
ENSG00000140323	143.695553	97.4613268	50.5717662	84.0997147	0.839280195	0.0317811	0.0975279	DISP2	dispatched RND transporter family member 2 [Source:HGNC Symbol;Acc:HGNC:19712]
ENSG00000179855	15.7474579	7.97410856	37.9288246	30.8720472	-1.543567097	0.0317923	0.0975279	GIPC3	GIPC PDZ domain containing family member 3 [Source:HGNC Symbol;Acc:HGNC:18183]
ENSG00000142208	3288.26605	2944.21808	3639.06001	3705.71021	-0.237120741	0.0317929	0.0975279	AKT1	AKT serine/threonine kinase 1 [Source:HGNC Symbol;Acc:HGNC:391]
ENSG00000181291	12.7948095	14.176193	0	3.19366005	3.082369361	0.0317955	0.0975279	TMEM132E	transmembrane protein 132E [Source:HGNC Symbol;Acc:HGNC:26991]
ENSG00000127334	714.540902	755.768289	577.360997	589.762556	0.333412851	0.0318565	0.0976973	DYRK2	dual specificity tyrosine phosphorylation regulated kinase 2 [Source:HGNC Symbol;Acc:HGNC:3093]
ENSG00000234664	79.7215056	25.6943498	125.375837	89.4224814	-1.033016873	0.0318716	0.0977256	HMGN2P5	high mobility group nucleosomal binding domain 2 pseudogene 5 [Source:HGNC Symbol;Acc:HGNC:33568]
ENSG00000108528	1650.53043	1518.62467	1787.92265	2041.81333	-0.27334962	0.0319038	0.0978065	SLC25A11	solute carrier family 25 member 11 [Source:HGNC Symbol;Acc:HGNC:10981]
ENSG00000084754	4769.51131	4767.6309	3917.20472	4250.76153	0.22359457	0.0319193	0.0978355	HADHA	hydroxyacyl-CoA dehydrogenase trifunctional multienzyme complex subunit alpha [Source:HGNC Symbol;Acc:HGNC:4801]
ENSG00000174500	26.5738352	31.0104222	8.4286277	9.58098015	1.677826367	0.0319248	0.0978355	GCSAM	germinal center associated signaling and motility [Source:HGNC Symbol;Acc:HGNC:20253]
ENSG00000155085	59.0529671	86.8291821	45.3038739	24.4847271	1.064890775	0.0319469	0.0978854	AK9	adenylate kinase 9 [Source:HGNC Symbol;Acc:HGNC:33814]
ENSG00000043514	431.08666	443.892043	531.003545	602.537196	-0.37329416	0.031983	0.0979783	TRIT1	tRNA isopentenyltransferase 1 [Source:HGNC Symbol;Acc:HGNC:20286]
ENSG00000213676	1792.25755	1549.6351	1974.40604	2051.39431	-0.26900666	0.0320082	0.0980379	ATF6B	activating transcription factor 6 beta [Source:HGNC Symbol;Acc:HGNC:2349]
ENSG00000131238	4284.29276	4321.96684	4871.74681	5137.53447	-0.217857197	0.0320594	0.0981767	PPT1	palmitoyl-protein thioesterase 1 [Source:HGNC Symbol;Acc:HGNC:9325]
ENSG00000100068	153.537714	92.1452544	60.0539724	78.7769479	0.821512037	0.03209	0.0982442	LRP5L	LDL receptor related protein 5 like [Source:HGNC Symbol;Acc:HGNC:25323]
ENSG00000232024	264.754136	280.865824	336.091529	409.85304	-0.450716188	0.032093	0.0982442	LSM12P1	LSM12 pseudogene 1 [Source:HGNC Symbol;Acc:HGNC:32453]
ENSG00000213341	766.704356	993.219521	732.237031	634.473797	0.365500002	0.0321711	0.0984473	CHUK	conserved helix-loop-helix ubiquitous kinase [Source:HGNC Symbol;Acc:HGNC:1974]
ENSG00000232112	561.003187	567.04772	693.254628	730.283598	-0.335567772	0.032175	0.0984473	TMA7	translation machinery associated 7 homolog [Source:HGNC Symbol;Acc:HGNC:26932]
ENSG00000231439	10.8263773	28.352386	47.4110308	51.0985608	-1.321972433	0.0321768	0.0984473	WASIR2	WASH and IL9R antisense RNA 2 [Source:HGNC Symbol;Acc:HGNC:38609]
ENSG00000135299	76.7688572	57.590784	32.6609323	33.0011539	1.031502706	0.0321929	0.0984786	ANKRD6	ankyrin repeat domain 6 [Source:HGNC Symbol;Acc:HGNC:17280]
ENSG00000272031	90.5478829	102.777399	45.3038739	61.7440943	0.853701528	0.0322012	0.0984862	ANKRD34A	ankyrin repeat domain 34A [Source:HGNC Symbol;Acc:HGNC:27639]
ENSG00000084676	601.356048	550.21349	480.431779	402.401166	0.382923206	0.0322087	0.0984911	NCOA1	nuclear receptor coactivator 1 [Source:HGNC Symbol;Acc:HGNC:7668]
ENSG00000032742	159.443011	189.606581	118.000788	108.584442	0.624212561	0.0322396	0.0985679	IFT88	intraflagellar transport 88 [Source:HGNC Symbol;Acc:HGNC:20606]
ENSG00000112640	796.23084	684.001312	875.523702	984.711849	-0.330249798	0.0322706	0.098645	PPP2R5D	protein phosphatase 2 regulatory subunit B'delta [Source:HGNC Symbol;Acc:HGNC:9312]
ENSG00000189060	20463.8215	19335.4412	16093.411	18144.2473	0.217142745	0.0322913	0.0986902	H1F0	H1 histone family member 0 [Source:HGNC Symbol;Acc:HGNC:4714]
ENSG00000111481	2811.90545	2890.17135	3299.80774	3375.69867	-0.227322023	0.0323079	0.098723	COPZ1	coatomer protein complex subunit zeta 1 [Source:HGNC Symbol;Acc:HGNC:2243]
ENSG00000124571	1584.58795	1446.8577	1729.97584	1927.90612	-0.271246789	0.032329	0.0987699	XPO5	exportin 5 [Source:HGNC Symbol;Acc:HGNC:17675]
ENSG00000137970	833.631052	948.032906	990.363755	1263.62483	-0.338761974	0.0323475	0.0988084	RPL7P9	ribosomal protein L7 pseudogene 9 [Source:HGNC Symbol;Acc:HGNC:37028]

ENSG00000180806	220.46441	196.694678	254.965988	340.657072	-0.514348088	0.0323764	0.0988789	HOXC9	homeobox C9 [Source:HGNC Symbol;Acc:HGNC:5130]
ENSG00000108187	74.800425	61.1348323	103.250689	128.810955	-0.77307775	0.032384	0.0988841	PBLD	phenazine biosynthesis like protein domain containing [Source:HGNC Symbol;Acc:HGNC:23301]
ENSG00000198515	7.87372895	4.43006031	0	0	4.97899711	0.0323883	1	CNGA1	cyclic nucleotide gated channel alpha 1 [Source:HGNC Symbol;Acc:HGNC:2148]
ENSG00000211460	1477.30839	1776.45418	2061.85305	1899.16318	-0.2833074	0.0323916	0.0988896	TSN	translin [Source:HGNC Symbol;Acc:HGNC:12379]
ENSG00000177054	591.513887	699.949529	527.84281	471.597134	0.370408727	0.0324035	0.0989078	ZDHHC13	zinc finger DHHC-type containing 13 [Source:HGNC Symbol;Acc:HGNC:18413]
ENSG00000138346	413.37077	532.493249	777.540905	518.437482	-0.45358535	0.0324096	0.0989087	DNA2	DNA replication helicase/nuclease 2 [Source:HGNC Symbol;Acc:HGNC:2939]
ENSG00000144290	5.90529671	14.176193	1.05357846	0	4.249044279	0.0324284	1	SLC4A10	solute carrier family 4 member 10 [Source:HGNC Symbol;Acc:HGNC:13811]
ENSG00000187325	374.002125	460.726272	657.43296	473.726241	-0.437669705	0.03246	0.0990445	TAF9B	TATA-box binding protein associated factor 9b [Source:HGNC Symbol;Acc:HGNC:17306]
ENSG00000003096	138.774473	152.394075	103.250689	76.6478412	0.694900435	0.0324781	0.0990821	KLHL13	kelch like family member 13 [Source:HGNC Symbol;Acc:HGNC:22931]
ENSG00000087206	671.235393	722.09983	796.505318	965.549889	-0.338338143	0.0325166	0.0991677	UIMC1	ubiquitin interaction motif containing 1 [Source:HGNC Symbol;Acc:HGNC:30298]
ENSG00000136758	2303.06572	2558.80283	2878.37636	2875.3586	-0.242741081	0.0325179	0.0991677	YME1L1	YME1 like 1 ATPase [Source:HGNC Symbol;Acc:HGNC:12843]
ENSG00000232934	8.85794506	3.54404825	0	0	4.989490481	0.0325425	1	AL157786.1	novel transcript, antisense to ACSL5
ENSG00000116962	239.164517	188.720569	155.929612	128.810955	0.586305421	0.0325491	0.0992449	NID1	nidogen 1 [Source:HGNC Symbol;Acc:HGNC:7821]
ENSG00000138698	759.814843	937.400761	692.20105	642.990224	0.346792833	0.0325777	0.0993141	RAP1GDS1	Rap1 GTPase-GDP dissociation stimulator 1 [Source:HGNC Symbol;Acc:HGNC:9859]
ENSG00000168505	91.532099	62.9068564	46.3574523	30.8720472	0.997691612	0.0326037	0.0993602	GBX2	gastrulation brain homeobox 2 [Source:HGNC Symbol;Acc:HGNC:4186]
ENSG00000110435	536.397784	545.78343	649.004333	726.025385	-0.345379239	0.0326046	0.0993602	PDHX	pyruvate dehydrogenase complex component X [Source:HGNC Symbol;Acc:HGNC:21350]
ENSG00000103507	1491.08742	1296.23565	1735.24373	1641.54127	-0.277224692	0.0326163	0.0993756	BCKDK	branched chain ketoacid dehydrogenase kinase [Source:HGNC Symbol;Acc:HGNC:16902]
ENSG00000257093	611.198209	703.493577	542.592908	479.049008	0.364321629	0.0326249	0.0993756	KIAA1147	KIAA1147 [Source:HGNC Symbol;Acc:HGNC:29472]
ENSG00000273559	398.607528	464.27032	523.628496	605.730856	-0.38742829	0.0326273	0.0993756	CWC25	CWC25 spliceosome associated protein homolog [Source:HGNC Symbol;Acc:HGNC:25989]
ENSG00000221953	6.88951283	5.31607237	0	0	4.968466777	0.0326864	1	C1orf229	chromosome 1 open reading frame 229 [Source:HGNC Symbol;Acc:HGNC:33759]
ENSG00000196693	212.590682	209.984859	326.609323	267.202891	-0.490984557	0.0327239	0.0996518	ZNF33B	zinc finger protein 33B [Source:HGNC Symbol;Acc:HGNC:13097]
ENSG00000087903	563.955836	458.954248	350.841628	416.24036	0.414514904	0.0327766	0.0997813	RFX2	regulatory factor X2 [Source:HGNC Symbol;Acc:HGNC:9983]
ENSG00000129946	20.6685385	13.2901809	4.21431385	2.1291067	2.417454352	0.0327782	0.0997813	SHC2	SHC adaptor protein 2 [Source:HGNC Symbol;Acc:HGNC:29869]
ENSG00000135838	124.011231	95.6893027	56.893237	69.1959678	0.79994978	0.0327884	0.0997835	NPL	N-acetylneuraminate pyruvate lyase [Source:HGNC Symbol;Acc:HGNC:16781]
ENSG00000073111	6119.85582	5334.67862	6894.61746	6551.26132	-0.23140074	0.0327907	0.0997835	MCM2	minichromosome maintenance complex component 2 [Source:HGNC Symbol;Acc:HGNC:6944]
ENSG00000246465	71.8477766	55.8187599	32.6609323	28.7429405	1.05454896	0.032798	0.0997875	AC138904.1	novel transcript LOC100506705
ENSG00000157870	245.069813	259.601534	312.912803	382.174653	-0.461393436	0.0328594	0.0999565	PRXL2B	peroxiredoxin like 2B [Source:HGNC Symbol;Acc:HGNC:28390]
ENSG00000134321	0.98421612	2.65803619	10.5357846	15.9683003	-2.846685995	0.0328796	0.1	RSAD2	radical S-adenosyl methionine domain containing 2 [Source:HGNC Symbol;Acc:HGNC:30908]
ENSG00000178149	452.739414	432.373886	572.093105	565.277829	-0.362038856	0.0328908	0.1000158	DALRD3	DALR anticodon binding domain containing 3 [Source:HGNC Symbol;Acc:HGNC:25536]
ENSG00000135720	2157.40173	2176.93164	1839.548	1821.45078	0.243593171	0.0328967	0.1000158	DYNC1LI2	dynein cytoplasmic 1 light intermediate chain 2 [Source:HGNC Symbol;Acc:HGNC:2966]
ENSG00000165660	259.833055	314.534282	417.217071	365.141799	-0.444814283	0.0329654	0.1001975	ABRAXAS2	abraxas 2, BRISC complex subunit [Source:HGNC Symbol;Acc:HGNC:28975]
ENSG00000122735	15.7474579	22.1503015	1.05357846	6.3873201	2.352086974	0.0329683	0.1001975	DNAI1	dynein axonemal intermediate chain 1 [Source:HGNC Symbol;Acc:HGNC:2954]
ENSG00000155657	230.306572	239.223257	155.929612	169.263983	0.5301836	0.0329916	0.1002503	TTN	titin [Source:HGNC Symbol;Acc:HGNC:12403]
ENSG00000206560	1348.37608	1531.91485	1849.0302	1648.99314	-0.279979668	0.0330167	0.1003086	ANKRD28	ankyrin repeat domain 28 [Source:HGNC Symbol;Acc:HGNC:29024]
ENSG00000183287	24.605403	26.5803619	7.37504924	7.45187345	1.787909637	0.0330538	0.1003919	CCBE1	collagen and calcium binding EGF domains 1 [Source:HGNC Symbol;Acc:HGNC:29426]
ENSG00000197362	195.859008	209.098847	280.251871	286.364851	-0.484136041	0.033056	0.1003919	ZNF786	zinc finger protein 786 [Source:HGNC Symbol;Acc:HGNC:21806]
ENSG00000178726	73.8162089	63.7928684	35.8216677	33.0011539	0.99875887	0.0330654	0.1004023	THBD	thrombomodulin [Source:HGNC Symbol;Acc:HGNC:11784]
ENSG00000214776	72.8319927	85.94317	34.7680893	47.9049008	0.94264259	0.0331535	0.1006518	AC092821.1	ovostatin
ENSG00000103274	251.959326	210.870871	325.555745	315.107792	-0.470245269	0.0331771	0.1007052	NUBP1	nucleotide binding protein 1 [Source:HGNC Symbol;Acc:HGNC:8041]
ENSG00000097021	2091.45925	1979.35095	1688.88628	1734.15741	0.249898736	0.0332593	0.1009367	ACOT7	acyl-CoA thioesterase 7 [Source:HGNC Symbol;Acc:HGNC:24157]
ENSG00000248019	40.3528608	57.590784	16.8572554	24.4847271	1.246443249	0.0332869	0.1010022	FAM13A-AS1	FAM13A antisense RNA 1 [Source:HGNC Symbol;Acc:HGNC:19370]
ENSG00000104853	2414.28214	2147.69324	2658.17846	2757.19318	-0.247672163	0.033305	0.101039	CLPTM1	CLPTM1, transmembrane protein [Source:HGNC Symbol;Acc:HGNC:2087]
ENSG00000101146	1387.74473	1348.51036	1635.15377	1639.41216	-0.259204051	0.0333213	0.1010703	RAE1	ribonucleic acid export 1 [Source:HGNC Symbol;Acc:HGNC:9828]
ENSG00000065135	1565.88784	1744.55775	2215.67551	1824.64444	-0.287224436	0.0333379	0.1010843	GNAI3	G protein subunit alpha i3 [Source:HGNC Symbol;Acc:HGNC:4387]
ENSG00000227081	607.261345	714.125722	831.273407	832.48072	-0.331650667	0.033338	0.1010843	AC005912.1	ribosomal protein S27 (RPS27) pseudogene
ENSG00000125482	542.303081	544.011406	617.396979	784.575819	-0.367908018	0.0333438	0.1010843	TTF1	transcription termination factor 1 [Source:HGNC Symbol;Acc:HGNC:12397]
ENSG00000007372	5.90529671	6.20208443	0	0	4.957875551	0.0333744	1	PAX6	paired box 6 [Source:HGNC Symbol;Acc:HGNC:8620]
ENSG00000101489	5.90529671	6.20208443	0	0	4.957875551	0.0333744	1	CELF4	CUGBP Elav-like family member 4 [Source:HGNC Symbol;Acc:HGNC:14015]
ENSG00000232104	5.90529671	6.20208443	0	0	4.957875551	0.0333744	1	RFX3-AS1	RFX3 antisense RNA 1 [Source:HGNC Symbol;Acc:HGNC:51197]
ENSG00000226648	5.90529671	6.20208443	0	0	4.957875551	0.0333744	1	PLCG1-AS1	PLCG1 antisense RNA 1 [Source:HGNC Symbol;Acc:HGNC:40450]
ENSG00000250007	5.90529671	6.20208443	0	0	4.957875551	0.0333744	1	AC087457.1	uncharacterized LOC101928174 [Source:NCBI gene;Acc:101928174]
ENSG00000189186	5.90529671	6.20208443	0	0	4.957875551	0.0333744	1	DCAF8L2	DDB1 and CUL4 associated factor 8 like 2 [Source:HGNC Symbol;Acc:HGNC:31811]
ENSG00000109079	2931.97982	2704.10881	2351.58713	2423.98798	0.238858628	0.0334185	0.1012924	TNFAIP1	TNF alpha induced protein 1 [Source:HGNC Symbol;Acc:HGNC:11894]

ENSG00000080603	116.137502	153.280087	230.733683	179.909516	-0.606355339	0.0334514	0.101374	SRCAP	Snf2 related CREBBP activator protein [Source:HGNC Symbol;Acc:HGNC:16974]
ENSG00000166924	189.953711	134.673833	108.518582	94.7452482	0.673705394	0.0334688	0.1013812	NYAP1	neuronal tyrosine phosphorylated phosphoinositide-3-kinase adaptor 1 [Source:HGNC Symbol;Acc:HGNC:22009]
ENSG00000246982	16.731674	8.86012062	1.05357846	2.1291067	3.006123859	0.0334694	0.1013812	Z84485.1	novel transcript, antisense to BRPF3
ENSG00000185046	8.85794506	13.2901809	1.05357846	1.06455335	3.388009325	0.0334718	0.1013812	ANKS1B	ankyrin repeat and sterile alpha motif domain containing 1B [Source:HGNC Symbol;Acc:HGNC:24600]
ENSG00000182489	32.4791319	21.2642895	8.4286277	7.45187345	1.756689034	0.0334851	0.101394	XKRX	XK related X-linked [Source:HGNC Symbol;Acc:HGNC:29845]
ENSG00000183914	402.544392	372.125066	553.128693	465.209814	-0.39507509	0.033488	0.101394	DNAH2	dynein axonemal heavy chain 2 [Source:HGNC Symbol;Acc:HGNC:2948]
ENSG00000180263	487.186979	551.099502	439.342219	340.657072	0.412996496	0.0335147	0.1014464	FGD6	FYVE, RhoGEF and PH domain containing 6 [Source:HGNC Symbol;Acc:HGNC:21740]
ENSG00000085733	4865.96449	4373.35554	5246.82074	5571.87224	-0.227807831	0.033522	0.1014464	CTTN	cortactin [Source:HGNC Symbol;Acc:HGNC:3338]
ENSG00000161677	447.818334	376.555126	535.217859	539.728549	-0.383831277	0.0335233	0.1014464	JOSD2	Josephin domain containing 2 [Source:HGNC Symbol;Acc:HGNC:28853]
ENSG00000005882	532.46092	477.560501	377.18109	396.013846	0.385014566	0.0335552	0.1015248	PDK2	pyruvate dehydrogenase kinase 2 [Source:HGNC Symbol;Acc:HGNC:8810]
ENSG00000165494	852.331158	995.877557	1164.2042	1117.78102	-0.303572228	0.0336114	0.101655	PCF11	PCF11, cleavage and polyadenylation factor subunit [Source:HGNC Symbol;Acc:HGNC:30097]
ENSG00000173540	609.229777	505.026875	686.933157	737.735472	-0.355394272	0.0336155	0.101655	GMPPB	GDP-mannose pyrophosphorylase B [Source:HGNC Symbol;Acc:HGNC:22932]
ENSG00000115368	818.86781	993.219521	1135.75758	1115.65191	-0.312461939	0.0336163	0.101655	WDR75	WD repeat domain 75 [Source:HGNC Symbol;Acc:HGNC:25725]
ENSG00000228474	1408.41327	1307.7538	1658.3325	1603.21735	-0.264256042	0.0336245	0.1016615	OST4	oligosaccharyltransferase complex subunit 4, non-catalytic [Source:HGNC Symbol;Acc:HGNC:32483]
ENSG00000165996	292.312187	318.07833	424.59212	390.69108	-0.417091312	0.0337243	0.1019452	HACD1	3-hydroxyacyl-CoA dehydratase 1 [Source:HGNC Symbol;Acc:HGNC:9639]
ENSG00000196295	316.91759	297.700053	226.519369	218.233437	0.466334614	0.0337369	0.1019651	GARS-DT	GARS divergent transcript [Source:HGNC Symbol;Acc:HGNC:48951]
ENSG00000143624	1967.44802	1917.3301	2207.24688	2402.69691	-0.246966832	0.0337722	0.1020533	INTS3	integrator complex subunit 3 [Source:HGNC Symbol;Acc:HGNC:26153]
ENSG00000213062	37.4002125	27.4663739	14.7500985	6.3873201	1.615351303	0.0338233	0.1021897	AL021068.1	novel transcript
ENSG00000112697	1900.52132	2290.34118	1952.28089	1315.78794	0.358970809	0.0338732	0.1023221	TMEM30A	transmembrane protein 30A [Source:HGNC Symbol;Acc:HGNC:16667]
ENSG00000159167	1535.37714	1604.56784	1320.13381	1297.69053	0.262501044	0.0339132	0.1024245	STC1	stanniocalcin 1 [Source:HGNC Symbol;Acc:HGNC:11373]
ENSG0000038358	1505.85066	1490.27229	1818.47643	1753.31937	-0.253600322	0.0339956	0.1026551	EDC4	enhancer of mRNA decapping 4 [Source:HGNC Symbol;Acc:HGNC:17157]
ENSG00000166949	1448.76613	1294.46362	1618.29652	1687.31706	-0.269403046	0.0340853	0.1029075	SMAD3	SMAD family member 3 [Source:HGNC Symbol;Acc:HGNC:6769]
ENSG00000159399	2518.60905	2023.65155	1875.36966	1862.96836	0.280604591	0.0341359	0.1030321	HK2	hexokinase 2 [Source:HGNC Symbol;Acc:HGNC:4923]
ENSG00000163293	82.6741539	92.1452544	52.6789231	42.582134	0.87628008	0.0341387	0.1030321	NIPAL1	NIPA like domain containing 1 [Source:HGNC Symbol;Acc:HGNC:27194]
ENSG00000174799	123.027015	143.533954	194.912016	202.265137	-0.574165799	0.0341523	0.1030547	CEP135	centrosomal protein 135 [Source:HGNC Symbol;Acc:HGNC:29086]
ENSG00000138180	1216.49112	1631.14821	1736.29731	1808.67614	-0.315322125	0.0341981	0.1031743	CEP55	centrosomal protein 55 [Source:HGNC Symbol;Acc:HGNC:1161]
ENSG00000121073	1000.94779	1033.97608	1198.97229	1273.20581	-0.280651669	0.0342044	0.103175	SLC35B1	solute carrier family 35 member B1 [Source:HGNC Symbol;Acc:HGNC:20798]
ENSG00000166925	1197.79102	1008.28173	1274.82994	1447.79256	-0.304034564	0.0342234	0.1032138	TSC22D4	TSC22 domain family member 4 [Source:HGNC Symbol;Acc:HGNC:21696]
ENSG00000146085	622.024587	704.379589	523.628496	518.437482	0.348589425	0.0342396	0.1032266	MUT	methylmalonyl-CoA mutase [Source:HGNC Symbol;Acc:HGNC:7526]
ENSG00000176244	40.3528608	71.766977	26.3394616	23.4201737	1.174494016	0.0342399	0.1032266	ACBD7	acyl-CoA binding domain containing 7 [Source:HGNC Symbol;Acc:HGNC:17715]
ENSG00000073670	137.790257	125.813713	93.7684831	64.9377544	0.731262523	0.0342795	0.1033275	ADAM11	ADAM metallopeptidase domain 11 [Source:HGNC Symbol;Acc:HGNC:189]
ENSG00000164366	744.067385	742.478108	830.219828	1046.45594	-0.336143628	0.0342975	0.1033635	CCDC127	coiled-coil domain containing 127 [Source:HGNC Symbol;Acc:HGNC:30520]
ENSG00000122971	336.601912	265.803619	418.27065	396.013846	-0.436162976	0.034393	0.1036329	ACADS	acyl-CoA dehydrogenase short chain [Source:HGNC Symbol;Acc:HGNC:90]
ENSG00000198768	44.2897253	46.0726272	71.6433354	96.8743549	-0.898596098	0.0344663	0.1038351	APCDD1L	APC down-regulated 1 like [Source:HGNC Symbol;Acc:HGNC:26892]
ENSG00000187630	149.60085	140.875918	200.179908	225.68531	-0.552341769	0.0345662	0.1041176	DHRS4L2	dehydrogenase/reductase 4 like 2 [Source:HGNC Symbol;Acc:HGNC:19731]
ENSG00000157538	1095.43254	1164.21985	916.613262	935.742395	0.286968658	0.0345995	0.1041992	VPS26C	VPS26 endosomal protein sorting factor C [Source:HGNC Symbol;Acc:HGNC:3044]
ENSG00000114784	696.825012	820.447169	577.360997	619.57005	0.342790135	0.0346098	0.1042117	EIF1B	eukaryotic translation initiation factor 1B [Source:HGNC Symbol;Acc:HGNC:30792]
ENSG00000089327	1545.21931	1401.67108	1701.52922	1840.61274	-0.265712917	0.0346755	0.104391	FXYD5	FXYD domain containing ion transport regulator 5 [Source:HGNC Symbol;Acc:HGNC:4029]
ENSG00000151422	682.06177	847.027531	1055.68562	887.837494	-0.345283503	0.034749	0.1045802	FER	FER tyrosine kinase [Source:HGNC Symbol;Acc:HGNC:3655]
ENSG00000099204	2299.12885	2477.28972	2144.03217	1837.41908	0.262744097	0.0347507	0.1045802	ABLIM1	actin binding LIM protein 1 [Source:HGNC Symbol;Acc:HGNC:78]
ENSG00000117899	1890.67916	1973.14886	2307.33683	2257.91766	-0.240545254	0.0347681	0.104614	MESD	mesoderm development LRP chaperone [Source:HGNC Symbol;Acc:HGNC:13520]
ENSG00000213862	186.016846	108.979484	223.358634	228.87897	-0.619354213	0.0348106	0.104723	AC044787.1	ribosomal protein L7a (RPL7A) pseudogene
ENSG00000146858	148.616634	164.798243	236.001576	218.233437	-0.534670132	0.0348362	0.1047721	ZC3HAV1L	zinc finger CCCH-type containing, antiviral 1 like [Source:HGNC Symbol;Acc:HGNC:22423]
ENSG00000004779	1721.39399	1891.63575	2151.40722	2145.075	-0.249673538	0.0348393	0.1047721	NDUFAB1	NADH:ubiquinone oxidoreductase subunit AB1 [Source:HGNC Symbol;Acc:HGNC:7694]
ENSG00000150456	108.263773	91.2592424	152.768877	158.618449	-0.643451114	0.0348848	0.1048902	EEF1AKMT1	EEF1A lysine methyltransferase 1 [Source:HGNC Symbol;Acc:HGNC:27351]
ENSG00000170581	957.642283	916.136472	741.719237	777.123946	0.302828996	0.0348931	0.1048965	STAT2	signal transducer and activator of transcription 2 [Source:HGNC Symbol;Acc:HGNC:11363]
ENSG00000170903	306.091213	357.948873	240.215889	243.782717	0.457041941	0.0349329	0.1049974	MSANTD4	Myb/SANT DNA binding domain containing 4 with coiled-coils [Source:HGNC Symbol;Acc:HGNC:29383]
ENSG00000089818	630.882532	631.7266	461.467366	526.953908	0.353292487	0.0349884	0.1051456	NECAP1	NECAP endocytosis associated 1 [Source:HGNC Symbol;Acc:HGNC:24539]
ENSG00000257671	350.380938	373.011078	223.358634	300.204045	0.466866206	0.0350136	0.1051659	KRT7-AS	KRT7 antisense RNA 1 [Source:HGNC Symbol;Acc:HGNC:52643]
ENSG00000144161	175.190469	183.404497	261.287459	246.976377	-0.502930602	0.0350149	0.1051659	ZC3H8	zinc finger CCCH-type containing 8 [Source:HGNC Symbol;Acc:HGNC:30941]
ENSG00000108511	140.742905	132.015797	194.912016	207.587903	-0.561802471	0.0350168	0.1051659	HOXB6	homeobox B6 [Source:HGNC Symbol;Acc:HGNC:5117]

ENSG00000149269	1172.2014	1286.48951	1015.64964	1004.93836	0.283423922	0.03502	0.1051659	PAK1	p21 (RAC1) activated kinase 1 [Source:HGNC Symbol;Acc:HGNC:8590]
ENSG00000119865	176.174685	194.922654	262.341037	261.880124	-0.497672773	0.0350547	0.1052512	CNRIP1	cannabinoid receptor interacting protein 1 [Source:HGNC Symbol;Acc:HGNC:24546]
ENSG00000026559	301.170132	256.057486	189.644123	205.458797	0.495289415	0.0350681	0.1052729	KCNG1	potassium voltage-gated channel modifier subfamily G member 1 [Source:HGNC Symbol;Acc:HGNC:6248]
ENSG00000155229	1521.59812	1372.43268	1699.42206	1773.54588	-0.263411248	0.0351129	0.1053886	MMS19	MMS19 homolog, cytosolic iron-sulfur assembly component [Source:HGNC Symbol;Acc:HGNC:13824]
ENSG00000273443	47.2423737	31.0104222	12.6429415	17.0328536	1.397185276	0.035201	0.1056343	AL645608.8	novel transcript
ENSG00000101266	2679.03627	3000.92285	3231.32514	3482.15401	-0.240941643	0.0352101	0.1056428	CSNK2A1	casein kinase 2 alpha 1 [Source:HGNC Symbol;Acc:HGNC:2457]
ENSG00000164985	863.157536	1111.94514	1162.09704	1320.04615	-0.328856799	0.0352442	0.1057262	PSIP1	PC4 and SFRS1 interacting protein 1 [Source:HGNC Symbol;Acc:HGNC:9527]
ENSG00000141577	736.193656	629.068564	820.737622	893.160261	-0.328761203	0.0352622	0.1057616	CEP131	centrosomal protein 131 [Source:HGNC Symbol;Acc:HGNC:29511]
ENSG00000166974	728.319927	699.063517	571.039527	565.277829	0.328823474	0.0353427	0.1059841	MAPRE2	microtubule associated protein RP/EB family member 2 [Source:HGNC Symbol;Acc:HGNC:6891]
ENSG00000165030	553.129458	630.840588	422.484963	491.823648	0.373495525	0.0353637	0.1060196	NFIL3	nuclear factor, interleukin 3 regulated [Source:HGNC Symbol;Acc:HGNC:7787]
ENSG00000279312	56.1003187	84.1711459	36.8752462	33.0011539	1.007222943	0.0353671	0.1060196	AL136164.4	TEC
ENSG00000113615	828.709972	1007.39571	765.951542	691.959678	0.333334457	0.0354469	0.10624	SEC24A	SEC24 homolog A, COPII coat complex component [Source:HGNC Symbol;Acc:HGNC:10703]
ENSG00000189337	248.022462	246.311353	154.876034	189.490496	0.521655754	0.0354808	0.1063229	KAZN	kazrin, periplakin interacting protein [Source:HGNC Symbol;Acc:HGNC:29173]
ENSG00000135972	539.350433	552.871527	642.682862	744.122792	-0.344310655	0.0355059	0.1063791	MRPS9	mitochondrial ribosomal protein S9 [Source:HGNC Symbol;Acc:HGNC:14501]
ENSG00000114857	1623.9566	1671.01875	1384.4021	1375.40293	0.25578618	0.0355661	0.1065407	NKTR	natural killer cell triggering receptor [Source:HGNC Symbol;Acc:HGNC:7833]
ENSG00000167578	11.8105934	19.4922654	4.21431385	1.06455335	2.568003595	0.0355762	0.1065521	RAB4B	RAB4B, member RAS oncogene family [Source:HGNC Symbol;Acc:HGNC:9782]
ENSG00000201544	0	0	9.48220616	2.1291067	-5.079346831	0.0355998	1	SNORA16B	small nucleolar RNA, H/ACA box 16B [Source:HGNC Symbol;Acc:HGNC:32606]
ENSG00000064547	244.085597	162.140207	293.948391	291.687618	-0.529996292	0.0356162	0.1066528	LPAR2	lysophosphatidic acid receptor 2 [Source:HGNC Symbol;Acc:HGNC:3168]
ENSG00000106066	855.283807	923.224568	732.237031	703.669765	0.308983705	0.0356439	0.1067171	CPVL	carboxypeptidase, vitellogenic like [Source:HGNC Symbol;Acc:HGNC:14399]
ENSG00000139445	23.6211868	18.6062533	5.26789231	5.32276675	1.994277994	0.0357368	0.1069762	FOXN4	forkhead box N4 [Source:HGNC Symbol;Acc:HGNC:21399]
ENSG00000261474	45.2739414	40.7565548	21.0715692	13.8391936	1.30018915	0.0357947	0.1071306	AC026471.4	novel transcript
ENSG00000134324	1033.42692	921.452544	746.98713	828.222506	0.311183418	0.0358536	0.1072878	LPIN1	lipin 1 [Source:HGNC Symbol;Acc:HGNC:13345]
ENSG00000249572	63.9740477	84.1711459	30.5537754	44.7112407	0.978657646	0.0358884	0.107373	AC034231.1	novel transcript
ENSG00000147586	146.648202	109.865496	182.269074	205.458797	-0.597931567	0.0359008	0.107391	MRPS28	mitochondrial ribosomal protein S28 [Source:HGNC Symbol;Acc:HGNC:14513]
ENSG00000103326	2016.65883	1689.625	2190.38962	2266.43408	-0.266469314	0.03591	0.107399	CAPN15	calpain 15 [Source:HGNC Symbol;Acc:HGNC:11182]
ENSG00000254290	52.1634543	30.1244101	17.9108339	13.8391936	1.371116274	0.0359224	0.107399	AC124067.4	novel transcript
ENSG00000135945	581.671726	647.674817	764.897964	776.059392	-0.32539731	0.0359225	0.107399	REV1	REV1, DNA directed polymerase [Source:HGNC Symbol;Acc:HGNC:14060]
ENSG00000185670	45.2739414	42.528579	61.1075508	107.519888	-0.941724623	0.0359334	0.1074125	ZBTB3	zinc finger and BTB domain containing 3 [Source:HGNC Symbol;Acc:HGNC:22918]
ENSG00000168286	835.599484	800.068892	1004.06027	999.615596	-0.292970674	0.0359547	0.107457	THAP11	THAP domain containing 11 [Source:HGNC Symbol;Acc:HGNC:23194]
ENSG00000165502	2919.18501	2725.3731	3086.98489	3629.06237	-0.250849339	0.0359909	0.1075462	RPL36AL	ribosomal protein L36a like [Source:HGNC Symbol;Acc:HGNC:10346]
ENSG00000071205	289.359539	290.611956	197.019172	220.362544	0.474719648	0.0360208	0.1076167	ARHGAP10	Rho GTPase activating protein 10 [Source:HGNC Symbol;Acc:HGNC:26099]
ENSG00000224677	3.93686447	7.97410856	0	0	4.936417391	0.0360565	1	PDIA3P2	protein disulfide isomerase family A member 3 pseudogene 2 [Source:HGNC Symbol;Acc:HGNC:49403]
ENSG00000121940	394.670663	347.316728	514.14629	458.822494	-0.391773648	0.0360998	0.1078337	CLCC1	chloride channel CLIC like 1 [Source:HGNC Symbol;Acc:HGNC:29675]
ENSG00000117245	50.195022	28.352386	15.8036769	13.8391936	1.403103651	0.0361161	0.1078631	KIF17	kinesin family member 17 [Source:HGNC Symbol;Acc:HGNC:19167]
ENSG00000076053	151.569282	197.58069	286.573342	223.556204	-0.545527947	0.0361725	0.1080124	RBM7	RNA binding motif protein 7 [Source:HGNC Symbol;Acc:HGNC:9904]
ENSG00000180596	118.105934	107.207459	72.6969139	60.679541	0.755749369	0.0361968	0.1080661	HIST1H2BC	histone cluster 1 H2B family member c [Source:HGNC Symbol;Acc:HGNC:4757]
ENSG00000119138	422.228715	456.296212	664.80801	495.017308	-0.400514545	0.0362385	0.1081714	KLF9	Kruppel like factor 9 [Source:HGNC Symbol;Acc:HGNC:1123]
ENSG00000035499	548.208378	558.187599	706.951148	683.443251	-0.329546387	0.0362616	0.1081784	DEPDC1B	DEP domain containing 1B [Source:HGNC Symbol;Acc:HGNC:24902]
ENSG00000164220	31.4949158	25.6943498	11.5893631	6.3873201	1.667923102	0.0362627	0.1081784	F2RL2	coagulation factor II thrombin receptor like 2 [Source:HGNC Symbol;Acc:HGNC:3539]
ENSG00000130520	5204.53483	4842.94193	5520.75114	6248.92817	-0.228316063	0.036266	0.1081784	LSM4	LSM4 homolog, U6 small nuclear RNA and mRNA degradation associated [Source:HGNC Symbol;Acc:HGNC:17259]
ENSG00000159063	837.567917	948.918918	1118.90033	1075.19888	-0.296006218	0.0362665	0.1081784	ALG8	ALG8, alpha-1,3-glucosyltransferase [Source:HGNC Symbol;Acc:HGNC:23161]
ENSG00000096654	266.722568	316.306306	234.947997	173.522196	0.513880307	0.0362824	0.1082069	ZNF184	zinc finger protein 184 [Source:HGNC Symbol;Acc:HGNC:12975]
ENSG00000242086	297.233268	275.549751	201.233486	210.781563	0.474932685	0.0363763	0.1084677	MUC20-OT1	MUC20 overlapping transcript [Source:HGNC Symbol;Acc:HGNC:53807]
ENSG00000205531	4043.15981	3992.37035	4411.33302	5000.20709	-0.228038739	0.0364092	0.1085468	NAP1L4	nucleosome assembly protein 1 like 4 [Source:HGNC Symbol;Acc:HGNC:7640]
ENSG00000105672	11.8105934	13.2901809	25.2858831	44.7112407	-1.478006792	0.0364173	0.1085516	ETV2	ETS variant 2 [Source:HGNC Symbol;Acc:HGNC:3491]
ENSG00000160917	732.256792	678.685239	821.791201	937.871502	-0.318926573	0.0364295	0.1085688	CPSF4	cleavage and polyadenylation specific factor 4 [Source:HGNC Symbol;Acc:HGNC:2327]
ENSG00000152939	82.6741539	74.4250132	115.893631	140.521042	-0.707526615	0.0364386	0.1085768	MARVELD2	MARVEL domain containing 2 [Source:HGNC Symbol;Acc:HGNC:26401]
ENSG00000149970	95.4689635	85.94317	59.0003939	40.4530273	0.866306238	0.0364539	0.1085952	CNKSR2	connector enhancer of kinase suppressor of Ras 2 [Source:HGNC Symbol;Acc:HGNC:19701]
ENSG00000112238	56.1003187	48.7306634	24.2323046	23.4201737	1.13666493	0.036464	0.1085952	PRDM13	PR/SET domain 13 [Source:HGNC Symbol;Acc:HGNC:13998]
ENSG00000110442	1299.16528	1329.01809	1511.88509	1641.54127	-0.262746528	0.036464	0.1085952	COMMD9	COMM domain containing 9 [Source:HGNC Symbol;Acc:HGNC:25014]

ENSG00000276023	1036.37957	1012.71179	834.434142	842.0617	0.289451397	0.036472	0.1085977	DUSP14	dual specificity phosphatase 14 [Source:HGNC Symbol;Acc:HGNC:17007]
ENSG00000113575	2531.40386	3092.1821	3488.39829	3252.21049	-0.26106451	0.0364777	0.1085977	PPP2CA	protein phosphatase 2 catalytic subunit alpha [Source:HGNC Symbol;Acc:HGNC:9299]
ENSG00000171345	1229.28593	1018.02786	1380.18779	1371.14472	-0.292559683	0.0364934	0.1086061	KRT19	keratin 19 [Source:HGNC Symbol;Acc:HGNC:6436]
ENSG00000135506	4965.37032	4705.61006	4060.49139	4264.60072	0.216127725	0.0364934	0.1086061	OS9	OS9, endoplasmic reticulum lectin [Source:HGNC Symbol;Acc:HGNC:16994]
ENSG00000160953	698.793444	649.446841	783.862376	903.805794	-0.324226834	0.0365324	0.108703	MUM1	melanoma associated antigen (mutated) 1 [Source:HGNC Symbol;Acc:HGNC:29641]
ENSG00000188766	50.195022	42.528579	15.8036769	23.4201737	1.240795254	0.0365651	0.1087813	SPRED3	sprouty related EVH1 domain containing 3 [Source:HGNC Symbol;Acc:HGNC:31041]
ENSG00000226478	0	0.88601206	13.69652	4.2582134	-4.276427363	0.0365781	1	UPF3AP1	UPF3A pseudogene 1 [Source:HGNC Symbol;Acc:HGNC:30568]
ENSG00000124486	6906.2445	7631.22189	6615.41916	5758.16907	0.232578944	0.0366531	0.1090237	USP9X	ubiquitin specific peptidase 9 X-linked [Source:HGNC Symbol;Acc:HGNC:12632]
ENSG00000171729	345.459857	345.544704	226.519369	278.912978	0.451318434	0.0366627	0.1090332	TMEM51	transmembrane protein 51 [Source:HGNC Symbol;Acc:HGNC:25488]
ENSG00000163075	23.6211868	15.0622051	6.32147077	2.1291067	2.191213951	0.0366697	0.1090333	CFAP221	cilia and flagella associated protein 221 [Source:HGNC Symbol;Acc:HGNC:33720]
ENSG00000113721	62.9898316	71.766977	126.429415	101.132568	-0.754977545	0.0366756	0.1090333	PDGFRB	platelet derived growth factor receptor beta [Source:HGNC Symbol;Acc:HGNC:8804]
ENSG00000154162	55.1161026	51.3886996	17.9108339	29.8074938	1.158468786	0.0366939	0.1090682	CDH12	cadherin 12 [Source:HGNC Symbol;Acc:HGNC:1751]
ENSG00000179698	8.85794506	22.1503015	2.10715692	3.19366005	2.552611039	0.0367519	0.1092136	WDR97	WD repeat domain 97 [Source:HGNC Symbol;Acc:HGNC:26959]
ENSG00000174099	1004.88466	1053.46834	882.898751	790.963139	0.298426451	0.0367557	0.1092136	MSRB3	methionine sulfoxide reductase B3 [Source:HGNC Symbol;Acc:HGNC:27375]
ENSG00000132026	5.90529671	13.2901809	0	1.06455335	4.181449938	0.0367636	1	RTBDN	retbindin [Source:HGNC Symbol;Acc:HGNC:30310]
ENSG00000170430	401.560176	363.264945	504.664083	488.629988	-0.37769219	0.0367937	0.1093072	MGMT	O-6-methylguanine-DNA methyltransferase [Source:HGNC Symbol;Acc:HGNC:7059]
ENSG00000185305	187.985279	238.337245	142.233092	146.908362	0.561372408	0.0368154	0.1093526	ARL15	ADP ribosylation factor like GTPase 15 [Source:HGNC Symbol;Acc:HGNC:25945]
ENSG00000172345	63.9740477	73.5390011	32.6609323	37.2593673	0.976655421	0.0368252	0.1093625	STARD5	StAR related lipid transfer domain containing 5 [Source:HGNC Symbol;Acc:HGNC:18065]
ENSG00000131174	4219.3345	4833.1958	3764.43585	3938.8474	0.233033462	0.0368707	0.1094784	COX7B	cytochrome c oxidase subunit 7B [Source:HGNC Symbol;Acc:HGNC:2291]
ENSG00000205336	6864.90742	6060.3225	5542.87629	5545.2584	0.221046151	0.0369411	0.109668	ADGRG1	adhesion G protein-coupled receptor G1 [Source:HGNC Symbol;Acc:HGNC:4512]
ENSG00000131143	9353.98999	8363.95386	9999.51319	10557.1756	-0.21448061	0.0369824	0.1097715	COX4I1	cytochrome c oxidase subunit 4I1 [Source:HGNC Symbol;Acc:HGNC:2265]
ENSG00000182957	222.432843	193.150629	136.9652	145.843809	0.554619323	0.0370038	0.1098155	SPATA13	spermatogenesis associated 13 [Source:HGNC Symbol;Acc:HGNC:23222]
ENSG00000030066	3304.01351	3344.69553	4139.50978	3664.19263	-0.23108347	0.0370153	0.1098305	NUP160	nucleoporin 160 [Source:HGNC Symbol;Acc:HGNC:18017]
ENSG00000168476	1486.16634	1282.94547	1610.92147	1737.35107	-0.27442245	0.0370627	0.1099518	REEP4	receptor accessory protein 4 [Source:HGNC Symbol;Acc:HGNC:26176]
ENSG00000162639	542.303081	557.301587	725.915561	660.023077	-0.333781289	0.037095	0.1100285	HENMT1	HEN methyltransferase 1 [Source:HGNC Symbol;Acc:HGNC:26400]
ENSG00000213593	1410.3817	1462.80591	1619.3501	1832.09632	-0.264396172	0.0371047	0.1100377	TMX2	thioredoxin related transmembrane protein 2 [Source:HGNC Symbol;Acc:HGNC:30739]
ENSG00000180900	5054.93398	4329.94095	5449.10781	5571.87224	-0.23203619	0.0371672	0.1102038	SCRIB	scribbled planar cell polarity protein [Source:HGNC Symbol;Acc:HGNC:30377]
ENSG00000011677	6.88951283	23.9223257	4.21431385	0	2.870462884	0.0371834	0.1102169	GABRA3	gamma-aminobutyric acid type A receptor alpha3 subunit [Source:HGNC Symbol;Acc:HGNC:4077]
ENSG00000250208	941.894825	793.866807	598.432567	759.026539	0.35424722	0.0371847	0.1102169	FZD10-DT	FZD10 divergent transcript [Source:HGNC Symbol;Acc:HGNC:48632]
ENSG00000113460	839.536349	1075.61864	1204.24018	1182.71877	-0.316902287	0.0372788	0.1104702	BRIX1	BRX1, biogenesis of ribosomes [Source:HGNC Symbol;Acc:HGNC:24170]
ENSG00000105321	413.37077	293.269992	479.3782	472.661688	-0.431687687	0.0372832	0.1104702	CCDC9	coiled-coil domain containing 9 [Source:HGNC Symbol;Acc:HGNC:24560]
ENSG00000152223	932.052664	956.007015	828.112671	685.572358	0.318839403	0.037295	0.1104859	EPG5	ectopic P-granules autophagy protein 5 homolog [Source:HGNC Symbol;Acc:HGNC:29331]
ENSG00000136816	1539.31401	1381.2928	1216.88312	1204.00984	0.270395944	0.0373857	0.1107351	TOR1B	torsin family 1 member B [Source:HGNC Symbol;Acc:HGNC:11995]
ENSG00000140367	275.580513	358.834885	518.360603	363.012692	-0.473288906	0.0373962	0.1107469	UBE2Q2	ubiquitin conjugating enzyme E2 Q2 [Source:HGNC Symbol;Acc:HGNC:19248]
ENSG00000108591	503.918653	506.798899	624.772028	652.571204	-0.337713681	0.0374895	0.1109956	DRG2	developmentally regulated GTP binding protein 2 [Source:HGNC Symbol;Acc:HGNC:3030]
ENSG00000049860	3473.29868	3798.33371	3113.32436	3106.36668	0.225591052	0.0374934	0.1109956	HEXB	hexosaminidase subunit beta [Source:HGNC Symbol;Acc:HGNC:4879]
ENSG00000007047	646.62999	517.431044	437.235062	457.757941	0.378371842	0.0375146	0.111039	MARK4	microtubule affinity regulating kinase 4 [Source:HGNC Symbol;Acc:HGNC:13538]
ENSG00000114544	1426.12916	1277.62939	1630.93946	1613.86288	-0.263539922	0.037554	0.1111209	SLC41A3	solute carrier family 41 member 3 [Source:HGNC Symbol;Acc:HGNC:31046]
ENSG00000163541	2001.89558	2071.4962	1647.79672	1784.19142	0.247301112	0.0375566	0.1111209	SUCLG1	succinate-CoA ligase alpha subunit [Source:HGNC Symbol;Acc:HGNC:11449]
ENSG00000175093	63.9740477	41.6425669	15.8036769	29.8074938	1.210062694	0.037571	0.1111209	SPSB4	splA/ryanodine receptor domain and SOCS box containing 4 [Source:HGNC Symbol;Acc:HGNC:30630]
ENSG00000154358	1229.28593	960.437075	1471.84911	1262.56027	-0.321210787	0.0375766	0.1111209	OBSCN	obscurin, cytoskeletal calmodulin and titin-interacting RhoGEF [Source:HGNC Symbol;Acc:HGNC:15719]
ENSG00000140497	1383.80786	1229.78474	1603.54642	1542.5378	-0.267934929	0.0375835	0.1111209	SCAMP2	secretory carrier membrane protein 2 [Source:HGNC Symbol;Acc:HGNC:10564]
ENSG00000163064	174.206253	202.01075	116.947209	134.133722	0.584289247	0.0375839	0.1111209	EN1	engrailed homeobox 1 [Source:HGNC Symbol;Acc:HGNC:3342]
ENSG00000276045	1302.11792	1156.24574	990.363755	1027.29398	0.284646335	0.0375883	0.1111209	ORAI1	ORAI calcium release-activated calcium modulator 1 [Source:HGNC Symbol;Acc:HGNC:25896]
ENSG00000111727	249.006678	217.958967	171.733289	152.231129	0.526706472	0.0376122	0.1111618	HCFC2	host cell factor C2 [Source:HGNC Symbol;Acc:HGNC:24972]
ENSG00000107331	3224.292	2875.10914	2714.01812	2422.92343	0.247535606	0.0376153	0.1111618	ABCA2	ATP binding cassette subfamily A member 2 [Source:HGNC Symbol;Acc:HGNC:32]
ENSG00000083838	70.8635605	58.4767961	118.000788	101.132568	-0.762294216	0.0376391	0.1112127	ZNF446	zinc finger protein 446 [Source:HGNC Symbol;Acc:HGNC:21036]
ENSG00000149609	26.5738352	17.7202412	5.26789231	6.3873201	1.92453426	0.0376651	0.1112701	C20orf144	chromosome 20 open reading frame 144 [Source:HGNC Symbol;Acc:HGNC:16137]
ENSG00000265808	1755.84155	1726.83751	1428.65239	1497.82656	0.25100056	0.0377012	0.1113574	SEC22B	SEC22 homolog B, vesicle trafficking protein (gene/pseudogene) [Source:HGNC Symbol;Acc:HGNC:10700]
ENSG00000186472	10.8263773	7.97410856	0	1.06455335	4.147391404	0.0377711	1	PCLO	piccolo presynaptic cytomatrix protein [Source:HGNC Symbol;Acc:HGNC:13406]
ENSG00000138750	483.250114	529.835213	687.986736	603.60175	-0.349982405	0.0377953	0.1116157	NUP54	nucleoporin 54 [Source:HGNC Symbol;Acc:HGNC:17359]

ENSG00000170379	105.311125	96.5753147	52.6789231	63.873201	0.792279566	0.0378244	0.111682	TCAF2	TRPM8 channel associated factor 2 [Source:HGNC Symbol;Acc:HGNC:26878]
ENSG00000114541	134.837608	139.989906	104.304268	60.679541	0.735969869	0.0379143	0.1119279	FRMD4B	FERM domain containing 4B [Source:HGNC Symbol;Acc:HGNC:24886]
ENSG00000130772	381.875854	405.793524	495.181877	522.695695	-0.369519139	0.0379442	0.1119968	MED18	mediator complex subunit 18 [Source:HGNC Symbol;Acc:HGNC:25944]
ENSG00000136932	146.648202	155.052111	231.787262	205.458797	-0.534998153	0.0379536	0.1120048	TRMO	tRNA methyltransferase O [Source:HGNC Symbol;Acc:HGNC:30967]
ENSG00000127995	71.8477766	145.305978	187.536966	169.263983	-0.71265324	0.0379739	0.1120451	CASD1	CAS1 domain containing 1 [Source:HGNC Symbol;Acc:HGNC:16014]
ENSG00000127220	1282.4336	1154.47372	969.292185	1031.5522	0.284124697	0.0380033	0.1121124	ABHD8	abhydrolase domain containing 8 [Source:HGNC Symbol;Acc:HGNC:23759]
ENSG00000164897	939.926393	833.73735	1111.52528	1061.35969	-0.293392489	0.0380867	0.1123389	TMUB1	transmembrane and ubiquitin like domain containing 1 [Source:HGNC Symbol;Acc:HGNC:21709]
ENSG00000137413	339.554561	329.596487	436.181483	439.660534	-0.388519281	0.0381599	0.112535	TAF8	TATA-box binding protein associated factor 8 [Source:HGNC Symbol;Acc:HGNC:17300]
ENSG00000238184	11.8105934	0.88601206	0	0	5.021161427	0.0382093	1	CD81-AS1	CD81 antisense RNA 1 [Source:HGNC Symbol;Acc:HGNC:49384]
ENSG00000171119	98.4216118	93.0312665	49.5181877	59.6149876	0.810767032	0.0382124	0.1126573	NRTN	neurturin [Source:HGNC Symbol;Acc:HGNC:8007]
ENSG00000125967	1277.51252	1189.02819	934.524096	1081.5862	0.290752695	0.0382147	0.1126573	NECAB3	N-terminal EF-hand calcium binding protein 3 [Source:HGNC Symbol;Acc:HGNC:15851]
ENSG00000256973	2.95264835	8.86012062	0	0	4.925493734	0.0383157	1	AC053513.1	novel transcript
ENSG00000121210	448.80255	390.731319	323.448588	309.785025	0.406170261	0.0383366	0.112997	TMEM131L	transmembrane 131 like [Source:HGNC Symbol;Acc:HGNC:29146]
ENSG00000115211	566.908484	474.016453	642.682862	684.507804	-0.351355031	0.0383649	0.1130592	EIF2B4	eukaryotic translation initiation factor 2B subunit delta [Source:HGNC Symbol;Acc:HGNC:3260]
ENSG00000116161	2257.79178	2672.21238	2927.89455	2934.97359	-0.249672018	0.0383711	0.1130592	CACYBP	calcyclin binding protein [Source:HGNC Symbol;Acc:HGNC:30423]
ENSG00000238287	14.7632418	9.74613268	1.05357846	2.1291067	2.944586978	0.0384261	0.1132015	AL603839.3	novel transcript
ENSG00000023909	985.200334	1097.76894	1234.79396	1294.49687	-0.279656953	0.038442	0.1132286	GCLM	glutamate-cysteine ligase modifier subunit [Source:HGNC Symbol;Acc:HGNC:4312]
ENSG00000180422	15.7474579	8.86012062	2.10715692	1.06455335	2.952105145	0.0384833	0.1133305	LINC00304	long intergenic non-protein coding RNA 304 [Source:HGNC Symbol;Acc:HGNC:26713]
ENSG00000111358	718.477766	765.514421	945.059881	884.643834	-0.30186027	0.0384966	0.1133499	GTF2H3	general transcription factor IIH subunit 3 [Source:HGNC Symbol;Acc:HGNC:4657]
ENSG00000181220	697.809228	677.799227	822.844779	878.256514	-0.306511727	0.0385042	0.1133526	ZNF746	zinc finger protein 746 [Source:HGNC Symbol;Acc:HGNC:21948]
ENSG00000117152	8.85794506	9.74613268	0	1.06455335	4.133791342	0.0386328	1	RGS4	regulator of G protein signaling 4 [Source:HGNC Symbol;Acc:HGNC:10000]
ENSG00000197748	12.7948095	11.5181568	2.10715692	1.06455335	2.936633869	0.0386492	0.1137596	CFAP43	cilia and flagella associated protein 43 [Source:HGNC Symbol;Acc:HGNC:26684]
ENSG00000143494	155.506147	168.342292	108.518582	103.261675	0.613140281	0.0386867	0.1138501	VASH2	vasohibin 2 [Source:HGNC Symbol;Acc:HGNC:25723]
ENSG00000145050	2799.11064	2655.37815	2124.01418	2466.57011	0.248703588	0.0388039	0.1141753	MANF	mesencephalic astrocyte derived neurotrophic factor [Source:HGNC Symbol;Acc:HGNC:15461]
ENSG00000166900	734.225224	758.426325	553.128693	635.53835	0.32873612	0.0388877	0.114402	STX3	syntaxin 3 [Source:HGNC Symbol;Acc:HGNC:11438]
ENSG00000149633	12.7948095	14.176193	2.10715692	2.1291067	2.671014227	0.0388973	0.1144101	KIAA1755	KIAA1755 [Source:HGNC Symbol;Acc:HGNC:29372]
ENSG00000125952	1086.57459	1200.54634	875.523702	990.034616	0.294299052	0.0389278	0.1144595	MAX	MYC associated factor X [Source:HGNC Symbol;Acc:HGNC:6913]
ENSG00000117151	70.8635605	73.5390011	42.1431385	33.0011539	0.942318706	0.0389314	0.1144595	CTBS	chitobiase [Source:HGNC Symbol;Acc:HGNC:2496]
ENSG00000278619	217.511762	179.860449	256.019566	303.397705	-0.494552554	0.0389344	0.1144595	MRM1	mitochondrial rRNA methyltransferase 1 [Source:HGNC Symbol;Acc:HGNC:26202]
ENSG00000160785	1165.31188	1026.88798	915.559684	872.933747	0.293184383	0.0389809	0.1145709	SLC25A44	solute carrier family 25 member 44 [Source:HGNC Symbol;Acc:HGNC:29036]
ENSG00000120500	23.6211868	15.9482171	4.21431385	5.32276675	2.051308212	0.0389858	0.1145709	ARR3	arrestin 3 [Source:HGNC Symbol;Acc:HGNC:710]
ENSG00000274211	1133.81697	1068.53055	915.559684	895.289368	0.28214867	0.0389948	0.1145773	SOCS7	suppressor of cytokine signaling 7 [Source:HGNC Symbol;Acc:HGNC:29846]
ENSG00000059122	2035.35893	1743.67174	1446.56323	1669.21965	0.278127201	0.0390026	0.1145802	FLYWCH1	FLYWCH-type zinc finger 1 [Source:HGNC Symbol;Acc:HGNC:25404]
ENSG00000140836	609.229777	616.664395	862.880761	690.895124	-0.341977325	0.039033	0.1146331	ZFHX3	zinc finger homeobox 3 [Source:HGNC Symbol;Acc:HGNC:777]
ENSG00000127947	1043.26909	1201.43236	1408.6344	1320.04615	-0.281204492	0.0390389	0.1146331	PTPN12	protein tyrosine phosphatase, non-receptor type 12 [Source:HGNC Symbol;Acc:HGNC:9645]
ENSG00000179361	342.507209	342.886668	226.519369	276.783871	0.445646256	0.0390409	0.1146331	ARID3B	AT-rich interaction domain 3B [Source:HGNC Symbol;Acc:HGNC:14350]
ENSG00000160796	1376.91835	1142.06955	1035.66763	1016.64845	0.295041867	0.0390723	0.1147053	NBEAL2	neurobeachin like 2 [Source:HGNC Symbol;Acc:HGNC:31928]
ENSG00000170634	80.7057217	77.9690614	116.947209	139.456489	-0.692516558	0.0390849	0.1147077	ACYP2	acylphosphatase 2 [Source:HGNC Symbol;Acc:HGNC:180]
ENSG00000198862	783.43603	979.92934	808.094681	508.856501	0.421447523	0.0390867	0.1147077	LTN1	listerin E3 ubiquitin protein ligase 1 [Source:HGNC Symbol;Acc:HGNC:13082]
ENSG00000135164	994.058279	1153.5877	910.291791	831.416167	0.302695672	0.0391312	0.1148185	DMTF1	cyclin D binding myb like transcription factor 1 [Source:HGNC Symbol;Acc:HGNC:14603]
ENSG00000099624	2319.79739	1907.58397	2446.40919	2632.64044	-0.265190859	0.0391765	0.1149313	ATP5F1D	ATP synthase F1 subunit delta [Source:HGNC Symbol;Acc:HGNC:837]
ENSG00000169660	475.376385	436.803946	537.325016	636.602903	-0.364310785	0.0392235	0.1150493	HEXDC	hexosaminidase D [Source:HGNC Symbol;Acc:HGNC:26307]
ENSG00000116717	594.466535	509.456936	400.359816	451.370621	0.37359705	0.0392379	0.1150714	GADD45A	growth arrest and DNA damage inducible alpha [Source:HGNC Symbol;Acc:HGNC:4095]
ENSG00000130758	730.28836	590.970045	503.610505	526.953908	0.357701352	0.0392485	0.1150827	MAP3K10	mitogen-activated protein kinase kinase kinase 10 [Source:HGNC Symbol;Acc:HGNC:6849]
ENSG00000136891	579.703294	645.016781	778.594484	751.574665	-0.320728431	0.0393283	0.1152967	TEX10	testis expressed 10 [Source:HGNC Symbol;Acc:HGNC:25988]
ENSG00000131470	374.986341	345.544704	440.395797	501.404628	-0.386765979	0.039342	0.1153167	PSMC3IP	PSMC3 interacting protein [Source:HGNC Symbol;Acc:HGNC:17928]
ENSG00000072195	308.059645	299.472077	401.413394	400.27206	-0.40025793	0.0393649	0.1153638	SPEG	SPEG complex locus [Source:HGNC Symbol;Acc:HGNC:16901]
ENSG00000130653	115.153286	66.4509046	51.6253447	45.7757941	0.896223557	0.039381	0.1153911	PNPLA7	patatin like phospholipase domain containing 7 [Source:HGNC Symbol;Acc:HGNC:24768]
ENSG00000181908	31.4949158	17.7202412	10.5357846	2.1291067	1.954403373	0.0394107	0.1154581	AP003774.1	uncharacterized LOC100996455 [Source:NCBI gene;Acc:100996455]
ENSG00000205133	136.80604	200.238726	110.625739	105.390782	0.643524744	0.0394846	0.1156545	TRIQK	triple QxxK/R motif containing [Source:HGNC Symbol;Acc:HGNC:27828]
ENSG00000235947	0.98421612	1.77202412	5.26789231	18.097407	-3.072443467	0.0395401	0.1157928	EGOT	eosinophil granule ontogeny transcript [Source:HGNC Symbol;Acc:HGNC:37129]
ENSG00000159339	17.7158901	16.8342292	3.16073539	4.2582134	2.219631546	0.0395455	0.1157928	PADI4	peptidyl arginine deiminase 4 [Source:HGNC Symbol;Acc:HGNC:18368]

ENSG00000076864	1151.53286	1004.73768	861.827182	895.289368	0.294883641	0.0395732	0.1158538	RAP1GAP	RAP1 GTPase activating protein [Source:HGNC Symbol;Acc:HGNC:9858]
ENSG00000114491	1115.11686	1197.0023	1369.652	1409.46864	-0.265131474	0.0396698	0.1161164	UMPS	uridine monophosphate synthetase [Source:HGNC Symbol;Acc:HGNC:12563]
ENSG00000228451	64.9582638	66.4509046	34.7680893	31.9366005	0.978241749	0.0396959	0.1161726	SDAD1P1	SDA1 domain containing 1 pseudogene 1 [Source:HGNC Symbol;Acc:HGNC:31403]
ENSG00000126882	1156.45394	1018.02786	1327.50886	1306.20696	-0.276908666	0.0397187	0.1162193	FAM78A	family with sequence similarity 78 member A [Source:HGNC Symbol;Acc:HGNC:25465]
ENSG00000230487	115.153286	89.4872182	59.0003939	59.6149876	0.785487447	0.0397882	0.1164026	PSMG3-AS1	PSMG3 antisense RNA 1 (head to head) [Source:HGNC Symbol;Acc:HGNC:22230]
ENSG00000107745	2570.7725	2861.81896	2242.01497	2363.30844	0.238569696	0.0398099	0.1164459	MICU1	mitochondrial calcium uptake 1 [Source:HGNC Symbol;Acc:HGNC:1530]
ENSG00000167363	62.0056154	61.1348323	85.3398554	127.746402	-0.791027626	0.0398183	0.1164504	FN3K	fructosamine 3 kinase [Source:HGNC Symbol;Acc:HGNC:24822]
ENSG00000215241	24.605403	8.86012062	5.26789231	0	2.661150419	0.0398607	0.1165541	LINC02449	long intergenic non-protein coding RNA 2449 [Source:HGNC Symbol;Acc:HGNC:53381]
ENSG00000182378	1387.74473	1298.89368	1497.13499	1749.06115	-0.273108413	0.039876	0.1165785	PLCXD1	phosphatidylinositol specific phospholipase C X domain containing 1 [Source:HGNC Symbol;Acc:HGNC:23148]
ENSG00000154639	436.991956	507.684911	398.252659	311.914132	0.412121865	0.0399183	0.1166602	CXADR	CXADR, Ig-like cell adhesion molecule [Source:HGNC Symbol;Acc:HGNC:2559]
ENSG00000075275	3826.63227	3426.20864	4743.21024	3905.84624	-0.254167328	0.0399243	0.1166602	CELSR1	cadherin EGF LAG seven-pass G-type receptor 1 [Source:HGNC Symbol;Acc:HGNC:1850]
ENSG00000204950	16.731674	17.7202412	4.21431385	3.19366005	2.217258787	0.0399246	0.1166602	LRRC10B	leucine rich repeat containing 10B [Source:HGNC Symbol;Acc:HGNC:37215]
ENSG00000139192	134.837608	124.927701	181.215496	202.265137	-0.562445491	0.0399716	0.1167774	TAPBPL	TAP binding protein like [Source:HGNC Symbol;Acc:HGNC:30683]
ENSG00000162068	49.2108059	31.0104222	16.8572554	14.9037469	1.334371987	0.0399785	0.1167774	NTN3	netrin 3 [Source:HGNC Symbol;Acc:HGNC:8030]
ENSG00000258884	7.87372895	14.176193	0	2.1291067	3.378864154	0.0399926	0.1167983	LINC02321	long intergenic non-protein coding RNA 2321 [Source:HGNC Symbol;Acc:HGNC:53240]
ENSG00000140632	1690.88329	1607.22588	1951.22731	1948.13263	-0.241762029	0.0400606	0.1169767	GLYR1	glyoxylate reductase 1 homolog [Source:HGNC Symbol;Acc:HGNC:24434]
ENSG00000183298	131.88496	120.49764	161.197505	219.29799	-0.592723281	0.0400835	0.1170232	RPSAP19	ribosomal protein SA pseudogene 19 [Source:HGNC Symbol;Acc:HGNC:36508]
ENSG00000137106	1463.52937	1141.18354	1577.20696	1619.18565	-0.295986314	0.0400928	0.1170304	GRHPR	glyoxylate and hydroxypyruvate reductase [Source:HGNC Symbol;Acc:HGNC:4570]
ENSG00000115944	1427.11337	1393.69697	1117.84675	1227.43001	0.266315147	0.0401028	0.1170393	COX7A2L	cytochrome c oxidase subunit 7A2 like [Source:HGNC Symbol;Acc:HGNC:2289]
ENSG00000180879	2459.55608	1997.9572	2588.64228	2765.7096	-0.264871513	0.040149	0.1171537	SSR4	signal sequence receptor subunit 4 [Source:HGNC Symbol;Acc:HGNC:11326]
ENSG00000054392	117.121718	107.207459	54.78608	77.7123946	0.759487482	0.0401634	0.1171576	HHAT	hedgehog acyltransferase [Source:HGNC Symbol;Acc:HGNC:18270]
ENSG00000166913	5833.44893	6315.49398	6797.68824	7263.44751	-0.210783805	0.0401661	0.1171576	YWHAB	tyrosine 3-monooxygenase/tryptophan 5-monooxygenase activation protein beta [Source:HGNC Symbol;Acc:HGNC:12849]
ENSG00000007944	271.643649	287.067908	189.644123	212.91067	0.473288445	0.0401743	0.1171576	MYLIP	myosin regulatory light chain interacting protein [Source:HGNC Symbol;Acc:HGNC:21155]
ENSG00000260196	203.732736	146.19199	101.143532	124.552742	0.631280498	0.040178	0.1171576	AC124798.1	antisense to KCNJ11 and overlapping to a novel gene
ENSG00000119900	436.991956	544.897418	413.002757	321.495112	0.419479738	0.0402432	0.1173274	OGFRL1	opioid growth factor receptor like 1 [Source:HGNC Symbol;Acc:HGNC:21378]
ENSG00000029993	1701.70967	1835.81699	1944.90584	2303.69345	-0.264016419	0.040264	0.1173536	HMGB3	high mobility group box 3 [Source:HGNC Symbol;Acc:HGNC:5004]
ENSG00000244617	2.95264835	1.77202412	12.6429415	14.9037469	-2.551863553	0.0402661	0.1173536	ASPRV1	aspartic peptidase retroviral like 1 [Source:HGNC Symbol;Acc:HGNC:26321]
ENSG00000197461	237.196084	226.819088	295.001969	338.527965	-0.449456968	0.0403339	0.117531	PDGFA	platelet derived growth factor subunit A [Source:HGNC Symbol;Acc:HGNC:8799]
ENSG00000145832	6.88951283	11.5181568	1.05357846	0	4.122386866	0.0403601	1	SLC25A48	solute carrier family 25 member 48 [Source:HGNC Symbol;Acc:HGNC:30451]
ENSG00000144840	190.937927	234.793196	301.32344	289.558511	-0.471623967	0.0403879	0.117668	RABL3	RAB, member of RAS oncogene family like 3 [Source:HGNC Symbol;Acc:HGNC:18072]
ENSG00000185842	212.590682	264.031594	175.947603	155.424789	0.52535885	0.0404187	0.1177376	DNAH14	dynein axonemal heavy chain 14 [Source:HGNC Symbol;Acc:HGNC:2945]
ENSG00000231574	6.88951283	11.5181568	0	1.06455335	4.120018194	0.0404266	1	LINC02015	long intergenic non-protein coding RNA 2015 [Source:HGNC Symbol;Acc:HGNC:52850]
ENSG00000182544	873.983913	839.053423	999.845961	1091.16718	-0.287787761	0.0405014	0.117958	MFSD5	major facilitator superfamily domain containing 5 [Source:HGNC Symbol;Acc:HGNC:28156]
ENSG00000179151	1366.09197	1206.74843	1562.45686	1528.69861	-0.26521378	0.0405316	0.1180257	EDC3	enhancer of mRNA decapping 3 [Source:HGNC Symbol;Acc:HGNC:26114]
ENSG00000262903	27.5580513	23.9223257	7.37504924	8.5164268	1.695263658	0.0405664	0.1181066	AC027796.4	uncharacterized LOC105371492 [Source:NCBI gene;Acc:105371492]
ENSG00000198668	7668.02778	8278.01069	7195.9409	6570.42328	0.212106422	0.0406601	0.1183565	CALM1	calmodulin 1 [Source:HGNC Symbol;Acc:HGNC:1442]
ENSG00000115762	1927.09516	2062.63608	1735.24373	1630.89573	0.245352443	0.0406662	0.1183565	PLEKHB2	pleckstrin homology domain containing B2 [Source:HGNC Symbol;Acc:HGNC:19236]
ENSG00000196656	111.216421	231.249148	93.7684831	111.778102	0.738996848	0.0407694	0.1186364	AC004057.1	ribosomal protein S26 (RPS26) pseudogene
ENSG00000084731	862.17332	839.053423	732.237031	637.667457	0.312327052	0.0407945	0.1186889	KIF3C	kinesin family member 3C [Source:HGNC Symbol;Acc:HGNC:6321]
ENSG00000103479	961.579147	1079.16269	857.612868	805.866886	0.295266863	0.0408058	0.1186976	RBL2	RB transcriptional corepressor like 2 [Source:HGNC Symbol;Acc:HGNC:9894]
ENSG00000204616	62.0056154	62.0208443	23.1787262	37.2593673	1.037575139	0.0408115	0.1186976	TRIM31	tripartite motif containing 31 [Source:HGNC Symbol;Acc:HGNC:16289]
ENSG00000145919	851.346942	872.721881	956.649244	1179.52511	-0.309048949	0.0408753	0.1188628	BOD1	biorientation of chromosomes in cell division 1 [Source:HGNC Symbol;Acc:HGNC:25114]
ENSG00000170270	294.280619	269.347667	224.412212	177.780409	0.486271204	0.0410151	0.1192486	GON7	GON7, KEOPS complex subunit [Source:HGNC Symbol;Acc:HGNC:20356]
ENSG00000009954	4897.4594	4896.10265	5583.96585	5680.45668	-0.201865683	0.0410328	0.1192797	BAZ1B	bromodomain adjacent to zinc finger domain 1B [Source:HGNC Symbol;Acc:HGNC:961]
ENSG00000215861	2.95264835	19.4922654	1.05357846	1.06455335	3.410781132	0.0411171	0.1195041	AC245297.1	phosphodiesterase 4D interacting protein-like [Source:NCBI gene;Acc:653513]
ENSG00000069509	474.392169	490.850682	611.075508	607.859963	-0.336470299	0.0411603	0.1196091	FUNDC1	FUN14 domain containing 1 [Source:HGNC Symbol;Acc:HGNC:28746]
ENSG00000177453	64.9582638	48.7306634	29.5001969	24.4847271	1.072806138	0.0412962	0.1199833	NIM1K	NIM1 serine/threonine protein kinase [Source:HGNC Symbol;Acc:HGNC:28646]
ENSG00000132561	284.438458	290.611956	204.394222	213.975223	0.45905804	0.0413085	0.1199985	MATN2	matrilin 2 [Source:HGNC Symbol;Acc:HGNC:6908]
ENSG00000169418	596.434968	549.327478	716.433354	714.315298	-0.320889032	0.0413195	0.1200098	NPR1	natriuretic peptide receptor 1 [Source:HGNC Symbol;Acc:HGNC:7943]
ENSG00000171311	583.640158	610.462311	709.058305	781.382159	-0.319543558	0.0413469	0.1200494	EXOSC1	exosome component 1 [Source:HGNC Symbol;Acc:HGNC:17286]
ENSG00000198753	146.648202	139.103894	214.930006	199.071476	-0.535304791	0.0413474	0.1200494	PLXNB3	plexin B3 [Source:HGNC Symbol;Acc:HGNC:9105]
ENSG00000246575	26.5738352	10.6321447	1.05357846	6.3873201	2.32080619	0.0414755	0.1204008	AC093162.2	retinol saturase (all-trans-retinol 13,14-reductase) (RESAT) pseudogene
ENSG00000146416	615.135074	575.90784	444.610111	491.823648	0.346747154	0.0415023	0.1204579	AIG1	androgen induced 1 [Source:HGNC Symbol;Acc:HGNC:21607]

ENSG00000184669	34.4475641	24.8083377	10.5357846	9.58098015	1.556841041	0.0415541	0.1205769	OR7E14P	olfactory receptor family 7 subfamily E member 14 pseudogene [Source:HGNC Symbol;Acc:HGNC:8385]
ENSG00000175106	23.6211868	13.2901809	38.9824031	48.9694541	-1.258027378	0.0415598	0.1205769	TVP23C	trans-golgi network vesicle protein 23 homolog C [Source:HGNC Symbol;Acc:HGNC:30453]
ENSG00000131797	423.212931	373.011078	292.894813	307.655918	0.406314569	0.0415671	0.1205769	CLUHP3	clustered mitochondria homolog pseudogene 3 [Source:HGNC Symbol;Acc:HGNC:28447]
ENSG00000133318	6639.52193	7282.13314	5822.07458	6202.08782	0.211493403	0.0415718	0.1205769	RTN3	reticulon 3 [Source:HGNC Symbol;Acc:HGNC:10469]
ENSG00000147027	6.88951283	4.43006031	0	0	4.859136496	0.0415797	1	TMEM47	transmembrane protein 47 [Source:HGNC Symbol;Acc:HGNC:18515]
ENSG00000223865	6.88951283	4.43006031	0	0	4.859136496	0.0415797	1	HLA-DPB1	major histocompatibility complex, class II, DP beta 1 [Source:HGNC Symbol;Acc:HGNC:4940]
ENSG00000198055	1305.07057	1059.67043	935.577675	981.518189	0.302180419	0.0415895	0.1206074	GRK6	G protein-coupled receptor kinase 6 [Source:HGNC Symbol;Acc:HGNC:4545]
ENSG00000168228	165.348308	186.062533	239.162311	256.557357	-0.495505671	0.0416424	0.1207402	ZCCHC4	zinc finger CCHC-type containing 4 [Source:HGNC Symbol;Acc:HGNC:22917]
ENSG00000189195	105.311125	128.471749	73.7504924	68.1314144	0.721464395	0.0416653	0.1207857	BTBD8	BTB domain containing 8 [Source:HGNC Symbol;Acc:HGNC:21019]
ENSG00000175782	633.83518	693.747444	537.325016	518.437482	0.330884268	0.041696	0.1208539	SLC35E3	solute carrier family 35 member E3 [Source:HGNC Symbol;Acc:HGNC:20864]
ENSG00000198053	298.217484	328.710475	225.465791	235.26629	0.444898754	0.0417067	0.1208644	SIRPA	signal regulatory protein alpha [Source:HGNC Symbol;Acc:HGNC:9662]
ENSG00000103942	748.988466	707.923637	527.84281	628.086477	0.333733777	0.0418193	0.1211698	HOMER2	homer scaffold protein 2 [Source:HGNC Symbol;Acc:HGNC:17513]
ENSG00000167257	213.574898	270.233679	313.966382	350.238052	-0.455734675	0.0418358	0.121197	RNF214	ring finger protein 214 [Source:HGNC Symbol;Acc:HGNC:25335]
ENSG00000117676	2949.69571	2756.38352	2385.30164	2489.99029	0.226880543	0.0418625	0.1212535	RPS6KA1	ribosomal protein S6 kinase A1 [Source:HGNC Symbol;Acc:HGNC:10430]
ENSG00000262919	389.749583	375.669114	468.842416	519.502035	-0.368926	0.0418874	0.1213049	CCNQ	cyclin Q [Source:HGNC Symbol;Acc:HGNC:28434]
ENSG00000105726	2004.84823	1747.21579	2255.71149	2199.36722	-0.248135199	0.041898	0.1213147	ATP13A1	ATPase 13A1 [Source:HGNC Symbol;Acc:HGNC:24215]
ENSG00000196636	366.128396	332.254523	246.53736	272.525658	0.427698955	0.0420003	0.1215737	SDHAF3	succinate dehydrogenase complex assembly factor 3 [Source:HGNC Symbol;Acc:HGNC:21752]
ENSG00000066279	1291.29155	1743.67174	3060.64543	2016.26405	-0.742173576	0.0420019	0.1215737	ASPM	abnormal spindle microtubule assembly [Source:HGNC Symbol;Acc:HGNC:19048]
ENSG00000121766	1019.6479	1048.15227	813.362573	883.579281	0.28530127	0.0420509	0.1216768	ZCCHC17	zinc finger CCHC-type containing 17 [Source:HGNC Symbol;Acc:HGNC:30246]
ENSG00000196199	947.800122	993.219521	824.951936	761.155645	0.291460325	0.0420519	0.1216768	MPHOSPH8	M-phase phosphoprotein 8 [Source:HGNC Symbol;Acc:HGNC:29810]
ENSG00000285601	8.85794506	2.65803619	0	0	4.881865022	0.0420585	1	AC084262.2	novel transcript
ENSG00000167244	8.85794506	2.65803619	0	0	4.881865022	0.0420585	1	IGF2	insulin like growth factor 2 [Source:HGNC Symbol;Acc:HGNC:5466]
ENSG00000145375	375.970557	365.036969	277.091136	281.042084	0.408738349	0.0420789	0.1217343	SPATA5	spermatogenesis associated 5 [Source:HGNC Symbol;Acc:HGNC:18119]
ENSG00000090487	1360.18668	1494.70235	1689.93985	1710.73723	-0.252068375	0.0422443	0.1221918	SPG21	SPG21, maspardin [Source:HGNC Symbol;Acc:HGNC:20373]
ENSG00000235034	19.6843224	33.6684583	11.5893631	4.2582134	1.752413529	0.0423849	0.1225774	C19orf81	chromosome 19 open reading frame 81 [Source:HGNC Symbol;Acc:HGNC:40041]
ENSG00000074527	1032.44271	1108.40109	937.684831	806.931439	0.295438444	0.0424165	0.1226479	NTN4	netrin 4 [Source:HGNC Symbol;Acc:HGNC:13658]
ENSG00000137507	13.7790257	7.08809649	1.05357846	1.06455335	3.297698214	0.0425013	0.122872	LRRC32	leucine rich repeat containing 32 [Source:HGNC Symbol;Acc:HGNC:4161]
ENSG00000165006	1569.82471	1538.11694	1777.38687	1903.42139	-0.244108757	0.042528	0.1229283	UBAP1	ubiquitin associated protein 1 [Source:HGNC Symbol;Acc:HGNC:12461]
ENSG00000138002	180.11155	126.699725	214.930006	234.201737	-0.551964232	0.0425716	0.1230333	IFT172	intraflagellar transport 172 [Source:HGNC Symbol;Acc:HGNC:30391]
ENSG00000070759	166.332524	168.342292	123.26868	96.8743549	0.604187992	0.0425953	0.1230805	TESK2	testis associated actin remodelling kinase 2 [Source:HGNC Symbol;Acc:HGNC:11732]
ENSG00000149483	729.304144	751.338228	897.64885	914.451328	-0.291288648	0.0427199	0.1234113	TMEM138	transmembrane protein 138 [Source:HGNC Symbol;Acc:HGNC:26944]
ENSG00000165669	267.706784	295.928029	375.073933	373.658226	-0.409056713	0.0427243	0.1234113	FAM204A	family with sequence similarity 204 member A [Source:HGNC Symbol;Acc:HGNC:25794]
ENSG00000172081	728.319927	645.016781	824.951936	875.062854	-0.30839674	0.0427622	0.1234753	MOB3A	MOB kinase activator 3A [Source:HGNC Symbol;Acc:HGNC:29802]
ENSG00000034510	19300.4781	17108.8929	19098.2168	24111.0688	-0.247058493	0.0427635	0.1234753	TMSB10	thymosin beta 10 [Source:HGNC Symbol;Acc:HGNC:11879]
ENSG00000205763	111.216421	95.6893027	146.447406	170.328536	-0.615532093	0.0427698	0.1234753	RP9P	RP9 pseudogene [Source:HGNC Symbol;Acc:HGNC:33969]
ENSG00000164045	839.536349	809.815024	982.988705	1024.10032	-0.283352688	0.0427758	0.1234753	CDC25A	cell division cycle 25A [Source:HGNC Symbol;Acc:HGNC:1725]
ENSG00000154319	432.070876	430.601862	284.466185	365.141799	0.40937924	0.0428033	0.1235339	FAM167A	family with sequence similarity 167 member A [Source:HGNC Symbol;Acc:HGNC:15549]
ENSG00000164663	270.659433	263.145582	201.233486	183.103176	0.473722982	0.0428178	0.1235544	USP49	ubiquitin specific peptidase 49 [Source:HGNC Symbol;Acc:HGNC:20078]
ENSG00000197608	180.11155	193.150629	132.750886	120.294529	0.561089313	0.0428605	0.1236566	ZNF841	zinc finger protein 841 [Source:HGNC Symbol;Acc:HGNC:27611]
ENSG00000143553	808.041433	847.027531	929.256204	1113.5228	-0.303390695	0.04288	0.1236919	SNAPIN	SNAP associated protein [Source:HGNC Symbol;Acc:HGNC:17145]
ENSG00000158966	450.770982	396.047392	359.270256	270.396551	0.426781098	0.042901	0.1237224	CACHD1	cache domain containing 1 [Source:HGNC Symbol;Acc:HGNC:29314]
ENSG00000182257	28.5422674	13.2901809	61.1075508	37.2593673	-1.24025716	0.042909	0.1237224	PRR34	proline rich 34 [Source:HGNC Symbol;Acc:HGNC:25606]
ENSG00000117971	35.4317803	22.1503015	8.4286277	10.6455335	1.592078925	0.0429126	0.1237224	CHRNB4	cholinergic receptor nicotinic beta 4 subunit [Source:HGNC Symbol;Acc:HGNC:1964]
ENSG00000188290	647.614206	544.897418	466.735259	466.274367	0.35332201	0.042953	0.1238156	HES4	hes family bHLH transcription factor 4 [Source:HGNC Symbol;Acc:HGNC:24149]
ENSG00000198919	270.659433	282.637848	204.394222	196.94237	0.463442518	0.0429652	0.1238156	DZIP3	DAZ interacting zinc finger protein 3 [Source:HGNC Symbol;Acc:HGNC:30938]
ENSG00000119684	399.591744	468.700381	597.378988	522.695695	-0.366586448	0.0429669	0.1238156	MLH3	mutL homolog 3 [Source:HGNC Symbol;Acc:HGNC:7128]
ENSG00000184708	372.033693	397.819416	489.913985	500.340075	-0.36280933	0.0429945	0.1238741	EIF4ENIF1	eukaryotic translation initiation factor 4E nuclear import factor 1 [Source:HGNC Symbol;Acc:HGNC:16687]
ENSG00000181751	256.880407	305.674161	218.090742	185.232283	0.480776961	0.0430972	0.1241462	C5orf30	chromosome 5 open reading frame 30 [Source:HGNC Symbol;Acc:HGNC:25052]
ENSG00000064932	2833.5582	2442.73525	3157.57465	3061.65544	-0.237517196	0.0431037	0.1241462	SBNO2	strawberry notch homolog 2 [Source:HGNC Symbol;Acc:HGNC:29158]
ENSG00000146374	89.5636668	129.357761	160.143926	177.780409	-0.623726516	0.0431126	0.1241507	RSPO3	R-spondin 3 [Source:HGNC Symbol;Acc:HGNC:20866]
ENSG00000088451	72.8319927	89.4872182	168.572554	101.132568	-0.731407212	0.0431375	0.1241962	TGDS	TDP-glucose 4,6-dehydratase [Source:HGNC Symbol;Acc:HGNC:20324]
ENSG00000083720	1525.53498	1891.63575	2024.9778	2098.23465	-0.270442921	0.0431431	0.1241962	OXCT1	3-oxoacid CoA-transferase 1 [Source:HGNC Symbol;Acc:HGNC:8527]

ENSG00000037241	1023.58476	894.872182	1130.48969	1205.07439	-0.284321557	0.0432357	0.124436	RPL26L1	ribosomal protein L26 like 1 [Source:HGNC Symbol;Acc:HGNC:17050]
ENSG00000153395	5102.17636	4959.89552	5828.39605	5730.49068	-0.200119846	0.0432455	0.124436	LPCAT1	lysophosphatidylcholine acyltransferase 1 [Source:HGNC Symbol;Acc:HGNC:25718]
ENSG00000124523	172.237821	133.787821	206.501379	237.395397	-0.538175128	0.0432485	0.124436	SIRT5	sirtuin 5 [Source:HGNC Symbol;Acc:HGNC:14933]
ENSG00000260916	185.03263	200.238726	145.393828	114.971762	0.565558077	0.0432706	0.1244784	CCPG1	cell cycle progression 1 [Source:HGNC Symbol;Acc:HGNC:24227]
ENSG00000132510	846.425862	808.043	1006.16743	1004.93836	-0.281833197	0.0433	0.1245306	KDM6B	lysine demethylase 6B [Source:HGNC Symbol;Acc:HGNC:29012]
ENSG00000173141	1077.71665	1088.90882	1239.00827	1370.08016	-0.268020115	0.0433035	0.1245306	MRPL57	mitochondrial ribosomal protein L57 [Source:HGNC Symbol;Acc:HGNC:14514]
ENSG00000159445	351.365154	377.441138	259.180302	287.429405	0.415448937	0.0434016	0.1247916	THEM4	thioesterase superfamily member 4 [Source:HGNC Symbol;Acc:HGNC:17947]
ENSG00000088448	2048.15374	2023.65155	1748.94025	1713.93089	0.233659785	0.0435331	0.1251485	ANKRD10	ankyrin repeat domain 10 [Source:HGNC Symbol;Acc:HGNC:20265]
ENSG00000133104	1127.91167	1265.22522	1412.84872	1462.6963	-0.264510565	0.0435419	0.1251523	SPART	spartin [Source:HGNC Symbol;Acc:HGNC:18514]
ENSG00000142156	1393.65002	1141.18354	1023.02469	1047.5205	0.291324769	0.043581	0.1252284	COL6A1	collagen type VI alpha 1 chain [Source:HGNC Symbol;Acc:HGNC:2211]
ENSG00000156958	454.707847	438.575971	348.734471	339.592519	0.375829431	0.0435832	0.1252284	GALK2	galactokinase 2 [Source:HGNC Symbol;Acc:HGNC:4119]
ENSG00000100985	9.84216118	1.77202412	0	0	4.89323722	0.0436155	1	MMP9	matrix metallopeptidase 9 [Source:HGNC Symbol;Acc:HGNC:7176]
ENSG00000163431	9.84216118	1.77202412	0	0	4.89323722	0.0436155	1	LMOD1	leiomodin 1 [Source:HGNC Symbol;Acc:HGNC:6647]
ENSG00000144596	31.4949158	51.3886996	14.7500985	19.1619603	1.291879135	0.0436405	0.1253572	GRIP2	glutamate receptor interacting protein 2 [Source:HGNC Symbol;Acc:HGNC:23841]
ENSG00000224846	14.7632418	11.5181568	2.10715692	2.1291067	2.632085276	0.0436428	0.1253572	AL133351.1	uncharacterized LOC105374888 [Source:NCBI gene;Acc:105374888]
ENSG00000100815	648.598422	722.985842	876.577281	819.70608	-0.306058785	0.0436632	0.1253944	TRIP11	thyroid hormone receptor interactor 11 [Source:HGNC Symbol;Acc:HGNC:12305]
ENSG00000175697	200.780088	192.264617	136.9652	133.069169	0.541290378	0.0436902	0.1254506	GPR156	G protein-coupled receptor 156 [Source:HGNC Symbol;Acc:HGNC:20844]
ENSG00000146918	2908.35863	3061.17167	3526.32711	3405.50617	-0.215516285	0.0437899	0.1256884	NCAPG2	non-SMC condensin II complex subunit G2 [Source:HGNC Symbol;Acc:HGNC:21904]
ENSG00000185825	2509.7511	2153.89532	2672.92856	2849.80932	-0.244240724	0.0437914	0.1256884	BCAP31	B cell receptor associated protein 31 [Source:HGNC Symbol;Acc:HGNC:16695]
ENSG00000128694	164.364092	186.062533	108.518582	125.617295	0.582537605	0.0437953	0.1256884	OSGEPL1	O-sialoglycoprotein endopeptidase like 1 [Source:HGNC Symbol;Acc:HGNC:23075]
ENSG00000134853	9.84216118	7.97410856	0	1.06455335	4.070125155	0.043809	1	PDGFRA	platelet derived growth factor receptor alpha [Source:HGNC Symbol;Acc:HGNC:8803]
ENSG00000116793	493.092275	539.581346	647.950754	649.377544	-0.328678308	0.0438091	0.1257066	PHTF1	putative homeodomain transcription factor 1 [Source:HGNC Symbol;Acc:HGNC:8939]
ENSG00000241769	21.6527546	10.6321447	2.10715692	4.2582134	2.3406658	0.0438459	0.1257909	LINC00893	long intergenic non-protein coding RNA 893 [Source:HGNC Symbol;Acc:HGNC:44265]
ENSG00000137273	424.197147	425.28579	540.485751	539.728549	-0.346646619	0.0438832	0.1258764	FOXF2	forkhead box F2 [Source:HGNC Symbol;Acc:HGNC:3810]
ENSG00000103248	398.607528	411.995609	478.324622	567.406936	-0.367184179	0.0439224	0.1259676	MTHFSD	methenyltetrahydrofolate synthetase domain containing [Source:HGNC Symbol;Acc:HGNC:25778]
ENSG00000212232	32.4791319	19.4922654	51.6253447	58.5504343	-1.088551469	0.0439504	0.1260265	SNORD17	small nucleolar RNA, C/D box 17 [Source:HGNC Symbol;Acc:HGNC:32713]
ENSG00000167748	3.93686447	21.2642895	2.10715692	1.06455335	2.993967131	0.044	0.1261474	KLK1	kallikrein 1 [Source:HGNC Symbol;Acc:HGNC:6357]
ENSG00000128534	711.588253	777.91859	643.73644	540.793102	0.330766181	0.04402	0.1261833	LSM8	LSM8 homolog, U6 small nuclear RNA associated [Source:HGNC Symbol;Acc:HGNC:20471]
ENSG00000005810	1341.48657	1331.67613	1221.09744	951.710695	0.29888394	0.0441127	0.1264275	MYCBP2	MYC binding protein 2, E3 ubiquitin protein ligase [Source:HGNC Symbol;Acc:HGNC:23386]
ENSG00000151502	1421.20807	1305.09577	1110.4717	1160.36315	0.263468792	0.0441374	0.1264769	VPS26B	VPS26, retromer complex component B [Source:HGNC Symbol;Acc:HGNC:28119]
ENSG00000135473	433.055092	408.45156	540.485751	531.212122	-0.349198153	0.044192	0.1266117	PAN2	poly(A) specific ribonuclease subunit PAN2 [Source:HGNC Symbol;Acc:HGNC:20074]
ENSG00000171421	1347.39187	1236.87284	1485.54563	1605.34645	-0.258539346	0.0442166	0.1266316	MRPL36	mitochondrial ribosomal protein L36 [Source:HGNC Symbol;Acc:HGNC:14490]
ENSG00000174607	95.4689635	98.3473389	66.3754431	44.7112407	0.80281242	0.0442183	0.1266316	UGT8	UDP glycosyltransferase 8 [Source:HGNC Symbol;Acc:HGNC:12555]
ENSG00000134253	243.101381	232.13516	148.554563	185.232283	0.509648854	0.0442214	0.1266316	TRIM45	tripartite motif containing 45 [Source:HGNC Symbol;Acc:HGNC:19018]
ENSG00000224715	26.5738352	31.8964342	11.5893631	8.5164268	1.540611402	0.044244	0.1266644	Z82186.1	uncharacterized LOC339685 [Source:NCBI gene;Acc:339685]
ENSG00000260287	9.84216118	8.86012062	15.8036769	42.582134	-1.643102484	0.0442478	0.1266644	TBC1D3G	TBC1 domain family member 3G [Source:HGNC Symbol;Acc:HGNC:29860]
ENSG00000261342	46.2581576	35.4404825	18.9644123	14.9037469	1.268773345	0.0442645	0.1266805	AC006538.1	novel transcript
ENSG00000218052	17.7158901	8.86012062	2.10715692	2.1291067	2.646383263	0.0442685	0.1266805	ADAMTS7P4	ADAMTS7 pseudogene 4 [Source:HGNC Symbol;Acc:HGNC:49410]
ENSG00000141480	845.441646	753.996265	666.915167	620.634603	0.31245316	0.0442873	0.126713	ARRB2	arrestin beta 2 [Source:HGNC Symbol;Acc:HGNC:712]
ENSG00000159131	3321.7294	3482.91341	4014.13394	3863.26411	-0.211113996	0.0443236	0.1267953	GART	phosphoribosylglycinamide formyltransferase, phosphoribosylglycinamide synthetase, phosphoribosylaminoimidazole synthetase [Source:HGNC Symbol;Acc:HGNC:4163]
ENSG00000100281	615.135074	626.410528	751.201444	783.511266	-0.305719076	0.0444011	0.1269955	HMGXB4	HMG-box containing 4 [Source:HGNC Symbol;Acc:HGNC:5003]
ENSG00000250305	118.105934	97.4613268	75.8576493	50.0340075	0.77467662	0.0444176	0.1270213	TRMT9B	tRNA methyltransferase 9B (putative) [Source:HGNC Symbol;Acc:HGNC:26725]
ENSG00000113593	533.445136	624.638504	750.147865	703.669765	-0.327383911	0.044436	0.1270523	PPWD1	peptidylprolyl isomerase domain and WD repeat containing 1 [Source:HGNC Symbol;Acc:HGNC:28954]
ENSG00000121022	781.467598	831.079314	969.292185	994.292829	-0.283857675	0.0445195	0.1272696	COPS5	COP9 signalosome subunit 5 [Source:HGNC Symbol;Acc:HGNC:2240]
ENSG00000118873	960.594931	1054.35435	1314.86592	1139.07208	-0.284064332	0.0445479	0.1273293	RAB3GAP2	RAB3 GTPase activating non-catalytic protein subunit 2 [Source:HGNC Symbol;Acc:HGNC:17168]
ENSG00000139197	1101.33784	953.348978	1261.13342	1228.49457	-0.277560631	0.0446026	0.127464	PEX5	peroxisomal biogenesis factor 5 [Source:HGNC Symbol;Acc:HGNC:9719]
ENSG00000137207	1048.19017	932.970701	1154.72199	1247.65653	-0.278538104	0.0447276	0.1277997	YIPF3	Yip1 domain family member 3 [Source:HGNC Symbol;Acc:HGNC:21023]
ENSG00000165389	1746.98361	1620.51606	1421.27735	1415.85596	0.247022298	0.0447434	0.1278231	SPTSSA	serine palmitoyltransferase small subunit A [Source:HGNC Symbol;Acc:HGNC:20361]
ENSG00000189334	158.458795	111.63752	204.394222	195.877816	-0.569833301	0.0447642	0.1278502	S100A14	S100 calcium binding protein A14 [Source:HGNC Symbol;Acc:HGNC:18901]
ENSG00000245498	34.4475641	33.6684583	17.9108339	6.3873201	1.485990943	0.044768	0.1278502	AP000866.1	uncharacterized LOC100507283 [Source:NCBI gene;Acc:100507283]
ENSG00000092295	16.731674	15.0622051	34.7680893	42.582134	-1.283644979	0.0448466	0.1280531	TGM1	transglutaminase 1 [Source:HGNC Symbol;Acc:HGNC:11777]

ENSG00000165688	2695.76795	2343.5019	2878.37636	3060.59088	-0.237277313	0.0448892	0.128142	PMPCA	peptidase, mitochondrial processing alpha subunit [Source:HGNC Symbol;Acc:HGNC:18667]
ENSG00000120093	87.5952345	86.8291821	114.840052	162.876663	-0.670887898	0.0448947	0.128142	HOXB3	homeobox B3 [Source:HGNC Symbol;Acc:HGNC:5114]
ENSG00000183935	124.011231	116.953592	83.2326985	64.9377544	0.701040688	0.0449005	0.128142	HTR7P1	5-hydroxytryptamine receptor 7 pseudogene 1 [Source:HGNC Symbol;Acc:HGNC:30411]
ENSG00000115363	161.411443	160.368183	241.269468	215.039777	-0.504052232	0.0449272	0.1281966	EVA1A	eva-1 homolog A, regulator of programmed cell death [Source:HGNC Symbol;Acc:HGNC:25816]
ENSG00000100445	99.4058279	85.94317	154.876034	133.069169	-0.636754419	0.0449356	0.1281988	SDR39U1	short chain dehydrogenase/reductase family 39U member 1 [Source:HGNC Symbol;Acc:HGNC:20275]
ENSG00000132405	370.06526	413.767633	572.093105	452.435174	-0.38588338	0.0449748	0.128289	TBC1D14	TBC1 domain family member 14 [Source:HGNC Symbol;Acc:HGNC:29246]
ENSG00000151208	2295.19199	2099.84859	2721.39317	2459.11824	-0.237453383	0.0450179	0.1283904	DLG5	discs large MAGUK scaffold protein 5 [Source:HGNC Symbol;Acc:HGNC:2904]
ENSG00000204991	962.563364	825.763242	711.165462	735.606365	0.3052347	0.0450934	0.1285838	SPIRE2	spire type actin nucleation factor 2 [Source:HGNC Symbol;Acc:HGNC:30623]
ENSG00000203739	38.3844286	29.238398	7.37504924	17.0328536	1.469812534	0.0451139	0.1286205	AL645568.1	uncharacterized LOC101928673 [Source:NCBI gene;Acc:101928673]
ENSG00000227268	70.8635605	73.5390011	31.6073539	44.7112407	0.920509748	0.0451226	0.1286237	KLLN	killin, p53 regulated DNA replication inhibitor [Source:HGNC Symbol;Acc:HGNC:37212]
ENSG00000204428	19.6843224	16.8342292	51.6253447	34.0657072	-1.232294497	0.0451467	0.1286709	LY6G5C	lymphocyte antigen 6 family member G5C [Source:HGNC Symbol;Acc:HGNC:13932]
ENSG00000162526	3.93686447	7.08809649	0	0	4.824548398	0.0451746	1	TSSK3	testis specific serine kinase 3 [Source:HGNC Symbol;Acc:HGNC:15473]
ENSG00000007350	3.93686447	7.08809649	0	0	4.824548398	0.0451746	1	TKTL1	transketolase like 1 [Source:HGNC Symbol;Acc:HGNC:11835]
ENSG00000143546	3.93686447	7.08809649	0	0	4.824548398	0.0451746	1	S100A8	S100 calcium binding protein A8 [Source:HGNC Symbol;Acc:HGNC:10498]
ENSG00000101452	292.312187	291.497968	382.448982	385.368313	-0.39527753	0.0451812	0.1287475	DHX35	DEAH-box helicase 35 [Source:HGNC Symbol;Acc:HGNC:15861]
ENSG00000214922	45.2739414	64.6788805	22.1251477	29.8074938	1.084225773	0.0452188	0.1288329	HLA-F-AS1	HLA-F antisense RNA 1 [Source:HGNC Symbol;Acc:HGNC:26645]
ENSG00000173120	4152.4078	3920.60337	4892.81838	4470.05952	-0.213950954	0.0452703	0.1289577	KDM2A	lysine demethylase 2A [Source:HGNC Symbol;Acc:HGNC:13606]
ENSG00000144362	0.98421612	4.43006031	35.8216677	1.06455335	-2.753178605	0.0453278	0.1290849	PHOSPHO2	phosphatase, orphan 2 [Source:HGNC Symbol;Acc:HGNC:28316]
ENSG00000104361	34.4475641	54.0467358	85.3398554	77.7123946	-0.878069762	0.0453302	0.1290849	NIPAL2	NIPA like domain containing 2 [Source:HGNC Symbol;Acc:HGNC:25854]
ENSG00000161013	4934.85962	4812.81752	5456.48286	5751.78175	-0.201462328	0.0453629	0.1291563	MGAT4B	alpha-1,3-mannosyl-glycoprotein 4-beta-N-acetylglucosaminyltransferase B [Source:HGNC Symbol;Acc:HGNC:7048]
ENSG00000135587	117.121718	129.357761	177.001182	186.296836	-0.558912887	0.0454514	0.1293865	SMPD2	sphingomyelin phosphodiesterase 2 [Source:HGNC Symbol;Acc:HGNC:11121]
ENSG00000104450	182.079982	222.389028	127.482994	149.037469	0.549762853	0.0455282	0.1295833	SPAG1	sperm associated antigen 1 [Source:HGNC Symbol;Acc:HGNC:11212]
ENSG00000122644	685.014418	895.758194	999.845961	978.324529	-0.322593768	0.0455472	0.1296156	ARL4A	ADP ribosylation factor like GTPase 4A [Source:HGNC Symbol;Acc:HGNC:695]
ENSG00000182040	13.7790257	18.6062533	1.05357846	5.32276675	2.347851851	0.0456406	0.1298485	USH1G	USH1 protein network component sans [Source:HGNC Symbol;Acc:HGNC:16356]
ENSG00000125733	2322.75004	2032.51167	2505.40958	2632.64044	-0.238766323	0.0456445	0.1298485	TRIP10	thyroid hormone receptor interactor 10 [Source:HGNC Symbol;Acc:HGNC:12304]
ENSG00000099992	275.580513	265.803619	190.697702	202.265137	0.462112867	0.045729	0.1300671	TBC1D10A	TBC1 domain family member 10A [Source:HGNC Symbol;Acc:HGNC:23609]
ENSG00000125450	1895.60024	1802.14853	2129.28207	2211.07731	-0.231306833	0.0457377	0.13007	NUP85	nucleoporin 85 [Source:HGNC Symbol;Acc:HGNC:8734]
ENSG00000122484	272.627865	372.125066	490.967563	384.303759	-0.439639903	0.0457828	0.1301764	RPAP2	RNA polymerase II associated protein 2 [Source:HGNC Symbol;Acc:HGNC:25791]
ENSG00000169592	602.340264	510.342948	642.682862	769.672072	-0.344720355	0.0458197	0.1302594	INO80E	INO80 complex subunit E [Source:HGNC Symbol;Acc:HGNC:26905]
ENSG00000106617	318.886022	391.617331	259.180302	269.331998	0.427883733	0.0458304	0.1302679	PRKAG2	protein kinase AMP-activated non-catalytic subunit gamma 2 [Source:HGNC Symbol;Acc:HGNC:9386]
ENSG00000134247	2514.67218	2409.0668	2934.21602	2799.77531	-0.219902411	0.0458909	0.1304178	PTGFRN	prostaglandin F2 receptor inhibitor [Source:HGNC Symbol;Acc:HGNC:9601]
ENSG00000213064	1280.46517	1535.4589	1133.65043	1189.10609	0.278281826	0.0459186	0.1304746	SFT2D2	SFT2 domain containing 2 [Source:HGNC Symbol;Acc:HGNC:25140]
ENSG00000144285	20.6685385	18.6062533	2.10715692	7.45187345	2.039781444	0.0459631	0.1305791	SCN1A	sodium voltage-gated channel alpha subunit 1 [Source:HGNC Symbol;Acc:HGNC:10585]
ENSG00000064419	2087.52239	1977.57892	2316.81904	2446.3436	-0.228763382	0.0459726	0.130584	TNPO3	transportin 3 [Source:HGNC Symbol;Acc:HGNC:17103]
ENSG00000123388	129.916528	136.445858	183.322652	204.394243	-0.541188453	0.0460052	0.1306548	HOXC11	homeobox C11 [Source:HGNC Symbol;Acc:HGNC:5123]
ENSG00000110756	1043.26909	1164.21985	938.73841	878.256514	0.281196391	0.0460267	0.1306938	HPS5	HPS5, biogenesis of lysosomal organelles complex 2 subunit 2 [Source:HGNC Symbol;Acc:HGNC:17022]
ENSG00000146094	161.411443	135.559845	96.9292185	95.8098015	0.622729897	0.0460437	0.1307039	DOK3	docking protein 3 [Source:HGNC Symbol;Acc:HGNC:24583]
ENSG00000128309	1483.21369	1298.89368	1134.704	1176.33145	0.267255674	0.0460513	0.1307039	MPST	mercaptopyruvate sulfurtransferase [Source:HGNC Symbol;Acc:HGNC:7223]
ENSG00000089902	1937.92154	2107.8227	2317.87262	2434.63351	-0.232044945	0.0460534	0.1307039	RCOR1	REST corepressor 1 [Source:HGNC Symbol;Acc:HGNC:17441]
ENSG00000132964	442.897253	465.156332	352.948785	350.238052	0.369103464	0.0460641	0.1307122	CDK8	cyclin dependent kinase 8 [Source:HGNC Symbol;Acc:HGNC:1779]
ENSG00000119729	633.83518	910.8204	672.183059	493.952755	0.406283513	0.0461159	0.1308372	RHOQ	ras homolog family member Q [Source:HGNC Symbol;Acc:HGNC:17736]
ENSG00000166263	328.728183	326.938451	249.698096	239.524504	0.422412722	0.0461474	0.1308894	STXBP4	syntaxin binding protein 4 [Source:HGNC Symbol;Acc:HGNC:19694]
ENSG00000177335	86.6110184	60.2488202	34.7680893	42.582134	0.923336406	0.0461498	0.1308894	C8orf31	chromosome 8 open reading frame 31 (putative) [Source:HGNC Symbol;Acc:HGNC:26731]
ENSG00000110871	498.013356	507.684911	590.003939	678.120484	-0.334334651	0.0461747	0.1309381	COQ5	coenzyme Q5, methyltransferase [Source:HGNC Symbol;Acc:HGNC:28722]
ENSG00000182247	499.981788	465.156332	313.966382	418.369467	0.39810321	0.0462192	0.1310205	UBE2E2	ubiquitin conjugating enzyme E2 E2 [Source:HGNC Symbol;Acc:HGNC:12478]
ENSG00000242125	887.762939	891.328134	977.720813	1213.59082	-0.300568375	0.0462193	0.1310205	SNHG3	small nucleolar RNA host gene 3 [Source:HGNC Symbol;Acc:HGNC:10118]
ENSG00000132205	205.701169	186.948545	251.805252	291.687618	-0.46957325	0.0462395	0.1310559	EMILIN2	elastin microfibril interfacer 2 [Source:HGNC Symbol;Acc:HGNC:19881]
ENSG00000132781	340.538777	295.928029	423.538542	409.85304	-0.389767249	0.046259	0.1310891	MUTYH	mutY DNA glycosylase [Source:HGNC Symbol;Acc:HGNC:7527]
ENSG00000102804	2945.75884	2931.81391	3603.23834	3244.75861	-0.220491943	0.0462906	0.1311567	TSC22D1	TSC22 domain family member 1 [Source:HGNC Symbol;Acc:HGNC:16826]
ENSG00000261488	25.5896191	24.8083377	9.48220616	6.3873201	1.666446437	0.0463718	0.1313647	TBILA	TGF-beta induced lncRNA [Source:HGNC Symbol;Acc:HGNC:53943]

ENSG00000181513	283.454242	240.995281	147.500985	217.168883	0.523772446	0.0463926	0.1314016	ACBD4	acyl-CoA binding domain containing 4 [Source:HGNC Symbol;Acc:HGNC:23337]
ENSG00000008710	2794.18956	2466.65758	3275.57544	2928.58627	-0.238205808	0.0464032	0.1314097	PKD1	polycystin 1, transient receptor potential channel interacting [Source:HGNC Symbol;Acc:HGNC:9008]
ENSG00000134077	1094.44832	1119.91925	1357.00906	1290.23866	-0.257526058	0.0464353	0.1314725	THUMPD3	THUMP domain containing 3 [Source:HGNC Symbol;Acc:HGNC:24493]
ENSG00000160991	951.736986	876.265929	1111.52528	1097.5545	-0.273534115	0.0464409	0.1314725	ORAI2	ORAI calcium release-activated calcium modulator 2 [Source:HGNC Symbol;Acc:HGNC:21667]
ENSG00000131459	1956.62164	1845.56312	1546.65318	1669.21965	0.241488574	0.0464683	0.1315279	GFPT2	glutamine-fructose-6-phosphate transaminase 2 [Source:HGNC Symbol;Acc:HGNC:4242]
ENSG00000130208	0	0	8.4286277	2.1291067	-4.942093949	0.0465095	1	APOC1	apolipoprotein C1 [Source:HGNC Symbol;Acc:HGNC:607]
ENSG00000107186	537.382001	502.368839	385.609717	426.885893	0.355561162	0.0465227	0.1316598	MPDZ	multiple PDZ domain crumbs cell polarity complex component [Source:HGNC Symbol;Acc:HGNC:7208]
ENSG00000109107	872.015481	871.835869	740.665659	682.378698	0.293252672	0.046564	0.1317545	ALDOC	aldolase, fructose-bisphosphate C [Source:HGNC Symbol;Acc:HGNC:418]
ENSG00000228343	39.3686447	27.4663739	69.5361785	60.679541	-0.965486476	0.0466444	0.13196	AC115618.2	novel transcript
ENSG00000183876	259.833055	248.083377	348.734471	328.946985	-0.416351813	0.046673	0.1320189	ARSI	arylsulfatase family member I [Source:HGNC Symbol;Acc:HGNC:32521]
ENSG00000140332	4422.08302	3762.00721	3456.79093	3522.60704	0.229503054	0.0466835	0.1320265	TLE3	transducin like enhancer of split 3 [Source:HGNC Symbol;Acc:HGNC:11839]
ENSG00000196182	558.050539	520.975092	665.861588	679.185037	-0.318282103	0.0467008	0.1320532	STK40	serine/threonine kinase 40 [Source:HGNC Symbol;Acc:HGNC:21373]
ENSG00000197479	33.463348	19.4922654	8.4286277	8.5164268	1.641424298	0.0467162	0.1320747	PCDHB11	protocadherin beta 11 [Source:HGNC Symbol;Acc:HGNC:8682]
ENSG00000180881	51.1792381	50.5026875	21.0715692	26.6138338	1.092616476	0.0467837	0.1322434	CAPS2	calcyphosine 2 [Source:HGNC Symbol;Acc:HGNC:16471]
ENSG00000250073	30.5106997	27.4663739	3.16073539	14.9037469	1.683373132	0.046803	0.1322757	AP000866.2	uncharacterized LOC101929340 [Source:NCBI gene;Acc:101929340]
ENSG00000196396	2334.56063	2169.84354	1891.17334	1947.06808	0.23072027	0.046812	0.1322792	PTPN1	protein tyrosine phosphatase, non-receptor type 1 [Source:HGNC Symbol;Acc:HGNC:9642]
ENSG00000168140	2216.4547	1964.28874	1727.86868	1804.41793	0.242882412	0.0468708	0.1324015	VASN	vasorin [Source:HGNC Symbol;Acc:HGNC:18517]
ENSG00000272886	554.113675	629.068564	804.933945	682.378698	-0.329531966	0.046871	0.1324015	DCP1A	decapping mRNA 1A [Source:HGNC Symbol;Acc:HGNC:18714]
ENSG00000064989	12.7948095	21.2642895	5.26789231	2.1291067	2.203911214	0.0468928	0.1324409	CALCRL	calcitonin receptor like receptor [Source:HGNC Symbol;Acc:HGNC:16709]
ENSG00000068697	3945.72242	4157.16859	4659.97754	4669.13099	-0.20321148	0.0469392	0.1325498	LAPTM4A	lysosomal protein transmembrane 4 alpha [Source:HGNC Symbol;Acc:HGNC:6924]
ENSG00000154330	33.463348	42.528579	12.6429415	18.097407	1.307302233	0.0470217	0.1327606	PGM5	phosphoglucomutase 5 [Source:HGNC Symbol;Acc:HGNC:8908]
ENSG00000126010	18.7001062	3.54404825	0	2.1291067	3.384483654	0.0471425	0.1330796	GRPR	gastrin releasing peptide receptor [Source:HGNC Symbol;Acc:HGNC:4609]
ENSG00000120149	91.532099	64.6788805	43.196717	41.5175807	0.881009269	0.0471598	0.1331062	MSX2	msh homeobox 2 [Source:HGNC Symbol;Acc:HGNC:7392]
ENSG00000178184	122.042799	165.684256	102.197111	79.8415013	0.661731809	0.0471811	0.1331441	PARD6G	par-6 family cell polarity regulator gamma [Source:HGNC Symbol;Acc:HGNC:16076]
ENSG00000154447	297.233268	276.435763	199.126329	220.362544	0.451286188	0.0472129	0.1332114	SH3RF1	SH3 domain containing ring finger 1 [Source:HGNC Symbol;Acc:HGNC:17650]
ENSG00000104691	77.7530733	70.8809649	115.893631	122.423635	-0.681838643	0.0472709	0.1333529	UBXN8	UBX domain protein 8 [Source:HGNC Symbol;Acc:HGNC:30307]
ENSG00000130005	516.713462	439.461983	383.50256	353.431712	0.374972826	0.0473334	0.1335068	GAMT	guanidinoacetate N-methyltransferase [Source:HGNC Symbol;Acc:HGNC:4136]
ENSG00000104325	886.778722	903.732303	713.272619	753.703772	0.287630121	0.0473543	0.1335437	DECR1	2,4-dienoyl-CoA reductase 1 [Source:HGNC Symbol;Acc:HGNC:2753]
ENSG00000117569	95.4689635	130.243773	180.161917	161.812109	-0.597296408	0.0474073	0.1336707	PTBP2	polypyrimidine tract binding protein 2 [Source:HGNC Symbol;Acc:HGNC:17662]
ENSG00000114026	613.166642	510.342948	718.540511	691.959678	-0.329077815	0.0474241	0.1336957	OGG1	8-oxoguanine DNA glycosylase [Source:HGNC Symbol;Acc:HGNC:8125]
ENSG00000124702	1497.97693	1150.04366	1583.52843	1653.25135	-0.290315677	0.0474413	0.1337218	KLHDC3	kelch domain containing 3 [Source:HGNC Symbol;Acc:HGNC:20704]
ENSG00000005175	738.162089	865.633784	1068.32856	916.580435	-0.307029521	0.0474738	0.1337912	RPAP3	RNA polymerase II associated protein 3 [Source:HGNC Symbol;Acc:HGNC:26151]
ENSG00000112308	2750.88405	3008.01095	3838.18634	3047.81624	-0.257772313	0.0474893	0.1338127	C6orf62	chromosome 6 open reading frame 62 [Source:HGNC Symbol;Acc:HGNC:20998]
ENSG00000170085	539.350433	455.4102	375.073933	394.949293	0.368735964	0.0475161	0.1338657	SIMC1	SUMO interacting motifs containing 1 [Source:HGNC Symbol;Acc:HGNC:24779]
ENSG00000120278	16.731674	29.238398	9.48220616	3.19366005	1.859657378	0.0475913	0.1340553	PLEKHG1	pleckstrin homology and RhoGEF domain containing G1 [Source:HGNC Symbol;Acc:HGNC:20884]
ENSG00000175390	4464.40431	4117.29805	4822.22862	5092.82323	-0.208473764	0.0476108	0.1340878	EIF3F	eukaryotic translation initiation factor 3 subunit F [Source:HGNC Symbol;Acc:HGNC:3275]
ENSG00000155034	603.32448	513.886996	476.217465	387.497419	0.370547513	0.0476692	0.1342208	FBXL18	F-box and leucine rich repeat protein 18 [Source:HGNC Symbol;Acc:HGNC:21874]
ENSG00000205089	2.95264835	3.54404825	13.69652	17.0328536	-2.238992413	0.0476775	0.1342208	CCNI2	cyclin I family member 2 [Source:HGNC Symbol;Acc:HGNC:33869]
ENSG00000144029	1361.17089	1262.56719	1444.45607	1717.12455	-0.269201676	0.0476818	0.1342208	MRPS5	mitochondrial ribosomal protein S5 [Source:HGNC Symbol;Acc:HGNC:14498]
ENSG00000159208	36.4159964	29.238398	51.6253447	77.7123946	-0.979869138	0.0477023	0.1342562	CIART	circadian associated repressor of transcription [Source:HGNC Symbol;Acc:HGNC:25200]
ENSG00000177733	7711.33329	6545.85711	7861.80249	8832.59915	-0.227793785	0.0477196	0.1342824	HNRNPA0	heterogeneous nuclear ribonucleoprotein A0 [Source:HGNC Symbol;Acc:HGNC:5030]
ENSG00000260430	27.5580513	38.0985187	9.48220616	14.9037469	1.430961677	0.0477343	0.1342835	AC099518.1	novel transcript
ENSG00000170006	250.97511	302.130113	207.554957	192.684156	0.467554445	0.0477359	0.1342835	TMEM154	transmembrane protein 154 [Source:HGNC Symbol;Acc:HGNC:26489]
ENSG00000156973	481.281682	500.596815	378.234668	389.626526	0.354907085	0.0477494	0.1342993	PDE6D	phosphodiesterase 6D [Source:HGNC Symbol;Acc:HGNC:8788]
ENSG00000128951	2164.29124	2286.79713	2534.90978	2660.31882	-0.222873489	0.0478268	0.134484	DUT	deoxyuridine triphosphatase [Source:HGNC Symbol;Acc:HGNC:3078]
ENSG00000171806	29.5264835	60.2488202	99.0363755	71.3250745	-0.919380193	0.047831	0.134484	METTL18	methyltransferase like 18 [Source:HGNC Symbol;Acc:HGNC:28793]
ENSG00000204228	170.269388	173.658364	109.57216	122.423635	0.568205105	0.0478861	0.1345959	HSD17B8	hydroxysteroid 17-beta dehydrogenase 8 [Source:HGNC Symbol;Acc:HGNC:3554]
ENSG00000225830	628.9141	715.011734	841.809191	816.51242	-0.302686315	0.0478867	0.1345959	ERCC6	ERCC excision repair 6, chromatin remodeling factor [Source:HGNC Symbol;Acc:HGNC:3438]
ENSG00000204764	126.963879	106.321447	64.2682862	78.7769479	0.704864488	0.0479388	0.1347197	RANBP17	RAN binding protein 17 [Source:HGNC Symbol;Acc:HGNC:14428]
ENSG00000171219	614.150858	577.679864	478.324622	469.468027	0.330246128	0.0480325	0.1349605	CDC42BPG	CDC42 binding protein kinase gamma [Source:HGNC Symbol;Acc:HGNC:29829]
ENSG00000033627	1693.83594	1645.3244	1437.08102	1389.24212	0.240457821	0.0480731	0.1350522	ATP6V0A1	ATPase H+ transporting V0 subunit a1 [Source:HGNC Symbol;Acc:HGNC:865]

ENSG00000241288	127.948095	100.119363	74.8040708	63.873201	0.716319531	0.0480863	0.135067	AC092902.2	uncharacterized LOC101927056 [Source:NCBI gene;Acc:101927056]
ENSG00000136158	168.300956	195.808666	110.625739	135.198275	0.567677113	0.0481378	0.1351696	SPRY2	sprouty RTK signaling antagonist 2 [Source:HGNC Symbol;Acc:HGNC:11270]
ENSG00000172731	525.571407	476.674489	363.48457	415.175807	0.363815919	0.0481389	0.1351696	LRRC20	leucine rich repeat containing 20 [Source:HGNC Symbol;Acc:HGNC:23421]
ENSG00000167196	1207.63318	1150.04366	1403.36651	1402.01676	-0.251021121	0.0482108	0.1353275	FBXO22	F-box protein 22 [Source:HGNC Symbol;Acc:HGNC:13593]
ENSG00000129003	946.815906	1119.91925	928.202625	721.767171	0.325270028	0.0482111	0.1353275	VPS13C	vacuolar protein sorting 13 homolog C [Source:HGNC Symbol;Acc:HGNC:23594]
ENSG00000256073	124.995447	115.181568	55.8396585	89.4224814	0.725415738	0.0482305	0.1353595	URB1-AS1	URB1 antisense RNA 1 (head to head) [Source:HGNC Symbol;Acc:HGNC:23128]
ENSG00000212123	3.93686447	10.6321447	24.2323046	24.4847271	-1.7307673	0.0482901	0.1355041	PRR22	proline rich 22 [Source:HGNC Symbol;Acc:HGNC:28354]
ENSG00000182149	1338.53392	1332.56214	1521.3673	1647.92859	-0.246719696	0.0483397	0.1356208	IST1	IST1, ESCRT-III associated factor [Source:HGNC Symbol;Acc:HGNC:28977]
ENSG00000137496	102.358476	108.979484	76.9112277	46.8403474	0.772006779	0.0483725	0.1356902	IL18BP	interleukin 18 binding protein [Source:HGNC Symbol;Acc:HGNC:5987]
ENSG00000065717	1137.75383	1006.5097	822.844779	931.484181	0.289217386	0.0483811	0.1356918	TLE2	transducin like enhancer of split 2 [Source:HGNC Symbol;Acc:HGNC:11838]
ENSG00000061794	912.368342	1138.5255	1292.74077	1218.91359	-0.291679791	0.0484019	0.1357278	MRPS35	mitochondrial ribosomal protein S35 [Source:HGNC Symbol;Acc:HGNC:16635]
ENSG00000110427	65.9424799	45.1866152	22.1251477	30.8720472	1.06672003	0.0484243	0.1357531	KIAA1549L	KIAA1549 like [Source:HGNC Symbol;Acc:HGNC:24836]
ENSG00000167711	15.7474579	4.43006031	26.3394616	34.0657072	-1.593445814	0.048427	0.1357531	SERPINF2	serpin family F member 2 [Source:HGNC Symbol;Acc:HGNC:9075]
ENSG00000177427	282.470026	211.756883	325.555745	347.044392	-0.446195821	0.0484496	0.1357718	MIEF2	mitochondrial elongation factor 2 [Source:HGNC Symbol;Acc:HGNC:17920]
ENSG00000244242	22.6369707	13.2901809	5.26789231	3.19366005	2.082996182	0.0484497	0.1357718	IFITM10	interferon induced transmembrane protein 10 [Source:HGNC Symbol;Acc:HGNC:40022]
ENSG00000165125	11.8105934	8.86012062	2.10715692	0	3.288914383	0.048482	0.1358398	TRPV6	transient receptor potential cation channel subfamily V member 6 [Source:HGNC Symbol;Acc:HGNC:14006]
ENSG00000119929	385.812718	408.45156	540.485751	476.919901	-0.356946112	0.0485179	0.1358884	CUTC	cutC copper transporter [Source:HGNC Symbol;Acc:HGNC:24271]
ENSG00000165891	1239.12809	1334.33417	1168.41851	934.677842	0.291313759	0.0485183	0.1358884	E2F7	E2F transcription factor 7 [Source:HGNC Symbol;Acc:HGNC:23820]
ENSG00000272473	7.87372895	7.08809649	24.2323046	23.4201737	-1.672574198	0.0485235	0.1358884	AC006273.1	novel transcript
ENSG00000136699	2596.36212	2317.80755	2867.84057	2876.42315	-0.225433982	0.0485437	0.1359224	SMPD4	sphingomyelin phosphodiesterase 4 [Source:HGNC Symbol;Acc:HGNC:32949]
ENSG00000171848	1739.10988	1986.43904	2132.44281	2277.07962	-0.242807231	0.0485819	0.1360069	RRM2	ribonucleotide reductase regulatory subunit M2 [Source:HGNC Symbol;Acc:HGNC:10452]
ENSG00000143373	1265.70193	1066.75852	1397.04504	1414.7914	-0.270229334	0.0485904	0.136008	ZNF687	zinc finger protein 687 [Source:HGNC Symbol;Acc:HGNC:29277]
ENSG00000103363	4621.87889	3911.74325	4765.33539	5254.63534	-0.231844561	0.0486159	0.1360525	ELOB	elongin B [Source:HGNC Symbol;Acc:HGNC:11619]
ENSG00000196923	2627.85704	2258.44475	2758.26841	3010.55687	-0.239825976	0.0486224	0.1360525	PDLIM7	PDZ and LIM domain 7 [Source:HGNC Symbol;Acc:HGNC:22958]
ENSG00000107672	520.650327	526.291165	634.254234	670.668611	-0.317698437	0.0486819	0.1361964	NSMCE4A	NSE4 homolog A, SMC5-SMC6 complex component [Source:HGNC Symbol;Acc:HGNC:25935]
ENSG00000280444	0.98421612	0	7.37504924	8.5164268	-4.073870113	0.0486969	1	AC073592.10	novel transcript
ENSG00000104231	271.643649	346.430716	449.878003	375.787333	-0.416581773	0.0487976	0.1364976	ZFAND1	zinc finger AN1-type containing 1 [Source:HGNC Symbol;Acc:HGNC:25858]
ENSG00000174343	73.8162089	83.2851338	123.26868	125.617295	-0.662809959	0.0488197	0.1365369	CHRNA9	cholinergic receptor nicotinic alpha 9 subunit [Source:HGNC Symbol;Acc:HGNC:14079]
ENSG00000121749	718.477766	956.893027	718.540511	594.020769	0.352813836	0.0490939	0.1372809	TBC1D15	TBC1 domain family member 15 [Source:HGNC Symbol;Acc:HGNC:25694]
ENSG00000278709	240.148733	254.285462	184.376231	171.393089	0.475084416	0.0491666	0.1374614	NKILA	NF-kappaB interacting lncRNA [Source:HGNC Symbol;Acc:HGNC:51599]
ENSG00000198663	1718.44134	1738.35567	1526.63519	1395.62944	0.242348868	0.0492131	0.1375604	C6orf89	chromosome 6 open reading frame 89 [Source:HGNC Symbol;Acc:HGNC:21114]
ENSG00000162231	1637.73562	1579.75951	1829.01221	1959.84272	-0.235917701	0.0492183	0.1375604	NXF1	nuclear RNA export factor 1 [Source:HGNC Symbol;Acc:HGNC:8071]
ENSG00000261305	17.7158901	19.4922654	33.7145108	52.1631142	-1.205488384	0.0492398	0.1375978	AC005586.2	novel transcript, overlapping LRRC61
ENSG00000141447	782.451814	862.089736	638.468548	696.217891	0.301605384	0.0492755	0.137675	OSBPL1A	oxysterol binding protein like 1A [Source:HGNC Symbol;Acc:HGNC:16398]
ENSG00000149474	218.495978	217.072955	289.734077	299.139491	-0.435082994	0.0493779	0.1379381	KAT14	lysine acetyltransferase 14 [Source:HGNC Symbol;Acc:HGNC:15904]
ENSG00000198276	1109.21157	918.794508	1185.27577	1284.91589	-0.285182096	0.049399	0.1379742	UCKL1	uridine-cytidine kinase 1 like 1 [Source:HGNC Symbol;Acc:HGNC:15938]
ENSG00000173068	33.463348	32.7824463	65.3218647	61.7440943	-0.93990089	0.0494168	0.137993	BNC2	basonuclin 2 [Source:HGNC Symbol;Acc:HGNC:30988]
ENSG00000196235	2467.42981	2024.53756	2650.80341	2681.60989	-0.247845477	0.049422	0.137993	SUPT5H	SPT5 homolog, DSIF elongation factor subunit [Source:HGNC Symbol;Acc:HGNC:11469]
ENSG00000167733	71.8477766	51.3886996	22.1251477	38.3239206	1.026557491	0.0494751	0.1381184	HSD11B1L	hydroxysteroid 11-beta dehydrogenase 1 like [Source:HGNC Symbol;Acc:HGNC:30419]
ENSG00000230733	648.598422	555.529563	462.520945	488.629988	0.339624885	0.0495309	0.1382512	AC092171.2	novel transcript
ENSG00000275557	36.4159964	28.352386	10.5357846	13.8391936	1.408957479	0.0496491	0.1385583	AC242842.1	novel transcript
ENSG00000131409	39.3686447	37.2125066	13.69652	18.097407	1.268257382	0.0497201	0.1387177	LRRC4B	leucine rich repeat containing 4B [Source:HGNC Symbol;Acc:HGNC:25042]
ENSG00000122783	503.918653	519.203068	634.254234	640.861117	-0.317481549	0.0497226	0.1387177	CYREN	cell cycle regulator of NHEJ [Source:HGNC Symbol;Acc:HGNC:22432]
ENSG00000261116	7.87372895	8.86012062	33.7145108	18.097407	-1.629466902	0.0497319	0.1387205	AL049555.1	novel transcript, overlapping to FAM83B
ENSG00000185122	2603.25163	2146.80723	2764.58989	2863.64851	-0.245113302	0.0498041	0.138899	HSF1	heat shock transcription factor 1 [Source:HGNC Symbol;Acc:HGNC:5224]
ENSG00000198015	512.776598	698.177505	889.220222	682.378698	-0.375152572	0.0498409	0.1389787	MRPL42	mitochondrial ribosomal protein L42 [Source:HGNC Symbol;Acc:HGNC:14493]
ENSG00000166275	20.6685385	31.8964342	55.8396585	53.2276675	-1.049133271	0.0498705	0.1390383	BORCS7	BLOC-1 related complex subunit 7 [Source:HGNC Symbol;Acc:HGNC:23516]
ENSG00000113108	270.659433	254.285462	198.072751	183.103176	0.461320721	0.0499254	0.1391684	APBB3	amyloid beta precursor protein binding family B member 3 [Source:HGNC Symbol;Acc:HGNC:20708]
ENSG00000204287	24.605403	11.5181568	5.26789231	3.19366005	2.090042446	0.0499743	0.1392819	HLA-DRA	major histocompatibility complex, class II, DR alpha [Source:HGNC Symbol;Acc:HGNC:4947]

Table S4. The secondary metabolites in metabolomics analysis

name	ID	mz	rt	ppm	formula	KEGG	Ctrl_Mean	Ctrl_Median	Ctrl_RSD	VPA_Mean	VPA_Median	VPA_RSD	FC	log2FC	pvalue	padj
Triethylamine	M101T110	101.0959	109.8	4.165410833	C6H15N	C14691	8876995.15	5765730.5	101.4	4570379.87	6171311.12	66.19	1.94	0.96	0.595314697	0.754830727
Methyl isobutyl ketone	M101T93	101.0962	92.6	0.085005668	C6H12O	C19263	14334818.17	7261247.1	85.8	15457036.12	17121251.62	59.34	0.93	-0.11	0.838640231	0.911775885
Methylmalonic acid	M101T967	101.071	967	3.106656133	C4H6O4	C02170	12718270.2	13281245.95	53.4	13095780.32	9166349.19	94.94	0.97	-0.04	0.785076121	0.880113422
Choline	M104T788	104.1075	787.8	0	C5H14NO	C00114	8343833.35	3364005.99	109.78	7115487.1	8991581.33	54.26	1.17	0.23	0.904202058	0.950495847
gamma-Aminobutyric acid	M104T97	104.0707	97.1	0.065806245	C4H9NO2	C00334	14529151.39	13968277.36	12.89	15171310.33	13345356.96	27.05	0.96	-0.06	0.883297452	0.938890207
m-Cresol	M109T549	109.1008	548.6	6.079862577	C7H8O	C01467	2385265.5	2399385.3	11.07	1944643.85	1945080.04	11.59	1.23	0.29	0.093111186	0.27802583
1,3-Benzenediol	M110T160	110.035	159.5	16.35816381	C6H6O2	C01751	5864173.97	5829564.78	9.2	7686109.4	7453576.1	7.47	0.76	-0.39	0.016140286	0.125612599
Hydroquinone	M110T403	110.0198	402.6	3.207124345	C6H6O2	C00530	61838365.13	70786114.17	79.25	24487443.56	33316441.25	75.13	2.53	1.34	0.442352854	0.634241079
Catechol	M110T63	110.0193	62.7	8.845742529	C6H6O2	C00090	49624783.24	32994641.62	63.82	26127180.67	26601942.77	6.73	1.9	0.93	0.263382499	0.473015928
Pyrrole-2-carboxylic acid	M111T990	111.0192	989.7	9.37480806	C5H5NO2	C05942	819514311.7	821104191.7	9.33	708300498.9	739744465.9	10.9	1.16	0.21	0.156853941	0.35648192
Cytosine	M112T208	112.0506	208.4	0.214188993	C4H5N3O	C00380	1796313.87	1790322.28	7.61	4552708.46	5006563.69	24.76	0.39	-1.34	0.004914034	0.072058219
Heptanoic acid	M113T153	113.0016	153.4	11.12088011	C7H14O2	C17714	580946.35	596788.35	9.51	656122.71	644778.12	11.23	0.89	-0.18	0.230569362	0.438674138
Beta-Guanidinopropionic acid	M114T192	114.0654	191.7	29.22884591	C4H9N3O2	C03065	68755492.14	75787919.86	17.85	35229657.55	38473672.33	26.88	1.95	0.96	0.027493911	0.160908503
1-Pyrroline-5-carboxylic acid	M114T199	114.0549	199.1	0.666345769	C5H7NO2	C04322	8993717.47	8979769.39	11.11	14059908.8	12735428.66	31.11	0.64	-0.64	0.085890961	0.267145815
3-Methylindole	M114T307_2	114.0672	306.7	9.941508164	C9H9N	C08313	36127027.58	30999111.52	35.96	12994959.85	17537648.32	72.28	2.78	1.48	0.141713739	0.338926631
Epsilon-caprolactam	M114T418	114.0921	418.2	1.368055702	C6H11NO	C06593	25940476.6	26102561.24	5.44	14001603.96	14021307.48	22.91	1.85	0.89	0.010281093	0.103262208
Creatinine	M114T465	114.0657	464.8	4.173033611	C4H7N3O	C00791	18929819.57	18054745.71	37.01	28499009.15	31368191.03	18.53	0.66	-0.59	0.145219647	0.343194049
1-Pyrroline-2-carboxylic acid	M114T623	114.0673	623.1	8.164955785	C5H7NO2	C03564	49664815.62	55117722.97	19.76	30019192.24	21258926.69	60.8	1.65	0.73	0.158461627	0.358029636
Dihydrouracil	M115T127	115.0503	127.4	0.574050081	C4H6N2O2	C00429	18599967.72	15855745.18	29.14	17277229.81	15897891.49	18.31	1.08	0.11	0.772707112	0.873189458
L-Proline	M116T76	116.0702	76.3	3.239418903	C5H9NO2	C00148	20850173.18	20314697.75	15.26	18462841.07	18418323.12	1.04	1.13	0.18	0.321120633	0.529393833
Aminomalonic acid	M119T106	119.0163	105.6	0.86491042	C3H5NO4	C00872	4132925.04	4168013.2	12.37	3380364.11	3428631.46	9.76	1.22	0.29	0.096778233	0.282756672
2-Methylserine	M119T190	119.0491	190.2	1.482846069	C4H9NO3	C02115	33779870.98	30777659.35	17.03	26384541.96	25460322.45	10.66	1.28	0.36	0.097297356	0.283555118
L-Cysteine	M122T93	122.0269	92.6	0.622813494	C3H7NO2S	C00097	7394985.06	7469051.06	3.33	17577797.55	17448966.08	8.7	0.42	-1.25	8.74415E-05	0.014294298
Niacinamide	M123T132	123.0553	132	0	C6H6N2O	C00153	1061569515	1076514456	2.99	1187809808	1154419330	5.54	0.89	-0.16	0.036145549	0.181688094
4-Hydroxybenzaldehyde	M123T190	123.044	190.1	0.617665226	C7H6O2	C00633	57621418.34	52207928.94	16.68	45561132.62	44984222.92	8.22	1.26	0.34	0.092715999	0.277492226
L-Histidinol	M124T760	124.086	760.3	26.06256951	C6H11N3O	C00860	14852453.08	14026817.44	13.98	15201787.79	16112166.15	25.56	0.98	-0.03	0.975822316	0.987733841
3-Hydroxybenzyl alcohol glucoside	M124T921	124.086	921.1	8.251596764	C7H8O2	C03351	50352165.39	57038882.43	69.04	23505441.76	11610874.95	90.5	2.14	1.1	0.367003662	0.569341045
Picolinic acid	M124T967	124.0865	967.3	3.93390422	C6H5NO2	C10164	169512698.7	171084306	18.99	163804432.3	168227402.4	10.37	1.03	0.05	0.850855551	0.920122996
Ciliatine	M126T541	125.9852	540.7	10.52892926	C2H8NO3P	C03557	9439161.09	8517090.78	31.71	9744458.46	10561269.17	24.48	0.97	-0.05	0.867000054	0.929375969
Taurine	M126T72	125.9858	72.5	4.010267951	C2H7NO3S	C00245	286101769.8	294502678.9	25.42	202560861.5	231214200.4	27.41	1.41	0.5	0.206885299	0.413700331
Triacetate lactone	M127T230	127.0385	229.8	3.746895626	C6H6O3	C02752	11172589.77	13618633.8	55.87	11616033.96	10391203.65	18.6	0.96	-0.06	0.701093536	0.827338597
Thymine	M127T242	127.0503	242.4	0.975991399	C5H6N2O2	C00178	22981508.99	18303110.83	39.25	20665989.15	18861773.48	16.61	1.11	0.15	0.781989046	0.878132304
Dihydrothymine	M129T236	129.0661	235.8	1.73554481	C5H8N2O2	C00906	16970340.81	14166217.72	34.18	12559215.29	11084654.82	24.5	1.35	0.43	0.282342391	0.491365897
Pipecolic acid	M130T153	130.05	153.4	20.27465398	C6H11NO2	C00408	269114958.1	271257633.1	10.47	272768337.6	279186868.8	11.79	0.99	-0.02	0.899820743	0.947794011
Pyrrolidonecarboxylic acid	M130T419	130.0491	419.3	5.966977088	C5H7NO3	C02237	17676650.35	19461049.53	66.59	7881947.14	4469561.52	76.02	2.24	1.17	0.312253276	0.52101209
Mesaconate	M130T72_3	130.0169	72.4	0.051826193	C5H6O4	C01732	30960188.14	32573510.36	10.15	21356482.99	21262415.52	8.79	1.45	0.54	0.009372214	0.097909995
Ketoleucine	M131T548	131.0856	547.7	27.76779205	C6H10O3	C00233	660890.51	654640.96	2.71	663979.54	397900.33	76.7	1	-0.01	0.710629543	0.833474064
L-Leucine	M132T323	132.0999	323.3	14.9583762	C6H13NO2	C00123	25578867.8	25991247.81	14.27	12886199.41	8167535.02	109.87	1.98	0.99	0.26916312	0.478941554
D-allo-Isoleucine	M132T476	132.1002	476.2	12.52806675	C6H13NO2	C21092	29713914.32	24076699.27	41.17	26593579.32	31756372.96	66.25	1.12	0.16	0.639663001	0.786799523
N,N-Diethylglycine	M132T494	132.1002	493.7	12.68733885	C6H13NO2	C16647	24373631.22	19541351.29	41.05	14678598.71	14904965.3	84.59	1.66	0.73	0.326525036	0.535007626
cis-4-Hydroxy-L-proline	M132T517	132.0995	517.2	18.38546655	C5H9NO3	C01015	25354361.73	30107101.46	84.39	37304832.49	35812288.12	10.7	0.68	-0.56	0.416792123	0.612470641
Leucine	M132T561	132.1039	560.5	15.32127363	C6H13NO2	C16439	15589466.12	4606186.62	123.3	13862838.66	13716800.54	75.9	1.12	0.17	0.889597962	0.94129832
cis-4-Hydroxy-D-proline	M132T61_2	131.9727	60.8	12.50240077	C5H9NO3	C03440	15591045.67	14383257.59	28.6	7901056.16	7212098.34	43.06	1.97	0.98	0.073364783	0.248966763
N-Carbamoylputrescine	M132T744	132.0996	744	17.55310385	C5H13N3O	C00436	25606307.41	28380290.7	39.71	29506534.43	26930612.66	29.77	0.87	-0.2	0.600689597	0.758999893
(R)-Pantolactone	M132T767	131.5328	767.5	6.081019457	C6H10O3	C01012	22827637.79	15667184.37	111.8	17495618.28	12219457.47	112.55	1.3	0.38	0.827490422	0.906137796
L-Asparagine	M133T85	133.0609	85.4	0.931904113	C4H8N2O3	C00152	24193952.93	22349386.86	21.82	15662650.99	14572202.69	20.08	1.54	0.63	0.059051947	0.225173793
Iminodiacetic acid	M134T100	134.0449	100.2	0.746018685	C4H7NO4	C19911	103342503.4	54104934.21	84.42	77124588.44	77531279.73	2.7	1.34	0.42	0.884326655	0.939415071
Chavicol	M135T738	135.0805	738.1	0.066165694	C9H10O	C16930	2952378.5	3231713.75	16.71	2439834.7	2542218.15	16.44	1.21	0.28	0.251744694	0.462015862
4-Hydroxyphenylacetaldehyde	M136T237	136.0624	237	2.437293652	C8H8O2	C03765	8684394.39	8590970.18	10.75	9555786.57	7725710.96	34.04	0.91	-0.14	0.757255761	0.863727087
p-Aminobenzoic acid	M137T145	137.0459	144.6	0.256091295	C7H7NO2	C00568	4139001315	4132587283	2.67	6104278858	6088388474	2.86	0.68	-0.56	6.67036E-05	0.012566956
Hypoxanthine	M137T277_1	137.0459	277	0.656643023	C5H4N4O	C00262	2373411333	2385282096	10.86	1085316162	963571118.7	27.97	2.19	1.13	0.008483558	0.09335878
1-Methylnicotinamide	M137T90	137.071	90.1	0.72954892	C7H9N2O	C02918	77353815.22	78369762.72	2.53	53883284.57	54405850.7	3.66	1.44	0.52	0.000152001	0.017928354
Acetylphosphate	M140T212	139.981	211.7	7.579224973	C2H5O5P	C00227	54167690.44	63225052.67	37.81	41319344.15	45935573.12	31.62	1.31	0.39	0.485901234	0.670984758
O-Phosphoethanolamine	M142T370	141.9571	370.2	11.12448211	C2H8NO4P	C00346	25382461.45	23840521.17	26.33	62707042.61	68641047.26	27.14	0.4	-1.3	0.016686312	0.127742603
2-Naphthylamine	M144T398	144.0808	398.3	0.166573201	C10H9N	C02227	3240927.46	3126354.2	9.92	2743596.5	2672231.35	14.13	1.18	0.24	0.15862662	0.358084324
3-Hydroxymethylglutaric acid	M145T968	144.9822	967.5	0.477900797	C6H10O5	C03761	504323583.8	493214012.1	6.34	371013707.1	358216086	10.59	1.36	0.44	0.011513089	0.108627102
4-Guanidinobutanoic acid	M146T260	146.0815	260.4	1.7949366	C5H11N3O2	C01035	4867590.06	4955941.44	4.47	4365332.59	4397934.74	1.46	1.12	0.16	0.016902138	0.128599554
Spermidine	M146T68	146.1653	68.5	0.130365449	C7H19N3	C00315	41308447.89	38095194.8	13.84	57990550.6	58269748.58	3.94	0.71	-0.49	0.012799687	0.113390047
Coumarin	M147T190	147.0444	190.1	0.477187346	C9H6O2	C05851	67033654.21	60823173.86	16.12	52746947.82	51070021.56	10.24	1.27	0.35	0.092458969	0.27696818
L-Lysine	M147T77_2	147.1129	77	0.001493594	C6H14N2O2	C00047	208493181.2	190343999.1	21.1	149367887.8	156100631.2	8.66	1.4	0.48	0.065209537	0.235281845
4-Hydroxycinnamic acid	M147T928	146.9813	928.5	8.769916119	C9H8O3	C00811	34346041.42	27948383.23	68.47	68502716.14	80499312.98	79.17	0.5	-1	0.663531193	0.803814794
L-2-Hydroxyglutaric acid	M148T128	148.0427	128.1	0.606794817	C5H8O5	C03196	20343017.59	20026332.77	7.08	16264210.57	21459489.66	59.83	1.25	0.32	0.485890179	0.670984758
3,4-Dihydro-2H-1-benzopyran-2-one	M149T340	149.06	339.8	1.975741312	C9H8O2	C02274	47702200.53	43414337.46	16.44	38614306.35	38221766.79	9.29	1.24	0.3	0.123523389	0.317087181
3-Methyladenine	M149T443	149.023	443.4	0.004306933	C6H7N5	C00913	20062293.06	16078169.14	96.88	12976152.78	10156128.9	39.66	1.55	0.63	0.992834492	0.995879218
(S)-4-Hydroxymandelate	M151T72	151.0353	72.4	2.873500433	C8H8O4	C03198	463441302	491190166.1	10.46	315741675.1	315199002.1	8.79	1.47	0.55	0.008969932	0.096123803

Guanine	M152T277	152.0571	277.3	2.630590745	C5H5N5O	C00242	37251838.26	37100942.48	4.24	49959019.99	48349415.98	7.93	0.75	-0.42	0.004650976	0.069993464
Loratadine	M152T462	152.1438	462.4	2.629091688	C10H17N	C06818	859842.19	976887.04	47.04	613652.72	566749.76	77.54	1.4	0.49	0.47523744	0.661925817
Xanthine	M153T318	153.041	318.4	1.960259016	C5H4N4O2	C00385	16032281.21	11610644.79	54.63	25045333.88	26376510.43	27.52	0.64	-0.64	0.205725954	0.412578434
2,3-Butanediol	M155T64_2	154.988	64.1	14.0559787	C4H10O2S2	C00265	31879396.57	30578338.82	9.68	28705351.14	30883572.42	15.14	1.11	0.15	0.361577698	0.565032879
Gentisic acid	M155T666	154.9923	665.9	14.50865926	C7H6O4	C00628	108839015.3	110779265.8	39.55	38428156.98	33582215.92	54.58	2.83	1.5	0.054931897	0.218539239
Uracil 5-carboxylate	M157T32	156.9646	31.8	8.260502336	C5H4N2O4	C03030	10257703	11401427.64	19.67	9948823.73	9366947.39	10.71	1.03	0.04	0.889572685	0.94129832
Phosphoglycolic acid	M157T765	156.9925	765.1	17.98812045	C2H5O6P	C00988	77767468.18	92229370.53	72.49	83266374.69	111795486.6	69.2	0.93	-0.1	0.934173427	0.966641074
Ascorbate	M159T145	159.0281	145.1	21.59366804	C6H8O6	C00072	8527546.69	8661819.93	3.38	10637342.56	10454404.92	4.04	0.8	-0.32	0.001897524	0.047941043
Serotonin	M159T399	159.0919	398.6	27.24211603	C10H12N2O	C00780	28555406.96	26718664.51	17.81	24574610	23481315.25	10.24	1.16	0.22	0.280843828	0.490206949
4,5-Dihydroorotic acid	M159T763	158.959	763.3	13.91653321	C5H6N2O4	C00337	17317043.71	14160223.64	34.98	7865432.63	5900389.53	68.93	2.2	1.14	0.104286675	0.293142897
N-methyl-L-glutamic Acid	M162T122	162.076	121.7	0.01510397	C6H11NO4	C01046	39490251.68	37768249.02	11.04	42930613.09	42828776.77	15.79	0.92	-0.12	0.51533987	0.694373151
L-Rhamnono-1,4-lactone	M162T282	162.0585	281.6	2.246186852	C6H10O5	C02991	158224291.4	135546425.3	37.61	143782262.4	133004873.7	17.9	1.1	0.14	0.800922705	0.890534967
Anabasine	M162T91_1	162.1124	91.2	20.35624665	C10H14N2	C06180	1060442338	1073233419	5.76	878062624.6	871377717.1	4.94	1.21	0.27	0.012791447	0.113390047
Nicotine	M163T142	163.1231	141.8	0.61303396	C10H14N2	C00745	61448993.65	54954885.94	34.6	43553889.2	43438232.17	19.66	1.41	0.5	0.228552085	0.436663646
2-Deoxystreptamine	M163T717	163.1118	717.1	0.007034813	C6H14N2O3	C02627	3295027	3218627.92	10.85	3104987.22	3287263.65	12.96	1.06	0.09	0.570221544	0.735054867
Pterin	M164T191	164.057	191.5	0.730754963	C6H5N5O	C00715	11948411.02	11941340.14	7.98	11823832.36	12300097.17	7.81	1.01	0.02	0.880566215	0.937673707
Acetylcysteine	M164T289	164.038	289.1	2.584766944	C5H9NO3S	C06809	11374701.1	11248903.55	25.03	15572549.38	14980167	19.28	0.73	-0.45	0.152494218	0.351462012
3,4-Dihydroxyphenylpropanoate	M165T190	165.0543	190.1	23.22871928	C9H10O4	C10447	677100677.1	618045109.4	16.65	542836551.1	526071093.5	9.05	1.25	0.32	0.111698892	0.303503716
Phenylpyruvic acid	M165T342	165.058	341.8	20.74465357	C9H8O3	C00166	5383053	5705267.09	13.64	8681058.33	8503409.2	20.67	0.62	-0.69	0.031558511	0.172247591
L-Methionine S-oxide	M166T134	166.0534	133.7	0.746747733	C5H11NO3S	C02989	29525176.82	31654318.87	41.37	37241851.75	38469212.09	16.23	0.79	-0.33	0.369016465	0.570904919
7-Methylguanine	M166T324	166.0725	324	0.005550642	C6H7N5O	C02242	928133.11	645349.76	55.67	1588104.27	1994897.45	49.76	0.58	-0.77	0.337134324	0.544175006
L-Phenylalanine	M166T402	166.085	402.4	6.148259457	C9H11NO2	C00079	24194381.72	16184512.03	58.52	7456139.73	3861355.13	86.35	3.24	1.7	0.078675284	0.256656452
Quinolinic acid	M167T666	167.0147	665.9	9.655285463	C7H5NO4	C03722	18127302.37	20834331.45	59.75	14182298.11	17474693.55	54.58	1.28	0.35	0.743026873	0.854742226
3-(2-Hydroxyphenyl)propanoic acid	M167T819	167.0703	819.5	0.004081545	C9H10O3	C01198	5250049.84	5458402.94	10.28	4939604.17	4971482.8	14.78	1.06	0.09	0.574166976	0.738040079
3-Methoxyanthranilate	M168T128	168.0656	127.6	0.468625144	C8H9NO3	C05831	38271000.89	37952432.19	15.83	23884004.82	22877773.62	17.1	1.6	0.68	0.023904058	0.151566662
8-Amino-7-oxononanoate	M170T485	170.1178	484.8	26.06429192	C9H17NO3	C01092	10888645.57	13156134	40.79	10163387.34	9094866.82	53.25	1.07	0.1	0.831931632	0.908583152
3-Dehydroshikimate	M172T43	171.991	43.2	11.79790159	C7H8O5	C02637	3046993.91	1397741.02	94.27	3863201.45	4238507.62	33.9	0.79	-0.34	0.441942272	0.633845341
Gabapentin	M172T516	172.1334	515.7	0.04453532	C9H17NO2	C07018	46851896.92	42494187.4	32.2	39182893.54	42672030.89	43.53	1.2	0.26	0.550790124	0.721297752
4-Quinolinecarboxylic acid	M173T101	172.9564	100.6	6.649356878	C10H7NO2	C06414	33764270.71	27014469.93	34.94	36005162.95	38148797.74	11.84	0.94	-0.09	0.656037744	0.798351017
Iminoarginine	M173T108	173.092	108.4	0.014572563	C6H12N4O2	C21026	14025966.73	14237689.87	5.9	9726515.34	9080749.04	24.86	1.44	0.53	0.055701443	0.219653212
2-Oxoarginine	M173T299	173.0788	299.5	6.933210076	C6H11N3O3	C03771	4451812.75	4237094.04	23.96	2814243.36	2711792.24	10.04	1.58	0.66	0.039915536	0.189862829
N-Acetylleucine	M174T498	174.1125	498.5	0	C8H15NO3	C02710	3303155.72	3994005.26	37	2585979.54	1909307.34	48.59	1.28	0.35	0.511092447	0.691498743
N-Acetyl-L-aspartic acid	M176T135	176.0555	134.6	0.194834913	C6H9NO5	C01042	202961491.8	196849306.7	6.64	136085594.2	132034197.3	7.51	1.49	0.58	0.002155252	0.049809806
D-Alanyl-D-serine	M176T383	176.0744	382.9	1.624129675	C6H12N2O4	C19719	127529367.5	118709676.3	28.3	157843354.5	165794386	26.03	0.81	-0.31	0.396310807	0.594239113
Rimantadine	M180T689	180.1746	689.1	0.419992607	C12H21N	C07236	1523510.04	1429922.54	28.11	1601644.2	1904511.43	39.96	0.95	-0.07	0.980240164	0.989805441
L-Tyrosine	M182T190_2	182.0812	190.1	0.131809325	C9H11NO3	C00082	3958343993	3572255534	17.24	3128383350	3061474629	10.17	1.27	0.34	0.109345282	0.299995005
Mannitol	M183T341	182.9846	341.4	0.405796603	C6H14O6	C00392	50740396.84	35482499.79	80.06	85724091.43	99420732.6	61.53	0.59	-0.76	0.455883269	0.645816349
Sorbitol	M183T381	182.9848	381.2	0.003187106	C6H14O6	C00794	107371359.4	71829352.06	97.59	42364879.33	55088895.7	57.28	2.53	1.34	0.404800449	0.60167941
Se-Methylselenocysteine	M183T420	182.9826	420.2	14.55850433	C4H9NO2Se	C05689	23668123.35	19468465.39	61.5	40156684.07	35112704.92	57.13	0.59	-0.76	0.331724964	0.539325019
4-Pyridoxic acid	M183T577	182.9819	577.3	18.89993467	C8H9NO4	C00847	16467077.51	17573476.71	37.6	9936454.59	9184452.36	22.19	1.66	0.73	0.160722646	0.361040216
Phosphorylcholine	M184T101	184.0728	100.6	0.827229813	C5H15NO4P	C00588	115306698.6	135348228.3	59.09	122758686.8	158774103.7	57.1	0.94	-0.09	0.922324438	0.960437458
3,4-Dihydroxymandelic acid	M185T420	184.983	420.3	13.8671421	C8H8O5	C05580	8643435.58	9692811.19	49.26	5709212.78	3339021.7	77.61	1.51	0.6	0.410917843	0.606873496
Undecanoic acid	M187T107	186.9567	107	1.299819445	C11H22O2	C17715	63024825.84	65360703.94	41.72	66083651.81	65863005.03	5.97	0.95	-0.07	0.714542848	0.835857776
Glycylleucine	M189T300_2	189.1236	299.7	1.184410618	C8H16N2O3	C02155	19022347.73	20091434.61	13.71	4660034.98	3497006.84	46.86	4.08	2.03	0.005075301	0.07347529
Isocitric acid	M192T110	192.0325	110.3	0.363921289	C6H8O7	C00311	90634707.36	87249353.42	9.17	140798179.2	141443077.3	2.54	0.64	-0.64	0.001192774	0.039127068
5,6-Dihydroxy-3-methyl-2-oxo-1,2,5,6-tetrahydroquinoline	M193T168	193.0678	168.3	1.158815406	C10H11NO3	C06726	625031813.9	615396032.1	3.11	369037843.6	360677204.3	9.65	1.69	0.76	0.000788438	0.032835656
3,4-Methylenedioxyamphetamine	M194T356	194.1156	356.3	0.201186726	C11H15NO2	C07577	20564711.82	13130118.8	78.66	19656039.16	19079515.37	63.06	1.05	0.07	0.983654546	0.991867461
gamma-Glutamyl-beta-aminopropiononitrile	M200T312	200.0918	311.6	0.549430427	C8H13N3O3	C06114	9860013.92	9869084.6	7.18	7183402.05	7064470.76	3.08	1.37	0.46	0.002213722	0.050370188
Dodecanoic acid	M200T647	200.1648	646.7	3.989388524	C12H24O2	C02679	9721599.77	9902013.89	10.51	8457950.4	8955718.81	14.68	1.15	0.2	0.255715176	0.465998018
Thiabendazole	M202T400	202.0446	400.3	0.245721994	C10H7N3S	C07131	1936258579	1943269464	4.26	1552988603	1585395450	4.15	1.25	0.32	0.003103131	0.058490175
N-Acetyl-D-glucosamine	M204T150	204.0863	149.6	18.78617036	C8H15NO6	C00140	114188030.1	119787972.6	15.46	105488228.3	102431904.4	7.06	1.08	0.11	0.514136309	0.693708215
L-Tryptophan	M204T859	204.1388	859.1	0.595919198	C11H12N2O2	C00078	7873039.2	6882868.6	27.64	9029355.09	8525623.75	9.75	0.87	-0.2	0.382254403	0.582320408
D-Ribose 5-phosphate	M213T112	213.0153	111.9	16.59035759	C5H11O8P	C00117	43125022.39	43437966.2	6.62	46085758.3	46159617.35	0.96	0.94	-0.1	0.218025013	0.425757505
N-a-Acetylcitrulline	M217T354	217.1056	354.2	3.224237422	C8H15N3O4	C15532	5724706.24	5463763.38	18.46	3785987.55	3886025.45	5.27	1.51	0.6	0.020742377	0.142290414
Propionylcarnitine	M218T276	218.139	275.9	1.448214212	C10H19NO4	C03017	1044484885	1175206322	24.87	429866666.6	395328981.2	39.17	2.43	1.28	0.029070318	0.166085876
gamma-Glutamylalanine	M219T111	219.0972	110.7	1.369255289	C7H11N2O5R	C03740	8475251.91	10174484.43	46.89	18437927.51	12352122.58	57.61	0.46	-1.12	0.162182523	0.362888211
D-Lysopine	M219T114	219.134	114	0.057376956	C9H18N2O4	C04020	5323732.58	5071451.63	12.66	3184380.35	3092549.63	5.67	1.67	0.74	0.002854283	0.056964719
1D-1-Guanidino-1-deoxy-3-dehydro-scyllo-inositol	M219T352	219.0799	352.4	25.56079704	C7H13N3O5	C04673	4629835.3	3713137.56	36.48	7325890.48	7921240.38	22.1	0.63	-0.66	0.114386068	0.306153604
Capsidiol	M219T692	219.1743	692.1	4.22085E-05	C15H24O2	C09627	2296165.54	2301596.89	8.37	2023750.05	1812520.02	27.47	1.13	0.18	0.406154094	0.602705035
5-Hydroxy-L-tryptophan	M220T218	220.0819	218.1	13.17691278	C11H12N2O3	C00643	7655945.22	7756045.77	4.49	6337081.21	6294467.14	5.08	1.21	0.27	0.00850717	0.093487564
1D-1-Guanidino-3-amino-1,3-dideoxy-scyllo-inositol	M220T357_2	220.1173	357	0.454303228	C7H16N4O4	C01298	1529240714	1549138947	8.86	1362168737	1420926774	10.59	1.12	0.17	0.2251557	0.433169743
N-Acetylmannosamine	M222T98	222.0976	97.7	1.909070607	C8H15NO6	C00645	13693185.86	12752489	12.4	10032428.17	10547338.84	10.14	1.36	0.45	0.02812384	0.162743559
Hydroxykynurenine	M224T306	224.1285	306.3	0.978725829	C10H12N2O4	C02794	12347563.11	10028313.92	34.29	8634646.77	7787315.94	23.06	1.43	0.52	0.205896989	0.412656024
Cerulenin	M224T668	224.1261	667.9	8.015567513	C12H17NO3	C12058	25653706.46	23786022.62	23.98	13097552.56	15945013.08	42.28	1.96	0.97	0.087330895	0.26891247
Methyl jasmonate	M224T777	223.9886	777	3.170781138	C13H20O3	C11512	216831033.8	245934234.3	39.77	205810203.3	170007516.5	35.51	1.05	0.08	0.941139885	0.970395984
Porphobilinogen	M226T873	226.1803	873.2	0.800056552	C10H14N2O4	C00931	29229699.7	28044667.84	42.09	47399972.28	30849520.88	69.84	0.62	-0.7	0.428085614	0.621389066
Deoxyuridine	M228T776	228.1957	776.2	0.018972733	C9H12N2O5	C00526	144946604.4	143654380.6	3.46	118153575.1	118861430.3	2.13	1.23	0.29	0.000946055	0.035590401

Butyryl-L-carnitine	M232T380_2	232.1546	379.5	0.008833687	C11H21NO4	C02862	905810732.4	919738345.6	9.79	253859402.7	255838843.4	4.94	3.57	1.84	3.84388E-05	0.010404993
4a-Carbinolamine tetrahydrobiopterin	M239T282	239.1026	281.6	0.128042246	C9H13N5O3	C00268	7516451.17	7771857.99	24.88	1115133.31	841067.91	45.18	6.74	2.75	0.002373226	0.052135705
N(alpha)-gamma-L-Glutamylhistamine	M241T479	241.1088	478.5	1.667317837	C10H16N4O3	C04138	1222338.66	1216448.06	2.34	1098210.76	1126028.94	5.3	1.11	0.15	0.033361463	0.176431195
Equol	M242T357	242.0996	357	19.75172518	C15H14O3	C14131	82289810.04	81161109.19	9.39	79313096.89	79468370.4	5.87	1.04	0.05	0.611033993	0.765987998
Uridine	M245T171	245.0765	170.8	1.126178969	C9H12N2O6	C00299	224165623.8	231505300	5.99	314954265.9	315626960.5	7.65	0.71	-0.49	0.003896228	0.064480793
Pyridoxal 5'-phosphate	M248T146	248.0322	145.7	0.077465212	C8H10NO6P	C00018	8816798.06	9036210.48	4.83	5542230.86	5468656.19	8.21	1.59	0.67	0.001059989	0.037374152
Alprenolol	M250T743	250.1769	743.2	13.01074855	C15H23NO2	D07156	141772072	142036765.9	7.88	111559243.4	111931697	1.62	1.27	0.35	0.006982839	0.085221777
(5-L-Glutamyl)-L-glutamate	M259T106	259.0916	106.1	12.48207198	C10H16N2O7	C05282	4123937.62	4170827.21	4.72	6840033.63	6636970.68	10.07	0.6	-0.73	0.001364886	0.041474911
Adenosine	M268T237_2	268.1043	236.6	0.835495738	C10H13N5O4	C00212	230299389.2	211524074.2	18.16	294456046.5	205802730	55.47	0.78	-0.35	0.626428896	0.777178406
Alpha-dimorphecolic acid	M279T277	279.2363	276.8	13.96666551	C18H32O3	C14767	8753702.02	8272516.26	14.99	4275575.13	4237156.75	6.86	2.05	1.03	0.001564123	0.044113895
13S-hydroxyoctadecadienoic acid	M279T93	279.231	92.9	11.9400776	C18H32O3	C14762	17074942.2	22190245.14	60.8	9667023.22	10869200	50.48	1.77	0.82	0.479426979	0.665691461
Linoleic acid	M280T939	280.2632	938.7	1.328038066	C18H32O2	C01595	12232525.95	12648636.19	27.69	10903995.17	10632198.98	27.7	1.12	0.17	0.65305931	0.796431703
1-Methyladenosine	M282T128	282.1185	127.6	3.63613981	C11H15N5O4	C02494	25807431.44	27576146.72	28.17	46596594.22	45207972.18	5.68	0.55	-0.85	0.025780272	0.156703226
(6Z)-Octadecenoic acid	M282T761	282.2792	761.2	2.575817166	C18H34O2	C08363	206582474.7	217487234.5	22.12	284144523.5	236949455.1	42.6	0.73	-0.46	0.357335545	0.561428519
Oleic acid	M282T811	282.2791	811.5	0.099002909	C18H34O2	C00712	17396567.18	8321846.41	118.84	20092836.9	21143771.04	62.2	0.87	-0.21	0.582467122	0.744725594
Oleamide	M282T955	282.2792	955.2	0.085022205	C18H35NO	C19670	8939235.38	3542317.05	125.14	11966801.45	13571033.23	53.77	0.75	-0.42	0.431365643	0.624482459
Vaccenic acid	M283T68	283.2624	68.1	2.739501869	C18H34O2	C08367	11278930.07	11346262.41	5.45	23167068.97	28789796.76	54.05	0.49	-1.04	0.295918002	0.504706424
Stearic acid	M284T442	284.1861	442.2	4.07590657	C18H36O2	C01530	14935501.5	15944513.64	15.4	21294964.47	19161577.16	18.14	0.7	-0.51	0.061677736	0.229537087
Octadecanamide	M284T924	284.294	924.5	2.729568686	C18H37NO	C13846	41924092.62	41570587.8	38.91	47707319.83	51190181.49	40.36	0.88	-0.19	0.752476673	0.860497526
Fisetin	M286T780	286.2732	779.9	4.987143564	C15H10O6	C10041	12127301.89	11897708.74	27.48	12181482.36	11136652.57	44.07	1	-0.01	0.912768936	0.955259274
Dehydroepiandrosterone	M288T750	288.2905	750.4	0.718883047	C19H28O2	C01227	48087600.75	49145051.18	45.55	63545892.52	71544097.15	32.96	0.76	-0.4	0.433087777	0.626141393
Aurin	M291T574	291.1008	574.3	2.665743275	C19H14O3	C14213	3615225.94	3596757.13	16.26	2441947.06	2480016.8	8.93	1.48	0.57	0.023216648	0.149426635
13(S)-HOT	M295T738	295.2255	738.1	4.322119871	C18H30O3	C16316	12525346.09	11732120.04	26.42	6135009.66	5866693.64	48.87	2.04	1.03	0.081253156	0.26037717
Sumatriptan	M296T381	296.1348	381.3	13.07470449	C14H21N3O2S	C07319	1860898.54	1778245.36	27.49	2415802.32	2427149.29	5.54	0.77	-0.38	0.151661824	0.350607239
Tridemorph	M298T926	298.3099	925.6	1.930877922	C19H39NO	C11285	2855515.97	1131870.18	117.42	16768928.51	17473757.24	17.04	0.17	-2.55	0.078369448	0.256307814
N-Acetyl-alpha-D-glucosamine 1-phosphate	M302T93	302.0628	92.6	0.522287657	C8H16NO9P	C04501	12044620.82	11035134.36	18.08	14941858.7	15029149.22	2.18	0.81	-0.31	0.151144829	0.349758349
Glutathione	M308T138	308.0909	137.5	0.342047133	C10H17N3O6S	C00051	13621215254	14182148478	8.94	17230293488	17664517464	4.82	0.79	-0.34	0.01689646	0.128599554
Phytosphingosine	M318T786	318.2994	786.1	2.733614955	C18H39NO3	C12144	5923051.75	6392112.83	17.11	4770434.31	4969099.82	25.63	1.24	0.31	0.289640293	0.498576383
S-(Hydroxymethyl)glutathione	M320T169	320.0911	169.4	12.91524004	C11H19N3O7S	C14180	40652162.37	19091743.81	96.41	107000357.5	102315185.4	7.81	0.38	-1.4	0.136275273	0.332499241
gamma-L-Glutamyl-L-cysteinyl-beta-alanine	M322T185_2	322.0769	185.4	0.026970832	C11H19N3O6S	C04544	894413857.3	884328315.3	2.17	1469781966	1448533300	5.09	0.61	-0.72	9.7114E-05	0.014709936
Nicotinamide ribotide	M335T101	335.0635	101.1	1.122175349	C11H15N2O8P	C00455	11059861.17	9133505.22	36.76	6922757.83	8744480.79	47.49	1.6	0.68	0.254105665	0.464358532
12-Keto-tetrahydro-leukotriene B4	M336T937	336.3107	936.7	2.332740436	C20H32O4	C02165	10332939.43	10664665.36	10.85	12040876.42	11211193.64	13.68	0.86	-0.22	0.204927492	0.411567763
Erucic acid	M338T974_1	338.3414	974	1.389985745	C22H42O2	C08316	366734910	173781476.8	119.05	822263301	923892737.1	59.07	0.45	-1.16	0.247252663	0.457048682
Fructose 1,6-bisphosphate	M341T937	341.2652	936.8	5.180057732	C6H14O12P2	C00354	13087154.31	12218476.77	29.56	8794411.08	8531755.3	40.77	1.49	0.57	0.22384024	0.432049639
AMP	M348T122_1	348.0692	122.3	3.378638501	C10H14N5O7P	C00020	3627045831	3539208285	10.13	4343849989	4488579166	12.52	0.83	-0.26	0.131214167	0.325993631
S-(Formylmethyl)glutathione	M350T325	350.1008	325	2.502130267	C12H19N3O7S	C14871	1781453.95	2206506.89	49.83	3603746.99	3584973.32	2	0.49	-1.02	0.155298001	0.354564245
Prostaglandin F2a	M355T967	355.2806	967.4	4.650429207	C20H34O5	C00639	15641463.92	15183648.89	14.29	13702245.03	12777276.83	19.95	1.14	0.19	0.37274874	0.574003323
Aldosterone	M361T875	361.2195	875.3	7.808428507	C21H28O5	C01780	11831455.24	11630969.14	23.93	13666617.93	13533803.4	4.73	0.87	-0.21	0.315603536	0.523434815
S-Lactoylglutathione	M362T336_1	362.1007	336.4	8.931296967	C13H21N3O8S	C03451	5391769.15	5518306.76	10.23	13850395.88	12968016.71	12.69	0.39	-1.36	0.000538031	0.028219663
GMP	M364T151	364.0734	151.4	22.24826093	C10H14N5O8P	C00144	12543333.76	12397309.93	8.45	5296154.74	4665315.28	50.01	2.37	1.24	0.032371948	0.174333265
Xanthylic acid	M365T133	365.0491	133.5	0.482126692	C10H13N4O9P	C00655	3881102.85	1509354.54	134.4	38180793.36	39368822.07	54.05	0.1	-3.3	0.049686222	0.208398036
Lathosterol	M369T643	369.3613	642.8	24.90786122	C27H46O	C01189	1822399.3	1916125.75	12.28	2337989.63	1569177.03	79.87	0.78	-0.36	0.923123794	0.960840088
5a-Cholest-8-en-3b-ol	M369T68	369.3487	68.4	3.341027389	C27H46O	C03845	40025322.77	51214923.06	64.56	15291824.82	15289470.22	4.43	2.62	1.39	0.319910842	0.528249918
Tetracosanoic acid	M369T729	369.3624	728.5	27.8201412	C24H48O2	C08320	2147065.34	1767990.04	41.93	4129444.78	2420790.3	96.18	0.52	-0.94	0.551611463	0.721811966
Cholesterol	M369T972	369.3604	972.3	22.47127738	C27H46O	C00187	61538723.21	84356817.15	68.98	127423891.4	81241455.82	66.51	0.48	-1.05	0.28559295	0.494461396
N-Acetyllactosamine	M384T98	384.1488	98.4	3.321618502	C14H25NO11	C00611	7096139.22	6898267.03	11.44	7572342.28	7598890.11	4.02	0.94	-0.09	0.376307231	0.577139435
7-Dehydrocholesterol	M385T318_2	385.3388	317.6	19.92013262	C27H44O	C01164	6777251.57	6355468.61	40.36	8664000.29	8231465.73	32.86	0.78	-0.35	0.432016816	0.625091915
Vitamin D3	M385T937	385.2925	936.7	2.347673562	C27H44O	C05443	7209035.33	7382583.47	38.1	15876316.7	12793320.04	55.02	0.45	-1.14	0.126153532	0.320053294
S-Hexyl-glutathione	M391T916_1	391.288	915.5	10.38616944	C16H29N3O6S	C02886	20133679.24	8653168.31	107.74	139569275.8	124084605.6	37.16	0.14	-2.79	0.023187263	0.149358599
Calcitriol	M399T958_2	399.3131	958	21.20140812	C27H44O3	C01673	24035776.44	24093945.29	12.57	13097003.68	8017162.64	79.93	1.84	0.88	0.141872129	0.338989095
20a,22b-Dihydroxycholesterol	M401T932_1	401.3411	932.1	2.24248152	C27H46O3	C05501	1361703.02	76390.87	163.52	25029043.79	22771040.49	26.82	0.05	-4.2	0.075275711	0.251776692
UDP	M405T132_1	405.0074	131.9	3.824786695	C9H14N2O12P2	C00015	58252672.65	56548526.17	17.75	91889535.89	93493091.64	55.72	0.63	-0.66	0.430294013	0.623552848
Sodium deoxycholate	M415T810	415.2127	810	5.591183244	C24H39O4. Na	C11171	5277981.33	5257419.81	1.77	3985416.34	4154515.71	15.18	1.32	0.41	0.084770246	0.265815424
S-Glutathionyl-L-cysteine	M427T97	427.0935	97	3.924183869	C13H22N4O8S2	C05526	3711990.41	3960299.44	16.44	6372440.07	6081764.78	23.88	0.58	-0.78	0.034590033	0.178836114
ADP	M428T159	428.0298	158.9	16.06430206	C10H15N5O10P2	C00008	4683181.48	4481385.43	8.81	11229794.95	13259039.63	34.59	0.42	-1.26	0.024548917	0.153303263
alpha-Tocopherol	M430T539_2	430.2414	538.8	3.223352792	C29H50O2	C02477	16844928.24	16076697.19	31.87	26514087.11	26246220.59	10.65	0.64	-0.65	0.067683262	0.239297661
Isovitexin	M432T799_2	432.246	799.1	16.16969073	C21H20O10	C01714	16041398.44	8994536.56	95.91	7026790.25	3758858.96	95.36	2.28	1.19	0.343338749	0.549183423
GDP	M444T157	444.0291	157.5	5.576180015	C10H15N5O11P2	C00035	15387655.26	17213064.81	32.77	31205411.31	30939207.42	8.8	0.49	-1.02	0.027004987	0.159449822
Folinic acid	M474T378_2	474.1694	378.2	7.963398735	C20H23N7O7	C03479	7159398.63	6220513.4	35.37	2485251.53	2663508.77	23.04	2.88	1.53	0.012583389	0.113053841
Antibiotic JI-20A	M482T805	482.3179	804.8	13.08968851	C19H39N5O9	C17704	2571036.58	1992772.47	69.59	12050287.74	13890375.1	61.02	0.21	-2.23	0.070188394	0.243624713
UDP-N-acetyl-D-mannosamine	M608T261_2	608.0963	260.7	12.08476697	C17H27N3O17P2	C01170	77483370.95	45113455.21	109.15	64674475.55	30439055.52	96.25	1.2	0.26	0.990334376	0.994841321
Rutin	M610T959	610.1788	959	8.154986702	C27H30O16	C05625	25979424.21	26510244.05	4.64	39230927.39	43582058.62	50.29	0.66	-0.59	0.479036904	0.665555009
NADP	M744T117_2	744.0905	117	9.676242339	C21H29N7O17P3	C00006	23347010.73	22226602.51	8.38	25696228.84	24829319.37	10.68	0.91	-0.14	0.286281038	0.495238037
FAD	M786T401_2	786.1589	400.8	6.965512952	C27H33N9O15P2	C00016	8813538.35	8384669.72	10.75	9943680.34	10285310.68	7.24	0.89	-0.17	0.17321904	0.37559234
2-Ketobutyric acid	M101T101	101.0239	100.8	4.949323873	C4H6O3	C00109	7718409.26	8826102.92	34.3	9262190.68	8525645.84	22	0.83	-0.26	0.452255597	0.758931224
Hypotaurine	M108T87	108.0126	86.7	1.629439528	C2H7NO2S	C00519	19335732.75	22073299.58	25.13	24055737.32	23404178.4	6.71	0.8	-0.32	0.215563692	0.615984909
Uracil	M112T129_1	112.0235	129.1	10.77371427	C4H4N2O2	C00106	2001128.56	612786.83	134.17	7001568.97	9477588.36	72.59	0.29	-1.81	0.223536477	0.621869044

2-Heptanone	M113T403	112.9842	402.9	6.014046097	C7H14O	C08380	1985262.35	1591300.87	42.97	1597240.83	1269288.5	37.7	1.24	0.31	0.543064482	0.804021132
Maleic acid	M115T133	115.0038	133.1	0.660847728	C4H4O4	C01384	2834334.13	3315393.14	53.32	2610817.35	2366854.07	17.36	1.09	0.12	0.924771911	0.976033449
Fumaric acid	M115T79	115.0039	78.6	1.530382883	C4H4O4	C00122	55635419.08	60120369.13	33.55	58132154.23	55972013.37	13.58	0.96	-0.06	0.74022023	0.899359655
Caproic acid	M115T870	114.9339	869.7	7.443986552	C6H12O2	C01585	98609730.07	123854469	48.38	58878151.07	32081509.24	82.92	1.67	0.74	0.336291848	0.697582541
L-Valine	M116T109	116.0717	109.4	0.206768747	C5H11NO2	C00183	34712563.85	36448182.95	16.01	42688794.31	42205765.49	13.67	0.81	-0.3	0.167646736	0.578199373
5-Aminopentanoic acid	M116T167	116.071	167.2	5.654866004	C5H11NO2	C00431	9209413.89	6386038.54	67.7	4781732.09	4980472.08	48.53	1.93	0.95	0.279836468	0.664346403
Betaine	M116T198	116.0709	198.1	6.892339079	C5H11NO2	C00719	6359822.2	7052923.78	56.72	4667557.79	3870402.14	53.95	1.36	0.45	0.641878029	0.8546565
L-Norvaline	M116T250	116.0708	250.1	1.909653866	C5H11NO2	C01826	2414506.77	2875542.79	79.03	8176946.75	7520044.41	41.66	0.3	-1.76	0.125967268	0.550950746
trans-1,2-Cyclohexanediol	M116T947	115.9215	947.2	15.3907485	C6H12O2	C03739	21144644.48	18873247.53	58.97	28713643.04	28581031.35	7.92	0.74	-0.44	0.355141785	0.707672298
Succinic acid	M117T113	117.0193	112.7	0.205094373	C4H6O4	C00042	3729546.08	3084130.59	31.8	3841550.75	3968458.03	32.2	0.97	-0.04	0.934922283	0.978853762
Guanidoacetic acid	M117T755	116.93	754.7	23.18213433	C3H7N3O2	C00581	15650484	18042399.16	28.68	48867035.09	28703855.12	114.64	0.32	-1.64	0.562489627	0.813988595
1-Naphthylamine	M124T62	123.902	61.8	7.878284886	C10H9N	C14790	3892825.77	3864918.92	25.46	4911883.22	4555895.75	28.03	0.79	-0.34	0.351594398	0.706626033
Pyroglutamic acid	M128T87	128.0354	87.3	0.593585836	C5H7NO3	C01879	38072786.61	35447420.31	21.29	40284953.92	38066615.61	14.96	0.95	-0.08	0.68394992	0.873173397
Itaconic acid	M129T118	129.0193	117.8	0	C5H6O4	C00490	6903243.71	7213339.63	28.94	10100563.56	9373941.14	21.41	0.68	-0.55	0.13856192	0.558084098
L-Isoleucine	M130T304	130.0863	304.2	0.145824172	C6H13NO2	C00407	20862699.74	19777398.79	57.34	16071136.19	11441969.87	59.28	1.3	0.38	0.64118692	0.854494219
Beta-Leucine	M130T335	130.0862	335	8.64042458	C6H13NO2	C02486	4046044.57	3774696.05	51.76	8050551.33	8667955	71.45	0.5	-0.99	0.473122911	0.770114926
Creatine	M130T96	130.0622	96.4	0.184527095	C4H9N3O2	C00300	38444610.33	41121386.74	32.57	43280946.89	44859193.88	9.91	0.89	-0.17	0.50926089	0.790184394
Glutaric acid	M131T302	130.9929	301.7	1.666396675	C5H8O4	C00489	1563393.9	1129792.32	77.1	3936347.06	4020944.88	26.08	0.4	-1.33	0.08052316	0.500795454
D-Asparagine	M131T85	131.0463	85.1	0.579947698	C4H8N2O3	C01905	18789256.8	20425339.44	25.42	20770613.05	19882347.57	14.4	0.9	-0.14	0.546542024	0.806321885
L-Malic acid	M133T157	133.0145	157.4	2.074961752	C4H6O5	C00149	4240918.25	4241578.55	65.59	2049602.99	1650448.08	38.57	2.07	1.05	0.3133122	0.683254328
Adenine	M134T247	134.0473	246.6	0.566964049	C5H5N5	C00147	5142357.28	5066759.99	22.83	4973817.29	3845375.63	39.54	1.03	0.05	0.813010009	0.931020181
Phenylacetic acid	M135T60	134.8654	60	12.42902269	C8H8O2	C07086	7634554.01	7532058.04	25.64	7722909.86	7319320.21	10.11	0.99	-0.02	0.858361697	0.951995562
Phenyl acetate	M136T887_2	135.9711	887.5	10.66205572	C8H8O2	C00548	29818692.51	27320808.05	64.39	59164900.57	48494771.77	40.45	0.5	-0.99	0.165425643	0.577347834
4-Nitrophenol	M138T600	138.0207	599.7	7.245290018	C6H5NO3	C00870	5140981.86	5391858.57	22.56	6164456.95	6042831.06	10.39	0.83	-0.26	0.260172217	0.651273828
4-Acetamidobutanoic acid	M144T119	144.0667	118.9	0.527533427	C6H11NO3	C02946	2511860.99	1963663.32	63.85	3468376.95	3934774.61	36.25	0.72	-0.47	0.405756207	0.733193823
L-Glutamic acid	M146T78	146.0462	77.6	1.88981295	C5H9NO4	C00025	1506271473	1711702517	24.36	1465111363	1439007426	4.13	1.03	0.04	0.973091518	0.991376945
L-Methionine	M148T129	148.0438	128.8	0	C5H11NO2S	C00073	18081413.71	6500868.29	135.72	35016537.02	45360387.68	73.22	0.52	-0.95	0.39969161	0.729504861
L-Histidine	M154T98	154.0625	97.6	1.79148073	C6H9N3O2	C00135	30599675.75	31520159.35	22	44308974.44	44535785.35	18.21	0.69	-0.53	0.091543893	0.516562749
Oxoadipic acid	M159T231	158.9779	231.4	4.675188388	C6H8O5	C00322	63045889.8	52503109.29	72.6	54337369.01	57620119.24	58.55	1.16	0.21	0.874256894	0.958779192
Fructose-1P	M161T168	161.0441	167.6	5.650626133	C6H12O6	C10906	10794578.58	12081680.26	79.07	9871092.32	9177470.73	17.47	1.09	0.13	0.724966308	0.89407469
D-Galactose	M161T303	161.0443	303.4	4.408705368	C6H12O6	C00984	10389435.58	14098998.47	67.14	14973786.05	13040413.68	24.99	0.69	-0.53	0.371362538	0.713709336
D-Glucose	M161T347	161.0441	346.9	5.650626133	C6H12O6	C00031	3555988.89	1293025.6	122.05	5753256.43	6179134.31	58.69	0.62	-0.69	0.35901906	0.710024392
D-(+)-Glucose	M161T473	161.0442	472.6	4.955896394	C6H12O6	C00293	2864026.25	1892487.34	85.73	5775690.48	6378458.56	32.92	0.5	-1.01	0.167485709	0.578199373
D-Glucopyranoside	M161T530	161.044	529.7	6.271538621	C6H12O6	C00738	2910723.67	1875348.71	87.17	3663591.02	4327548.23	44.44	0.79	-0.33	0.535829594	0.800583821
L-Gulose	M161T591	161.0442	591	5.029675083	C6H12O6	C15923	3967352.08	5003034.15	66.57	6039256.17	4972829.88	31.75	0.66	-0.61	0.343079977	0.70228301
D-Aldose	M161T674	161.0473	673.8	14.21962717	C6H12O6	C00737	1047137.81	715699.98	68.82	7700413.1	8825380.14	30.67	0.14	-2.88	0.007550248	0.239279076
Alpha-D-Glucose	M161T684	161.0442	684.3	5.029675083	C6H12O6	C00267	3559479.71	4402644.43	65.59	4873730.88	4460144.14	74.59	0.73	-0.45	0.695763721	0.879616833
Terephthalate	M165T155	165.0194	155.3	0.460551911	C8H6O4	C06337	5502214.64	4916099.86	38.22	8600274.14	7383713.88	48.8	0.64	-0.64	0.299917022	0.674413009
D-Phenylalanine	M165T302_1	165.0195	302.2	12.38608354	C9H11NO2	C02265	15597171.4	8851708.99	94.87	13098780.36	7187232.47	85.56	1.19	0.25	0.891408353	0.964428914
L-3-Phenyllactic acid	M165T332	165.0557	331.9	0	C9H10O3	C05607	2948818.38	3386700.24	41.08	5699928.52	5636373.06	8.67	0.52	-0.95	0.062706025	0.47242899
1-Methylxanthine	M165T854	165.0412	854	3.635455874	C6H6N4O2	C16358	16942129.01	13696005.4	34.71	12136347.97	9552442.61	38.41	1.4	0.48	0.286774868	0.667855096
Phthalic acid	M165T963	165.0411	963.5	5.957108096	C8H6O4	C01606	17060737.94	17982530.62	45.76	18650907.03	19022827.3	25.53	0.91	-0.13	0.681210346	0.871527928
L-Arginine	M173T101	173.1045	101	0.439041157	C6H14N4O2	C00062	79801602.56	85796518.57	22.62	80565966.23	83953079.42	8.91	0.99	-0.01	0.87282836	0.958135638
Shikimic acid	M173T372	173.0459	372.5	2.172833913	C7H10O5	C00493	17701175.02	20670043.89	35.51	23641163.09	19173271.7	35.7	0.75	-0.42	0.378827877	0.718982262
Guanidinosuccinic acid	M175T35	174.9584	34.9	19.85879438	C5H9N3O4	C03139	8213991.82	10030493.95	42.05	6938586.37	5363606.37	53.17	1.18	0.24	0.685207206	0.873599156
N-Formyl-L-methionine	M176T163	176.0391	163.1	2.135889129	C6H11NO3S	C03145	15798704.11	12470484.4	44.57	19768294.7	15794712.92	45.7	0.8	-0.32	0.556423382	0.811186517
Gluconolactone	M177T104	177.0408	103.6	1.694524652	C6H10O6	C00198	7026376.59	7176283.97	29.09	5508491.35	5696486.8	10.74	1.28	0.35	0.311271005	0.682484381
Pyrophosphate	M177T76	176.9362	75.8	1.559884297	H4P2O7	C00013	543622253.8	632873938.1	32.91	684182710.2	674917922	2.91	0.79	-0.33	0.334984612	0.69679673
4-Hydroxyphenylpyruvic acid	M179T328	179.035	328.1	0	C9H8O4	C01179	361081.74	395840.51	21	2419486.93	1980083.59	62.55	0.15	-2.74	0.009768327	0.274378771
myo-Inositol	M179T85_2	179.0563	85.1	0.982931067	C6H12O6	C00137	22502442.47	21559005.57	27.29	4846807.74	2830705.2	72.77	4.64	2.21	0.015886834	0.318461104
D-Mannose	M180T799	179.9892	798.9	8.325966136	C6H12O6	C00159	6840024.86	6331539.24	73.72	11874010.73	12852447.9	68.46	0.58	-0.8	0.501252315	0.786105151
Allose	M180T826	179.9892	825.5	8.156413863	C6H12O6	C01487	12355242.65	11363500.16	74.16	15414930.06	12561120	88.2	0.8	-0.32	0.893841873	0.964854042
Paraxanthine	M180T953	179.9891	952.8	5.735753148	C7H8N4O2	C13747	757042605.6	664791592.5	31.04	621233521.6	643326378.3	7.29	1.22	0.29	0.389932794	0.724372254
Hydroxyphenyllactic acid	M181T152	181.0509	152.1	1.524433184	C9H10O4	C03672	6266533.11	6281106.12	36.79	9757367.09	10806805.77	23.88	0.64	-0.64	0.156694723	0.571896277
Acetylcholine chloride	M181T92	180.9733	91.9	7.519966128	C7H16NO2. Cl	C08201	403602543.9	472245471.2	31.01	431306493.9	432714359.1	1.84	0.94	-0.1	0.658347762	0.861818291
2,4-Dinitrophenol	M183T444	183.0049	444	0.961722883	C6H4N2O5	C02496	19111640.91	20031632.31	28.56	24532519.68	25312393.51	6.75	0.78	-0.36	0.198975889	0.604312013
Azelaic acid	M187T305	187.0979	304.6	1.475163537	C9H16O4	C08261	11383742.34	12521102.05	81.16	8432779.36	11159680.88	59.71	1.35	0.43	0.947614856	0.982009993
10-Hydroxydecanoic acid	M187T574	187.1338	573.9	0.662627489	C10H20O3	C02774	1324562.23	1531159.47	39.2	1348009.67	1341409.36	20.85	0.98	-0.03	0.831125331	0.938466573
N-Acetyl-L-glutamine	M188T441	188.0756	440.8	21.83635049	C7H12N2O4	D07063	1404013.94	1432575.2	27.68	2219721.23	2173241.34	8.54	0.63	-0.66	0.050215347	0.447682596
N-Acetylglutamic acid	M188T80	188.0562	79.5	1.191133289	C7H11NO5	C00624	212149454.3	222299235.1	38.29	1189805209	1299289212	18.96	0.18	-2.49	0.002809714	0.173391915
Citric acid	M191T79	191.02	79.2	1.444874882	C6H8O7	C00158	129155240.7	155136326.2	35.88	107966047	102669378.7	12.5	1.2	0.26	0.628291515	0.846936723
Quinate	M191T80	191.0565	79.9	1.968004229	C7H12O6	C00296	16323008.85	5430871.79	123.8	8973347.17	4189071.13	127.93	1.82	0.86	0.521678766	0.796685673
N-Acetyl-L-phenylalanine	M206T372	206.0822	371.9	0.116458384	C11H13NO3	C03519	1739806.87	1895598.83	23.49	2700244.07	2677584.44	4.52	0.64	-0.63	0.036667988	0.416951966
Pantothenic acid	M218T157	218.1038	157.1	1.723949789	C9H17NO5	C00864	679260670	594470963	39.01	749125132.1	613945928	36.76	0.91	-0.14	0.739136677	0.898709108
L-Cystathionine	M221T83	221.06	83.1	0.560933683	C7H14N2O4S	C02291	188948522.9	209755759.4	26.21	216915184.5	220779974.7	3.79	0.87	-0.2	0.428805551	0.746433439
Thymidine	M223T842	223.0283	842	1.827647653	C10H14N2O5	C00214	20354029.73	23502274.07	30.11	16988393.25	14219371.58	39.17	1.2	0.26	0.541738079	0.8032273
Ribose 1-phosphate	M229T74	229.0119	74.5	0	C5H11O8P	C00620	106741496	120598986.5	31.69	50576509.5	45396902.92	21.04	2.11	1.08	0.037922955	0.418954145

gamma-Glutamylcysteine	M249T91	248.9602	91.3	0.034860231	C8H14N2O5S	C00669	1001430102	1096885346	29.02	982405858.9	980675858.6	3.2	1.02	0.03	0.95254513	0.984093508
Palmitic acid	M255T864	255.2334	864.2	1.86495968	C16H32O2	C00249	482599884.5	446021734.9	45.27	410082888.5	407070122.9	25.8	1.18	0.23	0.725940834	0.894538466
Galactose 1-phosphate	M259T73	259.0221	73	1.158202331	C6H13O9P	C00103	64888784.28	54908757.8	36.54	18682772.31	18642557.34	2.62	3.47	1.8	0.025698831	0.37071075
Inosine	M267T250_1	267.0739	250.1	1.407850037	C10H12N4O5	C00294	875142844.5	969493775.4	28.43	444038463.2	378478755.4	29.42	1.97	0.98	0.049304383	0.447085347
Pentostatin	M267T781	267.1091	780.9	2.995030869	C11H16N4O4	C02267	1845901.78	1560260.2	29.84	1204888.64	1332206.78	32.5	1.53	0.62	0.172129644	0.580865273
16-Hydroxy hexadecanoic acid	M271T965	271.2303	965.1	8.84856891	C16H32O3	C18218	2663402.45	2900288.11	35.01	21879007.14	21658438.25	29.58	0.12	-3.04	0.001755311	0.148012567
6-Phosphogluconic acid	M275T71	275.0211	70.8	13.4535132	C6H13O10P	C00345	52262480.73	47402712.23	21.05	6749829.55	5831700.37	38.75	7.74	2.95	0.001031318	0.11925627
Alpha-Linolenic acid	M277T923	277.2166	922.8	2.611676213	C18H30O2	C06427	5702597.72	6483154.4	29.35	1429725.88	1978165.32	76.77	3.99	2	0.10143714	0.529500036
Gamma-Linolenic acid	M277T930	277.2166	930	0.936085887	C18H30O2	C06426	4206625.1	5545978.12	56.78	6943247.2	7246763.94	16.67	0.61	-0.72	0.226743606	0.624390061
Sulfamethoxypyridazine	M279T328	279.0555	328.4	0.84199876	C11H12N4O3S	D02439	610912.25	663488.76	24.82	630918.72	628925.09	3.51	0.97	-0.05	0.759092186	0.907989009
Bovinic acid	M280T807	280.2324	806.6	27.8340406	C18H32O2	C04056	1668852.48	524649.48	132.53	2540797.43	1606081	70.08	0.66	-0.61	0.350869364	0.706199894
Guanosine	M282T269	282.0847	269.3	0.978429528	C10H13N5O5	C00387	11268335.4	12436571.88	22.3	15062024.74	19286498.54	86.77	0.75	-0.42	0.680019314	0.871292021
Xanthosine	M283T165	283.069	165	2.034839562	C10H12N4O6	C01762	2465584.66	2868363.98	69.29	7768373.02	8153010.17	30.15	0.32	-1.66	0.087726848	0.50985718
Cinchonidine	M294T649	294.1798	648.7	22.43525898	C19H22N2O	C11379	3530829.09	3969642.3	36.25	3636601.9	3528439.46	5.81	0.97	-0.04	0.768288111	0.909849121
9,10-Epoxyoctadecenoic acid	M295T829	295.2276	828.9	0.758736649	C18H32O3	C14825	6885483.15	7967474.5	38.32	10952135.91	11623467.39	19.18	0.63	-0.67	0.145743101	0.564489525
Nonadecanoic acid	M297T854	297.2379	854.2	18.93049527	C19H38O2	C16535	22951739.2	21208255.88	38.35	19587789.26	18843781.48	73.85	1.17	0.23	0.572552107	0.818573772
5'-Methylthioadenosine	M297T895	297.2455	894.8	7.139938041	C11H15N5O3S	C00170	13508583.49	14355761.88	19.88	21884303.68	23491215.07	28.34	0.62	-0.7	0.09584442	0.522503113
EPA (d5)	M301T910	301.2173	909.7	0.079676699	C20H30O2	C06428	8642308.26	8424257.65	24.82	13116690.22	12944658.64	27.98	0.66	-0.6	0.133458273	0.555466673
Isopimaric acid	M301T954	301.2174	953.7	0.252309461	C20H30O2	C09118	1747713.06	1078025.37	70.98	4353721.95	2254716.16	93.21	0.4	-1.32	0.279113382	0.663853677
Arachidonic acid	M303T975	303.2325	975.4	1.398267006	C20H32O2	C00219	15111566.63	13754501.6	80.39	31313907.62	30958082.55	34.06	0.48	-1.05	0.190943803	0.59700096
(-)-Epigallocatechin	M306T93	306.0753	92.7	4.247320839	C15H14O7	C12136	319580716.2	335656435.4	23.32	613506531.8	608734063.2	5.58	0.52	-0.94	0.010080437	0.275599721
N-Acetyl-a-neuraminic acid	M308T82	308.0988	81.8	0.246674119	C11H19NO9	C19909	377360383.3	436027452	79.79	484803607.2	528016627	85.07	0.78	-0.36	0.88962404	0.964089943
Arachidic acid	M311T768	311.1685	768.2	0.334236615	C20H40O2	C06425	15009584.15	15098240.03	15.07	18465257.26	16684071.11	19.49	0.81	-0.3	0.218356883	0.6176256
8-HETE	M319T847	319.2268	846.5	3.207750728	C20H32O3	C14776	4239470.59	4655586.46	60.18	5235082.71	5360810.6	35.9	0.81	-0.3	0.540368558	0.802792318
UMP	M323T87_2	323.0286	86.8	0.074296827	C9H13N2O9P	C00105	166120741.9	161868349.1	31.16	184841527.8	167872778.7	39.04	0.9	-0.15	0.770412923	0.910854394
N-Glycolylneuraminic acid	M324T80	324.0939	80.1	0.851605044	C11H19NO10	C03410	18011222.12	20900823.44	38.29	19008746.95	19193382.94	2	0.95	-0.08	0.701728615	0.883219172
11-Dehydrocorticosterone	M325T785	325.1839	785.2	10.73238866	C21H28O4	C05490	20101353.03	19656880.31	11	25428830.47	24546143.8	13.33	0.79	-0.34	0.076429276	0.49762741
Cyclic AMP	M328T313	328.0448	312.7	1.044673703	C10H12N5O6P	C00575	502274.48	579101.9	28.16	608158.65	602585.55	9.52	0.83	-0.28	0.311864409	0.682790036
Docosapentaenoic acid (22n-3)	M329T956	329.2497	956	3.268036387	C22H34O2	C16513	32214755.79	33473922.33	11.47	25613146.46	30413567.63	58.51	1.26	0.33	0.475519036	0.772031306
dAMP	M330T150	330.0616	149.9	2.048102536	C10H14N5O6P	C00360	551935.48	152541.57	145.91	2305061.13	3029687.31	73.86	0.24	-2.06	0.193151692	0.598352643
Adrenic acid	M331T858_1	331.2639	858.2	0.978072166	C22H36O2	C16527	35247906.61	30698438.43	106.31	67314648.34	64907342.98	25.6	0.52	-0.93	0.362395827	0.710160636
3'-AMP	M346T123_2	346.059	123.2	9.177625781	C10H14N5O7P	C01367	257088470	207536300.4	87.46	466713376.3	359094046.1	53.99	0.55	-0.86	0.286085051	0.667014537
IMP	M347T99	347.04	99	0.50714615	C10H13N4O8P	C00130	2723990.57	1876582.89	96.57	6113428.65	5545993.35	43.65	0.45	-1.17	0.178981682	0.586739329
S-Adenosylhomocysteine	M383T305_2	383.1132	305.2	2.35849178	C14H20N6O5S	C00021	42005571.7	43772779.24	25.84	45408491.04	45845666.64	5.98	0.93	-0.11	0.567019886	0.816443421
Mitragynine	M397T788_1	397.2252	788	29.89739825	C23H30N2O4	C09226	8942492.76	11051076.16	49.08	14296472.17	15481862.29	18.42	0.63	-0.68	0.206407611	0.607588486
Glycochenodeoxycholic acid	M449T839	449.3144	839.2	0.890245227	C26H43NO5	C05466	4173556.61	3054142.76	64.96	7869257.07	6958022.5	47.03	0.53	-0.91	0.195548952	0.59951654
5-Methyltetrahydrofolic acid	M458T307_1	458.1781	306.8	2.671450251	C20H25N7O6	C00440	5788581.28	6373671.62	25.49	6771351.17	6489835.07	26.12	0.85	-0.23	0.510230618	0.790681354
Glycocholic acid	M465T865_1	465.3028	865.1	3.759297283	C26H43NO6	C01921	69035462.42	72061636.89	11.74	87074240.88	85575251.63	14.37	0.79	-0.33	0.100678442	0.529130677
Adenosine diphosphate ribose	M540T148_2	540.0577	147.7	8.128761056	C15H23N5O14P2	C00301	1788361.28	1914658.17	29.47	1278976	1310160.11	8.4	1.4	0.48	0.186040804	0.591966981
Uridine diphosphate glucose	M565T79_2	565.0448	78.8	5.132336409	C15H24N2O17P2	C00029	109527645	130855229	41.47	158117274.5	158776171.5	16	0.69	-0.53	0.225957861	0.623751538
Uridine diphosphate glucuronic acid	M579T72_2	579.0256	71.8	2.4178551	C15H22N2O18P2	C00167	112912457.7	114510576.6	43.59	148801097.7	133455040.2	18.11	0.76	-0.4	0.313840128	0.683254328
Uridine diphosphate-N-acetylglucosamine	M606T80_3	606.0835	79.6	15.13982809	C17H27N3O17P2	C00043	817245095.4	665032057.1	55.96	517605730.4	398789623.2	76.2	1.58	0.66	0.369990536	0.713141151
Oxidized glutathione	M611T78_2	611.1522	77.7	12.23278171	C20H32N6O12S2	C00127	51694015.91	41405916.06	45.18	44316744.62	38924353.21	22.78	1.17	0.22	0.715134184	0.889606104
NAD	M662T148_3	662.1087	147.7	10.42124956	C21H28N7O14P2	C00003	22034050.31	20977618.94	43.67	13140438.88	11336390.89	30.41	1.68	0.75	0.197308054	0.601895645
Dephospho-CoA	M668T129_2	668.1187	129	17.67618725	C21H35N7O13P2S	C00882	508880.1	264766.75	88.89	1506967.78	1655576.15	48.11	0.34	-1.57	0.100589788	0.529056784
